# Gut microbiota and autoimmune neurologic disorders: a two-sample bidirectional Mendelian randomization study

**DOI:** 10.3389/fmicb.2024.1337632

**Published:** 2024-04-24

**Authors:** Mengyuan Zhang, Jie Fang, Chamou Zheng, Qing Lin, Jiawei Zhang

**Affiliations:** ^1^Department of Neurology and Department of Neuroscience, The First Affiliated Hospital of Xiamen University, School of Medicine, Xiamen University, Xiamen, China; ^2^Fujian Key Laboratory of Brain Tumors Diagnosis and Precision Treatment, Xiamen, China; ^3^Xiamen Key Laboratory of Brain Center, Xiamen, China; ^4^Xiamen Medical Quality Control Center for Neurology, Xiamen, China; ^5^Fujian Provincial Clinical Research Center for Brain Diseases, Xiamen, China; ^6^Xiamen Clinical Research Center for Neurological Diseases, Xiamen, China; ^7^Department of Neonatology, The First Affiliated Hospital of Xiamen University, Xiamen, China

**Keywords:** gut microbiota, Myasthenia Gravis, Multiple Sclerosis, Guillain-Barré Syndrome, Mendelian Randomization, causal relationship

## Abstract

**Background:**

Increasing evidence has suggested that alterations in the gut microbiome are correlated with autoimmune neurologic disorders, yet the causal relationship between them has yet to be established.

**Methods:**

From the published genome-wide association study (GWAS) summary statistics, we obtained data on the gut microbiota and three autoimmune neurologic disorders (Multiple Sclerosis, Guillain-Barré Syndrome, and Myasthenia Gravis). We then implemented a two-sample Mendelian Randomization (MR) to determine the causal relationship between the gut microbiota and the diseases. To validate the results, we conducted a series of sensitivity analyses. Finally, to verify the direction of causality, a reverse-causality analysis was done.

**Results:**

We discovered that a higher relative abundance of the genus *Ruminococcus2* (OR: 1.213, 95% CI: 1.006–1.462, *p* = 0.043, P_FDR_ = 0.048) and the genus *Roseburia* (OR: 1.255, 95% CI: 1.012–1.556, *p* = 0.038, P_FDR_ = 0.048) were associated with a higher risk of MS. Furthermore, the higher the abundance of the class *Mollicutes* (OR: 3.016, 95% CI: 1.228–7.411, *p* = 0.016, P_FDR_ = 0.021), the genus *Eubacterium (hallii group)* (OR: 2.787, 95% CI: 1.140–6.816, *p* = 0.025, P_FDR_ = 0.025), and the phylum *Tenericutes* (OR: 3.016, 95% CI: 1.228–7.411, *p* = 0.016, P_FDR_ = 0.021) was linked to a greater probability of GBS. Additionally, the higher the abundance of the genus *Ruminococcaceae UCG005* (OR: 2.450, 95% CI: 1.072–5.598, *p* = 0.034, P_FDR_ = 0.036), the genus *Holdemania* (OR: 2.437, 95% CI: 1.215–4.888, *p* = 0.012, P_FDR_ = 0.024), genus *Lachnoclostridium* (OR: 3.681, 95% CI: 1.288–10.521, *p* = 0.015, P_FDR_ = 0.025) and the genus *Eubacterium (ruminantium group)* (OR: 2.157, 95% CI: 1.211–3.843, *p* = 0.003, P_FDR_ = 0.016) correlated with a greater chance of MG occurrence. No SNPs were identified as outliers through sensitivity analysis. Then, the results of the reverse MR analysis did not indicate any reverse causality.

**Conclusion:**

Our findings demonstrate a causal relationship between the gut microbiota and three autoimmune neurologic disorders, providing novel insights into the mechanisms of these autoimmune neurologic disorders that are mediated by gut microbiota.

## 1 Introduction

Autoimmune neurologic disorders can affect both the central and peripheral nervous systems, such as Multiple Sclerosis (MS), Guillain-Barré Syndrome (GBS), and Myasthenia Gravis (MG) (Vass, 2012). Over the past few decades, the incidence of these diseases has risen steadily, and they have caused a heavy burden to patients and society due to their high mortality and disability rates (Rubin et al., 2018). Therefore, it is imperative to discover potential causal risk factors for autoimmune neurologic disorders.

Despite a lack of understanding of the exact causes of autoimmune neurologic disorders, genetics, environmental factors, and their interactions are believed to play a major role in their development (Diaz-Marugan et al., 2023; Kapoor et al., 2023). Evidence has been mounting that the gut microbiota is closely related to the host’s health and is implicated in the cause of a variety of complex human diseases, including autoimmune diseases (Wang et al., 2023). The gut microbiota is an intricate and ever-changing community of ecological microbes that inhabit the human digestive tract, including bacteria, archaea, viruses, parasites, and other microorganisms (Lozupone et al., 2012). The communication between the microbiota and the nervous system is likely facilitated by the microbiota-gut-brain axis, which involves a variety of pathways such as the immune system, the vagus and enteric nerves, and molecules or metabolites derived from the microbiota (Nunez et al., 2021). Alterations in the composition of gut microbiota, including decreased species diversity and increased presence of pathogens, have been observed in several autoimmune neurologic disorders. For example, an investigation found considerable disparities in the concentrations of operational taxa among MG patients, specifically *Firmicutes*, *Bacteroides*, and *Actinobacteria* (Kapoor et al., 2023). Reports showed that MS patients have a decreased presence of *Parabacteroides* and *Butyricicoccus* (Cekanaviciute et al., 2017; Diaz-Marugan et al., 2023), while *Amuciniphila*, *Eggerthellalenta*, *Bifidobacterium adolescentis*, and *Ruminococcus* taxa are more abundant. Additionally, a strong connection was observed between GBS and *Campylobacter jejuni* infection (Allos, 1997). Nevertheless, it is still unknown whether there is a causal association between the gut microbiota and autoimmune neurologic disorders.

Conventionally, Randomized Controlled Trials (RCTs) can potentially help establish a causal relationship. However, due to certain limitations such as technological constraints and ethical concerns, these trials can be difficult to implement. As an alternative, Mendelian Randomization (MR) analysis can provide a viable solution. Through the use of genetic variations that are present before the onset of any disease, MR reduces the potential of confounding factors, reverse causation, or other biases from affecting the accuracy of the results. By leveraging the Genome-Wide Association Study (GWAS), Single Nucleotide Polymorphisms (SNPs) associated with the exposure can be used as Instrumental Variables (IVs) in a two-sample MR analysis to determine the causal relationship between the exposure and the outcome (Davey Smith and Hemani, 2014; Bowden and Holmes, 2019).

In this study, we conducted a MR analysis using publicly available GWAS data to explore the potential causal relationship between the gut microbiota and three autoimmune neurologic disorders: MS, MG, and GBS. Our research may provide a novel perspective on developing new treatment strategies, such as probiotic therapy and dietary modulations for autoimmune neurologic disorders.

## 2 Methods

### 2.1 Study design

**Figure 1:**
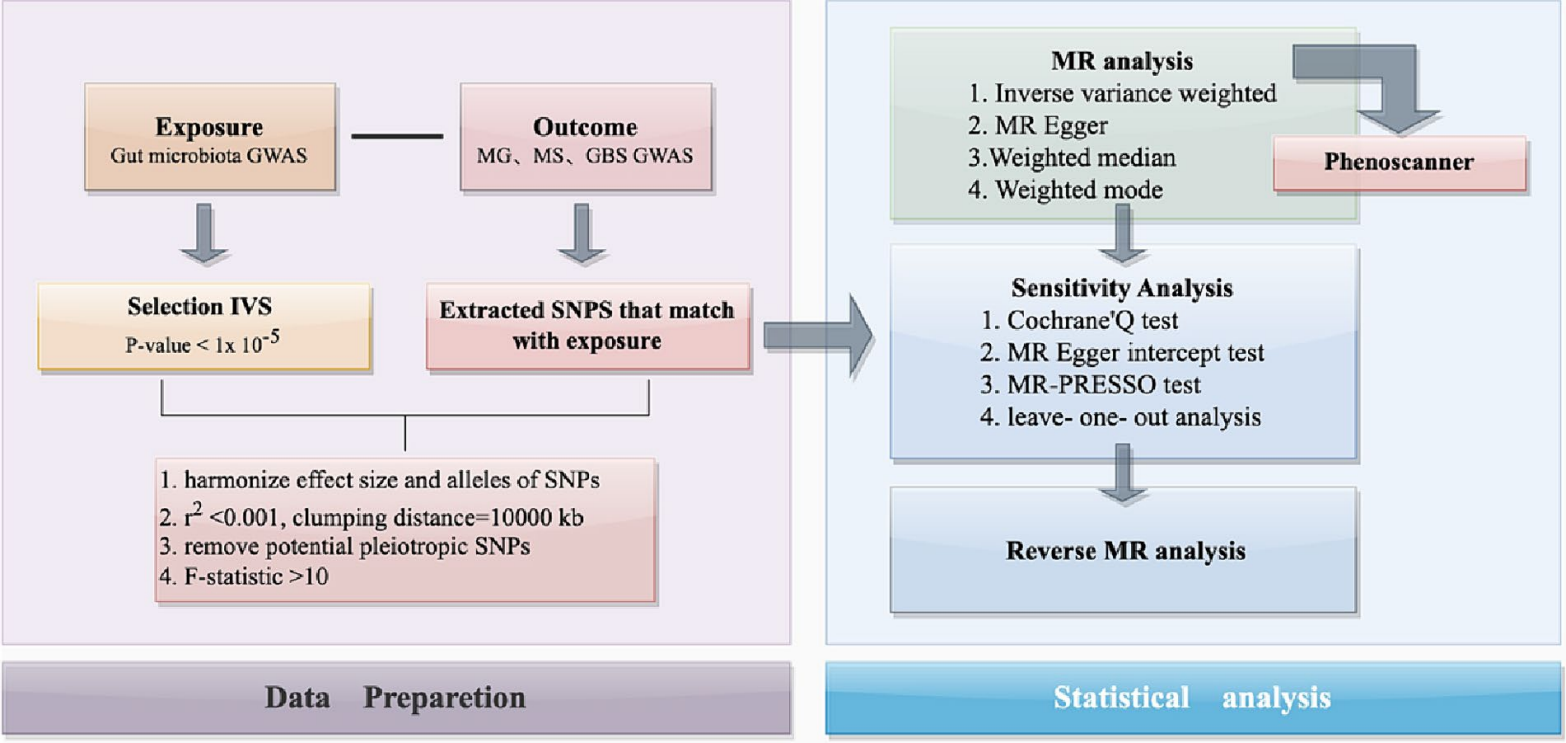
The flowchart of the present study. The whole workflow of MR analysis. MR, Mendelian randomization; MS, Multiple Sclerosis; GBS, Guillain-Barré Syndrome; MG, Myasthenia Gravis.

This study used publicly available data from a GWAS summary to identify suitable IVs for MR analysis to investigate the causal relationship between gut microbiota and three autoimmune neurologic disorders: MS, MG, and GBS (Figure 1). Three assumptions were strictly adhered to in order to ensure the accuracy of the results: (Vass, 2012) the IVs should relate to the exposure; (Rubin et al., 2018) they should not be related to any confounding factors; (Diaz-Marugan et al., 2023) their effects on outcomes should only be through the exposure. Ethical approval and informed consent have been provided in the original studies.

### 2.2 Data sources

#### 2.2.1 Exposure data

The MiBioGen consortium^1^ conducted a large-scale GWAS study to obtain summary statistics of the gut microbiome, comprising data from 24 cohorts from different countries and a total of 18,340 individuals (Kurilshikov et al., 2021). A total of 211 microbial community data were analyzed, encompassing 9 phyla, 16 classes, 20 orders, 35 families, and 131 genera. After removing 17 unknown groups, 194 microbial taxa were taken into consideration.

#### 2.2.2 Outcome data

The outcome data generated comes from a publicly accessible database, which contains a comprehensive description of the data in the original source. The MS data was from a GWAS conducted by the International Multiple Sclerosis Genetics Consortium (IMSGC) in 2019, involving 115,803 individuals, with 47,429 of them being MS patients and 68,374 being healthy controls (International Multiple Sclerosis Genetics Consortium, 2019). The Finnish database provided GWAS data for MG and GBS. There were 232 cases and 217,056 controls for MG, making a total of 217,288 individuals, and 213 cases and 215,718 controls for GBS, resulting in 215,931 individuals. To ensure consistency in the study, all outcome GWAS data used were from individuals of European ethnicity. All GWAS data utilized in the analysis is detailed in Table 1.

**Table 1:** All GWAS data information.

Datasets	Phenotype	Source	Year	Sample size	Case	Control	Population
Exposure	Gut microbiota	MiBioGen	2021	18,340	–	–	Mixed
Outcome	Multiple sclerosis	IMSGC	2019	115,803	47,429	68,374	European
	Guillain-Barre syndrome	Finn	2021	215,931	213	215,718	European
	Myasthenia gravis	Finn	2021	217,288	232	217,056	European

### 2.3 Instrumental variable selection (IVs)

To confirm the causal relationship between the gut microbiome and autoimmune neurologic disorders, we utilized quality control modalities to select the relevant IVs. Initially, SNPs that had a significant correlation with the gut microbiome were chosen to be IVs. Given that only a minor proportion of the gut microbiome exhibits more than three independent SNPs at a genome-wide significant threshold with a *p* < 5 × 10^−8^, we opted to raise the *p*-value to 1 × 10^−5^ to obtain an adequate number of IVs and yield more comprehensive outcomes. Secondly, SNPs with a minor allele frequency (MAF) of less than 0.01 were not included. Thirdly, to avoid IVs with linkage disequilibrium (LD), the SNPs chosen in our study were assessed against the European 1000 Genomes Project reference panel, with the requirements of *r*^2^ < 0.001 and clump distance >10,000 kb. According to the formula *F* = $\frac{R^{2}\times \left(N-1-K\right)}{\left(1-R^{2}\right)\times K}$ (*R*^2^ denotes the fraction of variance explained by genetic variation in the exposure, N represents the sample size, and K is the number of SNPs.) (Li et al., 2022), if the F-statistic>10, it can be deduced that there is no remarkable weak tool bias. Phenoscanner is employed to recognize potential confounding elements (Zhang et al., 2023).

### 2.4 Statistical analysis

#### 2.4.1 MR analysis

We applied a two-sample MR method to investigate the causal link between microbiome characteristics and three autoimmune neurological disorders. Four methods commonly used for features with multiple IVs are Inverse-Variance Weighted (IVW) tests, Weighted Modes (Hartwig et al., 2017), Weighted Median Estimator (WME) (Bowden et al., 2016), and MR-Egger regression (Hartwig et al., 2017). Evidence from prior studies has shown that the IVW method produces more meaningful results than other approaches (Burgess et al., 2013), thus this study primarily utilizes the IVW method. With the other methods providing supplementary explanations. The OR and 95% confidence interval (CI) were used to demonstrate the effect size. The Weighted Modes, WME method and the MR-Egger test were employed as additional approaches for MR analysis in the study. The Benjamini–Hochberg (BH) method was applied to calculate a false discovery rate *p*-value (P_FDR_) in order to adjust for multiple tests. Results were considered significant if the FDR < 0.05 (Korthauer et al., 2019; Luo et al., 2023).

#### 2.4.2 Sensitivity analysis

Cochran’s Q statistic (Greco et al., 2015) was employed to determine heterogeneity, with *p* < 0.05 indicating the presence of heterogeneity. MR-Egger intercept test and MR-PRESSO test can be used to determine if horizontal pleiotropy is present (Verbanck et al., 2018). In our study, when the MR-PRESSO global test indicated significant horizontal pleiotropy, we removed outliers with *p* < 0.05 and re-evaluated the remaining SNPs using the IVW analysis. And the MR-Egger regression intercept has a *p*-value >0.05, indicating no horizontal pleiotropy. Moreover, to evaluate if a single SNP was responsible for the causality in the two sample MR analysis, we performed a leave-one-out sensitivity analysis on the identified significant outcomes.

#### 2.4.3 Reverse MR analysis

To investigate if MS, MG, and GBS, the three neuroimmune diseases, are caused by gut microbiota, we performed a reverse MR analysis utilizing their related SNPs as IVs. We applied MS, MG, and GBS as exposures and the microbiome identified in MR analysis as the outcomes and then employed sensitivity analysis to assess the reliability of the results. Furthermore, the MR Steiger directionality test (Hemani et al., 2017) was utilized to identify if there was a directional causality between the exposure and the outcome.

To explore the causal relationship between the gut microbiome and MS, MG, and GBS, the “Two-Sample MR” R package and “MR-PRESSO” R package were used to carry out a MR study (Yavorska and Burgess, 2017).

## 3 Results

### 3.1 SNP selection from gut microbiome

Among the 194 taxa included in our study, the SNPs that were identified for each microbial taxa are provided (Supplementary Table S1). Details for each SNP include the effect allele, the other allele, the beta, the standard error, the *p*-value, and the *F*-value. Our query of the SNPs on PhenoScanner did not reveal any SNPs associated with the confounders specified (Supplementary Table S2).

### 3.2 Causal effects of the gut microbiome on the three autoimmune neurologic disorders

#### 3.2.1 MS

**Figure 2:**
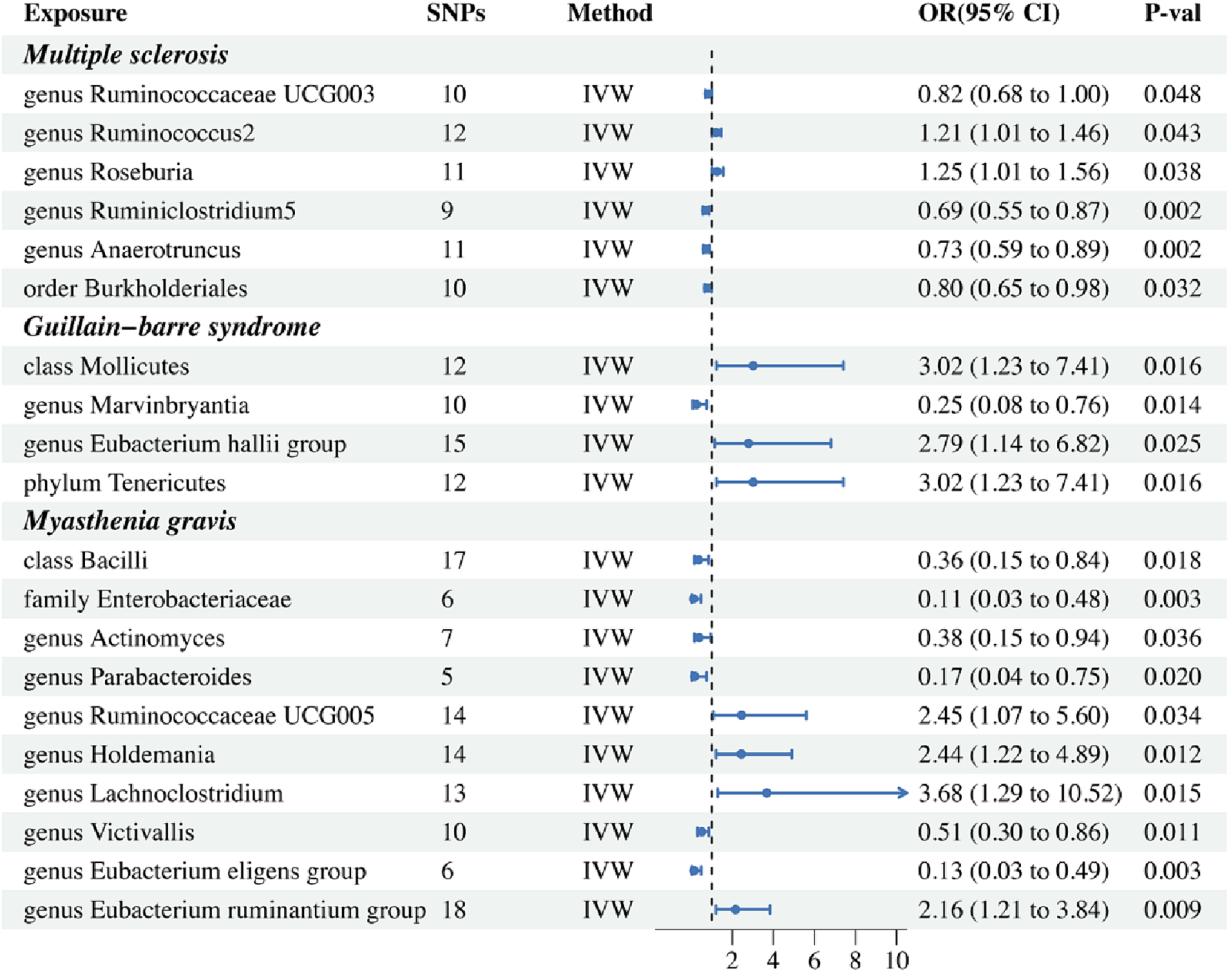
Forest plot of IVW analysis results of the effect of gut microbiome on MS, GBS, MG. IVW, Inverse variance weighted.

Results from the IVW-MR analysis indicated that four microbial taxa (genus *Ruminococcaceae UCG003*: OR: 0.824, 95% CI: 0.680–0.999, *p* = 0.048, P_FDR_ = 0.048; *genus Ruminiclostridium5*: OR: 0.695, 95% CI: 0.554–0.871, *p* = 0.002, P_FDR_ = 0.006; genus *Anaerotruncus*: OR: 0.726, 95% CI: 0.592–0.890, *p* = 0.002, P_FDR_ = 0.006; order *Burkholderiales*: OR: 0.800, 95% CI: 0.653–0.981, *p* = 0.032, P_FDR_ = 0.048) were found to have a negative correlation with MS. Two microbial taxa (genus *Ruminococcus 2*: OR: 1.213, 95% CI: 1.006–1.462, *p* = 0.043, P_FDR_ = 0.048; *genus Roseburia*: OR: 1.255, 95% CI: 1.012–1.556, *p* = 0.038, P_FDR_ = 0.048) were positively correlated with MS (Figure 2 and Table 2). All of them with *p* < 0.05 showed significant results. The MR-Egger analysis revealed a positive correlation between the genus *Ruminococcaceae UCG003* (OR: 1.159, 95% CI: 0.601–2.235, *p* = 0.043, P_FDR_ = 0.048) and MS, however, the result was not statistically significant as the *p* > 0.05.

**Table 2:** Positive MR results of causal links between gut microbiome and MS, GBS, MG.

Gut microbiome	Methods	SNPs	Beta	F-statistic	*p*-value	P_FDR_	OR	95%CI
*Multiple sclerosis*								
Genus Ruminococcaceae UCG003	IVW	10	−0.193	143.093	0.048	0.048	0.824	0.680–0.999	MR Egger	10	0.147	0.672	0.813	1.159	0.601–2.235	Weighted median	10	−0.046	0.694	0.694	0.955	0.759–1.201	Weighted mode	10	−0.009	0.952	0.952	0.991	0.742–1.324
Genus Ruminococcus2	IVW	12	0.193		171.688	0.043	0.048	1.213	1.006–1.462	MR Egger	12	0.236	0.446	0.813	1.266	0.707–2.268	Weighted median	12	0.185	0.141	0.169	1.203	0.940–1.538	Weighted mode	12	0.180	0.326	0.391	1.198	0.849–1.689
Genus Roseburia	IVW	11	0.227			157.391	0.038	0.048	1.255	1.012–1.556	MR Egger	11	0.796	0.124	0.744	2.216	0.884–5.555	Weighted median	11	0.256	0.086	0.129	1.292	0.965–1.730	Weighted mode	11	0.295	0.220	0.345	1.343	0.863–2.088
Genus Ruminiclostridium5	IVW	9	−0.364				128.793	0.002	0.006	0.695	0.554–0.871	MR Egger	9	−0.147	0.802	0.813	0.863	0.286–2.609	Weighted median	9	−0.335	0.043	0.129	0.715	0.517–0.990	Weighted mode	9	−0.326	0.230	0.345	0.722	0.441–1.181
Genus Anaerotruncus	IVW	11	−0.320	157.391				0.002	0.006	0.726	0.592–0.890	MR Egger	11	−0.096	0.813	0.813	0.909	0.421–1.963	Weighted median	11	−0.396	0.006	0.036	0.673	0.508–0.891	Weighted mode	11	−0.411	0.082	0.345	0.663	0.437–1.005
Order Burkholderiales	IVW	10	−0.223		143.093			0.032	0.048	0.800	0.653–0.981	MR Egger	10	−0.355	0.385	0.813	0.701	0.329–1.495	Weighted median	10	−0.239	0.078	0.129	0.787	0.604–1.027	Weighted mode	10	−0.285	0.201	0.345	0.752	0.502–1.127
*Guillain-barre syndrome*								
Class Mollicutes	IVW	12	1.104			171.688	0.016	0.021	3.016	1.228–7.411	MR Egger	12	1.941	0.258	0.344	6.963	0.292–166.094	Weighted median	12	1.204	0.048	0.084	3.332	1.008–11.017	Weighted mode	12	1.054	0.210	0.304	2.869	0.609–13.526
Genus Marvinbryantia	IVW	10	−1.398	143.093			0.014	0.021	0.247	0.081–0.755	MR Egger	10	0.323	0.891	0.891	1.382	0.016–121.625	Weighted median	10	−0.988	0.160	0.160	0.372	0.094–1.476	Weighted mode	10	−1.049	0.302	0.304	0.350	0.054–2.291
Genus Eubacterium (hallii group)	IVW	15	1.025		214.565		0.025	0.025	2.787	1.140–6.816	MR Egger	15	1.308	0.209	0.344	3.700	0.533–25.685	Weighted median	15	1.132	0.063	0.084	3.103	0.940–10.245	Weighted mode	15	0.977	0.304	0.304	2.656	0.442–15.969
Phylum Tenericutes	IVW	12	1.104				171.688	0.016	0.021	3.016	1.228–7.411	MR Egger	12	1.941	0.258	0.344	6.963	0.292–166.094	Weighted median	12	1.204	0.056	0.084	3.332	0.972–11.426	Weighted mode	12	1.054	0.266	0.304	2.869	0.492–16.730
*Myasthenia gravis*								
Class Bacilli	IVW	17	−1.026	243.139		0.018		0.025	0.358	0.153–0.838	MR Egger	17	−0.557	0.654	0.818	0.573	0.053–6.230	Weighted median	17	−0.384	0.533	0.533	0.681	0.203–2.280	Weighted mode	17	−0.279	0.756	0.756	0.757	0.134–4.261
Family Enterobacteriaceae	IVW	6	−2.198		85.880	0.003		0.015	0.111	0.026–0.477	MR Egger	6	−8.814	0.093	0.435	0.000	0–0.387	Weighted median	6	−1.903	0.055	0.092	0.149	0.021–1.045	Weighted mode	6	−3.391	0.070	0.254	0.034	0.002–0.605
Genus Actinomyces	IVW	7	−0.976			100.186	0.036	0.036	0.377	0.152–0.937	MR Egger	7	−1.941	0.152	0.435	0.144	0.015–1.369	Weighted median	7	−1.286	0.022	0.073	0.276	0.092–0.827	Weighted mode	7	−1.276	0.127	0.254	0.279	0.068–1.144
Genus Parabacteroides	IVW	5	−1.797				71.572	0.020	0.025	0.166	0.037–0.752	MR Egger	5	−3.702	0.497	0.818	0.025	0–302.019	Weighted median	5	−1.711	0.078	0.111	0.181	0.027–1.208	Weighted mode	5	−1.906	0.204	0.323	0.149	0.013–1.747
Genus Ruminococcaceae UCG005	IVW	14	0.896	200.274				0.034	0.036	2.450	1.072–5.598	MR Egger	14	0.323	0.788	0.831	1.381	0.138–13.855	Weighted median	14	0.672	0.239	0.266	1.958	0.640–5.985	Weighted mode	14	0.669	0.415	0.461	1.953	0.411–9.268
Genus Holdemania	IVW	14	0.891		200.274			0.012	0.024	2.437	1.215–4.888	MR Egger	14	1.934	0.089	0.435	6.917	0.892–53.642	Weighted median	14	0.949	0.045	0.092	2.582	1.021–6.529	Weighted mode	14	1.526	0.094	0.254	4.602	0.876–24.161
Genus Lachnoclostridium	IVW	13	1.303			185.982		0.015	0.025	3.681	1.288–10.521	MR Egger	13	2.701	0.174	0.435	14.888	0.391–567.030	Weighted median	13	1.375	0.055	0.092	3.955	0.971–16.103	Weighted mode	13	1.425	0.226	0.323	4.159	0.465–37.159
Genus Victivallis	IVW	10	−0.676				143.093	0.011	0.024	0.509	0.302–0.857	MR Egger	10	0.449	0.831	0.831	1.567	0.029–84.087	Weighted median	10	−0.925	0.011	0.060	0.397	0.195–0.808	Weighted mode	10	−1.156	0.102	0.254	0.315	0.091–1.093
Genus Eubacterium (eligens group)	IVW	6	−2.037	85.880				0.003	0.015	0.130	0.034–0.495	MR Egger	6	−2.123	0.451	0.818	0.120	0.001–17.489	Weighted median	6	−2.202	0.012	0.060	0.111	0.20–0.616	Weighted mode	6	−2.936	0.064	0.254	0.053	0.005–0.604
Genus Eubacterium (ruminantium group)	IVW	18	0.769		257.423			0.009	0.024	2.157	1.211–3.843	MR Egger	18	0.467	0.651	0.818	1.595	0.219–11.610	Weighted median	18	0.636	0.105	0.131	1.889	0.875–4.076	Weighted mode	18	0.607	0.349	0.436	1.836	0.533–6.363

#### 3.2.2 GBS

In addition, we conducted a causal analysis to determine if there is a correlation between gut microbiome and GBS. We found that in the IVW-MR method, there were three microbial taxa (class *Mollicutes*: OR: 3.016, 95% CI: 1.228–7.411, *p* = 0.016, P_FDR_ = 0.021; genus *Eubacterium (hallii group)*: OR: 2.787, 95% CI: 1.140–6.816, *p* = 0.025, P_FDR_ = 0.025; phylum *Tenericutes*: OR: 3.016, 95% CI: 1.228–7.411, *p* = 0.016, P_FDR_ = 0.021) which were positively correlated with GBS, and the genus *Marvinbryantia* (OR: 0.247, 95% CI: 0.081–0.755, *p* = 0.014, P_FDR_ = 0.021) was inversely associated with GBS (Figure 2 and Table 2). The outcomes of the analysis were all significant. The MR-Egger method revealed a positive correlation between the genus *Marvinbryantia* (OR: 1.382, 95% CI: 0.016–121.625, *p* = 0.891, P_FDR_ = 0.891) and GBS, though it was not considered significant.

#### 3.2.3 MG

The IVW-MR analysis of the gut microbiome and MG, showed that six microbial taxa (class *Bacilli*: OR: 0.358, 95% CI: 0.153–0.838, *p* = 0.018, P_FDR_ = 0.025; family *Enterobacteriaceae*: OR: 0.111, 95% CI: 0.026–0.477, *p* = 0.003, P_FDR_ = 0.016; genus *Actinomyces*: OR: 0.377, 95% CI: 0.152–0.937, *p* = 0.036, P_FDR_ = 0.036; genus *Parabacteroides*: OR: 0.166, 95% CI: 0.037–0.752, *p* = 0.020, P_FDR_ = 0.025; *genus Victivallis*: OR: 0.509, 95% CI: 0.302–0.857, *p* = 0.011, P_FDR_ = 0.024; genus *Eubacterium (eligens group)*: OR: 0.130, 95% CI: 0.034–0.495, *p* = 0.003, P_FDR_ = 0.016) were negatively correlated with MG; four microbial taxa (genus *Ruminococcaceae UCG005*: OR: 2.450, 95% CI: 1.072–5.598, *p* = 0.034, P_FDR_ = 0.036; genus *Holdemania*: OR: 2.437, 95% CI: 1.215–4.888, *p* = 0.012, P_FDR_ = 0.024; genus *Lachnoclostridium*: OR: 3.681, 95% CI: 1.288–10.521, *p* = 0.015, P_FDR_ = 0.025; genus *Eubacterium (ruminantium group)*: OR: 2.157, 95% CI: 1.211–3.843, *p* = 0.003, P_FDR_ = 0.016) were positively associated with MG. The results of all microbial taxa were significant (*p* < 0.05) (Figure 2 and Table 2). Results from the MR-weighted median analysis indicated that four microbial taxa (genus *Actinomyces*: OR: 0.276, 95% CI: 0.092–0.827, *p* = 0.022, P_FDR_ = 0.073; genus *Holdemania*: OR: 2.582, 95% CI: 1.021–6.529; genus *Victivallis*: OR: 0.397, 95% CI: 0.195–0.808, *p* = 0.045, P_FDR_ = 0.092; genus *Eubacterium (eligens group)*: OR: 0.110, 95% CI: 0.20–0.616, *p* = 0.012, P_FDR_ = 0.06) had a significant impact. The results of the MR-Egger analysis for the genus *Victivallis* are contrary to those of IVW-MR, yet not statistically significant. All other MR-Egger analyses and MR estimates of the weighted median were in agreement with the IVW-MR analysis but had no significance (*p* > 0.05). The results of analyses of all gut microbiota and the three autoimmune neurologic disorders have been included in the Supplementary Table S3.

### 3.3 Sensitivity analyses

**Figure 3:**
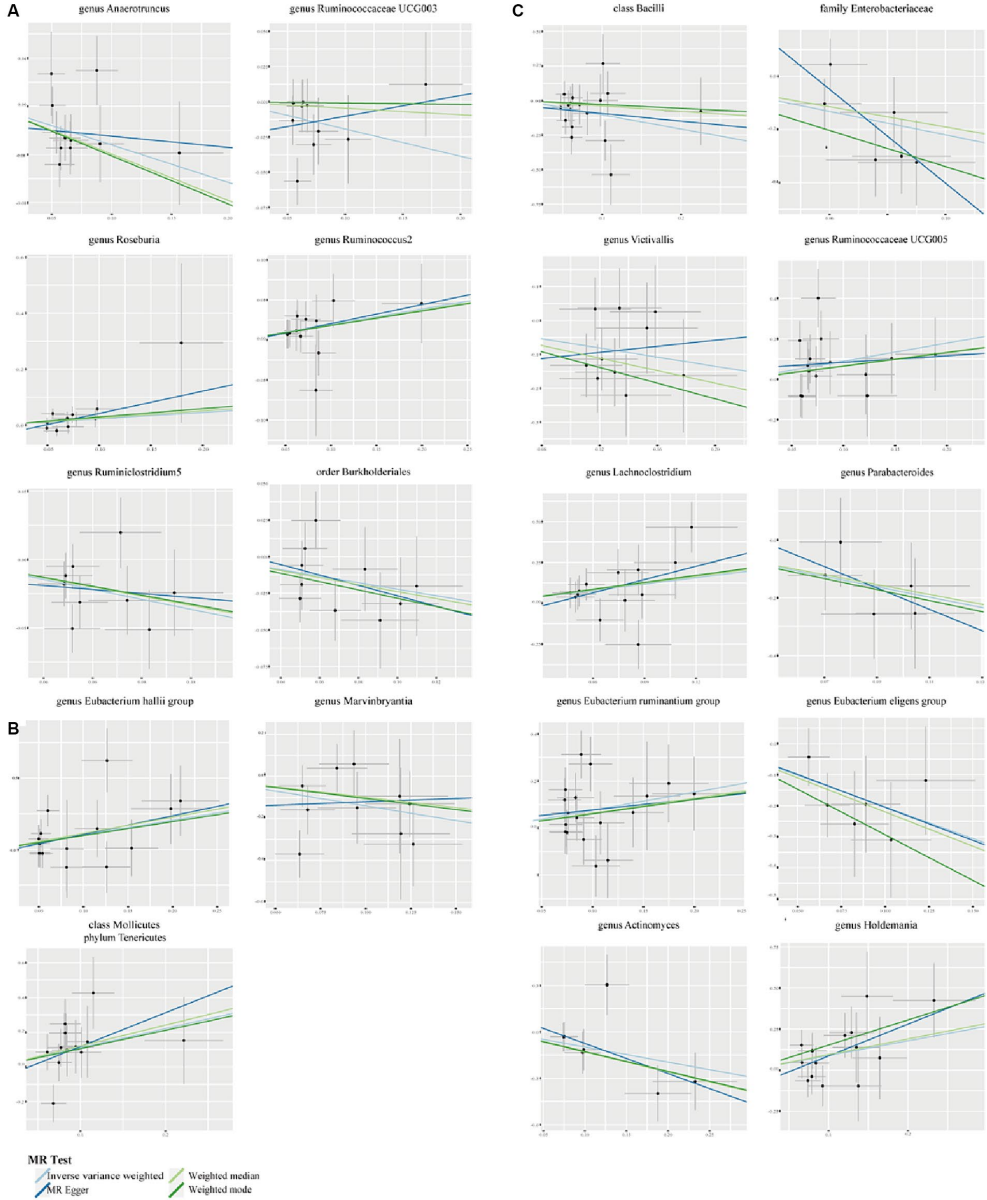
Scatter plots to show the associations of the gut microbiota against the three autoimmune neurologic disorders. **(A)** The effect of gut microbiota on MS. **(B)** The effect of gut microbiota on GBS. **(C)** The effect of gut microbiota on MG.

We employed Cochran’s Q test to detect heterogeneity and MR-PRESSO test and MR-Egger intercept to identify horizontal pleiotropy. The results of the test (*p* > 0.05) showed no evidence of heterogeneity or horizontal pleiotropy (Table 3). Moreover, four methods were implemented to measure the outcomes of MR analysis, and a scatter plot was formulated for the three diseases (Figure 3). Our main results were further substantiated through a leave-one-out analysis (Supplementary Figure S1).

**Table 3:** **Sensitivity analyses results** of gut microbiota and MS, GBS, MG.

Gut microbiota	Heterogeneity		Horizontal pleiotropy		MR-PRESSO
	Cochran’s Q	*p*-value	Egger intercept	*p*-value	Global Test$Pvalue
*Multiple sclerosis*					
Genus Ruminococcaceae UCG003	10.886	0.284	−0.025	0.319	0.312
Genus Ruminococcus2	5.400	0.910	−0.003	0.881	0.928
Genus Roseburia	10.347	0.411	−0.038	0.244	0.428
Genus Ruminiclostridium5	8.009	0.332	−0.013	0.705	0.483
Genus Anaerotruncus	10.813	0.372	−0.015	0.568	0.445
Order Burkholderiales	7.855	0.549	0.009	0.732	0.564
*Guillain-Barre Syndrome*					
Class Mollicutes	11.477	0.322	−0.076	0.601	0.432
Genus Marvinbryantia	10.675	0.299	−0.153	0.458	0.354
Genus Eubacterium (hallii group)	15.498	0.345	−0.025	0.750	0.358
Phylum Tenericutes	11.477	0.322	−0.076	0.601	0.424
*Myasthenia gravis*					
Class Bacilli	16.778	0.332	−0.037	0.685	0.406
Family Enterobacteriaceae	3.090	0.543	0.480	0.170	0.389
Genus Actinomyces	6.914	0.329	0.119	0.400	0.412
Genus Parabacteroides	1.249	0.870	0.167	0.715	0.901
Genus Ruminococcaceae UCG005	12.873	0.458	0.052	0.610	0.500
Genus Holdemania	10.624	0.642	−0.107	0.309	0.654
Genus Lachnoclostridium	13.594	0.327	−0.098	0.448	0.309
Genus Victivallis	6.803	0.658	−0.148	0.592	0.680
Genus Eubacterium (eligens group)	2.101	0.835	0.007	0.974	0.855
Genus Eubacterium (ruminantium group)	19.352	0.309	0.030	0.759	0.355

### 3.4 Reverse MR analysis

Taking into account cross-validation, we conducted reverse MR analyses for gut microbiota and three autoimmune neurologic disorders. The IVW-MR analysis findings indicated that there was no connection between the three autoimmune neurologic disorders, and the identified bacterial characteristics in a reverse causality fashion. The results of Cochran’s Q test indicated that there was no meaningful discrepancy among the incorporated independent variables. The MR-Egger intercept did not reveal any substantial horizontal pleiotropy (Table 4). Weighted Modes, Weighted Median Estimator, and MR-Egger results are presented in Supplementary Table S4.

**Table 4:** Reverse MR results of causal links between the three autoimmune neurologic disorders and gut microbiome.

Gut microbiome	Methods	SNPs	beta	*p*-value	OR	95%CI	steiger_test_pval	**Cochran’s Q**	**Q_pval**	Egger_intercept_pval
*Multiple sclerosis*										
Genus Anaerotruncus	IVW	118	−0.007	0.443	0.993	0.976–1.011	0	84.477	0.990	0.618
Genus Roseburia	IVW	116	−0.014	0.099	0.986	0.969–1.003	0	82.107	0.991	0.581
Genus Ruminiclostridium5	IVW	109	0.003	0.764	1.003	0.985–1.021	0	78.587	0.985	0.163
Genus Ruminococcaceae	IVW	115	0.005	0.631	1.005	0.986–1.024	0	79.648	0.994	0.435
Genus Ruminococcus2	IVW	117	0.000	0.959	1.000	0.981–1.018	0	89.900	0.965	0.990
Order Burkholderiales	IVW	115	−0.002	0.810	0.998	0.998–1.015	0	85.310	0.979	0.228
*Guillain-barre syndrome*										
Class Mollicutes	IVW	4	−0.002	0.902	0.998	0.970–1.027	0.558	3.204	0.361	0.252
Genus Eubacterium (hallii group)	IVW	6	−0.008	0.381	0.992	0.974–1.010	0.113	0.808	0.976	0.945
Genus Marvinbryantia	IVW	4	−0.015	0.256	0.985	0.959–1.011	0.267	0.295	0.961	0.757
Phylum Tenericutes	IVW	4	−0.002	0.902	0.998	0.970–1.027	0.558	3.204	0.361	0.252
*Myasthenia gravis*										
Class Bacilli	IVW	4	−0.003	0.783	0.997	0.974–1.020	0.274	1.406	0.704	0.889
Family Enterobacteriaceae	IVW	4	−0.005	0.718	0.995	0.968–1.023	0.218	1.004	0.800	0.871
Genus Actinomyces	IVW	4	0.007	0.722	1.006	0.969–1.046	0.188	0.891	0.828	0.595
Genus Eubacterium (eligens group)	IVW	3	−0.012	0.407	0.988	0.961–1.016	0.247	0.037	0.982	0.981
Genus Eubacterium (ruminantium group)	IVW	5	0.001	0.936	1.001	0.970–1.033	0.118	0.996	0.910	0.424
Genus Holdemania	IVW	4	0.019	0.214	1.020	0.989–1.051	0.311	0.106	0.991	0.933
Genus Lachnoclostridium	IVW	5	0.004	0.736	1.004	0.983–1.025	0.084	0.610	0.962	0.931
Genus Parabacteroides	IVW	5	0.007	0.533	1.007	0.985–1.030	0.419	2.811	0.590	0.617
Genus Ruminococcaceae UCG005	IVW	5	−0.010	0.371	0.990	0.969–1.012	0.202	0.835	0.934	0.509

## 4 Discussion

As far as we know, this is the first study to use published GWAS summary statistics to perform a two-sample MR analysis to explore the causal relationship between gut microbiota and autoimmune neurologic disorders. Through our analysis, we revealed causal relationships between certain microbial taxa and three common autoimmune neurologic disorders: MS, MG, and GBS.

Our research revealed that MS is associated with an increased presence of the genera *Ruminococcus2* and *Roseburia*. Study has shown that individuals with a higher abundance of *Ruminococcus* may be more likely to suffer from demyelinating optic neuritis, implying its involvement in autoimmune illnesses (Liu et al., 2023). Moreover, alterations in the abundance of *Ruminococcus* and *Roseburia* have been reported to be closely associated with the progression of Systemic Lupus Erythematosus (a typical autoimmune disease) (Vieira et al., 2021). On the other hand, we observed that a lower risk of MS was associated with higher levels of the genus *Ruminococcaceae UCG003*, the genus *Ruminiclostridium5*, the genus *Anaerotruncus*, and the order *Burkholderiales*. The genus *Ruminococcaceae* can increase the production of short-chain fatty acids (SCFAs), including acetic, propionic, and butyric acids, while reducing the release of inflammatory factors (Wang et al., 2023). Additionally, Ruminiclostridium5 and *Anaerotruncus* have been linked to CD8+ and CD4+ T cells, and their abundance is negatively related to the levels of inflammatory TNF-α, IL-1β, and IL-6 (Mao et al., 2023; Singh et al., 2023).

In the causal relationship between gut microbiota and GBS, we revealed that an increased risk of GBS was linked to a greater abundance of the phylum *Tenericutes*, class *Mollicutes*, and *genus Eubacterium (hallii group)*. Conversely, higher levels of the genus *Marvinbryantia* were associated with a lower risk of GBS. *Mycoplasma*, belonging to the phylum *Tenericutes* and class *Mollicutes*, is a widely known type of bacteria that causes mycoplasma pneumonia (Kuwahara et al., 2017; Gaspari et al., 2021). Previous research has established a strong correlation between *Mycoplasma* and the emergence of GBS, with many patients displaying a history of a mycoplasma infection before the onset of GBS. Our research has identified the genus *Marvinbryantia* in relation to GBS that has not been noted in prior studies. However, the exact mechanism of these bacteria leading to GBS remains poorly understood and warrants further study.

We discovered that the abundance of the genera *Ruminococcaceae UCG005*, *Holdemania*, *Eubacterium (ruminantium group)*, and *Lachnoclostridium* was associated with an increased risk of MG, while a high abundance of the class *Bacilli*, the family *Enterobacteriaceae*, and the genera *Parabacteroides*, *Victivallis*, and *Eubacterium (eligens group)* was related to a lower risk of MG. Qiu et al. observed that the genera *Clostridium*, *Lactobacillus*, and *Eubacterium* had significantly diminished in healthy individuals, with only one-third remaining (Qiu et al., 2018). A decrease in the concentration of *Clostridium* in the body leads to a decrease in the production of SCFAs, such as butyric acid and propionic acid, which affects the immune system. Studies have indicated that *lactobacilli* of the *Bacilli* class can stimulate the production of anti-inflammatory factors such as TGF-β, IL-10, and arginase 1 by regulating the dendritic cells (Rinaldi et al., 2019). Moreover, these same *lactobacilli* have been found to suppress AChR-reactive lymphocyte proliferation, anti-AChR-reactive IgG levels, and pro-inflammatory cytokines such as IL-6, IL-17, IFN-γ, and TNF-α (Chae et al., 2012; Teame et al., 2020). The *Enterobacteriaceae*, belonging to the phylum *Proteobacteria*, influences the production of propionic acid, thus impacting the immune response (Laursen, 2021). Our study has highlighted the significance of the genus *Holdemania* and genus *Parabacteroides* in the risk of MG development, a subject that has not been extensively discussed in earlier research.

The gut is an impressive network of nerve cells, with more than 100 million, second only to the central nervous system. The microbiota-gut-brain axis is a communication path between the gut and the central nervous system, which encompasses the central nervous system, the hypothalamic–pituitary–adrenal axis, the neuroimmune system, the autonomic sympathetic, the enteric and vagus nerves, and the intestinal microbiota and its metabolites (Cryan et al., 2019). SCFAs, the most studied microbial metabolites, have neuroactive properties that could be related to the communication along the microbiota-gut-brain axis, either directly or indirectly (Dalile et al., 2019; Zhang et al., 2023). It is interesting to note that certain SCFA-producing bacteria, such as *Ruminococcaceae UCG003*, *Ruminococcus2*, *Roseburia*, *Ruminiclostridium5*, *Lachnoclostridium*, and *Eubacterium*, are linked to autoimmune neurologic disorders. SCFAs are reported to play an anti-inflammatory role in the parenchymal mucosa, increase the number of Tregs, inhibit the differentiation of Th17 cells, and reduce the expression of inflammatory cytokines such as IL-17, IL-21, and IL-22 (Diller et al., 2016; Scott, 2021). A decrease in SCFA levels has been linked to autoimmune and inflammatory diseases, including rheumatoid arthritis, MS, colitis, type 1 diabetes, and rheumatoid arthritis (Schiweck et al., 2022). Although probiotics have been proven to reduce the symptoms of neuroimmune diseases and enhance functional performance, the exact ways in which they protect against these diseases are yet to be determined. Future studies should adopt a comprehensive approach, incorporating multi-omics analysis, to investigate the interplay between SCFAs, gut microbiota, and neuroimmune diseases, thus enhancing our understanding of the onset and development of these diseases.

This study has several advantages. By using the MR approach, we were able to reduce the potential for confounding factors that are often seen in epidemiological studies. Additionally, we sourced genetic variants from the most comprehensive GWAS meta-analysis for human gut microbiota composition, providing the accuracy of our MR analysis. Moreover, we covered three common autoimmune neurologic disorders. Finally, the causal associations identified in our MR analysis can be used to target particular gut bacteria for the prevention and treatment of autoimmune neurologic disorders.

Nevertheless, our study has certain limitations. Firstly, to reduce the risk of error due to mixed populations, we only used sample data from European ancestry, which limits the generality of the findings to all populations. Therefore, it is important for future studies to incorporate a more diverse range of populations in order to enhance the inclusivity of our conclusions. Secondly, the screening method may cause the exclusion of many SNPs of the gut microbiota at the IV selection stage, potentially preventing the discovery of crucial results. Thirdly, the pathogenesis of the specific gut microbiota in autoimmune neurologic disorders is still unclear, and more clinical and functional studies are still needed in the future to verify the accuracy of the results.

In conclusion, this is the first comprehensive assessment of the potential bidirectional relationship between gut microbiota and autoimmune neurologic disorders. However, further studies are needed to understand how gut microbiota affect autoimmune neurologic disorders and its precise biological mechanism.

## Data availability statement

The raw data supporting the conclusions of this article will be made available by the authors, without undue reservation.

## Author contributions

MZ: Formal analysis, Methodology, Writing – original draft. JF: Formal analysis, Methodology, Writing – original draft. CZ: Formal analysis, Writing – review & editing. QL: Funding acquisition, Methodology, Writing – review & editing. JZ: Methodology, Validation, Writing – review & editing.

## Footnotes

^1^ https://mibiogen.gcc.rug.nl
