# Supplementary material for: Gut microbiota and autoimmune neurologic disorders: a two-sample bidirectional Mendelian randomization study
Source: Front Microbiol. 2024 Apr 24;15:1337632. doi: 10.3389/fmicb.2024.1337632 (PMC11076763; doi:10.3389/fmicb.2024.1337632)
Supplement: Supplementary file 1 [file Table_1.DOCX]

***Supplementary Material***

**Gut microbiota and three autoimmune neurologic disorders: a two-sample bidirectional Mendelian randomization study**

**Mengyuan Zhang^1, 2, 3, 4, 5, 6#^, Jie Fang^1, 2, 3, 4, 5, 6#^, Chamou Zheng^7^, Qing Lin^1, 2, 3, 4, 5, 6*^, Jiawei Zhang^1, 2, 3, 4, 5, 6*^**

^1^Department of Neurology and Department of Neuroscience, The First Affiliated Hospital of Xiamen University, School of Medicine, Xiamen University, Xiamen, China.

^2^Fujian Key Laboratory of Brain Tumors Diagnosis and Precision Treatment, Xiamen, China.

^3^Xiamen Key Laboratory of Brain Center, Xiamen, China, Xiamen, China.

^4^Xiamen Medical Quality Control Center for Neurology, Xiamen, China.

^5^Fujian Provincial Clinical Research Center for Brain Diseases, Xiamen, China.

^6^Xiamen Clinical Research Center for Neurological Diseases, Xiamen, China.

^7^ Department of Neonatology, The First Affiliated Hospital of Xiamen University, Xiamen, China.

* Corresponding authors:

Jiawei Zhang, E-mail: zhangjiawei8102@163.com

Qing Lin, E-mail: linqing2005602@aliyun.com

\# Contributed equally.

**Table S1. Single-nucleotide polymorphisms (SNPs) associated with gut microbiome (P < 1×10^-5^).**

| **Classification** | **Exposure** | **SNP** | **Effect allele** | **Other allele** | **beta** | **SE** | **P** | **F** |
| --- | --- | --- | --- | --- | --- | --- | --- | --- |
| class | Actinobacteria | rs182549 | T | C | 0.111 | 0.012 | 3.79E-20 | 85.377 |
| class | Actinobacteria | rs4988235 | A | G | 0.110 | 0.012 | 1.50E-19 | 82.686 |
| class | Actinobacteria | rs6730157 | A | G | 0.108 | 0.012 | 2.43E-19 | 81.536 |
| class | Actinobacteria | rs6754311 | T | C | 0.108 | 0.012 | 4.18E-19 | 80.478 |
| class | Actinobacteria | rs7570971 | C | A | 0.107 | 0.012 | 2.56E-19 | 80.288 |
| class | Actinobacteria | rs1446585 | A | G | 0.109 | 0.012 | 7.71E-19 | 80.101 |
| class | Actinobacteria | rs1375131 | T | C | 0.106 | 0.012 | 9.16E-19 | 78.707 |
| class | Actinobacteria | rs12465802 | G | A | 0.106 | 0.012 | 8.79E-19 | 78.547 |
| class | Actinobacteria | rs687670 | T | C | 0.106 | 0.012 | 2.45E-17 | 73.562 |
| class | Actinobacteria | rs36084664 | A | G | -0.110 | 0.013 | 2.80E-19 | 73.503 |
| class | Actinobacteria | rs13404551 | T | C | 0.104 | 0.012 | 9.54E-17 | 71.497 |
| class | Actinobacteria | rs309134 | G | A | 0.104 | 0.012 | 3.71E-16 | 68.849 |
| class | Actinobacteria | rs309160 | C | T | 0.102 | 0.012 | 3.41E-16 | 68.751 |
| class | Actinobacteria | rs309168 | C | T | 0.102 | 0.012 | 4.88E-16 | 68.013 |
| class | Actinobacteria | rs218174 | A | G | 0.102 | 0.012 | 5.95E-16 | 67.950 |
| class | Actinobacteria | rs6759321 | G | T | 0.099 | 0.012 | 7.16E-16 | 66.697 |
| class | Actinobacteria | rs309180 | A | G | 0.100 | 0.012 | 1.26E-15 | 66.197 |
| class | Actinobacteria | rs13023281 | T | C | -0.104 | 0.013 | 1.58E-17 | 66.035 |
| class | Actinobacteria | rs12615624 | A | G | 0.101 | 0.012 | 1.57E-15 | 65.996 |
| class | Actinobacteria | rs632632 | T | C | 0.099 | 0.012 | 2.20E-15 | 65.262 |
| class | Actinobacteria | rs1561277 | C | A | 0.097 | 0.012 | 2.15E-15 | 64.682 |
| class | Actinobacteria | rs309125 | C | T | 0.098 | 0.012 | 3.91E-15 | 63.942 |
| class | Actinobacteria | rs309164 | T | C | 0.100 | 0.012 | 4.14E-15 | 63.872 |
| class | Actinobacteria | rs4988183 | T | C | 0.099 | 0.013 | 9.46E-15 | 62.498 |
| class | Actinobacteria | rs309149 | C | T | 0.098 | 0.012 | 1.20E-14 | 61.649 |
| class | Actinobacteria | rs309137 | T | C | 0.096 | 0.012 | 1.18E-14 | 61.553 |
| class | Actinobacteria | rs4954490 | A | G | 0.096 | 0.012 | 3.49E-14 | 59.725 |
| class | Actinobacteria | rs2236783 | A | G | 0.096 | 0.012 | 4.86E-14 | 59.258 |
| class | Actinobacteria | rs12620033 | G | C | 0.095 | 0.012 | 6.00E-14 | 58.827 |
| class | Actinobacteria | rs6716536 | C | T | 0.092 | 0.012 | 5.27E-14 | 58.005 |
| class | Actinobacteria | rs11681248 | T | C | 0.089 | 0.012 | 1.25E-13 | 56.250 |
| class | Actinobacteria | rs3754686 | C | T | 0.093 | 0.012 | 1.86E-13 | 56.204 |
| class | Actinobacteria | rs745500 | A | G | 0.093 | 0.012 | 2.42E-13 | 55.918 |
| class | Actinobacteria | rs6719488 | T | G | 0.092 | 0.012 | 3.10E-13 | 55.376 |
| class | Actinobacteria | rs892715 | C | T | 0.092 | 0.012 | 3.22E-13 | 55.281 |
| class | Actinobacteria | rs6742283 | G | A | 0.092 | 0.012 | 3.01E-13 | 55.265 |
| class | Actinobacteria | rs3739020 | T | G | 0.094 | 0.013 | 3.36E-13 | 55.236 |
| class | Actinobacteria | rs2322660 | T | C | 0.092 | 0.012 | 3.40E-13 | 55.075 |
| class | Actinobacteria | rs12988076 | A | C | 0.092 | 0.012 | 3.79E-13 | 54.979 |
| class | Actinobacteria | rs3769005 | G | C | 0.092 | 0.012 | 3.39E-13 | 54.873 |
| class | Actinobacteria | rs748841 | T | C | 0.092 | 0.012 | 4.03E-13 | 54.772 |
| class | Actinobacteria | rs17261772 | T | C | 0.092 | 0.012 | 4.30E-13 | 54.370 |
| class | Actinobacteria | rs2322659 | C | T | 0.093 | 0.013 | 5.97E-13 | 53.930 |
| class | Actinobacteria | rs1470457 | A | G | 0.093 | 0.013 | 7.35E-13 | 53.747 |
| class | Actinobacteria | rs12475139 | T | A | 0.089 | 0.012 | 7.64E-13 | 52.701 |
| class | Actinobacteria | rs2164210 | C | T | 0.090 | 0.012 | 1.12E-12 | 52.586 |
| class | Actinobacteria | rs313519 | A | C | 0.091 | 0.013 | 1.07E-12 | 52.149 |
| class | Actinobacteria | rs313518 | C | T | 0.091 | 0.013 | 1.22E-12 | 52.012 |
| class | Actinobacteria | rs10928546 | T | C | 0.091 | 0.013 | 1.62E-12 | 51.764 |
| class | Actinobacteria | rs2082729 | T | C | 0.091 | 0.013 | 2.16E-12 | 51.329 |
| class | Actinobacteria | rs749873 | T | C | 0.085 | 0.012 | 1.51E-12 | 51.275 |
| class | Actinobacteria | rs71348715 | T | G | 0.090 | 0.013 | 2.18E-12 | 51.233 |
| class | Actinobacteria | rs11684545 | T | G | 0.090 | 0.013 | 2.26E-12 | 51.042 |
| class | Actinobacteria | rs71348714 | A | G | 0.090 | 0.013 | 1.94E-12 | 51.027 |
| class | Actinobacteria | rs313517 | A | G | 0.090 | 0.013 | 1.97E-12 | 51.006 |
| class | Actinobacteria | rs28453840 | A | G | 0.090 | 0.013 | 2.50E-12 | 50.880 |
| class | Actinobacteria | rs313526 | C | T | 0.090 | 0.013 | 2.15E-12 | 50.864 |
| class | Actinobacteria | rs313528 | G | A | 0.090 | 0.013 | 2.21E-12 | 50.781 |
| class | Actinobacteria | rs4954280 | A | G | 0.090 | 0.013 | 2.26E-12 | 50.744 |
| class | Actinobacteria | rs2034276 | A | T | 0.090 | 0.013 | 2.21E-12 | 50.731 |
| class | Actinobacteria | rs1438305 | G | C | 0.090 | 0.013 | 2.92E-12 | 50.655 |
| class | Actinobacteria | rs1011361 | A | G | 0.090 | 0.013 | 3.03E-12 | 50.543 |
| class | Actinobacteria | rs1446584 | A | G | 0.089 | 0.013 | 2.48E-12 | 50.534 |
| class | Actinobacteria | rs10202489 | A | G | 0.090 | 0.013 | 3.05E-12 | 50.505 |
| class | Actinobacteria | rs313523 | G | A | 0.089 | 0.013 | 2.51E-12 | 50.468 |
| class | Actinobacteria | rs313522 | T | A | 0.089 | 0.013 | 2.59E-12 | 50.428 |
| class | Actinobacteria | rs12998016 | T | C | 0.090 | 0.013 | 3.55E-12 | 50.192 |
| class | Actinobacteria | rs1438304 | G | A | 0.089 | 0.013 | 3.70E-12 | 50.175 |
| class | Actinobacteria | rs10195620 | T | A | 0.089 | 0.013 | 3.61E-12 | 50.124 |
| class | Actinobacteria | rs656326 | A | C | 0.089 | 0.013 | 3.03E-12 | 50.102 |
| class | Actinobacteria | rs1438307 | T | G | 0.089 | 0.013 | 3.70E-12 | 50.102 |
| class | Actinobacteria | rs6720287 | A | G | 0.089 | 0.013 | 3.04E-12 | 50.084 |
| class | Actinobacteria | rs313524 | C | T | 0.089 | 0.013 | 3.16E-12 | 50.035 |
| class | Actinobacteria | rs6715856 | T | C | 0.089 | 0.013 | 3.37E-12 | 49.840 |
| class | Actinobacteria | rs6755383 | C | T | 0.089 | 0.013 | 3.45E-12 | 49.781 |
| class | Actinobacteria | rs6430589 | C | G | 0.089 | 0.013 | 3.74E-12 | 49.712 |
| class | Actinobacteria | rs7355359 | T | C | 0.089 | 0.013 | 3.78E-12 | 49.708 |
| class | Actinobacteria | rs10192827 | A | G | 0.089 | 0.013 | 5.09E-12 | 49.694 |
| class | Actinobacteria | rs3213889 | G | A | 0.089 | 0.013 | 4.86E-12 | 49.675 |
| class | Actinobacteria | rs12998387 | G | A | 0.089 | 0.013 | 4.84E-12 | 49.626 |
| class | Actinobacteria | rs10173394 | T | C | 0.089 | 0.013 | 5.02E-12 | 49.584 |
| class | Actinobacteria | rs4954276 | A | G | 0.089 | 0.013 | 4.56E-12 | 49.532 |
| class | Actinobacteria | rs13412397 | A | G | 0.089 | 0.013 | 4.80E-12 | 49.528 |
| class | Actinobacteria | rs10928542 | G | A | 0.088 | 0.013 | 4.39E-12 | 49.307 |
| class | Actinobacteria | rs2011946 | A | C | 0.084 | 0.012 | 4.82E-12 | 49.280 |
| class | Actinobacteria | rs7608045 | T | C | 0.088 | 0.013 | 4.58E-12 | 49.237 |
| class | Actinobacteria | rs12994270 | A | T | 0.088 | 0.013 | 6.73E-12 | 49.098 |
| class | Actinobacteria | rs4954279 | G | A | 0.088 | 0.013 | 5.01E-12 | 49.049 |
| class | Actinobacteria | rs11679508 | T | A | 0.088 | 0.013 | 6.11E-12 | 49.037 |
| class | Actinobacteria | rs12619365 | C | T | 0.088 | 0.013 | 5.05E-12 | 48.854 |
| class | Actinobacteria | rs13413639 | C | A | 0.088 | 0.013 | 6.40E-12 | 48.700 |
| class | Actinobacteria | rs12469709 | T | C | 0.088 | 0.013 | 7.78E-12 | 48.609 |
| class | Actinobacteria | rs1869829 | A | G | 0.087 | 0.012 | 5.20E-12 | 48.446 |
| class | Actinobacteria | rs1374329 | A | G | 0.088 | 0.013 | 1.29E-11 | 47.280 |
| class | Actinobacteria | rs9287442 | G | A | 0.089 | 0.013 | 2.26E-11 | 46.673 |
| class | Actinobacteria | rs6760329 | G | A | 0.089 | 0.013 | 2.09E-11 | 46.512 |
| class | Actinobacteria | rs10928545 | G | A | 0.088 | 0.013 | 2.73E-11 | 46.493 |
| class | Actinobacteria | rs12616520 | T | A | 0.088 | 0.013 | 2.96E-11 | 46.127 |
| class | Actinobacteria | rs10188066 | A | G | 0.088 | 0.013 | 2.62E-11 | 46.085 |
| class | Actinobacteria | rs2304599 | G | C | 0.088 | 0.013 | 2.88E-11 | 46.079 |
| class | Actinobacteria | rs11693502 | T | C | 0.081 | 0.012 | 1.96E-11 | 46.077 |
| class | Actinobacteria | rs10187402 | C | G | 0.085 | 0.013 | 1.65E-11 | 45.684 |
| class | Actinobacteria | rs2680880 | T | A | 0.077 | 0.012 | 4.86E-11 | 45.316 |
| class | Actinobacteria | rs6714750 | A | G | 0.085 | 0.013 | 4.33E-11 | 45.267 |
| class | Actinobacteria | rs11679933 | T | G | 0.080 | 0.012 | 3.67E-11 | 45.100 |
| class | Actinobacteria | rs2278544 | G | A | 0.087 | 0.013 | 5.15E-11 | 45.047 |
| class | Actinobacteria | rs1030764 | T | C | 0.087 | 0.013 | 6.07E-11 | 44.601 |
| class | Actinobacteria | rs3820794 | T | C | 0.086 | 0.013 | 7.33E-11 | 44.233 |
| class | Actinobacteria | rs62160860 | G | C | 0.113 | 0.017 | 2.68E-11 | 44.120 |
| class | Actinobacteria | rs932206 | T | C | 0.076 | 0.012 | 5.45E-11 | 43.500 |
| class | Actinobacteria | rs935613 | C | T | 0.083 | 0.013 | 6.41E-11 | 43.450 |
| class | Actinobacteria | rs1375132 | A | G | 0.082 | 0.012 | 7.46E-11 | 43.316 |
| class | Actinobacteria | rs7587285 | T | C | 0.110 | 0.017 | 3.94E-11 | 42.973 |
| class | Actinobacteria | rs2278683 | A | C | 0.110 | 0.017 | 4.71E-11 | 42.739 |
| class | Actinobacteria | rs4954551 | G | A | 0.110 | 0.017 | 4.36E-11 | 42.680 |
| class | Actinobacteria | rs11893318 | T | C | 0.109 | 0.017 | 5.76E-11 | 42.292 |
| class | Actinobacteria | rs6747073 | T | C | 0.081 | 0.012 | 1.55E-10 | 41.787 |
| class | Actinobacteria | rs6704561 | C | T | 0.078 | 0.012 | 3.07E-10 | 40.552 |
| class | Actinobacteria | rs2016636 | G | A | 0.080 | 0.013 | 3.27E-10 | 40.387 |
| class | Actinobacteria | rs1900741 | C | T | 0.086 | 0.014 | 8.46E-10 | 39.640 |
| class | Actinobacteria | rs2874739 | C | T | 0.097 | 0.016 | 2.66E-10 | 37.566 |
| class | Actinobacteria | rs12328663 | T | C | 0.097 | 0.016 | 2.41E-10 | 37.382 |
| class | Actinobacteria | rs4954567 | G | T | 0.073 | 0.012 | 1.22E-09 | 37.248 |
| class | Actinobacteria | rs4954513 | G | A | 0.106 | 0.017 | 1.94E-09 | 37.215 |
| class | Actinobacteria | rs10193348 | A | G | 0.096 | 0.016 | 3.07E-10 | 37.176 |
| class | Actinobacteria | rs13389481 | C | A | 0.098 | 0.016 | 3.88E-10 | 36.928 |
| class | Actinobacteria | rs10167901 | C | T | 0.096 | 0.016 | 4.18E-10 | 36.849 |
| class | Actinobacteria | rs3768998 | G | T | 0.105 | 0.017 | 1.09E-09 | 36.765 |
| class | Actinobacteria | rs10167809 | C | T | 0.096 | 0.016 | 4.44E-10 | 36.698 |
| class | Actinobacteria | rs4953948 | T | G | 0.096 | 0.016 | 4.45E-10 | 36.689 |
| class | Actinobacteria | rs4954216 | G | A | 0.096 | 0.016 | 4.60E-10 | 36.639 |
| class | Actinobacteria | rs3769002 | T | C | 0.105 | 0.017 | 2.46E-09 | 36.578 |
| class | Actinobacteria | rs1401687 | G | C | 0.073 | 0.012 | 1.74E-09 | 36.563 |
| class | Actinobacteria | rs62168942 | T | C | 0.096 | 0.016 | 5.19E-10 | 36.519 |
| class | Actinobacteria | rs7608980 | G | A | 0.105 | 0.017 | 3.25E-09 | 36.513 |
| class | Actinobacteria | rs3213871 | C | T | 0.106 | 0.017 | 2.34E-09 | 36.512 |
| class | Actinobacteria | rs10167884 | C | T | 0.096 | 0.016 | 4.92E-10 | 36.512 |
| class | Actinobacteria | rs56263017 | T | C | 0.105 | 0.017 | 2.52E-09 | 36.501 |
| class | Actinobacteria | rs7422031 | C | T | 0.095 | 0.016 | 4.81E-10 | 36.487 |
| class | Actinobacteria | rs4954215 | G | A | 0.096 | 0.016 | 4.92E-10 | 36.485 |
| class | Actinobacteria | rs10182750 | T | C | 0.096 | 0.016 | 5.26E-10 | 36.442 |
| class | Actinobacteria | rs6739713 | A | G | 0.077 | 0.013 | 2.97E-09 | 36.408 |
| class | Actinobacteria | rs16832138 | G | A | 0.105 | 0.017 | 3.50E-09 | 36.359 |
| class | Actinobacteria | rs16832275 | C | G | 0.101 | 0.017 | 2.05E-09 | 36.335 |
| class | Actinobacteria | rs4953947 | A | C | 0.096 | 0.016 | 5.37E-10 | 36.318 |
| class | Actinobacteria | rs10179271 | T | C | 0.072 | 0.012 | 1.95E-09 | 36.314 |
| class | Actinobacteria | rs13409400 | A | C | 0.095 | 0.016 | 5.81E-10 | 36.285 |
| class | Actinobacteria | rs62168941 | C | T | 0.095 | 0.016 | 6.05E-10 | 36.258 |
| class | Actinobacteria | rs7581382 | T | C | -0.085 | 0.014 | 6.88E-11 | 36.242 |
| class | Actinobacteria | rs9941547 | G | A | 0.072 | 0.012 | 2.26E-09 | 36.031 |
| class | Actinobacteria | rs3754692 | T | C | 0.077 | 0.013 | 3.68E-09 | 36.025 |
| class | Actinobacteria | rs10208624 | C | T | 0.095 | 0.016 | 4.46E-10 | 36.025 |
| class | Actinobacteria | rs7568525 | G | A | 0.095 | 0.016 | 4.39E-10 | 36.008 |
| class | Actinobacteria | rs10186594 | A | G | 0.095 | 0.016 | 6.87E-10 | 35.978 |
| class | Actinobacteria | rs4954217 | C | T | 0.095 | 0.016 | 6.85E-10 | 35.956 |
| class | Actinobacteria | rs13413872 | G | T | 0.095 | 0.016 | 6.77E-10 | 35.945 |
| class | Actinobacteria | rs13402112 | A | C | 0.095 | 0.016 | 6.77E-10 | 35.945 |
| class | Actinobacteria | rs7573600 | G | C | 0.095 | 0.016 | 5.15E-10 | 35.908 |
| class | Actinobacteria | rs56903548 | C | T | 0.095 | 0.016 | 6.24E-10 | 35.895 |
| class | Actinobacteria | rs4954214 | C | T | 0.095 | 0.016 | 6.86E-10 | 35.824 |
| class | Actinobacteria | rs7601696 | C | A | 0.095 | 0.016 | 7.76E-10 | 35.780 |
| class | Actinobacteria | rs921859 | A | G | 0.095 | 0.016 | 7.46E-10 | 35.770 |
| class | Actinobacteria | rs7577258 | G | A | 0.095 | 0.016 | 7.95E-10 | 35.724 |
| class | Actinobacteria | rs11898045 | T | C | 0.094 | 0.016 | 5.34E-10 | 35.703 |
| class | Actinobacteria | rs2017831 | C | T | 0.095 | 0.016 | 7.91E-10 | 35.681 |
| class | Actinobacteria | rs6760553 | A | G | 0.095 | 0.016 | 7.41E-10 | 35.659 |
| class | Actinobacteria | rs7565268 | T | C | 0.095 | 0.016 | 8.29E-10 | 35.653 |
| class | Actinobacteria | rs4954568 | T | A | 0.072 | 0.012 | 2.79E-09 | 35.619 |
| class | Actinobacteria | rs56174523 | A | C | 0.095 | 0.016 | 8.27E-10 | 35.556 |
| class | Actinobacteria | rs309132 | C | G | 0.102 | 0.017 | 5.74E-09 | 35.553 |
| class | Actinobacteria | rs58426504 | T | G | 0.094 | 0.016 | 6.12E-10 | 35.442 |
| class | Actinobacteria | rs58697996 | G | A | 0.094 | 0.016 | 6.37E-10 | 35.432 |
| class | Actinobacteria | rs61087958 | C | T | 0.094 | 0.016 | 7.35E-10 | 35.375 |
| class | Actinobacteria | rs16831498 | G | C | 0.095 | 0.016 | 8.35E-10 | 35.353 |
| class | Actinobacteria | rs4953945 | C | G | 0.095 | 0.016 | 1.12E-09 | 35.329 |
| class | Actinobacteria | rs4953946 | T | G | 0.094 | 0.016 | 8.54E-10 | 35.329 |
| class | Actinobacteria | rs56067372 | C | T | 0.104 | 0.017 | 5.97E-09 | 35.302 |
| class | Actinobacteria | rs7601683 | C | T | 0.094 | 0.016 | 1.01E-09 | 35.284 |
| class | Actinobacteria | rs11888951 | T | C | 0.094 | 0.016 | 9.10E-10 | 35.249 |
| class | Actinobacteria | rs7565149 | T | A | 0.094 | 0.016 | 1.04E-09 | 35.192 |
| class | Actinobacteria | rs60350840 | A | G | 0.094 | 0.016 | 1.08E-09 | 35.185 |
| class | Actinobacteria | rs10174944 | C | T | 0.094 | 0.016 | 1.02E-09 | 35.177 |
| class | Actinobacteria | rs62168934 | A | G | 0.094 | 0.016 | 1.03E-09 | 35.134 |
| class | Actinobacteria | rs7603805 | A | G | 0.094 | 0.016 | 9.44E-10 | 35.113 |
| class | Actinobacteria | rs309126 | T | C | 0.097 | 0.016 | 6.28E-09 | 35.028 |
| class | Actinobacteria | rs6715785 | G | A | 0.071 | 0.012 | 3.88E-09 | 34.966 |
| class | Actinobacteria | rs11884903 | T | C | 0.094 | 0.016 | 1.05E-09 | 34.852 |
| class | Actinobacteria | rs4954220 | C | A | 0.093 | 0.016 | 8.71E-10 | 34.786 |
| class | Actinobacteria | rs4954569 | C | T | 0.071 | 0.012 | 4.17E-09 | 34.781 |
| class | Actinobacteria | rs6752519 | C | T | 0.102 | 0.017 | 6.07E-09 | 34.716 |
| class | Actinobacteria | rs6741481 | A | G | 0.093 | 0.016 | 1.08E-09 | 34.632 |
| class | Actinobacteria | rs1551497 | T | C | 0.093 | 0.016 | 1.54E-09 | 34.463 |
| class | Actinobacteria | rs3754690 | C | T | 0.106 | 0.018 | 6.66E-09 | 34.462 |
| class | Actinobacteria | rs2290518 | G | A | 0.093 | 0.016 | 1.22E-09 | 34.427 |
| class | Actinobacteria | rs2164332 | G | C | 0.098 | 0.017 | 4.86E-09 | 34.398 |
| class | Actinobacteria | rs16832156 | G | A | 0.098 | 0.017 | 6.72E-09 | 34.224 |
| class | Actinobacteria | rs57829242 | A | G | 0.094 | 0.016 | 1.61E-09 | 34.209 |
| class | Actinobacteria | rs10209482 | A | C | 0.094 | 0.016 | 1.56E-09 | 34.205 |
| class | Actinobacteria | rs58327215 | G | C | 0.093 | 0.016 | 1.26E-09 | 34.197 |
| class | Actinobacteria | rs4954212 | A | G | 0.093 | 0.016 | 1.98E-09 | 34.181 |
| class | Actinobacteria | rs925966 | G | C | 0.071 | 0.012 | 8.72E-09 | 34.074 |
| class | Actinobacteria | rs6430561 | C | T | 0.092 | 0.016 | 1.44E-09 | 34.027 |
| class | Actinobacteria | rs2164331 | C | T | 0.102 | 0.017 | 7.56E-09 | 33.987 |
| class | Actinobacteria | rs11890844 | A | G | 0.093 | 0.016 | 1.87E-09 | 33.986 |
| class | Actinobacteria | rs2322725 | T | C | 0.098 | 0.017 | 5.81E-09 | 33.968 |
| class | Actinobacteria | rs4954392 | A | G | 0.070 | 0.012 | 6.15E-09 | 33.966 |
| class | Actinobacteria | rs13402101 | T | A | 0.092 | 0.016 | 2.15E-09 | 33.823 |
| class | Actinobacteria | rs6430593 | G | A | 0.098 | 0.017 | 9.44E-09 | 33.803 |
| class | Actinobacteria | rs4346445 | A | C | 0.092 | 0.016 | 1.22E-09 | 33.774 |
| class | Actinobacteria | rs950176 | A | G | 0.070 | 0.012 | 7.48E-09 | 33.770 |
| class | Actinobacteria | rs13399376 | A | G | 0.092 | 0.016 | 2.23E-09 | 33.734 |
| class | Actinobacteria | rs55634455 | T | C | 0.105 | 0.018 | 8.85E-09 | 33.727 |
| class | Actinobacteria | rs12616955 | C | T | 0.070 | 0.012 | 7.08E-09 | 33.692 |
| class | Actinobacteria | rs2874834 | A | G | 0.074 | 0.013 | 1.18E-08 | 33.674 |
| class | Actinobacteria | rs13410870 | G | A | 0.092 | 0.016 | 1.70E-09 | 33.661 |
| class | Actinobacteria | rs6709763 | C | A | 0.092 | 0.016 | 2.41E-09 | 33.579 |
| class | Actinobacteria | rs4954242 | A | G | 0.093 | 0.016 | 3.02E-09 | 33.500 |
| class | Actinobacteria | rs12478902 | T | C | 0.099 | 0.017 | 7.65E-09 | 33.499 |
| class | Actinobacteria | rs16832200 | T | C | 0.097 | 0.017 | 7.57E-09 | 33.484 |
| class | Actinobacteria | rs16831264 | G | C | 0.091 | 0.016 | 2.52E-09 | 33.440 |
| class | Actinobacteria | rs6721838 | A | G | 0.092 | 0.016 | 1.94E-09 | 33.360 |
| class | Actinobacteria | rs78141345 | G | A | 0.104 | 0.018 | 1.10E-08 | 33.334 |
| class | Actinobacteria | rs4954566 | C | T | 0.069 | 0.012 | 8.63E-09 | 33.306 |
| class | Actinobacteria | rs7371606 | A | G | 0.070 | 0.012 | 1.31E-08 | 33.276 |
| class | Actinobacteria | rs60433564 | A | C | 0.119 | 0.021 | 4.41E-09 | 33.246 |
| class | Actinobacteria | rs13427494 | G | A | 0.092 | 0.016 | 2.70E-09 | 33.220 |
| class | Actinobacteria | rs6723108 | G | T | 0.067 | 0.012 | 2.42E-09 | 33.147 |
| class | Actinobacteria | rs6737635 | G | T | 0.091 | 0.016 | 3.18E-09 | 33.105 |
| class | Actinobacteria | rs78793148 | C | A | 0.108 | 0.019 | 1.73E-08 | 33.092 |
| class | Actinobacteria | rs74266324 | G | A | 0.083 | 0.014 | 7.63E-09 | 33.090 |
| class | Actinobacteria | rs6749119 | C | G | 0.091 | 0.016 | 2.26E-09 | 33.078 |
| class | Actinobacteria | rs10496736 | C | G | 0.119 | 0.021 | 4.66E-09 | 32.980 |
| class | Actinobacteria | rs4954564 | A | G | 0.069 | 0.012 | 1.12E-08 | 32.955 |
| class | Actinobacteria | rs11892750 | G | A | 0.091 | 0.016 | 3.89E-09 | 32.901 |
| class | Actinobacteria | rs17293519 | A | G | 0.091 | 0.016 | 2.76E-09 | 32.889 |
| class | Actinobacteria | rs1530559 | A | G | 0.065 | 0.011 | 7.55E-09 | 32.804 |
| class | Actinobacteria | rs4954572 | G | T | 0.069 | 0.012 | 1.07E-08 | 32.770 |
| class | Actinobacteria | rs4954226 | T | C | 0.092 | 0.016 | 3.04E-09 | 32.756 |
| class | Actinobacteria | rs10803550 | C | T | 0.069 | 0.012 | 1.12E-08 | 32.732 |
| class | Actinobacteria | rs11897204 | T | C | 0.090 | 0.016 | 2.80E-09 | 32.686 |
| class | Actinobacteria | rs1979033 | G | A | 0.065 | 0.011 | 3.26E-09 | 32.665 |
| class | Actinobacteria | rs6706466 | A | G | 0.090 | 0.016 | 2.78E-09 | 32.623 |
| class | Actinobacteria | rs4954396 | A | C | 0.069 | 0.012 | 1.16E-08 | 32.623 |
| class | Actinobacteria | rs953387 | A | C | 0.069 | 0.012 | 1.20E-08 | 32.558 |
| class | Actinobacteria | rs2102896 | T | C | 0.069 | 0.012 | 1.21E-08 | 32.522 |
| class | Actinobacteria | rs2056297 | G | T | 0.068 | 0.012 | 1.30E-08 | 32.519 |
| class | Actinobacteria | rs4954570 | G | C | 0.073 | 0.013 | 7.97E-09 | 32.461 |
| class | Actinobacteria | rs13408096 | G | T | 0.090 | 0.016 | 4.30E-09 | 32.457 |
| class | Actinobacteria | rs56369660 | A | G | 0.090 | 0.016 | 4.01E-09 | 32.414 |
| class | Actinobacteria | rs1123848 | C | T | 0.069 | 0.012 | 1.32E-08 | 32.365 |
| class | Actinobacteria | rs56211644 | T | C | 0.102 | 0.018 | 1.75E-08 | 32.306 |
| class | Actinobacteria | rs4954213 | G | T | 0.090 | 0.016 | 4.93E-09 | 32.169 |
| class | Actinobacteria | rs4954221 | G | A | 0.090 | 0.016 | 3.73E-09 | 32.098 |
| class | Actinobacteria | rs11903663 | A | G | 0.090 | 0.016 | 2.13E-09 | 32.081 |
| class | Actinobacteria | rs4640444 | T | C | 0.090 | 0.016 | 2.30E-09 | 32.028 |
| class | Actinobacteria | rs4645053 | T | A | 0.090 | 0.016 | 2.31E-09 | 32.020 |
| class | Actinobacteria | rs12233092 | G | A | 0.095 | 0.017 | 1.66E-08 | 31.949 |
| class | Actinobacteria | rs7597802 | A | T | 0.090 | 0.016 | 6.12E-09 | 31.933 |
| class | Actinobacteria | rs11900832 | C | T | 0.090 | 0.016 | 6.06E-09 | 31.913 |
| class | Actinobacteria | rs584226 | T | C | 0.096 | 0.017 | 2.84E-08 | 31.889 |
| class | Actinobacteria | rs6742013 | T | C | 0.090 | 0.016 | 5.53E-09 | 31.761 |
| class | Actinobacteria | rs1964776 | C | T | 0.068 | 0.012 | 1.98E-08 | 31.670 |
| class | Actinobacteria | rs11889843 | G | A | 0.089 | 0.016 | 6.83E-09 | 31.642 |
| class | Actinobacteria | rs12473760 | C | A | 0.068 | 0.012 | 1.92E-08 | 31.597 |
| class | Actinobacteria | rs7563997 | T | A | 0.089 | 0.016 | 6.97E-09 | 31.596 |
| class | Actinobacteria | rs7565053 | A | T | 0.121 | 0.022 | 1.30E-08 | 31.589 |
| class | Actinobacteria | rs10928558 | T | C | 0.069 | 0.012 | 2.01E-08 | 31.585 |
| class | Actinobacteria | rs57543738 | T | C | 0.102 | 0.018 | 2.20E-08 | 31.583 |
| class | Actinobacteria | rs7605229 | C | T | 0.090 | 0.016 | 6.47E-09 | 31.568 |
| class | Actinobacteria | rs60376570 | G | A | 0.101 | 0.018 | 2.21E-08 | 31.542 |
| class | Actinobacteria | rs4494812 | A | G | 0.089 | 0.016 | 3.20E-09 | 31.530 |
| class | Actinobacteria | rs4954633 | C | T | 0.115 | 0.021 | 6.93E-09 | 31.494 |
| class | Actinobacteria | rs10928529 | G | A | 0.089 | 0.016 | 7.64E-09 | 31.475 |
| class | Actinobacteria | rs4954397 | A | G | 0.068 | 0.012 | 2.11E-08 | 31.460 |
| class | Actinobacteria | rs6727889 | C | T | 0.090 | 0.016 | 8.15E-09 | 31.447 |
| class | Actinobacteria | rs2305248 | A | G | 0.089 | 0.016 | 5.42E-09 | 31.395 |
| class | Actinobacteria | rs16855656 | A | G | 0.092 | 0.016 | 3.48E-08 | 31.271 |
| class | Actinobacteria | rs4954577 | A | G | 0.068 | 0.012 | 2.42E-08 | 31.222 |
| class | Actinobacteria | rs16833335 | G | A | 0.067 | 0.012 | 2.76E-08 | 31.008 |
| class | Actinobacteria | rs1519525 | C | T | 0.067 | 0.012 | 3.02E-08 | 30.944 |
| class | Actinobacteria | rs62170081 | A | G | 0.115 | 0.021 | 1.56E-08 | 30.895 |
| class | Actinobacteria | rs72972104 | G | A | 0.114 | 0.021 | 1.03E-08 | 30.737 |
| class | Actinobacteria | rs4954430 | C | T | 0.115 | 0.021 | 1.38E-08 | 30.713 |
| class | Actinobacteria | rs2874874 | A | C | 0.100 | 0.018 | 3.61E-08 | 30.697 |
| class | Actinobacteria | rs11888521 | G | C | 0.088 | 0.016 | 4.97E-09 | 30.690 |
| class | Actinobacteria | rs35215526 | G | A | 0.104 | 0.019 | 5.58E-08 | 30.513 |
| class | Actinobacteria | rs74697610 | A | T | 0.110 | 0.020 | 2.52E-08 | 30.481 |
| class | Actinobacteria | rs9677760 | A | T | 0.076 | 0.014 | 7.71E-08 | 30.456 |
| class | Actinobacteria | rs6720497 | G | A | 0.114 | 0.021 | 2.20E-08 | 30.351 |
| class | Actinobacteria | rs6707289 | A | G | 0.114 | 0.021 | 2.20E-08 | 30.350 |
| class | Actinobacteria | rs2322812 | A | G | 0.099 | 0.018 | 4.52E-08 | 30.315 |
| class | Actinobacteria | rs13010498 | G | C | -0.067 | 0.012 | 2.34E-08 | 30.282 |
| class | Actinobacteria | rs58080116 | A | G | 0.099 | 0.018 | 4.88E-08 | 30.222 |
| class | Actinobacteria | rs6729961 | T | G | 0.114 | 0.021 | 2.79E-08 | 30.130 |
| class | Actinobacteria | rs1865452 | C | T | 0.076 | 0.014 | 8.63E-08 | 30.083 |
| class | Actinobacteria | rs60963894 | T | G | 0.076 | 0.014 | 8.63E-08 | 30.082 |
| class | Actinobacteria | rs10496737 | A | G | 0.076 | 0.014 | 8.75E-08 | 30.046 |
| class | Actinobacteria | rs3806502 | C | T | 0.088 | 0.016 | 1.37E-08 | 29.927 |
| class | Actinobacteria | rs62168846 | G | A | 0.103 | 0.019 | 7.89E-08 | 29.907 |
| class | Actinobacteria | rs12469551 | G | T | 0.075 | 0.014 | 1.10E-07 | 29.881 |
| class | Actinobacteria | rs2322445 | T | A | 0.087 | 0.016 | 1.12E-08 | 29.880 |
| class | Actinobacteria | rs62168847 | T | C | 0.102 | 0.019 | 6.86E-08 | 29.853 |
| class | Actinobacteria | rs4954578 | T | C | 0.066 | 0.012 | 5.40E-08 | 29.851 |
| class | Actinobacteria | rs72970286 | T | G | 0.111 | 0.020 | 2.19E-08 | 29.836 |
| class | Actinobacteria | rs11897217 | C | T | 0.086 | 0.016 | 1.07E-08 | 29.826 |
| class | Melainabacteria | rs9864379 | C | T | -0.160 | 0.029 | 5.36E-08 | 29.812 |
| class | Actinobacteria | rs6738563 | G | T | 0.062 | 0.011 | 1.67E-08 | 29.786 |
| class | Actinobacteria | rs7594509 | G | T | 0.086 | 0.016 | 1.12E-08 | 29.785 |
| class | Actinobacteria | rs4954445 | T | A | 0.098 | 0.018 | 5.74E-08 | 29.722 |
| class | Actinobacteria | rs61663334 | A | G | 0.086 | 0.016 | 7.46E-09 | 29.708 |
| class | Actinobacteria | rs56982689 | C | T | 0.113 | 0.021 | 1.89E-08 | 29.679 |
| class | Actinobacteria | rs60453613 | G | C | 0.075 | 0.014 | 1.08E-07 | 29.652 |
| class | Actinobacteria | rs3769011 | A | T | 0.098 | 0.018 | 6.28E-08 | 29.645 |
| class | Actinobacteria | rs7595712 | C | T | 0.075 | 0.014 | 1.24E-07 | 29.635 |
| class | Actinobacteria | rs4954573 | T | G | 0.067 | 0.012 | 4.84E-08 | 29.629 |
| class | Actinobacteria | rs6705610 | T | G | 0.086 | 0.016 | 1.24E-08 | 29.597 |
| class | Actinobacteria | rs6716987 | C | A | 0.066 | 0.012 | 6.15E-08 | 29.587 |
| class | Actinobacteria | rs62168843 | T | C | 0.101 | 0.019 | 7.75E-08 | 29.536 |
| class | Melainabacteria | rs9830438 | A | C | -0.159 | 0.029 | 6.26E-08 | 29.498 |
| class | Actinobacteria | rs16831994 | T | C | 0.111 | 0.021 | 3.01E-08 | 29.369 |
| class | Actinobacteria | rs1469996 | A | G | 0.075 | 0.014 | 1.43E-07 | 29.328 |
| class | Actinobacteria | rs61470941 | T | C | 0.098 | 0.018 | 5.60E-08 | 29.312 |
| class | Actinobacteria | rs6708957 | C | T | 0.075 | 0.014 | 1.39E-07 | 29.147 |
| class | Actinobacteria | rs3820790 | A | T | 0.098 | 0.018 | 8.35E-08 | 28.916 |
| class | Melainabacteria | rs2005426 | C | A | -0.158 | 0.029 | 9.17E-08 | 28.831 |
| class | Melainabacteria | rs900188 | A | G | -0.157 | 0.029 | 9.54E-08 | 28.817 |
| class | Actinobacteria | rs56036623 | T | C | 0.071 | 0.013 | 5.37E-08 | 28.808 |
| class | Actinobacteria | rs7582173 | G | A | 0.078 | 0.015 | 3.10E-07 | 28.729 |
| class | Actinobacteria | rs147616090 | C | T | 0.074 | 0.014 | 1.76E-07 | 28.680 |
| class | Actinobacteria | rs13008147 | A | G | -0.069 | 0.013 | 1.81E-08 | 28.623 |
| class | Actinobacteria | rs7609188 | G | C | 0.110 | 0.021 | 4.03E-08 | 28.570 |
| class | Actinobacteria | rs1469995 | G | A | 0.074 | 0.014 | 1.83E-07 | 28.552 |
| class | Actinobacteria | rs6732236 | T | G | 0.074 | 0.014 | 2.06E-07 | 28.373 |
| class | Erysipelotrichia | rs62504403 | T | C | 0.068 | 0.013 | 1.12E-07 | 28.371 |
| class | Actinobacteria | rs62168844 | A | G | 0.099 | 0.019 | 1.45E-07 | 28.271 |
| class | Actinobacteria | rs10193587 | T | C | 0.066 | 0.012 | 9.46E-08 | 28.246 |
| class | Actinobacteria | rs7604460 | T | C | 0.083 | 0.016 | 2.02E-07 | 28.227 |
| class | Actinobacteria | rs12992815 | C | G | 0.095 | 0.018 | 1.13E-07 | 28.158 |
| class | Actinobacteria | rs3739022 | G | A | 0.096 | 0.018 | 1.07E-07 | 28.118 |
| class | Actinobacteria | rs6430585 | C | A | 0.073 | 0.014 | 2.66E-07 | 28.104 |
| class | Actinobacteria | rs10197646 | G | A | 0.077 | 0.015 | 4.17E-07 | 28.077 |
| class | Actinobacteria | rs6660520 | G | A | 0.071 | 0.013 | 1.11E-07 | 27.984 |
| class | Actinobacteria | rs10928513 | C | T | 0.061 | 0.012 | 6.28E-08 | 27.866 |
| class | Melainabacteria | rs12493406 | C | T | -0.154 | 0.029 | 2.41E-07 | 27.825 |
| class | Actinobacteria | rs6745996 | A | G | 0.088 | 0.017 | 5.86E-08 | 27.802 |
| class | Actinobacteria | rs998451 | A | G | 0.061 | 0.012 | 3.64E-08 | 27.739 |
| class | Actinobacteria | rs60274701 | G | A | 0.094 | 0.018 | 1.74E-07 | 27.711 |
| class | Actinobacteria | rs7513988 | T | C | 0.069 | 0.013 | 1.19E-07 | 27.671 |
| class | Actinobacteria | rs12022129 | A | G | 0.070 | 0.013 | 1.21E-07 | 27.655 |
| class | Actinobacteria | rs10174462 | C | G | 0.074 | 0.014 | 3.91E-07 | 27.628 |
| class | Actinobacteria | rs7575241 | C | A | 0.073 | 0.014 | 3.32E-07 | 27.457 |
| class | Actinobacteria | rs12471781 | G | A | 0.082 | 0.016 | 2.44E-07 | 27.456 |
| class | Melainabacteria | rs12486770 | T | A | -0.153 | 0.029 | 2.98E-07 | 27.368 |
| class | Melainabacteria | rs9863760 | C | T | -0.149 | 0.028 | 2.21E-07 | 27.285 |
| class | Melainabacteria | rs62231951 | A | T | -0.152 | 0.029 | 3.18E-07 | 27.238 |
| class | Melainabacteria | rs9864035 | G | A | -0.153 | 0.029 | 2.04E-07 | 27.232 |
| class | Melainabacteria | rs6773199 | G | A | -0.149 | 0.029 | 1.54E-07 | 27.141 |
| class | Actinobacteria | rs7556781 | A | G | 0.072 | 0.014 | 4.15E-07 | 27.138 |
| class | Actinobacteria | rs4845143 | T | G | 0.069 | 0.013 | 1.51E-07 | 27.106 |
| class | Melainabacteria | rs6442436 | C | T | -0.151 | 0.029 | 2.24E-07 | 26.992 |
| class | Actinobacteria | rs4954394 | A | G | 0.067 | 0.013 | 1.09E-07 | 26.918 |
| class | Actinobacteria | rs7565871 | G | C | 0.083 | 0.016 | 4.47E-08 | 26.895 |
| class | Erysipelotrichia | rs11990327 | G | A | 0.067 | 0.013 | 2.45E-07 | 26.880 |
| class | Actinobacteria | rs4954388 | T | C | 0.064 | 0.012 | 2.69E-07 | 26.865 |
| class | Actinobacteria | rs7589832 | A | C | 0.072 | 0.014 | 4.79E-07 | 26.865 |
| class | Melainabacteria | rs10510427 | G | A | -0.150 | 0.029 | 3.86E-07 | 26.849 |
| class | Melainabacteria | rs9883109 | T | C | -0.151 | 0.029 | 2.45E-07 | 26.842 |
| class | Methanobacteria | rs10202904 | G | T | -0.122 | 0.024 | 3.01E-07 | 26.761 |
| class | Erysipelotrichia | rs11990200 | G | A | 0.067 | 0.013 | 2.37E-07 | 26.711 |
| class | Melainabacteria | rs13072729 | T | C | -0.151 | 0.029 | 2.96E-07 | 26.661 |
| class | Melainabacteria | rs6780959 | A | G | -0.150 | 0.029 | 2.68E-07 | 26.635 |
| class | Actinobacteria | rs10928554 | C | T | 0.090 | 0.017 | 1.21E-06 | 26.511 |
| class | Actinobacteria | rs7568884 | C | T | 0.063 | 0.012 | 2.16E-07 | 26.508 |
| class | Actinobacteria | rs73958618 | A | G | 0.071 | 0.014 | 5.44E-07 | 26.464 |
| class | Actinobacteria | rs80274970 | C | T | 0.101 | 0.020 | 2.36E-07 | 26.431 |
| class | Melainabacteria | rs13062781 | G | A | -0.146 | 0.028 | 1.61E-07 | 26.359 |
| class | Actinobacteria | rs13415439 | C | A | 0.063 | 0.012 | 2.34E-07 | 26.356 |
| class | Bacteroidia | rs55773148 | A | G | -0.122 | 0.024 | 3.90E-07 | 26.341 |
| class | Bacteroidia | rs55993203 | G | A | -0.122 | 0.024 | 3.90E-07 | 26.341 |
| class | Bacteroidia | rs56358950 | T | C | -0.121 | 0.024 | 4.48E-07 | 26.308 |
| class | Bacteroidia | rs73512608 | A | G | -0.121 | 0.024 | 4.08E-07 | 26.280 |
| class | Bacteroidia | rs58269684 | C | T | -0.121 | 0.024 | 4.49E-07 | 26.243 |
| class | Actinobacteria | rs309124 | A | G | 0.075 | 0.015 | 4.06E-07 | 26.220 |
| class | Actinobacteria | rs6712127 | A | G | 0.072 | 0.014 | 7.56E-07 | 26.187 |
| class | Actinobacteria | rs4954575 | G | A | 0.063 | 0.012 | 2.59E-07 | 26.159 |
| class | Erysipelotrichia | rs62504402 | C | T | 0.066 | 0.013 | 2.88E-07 | 26.152 |
| class | Bacteroidia | rs60610351 | T | A | -0.121 | 0.024 | 4.85E-07 | 26.035 |
| class | Actinobacteria | rs13007153 | T | C | 0.063 | 0.012 | 4.26E-07 | 26.029 |
| class | Melainabacteria | rs1010755 | C | T | -0.148 | 0.029 | 3.90E-07 | 26.003 |
| class | Actinobacteria | rs1028182 | C | T | 0.068 | 0.013 | 2.87E-07 | 25.944 |
| class | Actinobacteria | rs872151 | C | T | 0.091 | 0.018 | 3.12E-07 | 25.936 |
| class | Bacteroidia | rs74090639 | G | A | -0.121 | 0.024 | 5.59E-07 | 25.928 |
| class | Actinobacteria | rs6713776 | C | G | 0.074 | 0.015 | 1.26E-06 | 25.875 |
| class | Melainabacteria | rs17039205 | A | G | -0.148 | 0.029 | 3.98E-07 | 25.827 |
| class | Gammaproteobacteria | rs6706173 | C | A | 0.074 | 0.015 | 1.99E-07 | 25.726 |
| class | Bacteroidia | rs17085771 | A | T | -0.120 | 0.024 | 6.24E-07 | 25.715 |
| class | Gammaproteobacteria | rs6432975 | C | G | 0.074 | 0.015 | 2.02E-07 | 25.695 |
| class | Actinobacteria | rs60241067 | A | T | 0.063 | 0.013 | 6.49E-07 | 25.618 |
| class | Bacteroidia | rs60614038 | T | A | -0.120 | 0.024 | 6.30E-07 | 25.596 |
| class | Bacteroidia | rs58298316 | T | G | -0.120 | 0.024 | 6.30E-07 | 25.596 |
| class | Actinobacteria | rs4954571 | G | A | 0.065 | 0.013 | 2.27E-07 | 25.588 |
| class | Melainabacteria | rs17039208 | T | C | -0.147 | 0.029 | 4.53E-07 | 25.583 |
| class | Alphaproteobacteria | rs140912403 | T | C | -0.161 | 0.032 | 6.20E-07 | 25.577 |
| class | Actinobacteria | rs6430580 | A | G | 0.084 | 0.017 | 1.90E-07 | 25.536 |
| class | Gammaproteobacteria | rs11899748 | C | T | 0.074 | 0.015 | 2.18E-07 | 25.529 |
| class | Actinobacteria | rs72976292 | T | A | 0.062 | 0.012 | 3.56E-07 | 25.504 |
| class | Actinobacteria | rs7564577 | C | T | 0.082 | 0.016 | 2.52E-07 | 25.488 |
| class | Actinobacteria | rs59869505 | A | C | 0.062 | 0.012 | 3.55E-07 | 25.465 |
| class | Bacteroidia | rs9788409 | T | C | -0.119 | 0.024 | 7.07E-07 | 25.430 |
| class | Methanobacteria | rs7601415 | T | A | -0.119 | 0.024 | 6.11E-07 | 25.420 |
| class | Actinobacteria | rs6540645 | C | G | 0.068 | 0.014 | 4.13E-07 | 25.417 |
| class | Actinobacteria | rs309122 | A | G | 0.073 | 0.015 | 6.29E-07 | 25.364 |
| class | Melainabacteria | rs13098772 | C | A | -0.147 | 0.029 | 6.58E-07 | 25.364 |
| class | Negativicutes | rs1643968 | C | T | -0.057 | 0.011 | 4.15E-07 | 25.339 |
| class | Clostridia | rs112334273 | A | G | 0.064 | 0.013 | 3.81E-07 | 25.314 |
| class | Actinobacteria | rs16833170 | A | G | 0.062 | 0.012 | 3.96E-07 | 25.291 |
| class | Bacteroidia | rs17085818 | A | C | -0.119 | 0.024 | 7.46E-07 | 25.273 |
| class | Methanobacteria | rs1869500 | A | C | -0.118 | 0.024 | 6.57E-07 | 25.249 |
| class | Clostridia | NA | T | C | 0.256 | 0.051 | 8.78E-07 | 25.245 |
| class | Melainabacteria | rs11150282 | C | T | 0.099 | 0.020 | 6.03E-07 | 25.236 |
| class | Actinobacteria | rs2056226 | C | G | 0.068 | 0.014 | 4.69E-07 | 25.212 |
| class | Gammaproteobacteria | rs7572454 | G | A | 0.072 | 0.014 | 3.44E-07 | 25.207 |
| class | Erysipelotrichia | rs62504370 | C | A | 0.063 | 0.013 | 4.80E-07 | 25.185 |
| class | Bacteroidia | rs2210451 | C | G | -0.119 | 0.024 | 7.02E-07 | 25.164 |
| class | Actinobacteria | rs6733547 | G | T | 0.080 | 0.016 | 2.67E-07 | 25.147 |
| class | Melainabacteria | rs13088024 | T | A | -0.146 | 0.029 | 6.15E-07 | 25.142 |
| class | Actinobacteria | rs2090661 | A | G | 0.062 | 0.012 | 6.15E-07 | 25.105 |
| class | Bacteroidia | rs2150960 | G | C | -0.119 | 0.024 | 7.16E-07 | 25.097 |
| class | Bacteroidia | rs2210454 | C | T | -0.119 | 0.024 | 7.69E-07 | 25.077 |
| class | Actinobacteria | rs4988243 | T | C | 0.112 | 0.022 | 4.65E-07 | 25.029 |
| class | Melainabacteria | rs13060131 | C | T | -0.146 | 0.029 | 6.46E-07 | 25.020 |
| class | Gammaproteobacteria | rs56730065 | A | G | 0.073 | 0.015 | 2.91E-07 | 25.008 |
| class | Bacteroidia | rs17085798 | C | A | -0.118 | 0.024 | 8.02E-07 | 24.996 |
| class | Actinobacteria | rs309123 | C | G | 0.074 | 0.015 | 6.86E-07 | 24.969 |
| class | Mollicutes | rs10108398 | A | G | 0.077 | 0.015 | 1.09E-06 | 24.960 |
| class | Coriobacteriia | rs719099 | G | A | 0.078 | 0.016 | 5.43E-07 | 24.957 |
| class | Melainabacteria | rs13072505 | A | G | -0.145 | 0.029 | 6.55E-07 | 24.949 |
| class | Bacteroidia | rs74090627 | C | T | -0.118 | 0.024 | 7.95E-07 | 24.946 |
| class | Verrucomicrobiae | rs11729256 | C | T | 0.075 | 0.015 | 6.73E-07 | 24.928 |
| class | Actinobacteria | rs1028181 | C | T | 0.067 | 0.014 | 5.42E-07 | 24.926 |
| class | Bacteroidia | rs56949730 | A | G | -0.118 | 0.024 | 7.98E-07 | 24.911 |
| class | Melainabacteria | rs12714900 | G | T | -0.145 | 0.029 | 6.82E-07 | 24.880 |
| class | Bacteroidia | rs112343530 | T | C | -0.118 | 0.024 | 7.32E-07 | 24.870 |
| class | Bacteroidia | rs80015632 | C | G | -0.118 | 0.024 | 8.23E-07 | 24.865 |
| class | Melainabacteria | rs12491989 | C | G | -0.145 | 0.029 | 7.10E-07 | 24.818 |
| class | Melainabacteria | rs12714903 | C | T | -0.145 | 0.029 | 7.17E-07 | 24.783 |
| class | Alphaproteobacteria | rs76784716 | G | A | 0.133 | 0.027 | 5.09E-07 | 24.777 |
| class | Betaproteobacteria | rs4033856 | T | C | 0.083 | 0.017 | 5.17E-07 | 24.776 |
| class | Actinobacteria | rs1807356 | T | C | 0.081 | 0.016 | 4.53E-07 | 24.763 |
| class | Deltaproteobacteria | rs6058181 | T | C | 0.083 | 0.017 | 3.40E-07 | 24.755 |
| class | Melainabacteria | rs7206242 | C | T | 0.100 | 0.020 | 8.60E-07 | 24.715 |
| class | Melainabacteria | rs13095126 | T | C | -0.145 | 0.029 | 7.67E-07 | 24.686 |
| class | Clostridia | NA | C | T | 0.252 | 0.051 | 1.23E-06 | 24.658 |
| class | Melainabacteria | rs13072444 | G | A | -0.145 | 0.029 | 7.80E-07 | 24.649 |
| class | Bacteroidia | rs2210452 | G | A | -0.117 | 0.024 | 9.11E-07 | 24.612 |
| class | Melainabacteria | rs12714902 | G | A | -0.144 | 0.029 | 7.91E-07 | 24.595 |
| class | Bacteroidia | rs112675712 | T | C | -0.117 | 0.024 | 8.12E-07 | 24.553 |
| class | Actinobacteria | rs4954556 | G | A | 0.123 | 0.025 | 2.90E-07 | 24.550 |
| class | Gammaproteobacteria | rs6432974 | G | T | 0.073 | 0.015 | 3.48E-07 | 24.542 |
| class | Actinobacteria | rs6685379 | A | G | 0.066 | 0.013 | 6.13E-07 | 24.527 |
| class | Actinobacteria | rs3098104 | T | A | 0.076 | 0.015 | 1.25E-06 | 24.492 |
| class | Alphaproteobacteria | rs77497586 | G | T | 0.132 | 0.027 | 5.63E-07 | 24.473 |
| class | Actinobacteria | rs908704 | A | G | 0.066 | 0.013 | 6.66E-07 | 24.468 |
| class | Coriobacteriia | rs2458655 | C | A | 0.078 | 0.016 | 7.77E-07 | 24.461 |
| class | Clostridia | rs13421739 | G | C | 0.089 | 0.018 | 9.54E-06 | 24.457 |
| class | Deltaproteobacteria | rs6060239 | T | C | 0.082 | 0.017 | 4.68E-07 | 24.333 |
| class | Actinobacteria | rs8047955 | G | A | 0.058 | 0.012 | 8.34E-07 | 24.320 |
| class | Methanobacteria | rs73457410 | G | A | 0.215 | 0.044 | 1.41E-06 | 24.316 |
| class | Betaproteobacteria | rs6087811 | G | T | -0.098 | 0.020 | 7.44E-07 | 24.278 |
| class | Actinobacteria | rs55809728 | G | A | 0.107 | 0.022 | 4.46E-07 | 24.249 |
| class | Melainabacteria | rs7644367 | G | A | -0.143 | 0.029 | 8.95E-07 | 24.237 |
| class | Clostridia | rs6815608 | C | T | 0.104 | 0.021 | 4.02E-07 | 24.229 |
| class | Actinobacteria | rs75667274 | C | T | 0.077 | 0.016 | 1.03E-06 | 24.214 |
| class | Lentisphaeria | rs62570196 | T | C | -0.216 | 0.044 | 1.08E-06 | 24.192 |
| class | Negativicutes | rs60274479 | C | T | -0.066 | 0.013 | 1.16E-06 | 24.182 |
| class | Melainabacteria | rs9832125 | C | T | -0.139 | 0.028 | 3.62E-07 | 24.180 |
| class | Deltaproteobacteria | rs6060238 | G | A | 0.082 | 0.017 | 4.88E-07 | 24.178 |
| class | Actinobacteria | rs112407062 | C | T | 0.076 | 0.015 | 1.03E-06 | 24.172 |
| class | Bacteroidia | rs7337863 | C | A | -0.117 | 0.024 | 8.25E-07 | 24.167 |
| class | Deltaproteobacteria | rs6060237 | A | G | 0.082 | 0.017 | 4.94E-07 | 24.166 |
| class | Melainabacteria | rs13072359 | A | C | -0.143 | 0.029 | 1.01E-06 | 24.146 |
| class | Actinobacteria | rs8057441 | T | C | 0.057 | 0.012 | 9.51E-07 | 24.116 |
| class | Betaproteobacteria | rs2367850 | C | G | 0.064 | 0.013 | 9.12E-07 | 24.102 |
| class | Melainabacteria | rs13072044 | G | T | -0.147 | 0.030 | 9.30E-07 | 24.100 |
| class | Erysipelotrichia | rs2300774 | A | G | 0.052 | 0.011 | 8.95E-07 | 24.094 |
| class | Actinobacteria | rs16832440 | G | A | 0.076 | 0.015 | 1.27E-06 | 24.092 |
| class | Verrucomicrobiae | rs113634144 | G | T | 0.074 | 0.015 | 1.12E-06 | 24.092 |
| class | Clostridia | rs6814436 | C | T | 0.074 | 0.015 | 9.65E-07 | 24.075 |
| class | Actinobacteria | rs75128007 | A | G | 0.064 | 0.013 | 1.50E-06 | 24.072 |
| class | Actinobacteria | rs309152 | T | C | 0.074 | 0.015 | 9.01E-07 | 24.055 |
| class | Melainabacteria | rs13084677 | A | T | -0.138 | 0.028 | 3.82E-07 | 24.023 |
| class | Actinobacteria | rs62168955 | C | T | 0.245 | 0.050 | 2.14E-06 | 24.022 |
| class | Bacilli | rs11730038 | A | G | -0.063 | 0.013 | 1.96E-06 | 24.012 |
| class | Actinobacteria | rs1964459 | C | G | 0.076 | 0.015 | 1.38E-06 | 23.986 |
| class | Actinobacteria | rs7322849 | C | T | 0.094 | 0.019 | 6.21E-07 | 23.953 |
| class | Actinobacteria | rs115553816 | G | A | 0.076 | 0.015 | 1.16E-06 | 23.949 |
| class | Actinobacteria | rs112396243 | C | T | 0.076 | 0.015 | 1.19E-06 | 23.937 |
| class | Erysipelotrichia | rs2300775 | C | T | 0.052 | 0.011 | 9.78E-07 | 23.919 |
| class | Actinobacteria | rs309133 | A | C | 0.073 | 0.015 | 1.06E-06 | 23.893 |
| class | Bacilli | rs4131679 | T | C | -0.063 | 0.013 | 2.14E-06 | 23.865 |
| class | Alphaproteobacteria | rs9813022 | G | A | -0.075 | 0.015 | 1.05E-06 | 23.840 |
| class | Melainabacteria | rs7204337 | G | A | 0.099 | 0.020 | 1.45E-06 | 23.840 |
| class | Actinobacteria | rs721765 | C | A | 0.076 | 0.015 | 1.49E-06 | 23.831 |
| class | Actinobacteria | rs11745923 | T | G | 0.056 | 0.012 | 1.58E-06 | 23.812 |
| class | Actinobacteria | rs12476127 | A | G | 0.074 | 0.015 | 1.50E-06 | 23.798 |
| class | Actinobacteria | rs2138992 | C | A | 0.066 | 0.014 | 8.92E-07 | 23.786 |
| class | Actinobacteria | rs111682961 | C | T | 0.075 | 0.015 | 1.33E-06 | 23.785 |
| class | Alphaproteobacteria | rs75071199 | C | A | 0.131 | 0.027 | 7.91E-07 | 23.783 |
| class | Coriobacteriia | rs7078773 | C | T | 0.076 | 0.016 | 1.12E-06 | 23.780 |
| class | Actinobacteria | rs79826902 | G | A | 0.075 | 0.015 | 1.31E-06 | 23.778 |
| class | Bacilli | rs35344081 | A | G | 0.062 | 0.013 | 1.01E-06 | 23.772 |
| class | Gammaproteobacteria | rs11181912 | A | G | -0.058 | 0.012 | 9.95E-07 | 23.767 |
| class | Bacilli | rs4293791 | T | C | -0.063 | 0.013 | 2.25E-06 | 23.758 |
| class | Bacilli | rs57872228 | T | C | -0.071 | 0.015 | 9.22E-07 | 23.757 |
| class | Alphaproteobacteria | rs55661963 | A | C | 0.130 | 0.027 | 9.33E-07 | 23.749 |
| class | Erysipelotrichia | rs7234058 | C | T | -0.095 | 0.019 | 9.12E-07 | 23.744 |
| class | Actinobacteria | rs1942055 | G | C | 0.056 | 0.012 | 5.83E-07 | 23.742 |
| class | Erysipelotrichia | rs9858727 | T | A | 0.052 | 0.011 | 1.08E-06 | 23.728 |
| class | Erysipelotrichia | rs2300780 | C | T | 0.052 | 0.011 | 1.10E-06 | 23.722 |
| class | Actinobacteria | rs4334924 | G | A | 0.057 | 0.012 | 1.88E-06 | 23.704 |
| class | Betaproteobacteria | rs12467854 | C | G | 0.064 | 0.013 | 1.10E-06 | 23.692 |
| class | Methanobacteria | rs62241835 | T | G | -0.203 | 0.042 | 1.63E-06 | 23.683 |
| class | Mollicutes | rs67374027 | T | A | 0.075 | 0.015 | 9.70E-07 | 23.634 |
| class | Actinobacteria | rs12464050 | G | T | 0.075 | 0.015 | 1.43E-06 | 23.634 |
| class | Negativicutes | rs2921533 | A | G | -0.054 | 0.011 | 1.02E-06 | 23.631 |
| class | Bacteroidia | rs73510191 | A | C | -0.115 | 0.024 | 1.65E-06 | 23.628 |
| class | Actinobacteria | rs586964 | G | A | 0.075 | 0.015 | 1.52E-06 | 23.609 |
| class | Bacteroidia | rs73846128 | G | A | -0.065 | 0.013 | 8.76E-07 | 23.602 |
| class | Erysipelotrichia | rs79996147 | A | T | -0.094 | 0.019 | 9.72E-07 | 23.597 |
| class | Actinobacteria | rs16832205 | G | A | 0.075 | 0.015 | 1.41E-06 | 23.592 |
| class | Erysipelotrichia | rs2300777 | C | A | 0.052 | 0.011 | 1.18E-06 | 23.592 |
| class | Bacteroidia | rs7631304 | A | G | -0.065 | 0.013 | 8.37E-07 | 23.590 |
| class | Actinobacteria | rs12474975 | A | T | 0.075 | 0.015 | 1.32E-06 | 23.587 |
| class | Actinobacteria | rs309119 | T | C | 0.073 | 0.015 | 1.09E-06 | 23.576 |
| class | Mollicutes | rs11890098 | G | A | 0.074 | 0.015 | 9.57E-07 | 23.551 |
| class | Actinobacteria | rs16832243 | C | T | 0.075 | 0.015 | 1.39E-06 | 23.549 |
| class | Mollicutes | rs10111506 | A | T | 0.069 | 0.014 | 1.60E-06 | 23.545 |
| class | Mollicutes | rs12674568 | A | G | 0.069 | 0.014 | 1.60E-06 | 23.535 |
| class | Coriobacteriia | rs56374305 | C | G | 0.100 | 0.021 | 1.17E-06 | 23.530 |
| class | Actinobacteria | rs2254780 | C | T | 0.071 | 0.015 | 1.14E-06 | 23.497 |
| class | Betaproteobacteria | rs62191117 | G | A | 0.064 | 0.013 | 1.17E-06 | 23.488 |
| class | Methanobacteria | rs6776814 | C | T | -0.200 | 0.041 | 1.63E-06 | 23.483 |
| class | Bacteroidia | rs4474577 | G | A | -0.118 | 0.024 | 1.47E-06 | 23.471 |
| class | Melainabacteria | rs4129395 | A | G | 0.090 | 0.019 | 1.48E-06 | 23.440 |
| class | Actinobacteria | rs77096272 | C | A | 0.075 | 0.015 | 1.60E-06 | 23.432 |
| class | Bacteroidia | rs56335733 | C | T | -0.115 | 0.024 | 1.66E-06 | 23.389 |
| class | Mollicutes | rs10102709 | C | T | 0.069 | 0.014 | 1.75E-06 | 23.383 |
| class | Betaproteobacteria | rs78428620 | C | T | -0.098 | 0.020 | 1.27E-06 | 23.375 |
| class | Bacilli | rs12642660 | G | A | -0.075 | 0.015 | 2.42E-06 | 23.375 |
| class | Bacteroidia | rs2225529 | C | T | -0.115 | 0.024 | 1.44E-06 | 23.364 |
| class | Bacilli | rs2952251 | G | A | -0.060 | 0.012 | 1.08E-06 | 23.361 |
| class | Melainabacteria | rs4129397 | A | G | 0.089 | 0.019 | 1.54E-06 | 23.360 |
| class | Actinobacteria | rs309161 | G | A | 0.072 | 0.015 | 1.42E-06 | 23.359 |
| class | Bacteroidia | rs60660711 | G | A | -0.115 | 0.024 | 1.52E-06 | 23.350 |
| class | Methanobacteria | rs894996 | A | C | 0.217 | 0.045 | 1.88E-06 | 23.349 |
| class | Actinobacteria | rs12448146 | C | A | 0.056 | 0.012 | 1.28E-06 | 23.343 |
| class | Actinobacteria | rs2441384 | T | C | 0.071 | 0.015 | 1.23E-06 | 23.337 |
| class | Actinobacteria | rs499483 | G | A | 0.074 | 0.015 | 1.74E-06 | 23.321 |
| class | Bacilli | rs1962325 | G | C | 0.055 | 0.011 | 1.38E-06 | 23.311 |
| class | Actinobacteria | rs3098103 | T | C | 0.075 | 0.015 | 1.82E-06 | 23.301 |
| class | Erysipelotrichia | rs2300778 | G | A | 0.052 | 0.011 | 1.37E-06 | 23.298 |
| class | Negativicutes | rs73232831 | A | G | -0.152 | 0.031 | 1.87E-06 | 23.242 |
| class | Gammaproteobacteria | rs12105903 | C | T | 0.070 | 0.014 | 8.40E-07 | 23.240 |
| class | Methanobacteria | rs9942108 | G | A | -0.203 | 0.042 | 1.85E-06 | 23.240 |
| class | Bacteroidia | rs1888900 | T | C | -0.114 | 0.024 | 2.03E-06 | 23.239 |
| class | Actinobacteria | rs6756200 | T | C | 0.055 | 0.011 | 1.20E-06 | 23.232 |
| class | Clostridia | rs72915163 | C | T | -0.058 | 0.012 | 1.34E-06 | 23.227 |
| class | Deltaproteobacteria | rs6060240 | C | A | 0.081 | 0.017 | 9.65E-07 | 23.225 |
| class | Methanobacteria | rs73031978 | G | C | -0.203 | 0.042 | 1.90E-06 | 23.225 |
| class | Actinobacteria | rs6711493 | A | G | 0.071 | 0.015 | 1.41E-06 | 23.218 |
| class | Bacteroidia | rs7338117 | A | G | -0.115 | 0.024 | 1.58E-06 | 23.218 |
| class | Bacteroidia | rs17085746 | A | G | -0.115 | 0.024 | 1.63E-06 | 23.214 |
| class | Bacteroidia | rs17085738 | T | G | -0.115 | 0.024 | 1.63E-06 | 23.214 |
| class | Bacilli | rs9581006 | T | C | 0.225 | 0.047 | 1.79E-06 | 23.207 |
| class | Mollicutes | rs12680996 | T | C | 0.069 | 0.014 | 1.92E-06 | 23.189 |
| class | Mollicutes | rs16923562 | A | G | 0.069 | 0.014 | 1.92E-06 | 23.174 |
| class | Bacilli | rs74352383 | A | T | -0.104 | 0.022 | 1.53E-06 | 23.171 |
| class | Gammaproteobacteria | rs67269716 | G | A | 0.069 | 0.014 | 9.00E-07 | 23.158 |
| class | Bacilli | rs77558518 | G | A | -0.107 | 0.022 | 1.34E-06 | 23.158 |
| class | Bacteroidia | rs11929346 | T | C | -0.064 | 0.013 | 1.10E-06 | 23.143 |
| class | Methanobacteria | rs76029318 | C | T | 0.215 | 0.045 | 1.58E-06 | 23.140 |
| class | Methanobacteria | rs62240385 | T | G | -0.201 | 0.042 | 1.77E-06 | 23.135 |
| class | Actinobacteria | rs309170 | C | T | 0.072 | 0.015 | 1.52E-06 | 23.125 |
| class | Actinobacteria | rs4440852 | A | C | 0.065 | 0.014 | 1.24E-06 | 23.120 |
| class | Erysipelotrichia | rs35161940 | C | T | -0.081 | 0.017 | 1.85E-06 | 23.118 |
| class | Bacteroidia | rs73510178 | G | A | -0.115 | 0.024 | 1.67E-06 | 23.113 |
| class | Mollicutes | rs16923564 | T | C | 0.068 | 0.014 | 1.99E-06 | 23.109 |
| class | Actinobacteria | rs6728946 | C | G | 0.073 | 0.015 | 1.64E-06 | 23.107 |
| class | Bacteroidia | rs12938514 | G | A | -0.200 | 0.042 | 1.42E-06 | 23.106 |
| class | Actinobacteria | rs309118 | G | C | 0.070 | 0.015 | 1.91E-06 | 23.105 |
| class | Melainabacteria | rs16851659 | C | G | -0.090 | 0.019 | 1.27E-06 | 23.103 |
| class | Methanobacteria | rs12711690 | G | A | -0.113 | 0.024 | 1.97E-06 | 23.103 |
| class | Bacteroidia | rs61070599 | C | T | -0.115 | 0.024 | 1.82E-06 | 23.094 |
| class | Mollicutes | rs11776003 | G | A | 0.068 | 0.014 | 2.03E-06 | 23.077 |
| class | Erysipelotrichia | rs2284890 | C | T | 0.051 | 0.011 | 1.54E-06 | 23.077 |
| class | Actinobacteria | rs10496738 | C | T | 0.074 | 0.015 | 2.19E-06 | 23.068 |
| class | Actinobacteria | rs309158 | A | G | 0.070 | 0.015 | 1.69E-06 | 23.067 |
| class | Gammaproteobacteria | rs7580360 | A | G | 0.070 | 0.015 | 8.87E-07 | 23.060 |
| class | Actinobacteria | rs2090660 | C | T | 0.062 | 0.013 | 2.21E-06 | 23.044 |
| class | Actinobacteria | rs309159 | C | T | 0.073 | 0.015 | 1.46E-06 | 23.043 |
| class | Bacteroidia | rs111845179 | C | T | 0.103 | 0.021 | 9.24E-07 | 23.041 |
| class | Actinobacteria | rs16832624 | T | C | 0.062 | 0.013 | 2.02E-06 | 23.039 |
| class | Bacteroidia | rs74937713 | A | T | -0.063 | 0.013 | 1.25E-06 | 23.034 |
| class | Bacteroidia | rs35800684 | G | A | -0.064 | 0.013 | 1.15E-06 | 23.032 |
| class | Melainabacteria | rs4129396 | A | G | 0.089 | 0.019 | 1.84E-06 | 23.021 |
| class | Actinobacteria | rs57243841 | A | G | 0.063 | 0.013 | 2.08E-06 | 23.009 |
| class | Erysipelotrichia | rs2284889 | A | G | 0.051 | 0.011 | 1.60E-06 | 23.005 |
| class | Bacilli | rs76717940 | A | T | 0.156 | 0.033 | 1.07E-06 | 23.004 |
| class | Melainabacteria | rs72765665 | C | T | -0.089 | 0.019 | 1.35E-06 | 23.003 |
| class | Lentisphaeria | rs77599476 | G | A | 0.230 | 0.048 | 1.86E-06 | 23.002 |
| class | Mollicutes | rs1991359 | A | G | 0.068 | 0.014 | 2.12E-06 | 22.997 |
| class | Mollicutes | rs28650441 | A | C | 0.068 | 0.014 | 2.13E-06 | 22.994 |
| class | Actinobacteria | rs59213715 | A | C | 0.074 | 0.015 | 1.74E-06 | 22.985 |
| class | Actinobacteria | rs41269821 | A | G | 0.073 | 0.015 | 1.56E-06 | 22.980 |
| class | Erysipelotrichia | rs2239641 | C | T | 0.051 | 0.011 | 1.62E-06 | 22.979 |
| class | Actinobacteria | rs10171677 | G | A | 0.055 | 0.011 | 8.34E-07 | 22.979 |
| class | Betaproteobacteria | rs4511740 | A | G | 0.061 | 0.013 | 1.66E-06 | 22.978 |
| class | Methanobacteria | rs62241834 | G | T | -0.201 | 0.042 | 2.44E-06 | 22.975 |
| class | Erysipelotrichia | rs79329601 | C | T | -0.093 | 0.019 | 1.40E-06 | 22.960 |
| class | Gammaproteobacteria | rs7586785 | C | G | 0.069 | 0.014 | 9.75E-07 | 22.960 |
| class | Actinobacteria | rs6743537 | G | A | 0.073 | 0.015 | 2.03E-06 | 22.959 |
| class | Bacteroidia | rs7546249 | A | T | -0.057 | 0.012 | 1.55E-06 | 22.948 |
| class | Erysipelotrichia | rs76420385 | G | A | -0.093 | 0.019 | 1.47E-06 | 22.941 |
| class | Bacteroidia | rs60869989 | G | A | -0.114 | 0.024 | 1.94E-06 | 22.938 |
| class | Mollicutes | rs7009248 | A | G | 0.068 | 0.014 | 2.24E-06 | 22.924 |
| class | Mollicutes | rs74603314 | C | T | 0.222 | 0.046 | 1.56E-06 | 22.924 |
| class | Methanobacteria | rs6784384 | G | A | -0.198 | 0.041 | 1.90E-06 | 22.909 |
| class | Mollicutes | rs6471729 | G | A | 0.068 | 0.014 | 2.22E-06 | 22.899 |
| class | Verrucomicrobiae | rs9349825 | G | A | -0.070 | 0.015 | 2.54E-06 | 22.898 |
| class | Actinobacteria | rs75753154 | T | C | 0.072 | 0.015 | 1.76E-06 | 22.896 |
| class | Actinobacteria | rs11738544 | G | A | 0.055 | 0.012 | 2.62E-06 | 22.892 |
| class | Bacteroidia | rs73512626 | C | A | -0.114 | 0.024 | 2.02E-06 | 22.891 |
| class | Mollicutes | rs7817515 | T | G | 0.068 | 0.014 | 2.23E-06 | 22.891 |
| class | Actinobacteria | rs177917 | A | G | 0.071 | 0.015 | 1.80E-06 | 22.887 |
| class | Actinobacteria | rs1961273 | T | C | 0.060 | 0.013 | 1.75E-06 | 22.884 |
| class | Actinobacteria | rs4954555 | T | C | 0.107 | 0.022 | 1.44E-06 | 22.870 |
| class | Verrucomicrobiae | rs2602429 | T | C | 0.075 | 0.016 | 2.58E-06 | 22.863 |
| class | Actinobacteria | rs134296 | A | C | 0.112 | 0.023 | 1.41E-06 | 22.862 |
| class | Bacteroidia | rs60722059 | A | G | -0.114 | 0.024 | 1.88E-06 | 22.839 |
| class | Actinobacteria | rs10180515 | T | A | 0.064 | 0.013 | 9.88E-07 | 22.836 |
| class | Methanobacteria | rs62240384 | C | T | -0.198 | 0.041 | 1.99E-06 | 22.817 |
| class | Actinobacteria | rs309166 | C | T | 0.071 | 0.015 | 1.79E-06 | 22.817 |
| class | Bacilli | rs80205102 | T | C | -0.061 | 0.013 | 3.63E-06 | 22.804 |
| class | Actinobacteria | rs11895609 | A | T | 0.076 | 0.016 | 5.27E-07 | 22.804 |
| class | Actinobacteria | rs80261410 | T | C | 0.062 | 0.013 | 2.91E-06 | 22.799 |
| class | Bacteroidia | rs61289215 | C | T | -0.114 | 0.024 | 2.19E-06 | 22.794 |
| class | Methanobacteria | rs56209241 | G | A | -0.202 | 0.042 | 2.53E-06 | 22.793 |
| class | Actinobacteria | rs908703 | C | G | 0.063 | 0.013 | 1.41E-06 | 22.792 |
| class | Methanobacteria | rs73457427 | G | A | 0.213 | 0.045 | 1.86E-06 | 22.790 |
| class | Actinobacteria | rs7606267 | T | C | 0.072 | 0.015 | 1.84E-06 | 22.789 |
| class | Verrucomicrobiae | rs4936098 | G | A | 0.065 | 0.014 | 1.12E-06 | 22.786 |
| class | Erysipelotrichia | rs2239640 | T | G | 0.051 | 0.011 | 1.79E-06 | 22.783 |
| class | Gammaproteobacteria | rs9967718 | G | A | 0.069 | 0.014 | 1.08E-06 | 22.778 |
| class | Mollicutes | rs1583268 | G | A | 0.073 | 0.015 | 1.45E-06 | 22.775 |
| class | Deltaproteobacteria | rs17791387 | G | A | -0.074 | 0.015 | 1.60E-06 | 22.761 |
| class | Actinobacteria | rs11678502 | T | C | 0.055 | 0.012 | 2.07E-06 | 22.758 |
| class | Erysipelotrichia | rs9852239 | C | T | 0.051 | 0.011 | 1.81E-06 | 22.752 |
| class | Mollicutes | rs28429491 | C | T | 0.068 | 0.014 | 2.39E-06 | 22.741 |
| class | Bacilli | rs11967379 | C | T | -0.103 | 0.022 | 2.40E-06 | 22.740 |
| class | Bacteroidia | rs7629132 | C | T | -0.063 | 0.013 | 1.48E-06 | 22.734 |
| class | Erysipelotrichia | rs78782849 | C | T | -0.092 | 0.019 | 1.61E-06 | 22.731 |
| class | Erysipelotrichia | rs9863872 | G | T | 0.051 | 0.011 | 1.87E-06 | 22.709 |
| class | Erysipelotrichia | rs9846149 | G | C | 0.051 | 0.011 | 1.87E-06 | 22.696 |
| class | Actinobacteria | rs16832162 | T | A | 0.072 | 0.015 | 1.98E-06 | 22.690 |
| class | Actinobacteria | rs11688302 | C | T | 0.058 | 0.012 | 1.67E-06 | 22.680 |
| class | Actinobacteria | rs12986776 | G | C | 0.055 | 0.012 | 2.21E-06 | 22.678 |
| class | Mollicutes | rs7825996 | A | T | 0.068 | 0.014 | 2.60E-06 | 22.677 |
| class | Bacteroidia | rs74090631 | A | G | -0.114 | 0.024 | 2.10E-06 | 22.673 |
| class | Bacteroidia | rs73510184 | C | T | -0.114 | 0.024 | 2.10E-06 | 22.673 |
| class | Actinobacteria | rs309167 | C | T | 0.073 | 0.015 | 2.31E-06 | 22.663 |
| class | Actinobacteria | rs134366 | A | G | 0.112 | 0.024 | 1.50E-06 | 22.662 |
| class | Betaproteobacteria | rs1928341 | G | A | 0.053 | 0.011 | 2.02E-06 | 22.661 |
| class | Bacteroidia | rs2032750 | C | T | -0.051 | 0.011 | 1.92E-06 | 22.657 |
| class | Melainabacteria | rs7859354 | C | T | 0.088 | 0.019 | 2.23E-06 | 22.653 |
| class | Actinobacteria | rs134372 | T | A | 0.112 | 0.024 | 1.55E-06 | 22.653 |
| class | Actinobacteria | rs56100046 | T | A | 0.074 | 0.015 | 2.20E-06 | 22.652 |
| class | Methanobacteria | rs4848258 | A | T | -0.112 | 0.024 | 2.44E-06 | 22.649 |
| class | Actinobacteria | rs3087343 | T | G | 0.072 | 0.015 | 2.23E-06 | 22.643 |
| class | Actinobacteria | rs4245838 | C | T | 0.107 | 0.022 | 1.63E-06 | 22.634 |
| class | Bacteroidia | rs60954548 | A | G | -0.113 | 0.024 | 2.38E-06 | 22.634 |
| class | Erysipelotrichia | rs10781552 | T | C | -0.055 | 0.012 | 2.33E-06 | 22.633 |
| class | Actinobacteria | rs309120 | G | C | 0.071 | 0.015 | 1.73E-06 | 22.630 |
| class | Actinobacteria | rs4954558 | A | G | 0.062 | 0.013 | 2.50E-06 | 22.630 |
| class | Coriobacteriia | rs240104 | C | T | -0.060 | 0.013 | 1.52E-06 | 22.630 |
| class | Mollicutes | rs7014398 | C | T | 0.068 | 0.014 | 2.55E-06 | 22.628 |
| class | Actinobacteria | rs309165 | T | C | 0.069 | 0.015 | 2.08E-06 | 22.622 |
| class | Methanobacteria | rs4848970 | G | T | 0.116 | 0.024 | 1.89E-06 | 22.619 |
| class | Actinobacteria | rs11684458 | A | G | 0.064 | 0.013 | 1.04E-06 | 22.608 |
| class | Actinobacteria | rs1057031 | G | A | 0.072 | 0.015 | 2.13E-06 | 22.606 |
| class | Methanobacteria | rs62240373 | G | A | -0.199 | 0.042 | 2.75E-06 | 22.598 |
| class | Actinobacteria | rs3098102 | C | T | 0.071 | 0.015 | 2.03E-06 | 22.588 |
| class | Actinobacteria | rs4954557 | C | T | 0.059 | 0.012 | 1.95E-06 | 22.584 |
| class | Clostridia | rs4690986 | C | G | 0.071 | 0.015 | 2.16E-06 | 22.576 |
| class | Methanobacteria | rs11147839 | G | A | 0.211 | 0.044 | 2.28E-06 | 22.574 |
| class | Methanobacteria | rs62241836 | C | T | -0.199 | 0.042 | 2.84E-06 | 22.571 |
| class | Melainabacteria | rs10739388 | T | C | 0.088 | 0.019 | 2.33E-06 | 22.570 |
| class | Lentisphaeria | rs2825714 | G | A | -0.137 | 0.029 | 1.72E-06 | 22.569 |
| class | Mollicutes | rs72647435 | G | T | 0.068 | 0.014 | 2.76E-06 | 22.564 |
| class | Gammaproteobacteria | rs59250079 | T | G | 0.070 | 0.015 | 1.39E-06 | 22.549 |
| class | Mollicutes | rs55774738 | T | C | 0.067 | 0.014 | 2.61E-06 | 22.544 |
| class | Negativicutes | rs71405394 | A | G | -0.114 | 0.024 | 2.17E-06 | 22.539 |
| class | Actinobacteria | rs134363 | A | G | 0.112 | 0.024 | 1.67E-06 | 22.532 |
| class | Negativicutes | rs13086907 | A | G | 0.063 | 0.013 | 1.95E-06 | 22.532 |
| class | Actinobacteria | rs7573555 | T | C | 0.072 | 0.015 | 2.10E-06 | 22.523 |
| class | Actinobacteria | rs2839740 | G | T | 0.073 | 0.015 | 2.40E-06 | 22.518 |
| class | Bacteroidia | rs7611599 | C | T | -0.063 | 0.013 | 1.49E-06 | 22.517 |
| class | Actinobacteria | rs2176716 | C | T | 0.064 | 0.013 | 1.11E-06 | 22.511 |
| class | Bacteroidia | rs74090626 | A | G | -0.113 | 0.024 | 2.30E-06 | 22.508 |
| class | Verrucomicrobiae | rs74542928 | C | T | 0.112 | 0.024 | 1.63E-06 | 22.508 |
| class | Bacteroidia | rs11920519 | A | G | -0.063 | 0.013 | 1.70E-06 | 22.504 |
| class | Mollicutes | rs10099153 | C | G | 0.068 | 0.014 | 2.83E-06 | 22.503 |
| class | Actinobacteria | rs7410699 | G | C | 0.111 | 0.023 | 1.64E-06 | 22.502 |
| class | Bacilli | rs17708276 | G | A | -0.079 | 0.017 | 1.84E-06 | 22.496 |
| class | Verrucomicrobiae | rs117107102 | G | A | 0.205 | 0.043 | 2.92E-06 | 22.493 |
| class | Bacteroidia | rs7337151 | A | C | -0.113 | 0.024 | 2.35E-06 | 22.491 |
| class | Bacteroidia | rs60814075 | C | T | -0.113 | 0.024 | 2.31E-06 | 22.479 |
| class | Bacteroidia | rs12486528 | G | A | -0.064 | 0.013 | 1.55E-06 | 22.469 |
| class | Bacilli | rs4028634 | C | T | 0.052 | 0.011 | 2.21E-06 | 22.465 |
| class | Actinobacteria | rs1960171 | C | T | 0.059 | 0.012 | 3.03E-06 | 22.461 |
| class | Negativicutes | rs4722181 | G | T | 0.050 | 0.011 | 2.00E-06 | 22.452 |
| class | Actinobacteria | rs134331 | C | G | 0.111 | 0.024 | 1.86E-06 | 22.452 |
| class | Bacilli | rs6813875 | G | A | -0.061 | 0.013 | 4.26E-06 | 22.451 |
| class | Bacteroidia | rs17085776 | A | G | -0.113 | 0.024 | 2.65E-06 | 22.450 |
| class | Actinobacteria | rs309162 | T | A | 0.071 | 0.015 | 2.34E-06 | 22.429 |
| class | Lentisphaeria | rs112022900 | G | A | 0.218 | 0.046 | 2.76E-06 | 22.423 |
| class | Mollicutes | rs4325706 | C | T | 0.073 | 0.015 | 1.73E-06 | 22.418 |
| class | Actinobacteria | rs614193 | A | T | 0.071 | 0.015 | 2.24E-06 | 22.414 |
| class | Actinobacteria | rs9939612 | C | T | 0.055 | 0.012 | 2.09E-06 | 22.411 |
| class | Melainabacteria | rs68053172 | G | A | -0.088 | 0.019 | 1.86E-06 | 22.410 |
| class | Actinobacteria | rs72847650 | C | A | -0.056 | 0.012 | 1.23E-06 | 22.408 |
| class | Bacilli | rs9445783 | A | T | -0.103 | 0.022 | 2.29E-06 | 22.408 |
| class | Bacteroidia | rs17085756 | G | A | -0.113 | 0.024 | 2.66E-06 | 22.399 |
| class | Actinobacteria | rs10841473 | C | G | -0.058 | 0.012 | 2.59E-06 | 22.396 |
| class | Lentisphaeria | rs45621736 | G | A | 0.218 | 0.046 | 2.75E-06 | 22.387 |
| class | Bacteroidia | rs7491419 | T | G | -0.113 | 0.024 | 2.53E-06 | 22.385 |
| class | Melainabacteria | rs72765667 | A | G | -0.088 | 0.019 | 1.85E-06 | 22.384 |
| class | Actinobacteria | rs134333 | A | G | 0.111 | 0.024 | 1.94E-06 | 22.375 |
| class | Actinobacteria | rs134371 | C | T | 0.111 | 0.024 | 1.76E-06 | 22.374 |
| class | Bacteroidia | rs74090642 | A | G | -0.113 | 0.024 | 2.72E-06 | 22.371 |
| class | Melainabacteria | rs10817461 | A | T | 0.088 | 0.019 | 2.56E-06 | 22.364 |
| class | Clostridia | rs2273429 | G | A | -0.072 | 0.015 | 4.52E-06 | 22.364 |
| class | Bacteroidia | rs11916046 | C | A | -0.063 | 0.013 | 1.79E-06 | 22.359 |
| class | Bacteroidia | rs1888899 | C | T | -0.112 | 0.024 | 3.35E-06 | 22.358 |
| class | Mollicutes | rs12335008 | T | A | 0.068 | 0.014 | 3.08E-06 | 22.358 |
| class | Betaproteobacteria | rs72747231 | G | C | -0.137 | 0.029 | 2.06E-06 | 22.357 |
| class | Actinobacteria | rs9926823 | A | C | 0.055 | 0.012 | 2.11E-06 | 22.355 |
| class | Actinobacteria | rs134332 | A | G | 0.111 | 0.024 | 1.91E-06 | 22.349 |
| class | Actinobacteria | rs2230437 | C | T | 0.054 | 0.011 | 3.27E-06 | 22.345 |
| class | Bacteroidia | rs17085809 | A | G | -0.113 | 0.024 | 2.60E-06 | 22.343 |
| class | Verrucomicrobiae | rs12908520 | A | G | 0.062 | 0.013 | 2.17E-06 | 22.340 |
| class | Actinobacteria | rs660002 | C | T | 0.069 | 0.015 | 2.37E-06 | 22.340 |
| class | Mollicutes | rs72901605 | C | T | -0.084 | 0.018 | 3.26E-06 | 22.338 |
| class | Bacteroidia | rs17085765 | G | C | -0.113 | 0.024 | 2.81E-06 | 22.332 |
| class | Actinobacteria | rs2413305 | G | A | 0.111 | 0.024 | 1.85E-06 | 22.328 |
| class | Actinobacteria | rs113974791 | T | G | 0.071 | 0.015 | 2.31E-06 | 22.328 |
| class | Bacilli | rs2009619 | G | A | -0.059 | 0.012 | 1.88E-06 | 22.317 |
| class | Actinobacteria | rs4889375 | A | C | 0.055 | 0.012 | 2.47E-06 | 22.314 |
| class | Actinobacteria | rs134286 | G | A | 0.111 | 0.023 | 1.67E-06 | 22.309 |
| class | Actinobacteria | rs10871420 | A | G | 0.055 | 0.012 | 2.38E-06 | 22.309 |
| class | Bacteroidia | rs1014718 | G | A | -0.056 | 0.012 | 2.21E-06 | 22.308 |
| class | Actinobacteria | rs78364332 | T | C | 0.071 | 0.015 | 2.51E-06 | 22.307 |
| class | Mollicutes | rs10099912 | G | T | 0.068 | 0.014 | 3.14E-06 | 22.301 |
| class | Bacteroidia | rs1022523 | A | G | -0.056 | 0.012 | 2.19E-06 | 22.297 |
| class | Betaproteobacteria | rs10788864 | A | T | 0.052 | 0.011 | 2.33E-06 | 22.286 |
| class | Mollicutes | rs9297995 | C | T | 0.067 | 0.014 | 3.00E-06 | 22.283 |
| class | Methanobacteria | rs6541968 | T | C | -0.111 | 0.024 | 2.96E-06 | 22.282 |
| class | Actinobacteria | rs112758679 | A | C | 0.071 | 0.015 | 2.35E-06 | 22.281 |
| class | Verrucomicrobiae | rs56161035 | C | T | 0.068 | 0.014 | 2.32E-06 | 22.280 |
| class | Bacteroidia | rs59082732 | T | G | -0.112 | 0.024 | 3.13E-06 | 22.268 |
| class | Actinobacteria | rs309171 | G | C | 0.071 | 0.015 | 2.44E-06 | 22.266 |
| class | Actinobacteria | rs11859935 | G | A | 0.055 | 0.012 | 2.50E-06 | 22.259 |
| class | Mollicutes | rs13025440 | T | G | 0.075 | 0.016 | 2.21E-06 | 22.254 |
| class | Bacilli | rs80327990 | A | G | -0.061 | 0.013 | 4.70E-06 | 22.241 |
| class | Actinobacteria | rs4954272 | A | G | 0.079 | 0.017 | 1.73E-06 | 22.235 |
| class | Actinobacteria | rs3099428 | C | G | 0.070 | 0.015 | 2.49E-06 | 22.234 |
| class | Actinobacteria | rs3099427 | A | G | 0.070 | 0.015 | 2.49E-06 | 22.233 |
| class | Methanobacteria | rs9594530 | T | C | 0.210 | 0.045 | 2.55E-06 | 22.227 |
| class | Bacteroidia | rs7319197 | A | G | -0.112 | 0.024 | 2.48E-06 | 22.207 |
| class | Actinobacteria | rs4988201 | A | G | 0.072 | 0.015 | 2.75E-06 | 22.206 |
| class | Bacteroidia | rs17085785 | C | T | -0.112 | 0.024 | 2.91E-06 | 22.204 |
| class | Bacteroidia | rs7320487 | T | C | -0.112 | 0.024 | 2.48E-06 | 22.204 |
| class | Mollicutes | rs10113750 | C | T | 0.067 | 0.014 | 3.15E-06 | 22.199 |
| class | Gammaproteobacteria | rs67074513 | T | C | 0.063 | 0.013 | 1.87E-06 | 22.196 |
| class | Actinobacteria | rs13387501 | C | T | 0.063 | 0.013 | 1.31E-06 | 22.193 |
| class | Clostridia | rs6508055 | G | A | -0.059 | 0.013 | 2.76E-06 | 22.192 |
| class | Bacteroidia | rs2875575 | G | C | -0.112 | 0.024 | 3.44E-06 | 22.187 |
| class | Actinobacteria | rs7561565 | T | G | 0.071 | 0.015 | 2.79E-06 | 22.180 |
| class | Gammaproteobacteria | rs61084716 | T | G | 0.069 | 0.015 | 1.52E-06 | 22.168 |
| class | Actinobacteria | rs134317 | G | A | 0.110 | 0.023 | 1.91E-06 | 22.166 |
| class | Actinobacteria | rs6430601 | G | T | 0.076 | 0.016 | 1.06E-06 | 22.165 |
| class | Gammaproteobacteria | rs17092798 | G | A | -0.064 | 0.014 | 1.89E-06 | 22.159 |
| class | Betaproteobacteria | rs11679161 | C | T | 0.062 | 0.013 | 2.25E-06 | 22.158 |
| class | Actinobacteria | rs309142 | T | C | 0.069 | 0.015 | 2.49E-06 | 22.158 |
| class | Mollicutes | rs7011095 | T | A | 0.068 | 0.014 | 3.41E-06 | 22.154 |
| class | Bacteroidia | rs35532963 | C | G | -0.062 | 0.013 | 2.01E-06 | 22.152 |
| class | Bacteroidia | rs36076607 | T | C | -0.062 | 0.013 | 1.90E-06 | 22.144 |
| class | Bacteroidia | rs75808613 | G | A | -0.112 | 0.024 | 2.81E-06 | 22.141 |
| class | Bacteroidia | rs78745799 | G | T | -0.112 | 0.024 | 2.81E-06 | 22.141 |
| class | Bacteroidia | rs1996069 | T | C | -0.063 | 0.013 | 1.99E-06 | 22.140 |
| class | Verrucomicrobiae | rs112769434 | A | G | 0.111 | 0.024 | 2.16E-06 | 22.139 |
| class | Actinobacteria | rs134316 | A | G | 0.110 | 0.023 | 1.81E-06 | 22.138 |
| class | Mollicutes | rs7009374 | C | T | 0.067 | 0.014 | 3.26E-06 | 22.138 |
| class | Actinobacteria | rs134307 | C | T | 0.110 | 0.023 | 1.86E-06 | 22.133 |
| class | Actinobacteria | rs11743825 | C | T | 0.054 | 0.012 | 3.75E-06 | 22.129 |
| class | Betaproteobacteria | rs2613606 | T | C | -0.051 | 0.011 | 2.20E-06 | 22.123 |
| class | Bacteroidia | rs71322866 | A | C | -0.063 | 0.013 | 1.93E-06 | 22.121 |
| class | Mollicutes | rs59979442 | A | G | 0.067 | 0.014 | 3.43E-06 | 22.119 |
| class | Betaproteobacteria | rs11683769 | A | T | 0.062 | 0.013 | 2.29E-06 | 22.115 |
| class | Mollicutes | rs13024040 | C | T | 0.074 | 0.016 | 2.13E-06 | 22.101 |
| class | Actinobacteria | rs2413308 | G | T | 0.110 | 0.023 | 1.85E-06 | 22.100 |
| class | Mollicutes | rs12676593 | A | G | 0.067 | 0.014 | 3.49E-06 | 22.092 |
| class | Bacteroidia | rs34519192 | A | G | -0.062 | 0.013 | 2.08E-06 | 22.080 |
| class | Actinobacteria | rs6518933 | A | G | 0.110 | 0.023 | 1.93E-06 | 22.079 |
| class | Actinobacteria | rs2413306 | A | G | 0.110 | 0.023 | 1.86E-06 | 22.077 |
| class | Lentisphaeria | rs17114848 | A | G | 0.152 | 0.032 | 4.06E-06 | 22.073 |
| class | Bacilli | rs80123130 | A | G | -0.060 | 0.013 | 5.23E-06 | 22.073 |
| class | Actinobacteria | rs79954860 | A | T | 0.109 | 0.023 | 2.33E-06 | 22.071 |
| class | Melainabacteria | rs4846956 | C | T | -0.088 | 0.019 | 2.15E-06 | 22.070 |
| class | Mollicutes | rs10090964 | A | T | 0.067 | 0.014 | 3.39E-06 | 22.065 |
| class | Betaproteobacteria | rs10047202 | C | T | 0.052 | 0.011 | 2.76E-06 | 22.047 |
| class | Methanobacteria | rs9942109 | G | A | -0.197 | 0.042 | 3.60E-06 | 22.046 |
| class | Methanobacteria | rs62240388 | A | G | -0.196 | 0.042 | 3.06E-06 | 22.046 |
| class | Bacteroidia | rs1555070 | C | T | -0.056 | 0.012 | 2.53E-06 | 22.036 |
| class | Bacteroidia | rs73846120 | A | G | -0.063 | 0.013 | 2.00E-06 | 22.035 |
| class | Negativicutes | rs35797647 | C | G | 0.050 | 0.011 | 2.47E-06 | 22.023 |
| class | Negativicutes | rs56371515 | G | C | 0.050 | 0.011 | 2.47E-06 | 22.023 |
| class | Gammaproteobacteria | rs7606905 | C | T | 0.068 | 0.015 | 1.48E-06 | 22.021 |
| class | Actinobacteria | rs2176718 | A | G | 0.057 | 0.012 | 2.45E-06 | 22.019 |
| class | Actinobacteria | rs6711997 | T | C | 0.063 | 0.013 | 1.42E-06 | 22.016 |
| class | Bacilli | rs4699544 | C | A | -0.060 | 0.013 | 5.35E-06 | 22.009 |
| class | Actinobacteria | rs12472293 | T | C | 0.070 | 0.015 | 2.79E-06 | 22.004 |
| class | Actinobacteria | rs12935228 | A | G | 0.054 | 0.012 | 2.57E-06 | 22.003 |
| class | Actinobacteria | rs35564151 | A | G | 0.054 | 0.011 | 1.87E-06 | 22.003 |
| class | Betaproteobacteria | rs1859743 | C | T | -0.051 | 0.011 | 2.20E-06 | 21.997 |
| class | Mollicutes | rs10094910 | T | C | 0.067 | 0.014 | 3.61E-06 | 21.991 |
| class | Actinobacteria | rs4072434 | G | C | 0.054 | 0.012 | 3.29E-06 | 21.973 |
| class | Actinobacteria | rs134294 | A | T | 0.110 | 0.023 | 1.92E-06 | 21.972 |
| class | Actinobacteria | . | C | T | 0.115 | 0.025 | 3.44E-06 | 21.967 |
| class | Actinobacteria | rs56146525 | A | C | 0.054 | 0.012 | 5.37E-06 | 21.964 |
| class | Negativicutes | rs35924322 | A | G | 0.050 | 0.011 | 2.58E-06 | 21.961 |
| class | Actinobacteria | rs309150 | T | A | 0.070 | 0.015 | 2.92E-06 | 21.960 |
| class | Actinobacteria | rs7581814 | C | G | 0.071 | 0.015 | 2.98E-06 | 21.955 |
| class | Erysipelotrichia | rs9859401 | A | C | 0.050 | 0.011 | 2.70E-06 | 21.954 |
| class | Verrucomicrobiae | rs1491244 | A | T | 0.111 | 0.024 | 2.47E-06 | 21.952 |
| class | Actinobacteria | rs134306 | G | A | 0.110 | 0.023 | 2.06E-06 | 21.950 |
| class | Bacteroidia | rs1325597 | G | T | -0.056 | 0.012 | 2.57E-06 | 21.946 |
| class | Negativicutes | rs9929374 | A | G | -0.050 | 0.011 | 3.11E-06 | 21.940 |
| class | Mollicutes | rs7590919 | G | A | 0.072 | 0.015 | 2.25E-06 | 21.936 |
| class | Gammaproteobacteria | rs7607028 | G | A | 0.068 | 0.015 | 1.64E-06 | 21.923 |
| class | Actinobacteria | rs79176913 | C | A | 0.071 | 0.015 | 3.14E-06 | 21.916 |
| class | Bacilli | rs78368499 | C | T | -0.102 | 0.022 | 2.81E-06 | 21.911 |
| class | Bacteroidia | rs34922683 | G | T | -0.062 | 0.013 | 2.32E-06 | 21.904 |
| class | Coriobacteriia | rs10995776 | C | T | 0.082 | 0.017 | 2.44E-06 | 21.903 |
| class | Methanobacteria | rs62240380 | C | T | -0.193 | 0.041 | 3.32E-06 | 21.900 |
| class | Actinobacteria | rs4988163 | G | T | 0.071 | 0.015 | 3.20E-06 | 21.893 |
| class | Erysipelotrichia | rs2300773 | A | G | 0.050 | 0.011 | 2.82E-06 | 21.891 |
| class | Bacteroidia | rs17085791 | G | C | -0.111 | 0.024 | 3.28E-06 | 21.890 |
| class | Actinobacteria | rs7599898 | C | T | 0.070 | 0.015 | 4.02E-06 | 21.887 |
| class | Bacteroidia | rs6794198 | C | T | -0.062 | 0.013 | 2.12E-06 | 21.879 |
| class | Actinobacteria | rs6750549 | A | G | 0.071 | 0.015 | 3.05E-06 | 21.879 |
| class | Bacteroidia | rs73975615 | A | G | -0.207 | 0.044 | 1.22E-06 | 21.874 |
| class | Deltaproteobacteria | rs16851319 | C | G | -0.071 | 0.015 | 5.68E-06 | 21.874 |
| class | Actinobacteria | rs503227 | T | C | 0.071 | 0.015 | 2.85E-06 | 21.872 |
| class | Actinobacteria | rs134295 | C | T | 0.110 | 0.023 | 1.95E-06 | 21.861 |
| class | Clostridia | rs11846847 | T | A | -0.072 | 0.015 | 6.15E-06 | 21.848 |
| class | Actinobacteria | rs55842567 | G | A | 0.054 | 0.012 | 5.35E-06 | 21.847 |
| class | Actinobacteria | rs4072435 | T | C | 0.054 | 0.012 | 3.40E-06 | 21.846 |
| class | Erysipelotrichia | rs9867473 | C | A | 0.050 | 0.011 | 2.36E-06 | 21.845 |
| class | Bacteroidia | rs2346325 | A | G | -0.062 | 0.013 | 2.40E-06 | 21.834 |
| class | Melainabacteria | rs73074665 | T | A | 0.166 | 0.036 | 2.87E-06 | 21.833 |
| class | Clostridia | rs13179700 | C | T | 0.051 | 0.011 | 3.37E-06 | 21.830 |
| class | Mollicutes | rs28809811 | G | T | 0.068 | 0.015 | 4.12E-06 | 21.828 |
| class | Actinobacteria | rs61253125 | T | C | 0.071 | 0.015 | 3.35E-06 | 21.813 |
| class | Bacteroidia | rs2183908 | T | C | -0.111 | 0.024 | 3.03E-06 | 21.811 |
| class | Bacteroidia | rs1040016 | G | T | -0.062 | 0.013 | 2.43E-06 | 21.810 |
| class | Actinobacteria | rs134289 | C | A | 0.110 | 0.023 | 2.12E-06 | 21.809 |
| class | Bacilli | rs60533369 | C | A | -0.060 | 0.013 | 5.91E-06 | 21.802 |
| class | Bacteroidia | rs11916939 | G | A | -0.062 | 0.013 | 2.39E-06 | 21.800 |
| class | Actinobacteria | rs139939202 | G | A | 0.072 | 0.015 | 4.33E-06 | 21.797 |
| class | Actinobacteria | rs134311 | G | A | 0.110 | 0.023 | 2.10E-06 | 21.790 |
| class | Actinobacteria | rs12928452 | C | A | 0.054 | 0.012 | 2.89E-06 | 21.787 |
| class | Bacteroidia | rs907713 | A | C | -0.062 | 0.013 | 2.24E-06 | 21.785 |
| class | Actinobacteria | rs10221893 | T | C | 0.054 | 0.012 | 3.44E-06 | 21.784 |
| class | Verrucomicrobiae | rs4242783 | A | G | 0.069 | 0.015 | 2.64E-06 | 21.781 |
| class | Verrucomicrobiae | rs2169719 | A | G | 0.068 | 0.015 | 3.06E-06 | 21.771 |
| class | Negativicutes | rs12113541 | A | G | 0.049 | 0.011 | 3.28E-06 | 21.763 |
| class | Actinobacteria | rs134308 | C | T | 0.109 | 0.023 | 2.17E-06 | 21.762 |
| class | Methanobacteria | rs58106718 | A | T | -0.193 | 0.041 | 3.43E-06 | 21.758 |
| class | Actinobacteria | rs76380759 | G | A | 0.070 | 0.015 | 4.34E-06 | 21.748 |
| class | Erysipelotrichia | rs13072608 | T | C | 0.050 | 0.011 | 3.05E-06 | 21.741 |
| class | Bacilli | rs11729948 | G | A | -0.060 | 0.013 | 6.14E-06 | 21.740 |
| class | Methanobacteria | rs62241832 | G | A | -0.196 | 0.042 | 4.58E-06 | 21.727 |
| class | Actinobacteria | rs3112496 | C | T | 0.070 | 0.015 | 2.79E-06 | 21.724 |
| class | Actinobacteria | rs4954559 | A | G | 0.060 | 0.013 | 3.80E-06 | 21.722 |
| class | Methanobacteria | rs56001610 | G | A | -0.195 | 0.042 | 4.49E-06 | 21.721 |
| class | Actinobacteria | rs62160866 | A | G | 0.118 | 0.025 | 1.51E-06 | 21.721 |
| class | Betaproteobacteria | rs1410861 | A | G | 0.051 | 0.011 | 3.29E-06 | 21.720 |
| class | Clostridia | rs11844758 | C | A | -0.071 | 0.015 | 6.03E-06 | 21.718 |
| class | Methanobacteria | rs75208022 | T | C | -0.227 | 0.049 | 5.92E-06 | 21.717 |
| class | Methanobacteria | rs62241845 | T | A | -0.195 | 0.042 | 4.55E-06 | 21.716 |
| class | Methanobacteria | rs62241844 | G | C | -0.195 | 0.042 | 4.55E-06 | 21.716 |
| class | Actinobacteria | rs134383 | A | G | 0.114 | 0.025 | 4.03E-06 | 21.715 |
| class | Actinobacteria | rs111837148 | G | A | 0.071 | 0.015 | 3.64E-06 | 21.708 |
| class | Lentisphaeria | rs11770843 | T | C | 0.109 | 0.023 | 1.91E-06 | 21.707 |
| class | Actinobacteria | rs2413309 | A | G | 0.109 | 0.023 | 2.08E-06 | 21.699 |
| class | Methanobacteria | rs3923545 | G | C | 0.207 | 0.045 | 3.20E-06 | 21.696 |
| class | Actinobacteria | rs134323 | A | C | 0.110 | 0.024 | 2.75E-06 | 21.692 |
| class | Actinobacteria | rs72711368 | C | T | 0.055 | 0.012 | 4.06E-06 | 21.691 |
| class | Mollicutes | rs11783174 | G | T | 0.067 | 0.014 | 4.33E-06 | 21.688 |
| class | Methanobacteria | rs62241837 | G | A | -0.195 | 0.042 | 4.61E-06 | 21.681 |
| class | Methanobacteria | rs62241838 | T | C | -0.195 | 0.042 | 4.61E-06 | 21.680 |
| class | Mollicutes | rs6981601 | C | T | 0.066 | 0.014 | 4.05E-06 | 21.679 |
| class | Actinobacteria | rs1438303 | C | T | 0.070 | 0.015 | 4.33E-06 | 21.679 |
| class | Actinobacteria | rs2289959 | C | T | 0.069 | 0.015 | 2.82E-06 | 21.677 |
| class | Betaproteobacteria | rs2094636 | C | G | 0.051 | 0.011 | 3.22E-06 | 21.675 |
| class | Bacilli | rs11110282 | G | A | -0.101 | 0.022 | 4.85E-06 | 21.669 |
| class | Actinobacteria | rs12899991 | T | A | 0.072 | 0.015 | 4.03E-06 | 21.668 |
| class | Bacteroidia | rs2346324 | A | G | -0.062 | 0.013 | 2.62E-06 | 21.667 |
| class | Deltaproteobacteria | rs4506934 | T | C | -0.094 | 0.020 | 3.59E-06 | 21.666 |
| class | Gammaproteobacteria | rs6469506 | A | T | 0.054 | 0.012 | 3.29E-06 | 21.661 |
| class | Alphaproteobacteria | rs10803434 | T | A | -0.069 | 0.015 | 3.55E-06 | 21.655 |
| class | Bacteroidia | rs3946805 | A | G | -0.062 | 0.013 | 2.60E-06 | 21.650 |
| class | Actinobacteria | rs134385 | T | C | 0.114 | 0.025 | 3.98E-06 | 21.650 |
| class | Methanobacteria | rs62241839 | A | G | -0.195 | 0.042 | 4.68E-06 | 21.650 |
| class | Actinobacteria | rs1056465 | T | A | 0.054 | 0.012 | 6.34E-06 | 21.639 |
| class | Bacteroidia | rs71322864 | C | A | -0.062 | 0.013 | 2.42E-06 | 21.637 |
| class | Actinobacteria | rs134288 | C | G | 0.109 | 0.023 | 2.42E-06 | 21.637 |
| class | Actinobacteria | rs3769016 | A | G | 0.070 | 0.015 | 4.57E-06 | 21.636 |
| class | Betaproteobacteria | rs62395635 | C | T | 0.110 | 0.024 | 2.94E-06 | 21.623 |
| class | Actinobacteria | rs56042615 | G | A | 0.054 | 0.012 | 6.08E-06 | 21.620 |
| class | Mollicutes | rs6986129 | A | T | 0.066 | 0.014 | 4.19E-06 | 21.619 |
| class | Actinobacteria | rs3099429 | T | A | 0.070 | 0.015 | 3.32E-06 | 21.614 |
| class | Mollicutes | rs1583269 | C | G | 0.072 | 0.015 | 2.62E-06 | 21.611 |
| class | Actinobacteria | rs2413310 | A | T | 0.109 | 0.023 | 2.21E-06 | 21.603 |
| class | Melainabacteria | rs79790072 | C | T | 0.227 | 0.049 | 3.29E-06 | 21.599 |
| class | Erysipelotrichia | rs55764236 | T | G | -0.054 | 0.012 | 3.89E-06 | 21.598 |
| class | Coriobacteriia | rs34739816 | T | G | 0.097 | 0.021 | 3.88E-06 | 21.594 |
| class | Erysipelotrichia | rs3761717 | C | G | 0.050 | 0.011 | 3.30E-06 | 21.588 |
| class | Actinobacteria | rs134293 | C | T | 0.109 | 0.023 | 2.33E-06 | 21.585 |
| class | Mollicutes | rs13027493 | T | A | 0.071 | 0.015 | 2.71E-06 | 21.580 |
| class | Gammaproteobacteria | rs1849782 | G | A | -0.063 | 0.014 | 2.43E-06 | 21.570 |
| class | Erysipelotrichia | rs7095708 | T | C | -0.053 | 0.011 | 4.01E-06 | 21.568 |
| class | Actinobacteria | rs1435577 | C | G | 0.070 | 0.015 | 3.83E-06 | 21.566 |
| class | Actinobacteria | rs134285 | T | C | 0.109 | 0.023 | 2.47E-06 | 21.558 |
| class | Actinobacteria | rs6430612 | T | C | 0.054 | 0.012 | 4.46E-06 | 21.557 |
| class | Gammaproteobacteria | rs1429930 | C | G | 0.069 | 0.015 | 2.15E-06 | 21.557 |
| class | Alphaproteobacteria | rs10803433 | G | T | -0.069 | 0.015 | 3.60E-06 | 21.555 |
| class | Verrucomicrobiae | rs11252894 | C | A | 0.077 | 0.017 | 2.94E-06 | 21.555 |
| class | Negativicutes | rs34884022 | A | G | 0.049 | 0.011 | 3.60E-06 | 21.553 |
| class | Erysipelotrichia | rs4617508 | T | A | -0.053 | 0.011 | 4.04E-06 | 21.541 |
| class | Negativicutes | rs35636125 | A | G | 0.049 | 0.011 | 3.21E-06 | 21.535 |
| class | Actinobacteria | rs4888172 | G | A | 0.054 | 0.012 | 3.35E-06 | 21.528 |
| class | Coriobacteriia | rs11250875 | C | T | 0.061 | 0.013 | 4.83E-06 | 21.526 |
| class | Erysipelotrichia | rs7911287 | G | T | -0.053 | 0.011 | 4.03E-06 | 21.524 |
| class | Gammaproteobacteria | rs12545601 | A | C | 0.054 | 0.012 | 3.66E-06 | 21.520 |
| class | Betaproteobacteria | rs6686895 | A | G | 0.051 | 0.011 | 3.66E-06 | 21.515 |
| class | Bacilli | rs78938557 | C | T | 0.108 | 0.023 | 1.07E-06 | 21.514 |
| class | Erysipelotrichia | rs11718657 | A | C | 0.050 | 0.011 | 3.40E-06 | 21.513 |
| class | Erysipelotrichia | rs11185506 | C | G | 0.050 | 0.011 | 3.40E-06 | 21.513 |
| class | Bacteroidia | rs13291434 | C | T | 0.069 | 0.015 | 4.28E-06 | 21.507 |
| class | Actinobacteria | rs134301 | T | C | 0.109 | 0.023 | 2.44E-06 | 21.506 |
| class | Betaproteobacteria | rs1511453 | G | A | 0.092 | 0.020 | 4.76E-06 | 21.502 |
| class | Actinobacteria | rs56104584 | T | C | 0.054 | 0.012 | 6.37E-06 | 21.500 |
| class | Actinobacteria | rs73957037 | C | A | 0.068 | 0.015 | 5.55E-06 | 21.494 |
| class | Bacteroidia | rs13291169 | G | C | 0.069 | 0.015 | 3.75E-06 | 21.493 |
| class | Actinobacteria | rs309172 | A | G | 0.069 | 0.015 | 2.93E-06 | 21.491 |
| class | Deltaproteobacteria | rs11599763 | C | T | -0.054 | 0.012 | 3.94E-06 | 21.490 |
| class | Actinobacteria | rs134300 | G | A | 0.109 | 0.023 | 2.43E-06 | 21.486 |
| class | Coriobacteriia | rs67561917 | G | A | -0.071 | 0.015 | 5.39E-06 | 21.486 |
| class | Gammaproteobacteria | rs4421375 | C | T | 0.054 | 0.012 | 3.59E-06 | 21.485 |
| class | Actinobacteria | rs4594504 | A | C | 0.070 | 0.015 | 4.00E-06 | 21.480 |
| class | Mollicutes | rs10111052 | T | G | 0.066 | 0.014 | 4.70E-06 | 21.479 |
| class | Methanobacteria | rs7635580 | G | A | -0.193 | 0.042 | 4.53E-06 | 21.474 |
| class | Actinobacteria | rs134290 | C | G | 0.109 | 0.023 | 2.44E-06 | 21.472 |
| class | Actinobacteria | rs134291 | G | A | 0.109 | 0.023 | 2.44E-06 | 21.472 |
| class | Actinobacteria | rs134339 | G | A | 0.109 | 0.024 | 3.12E-06 | 21.470 |
| class | Erysipelotrichia | rs12357537 | T | C | -0.054 | 0.012 | 4.15E-06 | 21.470 |
| class | Actinobacteria | rs72717432 | G | A | 0.054 | 0.012 | 5.72E-06 | 21.469 |
| class | Actinobacteria | rs2883034 | A | G | 0.062 | 0.013 | 2.61E-06 | 21.466 |
| class | Actinobacteria | rs134287 | A | C | 0.109 | 0.023 | 2.68E-06 | 21.461 |
| class | Negativicutes | rs1447205 | C | G | -0.050 | 0.011 | 2.93E-06 | 21.458 |
| class | Bacteroidia | rs34046877 | T | C | -0.061 | 0.013 | 2.67E-06 | 21.456 |
| class | Melainabacteria | rs7549147 | G | A | -0.086 | 0.019 | 3.13E-06 | 21.437 |
| class | Bacteroidia | rs12496485 | G | A | -0.062 | 0.013 | 2.72E-06 | 21.431 |
| class | Negativicutes | rs12112489 | C | T | 0.049 | 0.011 | 3.84E-06 | 21.430 |
| class | Actinobacteria | rs309154 | C | T | 0.071 | 0.015 | 4.39E-06 | 21.426 |
| class | Melainabacteria | rs202201936 | C | A | -0.141 | 0.031 | 6.88E-06 | 21.420 |
| class | Erysipelotrichia | rs9859260 | T | C | 0.049 | 0.011 | 3.63E-06 | 21.405 |
| class | Bacteroidia | rs13100590 | T | C | -0.061 | 0.013 | 3.00E-06 | 21.404 |
| class | Clostridia | rs10774377 | G | A | 0.053 | 0.011 | 3.24E-06 | 21.390 |
| class | Actinobacteria | rs895285 | A | G | 0.072 | 0.015 | 3.87E-06 | 21.382 |
| class | Actinobacteria | rs134407 | G | A | 0.113 | 0.025 | 4.34E-06 | 21.380 |
| class | Gammaproteobacteria | rs10497315 | A | G | 0.067 | 0.014 | 2.08E-06 | 21.377 |
| class | Mollicutes | rs6043847 | C | T | -0.115 | 0.025 | 4.55E-06 | 21.375 |
| class | Gammaproteobacteria | rs7569147 | G | A | 0.065 | 0.014 | 3.01E-06 | 21.363 |
| class | Negativicutes | rs56188349 | C | T | -0.064 | 0.014 | 4.41E-06 | 21.360 |
| class | Bacteroidia | rs17026797 | G | T | -0.061 | 0.013 | 3.07E-06 | 21.353 |
| class | Actinobacteria | rs134305 | G | A | 0.108 | 0.023 | 2.88E-06 | 21.352 |
| class | Bacteroidia | rs17026821 | T | C | -0.061 | 0.013 | 3.09E-06 | 21.345 |
| class | Actinobacteria | rs6753232 | T | C | 0.070 | 0.015 | 5.00E-06 | 21.344 |
| class | Gammaproteobacteria | rs1399856 | C | A | 0.054 | 0.012 | 3.96E-06 | 21.337 |
| class | Verrucomicrobiae | rs6832119 | A | G | 0.109 | 0.024 | 3.44E-06 | 21.336 |
| class | Lentisphaeria | rs2731834 | G | C | 0.109 | 0.024 | 4.24E-06 | 21.335 |
| class | Erysipelotrichia | rs9877119 | G | A | 0.049 | 0.011 | 3.74E-06 | 21.332 |
| class | Bacteroidia | rs35176045 | C | T | -0.061 | 0.013 | 2.70E-06 | 21.330 |
| class | Actinobacteria | rs72767435 | C | T | -0.126 | 0.027 | 2.57E-06 | 21.323 |
| class | Actinobacteria | rs134292 | A | G | 0.108 | 0.023 | 2.80E-06 | 21.302 |
| class | Actinobacteria | rs12187440 | G | A | 0.053 | 0.012 | 6.65E-06 | 21.290 |
| class | Actinobacteria | rs134304 | T | C | 0.108 | 0.023 | 2.86E-06 | 21.290 |
| class | Actinobacteria | rs60253740 | G | T | 0.070 | 0.015 | 6.71E-06 | 21.290 |
| class | Bacilli | rs2975635 | C | T | -0.056 | 0.012 | 3.58E-06 | 21.284 |
| class | Actinobacteria | rs4312299 | G | C | 0.054 | 0.012 | 3.81E-06 | 21.281 |
| class | Gammaproteobacteria | rs12619579 | G | A | 0.067 | 0.014 | 2.18E-06 | 21.281 |
| class | Methanobacteria | rs11123059 | A | G | -0.108 | 0.023 | 4.87E-06 | 21.279 |
| class | Methanobacteria | rs62241827 | T | G | -0.193 | 0.042 | 5.35E-06 | 21.274 |
| class | Actinobacteria | rs6889494 | C | T | 0.053 | 0.012 | 6.32E-06 | 21.268 |
| class | Methanobacteria | rs62241829 | C | T | -0.193 | 0.042 | 5.39E-06 | 21.263 |
| class | Actinobacteria | rs2413312 | G | A | 0.108 | 0.023 | 3.22E-06 | 21.255 |
| class | Verrucomicrobiae | rs111862613 | C | T | 0.091 | 0.020 | 3.74E-06 | 21.252 |
| class | Erysipelotrichia | rs8003149 | T | C | 0.054 | 0.012 | 4.08E-06 | 21.248 |
| class | Methanobacteria | rs62241828 | A | G | -0.193 | 0.042 | 5.45E-06 | 21.243 |
| class | Actinobacteria | rs134309 | G | A | 0.108 | 0.023 | 3.03E-06 | 21.241 |
| class | Negativicutes | rs72643860 | A | G | -0.050 | 0.011 | 3.35E-06 | 21.239 |
| class | Negativicutes | rs61249479 | C | A | 0.078 | 0.017 | 2.95E-06 | 21.236 |
| class | Actinobacteria | rs4888173 | A | G | 0.053 | 0.012 | 3.88E-06 | 21.235 |
| class | Bacilli | rs2131006 | A | G | -0.090 | 0.020 | 7.23E-06 | 21.233 |
| class | Verrucomicrobiae | rs11184341 | C | G | 0.066 | 0.014 | 4.13E-06 | 21.230 |
| class | Actinobacteria | rs13024450 | T | C | 0.054 | 0.012 | 4.77E-06 | 21.224 |
| class | Verrucomicrobiae | rs77055143 | C | T | 0.067 | 0.014 | 3.88E-06 | 21.222 |
| class | Betaproteobacteria | rs9964679 | G | A | 0.053 | 0.011 | 4.85E-06 | 21.218 |
| class | Verrucomicrobiae | rs12416161 | C | T | 0.076 | 0.017 | 3.50E-06 | 21.210 |
| class | Bacilli | rs4459992 | C | T | 0.054 | 0.012 | 4.30E-06 | 21.207 |
| class | Actinobacteria | rs134302 | T | C | 0.108 | 0.023 | 3.04E-06 | 21.203 |
| class | Betaproteobacteria | rs11128180 | G | A | 0.059 | 0.013 | 3.67E-06 | 21.203 |
| class | Bacilli | rs113280052 | T | C | -0.103 | 0.022 | 4.48E-06 | 21.203 |
| class | Actinobacteria | rs134310 | T | A | 0.108 | 0.023 | 2.92E-06 | 21.202 |
| class | Actinobacteria | rs134377 | G | A | 0.108 | 0.024 | 3.40E-06 | 21.200 |
| class | Actinobacteria | rs309157 | G | A | 0.071 | 0.015 | 4.60E-06 | 21.199 |
| class | Mollicutes | rs12476593 | C | G | 0.073 | 0.016 | 3.84E-06 | 21.194 |
| class | Gammaproteobacteria | rs61926436 | A | C | -0.062 | 0.014 | 3.10E-06 | 21.187 |
| class | Alphaproteobacteria | . | A | C | -0.069 | 0.015 | 4.36E-06 | 21.184 |
| class | Bacteroidia | rs13060907 | G | A | -0.061 | 0.013 | 3.17E-06 | 21.181 |
| class | Methanobacteria | rs55721046 | A | G | -0.191 | 0.041 | 6.06E-06 | 21.171 |
| class | Actinobacteria | rs134282 | A | G | 0.108 | 0.023 | 3.07E-06 | 21.168 |
| class | Actinobacteria | rs34847072 | G | C | 0.053 | 0.012 | 7.60E-06 | 21.166 |
| class | Bacilli | rs9445784 | C | T | -0.100 | 0.022 | 4.05E-06 | 21.162 |
| class | Verrucomicrobiae | rs12498518 | C | T | 0.067 | 0.014 | 3.94E-06 | 21.153 |
| class | Actinobacteria | rs115554641 | C | G | 0.053 | 0.012 | 7.53E-06 | 21.150 |
| class | Bacteroidia | rs6777119 | T | C | -0.061 | 0.013 | 3.35E-06 | 21.148 |
| class | Bacteroidia | rs73510192 | T | C | -0.111 | 0.024 | 4.18E-06 | 21.147 |
| class | Actinobacteria | rs134389 | T | G | 0.113 | 0.025 | 4.76E-06 | 21.147 |
| class | Gammaproteobacteria | rs6984998 | C | T | -0.056 | 0.012 | 3.79E-06 | 21.141 |
| class | Gammaproteobacteria | rs1346722 | T | C | 0.061 | 0.013 | 3.25E-06 | 21.131 |
| class | Bacilli | rs12368593 | A | C | -0.101 | 0.022 | 6.18E-06 | 21.130 |
| class | Actinobacteria | rs72717440 | A | T | 0.053 | 0.012 | 6.96E-06 | 21.129 |
| class | Betaproteobacteria | rs1122678 | T | C | -0.050 | 0.011 | 3.76E-06 | 21.126 |
| class | Actinobacteria | rs134374 | T | C | 0.108 | 0.024 | 3.50E-06 | 21.115 |
| class | Betaproteobacteria | rs2528680 | C | T | -0.050 | 0.011 | 3.74E-06 | 21.114 |
| class | Negativicutes | rs34660854 | G | A | 0.061 | 0.013 | 4.26E-06 | 21.114 |
| class | Deltaproteobacteria | rs55640204 | A | G | -0.092 | 0.020 | 4.81E-06 | 21.113 |
| class | Gammaproteobacteria | rs7000905 | C | T | 0.053 | 0.012 | 4.39E-06 | 21.094 |
| class | Mollicutes | rs13015145 | G | A | 0.071 | 0.015 | 3.45E-06 | 21.094 |
| class | Bacteroidia | rs11871360 | A | G | -0.203 | 0.044 | 1.86E-06 | 21.080 |
| class | Actinobacteria | rs134376 | A | G | 0.108 | 0.024 | 3.64E-06 | 21.080 |
| class | Actinobacteria | rs10200263 | T | C | 0.053 | 0.012 | 5.24E-06 | 21.074 |
| class | Bacteroidia | rs17343978 | C | A | -0.055 | 0.012 | 8.36E-06 | 21.067 |
| class | Actinobacteria | rs134373 | C | T | 0.108 | 0.023 | 3.45E-06 | 21.053 |
| class | Methanobacteria | rs12303159 | C | A | -0.223 | 0.049 | 8.56E-06 | 21.045 |
| class | Actinobacteria | rs59413285 | G | T | 0.069 | 0.015 | 5.73E-06 | 21.042 |
| class | Erysipelotrichia | rs17530232 | G | A | 0.103 | 0.022 | 2.79E-06 | 21.042 |
| class | Betaproteobacteria | rs8119189 | C | A | -0.093 | 0.020 | 4.31E-06 | 21.039 |
| class | Actinobacteria | rs2034261 | G | T | 0.052 | 0.011 | 3.52E-06 | 21.036 |
| class | Actinobacteria | rs6708336 | T | A | 0.052 | 0.011 | 3.60E-06 | 21.034 |
| class | Betaproteobacteria | rs56386628 | T | C | -0.062 | 0.014 | 5.87E-06 | 21.031 |
| class | Verrucomicrobiae | rs12501826 | G | C | 0.066 | 0.014 | 4.30E-06 | 21.016 |
| class | Gammaproteobacteria | rs12675001 | G | T | 0.053 | 0.012 | 5.10E-06 | 21.011 |
| class | Bacteroidia | rs6791217 | G | T | -0.061 | 0.013 | 3.44E-06 | 21.010 |
| class | Actinobacteria | rs34453673 | G | C | 0.053 | 0.012 | 7.50E-06 | 20.997 |
| class | Verrucomicrobiae | rs6832927 | C | A | 0.108 | 0.024 | 4.23E-06 | 20.989 |
| class | Actinobacteria | rs134347 | C | T | 0.108 | 0.023 | 3.86E-06 | 20.981 |
| class | Actinobacteria | rs57530667 | A | G | 0.069 | 0.015 | 5.90E-06 | 20.980 |
| class | Actinobacteria | rs62168830 | A | T | 0.069 | 0.015 | 5.90E-06 | 20.979 |
| class | Clostridia | rs6126494 | T | G | -0.097 | 0.021 | 9.12E-06 | 20.967 |
| class | Actinobacteria | rs73958638 | A | G | 0.106 | 0.023 | 2.70E-06 | 20.966 |
| class | Clostridia | rs4764517 | T | C | 0.052 | 0.011 | 3.97E-06 | 20.961 |
| class | Actinobacteria | rs11150406 | T | C | 0.053 | 0.012 | 4.61E-06 | 20.956 |
| class | Actinobacteria | rs62168897 | C | T | 0.052 | 0.011 | 3.77E-06 | 20.954 |
| class | Bacilli | rs11248956 | T | C | 0.058 | 0.013 | 3.22E-06 | 20.952 |
| class | Verrucomicrobiae | rs11724791 | G | A | 0.066 | 0.014 | 4.86E-06 | 20.946 |
| class | Actinobacteria | rs11742144 | G | A | 0.053 | 0.012 | 6.91E-06 | 20.945 |
| class | Bacilli | rs9345899 | G | A | -0.080 | 0.018 | 6.67E-06 | 20.943 |
| class | Gammaproteobacteria | rs10467189 | T | C | -0.062 | 0.014 | 3.46E-06 | 20.937 |
| class | Methanobacteria | rs62241826 | T | C | -0.190 | 0.041 | 6.95E-06 | 20.937 |
| class | Gammaproteobacteria | rs12404135 | G | A | -0.079 | 0.017 | 8.89E-06 | 20.933 |
| class | Betaproteobacteria | rs36119524 | C | T | 0.061 | 0.013 | 4.40E-06 | 20.923 |
| class | Deltaproteobacteria | rs9928243 | A | C | -0.054 | 0.012 | 5.02E-06 | 20.923 |
| class | Actinobacteria | rs3814355 | C | T | 0.052 | 0.011 | 3.58E-06 | 20.919 |
| class | Bacteroidia | rs1996068 | A | G | -0.060 | 0.013 | 3.94E-06 | 20.911 |
| class | Erysipelotrichia | rs3804140 | C | T | 0.049 | 0.011 | 4.68E-06 | 20.907 |
| class | Gammaproteobacteria | rs13281859 | C | T | 0.053 | 0.012 | 5.21E-06 | 20.901 |
| class | Betaproteobacteria | rs1544475 | C | T | -0.050 | 0.011 | 4.22E-06 | 20.901 |
| class | Actinobacteria | rs309113 | C | A | 0.068 | 0.015 | 4.50E-06 | 20.899 |
| class | Actinobacteria | rs75524146 | C | T | 0.071 | 0.015 | 5.28E-06 | 20.897 |
| class | Bacilli | rs80243853 | A | G | -0.101 | 0.022 | 7.15E-06 | 20.892 |
| class | Betaproteobacteria | rs6087810 | C | T | -0.093 | 0.020 | 4.82E-06 | 20.889 |
| class | Mollicutes | rs3768491 | G | A | -0.068 | 0.015 | 4.23E-06 | 20.875 |
| class | Actinobacteria | rs11739543 | G | A | 0.053 | 0.012 | 8.02E-06 | 20.869 |
| class | Actinobacteria | rs10165053 | T | C | 0.053 | 0.012 | 6.00E-06 | 20.866 |
| class | Gammaproteobacteria | rs12089016 | C | A | -0.079 | 0.017 | 9.53E-06 | 20.863 |
| class | Verrucomicrobiae | rs59317921 | C | T | 0.063 | 0.014 | 4.62E-06 | 20.858 |
| class | Verrucomicrobiae | rs1605923 | T | G | 0.066 | 0.014 | 4.84E-06 | 20.856 |
| class | Deltaproteobacteria | rs55744759 | G | A | -0.078 | 0.017 | 7.31E-06 | 20.854 |
| class | Gammaproteobacteria | rs4281140 | G | T | 0.053 | 0.012 | 5.41E-06 | 20.847 |
| class | Mollicutes | rs3739327 | G | A | 0.066 | 0.014 | 5.75E-06 | 20.843 |
| class | Actinobacteria | rs16832417 | C | T | 0.071 | 0.015 | 6.58E-06 | 20.835 |
| class | Betaproteobacteria | rs2040996 | C | T | -0.050 | 0.011 | 4.66E-06 | 20.826 |
| class | Mollicutes | rs78169027 | G | A | -0.108 | 0.024 | 5.88E-06 | 20.824 |
| class | Melainabacteria | rs28678345 | C | T | 0.215 | 0.047 | 6.69E-06 | 20.818 |
| class | Negativicutes | rs56249650 | C | A | 0.048 | 0.011 | 5.35E-06 | 20.813 |
| class | Mollicutes | rs10097590 | G | A | 0.067 | 0.015 | 8.52E-06 | 20.812 |
| class | Bacteroidia | rs73510188 | G | A | -0.110 | 0.024 | 4.75E-06 | 20.810 |
| class | Bacteroidia | rs73510190 | G | C | -0.110 | 0.024 | 4.75E-06 | 20.810 |
| class | Actinobacteria | rs134419 | T | C | 0.112 | 0.025 | 5.96E-06 | 20.809 |
| class | Gammaproteobacteria | rs12679016 | A | T | 0.053 | 0.012 | 5.69E-06 | 20.808 |
| class | Verrucomicrobiae | rs6668273 | G | A | 0.065 | 0.014 | 5.26E-06 | 20.805 |
| class | Actinobacteria | rs62168833 | G | A | 0.069 | 0.015 | 6.81E-06 | 20.800 |
| class | Bacilli | rs11110281 | C | T | -0.101 | 0.022 | 7.39E-06 | 20.799 |
| class | Actinobacteria | rs134283 | G | A | 0.107 | 0.023 | 3.90E-06 | 20.798 |
| class | Negativicutes | rs4236917 | G | C | -0.049 | 0.011 | 4.15E-06 | 20.795 |
| class | Clostridia | rs9986136 | G | A | 0.050 | 0.011 | 5.34E-06 | 20.794 |
| class | Negativicutes | rs4736971 | C | A | -0.049 | 0.011 | 4.19E-06 | 20.794 |
| class | Actinobacteria | rs77985886 | C | T | 0.088 | 0.019 | 7.32E-06 | 20.792 |
| class | Verrucomicrobiae | rs113653192 | C | T | 0.108 | 0.024 | 4.68E-06 | 20.789 |
| class | Betaproteobacteria | rs7638039 | C | T | 0.058 | 0.013 | 5.39E-06 | 20.788 |
| class | Verrucomicrobiae | rs72663787 | G | A | 0.066 | 0.015 | 5.08E-06 | 20.783 |
| class | Bacilli | rs2271029 | C | A | 0.050 | 0.011 | 5.33E-06 | 20.783 |
| class | Mollicutes | rs10085973 | C | G | 0.065 | 0.014 | 6.68E-06 | 20.779 |
| class | Actinobacteria | rs134381 | C | T | 0.107 | 0.024 | 3.68E-06 | 20.778 |
| class | Gammaproteobacteria | rs13266408 | T | C | 0.053 | 0.012 | 5.78E-06 | 20.777 |
| class | Deltaproteobacteria | rs72647048 | C | T | -0.078 | 0.017 | 7.32E-06 | 20.772 |
| class | Betaproteobacteria | rs2528681 | G | C | -0.050 | 0.011 | 4.53E-06 | 20.759 |
| class | Erysipelotrichia | rs3804139 | C | T | 0.049 | 0.011 | 5.08E-06 | 20.748 |
| class | Gammaproteobacteria | rs9973122 | A | T | 0.074 | 0.016 | 6.77E-06 | 20.747 |
| class | Alphaproteobacteria | rs12740709 | C | A | -0.068 | 0.015 | 5.42E-06 | 20.746 |
| class | Mollicutes | rs12711448 | T | C | -0.068 | 0.015 | 4.51E-06 | 20.744 |
| class | Mollicutes | rs4282557 | C | T | 0.065 | 0.014 | 6.79E-06 | 20.736 |
| class | Actinobacteria | rs68005421 | G | C | 0.054 | 0.012 | 6.68E-06 | 20.732 |
| class | Melainabacteria | rs12746891 | A | G | -0.085 | 0.019 | 4.43E-06 | 20.731 |
| class | Deltaproteobacteria | rs7936267 | G | A | -0.056 | 0.012 | 9.72E-06 | 20.731 |
| class | Actinobacteria | rs2322813 | A | G | 0.069 | 0.015 | 7.54E-06 | 20.730 |
| class | Mollicutes | rs10099240 | G | C | 0.065 | 0.014 | 6.96E-06 | 20.730 |
| class | Actinobacteria | rs72717436 | C | A | 0.053 | 0.012 | 8.84E-06 | 20.725 |
| class | Coriobacteriia | rs80046645 | G | C | 0.256 | 0.056 | 4.66E-06 | 20.724 |
| class | Erysipelotrichia | rs4078432 | T | C | -0.061 | 0.013 | 4.23E-06 | 20.723 |
| class | Methanobacteria | rs62240390 | C | G | -0.189 | 0.042 | 6.79E-06 | 20.722 |
| class | Methanobacteria | rs73248946 | A | G | -0.222 | 0.049 | 9.83E-06 | 20.722 |
| class | Clostridia | rs10774378 | C | T | 0.052 | 0.011 | 4.68E-06 | 20.715 |
| class | Betaproteobacteria | rs2528678 | C | T | -0.050 | 0.011 | 4.62E-06 | 20.712 |
| class | Actinobacteria | rs12475398 | C | T | 0.069 | 0.015 | 8.23E-06 | 20.709 |
| class | Bacilli | rs7764486 | A | G | -0.099 | 0.022 | 6.30E-06 | 20.709 |
| class | Betaproteobacteria | rs4591362 | G | A | 0.061 | 0.013 | 4.44E-06 | 20.707 |
| class | Negativicutes | rs2354569 | T | C | -0.049 | 0.011 | 4.39E-06 | 20.707 |
| class | Actinobacteria | rs13018756 | T | C | 0.053 | 0.012 | 6.07E-06 | 20.704 |
| class | Actinobacteria | rs3814354 | G | A | 0.052 | 0.011 | 4.38E-06 | 20.702 |
| class | Clostridia | rs72738886 | C | T | 0.087 | 0.019 | 8.24E-06 | 20.697 |
| class | Mollicutes | rs638542 | A | G | -0.068 | 0.015 | 4.49E-06 | 20.696 |
| class | Bacteroidia | rs60475411 | T | C | -0.060 | 0.013 | 4.32E-06 | 20.694 |
| class | Actinobacteria | rs10166142 | A | G | 0.052 | 0.011 | 4.42E-06 | 20.693 |
| class | Verrucomicrobiae | rs3995795 | T | C | 0.064 | 0.014 | 5.19E-06 | 20.686 |
| class | Erysipelotrichia | rs34221479 | C | T | 0.049 | 0.011 | 5.79E-06 | 20.675 |
| class | Clostridia | rs7269302 | T | A | -0.096 | 0.021 | 9.04E-06 | 20.675 |
| class | Actinobacteria | rs3769001 | A | G | 0.069 | 0.015 | 6.06E-06 | 20.675 |
| class | Bacteroidia | rs35665203 | T | C | -0.061 | 0.013 | 4.04E-06 | 20.672 |
| class | Actinobacteria | rs12446319 | T | C | 0.051 | 0.011 | 5.24E-06 | 20.672 |
| class | Methanobacteria | rs56131665 | A | G | 0.179 | 0.039 | 6.18E-06 | 20.669 |
| class | Actinobacteria | rs12188678 | T | C | 0.053 | 0.012 | 8.21E-06 | 20.667 |
| class | Gammaproteobacteria | rs9494710 | T | C | -0.055 | 0.012 | 4.55E-06 | 20.663 |
| class | Lentisphaeria | rs73113483 | A | T | -0.131 | 0.029 | 8.66E-06 | 20.656 |
| class | Coriobacteriia | rs1816223 | G | A | -0.059 | 0.013 | 4.84E-06 | 20.652 |
| class | Erysipelotrichia | rs9990392 | T | C | 0.049 | 0.011 | 4.94E-06 | 20.647 |
| class | Actinobacteria | rs72717435 | G | A | 0.052 | 0.012 | 9.34E-06 | 20.633 |
| class | Gammaproteobacteria | rs6994489 | C | A | -0.055 | 0.012 | 5.37E-06 | 20.629 |
| class | Negativicutes | rs9423647 | A | G | 0.048 | 0.011 | 6.06E-06 | 20.628 |
| class | Bacilli | rs7959495 | A | C | -0.099 | 0.022 | 8.73E-06 | 20.624 |
| class | Actinobacteria | rs12188164 | C | A | 0.053 | 0.012 | 8.43E-06 | 20.622 |
| class | Gammaproteobacteria | rs11775846 | G | A | 0.053 | 0.012 | 6.07E-06 | 20.620 |
| class | Actinobacteria | rs1533649 | G | A | 0.059 | 0.013 | 8.18E-06 | 20.620 |
| class | Actinobacteria | rs2289963 | G | A | 0.088 | 0.019 | 8.06E-06 | 20.612 |
| class | Betaproteobacteria | rs976935 | C | A | -0.050 | 0.011 | 4.89E-06 | 20.612 |
| class | Actinobacteria | rs134330 | G | C | 0.107 | 0.024 | 4.97E-06 | 20.611 |
| class | Erysipelotrichia | rs11146160 | A | G | -0.052 | 0.011 | 6.47E-06 | 20.611 |
| class | Bacilli | rs11110276 | G | T | -0.099 | 0.022 | 8.19E-06 | 20.608 |
| class | Betaproteobacteria | rs4507125 | A | C | 0.060 | 0.013 | 5.73E-06 | 20.603 |
| class | Actinobacteria | rs79633114 | C | T | 0.069 | 0.015 | 8.26E-06 | 20.590 |
| class | Mollicutes | rs6867857 | C | A | 0.122 | 0.027 | 2.42E-06 | 20.589 |
| class | Deltaproteobacteria | rs1035691 | G | A | -0.055 | 0.012 | 9.65E-06 | 20.588 |
| class | Gammaproteobacteria | rs10111899 | C | T | -0.055 | 0.012 | 5.46E-06 | 20.578 |
| class | Actinobacteria | rs2278682 | G | C | 0.068 | 0.015 | 5.69E-06 | 20.571 |
| class | Betaproteobacteria | rs76625259 | T | G | -0.091 | 0.020 | 5.39E-06 | 20.570 |
| class | Gammaproteobacteria | rs998899 | C | T | 0.060 | 0.013 | 4.14E-06 | 20.565 |
| class | Betaproteobacteria | rs320161 | G | A | 0.057 | 0.013 | 7.33E-06 | 20.564 |
| class | Actinobacteria | rs4988274 | T | A | 0.070 | 0.015 | 6.56E-06 | 20.553 |
| class | Mollicutes | rs4970749 | A | G | -0.068 | 0.015 | 5.01E-06 | 20.550 |
| class | Actinobacteria | rs134391 | C | T | 0.107 | 0.024 | 3.90E-06 | 20.549 |
| class | Actinobacteria | rs11743639 | C | T | 0.052 | 0.012 | 9.44E-06 | 20.549 |
| class | Verrucomicrobiae | rs72663782 | G | C | 0.066 | 0.014 | 5.35E-06 | 20.548 |
| class | Actinobacteria | rs72717410 | A | T | 0.052 | 0.012 | 8.94E-06 | 20.546 |
| class | Actinobacteria | rs56271357 | C | T | 0.051 | 0.011 | 4.66E-06 | 20.537 |
| class | Lentisphaeria | rs11764871 | T | G | 0.106 | 0.023 | 6.39E-06 | 20.535 |
| class | Gammaproteobacteria | rs79795896 | G | A | -0.159 | 0.035 | 7.92E-06 | 20.531 |
| class | Clostridia | rs11167476 | C | T | 0.050 | 0.011 | 6.24E-06 | 20.519 |
| class | Erysipelotrichia | rs12258727 | T | C | -0.052 | 0.011 | 6.86E-06 | 20.518 |
| class | Actinobacteria | rs72717438 | C | T | 0.052 | 0.012 | 9.84E-06 | 20.516 |
| class | Actinobacteria | rs4452212 | A | G | 0.053 | 0.012 | 7.01E-06 | 20.514 |
| class | Lentisphaeria | rs72640280 | G | A | 0.220 | 0.049 | 5.18E-06 | 20.513 |
| class | Gammaproteobacteria | rs7589167 | C | T | 0.067 | 0.015 | 3.81E-06 | 20.511 |
| class | Actinobacteria | rs12931368 | C | T | 0.051 | 0.011 | 5.68E-06 | 20.509 |
| class | Actinobacteria | rs11745789 | T | C | 0.052 | 0.012 | 9.68E-06 | 20.501 |
| class | Gammaproteobacteria | rs2176115 | A | G | -0.055 | 0.012 | 5.76E-06 | 20.493 |
| class | Bacteroidia | rs13097740 | G | A | -0.060 | 0.013 | 4.05E-06 | 20.493 |
| class | Clostridia | rs7731599 | T | C | 0.050 | 0.011 | 6.25E-06 | 20.492 |
| class | Bacilli | rs2952176 | A | G | -0.055 | 0.012 | 5.65E-06 | 20.491 |
| class | Lentisphaeria | rs2031282 | G | A | 0.122 | 0.027 | 4.38E-06 | 20.491 |
| class | Actinobacteria | rs1376754 | A | G | 0.051 | 0.011 | 6.71E-06 | 20.489 |
| class | Deltaproteobacteria | rs3935584 | T | C | -0.052 | 0.012 | 7.50E-06 | 20.485 |
| class | Erysipelotrichia | rs921306 | C | T | 0.049 | 0.011 | 7.20E-06 | 20.481 |
| class | Deltaproteobacteria | rs62020470 | G | A | -0.059 | 0.013 | 4.85E-06 | 20.480 |
| class | Deltaproteobacteria | rs2838334 | A | G | 0.056 | 0.012 | 5.45E-06 | 20.480 |
| class | Actinobacteria | rs1879087 | T | C | 0.104 | 0.023 | 6.20E-07 | 20.478 |
| class | Actinobacteria | rs28815269 | A | G | 0.053 | 0.012 | 6.57E-06 | 20.472 |
| class | Lentisphaeria | rs2546105 | T | A | 0.106 | 0.023 | 6.53E-06 | 20.472 |
| class | Gammaproteobacteria | rs13273646 | G | A | 0.053 | 0.012 | 6.78E-06 | 20.466 |
| class | Actinobacteria | rs72974159 | C | T | 0.060 | 0.013 | 7.21E-06 | 20.465 |
| class | Bacilli | rs7689028 | T | A | -0.051 | 0.011 | 6.61E-06 | 20.465 |
| class | Mollicutes | rs4970840 | G | A | -0.068 | 0.015 | 5.85E-06 | 20.463 |
| class | Erysipelotrichia | rs1074800 | G | A | 0.049 | 0.011 | 6.15E-06 | 20.459 |
| class | Actinobacteria | rs72717412 | C | T | 0.052 | 0.012 | 9.65E-06 | 20.448 |
| class | Deltaproteobacteria | rs112381107 | T | C | 0.207 | 0.046 | 4.63E-06 | 20.445 |
| class | Gammaproteobacteria | rs7017964 | G | A | 0.053 | 0.012 | 6.65E-06 | 20.441 |
| class | Verrucomicrobiae | rs2199602 | G | T | 0.066 | 0.015 | 7.12E-06 | 20.438 |
| class | Verrucomicrobiae | rs61779207 | A | G | -0.076 | 0.017 | 6.72E-06 | 20.432 |
| class | Betaproteobacteria | rs2321387 | A | G | -0.049 | 0.011 | 5.80E-06 | 20.430 |
| class | Actinobacteria | rs134387 | T | C | 0.106 | 0.024 | 4.05E-06 | 20.421 |
| class | Erysipelotrichia | rs9852883 | C | A | 0.048 | 0.011 | 6.33E-06 | 20.416 |
| class | Coriobacteriia | rs4880783 | T | G | 0.057 | 0.013 | 5.90E-06 | 20.414 |
| class | Bacteroidia | rs7857688 | G | A | 0.064 | 0.014 | 5.79E-06 | 20.408 |
| class | Methanobacteria | rs62240389 | A | G | -0.188 | 0.042 | 7.64E-06 | 20.402 |
| class | Verrucomicrobiae | rs4497215 | C | T | 0.064 | 0.014 | 6.55E-06 | 20.401 |
| class | Mollicutes | rs13020096 | T | C | 0.064 | 0.014 | 5.47E-06 | 20.399 |
| class | Clostridia | rs6934062 | C | G | 0.052 | 0.012 | 6.53E-06 | 20.396 |
| class | Verrucomicrobiae | rs72663756 | C | T | 0.066 | 0.015 | 5.39E-06 | 20.394 |
| class | Bacilli | rs12595929 | C | T | 0.057 | 0.013 | 4.57E-06 | 20.388 |
| class | Negativicutes | rs34058443 | T | C | 0.060 | 0.013 | 6.37E-06 | 20.381 |
| class | Methanobacteria | rs74599069 | C | A | 0.221 | 0.049 | 8.22E-06 | 20.378 |
| class | Bacilli | rs34989881 | G | A | 0.111 | 0.025 | 6.55E-06 | 20.374 |
| class | Bacteroidia | rs929878 | T | C | -0.055 | 0.012 | 4.73E-06 | 20.372 |
| class | Betaproteobacteria | rs320179 | A | C | 0.057 | 0.013 | 9.17E-06 | 20.371 |
| class | Melainabacteria | rs113884518 | C | T | -0.205 | 0.045 | 8.06E-06 | 20.371 |
| class | Bacilli | rs11835118 | C | T | -0.098 | 0.022 | 9.31E-06 | 20.365 |
| class | Actinobacteria | rs56051594 | C | T | 0.052 | 0.011 | 8.45E-06 | 20.364 |
| class | Mollicutes | rs4885016 | C | T | -0.082 | 0.018 | 7.27E-06 | 20.363 |
| class | Bacilli | rs12797734 | C | T | 0.057 | 0.013 | 7.21E-06 | 20.363 |
| class | Actinobacteria | rs7560535 | G | A | 0.080 | 0.018 | 8.11E-06 | 20.355 |
| class | Actinobacteria | rs6430594 | A | G | 0.068 | 0.015 | 7.33E-06 | 20.353 |
| class | Actinobacteria | rs11655079 | C | T | -0.056 | 0.012 | 5.93E-06 | 20.351 |
| class | Betaproteobacteria | rs34203874 | C | T | 0.090 | 0.020 | 8.91E-06 | 20.350 |
| class | Mollicutes | rs11678196 | A | G | 0.064 | 0.014 | 5.61E-06 | 20.344 |
| class | Verrucomicrobiae | rs72663785 | C | G | 0.066 | 0.015 | 7.41E-06 | 20.338 |
| class | Actinobacteria | rs134329 | G | A | 0.106 | 0.024 | 5.82E-06 | 20.333 |
| class | Deltaproteobacteria | rs7164160 | T | A | -0.058 | 0.013 | 5.28E-06 | 20.329 |
| class | Coriobacteriia | rs8010111 | A | G | -0.103 | 0.023 | 6.90E-06 | 20.328 |
| class | Verrucomicrobiae | rs12045978 | G | A | 0.064 | 0.014 | 6.87E-06 | 20.326 |
| class | Bacilli | rs78017832 | G | T | -0.100 | 0.022 | 9.59E-06 | 20.326 |
| class | Coriobacteriia | rs76779974 | G | C | 0.077 | 0.017 | 5.63E-06 | 20.326 |
| class | Melainabacteria | rs367480 | A | G | -0.084 | 0.019 | 8.20E-06 | 20.323 |
| class | Alphaproteobacteria | rs150933400 | G | A | -0.110 | 0.024 | 9.11E-06 | 20.319 |
| class | Methanobacteria | rs4257531 | A | G | 0.164 | 0.036 | 7.44E-06 | 20.316 |
| class | Actinobacteria | rs7607920 | T | C | 0.068 | 0.015 | 7.91E-06 | 20.315 |
| class | Actinobacteria | rs56346498 | C | T | 0.052 | 0.012 | 9.76E-06 | 20.315 |
| class | Bacteroidia | rs72706335 | C | T | -0.222 | 0.049 | 7.66E-06 | 20.315 |
| class | Bacilli | rs694949 | G | A | -0.081 | 0.018 | 7.60E-06 | 20.314 |
| class | Actinobacteria | rs4953938 | G | A | 0.051 | 0.011 | 4.87E-06 | 20.313 |
| class | Bacteroidia | rs7157380 | G | C | 0.096 | 0.021 | 4.63E-06 | 20.312 |
| class | Verrucomicrobiae | rs7693865 | C | T | 0.107 | 0.024 | 6.08E-06 | 20.307 |
| class | Alphaproteobacteria | rs7960664 | A | G | 0.097 | 0.022 | 8.84E-06 | 20.305 |
| class | Actinobacteria | rs6432655 | G | A | 0.050 | 0.011 | 7.23E-06 | 20.301 |
| class | Mollicutes | rs3931560 | C | A | -0.067 | 0.015 | 5.79E-06 | 20.299 |
| class | Deltaproteobacteria | rs2873556 | A | G | -0.077 | 0.017 | 9.29E-06 | 20.295 |
| class | Actinobacteria | rs108272 | C | G | 0.106 | 0.024 | 4.33E-06 | 20.290 |
| class | Bacilli | rs2559847 | C | T | -0.099 | 0.022 | 9.02E-06 | 20.289 |
| class | Gammaproteobacteria | rs6469507 | T | C | -0.055 | 0.012 | 6.31E-06 | 20.285 |
| class | Mollicutes | rs652651 | G | A | -0.067 | 0.015 | 5.50E-06 | 20.278 |
| class | Gammaproteobacteria | rs4876552 | G | C | 0.052 | 0.012 | 7.74E-06 | 20.272 |
| class | Coriobacteriia | rs2442778 | A | G | -0.116 | 0.026 | 9.03E-06 | 20.272 |
| class | Actinobacteria | rs1947112 | A | G | 0.051 | 0.011 | 5.39E-06 | 20.271 |
| class | Bacteroidia | rs62575403 | T | C | 0.140 | 0.031 | 7.06E-06 | 20.264 |
| class | Mollicutes | rs11102971 | T | C | -0.067 | 0.015 | 5.84E-06 | 20.254 |
| class | Negativicutes | rs1649999 | G | A | 0.075 | 0.017 | 7.58E-06 | 20.246 |
| class | Bacilli | rs2952228 | C | T | -0.055 | 0.012 | 5.57E-06 | 20.245 |
| class | Gammaproteobacteria | rs12550281 | A | G | 0.052 | 0.012 | 7.18E-06 | 20.237 |
| class | Lentisphaeria | rs1002941 | A | G | 0.105 | 0.023 | 8.15E-06 | 20.233 |
| class | Bacilli | rs11837200 | T | C | -0.098 | 0.022 | 9.98E-06 | 20.224 |
| class | Mollicutes | rs17214486 | A | C | 0.061 | 0.014 | 6.61E-06 | 20.223 |
| class | Bacilli | rs11110283 | C | A | -0.100 | 0.022 | 9.53E-06 | 20.218 |
| class | Actinobacteria | rs5001584 | G | A | 0.052 | 0.012 | 6.20E-06 | 20.213 |
| class | Clostridia | rs7964593 | T | C | 0.051 | 0.011 | 5.91E-06 | 20.212 |
| class | Methanobacteria | rs10424197 | A | G | -0.111 | 0.025 | 9.28E-06 | 20.211 |
| class | Mollicutes | rs10504256 | G | A | 0.063 | 0.014 | 8.96E-06 | 20.210 |
| class | Methanobacteria | rs73068003 | T | G | -0.158 | 0.035 | 8.45E-06 | 20.206 |
| class | Actinobacteria | rs12474093 | G | A | 0.072 | 0.016 | 7.62E-06 | 20.201 |
| class | Gammaproteobacteria | rs4548204 | A | C | -0.054 | 0.012 | 6.72E-06 | 20.200 |
| class | Lentisphaeria | rs6951588 | T | C | 0.104 | 0.023 | 7.63E-06 | 20.199 |
| class | Gammaproteobacteria | rs7818533 | G | A | 0.052 | 0.012 | 7.99E-06 | 20.192 |
| class | Negativicutes | rs2834062 | G | A | 0.049 | 0.011 | 8.44E-06 | 20.190 |
| class | Actinobacteria | rs11674937 | G | C | 0.060 | 0.013 | 3.78E-06 | 20.187 |
| class | Bacteroidia | rs11146701 | G | A | 0.047 | 0.011 | 7.08E-06 | 20.186 |
| class | Mollicutes | rs10745352 | C | T | -0.067 | 0.015 | 6.13E-06 | 20.181 |
| class | Actinobacteria | rs34007021 | C | T | 0.068 | 0.015 | 8.58E-06 | 20.166 |
| class | Actinobacteria | rs4988145 | A | C | 0.068 | 0.015 | 7.51E-06 | 20.165 |
| class | Betaproteobacteria | rs12477314 | C | T | 0.060 | 0.013 | 5.96E-06 | 20.164 |
| class | Gammaproteobacteria | rs10110536 | C | T | -0.054 | 0.012 | 6.78E-06 | 20.162 |
| class | Gammaproteobacteria | rs13257428 | G | A | 0.052 | 0.012 | 7.91E-06 | 20.161 |
| class | Deltaproteobacteria | rs35912151 | A | T | -0.077 | 0.017 | 9.95E-06 | 20.159 |
| class | Actinobacteria | rs2413311 | C | T | 0.106 | 0.024 | 4.06E-06 | 20.158 |
| class | Methanobacteria | rs41288287 | C | T | 0.199 | 0.044 | 8.70E-06 | 20.154 |
| class | Actinobacteria | rs2304366 | C | T | 0.068 | 0.015 | 8.68E-06 | 20.153 |
| class | Gammaproteobacteria | rs2357360 | T | A | 0.052 | 0.012 | 7.80E-06 | 20.148 |
| class | Verrucomicrobiae | rs12026127 | G | A | 0.064 | 0.014 | 7.05E-06 | 20.144 |
| class | Betaproteobacteria | rs62191107 | T | C | 0.060 | 0.013 | 6.67E-06 | 20.140 |
| class | Betaproteobacteria | rs62191106 | C | T | 0.060 | 0.013 | 6.63E-06 | 20.136 |
| class | Actinobacteria | rs1515761 | C | T | 0.076 | 0.017 | 4.96E-06 | 20.136 |
| class | Actinobacteria | rs76097170 | C | T | 0.067 | 0.015 | 8.88E-06 | 20.135 |
| class | Actinobacteria | rs2459217 | A | G | 0.080 | 0.018 | 5.55E-06 | 20.135 |
| class | Deltaproteobacteria | rs72821212 | G | C | -0.091 | 0.020 | 7.73E-06 | 20.135 |
| class | Alphaproteobacteria | rs12977163 | C | G | 0.069 | 0.015 | 7.14E-06 | 20.133 |
| class | Alphaproteobacteria | rs8048637 | G | T | 0.078 | 0.017 | 9.08E-06 | 20.127 |
| class | Deltaproteobacteria | rs72647049 | A | G | -0.076 | 0.017 | 9.56E-06 | 20.126 |
| class | Actinobacteria | rs134396 | G | T | 0.106 | 0.024 | 4.11E-06 | 20.119 |
| class | Erysipelotrichia | rs12938302 | C | T | -0.075 | 0.017 | 9.17E-06 | 20.119 |
| class | Actinobacteria | rs134409 | G | A | 0.106 | 0.024 | 4.33E-06 | 20.115 |
| class | Actinobacteria | rs134408 | A | G | 0.106 | 0.024 | 4.33E-06 | 20.115 |
| class | Erysipelotrichia | rs35587059 | C | T | 0.048 | 0.011 | 7.13E-06 | 20.114 |
| class | Bacteroidia | rs73846124 | G | A | -0.060 | 0.013 | 5.11E-06 | 20.110 |
| class | Bacteroidia | rs71322865 | C | T | -0.059 | 0.013 | 4.90E-06 | 20.108 |
| class | Erysipelotrichia | rs41300431 | G | A | 0.048 | 0.011 | 7.12E-06 | 20.106 |
| class | Actinobacteria | rs134404 | T | A | 0.107 | 0.024 | 5.55E-06 | 20.102 |
| class | Mollicutes | rs4970754 | G | A | -0.067 | 0.015 | 6.38E-06 | 20.100 |
| class | Actinobacteria | rs134400 | G | A | 0.106 | 0.024 | 4.28E-06 | 20.099 |
| class | Mollicutes | rs10497163 | G | T | 0.069 | 0.015 | 5.94E-06 | 20.088 |
| class | Negativicutes | rs58649672 | C | G | 0.047 | 0.011 | 7.78E-06 | 20.084 |
| class | Mollicutes | rs7843989 | T | G | 0.063 | 0.014 | 9.55E-06 | 20.084 |
| class | Gammaproteobacteria | rs12827899 | G | C | -0.061 | 0.014 | 5.44E-06 | 20.081 |
| class | Coriobacteriia | rs13307134 | T | C | 0.057 | 0.013 | 7.80E-06 | 20.072 |
| class | Bacteroidia | rs34986288 | G | A | -0.061 | 0.014 | 4.69E-06 | 20.069 |
| class | Actinobacteria | rs10187990 | A | G | 0.051 | 0.011 | 5.67E-06 | 20.061 |
| class | Mollicutes | rs7827910 | T | A | 0.063 | 0.014 | 9.57E-06 | 20.058 |
| class | Actinobacteria | rs134393 | C | T | 0.105 | 0.024 | 4.43E-06 | 20.058 |
| class | Coriobacteriia | rs7977399 | C | T | -0.058 | 0.013 | 6.07E-06 | 20.058 |
| class | Bacteroidia | rs2418267 | G | A | 0.061 | 0.014 | 7.16E-06 | 20.054 |
| class | Actinobacteria | rs1374289 | C | T | 0.051 | 0.011 | 5.79E-06 | 20.049 |
| class | Clostridia | rs13105690 | C | T | -0.053 | 0.012 | 8.78E-06 | 20.044 |
| class | Actinobacteria | rs12465544 | C | T | 0.067 | 0.015 | 9.70E-06 | 20.044 |
| class | Actinobacteria | rs80083040 | G | T | 0.156 | 0.035 | 8.62E-06 | 20.038 |
| class | Betaproteobacteria | rs12867878 | G | A | -0.050 | 0.011 | 6.55E-06 | 20.029 |
| class | Alphaproteobacteria | rs34569731 | G | A | 0.071 | 0.016 | 7.38E-06 | 20.028 |
| class | Betaproteobacteria | rs1419746 | G | A | -0.049 | 0.011 | 6.69E-06 | 20.024 |
| class | Bacilli | rs11932798 | C | T | -0.051 | 0.011 | 8.58E-06 | 20.023 |
| class | Deltaproteobacteria | rs12546457 | A | G | -0.077 | 0.017 | 8.53E-06 | 20.023 |
| class | Mollicutes | rs6664236 | A | C | -0.067 | 0.015 | 6.74E-06 | 20.014 |
| class | Gammaproteobacteria | rs4896254 | A | T | -0.054 | 0.012 | 6.43E-06 | 20.010 |
| class | Actinobacteria | rs3769023 | G | A | 0.051 | 0.011 | 6.29E-06 | 20.010 |
| class | Gammaproteobacteria | rs75101789 | T | C | 0.073 | 0.016 | 8.79E-06 | 20.002 |
| class | Clostridia | rs76767978 | A | G | 0.070 | 0.016 | 8.40E-06 | 19.989 |
| class | Methanobacteria | rs9532770 | T | C | 0.124 | 0.028 | 9.34E-06 | 19.985 |
| class | Actinobacteria | rs28579 | G | C | 0.105 | 0.024 | 4.45E-06 | 19.979 |
| class | Verrucomicrobiae | rs7538356 | A | C | 0.064 | 0.014 | 8.12E-06 | 19.978 |
| class | Betaproteobacteria | rs2528688 | G | A | -0.049 | 0.011 | 6.95E-06 | 19.976 |
| class | Erysipelotrichia | rs7906464 | T | A | -0.053 | 0.012 | 7.35E-06 | 19.973 |
| class | Actinobacteria | rs134392 | C | T | 0.105 | 0.024 | 4.54E-06 | 19.970 |
| class | Gammaproteobacteria | rs1473985 | A | T | -0.054 | 0.012 | 7.53E-06 | 19.961 |
| class | Alphaproteobacteria | rs17061716 | C | G | 0.093 | 0.021 | 9.33E-06 | 19.960 |
| class | Betaproteobacteria | rs2528679 | T | C | -0.049 | 0.011 | 7.00E-06 | 19.958 |
| class | Actinobacteria | rs2243193 | G | A | 0.058 | 0.013 | 7.50E-06 | 19.956 |
| class | Betaproteobacteria | rs4585927 | T | C | 0.049 | 0.011 | 8.30E-06 | 19.948 |
| class | Erysipelotrichia | rs290833 | G | T | -0.050 | 0.011 | 8.03E-06 | 19.942 |
| class | Betaproteobacteria | rs2906622 | T | C | -0.049 | 0.011 | 7.28E-06 | 19.942 |
| class | Actinobacteria | rs12049045 | G | A | 0.051 | 0.011 | 8.63E-06 | 19.936 |
| class | Erysipelotrichia | rs8096144 | C | G | -0.086 | 0.019 | 9.53E-06 | 19.930 |
| class | Actinobacteria | rs12467569 | T | C | 0.067 | 0.015 | 9.48E-06 | 19.924 |
| class | Actinobacteria | rs11682960 | C | T | 0.050 | 0.011 | 8.92E-06 | 19.923 |
| class | Actinobacteria | rs78274583 | C | T | 0.069 | 0.015 | 8.93E-06 | 19.918 |
| class | Coriobacteriia | rs11073596 | G | T | 0.051 | 0.011 | 8.14E-06 | 19.912 |
| class | Bacilli | rs111552159 | G | C | 0.128 | 0.029 | 9.13E-06 | 19.912 |
| class | Deltaproteobacteria | rs7199026 | A | C | -0.052 | 0.012 | 8.58E-06 | 19.910 |
| class | Verrucomicrobiae | rs4595371 | A | G | 0.063 | 0.014 | 8.42E-06 | 19.894 |
| class | Lentisphaeria | rs1895249 | G | T | 0.105 | 0.023 | 8.27E-06 | 19.893 |
| class | Coriobacteriia | rs62448869 | A | T | -0.049 | 0.011 | 7.87E-06 | 19.891 |
| class | Bacilli | rs7627280 | T | C | 0.051 | 0.011 | 8.81E-06 | 19.882 |
| class | Verrucomicrobiae | rs79837054 | T | C | 0.105 | 0.024 | 7.58E-06 | 19.879 |
| class | Bacilli | rs11097541 | G | A | -0.051 | 0.011 | 8.99E-06 | 19.875 |
| class | Mollicutes | rs2464826 | C | A | 0.094 | 0.021 | 8.40E-06 | 19.874 |
| class | Melainabacteria | rs10148250 | A | G | 0.086 | 0.019 | 8.67E-06 | 19.866 |
| class | Mollicutes | rs12711447 | T | C | -0.067 | 0.015 | 8.10E-06 | 19.866 |
| class | Coriobacteriia | rs12974142 | A | G | 0.079 | 0.018 | 8.51E-06 | 19.865 |
| class | Mollicutes | rs7551176 | T | C | -0.067 | 0.015 | 8.14E-06 | 19.863 |
| class | Betaproteobacteria | rs56104821 | C | A | 0.057 | 0.013 | 8.76E-06 | 19.860 |
| class | Methanobacteria | rs6508769 | C | T | 0.154 | 0.034 | 8.23E-06 | 19.856 |
| class | Bacilli | rs28564647 | G | T | -0.061 | 0.014 | 7.81E-06 | 19.855 |
| class | Clostridia | rs6536525 | G | C | 0.066 | 0.015 | 7.84E-06 | 19.853 |
| class | Deltaproteobacteria | rs17084793 | A | G | -0.071 | 0.016 | 5.69E-06 | 19.850 |
| class | Coriobacteriia | rs7898585 | C | T | 0.073 | 0.016 | 5.25E-06 | 19.850 |
| class | Lentisphaeria | rs1363428 | A | G | 0.104 | 0.023 | 9.33E-06 | 19.850 |
| class | Gammaproteobacteria | rs10095416 | T | A | -0.054 | 0.012 | 7.92E-06 | 19.844 |
| class | Actinobacteria | rs4945008 | G | A | 0.054 | 0.012 | 5.39E-06 | 19.842 |
| class | Actinobacteria | rs961091 | A | G | 0.050 | 0.011 | 8.68E-06 | 19.835 |
| class | Erysipelotrichia | rs28703642 | G | T | 0.053 | 0.012 | 7.64E-06 | 19.830 |
| class | Betaproteobacteria | rs9537878 | G | A | -0.049 | 0.011 | 7.93E-06 | 19.830 |
| class | Mollicutes | rs6695482 | G | C | -0.066 | 0.015 | 7.47E-06 | 19.816 |
| class | Alphaproteobacteria | rs62285697 | T | C | 0.081 | 0.018 | 9.76E-06 | 19.812 |
| class | Verrucomicrobiae | rs4294419 | G | A | 0.063 | 0.014 | 8.19E-06 | 19.808 |
| class | Coriobacteriia | rs45480394 | G | T | -0.050 | 0.011 | 9.71E-06 | 19.806 |
| class | Mollicutes | rs10858095 | C | G | -0.066 | 0.015 | 7.51E-06 | 19.802 |
| class | Methanobacteria | rs11018665 | T | A | 0.111 | 0.025 | 6.52E-06 | 19.797 |
| class | Bacteroidia | rs1505366 | G | T | -0.107 | 0.024 | 8.89E-06 | 19.796 |
| class | Clostridia | rs11082866 | A | T | -0.051 | 0.012 | 8.06E-06 | 19.792 |
| class | Verrucomicrobiae | rs12024893 | A | G | 0.063 | 0.014 | 8.96E-06 | 19.784 |
| class | Bacilli | rs2975647 | G | A | -0.055 | 0.012 | 7.37E-06 | 19.782 |
| class | Bacilli | rs11249980 | C | T | -0.055 | 0.012 | 8.01E-06 | 19.776 |
| class | Mollicutes | rs1465607 | C | T | -0.066 | 0.015 | 8.59E-06 | 19.775 |
| class | Coriobacteriia | rs7303392 | C | T | -0.057 | 0.013 | 7.32E-06 | 19.770 |
| class | Actinobacteria | rs134416 | G | A | 0.105 | 0.024 | 5.70E-06 | 19.764 |
| class | Negativicutes | rs1135612 | A | G | 0.053 | 0.012 | 9.26E-06 | 19.761 |
| class | Erysipelotrichia | rs1884466 | T | C | -0.048 | 0.011 | 9.53E-06 | 19.760 |
| class | Negativicutes | rs12572508 | C | T | 0.047 | 0.011 | 9.58E-06 | 19.757 |
| class | Mollicutes | rs10745356 | A | C | -0.066 | 0.015 | 8.64E-06 | 19.756 |
| class | Actinobacteria | rs6893855 | A | C | -0.077 | 0.017 | 8.84E-06 | 19.754 |
| class | Verrucomicrobiae | rs36010840 | C | T | 0.063 | 0.014 | 9.11E-06 | 19.754 |
| class | Actinobacteria | rs7569447 | A | C | 0.072 | 0.016 | 4.83E-06 | 19.754 |
| class | Actinobacteria | rs58110576 | C | A | 0.052 | 0.012 | 9.09E-06 | 19.752 |
| class | Deltaproteobacteria | rs7163129 | T | C | -0.057 | 0.013 | 7.15E-06 | 19.751 |
| class | Melainabacteria | rs1221147 | A | T | 0.124 | 0.028 | 7.11E-06 | 19.749 |
| class | Actinobacteria | rs134394 | C | T | 0.105 | 0.024 | 5.05E-06 | 19.739 |
| class | Actinobacteria | rs134395 | C | A | 0.105 | 0.024 | 5.05E-06 | 19.738 |
| class | Actinobacteria | rs309143 | A | G | 0.067 | 0.015 | 9.72E-06 | 19.728 |
| class | Verrucomicrobiae | rs72663769 | T | C | 0.065 | 0.015 | 7.43E-06 | 19.727 |
| class | Lentisphaeria | rs62481809 | C | G | 0.103 | 0.023 | 9.20E-06 | 19.726 |
| class | Actinobacteria | rs625118 | C | A | 0.074 | 0.017 | 8.62E-06 | 19.725 |
| class | Erysipelotrichia | rs7100916 | T | G | -0.053 | 0.012 | 7.62E-06 | 19.721 |
| class | Verrucomicrobiae | rs72663802 | C | T | 0.065 | 0.015 | 8.44E-06 | 19.707 |
| class | Actinobacteria | rs4957061 | C | T | 0.050 | 0.011 | 9.66E-06 | 19.692 |
| class | Gammaproteobacteria | rs2234691 | C | T | 0.076 | 0.017 | 8.39E-06 | 19.682 |
| class | Coriobacteriia | rs1397793 | A | G | -0.050 | 0.011 | 9.77E-06 | 19.681 |
| class | Verrucomicrobiae | rs4107636 | G | C | 0.063 | 0.014 | 7.95E-06 | 19.674 |
| class | Actinobacteria | rs9937275 | G | A | 0.052 | 0.012 | 8.63E-06 | 19.674 |
| class | Actinobacteria | rs11895319 | T | C | 0.065 | 0.015 | 9.98E-06 | 19.670 |
| class | Betaproteobacteria | rs7326330 | T | C | -0.049 | 0.011 | 8.60E-06 | 19.662 |
| class | Verrucomicrobiae | rs4600885 | A | C | 0.063 | 0.014 | 7.86E-06 | 19.661 |
| class | Erysipelotrichia | rs61865709 | G | A | -0.053 | 0.012 | 8.00E-06 | 19.660 |
| class | Verrucomicrobiae | rs12411321 | T | C | 0.074 | 0.017 | 7.24E-06 | 19.656 |
| class | Betaproteobacteria | rs7988877 | T | A | -0.050 | 0.011 | 8.50E-06 | 19.651 |
| class | Methanobacteria | rs4753222 | G | A | 0.111 | 0.025 | 7.45E-06 | 19.651 |
| class | Actinobacteria | rs2243174 | A | G | 0.057 | 0.013 | 8.66E-06 | 19.649 |
| class | Bacteroidia | rs4916508 | A | G | -0.047 | 0.011 | 8.47E-06 | 19.641 |
| class | Mollicutes | rs12480021 | G | A | -0.111 | 0.025 | 9.55E-06 | 19.640 |
| class | Deltaproteobacteria | rs7162535 | T | C | -0.057 | 0.013 | 7.63E-06 | 19.632 |
| class | Coriobacteriia | rs7540303 | T | C | 0.048 | 0.011 | 9.62E-06 | 19.632 |
| class | Coriobacteriia | rs60426889 | G | T | -0.050 | 0.011 | 8.69E-06 | 19.632 |
| class | Negativicutes | rs2103109 | A | C | 0.047 | 0.011 | 9.81E-06 | 19.622 |
| class | Verrucomicrobiae | rs736124 | T | A | -0.074 | 0.017 | 8.63E-06 | 19.620 |
| class | Betaproteobacteria | rs2528684 | T | C | -0.048 | 0.011 | 8.25E-06 | 19.613 |
| class | Betaproteobacteria | rs13385989 | A | C | 0.060 | 0.014 | 9.69E-06 | 19.613 |
| class | Mollicutes | rs10858094 | C | T | -0.066 | 0.015 | 8.13E-06 | 19.603 |
| class | Actinobacteria | rs67665078 | G | A | 0.049 | 0.011 | 9.71E-06 | 19.591 |
| class | Betaproteobacteria | rs2613608 | G | A | -0.048 | 0.011 | 8.40E-06 | 19.591 |
| class | Betaproteobacteria | rs34177861 | G | A | 0.060 | 0.013 | 7.65E-06 | 19.589 |
| class | Betaproteobacteria | rs4521068 | G | A | 0.059 | 0.013 | 9.11E-06 | 19.585 |
| class | Actinobacteria | rs7700479 | A | T | -0.077 | 0.017 | 8.62E-06 | 19.581 |
| class | Actinobacteria | rs72847647 | G | A | -0.056 | 0.013 | 6.51E-06 | 19.575 |
| class | Mollicutes | rs74338080 | C | G | -0.110 | 0.025 | 9.95E-06 | 19.574 |
| class | Mollicutes | rs417270 | C | T | -0.066 | 0.015 | 8.60E-06 | 19.573 |
| class | Lentisphaeria | rs253710 | C | T | 0.104 | 0.023 | 9.95E-06 | 19.571 |
| class | Coriobacteriia | rs3025411 | G | A | 0.093 | 0.021 | 8.27E-06 | 19.566 |
| class | Bacilli | rs2975649 | C | T | -0.055 | 0.012 | 8.32E-06 | 19.559 |
| class | Erysipelotrichia | rs2638904 | A | G | 0.047 | 0.011 | 9.64E-06 | 19.553 |
| class | Clostridia | rs6536526 | T | A | 0.066 | 0.015 | 9.19E-06 | 19.545 |
| class | Mollicutes | rs367586 | A | C | -0.066 | 0.015 | 8.77E-06 | 19.543 |
| class | Actinobacteria | rs134410 | C | T | 0.104 | 0.024 | 5.84E-06 | 19.541 |
| class | Actinobacteria | rs79931555 | A | G | 0.097 | 0.022 | 7.13E-06 | 19.539 |
| class | Lentisphaeria | rs35004924 | A | C | 0.104 | 0.024 | 6.42E-06 | 19.533 |
| class | Melainabacteria | rs789069 | C | A | -0.104 | 0.023 | 6.85E-06 | 19.531 |
| class | Gammaproteobacteria | rs6432976 | C | T | 0.070 | 0.016 | 7.44E-06 | 19.525 |
| class | Gammaproteobacteria | rs12692857 | G | A | 0.065 | 0.015 | 6.39E-06 | 19.523 |
| class | Bacilli | rs2975655 | G | A | -0.055 | 0.012 | 8.92E-06 | 19.523 |
| class | Actinobacteria | rs134417 | C | A | 0.104 | 0.024 | 6.28E-06 | 19.516 |
| class | Melainabacteria | rs10738747 | A | G | 0.081 | 0.018 | 9.96E-06 | 19.514 |
| class | Mollicutes | rs13025367 | C | G | 0.063 | 0.014 | 8.53E-06 | 19.512 |
| class | Actinobacteria | rs857444 | T | C | 0.051 | 0.012 | 8.92E-06 | 19.500 |
| class | Bacteroidia | rs1365795 | T | C | 0.090 | 0.020 | 8.08E-06 | 19.500 |
| class | Bacteroidia | rs73975618 | A | G | -0.189 | 0.043 | 6.90E-06 | 19.496 |
| class | Coriobacteriia | rs62448870 | A | G | -0.048 | 0.011 | 9.50E-06 | 19.495 |
| class | Actinobacteria | rs134411 | T | C | 0.104 | 0.024 | 6.18E-06 | 19.494 |
| class | Deltaproteobacteria | rs17791393 | A | G | -0.067 | 0.015 | 8.23E-06 | 19.494 |
| class | Actinobacteria | rs1496374 | G | A | -0.077 | 0.017 | 9.18E-06 | 19.490 |
| class | Gammaproteobacteria | rs2090256 | T | C | -0.053 | 0.012 | 9.68E-06 | 19.485 |
| class | Betaproteobacteria | rs1581853 | G | A | -0.049 | 0.011 | 9.17E-06 | 19.484 |
| class | Verrucomicrobiae | rs72663751 | G | A | 0.065 | 0.015 | 8.50E-06 | 19.484 |
| class | Actinobacteria | rs134327 | G | A | 0.104 | 0.024 | 9.02E-06 | 19.482 |
| class | Gammaproteobacteria | rs7003100 | T | C | -0.054 | 0.012 | 9.14E-06 | 19.468 |
| class | Mollicutes | rs443345 | G | A | -0.066 | 0.015 | 9.11E-06 | 19.464 |
| class | Mollicutes | rs67450615 | T | G | 0.072 | 0.016 | 9.40E-06 | 19.456 |
| class | Betaproteobacteria | rs7324740 | A | G | -0.050 | 0.011 | 9.29E-06 | 19.454 |
| class | Coriobacteriia | rs7912120 | G | A | 0.072 | 0.016 | 6.24E-06 | 19.451 |
| class | Bacilli | rs4260895 | A | C | -0.054 | 0.012 | 6.87E-06 | 19.449 |
| class | Mollicutes | rs10776807 | A | G | -0.066 | 0.015 | 9.12E-06 | 19.444 |
| class | Mollicutes | rs13023735 | A | G | 0.063 | 0.014 | 8.86E-06 | 19.441 |
| class | Actinobacteria | rs134268 | T | C | 0.103 | 0.023 | 7.25E-06 | 19.435 |
| class | Clostridia | rs10517722 | G | C | 0.065 | 0.015 | 9.58E-06 | 19.432 |
| class | Betaproteobacteria | rs11124193 | A | G | 0.059 | 0.013 | 9.95E-06 | 19.426 |
| class | Clostridia | rs7687278 | T | A | 0.065 | 0.015 | 9.65E-06 | 19.418 |
| class | Betaproteobacteria | rs1926544 | A | C | -0.048 | 0.011 | 9.84E-06 | 19.418 |
| class | Verrucomicrobiae | rs12904881 | G | A | 0.058 | 0.013 | 9.30E-06 | 19.415 |
| class | Bacilli | rs13068444 | G | A | 0.060 | 0.014 | 9.53E-06 | 19.414 |
| class | Verrucomicrobiae | rs28613890 | C | T | 0.063 | 0.014 | 9.91E-06 | 19.405 |
| class | Verrucomicrobiae | rs28858161 | T | C | 0.062 | 0.014 | 9.03E-06 | 19.396 |
| class | Coriobacteriia | rs11656361 | C | A | 0.077 | 0.018 | 8.02E-06 | 19.394 |
| class | Verrucomicrobiae | rs28811282 | G | A | 0.062 | 0.014 | 9.06E-06 | 19.391 |
| class | Erysipelotrichia | rs56970041 | G | T | 0.072 | 0.016 | 5.40E-06 | 19.385 |
| class | Mollicutes | rs12136140 | A | G | -0.066 | 0.015 | 9.28E-06 | 19.385 |
| class | Verrucomicrobiae | rs10005131 | T | G | 0.061 | 0.014 | 9.81E-06 | 19.381 |
| class | Gammaproteobacteria | rs6720763 | T | C | 0.061 | 0.014 | 6.95E-06 | 19.379 |
| class | Clostridia | rs6797343 | T | G | 0.059 | 0.013 | 9.36E-06 | 19.375 |
| class | Actinobacteria | rs861392 | T | C | 0.051 | 0.012 | 9.85E-06 | 19.364 |
| class | Mollicutes | rs11102969 | A | G | -0.066 | 0.015 | 9.44E-06 | 19.357 |
| class | Methanobacteria | rs3912664 | G | T | 0.110 | 0.025 | 8.67E-06 | 19.355 |
| class | Verrucomicrobiae | rs1988252 | G | A | 0.062 | 0.014 | 9.56E-06 | 19.347 |
| class | Bacilli | rs7187700 | G | A | 0.056 | 0.013 | 8.87E-06 | 19.339 |
| class | Clostridia | rs7667168 | T | C | 0.066 | 0.015 | 9.17E-06 | 19.338 |
| class | Actinobacteria | rs7732645 | T | C | -0.076 | 0.017 | 9.50E-06 | 19.331 |
| class | Actinobacteria | rs134413 | C | T | 0.104 | 0.024 | 7.36E-06 | 19.331 |
| class | Melainabacteria | rs11806158 | C | A | -0.082 | 0.019 | 8.98E-06 | 19.316 |
| class | Actinobacteria | rs7721660 | T | G | -0.077 | 0.017 | 9.97E-06 | 19.310 |
| class | Gammaproteobacteria | rs10171261 | G | A | 0.065 | 0.015 | 6.86E-06 | 19.307 |
| class | Coriobacteriia | rs1834859 | T | G | -0.057 | 0.013 | 7.97E-06 | 19.301 |
| class | Bacilli | rs1595463 | A | C | 0.048 | 0.011 | 7.97E-06 | 19.300 |
| class | Mollicutes | rs10858092 | T | C | -0.065 | 0.015 | 9.40E-06 | 19.298 |
| class | Methanobacteria | rs12825290 | G | C | -0.217 | 0.049 | 6.08E-06 | 19.296 |
| class | Verrucomicrobiae | rs941682 | A | G | -0.063 | 0.014 | 9.61E-06 | 19.290 |
| class | Gammaproteobacteria | rs60188538 | C | T | 0.063 | 0.014 | 7.60E-06 | 19.289 |
| class | Verrucomicrobiae | rs2865331 | G | A | 0.062 | 0.014 | 9.67E-06 | 19.282 |
| class | Alphaproteobacteria | rs55876211 | T | C | -0.081 | 0.018 | 7.54E-06 | 19.255 |
| class | Methanobacteria | rs11018664 | G | A | 0.110 | 0.025 | 9.00E-06 | 19.239 |
| class | Deltaproteobacteria | rs4321143 | A | G | -0.057 | 0.013 | 9.45E-06 | 19.230 |
| class | Methanobacteria | rs10830292 | T | G | 0.110 | 0.025 | 9.56E-06 | 19.227 |
| class | Bacteroidia | NA | T | C | -0.223 | 0.051 | 9.93E-06 | 19.221 |
| class | Mollicutes | rs12566890 | G | T | -0.101 | 0.023 | 3.65E-06 | 19.176 |
| class | Methanobacteria | rs4385899 | C | T | 0.110 | 0.025 | 9.69E-06 | 19.161 |
| class | Actinobacteria | rs134422 | T | C | 0.103 | 0.024 | 7.77E-06 | 19.153 |
| class | Bacteroidia | rs62531359 | G | T | 0.066 | 0.015 | 9.09E-06 | 19.138 |
| class | Bacteroidia | rs12486971 | T | C | -0.060 | 0.014 | 8.56E-06 | 19.137 |
| class | Betaproteobacteria | rs9553336 | T | G | 0.089 | 0.020 | 9.14E-06 | 19.121 |
| class | Methanobacteria | rs10501707 | A | G | 0.110 | 0.025 | 9.72E-06 | 19.112 |
| class | Bacteroidia | rs4146051 | G | A | -0.107 | 0.025 | 8.76E-06 | 19.105 |
| class | Bacilli | rs74663707 | T | C | 0.098 | 0.022 | 8.46E-06 | 19.095 |
| class | Gammaproteobacteria | rs16852925 | C | G | 0.063 | 0.014 | 8.85E-06 | 19.082 |
| class | Coriobacteriia | rs7896306 | T | G | 0.072 | 0.016 | 7.82E-06 | 19.059 |
| class | Actinobacteria | rs5755641 | T | C | 0.103 | 0.024 | 8.25E-06 | 19.026 |
| class | Clostridia | rs3792064 | A | G | 0.080 | 0.018 | 9.98E-06 | 19.003 |
| class | Mollicutes | rs28537087 | A | G | 0.082 | 0.019 | 8.07E-06 | 19.002 |
| class | Deltaproteobacteria | rs2692012 | G | A | 0.110 | 0.025 | 3.14E-06 | 18.968 |
| class | Clostridia | rs10209007 | C | G | 0.079 | 0.018 | 9.89E-06 | 18.952 |
| class | Actinobacteria | rs638382 | C | T | -0.051 | 0.012 | 7.72E-06 | 18.945 |
| class | Bacteroidia | rs79585701 | C | A | 0.065 | 0.015 | 9.99E-06 | 18.687 |
| class | Erysipelotrichia | rs10134361 | G | A | 0.071 | 0.016 | 8.66E-06 | 18.642 |
| class | Betaproteobacteria | rs75242906 | T | C | -0.121 | 0.028 | 9.27E-06 | 18.601 |
| class | Actinobacteria | rs9800013 | A | G | 0.060 | 0.014 | 9.78E-06 | 18.576 |
| class | Bacilli | rs2952183 | C | T | -0.065 | 0.015 | 9.99E-06 | 18.552 |
| class | Clostridia | rs1842454 | A | G | -0.055 | 0.013 | 8.72E-06 | 18.466 |
| class | Negativicutes | rs10787507 | C | G | -0.055 | 0.013 | 9.14E-06 | 18.411 |
| class | Betaproteobacteria | rs9520346 | G | C | 0.142 | 0.033 | 7.59E-06 | 18.336 |
| class | Erysipelotrichia | rs10144017 | G | A | 0.069 | 0.016 | 8.50E-06 | 18.267 |
| class | Erysipelotrichia | rs7826267 | G | T | -0.084 | 0.020 | 9.28E-06 | 17.755 |
| class | Bacilli | rs10034695 | T | C | -0.104 | 0.025 | 9.62E-06 | 17.750 |
| class | Clostridia | rs76860606 | G | C | 0.095 | 0.023 | 7.53E-06 | 17.698 |
| class | Negativicutes | rs4463806 | C | T | -0.054 | 0.013 | 7.81E-06 | 17.681 |
| class | Bacilli | rs11726025 | G | A | -0.104 | 0.025 | 8.53E-06 | 17.637 |
| class | Bacilli | rs7666190 | A | C | -0.104 | 0.025 | 8.47E-06 | 17.610 |
| class | Negativicutes | rs10885514 | A | G | -0.053 | 0.013 | 8.58E-06 | 17.172 |
| family | Bifidobacteriaceae | rs182549 | T | C | 0.117 | 0.013 | 5.94E-20 | 85.372 |
| family | Bifidobacteriaceae | rs4988235 | A | G | 0.116 | 0.013 | 1.30E-19 | 84.054 |
| family | Bifidobacteriaceae | rs7570971 | C | A | 0.113 | 0.013 | 2.53E-19 | 80.781 |
| family | Bifidobacteriaceae | rs6754311 | T | C | 0.113 | 0.013 | 8.77E-19 | 79.915 |
| family | Bifidobacteriaceae | rs1446585 | A | G | 0.112 | 0.013 | 4.82E-18 | 76.954 |
| family | Bifidobacteriaceae | rs1375131 | T | C | 0.110 | 0.013 | 3.94E-18 | 76.803 |
| family | Bifidobacteriaceae | rs6730157 | A | G | 0.109 | 0.013 | 4.20E-18 | 76.629 |
| family | Bifidobacteriaceae | rs12465802 | G | A | 0.110 | 0.013 | 6.15E-18 | 75.683 |
| family | Bifidobacteriaceae | rs687670 | T | C | 0.111 | 0.013 | 2.49E-17 | 74.007 |
| family | Bifidobacteriaceae | rs13404551 | T | C | 0.110 | 0.013 | 7.02E-17 | 72.595 |
| family | Bifidobacteriaceae | rs36084664 | A | G | -0.113 | 0.014 | 1.45E-18 | 70.438 |
| family | Bifidobacteriaceae | rs309160 | C | T | 0.108 | 0.013 | 3.14E-16 | 69.414 |
| family | Bifidobacteriaceae | rs309168 | C | T | 0.107 | 0.013 | 5.10E-16 | 68.411 |
| family | Bifidobacteriaceae | rs218174 | A | G | 0.107 | 0.013 | 7.25E-16 | 68.090 |
| family | Bifidobacteriaceae | rs309134 | G | A | 0.107 | 0.013 | 1.35E-15 | 66.643 |
| family | Bifidobacteriaceae | rs309180 | A | G | 0.105 | 0.013 | 1.36E-15 | 66.493 |
| family | Bifidobacteriaceae | rs632632 | T | C | 0.105 | 0.013 | 2.42E-15 | 65.527 |
| family | Bifidobacteriaceae | rs12615624 | A | G | 0.105 | 0.013 | 4.78E-15 | 64.191 |
| family | Bifidobacteriaceae | rs6759321 | G | T | 0.101 | 0.013 | 4.00E-15 | 63.872 |
| family | Bifidobacteriaceae | rs309125 | C | T | 0.103 | 0.013 | 6.64E-15 | 63.343 |
| family | Bifidobacteriaceae | rs13023281 | T | C | -0.106 | 0.013 | 1.12E-16 | 62.561 |
| family | Bifidobacteriaceae | rs1561277 | C | A | 0.100 | 0.013 | 1.28E-14 | 61.678 |
| family | Bifidobacteriaceae | rs309164 | T | C | 0.103 | 0.013 | 1.74E-14 | 61.436 |
| family | Bifidobacteriaceae | rs309149 | C | T | 0.101 | 0.013 | 4.39E-14 | 59.450 |
| family | Bifidobacteriaceae | rs4988183 | T | C | 0.102 | 0.013 | 5.12E-14 | 59.384 |
| family | Bifidobacteriaceae | rs4954490 | A | G | 0.100 | 0.013 | 5.25E-14 | 59.153 |
| family | Bifidobacteriaceae | rs6716536 | C | T | 0.098 | 0.013 | 3.56E-14 | 59.134 |
| family | Bifidobacteriaceae | rs309137 | T | C | 0.099 | 0.013 | 5.22E-14 | 58.752 |
| family | Bifidobacteriaceae | rs2236783 | A | G | 0.099 | 0.013 | 1.15E-13 | 57.801 |
| family | Bifidobacteriaceae | rs11681248 | T | C | 0.094 | 0.012 | 9.61E-14 | 57.117 |
| family | Bifidobacteriaceae | rs12620033 | G | C | 0.098 | 0.013 | 1.68E-13 | 56.975 |
| family | Bifidobacteriaceae | rs3754686 | C | T | 0.097 | 0.013 | 2.82E-13 | 55.615 |
| family | Bifidobacteriaceae | rs3769005 | G | C | 0.096 | 0.013 | 5.29E-13 | 54.165 |
| family | Bifidobacteriaceae | rs745500 | A | G | 0.096 | 0.013 | 6.29E-13 | 54.148 |
| family | Bifidobacteriaceae | rs6719488 | T | G | 0.096 | 0.013 | 6.53E-13 | 53.989 |
| family | Bifidobacteriaceae | rs12475139 | T | A | 0.094 | 0.013 | 5.51E-13 | 53.702 |
| family | Bifidobacteriaceae | rs892715 | C | T | 0.095 | 0.013 | 7.89E-13 | 53.593 |
| family | Bifidobacteriaceae | rs12988076 | A | C | 0.095 | 0.013 | 8.81E-13 | 53.419 |
| family | Bifidobacteriaceae | rs2322660 | T | C | 0.095 | 0.013 | 9.97E-13 | 53.059 |
| family | Bifidobacteriaceae | rs6742283 | G | A | 0.095 | 0.013 | 1.13E-12 | 52.793 |
| family | Bifidobacteriaceae | rs2164210 | C | T | 0.094 | 0.013 | 1.14E-12 | 52.750 |
| family | Bifidobacteriaceae | rs749873 | T | C | 0.090 | 0.012 | 8.79E-13 | 52.662 |
| family | Bifidobacteriaceae | rs748841 | T | C | 0.094 | 0.013 | 1.26E-12 | 52.652 |
| family | Bifidobacteriaceae | rs17261772 | T | C | 0.095 | 0.013 | 1.55E-12 | 52.416 |
| family | Bifidobacteriaceae | rs2011946 | A | C | 0.090 | 0.013 | 1.21E-12 | 52.215 |
| family | Bifidobacteriaceae | rs3739020 | T | G | 0.095 | 0.013 | 3.46E-12 | 50.709 |
| family | Bifidobacteriaceae | rs2082729 | T | C | 0.093 | 0.013 | 6.59E-12 | 49.356 |
| family | Bifidobacteriaceae | rs11693502 | T | C | 0.088 | 0.013 | 4.47E-12 | 49.178 |
| family | Bifidobacteriaceae | rs71348715 | T | G | 0.092 | 0.013 | 8.94E-12 | 48.731 |
| family | Bifidobacteriaceae | rs1438305 | G | C | 0.092 | 0.013 | 9.19E-12 | 48.673 |
| family | Bifidobacteriaceae | rs1011361 | A | G | 0.092 | 0.013 | 8.86E-12 | 48.660 |
| family | Bifidobacteriaceae | rs10202489 | A | G | 0.092 | 0.013 | 9.56E-12 | 48.545 |
| family | Bifidobacteriaceae | rs313519 | A | C | 0.092 | 0.013 | 8.33E-12 | 48.420 |
| family | Bifidobacteriaceae | rs11684545 | T | G | 0.092 | 0.013 | 1.02E-11 | 48.386 |
| family | Bifidobacteriaceae | rs71348714 | A | G | 0.092 | 0.013 | 9.53E-12 | 48.288 |
| family | Bifidobacteriaceae | rs12998016 | T | C | 0.092 | 0.013 | 1.05E-11 | 48.278 |
| family | Bifidobacteriaceae | rs6430589 | C | G | 0.092 | 0.013 | 9.08E-12 | 48.250 |
| family | Bifidobacteriaceae | rs1438304 | G | A | 0.092 | 0.013 | 1.18E-11 | 48.171 |
| family | Bifidobacteriaceae | rs313518 | C | T | 0.092 | 0.013 | 1.03E-11 | 48.167 |
| family | Bifidobacteriaceae | rs10928546 | T | C | 0.092 | 0.013 | 1.18E-11 | 48.156 |
| family | Bifidobacteriaceae | rs1438307 | T | G | 0.092 | 0.013 | 1.16E-11 | 48.137 |
| family | Bifidobacteriaceae | rs28453840 | A | G | 0.092 | 0.013 | 1.24E-11 | 48.074 |
| family | Bifidobacteriaceae | rs313528 | G | A | 0.091 | 0.013 | 1.04E-11 | 48.053 |
| family | Bifidobacteriaceae | rs10195620 | T | A | 0.092 | 0.013 | 1.21E-11 | 48.023 |
| family | Bifidobacteriaceae | rs313526 | C | T | 0.091 | 0.013 | 1.09E-11 | 47.983 |
| family | Bifidobacteriaceae | rs11679933 | T | G | 0.087 | 0.013 | 1.00E-11 | 47.873 |
| family | Bifidobacteriaceae | rs13412397 | A | G | 0.091 | 0.013 | 1.33E-11 | 47.857 |
| family | Bifidobacteriaceae | rs12998387 | G | A | 0.091 | 0.013 | 1.50E-11 | 47.708 |
| family | Bifidobacteriaceae | rs313524 | C | T | 0.091 | 0.013 | 1.33E-11 | 47.520 |
| family | Bifidobacteriaceae | rs3213889 | G | A | 0.091 | 0.013 | 1.68E-11 | 47.511 |
| family | Bifidobacteriaceae | rs313517 | A | G | 0.091 | 0.013 | 1.38E-11 | 47.493 |
| family | Bifidobacteriaceae | rs1470457 | A | G | 0.092 | 0.013 | 1.74E-11 | 47.404 |
| family | Bifidobacteriaceae | rs7355359 | T | C | 0.091 | 0.013 | 1.50E-11 | 47.383 |
| family | Bifidobacteriaceae | rs12994270 | A | T | 0.091 | 0.013 | 2.13E-11 | 47.110 |
| family | Bifidobacteriaceae | rs4954280 | A | G | 0.091 | 0.013 | 1.70E-11 | 47.083 |
| family | Bifidobacteriaceae | rs1446584 | A | G | 0.090 | 0.013 | 1.75E-11 | 47.002 |
| family | Bifidobacteriaceae | rs10173394 | T | C | 0.091 | 0.013 | 2.21E-11 | 46.996 |
| family | Bifidobacteriaceae | rs2034276 | A | T | 0.090 | 0.013 | 1.77E-11 | 46.968 |
| family | Bifidobacteriaceae | rs11679508 | T | A | 0.090 | 0.013 | 2.21E-11 | 46.843 |
| family | Bifidobacteriaceae | rs6720287 | A | G | 0.090 | 0.013 | 1.88E-11 | 46.839 |
| family | Bifidobacteriaceae | rs313522 | T | A | 0.090 | 0.013 | 2.09E-11 | 46.597 |
| family | Bifidobacteriaceae | rs313523 | G | A | 0.090 | 0.013 | 2.08E-11 | 46.591 |
| family | Bifidobacteriaceae | rs6715856 | T | C | 0.090 | 0.013 | 2.26E-11 | 46.471 |
| family | Bifidobacteriaceae | rs6755383 | C | T | 0.090 | 0.013 | 2.25E-11 | 46.446 |
| family | Bifidobacteriaceae | rs12469709 | T | C | 0.090 | 0.013 | 2.72E-11 | 46.390 |
| family | Bifidobacteriaceae | rs656326 | A | C | 0.090 | 0.013 | 2.34E-11 | 46.376 |
| family | Bifidobacteriaceae | rs10192827 | A | G | 0.090 | 0.013 | 3.91E-11 | 45.970 |
| family | Bifidobacteriaceae | rs932206 | T | C | 0.082 | 0.012 | 1.88E-11 | 45.838 |
| family | Bifidobacteriaceae | rs4954276 | A | G | 0.089 | 0.013 | 3.83E-11 | 45.669 |
| family | Bifidobacteriaceae | rs10928542 | G | A | 0.089 | 0.013 | 3.35E-11 | 45.654 |
| family | Bifidobacteriaceae | rs7608045 | T | C | 0.089 | 0.013 | 3.58E-11 | 45.505 |
| family | Bifidobacteriaceae | rs4954279 | G | A | 0.089 | 0.013 | 4.26E-11 | 45.169 |
| family | Bifidobacteriaceae | rs2322659 | C | T | 0.089 | 0.013 | 4.94E-11 | 44.875 |
| family | Bifidobacteriaceae | rs13413639 | C | A | 0.088 | 0.013 | 5.78E-11 | 44.688 |
| family | Bifidobacteriaceae | rs12619365 | C | T | 0.088 | 0.013 | 5.99E-11 | 44.387 |
| family | Bifidobacteriaceae | rs2680880 | T | A | 0.080 | 0.012 | 9.86E-11 | 44.287 |
| family | Bifidobacteriaceae | rs1869829 | A | G | 0.086 | 0.013 | 6.87E-11 | 43.596 |
| family | Bifidobacteriaceae | rs7587285 | T | C | 0.116 | 0.018 | 3.39E-11 | 43.348 |
| family | Bifidobacteriaceae | rs2278683 | A | C | 0.115 | 0.018 | 5.39E-11 | 42.739 |
| family | Bifidobacteriaceae | rs10187402 | C | G | 0.086 | 0.013 | 9.54E-11 | 42.692 |
| family | Bifidobacteriaceae | rs4954551 | G | A | 0.115 | 0.018 | 5.00E-11 | 42.631 |
| family | Bifidobacteriaceae | rs11893318 | T | C | 0.115 | 0.018 | 5.47E-11 | 42.486 |
| family | Bifidobacteriaceae | rs1375132 | A | G | 0.085 | 0.013 | 1.49E-10 | 42.274 |
| family | Bifidobacteriaceae | rs62160860 | G | C | 0.116 | 0.018 | 7.86E-11 | 42.149 |
| family | Bifidobacteriaceae | rs935613 | C | T | 0.084 | 0.013 | 2.63E-10 | 40.991 |
| family | Bifidobacteriaceae | rs2016636 | G | A | 0.084 | 0.013 | 3.23E-10 | 40.741 |
| family | Bifidobacteriaceae | rs6714750 | A | G | 0.084 | 0.013 | 6.28E-10 | 39.957 |
| family | Bifidobacteriaceae | rs1374329 | A | G | 0.085 | 0.013 | 5.57E-10 | 39.908 |
| family | Bifidobacteriaceae | rs10928545 | G | A | 0.086 | 0.014 | 8.21E-10 | 39.645 |
| family | Bifidobacteriaceae | rs1979033 | G | A | 0.075 | 0.012 | 1.05E-10 | 39.424 |
| family | Bifidobacteriaceae | rs9287442 | G | A | 0.085 | 0.014 | 1.05E-09 | 39.050 |
| family | Bifidobacteriaceae | rs12328663 | T | C | 0.103 | 0.017 | 1.19E-10 | 38.929 |
| family | Bifidobacteriaceae | rs2304599 | G | C | 0.085 | 0.014 | 1.12E-09 | 38.856 |
| family | Bifidobacteriaceae | rs2874739 | C | T | 0.103 | 0.017 | 1.71E-10 | 38.596 |
| family | Bifidobacteriaceae | rs12616520 | T | A | 0.084 | 0.014 | 1.34E-09 | 38.570 |
| family | Bifidobacteriaceae | rs6760329 | G | A | 0.085 | 0.014 | 1.21E-09 | 38.501 |
| family | Bifidobacteriaceae | rs10193348 | A | G | 0.103 | 0.017 | 1.70E-10 | 38.500 |
| family | Bifidobacteriaceae | rs4953945 | C | G | 0.103 | 0.017 | 2.92E-10 | 38.497 |
| family | Bifidobacteriaceae | rs6747073 | T | C | 0.081 | 0.013 | 9.76E-10 | 38.409 |
| family | Bifidobacteriaceae | rs10188066 | A | G | 0.084 | 0.014 | 1.35E-09 | 38.317 |
| family | Bifidobacteriaceae | rs1530559 | A | G | 0.074 | 0.012 | 5.23E-10 | 38.029 |
| family | Bifidobacteriaceae | rs4954513 | G | A | 0.112 | 0.018 | 1.50E-09 | 37.669 |
| family | Bifidobacteriaceae | rs309126 | T | C | 0.105 | 0.017 | 1.84E-09 | 37.634 |
| family | Bifidobacteriaceae | rs7568525 | G | A | 0.102 | 0.017 | 2.11E-10 | 37.603 |
| family | Bifidobacteriaceae | rs13389481 | C | A | 0.104 | 0.017 | 3.31E-10 | 37.518 |
| family | Bifidobacteriaceae | rs1030764 | T | C | 0.084 | 0.014 | 2.11E-09 | 37.511 |
| family | Bifidobacteriaceae | rs4346445 | A | C | 0.102 | 0.017 | 2.30E-10 | 37.502 |
| family | Bifidobacteriaceae | rs10208624 | C | T | 0.101 | 0.017 | 2.26E-10 | 37.480 |
| family | Bifidobacteriaceae | rs62168942 | T | C | 0.102 | 0.017 | 3.65E-10 | 37.442 |
| family | Bifidobacteriaceae | rs4954217 | C | T | 0.101 | 0.017 | 3.68E-10 | 37.435 |
| family | Bifidobacteriaceae | rs58697996 | G | A | 0.101 | 0.017 | 3.09E-10 | 37.287 |
| family | Bifidobacteriaceae | rs6704561 | C | T | 0.079 | 0.013 | 1.57E-09 | 37.236 |
| family | Bifidobacteriaceae | rs4954216 | G | A | 0.101 | 0.017 | 3.98E-10 | 37.231 |
| family | Bifidobacteriaceae | rs10167901 | C | T | 0.101 | 0.017 | 3.96E-10 | 37.230 |
| family | Bifidobacteriaceae | rs7581382 | T | C | -0.090 | 0.015 | 5.00E-11 | 37.205 |
| family | Bifidobacteriaceae | rs4953948 | T | G | 0.101 | 0.017 | 3.99E-10 | 37.187 |
| family | Bifidobacteriaceae | rs1551497 | T | C | 0.101 | 0.017 | 4.34E-10 | 37.157 |
| family | Bifidobacteriaceae | rs10167809 | C | T | 0.101 | 0.017 | 4.08E-10 | 37.153 |
| family | Bifidobacteriaceae | rs10167884 | C | T | 0.101 | 0.017 | 4.08E-10 | 37.151 |
| family | Bifidobacteriaceae | rs7422031 | C | T | 0.101 | 0.017 | 3.80E-10 | 37.135 |
| family | Bifidobacteriaceae | rs921859 | A | G | 0.101 | 0.017 | 4.38E-10 | 37.106 |
| family | Bifidobacteriaceae | rs7601696 | C | A | 0.101 | 0.017 | 4.52E-10 | 37.101 |
| family | Bifidobacteriaceae | rs4954215 | G | A | 0.101 | 0.017 | 4.26E-10 | 37.077 |
| family | Bifidobacteriaceae | rs7577258 | G | A | 0.101 | 0.017 | 4.61E-10 | 37.065 |
| family | Bifidobacteriaceae | rs62168941 | C | T | 0.101 | 0.017 | 4.64E-10 | 37.021 |
| family | Bifidobacteriaceae | rs4953947 | A | C | 0.101 | 0.017 | 4.52E-10 | 36.964 |
| family | Bifidobacteriaceae | rs2017831 | C | T | 0.101 | 0.017 | 4.80E-10 | 36.958 |
| family | Bifidobacteriaceae | rs61087958 | C | T | 0.101 | 0.017 | 3.67E-10 | 36.944 |
| family | Bifidobacteriaceae | rs7573600 | G | C | 0.100 | 0.017 | 3.51E-10 | 36.934 |
| family | Bifidobacteriaceae | rs3820794 | T | C | 0.083 | 0.014 | 2.82E-09 | 36.933 |
| family | Bifidobacteriaceae | rs7565268 | T | C | 0.101 | 0.017 | 5.02E-10 | 36.923 |
| family | Bifidobacteriaceae | rs13409400 | A | C | 0.101 | 0.017 | 4.93E-10 | 36.917 |
| family | Bifidobacteriaceae | rs56903548 | C | T | 0.100 | 0.017 | 4.29E-10 | 36.876 |
| family | Bifidobacteriaceae | rs10182750 | T | C | 0.101 | 0.017 | 4.99E-10 | 36.839 |
| family | Bifidobacteriaceae | rs13402101 | T | A | 0.101 | 0.017 | 5.19E-10 | 36.787 |
| family | Bifidobacteriaceae | rs13399376 | A | G | 0.101 | 0.017 | 5.19E-10 | 36.787 |
| family | Bifidobacteriaceae | rs11890844 | A | G | 0.101 | 0.017 | 5.28E-10 | 36.745 |
| family | Bifidobacteriaceae | rs3213871 | C | T | 0.111 | 0.018 | 1.92E-09 | 36.716 |
| family | Bifidobacteriaceae | rs7608980 | G | A | 0.110 | 0.018 | 2.78E-09 | 36.715 |
| family | Bifidobacteriaceae | rs16832138 | G | A | 0.110 | 0.018 | 2.83E-09 | 36.710 |
| family | Bifidobacteriaceae | rs2278544 | G | A | 0.082 | 0.014 | 3.42E-09 | 36.696 |
| family | Bifidobacteriaceae | rs3769002 | T | C | 0.110 | 0.018 | 2.22E-09 | 36.695 |
| family | Bifidobacteriaceae | rs6738563 | G | T | 0.073 | 0.012 | 4.67E-10 | 36.687 |
| family | Bifidobacteriaceae | rs7603805 | A | G | 0.100 | 0.017 | 4.43E-10 | 36.674 |
| family | Bifidobacteriaceae | rs4954220 | C | A | 0.100 | 0.017 | 3.75E-10 | 36.622 |
| family | Bifidobacteriaceae | rs13402112 | A | C | 0.100 | 0.017 | 5.56E-10 | 36.589 |
| family | Bifidobacteriaceae | rs4954214 | C | T | 0.100 | 0.017 | 5.48E-10 | 36.573 |
| family | Bifidobacteriaceae | rs6741481 | A | G | 0.100 | 0.017 | 4.46E-10 | 36.504 |
| family | Bifidobacteriaceae | rs10186594 | A | G | 0.100 | 0.017 | 6.00E-10 | 36.499 |
| family | Bifidobacteriaceae | rs56263017 | T | C | 0.110 | 0.018 | 2.38E-09 | 36.485 |
| family | Bifidobacteriaceae | rs4954212 | A | G | 0.100 | 0.017 | 6.59E-10 | 36.479 |
| family | Bifidobacteriaceae | rs7601683 | C | T | 0.100 | 0.017 | 6.26E-10 | 36.474 |
| family | Bifidobacteriaceae | rs11898045 | T | C | 0.100 | 0.017 | 4.32E-10 | 36.379 |
| family | Bifidobacteriaceae | rs6430561 | C | T | 0.100 | 0.017 | 4.49E-10 | 36.360 |
| family | Bifidobacteriaceae | rs58426504 | T | G | 0.100 | 0.017 | 4.37E-10 | 36.349 |
| family | Bifidobacteriaceae | rs7565149 | T | A | 0.100 | 0.017 | 6.78E-10 | 36.315 |
| family | Bifidobacteriaceae | rs6760553 | A | G | 0.100 | 0.017 | 6.22E-10 | 36.287 |
| family | Bifidobacteriaceae | rs56174523 | A | C | 0.100 | 0.017 | 6.61E-10 | 36.268 |
| family | Bifidobacteriaceae | rs13413872 | G | T | 0.100 | 0.017 | 6.60E-10 | 36.262 |
| family | Bifidobacteriaceae | rs11884903 | T | C | 0.101 | 0.017 | 5.72E-10 | 36.251 |
| family | Bifidobacteriaceae | rs16832275 | C | G | 0.106 | 0.018 | 2.11E-09 | 36.249 |
| family | Bifidobacteriaceae | rs16832156 | G | A | 0.106 | 0.018 | 2.47E-09 | 36.226 |
| family | Bifidobacteriaceae | rs4953946 | T | G | 0.100 | 0.017 | 6.49E-10 | 36.166 |
| family | Bifidobacteriaceae | rs56067372 | C | T | 0.109 | 0.018 | 3.81E-09 | 36.094 |
| family | Bifidobacteriaceae | rs58327215 | G | C | 0.099 | 0.017 | 4.92E-10 | 36.066 |
| family | Bifidobacteriaceae | rs57829242 | A | G | 0.101 | 0.017 | 6.48E-10 | 36.060 |
| family | Bifidobacteriaceae | rs11888951 | T | C | 0.100 | 0.017 | 7.06E-10 | 36.044 |
| family | Bifidobacteriaceae | rs60350840 | A | G | 0.100 | 0.017 | 8.18E-10 | 35.982 |
| family | Bifidobacteriaceae | rs6723108 | G | T | 0.073 | 0.012 | 7.70E-10 | 35.971 |
| family | Bifidobacteriaceae | rs11892750 | G | A | 0.100 | 0.017 | 9.41E-10 | 35.970 |
| family | Bifidobacteriaceae | rs3768998 | G | T | 0.109 | 0.018 | 1.71E-09 | 35.968 |
| family | Bifidobacteriaceae | rs11903663 | A | G | 0.099 | 0.017 | 3.66E-10 | 35.926 |
| family | Bifidobacteriaceae | rs2290518 | G | A | 0.099 | 0.017 | 6.07E-10 | 35.918 |
| family | Bifidobacteriaceae | rs10174944 | C | T | 0.099 | 0.017 | 8.15E-10 | 35.909 |
| family | Bifidobacteriaceae | rs10209482 | A | C | 0.100 | 0.017 | 7.31E-10 | 35.786 |
| family | Bifidobacteriaceae | rs4640444 | T | C | 0.099 | 0.017 | 4.39E-10 | 35.678 |
| family | Bifidobacteriaceae | rs62168934 | A | G | 0.099 | 0.017 | 9.43E-10 | 35.644 |
| family | Bifidobacteriaceae | rs16831264 | G | C | 0.099 | 0.017 | 9.62E-10 | 35.529 |
| family | Bifidobacteriaceae | rs4645053 | T | A | 0.099 | 0.017 | 4.77E-10 | 35.526 |
| family | Bifidobacteriaceae | rs6721838 | A | G | 0.099 | 0.017 | 6.53E-10 | 35.525 |
| family | Bifidobacteriaceae | rs2164332 | G | C | 0.104 | 0.018 | 3.03E-09 | 35.435 |
| family | Bifidobacteriaceae | rs925966 | G | C | 0.076 | 0.013 | 4.52E-09 | 35.335 |
| family | Bifidobacteriaceae | rs13427494 | G | A | 0.100 | 0.017 | 9.35E-10 | 35.309 |
| family | Bifidobacteriaceae | rs309132 | C | G | 0.107 | 0.018 | 5.94E-09 | 35.301 |
| family | Bifidobacteriaceae | rs16831498 | G | C | 0.100 | 0.017 | 9.40E-10 | 35.267 |
| family | Bifidobacteriaceae | rs13410870 | G | A | 0.098 | 0.017 | 8.43E-10 | 35.171 |
| family | Bifidobacteriaceae | rs6709763 | C | A | 0.098 | 0.017 | 1.36E-09 | 35.038 |
| family | Bifidobacteriaceae | rs6752519 | C | T | 0.107 | 0.018 | 4.92E-09 | 34.988 |
| family | Bifidobacteriaceae | rs6739713 | A | G | 0.079 | 0.013 | 7.39E-09 | 34.953 |
| family | Bifidobacteriaceae | rs13408096 | G | T | 0.098 | 0.017 | 1.37E-09 | 34.911 |
| family | Bifidobacteriaceae | rs4494812 | A | G | 0.098 | 0.017 | 7.34E-10 | 34.837 |
| family | Bifidobacteriaceae | rs4954226 | T | C | 0.099 | 0.017 | 1.17E-09 | 34.697 |
| family | Bifidobacteriaceae | rs6430593 | G | A | 0.103 | 0.018 | 6.67E-09 | 34.693 |
| family | Bifidobacteriaceae | rs7371606 | A | G | 0.075 | 0.013 | 6.42E-09 | 34.659 |
| family | Bifidobacteriaceae | rs6737635 | G | T | 0.098 | 0.017 | 1.65E-09 | 34.645 |
| family | Bifidobacteriaceae | rs2322725 | T | C | 0.103 | 0.018 | 4.38E-09 | 34.604 |
| family | Bifidobacteriaceae | rs4954213 | G | T | 0.097 | 0.017 | 1.58E-09 | 34.601 |
| family | Bifidobacteriaceae | rs6749119 | C | G | 0.097 | 0.017 | 1.10E-09 | 34.550 |
| family | Bifidobacteriaceae | rs11897204 | T | C | 0.097 | 0.017 | 1.10E-09 | 34.525 |
| family | Bifidobacteriaceae | rs3754692 | T | C | 0.079 | 0.013 | 9.24E-09 | 34.474 |
| family | Bifidobacteriaceae | rs7597802 | A | T | 0.097 | 0.017 | 2.04E-09 | 34.314 |
| family | Bifidobacteriaceae | rs4954221 | G | A | 0.097 | 0.017 | 1.19E-09 | 34.256 |
| family | Bifidobacteriaceae | rs7563997 | T | A | 0.097 | 0.017 | 2.04E-09 | 34.202 |
| family | Bifidobacteriaceae | rs11888521 | G | C | 0.097 | 0.017 | 1.02E-09 | 34.158 |
| family | Bifidobacteriaceae | rs10928529 | G | A | 0.097 | 0.017 | 2.20E-09 | 34.128 |
| family | Bifidobacteriaceae | rs17293519 | A | G | 0.096 | 0.017 | 1.72E-09 | 34.114 |
| family | Bifidobacteriaceae | rs11900832 | C | T | 0.097 | 0.017 | 2.27E-09 | 34.111 |
| family | Bifidobacteriaceae | rs4954242 | A | G | 0.098 | 0.017 | 2.41E-09 | 34.080 |
| family | Bifidobacteriaceae | rs3754690 | C | T | 0.110 | 0.019 | 8.00E-09 | 33.984 |
| family | Bifidobacteriaceae | rs2164331 | C | T | 0.106 | 0.018 | 7.53E-09 | 33.959 |
| family | Bifidobacteriaceae | rs11889843 | G | A | 0.096 | 0.017 | 2.45E-09 | 33.863 |
| family | Bifidobacteriaceae | rs16832200 | T | C | 0.102 | 0.018 | 6.78E-09 | 33.849 |
| family | Bifidobacteriaceae | rs6706466 | A | G | 0.096 | 0.017 | 1.54E-09 | 33.819 |
| family | Bifidobacteriaceae | rs61663334 | A | G | 0.096 | 0.017 | 1.18E-09 | 33.656 |
| family | Bifidobacteriaceae | rs6742013 | T | C | 0.097 | 0.017 | 2.16E-09 | 33.637 |
| family | Bifidobacteriaceae | rs1900741 | C | T | 0.083 | 0.014 | 1.91E-08 | 33.624 |
| family | Bifidobacteriaceae | rs2305248 | A | G | 0.096 | 0.017 | 1.82E-09 | 33.567 |
| family | Bifidobacteriaceae | rs56369660 | A | G | 0.096 | 0.017 | 2.64E-09 | 33.526 |
| family | Bifidobacteriaceae | rs12233092 | G | A | 0.102 | 0.018 | 8.26E-09 | 33.473 |
| family | Streptococcaceae | rs11110281 | C | T | -0.131 | 0.023 | 1.40E-08 | 33.387 |
| family | Streptococcaceae | rs11110282 | G | A | -0.128 | 0.022 | 1.44E-08 | 33.345 |
| family | Bifidobacteriaceae | rs7605229 | C | T | 0.097 | 0.017 | 2.77E-09 | 33.296 |
| family | Bifidobacteriaceae | rs12478902 | T | C | 0.103 | 0.018 | 9.81E-09 | 33.170 |
| family | Bifidobacteriaceae | rs2874834 | A | G | 0.077 | 0.013 | 1.94E-08 | 33.095 |
| family | Bifidobacteriaceae | rs16855656 | A | G | 0.099 | 0.017 | 1.42E-08 | 33.086 |
| family | Bifidobacteriaceae | rs4954567 | G | T | 0.072 | 0.013 | 9.64E-09 | 33.032 |
| family | Streptococcaceae | rs12368593 | A | C | -0.129 | 0.022 | 1.72E-08 | 33.001 |
| family | Bifidobacteriaceae | rs3806502 | C | T | 0.096 | 0.017 | 2.90E-09 | 32.981 |
| family | Bifidobacteriaceae | rs55634455 | T | C | 0.108 | 0.019 | 1.32E-08 | 32.844 |
| family | Bifidobacteriaceae | rs10179271 | T | C | 0.072 | 0.013 | 1.16E-08 | 32.657 |
| family | Streptococcaceae | rs80243853 | A | G | -0.128 | 0.022 | 2.12E-08 | 32.639 |
| family | Bifidobacteriaceae | rs6727889 | C | T | 0.096 | 0.017 | 4.85E-09 | 32.629 |
| family | Bifidobacteriaceae | rs584226 | T | C | 0.101 | 0.018 | 1.91E-08 | 32.529 |
| family | Streptococcaceae | rs11110283 | C | A | -0.129 | 0.023 | 2.04E-08 | 32.508 |
| family | Peptostreptococcaceae | rs61841503 | A | G | 0.092 | 0.016 | 9.80E-09 | 32.495 |
| family | Streptococcaceae | rs78017832 | G | T | -0.128 | 0.023 | 2.37E-08 | 32.425 |
| family | Bifidobacteriaceae | rs1401687 | G | C | 0.072 | 0.013 | 1.31E-08 | 32.413 |
| family | Streptococcaceae | rs117905191 | G | A | -0.128 | 0.023 | 2.39E-08 | 32.399 |
| family | Bifidobacteriaceae | rs78141345 | G | A | 0.107 | 0.019 | 1.77E-08 | 32.303 |
| family | Streptococcaceae | rs11110268 | T | C | -0.128 | 0.023 | 2.69E-08 | 32.174 |
| family | Bifidobacteriaceae | rs4954568 | T | A | 0.071 | 0.013 | 1.51E-08 | 32.136 |
| family | Bifidobacteriaceae | rs60433564 | A | C | 0.123 | 0.022 | 1.05E-08 | 32.085 |
| family | Bifidobacteriaceae | rs6745996 | A | G | 0.099 | 0.017 | 8.06E-09 | 32.055 |
| family | Bifidobacteriaceae | rs7565053 | A | T | 0.128 | 0.023 | 1.04E-08 | 32.053 |
| family | Bifidobacteriaceae | rs11897217 | C | T | 0.094 | 0.017 | 3.48E-09 | 32.002 |
| family | Bifidobacteriaceae | rs9941547 | G | A | 0.071 | 0.013 | 1.72E-08 | 31.867 |
| family | Bifidobacteriaceae | rs10496736 | C | G | 0.122 | 0.022 | 1.09E-08 | 31.818 |
| family | Bifidobacteriaceae | rs2322445 | T | A | 0.093 | 0.017 | 4.22E-09 | 31.767 |
| family | Bifidobacteriaceae | rs7594509 | G | T | 0.093 | 0.017 | 4.01E-09 | 31.747 |
| family | Bifidobacteriaceae | rs6705610 | T | G | 0.093 | 0.017 | 4.25E-09 | 31.718 |
| family | Streptococcaceae | rs17030005 | C | T | -0.126 | 0.022 | 3.84E-08 | 31.551 |
| family | Streptococcaceae | rs9669453 | G | A | -0.125 | 0.022 | 4.12E-08 | 31.421 |
| family | Streptococcaceae | rs78843973 | C | T | -0.126 | 0.023 | 3.84E-08 | 31.407 |
| family | Streptococcaceae | rs17851246 | G | A | -0.126 | 0.022 | 4.10E-08 | 31.403 |
| family | Bifidobacteriaceae | rs998451 | A | G | 0.068 | 0.012 | 7.44E-09 | 31.361 |
| family | Streptococcaceae | rs11110276 | G | T | -0.124 | 0.022 | 3.96E-08 | 31.335 |
| family | Bifidobacteriaceae | rs57543738 | T | C | 0.106 | 0.019 | 2.16E-08 | 31.257 |
| family | Peptostreptococcaceae | rs11595451 | C | T | 0.090 | 0.016 | 1.85E-08 | 31.155 |
| family | Streptococcaceae | rs7959495 | A | C | -0.125 | 0.022 | 4.83E-08 | 31.127 |
| family | Streptococcaceae | rs74348118 | T | C | -0.124 | 0.022 | 4.96E-08 | 31.100 |
| family | Streptococcaceae | rs11110264 | A | G | -0.126 | 0.023 | 4.47E-08 | 31.096 |
| family | Streptococcaceae | rs7294396 | C | G | -0.120 | 0.022 | 4.83E-08 | 31.019 |
| family | Bifidobacteriaceae | rs56211644 | T | C | 0.105 | 0.019 | 3.18E-08 | 30.950 |
| family | Streptococcaceae | rs11835118 | C | T | -0.123 | 0.022 | 4.95E-08 | 30.938 |
| family | Streptococcaceae | rs17029986 | T | G | -0.120 | 0.022 | 5.03E-08 | 30.911 |
| family | Bifidobacteriaceae | rs4954569 | C | T | 0.070 | 0.013 | 2.86E-08 | 30.854 |
| family | Streptococcaceae | rs17030023 | A | T | -0.124 | 0.022 | 5.66E-08 | 30.804 |
| family | Bifidobacteriaceae | rs6715785 | G | A | 0.070 | 0.013 | 3.00E-08 | 30.788 |
| family | Streptococcaceae | rs11837200 | T | C | -0.123 | 0.022 | 5.49E-08 | 30.717 |
| family | Streptococcaceae | rs116878826 | T | C | -0.124 | 0.022 | 5.65E-08 | 30.605 |
| family | Bifidobacteriaceae | rs4954430 | C | T | 0.120 | 0.022 | 1.37E-08 | 30.596 |
| family | Streptococcaceae | rs75132655 | T | G | -0.111 | 0.020 | 5.20E-08 | 30.575 |
| family | Streptococcaceae | rs79728246 | T | C | -0.123 | 0.022 | 6.48E-08 | 30.575 |
| family | Bifidobacteriaceae | rs950176 | A | G | 0.070 | 0.013 | 3.52E-08 | 30.563 |
| family | Bifidobacteriaceae | rs4954392 | A | G | 0.070 | 0.013 | 3.21E-08 | 30.559 |
| family | Bifidobacteriaceae | rs4954570 | G | C | 0.074 | 0.013 | 2.61E-08 | 30.464 |
| family | Streptococcaceae | rs11522881 | C | T | -0.124 | 0.022 | 6.67E-08 | 30.461 |
| family | Bifidobacteriaceae | rs12616955 | C | T | 0.069 | 0.013 | 3.45E-08 | 30.424 |
| family | Streptococcaceae | rs4374023 | G | A | -0.123 | 0.022 | 6.73E-08 | 30.416 |
| family | Bifidobacteriaceae | rs60376570 | G | A | 0.104 | 0.019 | 3.46E-08 | 30.350 |
| family | Bifidobacteriaceae | rs7322849 | C | T | 0.111 | 0.020 | 1.74E-08 | 30.320 |
| family | Streptococcaceae | rs12371930 | G | A | -0.130 | 0.024 | 5.39E-08 | 30.310 |
| family | Streptococcaceae | rs12369047 | T | G | -0.111 | 0.020 | 6.20E-08 | 30.310 |
| family | Streptococcaceae | rs12370758 | T | C | -0.125 | 0.023 | 6.85E-08 | 30.304 |
| family | Streptococcaceae | rs11110145 | A | T | -0.111 | 0.020 | 6.51E-08 | 30.263 |
| family | Bifidobacteriaceae | rs6430580 | A | G | 0.096 | 0.017 | 1.96E-08 | 30.256 |
| family | Streptococcaceae | rs7313123 | T | C | -0.110 | 0.020 | 6.13E-08 | 30.240 |
| family | Bifidobacteriaceae | rs4954633 | C | T | 0.118 | 0.022 | 1.67E-08 | 30.235 |
| family | Bifidobacteriaceae | rs62170081 | A | G | 0.119 | 0.022 | 2.78E-08 | 30.230 |
| family | Streptococcaceae | rs117585280 | T | A | -0.124 | 0.023 | 7.12E-08 | 30.212 |
| family | Bifidobacteriaceae | rs10928554 | C | T | 0.100 | 0.018 | 1.56E-07 | 30.162 |
| family | Bifidobacteriaceae | rs4954566 | C | T | 0.069 | 0.013 | 4.15E-08 | 30.081 |
| family | Bifidobacteriaceae | rs35215526 | G | A | 0.107 | 0.020 | 7.28E-08 | 30.069 |
| family | Bifidobacteriaceae | rs4954564 | A | G | 0.069 | 0.013 | 4.58E-08 | 30.042 |
| family | Streptococcaceae | rs12372388 | G | A | -0.122 | 0.022 | 7.95E-08 | 30.038 |
| family | Streptococcaceae | rs12368073 | C | A | -0.117 | 0.021 | 7.73E-08 | 30.022 |
| family | Bifidobacteriaceae | rs10928513 | C | T | 0.066 | 0.012 | 2.74E-08 | 29.965 |
| family | Oxalobacteraceae | rs4428215 | A | G | 0.126 | 0.023 | 4.88E-08 | 29.812 |
| family | Bifidobacteriaceae | rs6729961 | T | G | 0.118 | 0.022 | 4.11E-08 | 29.806 |
| family | Streptococcaceae | rs11110235 | T | C | -0.121 | 0.022 | 9.83E-08 | 29.773 |
| family | Bifidobacteriaceae | rs7565871 | G | C | 0.091 | 0.017 | 1.13E-08 | 29.729 |
| family | Streptococcaceae | rs11110158 | G | A | -0.126 | 0.023 | 8.06E-08 | 29.698 |
| family | Streptococcaceae | rs7296331 | C | T | -0.121 | 0.022 | 1.03E-07 | 29.676 |
| family | Streptococcaceae | rs11110288 | C | T | -0.121 | 0.022 | 9.54E-08 | 29.647 |
| family | Streptococcaceae | rs12367656 | T | C | -0.122 | 0.022 | 1.03E-07 | 29.646 |
| family | Bifidobacteriaceae | rs56982689 | C | T | 0.118 | 0.022 | 1.78E-08 | 29.630 |
| family | Streptococcaceae | rs7967270 | A | C | -0.110 | 0.020 | 8.93E-08 | 29.624 |
| family | Bifidobacteriaceae | rs2874874 | A | C | 0.103 | 0.019 | 5.28E-08 | 29.597 |
| family | Bifidobacteriaceae | rs10803550 | C | T | 0.068 | 0.013 | 5.22E-08 | 29.583 |
| family | Bifidobacteriaceae | rs6720497 | G | A | 0.118 | 0.022 | 4.10E-08 | 29.582 |
| family | Bifidobacteriaceae | rs6707289 | A | G | 0.118 | 0.022 | 4.10E-08 | 29.581 |
| family | Peptostreptococcaceae | rs61844362 | T | C | 0.081 | 0.015 | 5.78E-08 | 29.537 |
| family | Streptococcaceae | rs11110148 | G | C | -0.109 | 0.020 | 9.40E-08 | 29.516 |
| family | Streptococcaceae | rs2559847 | C | T | -0.122 | 0.022 | 9.86E-08 | 29.491 |
| family | Bifidobacteriaceae | rs4954396 | A | C | 0.069 | 0.013 | 5.27E-08 | 29.456 |
| family | Bifidobacteriaceae | rs4954572 | G | T | 0.069 | 0.013 | 5.44E-08 | 29.403 |
| family | Bifidobacteriaceae | rs953387 | A | C | 0.068 | 0.013 | 5.67E-08 | 29.387 |
| family | Bifidobacteriaceae | rs58080116 | A | G | 0.102 | 0.019 | 6.55E-08 | 29.305 |
| family | Streptococcaceae | rs78367981 | G | C | -0.121 | 0.022 | 1.14E-07 | 29.288 |
| family | Bifidobacteriaceae | rs2102896 | T | C | 0.068 | 0.013 | 5.88E-08 | 29.285 |
| family | Bifidobacteriaceae | rs72972104 | G | A | 0.116 | 0.022 | 2.73E-08 | 29.249 |
| family | Bifidobacteriaceae | rs74266324 | G | A | 0.082 | 0.015 | 5.45E-08 | 29.201 |
| family | Streptococcaceae | rs11110241 | T | C | -0.116 | 0.021 | 1.10E-07 | 29.192 |
| family | Streptococcaceae | rs114272490 | T | C | -0.109 | 0.020 | 1.21E-07 | 29.084 |
| family | Streptococcaceae | rs74767263 | C | G | -0.109 | 0.020 | 1.18E-07 | 29.079 |
| family | Bifidobacteriaceae | rs2322812 | A | G | 0.102 | 0.019 | 7.07E-08 | 29.075 |
| family | Bifidobacteriaceae | rs72970286 | T | G | 0.115 | 0.021 | 3.92E-08 | 29.057 |
| family | Bifidobacteriaceae | rs2056297 | G | T | 0.068 | 0.013 | 7.24E-08 | 29.015 |
| family | Streptococcaceae | rs116172730 | G | A | -0.120 | 0.022 | 1.39E-07 | 29.004 |
| family | Streptococcaceae | rs11110138 | T | C | -0.109 | 0.020 | 1.18E-07 | 28.977 |
| family | Bifidobacteriaceae | rs1807356 | T | C | 0.092 | 0.017 | 4.88E-08 | 28.969 |
| family | Bifidobacteriaceae | rs62168847 | T | C | 0.105 | 0.019 | 1.08E-07 | 28.935 |
| family | Bifidobacteriaceae | rs62168846 | G | A | 0.105 | 0.020 | 1.38E-07 | 28.934 |
| family | Streptococcaceae | rs7138882 | A | G | -0.120 | 0.022 | 1.40E-07 | 28.873 |
| family | Bifidobacteriaceae | rs13010498 | G | C | -0.069 | 0.013 | 6.09E-08 | 28.853 |
| family | Streptococcaceae | rs57062458 | C | T | -0.115 | 0.021 | 1.18E-07 | 28.840 |
| family | Streptococcaceae | rs11110129 | A | G | -0.108 | 0.020 | 1.37E-07 | 28.765 |
| family | Oxalobacteraceae | rs7643618 | G | A | 0.123 | 0.023 | 8.82E-08 | 28.757 |
| family | Bifidobacteriaceae | rs74697610 | A | T | 0.112 | 0.021 | 7.76E-08 | 28.663 |
| family | Streptococcaceae | rs12370204 | C | T | -0.108 | 0.020 | 1.41E-07 | 28.652 |
| family | Streptococcaceae | rs114181124 | G | A | -0.108 | 0.020 | 1.49E-07 | 28.643 |
| family | Bifidobacteriaceae | rs62168843 | T | C | 0.104 | 0.019 | 1.20E-07 | 28.624 |
| family | Streptococcaceae | rs11110204 | A | G | -0.120 | 0.022 | 1.76E-07 | 28.589 |
| family | Oxalobacteraceae | rs7644098 | G | C | 0.123 | 0.023 | 9.42E-08 | 28.586 |
| family | Bifidobacteriaceae | rs3769011 | A | T | 0.101 | 0.019 | 9.05E-08 | 28.569 |
| family | Streptococcaceae | rs11110250 | T | C | -0.118 | 0.022 | 1.75E-07 | 28.516 |
| family | Bifidobacteriaceae | rs4954577 | A | G | 0.068 | 0.013 | 8.89E-08 | 28.491 |
| family | Streptococcaceae | rs115024257 | G | A | -0.108 | 0.020 | 1.44E-07 | 28.459 |
| family | Bifidobacteriaceae | rs4954445 | T | A | 0.101 | 0.019 | 9.27E-08 | 28.440 |
| family | Streptococcaceae | rs11110251 | A | C | -0.118 | 0.022 | 1.83E-07 | 28.423 |
| family | Bifidobacteriaceae | rs1123848 | C | T | 0.068 | 0.013 | 8.89E-08 | 28.401 |
| family | Erysipelotrichaceae | rs62504403 | T | C | 0.068 | 0.013 | 1.12E-07 | 28.371 |
| family | Bifidobacteriaceae | rs4954397 | A | G | 0.068 | 0.013 | 9.39E-08 | 28.368 |
| family | Bifidobacteriaceae | rs12473760 | C | A | 0.067 | 0.013 | 9.33E-08 | 28.361 |
| family | Bifidobacteriaceae | rs16831994 | T | C | 0.114 | 0.022 | 6.36E-08 | 28.314 |
| family | Bifidobacteriaceae | rs1964776 | C | T | 0.067 | 0.013 | 9.83E-08 | 28.275 |
| family | Bifidobacteriaceae | rs61470941 | T | C | 0.100 | 0.019 | 8.01E-08 | 28.264 |
| family | Streptococcaceae | rs11110211 | T | G | -0.118 | 0.022 | 2.17E-07 | 28.162 |
| family | Streptococcaceae | rs150200257 | G | A | -0.123 | 0.023 | 1.82E-07 | 28.112 |
| family | Victivallaceae | rs4396289 | T | C | -0.153 | 0.029 | 1.54E-07 | 28.090 |
| family | Bifidobacteriaceae | rs16833335 | G | A | 0.067 | 0.013 | 1.13E-07 | 28.086 |
| family | Peptostreptococcaceae | rs76209010 | C | A | 0.078 | 0.015 | 1.21E-07 | 28.035 |
| family | Peptostreptococcaceae | rs41335345 | C | T | 0.086 | 0.016 | 9.59E-08 | 28.009 |
| family | Bifidobacteriaceae | rs4954388 | T | C | 0.068 | 0.013 | 1.43E-07 | 28.006 |
| family | Streptococcaceae | rs10860540 | A | C | -0.105 | 0.020 | 2.02E-07 | 28.005 |
| family | Streptococcaceae | rs11832216 | C | T | -0.117 | 0.022 | 2.44E-07 | 27.999 |
| family | Peptostreptococcaceae | rs61844361 | T | C | 0.079 | 0.015 | 1.36E-07 | 27.986 |
| family | Streptococcaceae | rs112118907 | C | A | -0.118 | 0.022 | 2.50E-07 | 27.955 |
| family | Bifidobacteriaceae | rs7564577 | C | T | 0.089 | 0.017 | 5.26E-08 | 27.907 |
| family | Bifidobacteriaceae | rs1942055 | G | C | 0.064 | 0.012 | 8.32E-08 | 27.889 |
| family | Oxalobacteraceae | rs4481179 | C | G | 0.121 | 0.023 | 1.32E-07 | 27.871 |
| family | Victivallaceae | rs56349194 | G | A | -0.152 | 0.029 | 1.78E-07 | 27.864 |
| family | Bifidobacteriaceae | rs13008147 | A | G | -0.071 | 0.013 | 2.50E-08 | 27.857 |
| family | Bifidobacteriaceae | rs4954578 | T | C | 0.067 | 0.013 | 1.37E-07 | 27.842 |
| family | Streptococcaceae | rs11110140 | T | C | -0.106 | 0.020 | 2.19E-07 | 27.842 |
| family | Streptococcaceae | rs11110257 | C | T | -0.117 | 0.022 | 2.55E-07 | 27.813 |
| family | Prevotellaceae | rs9958960 | A | G | -0.091 | 0.017 | 1.06E-07 | 27.765 |
| family | Streptococcaceae | rs2131006 | A | G | -0.105 | 0.020 | 2.79E-07 | 27.760 |
| family | Oxalobacteraceae | rs736744 | T | C | 0.106 | 0.020 | 1.49E-07 | 27.721 |
| family | Streptococcaceae | rs11110118 | C | T | -0.106 | 0.020 | 2.28E-07 | 27.663 |
| family | Streptococcaceae | rs74484094 | C | T | -0.117 | 0.022 | 3.07E-07 | 27.632 |
| family | Streptococcaceae | rs75007764 | G | A | -0.116 | 0.022 | 2.70E-07 | 27.595 |
| family | Oxalobacteraceae | rs12635823 | A | G | 0.121 | 0.023 | 1.55E-07 | 27.529 |
| family | Bifidobacteriaceae | rs7609188 | G | C | 0.113 | 0.022 | 8.16E-08 | 27.529 |
| family | Streptococcaceae | rs56843411 | G | A | -0.115 | 0.022 | 2.64E-07 | 27.513 |
| family | Streptococcaceae | rs10860539 | C | T | -0.104 | 0.020 | 2.67E-07 | 27.467 |
| family | Streptococcaceae | rs12370791 | T | G | -0.116 | 0.022 | 2.95E-07 | 27.448 |
| family | Oxalobacteraceae | rs2132164 | G | C | 0.123 | 0.023 | 1.83E-07 | 27.445 |
| family | Streptococcaceae | rs10860537 | C | T | -0.106 | 0.020 | 2.52E-07 | 27.440 |
| family | Streptococcaceae | rs11110160 | A | G | -0.105 | 0.020 | 2.91E-07 | 27.405 |
| family | Peptostreptococcaceae | rs74668791 | T | G | 0.078 | 0.015 | 1.95E-07 | 27.397 |
| family | Oxalobacteraceae | rs4525896 | G | A | 0.123 | 0.023 | 1.97E-07 | 27.345 |
| family | Bifidobacteriaceae | rs13007153 | T | C | 0.068 | 0.013 | 2.09E-07 | 27.334 |
| family | Bifidobacteriaceae | rs1961273 | T | C | 0.069 | 0.013 | 1.70E-07 | 27.300 |
| family | Oxalobacteraceae | rs4423796 | C | T | 0.122 | 0.023 | 1.91E-07 | 27.286 |
| family | Bifidobacteriaceae | rs1519525 | C | T | 0.066 | 0.013 | 1.72E-07 | 27.280 |
| family | Streptococcaceae | rs7312868 | G | A | -0.105 | 0.020 | 3.02E-07 | 27.273 |
| family | Oxalobacteraceae | rs7428410 | C | T | 0.122 | 0.023 | 2.05E-07 | 27.225 |
| family | Peptostreptococcaceae | rs61844360 | G | A | 0.078 | 0.015 | 2.00E-07 | 27.224 |
| family | Streptococcaceae | rs11110196 | C | A | -0.105 | 0.020 | 2.95E-07 | 27.218 |
| family | Streptococcaceae | rs7298009 | T | C | -0.105 | 0.020 | 3.15E-07 | 27.187 |
| family | Ruminococcaceae | rs117677831 | A | G | 0.098 | 0.019 | 1.76E-07 | 27.172 |
| family | Streptococcaceae | rs11110198 | G | A | -0.105 | 0.020 | 2.95E-07 | 27.158 |
| family | Bifidobacteriaceae | rs80274970 | C | T | 0.108 | 0.021 | 2.18E-07 | 27.153 |
| family | Veillonellaceae | rs12741784 | T | C | -0.062 | 0.012 | 1.28E-07 | 27.151 |
| family | Oxalobacteraceae | rs13073573 | C | T | 0.122 | 0.023 | 2.12E-07 | 27.146 |
| family | Oxalobacteraceae | rs34124854 | C | T | 0.122 | 0.023 | 2.24E-07 | 27.126 |
| family | BacteroidalesS24 | rs941000 | T | C | 0.085 | 0.016 | 3.16E-07 | 27.083 |
| family | Streptococcaceae | rs138155218 | C | G | -0.105 | 0.020 | 3.23E-07 | 27.079 |
| family | Peptostreptococcaceae | rs61841463 | C | T | 0.077 | 0.015 | 2.10E-07 | 27.051 |
| family | Bifidobacteriaceae | rs62168844 | A | G | 0.101 | 0.019 | 2.74E-07 | 27.040 |
| family | Oxalobacteraceae | rs4894821 | G | A | 0.122 | 0.023 | 2.29E-07 | 27.035 |
| family | Streptococcaceae | rs11110110 | G | A | -0.105 | 0.020 | 3.21E-07 | 27.004 |
| family | Ruminococcaceae | rs55793120 | C | T | 0.138 | 0.027 | 1.44E-07 | 26.970 |
| family | Peptostreptococcaceae | rs61841475 | G | A | 0.074 | 0.014 | 1.73E-07 | 26.954 |
| family | Pasteurellaceae | rs9382510 | T | C | -0.088 | 0.017 | 2.48E-07 | 26.921 |
| family | Peptostreptococcaceae | rs11591519 | G | A | 0.084 | 0.016 | 1.79E-07 | 26.914 |
| family | Bifidobacteriaceae | rs3739022 | G | A | 0.098 | 0.019 | 1.66E-07 | 26.904 |
| family | Oxalobacteraceae | rs1317940 | A | G | 0.122 | 0.023 | 2.39E-07 | 26.885 |
| family | Erysipelotrichaceae | rs11990327 | G | A | 0.067 | 0.013 | 2.45E-07 | 26.880 |
| family | Pasteurellaceae | rs12191680 | G | C | 0.102 | 0.020 | 3.05E-07 | 26.869 |
| family | Streptococcaceae | rs11110130 | A | T | -0.105 | 0.020 | 3.56E-07 | 26.845 |
| family | Bifidobacteriaceae | rs60274701 | G | A | 0.097 | 0.019 | 2.25E-07 | 26.844 |
| family | Victivallaceae | rs12575328 | G | A | -0.150 | 0.029 | 2.97E-07 | 26.821 |
| family | Oxalobacteraceae | rs6445047 | C | T | 0.120 | 0.023 | 2.52E-07 | 26.808 |
| family | Peptostreptococcaceae | rs11594134 | T | C | 0.084 | 0.016 | 1.97E-07 | 26.804 |
| family | Bifidobacteriaceae | rs6733547 | G | T | 0.087 | 0.017 | 1.45E-07 | 26.789 |
| family | Peptostreptococcaceae | rs7913601 | C | T | 0.077 | 0.015 | 2.47E-07 | 26.784 |
| family | Methanobacteriaceae | rs10202904 | G | T | -0.122 | 0.024 | 3.01E-07 | 26.761 |
| family | Bifidobacteriaceae | rs872151 | C | T | 0.096 | 0.019 | 1.81E-07 | 26.746 |
| family | Porphyromonadaceae | rs10119172 | G | C | -0.074 | 0.014 | 1.32E-07 | 26.739 |
| family | Erysipelotrichaceae | rs11990200 | G | A | 0.067 | 0.013 | 2.37E-07 | 26.711 |
| family | Peptostreptococcaceae | rs61841466 | G | A | 0.077 | 0.015 | 2.45E-07 | 26.668 |
| family | Oxalobacteraceae | rs4622921 | A | G | 0.121 | 0.023 | 2.79E-07 | 26.667 |
| family | Peptostreptococcaceae | rs78114045 | C | T | 0.077 | 0.015 | 2.47E-07 | 26.663 |
| family | Streptococcaceae | rs12366744 | T | A | -0.114 | 0.022 | 4.21E-07 | 26.656 |
| family | Peptostreptococcaceae | rs61841473 | G | A | 0.073 | 0.014 | 2.04E-07 | 26.636 |
| family | Oxalobacteraceae | rs6445049 | T | G | 0.120 | 0.023 | 2.64E-07 | 26.621 |
| family | Alcaligenaceae | rs9537886 | C | A | -0.057 | 0.011 | 2.35E-07 | 26.604 |
| family | Bifidobacteriaceae | rs10928558 | T | C | 0.066 | 0.013 | 2.34E-07 | 26.584 |
| family | Streptococcaceae | rs11110249 | T | A | -0.116 | 0.022 | 4.76E-07 | 26.566 |
| family | Bifidobacteriaceae | rs6756200 | T | C | 0.062 | 0.012 | 2.33E-07 | 26.528 |
| family | Rhodospirillaceae | rs9813022 | G | A | -0.084 | 0.016 | 2.53E-07 | 26.522 |
| family | Alcaligenaceae | rs2321387 | A | G | -0.057 | 0.011 | 2.46E-07 | 26.513 |
| family | Oxalobacteraceae | rs6772385 | C | A | 0.120 | 0.023 | 2.77E-07 | 26.512 |
| family | BacteroidalesS24 | rs696266 | G | A | 0.084 | 0.016 | 4.27E-07 | 26.506 |
| family | Pasteurellaceae | rs7740611 | C | A | -0.088 | 0.017 | 3.08E-07 | 26.505 |
| family | Oxalobacteraceae | rs6782568 | C | A | 0.121 | 0.023 | 2.73E-07 | 26.492 |
| family | Rikenellaceae | rs9603208 | T | G | 0.082 | 0.016 | 1.92E-07 | 26.474 |
| family | Streptococcaceae | rs12369165 | C | T | -0.103 | 0.020 | 4.37E-07 | 26.472 |
| family | Peptostreptococcaceae | rs61841474 | A | T | 0.073 | 0.014 | 2.22E-07 | 26.465 |
| family | Streptococcaceae | rs12371009 | A | G | -0.103 | 0.020 | 4.62E-07 | 26.464 |
| family | Peptostreptococcaceae | rs7899152 | A | G | 0.073 | 0.014 | 2.24E-07 | 26.441 |
| family | Peptostreptococcaceae | rs61841467 | A | G | 0.076 | 0.015 | 2.78E-07 | 26.430 |
| family | BacteroidalesS24 | rs17043770 | A | G | -0.177 | 0.034 | 7.10E-07 | 26.426 |
| family | Peptostreptococcaceae | rs113922985 | C | T | 0.073 | 0.014 | 2.27E-07 | 26.417 |
| family | Rikenellaceae | rs9594211 | C | T | 0.082 | 0.016 | 1.98E-07 | 26.400 |
| family | Streptococcaceae | rs11110200 | A | T | -0.116 | 0.023 | 5.23E-07 | 26.396 |
| family | ClostridialesvadinBB60group | rs28691777 | T | C | 0.137 | 0.027 | 6.96E-07 | 26.380 |
| family | Streptococcaceae | rs11495178 | C | A | -0.103 | 0.020 | 4.93E-07 | 26.340 |
| family | Bifidobacteriaceae | rs10171677 | G | A | 0.062 | 0.012 | 1.40E-07 | 26.317 |
| family | Oxalobacteraceae | rs4243424 | C | T | 0.120 | 0.023 | 3.36E-07 | 26.304 |
| family | Bifidobacteriaceae | rs10193587 | T | C | 0.066 | 0.013 | 2.40E-07 | 26.304 |
| family | Rikenellaceae | rs1107244 | A | G | 0.087 | 0.017 | 1.92E-07 | 26.296 |
| family | Oxalobacteraceae | rs35327886 | A | G | 0.120 | 0.023 | 3.39E-07 | 26.285 |
| family | Lachnospiraceae | rs9929145 | A | G | -0.126 | 0.025 | 2.84E-07 | 26.282 |
| family | Oxalobacteraceae | rs6785343 | C | T | 0.120 | 0.023 | 3.09E-07 | 26.280 |
| family | Prevotellaceae | rs4493272 | C | T | -0.060 | 0.012 | 3.02E-07 | 26.275 |
| family | Streptococcaceae | rs113804138 | C | T | -0.113 | 0.022 | 5.44E-07 | 26.266 |
| family | Peptostreptococcaceae | rs7075681 | G | C | 0.076 | 0.015 | 3.51E-07 | 26.250 |
| family | Bifidobacteriaceae | rs3820790 | A | T | 0.098 | 0.019 | 3.11E-07 | 26.246 |
| family | Bifidobacteriaceae | rs12992815 | C | G | 0.096 | 0.019 | 2.38E-07 | 26.243 |
| family | Oxalobacteraceae | rs4280664 | G | A | 0.120 | 0.023 | 3.47E-07 | 26.237 |
| family | Prevotellaceae | rs9952932 | C | T | -0.088 | 0.017 | 3.01E-07 | 26.232 |
| family | Alcaligenaceae | rs62191117 | G | A | 0.068 | 0.013 | 2.76E-07 | 26.221 |
| family | Rikenellaceae | rs1939881 | A | G | -0.106 | 0.021 | 5.64E-07 | 26.219 |
| family | Oxalobacteraceae | rs13060354 | A | G | 0.120 | 0.023 | 3.56E-07 | 26.198 |
| family | Bifidobacteriaceae | rs2090661 | A | G | 0.066 | 0.013 | 3.34E-07 | 26.189 |
| family | Alcaligenaceae | rs9537885 | G | A | -0.057 | 0.011 | 2.90E-07 | 26.187 |
| family | Bacteroidaceae | rs6795673 | T | C | 0.054 | 0.011 | 3.38E-07 | 26.183 |
| family | Streptococcaceae | rs76452890 | G | C | -0.113 | 0.022 | 5.85E-07 | 26.168 |
| family | Bifidobacteriaceae | rs4954573 | T | G | 0.066 | 0.013 | 2.68E-07 | 26.160 |
| family | Rikenellaceae | rs9594210 | C | T | 0.082 | 0.016 | 2.25E-07 | 26.155 |
| family | Erysipelotrichaceae | rs62504402 | C | T | 0.066 | 0.013 | 2.88E-07 | 26.152 |
| family | Defluviitaleaceae | rs2357086 | C | T | -0.112 | 0.022 | 8.47E-07 | 26.152 |
| family | Streptococcaceae | rs11110184 | A | G | -0.103 | 0.020 | 5.56E-07 | 26.146 |
| family | Rikenellaceae | rs9594206 | G | A | 0.082 | 0.016 | 2.13E-07 | 26.145 |
| family | Streptococcaceae | rs11110214 | A | G | -0.112 | 0.022 | 5.52E-07 | 26.110 |
| family | BacteroidalesS24 | rs738193 | C | T | 0.085 | 0.017 | 3.82E-07 | 26.100 |
| family | Prevotellaceae | rs6733556 | G | C | -0.060 | 0.012 | 3.37E-07 | 26.099 |
| family | Rikenellaceae | rs9603209 | T | C | 0.082 | 0.016 | 2.20E-07 | 26.097 |
| family | Oxalobacteraceae | rs4243423 | A | G | 0.120 | 0.023 | 3.76E-07 | 26.086 |
| family | Bifidobacteriaceae | rs4954272 | A | G | 0.089 | 0.018 | 3.17E-07 | 26.076 |
| family | Prevotellaceae | rs12118202 | C | T | -0.075 | 0.015 | 5.54E-07 | 26.075 |
| family | Oxalobacteraceae | rs7625948 | C | G | 0.119 | 0.023 | 3.53E-07 | 26.063 |
| family | Streptococcaceae | rs11110190 | T | C | -0.103 | 0.020 | 5.84E-07 | 26.048 |
| family | Defluviitaleaceae | rs72731813 | T | C | -0.150 | 0.029 | 2.76E-07 | 26.048 |
| family | Defluviitaleaceae | rs12501518 | A | G | -0.111 | 0.022 | 9.06E-07 | 26.012 |
| family | Streptococcaceae | rs11110212 | T | C | -0.112 | 0.022 | 5.91E-07 | 26.005 |
| family | Streptococcaceae | rs2029585 | T | C | -0.112 | 0.022 | 5.91E-07 | 26.004 |
| family | Rikenellaceae | rs34299625 | A | T | -0.105 | 0.021 | 5.80E-07 | 25.979 |
| family | Lactobacillaceae | rs16861661 | A | G | -0.193 | 0.038 | 2.70E-07 | 25.969 |
| family | Rikenellaceae | rs9603207 | T | A | 0.081 | 0.016 | 2.52E-07 | 25.962 |
| family | Victivallaceae | rs62570196 | T | C | -0.246 | 0.048 | 2.70E-07 | 25.953 |
| family | Alcaligenaceae | rs9537878 | G | A | -0.056 | 0.011 | 3.27E-07 | 25.949 |
| family | Alcaligenaceae | rs7988877 | T | A | -0.058 | 0.011 | 3.15E-07 | 25.946 |
| family | Rikenellaceae | rs6563551 | T | G | 0.087 | 0.017 | 2.32E-07 | 25.937 |
| family | Streptococcaceae | rs11110191 | C | T | -0.102 | 0.020 | 6.22E-07 | 25.929 |
| family | Defluviitaleaceae | rs12500663 | G | T | -0.116 | 0.023 | 1.09E-06 | 25.927 |
| family | Rikenellaceae | rs9603206 | T | G | 0.081 | 0.016 | 2.58E-07 | 25.910 |
| family | Rikenellaceae | rs56406841 | T | A | 0.081 | 0.016 | 2.40E-07 | 25.890 |
| family | Peptostreptococcaceae | rs61841472 | T | C | 0.072 | 0.014 | 3.18E-07 | 25.889 |
| family | Rikenellaceae | rs17055747 | A | C | 0.081 | 0.016 | 2.45E-07 | 25.886 |
| family | Rikenellaceae | rs9594205 | G | A | 0.081 | 0.016 | 2.53E-07 | 25.864 |
| family | Rikenellaceae | rs4575409 | C | T | 0.081 | 0.016 | 2.53E-07 | 25.864 |
| family | Rikenellaceae | rs1575383 | A | G | 0.081 | 0.016 | 2.53E-07 | 25.864 |
| family | Rikenellaceae | rs9603200 | G | A | 0.081 | 0.016 | 2.53E-07 | 25.864 |
| family | Rikenellaceae | rs1575381 | A | T | 0.081 | 0.016 | 2.53E-07 | 25.864 |
| family | Prevotellaceae | rs10779530 | G | A | -0.075 | 0.015 | 6.23E-07 | 25.858 |
| family | Rikenellaceae | rs1575382 | G | A | 0.081 | 0.016 | 2.61E-07 | 25.833 |
| family | ClostridialesvadinBB60group | rs7218114 | G | A | 0.136 | 0.027 | 9.08E-07 | 25.826 |
| family | Bifidobacteriaceae | rs6716987 | C | A | 0.064 | 0.013 | 3.84E-07 | 25.792 |
| family | Rikenellaceae | rs2323487 | A | T | 0.086 | 0.017 | 2.92E-07 | 25.789 |
| family | Streptococcaceae | rs11110208 | T | C | -0.112 | 0.022 | 6.59E-07 | 25.788 |
| family | Alcaligenaceae | rs7326330 | T | C | -0.056 | 0.011 | 3.54E-07 | 25.786 |
| family | Rikenellaceae | rs9805695 | T | C | 0.081 | 0.016 | 2.61E-07 | 25.780 |
| family | Rikenellaceae | rs7989845 | A | G | 0.081 | 0.016 | 2.61E-07 | 25.780 |
| family | Prevotellaceae | rs9953173 | A | G | -0.088 | 0.017 | 3.76E-07 | 25.764 |
| family | BacteroidalesS24 | rs17043785 | C | T | -0.176 | 0.035 | 5.12E-07 | 25.761 |
| family | Rikenellaceae | rs7988812 | C | A | 0.081 | 0.016 | 2.61E-07 | 25.751 |
| family | Rikenellaceae | rs7335244 | A | G | 0.081 | 0.016 | 2.59E-07 | 25.733 |
| family | Streptococcaceae | rs11110125 | A | G | -0.101 | 0.020 | 5.76E-07 | 25.729 |
| family | Rikenellaceae | rs9594209 | T | C | 0.081 | 0.016 | 2.62E-07 | 25.727 |
| family | Rikenellaceae | rs4421867 | T | C | 0.081 | 0.016 | 2.75E-07 | 25.714 |
| family | Rikenellaceae | rs7994056 | T | C | 0.081 | 0.016 | 2.69E-07 | 25.696 |
| family | Prevotellaceae | rs7226722 | C | T | -0.087 | 0.017 | 3.84E-07 | 25.694 |
| family | Streptococcaceae | rs11110091 | G | A | -0.102 | 0.020 | 5.93E-07 | 25.684 |
| family | Streptococcaceae | rs7308069 | C | A | -0.101 | 0.020 | 6.20E-07 | 25.683 |
| family | Rikenellaceae | rs9594207 | A | G | 0.081 | 0.016 | 2.69E-07 | 25.679 |
| family | Rikenellaceae | rs1575380 | C | T | 0.081 | 0.016 | 2.76E-07 | 25.678 |
| family | Peptostreptococcaceae | rs41526049 | C | T | 0.072 | 0.014 | 3.55E-07 | 25.669 |
| family | Streptococcaceae | rs11110164 | C | G | -0.102 | 0.020 | 6.37E-07 | 25.658 |
| family | Peptostreptococcaceae | rs61841471 | G | T | 0.072 | 0.014 | 3.61E-07 | 25.648 |
| family | ClostridialesvadinBB60group | rs8066769 | G | A | 0.135 | 0.027 | 1.01E-06 | 25.647 |
| family | Rikenellaceae | rs17055728 | A | G | 0.081 | 0.016 | 2.85E-07 | 25.646 |
| family | Porphyromonadaceae | rs60884758 | T | C | -0.071 | 0.014 | 2.66E-07 | 25.636 |
| family | Rikenellaceae | rs4435115 | G | A | 0.081 | 0.016 | 2.84E-07 | 25.630 |
| family | Oxalobacteraceae | rs6445048 | A | G | 0.118 | 0.023 | 4.24E-07 | 25.625 |
| family | Rikenellaceae | rs9594208 | T | C | 0.081 | 0.016 | 2.85E-07 | 25.613 |
| family | Peptostreptococcaceae | rs4692811 | T | C | 0.064 | 0.013 | 4.21E-07 | 25.595 |
| family | Rikenellaceae | rs1590303 | C | A | 0.081 | 0.016 | 2.84E-07 | 25.590 |
| family | Pasteurellaceae | rs7771624 | C | A | -0.086 | 0.017 | 4.94E-07 | 25.576 |
| family | Rikenellaceae | rs9603201 | G | C | 0.081 | 0.016 | 2.92E-07 | 25.575 |
| family | Rikenellaceae | rs9603203 | G | A | 0.081 | 0.016 | 2.87E-07 | 25.566 |
| family | Rikenellaceae | rs9603202 | A | G | 0.081 | 0.016 | 2.94E-07 | 25.561 |
| family | BacteroidalesS24 | rs6742712 | T | C | -0.174 | 0.034 | 5.79E-07 | 25.552 |
| family | Streptococcaceae | rs12371224 | A | T | -0.101 | 0.020 | 6.50E-07 | 25.546 |
| family | Alcaligenaceae | rs9537883 | G | T | -0.056 | 0.011 | 4.06E-07 | 25.540 |
| family | Streptococcaceae | rs2952251 | G | A | -0.064 | 0.013 | 3.72E-07 | 25.530 |
| family | Streptococcaceae | rs60861038 | C | T | -0.101 | 0.020 | 7.48E-07 | 25.518 |
| family | Bifidobacteriaceae | rs56036623 | T | C | 0.070 | 0.014 | 3.06E-07 | 25.512 |
| family | Streptococcaceae | rs11110206 | T | C | -0.111 | 0.022 | 7.65E-07 | 25.506 |
| family | Prevotellaceae | rs6720263 | A | G | -0.060 | 0.012 | 4.59E-07 | 25.500 |
| family | Rikenellaceae | rs55963355 | C | T | 0.081 | 0.016 | 3.02E-07 | 25.495 |
| family | Peptostreptococcaceae | rs61841477 | C | A | 0.081 | 0.016 | 3.70E-07 | 25.472 |
| family | Rikenellaceae | rs55796046 | G | T | 0.081 | 0.016 | 3.11E-07 | 25.464 |
| family | Alcaligenaceae | rs1581853 | G | A | -0.057 | 0.011 | 4.03E-07 | 25.452 |
| family | Rikenellaceae | rs4514540 | C | T | 0.081 | 0.016 | 3.18E-07 | 25.441 |
| family | Rikenellaceae | rs4578521 | C | T | 0.081 | 0.016 | 3.18E-07 | 25.441 |
| family | FamilyXI | rs10759623 | T | C | -0.162 | 0.032 | 5.78E-07 | 25.425 |
| family | Methanobacteriaceae | rs7601415 | T | A | -0.119 | 0.024 | 6.11E-07 | 25.420 |
| family | Bifidobacteriaceae | rs6708336 | T | A | 0.060 | 0.012 | 3.72E-07 | 25.411 |
| family | Veillonellaceae | rs12738636 | G | A | -0.059 | 0.012 | 4.46E-07 | 25.400 |
| family | Lachnospiraceae | rs8060299 | T | G | -0.124 | 0.025 | 4.61E-07 | 25.383 |
| family | Rikenellaceae | rs9603205 | C | A | 0.080 | 0.016 | 3.19E-07 | 25.373 |
| family | Rikenellaceae | rs4436664 | G | C | 0.080 | 0.016 | 3.25E-07 | 25.372 |
| family | Bifidobacteriaceae | rs10496737 | A | G | 0.073 | 0.015 | 1.04E-06 | 25.356 |
| family | Rikenellaceae | rs5000542 | G | A | 0.080 | 0.016 | 3.28E-07 | 25.351 |
| family | Rikenellaceae | rs17055723 | C | T | 0.080 | 0.016 | 3.34E-07 | 25.342 |
| family | Rikenellaceae | rs1923718 | C | T | 0.080 | 0.016 | 3.12E-07 | 25.342 |
| family | Streptococcaceae | rs118038800 | C | T | -0.101 | 0.020 | 8.68E-07 | 25.339 |
| family | ClostridialesvadinBB60group | rs55682560 | T | C | -0.132 | 0.026 | 4.97E-07 | 25.330 |
| family | Bifidobacteriaceae | rs3814354 | G | A | 0.060 | 0.012 | 3.95E-07 | 25.328 |
| family | Streptococcaceae | rs76118421 | T | A | -0.102 | 0.020 | 7.01E-07 | 25.323 |
| family | Alcaligenaceae | rs1926544 | A | C | -0.056 | 0.011 | 4.53E-07 | 25.322 |
| family | Rikenellaceae | rs7334359 | C | T | 0.080 | 0.016 | 3.21E-07 | 25.309 |
| family | Rikenellaceae | rs1923717 | A | T | 0.080 | 0.016 | 3.19E-07 | 25.306 |
| family | Rikenellaceae | rs35078720 | C | G | 0.080 | 0.016 | 3.37E-07 | 25.301 |
| family | Rikenellaceae | rs35308611 | T | C | 0.080 | 0.016 | 3.30E-07 | 25.287 |
| family | BacteroidalesS24 | rs17043468 | T | C | -0.173 | 0.034 | 7.39E-07 | 25.284 |
| family | Bifidobacteriaceae | rs60963894 | T | G | 0.073 | 0.015 | 1.08E-06 | 25.276 |
| family | Bifidobacteriaceae | rs4988243 | T | C | 0.119 | 0.024 | 3.85E-07 | 25.271 |
| family | Rikenellaceae | rs1590305 | C | T | 0.080 | 0.016 | 3.37E-07 | 25.261 |
| family | Desulfovibrionaceae | rs6058181 | T | C | 0.083 | 0.017 | 2.70E-07 | 25.253 |
| family | Methanobacteriaceae | rs1869500 | A | C | -0.118 | 0.024 | 6.57E-07 | 25.249 |
| family | Streptococcaceae | rs7316887 | G | A | -0.101 | 0.020 | 7.64E-07 | 25.247 |
| family | Bacteroidaceae | rs6810046 | A | G | 0.053 | 0.011 | 5.56E-07 | 25.228 |
| family | Bifidobacteriaceae | rs1865452 | C | T | 0.073 | 0.015 | 1.12E-06 | 25.205 |
| family | Bifidobacteriaceae | rs62168897 | C | T | 0.060 | 0.012 | 4.15E-07 | 25.199 |
| family | Erysipelotrichaceae | rs62504370 | C | A | 0.063 | 0.013 | 4.80E-07 | 25.185 |
| family | Bacteroidaceae | rs11585893 | G | A | -0.074 | 0.015 | 1.80E-06 | 25.175 |
| family | Streptococcaceae | rs11110171 | G | A | -0.101 | 0.020 | 7.94E-07 | 25.170 |
| family | Rikenellaceae | rs6563550 | C | T | 0.086 | 0.017 | 2.81E-07 | 25.162 |
| family | Oxalobacteraceae | rs4605578 | A | G | 0.118 | 0.023 | 5.89E-07 | 25.162 |
| family | Bifidobacteriaceae | rs9677760 | A | T | 0.073 | 0.014 | 1.21E-06 | 25.154 |
| family | Rikenellaceae | rs34027519 | C | T | 0.080 | 0.016 | 3.70E-07 | 25.142 |
| family | Bifidobacteriaceae | rs7560535 | G | A | 0.093 | 0.018 | 9.58E-07 | 25.130 |
| family | BacteroidalesS24 | rs697394 | A | T | 0.085 | 0.017 | 8.04E-07 | 25.129 |
| family | Rikenellaceae | rs61946592 | T | C | 0.085 | 0.017 | 3.24E-07 | 25.121 |
| family | Bifidobacteriaceae | rs4954557 | C | T | 0.065 | 0.013 | 5.19E-07 | 25.121 |
| family | Defluviitaleaceae | rs2357084 | A | T | -0.109 | 0.022 | 1.42E-06 | 25.099 |
| family | Bifidobacteriaceae | rs2034261 | G | T | 0.060 | 0.012 | 4.26E-07 | 25.098 |
| family | Rikenellaceae | rs1923719 | T | C | 0.080 | 0.016 | 3.61E-07 | 25.070 |
| family | Rikenellaceae | rs9603204 | C | T | 0.080 | 0.016 | 3.61E-07 | 25.070 |
| family | Rikenellaceae | rs12787142 | C | G | -0.104 | 0.021 | 1.27E-06 | 25.068 |
| family | Peptostreptococcaceae | rs41289305 | T | C | 0.075 | 0.015 | 6.75E-07 | 25.068 |
| family | Rikenellaceae | rs7329262 | A | G | 0.080 | 0.016 | 3.66E-07 | 25.066 |
| family | Rikenellaceae | rs7140071 | T | C | 0.080 | 0.016 | 3.74E-07 | 25.059 |
| family | ClostridialesvadinBB60group | rs8067909 | G | A | 0.134 | 0.027 | 1.27E-06 | 25.051 |
| family | Rikenellaceae | rs7324216 | C | T | 0.085 | 0.017 | 3.70E-07 | 25.040 |
| family | Streptococcaceae | rs11110280 | G | A | -0.100 | 0.020 | 1.01E-06 | 25.028 |
| family | Desulfovibrionaceae | rs6060239 | T | C | 0.083 | 0.017 | 3.36E-07 | 25.026 |
| family | Peptostreptococcaceae | rs61841445 | G | C | 0.075 | 0.015 | 6.91E-07 | 24.995 |
| family | Peptostreptococcaceae | rs80159630 | T | C | 0.063 | 0.013 | 5.66E-07 | 24.993 |
| family | Prevotellaceae | rs3860225 | G | A | 0.084 | 0.017 | 5.50E-07 | 24.981 |
| family | ClostridialesvadinBB60group | rs6503942 | G | A | 0.134 | 0.027 | 1.30E-06 | 24.980 |
| family | Bifidobacteriaceae | rs78793148 | C | A | 0.099 | 0.020 | 1.03E-06 | 24.978 |
| family | Prevotellaceae | rs11102139 | T | C | 0.084 | 0.017 | 6.16E-07 | 24.969 |
| family | Bifidobacteriaceae | rs625118 | C | A | 0.087 | 0.017 | 6.58E-07 | 24.964 |
| family | Coriobacteriaceae | rs719099 | G | A | 0.078 | 0.016 | 5.43E-07 | 24.957 |
| family | Peptostreptococcaceae | rs61841447 | T | C | 0.075 | 0.015 | 7.14E-07 | 24.951 |
| family | Peptostreptococcaceae | rs17661170 | A | G | 0.063 | 0.013 | 5.95E-07 | 24.935 |
| family | ClostridialesvadinBB60group | rs9895182 | T | C | 0.131 | 0.026 | 1.75E-06 | 24.934 |
| family | Bifidobacteriaceae | rs10166142 | A | G | 0.059 | 0.012 | 4.86E-07 | 24.932 |
| family | Verrucomicrobiaceae | rs11729256 | C | T | 0.075 | 0.015 | 6.73E-07 | 24.928 |
| family | Streptococcaceae | rs4260895 | A | C | -0.063 | 0.013 | 4.50E-07 | 24.927 |
| family | Alcaligenaceae | rs12867878 | G | A | -0.057 | 0.011 | 5.06E-07 | 24.923 |
| family | Alcaligenaceae | rs7324740 | A | G | -0.057 | 0.011 | 5.32E-07 | 24.921 |
| family | Bifidobacteriaceae | rs6430601 | G | T | 0.085 | 0.017 | 2.46E-07 | 24.873 |
| family | BacteroidalesS24 | rs2356673 | A | G | -0.173 | 0.035 | 1.04E-06 | 24.870 |
| family | Bifidobacteriaceae | rs1469996 | A | G | 0.073 | 0.015 | 1.53E-06 | 24.863 |
| family | Streptococcaceae | rs11614784 | G | A | -0.100 | 0.020 | 1.09E-06 | 24.859 |
| family | Bifidobacteriaceae | rs35564151 | A | G | 0.060 | 0.012 | 4.51E-07 | 24.846 |
| family | Rikenellaceae | rs61946576 | T | C | 0.085 | 0.017 | 3.60E-07 | 24.842 |
| family | Desulfovibrionaceae | rs6060238 | G | A | 0.083 | 0.017 | 3.60E-07 | 24.825 |
| family | Streptococcaceae | rs76222580 | T | A | -0.100 | 0.020 | 1.10E-06 | 24.817 |
| family | FamilyXI | rs2155352 | G | A | -0.151 | 0.030 | 6.63E-07 | 24.795 |
| family | Enterobacteriaceae | rs78143293 | G | A | -0.085 | 0.017 | 1.20E-06 | 24.792 |
| family | Alcaligenaceae | rs9527747 | C | G | -0.055 | 0.011 | 5.96E-07 | 24.768 |
| family | Oxalobacteraceae | rs4287929 | G | A | 0.117 | 0.023 | 7.21E-07 | 24.766 |
| family | Alcaligenaceae | rs9537916 | G | A | -0.056 | 0.011 | 5.82E-07 | 24.762 |
| family | Peptostreptococcaceae | rs2644627 | G | C | -0.056 | 0.011 | 6.33E-07 | 24.761 |
| family | Bifidobacteriaceae | rs3814355 | C | T | 0.059 | 0.012 | 4.83E-07 | 24.742 |
| family | Rikenellaceae | rs67705352 | G | T | -0.055 | 0.011 | 6.58E-07 | 24.731 |
| family | Oxalobacteraceae | rs1569853 | C | T | -0.140 | 0.028 | 7.45E-07 | 24.714 |
| family | Alcaligenaceae | rs9537872 | A | G | -0.055 | 0.011 | 6.15E-07 | 24.701 |
| family | Victivallaceae | rs34296005 | A | T | 0.128 | 0.026 | 7.92E-07 | 24.679 |
| family | Desulfovibrionaceae | rs6060237 | A | G | 0.082 | 0.017 | 3.97E-07 | 24.670 |
| family | Bifidobacteriaceae | rs7595712 | C | T | 0.072 | 0.015 | 1.70E-06 | 24.663 |
| family | Bifidobacteriaceae | rs7592990 | A | G | 0.087 | 0.017 | 7.55E-07 | 24.662 |
| family | Bifidobacteriaceae | rs2117511 | T | C | 0.086 | 0.017 | 7.45E-07 | 24.661 |
| family | Bifidobacteriaceae | rs56271357 | C | T | 0.059 | 0.012 | 5.47E-07 | 24.643 |
| family | Oxalobacteraceae | rs4364195 | A | G | 0.116 | 0.023 | 7.67E-07 | 24.639 |
| family | ClostridialesvadinBB60group | rs56827745 | A | G | 0.133 | 0.027 | 1.74E-06 | 24.625 |
| family | BacteroidalesS24 | rs149498972 | G | A | -0.172 | 0.035 | 1.25E-06 | 24.599 |
| family | Defluviitaleaceae | rs4677103 | G | A | 0.098 | 0.020 | 9.42E-07 | 24.598 |
| family | Alcaligenaceae | rs1036449 | C | T | -0.055 | 0.011 | 6.51E-07 | 24.597 |
| family | Alcaligenaceae | rs2217928 | T | C | -0.055 | 0.011 | 6.58E-07 | 24.586 |
| family | Streptococcaceae | rs11524043 | G | A | -0.099 | 0.020 | 1.16E-06 | 24.579 |
| family | Bifidobacteriaceae | rs1469995 | G | A | 0.072 | 0.015 | 1.55E-06 | 24.574 |
| family | Bifidobacteriaceae | rs60453613 | G | C | 0.072 | 0.015 | 1.55E-06 | 24.573 |
| family | Bifidobacteriaceae | rs309124 | A | G | 0.076 | 0.015 | 9.62E-07 | 24.560 |
| family | Lactobacillaceae | rs921925 | C | A | 0.100 | 0.020 | 5.77E-07 | 24.555 |
| family | Alcaligenaceae | rs9537941 | A | C | -0.055 | 0.011 | 6.52E-07 | 24.548 |
| family | Bifidobacteriaceae | rs4954394 | A | G | 0.067 | 0.014 | 4.30E-07 | 24.546 |
| family | Victivallaceae | rs11764871 | T | G | 0.127 | 0.026 | 7.49E-07 | 24.542 |
| family | Rikenellaceae | rs9603195 | G | T | 0.079 | 0.016 | 5.21E-07 | 24.535 |
| family | Rikenellaceae | rs2224659 | A | G | 0.079 | 0.016 | 5.21E-07 | 24.535 |
| family | Lachnospiraceae | rs28456161 | G | A | -0.131 | 0.026 | 6.10E-07 | 24.527 |
| family | Prevotellaceae | rs9967134 | T | A | -0.086 | 0.017 | 6.70E-07 | 24.522 |
| family | Bifidobacteriaceae | rs12469551 | G | T | 0.072 | 0.014 | 1.80E-06 | 24.520 |
| family | Alcaligenaceae | rs1470456 | A | G | -0.055 | 0.011 | 6.67E-07 | 24.519 |
| family | Bifidobacteriaceae | rs11895609 | A | T | 0.083 | 0.017 | 2.89E-07 | 24.515 |
| family | Coriobacteriaceae | rs2458655 | C | A | 0.078 | 0.016 | 7.77E-07 | 24.461 |
| family | Victivallaceae | rs34702749 | A | T | -0.141 | 0.028 | 1.17E-06 | 24.450 |
| family | Peptococcaceae | rs35703006 | T | G | 0.081 | 0.016 | 4.95E-07 | 24.448 |
| family | Bifidobacteriaceae | rs3769023 | G | A | 0.059 | 0.012 | 6.29E-07 | 24.435 |
| family | ClostridialesvadinBB60group | rs8067650 | T | C | 0.130 | 0.026 | 2.37E-06 | 24.424 |
| family | Rikenellaceae | rs17055871 | T | A | 0.084 | 0.017 | 4.49E-07 | 24.423 |
| family | Alcaligenaceae | rs9537922 | T | C | -0.055 | 0.011 | 7.07E-07 | 24.420 |
| family | Bifidobacteriaceae | rs1964459 | C | G | 0.080 | 0.016 | 1.17E-06 | 24.419 |
| family | Alcaligenaceae | rs9569804 | G | A | -0.055 | 0.011 | 7.10E-07 | 24.410 |
| family | Streptococcaceae | rs7308947 | A | T | -0.098 | 0.020 | 1.15E-06 | 24.404 |
| family | Bacteroidaceae | rs66474973 | T | G | 0.081 | 0.016 | 6.81E-07 | 24.403 |
| family | Alcaligenaceae | rs9537900 | A | G | -0.055 | 0.011 | 7.38E-07 | 24.391 |
| family | Bifidobacteriaceae | rs10187990 | A | G | 0.059 | 0.012 | 5.95E-07 | 24.385 |
| family | Alcaligenaceae | rs12467854 | C | G | 0.066 | 0.013 | 7.63E-07 | 24.385 |
| family | Streptococcaceae | rs111326382 | A | G | -0.097 | 0.020 | 1.17E-06 | 24.384 |
| family | Rikenellaceae | rs7337526 | C | T | 0.084 | 0.017 | 4.72E-07 | 24.377 |
| family | Rikenellaceae | rs1322804 | T | G | 0.079 | 0.016 | 5.67E-07 | 24.369 |
| family | Alcaligenaceae | rs9537940 | C | A | -0.055 | 0.011 | 7.14E-07 | 24.367 |
| family | Veillonellaceae | rs12024294 | C | T | -0.058 | 0.012 | 6.02E-07 | 24.367 |
| family | BacteroidalesS24 | rs138088344 | G | A | -0.172 | 0.035 | 1.33E-06 | 24.357 |
| family | Bacteroidaceae | rs750423 | G | A | 0.052 | 0.011 | 8.89E-07 | 24.336 |
| family | Alcaligenaceae | rs9537904 | G | A | -0.055 | 0.011 | 7.46E-07 | 24.328 |
| family | Methanobacteriaceae | rs73457410 | G | A | 0.215 | 0.044 | 1.41E-06 | 24.316 |
| family | Oxalobacteraceae | rs10868238 | C | T | 0.099 | 0.020 | 8.33E-07 | 24.316 |
| family | Ruminococcaceae | rs17376049 | C | T | 0.085 | 0.017 | 7.30E-07 | 24.306 |
| family | Alcaligenaceae | rs1953283 | C | A | -0.055 | 0.011 | 7.66E-07 | 24.301 |
| family | Bifidobacteriaceae | rs6708957 | C | T | 0.072 | 0.015 | 1.76E-06 | 24.294 |
| family | Prevotellaceae | rs13005458 | G | A | -0.058 | 0.012 | 8.60E-07 | 24.266 |
| family | Victivallaceae | rs2546105 | T | A | 0.127 | 0.026 | 1.03E-06 | 24.262 |
| family | Peptostreptococcaceae | rs12377846 | A | C | -0.252 | 0.051 | 7.26E-07 | 24.260 |
| family | Prevotellaceae | rs12612711 | G | A | -0.058 | 0.012 | 8.75E-07 | 24.258 |
| family | Bifidobacteriaceae | rs1947112 | A | G | 0.059 | 0.012 | 6.85E-07 | 24.246 |
| family | Bifidobacteriaceae | rs6732236 | T | G | 0.072 | 0.015 | 1.82E-06 | 24.238 |
| family | Alcaligenaceae | rs9537921 | A | G | -0.054 | 0.011 | 7.75E-07 | 24.235 |
| family | Alcaligenaceae | rs1409526 | A | G | -0.054 | 0.011 | 7.78E-07 | 24.234 |
| family | Streptococcaceae | rs11110266 | C | T | -0.091 | 0.018 | 1.72E-06 | 24.232 |
| family | Bifidobacteriaceae | rs638382 | C | T | -0.060 | 0.012 | 5.56E-07 | 24.230 |
| family | Streptococcaceae | rs138049946 | T | C | -0.098 | 0.020 | 1.37E-06 | 24.230 |
| family | Bifidobacteriaceae | rs4567981 | A | T | 0.058 | 0.012 | 8.88E-07 | 24.224 |
| family | Alcaligenaceae | rs7997285 | C | T | -0.054 | 0.011 | 7.97E-07 | 24.219 |
| family | Alcaligenaceae | rs7996696 | G | A | -0.054 | 0.011 | 7.95E-07 | 24.217 |
| family | Porphyromonadaceae | rs10217465 | G | A | -0.071 | 0.015 | 5.48E-07 | 24.198 |
| family | Alcaligenaceae | rs9527748 | G | A | -0.054 | 0.011 | 7.96E-07 | 24.198 |
| family | Bifidobacteriaceae | rs72847650 | C | A | -0.061 | 0.012 | 5.13E-07 | 24.193 |
| family | Alcaligenaceae | rs9537908 | T | C | -0.054 | 0.011 | 8.02E-07 | 24.190 |
| family | Streptococcaceae | rs2975635 | C | T | -0.061 | 0.012 | 8.18E-07 | 24.188 |
| family | Alcaligenaceae | rs9563562 | T | C | -0.054 | 0.011 | 8.07E-07 | 24.183 |
| family | Alcaligenaceae | rs9537865 | C | T | -0.054 | 0.011 | 8.31E-07 | 24.177 |
| family | Alcaligenaceae | rs9563572 | C | T | -0.054 | 0.011 | 8.10E-07 | 24.167 |
| family | Streptococcaceae | rs11610034 | T | C | -0.098 | 0.020 | 1.53E-06 | 24.158 |
| family | Porphyromonadaceae | rs10965294 | C | G | -0.070 | 0.014 | 5.34E-07 | 24.156 |
| family | Peptostreptococcaceae | rs34683673 | G | C | 0.074 | 0.015 | 9.70E-07 | 24.148 |
| family | Alcaligenaceae | rs10444649 | C | T | -0.054 | 0.011 | 8.26E-07 | 24.134 |
| family | Rikenellaceae | rs73180681 | C | T | 0.084 | 0.017 | 4.75E-07 | 24.131 |
| family | Alcaligenaceae | rs9569779 | A | G | -0.054 | 0.011 | 8.53E-07 | 24.125 |
| family | Streptococcaceae | rs7306985 | C | T | -0.098 | 0.020 | 1.45E-06 | 24.123 |
| family | Streptococcaceae | rs2952176 | A | G | -0.061 | 0.012 | 8.79E-07 | 24.117 |
| family | BacteroidalesS24 | rs696267 | T | C | 0.083 | 0.017 | 1.42E-06 | 24.112 |
| family | Rikenellaceae | rs3764077 | A | G | 0.084 | 0.017 | 5.28E-07 | 24.109 |
| family | Verrucomicrobiaceae | rs113634144 | G | T | 0.074 | 0.015 | 1.11E-06 | 24.099 |
| family | Erysipelotrichaceae | rs2300774 | A | G | 0.052 | 0.011 | 8.95E-07 | 24.094 |
| family | Alcaligenaceae | rs1036450 | T | A | -0.054 | 0.011 | 8.44E-07 | 24.085 |
| family | Streptococcaceae | rs10028567 | T | C | -0.093 | 0.019 | 3.72E-06 | 24.079 |
| family | Alcaligenaceae | rs1036451 | T | C | -0.054 | 0.011 | 8.48E-07 | 24.078 |
| family | Alcaligenaceae | rs9563571 | T | C | -0.054 | 0.011 | 8.40E-07 | 24.075 |
| family | Bifidobacteriaceae | rs147616090 | C | T | 0.071 | 0.015 | 2.04E-06 | 24.068 |
| family | Lactobacillaceae | rs328312 | A | T | 0.083 | 0.017 | 8.69E-07 | 24.068 |
| family | Bifidobacteriaceae | rs2289963 | G | A | 0.099 | 0.020 | 1.70E-06 | 24.061 |
| family | Oxalobacteraceae | rs6000536 | T | C | -0.118 | 0.024 | 7.39E-07 | 24.058 |
| family | Christensenellaceae | rs62573205 | A | G | -0.065 | 0.013 | 1.49E-06 | 24.057 |
| family | Pasteurellaceae | rs4132989 | T | C | -0.084 | 0.017 | 1.18E-06 | 24.055 |
| family | Bifidobacteriaceae | rs4953938 | G | A | 0.058 | 0.012 | 6.84E-07 | 24.054 |
| family | Peptostreptococcaceae | rs2883972 | A | G | 0.073 | 0.015 | 9.80E-07 | 24.051 |
| family | Ruminococcaceae | rs1572312 | G | T | 0.084 | 0.017 | 8.10E-07 | 24.046 |
| family | Bifidobacteriaceae | rs309122 | A | G | 0.075 | 0.015 | 1.25E-06 | 24.038 |
| family | BacteroidalesS24 | rs72791497 | G | T | -0.173 | 0.035 | 2.42E-06 | 24.037 |
| family | Peptococcaceae | rs12634826 | G | T | -0.074 | 0.015 | 1.01E-06 | 24.031 |
| family | Peptostreptococcaceae | rs117020988 | T | C | 0.182 | 0.037 | 1.03E-06 | 24.028 |
| family | Peptococcaceae | rs9644052 | T | C | 0.082 | 0.017 | 7.34E-07 | 24.028 |
| family | Defluviitaleaceae | rs55658617 | C | T | 0.177 | 0.036 | 1.41E-06 | 24.013 |
| family | Peptococcaceae | rs4990837 | G | A | 0.091 | 0.019 | 1.74E-06 | 23.990 |
| family | Alcaligenaceae | rs7322915 | A | T | -0.056 | 0.011 | 8.46E-07 | 23.985 |
| family | ClostridialesvadinBB60group | rs9979874 | C | G | -0.074 | 0.015 | 1.05E-06 | 23.975 |
| family | Alcaligenaceae | rs9527759 | T | C | -0.054 | 0.011 | 8.83E-07 | 23.962 |
| family | Alcaligenaceae | rs7988274 | A | C | -0.054 | 0.011 | 8.99E-07 | 23.958 |
| family | Alcaligenaceae | rs9537907 | T | A | -0.054 | 0.011 | 9.07E-07 | 23.952 |
| family | Erysipelotrichaceae | rs2300775 | C | T | 0.052 | 0.011 | 9.78E-07 | 23.919 |
| family | Victivallaceae | rs6951588 | T | C | 0.125 | 0.026 | 1.11E-06 | 23.919 |
| family | Alcaligenaceae | rs9563573 | C | T | -0.054 | 0.011 | 9.04E-07 | 23.913 |
| family | Alcaligenaceae | rs986833 | A | G | -0.054 | 0.011 | 9.31E-07 | 23.905 |
| family | Streptococcaceae | rs4968759 | G | A | -0.054 | 0.011 | 8.92E-07 | 23.905 |
| family | Prevotellaceae | rs74442976 | G | A | 0.091 | 0.019 | 1.72E-06 | 23.899 |
| family | Prevotellaceae | rs13026139 | G | A | -0.058 | 0.012 | 1.06E-06 | 23.890 |
| family | ClostridialesvadinBB60group | rs6503943 | G | T | 0.128 | 0.026 | 3.10E-06 | 23.890 |
| family | Alcaligenaceae | rs1536384 | T | C | -0.054 | 0.011 | 9.40E-07 | 23.884 |
| family | Alcaligenaceae | rs11679161 | C | T | 0.065 | 0.013 | 8.91E-07 | 23.871 |
| family | Rikenellaceae | rs5010916 | G | T | 0.078 | 0.016 | 7.24E-07 | 23.865 |
| family | ClostridialesvadinBB60group | rs8082388 | T | C | 0.130 | 0.027 | 2.42E-06 | 23.860 |
| family | ClostridialesvadinBB60group | rs2302201 | A | T | 0.128 | 0.026 | 3.17E-06 | 23.859 |
| family | Streptococcaceae | rs17708276 | G | A | -0.083 | 0.017 | 9.74E-07 | 23.849 |
| family | Ruminococcaceae | rs17304036 | G | A | 0.085 | 0.017 | 9.12E-07 | 23.846 |
| family | Peptococcaceae | rs2720807 | G | C | 0.093 | 0.019 | 1.77E-06 | 23.839 |
| family | Alcaligenaceae | rs7990795 | G | T | -0.054 | 0.011 | 9.68E-07 | 23.833 |
| family | Streptococcaceae | rs57497665 | C | T | -0.091 | 0.019 | 2.08E-06 | 23.829 |
| family | ClostridialesvadinBB60group | rs16943991 | G | A | 0.130 | 0.027 | 2.33E-06 | 23.827 |
| family | Streptococcaceae | rs74659220 | A | C | -0.089 | 0.018 | 1.14E-06 | 23.824 |
| family | Victivallaceae | rs12793955 | G | T | -0.141 | 0.029 | 1.44E-06 | 23.822 |
| family | Alcaligenaceae | rs9537948 | G | A | -0.056 | 0.011 | 9.71E-07 | 23.821 |
| family | Alcaligenaceae | rs7983206 | T | C | -0.054 | 0.011 | 9.79E-07 | 23.817 |
| family | ClostridialesvadinBB60group | rs9900396 | C | T | 0.130 | 0.027 | 2.29E-06 | 23.800 |
| family | Peptococcaceae | rs3811732 | G | A | -0.072 | 0.015 | 1.26E-06 | 23.781 |
| family | Coriobacteriaceae | rs7078773 | C | T | 0.076 | 0.016 | 1.12E-06 | 23.780 |
| family | Alcaligenaceae | rs9569799 | A | G | -0.054 | 0.011 | 9.88E-07 | 23.779 |
| family | Streptococcaceae | rs241077 | A | T | -0.054 | 0.011 | 1.19E-06 | 23.777 |
| family | BacteroidalesS24 | rs689695 | A | C | 0.081 | 0.017 | 1.28E-06 | 23.776 |
| family | Bifidobacteriaceae | rs76671854 | G | C | -0.089 | 0.018 | 1.04E-06 | 23.765 |
| family | ClostridialesvadinBB60group | rs10515176 | T | C | 0.128 | 0.026 | 3.02E-06 | 23.764 |
| family | Bifidobacteriaceae | rs4957061 | C | T | 0.057 | 0.012 | 1.15E-06 | 23.761 |
| family | Bifidobacteriaceae | rs77985886 | C | T | 0.098 | 0.020 | 2.02E-06 | 23.758 |
| family | Desulfovibrionaceae | rs6060240 | C | A | 0.082 | 0.017 | 7.33E-07 | 23.754 |
| family | Erysipelotrichaceae | rs7234058 | C | T | -0.095 | 0.019 | 9.12E-07 | 23.744 |
| family | Bifidobacteriaceae | rs60902646 | T | C | 0.095 | 0.020 | 2.47E-06 | 23.740 |
| family | Veillonellaceae | rs66716149 | T | C | -0.058 | 0.012 | 8.34E-07 | 23.735 |
| family | Streptococcaceae | rs2975662 | G | C | -0.060 | 0.012 | 1.02E-06 | 23.732 |
| family | Erysipelotrichaceae | rs9858727 | T | A | 0.052 | 0.011 | 1.08E-06 | 23.728 |
| family | Erysipelotrichaceae | rs2300780 | C | T | 0.052 | 0.011 | 1.10E-06 | 23.722 |
| family | Oxalobacteraceae | rs73733937 | A | G | -0.137 | 0.028 | 1.30E-06 | 23.708 |
| family | Alcaligenaceae | rs67737557 | G | C | 0.074 | 0.015 | 1.04E-06 | 23.700 |
| family | Oxalobacteraceae | rs73733936 | T | C | -0.137 | 0.028 | 1.32E-06 | 23.688 |
| family | Peptostreptococcaceae | rs483392 | G | A | -0.055 | 0.011 | 1.12E-06 | 23.683 |
| family | Methanobacteriaceae | rs62241835 | T | G | -0.203 | 0.042 | 1.63E-06 | 23.683 |
| family | Streptococcaceae | rs76734283 | G | C | -0.097 | 0.020 | 1.76E-06 | 23.679 |
| family | Alcaligenaceae | rs7333865 | C | T | -0.055 | 0.011 | 9.44E-07 | 23.671 |
| family | Alcaligenaceae | rs9537913 | A | G | -0.054 | 0.011 | 1.05E-06 | 23.655 |
| family | Veillonellaceae | rs79535861 | C | A | 0.101 | 0.021 | 1.58E-06 | 23.646 |
| family | Alcaligenaceae | rs7986644 | A | G | -0.054 | 0.011 | 1.07E-06 | 23.626 |
| family | Streptococcaceae | rs10026175 | A | G | -0.092 | 0.019 | 4.59E-06 | 23.621 |
| family | Alcaligenaceae | rs17000015 | T | A | -0.061 | 0.013 | 1.13E-06 | 23.619 |
| family | Streptococcaceae | rs10025825 | A | G | -0.093 | 0.019 | 4.60E-06 | 23.614 |
| family | Streptococcaceae | rs11110259 | C | A | -0.097 | 0.020 | 1.99E-06 | 23.613 |
| family | FamilyXIII | rs6501525 | G | A | 0.056 | 0.012 | 1.24E-06 | 23.603 |
| family | Victivallaceae | rs62481809 | C | G | 0.124 | 0.026 | 1.19E-06 | 23.601 |
| family | Erysipelotrichaceae | rs79996147 | A | T | -0.094 | 0.019 | 9.72E-07 | 23.597 |
| family | Alcaligenaceae | rs980610 | A | G | -0.054 | 0.011 | 1.10E-06 | 23.596 |
| family | Erysipelotrichaceae | rs2300777 | C | A | 0.052 | 0.011 | 1.18E-06 | 23.592 |
| family | Alcaligenaceae | rs1887128 | A | G | -0.054 | 0.011 | 1.08E-06 | 23.584 |
| family | Ruminococcaceae | rs56199908 | C | T | -0.199 | 0.041 | 1.66E-06 | 23.584 |
| family | Rhodospirillaceae | rs3754624 | T | C | 0.097 | 0.020 | 1.71E-06 | 23.575 |
| family | Peptostreptococcaceae | rs9573934 | T | C | -0.069 | 0.014 | 1.73E-06 | 23.574 |
| family | Streptococcaceae | rs2009619 | G | A | -0.062 | 0.013 | 1.07E-06 | 23.565 |
| family | Bifidobacteriaceae | rs10174462 | C | G | 0.071 | 0.015 | 3.08E-06 | 23.565 |
| family | Pasteurellaceae | rs76022354 | T | C | 0.243 | 0.050 | 1.83E-06 | 23.560 |
| family | Peptostreptococcaceae | rs9573937 | G | A | -0.069 | 0.014 | 1.71E-06 | 23.559 |
| family | Desulfovibrionaceae | rs16851319 | C | G | -0.073 | 0.015 | 2.40E-06 | 23.556 |
| family | Porphyromonadaceae | rs10965204 | G | A | -0.064 | 0.013 | 1.04E-06 | 23.552 |
| family | Bifidobacteriaceae | rs578384 | C | T | -0.059 | 0.012 | 7.43E-07 | 23.541 |
| family | Lachnospiraceae | rs35524804 | C | T | -0.061 | 0.013 | 2.45E-06 | 23.537 |
| family | Coriobacteriaceae | rs56374305 | C | G | 0.100 | 0.021 | 1.17E-06 | 23.530 |
| family | Victivallaceae | rs61702987 | C | T | 0.146 | 0.030 | 3.08E-06 | 23.522 |
| family | Alcaligenaceae | rs9537910 | A | G | -0.054 | 0.011 | 1.12E-06 | 23.520 |
| family | Streptococcaceae | rs55657347 | T | G | -0.092 | 0.019 | 4.70E-06 | 23.520 |
| family | Rikenellaceae | rs4264350 | C | T | -0.053 | 0.011 | 1.35E-06 | 23.514 |
| family | Lactobacillaceae | rs6092149 | T | A | -0.083 | 0.017 | 1.38E-06 | 23.510 |
| family | FamilyXI | rs11021253 | G | C | -0.146 | 0.030 | 1.25E-06 | 23.503 |
| family | Streptococcaceae | rs11110294 | A | G | -0.097 | 0.020 | 2.15E-06 | 23.503 |
| family | Peptococcaceae | rs12992764 | G | T | 0.068 | 0.014 | 1.46E-06 | 23.502 |
| family | Bifidobacteriaceae | rs4954575 | G | A | 0.062 | 0.013 | 9.86E-07 | 23.488 |
| family | Bifidobacteriaceae | rs1374289 | C | T | 0.058 | 0.012 | 9.62E-07 | 23.485 |
| family | Methanobacteriaceae | rs6776814 | C | T | -0.200 | 0.041 | 1.63E-06 | 23.483 |
| family | Alcaligenaceae | rs9563570 | G | T | -0.054 | 0.011 | 1.15E-06 | 23.473 |
| family | Bifidobacteriaceae | rs16832440 | G | A | 0.079 | 0.016 | 1.90E-06 | 23.467 |
| family | Alcaligenaceae | rs4033856 | T | C | 0.082 | 0.017 | 1.03E-06 | 23.466 |
| family | Prevotellaceae | rs2206482 | G | T | -0.057 | 0.012 | 1.30E-06 | 23.464 |
| family | Peptostreptococcaceae | rs117680446 | G | A | 0.183 | 0.038 | 1.39E-06 | 23.458 |
| family | Alcaligenaceae | rs11683769 | A | T | 0.064 | 0.013 | 1.10E-06 | 23.436 |
| family | Pasteurellaceae | rs78909003 | C | T | -0.241 | 0.050 | 2.05E-06 | 23.415 |
| family | Streptococcaceae | rs2197262 | T | A | -0.054 | 0.011 | 1.40E-06 | 23.411 |
| family | Porphyromonadaceae | rs17065783 | G | A | -0.059 | 0.012 | 1.79E-06 | 23.403 |
| family | Oxalobacteraceae | rs11140800 | A | C | 0.097 | 0.020 | 1.35E-06 | 23.393 |
| family | Alcaligenaceae | rs1887129 | C | T | -0.054 | 0.011 | 1.19E-06 | 23.381 |
| family | ClostridialesvadinBB60group | rs7725895 | G | A | -0.116 | 0.024 | 3.94E-06 | 23.380 |
| family | Streptococcaceae | rs72739637 | G | A | 0.093 | 0.019 | 1.82E-06 | 23.355 |
| family | Methanobacteriaceae | rs894996 | A | C | 0.217 | 0.045 | 1.88E-06 | 23.349 |
| family | Porphyromonadaceae | rs2093835 | T | G | -0.068 | 0.014 | 7.71E-07 | 23.349 |
| family | Victivallaceae | rs2944282 | C | T | -0.124 | 0.026 | 1.57E-06 | 23.349 |
| family | Alcaligenaceae | rs9527764 | T | A | -0.055 | 0.011 | 1.20E-06 | 23.340 |
| family | Bifidobacteriaceae | rs6430585 | C | A | 0.070 | 0.014 | 3.17E-06 | 23.337 |
| family | Alcaligenaceae | rs11618348 | A | C | -0.053 | 0.011 | 1.25E-06 | 23.330 |
| family | Lachnospiraceae | . | T | G | -0.052 | 0.011 | 1.43E-06 | 23.323 |
| family | Porphyromonadaceae | rs10811676 | C | T | -0.069 | 0.014 | 7.46E-07 | 23.310 |
| family | Erysipelotrichaceae | rs2300778 | G | A | 0.052 | 0.011 | 1.37E-06 | 23.298 |
| family | Bifidobacteriaceae | rs309123 | C | G | 0.075 | 0.016 | 1.64E-06 | 23.297 |
| family | Oxalobacteraceae | rs73733942 | C | T | -0.136 | 0.028 | 1.54E-06 | 23.295 |
| family | Oxalobacteraceae | rs76405250 | T | C | -0.136 | 0.028 | 1.62E-06 | 23.284 |
| family | Bifidobacteriaceae | rs7604460 | T | C | 0.079 | 0.016 | 2.38E-06 | 23.274 |
| family | Bacteroidaceae | rs195028 | A | G | 0.080 | 0.017 | 1.33E-06 | 23.274 |
| family | Bifidobacteriaceae | rs6733130 | A | C | 0.058 | 0.012 | 9.71E-07 | 23.265 |
| family | Streptococcaceae | rs11110265 | G | A | -0.084 | 0.017 | 1.78E-06 | 23.256 |
| family | Alcaligenaceae | rs9537912 | A | T | -0.053 | 0.011 | 1.29E-06 | 23.248 |
| family | ClostridialesvadinBB60group | rs74692182 | T | A | 0.107 | 0.022 | 2.05E-06 | 23.243 |
| family | Streptococcaceae | rs1518775 | G | T | -0.053 | 0.011 | 1.32E-06 | 23.241 |
| family | Methanobacteriaceae | rs9942108 | G | A | -0.203 | 0.042 | 1.85E-06 | 23.240 |
| family | Bifidobacteriaceae | rs12471781 | G | A | 0.079 | 0.016 | 2.00E-06 | 23.237 |
| family | Methanobacteriaceae | rs73031978 | G | C | -0.203 | 0.042 | 1.90E-06 | 23.225 |
| family | Alcaligenaceae | rs7139988 | G | T | -0.055 | 0.011 | 1.23E-06 | 23.216 |
| family | Victivallaceae | rs6545794 | G | A | -0.198 | 0.041 | 5.97E-07 | 23.215 |
| family | Porphyromonadaceae | rs1125465 | C | T | -0.059 | 0.012 | 1.93E-06 | 23.211 |
| family | Oxalobacteraceae | rs10465181 | T | C | 0.096 | 0.020 | 1.51E-06 | 23.206 |
| family | Rikenellaceae | rs34733323 | G | A | -0.100 | 0.021 | 2.71E-06 | 23.205 |
| family | Peptostreptococcaceae | rs9573936 | G | C | -0.068 | 0.014 | 2.02E-06 | 23.201 |
| family | Bifidobacteriaceae | rs309152 | T | C | 0.076 | 0.016 | 1.48E-06 | 23.199 |
| family | Streptococcaceae | rs2440146 | T | C | -0.053 | 0.011 | 1.58E-06 | 23.182 |
| family | Streptococcaceae | rs7970304 | T | G | -0.087 | 0.018 | 1.44E-06 | 23.179 |
| family | Victivallaceae | rs1603450 | C | T | -0.144 | 0.030 | 2.28E-06 | 23.178 |
| family | Victivallaceae | rs253710 | C | T | 0.124 | 0.026 | 2.11E-06 | 23.177 |
| family | Bifidobacteriaceae | rs309133 | A | C | 0.075 | 0.016 | 1.65E-06 | 23.174 |
| family | Victivallaceae | rs11770843 | T | C | 0.124 | 0.026 | 1.06E-06 | 23.164 |
| family | Alcaligenaceae | rs7323027 | G | A | -0.055 | 0.011 | 1.27E-06 | 23.158 |
| family | Peptococcaceae | rs7837830 | A | G | 0.089 | 0.019 | 2.70E-06 | 23.153 |
| family | ClostridialesvadinBB60group | rs8082356 | G | T | 0.131 | 0.027 | 3.42E-06 | 23.151 |
| family | Christensenellaceae | rs4076564 | A | G | -0.189 | 0.039 | 8.06E-06 | 23.143 |
| family | Bifidobacteriaceae | rs4954571 | G | A | 0.065 | 0.014 | 9.01E-07 | 23.140 |
| family | Methanobacteriaceae | rs76029318 | C | T | 0.215 | 0.045 | 1.58E-06 | 23.140 |
| family | Alcaligenaceae | rs9569803 | A | G | -0.053 | 0.011 | 1.37E-06 | 23.137 |
| family | Methanobacteriaceae | rs62240385 | T | G | -0.201 | 0.042 | 1.77E-06 | 23.135 |
| family | Streptococcaceae | rs11110252 | G | A | -0.096 | 0.020 | 2.64E-06 | 23.132 |
| family | Pasteurellaceae | rs10840326 | G | C | -0.072 | 0.015 | 1.44E-06 | 23.126 |
| family | Erysipelotrichaceae | rs35161940 | C | T | -0.081 | 0.017 | 1.85E-06 | 23.118 |
| family | Alcaligenaceae | rs9537930 | A | G | -0.053 | 0.011 | 1.42E-06 | 23.118 |
| family | Bifidobacteriaceae | rs1374287 | C | T | 0.057 | 0.012 | 1.06E-06 | 23.114 |
| family | Methanobacteriaceae | rs12711690 | G | A | -0.113 | 0.024 | 1.97E-06 | 23.103 |
| family | Oxalobacteraceae | rs73733941 | C | T | -0.135 | 0.028 | 1.72E-06 | 23.093 |
| family | Peptostreptococcaceae | rs73235371 | C | T | -0.068 | 0.014 | 1.86E-06 | 23.089 |
| family | Bacteroidaceae | rs28757219 | A | T | 0.082 | 0.017 | 1.29E-06 | 23.089 |
| family | BacteroidalesS24 | rs965441 | C | A | -0.159 | 0.033 | 5.09E-06 | 23.084 |
| family | Erysipelotrichaceae | rs2284890 | C | T | 0.051 | 0.011 | 1.54E-06 | 23.077 |
| family | Bifidobacteriaceae | rs7575241 | C | A | 0.071 | 0.015 | 3.17E-06 | 23.075 |
| family | ClostridialesvadinBB60group | rs12520828 | G | A | -0.115 | 0.024 | 4.41E-06 | 23.075 |
| family | Porphyromonadaceae | rs3111851 | G | C | 0.058 | 0.012 | 2.19E-06 | 23.071 |
| family | Veillonellaceae | rs111810795 | T | C | -0.087 | 0.018 | 1.73E-06 | 23.059 |
| family | Bifidobacteriaceae | rs112407062 | C | T | 0.078 | 0.016 | 2.02E-06 | 23.056 |
| family | Bifidobacteriaceae | rs2322251 | C | T | 0.057 | 0.012 | 1.24E-06 | 23.047 |
| family | Defluviitaleaceae | rs1582238 | C | T | 0.080 | 0.017 | 1.69E-06 | 23.042 |
| family | Actinomycetaceae | rs35011108 | G | A | 0.242 | 0.050 | 1.83E-06 | 23.041 |
| family | Streptococcaceae | rs9895557 | C | T | -0.053 | 0.011 | 1.58E-06 | 23.037 |
| family | Rikenellaceae | rs6837275 | G | A | 0.057 | 0.012 | 1.45E-06 | 23.022 |
| family | Bifidobacteriaceae | rs75667274 | C | T | 0.078 | 0.016 | 2.13E-06 | 23.019 |
| family | Erysipelotrichaceae | rs2284889 | A | G | 0.051 | 0.011 | 1.60E-06 | 23.005 |
| family | Streptococcaceae | rs2952168 | G | C | -0.061 | 0.013 | 1.50E-06 | 23.000 |
| family | ClostridialesvadinBB60group | rs8070466 | C | T | 0.128 | 0.027 | 3.71E-06 | 22.992 |
| family | Bifidobacteriaceae | rs1374288 | C | T | 0.057 | 0.012 | 1.14E-06 | 22.988 |
| family | ClostridialesvadinBB60group | rs6588624 | A | G | -0.066 | 0.014 | 1.79E-06 | 22.985 |
| family | Bifidobacteriaceae | rs7568884 | C | T | 0.061 | 0.013 | 1.26E-06 | 22.985 |
| family | Alcaligenaceae | rs56050831 | T | C | 0.073 | 0.015 | 1.48E-06 | 22.983 |
| family | Erysipelotrichaceae | rs2239641 | C | T | 0.051 | 0.011 | 1.62E-06 | 22.979 |
| family | Methanobacteriaceae | rs62241834 | G | T | -0.201 | 0.042 | 2.44E-06 | 22.975 |
| family | Streptococcaceae | rs10028558 | T | C | -0.092 | 0.019 | 6.04E-06 | 22.973 |
| family | Streptococcaceae | rs395407 | C | G | -0.083 | 0.017 | 1.33E-06 | 22.967 |
| family | Erysipelotrichaceae | rs79329601 | C | T | -0.093 | 0.019 | 1.40E-06 | 22.960 |
| family | Bifidobacteriaceae | rs115553816 | G | A | 0.078 | 0.016 | 2.18E-06 | 22.959 |
| family | FamilyXI | rs75969889 | G | T | -0.145 | 0.030 | 1.65E-06 | 22.959 |
| family | Bifidobacteriaceae | rs11745923 | T | G | 0.058 | 0.012 | 2.49E-06 | 22.949 |
| family | Erysipelotrichaceae | rs76420385 | G | A | -0.093 | 0.019 | 1.47E-06 | 22.941 |
| family | Rikenellaceae | rs56098078 | A | C | 0.077 | 0.016 | 1.29E-06 | 22.941 |
| family | Bifidobacteriaceae | rs10928523 | C | T | 0.057 | 0.012 | 1.16E-06 | 22.940 |
| family | Bifidobacteriaceae | rs12476127 | A | G | 0.076 | 0.016 | 2.51E-06 | 22.921 |
| family | Verrucomicrobiaceae | rs9349825 | G | A | -0.070 | 0.015 | 2.51E-06 | 22.919 |
| family | ClostridialesvadinBB60group | rs9305614 | A | G | -0.067 | 0.014 | 1.85E-06 | 22.911 |
| family | Methanobacteriaceae | rs6784384 | G | A | -0.198 | 0.041 | 1.90E-06 | 22.909 |
| family | ClostridialesvadinBB60group | rs72840348 | T | C | 0.128 | 0.027 | 4.11E-06 | 22.908 |
| family | Streptococcaceae | rs4865417 | A | G | -0.089 | 0.019 | 6.85E-06 | 22.903 |
| family | BacteroidalesS24 | rs803053 | T | A | 0.081 | 0.017 | 3.13E-06 | 22.898 |
| family | Bifidobacteriaceae | rs7589832 | A | C | 0.070 | 0.015 | 3.88E-06 | 22.890 |
| family | Bifidobacteriaceae | rs13020688 | A | G | 0.058 | 0.012 | 1.57E-06 | 22.887 |
| family | Alcaligenaceae | rs9537937 | A | T | -0.053 | 0.011 | 1.60E-06 | 22.861 |
| family | FamilyXIII | rs118170811 | G | A | 0.152 | 0.032 | 1.80E-06 | 22.860 |
| family | Streptococcaceae | rs991041 | T | A | -0.053 | 0.011 | 1.77E-06 | 22.860 |
| family | Prevotellaceae | rs8095689 | T | A | -0.082 | 0.017 | 1.55E-06 | 22.860 |
| family | Porphyromonadaceae | rs7851755 | G | T | -0.068 | 0.014 | 9.93E-07 | 22.827 |
| family | BacteroidalesS24 | rs17043777 | T | G | -0.158 | 0.033 | 5.79E-06 | 22.825 |
| family | Porphyromonadaceae | rs17065781 | T | G | -0.058 | 0.012 | 2.40E-06 | 22.823 |
| family | Rikenellaceae | rs55903929 | C | G | 0.076 | 0.016 | 1.36E-06 | 22.820 |
| family | Methanobacteriaceae | rs62240384 | C | T | -0.198 | 0.041 | 1.99E-06 | 22.817 |
| family | Alcaligenaceae | rs2367850 | C | G | 0.063 | 0.013 | 1.85E-06 | 22.814 |
| family | Streptococcaceae | rs11249980 | C | T | -0.060 | 0.013 | 1.68E-06 | 22.798 |
| family | Methanobacteriaceae | rs56209241 | G | A | -0.202 | 0.042 | 2.53E-06 | 22.793 |
| family | Methanobacteriaceae | rs73457427 | G | A | 0.213 | 0.045 | 1.86E-06 | 22.790 |
| family | Bifidobacteriaceae | rs13415439 | C | A | 0.061 | 0.013 | 1.40E-06 | 22.788 |
| family | Erysipelotrichaceae | rs2239640 | T | G | 0.051 | 0.011 | 1.79E-06 | 22.783 |
| family | Bifidobacteriaceae | rs72976292 | T | A | 0.061 | 0.013 | 1.41E-06 | 22.783 |
| family | Verrucomicrobiaceae | rs2602429 | T | C | 0.075 | 0.016 | 2.70E-06 | 22.781 |
| family | Verrucomicrobiaceae | rs4936098 | G | A | 0.065 | 0.014 | 1.13E-06 | 22.775 |
| family | Bifidobacteriaceae | rs1348790 | C | G | 0.057 | 0.012 | 1.26E-06 | 22.770 |
| family | Bifidobacteriaceae | rs16833170 | A | G | 0.061 | 0.013 | 1.44E-06 | 22.754 |
| family | Erysipelotrichaceae | rs9852239 | C | T | 0.051 | 0.011 | 1.81E-06 | 22.752 |
| family | Bifidobacteriaceae | rs150183636 | C | T | 0.096 | 0.020 | 4.08E-06 | 22.750 |
| family | Victivallaceae | rs13234582 | A | G | 0.122 | 0.026 | 1.67E-06 | 22.748 |
| family | Erysipelotrichaceae | rs78782849 | C | T | -0.092 | 0.019 | 1.61E-06 | 22.731 |
| family | Lachnospiraceae | rs11979110 | C | T | -0.050 | 0.011 | 1.82E-06 | 22.730 |
| family | Rhodospirillaceae | rs16855101 | T | C | 0.119 | 0.025 | 1.99E-06 | 22.728 |
| family | Oxalobacteraceae | rs10746751 | T | C | 0.095 | 0.020 | 1.95E-06 | 22.722 |
| family | Erysipelotrichaceae | rs9863872 | G | T | 0.051 | 0.011 | 1.87E-06 | 22.709 |
| family | Rikenellaceae | rs9594204 | C | T | 0.076 | 0.016 | 1.54E-06 | 22.705 |
| family | Porphyromonadaceae | rs6953849 | G | A | 0.072 | 0.015 | 2.44E-06 | 22.702 |
| family | Ruminococcaceae | rs2113833 | C | T | 0.169 | 0.036 | 1.14E-06 | 22.701 |
| family | FamilyXI | rs11547158 | G | A | -0.178 | 0.037 | 2.70E-06 | 22.696 |
| family | Erysipelotrichaceae | rs9846149 | G | C | 0.051 | 0.011 | 1.87E-06 | 22.696 |
| family | Defluviitaleaceae | rs112893842 | C | T | 0.111 | 0.023 | 2.75E-06 | 22.686 |
| family | Bifidobacteriaceae | rs10496738 | C | T | 0.077 | 0.016 | 2.89E-06 | 22.685 |
| family | Alcaligenaceae | rs9569823 | C | T | -0.054 | 0.011 | 1.71E-06 | 22.684 |
| family | Victivallaceae | rs2731834 | G | C | 0.124 | 0.026 | 2.33E-06 | 22.678 |
| family | Bifidobacteriaceae | rs59869505 | A | C | 0.061 | 0.013 | 1.46E-06 | 22.671 |
| family | Peptostreptococcaceae | rs11616274 | C | G | -0.068 | 0.014 | 2.41E-06 | 22.670 |
| family | Streptococcaceae | rs2952183 | C | T | -0.073 | 0.015 | 1.40E-06 | 22.669 |
| family | Streptococcaceae | rs2975655 | G | A | -0.060 | 0.013 | 1.74E-06 | 22.667 |
| family | Victivallaceae | rs59078374 | C | G | 0.143 | 0.030 | 4.98E-06 | 22.666 |
| family | Oxalobacteraceae | rs17543178 | A | G | -0.134 | 0.028 | 2.07E-06 | 22.666 |
| family | Rhodospirillaceae | rs76784716 | G | A | 0.136 | 0.029 | 1.49E-06 | 22.666 |
| family | ClostridialesvadinBB60group | rs6503944 | A | G | 0.127 | 0.027 | 3.97E-06 | 22.663 |
| family | Methanobacteriaceae | rs4848258 | A | T | -0.112 | 0.024 | 2.44E-06 | 22.649 |
| family | Peptococcaceae | rs7009013 | T | C | 0.088 | 0.019 | 3.59E-06 | 22.646 |
| family | Streptococcaceae | rs74476543 | G | A | -0.095 | 0.020 | 3.32E-06 | 22.646 |
| family | Bifidobacteriaceae | rs10841473 | C | G | -0.061 | 0.013 | 2.28E-06 | 22.638 |
| family | Bifidobacteriaceae | rs112396243 | C | T | 0.077 | 0.016 | 2.60E-06 | 22.636 |
| family | ClostridialesvadinBB60group | rs7538333 | T | C | -0.067 | 0.014 | 2.18E-06 | 22.635 |
| family | Erysipelotrichaceae | rs10781552 | T | C | -0.055 | 0.012 | 2.33E-06 | 22.633 |
| family | Coriobacteriaceae | rs240104 | C | T | -0.060 | 0.013 | 1.52E-06 | 22.630 |
| family | ClostridialesvadinBB60group | rs7220068 | A | T | 0.127 | 0.027 | 3.97E-06 | 22.626 |
| family | Streptococcaceae | rs11110092 | C | T | -0.093 | 0.020 | 2.33E-06 | 22.624 |
| family | Methanobacteriaceae | rs4848970 | G | T | 0.116 | 0.024 | 1.89E-06 | 22.619 |
| family | ClostridialesvadinBB60group | rs13185255 | A | G | -0.114 | 0.024 | 5.49E-06 | 22.615 |
| family | BacteroidalesS24 | rs12748533 | T | G | -0.082 | 0.017 | 2.59E-06 | 22.605 |
| family | Bifidobacteriaceae | rs721765 | C | A | 0.077 | 0.016 | 2.94E-06 | 22.598 |
| family | Methanobacteriaceae | rs62240373 | G | A | -0.199 | 0.042 | 2.75E-06 | 22.598 |
| family | Streptococcaceae | rs10860564 | T | C | -0.095 | 0.020 | 3.14E-06 | 22.596 |
| family | Victivallaceae | rs1363428 | A | G | 0.122 | 0.026 | 2.47E-06 | 22.592 |
| family | Streptococcaceae | rs2952228 | C | T | -0.060 | 0.013 | 1.70E-06 | 22.577 |
| family | Methanobacteriaceae | rs11147839 | G | A | 0.211 | 0.044 | 2.28E-06 | 22.574 |
| family | Methanobacteriaceae | rs62241836 | C | T | -0.199 | 0.042 | 2.84E-06 | 22.571 |
| family | Streptococcaceae | rs10000595 | C | A | -0.088 | 0.019 | 7.78E-06 | 22.569 |
| family | FamilyXI | rs112933391 | G | A | -0.175 | 0.037 | 3.08E-06 | 22.567 |
| family | Bifidobacteriaceae | rs73958618 | A | G | 0.069 | 0.015 | 4.27E-06 | 22.566 |
| family | Victivallaceae | rs1895249 | G | T | 0.122 | 0.026 | 2.42E-06 | 22.564 |
| family | Lactobacillaceae | rs78414635 | T | G | -0.181 | 0.038 | 1.63E-06 | 22.563 |
| family | Alcaligenaceae | rs74692987 | G | A | -0.060 | 0.013 | 1.35E-06 | 22.556 |
| family | Bifidobacteriaceae | rs12474975 | A | T | 0.077 | 0.016 | 2.48E-06 | 22.549 |
| family | Bifidobacteriaceae | rs7570283 | T | C | 0.092 | 0.019 | 4.26E-06 | 22.547 |
| family | Alcaligenaceae | rs1409517 | A | G | -0.053 | 0.011 | 1.95E-06 | 22.545 |
| family | Acidaminococcaceae | rs6589457 | G | A | 0.166 | 0.035 | 2.32E-06 | 22.533 |
| family | Oxalobacteraceae | rs10780691 | T | C | 0.093 | 0.020 | 2.17E-06 | 22.532 |
| family | Lactobacillaceae | rs7399658 | A | G | -0.105 | 0.022 | 4.42E-06 | 22.529 |
| family | Alcaligenaceae | rs35781367 | A | G | -0.053 | 0.011 | 1.90E-06 | 22.527 |
| family | Alcaligenaceae | rs7318351 | A | T | -0.053 | 0.011 | 1.96E-06 | 22.514 |
| family | Bifidobacteriaceae | rs6746633 | G | A | 0.096 | 0.020 | 4.44E-06 | 22.508 |
| family | Bifidobacteriaceae | rs7574366 | G | A | 0.096 | 0.020 | 4.08E-06 | 22.499 |
| family | Bifidobacteriaceae | rs56108664 | C | T | 0.075 | 0.016 | 1.37E-06 | 22.497 |
| family | Streptococcaceae | rs11110098 | G | A | -0.092 | 0.019 | 2.99E-06 | 22.494 |
| family | ClostridialesvadinBB60group | rs7212702 | G | A | 0.124 | 0.026 | 6.64E-06 | 22.494 |
| family | Verrucomicrobiaceae | rs117107102 | G | A | 0.205 | 0.043 | 2.92E-06 | 22.493 |
| family | Verrucomicrobiaceae | rs74542928 | C | T | 0.112 | 0.024 | 1.65E-06 | 22.492 |
| family | Streptococcaceae | rs11110086 | G | A | -0.095 | 0.020 | 3.01E-06 | 22.487 |
| family | Alcaligenaceae | rs35329616 | C | T | -0.052 | 0.011 | 1.99E-06 | 22.486 |
| family | FamilyXI | rs7932399 | G | C | -0.143 | 0.030 | 2.11E-06 | 22.481 |
| family | Prevotellaceae | rs912860 | A | G | -0.229 | 0.048 | 9.30E-07 | 22.476 |
| family | FamilyXI | rs55893383 | C | T | -0.175 | 0.037 | 3.00E-06 | 22.474 |
| family | Alcaligenaceae | rs1575903 | A | G | -0.052 | 0.011 | 2.01E-06 | 22.470 |
| family | Porphyromonadaceae | rs10757293 | T | C | -0.067 | 0.014 | 1.34E-06 | 22.465 |
| family | Bifidobacteriaceae | rs6710892 | G | A | 0.085 | 0.018 | 1.90E-06 | 22.439 |
| family | Lactobacillaceae | rs11674854 | T | C | -0.083 | 0.018 | 2.60E-06 | 22.438 |
| family | Bifidobacteriaceae | rs10928522 | C | A | 0.057 | 0.012 | 1.43E-06 | 22.435 |
| family | Lactobacillaceae | rs7399816 | G | A | -0.104 | 0.022 | 4.79E-06 | 22.434 |
| family | Rikenellaceae | rs36125440 | C | T | -0.098 | 0.021 | 4.53E-06 | 22.428 |
| family | Peptococcaceae | rs75898026 | G | A | -0.082 | 0.017 | 2.02E-06 | 22.423 |
| family | ClostridialesvadinBB60group | rs7538034 | G | T | -0.079 | 0.017 | 2.37E-06 | 22.423 |
| family | Victivallaceae | rs72960925 | C | T | -0.193 | 0.041 | 1.20E-06 | 22.421 |
| family | Rhodospirillaceae | rs75071199 | C | A | 0.135 | 0.029 | 1.61E-06 | 22.413 |
| family | Streptococcaceae | rs11110096 | A | G | -0.094 | 0.020 | 3.07E-06 | 22.403 |
| family | Streptococcaceae | rs11110085 | C | A | -0.094 | 0.020 | 3.16E-06 | 22.402 |
| family | Bifidobacteriaceae | rs7556781 | A | G | 0.068 | 0.014 | 4.97E-06 | 22.402 |
| family | Christensenellaceae | rs117186816 | A | G | -0.206 | 0.044 | 6.52E-06 | 22.389 |
| family | Desulfovibrionaceae | rs11599763 | C | T | -0.056 | 0.012 | 2.50E-06 | 22.388 |
| family | Bifidobacteriaceae | rs1446524 | A | G | 0.057 | 0.012 | 1.39E-06 | 22.385 |
| family | Streptococcaceae | rs17613967 | T | G | -0.088 | 0.019 | 8.33E-06 | 22.376 |
| family | Bifidobacteriaceae | rs6712127 | A | G | 0.069 | 0.015 | 5.30E-06 | 22.375 |
| family | Victivallaceae | rs33893 | A | G | 0.123 | 0.026 | 2.86E-06 | 22.375 |
| family | Bifidobacteriaceae | rs6755391 | C | T | 0.085 | 0.018 | 2.00E-06 | 22.374 |
| family | FamilyXIII | rs12643275 | T | A | -0.055 | 0.012 | 2.42E-06 | 22.372 |
| family | Peptostreptococcaceae | rs4328317 | T | G | -0.067 | 0.014 | 2.89E-06 | 22.370 |
| family | Prevotellaceae | rs12143482 | C | T | -0.076 | 0.016 | 6.66E-06 | 22.357 |
| family | Verrucomicrobiaceae | rs12908520 | A | G | 0.062 | 0.013 | 2.15E-06 | 22.353 |
| family | ClostridialesvadinBB60group | rs72840345 | G | A | 0.127 | 0.027 | 5.08E-06 | 22.346 |
| family | BacteroidalesS24 | rs1850003 | G | A | 0.084 | 0.018 | 2.41E-06 | 22.344 |
| family | FamilyXI | rs11826840 | A | G | -0.143 | 0.030 | 2.31E-06 | 22.339 |
| family | Porphyromonadaceae | rs10811655 | C | G | -0.066 | 0.014 | 1.50E-06 | 22.334 |
| family | Rikenellaceae | rs7995332 | C | A | 0.080 | 0.017 | 7.77E-07 | 22.331 |
| family | Oxalobacteraceae | rs4877289 | G | A | 0.093 | 0.020 | 2.31E-06 | 22.320 |
| family | Acidaminococcaceae | rs2933324 | G | A | -0.066 | 0.014 | 2.24E-06 | 22.315 |
| family | Bifidobacteriaceae | rs16831899 | G | A | 0.085 | 0.018 | 2.06E-06 | 22.314 |
| family | Desulfovibrionaceae | rs17791387 | G | A | -0.073 | 0.015 | 2.10E-06 | 22.314 |
| family | Bifidobacteriaceae | rs177917 | A | G | 0.074 | 0.016 | 2.37E-06 | 22.312 |
| family | Bifidobacteriaceae | rs62168871 | A | G | 0.057 | 0.012 | 1.62E-06 | 22.305 |
| family | Lachnospiraceae | rs79086868 | C | T | 0.078 | 0.016 | 3.01E-06 | 22.302 |
| family | Streptococcaceae | rs2952172 | T | C | -0.058 | 0.012 | 2.19E-06 | 22.302 |
| family | Alcaligenaceae | rs7638039 | C | T | 0.060 | 0.013 | 2.70E-06 | 22.295 |
| family | Porphyromonadaceae | rs13221186 | G | C | 0.088 | 0.019 | 3.28E-06 | 22.290 |
| family | Victivallaceae | rs907574 | G | A | -0.193 | 0.041 | 1.60E-06 | 22.288 |
| family | Methanobacteriaceae | rs6541968 | T | C | -0.111 | 0.024 | 2.96E-06 | 22.282 |
| family | Streptococcaceae | rs7224098 | A | G | -0.052 | 0.011 | 2.44E-06 | 22.281 |
| family | Streptococcaceae | rs12370772 | G | C | -0.094 | 0.020 | 4.17E-06 | 22.281 |
| family | Bifidobacteriaceae | rs4954193 | A | G | 0.056 | 0.012 | 1.62E-06 | 22.277 |
| family | Streptococcaceae | rs12367581 | T | A | -0.094 | 0.020 | 3.77E-06 | 22.276 |
| family | Oxalobacteraceae | rs75858443 | C | T | -0.133 | 0.028 | 2.69E-06 | 22.273 |
| family | Bifidobacteriaceae | rs10176573 | C | A | 0.056 | 0.012 | 1.76E-06 | 22.271 |
| family | Verrucomicrobiaceae | rs56161035 | C | T | 0.068 | 0.014 | 2.33E-06 | 22.270 |
| family | Streptococcaceae | rs1120106 | C | T | -0.052 | 0.011 | 2.22E-06 | 22.261 |
| family | Bifidobacteriaceae | rs6743537 | G | A | 0.075 | 0.016 | 3.22E-06 | 22.260 |
| family | Streptococcaceae | rs11110165 | T | G | -0.081 | 0.017 | 2.13E-06 | 22.257 |
| family | Prevotellaceae | rs148376875 | G | T | 0.085 | 0.018 | 2.08E-06 | 22.252 |
| family | Streptococcaceae | rs2460111 | G | A | -0.052 | 0.011 | 2.36E-06 | 22.248 |
| family | Bifidobacteriaceae | rs60241067 | A | T | 0.062 | 0.013 | 4.15E-06 | 22.241 |
| family | Bifidobacteriaceae | rs79826902 | G | A | 0.077 | 0.016 | 3.24E-06 | 22.239 |
| family | Lactobacillaceae | rs1590596 | G | C | -0.104 | 0.022 | 3.90E-06 | 22.238 |
| family | Bifidobacteriaceae | rs6430554 | C | T | 0.056 | 0.012 | 1.76E-06 | 22.230 |
| family | Methanobacteriaceae | rs9594530 | T | C | 0.210 | 0.045 | 2.55E-06 | 22.227 |
| family | Bifidobacteriaceae | rs111682961 | C | T | 0.077 | 0.016 | 3.32E-06 | 22.225 |
| family | Bifidobacteriaceae | rs7589297 | C | A | 0.056 | 0.012 | 1.81E-06 | 22.225 |
| family | Peptostreptococcaceae | rs9565296 | C | T | -0.067 | 0.014 | 3.20E-06 | 22.215 |
| family | Peptococcaceae | rs6558812 | G | A | 0.088 | 0.019 | 4.35E-06 | 22.213 |
| family | Oxalobacteraceae | rs77722911 | C | A | -0.133 | 0.028 | 2.74E-06 | 22.212 |
| family | ClostridialesvadinBB60group | rs10515175 | T | C | 0.126 | 0.027 | 5.14E-06 | 22.211 |
| family | ClostridialesvadinBB60group | rs118104867 | T | C | 0.214 | 0.046 | 3.44E-06 | 22.207 |
| family | Rikenellaceae | rs1923716 | C | A | 0.075 | 0.016 | 1.83E-06 | 22.206 |
| family | Bifidobacteriaceae | rs16832243 | C | T | 0.077 | 0.016 | 3.09E-06 | 22.206 |
| family | FamilyXI | rs35293957 | G | A | -0.169 | 0.036 | 3.54E-06 | 22.204 |
| family | Bacteroidaceae | rs17619981 | G | T | 0.088 | 0.019 | 2.69E-06 | 22.194 |
| family | Lachnospiraceae | rs13234407 | G | A | -0.049 | 0.011 | 2.41E-06 | 22.193 |
| family | Rikenellaceae | rs9712594 | A | C | -0.084 | 0.018 | 2.90E-06 | 22.190 |
| family | Porphyromonadaceae | rs10811677 | G | T | -0.067 | 0.014 | 1.50E-06 | 22.187 |
| family | Bifidobacteriaceae | rs309161 | G | A | 0.074 | 0.016 | 2.67E-06 | 22.180 |
| family | Bifidobacteriaceae | rs4334924 | G | A | 0.057 | 0.012 | 4.16E-06 | 22.177 |
| family | Veillonellaceae | rs114889439 | G | A | -0.254 | 0.054 | 6.19E-06 | 22.160 |
| family | Victivallaceae | rs1363429 | G | T | 0.118 | 0.025 | 3.60E-06 | 22.159 |
| family | Bifidobacteriaceae | rs41389446 | G | T | -0.087 | 0.018 | 2.47E-06 | 22.153 |
| family | Victivallaceae | rs7131017 | G | A | -0.134 | 0.028 | 3.93E-06 | 22.151 |
| family | Bifidobacteriaceae | rs309119 | T | C | 0.074 | 0.016 | 2.33E-06 | 22.149 |
| family | Peptostreptococcaceae | rs117644882 | C | T | 0.178 | 0.038 | 2.80E-06 | 22.142 |
| family | Acidaminococcaceae | rs6923842 | C | T | -0.080 | 0.017 | 2.21E-06 | 22.140 |
| family | Bifidobacteriaceae | rs1446525 | G | A | 0.056 | 0.012 | 1.84E-06 | 22.137 |
| family | BacteroidalesS24 | rs12438801 | A | G | 0.082 | 0.017 | 2.47E-06 | 22.135 |
| family | Peptostreptococcaceae | rs75819860 | A | T | 0.126 | 0.027 | 2.49E-06 | 22.133 |
| family | Streptococcaceae | rs2447442 | C | T | -0.052 | 0.011 | 2.65E-06 | 22.127 |
| family | Verrucomicrobiaceae | rs112769434 | A | G | 0.111 | 0.024 | 2.18E-06 | 22.123 |
| family | FamilyXIII | rs6797051 | T | C | -0.081 | 0.017 | 4.89E-06 | 22.121 |
| family | Bifidobacteriaceae | rs2166480 | G | A | 0.056 | 0.012 | 1.89E-06 | 22.115 |
| family | Defluviitaleaceae | rs73217031 | C | T | 0.170 | 0.036 | 3.15E-06 | 22.113 |
| family | Bifidobacteriaceae | rs766270 | G | C | 0.056 | 0.012 | 2.00E-06 | 22.111 |
| family | Rikenellaceae | rs9603191 | G | A | 0.075 | 0.016 | 1.91E-06 | 22.107 |
| family | ClostridialesvadinBB60group | rs2302202 | T | C | 0.123 | 0.026 | 7.79E-06 | 22.101 |
| family | Peptostreptococcaceae | rs76762387 | G | T | 0.175 | 0.037 | 2.73E-06 | 22.097 |
| family | Bifidobacteriaceae | rs16832205 | G | A | 0.076 | 0.016 | 3.40E-06 | 22.096 |
| family | Lachnospiraceae | rs13230111 | A | G | -0.049 | 0.011 | 2.58E-06 | 22.095 |
| family | Veillonellaceae | rs12668619 | G | A | 0.055 | 0.012 | 2.57E-06 | 22.093 |
| family | Porphyromonadaceae | rs2675411 | G | A | 0.057 | 0.012 | 3.45E-06 | 22.093 |
| family | Bifidobacteriaceae | rs6706537 | T | C | 0.056 | 0.012 | 1.74E-06 | 22.090 |
| family | Victivallaceae | rs35004924 | A | C | 0.122 | 0.026 | 1.98E-06 | 22.081 |
| family | Bifidobacteriaceae | rs1374292 | G | A | 0.056 | 0.012 | 1.80E-06 | 22.079 |
| family | Bifidobacteriaceae | rs2164918 | T | G | 0.091 | 0.019 | 5.28E-06 | 22.074 |
| family | Streptococcaceae | rs35344081 | A | G | 0.061 | 0.013 | 2.64E-06 | 22.071 |
| family | Streptococcaceae | rs79222628 | G | A | -0.087 | 0.019 | 2.49E-06 | 22.068 |
| family | Peptostreptococcaceae | rs17747079 | A | G | -0.066 | 0.014 | 3.00E-06 | 22.067 |
| family | Veillonellaceae | rs17263892 | C | A | 0.099 | 0.021 | 3.69E-06 | 22.066 |
| family | ClostridialesvadinBB60group | rs13409132 | G | A | -0.165 | 0.035 | 4.37E-06 | 22.065 |
| family | Bifidobacteriaceae | rs309170 | C | T | 0.073 | 0.016 | 2.80E-06 | 22.050 |
| family | Methanobacteriaceae | rs9942109 | G | A | -0.197 | 0.042 | 3.60E-06 | 22.046 |
| family | Methanobacteriaceae | rs62240388 | A | G | -0.196 | 0.042 | 3.06E-06 | 22.046 |
| family | Rikenellaceae | rs62532512 | A | C | -0.050 | 0.011 | 2.76E-06 | 22.045 |
| family | Bifidobacteriaceae | rs6430551 | T | C | 0.056 | 0.012 | 1.64E-06 | 22.044 |
| family | Streptococcaceae | rs2440137 | T | C | -0.052 | 0.011 | 2.48E-06 | 22.038 |
| family | Peptococcaceae | rs117452796 | G | A | -0.258 | 0.055 | 3.15E-06 | 22.036 |
| family | Lachnospiraceae | rs112040820 | G | A | 0.055 | 0.012 | 2.42E-06 | 22.032 |
| family | Bifidobacteriaceae | rs55842567 | G | A | 0.057 | 0.012 | 4.73E-06 | 22.032 |
| family | Peptostreptococcaceae | rs59987323 | T | C | -0.054 | 0.011 | 2.70E-06 | 22.029 |
| family | Lachnospiraceae | rs11755180 | G | C | -0.049 | 0.010 | 2.69E-06 | 22.024 |
| family | Rhodospirillaceae | rs4531902 | T | C | 0.117 | 0.025 | 2.74E-06 | 22.016 |
| family | ClostridialesvadinBB60group | rs16943994 | G | A | 0.126 | 0.027 | 6.01E-06 | 22.009 |
| family | Victivallaceae | rs2194089 | C | T | 0.117 | 0.025 | 4.03E-06 | 22.009 |
| family | Bifidobacteriaceae | rs62168872 | C | T | 0.056 | 0.012 | 2.05E-06 | 22.008 |
| family | FamilyXIII | rs482905 | T | G | 0.060 | 0.013 | 3.72E-06 | 22.003 |
| family | Defluviitaleaceae | rs10026953 | G | A | -0.105 | 0.022 | 7.90E-06 | 22.003 |
| family | Prevotellaceae | rs55780618 | C | G | -0.075 | 0.016 | 7.80E-06 | 21.999 |
| family | ClostridialesvadinBB60group | rs2191834 | T | G | 0.075 | 0.016 | 2.50E-06 | 21.998 |
| family | Streptococcaceae | rs4865415 | T | C | -0.089 | 0.019 | 8.33E-06 | 21.992 |
| family | Victivallaceae | rs2944279 | G | A | -0.120 | 0.026 | 2.95E-06 | 21.990 |
| family | Bifidobacteriaceae | rs4954197 | G | A | 0.056 | 0.012 | 2.07E-06 | 21.990 |
| family | Defluviitaleaceae | rs9725395 | G | A | -0.138 | 0.030 | 3.41E-06 | 21.969 |
| family | Porphyromonadaceae | rs17140890 | T | C | 0.071 | 0.015 | 4.47E-06 | 21.964 |
| family | Peptococcaceae | rs150600492 | C | A | 0.136 | 0.029 | 2.31E-06 | 21.957 |
| family | BacteroidalesS24 | rs17043487 | T | G | -0.163 | 0.035 | 3.39E-06 | 21.955 |
| family | Erysipelotrichaceae | rs9859401 | A | C | 0.050 | 0.011 | 2.70E-06 | 21.954 |
| family | Alcaligenaceae | rs59349789 | A | T | -0.059 | 0.013 | 2.82E-06 | 21.952 |
| family | Peptostreptococcaceae | rs1981301 | G | A | -0.067 | 0.014 | 3.80E-06 | 21.946 |
| family | Desulfovibrionaceae | rs4506934 | T | C | -0.094 | 0.020 | 3.16E-06 | 21.945 |
| family | Verrucomicrobiaceae | rs1491244 | A | T | 0.111 | 0.024 | 2.49E-06 | 21.936 |
| family | FamilyXIII | rs7514702 | C | T | -0.066 | 0.014 | 3.92E-06 | 21.931 |
| family | Actinomycetaceae | rs34583783 | T | G | 0.124 | 0.026 | 5.48E-06 | 21.930 |
| family | FamilyXI | rs13228115 | C | T | -0.173 | 0.037 | 4.14E-06 | 21.926 |
| family | Rhodospirillaceae | rs11591293 | T | G | 0.074 | 0.016 | 2.67E-06 | 21.923 |
| family | Prevotellaceae | rs12142231 | A | G | -0.076 | 0.016 | 4.12E-06 | 21.921 |
| family | Prevotellaceae | rs11151192 | G | T | -0.081 | 0.017 | 2.54E-06 | 21.920 |
| family | Prevotellaceae | rs11151195 | A | G | -0.081 | 0.017 | 2.77E-06 | 21.913 |
| family | Bacteroidaceae | rs9507307 | T | C | 0.060 | 0.013 | 2.13E-06 | 21.912 |
| family | Streptococcaceae | rs2120255 | C | A | -0.052 | 0.011 | 2.95E-06 | 21.907 |
| family | Bifidobacteriaceae | rs4954196 | A | G | 0.056 | 0.012 | 2.16E-06 | 21.903 |
| family | Coriobacteriaceae | rs10995776 | C | T | 0.082 | 0.017 | 2.44E-06 | 21.903 |
| family | Methanobacteriaceae | rs62240380 | C | T | -0.193 | 0.041 | 3.32E-06 | 21.900 |
| family | Ruminococcaceae | rs4347803 | G | A | 0.162 | 0.035 | 2.15E-06 | 21.895 |
| family | FamilyXI | rs697771 | G | A | -0.118 | 0.025 | 3.19E-06 | 21.895 |
| family | Erysipelotrichaceae | rs2300773 | A | G | 0.050 | 0.011 | 2.82E-06 | 21.891 |
| family | Victivallaceae | rs7077363 | A | G | 0.149 | 0.032 | 2.83E-06 | 21.887 |
| family | Victivallaceae | rs4079618 | G | C | -0.191 | 0.041 | 1.76E-06 | 21.882 |
| family | Bacteroidaceae | rs495004 | G | C | -0.061 | 0.013 | 3.42E-06 | 21.881 |
| family | Alcaligenaceae | rs112159068 | T | A | 0.131 | 0.028 | 3.56E-06 | 21.881 |
| family | Peptostreptococcaceae | rs1963247 | G | T | -0.053 | 0.011 | 2.83E-06 | 21.878 |
| family | Bifidobacteriaceae | rs11742144 | G | A | 0.057 | 0.012 | 4.22E-06 | 21.878 |
| family | ClostridialesvadinBB60group | rs13020025 | A | G | 0.075 | 0.016 | 2.79E-06 | 21.876 |
| family | Defluviitaleaceae | rs2102252 | C | G | -0.105 | 0.022 | 8.66E-06 | 21.876 |
| family | Bifidobacteriaceae | rs56146525 | A | C | 0.057 | 0.012 | 5.54E-06 | 21.868 |
| family | Streptococcaceae | rs67455183 | T | G | -0.075 | 0.016 | 2.70E-06 | 21.857 |
| family | Erysipelotrichaceae | rs9867473 | C | A | 0.050 | 0.011 | 2.36E-06 | 21.845 |
| family | Peptostreptococcaceae | rs1520207 | T | C | 0.053 | 0.011 | 3.17E-06 | 21.842 |
| family | Prevotellaceae | rs4685827 | C | T | -0.068 | 0.015 | 2.77E-06 | 21.840 |
| family | Peptostreptococcaceae | rs2328994 | C | T | -0.065 | 0.014 | 3.97E-06 | 21.833 |
| family | FamilyXI | rs56205120 | C | T | -0.171 | 0.037 | 4.48E-06 | 21.829 |
| family | Lachnospiraceae | rs13233731 | G | A | -0.049 | 0.011 | 2.91E-06 | 21.824 |
| family | Peptococcaceae | rs11136654 | T | G | 0.086 | 0.018 | 5.26E-06 | 21.822 |
| family | Peptostreptococcaceae | rs4747289 | A | G | 0.065 | 0.014 | 2.94E-06 | 21.816 |
| family | Oxalobacteraceae | rs73733958 | G | T | -0.132 | 0.028 | 3.33E-06 | 21.811 |
| family | Acidaminococcaceae | rs6924018 | C | A | -0.079 | 0.017 | 2.63E-06 | 21.805 |
| family | Bifidobacteriaceae | rs3769018 | T | C | 0.094 | 0.020 | 5.76E-06 | 21.804 |
| family | Bifidobacteriaceae | rs79769806 | A | G | -0.086 | 0.018 | 2.93E-06 | 21.803 |
| family | BacteroidalesS24 | rs17280811 | A | C | 0.081 | 0.017 | 3.28E-06 | 21.800 |
| family | Streptococcaceae | rs11110084 | G | A | -0.087 | 0.019 | 2.94E-06 | 21.799 |
| family | Lachnospiraceae | rs1986141 | G | A | -0.054 | 0.012 | 4.27E-06 | 21.799 |
| family | Oxalobacteraceae | rs10780692 | T | C | 0.094 | 0.020 | 3.02E-06 | 21.796 |
| family | FamilyXI | rs3733511 | G | A | 0.128 | 0.027 | 3.39E-06 | 21.795 |
| family | Enterobacteriaceae | rs6092361 | G | A | 0.060 | 0.013 | 3.27E-06 | 21.786 |
| family | ClostridialesvadinBB60group | rs72840351 | G | C | 0.138 | 0.030 | 6.48E-06 | 21.783 |
| family | ClostridialesvadinBB60group | rs2894671 | C | T | 0.074 | 0.016 | 2.81E-06 | 21.782 |
| family | Bacteroidaceae | rs2023437 | C | T | -0.078 | 0.017 | 5.02E-06 | 21.780 |
| family | Bifidobacteriaceae | rs12464050 | G | T | 0.076 | 0.016 | 4.03E-06 | 21.777 |
| family | Bifidobacteriaceae | rs3098103 | T | C | 0.076 | 0.016 | 4.27E-06 | 21.771 |
| family | Defluviitaleaceae | rs6840503 | G | A | -0.103 | 0.022 | 8.27E-06 | 21.771 |
| family | BacteroidalesS24 | rs601978 | A | T | 0.083 | 0.018 | 3.71E-06 | 21.771 |
| family | Pasteurellaceae | rs6092684 | T | A | 0.068 | 0.015 | 3.44E-06 | 21.769 |
| family | Streptococcaceae | rs2447443 | G | C | -0.052 | 0.011 | 3.21E-06 | 21.765 |
| family | Bifidobacteriaceae | rs62168891 | C | T | 0.056 | 0.012 | 2.43E-06 | 21.763 |
| family | Prevotellaceae | rs17260438 | G | A | -0.075 | 0.016 | 7.65E-06 | 21.763 |
| family | Bifidobacteriaceae | rs56104584 | T | C | 0.057 | 0.012 | 5.43E-06 | 21.762 |
| family | Methanobacteriaceae | rs58106718 | A | T | -0.193 | 0.041 | 3.43E-06 | 21.758 |
| family | Oxalobacteraceae | rs11246212 | C | T | 0.136 | 0.029 | 4.51E-06 | 21.745 |
| family | Enterobacteriaceae | rs62210023 | G | A | 0.061 | 0.013 | 3.13E-06 | 21.742 |
| family | BacteroidalesS24 | rs17043784 | C | G | -0.155 | 0.033 | 7.28E-06 | 21.741 |
| family | Erysipelotrichaceae | rs13072608 | T | C | 0.050 | 0.011 | 3.05E-06 | 21.741 |
| family | Oxalobacteraceae | rs73733956 | G | A | -0.131 | 0.028 | 3.48E-06 | 21.733 |
| family | Alcaligenaceae | rs72982722 | C | T | 0.131 | 0.028 | 3.85E-06 | 21.732 |
| family | Methanobacteriaceae | rs62241832 | G | A | -0.196 | 0.042 | 4.58E-06 | 21.727 |
| family | BacteroidalesS24 | rs6831034 | A | T | -0.096 | 0.021 | 6.10E-06 | 21.724 |
| family | Methanobacteriaceae | rs56001610 | G | A | -0.195 | 0.042 | 4.49E-06 | 21.721 |
| family | Bifidobacteriaceae | rs11743825 | C | T | 0.056 | 0.012 | 4.71E-06 | 21.720 |
| family | Methanobacteriaceae | rs75208022 | T | C | -0.227 | 0.049 | 5.92E-06 | 21.717 |
| family | Methanobacteriaceae | rs62241844 | G | C | -0.195 | 0.042 | 4.55E-06 | 21.716 |
| family | Methanobacteriaceae | rs62241845 | T | A | -0.195 | 0.042 | 4.55E-06 | 21.716 |
| family | Verrucomicrobiaceae | rs2169719 | A | G | 0.068 | 0.015 | 3.16E-06 | 21.712 |
| family | Peptococcaceae | rs75430375 | T | C | -0.148 | 0.032 | 3.41E-06 | 21.712 |
| family | Bifidobacteriaceae | rs57243841 | A | G | 0.065 | 0.014 | 4.05E-06 | 21.710 |
| family | Bifidobacteriaceae | rs12469941 | C | T | 0.056 | 0.012 | 2.02E-06 | 21.704 |
| family | Rikenellaceae | rs74474130 | G | T | 0.138 | 0.030 | 3.61E-06 | 21.702 |
| family | Verrucomicrobiaceae | rs4242783 | A | G | 0.069 | 0.015 | 2.75E-06 | 21.699 |
| family | Peptostreptococcaceae | rs12586021 | C | T | -0.066 | 0.014 | 4.24E-06 | 21.698 |
| family | Methanobacteriaceae | rs3923545 | G | C | 0.207 | 0.045 | 3.20E-06 | 21.696 |
| family | Bifidobacteriaceae | rs12187440 | G | A | 0.056 | 0.012 | 5.25E-06 | 21.691 |
| family | ClostridialesvadinBB60group | rs34367586 | A | G | 0.125 | 0.027 | 7.05E-06 | 21.691 |
| family | Peptostreptococcaceae | rs4142197 | C | G | -0.066 | 0.014 | 4.01E-06 | 21.690 |
| family | Methanobacteriaceae | rs62241837 | G | A | -0.195 | 0.042 | 4.61E-06 | 21.681 |
| family | Methanobacteriaceae | rs62241838 | T | C | -0.195 | 0.042 | 4.61E-06 | 21.680 |
| family | Rhodospirillaceae | rs55661963 | A | C | 0.133 | 0.029 | 2.76E-06 | 21.674 |
| family | Rhodospirillaceae | rs77497586 | G | T | 0.133 | 0.029 | 2.30E-06 | 21.669 |
| family | Prevotellaceae | rs9586501 | A | G | 0.059 | 0.013 | 2.59E-06 | 21.666 |
| family | Bifidobacteriaceae | rs11896918 | A | G | 0.094 | 0.020 | 5.57E-06 | 21.662 |
| family | FamilyXI | rs3733512 | G | C | 0.128 | 0.027 | 3.70E-06 | 21.659 |
| family | Peptostreptococcaceae | rs76982728 | C | T | 0.124 | 0.027 | 3.24E-06 | 21.652 |
| family | Methanobacteriaceae | rs62241839 | A | G | -0.195 | 0.042 | 4.68E-06 | 21.650 |
| family | Bifidobacteriaceae | rs56042615 | G | A | 0.056 | 0.012 | 5.83E-06 | 21.649 |
| family | FamilyXIII | rs476048 | A | C | 0.059 | 0.013 | 4.46E-06 | 21.643 |
| family | Ruminococcaceae | rs56697967 | A | G | 0.080 | 0.017 | 3.52E-06 | 21.632 |
| family | Streptococcaceae | rs5012592 | C | T | -0.090 | 0.019 | 8.42E-06 | 21.628 |
| family | ClostridialesvadinBB60group | rs7726056 | G | A | -0.111 | 0.024 | 7.97E-06 | 21.625 |
| family | Alcaligenaceae | rs7593823 | G | A | 0.061 | 0.013 | 3.31E-06 | 21.625 |
| family | Rhodospirillaceae | rs3754623 | G | T | 0.093 | 0.020 | 4.75E-06 | 21.614 |
| family | Bifidobacteriaceae | rs7588568 | C | A | -0.061 | 0.013 | 3.73E-06 | 21.612 |
| family | Victivallaceae | rs253712 | G | A | 0.120 | 0.026 | 4.05E-06 | 21.599 |
| family | Erysipelotrichaceae | rs55764236 | T | G | -0.054 | 0.012 | 3.89E-06 | 21.598 |
| family | Coriobacteriaceae | rs34739816 | T | G | 0.097 | 0.021 | 3.88E-06 | 21.594 |
| family | Acidaminococcaceae | rs3012351 | C | T | -0.065 | 0.014 | 3.31E-06 | 21.591 |
| family | Lachnospiraceae | rs34148016 | T | A | -0.054 | 0.012 | 5.30E-06 | 21.590 |
| family | Alcaligenaceae | rs7270486 | C | A | 0.069 | 0.015 | 2.86E-06 | 21.590 |
| family | Bifidobacteriaceae | rs7560412 | C | T | 0.096 | 0.021 | 6.82E-06 | 21.590 |
| family | Erysipelotrichaceae | rs3761717 | C | G | 0.050 | 0.011 | 3.30E-06 | 21.588 |
| family | ClostridialesvadinBB60group | rs17613877 | G | C | 0.074 | 0.016 | 3.23E-06 | 21.588 |
| family | Bifidobacteriaceae | rs77096272 | C | A | 0.076 | 0.016 | 4.47E-06 | 21.587 |
| family | Veillonellaceae | rs4461038 | G | A | -0.055 | 0.012 | 3.73E-06 | 21.579 |
| family | Defluviitaleaceae | rs2102251 | G | A | -0.104 | 0.022 | 9.83E-06 | 21.576 |
| family | Lactobacillaceae | rs768253 | G | T | -0.079 | 0.017 | 3.61E-06 | 21.570 |
| family | Erysipelotrichaceae | rs7095708 | T | C | -0.053 | 0.011 | 4.01E-06 | 21.568 |
| family | Streptococcaceae | rs2952262 | T | A | -0.059 | 0.013 | 3.23E-06 | 21.567 |
| family | Enterobacteriaceae | rs73280755 | A | G | 0.060 | 0.013 | 3.43E-06 | 21.562 |
| family | Pasteurellaceae | rs10965428 | A | C | -0.120 | 0.026 | 4.29E-06 | 21.561 |
| family | Rhodospirillaceae | rs55876211 | T | C | -0.091 | 0.020 | 2.87E-06 | 21.557 |
| family | Defluviitaleaceae | rs9608282 | G | T | 0.139 | 0.030 | 4.61E-06 | 21.554 |
| family | Rikenellaceae | rs77885767 | T | C | -0.156 | 0.034 | 2.85E-06 | 21.552 |
| family | Verrucomicrobiaceae | rs11252894 | C | A | 0.077 | 0.017 | 2.95E-06 | 21.551 |
| family | Bifidobacteriaceae | rs55809728 | G | A | 0.106 | 0.023 | 1.90E-06 | 21.550 |
| family | Bifidobacteriaceae | rs6430555 | A | C | 0.055 | 0.012 | 2.62E-06 | 21.547 |
| family | Bifidobacteriaceae | rs6430550 | C | T | 0.055 | 0.012 | 2.05E-06 | 21.544 |
| family | Erysipelotrichaceae | rs4617508 | T | A | -0.053 | 0.011 | 4.04E-06 | 21.541 |
| family | Bifidobacteriaceae | rs72717412 | C | T | 0.056 | 0.012 | 5.42E-06 | 21.541 |
| family | ClostridialesvadinBB60group | rs7226487 | G | A | -0.064 | 0.014 | 3.58E-06 | 21.537 |
| family | ClostridialesvadinBB60group | rs12612757 | T | C | 0.074 | 0.016 | 3.34E-06 | 21.537 |
| family | Victivallaceae | rs1895248 | T | A | 0.120 | 0.026 | 4.31E-06 | 21.533 |
| family | Bifidobacteriaceae | rs72717410 | A | T | 0.056 | 0.012 | 5.35E-06 | 21.531 |
| family | FamilyXI | rs11976830 | T | C | -0.169 | 0.036 | 5.89E-06 | 21.529 |
| family | Coriobacteriaceae | rs11250875 | C | T | 0.061 | 0.013 | 4.83E-06 | 21.526 |
| family | Erysipelotrichaceae | rs7911287 | G | T | -0.053 | 0.011 | 4.03E-06 | 21.524 |
| family | Erysipelotrichaceae | rs11185506 | C | G | 0.050 | 0.011 | 3.40E-06 | 21.513 |
| family | Erysipelotrichaceae | rs11718657 | A | C | 0.050 | 0.011 | 3.40E-06 | 21.513 |
| family | Bifidobacteriaceae | rs2053727 | C | T | 0.055 | 0.012 | 2.62E-06 | 21.513 |
| family | Rhodospirillaceae | rs3768869 | A | G | 0.093 | 0.020 | 4.92E-06 | 21.508 |
| family | Rhodospirillaceae | rs10181850 | A | C | 0.093 | 0.020 | 4.98E-06 | 21.508 |
| family | Prevotellaceae | rs2206481 | C | A | -0.054 | 0.012 | 3.56E-06 | 21.507 |
| family | ClostridialesvadinBB60group | rs59445772 | A | G | 0.124 | 0.027 | 8.01E-06 | 21.506 |
| family | Peptococcaceae | rs61870295 | A | G | 0.134 | 0.029 | 2.76E-06 | 21.503 |
| family | Victivallaceae | rs33892 | G | A | 0.121 | 0.026 | 4.41E-06 | 21.503 |
| family | Bifidobacteriaceae | rs41269821 | A | G | 0.074 | 0.016 | 3.87E-06 | 21.502 |
| family | Alcaligenaceae | rs62395635 | C | T | 0.111 | 0.024 | 3.35E-06 | 21.495 |
| family | Rikenellaceae | rs2044028 | C | G | -0.050 | 0.011 | 3.65E-06 | 21.486 |
| family | Coriobacteriaceae | rs67561917 | G | A | -0.071 | 0.015 | 5.39E-06 | 21.486 |
| family | Bifidobacteriaceae | rs2197579 | C | T | 0.055 | 0.012 | 2.72E-06 | 21.486 |
| family | Methanobacteriaceae | rs7635580 | G | A | -0.193 | 0.042 | 4.53E-06 | 21.474 |
| family | Bifidobacteriaceae | rs6728946 | C | G | 0.073 | 0.016 | 4.39E-06 | 21.472 |
| family | Erysipelotrichaceae | rs12357537 | T | C | -0.054 | 0.012 | 4.15E-06 | 21.470 |
| family | Oxalobacteraceae | rs58167057 | G | A | -0.131 | 0.028 | 4.03E-06 | 21.468 |
| family | Bifidobacteriaceae | rs2230437 | C | T | 0.055 | 0.012 | 5.16E-06 | 21.462 |
| family | Peptostreptococcaceae | rs4557228 | C | T | 0.058 | 0.012 | 3.39E-06 | 21.462 |
| family | Streptococcaceae | rs2952257 | T | C | -0.058 | 0.013 | 3.15E-06 | 21.459 |
| family | ClostridialesvadinBB60group | rs16944016 | A | T | 0.137 | 0.030 | 7.23E-06 | 21.457 |
| family | Pasteurellaceae | rs35510 | G | A | 0.123 | 0.026 | 4.02E-06 | 21.456 |
| family | Bifidobacteriaceae | rs1592 | A | C | 0.055 | 0.012 | 4.46E-06 | 21.453 |
| family | Streptococcaceae | rs9916518 | C | T | -0.051 | 0.011 | 3.67E-06 | 21.453 |
| family | Streptococcaceae | rs7818437 | T | C | -0.061 | 0.013 | 3.42E-06 | 21.449 |
| family | ClostridialesvadinBB60group | rs66714985 | C | A | 0.117 | 0.025 | 4.85E-06 | 21.446 |
| family | Victivallaceae | rs67832247 | T | C | -0.123 | 0.027 | 3.24E-06 | 21.440 |
| family | Bifidobacteriaceae | rs309159 | C | T | 0.073 | 0.016 | 3.51E-06 | 21.435 |
| family | ClostridialesvadinBB60group | rs2215602 | T | G | 0.074 | 0.016 | 3.39E-06 | 21.434 |
| family | Bifidobacteriaceae | rs1374291 | T | C | 0.055 | 0.012 | 2.73E-06 | 21.433 |
| family | Alcaligenaceae | rs9964679 | G | A | 0.054 | 0.012 | 4.47E-06 | 21.432 |
| family | Clostridiaceae1 | rs2795528 | A | G | -0.181 | 0.039 | 3.81E-06 | 21.432 |
| family | Rhodospirillaceae | rs7098347 | A | G | 0.073 | 0.016 | 3.06E-06 | 21.431 |
| family | Streptococcaceae | rs9904474 | T | G | -0.051 | 0.011 | 4.04E-06 | 21.428 |
| family | Bifidobacteriaceae | rs586964 | G | A | 0.075 | 0.016 | 4.90E-06 | 21.428 |
| family | Streptococcaceae | rs11248956 | T | C | 0.060 | 0.013 | 2.68E-06 | 21.427 |
| family | Porphyromonadaceae | rs1029811 | G | T | 0.067 | 0.015 | 2.91E-06 | 21.426 |
| family | Lactobacillaceae | rs77478751 | G | A | -0.219 | 0.047 | 5.96E-06 | 21.421 |
| family | Streptococcaceae | rs111923753 | C | T | -0.085 | 0.018 | 3.71E-06 | 21.420 |
| family | Prevotellaceae | rs111996655 | C | T | -0.075 | 0.016 | 6.02E-06 | 21.419 |
| family | Peptostreptococcaceae | rs57768785 | G | A | 0.058 | 0.012 | 3.44E-06 | 21.418 |
| family | Desulfovibrionaceae | rs112381107 | T | C | 0.211 | 0.046 | 2.82E-06 | 21.409 |
| family | Erysipelotrichaceae | rs9859260 | T | C | 0.049 | 0.011 | 3.63E-06 | 21.405 |
| family | Bifidobacteriaceae | rs62181700 | A | G | -0.060 | 0.013 | 4.12E-06 | 21.403 |
| family | Bifidobacteriaceae | rs72711368 | C | T | 0.057 | 0.012 | 4.75E-06 | 21.403 |
| family | Bifidobacteriaceae | rs56230617 | G | A | 0.073 | 0.016 | 2.30E-06 | 21.402 |
| family | ClostridialesvadinBB60group | rs9907155 | G | A | 0.137 | 0.030 | 7.69E-06 | 21.399 |
| family | Rikenellaceae | rs67281112 | C | G | 0.064 | 0.014 | 3.63E-06 | 21.397 |
| family | ClostridialesvadinBB60group | rs740646 | A | G | 0.124 | 0.027 | 9.11E-06 | 21.391 |
| family | Alcaligenaceae | rs6087811 | G | T | -0.093 | 0.020 | 3.37E-06 | 21.387 |
| family | Bifidobacteriaceae | rs73958638 | A | G | 0.112 | 0.024 | 2.36E-06 | 21.387 |
| family | FamilyXI | rs488164 | G | T | 0.118 | 0.026 | 4.80E-06 | 21.386 |
| family | Streptococcaceae | rs9989494 | G | A | -0.051 | 0.011 | 3.89E-06 | 21.385 |
| family | Bifidobacteriaceae | rs499483 | G | A | 0.075 | 0.016 | 4.99E-06 | 21.383 |
| family | Peptostreptococcaceae | rs7326261 | G | A | -0.065 | 0.014 | 4.75E-06 | 21.374 |
| family | Bifidobacteriaceae | rs4954191 | C | G | 0.055 | 0.012 | 2.34E-06 | 21.372 |
| family | Rikenellaceae | rs12787515 | G | A | -0.096 | 0.021 | 6.08E-06 | 21.372 |
| family | Bifidobacteriaceae | rs309158 | A | G | 0.071 | 0.015 | 4.45E-06 | 21.372 |
| family | Prevotellaceae | rs77380553 | G | A | -0.077 | 0.017 | 3.09E-06 | 21.371 |
| family | Veillonellaceae | rs7324328 | C | T | 0.094 | 0.020 | 3.79E-06 | 21.369 |
| family | FamilyXIII | rs7539202 | T | C | 0.059 | 0.013 | 5.07E-06 | 21.368 |
| family | Bifidobacteriaceae | rs520449 | T | C | -0.057 | 0.012 | 1.83E-06 | 21.366 |
| family | Enterobacteriaceae | rs2374343 | G | C | 0.058 | 0.013 | 4.93E-06 | 21.365 |
| family | Peptostreptococcaceae | rs1098907 | A | T | 0.052 | 0.011 | 3.88E-06 | 21.365 |
| family | Porphyromonadaceae | rs34701787 | C | T | 0.079 | 0.017 | 5.82E-06 | 21.361 |
| family | Streptococcaceae | rs2975649 | C | T | -0.058 | 0.013 | 3.44E-06 | 21.360 |
| family | Bifidobacteriaceae | rs1056465 | T | A | 0.056 | 0.012 | 7.13E-06 | 21.360 |
| family | Bifidobacteriaceae | rs59213715 | A | C | 0.075 | 0.016 | 4.30E-06 | 21.355 |
| family | Prevotellaceae | rs111795168 | C | T | -0.077 | 0.017 | 6.57E-06 | 21.352 |
| family | Lachnospiraceae | rs28578977 | A | G | -0.113 | 0.025 | 3.68E-06 | 21.349 |
| family | Lachnospiraceae | rs147922050 | C | G | 0.058 | 0.013 | 5.89E-06 | 21.348 |
| family | Victivallaceae | rs185249 | G | A | 0.119 | 0.026 | 4.42E-06 | 21.340 |
| family | Bifidobacteriaceae | rs34847072 | G | C | 0.056 | 0.012 | 6.71E-06 | 21.338 |
| family | Enterobacteriaceae | rs2374342 | A | C | 0.058 | 0.013 | 4.52E-06 | 21.338 |
| family | Bifidobacteriaceae | rs56302516 | C | T | 0.062 | 0.013 | 3.49E-06 | 21.336 |
| family | Erysipelotrichaceae | rs9877119 | G | A | 0.049 | 0.011 | 3.74E-06 | 21.332 |
| family | Desulfovibrionaceae | rs55640204 | A | G | -0.093 | 0.020 | 4.33E-06 | 21.327 |
| family | Bifidobacteriaceae | rs309120 | G | C | 0.072 | 0.016 | 3.59E-06 | 21.323 |
| family | Verrucomicrobiaceae | rs6832119 | A | G | 0.109 | 0.024 | 3.47E-06 | 21.320 |
| family | Streptococcaceae | rs114279530 | G | A | -0.083 | 0.018 | 2.89E-06 | 21.320 |
| family | FamilyXIII | rs3098182 | T | G | 0.051 | 0.011 | 4.04E-06 | 21.316 |
| family | Acidaminococcaceae | rs262812 | C | T | -0.066 | 0.014 | 3.25E-06 | 21.314 |
| family | FamilyXI | rs17379710 | C | T | -0.116 | 0.025 | 3.97E-06 | 21.308 |
| family | Pasteurellaceae | rs72756943 | A | G | 0.140 | 0.030 | 3.35E-06 | 21.308 |
| family | Peptostreptococcaceae | rs10805326 | A | G | 0.057 | 0.012 | 4.03E-06 | 21.306 |
| family | Streptococcaceae | rs2052119 | G | A | -0.051 | 0.011 | 3.97E-06 | 21.305 |
| family | FamilyXIII | rs13142827 | C | T | -0.063 | 0.014 | 2.69E-06 | 21.305 |
| family | Christensenellaceae | rs892686 | G | A | 0.051 | 0.011 | 4.12E-06 | 21.304 |
| family | Alcaligenaceae | rs113310015 | C | T | 0.070 | 0.015 | 3.54E-06 | 21.304 |
| family | Verrucomicrobiaceae | rs77055143 | C | T | 0.067 | 0.014 | 3.73E-06 | 21.303 |
| family | Peptococcaceae | rs61870296 | G | A | 0.134 | 0.029 | 3.20E-06 | 21.300 |
| family | Pasteurellaceae | rs35509 | A | G | 0.122 | 0.027 | 4.39E-06 | 21.298 |
| family | FamilyXI | rs10817437 | C | T | -0.152 | 0.033 | 5.06E-06 | 21.294 |
| family | Streptococcaceae | rs2460109 | C | T | -0.051 | 0.011 | 4.15E-06 | 21.292 |
| family | Streptococcaceae | rs4968752 | G | A | -0.051 | 0.011 | 4.19E-06 | 21.284 |
| family | Actinomycetaceae | rs6488662 | T | C | 0.081 | 0.018 | 4.08E-06 | 21.281 |
| family | Methanobacteriaceae | rs11123059 | A | G | -0.108 | 0.023 | 4.87E-06 | 21.279 |
| family | Streptococcaceae | rs2975647 | G | A | -0.058 | 0.013 | 3.53E-06 | 21.278 |
| family | Victivallaceae | rs60597389 | A | G | 0.138 | 0.030 | 9.10E-06 | 21.274 |
| family | Methanobacteriaceae | rs62241827 | T | G | -0.193 | 0.042 | 5.35E-06 | 21.274 |
| family | Defluviitaleaceae | rs2357087 | T | C | -0.104 | 0.023 | 9.71E-06 | 21.272 |
| family | Bifidobacteriaceae | rs73958614 | A | C | 0.093 | 0.020 | 6.78E-06 | 21.270 |
| family | Bifidobacteriaceae | rs309167 | C | T | 0.075 | 0.016 | 5.12E-06 | 21.266 |
| family | Bifidobacteriaceae | rs6711493 | A | G | 0.071 | 0.015 | 4.13E-06 | 21.265 |
| family | Bifidobacteriaceae | rs309166 | C | T | 0.072 | 0.016 | 4.11E-06 | 21.264 |
| family | Methanobacteriaceae | rs62241829 | C | T | -0.193 | 0.042 | 5.39E-06 | 21.263 |
| family | Streptococcaceae | rs11110070 | G | A | -0.083 | 0.018 | 3.80E-06 | 21.257 |
| family | Lachnospiraceae | rs4731702 | C | T | -0.048 | 0.011 | 3.96E-06 | 21.256 |
| family | Verrucomicrobiaceae | rs111862613 | C | T | 0.091 | 0.020 | 3.73E-06 | 21.255 |
| family | Bifidobacteriaceae | rs34453673 | G | C | 0.056 | 0.012 | 6.43E-06 | 21.255 |
| family | FamilyXI | rs34155035 | T | C | -0.139 | 0.030 | 4.02E-06 | 21.251 |
| family | Erysipelotrichaceae | rs8003149 | T | C | 0.054 | 0.012 | 4.08E-06 | 21.248 |
| family | Methanobacteriaceae | rs62241828 | A | G | -0.193 | 0.042 | 5.45E-06 | 21.243 |
| family | Victivallaceae | rs72960932 | G | A | -0.193 | 0.042 | 2.47E-06 | 21.242 |
| family | ClostridialesvadinBB60group | rs9889426 | A | C | 0.136 | 0.029 | 7.49E-06 | 21.239 |
| family | FamilyXIII | rs4514220 | G | A | 0.059 | 0.013 | 5.42E-06 | 21.236 |
| family | Alcaligenaceae | rs77984434 | C | A | 0.130 | 0.028 | 5.18E-06 | 21.229 |
| family | Verrucomicrobiaceae | rs11184341 | C | G | 0.066 | 0.014 | 4.14E-06 | 21.227 |
| family | Bifidobacteriaceae | rs12469411 | C | T | 0.055 | 0.012 | 2.81E-06 | 21.219 |
| family | Lachnospiraceae | rs56077123 | C | A | 0.055 | 0.012 | 3.05E-06 | 21.213 |
| family | Clostridiaceae1 | rs12186080 | A | G | 0.075 | 0.016 | 5.34E-06 | 21.212 |
| family | Actinomycetaceae | rs2110087 | G | A | 0.079 | 0.017 | 4.47E-06 | 21.211 |
| family | Verrucomicrobiaceae | rs12416161 | C | T | 0.076 | 0.017 | 3.51E-06 | 21.208 |
| family | Bifidobacteriaceae | rs12188164 | C | A | 0.056 | 0.012 | 6.17E-06 | 21.200 |
| family | Rhodospirillaceae | rs11599418 | T | C | 0.073 | 0.016 | 3.79E-06 | 21.200 |
| family | Bifidobacteriaceae | rs56346498 | C | T | 0.056 | 0.012 | 6.30E-06 | 21.198 |
| family | Bifidobacteriaceae | rs2839740 | G | T | 0.075 | 0.016 | 5.22E-06 | 21.182 |
| family | Lachnospiraceae | rs4545208 | T | C | -0.049 | 0.011 | 4.74E-06 | 21.175 |
| family | Methanobacteriaceae | rs55721046 | A | G | -0.191 | 0.041 | 6.06E-06 | 21.171 |
| family | Bifidobacteriaceae | rs12188678 | T | C | 0.056 | 0.012 | 6.26E-06 | 21.161 |
| family | Lactobacillaceae | rs768252 | G | T | -0.078 | 0.017 | 4.47E-06 | 21.159 |
| family | Desulfovibrionaceae | rs2838334 | A | G | 0.057 | 0.012 | 3.82E-06 | 21.157 |
| family | Pasteurellaceae | rs4822728 | C | T | 0.069 | 0.015 | 4.72E-06 | 21.156 |
| family | Oxalobacteraceae | rs934049 | A | G | 0.110 | 0.024 | 4.21E-06 | 21.152 |
| family | Prevotellaceae | rs11151191 | T | C | -0.079 | 0.017 | 4.02E-06 | 21.149 |
| family | Lactobacillaceae | rs59849860 | A | G | -0.101 | 0.022 | 5.96E-06 | 21.148 |
| family | Verrucomicrobiaceae | rs12498518 | C | T | 0.067 | 0.014 | 3.96E-06 | 21.144 |
| family | Desulfovibrionaceae | rs9928243 | A | C | -0.054 | 0.012 | 4.48E-06 | 21.142 |
| family | Peptostreptococcaceae | rs9318445 | C | T | -0.065 | 0.014 | 5.48E-06 | 21.142 |
| family | Victivallaceae | rs58593843 | G | A | -0.187 | 0.041 | 2.94E-06 | 21.141 |
| family | FamilyXIII | rs7076829 | A | T | -0.052 | 0.011 | 4.76E-06 | 21.140 |
| family | Streptococcaceae | rs7971435 | T | G | -0.084 | 0.018 | 4.57E-06 | 21.139 |
| family | Acidaminococcaceae | rs6427992 | C | G | -0.060 | 0.013 | 4.24E-06 | 21.138 |
| family | Rhodospirillaceae | rs4918686 | T | G | 0.072 | 0.016 | 4.16E-06 | 21.132 |
| family | Bifidobacteriaceae | rs72717432 | G | A | 0.056 | 0.012 | 6.69E-06 | 21.129 |
| family | Defluviitaleaceae | rs17051335 | T | C | -0.134 | 0.029 | 4.58E-06 | 21.129 |
| family | Pasteurellaceae | rs55676407 | A | G | 0.134 | 0.029 | 3.41E-06 | 21.127 |
| family | Alcaligenaceae | rs6969323 | C | A | -0.059 | 0.013 | 3.89E-06 | 21.127 |
| family | Streptococcaceae | rs7970539 | T | C | -0.084 | 0.018 | 4.39E-06 | 21.127 |
| family | ClostridialesvadinBB60group | rs7544734 | T | C | -0.064 | 0.014 | 4.75E-06 | 21.122 |
| family | Porphyromonadaceae | rs10811672 | A | T | -0.068 | 0.015 | 2.10E-06 | 21.117 |
| family | Porphyromonadaceae | rs2137100 | T | G | 0.056 | 0.012 | 5.74E-06 | 21.116 |
| family | Bacteroidaceae | rs68118234 | T | C | -0.077 | 0.017 | 5.65E-06 | 21.115 |
| family | Bifidobacteriaceae | rs309118 | G | C | 0.071 | 0.015 | 5.44E-06 | 21.109 |
| family | Streptococcaceae | rs2460110 | G | A | -0.051 | 0.011 | 4.19E-06 | 21.105 |
| family | Verrucomicrobiaceae | rs12501826 | G | C | 0.067 | 0.014 | 4.14E-06 | 21.097 |
| family | FamilyXIII | rs7674904 | G | C | -0.050 | 0.011 | 4.55E-06 | 21.095 |
| family | Bifidobacteriaceae | rs309165 | T | C | 0.070 | 0.015 | 5.00E-06 | 21.093 |
| family | Pasteurellaceae | rs73139353 | C | A | -0.223 | 0.048 | 8.71E-06 | 21.092 |
| family | Bifidobacteriaceae | rs7573555 | T | C | 0.073 | 0.016 | 5.12E-06 | 21.086 |
| family | Streptococcaceae | rs2975654 | G | A | -0.058 | 0.013 | 4.27E-06 | 21.086 |
| family | Bifidobacteriaceae | rs79954860 | A | T | 0.112 | 0.024 | 3.72E-06 | 21.085 |
| family | Victivallaceae | rs17017706 | G | T | -0.188 | 0.041 | 1.57E-06 | 21.084 |
| family | Streptococcaceae | rs2952258 | C | T | -0.058 | 0.013 | 3.98E-06 | 21.081 |
| family | Streptococcaceae | rs75088533 | C | T | -0.085 | 0.018 | 4.59E-06 | 21.078 |
| family | Bifidobacteriaceae | rs7600925 | A | G | 0.093 | 0.020 | 7.06E-06 | 21.077 |
| family | Lachnospiraceae | rs34533733 | G | C | -0.049 | 0.011 | 5.16E-06 | 21.076 |
| family | Bifidobacteriaceae | rs857444 | T | C | 0.055 | 0.012 | 3.82E-06 | 21.075 |
| family | Peptostreptococcaceae | rs77297228 | G | A | 0.159 | 0.035 | 6.02E-06 | 21.075 |
| family | Rhodospirillaceae | rs56752332 | G | T | -0.090 | 0.020 | 3.80E-06 | 21.074 |
| family | Veillonellaceae | rs11838712 | G | A | 0.094 | 0.020 | 4.57E-06 | 21.068 |
| family | BacteroidalesS24 | rs690117 | G | A | 0.081 | 0.018 | 4.62E-06 | 21.065 |
| family | Rhodospirillaceae | rs16855133 | A | G | 0.115 | 0.025 | 3.93E-06 | 21.065 |
| family | Streptococcaceae | rs77628647 | G | A | -0.085 | 0.018 | 4.64E-06 | 21.065 |
| family | BacteroidalesS24 | rs697400 | A | G | 0.076 | 0.017 | 7.70E-06 | 21.063 |
| family | Acidaminococcaceae | rs3000667 | G | A | -0.064 | 0.014 | 4.37E-06 | 21.063 |
| family | Lachnospiraceae | rs62078074 | A | G | 0.054 | 0.012 | 4.05E-06 | 21.060 |
| family | Bifidobacteriaceae | rs3098102 | C | T | 0.072 | 0.016 | 4.70E-06 | 21.060 |
| family | Rhodospirillaceae | rs71498364 | C | T | 0.073 | 0.016 | 4.02E-06 | 21.059 |
| family | Bifidobacteriaceae | rs1446523 | G | A | 0.055 | 0.012 | 2.94E-06 | 21.057 |
| family | Porphyromonadaceae | rs2675423 | C | A | 0.056 | 0.012 | 5.99E-06 | 21.053 |
| family | Enterobacteriaceae | rs6069871 | G | A | 0.060 | 0.013 | 4.49E-06 | 21.049 |
| family | Enterobacteriaceae | rs80319214 | G | C | 0.099 | 0.022 | 6.95E-06 | 21.048 |
| family | Peptostreptococcaceae | rs842523 | T | C | 0.052 | 0.011 | 4.56E-06 | 21.047 |
| family | Bifidobacteriaceae | rs660002 | C | T | 0.070 | 0.015 | 4.93E-06 | 21.046 |
| family | Methanobacteriaceae | rs12303159 | C | A | -0.223 | 0.049 | 8.56E-06 | 21.045 |
| family | Lachnospiraceae | rs10980809 | T | C | -0.049 | 0.011 | 4.91E-06 | 21.045 |
| family | Erysipelotrichaceae | rs17530232 | G | A | 0.103 | 0.022 | 2.79E-06 | 21.042 |
| family | Bifidobacteriaceae | rs72717407 | G | A | 0.056 | 0.012 | 6.68E-06 | 21.042 |
| family | Rikenellaceae | rs7242694 | T | C | -0.062 | 0.013 | 5.81E-06 | 21.038 |
| family | BacteroidalesS24 | rs12148053 | T | C | 0.080 | 0.017 | 5.01E-06 | 21.036 |
| family | Clostridiaceae1 | rs941434 | C | A | -0.179 | 0.039 | 4.81E-06 | 21.032 |
| family | Bifidobacteriaceae | rs1057031 | G | A | 0.073 | 0.016 | 5.56E-06 | 21.027 |
| family | Bifidobacteriaceae | rs1960171 | C | T | 0.060 | 0.013 | 5.82E-06 | 21.026 |
| family | Veillonellaceae | rs2175069 | G | A | -0.053 | 0.011 | 4.64E-06 | 21.022 |
| family | Alcaligenaceae | rs7302582 | C | G | -0.107 | 0.023 | 3.34E-06 | 21.020 |
| family | ClostridialesvadinBB60group | rs10179973 | C | A | 0.064 | 0.014 | 4.90E-06 | 21.019 |
| family | Bifidobacteriaceae | rs79106992 | C | T | -0.084 | 0.018 | 4.83E-06 | 21.017 |
| family | Veillonellaceae | rs1442060 | A | G | -0.051 | 0.011 | 4.51E-06 | 21.017 |
| family | Veillonellaceae | rs12186441 | A | G | 0.208 | 0.045 | 4.53E-06 | 21.010 |
| family | Bifidobacteriaceae | rs7606267 | T | C | 0.073 | 0.016 | 5.37E-06 | 21.009 |
| family | Bifidobacteriaceae | rs189135960 | C | T | 0.093 | 0.020 | 8.88E-06 | 21.008 |
| family | BacteroidalesS24 | rs11135366 | G | C | 0.084 | 0.018 | 8.78E-06 | 21.007 |
| family | Alcaligenaceae | rs7991472 | T | C | -0.051 | 0.011 | 4.23E-06 | 21.005 |
| family | Bifidobacteriaceae | rs12986776 | G | C | 0.056 | 0.012 | 5.91E-06 | 20.999 |
| family | Bifidobacteriaceae | rs72717436 | C | A | 0.055 | 0.012 | 7.43E-06 | 20.995 |
| family | Veillonellaceae | rs4797169 | C | T | 0.059 | 0.013 | 4.49E-06 | 20.991 |
| family | Rhodospirillaceae | rs16855116 | C | T | 0.115 | 0.025 | 4.85E-06 | 20.989 |
| family | Bifidobacteriaceae | rs75753154 | T | C | 0.073 | 0.016 | 5.47E-06 | 20.982 |
| family | Rhodospirillaceae | rs11818728 | C | T | 0.073 | 0.016 | 4.12E-06 | 20.979 |
| family | Victivallaceae | rs12806744 | G | A | -0.130 | 0.028 | 6.29E-06 | 20.978 |
| family | FamilyXI | rs34601953 | T | A | -0.138 | 0.030 | 4.62E-06 | 20.977 |
| family | Acidaminococcaceae | rs45497800 | C | T | -0.118 | 0.026 | 5.86E-06 | 20.975 |
| family | Oxalobacteraceae | rs7993559 | A | C | 0.092 | 0.020 | 5.04E-06 | 20.975 |
| family | Oxalobacteraceae | rs12360820 | C | A | 0.120 | 0.026 | 4.92E-06 | 20.974 |
| family | Verrucomicrobiaceae | rs6832927 | C | A | 0.108 | 0.024 | 4.27E-06 | 20.973 |
| family | Bifidobacteriaceae | rs3099428 | C | G | 0.072 | 0.016 | 4.96E-06 | 20.972 |
| family | Lachnospiraceae | rs959845 | T | C | -0.049 | 0.011 | 5.17E-06 | 20.971 |
| family | Victivallaceae | rs11671100 | C | A | -0.160 | 0.035 | 4.08E-06 | 20.970 |
| family | Streptococcaceae | rs6601443 | G | A | -0.062 | 0.014 | 3.76E-06 | 20.969 |
| family | Alcaligenaceae | rs13385989 | A | C | 0.063 | 0.014 | 4.84E-06 | 20.968 |
| family | Rikenellaceae | rs8130320 | G | A | -0.049 | 0.011 | 4.73E-06 | 20.968 |
| family | Bifidobacteriaceae | rs2305165 | A | C | 0.093 | 0.020 | 8.48E-06 | 20.966 |
| family | Ruminococcaceae | rs10093275 | T | C | 0.053 | 0.012 | 5.35E-06 | 20.960 |
| family | Alcaligenaceae | rs7330191 | C | T | -0.051 | 0.011 | 4.34E-06 | 20.959 |
| family | Bifidobacteriaceae | rs540489 | G | T | -0.063 | 0.014 | 5.37E-06 | 20.957 |
| family | FamilyXIII | rs497142 | T | G | 0.058 | 0.013 | 6.27E-06 | 20.954 |
| family | ClostridialesvadinBB60group | rs7215860 | G | A | 0.136 | 0.030 | 8.79E-06 | 20.954 |
| family | Oxalobacteraceae | rs111966731 | C | T | 0.204 | 0.045 | 4.56E-06 | 20.952 |
| family | Porphyromonadaceae | rs10858364 | T | G | 0.055 | 0.012 | 4.31E-06 | 20.951 |
| family | Alcaligenaceae | rs1153990 | G | A | -0.059 | 0.013 | 5.97E-06 | 20.950 |
| family | Rhodospirillaceae | rs34142542 | C | T | 0.073 | 0.016 | 4.33E-06 | 20.948 |
| family | Bifidobacteriaceae | rs614193 | A | T | 0.072 | 0.016 | 5.14E-06 | 20.947 |
| family | FamilyXIII | rs1999289 | T | A | -0.064 | 0.014 | 2.54E-06 | 20.944 |
| family | Peptostreptococcaceae | rs12640117 | C | T | 0.057 | 0.012 | 4.40E-06 | 20.942 |
| family | ClostridialesvadinBB60group | rs1860762 | T | C | 0.073 | 0.016 | 4.66E-06 | 20.941 |
| family | Verrucomicrobiaceae | rs1605923 | T | G | 0.066 | 0.014 | 4.64E-06 | 20.941 |
| family | Bifidobacteriaceae | rs72717411 | C | G | 0.055 | 0.012 | 7.15E-06 | 20.939 |
| family | Methanobacteriaceae | rs62241826 | T | C | -0.190 | 0.041 | 6.95E-06 | 20.937 |
| family | Prevotellaceae | rs7252711 | G | A | -0.074 | 0.016 | 5.57E-06 | 20.934 |
| family | Victivallaceae | rs56311698 | G | T | -0.195 | 0.043 | 3.96E-06 | 20.930 |
| family | Bifidobacteriaceae | rs112758679 | A | C | 0.073 | 0.016 | 5.42E-06 | 20.930 |
| family | Verrucomicrobiaceae | rs11724791 | G | A | 0.066 | 0.014 | 4.92E-06 | 20.928 |
| family | Rhodospirillaceae | rs4822789 | C | G | 0.073 | 0.016 | 7.01E-06 | 20.927 |
| family | Bifidobacteriaceae | rs13410301 | T | C | 0.054 | 0.012 | 5.79E-06 | 20.925 |
| family | Enterobacteriaceae | rs111229068 | T | A | 0.111 | 0.024 | 3.65E-06 | 20.921 |
| family | Bifidobacteriaceae | rs766271 | C | T | 0.054 | 0.012 | 5.98E-06 | 20.918 |
| family | ClostridialesvadinBB60group | rs12997335 | T | A | 0.073 | 0.016 | 4.62E-06 | 20.918 |
| family | Rhodospirillaceae | rs17793087 | T | C | 0.073 | 0.016 | 4.38E-06 | 20.916 |
| family | Streptococcaceae | rs8079516 | A | G | -0.051 | 0.011 | 5.10E-06 | 20.915 |
| family | Alcaligenaceae | rs67553427 | T | A | 0.069 | 0.015 | 4.35E-06 | 20.910 |
| family | Erysipelotrichaceae | rs3804140 | C | T | 0.049 | 0.011 | 4.68E-06 | 20.907 |
| family | Bifidobacteriaceae | rs67665078 | G | A | 0.053 | 0.012 | 4.93E-06 | 20.906 |
| family | Lactobacillaceae | rs1434764 | G | T | -0.078 | 0.017 | 5.11E-06 | 20.905 |
| family | Bifidobacteriaceae | rs55932239 | G | A | 0.055 | 0.012 | 7.30E-06 | 20.904 |
| family | Enterobacteriaceae | rs7271805 | C | A | 0.059 | 0.013 | 4.77E-06 | 20.898 |
| family | Bifidobacteriaceae | rs75128007 | A | G | 0.062 | 0.014 | 6.87E-06 | 20.892 |
| family | Rhodospirillaceae | rs1549633 | C | A | 0.100 | 0.022 | 4.70E-06 | 20.891 |
| family | Streptococcaceae | rs77558518 | G | A | -0.104 | 0.023 | 3.72E-06 | 20.891 |
| family | BacteroidalesS24 | rs28494831 | C | T | 0.080 | 0.017 | 4.72E-06 | 20.889 |
| family | Verrucomicrobiaceae | rs59317921 | C | T | 0.063 | 0.014 | 4.55E-06 | 20.888 |
| family | Rhodospirillaceae | rs62255666 | G | A | -0.082 | 0.018 | 5.50E-06 | 20.886 |
| family | Rhodospirillaceae | rs61933850 | A | G | 0.165 | 0.036 | 7.23E-06 | 20.883 |
| family | FamilyXI | rs3928987 | G | A | -0.115 | 0.025 | 5.28E-06 | 20.883 |
| family | ClostridialesvadinBB60group | rs10904722 | T | C | -0.067 | 0.015 | 5.05E-06 | 20.882 |
| family | Bifidobacteriaceae | rs895285 | A | G | 0.074 | 0.016 | 5.52E-06 | 20.882 |
| family | Verrucomicrobiaceae | rs6668273 | G | A | 0.065 | 0.014 | 5.08E-06 | 20.871 |
| family | Bifidobacteriaceae | rs6430549 | A | G | 0.055 | 0.012 | 3.22E-06 | 20.869 |
| family | Peptostreptococcaceae | rs2328996 | T | C | -0.064 | 0.014 | 6.32E-06 | 20.864 |
| family | Streptococcaceae | rs2370083 | T | G | -0.084 | 0.018 | 4.26E-06 | 20.862 |
| family | Ruminococcaceae | rs1612733 | C | T | 0.109 | 0.024 | 4.22E-06 | 20.862 |
| family | Bifidobacteriaceae | rs2053726 | A | G | 0.055 | 0.012 | 3.21E-06 | 20.847 |
| family | Peptostreptococcaceae | rs11149051 | C | T | -0.065 | 0.014 | 5.61E-06 | 20.846 |
| family | Rikenellaceae | rs59663348 | A | G | 0.057 | 0.013 | 6.12E-06 | 20.845 |
| family | Rhodospirillaceae | rs3768870 | A | C | 0.091 | 0.020 | 6.99E-06 | 20.841 |
| family | ClostridialesvadinBB60group | rs7211063 | C | T | 0.135 | 0.030 | 9.90E-06 | 20.839 |
| family | Victivallaceae | rs2434250 | A | G | 0.114 | 0.025 | 6.51E-06 | 20.832 |
| family | Bifidobacteriaceae | rs4954275 | C | T | 0.092 | 0.020 | 8.43E-06 | 20.826 |
| family | Bifidobacteriaceae | rs115554641 | C | G | 0.055 | 0.012 | 8.60E-06 | 20.824 |
| family | Alcaligenaceae | rs368042645 | G | A | 0.069 | 0.015 | 4.71E-06 | 20.820 |
| family | Acidaminococcaceae | rs3012352 | T | C | -0.064 | 0.014 | 4.76E-06 | 20.819 |
| family | ClostridialesvadinBB60group | rs78285863 | C | A | 0.073 | 0.016 | 4.83E-06 | 20.818 |
| family | Bifidobacteriaceae | rs56182240 | C | T | 0.055 | 0.012 | 7.67E-06 | 20.818 |
| family | Christensenellaceae | rs5763231 | T | C | -0.068 | 0.015 | 3.72E-06 | 20.815 |
| family | Rhodospirillaceae | rs4278423 | C | T | 0.108 | 0.024 | 3.12E-06 | 20.809 |
| family | Streptococcaceae | rs6806351 | C | T | -0.062 | 0.014 | 6.94E-06 | 20.808 |
| family | Streptococcaceae | rs2952189 | C | T | -0.070 | 0.015 | 4.09E-06 | 20.808 |
| family | Bifidobacteriaceae | rs309142 | T | C | 0.070 | 0.015 | 5.19E-06 | 20.807 |
| family | Lachnospiraceae | rs13241538 | G | C | -0.048 | 0.011 | 5.19E-06 | 20.801 |
| family | ClostridialesvadinBB60group | rs9914051 | G | A | 0.135 | 0.030 | 9.77E-06 | 20.798 |
| family | Pasteurellaceae | rs731534 | G | C | -0.099 | 0.022 | 9.46E-06 | 20.798 |
| family | Bifidobacteriaceae | rs3099427 | A | G | 0.071 | 0.016 | 5.48E-06 | 20.797 |
| family | Peptostreptococcaceae | rs1351267 | C | T | 0.051 | 0.011 | 5.20E-06 | 20.797 |
| family | Victivallaceae | rs12703879 | G | A | 0.117 | 0.026 | 4.31E-06 | 20.794 |
| family | Verrucomicrobiaceae | rs72663787 | G | A | 0.066 | 0.015 | 5.06E-06 | 20.793 |
| family | Streptococcaceae | rs2975653 | G | A | -0.057 | 0.013 | 4.62E-06 | 20.791 |
| family | Christensenellaceae | rs9608766 | C | T | -0.068 | 0.015 | 3.33E-06 | 20.791 |
| family | Peptococcaceae | rs12144792 | T | C | 0.064 | 0.014 | 5.82E-06 | 20.788 |
| family | FamilyXIII | rs34583378 | G | T | -0.068 | 0.015 | 5.00E-06 | 20.785 |
| family | Bifidobacteriaceae | rs113974791 | T | G | 0.072 | 0.016 | 5.92E-06 | 20.784 |
| family | Actinomycetaceae | rs6488660 | C | T | 0.080 | 0.017 | 5.27E-06 | 20.783 |
| family | ClostridialesvadinBB60group | rs7215412 | G | A | 0.134 | 0.029 | 9.10E-06 | 20.781 |
| family | Alcaligenaceae | rs1217467 | C | T | -0.059 | 0.013 | 8.05E-06 | 20.775 |
| family | Verrucomicrobiaceae | rs113653192 | C | T | 0.108 | 0.024 | 4.72E-06 | 20.774 |
| family | FamilyXIII | rs34460632 | G | A | -0.062 | 0.014 | 5.61E-06 | 20.763 |
| family | Streptococcaceae | rs2927291 | G | A | -0.050 | 0.011 | 5.00E-06 | 20.763 |
| family | BacteroidalesS24 | rs11135367 | T | C | 0.084 | 0.018 | 9.78E-06 | 20.761 |
| family | Bacteroidaceae | rs480694 | T | A | -0.059 | 0.013 | 6.16E-06 | 20.760 |
| family | Bifidobacteriaceae | rs6889494 | C | T | 0.055 | 0.012 | 7.91E-06 | 20.759 |
| family | FamilyXIII | rs34025726 | T | A | -0.062 | 0.014 | 5.56E-06 | 20.757 |
| family | Bifidobacteriaceae | rs479980 | A | C | -0.056 | 0.012 | 4.29E-06 | 20.754 |
| family | Actinomycetaceae | rs11055861 | A | G | 0.079 | 0.017 | 5.38E-06 | 20.752 |
| family | Actinomycetaceae | rs6488659 | T | C | 0.079 | 0.017 | 5.36E-06 | 20.751 |
| family | ClostridialesvadinBB60group | rs12614624 | T | C | 0.073 | 0.016 | 5.15E-06 | 20.751 |
| family | Erysipelotrichaceae | rs3804139 | C | T | 0.049 | 0.011 | 5.08E-06 | 20.748 |
| family | Bifidobacteriaceae | rs56784995 | T | C | 0.092 | 0.020 | 8.31E-06 | 20.748 |
| family | Desulfovibrionaceae | rs35236973 | T | A | -0.053 | 0.012 | 6.22E-06 | 20.746 |
| family | Verrucomicrobiaceae | rs3995795 | T | C | 0.064 | 0.014 | 5.03E-06 | 20.745 |
| family | Actinomycetaceae | rs11055860 | A | G | 0.079 | 0.017 | 5.40E-06 | 20.744 |
| family | Oxalobacteraceae | rs36057338 | T | G | 0.182 | 0.040 | 6.26E-06 | 20.743 |
| family | Bifidobacteriaceae | rs3814358 | A | C | 0.054 | 0.012 | 3.52E-06 | 20.741 |
| family | Streptococcaceae | rs2952184 | T | C | -0.070 | 0.015 | 4.31E-06 | 20.736 |
| family | Streptococcaceae | rs4968638 | C | T | -0.050 | 0.011 | 5.39E-06 | 20.736 |
| family | Bifidobacteriaceae | rs72717415 | G | A | 0.055 | 0.012 | 7.98E-06 | 20.733 |
| family | Prevotellaceae | rs74999593 | G | A | -0.075 | 0.017 | 8.86E-06 | 20.732 |
| family | Pasteurellaceae | rs62568866 | A | T | -0.118 | 0.026 | 7.23E-06 | 20.730 |
| family | Peptostreptococcaceae | rs6721459 | G | A | 0.051 | 0.011 | 5.08E-06 | 20.730 |
| family | Ruminococcaceae | rs4347804 | G | A | 0.158 | 0.035 | 3.94E-06 | 20.725 |
| family | Lactobacillaceae | rs12542112 | T | C | -0.078 | 0.017 | 5.38E-06 | 20.725 |
| family | Coriobacteriaceae | rs80046645 | G | C | 0.256 | 0.056 | 4.66E-06 | 20.724 |
| family | Rhodospirillaceae | rs11598143 | T | C | 0.071 | 0.016 | 4.65E-06 | 20.723 |
| family | Erysipelotrichaceae | rs4078432 | T | C | -0.061 | 0.013 | 4.23E-06 | 20.723 |
| family | Methanobacteriaceae | rs62240390 | C | G | -0.189 | 0.042 | 6.79E-06 | 20.722 |
| family | Methanobacteriaceae | rs73248946 | A | G | -0.222 | 0.049 | 9.83E-06 | 20.722 |
| family | Actinomycetaceae | rs7488453 | A | G | 0.079 | 0.017 | 5.45E-06 | 20.721 |
| family | FamilyXIII | rs6851324 | C | T | -0.050 | 0.011 | 5.61E-06 | 20.720 |
| family | Peptostreptococcaceae | rs12645967 | G | C | 0.056 | 0.012 | 4.97E-06 | 20.718 |
| family | Defluviitaleaceae | rs7664875 | G | A | -0.133 | 0.029 | 5.48E-06 | 20.717 |
| family | Streptococcaceae | rs6504149 | G | C | -0.050 | 0.011 | 5.08E-06 | 20.714 |
| family | Actinomycetaceae | rs2889192 | T | G | 0.089 | 0.020 | 3.64E-06 | 20.714 |
| family | Bifidobacteriaceae | rs7593370 | C | T | 0.054 | 0.012 | 3.60E-06 | 20.708 |
| family | Lachnospiraceae | rs2159863 | G | A | -0.059 | 0.013 | 3.70E-06 | 20.706 |
| family | Rhodospirillaceae | rs2086613 | G | A | -0.089 | 0.020 | 4.73E-06 | 20.704 |
| family | Bifidobacteriaceae | rs72717440 | A | T | 0.055 | 0.012 | 8.33E-06 | 20.704 |
| family | Oxalobacteraceae | rs73733961 | T | C | -0.128 | 0.028 | 5.86E-06 | 20.700 |
| family | Actinomycetaceae | rs114313061 | A | C | 0.080 | 0.018 | 4.89E-06 | 20.700 |
| family | Alcaligenaceae | rs7317710 | T | C | 0.050 | 0.011 | 5.82E-06 | 20.697 |
| family | Lachnospiraceae | rs13005175 | G | A | 0.099 | 0.022 | 8.37E-06 | 20.694 |
| family | FamilyXIII | rs4336803 | G | T | -0.064 | 0.014 | 7.21E-06 | 20.679 |
| family | Acidaminococcaceae | rs6701725 | G | A | -0.059 | 0.013 | 5.31E-06 | 20.679 |
| family | Actinomycetaceae | rs2377095 | C | A | 0.079 | 0.017 | 5.58E-06 | 20.679 |
| family | Acidaminococcaceae | rs1109873 | G | A | -0.064 | 0.014 | 5.17E-06 | 20.678 |
| family | Rhodospirillaceae | rs35982688 | T | C | 0.072 | 0.016 | 4.68E-06 | 20.678 |
| family | Bifidobacteriaceae | rs16832162 | T | A | 0.072 | 0.016 | 6.39E-06 | 20.677 |
| family | Actinomycetaceae | rs6488665 | T | C | 0.079 | 0.017 | 5.63E-06 | 20.676 |
| family | Lactobacillaceae | rs6024243 | T | A | -0.078 | 0.017 | 5.98E-06 | 20.676 |
| family | Erysipelotrichaceae | rs34221479 | C | T | 0.049 | 0.011 | 5.79E-06 | 20.675 |
| family | Rikenellaceae | rs7994129 | G | A | 0.075 | 0.017 | 3.63E-06 | 20.670 |
| family | Bifidobacteriaceae | rs11739543 | G | A | 0.055 | 0.012 | 8.63E-06 | 20.670 |
| family | Streptococcaceae | rs11720390 | A | G | 0.103 | 0.023 | 7.24E-06 | 20.670 |
| family | Methanobacteriaceae | rs56131665 | A | G | 0.179 | 0.039 | 6.18E-06 | 20.669 |
| family | Clostridiaceae1 | rs2817172 | T | C | 0.056 | 0.012 | 5.27E-06 | 20.668 |
| family | Alcaligenaceae | rs78428620 | C | T | -0.093 | 0.021 | 5.12E-06 | 20.667 |
| family | Bifidobacteriaceae | rs3087343 | T | G | 0.072 | 0.016 | 6.64E-06 | 20.663 |
| family | Victivallaceae | rs13009241 | G | A | 0.118 | 0.026 | 6.20E-06 | 20.659 |
| family | Porphyromonadaceae | rs2851493 | G | T | 0.066 | 0.015 | 4.84E-06 | 20.659 |
| family | Streptococcaceae | rs1518772 | G | A | -0.050 | 0.011 | 6.76E-06 | 20.656 |
| family | ClostridialesvadinBB60group | rs7214077 | G | A | 0.134 | 0.029 | 9.44E-06 | 20.655 |
| family | Streptococcaceae | rs11925530 | A | C | 0.103 | 0.023 | 7.18E-06 | 20.655 |
| family | Coriobacteriaceae | rs1816223 | G | A | -0.059 | 0.013 | 4.84E-06 | 20.652 |
| family | Christensenellaceae | rs12657403 | G | A | 0.078 | 0.017 | 5.59E-06 | 20.649 |
| family | Erysipelotrichaceae | rs9990392 | T | C | 0.049 | 0.011 | 4.94E-06 | 20.647 |
| family | Peptococcaceae | rs9603802 | C | G | -0.081 | 0.018 | 5.04E-06 | 20.646 |
| family | Bacteroidaceae | rs66710942 | T | C | 0.049 | 0.011 | 5.86E-06 | 20.644 |
| family | Bifidobacteriaceae | rs3112496 | C | T | 0.071 | 0.016 | 5.05E-06 | 20.643 |
| family | Bifidobacteriaceae | rs4954555 | T | C | 0.107 | 0.023 | 3.48E-06 | 20.639 |
| family | Bifidobacteriaceae | rs7569447 | A | C | 0.077 | 0.017 | 2.95E-06 | 20.635 |
| family | Desulfovibrionaceae | rs3935584 | T | C | -0.053 | 0.012 | 6.78E-06 | 20.635 |
| family | Clostridiaceae1 | rs12341505 | A | G | 0.081 | 0.018 | 4.54E-06 | 20.627 |
| family | Bifidobacteriaceae | rs11740891 | A | G | 0.054 | 0.012 | 6.11E-06 | 20.625 |
| family | Rikenellaceae | rs4306975 | G | T | 0.053 | 0.012 | 6.36E-06 | 20.624 |
| family | Bifidobacteriaceae | rs11678502 | T | C | 0.055 | 0.012 | 6.90E-06 | 20.618 |
| family | Bifidobacteriaceae | rs78364332 | T | C | 0.072 | 0.016 | 6.82E-06 | 20.617 |
| family | Bifidobacteriaceae | rs309171 | G | C | 0.072 | 0.016 | 6.48E-06 | 20.613 |
| family | Peptostreptococcaceae | rs12986312 | G | T | 0.057 | 0.013 | 5.77E-06 | 20.613 |
| family | Defluviitaleaceae | rs4344384 | T | G | 0.071 | 0.016 | 5.86E-06 | 20.612 |
| family | Erysipelotrichaceae | rs11146160 | A | G | -0.052 | 0.011 | 6.47E-06 | 20.611 |
| family | Prevotellaceae | rs6087027 | T | C | -0.053 | 0.012 | 5.71E-06 | 20.608 |
| family | Actinomycetaceae | rs4073240 | A | G | 0.075 | 0.016 | 6.05E-06 | 20.605 |
| family | Ruminococcaceae | rs11690053 | T | C | 0.160 | 0.035 | 3.76E-06 | 20.602 |
| family | Defluviitaleaceae | rs7677780 | G | T | -0.133 | 0.029 | 6.32E-06 | 20.601 |
| family | Christensenellaceae | rs12380890 | G | A | -0.050 | 0.011 | 5.78E-06 | 20.597 |
| family | Rikenellaceae | rs2836934 | A | C | -0.048 | 0.011 | 5.76E-06 | 20.590 |
| family | FamilyXIII | rs67320257 | C | A | -0.062 | 0.014 | 5.97E-06 | 20.589 |
| family | Peptococcaceae | rs114332351 | A | G | -0.136 | 0.030 | 6.60E-06 | 20.571 |
| family | Porphyromonadaceae | rs12698860 | G | A | 0.077 | 0.017 | 7.02E-06 | 20.563 |
| family | Bifidobacteriaceae | rs12472293 | T | C | 0.072 | 0.016 | 6.61E-06 | 20.561 |
| family | BacteroidalesS24 | rs1949158 | C | T | 0.079 | 0.017 | 6.64E-06 | 20.559 |
| family | Rhodospirillaceae | rs6759037 | G | A | 0.114 | 0.025 | 6.03E-06 | 20.558 |
| family | FamilyXIII | rs6693896 | G | A | -0.064 | 0.014 | 7.58E-06 | 20.553 |
| family | BacteroidalesS24 | rs17346776 | T | G | 0.079 | 0.017 | 6.47E-06 | 20.551 |
| family | Alcaligenaceae | rs9527726 | A | G | -0.050 | 0.011 | 5.12E-06 | 20.550 |
| family | Rhodospirillaceae | rs11596757 | C | G | 0.072 | 0.016 | 5.66E-06 | 20.546 |
| family | Bifidobacteriaceae | rs72717414 | C | G | 0.055 | 0.012 | 8.75E-06 | 20.544 |
| family | Bacteroidaceae | rs6015624 | A | G | 0.075 | 0.017 | 5.21E-06 | 20.540 |
| family | Verrucomicrobiaceae | rs72663782 | G | C | 0.066 | 0.014 | 5.38E-06 | 20.540 |
| family | Peptostreptococcaceae | rs10208182 | A | G | 0.051 | 0.011 | 5.50E-06 | 20.539 |
| family | Victivallaceae | rs2291840 | C | A | -0.133 | 0.029 | 6.79E-06 | 20.536 |
| family | Pasteurellaceae | rs7270573 | G | C | -0.068 | 0.015 | 5.58E-06 | 20.534 |
| family | Porphyromonadaceae | rs2675416 | T | A | 0.055 | 0.012 | 8.01E-06 | 20.533 |
| family | Pasteurellaceae | rs7115142 | A | G | -0.068 | 0.015 | 5.97E-06 | 20.533 |
| family | Oxalobacteraceae | rs62435498 | A | C | 0.182 | 0.040 | 7.46E-06 | 20.531 |
| family | Rhodospirillaceae | rs12632655 | G | A | -0.089 | 0.020 | 5.17E-06 | 20.531 |
| family | Rikenellaceae | rs2833282 | A | G | 0.071 | 0.016 | 4.31E-06 | 20.526 |
| family | Victivallaceae | rs41121 | T | G | 0.117 | 0.026 | 7.53E-06 | 20.520 |
| family | Bifidobacteriaceae | rs861392 | T | C | 0.055 | 0.012 | 5.21E-06 | 20.520 |
| family | Bifidobacteriaceae | rs842356 | A | G | 0.074 | 0.016 | 5.09E-06 | 20.519 |
| family | Erysipelotrichaceae | rs12258727 | T | C | -0.052 | 0.011 | 6.86E-06 | 20.518 |
| family | Bifidobacteriaceae | rs1992764 | C | T | 0.054 | 0.012 | 5.09E-06 | 20.515 |
| family | Ruminococcaceae | rs2426816 | A | T | -0.048 | 0.011 | 6.04E-06 | 20.514 |
| family | Streptococcaceae | rs1601915 | C | T | -0.082 | 0.018 | 5.74E-06 | 20.507 |
| family | ClostridialesvadinBB60group | rs12997748 | T | C | 0.072 | 0.016 | 5.68E-06 | 20.507 |
| family | Enterobacteriaceae | rs61973590 | G | C | -0.061 | 0.013 | 8.54E-06 | 20.504 |
| family | FamilyXI | rs6025153 | C | G | 0.119 | 0.026 | 7.07E-06 | 20.502 |
| family | Rikenellaceae | rs36021379 | G | A | -0.066 | 0.014 | 7.20E-06 | 20.496 |
| family | Porphyromonadaceae | rs210602 | G | C | 0.063 | 0.014 | 8.72E-06 | 20.493 |
| family | Bifidobacteriaceae | rs80261410 | T | C | 0.062 | 0.014 | 8.48E-06 | 20.492 |
| family | Lactobacillaceae | rs6092148 | T | G | -0.077 | 0.017 | 6.66E-06 | 20.490 |
| family | Streptococcaceae | rs4968747 | G | T | -0.050 | 0.011 | 6.05E-06 | 20.489 |
| family | Verrucomicrobiaceae | rs72663756 | C | T | 0.067 | 0.015 | 5.12E-06 | 20.489 |
| family | Oxalobacteraceae | rs12509763 | G | C | -0.163 | 0.036 | 8.05E-06 | 20.487 |
| family | Prevotellaceae | rs3758087 | C | T | 0.056 | 0.012 | 8.61E-06 | 20.485 |
| family | Rhodospirillaceae | rs1035406 | A | G | -0.114 | 0.025 | 5.84E-06 | 20.485 |
| family | Erysipelotrichaceae | rs921306 | C | T | 0.049 | 0.011 | 7.20E-06 | 20.481 |
| family | Porphyromonadaceae | rs9580476 | T | C | -0.110 | 0.024 | 4.11E-06 | 20.479 |
| family | Streptococcaceae | rs241062 | A | T | -0.050 | 0.011 | 6.40E-06 | 20.478 |
| family | Prevotellaceae | rs926496 | T | C | -0.053 | 0.012 | 6.46E-06 | 20.475 |
| family | Bifidobacteriaceae | rs2090660 | C | T | 0.062 | 0.014 | 7.78E-06 | 20.475 |
| family | Enterobacteriaceae | rs62210022 | C | T | 0.070 | 0.016 | 5.30E-06 | 20.472 |
| family | Porphyromonadaceae | rs2675420 | G | A | 0.055 | 0.012 | 8.04E-06 | 20.471 |
| family | Desulfovibrionaceae | rs72821212 | G | C | -0.092 | 0.020 | 6.60E-06 | 20.467 |
| family | Actinomycetaceae | rs17072071 | A | C | -0.112 | 0.025 | 7.13E-06 | 20.466 |
| family | Ruminococcaceae | rs10172096 | G | A | 0.160 | 0.035 | 3.51E-06 | 20.463 |
| family | Streptococcaceae | rs12595929 | C | T | 0.058 | 0.013 | 4.89E-06 | 20.461 |
| family | Erysipelotrichaceae | rs1074800 | G | A | 0.049 | 0.011 | 6.15E-06 | 20.459 |
| family | Verrucomicrobiaceae | rs61779207 | A | G | -0.076 | 0.017 | 6.63E-06 | 20.459 |
| family | Lachnospiraceae | rs62078073 | T | G | 0.053 | 0.012 | 5.88E-06 | 20.458 |
| family | Rhodospirillaceae | rs62255732 | C | T | -0.089 | 0.020 | 5.36E-06 | 20.454 |
| family | Bifidobacteriaceae | rs73797465 | G | T | -0.094 | 0.021 | 4.85E-06 | 20.454 |
| family | Peptostreptococcaceae | rs9573928 | T | C | -0.064 | 0.014 | 7.01E-06 | 20.452 |
| family | Bifidobacteriaceae | rs75524146 | C | T | 0.074 | 0.016 | 7.23E-06 | 20.451 |
| family | Actinomycetaceae | rs2110088 | A | T | 0.079 | 0.017 | 6.40E-06 | 20.449 |
| family | Alcaligenaceae | rs35945722 | G | A | 0.063 | 0.014 | 4.39E-06 | 20.447 |
| family | FamilyXIII | rs10911971 | G | C | -0.064 | 0.014 | 7.98E-06 | 20.445 |
| family | Christensenellaceae | rs5752918 | G | A | -0.068 | 0.015 | 4.73E-06 | 20.445 |
| family | FamilyXI | rs11547159 | T | C | -0.165 | 0.036 | 9.32E-06 | 20.444 |
| family | Clostridiaceae1 | rs62397761 | G | A | 0.062 | 0.014 | 9.08E-06 | 20.443 |
| family | ClostridialesvadinBB60group | rs13004970 | T | A | 0.072 | 0.016 | 6.00E-06 | 20.441 |
| family | Alcaligenaceae | . | T | A | -0.056 | 0.012 | 6.08E-06 | 20.437 |
| family | Ruminococcaceae | rs10172224 | C | A | 0.160 | 0.035 | 3.49E-06 | 20.436 |
| family | Verrucomicrobiaceae | rs4497215 | C | T | 0.064 | 0.014 | 6.43E-06 | 20.433 |
| family | Prevotellaceae | rs34660375 | G | A | -0.081 | 0.018 | 7.40E-06 | 20.431 |
| family | Bifidobacteriaceae | rs11688414 | A | G | 0.054 | 0.012 | 6.08E-06 | 20.423 |
| family | Veillonellaceae | rs6692542 | G | A | 0.053 | 0.012 | 8.68E-06 | 20.422 |
| family | Actinomycetaceae | rs61937253 | C | T | 0.079 | 0.018 | 5.68E-06 | 20.422 |
| family | Peptostreptococcaceae | rs117127341 | C | T | 0.158 | 0.035 | 7.91E-06 | 20.418 |
| family | Veillonellaceae | rs79596873 | G | A | 0.205 | 0.045 | 6.63E-06 | 20.416 |
| family | Erysipelotrichaceae | rs9852883 | C | A | 0.048 | 0.011 | 6.33E-06 | 20.416 |
| family | Rhodospirillaceae | rs72714493 | G | A | 0.082 | 0.018 | 7.35E-06 | 20.414 |
| family | Coriobacteriaceae | rs4880783 | T | G | 0.057 | 0.013 | 5.90E-06 | 20.414 |
| family | ClostridialesvadinBB60group | rs989682 | G | A | 0.070 | 0.016 | 6.85E-06 | 20.413 |
| family | Streptococcaceae | rs7316693 | A | G | -0.055 | 0.012 | 5.87E-06 | 20.412 |
| family | Alcaligenaceae | rs112135816 | G | T | -0.078 | 0.017 | 5.28E-06 | 20.411 |
| family | Rhodospirillaceae | rs11892008 | C | T | 0.114 | 0.025 | 6.34E-06 | 20.410 |
| family | Bifidobacteriaceae | rs12188321 | C | G | 0.053 | 0.012 | 6.41E-06 | 20.410 |
| family | Bifidobacteriaceae | rs16832624 | T | C | 0.061 | 0.014 | 7.07E-06 | 20.409 |
| family | Streptococcaceae | rs7219218 | T | A | -0.050 | 0.011 | 6.33E-06 | 20.406 |
| family | Lactobacillaceae | rs12547259 | A | G | -0.077 | 0.017 | 6.51E-06 | 20.405 |
| family | Methanobacteriaceae | rs62240389 | A | G | -0.188 | 0.042 | 7.64E-06 | 20.402 |
| family | Veillonellaceae | rs17056612 | G | A | 0.093 | 0.021 | 8.10E-06 | 20.401 |
| family | Prevotellaceae | rs12873417 | T | C | 0.058 | 0.013 | 4.67E-06 | 20.394 |
| family | Bifidobacteriaceae | rs4072434 | G | C | 0.055 | 0.012 | 8.36E-06 | 20.394 |
| family | Streptococcaceae | rs16950051 | G | A | 0.107 | 0.024 | 5.34E-06 | 20.391 |
| family | Veillonellaceae | rs2585520 | T | G | -0.090 | 0.020 | 5.27E-06 | 20.387 |
| family | Victivallaceae | rs253708 | G | A | 0.118 | 0.026 | 7.81E-06 | 20.384 |
| family | Verrucomicrobiaceae | rs2199602 | G | T | 0.066 | 0.015 | 7.34E-06 | 20.382 |
| family | Lactobacillaceae | rs62314653 | A | C | 0.177 | 0.039 | 6.59E-06 | 20.379 |
| family | Methanobacteriaceae | rs74599069 | C | A | 0.221 | 0.049 | 8.22E-06 | 20.378 |
| family | Rhodospirillaceae | rs12772311 | T | C | 0.072 | 0.016 | 5.76E-06 | 20.377 |
| family | Defluviitaleaceae | rs12122999 | T | G | -0.082 | 0.018 | 6.18E-06 | 20.377 |
| family | FamilyXIII | rs66753613 | A | G | 0.065 | 0.014 | 8.08E-06 | 20.375 |
| family | Rhodospirillaceae | rs35396261 | T | G | 0.072 | 0.016 | 5.97E-06 | 20.367 |
| family | Bifidobacteriaceae | rs6899771 | G | A | -0.091 | 0.020 | 7.28E-06 | 20.365 |
| family | Bacteroidaceae | rs13207588 | G | A | -0.059 | 0.013 | 7.49E-06 | 20.365 |
| family | Lachnospiraceae | rs146660815 | C | T | -0.215 | 0.048 | 6.53E-06 | 20.357 |
| family | ClostridialesvadinBB60group | rs4972882 | C | A | 0.072 | 0.016 | 6.32E-06 | 20.351 |
| family | Peptostreptococcaceae | rs59865771 | T | C | -0.057 | 0.013 | 7.69E-06 | 20.349 |
| family | FamilyXIII | rs10158652 | C | T | -0.064 | 0.014 | 8.22E-06 | 20.346 |
| family | Bifidobacteriaceae | rs11655079 | C | T | -0.059 | 0.013 | 5.92E-06 | 20.345 |
| family | Alcaligenaceae | rs11128180 | G | A | 0.059 | 0.013 | 6.24E-06 | 20.342 |
| family | FamilyXIII | rs35112465 | C | A | -0.061 | 0.014 | 4.77E-06 | 20.341 |
| family | Prevotellaceae | rs13069367 | C | A | -0.054 | 0.012 | 7.39E-06 | 20.338 |
| family | Bacteroidaceae | rs7322219 | T | C | 0.052 | 0.012 | 5.83E-06 | 20.337 |
| family | Bifidobacteriaceae | rs79176913 | C | A | 0.072 | 0.016 | 8.15E-06 | 20.335 |
| family | Bacteroidaceae | rs2366421 | A | T | -0.053 | 0.012 | 7.65E-06 | 20.334 |
| family | Streptococcaceae | rs11110079 | C | A | -0.080 | 0.018 | 5.49E-06 | 20.333 |
| family | Bifidobacteriaceae | rs503227 | T | C | 0.072 | 0.016 | 6.71E-06 | 20.332 |
| family | Rikenellaceae | rs7832304 | G | T | -0.072 | 0.016 | 8.71E-06 | 20.330 |
| family | Verrucomicrobiaceae | rs12045978 | G | A | 0.064 | 0.014 | 6.86E-06 | 20.330 |
| family | Bifidobacteriaceae | rs4988201 | A | G | 0.072 | 0.016 | 7.71E-06 | 20.329 |
| family | Coriobacteriaceae | rs8010111 | A | G | -0.103 | 0.023 | 6.90E-06 | 20.328 |
| family | Desulfovibrionaceae | rs7199026 | A | C | -0.053 | 0.012 | 6.90E-06 | 20.327 |
| family | Coriobacteriaceae | rs76779974 | G | C | 0.077 | 0.017 | 5.63E-06 | 20.326 |
| family | Lactobacillaceae | rs74599091 | G | A | 0.192 | 0.043 | 7.70E-06 | 20.323 |
| family | Streptococcaceae | rs76717940 | A | T | 0.151 | 0.033 | 3.09E-06 | 20.323 |
| family | Methanobacteriaceae | rs4257531 | A | G | 0.164 | 0.036 | 7.44E-06 | 20.316 |
| family | Alcaligenaceae | rs148149311 | G | A | 0.127 | 0.028 | 8.36E-06 | 20.315 |
| family | Rhodospirillaceae | rs4266206 | G | A | -0.088 | 0.020 | 5.84E-06 | 20.312 |
| family | FamilyXIII | rs10404377 | A | C | -0.050 | 0.011 | 6.99E-06 | 20.311 |
| family | Victivallaceae | rs253719 | G | A | 0.118 | 0.026 | 8.50E-06 | 20.309 |
| family | Desulfovibrionaceae | rs72647048 | C | T | -0.077 | 0.017 | 9.61E-06 | 20.307 |
| family | Alcaligenaceae | rs6561944 | T | C | -0.050 | 0.011 | 5.96E-06 | 20.304 |
| family | Bifidobacteriaceae | rs12465599 | G | A | 0.053 | 0.012 | 7.13E-06 | 20.303 |
| family | Clostridiaceae1 | rs12490337 | G | C | -0.062 | 0.014 | 6.91E-06 | 20.300 |
| family | Verrucomicrobiaceae | rs72663785 | C | G | 0.066 | 0.015 | 7.56E-06 | 20.300 |
| family | Veillonellaceae | rs6909981 | T | C | -0.064 | 0.014 | 5.48E-06 | 20.298 |
| family | Rikenellaceae | rs2447496 | A | G | -0.055 | 0.012 | 6.09E-06 | 20.297 |
| family | Actinomycetaceae | rs58484246 | C | T | 0.076 | 0.017 | 6.12E-06 | 20.297 |
| family | Desulfovibrionaceae | rs2692012 | G | A | 0.114 | 0.025 | 1.56E-06 | 20.294 |
| family | Porphyromonadaceae | rs12486680 | C | A | 0.098 | 0.022 | 4.29E-06 | 20.294 |
| family | Verrucomicrobiaceae | rs7693865 | C | T | 0.107 | 0.024 | 6.13E-06 | 20.292 |
| family | Bifidobacteriaceae | rs7581814 | C | G | 0.071 | 0.016 | 8.01E-06 | 20.291 |
| family | Prevotellaceae | rs34071565 | A | T | -0.157 | 0.035 | 5.60E-06 | 20.290 |
| family | Victivallaceae | rs2241426 | G | C | 0.117 | 0.026 | 8.55E-06 | 20.289 |
| family | Pasteurellaceae | rs40513 | T | G | 0.118 | 0.026 | 7.08E-06 | 20.288 |
| family | Streptococcaceae | rs7224059 | C | A | -0.050 | 0.011 | 6.38E-06 | 20.287 |
| family | Rikenellaceae | rs11088466 | T | A | -0.048 | 0.011 | 6.80E-06 | 20.284 |
| family | Streptococcaceae | rs7134384 | T | C | -0.080 | 0.018 | 4.95E-06 | 20.283 |
| family | Rhodospirillaceae | rs67824877 | T | G | 0.071 | 0.016 | 5.76E-06 | 20.280 |
| family | FamilyXIII | rs10911961 | A | G | -0.064 | 0.014 | 8.81E-06 | 20.276 |
| family | Alcaligenaceae | rs58598064 | G | T | 0.069 | 0.015 | 5.73E-06 | 20.273 |
| family | Coriobacteriaceae | rs2442778 | A | G | -0.116 | 0.026 | 9.03E-06 | 20.272 |
| family | FamilyXIII | rs13143273 | C | A | -0.067 | 0.015 | 6.96E-06 | 20.270 |
| family | Veillonellaceae | rs1447205 | C | G | -0.052 | 0.012 | 7.35E-06 | 20.269 |
| family | Streptococcaceae | rs957755 | G | T | -0.064 | 0.014 | 7.42E-06 | 20.268 |
| family | Streptococcaceae | rs8072812 | G | A | -0.050 | 0.011 | 7.23E-06 | 20.265 |
| family | Streptococcaceae | rs11110057 | G | A | -0.084 | 0.019 | 7.33E-06 | 20.263 |
| family | Bifidobacteriaceae | rs621341 | C | T | -0.055 | 0.012 | 3.67E-06 | 20.263 |
| family | Alcaligenaceae | rs28437793 | C | G | -0.057 | 0.013 | 6.70E-06 | 20.262 |
| family | Veillonellaceae | rs2036713 | A | C | -0.052 | 0.011 | 6.91E-06 | 20.262 |
| family | Acidaminococcaceae | rs3000673 | C | G | -0.063 | 0.014 | 6.63E-06 | 20.259 |
| family | BacteroidalesS24 | rs7217209 | T | C | 0.084 | 0.019 | 8.43E-06 | 20.256 |
| family | Bifidobacteriaceae | rs7561565 | T | G | 0.071 | 0.016 | 8.42E-06 | 20.254 |
| family | FamilyXIII | rs12409191 | A | G | -0.064 | 0.014 | 8.52E-06 | 20.253 |
| family | Rikenellaceae | rs61978199 | T | C | -0.152 | 0.034 | 5.42E-06 | 20.252 |
| family | Rikenellaceae | rs61978202 | G | A | -0.151 | 0.034 | 5.68E-06 | 20.252 |
| family | ClostridialesvadinBB60group | rs4972883 | C | A | 0.072 | 0.016 | 6.67E-06 | 20.251 |
| family | BacteroidalesS24 | rs61508842 | C | T | 0.123 | 0.027 | 7.83E-06 | 20.250 |
| family | Prevotellaceae | rs2322142 | C | T | -0.060 | 0.013 | 8.39E-06 | 20.250 |
| family | Alcaligenaceae | rs41293110 | G | T | 0.068 | 0.015 | 6.12E-06 | 20.249 |
| family | Porphyromonadaceae | rs2137102 | G | C | 0.054 | 0.012 | 9.52E-06 | 20.246 |
| family | FamilyXIII | rs34648645 | A | T | -0.067 | 0.015 | 6.33E-06 | 20.243 |
| family | FamilyXI | rs2756218 | C | T | -0.113 | 0.025 | 7.12E-06 | 20.241 |
| family | ClostridialesvadinBB60group | rs10517600 | G | T | 0.063 | 0.014 | 6.83E-06 | 20.241 |
| family | Alcaligenaceae | rs55995696 | T | C | 0.068 | 0.015 | 6.23E-06 | 20.229 |
| family | Lachnospiraceae | rs13241165 | A | T | -0.047 | 0.011 | 6.71E-06 | 20.228 |
| family | Bifidobacteriaceae | rs6750549 | A | G | 0.072 | 0.016 | 8.10E-06 | 20.226 |
| family | Bacteroidaceae | rs79471440 | G | T | 0.058 | 0.013 | 5.24E-06 | 20.226 |
| family | Streptococcaceae | rs9903102 | A | C | -0.069 | 0.015 | 4.92E-06 | 20.226 |
| family | Oxalobacteraceae | rs111624546 | C | T | -0.128 | 0.028 | 7.50E-06 | 20.225 |
| family | Defluviitaleaceae | rs28696126 | T | A | -0.107 | 0.024 | 5.56E-06 | 20.223 |
| family | Pasteurellaceae | rs77529231 | T | C | -0.224 | 0.050 | 8.54E-06 | 20.218 |
| family | Prevotellaceae | rs2278540 | A | G | 0.055 | 0.012 | 8.44E-06 | 20.218 |
| family | Bifidobacteriaceae | rs4245838 | C | T | 0.105 | 0.023 | 4.34E-06 | 20.214 |
| family | Porphyromonadaceae | rs2667697 | G | A | 0.054 | 0.012 | 9.03E-06 | 20.212 |
| family | Streptococcaceae | rs7187700 | G | A | 0.058 | 0.013 | 5.25E-06 | 20.211 |
| family | Methanobacteriaceae | rs10424197 | A | G | -0.111 | 0.025 | 9.28E-06 | 20.211 |
| family | Pasteurellaceae | rs9938097 | C | T | -0.071 | 0.016 | 8.23E-06 | 20.209 |
| family | Bifidobacteriaceae | rs10180515 | T | A | 0.063 | 0.014 | 4.10E-06 | 20.209 |
| family | Peptostreptococcaceae | . | T | A | 0.056 | 0.012 | 6.37E-06 | 20.208 |
| family | Veillonellaceae | rs9345168 | A | C | 0.051 | 0.011 | 8.49E-06 | 20.207 |
| family | Methanobacteriaceae | rs73068003 | T | G | -0.158 | 0.035 | 8.45E-06 | 20.206 |
| family | FamilyXIII | rs6831434 | A | G | -0.049 | 0.011 | 7.27E-06 | 20.204 |
| family | Clostridiaceae1 | rs10875374 | T | C | -0.054 | 0.012 | 8.10E-06 | 20.197 |
| family | Rhodospirillaceae | rs2162883 | T | C | 0.088 | 0.019 | 8.90E-06 | 20.196 |
| family | Bifidobacteriaceae | rs309150 | T | A | 0.071 | 0.016 | 8.14E-06 | 20.192 |
| family | Porphyromonadaceae | rs864093 | C | A | -0.053 | 0.012 | 9.60E-06 | 20.188 |
| family | Veillonellaceae | rs4736971 | C | A | -0.052 | 0.012 | 7.73E-06 | 20.186 |
| family | Clostridiaceae1 | rs2123173 | C | T | 0.073 | 0.016 | 8.78E-06 | 20.184 |
| family | Bifidobacteriaceae | rs11684458 | A | G | 0.063 | 0.014 | 3.84E-06 | 20.179 |
| family | Rhodospirillaceae | rs56250014 | T | A | 0.115 | 0.026 | 6.63E-06 | 20.177 |
| family | Rhodospirillaceae | rs7001029 | T | C | 0.117 | 0.026 | 5.35E-06 | 20.171 |
| family | Actinomycetaceae | rs34951409 | C | G | 0.078 | 0.017 | 7.29E-06 | 20.169 |
| family | Streptococcaceae | rs6563952 | C | G | 0.080 | 0.018 | 8.71E-06 | 20.166 |
| family | Rhodospirillaceae | rs1572583 | A | C | 0.071 | 0.016 | 6.45E-06 | 20.166 |
| family | FamilyXIII | rs28575314 | T | C | 0.049 | 0.011 | 6.96E-06 | 20.166 |
| family | Bifidobacteriaceae | rs9800013 | A | G | 0.065 | 0.015 | 4.84E-06 | 20.164 |
| family | Actinomycetaceae | rs7958229 | C | T | 0.078 | 0.017 | 6.80E-06 | 20.161 |
| family | Bifidobacteriaceae | rs6880820 | T | C | 0.064 | 0.014 | 4.07E-06 | 20.160 |
| family | Streptococcaceae | rs2370086 | T | G | -0.083 | 0.018 | 5.77E-06 | 20.159 |
| family | FamilyXI | rs10831388 | C | T | -0.130 | 0.029 | 7.63E-06 | 20.158 |
| family | Alcaligenaceae | rs73119510 | C | T | 0.068 | 0.015 | 6.50E-06 | 20.158 |
| family | Oxalobacteraceae | rs56097943 | G | A | 0.180 | 0.040 | 8.90E-06 | 20.156 |
| family | Peptostreptococcaceae | rs4698733 | T | C | 0.055 | 0.012 | 6.32E-06 | 20.155 |
| family | Lachnospiraceae | rs12760724 | C | A | -0.048 | 0.011 | 7.27E-06 | 20.155 |
| family | Methanobacteriaceae | rs41288287 | C | T | 0.199 | 0.044 | 8.70E-06 | 20.154 |
| family | FamilyXI | rs576386 | G | C | 0.114 | 0.026 | 9.07E-06 | 20.152 |
| family | BacteroidalesS24 | rs28434476 | G | C | 0.078 | 0.017 | 7.07E-06 | 20.151 |
| family | Streptococcaceae | rs7212556 | G | T | -0.050 | 0.011 | 7.03E-06 | 20.147 |
| family | Veillonellaceae | rs1693340 | C | T | 0.082 | 0.018 | 9.25E-06 | 20.147 |
| family | Bifidobacteriaceae | rs309162 | T | A | 0.071 | 0.016 | 8.46E-06 | 20.146 |
| family | Alcaligenaceae | rs7768419 | A | G | 0.126 | 0.028 | 8.85E-06 | 20.145 |
| family | Alcaligenaceae | rs67623980 | C | T | -0.061 | 0.013 | 8.95E-06 | 20.144 |
| family | Streptococcaceae | rs386474 | T | C | -0.050 | 0.011 | 7.56E-06 | 20.141 |
| family | Lachnospiraceae | rs3127230 | T | C | -0.050 | 0.011 | 6.20E-06 | 20.141 |
| family | Streptococcaceae | rs6504148 | A | G | -0.050 | 0.011 | 7.62E-06 | 20.140 |
| family | Lachnospiraceae | rs72861044 | A | G | 0.056 | 0.013 | 5.53E-06 | 20.138 |
| family | Bifidobacteriaceae | rs2176716 | C | T | 0.063 | 0.014 | 4.01E-06 | 20.135 |
| family | FamilyXIII | rs6704304 | G | A | -0.063 | 0.014 | 9.31E-06 | 20.135 |
| family | Actinomycetaceae | rs59800030 | C | T | 0.076 | 0.017 | 6.71E-06 | 20.134 |
| family | Verrucomicrobiaceae | rs12026127 | G | A | 0.064 | 0.014 | 7.09E-06 | 20.133 |
| family | Veillonellaceae | rs75768969 | G | C | -0.070 | 0.016 | 7.19E-06 | 20.131 |
| family | Bifidobacteriaceae | rs309172 | A | G | 0.070 | 0.016 | 6.64E-06 | 20.129 |
| family | Veillonellaceae | rs72643860 | A | G | -0.052 | 0.012 | 8.00E-06 | 20.128 |
| family | Alcaligenaceae | rs4511740 | A | G | 0.058 | 0.013 | 7.12E-06 | 20.127 |
| family | Enterobacteriaceae | rs4792380 | T | A | 0.116 | 0.026 | 9.49E-06 | 20.125 |
| family | Bifidobacteriaceae | rs68005421 | G | C | 0.055 | 0.012 | 9.22E-06 | 20.120 |
| family | Erysipelotrichaceae | rs12938302 | C | T | -0.075 | 0.017 | 9.17E-06 | 20.119 |
| family | ClostridialesvadinBB60group | rs35380989 | C | A | 0.072 | 0.016 | 6.96E-06 | 20.114 |
| family | Erysipelotrichaceae | rs35587059 | C | T | 0.048 | 0.011 | 7.13E-06 | 20.114 |
| family | ClostridialesvadinBB60group | rs3845824 | A | G | 0.071 | 0.016 | 7.22E-06 | 20.113 |
| family | Porphyromonadaceae | rs28868966 | C | T | -0.053 | 0.012 | 9.69E-06 | 20.110 |
| family | Defluviitaleaceae | rs1908593 | C | T | 0.070 | 0.016 | 7.86E-06 | 20.108 |
| family | Actinomycetaceae | rs56236273 | T | A | 0.076 | 0.017 | 6.71E-06 | 20.108 |
| family | Erysipelotrichaceae | rs41300431 | G | A | 0.048 | 0.011 | 7.12E-06 | 20.106 |
| family | Porphyromonadaceae | rs12698889 | G | A | 0.075 | 0.017 | 7.21E-06 | 20.102 |
| family | Clostridiaceae1 | rs11166591 | A | G | -0.054 | 0.012 | 8.47E-06 | 20.093 |
| family | Streptococcaceae | rs11769597 | T | C | -0.064 | 0.014 | 8.09E-06 | 20.093 |
| family | Peptostreptococcaceae | rs12647693 | G | A | 0.056 | 0.012 | 6.94E-06 | 20.088 |
| family | Bifidobacteriaceae | rs3099429 | T | A | 0.071 | 0.016 | 7.65E-06 | 20.086 |
| family | Pasteurellaceae | rs6100166 | G | C | 0.067 | 0.015 | 8.38E-06 | 20.086 |
| family | Victivallaceae | rs7314815 | A | G | 0.101 | 0.023 | 6.40E-06 | 20.079 |
| family | Streptococcaceae | . | A | T | -0.050 | 0.011 | 7.09E-06 | 20.075 |
| family | Coriobacteriaceae | rs13307134 | T | C | 0.057 | 0.013 | 7.80E-06 | 20.072 |
| family | Acidaminococcaceae | rs1565606 | T | C | -0.063 | 0.014 | 7.33E-06 | 20.070 |
| family | Bifidobacteriaceae | rs61253125 | T | C | 0.071 | 0.016 | 8.75E-06 | 20.069 |
| family | FamilyXI | rs2156611 | C | T | -0.112 | 0.025 | 9.43E-06 | 20.068 |
| family | Bifidobacteriaceae | rs309154 | C | T | 0.072 | 0.016 | 9.29E-06 | 20.066 |
| family | Rikenellaceae | rs9578457 | A | G | -0.141 | 0.032 | 3.99E-06 | 20.064 |
| family | Alcaligenaceae | rs10203027 | T | C | 0.062 | 0.014 | 8.46E-06 | 20.064 |
| family | Verrucomicrobiaceae | rs7538356 | A | C | 0.064 | 0.014 | 7.77E-06 | 20.063 |
| family | Victivallaceae | rs2944276 | C | G | -0.118 | 0.026 | 7.82E-06 | 20.062 |
| family | Clostridiaceae1 | rs881532 | G | A | -0.053 | 0.012 | 7.90E-06 | 20.060 |
| family | Coriobacteriaceae | rs7977399 | C | T | -0.058 | 0.013 | 6.07E-06 | 20.058 |
| family | Streptococcaceae | rs7838901 | A | G | -0.061 | 0.014 | 6.32E-06 | 20.040 |
| family | Bacteroidaceae | rs1340391 | C | T | -0.059 | 0.013 | 6.73E-06 | 20.040 |
| family | Alcaligenaceae | rs28480294 | C | T | -0.052 | 0.012 | 6.61E-06 | 20.033 |
| family | FamilyXIII | rs6675578 | G | A | -0.063 | 0.014 | 9.69E-06 | 20.032 |
| family | Actinomycetaceae | rs12230967 | A | G | 0.078 | 0.017 | 7.79E-06 | 20.031 |
| family | Streptococcaceae | rs239358 | A | G | -0.050 | 0.011 | 8.48E-06 | 20.028 |
| family | Alcaligenaceae | rs66514327 | A | T | 0.068 | 0.015 | 6.58E-06 | 20.026 |
| family | FamilyXIII | rs1887048 | A | G | 0.058 | 0.013 | 9.65E-06 | 20.026 |
| family | Oxalobacteraceae | rs62341594 | C | T | 0.179 | 0.040 | 9.12E-06 | 20.024 |
| family | Alcaligenaceae | rs6118993 | C | T | 0.071 | 0.016 | 8.44E-06 | 20.023 |
| family | Lachnospiraceae | rs11139361 | C | T | 0.049 | 0.011 | 4.26E-06 | 20.022 |
| family | Alcaligenaceae | rs8115462 | T | C | 0.064 | 0.014 | 6.42E-06 | 20.022 |
| family | Rikenellaceae | rs10217435 | T | C | -0.088 | 0.020 | 6.51E-06 | 20.022 |
| family | Acidaminococcaceae | rs74540770 | A | G | -0.109 | 0.024 | 7.09E-06 | 20.021 |
| family | Bifidobacteriaceae | rs10221893 | T | C | 0.054 | 0.012 | 9.68E-06 | 20.020 |
| family | Prevotellaceae | rs3805026 | T | C | -0.065 | 0.014 | 8.52E-06 | 20.016 |
| family | Lachnospiraceae | rs2910921 | C | T | 0.160 | 0.036 | 8.42E-06 | 20.014 |
| family | Lachnospiraceae | rs7359994 | C | T | -0.050 | 0.011 | 5.36E-06 | 20.009 |
| family | Lactobacillaceae | rs1978760 | T | C | -0.076 | 0.017 | 7.99E-06 | 20.008 |
| family | Clostridiaceae1 | rs4908133 | T | C | -0.053 | 0.012 | 8.87E-06 | 20.008 |
| family | FamilyXI | rs34205092 | A | G | -0.135 | 0.030 | 7.79E-06 | 20.005 |
| family | Streptococcaceae | rs13379080 | T | C | -0.082 | 0.018 | 6.72E-06 | 20.004 |
| family | Lachnospiraceae | rs887732 | G | C | -0.059 | 0.013 | 7.55E-06 | 19.999 |
| family | Rhodospirillaceae | rs11127841 | G | A | -0.088 | 0.020 | 6.82E-06 | 19.998 |
| family | Victivallaceae | rs2731835 | G | T | 0.117 | 0.026 | 9.44E-06 | 19.997 |
| family | Peptostreptococcaceae | rs1467258 | A | G | 0.073 | 0.016 | 7.90E-06 | 19.995 |
| family | ClostridialesvadinBB60group | rs13142641 | G | C | 0.062 | 0.014 | 7.42E-06 | 19.990 |
| family | Rhodospirillaceae | rs12977163 | C | G | 0.073 | 0.016 | 7.63E-06 | 19.989 |
| family | Lachnospiraceae | rs1205443 | G | A | 0.050 | 0.011 | 7.29E-06 | 19.987 |
| family | Methanobacteriaceae | rs9532770 | T | C | 0.124 | 0.028 | 9.34E-06 | 19.985 |
| family | BacteroidalesS24 | rs28545668 | G | C | 0.078 | 0.017 | 8.66E-06 | 19.980 |
| family | Erysipelotrichaceae | rs7906464 | T | A | -0.053 | 0.012 | 7.35E-06 | 19.973 |
| family | Clostridiaceae1 | rs7629156 | C | T | -0.061 | 0.014 | 7.90E-06 | 19.971 |
| family | Streptococcaceae | rs8076071 | A | T | -0.049 | 0.011 | 6.95E-06 | 19.970 |
| family | Enterobacteriaceae | rs6069866 | G | C | 0.058 | 0.013 | 7.83E-06 | 19.968 |
| family | FamilyXI | rs11021252 | C | A | -0.130 | 0.029 | 8.05E-06 | 19.968 |
| family | Bifidobacteriaceae | rs4988163 | G | T | 0.071 | 0.016 | 9.50E-06 | 19.964 |
| family | Bifidobacteriaceae | rs4954274 | G | A | 0.089 | 0.020 | 9.60E-06 | 19.963 |
| family | Victivallaceae | rs34962571 | C | A | -0.187 | 0.042 | 6.25E-06 | 19.963 |
| family | Veillonellaceae | rs2354569 | T | C | -0.052 | 0.012 | 8.67E-06 | 19.962 |
| family | Veillonellaceae | rs2561116 | G | T | -0.084 | 0.019 | 7.89E-06 | 19.960 |
| family | Christensenellaceae | rs72706624 | T | G | 0.088 | 0.020 | 6.38E-06 | 19.957 |
| family | Porphyromonadaceae | rs35233670 | C | T | -0.047 | 0.011 | 7.91E-06 | 19.953 |
| family | ClostridialesvadinBB60group | rs9954215 | C | T | -0.062 | 0.014 | 8.04E-06 | 19.951 |
| family | Actinomycetaceae | rs11055873 | A | G | 0.078 | 0.017 | 7.85E-06 | 19.950 |
| family | FamilyXIII | rs35559104 | G | A | -0.060 | 0.014 | 5.90E-06 | 19.948 |
| family | ClostridialesvadinBB60group | rs17121075 | A | G | 0.077 | 0.017 | 7.91E-06 | 19.948 |
| family | Rikenellaceae | rs61978203 | C | T | -0.151 | 0.034 | 6.19E-06 | 19.948 |
| family | Oxalobacteraceae | rs7617946 | T | C | 0.098 | 0.022 | 7.73E-06 | 19.946 |
| family | Veillonellaceae | rs4236917 | G | C | -0.052 | 0.012 | 8.71E-06 | 19.944 |
| family | Erysipelotrichaceae | rs290833 | G | T | -0.050 | 0.011 | 8.03E-06 | 19.942 |
| family | Clostridiaceae1 | rs2038365 | C | G | -0.053 | 0.012 | 9.15E-06 | 19.942 |
| family | ClostridialesvadinBB60group | rs12123324 | C | T | -0.074 | 0.017 | 8.02E-06 | 19.938 |
| family | Clostridiaceae1 | rs7539216 | A | G | -0.053 | 0.012 | 9.29E-06 | 19.937 |
| family | Oxalobacteraceae | rs6445050 | A | G | 0.098 | 0.022 | 7.79E-06 | 19.937 |
| family | Ruminococcaceae | rs1158100 | G | A | -0.049 | 0.011 | 8.61E-06 | 19.936 |
| family | BacteroidalesS24 | rs10872669 | G | A | -0.123 | 0.028 | 9.49E-06 | 19.932 |
| family | Erysipelotrichaceae | rs8096144 | C | G | -0.086 | 0.019 | 9.53E-06 | 19.930 |
| family | Porphyromonadaceae | rs7788868 | G | A | 0.076 | 0.017 | 9.58E-06 | 19.927 |
| family | Veillonellaceae | rs2561115 | C | A | -0.083 | 0.019 | 8.99E-06 | 19.924 |
| family | Peptococcaceae | rs11787651 | G | A | 0.129 | 0.029 | 8.29E-06 | 19.922 |
| family | Prevotellaceae | rs2278541 | C | T | 0.055 | 0.012 | 9.80E-06 | 19.920 |
| family | Defluviitaleaceae | rs6952956 | C | T | -0.108 | 0.024 | 8.02E-06 | 19.920 |
| family | Lachnospiraceae | rs1205444 | G | A | 0.050 | 0.011 | 7.53E-06 | 19.916 |
| family | Streptococcaceae | rs7835507 | C | A | -0.073 | 0.016 | 6.92E-06 | 19.916 |
| family | ClostridialesvadinBB60group | rs968104 | T | A | 0.111 | 0.025 | 8.24E-06 | 19.914 |
| family | Coriobacteriaceae | rs11073596 | G | T | 0.051 | 0.011 | 8.14E-06 | 19.912 |
| family | Porphyromonadaceae | rs7038649 | T | C | -0.058 | 0.013 | 7.69E-06 | 19.912 |
| family | Christensenellaceae | rs9613908 | C | T | -0.067 | 0.015 | 5.22E-06 | 19.901 |
| family | Lachnospiraceae | rs12709872 | C | T | -0.050 | 0.011 | 6.15E-06 | 19.899 |
| family | Porphyromonadaceae | rs10757280 | G | A | -0.065 | 0.015 | 6.90E-06 | 19.898 |
| family | Rhodospirillaceae | rs7623329 | C | A | -0.071 | 0.016 | 7.51E-06 | 19.897 |
| family | Christensenellaceae | rs4805124 | G | C | 0.049 | 0.011 | 8.36E-06 | 19.897 |
| family | Lactobacillaceae | rs1434765 | C | A | -0.076 | 0.017 | 8.52E-06 | 19.896 |
| family | BacteroidalesS24 | rs1398206 | G | A | 0.078 | 0.017 | 8.24E-06 | 19.895 |
| family | Rhodospirillaceae | rs6790948 | T | C | -0.129 | 0.029 | 9.33E-06 | 19.893 |
| family | Coriobacteriaceae | rs62448869 | A | T | -0.049 | 0.011 | 7.87E-06 | 19.891 |
| family | Rhodospirillaceae | rs6679026 | C | T | 0.112 | 0.025 | 9.95E-06 | 19.891 |
| family | Prevotellaceae | rs12057990 | T | C | 0.059 | 0.013 | 8.97E-06 | 19.889 |
| family | Streptococcaceae | rs2907466 | G | A | -0.049 | 0.011 | 7.76E-06 | 19.887 |
| family | BacteroidalesS24 | rs3920216 | A | G | 0.078 | 0.017 | 8.35E-06 | 19.887 |
| family | Verrucomicrobiaceae | rs12024893 | A | G | 0.063 | 0.014 | 8.50E-06 | 19.884 |
| family | Streptococcaceae | rs2952186 | T | C | -0.068 | 0.015 | 6.41E-06 | 19.878 |
| family | Pasteurellaceae | rs6972479 | G | A | -0.078 | 0.018 | 7.75E-06 | 19.878 |
| family | Verrucomicrobiaceae | rs4595371 | A | G | 0.063 | 0.014 | 8.52E-06 | 19.871 |
| family | ClostridialesvadinBB60group | rs2938236 | C | A | 0.114 | 0.026 | 9.13E-06 | 19.869 |
| family | Clostridiaceae1 | rs12123735 | C | T | -0.053 | 0.012 | 9.53E-06 | 19.867 |
| family | Porphyromonadaceae | rs10965284 | C | T | -0.064 | 0.014 | 4.96E-06 | 19.865 |
| family | Coriobacteriaceae | rs12974142 | A | G | 0.079 | 0.018 | 8.51E-06 | 19.865 |
| family | Oxalobacteraceae | rs561239 | G | A | 0.106 | 0.024 | 7.19E-06 | 19.864 |
| family | Verrucomicrobiaceae | rs79837054 | T | C | 0.105 | 0.024 | 7.64E-06 | 19.864 |
| family | Lactobacillaceae | rs820999 | G | T | -0.076 | 0.017 | 8.89E-06 | 19.861 |
| family | Alcaligenaceae | rs4584714 | A | G | -0.049 | 0.011 | 7.63E-06 | 19.860 |
| family | Porphyromonadaceae | rs10965207 | C | T | -0.066 | 0.015 | 7.80E-06 | 19.858 |
| family | Rikenellaceae | rs2150411 | G | T | -0.048 | 0.011 | 8.51E-06 | 19.858 |
| family | Methanobacteriaceae | rs6508769 | C | T | 0.154 | 0.034 | 8.23E-06 | 19.856 |
| family | Clostridiaceae1 | rs76185991 | C | G | -0.061 | 0.014 | 8.28E-06 | 19.856 |
| family | Christensenellaceae | rs5752917 | G | A | -0.067 | 0.015 | 6.62E-06 | 19.855 |
| family | Verrucomicrobiaceae | rs36010840 | C | T | 0.063 | 0.014 | 8.64E-06 | 19.855 |
| family | Rikenellaceae | rs2297256 | G | A | -0.048 | 0.011 | 8.52E-06 | 19.853 |
| family | Lachnospiraceae | rs10402491 | T | C | 0.066 | 0.015 | 7.58E-06 | 19.852 |
| family | Coriobacteriaceae | rs7898585 | C | T | 0.073 | 0.016 | 5.25E-06 | 19.850 |
| family | Verrucomicrobiaceae | rs4294419 | G | A | 0.063 | 0.014 | 8.04E-06 | 19.842 |
| family | Bifidobacteriaceae | rs4954556 | G | A | 0.114 | 0.026 | 4.45E-06 | 19.840 |
| family | Streptococcaceae | rs7916711 | G | A | 0.096 | 0.022 | 6.33E-06 | 19.839 |
| family | Alcaligenaceae | rs2040996 | C | T | -0.049 | 0.011 | 7.80E-06 | 19.834 |
| family | Streptococcaceae | rs2533008 | G | C | -0.049 | 0.011 | 8.91E-06 | 19.832 |
| family | Erysipelotrichaceae | rs28703642 | G | T | 0.053 | 0.012 | 7.64E-06 | 19.830 |
| family | Bifidobacteriaceae | rs13387501 | C | T | 0.062 | 0.014 | 4.78E-06 | 19.827 |
| family | Christensenellaceae | rs870002 | T | C | 0.049 | 0.011 | 6.54E-06 | 19.824 |
| family | Alcaligenaceae | rs371834896 | C | T | 0.068 | 0.015 | 8.10E-06 | 19.823 |
| family | ClostridialesvadinBB60group | rs276167 | A | G | 0.062 | 0.014 | 9.21E-06 | 19.819 |
| family | Oxalobacteraceae | rs10868235 | T | C | 0.088 | 0.020 | 8.95E-06 | 19.818 |
| family | Ruminococcaceae | rs7571324 | T | C | 0.157 | 0.035 | 6.01E-06 | 19.816 |
| family | Bifidobacteriaceae | rs55888705 | G | A | 0.054 | 0.012 | 8.66E-06 | 19.811 |
| family | Oxalobacteraceae | rs56133825 | T | C | -0.125 | 0.028 | 9.46E-06 | 19.811 |
| family | Lachnospiraceae | rs1345833 | G | A | -0.050 | 0.011 | 6.69E-06 | 19.811 |
| family | Victivallaceae | rs7627405 | T | C | -0.134 | 0.030 | 8.19E-06 | 19.809 |
| family | Coriobacteriaceae | rs45480394 | G | T | -0.050 | 0.011 | 9.71E-06 | 19.806 |
| family | Rhodospirillaceae | rs74354280 | T | C | -0.091 | 0.020 | 6.67E-06 | 19.805 |
| family | Clostridiaceae1 | rs56188186 | G | A | 0.097 | 0.022 | 8.24E-06 | 19.804 |
| family | Rikenellaceae | rs12350642 | A | G | -0.088 | 0.020 | 6.75E-06 | 19.802 |
| family | Rikenellaceae | rs12346296 | T | C | -0.088 | 0.020 | 6.75E-06 | 19.802 |
| family | BacteroidalesS24 | rs28410286 | A | G | 0.077 | 0.017 | 9.82E-06 | 19.801 |
| family | Alcaligenaceae | rs79846372 | G | A | 0.088 | 0.020 | 6.99E-06 | 19.798 |
| family | Methanobacteriaceae | rs11018665 | T | A | 0.111 | 0.025 | 6.52E-06 | 19.797 |
| family | Bifidobacteriaceae | rs10432371 | G | A | 0.053 | 0.012 | 6.02E-06 | 19.791 |
| family | Rikenellaceae | rs11841382 | T | G | 0.080 | 0.018 | 3.98E-06 | 19.790 |
| family | Prevotellaceae | rs75982374 | A | G | 0.080 | 0.018 | 8.04E-06 | 19.789 |
| family | Lachnospiraceae | rs7359957 | T | C | -0.050 | 0.011 | 6.07E-06 | 19.788 |
| family | ClostridialesvadinBB60group | rs150027448 | G | A | 0.114 | 0.026 | 9.54E-06 | 19.785 |
| family | Alcaligenaceae | rs73117396 | G | A | 0.068 | 0.015 | 8.15E-06 | 19.780 |
| family | Acidaminococcaceae | rs78702810 | C | T | -0.144 | 0.032 | 9.16E-06 | 19.778 |
| family | Clostridiaceae1 | rs10804492 | C | T | -0.061 | 0.014 | 8.87E-06 | 19.778 |
| family | Lactobacillaceae | rs9345899 | G | A | -0.124 | 0.028 | 9.45E-06 | 19.774 |
| family | Bifidobacteriaceae | rs10857328 | A | T | 0.056 | 0.013 | 7.24E-06 | 19.770 |
| family | Coriobacteriaceae | rs7303392 | C | T | -0.057 | 0.013 | 7.32E-06 | 19.770 |
| family | Streptococcaceae | rs6601426 | G | C | -0.068 | 0.015 | 8.26E-06 | 19.770 |
| family | Rhodospirillaceae | rs57340984 | C | T | 0.112 | 0.025 | 8.17E-06 | 19.766 |
| family | Rikenellaceae | rs8132424 | A | T | -0.047 | 0.011 | 8.94E-06 | 19.760 |
| family | Erysipelotrichaceae | rs1884466 | T | C | -0.048 | 0.011 | 9.53E-06 | 19.760 |
| family | Pasteurellaceae | rs111582866 | A | G | -0.114 | 0.026 | 7.07E-06 | 19.753 |
| family | Rhodospirillaceae | rs77304857 | A | C | -0.100 | 0.022 | 6.95E-06 | 19.737 |
| family | Lactobacillaceae | rs1529819 | G | A | -0.076 | 0.017 | 9.23E-06 | 19.736 |
| family | Ruminococcaceae | rs138850552 | C | T | 0.102 | 0.023 | 6.42E-06 | 19.733 |
| family | Streptococcaceae | rs11764382 | G | A | -0.063 | 0.014 | 8.85E-06 | 19.732 |
| family | Verrucomicrobiaceae | rs72663769 | T | C | 0.065 | 0.015 | 7.41E-06 | 19.732 |
| family | ClostridialesvadinBB60group | rs276175 | C | A | 0.062 | 0.014 | 9.38E-06 | 19.731 |
| family | Enterobacteriaceae | rs504442 | G | T | 0.084 | 0.019 | 5.17E-06 | 19.728 |
| family | Prevotellaceae | rs3805025 | A | G | -0.065 | 0.015 | 9.65E-06 | 19.727 |
| family | Bifidobacteriaceae | rs606101 | G | A | -0.054 | 0.012 | 7.54E-06 | 19.721 |
| family | Erysipelotrichaceae | rs7100916 | T | G | -0.053 | 0.012 | 7.62E-06 | 19.721 |
| family | Porphyromonadaceae | rs1980561 | G | A | -0.049 | 0.011 | 8.95E-06 | 19.719 |
| family | Rikenellaceae | rs9389714 | T | C | -0.064 | 0.014 | 8.79E-06 | 19.718 |
| family | Prevotellaceae | rs7975087 | A | C | -0.060 | 0.014 | 7.59E-06 | 19.716 |
| family | Rhodospirillaceae | rs13336560 | T | C | -0.070 | 0.016 | 9.17E-06 | 19.709 |
| family | Clostridiaceae1 | rs876740 | C | T | 0.054 | 0.012 | 8.03E-06 | 19.706 |
| family | Streptococcaceae | rs4968647 | T | C | -0.049 | 0.011 | 8.44E-06 | 19.705 |
| family | Alcaligenaceae | rs7331960 | A | G | -0.050 | 0.011 | 8.20E-06 | 19.704 |
| family | Rikenellaceae | rs35909684 | C | A | -0.085 | 0.019 | 7.10E-06 | 19.702 |
| family | BacteroidalesS24 | rs17281048 | A | G | 0.077 | 0.017 | 9.88E-06 | 19.700 |
| family | Alcaligenaceae | rs1859743 | C | T | -0.049 | 0.011 | 7.47E-06 | 19.696 |
| family | FamilyXI | rs11021251 | T | C | -0.129 | 0.029 | 9.18E-06 | 19.695 |
| family | Actinomycetaceae | rs12831981 | T | G | 0.077 | 0.017 | 8.84E-06 | 19.691 |
| family | Lachnospiraceae | rs1205445 | G | A | 0.050 | 0.011 | 9.14E-06 | 19.690 |
| family | Porphyromonadaceae | rs2851520 | T | C | 0.065 | 0.015 | 8.21E-06 | 19.689 |
| family | Desulfovibrionaceae | rs7164160 | T | A | -0.057 | 0.013 | 7.34E-06 | 19.687 |
| family | Ruminococcaceae | rs10166469 | C | T | -0.053 | 0.012 | 8.52E-06 | 19.686 |
| family | Alcaligenaceae | rs147968 | T | C | 0.049 | 0.011 | 9.13E-06 | 19.685 |
| family | Christensenellaceae | rs7211194 | T | C | 0.049 | 0.011 | 9.94E-06 | 19.684 |
| family | Streptococcaceae | rs28510567 | A | G | -0.049 | 0.011 | 9.63E-06 | 19.683 |
| family | ClostridialesvadinBB60group | rs276172 | A | G | 0.062 | 0.014 | 9.88E-06 | 19.683 |
| family | Ruminococcaceae | rs76724913 | G | T | 0.090 | 0.020 | 9.60E-06 | 19.682 |
| family | Coriobacteriaceae | rs1397793 | A | G | -0.050 | 0.011 | 9.77E-06 | 19.681 |
| family | Streptococcaceae | rs62077966 | C | T | -0.049 | 0.011 | 8.16E-06 | 19.680 |
| family | Actinomycetaceae | rs35110659 | G | T | 0.076 | 0.017 | 9.97E-06 | 19.673 |
| family | Streptococcaceae | rs239363 | G | A | -0.049 | 0.011 | 9.19E-06 | 19.671 |
| family | Rikenellaceae | rs6744030 | T | C | 0.070 | 0.016 | 9.32E-06 | 19.669 |
| family | Defluviitaleaceae | rs76603889 | G | A | -0.107 | 0.024 | 7.61E-06 | 19.669 |
| family | Verrucomicrobiaceae | rs4107636 | G | C | 0.063 | 0.014 | 7.97E-06 | 19.668 |
| family | Lactobacillaceae | rs7832262 | G | A | -0.076 | 0.017 | 9.53E-06 | 19.667 |
| family | Veillonellaceae | rs11633014 | C | T | -0.053 | 0.012 | 9.62E-06 | 19.666 |
| family | Bifidobacteriaceae | rs6711997 | T | C | 0.062 | 0.014 | 5.10E-06 | 19.666 |
| family | Desulfovibrionaceae | rs62020470 | G | A | -0.057 | 0.013 | 7.38E-06 | 19.664 |
| family | Rikenellaceae | rs8132143 | A | T | -0.047 | 0.011 | 9.41E-06 | 19.663 |
| family | Actinomycetaceae | rs11055874 | C | T | 0.077 | 0.017 | 9.14E-06 | 19.662 |
| family | Erysipelotrichaceae | rs61865709 | G | A | -0.053 | 0.012 | 8.00E-06 | 19.660 |
| family | Rhodospirillaceae | rs1414885 | C | T | 0.070 | 0.016 | 8.43E-06 | 19.659 |
| family | Peptostreptococcaceae | rs12993096 | C | T | -0.057 | 0.013 | 6.31E-06 | 19.659 |
| family | Bacteroidaceae | rs17788984 | A | G | 0.075 | 0.017 | 8.47E-06 | 19.658 |
| family | Lachnospiraceae | rs34072724 | G | A | -0.047 | 0.011 | 9.04E-06 | 19.658 |
| family | Verrucomicrobiaceae | rs72663802 | C | T | 0.064 | 0.015 | 8.67E-06 | 19.654 |
| family | Enterobacteriaceae | rs35673018 | A | G | 0.090 | 0.020 | 7.63E-06 | 19.653 |
| family | Streptococcaceae | rs2975675 | T | C | -0.068 | 0.015 | 6.83E-06 | 19.653 |
| family | Methanobacteriaceae | rs4753222 | G | A | 0.111 | 0.025 | 7.45E-06 | 19.651 |
| family | Veillonellaceae | rs4263802 | A | G | 0.051 | 0.011 | 7.45E-06 | 19.649 |
| family | Victivallaceae | rs4764863 | A | G | 0.100 | 0.023 | 9.12E-06 | 19.649 |
| family | Bifidobacteriaceae | rs10928519 | C | T | 0.053 | 0.012 | 6.26E-06 | 19.645 |
| family | Lachnospiraceae | rs7359921 | T | C | -0.050 | 0.011 | 7.06E-06 | 19.641 |
| family | Coriobacteriaceae | rs7540303 | T | C | 0.048 | 0.011 | 9.62E-06 | 19.632 |
| family | Coriobacteriaceae | rs60426889 | G | T | -0.050 | 0.011 | 8.69E-06 | 19.632 |
| family | Verrucomicrobiaceae | rs736124 | T | A | -0.074 | 0.017 | 8.58E-06 | 19.631 |
| family | Actinomycetaceae | rs7133400 | C | G | 0.077 | 0.017 | 9.12E-06 | 19.630 |
| family | BacteroidalesS24 | rs78609301 | G | A | -0.087 | 0.020 | 7.09E-06 | 19.628 |
| family | BacteroidalesS24 | rs56733378 | C | T | 0.121 | 0.027 | 9.89E-06 | 19.626 |
| family | Verrucomicrobiaceae | rs12411321 | T | C | 0.074 | 0.017 | 7.37E-06 | 19.624 |
| family | Rhodospirillaceae | rs80346366 | C | T | -0.099 | 0.022 | 7.25E-06 | 19.617 |
| family | Prevotellaceae | rs10131942 | G | C | 0.070 | 0.016 | 8.61E-06 | 19.615 |
| family | Enterobacteriaceae | rs79757635 | A | C | 0.076 | 0.017 | 9.32E-06 | 19.615 |
| family | Streptococcaceae | rs57646748 | A | G | -0.088 | 0.020 | 7.88E-06 | 19.612 |
| family | Oxalobacteraceae | rs7616207 | G | A | 0.097 | 0.022 | 9.25E-06 | 19.611 |
| family | Streptococcaceae | rs4968776 | G | A | 0.050 | 0.011 | 8.30E-06 | 19.607 |
| family | Verrucomicrobiaceae | rs4600885 | A | C | 0.063 | 0.014 | 8.12E-06 | 19.598 |
| family | Oxalobacteraceae | rs80330081 | C | A | -0.188 | 0.042 | 6.64E-06 | 19.597 |
| family | Alcaligenaceae | rs74776516 | G | T | -0.094 | 0.021 | 6.85E-06 | 19.591 |
| family | Bifidobacteriaceae | rs7174549 | T | C | 0.055 | 0.012 | 6.87E-06 | 19.590 |
| family | Christensenellaceae | rs7343112 | C | T | 0.048 | 0.011 | 9.84E-06 | 19.586 |
| family | Rhodospirillaceae | rs12771052 | A | G | 0.070 | 0.016 | 8.89E-06 | 19.583 |
| family | Clostridiaceae1 | rs6784441 | T | C | -0.061 | 0.014 | 9.67E-06 | 19.577 |
| family | Verrucomicrobiaceae | rs72663751 | G | A | 0.065 | 0.015 | 8.08E-06 | 19.577 |
| family | Veillonellaceae | rs11700976 | A | C | 0.050 | 0.011 | 9.25E-06 | 19.576 |
| family | Defluviitaleaceae | rs2189340 | G | A | -0.107 | 0.024 | 9.96E-06 | 19.575 |
| family | Streptococcaceae | rs10826446 | T | C | 0.095 | 0.022 | 7.11E-06 | 19.567 |
| family | Coriobacteriaceae | rs3025411 | G | A | 0.093 | 0.021 | 8.27E-06 | 19.566 |
| family | Streptococcaceae | rs4261605 | C | T | -0.049 | 0.011 | 9.75E-06 | 19.559 |
| family | Rhodospirillaceae | rs72714481 | A | G | 0.114 | 0.026 | 7.78E-06 | 19.559 |
| family | FamilyXIII | rs17040587 | A | C | -0.060 | 0.013 | 6.27E-06 | 19.559 |
| family | Streptococcaceae | rs57226474 | G | A | -0.049 | 0.011 | 8.41E-06 | 19.558 |
| family | Rhodospirillaceae | rs72714475 | G | C | 0.114 | 0.026 | 9.33E-06 | 19.556 |
| family | Clostridiaceae1 | rs2156323 | G | A | 0.080 | 0.018 | 8.40E-06 | 19.555 |
| family | Erysipelotrichaceae | rs2638904 | A | G | 0.047 | 0.011 | 9.64E-06 | 19.553 |
| family | FamilyXIII | rs12904405 | G | A | -0.085 | 0.019 | 7.67E-06 | 19.546 |
| family | Bifidobacteriaceae | rs669759 | T | C | -0.054 | 0.012 | 5.33E-06 | 19.545 |
| family | Rhodospirillaceae | rs67973976 | T | C | 0.116 | 0.026 | 7.23E-06 | 19.543 |
| family | Veillonellaceae | rs7814375 | A | C | 0.051 | 0.011 | 7.87E-06 | 19.542 |
| family | Bifidobacteriaceae | rs677208 | C | G | 0.073 | 0.016 | 6.00E-06 | 19.541 |
| family | Bifidobacteriaceae | rs13424016 | G | A | 0.053 | 0.012 | 9.54E-06 | 19.539 |
| family | ClostridialesvadinBB60group | rs740990 | C | T | 0.071 | 0.016 | 9.31E-06 | 19.529 |
| family | FamilyXIII | rs17366206 | T | G | -0.066 | 0.015 | 8.12E-06 | 19.524 |
| family | Streptococcaceae | rs28718126 | G | A | 0.109 | 0.025 | 9.41E-06 | 19.520 |
| family | Alcaligenaceae | rs4354244 | C | T | -0.056 | 0.013 | 9.92E-06 | 19.519 |
| family | Bifidobacteriaceae | rs11688302 | C | T | 0.057 | 0.013 | 8.32E-06 | 19.515 |
| family | Streptococcaceae | rs77968078 | A | G | -0.099 | 0.022 | 7.93E-06 | 19.515 |
| family | Rhodospirillaceae | rs12771420 | A | T | 0.070 | 0.016 | 9.05E-06 | 19.514 |
| family | ClostridialesvadinBB60group | rs6755871 | G | C | -0.061 | 0.014 | 9.33E-06 | 19.508 |
| family | Streptococcaceae | rs10826445 | G | A | 0.095 | 0.022 | 7.24E-06 | 19.499 |
| family | Coriobacteriaceae | rs62448870 | A | G | -0.048 | 0.011 | 9.50E-06 | 19.495 |
| family | Bifidobacteriaceae | rs10496759 | C | G | 0.064 | 0.014 | 8.80E-06 | 19.494 |
| family | Porphyromonadaceae | rs35961441 | C | A | 0.092 | 0.021 | 8.37E-06 | 19.492 |
| family | Ruminococcaceae | rs3009418 | A | C | 0.093 | 0.021 | 8.69E-06 | 19.489 |
| family | Alcaligenaceae | rs8000825 | G | A | -0.049 | 0.011 | 8.99E-06 | 19.487 |
| family | Veillonellaceae | rs4909362 | C | T | 0.051 | 0.011 | 7.82E-06 | 19.472 |
| family | Enterobacteriaceae | rs11026530 | C | T | 0.082 | 0.019 | 9.43E-06 | 19.471 |
| family | Rhodospirillaceae | rs12773098 | T | C | 0.070 | 0.016 | 9.27E-06 | 19.468 |
| family | Bifidobacteriaceae | rs1942051 | A | G | 0.053 | 0.012 | 6.80E-06 | 19.468 |
| family | Veillonellaceae | rs10086225 | G | A | 0.051 | 0.011 | 7.97E-06 | 19.468 |
| family | Alcaligenaceae | rs2613606 | T | C | -0.049 | 0.011 | 8.89E-06 | 19.467 |
| family | Alcaligenaceae | rs1344064 | C | T | -0.049 | 0.011 | 9.18E-06 | 19.466 |
| family | Alcaligenaceae | rs6650396 | A | G | -0.049 | 0.011 | 9.12E-06 | 19.465 |
| family | Oxalobacteraceae | rs79806081 | G | A | -0.187 | 0.042 | 7.08E-06 | 19.463 |
| family | Bacteroidaceae | rs1340392 | G | A | -0.058 | 0.013 | 9.60E-06 | 19.463 |
| family | Coriobacteriaceae | rs7912120 | G | A | 0.072 | 0.016 | 6.24E-06 | 19.451 |
| family | Alcaligenaceae | rs9537817 | A | C | -0.049 | 0.011 | 9.31E-06 | 19.445 |
| family | Pasteurellaceae | rs8125536 | C | T | -0.066 | 0.015 | 9.93E-06 | 19.445 |
| family | Rhodospirillaceae | rs2095588 | T | C | 0.070 | 0.016 | 9.78E-06 | 19.439 |
| family | Lachnospiraceae | rs889359 | C | T | -0.050 | 0.011 | 8.16E-06 | 19.432 |
| family | Alcaligenaceae | rs9537819 | A | G | -0.049 | 0.011 | 9.19E-06 | 19.428 |
| family | Porphyromonadaceae | rs10762312 | A | G | -0.052 | 0.012 | 8.70E-06 | 19.427 |
| family | Rhodospirillaceae | rs71481160 | C | T | 0.070 | 0.016 | 9.64E-06 | 19.424 |
| family | Christensenellaceae | rs5752916 | A | G | -0.066 | 0.015 | 8.29E-06 | 19.423 |
| family | Rhodospirillaceae | rs72714480 | C | T | 0.113 | 0.026 | 8.35E-06 | 19.421 |
| family | Veillonellaceae | rs10086221 | T | A | 0.051 | 0.011 | 8.22E-06 | 19.419 |
| family | Verrucomicrobiaceae | rs12904881 | G | A | 0.058 | 0.013 | 9.27E-06 | 19.419 |
| family | Streptococcaceae | rs28587507 | C | G | -0.073 | 0.016 | 9.18E-06 | 19.413 |
| family | Oxalobacteraceae | rs57363691 | A | T | -0.187 | 0.042 | 7.50E-06 | 19.413 |
| family | Oxalobacteraceae | rs12002250 | C | A | 0.196 | 0.045 | 5.53E-06 | 19.408 |
| family | Oxalobacteraceae | rs12004269 | T | G | 0.196 | 0.045 | 5.53E-06 | 19.408 |
| family | Oxalobacteraceae | rs62304913 | C | T | -0.193 | 0.044 | 7.17E-06 | 19.395 |
| family | FamilyXIII | rs34319585 | T | C | -0.060 | 0.014 | 8.51E-06 | 19.394 |
| family | FamilyXIII | rs34246808 | A | G | -0.060 | 0.014 | 8.51E-06 | 19.394 |
| family | Coriobacteriaceae | rs11656361 | C | A | 0.077 | 0.018 | 8.02E-06 | 19.394 |
| family | Alcaligenaceae | rs2320923 | G | A | -0.049 | 0.011 | 9.56E-06 | 19.391 |
| family | Lachnospiraceae | rs140737552 | C | T | 0.055 | 0.013 | 8.30E-06 | 19.388 |
| family | Oxalobacteraceae | rs17138946 | T | G | -0.189 | 0.043 | 8.09E-06 | 19.388 |
| family | Prevotellaceae | rs11045907 | A | T | -0.060 | 0.014 | 9.00E-06 | 19.387 |
| family | Erysipelotrichaceae | rs56970041 | G | T | 0.072 | 0.016 | 5.40E-06 | 19.385 |
| family | Pasteurellaceae | rs12050685 | G | A | -0.067 | 0.015 | 9.19E-06 | 19.385 |
| family | Ruminococcaceae | rs499272 | C | G | 0.071 | 0.016 | 9.29E-06 | 19.382 |
| family | Streptococcaceae | rs10111917 | C | T | -0.073 | 0.016 | 9.32E-06 | 19.381 |
| family | Oxalobacteraceae | rs62304910 | G | A | -0.186 | 0.042 | 7.27E-06 | 19.378 |
| family | Verrucomicrobiaceae | rs10005131 | T | G | 0.061 | 0.014 | 9.83E-06 | 19.376 |
| family | Streptococcaceae | rs7901835 | T | C | 0.095 | 0.022 | 8.03E-06 | 19.373 |
| family | Rhodospirillaceae | rs80344265 | C | T | -0.099 | 0.022 | 8.17E-06 | 19.369 |
| family | Alcaligenaceae | rs2906622 | T | C | -0.049 | 0.011 | 9.79E-06 | 19.367 |
| family | Verrucomicrobiaceae | rs28858161 | T | C | 0.062 | 0.014 | 9.24E-06 | 19.355 |
| family | Methanobacteriaceae | rs3912664 | G | T | 0.110 | 0.025 | 8.67E-06 | 19.355 |
| family | Prevotellaceae | rs11045908 | G | A | -0.060 | 0.014 | 9.12E-06 | 19.353 |
| family | Verrucomicrobiaceae | rs28811282 | G | A | 0.062 | 0.014 | 9.27E-06 | 19.349 |
| family | Rhodospirillaceae | rs12518525 | A | G | -0.110 | 0.025 | 9.78E-06 | 19.347 |
| family | Verrucomicrobiaceae | rs1988252 | G | A | 0.062 | 0.014 | 9.56E-06 | 19.347 |
| family | FamilyXIII | rs35924848 | A | C | -0.065 | 0.015 | 9.95E-06 | 19.341 |
| family | Lachnospiraceae | rs10852797 | C | T | 0.053 | 0.012 | 7.44E-06 | 19.340 |
| family | Christensenellaceae | rs6471946 | G | A | 0.048 | 0.011 | 9.87E-06 | 19.334 |
| family | FamilyXIII | rs13143795 | C | A | -0.059 | 0.014 | 8.24E-06 | 19.332 |
| family | Clostridiaceae1 | rs4723021 | C | T | -0.106 | 0.024 | 7.42E-06 | 19.331 |
| family | Rikenellaceae | rs61741563 | G | A | -0.148 | 0.034 | 9.13E-06 | 19.329 |
| family | Bifidobacteriaceae | rs2349959 | A | T | 0.056 | 0.013 | 9.28E-06 | 19.327 |
| family | Rhodospirillaceae | rs11944431 | G | A | -0.099 | 0.022 | 8.19E-06 | 19.326 |
| family | Rhodospirillaceae | rs56021351 | G | C | 0.104 | 0.024 | 6.71E-06 | 19.326 |
| family | Lachnospiraceae | rs1991016 | C | T | -0.049 | 0.011 | 8.19E-06 | 19.322 |
| family | Peptostreptococcaceae | rs10904854 | A | G | 0.053 | 0.012 | 8.61E-06 | 19.321 |
| family | Rhodospirillaceae | rs1923415 | G | A | -0.100 | 0.023 | 9.64E-06 | 19.321 |
| family | Bacteroidaceae | rs79325005 | A | G | 0.057 | 0.013 | 8.58E-06 | 19.317 |
| family | Victivallaceae | rs7860510 | C | G | 0.165 | 0.038 | 9.60E-06 | 19.313 |
| family | Rikenellaceae | rs61424289 | T | C | -0.087 | 0.020 | 8.88E-06 | 19.310 |
| family | FamilyXIII | rs34495163 | A | G | -0.059 | 0.014 | 8.88E-06 | 19.309 |
| family | FamilyXI | rs78222136 | C | G | 0.225 | 0.051 | 8.41E-06 | 19.308 |
| family | Alcaligenaceae | rs2320924 | T | C | -0.049 | 0.011 | 9.99E-06 | 19.307 |
| family | Streptococcaceae | rs7916740 | C | T | 0.095 | 0.022 | 8.25E-06 | 19.303 |
| family | Coriobacteriaceae | rs1834859 | T | G | -0.057 | 0.013 | 7.97E-06 | 19.301 |
| family | Methanobacteriaceae | rs12825290 | G | C | -0.217 | 0.049 | 6.08E-06 | 19.296 |
| family | Verrucomicrobiaceae | rs941682 | A | G | -0.063 | 0.014 | 9.58E-06 | 19.296 |
| family | Veillonellaceae | rs73557284 | G | A | 0.205 | 0.047 | 7.16E-06 | 19.292 |
| family | Verrucomicrobiaceae | rs2865331 | G | A | 0.062 | 0.014 | 9.62E-06 | 19.291 |
| family | Desulfovibrionaceae | rs7163129 | T | C | -0.057 | 0.013 | 9.07E-06 | 19.284 |
| family | Alcaligenaceae | rs9520346 | G | C | 0.147 | 0.034 | 4.56E-06 | 19.280 |
| family | Rikenellaceae | rs12584670 | T | C | 0.082 | 0.019 | 8.50E-06 | 19.278 |
| family | Bifidobacteriaceae | rs1879087 | T | C | 0.105 | 0.024 | 1.05E-06 | 19.266 |
| family | FamilyXIII | rs62326992 | G | T | -0.059 | 0.013 | 7.60E-06 | 19.263 |
| family | Bifidobacteriaceae | rs676239 | A | T | 0.072 | 0.016 | 6.80E-06 | 19.258 |
| family | Rhodospirillaceae | rs72714479 | G | A | 0.113 | 0.026 | 8.56E-06 | 19.252 |
| family | Rhodospirillaceae | rs11945729 | G | A | -0.099 | 0.022 | 8.61E-06 | 19.241 |
| family | Rhodospirillaceae | rs1593368 | T | C | -0.098 | 0.022 | 9.08E-06 | 19.241 |
| family | Methanobacteriaceae | rs11018664 | G | A | 0.110 | 0.025 | 9.00E-06 | 19.239 |
| family | Bacteroidaceae | rs78280928 | G | A | 0.057 | 0.013 | 8.95E-06 | 19.234 |
| family | Methanobacteriaceae | rs4385899 | C | T | 0.110 | 0.025 | 9.33E-06 | 19.233 |
| family | Methanobacteriaceae | rs10830292 | T | G | 0.110 | 0.025 | 9.56E-06 | 19.227 |
| family | Oxalobacteraceae | rs62304912 | G | A | -0.185 | 0.042 | 8.29E-06 | 19.201 |
| family | FamilyXIII | rs11490084 | G | A | -0.059 | 0.014 | 8.63E-06 | 19.199 |
| family | Rhodospirillaceae | rs61350907 | T | A | -0.098 | 0.022 | 8.90E-06 | 19.188 |
| family | Veillonellaceae | rs11166707 | G | C | 0.050 | 0.011 | 9.61E-06 | 19.180 |
| family | Porphyromonadaceae | rs7873444 | G | C | -0.061 | 0.014 | 7.49E-06 | 19.169 |
| family | Porphyromonadaceae | rs115602804 | A | G | 0.095 | 0.022 | 6.65E-06 | 19.166 |
| family | Rikenellaceae | rs4783173 | G | C | 0.048 | 0.011 | 7.06E-06 | 19.160 |
| family | Rhodospirillaceae | rs79569783 | G | A | -0.099 | 0.023 | 9.19E-06 | 19.152 |
| family | Rhodospirillaceae | rs17022723 | T | C | -0.098 | 0.022 | 9.01E-06 | 19.151 |
| family | Porphyromonadaceae | rs1003404 | C | T | 0.064 | 0.015 | 9.70E-06 | 19.150 |
| family | ClostridialesvadinBB60group | rs34088226 | G | A | -0.118 | 0.027 | 7.66E-06 | 19.145 |
| family | Porphyromonadaceae | rs7330827 | C | T | -0.104 | 0.024 | 8.05E-06 | 19.140 |
| family | Desulfovibrionaceae | rs7162535 | T | C | -0.057 | 0.013 | 9.89E-06 | 19.124 |
| family | FamilyXIII | rs13128396 | C | G | -0.059 | 0.014 | 9.01E-06 | 19.117 |
| family | Bifidobacteriaceae | rs4954187 | C | T | 0.052 | 0.012 | 8.71E-06 | 19.115 |
| family | Methanobacteriaceae | rs10501707 | A | G | 0.110 | 0.025 | 9.72E-06 | 19.112 |
| family | Victivallaceae | rs111911665 | G | A | -0.180 | 0.041 | 5.51E-06 | 19.105 |
| family | FamilyXIII | rs13147741 | G | A | -0.059 | 0.014 | 9.26E-06 | 19.104 |
| family | Oxalobacteraceae | rs62304911 | T | G | -0.185 | 0.042 | 8.68E-06 | 19.104 |
| family | Rhodospirillaceae | rs7078649 | C | A | 0.105 | 0.024 | 8.17E-06 | 19.093 |
| family | Streptococcaceae | rs73195303 | C | T | -0.058 | 0.013 | 9.52E-06 | 19.092 |
| family | FamilyXIII | rs36010286 | C | T | -0.059 | 0.014 | 9.73E-06 | 19.091 |
| family | FamilyXIII | rs35190754 | T | A | -0.059 | 0.014 | 9.26E-06 | 19.089 |
| family | Porphyromonadaceae | rs114875383 | C | T | 0.095 | 0.022 | 6.93E-06 | 19.082 |
| family | FamilyXIII | rs17374483 | C | T | -0.059 | 0.014 | 9.36E-06 | 19.070 |
| family | FamilyXIII | rs35404408 | C | T | -0.059 | 0.014 | 9.36E-06 | 19.070 |
| family | Lactobacillaceae | rs1530559 | A | G | 0.077 | 0.018 | 9.65E-06 | 19.063 |
| family | Lachnospiraceae | rs889344 | C | G | -0.049 | 0.011 | 8.93E-06 | 19.060 |
| family | Coriobacteriaceae | rs7896306 | T | G | 0.072 | 0.016 | 7.82E-06 | 19.059 |
| family | FamilyXIII | rs36018865 | G | A | -0.059 | 0.014 | 9.26E-06 | 19.054 |
| family | Clostridiaceae1 | rs550843 | C | T | -0.073 | 0.017 | 7.09E-06 | 19.043 |
| family | FamilyXIII | rs34664888 | C | T | -0.059 | 0.013 | 8.22E-06 | 19.042 |
| family | Lachnospiraceae | rs11737564 | A | G | -0.056 | 0.013 | 9.05E-06 | 19.041 |
| family | FamilyXIII | rs62326988 | T | A | -0.059 | 0.014 | 9.57E-06 | 19.039 |
| family | Streptococcaceae | rs11110077 | C | G | -0.077 | 0.018 | 9.43E-06 | 19.034 |
| family | Pasteurellaceae | rs16970009 | G | A | 0.187 | 0.043 | 7.32E-06 | 19.028 |
| family | Rikenellaceae | rs10832801 | C | A | -0.053 | 0.012 | 7.50E-06 | 19.005 |
| family | FamilyXIII | rs12186153 | T | G | -0.059 | 0.014 | 9.42E-06 | 18.994 |
| family | Veillonellaceae | rs61264131 | C | A | 0.202 | 0.046 | 6.75E-06 | 18.990 |
| family | FamilyXIII | rs13148852 | A | G | -0.059 | 0.013 | 8.80E-06 | 18.987 |
| family | FamilyXIII | rs35239169 | T | C | -0.059 | 0.013 | 8.34E-06 | 18.982 |
| family | Bifidobacteriaceae | rs659440 | T | A | 0.072 | 0.016 | 7.93E-06 | 18.981 |
| family | Oxalobacteraceae | rs17788332 | A | G | -0.185 | 0.042 | 9.05E-06 | 18.973 |
| family | FamilyXIII | rs35248316 | G | A | -0.059 | 0.013 | 8.49E-06 | 18.972 |
| family | Oxalobacteraceae | rs17731697 | T | C | -0.185 | 0.042 | 9.09E-06 | 18.965 |
| family | Oxalobacteraceae | rs62304905 | T | C | -0.185 | 0.042 | 9.09E-06 | 18.965 |
| family | Oxalobacteraceae | rs79967238 | G | A | -0.184 | 0.042 | 9.70E-06 | 18.953 |
| family | FamilyXIII | rs11937660 | T | A | -0.059 | 0.013 | 8.80E-06 | 18.951 |
| family | FamilyXIII | rs34915491 | G | T | -0.059 | 0.013 | 8.76E-06 | 18.947 |
| family | Oxalobacteraceae | rs79615800 | C | T | -0.186 | 0.043 | 9.32E-06 | 18.940 |
| family | FamilyXIII | rs17301876 | T | G | -0.059 | 0.013 | 9.08E-06 | 18.935 |
| family | FamilyXIII | rs36049157 | G | C | -0.059 | 0.014 | 9.86E-06 | 18.921 |
| family | FamilyXIII | rs36068795 | C | T | -0.059 | 0.014 | 9.95E-06 | 18.918 |
| family | FamilyXIII | rs34402008 | A | G | -0.059 | 0.013 | 9.59E-06 | 18.913 |
| family | Rhodospirillaceae | rs11598665 | G | A | 0.104 | 0.024 | 9.02E-06 | 18.913 |
| family | Bifidobacteriaceae | rs4954189 | G | A | 0.052 | 0.012 | 9.83E-06 | 18.909 |
| family | Lachnospiraceae | rs4744919 | T | C | 0.049 | 0.011 | 7.10E-06 | 18.885 |
| family | Peptostreptococcaceae | rs77540684 | G | T | 0.107 | 0.025 | 8.14E-06 | 18.877 |
| family | Bifidobacteriaceae | rs10831953 | A | G | 0.054 | 0.012 | 9.95E-06 | 18.869 |
| family | Rhodospirillaceae | rs28406547 | C | T | 0.104 | 0.024 | 9.66E-06 | 18.787 |
| family | Bifidobacteriaceae | rs558520 | T | C | 0.071 | 0.016 | 8.95E-06 | 18.755 |
| family | FamilyXIII | rs7677453 | G | C | -0.058 | 0.013 | 9.78E-06 | 18.722 |
| family | Lachnospiraceae | rs7350265 | G | T | 0.048 | 0.011 | 7.97E-06 | 18.691 |
| family | Erysipelotrichaceae | rs10134361 | G | A | 0.071 | 0.016 | 8.66E-06 | 18.642 |
| family | Bifidobacteriaceae | rs6707350 | C | T | 0.081 | 0.019 | 7.70E-06 | 18.554 |
| family | Rikenellaceae | rs2450745 | C | A | -0.079 | 0.018 | 8.61E-06 | 18.523 |
| family | Pasteurellaceae | rs9895850 | C | T | -0.176 | 0.041 | 9.08E-06 | 18.497 |
| family | Porphyromonadaceae | rs7045985 | G | A | -0.060 | 0.014 | 9.27E-06 | 18.480 |
| family | Bifidobacteriaceae | rs11674937 | G | C | 0.060 | 0.014 | 9.88E-06 | 18.448 |
| family | Clostridiaceae1 | rs6934446 | G | A | -0.072 | 0.017 | 9.28E-06 | 18.405 |
| family | Defluviitaleaceae | rs540220 | C | T | -0.124 | 0.029 | 9.48E-06 | 18.293 |
| family | Rhodospirillaceae | rs78250451 | T | A | -0.085 | 0.020 | 9.55E-06 | 18.281 |
| family | Erysipelotrichaceae | rs10144017 | G | A | 0.069 | 0.016 | 8.50E-06 | 18.267 |
| family | Bifidobacteriaceae | rs7572152 | C | G | 0.081 | 0.019 | 8.42E-06 | 18.201 |
| family | Bifidobacteriaceae | rs4954218 | T | G | -0.059 | 0.014 | 6.71E-06 | 18.113 |
| family | Veillonellaceae | rs10013922 | T | G | 0.055 | 0.013 | 7.25E-06 | 18.094 |
| family | Bifidobacteriaceae | rs12446429 | C | T | 0.081 | 0.019 | 8.53E-06 | 18.040 |
| family | Christensenellaceae | rs77867022 | T | G | -0.160 | 0.038 | 8.82E-06 | 17.866 |
| family | Ruminococcaceae | rs2086165 | C | T | 0.154 | 0.036 | 9.60E-06 | 17.850 |
| family | Victivallaceae | rs17021086 | T | C | -0.175 | 0.042 | 9.62E-06 | 17.807 |
| family | Peptostreptococcaceae | rs3012501 | C | G | 0.058 | 0.014 | 9.11E-06 | 17.791 |
| family | Erysipelotrichaceae | rs7826267 | G | T | -0.084 | 0.020 | 9.28E-06 | 17.755 |
| family | FamilyXIII | rs4293737 | G | A | -0.112 | 0.027 | 8.38E-06 | 17.070 |
| family | Lachnospiraceae | rs11841382 | T | G | -0.072 | 0.017 | 9.58E-06 | 16.913 |
| genus | Bifidobacterium | rs182549 | T | C | 0.120 | 0.013 | 1.28E-20 | 88.430 |
| genus | Bifidobacterium | rs4988235 | A | G | 0.119 | 0.013 | 2.49E-20 | 87.276 |
| genus | Bifidobacterium | rs6754311 | T | C | 0.117 | 0.013 | 6.96E-20 | 84.807 |
| genus | Bifidobacterium | rs7570971 | C | A | 0.113 | 0.013 | 2.01E-19 | 81.233 |
| genus | Bifidobacterium | rs1375131 | T | C | 0.112 | 0.013 | 1.02E-18 | 79.652 |
| genus | Bifidobacterium | rs1446585 | A | G | 0.114 | 0.013 | 1.94E-18 | 78.814 |
| genus | Bifidobacterium | rs6730157 | A | G | 0.111 | 0.013 | 1.72E-18 | 78.498 |
| genus | Bifidobacterium | rs12465802 | G | A | 0.112 | 0.013 | 1.73E-18 | 78.276 |
| genus | Bifidobacterium | rs687670 | T | C | 0.111 | 0.013 | 2.60E-17 | 73.813 |
| genus | Bifidobacterium | rs13404551 | T | C | 0.110 | 0.013 | 6.82E-17 | 72.636 |
| genus | Bifidobacterium | rs309160 | C | T | 0.108 | 0.013 | 3.68E-16 | 68.951 |
| genus | Bifidobacterium | rs309168 | C | T | 0.107 | 0.013 | 6.85E-16 | 67.700 |
| genus | Bifidobacterium | rs36084664 | A | G | -0.111 | 0.014 | 7.39E-18 | 67.428 |
| genus | Bifidobacterium | rs218174 | A | G | 0.106 | 0.013 | 1.06E-15 | 67.303 |
| genus | Bifidobacterium | rs309134 | G | A | 0.107 | 0.013 | 1.32E-15 | 66.754 |
| genus | Bifidobacterium | rs309180 | A | G | 0.105 | 0.013 | 3.11E-15 | 64.741 |
| genus | Bifidobacterium | rs12615624 | A | G | 0.105 | 0.013 | 5.81E-15 | 63.838 |
| genus | Bifidobacterium | rs6759321 | G | T | 0.102 | 0.013 | 4.60E-15 | 63.620 |
| genus | Bifidobacterium | rs632632 | T | C | 0.103 | 0.013 | 6.21E-15 | 63.554 |
| genus | Bifidobacterium | rs309125 | C | T | 0.102 | 0.013 | 1.11E-14 | 62.233 |
| genus | Bifidobacterium | rs6716536 | C | T | 0.101 | 0.013 | 7.72E-15 | 62.212 |
| genus | Bifidobacterium | rs309164 | T | C | 0.103 | 0.013 | 1.85E-14 | 61.327 |
| genus | Bifidobacterium | rs1561277 | C | A | 0.100 | 0.013 | 1.60E-14 | 61.299 |
| genus | Bifidobacterium | rs309149 | C | T | 0.102 | 0.013 | 4.14E-14 | 59.580 |
| genus | Bifidobacterium | rs11681248 | T | C | 0.096 | 0.012 | 3.09E-14 | 59.343 |
| genus | Bifidobacterium | rs309137 | T | C | 0.100 | 0.013 | 4.04E-14 | 59.217 |
| genus | Bifidobacterium | rs13023281 | T | C | -0.104 | 0.013 | 7.23E-16 | 59.011 |
| genus | Bifidobacterium | rs4988183 | T | C | 0.101 | 0.013 | 8.17E-14 | 58.486 |
| genus | Bifidobacterium | rs4954490 | A | G | 0.100 | 0.013 | 8.13E-14 | 58.167 |
| genus | Bifidobacterium | rs12475139 | T | A | 0.097 | 0.013 | 1.33E-13 | 56.511 |
| genus | Bifidobacterium | rs2236783 | A | G | 0.098 | 0.013 | 2.16E-13 | 56.416 |
| genus | Bifidobacterium | rs12620033 | G | C | 0.097 | 0.013 | 3.36E-13 | 55.470 |
| genus | Bifidobacterium | rs749873 | T | C | 0.093 | 0.012 | 3.09E-13 | 54.874 |
| genus | Bifidobacterium | rs3754686 | C | T | 0.096 | 0.013 | 5.30E-13 | 54.234 |
| genus | Bifidobacterium | rs2011946 | A | C | 0.092 | 0.013 | 5.60E-13 | 53.868 |
| genus | Bifidobacterium | rs6719488 | T | G | 0.095 | 0.013 | 1.04E-12 | 52.972 |
| genus | Bifidobacterium | rs892715 | C | T | 0.095 | 0.013 | 1.08E-12 | 52.886 |
| genus | Bifidobacterium | rs17261772 | T | C | 0.096 | 0.013 | 1.25E-12 | 52.857 |
| genus | Bifidobacterium | rs3769005 | G | C | 0.095 | 0.013 | 9.71E-13 | 52.854 |
| genus | Bifidobacterium | rs745500 | A | G | 0.095 | 0.013 | 1.16E-12 | 52.826 |
| genus | Bifidobacterium | rs12988076 | A | C | 0.095 | 0.013 | 1.47E-12 | 52.311 |
| genus | Bifidobacterium | rs6742283 | G | A | 0.095 | 0.013 | 1.50E-12 | 52.237 |
| genus | Bifidobacterium | rs2322660 | T | C | 0.094 | 0.013 | 1.60E-12 | 52.048 |
| genus | Bifidobacterium | rs2164210 | C | T | 0.094 | 0.013 | 1.90E-12 | 51.666 |
| genus | Bifidobacterium | rs748841 | T | C | 0.094 | 0.013 | 2.11E-12 | 51.536 |
| genus | Bifidobacterium | rs11693502 | T | C | 0.090 | 0.013 | 1.71E-12 | 51.325 |
| genus | Bifidobacterium | rs3739020 | T | G | 0.095 | 0.013 | 3.84E-12 | 50.589 |
| genus | Bifidobacterium | rs2082729 | T | C | 0.094 | 0.013 | 5.73E-12 | 49.534 |
| genus | Bifidobacterium | rs11679933 | T | G | 0.089 | 0.013 | 4.80E-12 | 49.483 |
| genus | Bifidobacterium | rs1438305 | G | C | 0.093 | 0.013 | 6.39E-12 | 49.268 |
| genus | Bifidobacterium | rs1011361 | A | G | 0.093 | 0.013 | 6.57E-12 | 49.206 |
| genus | Bifidobacterium | rs71348714 | A | G | 0.093 | 0.013 | 6.39E-12 | 49.066 |
| genus | Bifidobacterium | rs12998016 | T | C | 0.093 | 0.013 | 7.30E-12 | 48.910 |
| genus | Bifidobacterium | rs313519 | A | C | 0.093 | 0.013 | 6.68E-12 | 48.860 |
| genus | Bifidobacterium | rs313526 | C | T | 0.093 | 0.013 | 6.94E-12 | 48.857 |
| genus | Bifidobacterium | rs10928546 | T | C | 0.093 | 0.013 | 7.97E-12 | 48.827 |
| genus | Bifidobacterium | rs313528 | G | A | 0.093 | 0.013 | 7.01E-12 | 48.811 |
| genus | Bifidobacterium | rs1438304 | G | A | 0.093 | 0.013 | 8.41E-12 | 48.712 |
| genus | Bifidobacterium | rs11684545 | T | G | 0.093 | 0.013 | 8.79E-12 | 48.592 |
| genus | Bifidobacterium | rs71348715 | T | G | 0.093 | 0.013 | 9.30E-12 | 48.547 |
| genus | Bifidobacterium | rs313518 | C | T | 0.092 | 0.013 | 8.83E-12 | 48.464 |
| genus | Bifidobacterium | rs6430589 | C | G | 0.093 | 0.013 | 8.22E-12 | 48.399 |
| genus | Bifidobacterium | rs28453840 | A | G | 0.093 | 0.013 | 9.95E-12 | 48.372 |
| genus | Bifidobacterium | rs13412397 | A | G | 0.092 | 0.013 | 1.03E-11 | 48.353 |
| genus | Bifidobacterium | rs10195620 | T | A | 0.093 | 0.013 | 1.03E-11 | 48.241 |
| genus | Bifidobacterium | rs7355359 | T | C | 0.092 | 0.013 | 9.81E-12 | 48.164 |
| genus | Bifidobacterium | rs1438307 | T | G | 0.092 | 0.013 | 1.17E-11 | 48.021 |
| genus | Bifidobacterium | rs313524 | C | T | 0.092 | 0.013 | 1.03E-11 | 48.010 |
| genus | Bifidobacterium | rs12998387 | G | A | 0.092 | 0.013 | 1.22E-11 | 47.983 |
| genus | Bifidobacterium | rs10202489 | A | G | 0.092 | 0.013 | 1.22E-11 | 47.974 |
| genus | Bifidobacterium | rs313517 | A | G | 0.092 | 0.013 | 1.19E-11 | 47.784 |
| genus | Bifidobacterium | rs11679508 | T | A | 0.092 | 0.013 | 1.40E-11 | 47.727 |
| genus | Bifidobacterium | rs1470457 | A | G | 0.092 | 0.013 | 1.53E-11 | 47.722 |
| genus | Bifidobacterium | rs3213889 | G | A | 0.092 | 0.013 | 1.58E-11 | 47.521 |
| genus | Bifidobacterium | rs1446584 | A | G | 0.091 | 0.013 | 1.35E-11 | 47.520 |
| genus | Bifidobacterium | rs4954280 | A | G | 0.091 | 0.013 | 1.38E-11 | 47.488 |
| genus | Bifidobacterium | rs6720287 | A | G | 0.091 | 0.013 | 1.36E-11 | 47.463 |
| genus | Bifidobacterium | rs2034276 | A | T | 0.091 | 0.013 | 1.39E-11 | 47.408 |
| genus | Bifidobacterium | rs10173394 | T | C | 0.092 | 0.013 | 1.78E-11 | 47.300 |
| genus | Bifidobacterium | rs6755383 | C | T | 0.091 | 0.013 | 1.49E-11 | 47.262 |
| genus | Bifidobacterium | rs313523 | G | A | 0.091 | 0.013 | 1.54E-11 | 47.177 |
| genus | Bifidobacterium | rs313522 | T | A | 0.091 | 0.013 | 1.59E-11 | 47.143 |
| genus | Bifidobacterium | rs12994270 | A | T | 0.091 | 0.013 | 2.01E-11 | 47.081 |
| genus | Bifidobacterium | rs6715856 | T | C | 0.091 | 0.013 | 1.83E-11 | 46.879 |
| genus | Bifidobacterium | rs656326 | A | C | 0.091 | 0.013 | 1.94E-11 | 46.715 |
| genus | Bifidobacterium | rs932206 | T | C | 0.083 | 0.012 | 1.18E-11 | 46.667 |
| genus | Bifidobacterium | rs12469709 | T | C | 0.091 | 0.013 | 2.24E-11 | 46.666 |
| genus | Bifidobacterium | rs10928542 | G | A | 0.090 | 0.013 | 2.10E-11 | 46.532 |
| genus | Bifidobacterium | rs7608045 | T | C | 0.090 | 0.013 | 2.42E-11 | 46.248 |
| genus | Bifidobacterium | rs4954276 | A | G | 0.090 | 0.013 | 2.98E-11 | 46.164 |
| genus | Bifidobacterium | rs10192827 | A | G | 0.090 | 0.013 | 3.74E-11 | 45.959 |
| genus | Bifidobacterium | rs4954279 | G | A | 0.090 | 0.013 | 2.84E-11 | 45.952 |
| genus | Bifidobacterium | rs12619365 | C | T | 0.090 | 0.013 | 3.77E-11 | 45.306 |
| genus | Bifidobacterium | rs13413639 | C | A | 0.089 | 0.013 | 4.36E-11 | 45.219 |
| genus | Bifidobacterium | rs2322659 | C | T | 0.090 | 0.013 | 4.47E-11 | 45.178 |
| genus | Bifidobacterium | rs2680880 | T | A | 0.080 | 0.012 | 1.69E-10 | 43.091 |
| genus | Bifidobacterium | rs1869829 | A | G | 0.086 | 0.013 | 9.60E-11 | 42.864 |
| genus | Bifidobacterium | rs1375132 | A | G | 0.086 | 0.013 | 1.40E-10 | 42.343 |
| genus | Bifidobacterium | rs1979033 | G | A | 0.078 | 0.012 | 2.40E-11 | 42.290 |
| genus | Bifidobacterium | rs11893318 | T | C | 0.115 | 0.018 | 6.54E-11 | 42.215 |
| genus | Bifidobacterium | rs10187402 | C | G | 0.085 | 0.013 | 1.76E-10 | 41.361 |
| genus | Bifidobacterium | rs7587285 | T | C | 0.113 | 0.018 | 1.11E-10 | 41.162 |
| genus | Bifidobacterium | rs6714750 | A | G | 0.085 | 0.013 | 3.86E-10 | 41.024 |
| genus | Bifidobacterium | rs1374329 | A | G | 0.086 | 0.014 | 3.44E-10 | 40.979 |
| genus | Bifidobacterium | rs10928545 | G | A | 0.087 | 0.014 | 4.26E-10 | 40.961 |
| genus | Bifidobacterium | rs2278683 | A | C | 0.113 | 0.018 | 1.48E-10 | 40.802 |
| genus | Bifidobacterium | rs935613 | C | T | 0.084 | 0.013 | 3.02E-10 | 40.683 |
| genus | Bifidobacterium | rs4954551 | G | A | 0.113 | 0.018 | 1.48E-10 | 40.603 |
| genus | Bifidobacterium | rs6760329 | G | A | 0.087 | 0.014 | 5.08E-10 | 40.252 |
| genus | Bifidobacterium | rs10188066 | A | G | 0.087 | 0.014 | 5.21E-10 | 40.208 |
| genus | Bifidobacterium | rs9287442 | G | A | 0.086 | 0.014 | 5.92E-10 | 40.148 |
| genus | Bifidobacterium | rs62160860 | G | C | 0.113 | 0.018 | 2.18E-10 | 40.134 |
| genus | Bifidobacterium | rs2304599 | G | C | 0.087 | 0.014 | 6.29E-10 | 40.023 |
| genus | Bifidobacterium | rs6723108 | G | T | 0.077 | 0.012 | 8.12E-11 | 39.843 |
| genus | Bifidobacterium | rs1530559 | A | G | 0.076 | 0.012 | 2.11E-10 | 39.725 |
| genus | Bifidobacterium | rs2016636 | G | A | 0.083 | 0.013 | 5.45E-10 | 39.663 |
| genus | Bifidobacterium | rs1030764 | T | C | 0.086 | 0.014 | 8.26E-10 | 39.417 |
| genus | Bifidobacterium | rs6738563 | G | T | 0.076 | 0.012 | 1.12E-10 | 39.414 |
| genus | Bifidobacterium | rs12616520 | T | A | 0.086 | 0.014 | 9.38E-10 | 39.272 |
| genus | Bifidobacterium | rs2278544 | G | A | 0.085 | 0.014 | 1.23E-09 | 38.767 |
| genus | Bifidobacterium | rs6704561 | C | T | 0.081 | 0.013 | 7.90E-10 | 38.666 |
| genus | Bifidobacterium | rs6747073 | T | C | 0.082 | 0.013 | 9.04E-10 | 38.465 |
| genus | Bifidobacterium | rs3820794 | T | C | 0.085 | 0.014 | 1.49E-09 | 38.240 |
| genus | Bifidobacterium | rs7581382 | T | C | -0.091 | 0.015 | 3.03E-11 | 37.851 |
| genus | Bifidobacterium | rs925966 | G | C | 0.079 | 0.013 | 1.45E-09 | 37.661 |
| genus | Bifidobacterium | rs7371606 | A | G | 0.078 | 0.013 | 2.20E-09 | 36.860 |
| genus | Streptococcus | rs11110281 | C | T | -0.138 | 0.023 | 2.58E-09 | 36.572 |
| genus | Streptococcus | rs11110282 | G | A | -0.135 | 0.022 | 2.97E-09 | 36.462 |
| genus | Streptococcus | rs12368593 | A | C | -0.136 | 0.023 | 2.91E-09 | 36.403 |
| genus | Streptococcus | rs80243853 | A | G | -0.136 | 0.023 | 3.37E-09 | 36.148 |
| genus | Bifidobacterium | rs1900741 | C | T | 0.086 | 0.014 | 6.78E-09 | 35.957 |
| genus | Streptococcus | rs11110283 | C | A | -0.136 | 0.023 | 3.74E-09 | 35.723 |
| genus | Bifidobacterium | rs6739713 | A | G | 0.080 | 0.013 | 5.29E-09 | 35.675 |
| genus | Bifidobacterium | rs998451 | A | G | 0.073 | 0.012 | 6.17E-10 | 35.558 |
| genus | Streptococcus | rs11110268 | T | C | -0.135 | 0.023 | 4.77E-09 | 35.444 |
| genus | Tyzzerella3 | rs67476743 | G | T | 0.132 | 0.022 | 3.74E-09 | 35.417 |
| genus | Streptococcus | rs117905191 | G | A | -0.135 | 0.023 | 4.85E-09 | 35.409 |
| genus | Streptococcus | rs78017832 | G | T | -0.135 | 0.023 | 4.94E-09 | 35.381 |
| genus | Enterorhabdus | rs11098863 | A | T | -0.097 | 0.016 | 3.06E-09 | 35.262 |
| genus | Bifidobacterium | rs3754692 | T | C | 0.080 | 0.013 | 6.80E-09 | 35.107 |
| genus | Streptococcus | rs17030005 | C | T | -0.133 | 0.023 | 6.37E-09 | 34.976 |
| genus | Streptococcus | rs9669453 | G | A | -0.133 | 0.023 | 6.45E-09 | 34.956 |
| genus | Bifidobacterium | rs4953945 | C | G | 0.099 | 0.017 | 1.61E-09 | 34.901 |
| genus | Bifidobacterium | rs309126 | T | C | 0.101 | 0.017 | 7.22E-09 | 34.802 |
| genus | Enterorhabdus | rs12647099 | A | G | -0.096 | 0.016 | 3.90E-09 | 34.790 |
| genus | Streptococcus | rs78843973 | C | T | -0.134 | 0.023 | 6.72E-09 | 34.721 |
| genus | Streptococcus | rs74348118 | T | C | -0.132 | 0.022 | 7.92E-09 | 34.627 |
| genus | Erysipelatoclostridium | rs7221249 | G | A | 0.084 | 0.014 | 4.31E-09 | 34.619 |
| genus | Bifidobacterium | rs12328663 | T | C | 0.098 | 0.017 | 9.93E-10 | 34.549 |
| genus | Bifidobacterium | rs2874834 | A | G | 0.079 | 0.013 | 9.46E-09 | 34.514 |
| genus | Bifidobacterium | rs4954513 | G | A | 0.107 | 0.018 | 8.77E-09 | 34.491 |
| genus | Bifidobacterium | rs2874739 | C | T | 0.097 | 0.017 | 1.32E-09 | 34.375 |
| genus | Streptococcus | rs11110276 | G | T | -0.131 | 0.022 | 8.78E-09 | 34.307 |
| genus | Bifidobacterium | rs16832275 | C | G | 0.103 | 0.018 | 6.33E-09 | 34.261 |
| genus | Bifidobacterium | rs10193348 | A | G | 0.097 | 0.017 | 1.36E-09 | 34.206 |
| genus | Streptococcus | rs17851246 | G | A | -0.132 | 0.023 | 9.29E-09 | 34.201 |
| genus | Bifidobacterium | rs3768998 | G | T | 0.106 | 0.018 | 4.64E-09 | 34.176 |
| genus | Streptococcus | rs7294396 | C | G | -0.127 | 0.022 | 9.82E-09 | 34.174 |
| genus | Bifidobacterium | rs16832156 | G | A | 0.103 | 0.018 | 7.28E-09 | 34.153 |
| genus | Streptococcus | rs11110264 | A | G | -0.133 | 0.023 | 9.01E-09 | 34.135 |
| genus | Streptococcus | rs17030023 | A | T | -0.132 | 0.023 | 9.85E-09 | 34.131 |
| genus | Tyzzerella3 | rs55725239 | A | T | 0.129 | 0.022 | 6.75E-09 | 34.128 |
| genus | Streptococcus | rs116878826 | T | C | -0.131 | 0.022 | 8.84E-09 | 34.115 |
| genus | Streptococcus | rs17029986 | T | G | -0.127 | 0.022 | 1.01E-08 | 34.093 |
| genus | Streptococcus | rs7959495 | A | C | -0.131 | 0.022 | 1.10E-08 | 34.035 |
| genus | Bifidobacterium | rs4346445 | A | C | 0.097 | 0.017 | 1.32E-09 | 33.986 |
| genus | Bifidobacterium | rs13389481 | C | A | 0.099 | 0.017 | 1.88E-09 | 33.859 |
| genus | Ruminococcus1 | rs10769159 | C | G | -0.064 | 0.011 | 5.29E-09 | 33.844 |
| genus | Bifidobacterium | rs7608980 | G | A | 0.106 | 0.018 | 1.42E-08 | 33.781 |
| genus | Streptococcus | rs12372388 | G | A | -0.130 | 0.022 | 1.16E-08 | 33.763 |
| genus | Bifidobacterium | rs3213871 | C | T | 0.107 | 0.018 | 1.02E-08 | 33.762 |
| genus | Streptococcus | rs11835118 | C | T | -0.130 | 0.022 | 1.20E-08 | 33.743 |
| genus | Bifidobacterium | rs16832138 | G | A | 0.106 | 0.018 | 1.47E-08 | 33.725 |
| genus | Enterorhabdus | rs10857113 | T | C | -0.094 | 0.016 | 6.62E-09 | 33.721 |
| genus | Bifidobacterium | rs1551497 | T | C | 0.097 | 0.017 | 2.25E-09 | 33.717 |
| genus | Streptococcus | rs7313123 | T | C | -0.117 | 0.020 | 9.80E-09 | 33.712 |
| genus | Streptococcus | rs12369047 | T | G | -0.118 | 0.020 | 1.05E-08 | 33.711 |
| genus | Bifidobacterium | rs10167901 | C | T | 0.097 | 0.017 | 2.14E-09 | 33.704 |
| genus | Bifidobacterium | rs4954216 | G | A | 0.097 | 0.017 | 2.15E-09 | 33.704 |
| genus | Streptococcus | rs79728246 | T | C | -0.130 | 0.022 | 1.25E-08 | 33.695 |
| genus | Enterorhabdus | rs1355109 | T | C | -0.095 | 0.016 | 7.09E-09 | 33.683 |
| genus | Streptococcus | rs75132655 | T | G | -0.117 | 0.020 | 1.02E-08 | 33.658 |
| genus | Bifidobacterium | rs62168942 | T | C | 0.097 | 0.017 | 2.37E-09 | 33.617 |
| genus | Bifidobacterium | rs3769002 | T | C | 0.106 | 0.018 | 1.24E-08 | 33.609 |
| genus | Ruminococcus1 | rs895733 | T | G | -0.064 | 0.011 | 7.05E-09 | 33.548 |
| genus | Bifidobacterium | rs4954215 | G | A | 0.097 | 0.017 | 2.32E-09 | 33.535 |
| genus | Streptococcus | rs4374023 | G | A | -0.130 | 0.022 | 1.37E-08 | 33.532 |
| genus | Streptococcus | rs11837200 | T | C | -0.129 | 0.022 | 1.34E-08 | 33.496 |
| genus | Bifidobacterium | rs4953948 | T | G | 0.096 | 0.017 | 2.38E-09 | 33.483 |
| genus | Bifidobacterium | rs10167809 | C | T | 0.096 | 0.017 | 2.41E-09 | 33.467 |
| genus | Bifidobacterium | rs10167884 | C | T | 0.096 | 0.017 | 2.41E-09 | 33.466 |
| genus | Erysipelatoclostridium | rs7211274 | C | T | -0.083 | 0.014 | 8.02E-09 | 33.461 |
| genus | Bifidobacterium | rs4954567 | G | T | 0.073 | 0.013 | 7.93E-09 | 33.454 |
| genus | Faecalibacterium | rs12320842 | G | C | 0.095 | 0.016 | 7.57E-09 | 33.453 |
| genus | Bifidobacterium | rs7577258 | G | A | 0.096 | 0.017 | 2.63E-09 | 33.442 |
| genus | Bifidobacterium | rs4953947 | A | C | 0.096 | 0.017 | 2.45E-09 | 33.431 |
| genus | Streptococcus | rs12370758 | T | C | -0.132 | 0.023 | 1.32E-08 | 33.408 |
| genus | Streptococcus | rs117585280 | T | A | -0.132 | 0.023 | 1.31E-08 | 33.396 |
| genus | Bifidobacterium | rs56263017 | T | C | 0.106 | 0.018 | 1.34E-08 | 33.384 |
| genus | Bifidobacterium | rs4954217 | C | T | 0.096 | 0.017 | 2.62E-09 | 33.381 |
| genus | Streptococcus | rs11522881 | C | T | -0.130 | 0.023 | 1.46E-08 | 33.374 |
| genus | Streptococcus | rs11110145 | A | T | -0.117 | 0.020 | 1.30E-08 | 33.333 |
| genus | Bifidobacterium | rs10182750 | T | C | 0.096 | 0.017 | 2.67E-09 | 33.331 |
| genus | Tyzzerella3 | rs4315451 | C | T | 0.128 | 0.022 | 1.03E-08 | 33.329 |
| genus | Bifidobacterium | rs62168941 | C | T | 0.096 | 0.017 | 2.78E-09 | 33.326 |
| genus | Bifidobacterium | rs13409400 | A | C | 0.096 | 0.017 | 2.88E-09 | 33.242 |
| genus | Bifidobacterium | rs57829242 | A | G | 0.097 | 0.017 | 2.78E-09 | 33.217 |
| genus | Enterorhabdus | rs11098866 | G | A | -0.094 | 0.016 | 8.63E-09 | 33.210 |
| genus | Bifidobacterium | rs921859 | A | G | 0.096 | 0.017 | 2.90E-09 | 33.185 |
| genus | Bifidobacterium | rs11884903 | T | C | 0.097 | 0.017 | 2.77E-09 | 33.178 |
| genus | Bifidobacterium | rs4954212 | A | G | 0.096 | 0.017 | 3.18E-09 | 33.169 |
| genus | Bifidobacterium | rs7565268 | T | C | 0.096 | 0.017 | 3.07E-09 | 33.159 |
| genus | Bifidobacterium | rs13399376 | A | G | 0.096 | 0.017 | 3.02E-09 | 33.139 |
| genus | Bifidobacterium | rs13402101 | T | A | 0.096 | 0.017 | 3.02E-09 | 33.139 |
| genus | Bifidobacterium | rs7601696 | C | A | 0.096 | 0.017 | 3.07E-09 | 33.137 |
| genus | Bifidobacterium | rs6721838 | A | G | 0.096 | 0.017 | 2.35E-09 | 33.123 |
| genus | Streptococcus | rs2559847 | C | T | -0.130 | 0.023 | 1.47E-08 | 33.109 |
| genus | Bifidobacterium | rs11890844 | A | G | 0.096 | 0.017 | 3.08E-09 | 33.100 |
| genus | Bifidobacterium | rs4954214 | C | T | 0.096 | 0.017 | 2.97E-09 | 33.058 |
| genus | Streptococcus | rs11110235 | T | C | -0.129 | 0.022 | 1.76E-08 | 33.045 |
| genus | Bifidobacterium | rs10208624 | C | T | 0.096 | 0.017 | 2.03E-09 | 33.030 |
| genus | Bifidobacterium | rs13402112 | A | C | 0.096 | 0.017 | 3.09E-09 | 33.023 |
| genus | Bifidobacterium | rs7422031 | C | T | 0.095 | 0.017 | 2.75E-09 | 33.010 |
| genus | Bifidobacterium | rs2164332 | G | C | 0.101 | 0.018 | 1.11E-08 | 32.988 |
| genus | Bifidobacterium | rs10186594 | A | G | 0.096 | 0.017 | 3.30E-09 | 32.961 |
| genus | Bifidobacterium | rs56903548 | C | T | 0.095 | 0.017 | 2.85E-09 | 32.950 |
| genus | Bifidobacterium | rs58697996 | G | A | 0.096 | 0.017 | 2.58E-09 | 32.924 |
| genus | Bifidobacterium | rs7568525 | G | A | 0.096 | 0.017 | 2.15E-09 | 32.916 |
| genus | Bifidobacterium | rs56067372 | C | T | 0.105 | 0.018 | 2.23E-08 | 32.908 |
| genus | Streptococcus | rs11110148 | G | C | -0.116 | 0.020 | 1.61E-08 | 32.884 |
| genus | Bifidobacterium | rs7601683 | C | T | 0.096 | 0.017 | 3.63E-09 | 32.847 |
| genus | Bifidobacterium | rs1401687 | G | C | 0.072 | 0.013 | 1.08E-08 | 32.832 |
| genus | Bifidobacterium | rs2017831 | C | T | 0.096 | 0.017 | 3.57E-09 | 32.830 |
| genus | Bifidobacterium | rs6430593 | G | A | 0.101 | 0.018 | 1.74E-08 | 32.830 |
| genus | Streptococcus | rs7296331 | C | T | -0.128 | 0.022 | 1.98E-08 | 32.829 |
| genus | Bifidobacterium | rs10928513 | C | T | 0.070 | 0.012 | 5.22E-09 | 32.815 |
| genus | Bifidobacterium | rs6760553 | A | G | 0.096 | 0.017 | 3.31E-09 | 32.809 |
| genus | Streptococcus | rs78367981 | G | C | -0.128 | 0.022 | 1.88E-08 | 32.796 |
| genus | Tyzzerella3 | rs55784632 | C | T | 0.127 | 0.022 | 1.34E-08 | 32.782 |
| genus | Peptococcus | rs57905414 | A | T | 0.185 | 0.032 | 1.30E-08 | 32.770 |
| genus | Bifidobacterium | rs10179271 | T | C | 0.072 | 0.013 | 1.12E-08 | 32.767 |
| genus | Streptococcus | rs7967270 | A | C | -0.116 | 0.020 | 1.74E-08 | 32.745 |
| genus | Bifidobacterium | rs13413872 | G | T | 0.095 | 0.017 | 3.64E-09 | 32.711 |
| genus | Bifidobacterium | rs10209482 | A | C | 0.096 | 0.017 | 3.75E-09 | 32.668 |
| genus |  | rs17159861 | T | C | 0.096 | 0.017 | 1.04E-08 | 32.654 |
| genus | Bifidobacterium | rs16831498 | G | C | 0.096 | 0.017 | 3.74E-09 | 32.647 |
| genus | Streptococcus | rs114272490 | T | C | -0.116 | 0.020 | 1.89E-08 | 32.645 |
| genus | Bifidobacterium | rs13427494 | G | A | 0.096 | 0.017 | 3.69E-09 | 32.645 |
| genus | Bifidobacterium | rs60350840 | A | G | 0.095 | 0.017 | 4.18E-09 | 32.610 |
| genus | Bifidobacterium | rs60433564 | A | C | 0.124 | 0.022 | 9.61E-09 | 32.608 |
| genus | Streptococcus | rs12367656 | T | C | -0.129 | 0.023 | 2.15E-08 | 32.602 |
| genus | Bifidobacterium | rs7565149 | T | A | 0.095 | 0.017 | 4.09E-09 | 32.587 |
| genus | Bifidobacterium | rs11888951 | T | C | 0.095 | 0.017 | 3.73E-09 | 32.579 |
| genus | Bifidobacterium | rs7573600 | G | C | 0.095 | 0.017 | 2.97E-09 | 32.577 |
| genus | Bifidobacterium | rs6752519 | C | T | 0.104 | 0.018 | 1.78E-08 | 32.565 |
| genus | Streptococcus | rs74767263 | C | G | -0.116 | 0.020 | 1.92E-08 | 32.560 |
| genus | Streptococcus | rs11110288 | C | T | -0.128 | 0.022 | 2.15E-08 | 32.559 |
| genus | Erysipelatoclostridium | rs9914071 | T | C | -0.082 | 0.014 | 1.28E-08 | 32.546 |
| genus | Bifidobacterium | rs56174523 | A | C | 0.095 | 0.017 | 4.02E-09 | 32.542 |
| genus | Bifidobacterium | rs4953946 | T | G | 0.095 | 0.017 | 3.78E-09 | 32.535 |
| genus | Bifidobacterium | rs10174944 | C | T | 0.095 | 0.017 | 4.08E-09 | 32.534 |
| genus | Streptococcus | rs12368073 | C | A | -0.123 | 0.022 | 2.18E-08 | 32.525 |
| genus | Bifidobacterium | rs11892750 | G | A | 0.095 | 0.017 | 5.09E-09 | 32.455 |
| genus | Bifidobacterium | rs4954568 | T | A | 0.072 | 0.013 | 1.31E-08 | 32.442 |
| genus | Bifidobacterium | rs61087958 | C | T | 0.095 | 0.017 | 3.38E-09 | 32.439 |
| genus | Bifidobacterium | rs11903663 | A | G | 0.095 | 0.017 | 2.16E-09 | 32.430 |
| genus | Bifidobacterium | rs7603805 | A | G | 0.095 | 0.017 | 3.75E-09 | 32.426 |
| genus | Streptococcus | rs7138882 | A | G | -0.128 | 0.022 | 2.29E-08 | 32.410 |
| genus | Enterorhabdus | rs2391050 | T | A | -0.093 | 0.016 | 1.32E-08 | 32.408 |
| genus | Allisonella | rs602075 | G | A | 0.169 | 0.030 | 3.57E-08 | 32.374 |
| genus | Bifidobacterium | rs9941547 | G | A | 0.072 | 0.013 | 1.37E-08 | 32.355 |
| genus | Bifidobacterium | rs10496736 | C | G | 0.124 | 0.022 | 1.03E-08 | 32.328 |
| genus | Streptococcus | rs116172730 | G | A | -0.127 | 0.022 | 2.51E-08 | 32.302 |
| genus | Bifidobacterium | rs2322725 | T | C | 0.100 | 0.018 | 1.52E-08 | 32.289 |
| genus | Peptococcus | rs75754569 | G | C | 0.181 | 0.032 | 1.10E-08 | 32.262 |
| genus | Streptococcus | rs12371930 | G | A | -0.135 | 0.024 | 1.99E-08 | 32.257 |
| genus | Bifidobacterium | rs16831264 | G | C | 0.094 | 0.017 | 4.61E-09 | 32.255 |
| genus | Bifidobacterium | rs11898045 | T | C | 0.094 | 0.017 | 3.25E-09 | 32.248 |
| genus | Bifidobacterium | rs4954220 | C | A | 0.095 | 0.017 | 3.38E-09 | 32.228 |
| genus | Bifidobacterium | rs62168934 | A | G | 0.095 | 0.017 | 4.95E-09 | 32.225 |
| genus | Bifidobacterium | rs6741481 | A | G | 0.094 | 0.017 | 3.79E-09 | 32.195 |
| genus | Bifidobacterium | rs4645053 | T | A | 0.094 | 0.017 | 2.63E-09 | 32.159 |
| genus | Enterorhabdus | rs4833344 | C | T | 0.092 | 0.016 | 1.48E-08 | 32.155 |
| genus | Bifidobacterium | rs4954226 | T | C | 0.095 | 0.017 | 4.61E-09 | 32.121 |
| genus | Streptococcus | rs11110138 | T | C | -0.115 | 0.020 | 2.28E-08 | 32.121 |
| genus | Streptococcus | rs115024257 | G | A | -0.115 | 0.020 | 2.16E-08 | 32.098 |
| genus | Bifidobacterium | rs4640444 | T | C | 0.094 | 0.017 | 2.72E-09 | 32.078 |
| genus | Allisonella | rs785928 | T | G | 0.168 | 0.030 | 4.10E-08 | 32.068 |
| genus | Bifidobacterium | rs58426504 | T | G | 0.094 | 0.017 | 3.62E-09 | 32.046 |
| genus | Bifidobacterium | rs7565053 | A | T | 0.128 | 0.023 | 1.48E-08 | 31.998 |
| genus | Bifidobacterium | rs6430561 | C | T | 0.094 | 0.017 | 4.07E-09 | 31.964 |
| genus | Streptococcus | rs11110198 | G | A | -0.115 | 0.020 | 2.36E-08 | 31.944 |
| genus | Bifidobacterium | rs58327215 | G | C | 0.094 | 0.017 | 3.91E-09 | 31.937 |
| genus | Bifidobacterium | rs309132 | C | G | 0.102 | 0.018 | 3.68E-08 | 31.928 |
| genus | Streptococcus | rs11110196 | C | A | -0.115 | 0.020 | 2.45E-08 | 31.927 |
| genus | Streptococcus | rs12370204 | C | T | -0.115 | 0.020 | 2.54E-08 | 31.925 |
| genus | Streptococcus | rs11110241 | T | C | -0.122 | 0.022 | 2.72E-08 | 31.908 |
| genus | Streptococcus | rs11110129 | A | G | -0.115 | 0.020 | 2.71E-08 | 31.865 |
| genus | Bifidobacterium | rs3754690 | C | T | 0.107 | 0.019 | 2.92E-08 | 31.862 |
| genus | Streptococcus | rs11110158 | G | A | -0.132 | 0.023 | 2.71E-08 | 31.809 |
| genus | Streptococcus | rs114181124 | G | A | -0.114 | 0.020 | 2.96E-08 | 31.732 |
| genus | Bifidobacterium | rs2290518 | G | A | 0.093 | 0.017 | 4.93E-09 | 31.621 |
| genus | Allisonella | rs2480213 | G | A | 0.167 | 0.030 | 4.77E-08 | 31.591 |
| genus | Bifidobacterium | rs6709763 | C | A | 0.094 | 0.017 | 7.39E-09 | 31.509 |
| genus | Streptococcus | rs12369165 | C | T | -0.113 | 0.020 | 3.18E-08 | 31.474 |
| genus | Enterorhabdus | rs11098865 | T | C | -0.091 | 0.016 | 2.14E-08 | 31.436 |
| genus | Bifidobacterium | rs12233092 | G | A | 0.100 | 0.018 | 2.40E-08 | 31.431 |
| genus | Streptococcus | rs11110250 | T | C | -0.125 | 0.022 | 3.98E-08 | 31.427 |
| genus | Streptococcus | rs57062458 | C | T | -0.121 | 0.022 | 3.19E-08 | 31.417 |
| genus | Streptococcus | rs11110204 | A | G | -0.127 | 0.023 | 3.93E-08 | 31.417 |
| genus | Allisonella | rs785916 | G | A | 0.167 | 0.030 | 5.38E-08 | 31.415 |
| genus | Streptococcus | rs11110118 | C | T | -0.114 | 0.020 | 3.28E-08 | 31.395 |
| genus | Bifidobacterium | rs2164331 | C | T | 0.103 | 0.018 | 3.12E-08 | 31.395 |
| genus | Streptococcus | rs11110140 | T | C | -0.114 | 0.020 | 3.44E-08 | 31.392 |
| genus | Streptococcus | rs11110251 | A | C | -0.125 | 0.022 | 4.06E-08 | 31.386 |
| genus | Bifidobacterium | rs6737635 | G | T | 0.093 | 0.017 | 7.96E-09 | 31.320 |
| genus | Bifidobacterium | rs16832200 | T | C | 0.099 | 0.018 | 2.68E-08 | 31.304 |
| genus | Streptococcus | rs11110211 | T | G | -0.125 | 0.022 | 4.23E-08 | 31.291 |
| genus |  | rs35866622 | C | T | -0.061 | 0.011 | 2.21E-08 | 31.285 |
| genus | CandidatusSoleaferrea | . | A | G | 0.195 | 0.035 | 4.26E-08 | 31.269 |
| genus | Enterorhabdus | rs2136230 | T | A | -0.091 | 0.016 | 2.39E-08 | 31.267 |
| genus | Allisonella | rs513301 | C | T | 0.166 | 0.030 | 6.73E-08 | 31.257 |
| genus | Allisonella | rs502417 | C | T | 0.166 | 0.030 | 5.71E-08 | 31.248 |
| genus | Streptococcus | rs12371009 | A | G | -0.113 | 0.020 | 3.79E-08 | 31.248 |
| genus | Streptococcus | rs10860540 | A | C | -0.112 | 0.020 | 3.89E-08 | 31.210 |
| genus | RuminococcaceaeUCG013 | rs12781711 | T | C | -0.066 | 0.012 | 2.55E-08 | 31.194 |
| genus | Streptococcus | rs10860537 | C | T | -0.113 | 0.020 | 3.57E-08 | 31.182 |
| genus | Bifidobacterium | rs7597802 | A | T | 0.093 | 0.017 | 8.96E-09 | 31.179 |
| genus | Streptococcus | rs112118907 | C | A | -0.125 | 0.022 | 4.70E-08 | 31.158 |
| genus | Oxalobacter | rs736744 | T | C | 0.118 | 0.021 | 2.57E-08 | 31.135 |
| genus | Streptococcus | rs11110160 | A | G | -0.113 | 0.020 | 4.14E-08 | 31.130 |
| genus | Bifidobacterium | rs13408096 | G | T | 0.093 | 0.017 | 8.50E-09 | 31.121 |
| genus | Bifidobacterium | rs62170081 | A | G | 0.121 | 0.022 | 2.14E-08 | 31.116 |
| genus | Bifidobacterium | rs6742013 | T | C | 0.094 | 0.017 | 8.37E-09 | 31.072 |
| genus | Bifidobacterium | rs4494812 | A | G | 0.093 | 0.017 | 5.01E-09 | 31.056 |
| genus | Bifidobacterium | rs6715785 | G | A | 0.070 | 0.013 | 2.69E-08 | 31.045 |
| genus | Bifidobacterium | rs7322849 | C | T | 0.112 | 0.020 | 1.08E-08 | 31.035 |
| genus | Bifidobacterium | rs4954213 | G | T | 0.093 | 0.017 | 8.62E-09 | 31.023 |
| genus | Bifidobacterium | rs7563997 | T | A | 0.093 | 0.017 | 9.05E-09 | 31.006 |
| genus | Bifidobacterium | rs10928554 | C | T | 0.102 | 0.018 | 1.03E-07 | 30.973 |
| genus | Streptococcus | rs11832216 | C | T | -0.124 | 0.022 | 5.27E-08 | 30.965 |
| genus | Ruminococcus1 | rs4148899 | T | C | -0.061 | 0.011 | 2.53E-08 | 30.946 |
| genus | Bifidobacterium | rs74266324 | G | A | 0.085 | 0.015 | 2.13E-08 | 30.904 |
| genus | Bifidobacterium | rs4954430 | C | T | 0.121 | 0.022 | 1.70E-08 | 30.875 |
| genus | Bifidobacterium | rs13410870 | G | A | 0.092 | 0.017 | 7.17E-09 | 30.853 |
| genus | Streptococcus | rs12371224 | A | T | -0.112 | 0.020 | 4.03E-08 | 30.850 |
| genus | Bifidobacterium | rs4954633 | C | T | 0.120 | 0.022 | 1.60E-08 | 30.848 |
| genus | RuminococcaceaeUCG009 | rs8009993 | C | G | -0.136 | 0.024 | 4.42E-08 | 30.821 |
| genus | Bifidobacterium | rs11900832 | C | T | 0.093 | 0.017 | 1.09E-08 | 30.809 |
| genus | Intestinibacter | rs10805326 | A | G | 0.078 | 0.014 | 3.55E-08 | 30.803 |
| genus | Streptococcus | rs11110257 | C | T | -0.124 | 0.022 | 5.62E-08 | 30.797 |
| genus | Bifidobacterium | rs4954242 | A | G | 0.094 | 0.017 | 1.31E-08 | 30.790 |
| genus | Streptococcus | rs10860539 | C | T | -0.111 | 0.020 | 4.83E-08 | 30.788 |
| genus | Bifidobacterium | rs10928529 | G | A | 0.093 | 0.017 | 1.09E-08 | 30.786 |
| genus | Bifidobacterium | rs4954392 | A | G | 0.070 | 0.013 | 2.94E-08 | 30.785 |
| genus | Bifidobacterium | rs55634455 | T | C | 0.105 | 0.019 | 4.66E-08 | 30.775 |
| genus | Bifidobacterium | rs11888521 | G | C | 0.092 | 0.017 | 5.66E-09 | 30.751 |
| genus | Allisonella | rs785915 | A | G | 0.165 | 0.030 | 8.27E-08 | 30.738 |
| genus | Bifidobacterium | rs4954569 | C | T | 0.070 | 0.013 | 3.15E-08 | 30.718 |
| genus | Bifidobacterium | rs7605229 | C | T | 0.093 | 0.017 | 1.07E-08 | 30.699 |
| genus | Streptococcus | rs11495178 | C | A | -0.112 | 0.020 | 5.01E-08 | 30.695 |
| genus | Ruminococcus1 | rs11603055 | C | T | -0.061 | 0.011 | 2.92E-08 | 30.691 |
| genus | Bifidobacterium | rs12478902 | T | C | 0.099 | 0.018 | 3.69E-08 | 30.664 |
| genus | Bifidobacterium | rs4954221 | G | A | 0.092 | 0.017 | 7.81E-09 | 30.647 |
| genus | Streptococcus | rs74484094 | C | T | -0.123 | 0.022 | 6.75E-08 | 30.644 |
| genus | Streptococcus | rs75007764 | G | A | -0.123 | 0.022 | 5.91E-08 | 30.614 |
| genus | Allisonella | rs512269 | C | T | 0.164 | 0.030 | 8.90E-08 | 30.613 |
| genus | Streptococcus | rs11110171 | G | A | -0.112 | 0.020 | 4.56E-08 | 30.609 |
| genus |  | rs281379 | G | A | -0.060 | 0.011 | 3.35E-08 | 30.598 |
| genus | Bifidobacterium | rs4954564 | A | G | 0.070 | 0.013 | 3.58E-08 | 30.593 |
| genus | Streptococcus | rs11110110 | G | A | -0.112 | 0.020 | 5.02E-08 | 30.574 |
| genus | Bifidobacterium | rs950176 | A | G | 0.070 | 0.013 | 3.70E-08 | 30.548 |
| genus | Streptococcus | rs56843411 | G | A | -0.122 | 0.022 | 5.76E-08 | 30.536 |
| genus | Bifidobacterium | rs3806502 | C | T | 0.093 | 0.017 | 1.09E-08 | 30.512 |
| genus | Streptococcus | rs11110184 | A | G | -0.112 | 0.020 | 5.63E-08 | 30.510 |
| genus | Ruminococcus1 | rs12420703 | T | C | -0.061 | 0.011 | 3.04E-08 | 30.467 |
| genus | Bifidobacterium | rs12616955 | C | T | 0.070 | 0.013 | 3.48E-08 | 30.448 |
| genus | Streptococcus | rs138155218 | C | G | -0.112 | 0.020 | 5.55E-08 | 30.441 |
| genus | Streptococcus | rs7298009 | T | C | -0.112 | 0.020 | 5.73E-08 | 30.435 |
| genus | Bifidobacterium | rs16855656 | A | G | 0.095 | 0.017 | 5.47E-08 | 30.420 |
| genus | Streptococcus | rs11110190 | T | C | -0.111 | 0.020 | 5.94E-08 | 30.404 |
| genus | Bifidobacterium | rs11889843 | G | A | 0.092 | 0.017 | 1.28E-08 | 30.400 |
| genus | Bifidobacterium | rs11897204 | T | C | 0.092 | 0.017 | 8.84E-09 | 30.396 |
| genus | Streptococcus | rs7312868 | G | A | -0.112 | 0.020 | 5.91E-08 | 30.395 |
| genus | Bifidobacterium | rs2305248 | A | G | 0.092 | 0.017 | 9.52E-09 | 30.392 |
| genus | Bifidobacterium | rs4954566 | C | T | 0.070 | 0.013 | 3.69E-08 | 30.372 |
| genus | Streptococcus | rs11110191 | C | T | -0.111 | 0.020 | 6.05E-08 | 30.362 |
| genus | Bifidobacterium | rs78141345 | G | A | 0.104 | 0.019 | 5.92E-08 | 30.348 |
| genus | Bifidobacterium | rs17293519 | A | G | 0.091 | 0.017 | 1.05E-08 | 30.344 |
| genus | Bifidobacterium | rs6729961 | T | G | 0.120 | 0.022 | 3.87E-08 | 30.341 |
| genus | Bifidobacterium | rs6749119 | C | G | 0.091 | 0.017 | 9.10E-09 | 30.339 |
| genus | Streptococcus | rs11110130 | A | T | -0.112 | 0.020 | 5.83E-08 | 30.330 |
| genus | Ruminococcus1 | rs4148901 | T | G | -0.061 | 0.011 | 3.48E-08 | 30.304 |
| genus | Streptococcus | rs12370791 | T | G | -0.123 | 0.022 | 7.08E-08 | 30.267 |
| genus | Bifidobacterium | rs6720497 | G | A | 0.120 | 0.022 | 3.55E-08 | 30.248 |
| genus | Bifidobacterium | rs6707289 | A | G | 0.120 | 0.022 | 3.55E-08 | 30.247 |
| genus | Bifidobacterium | rs61663334 | A | G | 0.091 | 0.017 | 6.77E-09 | 30.166 |
| genus | Streptococcus | rs150200257 | G | A | -0.128 | 0.023 | 6.21E-08 | 30.146 |
| genus | Bifidobacterium | rs56369660 | A | G | 0.091 | 0.017 | 1.33E-08 | 30.140 |
| genus | Bifidobacterium | rs35215526 | G | A | 0.108 | 0.020 | 8.84E-08 | 30.076 |
| genus | Bifidobacterium | rs1942055 | G | C | 0.067 | 0.012 | 2.48E-08 | 30.059 |
| genus | Bifidobacterium | rs72972104 | G | A | 0.118 | 0.022 | 2.26E-08 | 30.052 |
| genus | Bifidobacterium | rs72970286 | T | G | 0.117 | 0.021 | 3.01E-08 | 29.995 |
| genus | Ruminococcus1 | rs45604433 | C | G | -0.063 | 0.011 | 5.09E-08 | 29.992 |
| genus |  | rs34297067 | G | A | -0.187 | 0.034 | 6.60E-08 | 29.959 |
| genus | Bifidobacterium | rs56982689 | C | T | 0.119 | 0.022 | 2.13E-08 | 29.935 |
| genus | Ruminococcus1 | rs4148895 | G | C | -0.060 | 0.011 | 4.70E-08 | 29.912 |
| genus | Allisonella | rs785907 | T | G | 0.161 | 0.030 | 1.59E-07 | 29.892 |
| genus |  | rs2287922 | G | A | -0.059 | 0.011 | 4.90E-08 | 29.877 |
| genus | Streptococcus | rs60861038 | C | T | -0.110 | 0.020 | 7.95E-08 | 29.815 |
| genus | Bifidobacterium | rs6706466 | A | G | 0.091 | 0.017 | 1.17E-08 | 29.811 |
| genus | Peptococcus | rs77922631 | A | T | 0.179 | 0.033 | 7.05E-08 | 29.805 |
| genus | Ruminococcus1 | rs36106594 | C | T | -0.062 | 0.011 | 5.55E-08 | 29.780 |
| genus | Bifidobacterium | rs4954572 | G | T | 0.069 | 0.013 | 4.55E-08 | 29.778 |
| genus | Bifidobacterium | rs4954396 | A | C | 0.069 | 0.013 | 4.57E-08 | 29.776 |
| genus | Bifidobacterium | rs10803550 | C | T | 0.069 | 0.013 | 4.80E-08 | 29.775 |
| genus | Bifidobacterium | rs4954388 | T | C | 0.071 | 0.013 | 6.05E-08 | 29.764 |
| genus |  | rs12147432 | C | A | -0.186 | 0.034 | 7.48E-08 | 29.746 |
| genus | Bifidobacterium | rs6745996 | A | G | 0.096 | 0.018 | 2.70E-08 | 29.743 |
| genus |  | rs13163520 | A | G | -0.127 | 0.023 | 5.61E-08 | 29.661 |
| genus | Streptococcus | rs11110125 | A | G | -0.109 | 0.020 | 7.71E-08 | 29.650 |
| genus | Bifidobacterium | rs584226 | T | C | 0.097 | 0.018 | 8.88E-08 | 29.578 |
| genus | Streptococcus | rs111326382 | A | G | -0.108 | 0.020 | 8.51E-08 | 29.566 |
| genus | Bifidobacterium | rs6727889 | C | T | 0.092 | 0.017 | 2.37E-08 | 29.530 |
| genus | Bifidobacterium | rs953387 | A | C | 0.069 | 0.013 | 5.45E-08 | 29.498 |
| genus | Bifidobacterium | rs6756200 | T | C | 0.065 | 0.012 | 4.63E-08 | 29.483 |
| genus | Bifidobacterium | rs2102896 | T | C | 0.069 | 0.013 | 5.49E-08 | 29.455 |
| genus | Streptococcus | rs7308069 | C | A | -0.109 | 0.020 | 8.94E-08 | 29.449 |
| genus | Bifidobacterium | rs57543738 | T | C | 0.103 | 0.019 | 6.87E-08 | 29.431 |
| genus | Bifidobacterium | rs2056297 | G | T | 0.069 | 0.013 | 6.04E-08 | 29.415 |
| genus | Streptococcus | rs11110200 | A | T | -0.123 | 0.023 | 1.11E-07 | 29.380 |
| genus | Bifidobacterium | rs16831994 | T | C | 0.117 | 0.022 | 4.59E-08 | 29.377 |
| genus | LachnospiraceaeUCG001 | rs437876 | C | T | 0.078 | 0.014 | 7.17E-08 | 29.376 |
| genus | Peptococcus | rs56249239 | T | C | 0.175 | 0.032 | 6.07E-08 | 29.371 |
| genus | Romboutsia | rs61841503 | A | G | 0.093 | 0.017 | 4.00E-08 | 29.351 |
| genus | Haemophilus | rs9382510 | T | C | -0.094 | 0.017 | 7.12E-08 | 29.342 |
| genus | Streptococcus | rs11110249 | T | A | -0.122 | 0.023 | 1.20E-07 | 29.263 |
| genus | Streptococcus | rs11110164 | C | G | -0.109 | 0.020 | 9.75E-08 | 29.252 |
| genus | Haemophilus | rs7740611 | C | A | -0.093 | 0.017 | 7.47E-08 | 29.239 |
| genus | Faecalibacterium | rs7966283 | A | G | 0.085 | 0.016 | 6.24E-08 | 29.222 |
| genus | Streptococcus | rs11110091 | G | A | -0.110 | 0.020 | 9.55E-08 | 29.182 |
| genus | Bifidobacterium | rs2322445 | T | A | 0.090 | 0.017 | 1.66E-08 | 29.165 |
| genus | Bifidobacterium | rs62168846 | G | A | 0.106 | 0.020 | 1.47E-07 | 29.143 |
| genus | RuminococcaceaeUCG004 | rs6769553 | G | A | 0.085 | 0.016 | 7.91E-08 | 29.126 |
| genus | Streptococcus | rs76452890 | G | C | -0.120 | 0.022 | 1.36E-07 | 29.042 |
| genus | Ruminococcus1 | rs10838504 | C | T | 0.060 | 0.011 | 7.86E-08 | 29.036 |
| genus | Bifidobacterium | rs13007153 | T | C | 0.070 | 0.013 | 9.07E-08 | 29.029 |
| genus | Turicibacter | rs149744580 | G | A | 0.170 | 0.032 | 7.01E-08 | 28.997 |
| genus | Bifidobacterium | rs56211644 | T | C | 0.102 | 0.019 | 1.07E-07 | 28.994 |
| genus | Streptococcus | rs12366744 | T | A | -0.119 | 0.022 | 1.26E-07 | 28.993 |
| genus | Peptococcus | rs139136610 | C | T | 0.172 | 0.032 | 5.63E-08 | 28.992 |
| genus | Streptococcus | rs76118421 | T | A | -0.110 | 0.020 | 1.06E-07 | 28.950 |
| genus | Oxalobacter | rs4428215 | A | G | 0.130 | 0.024 | 7.51E-08 | 28.931 |
| genus | Erysipelatoclostridium | rs4791400 | A | G | -0.077 | 0.014 | 8.56E-08 | 28.897 |
| genus | LachnospiraceaeUCG008 | rs10793103 | T | C | 0.097 | 0.018 | 9.35E-08 | 28.889 |
| genus | Bifidobacterium | rs10171677 | G | A | 0.065 | 0.012 | 3.28E-08 | 28.862 |
| genus | Streptococcus | rs2029585 | T | C | -0.119 | 0.022 | 1.37E-07 | 28.857 |
| genus | Bifidobacterium | rs62168847 | T | C | 0.105 | 0.020 | 1.34E-07 | 28.845 |
| genus | Ruminococcus1 | rs7121696 | C | T | -0.061 | 0.011 | 9.39E-08 | 28.843 |
| genus | Oxalobacter | rs10868238 | C | T | 0.113 | 0.021 | 8.22E-08 | 28.841 |
| genus | Streptococcus | rs113804138 | C | T | -0.119 | 0.022 | 1.48E-07 | 28.811 |
| genus | Peptococcus | rs10031059 | C | T | -0.121 | 0.023 | 1.24E-07 | 28.784 |
| genus |  | rs35326214 | T | A | -0.124 | 0.023 | 1.19E-07 | 28.779 |
| genus | RuminococcaceaeUCG011 | rs1416041 | C | A | -0.182 | 0.034 | 7.04E-08 | 28.778 |
| genus | Bifidobacterium | rs4954570 | G | C | 0.073 | 0.014 | 5.73E-08 | 28.774 |
| genus | Streptococcus | rs11524043 | G | A | -0.108 | 0.020 | 1.30E-07 | 28.761 |
| genus | Bifidobacterium | rs6705610 | T | G | 0.089 | 0.017 | 2.02E-08 | 28.745 |
| genus | Bifidobacterium | rs11897217 | C | T | 0.089 | 0.017 | 1.98E-08 | 28.707 |
| genus | Bifidobacterium | rs62168843 | T | C | 0.105 | 0.020 | 1.43E-07 | 28.639 |
| genus | Streptococcus | rs11110212 | T | C | -0.118 | 0.022 | 1.60E-07 | 28.591 |
| genus | Bifidobacterium | rs60376570 | G | A | 0.101 | 0.019 | 1.09E-07 | 28.589 |
| genus | Holdemanella | rs761624 | G | C | 0.096 | 0.018 | 1.38E-07 | 28.579 |
| genus | Streptococcus | rs7316887 | G | A | -0.108 | 0.020 | 1.35E-07 | 28.563 |
| genus | Bifidobacterium | rs12473760 | C | A | 0.068 | 0.013 | 8.67E-08 | 28.546 |
| genus | Streptococcus | rs11110214 | A | G | -0.118 | 0.022 | 1.60E-07 | 28.537 |
| genus | Lactococcus | rs34757988 | C | G | 0.122 | 0.023 | 8.95E-08 | 28.525 |
| genus | Streptococcus | rs11110208 | T | C | -0.118 | 0.022 | 1.65E-07 | 28.523 |
| genus | LachnospiraceaeUCG001 | rs985416 | T | C | 0.097 | 0.018 | 1.46E-07 | 28.481 |
| genus | Bifidobacterium | rs7594509 | G | T | 0.089 | 0.017 | 2.26E-08 | 28.475 |
| genus | RuminococcaceaeUCG003 | rs73341548 | G | T | -0.170 | 0.032 | 1.65E-07 | 28.473 |
| genus | RuminococcaceaeUCG003 | rs73341549 | C | T | -0.170 | 0.032 | 1.51E-07 | 28.393 |
| genus | Coprococcus1 | rs4277593 | A | G | -0.059 | 0.011 | 1.14E-07 | 28.390 |
| genus | Streptococcus | rs118038800 | C | T | -0.107 | 0.020 | 1.66E-07 | 28.385 |
| genus |  | rs660745 | T | C | -0.057 | 0.011 | 9.88E-08 | 28.366 |
| genus | Bifidobacterium | rs2874874 | A | C | 0.101 | 0.019 | 1.27E-07 | 28.356 |
| genus | Peptococcus | rs114500003 | T | C | 0.172 | 0.032 | 1.32E-07 | 28.343 |
| genus | Bifidobacterium | rs7609188 | G | C | 0.115 | 0.022 | 6.86E-08 | 28.321 |
| genus | Streptococcus | rs138049946 | T | C | -0.107 | 0.020 | 1.63E-07 | 28.300 |
| genus | Peptococcus | rs12650638 | G | T | -0.121 | 0.023 | 1.64E-07 | 28.295 |
| genus | Haemophilus | rs12191680 | G | C | 0.107 | 0.020 | 1.47E-07 | 28.288 |
| genus | Streptococcus | rs11110206 | T | C | -0.118 | 0.022 | 1.86E-07 | 28.287 |
| genus | Bifidobacterium | rs4954397 | A | G | 0.068 | 0.013 | 1.00E-07 | 28.274 |
| genus | Streptococcus | rs11110280 | G | A | -0.107 | 0.020 | 1.76E-07 | 28.255 |
| genus |  | rs2287921 | T | C | -0.057 | 0.011 | 1.13E-07 | 28.219 |
| genus | Haemophilus | rs7771624 | C | A | -0.092 | 0.017 | 1.27E-07 | 28.203 |
| genus | Bifidobacterium | rs4954577 | A | G | 0.068 | 0.013 | 1.08E-07 | 28.164 |
| genus | Romboutsia | rs61844362 | T | C | 0.084 | 0.016 | 1.08E-07 | 28.151 |
| genus | Faecalibacterium | rs7960298 | C | T | 0.085 | 0.016 | 1.03E-07 | 28.140 |
| genus | Streptococcus | rs7308947 | A | T | -0.106 | 0.020 | 1.71E-07 | 28.103 |
| genus | Oxalobacter | rs10465181 | T | C | 0.112 | 0.021 | 1.21E-07 | 28.094 |
| genus | Peptococcus | rs13105343 | T | C | -0.119 | 0.023 | 1.79E-07 | 28.079 |
| genus |  | rs11882796 | A | T | -0.057 | 0.011 | 1.22E-07 | 28.049 |
| genus | Allisonella | rs785914 | C | T | 0.158 | 0.030 | 3.38E-07 | 28.047 |
| genus | Ruminococcus1 | rs45486999 | C | T | -0.061 | 0.011 | 1.58E-07 | 28.039 |
| genus | Enterorhabdus | rs4834119 | G | A | 0.086 | 0.016 | 1.23E-07 | 28.017 |
| genus | Streptococcus | rs11614784 | G | A | -0.107 | 0.020 | 1.96E-07 | 28.014 |
| genus | Peptococcus | rs4280753 | C | T | -0.119 | 0.022 | 1.66E-07 | 27.999 |
| genus | Bifidobacterium | rs638382 | C | T | -0.065 | 0.012 | 9.45E-08 | 27.997 |
| genus | RuminococcaceaeUCG013 | rs12773579 | G | A | -0.063 | 0.012 | 1.32E-07 | 27.994 |
| genus | Streptococcus | rs76734283 | G | C | -0.106 | 0.020 | 1.86E-07 | 27.974 |
| genus | Streptococcus | rs7306985 | C | T | -0.106 | 0.020 | 1.93E-07 | 27.970 |
| genus | Streptococcus | rs76222580 | T | A | -0.107 | 0.020 | 2.01E-07 | 27.951 |
| genus | Peptococcus | rs1372155 | A | C | -0.119 | 0.022 | 1.71E-07 | 27.944 |
| genus | RuminococcaceaeUCG011 | rs35202762 | C | T | -0.160 | 0.030 | 9.98E-08 | 27.929 |
| genus | Bifidobacterium | rs35564151 | A | G | 0.064 | 0.012 | 8.56E-08 | 27.928 |
| genus | Peptococcus | rs6531356 | G | A | -0.119 | 0.022 | 1.78E-07 | 27.919 |
| genus | Bifidobacterium | rs16833335 | G | A | 0.067 | 0.013 | 1.29E-07 | 27.869 |
| genus |  | rs11666792 | G | A | -0.056 | 0.011 | 1.37E-07 | 27.844 |
| genus | Butyricimonas | rs113054641 | A | G | -0.145 | 0.027 | 1.74E-07 | 27.842 |
| genus | RikenellaceaeRC9gutgroup | rs2900503 | T | G | -0.172 | 0.033 | 1.55E-07 | 27.829 |
| genus | RikenellaceaeRC9gutgroup | rs7020474 | A | G | -0.180 | 0.034 | 1.62E-07 | 27.823 |
| genus | Peptococcus | rs9991605 | T | G | -0.119 | 0.022 | 1.91E-07 | 27.818 |
| genus | Peptococcus | rs10015798 | C | T | -0.118 | 0.022 | 1.84E-07 | 27.815 |
| genus | Bifidobacterium | rs58080116 | A | G | 0.100 | 0.019 | 1.81E-07 | 27.807 |
| genus | Bifidobacterium | rs1123848 | C | T | 0.067 | 0.013 | 1.24E-07 | 27.802 |
| genus | Peptococcus | rs28778129 | C | A | -0.118 | 0.022 | 1.94E-07 | 27.725 |
| genus | Butyricimonas | rs111284490 | C | T | -0.144 | 0.027 | 1.77E-07 | 27.690 |
| genus | Ruminococcus1 | rs10769160 | C | T | -0.059 | 0.011 | 1.69E-07 | 27.686 |
| genus | Faecalibacterium | rs10862584 | G | A | 0.084 | 0.016 | 1.22E-07 | 27.659 |
| genus | Bifidobacterium | rs1964776 | C | T | 0.067 | 0.013 | 1.38E-07 | 27.647 |
| genus | Peptococcus | rs2375445 | C | G | -0.118 | 0.022 | 2.00E-07 | 27.645 |
| genus | Bifidobacterium | rs6430580 | A | G | 0.092 | 0.018 | 8.04E-08 | 27.644 |
| genus | Peptococcus | rs7441225 | T | C | -0.118 | 0.022 | 2.04E-07 | 27.634 |
| genus | Peptococcus | rs6856177 | T | G | -0.118 | 0.022 | 1.99E-07 | 27.621 |
| genus | Peptococcus | rs1899284 | G | C | -0.118 | 0.022 | 2.04E-07 | 27.603 |
| genus | Erysipelatoclostridium | rs4791963 | G | A | -0.076 | 0.015 | 1.65E-07 | 27.585 |
| genus |  | rs12979891 | C | T | -0.056 | 0.011 | 1.58E-07 | 27.571 |
| genus | Bifidobacterium | rs4954578 | T | C | 0.067 | 0.013 | 1.63E-07 | 27.564 |
| genus | Bifidobacterium | rs6708336 | T | A | 0.063 | 0.012 | 1.20E-07 | 27.563 |
| genus | Enterorhabdus | rs13126578 | A | C | 0.087 | 0.016 | 1.44E-07 | 27.558 |
| genus | Intestinibacter | rs4327025 | A | G | -0.081 | 0.015 | 1.64E-07 | 27.546 |
| genus | RuminococcaceaeUCG011 | rs35521499 | A | G | -0.178 | 0.034 | 1.33E-07 | 27.545 |
| genus | Peptococcus | rs6531357 | C | T | -0.119 | 0.023 | 2.25E-07 | 27.534 |
| genus | Peptococcus | rs4467591 | G | A | -0.118 | 0.022 | 2.12E-07 | 27.529 |
| genus | Bifidobacterium | rs2322812 | A | G | 0.099 | 0.019 | 2.00E-07 | 27.527 |
| genus | Peptococcus | rs4277795 | T | A | -0.118 | 0.022 | 2.18E-07 | 27.493 |
| genus |  | rs6893333 | C | G | -0.121 | 0.023 | 1.56E-07 | 27.477 |
| genus | Peptococcus | rs9684906 | C | T | -0.118 | 0.022 | 2.22E-07 | 27.464 |
| genus | Bifidobacterium | rs2090661 | A | G | 0.068 | 0.013 | 1.80E-07 | 27.457 |
| genus | RuminococcaceaeUCG013 | rs10903693 | C | A | -0.060 | 0.011 | 1.82E-07 | 27.430 |
| genus | Bifidobacterium | rs3769011 | A | T | 0.099 | 0.019 | 2.08E-07 | 27.418 |
| genus | Oxalobacter | rs10746751 | T | C | 0.110 | 0.021 | 1.72E-07 | 27.418 |
| genus | Oxalobacter | rs11140800 | A | C | 0.111 | 0.021 | 1.71E-07 | 27.402 |
| genus | Peptococcus | rs988769 | C | G | -0.118 | 0.022 | 2.28E-07 | 27.390 |
| genus | Bifidobacterium | rs9677760 | A | T | 0.076 | 0.015 | 4.01E-07 | 27.384 |
| genus | Ruminococcus1 | rs34264431 | A | G | -0.060 | 0.011 | 2.07E-07 | 27.381 |
| genus | Holdemanella | rs9365544 | T | C | 0.094 | 0.018 | 2.52E-07 | 27.376 |
| genus | Oxalobacter | rs7644098 | G | C | 0.127 | 0.024 | 1.69E-07 | 27.373 |
| genus | Peptococcus | rs11939412 | T | C | -0.118 | 0.022 | 2.39E-07 | 27.365 |
| genus |  | rs4583233 | C | A | 0.067 | 0.013 | 2.84E-07 | 27.363 |
| genus | Bifidobacterium | rs578384 | C | T | -0.064 | 0.012 | 1.19E-07 | 27.339 |
| genus | Peptococcus | rs2165453 | A | G | -0.118 | 0.023 | 2.41E-07 | 27.330 |
| genus | Oxalobacter | rs7643618 | G | A | 0.127 | 0.024 | 1.75E-07 | 27.328 |
| genus | Peptococcus | rs10028591 | G | A | -0.118 | 0.023 | 2.75E-07 | 27.317 |
| genus | Peptococcus | rs1583888 | T | C | -0.118 | 0.023 | 2.47E-07 | 27.316 |
| genus | Peptococcus | . | C | T | -0.118 | 0.023 | 2.47E-07 | 27.316 |
| genus |  | rs8112983 | T | C | -0.056 | 0.011 | 1.86E-07 | 27.281 |
| genus | Bifidobacterium | rs62168844 | A | G | 0.102 | 0.020 | 2.87E-07 | 27.272 |
| genus | Peptococcus | rs6838889 | G | A | -0.117 | 0.022 | 2.46E-07 | 27.266 |
| genus |  | rs2638282 | G | A | -0.056 | 0.011 | 1.75E-07 | 27.258 |
| genus | Oxalobacter | rs4481179 | C | G | 0.126 | 0.024 | 1.84E-07 | 27.254 |
| genus | Bifidobacterium | rs309124 | A | G | 0.080 | 0.015 | 2.73E-07 | 27.235 |
| genus | Peptococcus | rs7662185 | C | A | -0.114 | 0.022 | 2.27E-07 | 27.228 |
| genus | Peptococcus | rs425330 | C | T | -0.122 | 0.023 | 3.47E-07 | 27.201 |
| genus | RuminococcaceaeUCG011 | rs17522882 | T | G | -0.177 | 0.034 | 1.61E-07 | 27.194 |
| genus | Peptococcus | rs13120223 | T | G | -0.117 | 0.022 | 2.58E-07 | 27.184 |
| genus |  | rs11878908 | C | T | -0.056 | 0.011 | 1.96E-07 | 27.180 |
| genus | Gordonibacter | rs76287110 | T | A | -0.243 | 0.047 | 1.67E-07 | 27.163 |
| genus | Enterorhabdus | rs62323861 | G | A | 0.086 | 0.016 | 1.83E-07 | 27.137 |
| genus | RuminococcaceaeUCG011 | rs60647205 | C | T | -0.158 | 0.030 | 1.58E-07 | 27.132 |
| genus | Peptococcus | rs10015792 | C | T | -0.117 | 0.022 | 2.64E-07 | 27.130 |
| genus | Peptococcus | rs10006991 | C | A | -0.117 | 0.022 | 2.56E-07 | 27.124 |
| genus | RuminococcaceaeUCG013 | rs12416132 | T | C | -0.062 | 0.012 | 2.20E-07 | 27.122 |
| genus | Peptococcus | rs6834090 | C | A | -0.117 | 0.022 | 2.65E-07 | 27.116 |
| genus | Peptococcus | rs10026737 | T | A | -0.117 | 0.022 | 2.80E-07 | 27.100 |
| genus | Peptococcus | rs6827461 | G | A | -0.117 | 0.022 | 2.67E-07 | 27.096 |
| genus | RuminococcaceaeUCG004 | rs12125734 | T | G | 0.134 | 0.026 | 2.09E-07 | 27.086 |
| genus | Peptococcus | rs7658271 | C | G | -0.114 | 0.022 | 2.67E-07 | 27.084 |
| genus | Bifidobacterium | rs62168897 | C | T | 0.062 | 0.012 | 1.56E-07 | 27.073 |
| genus | Peptococcus | rs7664779 | T | C | -0.117 | 0.022 | 2.71E-07 | 27.070 |
| genus |  | rs519757 | C | A | 0.056 | 0.011 | 1.88E-07 | 27.070 |
| genus | Streptococcus | rs11610034 | T | C | -0.105 | 0.020 | 3.13E-07 | 27.067 |
| genus | Ruminiclostridium9 | rs57665991 | G | C | -0.064 | 0.012 | 2.07E-07 | 27.052 |
| genus | Peptococcus | rs4634245 | C | T | -0.117 | 0.022 | 2.69E-07 | 27.051 |
| genus | Oxalobacter | rs12635823 | A | G | 0.126 | 0.024 | 2.03E-07 | 27.049 |
| genus | Bifidobacterium | rs4954445 | T | A | 0.099 | 0.019 | 2.46E-07 | 27.048 |
| genus | Odoribacter | rs503751 | G | C | 0.062 | 0.012 | 2.08E-07 | 27.047 |
| genus | Peptococcus | rs1899283 | C | G | -0.117 | 0.022 | 2.73E-07 | 27.043 |
| genus | LachnospiraceaeUCG010 | rs11192447 | G | A | 0.127 | 0.024 | 4.69E-07 | 27.039 |
| genus | Gordonibacter | rs74484720 | C | T | -0.243 | 0.047 | 1.84E-07 | 27.030 |
| genus | Holdemania | rs1867876 | C | T | 0.084 | 0.016 | 2.74E-07 | 27.009 |
| genus | Peptococcus | rs11944235 | C | T | -0.114 | 0.022 | 2.79E-07 | 26.987 |
| genus | Lachnoclostridium | rs6112314 | C | A | -0.056 | 0.011 | 2.43E-07 | 26.964 |
| genus | Peptococcus | rs11929973 | A | G | -0.117 | 0.023 | 3.02E-07 | 26.956 |
| genus | Peptococcus | rs1114490 | C | A | -0.117 | 0.022 | 2.85E-07 | 26.947 |
| genus | Peptococcus | rs1899282 | G | C | -0.117 | 0.022 | 2.88E-07 | 26.944 |
| genus | Ruminococcus1 | rs895734 | T | C | -0.058 | 0.011 | 2.57E-07 | 26.935 |
| genus | LachnospiraceaeUCG008 | rs9873555 | C | G | -0.121 | 0.023 | 2.41E-07 | 26.933 |
| genus | Peptococcus | rs9681332 | G | A | 0.167 | 0.032 | 2.70E-07 | 26.930 |
| genus | RuminococcaceaeUCG011 | rs35283097 | C | T | -0.157 | 0.030 | 1.76E-07 | 26.917 |
| genus | RuminococcaceaeUCG011 | rs34705704 | T | C | -0.157 | 0.030 | 1.76E-07 | 26.917 |
| genus | Peptococcus | rs10026574 | G | A | -0.117 | 0.022 | 3.08E-07 | 26.914 |
| genus | Peptococcus | rs56057697 | G | T | 0.167 | 0.032 | 2.68E-07 | 26.902 |
| genus |  | rs13116360 | C | T | 0.154 | 0.030 | 2.94E-07 | 26.896 |
| genus | Bifidobacterium | rs1865452 | C | T | 0.076 | 0.015 | 4.63E-07 | 26.884 |
| genus |  | rs3859439 | C | T | 0.055 | 0.011 | 2.08E-07 | 26.876 |
| genus | Bifidobacterium | rs10496737 | A | G | 0.076 | 0.015 | 4.65E-07 | 26.875 |
| genus | Bifidobacterium | rs61470941 | T | C | 0.098 | 0.019 | 2.13E-07 | 26.874 |
| genus | Peptococcus | rs77534638 | G | A | 0.171 | 0.033 | 2.63E-07 | 26.866 |
| genus | Bifidobacterium | rs10166142 | A | G | 0.062 | 0.012 | 1.76E-07 | 26.865 |
| genus |  | rs838145 | A | G | -0.058 | 0.011 | 1.90E-07 | 26.864 |
| genus | RuminococcaceaeUCG011 | rs12526170 | G | A | -0.157 | 0.030 | 1.74E-07 | 26.862 |
| genus | Bifidobacterium | rs1469996 | A | G | 0.076 | 0.015 | 5.53E-07 | 26.861 |
| genus | Peptococcus | rs2375441 | T | A | -0.117 | 0.023 | 3.12E-07 | 26.855 |
| genus | Paraprevotella | rs2081023 | G | A | -0.123 | 0.024 | 2.64E-07 | 26.854 |
| genus | Bifidobacterium | rs13010498 | G | C | -0.066 | 0.013 | 1.78E-07 | 26.848 |
| genus | Bifidobacterium | rs2034261 | G | T | 0.062 | 0.012 | 1.72E-07 | 26.844 |
| genus | Peptococcus | rs1583890 | T | C | -0.117 | 0.023 | 3.14E-07 | 26.843 |
| genus | Bifidobacterium | rs309122 | A | G | 0.080 | 0.015 | 3.35E-07 | 26.842 |
| genus | Holdemania | rs9500080 | T | C | 0.093 | 0.018 | 4.09E-07 | 26.840 |
| genus | Bifidobacterium | rs3814354 | G | A | 0.062 | 0.012 | 1.79E-07 | 26.831 |
| genus | Peptococcus | rs7683780 | A | G | -0.116 | 0.022 | 3.00E-07 | 26.831 |
| genus | RuminococcaceaeUCG004 | rs9725226 | G | A | 0.132 | 0.025 | 2.58E-07 | 26.829 |
| genus | Peptococcus | rs10026473 | G | A | -0.116 | 0.022 | 3.23E-07 | 26.827 |
| genus | Peptococcus | rs10010382 | G | A | -0.117 | 0.023 | 3.15E-07 | 26.822 |
| genus | Peptococcus | rs1583891 | G | A | -0.117 | 0.023 | 3.20E-07 | 26.815 |
| genus | Peptococcus | rs1837735 | A | G | -0.117 | 0.023 | 3.20E-07 | 26.814 |
| genus |  | rs6048195 | T | A | -0.060 | 0.012 | 2.50E-07 | 26.807 |
| genus | Bifidobacterium | rs74697610 | A | T | 0.109 | 0.021 | 2.00E-07 | 26.805 |
| genus | Peptococcus | rs12646431 | G | T | -0.117 | 0.023 | 3.21E-07 | 26.802 |
| genus | Peptococcus | rs12646436 | C | T | -0.117 | 0.023 | 3.21E-07 | 26.801 |
| genus | Haemophilus | rs4132989 | T | C | -0.090 | 0.017 | 2.70E-07 | 26.797 |
| genus | Bifidobacterium | rs60963894 | T | G | 0.076 | 0.015 | 4.86E-07 | 26.793 |
| genus | Peptococcus | rs7659069 | G | C | -0.117 | 0.023 | 3.25E-07 | 26.784 |
| genus | Peptococcus | rs6839264 | G | A | -0.116 | 0.022 | 3.14E-07 | 26.780 |
| genus | Peptococcus | rs2119531 | C | G | -0.117 | 0.023 | 3.33E-07 | 26.771 |
| genus | Peptococcus | rs1899277 | G | A | -0.117 | 0.023 | 3.35E-07 | 26.771 |
| genus | Streptococcus | rs11110092 | C | T | -0.102 | 0.020 | 2.75E-07 | 26.763 |
| genus | Bifidobacterium | rs1519525 | C | T | 0.066 | 0.013 | 2.32E-07 | 26.758 |
| genus |  | rs17830032 | A | G | -0.161 | 0.031 | 2.39E-07 | 26.749 |
| genus | Bifidobacterium | rs1807356 | T | C | 0.089 | 0.017 | 1.72E-07 | 26.749 |
| genus | Peptococcus | rs77681628 | T | C | 0.200 | 0.039 | 2.69E-07 | 26.745 |
| genus | Peptococcus | rs2375442 | A | G | -0.117 | 0.023 | 3.33E-07 | 26.737 |
| genus | Peptococcus | rs10013010 | G | T | -0.117 | 0.023 | 3.30E-07 | 26.731 |
| genus | Peptococcus | rs7685367 | A | C | -0.116 | 0.022 | 3.15E-07 | 26.722 |
| genus | Tyzzerella3 | rs4147909 | C | T | 0.117 | 0.023 | 3.34E-07 | 26.717 |
| genus |  | rs629504 | G | C | 0.055 | 0.011 | 2.26E-07 | 26.715 |
| genus | Peptococcus | rs4627887 | C | T | -0.116 | 0.022 | 3.17E-07 | 26.714 |
| genus | Lachnoclostridium | rs6081571 | C | T | -0.057 | 0.011 | 2.76E-07 | 26.708 |
| genus |  | rs35337287 | A | T | -0.176 | 0.034 | 4.47E-07 | 26.699 |
| genus | RuminococcaceaeUCG013 | rs12761870 | C | T | -0.062 | 0.012 | 2.68E-07 | 26.699 |
| genus | Peptococcus | rs10023010 | T | A | -0.116 | 0.023 | 3.48E-07 | 26.695 |
| genus | Peptococcus | rs10014912 | T | C | -0.116 | 0.022 | 3.46E-07 | 26.692 |
| genus | Romboutsia | rs61844361 | T | C | 0.082 | 0.016 | 2.28E-07 | 26.680 |
| genus | Bifidobacterium | rs13008147 | A | G | -0.070 | 0.014 | 4.70E-08 | 26.680 |
| genus | RuminococcaceaeUCG003 | rs57295596 | G | T | -0.165 | 0.032 | 3.55E-07 | 26.678 |
| genus | Bifidobacterium | rs56271357 | C | T | 0.062 | 0.012 | 1.87E-07 | 26.676 |
| genus | Ruminococcus1 | rs55681357 | C | T | -0.060 | 0.012 | 3.04E-07 | 26.674 |
| genus |  | rs35398954 | G | A | -0.090 | 0.017 | 5.40E-07 | 26.653 |
| genus | Bifidobacterium | rs3814355 | C | T | 0.062 | 0.012 | 1.79E-07 | 26.650 |
| genus | Oxalobacter | rs6000536 | T | C | -0.131 | 0.025 | 2.06E-07 | 26.637 |
| genus | Peptococcus | rs2375444 | T | C | -0.116 | 0.022 | 3.30E-07 | 26.637 |
| genus | Odoribacter | rs580930 | C | T | 0.061 | 0.012 | 2.56E-07 | 26.628 |
| genus | Peptococcus | rs6858045 | G | T | -0.117 | 0.023 | 3.57E-07 | 26.621 |
| genus | LachnospiraceaeUCG008 | rs7111583 | A | G | 0.092 | 0.018 | 3.08E-07 | 26.617 |
| genus | Peptococcus | rs7678395 | A | G | -0.116 | 0.022 | 3.53E-07 | 26.608 |
| genus | Peptococcus | rs1837737 | A | G | -0.116 | 0.023 | 3.59E-07 | 26.592 |
| genus | Bifidobacterium | rs10928558 | T | C | 0.066 | 0.013 | 2.39E-07 | 26.585 |
| genus | Peptococcus | rs1837736 | T | G | -0.116 | 0.023 | 3.66E-07 | 26.559 |
| genus | Bifidobacterium | rs7565871 | G | C | 0.086 | 0.017 | 5.37E-08 | 26.559 |
| genus | Peptococcus | rs2165455 | C | A | -0.116 | 0.023 | 3.61E-07 | 26.555 |
| genus | Peptococcus | rs7683635 | G | A | -0.116 | 0.023 | 3.68E-07 | 26.551 |
| genus | Streptococcus | rs2131006 | A | G | -0.104 | 0.020 | 4.50E-07 | 26.545 |
| genus | Oxalobacter | rs6445049 | T | G | 0.126 | 0.024 | 2.82E-07 | 26.528 |
| genus | Streptococcus | rs11110294 | A | G | -0.104 | 0.020 | 4.14E-07 | 26.522 |
| genus |  | rs2452170 | G | A | -0.055 | 0.011 | 2.69E-07 | 26.509 |
| genus | Peptococcus | rs2028446 | C | T | -0.116 | 0.022 | 3.64E-07 | 26.506 |
| genus | Peptococcus | rs6826721 | T | G | -0.116 | 0.023 | 3.79E-07 | 26.504 |
| genus | Butyricimonas | rs17002267 | C | G | -0.141 | 0.027 | 5.01E-07 | 26.503 |
| genus | Gordonibacter | rs7294633 | T | C | 0.129 | 0.025 | 3.44E-07 | 26.501 |
| genus | Oxalobacter | rs6445047 | C | T | 0.126 | 0.024 | 2.78E-07 | 26.500 |
| genus | Peptococcus | rs11945212 | C | T | -0.116 | 0.022 | 3.81E-07 | 26.499 |
| genus | RuminococcaceaeNK4A214group | rs5994253 | G | A | -0.081 | 0.016 | 2.35E-07 | 26.493 |
| genus | Bifidobacterium | rs10193587 | T | C | 0.067 | 0.013 | 2.24E-07 | 26.492 |
| genus | Odoribacter | rs472234 | G | C | 0.061 | 0.012 | 2.77E-07 | 26.492 |
| genus | Peptococcus | rs13117468 | C | T | -0.116 | 0.023 | 3.82E-07 | 26.481 |
| genus | Bifidobacterium | rs3769023 | G | A | 0.062 | 0.012 | 2.16E-07 | 26.473 |
| genus | Peptococcus | rs28414959 | G | A | -0.116 | 0.022 | 3.87E-07 | 26.467 |
| genus | Bifidobacterium | rs1964459 | C | G | 0.084 | 0.016 | 4.08E-07 | 26.461 |
| genus | Bifidobacterium | rs7595712 | C | T | 0.075 | 0.015 | 6.95E-07 | 26.457 |
| genus | Peptococcus | rs1899279 | A | C | -0.116 | 0.022 | 3.74E-07 | 26.453 |
| genus |  | rs546308 | A | G | 0.055 | 0.011 | 2.61E-07 | 26.448 |
| genus | Streptococcus | rs11110096 | A | G | -0.103 | 0.020 | 3.77E-07 | 26.438 |
| genus | Peptococcus | rs6839270 | G | A | -0.116 | 0.022 | 3.77E-07 | 26.425 |
| genus | Streptococcus | rs11110259 | C | A | -0.104 | 0.020 | 4.25E-07 | 26.422 |
| genus | Bifidobacterium | rs12469551 | G | T | 0.075 | 0.015 | 7.06E-07 | 26.419 |
| genus | RuminococcaceaeUCG004 | rs12047707 | G | A | 0.135 | 0.026 | 2.64E-07 | 26.415 |
| genus | Oxalobacter | rs6772385 | C | A | 0.126 | 0.024 | 2.97E-07 | 26.413 |
| genus |  | rs12888970 | G | A | -0.176 | 0.034 | 3.88E-07 | 26.401 |
| genus | Peptococcus | rs10015291 | A | C | -0.115 | 0.022 | 3.99E-07 | 26.395 |
| genus |  | rs11672900 | A | G | -0.055 | 0.011 | 2.87E-07 | 26.394 |
| genus | Peptococcus | rs2375443 | C | T | -0.116 | 0.023 | 4.00E-07 | 26.377 |
| genus | Peptococcus | rs317065 | C | T | -0.117 | 0.023 | 4.43E-07 | 26.373 |
| genus | Peptococcus | rs10009627 | C | T | -0.116 | 0.023 | 4.07E-07 | 26.357 |
| genus | Subdoligranulum | rs12638227 | C | G | -0.056 | 0.011 | 2.48E-07 | 26.356 |
| genus |  | rs34273878 | C | T | -0.175 | 0.034 | 5.30E-07 | 26.354 |
| genus | RuminococcaceaeUCG004 | rs12032687 | G | A | 0.132 | 0.026 | 3.05E-07 | 26.349 |
| genus | RuminococcaceaeUCG011 | rs6930304 | C | T | -0.175 | 0.034 | 2.53E-07 | 26.342 |
| genus | Peptococcus | rs7679824 | T | C | -0.115 | 0.022 | 4.07E-07 | 26.340 |
| genus | Peptococcus | rs4859297 | A | G | -0.119 | 0.023 | 4.03E-07 | 26.326 |
| genus | LachnospiraceaeUCG008 | rs7111585 | T | G | 0.092 | 0.018 | 3.61E-07 | 26.300 |
| genus | RuminococcaceaeUCG004 | rs10874691 | C | A | 0.132 | 0.026 | 2.96E-07 | 26.299 |
| genus | Anaerofilum | rs816292 | C | T | -0.113 | 0.022 | 2.64E-07 | 26.288 |
| genus | Streptococcus | rs74476543 | G | A | -0.103 | 0.020 | 4.68E-07 | 26.279 |
| genus | Bifidobacterium | rs1947112 | A | G | 0.061 | 0.012 | 2.35E-07 | 26.275 |
| genus | Ruminiclostridium9 | rs73166358 | C | T | -0.063 | 0.012 | 2.87E-07 | 26.270 |
| genus |  | rs33988101 | G | T | -0.055 | 0.011 | 3.09E-07 | 26.257 |
| genus | Peptococcus | rs2165454 | T | C | -0.116 | 0.023 | 4.23E-07 | 26.247 |
| genus | Peptococcus | rs6531359 | C | T | -0.116 | 0.023 | 4.35E-07 | 26.235 |
| genus | RuminococcaceaeUCG003 | rs6759615 | G | A | 0.103 | 0.020 | 7.86E-07 | 26.224 |
| genus | Bifidobacterium | rs4954557 | C | T | 0.067 | 0.013 | 3.06E-07 | 26.222 |
| genus | RuminococcaceaeUCG004 | rs9843412 | G | C | 0.081 | 0.016 | 3.58E-07 | 26.218 |
| genus | Ruminiclostridium9 | rs79990961 | G | A | -0.063 | 0.012 | 3.20E-07 | 26.218 |
| genus | Bifidobacterium | rs4954573 | T | G | 0.066 | 0.013 | 2.65E-07 | 26.211 |
| genus | Peptococcus | rs10026840 | G | A | -0.115 | 0.022 | 4.39E-07 | 26.211 |
| genus | Romboutsia | rs61841467 | A | G | 0.081 | 0.016 | 2.75E-07 | 26.187 |
| genus | Bacteroides | rs6795673 | T | C | 0.054 | 0.011 | 3.38E-07 | 26.183 |
| genus | Peptococcus | rs13115721 | C | T | -0.115 | 0.022 | 4.11E-07 | 26.183 |
| genus | Bifidobacterium | rs6732236 | T | G | 0.075 | 0.015 | 6.53E-07 | 26.182 |
| genus | Bifidobacterium | rs60453613 | G | C | 0.075 | 0.015 | 6.64E-07 | 26.178 |
| genus | Peptococcus | rs2028447 | G | T | -0.115 | 0.022 | 4.30E-07 | 26.174 |
| genus | Peptococcus | rs7696990 | C | T | -0.115 | 0.023 | 4.47E-07 | 26.170 |
| genus | Ruminiclostridium9 | rs73172105 | A | G | -0.064 | 0.012 | 3.75E-07 | 26.165 |
| genus | Oxalobacter | rs6445048 | A | G | 0.125 | 0.024 | 3.45E-07 | 26.162 |
| genus | Bifidobacterium | rs10187990 | A | G | 0.061 | 0.012 | 2.37E-07 | 26.160 |
| genus | RuminococcaceaeUCG011 | rs12525491 | C | T | -0.155 | 0.030 | 2.74E-07 | 26.158 |
| genus | Romboutsia | rs76209010 | C | A | 0.080 | 0.016 | 3.09E-07 | 26.143 |
| genus | Peptococcus | rs12649458 | T | G | -0.115 | 0.022 | 4.42E-07 | 26.130 |
| genus | Anaerofilum | rs79598899 | T | C | 0.183 | 0.036 | 3.75E-07 | 26.116 |
| genus | Tyzzerella3 | rs17706273 | C | T | -0.140 | 0.027 | 5.88E-07 | 26.109 |
| genus | Bifidobacterium | rs1469995 | G | A | 0.075 | 0.015 | 6.84E-07 | 26.108 |
| genus | Ruminiclostridium9 | rs12493128 | T | C | -0.063 | 0.012 | 4.15E-07 | 26.081 |
| genus | Romboutsia | rs78114045 | C | T | 0.081 | 0.016 | 2.91E-07 | 26.077 |
| genus | Lactococcus | rs757872 | C | G | 0.141 | 0.028 | 4.37E-07 | 26.058 |
| genus | Faecalibacterium | rs7970542 | A | G | 0.081 | 0.016 | 3.70E-07 | 26.049 |
| genus | Streptococcus | rs11110098 | G | A | -0.099 | 0.019 | 4.91E-07 | 26.044 |
| genus | Ruminiclostridium9 | rs73170375 | A | C | -0.063 | 0.012 | 3.54E-07 | 26.038 |
| genus | RuminococcaceaeUCG011 | rs7752187 | C | G | -0.154 | 0.030 | 2.93E-07 | 26.029 |
| genus | Peptococcus | rs6531354 | A | G | -0.115 | 0.023 | 4.81E-07 | 26.025 |
| genus | Holdemania | rs6133067 | C | T | 0.091 | 0.018 | 5.17E-07 | 26.020 |
| genus | Peptococcus | rs6851451 | C | T | -0.115 | 0.023 | 4.86E-07 | 26.008 |
| genus | Roseburia | rs2034589 | C | G | 0.063 | 0.012 | 5.01E-07 | 26.008 |
| genus | Oxalobacter | rs10780692 | T | C | 0.108 | 0.021 | 3.39E-07 | 26.001 |
| genus | Peptococcus | rs6814618 | T | C | -0.115 | 0.022 | 4.68E-07 | 26.000 |
| genus | Gordonibacter | rs75773691 | T | G | -0.238 | 0.047 | 3.25E-07 | 25.997 |
| genus | Romboutsia | rs61841466 | G | A | 0.080 | 0.016 | 3.04E-07 | 25.992 |
| genus | FamilyXIIIAD3011group | rs9852893 | G | C | 0.066 | 0.013 | 3.88E-07 | 25.982 |
| genus | Ruminiclostridium9 | rs73170306 | G | A | -0.063 | 0.012 | 3.28E-07 | 25.968 |
| genus | Bifidobacterium | rs1961273 | T | C | 0.067 | 0.013 | 3.51E-07 | 25.949 |
| genus | Oxalobacter | rs6782568 | C | A | 0.126 | 0.025 | 3.38E-07 | 25.945 |
| genus | Ruminiclostridium9 | rs73170303 | G | A | -0.063 | 0.012 | 3.35E-07 | 25.943 |
| genus | Erysipelatoclostridium | rs710230 | C | T | 0.143 | 0.028 | 6.33E-07 | 25.941 |
| genus | Peptococcus | rs1899281 | T | G | -0.114 | 0.022 | 4.90E-07 | 25.920 |
| genus | Ruminiclostridium9 | rs75936762 | A | G | -0.062 | 0.012 | 3.95E-07 | 25.917 |
| genus | Ruminiclostridium9 | rs4680425 | T | C | -0.063 | 0.012 | 3.25E-07 | 25.915 |
| genus | Peptococcus | rs7690774 | C | T | -0.115 | 0.023 | 5.21E-07 | 25.908 |
| genus | Peptococcus | rs7685819 | C | A | -0.115 | 0.023 | 5.21E-07 | 25.907 |
| genus | LachnospiraceaeFCS020group | rs7249113 | A | G | 0.068 | 0.013 | 3.72E-07 | 25.907 |
| genus | Ruminiclostridium9 | rs10936139 | T | C | -0.063 | 0.012 | 4.38E-07 | 25.900 |
| genus | Coprococcus3 | rs8100692 | C | T | 0.058 | 0.011 | 4.16E-07 | 25.892 |
| genus | Bifidobacterium | rs3739022 | G | A | 0.096 | 0.019 | 3.60E-07 | 25.889 |
| genus | Romboutsia | rs74668791 | T | G | 0.080 | 0.016 | 3.66E-07 | 25.884 |
| genus | Peptococcus | rs6531353 | G | C | -0.115 | 0.023 | 5.21E-07 | 25.882 |
| genus | Peptococcus | rs74429934 | A | G | 0.164 | 0.032 | 4.66E-07 | 25.877 |
| genus | Oxalobacter | rs7625948 | C | G | 0.124 | 0.024 | 3.84E-07 | 25.868 |
| genus | Ruminiclostridium9 | rs6784968 | T | C | -0.063 | 0.012 | 3.48E-07 | 25.867 |
| genus | Streptococcus | rs11110086 | G | A | -0.102 | 0.020 | 5.15E-07 | 25.864 |
| genus | Lachnoclostridium | rs6112304 | G | T | -0.057 | 0.011 | 3.90E-07 | 25.861 |
| genus | Enterorhabdus | rs7923280 | T | A | 0.086 | 0.017 | 5.24E-07 | 25.851 |
| genus | Lachnoclostridium | rs6075500 | G | A | -0.057 | 0.011 | 3.95E-07 | 25.847 |
| genus | Peptococcus | rs12649442 | A | G | -0.114 | 0.022 | 4.93E-07 | 25.836 |
| genus | Phascolarctobacterium | rs75882962 | C | T | 0.097 | 0.019 | 3.19E-07 | 25.835 |
| genus | Bifidobacterium | rs4953938 | G | A | 0.061 | 0.012 | 2.71E-07 | 25.830 |
| genus | Ruminiclostridium9 | rs6800218 | C | T | -0.062 | 0.012 | 3.27E-07 | 25.826 |
| genus | RuminococcaceaeUCG004 | rs4908294 | A | C | 0.129 | 0.025 | 4.33E-07 | 25.826 |
| genus | Streptococcus | rs11110085 | C | A | -0.102 | 0.020 | 5.29E-07 | 25.817 |
| genus |  | rs584768 | G | A | -0.054 | 0.011 | 3.83E-07 | 25.816 |
| genus | RuminococcaceaeNK4A214group | rs11586410 | A | G | -0.086 | 0.017 | 3.66E-07 | 25.815 |
| genus | Ruminiclostridium9 | rs73164102 | G | A | -0.063 | 0.012 | 3.91E-07 | 25.805 |
| genus | FamilyXIIIAD3011group | rs72730932 | A | C | -0.090 | 0.018 | 6.89E-07 | 25.798 |
| genus | RuminococcaceaeUCG004 | rs9874831 | A | G | 0.080 | 0.016 | 4.59E-07 | 25.794 |
| genus | Peptococcus | rs6531358 | T | C | -0.115 | 0.023 | 5.40E-07 | 25.794 |
| genus | Streptococcus | rs10860564 | T | C | -0.102 | 0.020 | 5.55E-07 | 25.784 |
| genus | Gordonibacter | rs77299262 | T | C | -0.237 | 0.047 | 3.50E-07 | 25.784 |
| genus | LachnospiraceaeUCG008 | rs7119296 | G | A | 0.092 | 0.018 | 4.75E-07 | 25.776 |
| genus | Lachnoclostridium | rs6035270 | G | T | -0.056 | 0.011 | 4.50E-07 | 25.771 |
| genus | Sellimonas | rs2371572 | C | A | 0.127 | 0.025 | 4.46E-07 | 25.770 |
| genus | Ruminiclostridium9 | rs11715599 | G | A | -0.063 | 0.012 | 3.96E-07 | 25.756 |
| genus | Streptococcus | rs11110252 | G | A | -0.102 | 0.020 | 6.22E-07 | 25.751 |
| genus | RuminococcaceaeUCG004 | rs1358460 | A | T | 0.129 | 0.025 | 4.30E-07 | 25.739 |
| genus |  | rs71386687 | G | T | 0.121 | 0.024 | 2.91E-07 | 25.733 |
| genus | Bifidobacterium | rs6708957 | C | T | 0.074 | 0.015 | 8.24E-07 | 25.733 |
| genus |  | rs8063637 | C | G | 0.063 | 0.012 | 5.63E-07 | 25.729 |
| genus | Lachnoclostridium | rs4813352 | A | G | -0.055 | 0.011 | 4.60E-07 | 25.728 |
| genus | Ruminiclostridium9 | rs11717618 | G | C | -0.063 | 0.012 | 4.14E-07 | 25.711 |
| genus | Peptococcus | rs7660143 | A | G | -0.114 | 0.023 | 5.77E-07 | 25.709 |
| genus | Ruminiclostridium9 | rs12496934 | G | A | -0.063 | 0.012 | 4.75E-07 | 25.708 |
| genus | Howardella | rs901099 | G | T | -0.127 | 0.025 | 6.53E-07 | 25.689 |
| genus | Ruminiclostridium6 | rs1756364 | T | G | 0.100 | 0.020 | 2.54E-07 | 25.681 |
| genus | Romboutsia | rs11595451 | C | T | 0.087 | 0.017 | 2.71E-07 | 25.677 |
| genus | Oxalobacter | rs6785343 | C | T | 0.125 | 0.025 | 3.98E-07 | 25.669 |
| genus |  | rs12978752 | A | C | -0.054 | 0.011 | 4.20E-07 | 25.668 |
| genus |  | rs4002471 | C | T | -0.054 | 0.011 | 4.28E-07 | 25.651 |
| genus | Oxalobacter | rs4423796 | C | T | 0.125 | 0.025 | 4.04E-07 | 25.646 |
| genus | Peptococcus | rs6854595 | C | T | -0.114 | 0.023 | 5.87E-07 | 25.638 |
| genus | RuminococcaceaeUCG002 | rs77564310 | C | A | -0.071 | 0.014 | 3.29E-07 | 25.630 |
| genus | Coprococcus3 | rs62481985 | C | G | -0.058 | 0.012 | 4.18E-07 | 25.623 |
| genus | RuminococcaceaeUCG005 | rs10950694 | C | T | 0.058 | 0.011 | 4.30E-07 | 25.620 |
| genus | Romboutsia | rs41335345 | C | T | 0.087 | 0.017 | 2.73E-07 | 25.617 |
| genus | Ruminiclostridium9 | rs11719124 | G | A | -0.063 | 0.012 | 5.40E-07 | 25.616 |
| genus | Lachnoclostridium | rs6035284 | A | G | -0.055 | 0.011 | 4.90E-07 | 25.614 |
| genus | Lachnoclostridium | rs6081562 | A | T | -0.057 | 0.011 | 4.51E-07 | 25.603 |
| genus | Parasutterella | rs2387977 | C | T | -0.068 | 0.013 | 5.38E-07 | 25.597 |
| genus | Ruminiclostridium9 | rs1851062 | G | A | -0.062 | 0.012 | 4.03E-07 | 25.559 |
| genus | Ruminiclostridium9 | rs73168799 | T | C | -0.063 | 0.012 | 4.25E-07 | 25.555 |
| genus |  | rs2047242 | G | A | -0.068 | 0.013 | 2.46E-07 | 25.552 |
| genus | Bifidobacterium | rs16832440 | G | A | 0.083 | 0.016 | 6.68E-07 | 25.546 |
| genus | Ruminiclostridium9 | rs6767494 | G | A | -0.062 | 0.012 | 4.61E-07 | 25.545 |
| genus |  | rs418464 | T | C | -0.055 | 0.011 | 4.17E-07 | 25.531 |
| genus | Holdemanella | rs2763994 | G | A | 0.093 | 0.018 | 7.02E-07 | 25.530 |
| genus | RuminococcaceaeUCG004 | rs10782929 | C | A | 0.129 | 0.025 | 5.12E-07 | 25.527 |
| genus | Oxalobacter | rs4525896 | G | A | 0.125 | 0.025 | 4.45E-07 | 25.517 |
| genus | Ruminiclostridium9 | rs112014459 | G | T | -0.062 | 0.012 | 4.21E-07 | 25.509 |
| genus | Anaerofilum | rs1384873 | C | G | -0.112 | 0.022 | 3.88E-07 | 25.491 |
| genus |  | rs10071259 | A | C | -0.115 | 0.023 | 6.06E-07 | 25.487 |
| genus | Ruminiclostridium9 | rs4680421 | G | A | -0.062 | 0.012 | 4.11E-07 | 25.485 |
| genus | Bifidobacterium | rs872151 | C | T | 0.094 | 0.019 | 4.14E-07 | 25.470 |
| genus |  | rs12978750 | A | G | -0.054 | 0.011 | 4.66E-07 | 25.462 |
| genus |  | rs9648214 | C | T | -0.083 | 0.016 | 2.52E-07 | 25.438 |
| genus | Romboutsia | rs61841463 | C | T | 0.079 | 0.016 | 4.38E-07 | 25.431 |
| genus | Ruminiclostridium9 | rs4680435 | G | A | -0.063 | 0.012 | 6.11E-07 | 25.422 |
| genus | LachnospiraceaeUCG008 | rs10899031 | C | T | 0.091 | 0.018 | 5.80E-07 | 25.422 |
| genus | Oxalobacter | rs7428410 | C | T | 0.125 | 0.025 | 4.62E-07 | 25.414 |
| genus | Ruminiclostridium9 | rs6441193 | A | G | -0.062 | 0.012 | 5.03E-07 | 25.412 |
| genus | Peptococcus | rs9684110 | T | C | -0.110 | 0.022 | 5.84E-07 | 25.409 |
| genus | Bifidobacterium | rs6716987 | C | A | 0.064 | 0.013 | 4.77E-07 | 25.405 |
| genus |  | rs12659196 | T | C | -0.116 | 0.023 | 5.21E-07 | 25.405 |
| genus |  | rs12979278 | C | T | -0.054 | 0.011 | 4.88E-07 | 25.387 |
| genus | Ruminiclostridium9 | rs12053865 | A | T | -0.062 | 0.012 | 5.07E-07 | 25.382 |
| genus | Bifidobacterium | rs7564577 | C | T | 0.086 | 0.017 | 2.19E-07 | 25.381 |
| genus | Peptococcus | rs1348141 | C | T | -0.111 | 0.022 | 6.59E-07 | 25.381 |
| genus | Bifidobacterium | rs6430585 | C | A | 0.073 | 0.015 | 1.15E-06 | 25.375 |
| genus | DefluviitaleaceaeUCG011 | rs12500663 | G | T | -0.115 | 0.023 | 1.51E-06 | 25.374 |
| genus | Romboutsia | rs61844360 | G | A | 0.080 | 0.016 | 4.86E-07 | 25.368 |
| genus | Flavonifractor | rs12030302 | G | A | -0.069 | 0.014 | 5.61E-07 | 25.361 |
| genus | DefluviitaleaceaeUCG011 | rs2357086 | C | T | -0.110 | 0.022 | 1.33E-06 | 25.356 |
| genus |  | rs55750250 | A | G | -0.082 | 0.016 | 2.68E-07 | 25.354 |
| genus | Bifidobacterium | rs56036623 | T | C | 0.070 | 0.014 | 3.31E-07 | 25.328 |
| genus | Bifidobacterium | rs1374289 | C | T | 0.060 | 0.012 | 3.66E-07 | 25.328 |
| genus | Butyricimonas | rs8127278 | A | G | -0.138 | 0.027 | 5.67E-07 | 25.325 |
| genus | Ruminiclostridium9 | rs4680413 | A | G | -0.062 | 0.012 | 5.00E-07 | 25.320 |
| genus |  | rs7084821 | A | G | -0.067 | 0.013 | 2.99E-07 | 25.310 |
| genus | Ruminiclostridium9 | rs73170310 | T | C | -0.062 | 0.012 | 4.73E-07 | 25.310 |
| genus | Romboutsia | rs7913601 | C | T | 0.079 | 0.016 | 4.76E-07 | 25.307 |
| genus | Bifidobacterium | rs147616090 | C | T | 0.074 | 0.015 | 1.05E-06 | 25.306 |
| genus | Ruminiclostridium9 | rs73164066 | T | C | -0.060 | 0.012 | 6.18E-07 | 25.306 |
| genus | RuminococcaceaeUCG004 | rs12129963 | T | A | 0.128 | 0.025 | 5.24E-07 | 25.301 |
| genus | Ruminiclostridium9 | rs17627933 | A | G | -0.062 | 0.012 | 6.03E-07 | 25.286 |
| genus | Ruminiclostridium9 | rs60962704 | C | T | -0.062 | 0.012 | 4.84E-07 | 25.282 |
| genus | Victivallis | rs56349194 | G | A | -0.159 | 0.032 | 6.26E-07 | 25.282 |
| genus | Blautia | rs11149971 | T | C | 0.118 | 0.023 | 1.04E-06 | 25.281 |
| genus | Lachnoclostridium | rs2876535 | G | A | -0.056 | 0.011 | 5.23E-07 | 25.273 |
| genus | RuminococcaceaeUCG004 | rs35091045 | T | C | 0.129 | 0.026 | 5.03E-07 | 25.262 |
| genus | Ruminiclostridium9 | rs12053869 | A | C | -0.062 | 0.012 | 5.50E-07 | 25.253 |
| genus |  | rs57381059 | G | A | -0.083 | 0.016 | 2.73E-07 | 25.248 |
| genus |  | rs10039058 | C | A | -0.114 | 0.023 | 6.93E-07 | 25.241 |
| genus | DefluviitaleaceaeUCG011 | rs12501518 | A | G | -0.110 | 0.022 | 1.40E-06 | 25.237 |
| genus | Bacteroides | rs6810046 | A | G | 0.053 | 0.011 | 5.56E-07 | 25.228 |
| genus | Lachnoclostridium | rs2143855 | G | A | -0.054 | 0.011 | 5.88E-07 | 25.225 |
| genus | Tyzzerella3 | rs17706248 | C | T | -0.138 | 0.027 | 1.02E-06 | 25.223 |
| genus | Ruminiclostridium9 | rs7652970 | G | A | -0.062 | 0.012 | 5.70E-07 | 25.205 |
| genus |  | rs479486 | G | A | -0.053 | 0.011 | 5.29E-07 | 25.203 |
| genus | Bifidobacterium | rs309133 | A | C | 0.079 | 0.016 | 5.74E-07 | 25.198 |
| genus | Bifidobacterium | rs60274701 | G | A | 0.094 | 0.019 | 6.43E-07 | 25.189 |
| genus | Marvinbryantia | rs2724813 | G | A | -0.084 | 0.017 | 6.28E-07 | 25.180 |
| genus | Ruminiclostridium9 | rs12637649 | A | G | -0.062 | 0.012 | 5.90E-07 | 25.178 |
| genus | Bacteroides | rs11585893 | G | A | -0.074 | 0.015 | 1.80E-06 | 25.175 |
| genus | Bifidobacterium | rs6733130 | A | C | 0.060 | 0.012 | 3.57E-07 | 25.168 |
| genus | DefluviitaleaceaeUCG011 | rs72731813 | T | C | -0.147 | 0.029 | 4.33E-07 | 25.165 |
| genus | Alistipes | rs9712594 | A | C | -0.090 | 0.018 | 4.78E-07 | 25.165 |
| genus | RuminococcaceaeUCG004 | rs12030718 | T | C | 0.129 | 0.026 | 5.29E-07 | 25.164 |
| genus | RuminococcaceaeUCG004 | rs11164703 | A | T | 0.129 | 0.026 | 5.33E-07 | 25.162 |
| genus | RuminococcaceaeUCG004 | rs12143297 | C | A | 0.129 | 0.026 | 5.21E-07 | 25.156 |
| genus | Victivallis | rs4396289 | T | C | -0.158 | 0.032 | 6.54E-07 | 25.153 |
| genus | Peptococcus | rs11001948 | A | C | -0.191 | 0.038 | 1.96E-06 | 25.132 |
| genus | ErysipelotrichaceaeUCG003 | rs11877693 | G | C | -0.095 | 0.019 | 1.39E-06 | 25.120 |
| genus | RuminococcaceaeUCG002 | rs55793120 | C | T | 0.137 | 0.027 | 4.81E-07 | 25.119 |
| genus | Bifidobacterium | rs309123 | C | G | 0.078 | 0.016 | 6.42E-07 | 25.116 |
| genus | Alloprevotella | rs12675596 | T | G | 0.146 | 0.029 | 9.64E-07 | 25.114 |
| genus | Dorea | rs62503162 | G | A | -0.097 | 0.019 | 7.47E-07 | 25.113 |
| genus | Oxalobacter | rs4622921 | A | G | 0.124 | 0.025 | 5.48E-07 | 25.098 |
| genus | Sellimonas | rs35344361 | T | G | 0.126 | 0.025 | 6.34E-07 | 25.095 |
| genus | Bifidobacterium | rs12476127 | A | G | 0.080 | 0.016 | 8.06E-07 | 25.092 |
| genus | Bifidobacterium | rs80274970 | C | T | 0.104 | 0.021 | 6.53E-07 | 25.087 |
| genus | Marvinbryantia | rs61884471 | A | G | 0.124 | 0.025 | 1.01E-06 | 25.085 |
| genus |  | rs11617697 | G | A | -0.143 | 0.029 | 7.22E-07 | 25.080 |
| genus | LachnospiraceaeUCG008 | rs13087616 | G | A | -0.116 | 0.023 | 5.82E-07 | 25.076 |
| genus | Bifidobacterium | rs12471781 | G | A | 0.083 | 0.016 | 7.76E-07 | 25.076 |
| genus | RuminococcaceaeUCG004 | rs2056752 | T | G | 0.128 | 0.025 | 6.18E-07 | 25.055 |
| genus | Ruminiclostridium9 | rs73170352 | A | C | -0.062 | 0.012 | 5.97E-07 | 25.039 |
| genus | Coprococcus2 | rs59936925 | T | A | 0.117 | 0.023 | 9.38E-07 | 25.038 |
| genus | Bifidobacterium | rs12992815 | C | G | 0.094 | 0.019 | 5.79E-07 | 25.031 |
| genus | Slackia | rs8901 | T | C | 0.093 | 0.019 | 6.07E-07 | 25.028 |
| genus | Tyzzerella3 | rs12979724 | A | G | -0.100 | 0.020 | 8.15E-07 | 25.017 |
| genus | Peptococcus | rs10020396 | C | T | -0.113 | 0.023 | 8.07E-07 | 25.015 |
| genus | Lachnoclostridium | rs6081572 | G | A | -0.056 | 0.011 | 5.86E-07 | 25.014 |
| genus | Ruminiclostridium9 | rs6800853 | G | A | -0.062 | 0.012 | 6.18E-07 | 25.011 |
| genus | Lachnoclostridium | rs8121292 | G | A | -0.054 | 0.011 | 6.52E-07 | 25.006 |
| genus | LachnospiraceaeUCG008 | rs10741777 | C | T | -0.097 | 0.019 | 7.69E-07 | 24.994 |
| genus | RuminococcaceaeUCG004 | rs11164705 | A | G | 0.129 | 0.026 | 5.88E-07 | 24.992 |
| genus | Oxalobacter | rs4877289 | G | A | 0.104 | 0.021 | 5.69E-07 | 24.990 |
| genus | Streptococcus | rs12370772 | G | C | -0.101 | 0.020 | 9.54E-07 | 24.986 |
| genus | RuminococcaceaeUCG004 | rs11164723 | G | A | 0.129 | 0.026 | 5.88E-07 | 24.984 |
| genus |  | rs2116427 | G | A | 0.091 | 0.018 | 4.67E-07 | 24.983 |
| genus | Peptococcus | rs7690572 | C | T | -0.113 | 0.023 | 8.44E-07 | 24.983 |
| genus | Akkermansia | rs11729256 | C | T | 0.075 | 0.015 | 6.58E-07 | 24.970 |
| genus |  | rs17631095 | C | T | -0.114 | 0.023 | 8.40E-07 | 24.966 |
| genus | Barnesiella | rs2276875 | G | A | -0.070 | 0.014 | 4.65E-07 | 24.963 |
| genus | Oxalobacter | rs34124854 | C | T | 0.123 | 0.025 | 6.03E-07 | 24.962 |
| genus | ErysipelotrichaceaeUCG003 | rs76502207 | C | T | 0.145 | 0.029 | 6.41E-07 | 24.958 |
| genus | Peptococcus | rs1348138 | T | C | -0.113 | 0.023 | 8.27E-07 | 24.957 |
| genus | Romboutsia | rs7075681 | G | C | 0.079 | 0.016 | 5.99E-07 | 24.956 |
| genus |  | rs13180307 | C | T | -0.114 | 0.023 | 8.46E-07 | 24.943 |
| genus | LachnospiraceaeUCG001 | rs2091024 | T | C | 0.093 | 0.019 | 8.21E-07 | 24.942 |
| genus | Lachnoclostridium | rs6035293 | A | G | -0.054 | 0.011 | 6.93E-07 | 24.937 |
| genus |  | rs10953312 | A | C | -0.056 | 0.011 | 6.10E-07 | 24.936 |
| genus | Oxalobacter | rs2132164 | G | C | 0.123 | 0.025 | 5.77E-07 | 24.934 |
| genus | Parasutterella | rs7572229 | A | G | 0.066 | 0.013 | 6.32E-07 | 24.929 |
| genus | Butyricimonas | rs2823125 | A | G | -0.136 | 0.027 | 9.77E-07 | 24.921 |
| genus | LachnospiraceaeFCS020group | rs12981905 | T | C | 0.067 | 0.013 | 6.05E-07 | 24.915 |
| genus | Butyricimonas | rs62390301 | C | T | -0.087 | 0.017 | 7.42E-07 | 24.915 |
| genus | RuminococcaceaeUCG004 | rs12129353 | T | A | 0.128 | 0.026 | 6.10E-07 | 24.912 |
| genus | Ruminiclostridium9 | rs7624303 | A | G | -0.062 | 0.012 | 6.24E-07 | 24.910 |
| genus | Bifidobacterium | rs78793148 | C | A | 0.099 | 0.020 | 9.44E-07 | 24.906 |
| genus | Ruminiclostridium9 | rs4680428 | C | T | -0.061 | 0.012 | 5.83E-07 | 24.906 |
| genus | Ruminiclostridium9 | rs11716969 | A | G | -0.062 | 0.012 | 6.95E-07 | 24.896 |
| genus | Streptococcus | rs57497665 | C | T | -0.093 | 0.019 | 9.91E-07 | 24.895 |
| genus | Peptococcus | rs317066 | A | G | -0.112 | 0.022 | 8.14E-07 | 24.891 |
| genus | Tyzzerella3 | rs4147935 | C | T | 0.100 | 0.020 | 7.85E-07 | 24.888 |
| genus | RikenellaceaeRC9gutgroup | rs10120177 | C | A | -0.170 | 0.034 | 6.66E-07 | 24.885 |
| genus | Bifidobacterium | rs10928523 | C | T | 0.060 | 0.012 | 4.18E-07 | 24.882 |
| genus | RuminococcaceaeUCG004 | rs11164727 | G | T | 0.128 | 0.026 | 6.10E-07 | 24.879 |
| genus | Ruminiclostridium9 | rs55969621 | A | T | -0.061 | 0.012 | 6.52E-07 | 24.878 |
| genus | Peptococcus | rs1978931 | T | A | -0.112 | 0.023 | 8.76E-07 | 24.875 |
| genus | Peptococcus | rs403094 | A | G | -0.112 | 0.022 | 8.58E-07 | 24.873 |
| genus | Peptococcus | rs11001941 | A | G | -0.196 | 0.039 | 1.33E-06 | 24.873 |
| genus | Lachnoclostridium | rs6081560 | A | G | -0.054 | 0.011 | 7.04E-07 | 24.870 |
| genus | Butyrivibrio | rs72723662 | T | C | 0.224 | 0.045 | 7.86E-07 | 24.869 |
| genus | Ruminiclostridium9 | rs138824716 | A | G | -0.062 | 0.012 | 6.33E-07 | 24.857 |
| genus | Ruminiclostridium9 | rs7640075 | T | C | -0.061 | 0.012 | 7.01E-07 | 24.856 |
| genus | Peptococcus | rs430940 | G | T | -0.113 | 0.023 | 9.48E-07 | 24.853 |
| genus | Ruminiclostridium9 | rs145049570 | A | G | -0.062 | 0.012 | 5.43E-07 | 24.852 |
| genus | RuminococcaceaeUCG009 | rs1550196 | A | G | 0.131 | 0.026 | 1.13E-06 | 24.849 |
| genus | Bifidobacterium | rs1374288 | C | T | 0.060 | 0.012 | 4.30E-07 | 24.846 |
| genus | Oxalobacter | rs4243424 | C | T | 0.123 | 0.025 | 6.27E-07 | 24.845 |
| genus | Oxalobacter | rs35327886 | A | G | 0.123 | 0.025 | 6.36E-07 | 24.832 |
| genus | Oxalobacter | rs1317940 | A | G | 0.123 | 0.025 | 6.14E-07 | 24.828 |
| genus | Bifidobacterium | rs1374287 | C | T | 0.060 | 0.012 | 4.31E-07 | 24.828 |
| genus | Peptococcus | rs6531352 | A | G | -0.113 | 0.023 | 8.94E-07 | 24.827 |
| genus | Ruminiclostridium9 | rs11716327 | G | T | -0.062 | 0.012 | 7.80E-07 | 24.815 |
| genus | Ruminiclostridium9 | rs73168723 | G | C | -0.062 | 0.012 | 5.78E-07 | 24.804 |
| genus | Lachnoclostridium | rs6075497 | A | T | -0.056 | 0.011 | 6.36E-07 | 24.803 |
| genus | Anaerostipes | rs7193624 | T | C | 0.075 | 0.015 | 5.35E-07 | 24.803 |
| genus | Streptococcus | rs74659220 | A | C | -0.091 | 0.018 | 7.37E-07 | 24.803 |
| genus | Bifidobacterium | rs2322251 | C | T | 0.060 | 0.012 | 4.94E-07 | 24.800 |
| genus | Terrisporobacter | rs1883097 | T | C | 0.226 | 0.045 | 4.16E-07 | 24.798 |
| genus | Ruminiclostridium9 | rs11707386 | G | A | -0.062 | 0.012 | 7.75E-07 | 24.795 |
| genus | RuminococcaceaeUCG004 | rs12136508 | A | G | 0.128 | 0.026 | 6.75E-07 | 24.789 |
| genus | FamilyXIIIAD3011group | rs16840310 | G | A | -0.061 | 0.012 | 6.75E-07 | 24.780 |
| genus | Gordonibacter | rs7220558 | T | A | 0.117 | 0.023 | 6.71E-07 | 24.774 |
| genus | Ruminococcus1 | rs4148900 | A | C | -0.058 | 0.012 | 7.83E-07 | 24.773 |
| genus | Bifidobacterium | rs7589832 | A | C | 0.073 | 0.015 | 1.50E-06 | 24.771 |
| genus | Lactococcus | rs123059 | C | T | -0.137 | 0.027 | 1.27E-06 | 24.769 |
| genus | Ruminococcus1 | rs386753175 | T | A | -0.055 | 0.011 | 6.45E-07 | 24.766 |
| genus | LachnospiraceaeUCG001 | rs9824897 | C | A | 0.093 | 0.019 | 9.99E-07 | 24.761 |
| genus | Ruminiclostridium9 | rs73170384 | A | C | -0.062 | 0.012 | 7.75E-07 | 24.760 |
| genus | Ruminiclostridium9 | rs12639310 | G | A | -0.061 | 0.012 | 6.91E-07 | 24.754 |
| genus | Oxalobacter | rs4280664 | G | A | 0.123 | 0.025 | 6.54E-07 | 24.753 |
| genus | RuminococcaceaeUCG004 | rs1528208 | C | G | 0.127 | 0.025 | 7.12E-07 | 24.745 |
| genus | Ruminiclostridium9 | rs12639261 | G | T | -0.061 | 0.012 | 6.97E-07 | 24.738 |
| genus | RuminococcaceaeUCG003 | rs62177720 | C | T | 0.099 | 0.020 | 1.16E-06 | 24.729 |
| genus | Bifidobacterium | rs112407062 | C | T | 0.081 | 0.016 | 8.70E-07 | 24.729 |
| genus | Ruminiclostridium9 | rs73170364 | C | A | -0.061 | 0.012 | 7.25E-07 | 24.725 |
| genus | Ruminiclostridium9 | rs4402974 | A | C | -0.062 | 0.012 | 7.97E-07 | 24.724 |
| genus | Ruminiclostridium9 | rs4516653 | T | C | -0.062 | 0.012 | 7.97E-07 | 24.724 |
| genus |  | rs62349875 | T | A | -0.113 | 0.023 | 1.01E-06 | 24.721 |
| genus | Peptococcus | rs2889085 | T | C | -0.112 | 0.023 | 9.40E-07 | 24.715 |
| genus | Odoribacter | rs77779484 | A | G | -0.133 | 0.027 | 6.56E-07 | 24.713 |
| genus | Coprococcus1 | rs74101919 | C | T | -0.072 | 0.014 | 1.03E-06 | 24.706 |
| genus | Marvinbryantia | rs61193268 | A | G | 0.123 | 0.025 | 1.31E-06 | 24.702 |
| genus | Dialister | rs11166701 | A | G | -0.066 | 0.013 | 5.51E-07 | 24.698 |
| genus | RuminococcaceaeUCG004 | rs1406124 | C | T | 0.127 | 0.025 | 7.36E-07 | 24.696 |
| genus | LachnospiraceaeUCG008 | rs10766567 | C | T | -0.097 | 0.019 | 8.94E-07 | 24.691 |
| genus | RuminococcaceaeUCG004 | rs10782928 | A | G | 0.127 | 0.025 | 7.42E-07 | 24.686 |
| genus | Butyrivibrio | rs7752361 | G | A | -0.119 | 0.024 | 7.69E-07 | 24.685 |
| genus | Streptococcus | rs72739637 | G | A | 0.096 | 0.019 | 1.03E-06 | 24.684 |
| genus | Peptococcus | rs317067 | T | A | -0.112 | 0.022 | 9.07E-07 | 24.681 |
| genus | RuminococcaceaeUCG004 | rs71655813 | T | C | 0.128 | 0.026 | 7.00E-07 | 24.676 |
| genus | Lachnoclostridium | rs6046001 | C | T | -0.054 | 0.011 | 7.79E-07 | 24.669 |
| genus | RuminococcaceaeUCG004 | rs12024454 | T | C | 0.128 | 0.026 | 6.90E-07 | 24.669 |
| genus | Flavonifractor | rs10493606 | A | G | -0.068 | 0.014 | 8.08E-07 | 24.661 |
| genus | Anaerofilum | rs712981 | C | A | 0.101 | 0.020 | 6.83E-07 | 24.660 |
| genus | Ruminiclostridium9 | rs2139696 | A | G | -0.061 | 0.012 | 7.40E-07 | 24.658 |
| genus | Oxalobacter | rs4894821 | G | A | 0.123 | 0.025 | 6.77E-07 | 24.657 |
| genus | Bifidobacterium | rs73958618 | A | G | 0.072 | 0.015 | 1.43E-06 | 24.655 |
| genus | Ruminiclostridium9 | rs7621889 | T | C | -0.057 | 0.012 | 6.74E-07 | 24.651 |
| genus | Bifidobacterium | rs7604460 | T | C | 0.081 | 0.016 | 1.21E-06 | 24.651 |
| genus | Oxalobacter | rs13060354 | A | G | 0.123 | 0.025 | 6.96E-07 | 24.650 |
| genus | Intestinibacter | rs78948008 | A | T | 0.144 | 0.029 | 1.04E-06 | 24.650 |
| genus | Ruminiclostridium9 | rs58195752 | T | C | -0.061 | 0.012 | 7.53E-07 | 24.641 |
| genus | LachnospiraceaeNK4A136group | rs12362320 | C | G | 0.057 | 0.012 | 8.04E-07 | 24.641 |
| genus | Erysipelatoclostridium | rs4697572 | G | A | -0.081 | 0.016 | 7.59E-07 | 24.640 |
| genus | Oxalobacter | rs13073573 | C | T | 0.123 | 0.025 | 6.69E-07 | 24.631 |
| genus | LachnospiraceaeUCG010 | rs9981767 | C | A | 0.066 | 0.013 | 9.96E-07 | 24.629 |
| genus | Anaerotruncus | rs6563550 | C | T | 0.088 | 0.018 | 2.35E-07 | 24.629 |
| genus | RuminococcaceaeUCG003 | rs10204449 | A | G | 0.099 | 0.020 | 1.42E-06 | 24.625 |
| genus | Butyricimonas | rs78496505 | C | T | -0.087 | 0.017 | 8.60E-07 | 24.622 |
| genus | Ruminiclostridium9 | rs113849803 | C | T | -0.061 | 0.012 | 9.29E-07 | 24.615 |
| genus | Eggerthella | rs2240838 | G | A | 0.098 | 0.020 | 7.36E-07 | 24.613 |
| genus | Oscillibacter | rs11627628 | C | T | 0.144 | 0.029 | 1.01E-06 | 24.605 |
| genus | Peptococcus | rs1348139 | T | C | -0.112 | 0.023 | 9.92E-07 | 24.605 |
| genus | Oxalobacter | rs10780691 | T | C | 0.103 | 0.021 | 7.38E-07 | 24.600 |
| genus | Ruminiclostridium9 | rs73170346 | G | A | -0.061 | 0.012 | 7.48E-07 | 24.599 |
| genus | Ruminiclostridium9 | rs73170337 | C | T | -0.061 | 0.012 | 7.53E-07 | 24.594 |
| genus | Ruminiclostridium9 | rs73170339 | G | A | -0.061 | 0.012 | 7.53E-07 | 24.594 |
| genus | Butyricimonas | rs138323763 | A | G | -0.136 | 0.027 | 8.68E-07 | 24.589 |
| genus |  | rs2909242 | A | C | -0.091 | 0.018 | 7.41E-07 | 24.588 |
| genus | Ruminiclostridium9 | rs73170373 | T | A | -0.061 | 0.012 | 9.26E-07 | 24.582 |
| genus | RuminococcaceaeUCG004 | rs4974363 | G | C | 0.078 | 0.016 | 8.51E-07 | 24.581 |
| genus | Streptococcus | rs12367581 | T | A | -0.100 | 0.020 | 1.04E-06 | 24.578 |
| genus | DefluviitaleaceaeUCG011 | rs4677103 | G | A | 0.098 | 0.020 | 9.60E-07 | 24.576 |
| genus | RuminococcaceaeUCG003 | rs646327 | A | G | 0.059 | 0.012 | 7.83E-07 | 24.567 |
| genus |  | rs12897317 | C | T | -0.169 | 0.034 | 1.06E-06 | 24.562 |
| genus | RuminococcaceaeUCG004 | rs9827785 | C | T | 0.078 | 0.016 | 8.56E-07 | 24.560 |
| genus | LachnospiraceaeUCG008 | rs67078837 | C | T | -0.085 | 0.017 | 7.68E-07 | 24.556 |
| genus | Bifidobacterium | rs3820790 | A | T | 0.095 | 0.019 | 8.74E-07 | 24.525 |
| genus | Oxalobacter | rs4243423 | A | G | 0.122 | 0.025 | 7.41E-07 | 24.520 |
| genus | Marvinbryantia | rs2842896 | T | C | -0.065 | 0.013 | 7.25E-07 | 24.519 |
| genus | LachnospiraceaeUCG008 | rs10734277 | G | A | -0.096 | 0.019 | 7.84E-07 | 24.518 |
| genus | ChristensenellaceaeR | rs999354 | A | T | 0.058 | 0.012 | 7.01E-07 | 24.518 |
| genus | Holdemanella | rs607782 | C | T | -0.085 | 0.017 | 7.19E-07 | 24.518 |
| genus | RuminococcaceaeUCG004 | rs731508 | A | C | 0.078 | 0.016 | 8.96E-07 | 24.514 |
| genus | RuminococcaceaeUCG004 | rs9811826 | G | T | 0.078 | 0.016 | 8.73E-07 | 24.514 |
| genus | RuminococcaceaeUCG004 | rs6445659 | A | G | 0.079 | 0.016 | 8.76E-07 | 24.512 |
| genus | RuminococcaceaeUCG004 | rs9880729 | C | A | 0.078 | 0.016 | 8.68E-07 | 24.512 |
| genus | Ruminiclostridium9 | rs6807468 | T | C | -0.061 | 0.012 | 7.71E-07 | 24.503 |
| genus | Erysipelatoclostridium | rs349442 | C | T | 0.087 | 0.018 | 8.75E-07 | 24.493 |
| genus | Desulfovibrio | . | C | A | 0.085 | 0.017 | 8.55E-07 | 24.492 |
| genus | LachnospiraceaeUCG004 | rs12747809 | A | G | -0.062 | 0.013 | 8.65E-07 | 24.489 |
| genus | Ruminiclostridium9 | rs17683939 | C | T | -0.061 | 0.012 | 6.30E-07 | 24.478 |
| genus | Gordonibacter | rs41268621 | C | G | -0.232 | 0.047 | 6.43E-07 | 24.468 |
| genus | RuminococcaceaeUCG005 | rs60081663 | G | C | 0.158 | 0.032 | 9.28E-07 | 24.467 |
| genus | Butyricicoccus | rs56221232 | C | T | 0.083 | 0.017 | 7.62E-07 | 24.467 |
| genus | Peptococcus | rs1978930 | G | T | -0.112 | 0.023 | 1.07E-06 | 24.458 |
| genus | Bifidobacterium | rs1446524 | A | G | 0.059 | 0.012 | 4.75E-07 | 24.457 |
| genus | Lachnospira | rs13157098 | G | A | -0.077 | 0.016 | 5.99E-07 | 24.456 |
| genus | Ruminiclostridium9 | rs76757621 | T | C | -0.061 | 0.012 | 7.38E-07 | 24.446 |
| genus | Peptococcus | rs1530246 | G | C | -0.113 | 0.023 | 9.49E-07 | 24.446 |
| genus |  | rs73615400 | C | T | -0.096 | 0.019 | 9.54E-07 | 24.443 |
| genus | Peptococcus | rs1441209 | T | C | -0.112 | 0.023 | 1.08E-06 | 24.440 |
| genus | RuminococcaceaeUCG004 | rs12128315 | G | A | 0.127 | 0.026 | 7.51E-07 | 24.440 |
| genus | Butyrivibrio | rs7764591 | C | G | -0.119 | 0.024 | 8.73E-07 | 24.440 |
| genus | Butyrivibrio | rs7750792 | C | T | -0.119 | 0.024 | 8.73E-07 | 24.440 |
| genus | Ruminiclostridium9 | rs34434866 | C | T | -0.061 | 0.012 | 8.26E-07 | 24.435 |
| genus | Bifidobacterium | rs721765 | C | A | 0.081 | 0.016 | 1.16E-06 | 24.433 |
| genus | Lachnoclostridium | rs3790176 | G | A | -0.055 | 0.011 | 8.11E-07 | 24.432 |
| genus | Ruminiclostridium6 | rs71414120 | G | T | 0.201 | 0.041 | 1.08E-06 | 24.432 |
| genus | LachnospiraceaeUCG008 | rs6765500 | T | G | -0.114 | 0.023 | 8.22E-07 | 24.428 |
| genus | Ruminiclostridium9 | rs12054114 | T | C | -0.061 | 0.012 | 8.19E-07 | 24.426 |
| genus |  | rs439523 | T | C | -0.053 | 0.011 | 7.96E-07 | 24.425 |
| genus | Bifidobacterium | rs115553816 | G | A | 0.081 | 0.016 | 1.05E-06 | 24.422 |
| genus | Ruminiclostridium9 | rs12053861 | G | A | -0.061 | 0.012 | 8.21E-07 | 24.420 |
| genus |  | rs838147 | G | A | -0.053 | 0.011 | 8.36E-07 | 24.418 |
| genus | RuminococcaceaeUCG004 | rs1375521 | G | A | 0.078 | 0.016 | 9.06E-07 | 24.415 |
| genus | Bifidobacterium | rs10496738 | C | T | 0.081 | 0.016 | 1.22E-06 | 24.414 |
| genus |  | rs4609756 | G | T | -0.169 | 0.034 | 1.39E-06 | 24.412 |
| genus |  | rs4432189 | A | T | -0.169 | 0.034 | 1.39E-06 | 24.412 |
| genus | Bifidobacterium | rs4954272 | A | G | 0.087 | 0.018 | 8.24E-07 | 24.411 |
| genus | Lachnoclostridium | rs6075498 | A | C | -0.055 | 0.011 | 7.73E-07 | 24.408 |
| genus | Ruminiclostridium9 | rs58925169 | A | G | -0.061 | 0.012 | 8.32E-07 | 24.407 |
| genus | Victivallis | rs4764863 | A | G | 0.122 | 0.025 | 8.22E-07 | 24.407 |
| genus | Bacteroides | rs66474973 | T | G | 0.081 | 0.016 | 6.81E-07 | 24.403 |
| genus | Odoribacter | rs9712594 | A | C | -0.097 | 0.020 | 5.31E-07 | 24.401 |
| genus | Parabacteroides | rs60884758 | T | C | -0.070 | 0.014 | 5.71E-07 | 24.401 |
| genus | Ruminiclostridium9 | rs4680432 | C | T | -0.061 | 0.012 | 9.29E-07 | 24.391 |
| genus | Ruminiclostridium6 | rs61060922 | G | T | 0.159 | 0.032 | 1.09E-06 | 24.380 |
| genus | Ruminiclostridium9 | . | C | A | -0.081 | 0.016 | 7.07E-07 | 24.373 |
| genus | Coprococcus3 | rs178271 | C | T | 0.145 | 0.029 | 7.81E-07 | 24.371 |
| genus | Adlercreutzia | rs7680684 | T | C | -0.083 | 0.017 | 9.77E-07 | 24.371 |
| genus | Ruminiclostridium9 | rs11717923 | G | A | -0.061 | 0.012 | 8.43E-07 | 24.369 |
| genus | DefluviitaleaceaeUCG011 | rs2357084 | A | T | -0.108 | 0.022 | 2.16E-06 | 24.361 |
| genus | Allisonella | rs35778461 | T | C | 0.147 | 0.030 | 1.21E-06 | 24.360 |
| genus | Bifidobacterium | rs1348790 | C | G | 0.059 | 0.012 | 5.57E-07 | 24.350 |
| genus |  | rs62349877 | C | T | -0.114 | 0.023 | 7.62E-07 | 24.348 |
| genus | Oscillospira | rs73038677 | A | T | -0.083 | 0.017 | 1.09E-06 | 24.339 |
| genus | Romboutsia | rs11594134 | T | C | 0.085 | 0.017 | 6.27E-07 | 24.339 |
| genus | Sellimonas | rs13417181 | C | T | 0.167 | 0.034 | 7.62E-07 | 24.337 |
| genus | Bacteroides | rs750423 | G | A | 0.052 | 0.011 | 8.89E-07 | 24.336 |
| genus | FamilyXIIIAD3011group | rs3828024 | A | G | -0.060 | 0.012 | 8.55E-07 | 24.332 |
| genus | Bifidobacterium | rs75667274 | C | T | 0.081 | 0.016 | 1.11E-06 | 24.332 |
| genus | Butyrivibrio | rs12211763 | G | A | -0.118 | 0.024 | 9.24E-07 | 24.330 |
| genus | LachnospiraceaeUCG008 | rs2186956 | C | G | 0.088 | 0.018 | 1.01E-06 | 24.329 |
| genus | RuminococcaceaeUCG004 | rs11164699 | A | G | 0.127 | 0.026 | 8.91E-07 | 24.328 |
| genus | LachnospiraceaeUCG010 | rs72825440 | G | A | 0.120 | 0.024 | 1.83E-06 | 24.327 |
| genus | Oxalobacter | rs36057338 | T | G | 0.208 | 0.042 | 8.80E-07 | 24.323 |
| genus | Oscillospira | rs12206468 | A | G | -0.133 | 0.027 | 1.04E-06 | 24.319 |
| genus | Ruminiclostridium9 | rs12636733 | C | T | -0.061 | 0.012 | 8.47E-07 | 24.316 |
| genus |  | rs9870933 | G | A | 0.062 | 0.013 | 8.49E-07 | 24.313 |
| genus | Ruminiclostridium9 | rs9681202 | A | G | -0.057 | 0.012 | 8.34E-07 | 24.312 |
| genus | RuminococcaceaeNK4A214group | rs136761 | A | G | -0.059 | 0.012 | 8.15E-07 | 24.312 |
| genus | ErysipelotrichaceaeUCG003 | rs2302840 | C | T | -0.087 | 0.018 | 1.29E-06 | 24.305 |
| genus |  | rs11007308 | T | C | -0.067 | 0.014 | 4.98E-07 | 24.298 |
| genus | Ruminiclostridium9 | rs10936131 | C | T | -0.061 | 0.012 | 8.71E-07 | 24.297 |
| genus | Oscillibacter | rs9393920 | G | A | -0.074 | 0.015 | 9.92E-07 | 24.294 |
| genus | Peptococcus | rs1441208 | C | A | -0.111 | 0.023 | 1.17E-06 | 24.291 |
| genus | Intestinimonas | rs716604 | G | A | 0.082 | 0.017 | 8.57E-07 | 24.289 |
| genus | Ruminiclostridium9 | rs56123505 | C | A | -0.061 | 0.012 | 9.53E-07 | 24.289 |
| genus | Anaerotruncus | rs7324216 | C | T | 0.087 | 0.018 | 2.85E-07 | 24.287 |
| genus | Bifidobacterium | rs4954193 | A | G | 0.059 | 0.012 | 5.68E-07 | 24.287 |
| genus | Bifidobacterium | rs7589297 | C | A | 0.059 | 0.012 | 6.14E-07 | 24.285 |
| genus | Streptococcus | rs79222628 | G | A | -0.092 | 0.019 | 8.01E-07 | 24.282 |
| genus | Bifidobacterium | rs6733547 | G | T | 0.083 | 0.017 | 5.10E-07 | 24.277 |
| genus | Akkermansia | rs113634144 | G | T | 0.074 | 0.015 | 1.02E-06 | 24.266 |
| genus |  | rs58877550 | G | T | -0.092 | 0.019 | 5.90E-07 | 24.265 |
| genus | Intestinimonas | rs11258178 | G | A | 0.066 | 0.013 | 6.98E-07 | 24.264 |
| genus | Anaerotruncus | rs6563551 | T | G | 0.087 | 0.018 | 2.72E-07 | 24.259 |
| genus | Bifidobacterium | rs7556781 | A | G | 0.071 | 0.014 | 1.98E-06 | 24.253 |
| genus |  | rs71599205 | T | C | -0.114 | 0.023 | 8.23E-07 | 24.251 |
| genus | Intestinimonas | rs12226153 | G | A | -0.151 | 0.031 | 5.12E-07 | 24.250 |
| genus | Parasutterella | rs78383039 | C | T | -0.146 | 0.030 | 1.57E-06 | 24.250 |
| genus | LachnospiraceaeUCG001 | rs6769749 | G | A | 0.090 | 0.018 | 1.18E-06 | 24.245 |
| genus | Bifidobacterium | rs10176573 | C | A | 0.059 | 0.012 | 6.24E-07 | 24.243 |
| genus | Ruminiclostridium9 | rs7641036 | T | C | -0.061 | 0.012 | 1.02E-06 | 24.242 |
| genus | Butyricimonas | rs2205439 | A | G | -0.132 | 0.027 | 1.58E-06 | 24.240 |
| genus | Fusicatenibacter | rs4378146 | C | A | -0.062 | 0.013 | 7.20E-07 | 24.239 |
| genus | Peptococcus | rs1978933 | C | T | -0.111 | 0.023 | 1.21E-06 | 24.237 |
| genus | Prevotella9 | rs111509883 | C | T | 0.171 | 0.035 | 1.24E-06 | 24.235 |
| genus | Phascolarctobacterium | rs56157888 | C | A | 0.095 | 0.019 | 1.09E-06 | 24.232 |
| genus |  | rs139749 | T | C | -0.085 | 0.017 | 8.59E-07 | 24.219 |
| genus | Enterorhabdus | rs10764181 | A | C | 0.083 | 0.017 | 1.21E-06 | 24.211 |
| genus | Romboutsia | rs11591519 | G | A | 0.085 | 0.017 | 6.85E-07 | 24.209 |
| genus | Peptococcus | rs1348140 | A | G | -0.111 | 0.023 | 1.22E-06 | 24.208 |
| genus | Anaerotruncus | rs73180681 | C | T | 0.087 | 0.018 | 2.48E-07 | 24.204 |
| genus | Prevotella7 | rs57404562 | A | C | 0.155 | 0.032 | 6.22E-07 | 24.202 |
| genus | Butyrivibrio | rs6568680 | T | C | -0.118 | 0.024 | 1.00E-06 | 24.201 |
| genus | Ruminiclostridium9 | rs12634807 | A | G | -0.061 | 0.012 | 9.30E-07 | 24.196 |
| genus | Ruminococcus2 | rs78120384 | G | A | -0.193 | 0.039 | 3.31E-07 | 24.189 |
| genus | Intestinimonas | rs1870850 | A | G | 0.082 | 0.017 | 8.96E-07 | 24.186 |
| genus | Lachnoclostridium | rs4813351 | C | T | -0.054 | 0.011 | 9.84E-07 | 24.183 |
| genus | Bifidobacterium | rs1446525 | G | A | 0.059 | 0.012 | 6.24E-07 | 24.183 |
| genus | ErysipelotrichaceaeUCG003 | rs28568391 | G | A | -0.058 | 0.012 | 6.42E-07 | 24.182 |
| genus | Barnesiella | rs2428166 | A | G | -0.166 | 0.034 | 8.51E-07 | 24.178 |
| genus | Howardella | rs1484873 | G | A | -0.228 | 0.046 | 2.56E-06 | 24.177 |
| genus | Bifidobacterium | rs625118 | C | A | 0.086 | 0.018 | 1.09E-06 | 24.174 |
| genus | Bifidobacterium | rs479980 | A | C | -0.060 | 0.012 | 8.05E-07 | 24.167 |
| genus | Bifidobacterium | rs10174462 | C | G | 0.073 | 0.015 | 2.09E-06 | 24.162 |
| genus | Peptococcus | rs1978932 | G | T | -0.111 | 0.023 | 1.25E-06 | 24.161 |
| genus | Intestinimonas | rs716605 | A | G | 0.082 | 0.017 | 8.75E-07 | 24.160 |
| genus | Lachnoclostridium | rs6112307 | C | T | -0.055 | 0.011 | 9.97E-07 | 24.156 |
| genus | Odoribacter | rs116516801 | T | C | -0.132 | 0.027 | 8.83E-07 | 24.154 |
| genus | RuminococcaceaeUCG002 | rs67746927 | G | C | -0.054 | 0.011 | 9.17E-07 | 24.154 |
| genus | Bifidobacterium | rs10928522 | C | A | 0.059 | 0.012 | 5.82E-07 | 24.153 |
| genus | Phascolarctobacterium | rs73094691 | C | G | 0.119 | 0.024 | 8.40E-07 | 24.149 |
| genus | Sellimonas | rs2016057 | C | A | -0.126 | 0.026 | 1.03E-06 | 24.149 |
| genus | Turicibacter | rs12603364 | C | T | 0.111 | 0.023 | 8.67E-07 | 24.148 |
| genus |  | rs12890135 | A | G | -0.165 | 0.034 | 1.24E-06 | 24.148 |
| genus | Allisonella | rs62559861 | G | C | 0.147 | 0.030 | 1.41E-06 | 24.148 |
| genus | Bifidobacterium | rs2289963 | G | A | 0.100 | 0.020 | 1.85E-06 | 24.145 |
| genus | RuminococcaceaeUCG004 | rs6445655 | T | A | 0.078 | 0.016 | 1.05E-06 | 24.143 |
| genus | Intestinimonas | rs1037944 | G | A | 0.082 | 0.017 | 8.57E-07 | 24.142 |
| genus | Ruminiclostridium9 | rs73170326 | G | A | -0.061 | 0.012 | 9.52E-07 | 24.137 |
| genus | RuminococcaceaeUCG003 | rs2452170 | G | A | 0.058 | 0.012 | 1.01E-06 | 24.134 |
| genus | Bifidobacterium | rs520449 | T | C | -0.061 | 0.012 | 4.54E-07 | 24.129 |
| genus |  | rs2047241 | A | G | -0.066 | 0.013 | 5.64E-07 | 24.128 |
| genus | Bifidobacterium | rs6706537 | T | C | 0.059 | 0.012 | 5.98E-07 | 24.124 |
| genus | LachnospiraceaeUCG008 | rs9877253 | T | G | -0.109 | 0.022 | 8.38E-07 | 24.123 |
| genus | Ruminiclostridium5 | rs79968837 | G | A | -0.095 | 0.019 | 1.15E-06 | 24.118 |
| genus | Intestinimonas | rs1350892 | G | C | 0.082 | 0.017 | 8.60E-07 | 24.117 |
| genus | Erysipelatoclostridium | rs9799756 | T | G | -0.080 | 0.016 | 9.80E-07 | 24.117 |
| genus | Oscillibacter | rs234108 | G | A | 0.075 | 0.015 | 9.16E-07 | 24.116 |
| genus | Oxalobacter | rs6071435 | A | T | -0.106 | 0.021 | 1.07E-06 | 24.109 |
| genus | Lachnoclostridium | rs6081565 | G | A | -0.055 | 0.011 | 1.09E-06 | 24.105 |
| genus | LachnospiraceaeUCG008 | rs9814084 | A | G | -0.115 | 0.023 | 9.90E-07 | 24.102 |
| genus | Butyrivibrio | rs6913769 | A | G | -0.118 | 0.024 | 1.06E-06 | 24.098 |
| genus | Ruminiclostridium9 | rs1918000 | A | G | -0.061 | 0.012 | 9.53E-07 | 24.092 |
| genus | Bifidobacterium | rs7560535 | G | A | 0.091 | 0.019 | 1.76E-06 | 24.092 |
| genus | LachnospiraceaeUCG008 | rs955844 | C | A | 0.112 | 0.023 | 1.81E-06 | 24.080 |
| genus | ChristensenellaceaeR | rs8110909 | A | G | 0.058 | 0.012 | 8.75E-07 | 24.080 |
| genus | ChristensenellaceaeR | rs6510389 | C | A | 0.057 | 0.012 | 1.04E-06 | 24.075 |
| genus | Ruminiclostridium9 | rs10936132 | C | T | -0.060 | 0.012 | 9.96E-07 | 24.075 |
| genus |  | rs3771393 | T | C | 0.131 | 0.027 | 7.38E-07 | 24.074 |
| genus | Bifidobacterium | rs62168871 | A | G | 0.059 | 0.012 | 6.42E-07 | 24.073 |
| genus | Butyricimonas | rs2642760 | C | G | 0.071 | 0.015 | 8.58E-07 | 24.072 |
| genus | Streptococcus | rs11110265 | G | A | -0.086 | 0.018 | 9.75E-07 | 24.070 |
| genus |  | rs10950610 | C | G | -0.081 | 0.016 | 5.84E-07 | 24.069 |
| genus | RuminococcaceaeUCG014 | . | A | G | 0.131 | 0.027 | 1.24E-06 | 24.069 |
| genus |  | rs12666868 | T | C | -0.080 | 0.016 | 5.37E-07 | 24.065 |
| genus | Methanobrevibacter | rs73457410 | G | A | 0.218 | 0.044 | 1.47E-06 | 24.064 |
| genus | RuminococcaceaeUCG004 | rs9880873 | G | T | 0.078 | 0.016 | 1.10E-06 | 24.062 |
| genus | Tyzzerella3 | rs4147931 | G | C | 0.098 | 0.020 | 1.19E-06 | 24.061 |
| genus | Methanobrevibacter | rs76029318 | C | T | 0.223 | 0.045 | 1.08E-06 | 24.060 |
| genus | Bifidobacterium | rs12474975 | A | T | 0.080 | 0.016 | 1.15E-06 | 24.060 |
| genus |  | rs17169426 | A | G | -0.081 | 0.016 | 5.94E-07 | 24.055 |
| genus | Oscillibacter | rs9501180 | C | T | -0.074 | 0.015 | 1.13E-06 | 24.054 |
| genus | Oxalobacter | rs4364195 | A | G | 0.121 | 0.025 | 9.31E-07 | 24.054 |
| genus | Anaerotruncus | rs7337526 | C | T | 0.086 | 0.018 | 3.02E-07 | 24.052 |
| genus | Ruminiclostridium9 | rs11712563 | G | A | -0.061 | 0.012 | 8.73E-07 | 24.048 |
| genus |  | rs139741 | G | A | -0.084 | 0.017 | 9.30E-07 | 24.048 |
| genus | Intestinibacter | rs9999821 | C | G | 0.070 | 0.014 | 1.07E-06 | 24.036 |
| genus |  | rs4852730 | C | T | 0.131 | 0.027 | 7.46E-07 | 24.035 |
| genus | Peptococcus | rs3781148 | C | T | -0.198 | 0.040 | 2.75E-06 | 24.032 |
| genus | RuminococcaceaeUCG004 | rs10874693 | G | A | 0.125 | 0.025 | 9.67E-07 | 24.028 |
| genus | Bilophila | rs116261629 | C | G | 0.128 | 0.026 | 8.62E-07 | 24.028 |
| genus | Bifidobacterium | rs4954197 | G | A | 0.059 | 0.012 | 7.07E-07 | 24.018 |
| genus | Lachnoclostridium | rs6081568 | C | G | -0.055 | 0.011 | 9.67E-07 | 24.015 |
| genus |  | rs6595327 | C | A | 0.096 | 0.020 | 6.92E-07 | 24.014 |
| genus | LachnospiraceaeUCG001 | rs770061 | A | G | 0.092 | 0.019 | 1.57E-06 | 24.013 |
| genus | Oxalobacter | rs4605578 | A | G | 0.121 | 0.025 | 9.53E-07 | 24.010 |
| genus | Streptococcus | rs7970304 | T | G | -0.090 | 0.018 | 9.95E-07 | 24.009 |
| genus | Olsenella | rs62112538 | T | C | -0.199 | 0.041 | 1.19E-06 | 24.006 |
| genus |  | rs12531973 | A | G | -0.080 | 0.016 | 5.27E-07 | 24.005 |
| genus | Victivallis | rs7314815 | A | G | 0.121 | 0.025 | 8.54E-07 | 24.004 |
| genus | Bifidobacterium | rs766270 | G | C | 0.059 | 0.012 | 7.40E-07 | 23.996 |
| genus | RuminococcaceaeUCG005 | rs60317497 | G | T | 0.157 | 0.032 | 1.17E-06 | 23.995 |
| genus | Coprococcus2 | rs6677933 | T | C | -0.080 | 0.016 | 1.19E-06 | 23.995 |
| genus | Oscillibacter | rs133832 | C | A | -0.080 | 0.016 | 1.15E-06 | 23.993 |
| genus | Bifidobacterium | rs309152 | T | C | 0.078 | 0.016 | 1.01E-06 | 23.990 |
| genus |  | rs139747 | A | G | -0.084 | 0.017 | 9.70E-07 | 23.990 |
| genus | Bifidobacterium | rs6430554 | C | T | 0.059 | 0.012 | 6.89E-07 | 23.989 |
| genus | ChristensenellaceaeR | rs10423276 | C | T | 0.057 | 0.012 | 1.02E-06 | 23.982 |
| genus | LachnospiraceaeUCG008 | rs1487192 | G | A | -0.096 | 0.020 | 1.21E-06 | 23.982 |
| genus | Bifidobacterium | rs6430551 | T | C | 0.059 | 0.012 | 6.04E-07 | 23.982 |
| genus | Peptococcus | rs75330476 | C | A | 0.158 | 0.032 | 9.89E-07 | 23.973 |
| genus | Butyrivibrio | rs10872067 | C | T | -0.117 | 0.024 | 1.11E-06 | 23.970 |
| genus | Roseburia | rs2160994 | C | T | 0.055 | 0.011 | 9.70E-07 | 23.969 |
| genus | RuminococcaceaeUCG003 | rs10181077 | C | T | 0.098 | 0.020 | 1.98E-06 | 23.964 |
| genus |  | rs1472354 | A | G | -0.112 | 0.023 | 1.38E-06 | 23.959 |
| genus | RuminococcaceaeUCG004 | rs11164698 | G | A | 0.126 | 0.026 | 1.10E-06 | 23.958 |
| genus | Intestinimonas | rs28373400 | C | T | 0.081 | 0.017 | 9.94E-07 | 23.956 |
| genus | Lachnoclostridium | rs2143856 | T | C | -0.053 | 0.011 | 1.20E-06 | 23.955 |
| genus |  | rs1231281 | G | A | -0.053 | 0.011 | 9.05E-07 | 23.950 |
| genus | Ruminiclostridium9 | rs9845388 | C | T | -0.057 | 0.012 | 1.11E-06 | 23.943 |
| genus | Fusicatenibacter | rs4425931 | G | T | -0.061 | 0.013 | 8.72E-07 | 23.940 |
| genus | Fusicatenibacter | rs704418 | C | T | 0.074 | 0.015 | 7.77E-07 | 23.937 |
| genus | Holdemania | rs11024781 | A | T | 0.078 | 0.016 | 1.27E-06 | 23.934 |
| genus | FamilyXIIIAD3011group | rs9876298 | C | T | 0.063 | 0.013 | 1.13E-06 | 23.929 |
| genus | RuminococcaceaeUCG003 | rs676388 | T | C | 0.058 | 0.012 | 1.09E-06 | 23.923 |
| genus | Victivallis | rs12575328 | G | A | -0.154 | 0.032 | 1.24E-06 | 23.921 |
| genus |  | rs973579 | A | G | -0.053 | 0.011 | 8.91E-07 | 23.920 |
| genus | Intestinimonas | rs13398390 | C | G | 0.081 | 0.017 | 9.53E-07 | 23.920 |
| genus | Ruminiclostridium9 | rs11719153 | G | A | -0.061 | 0.012 | 9.93E-07 | 23.920 |
| genus | Bifidobacterium | rs309158 | A | G | 0.075 | 0.015 | 1.35E-06 | 23.920 |
| genus | Oscillibacter | rs380914 | C | T | -0.074 | 0.015 | 1.17E-06 | 23.919 |
| genus | LachnospiraceaeUCG008 | rs2158198 | G | A | -0.083 | 0.017 | 1.08E-06 | 23.917 |
| genus |  | rs4615457 | A | G | -0.079 | 0.016 | 5.48E-07 | 23.916 |
| genus | Streptococcus | rs11110266 | C | T | -0.091 | 0.019 | 1.58E-06 | 23.916 |
| genus | Anaerotruncus | rs2323487 | A | T | 0.086 | 0.018 | 3.57E-07 | 23.911 |
| genus | Bifidobacterium | rs2166480 | G | A | 0.059 | 0.012 | 7.28E-07 | 23.905 |
| genus | Prevotella7 | rs73915877 | T | G | 0.155 | 0.032 | 7.38E-07 | 23.902 |
| genus | Faecalibacterium | rs75499067 | T | C | 0.228 | 0.047 | 1.76E-06 | 23.900 |
| genus | DefluviitaleaceaeUCG011 | rs112893842 | C | T | 0.114 | 0.023 | 1.45E-06 | 23.899 |
| genus |  | rs57199565 | C | T | 0.078 | 0.016 | 7.97E-07 | 23.896 |
| genus | FamilyXIIIAD3011group | rs62200412 | T | C | -0.080 | 0.016 | 5.80E-07 | 23.894 |
| genus | Bifidobacterium | rs57243841 | A | G | 0.068 | 0.014 | 1.34E-06 | 23.894 |
| genus | RuminococcaceaeUCG004 | rs9880860 | G | T | 0.077 | 0.016 | 1.20E-06 | 23.893 |
| genus | Peptococcus | rs13143446 | T | G | -0.111 | 0.023 | 1.25E-06 | 23.893 |
| genus | Intestinimonas | rs59872056 | G | A | 0.081 | 0.017 | 9.66E-07 | 23.886 |
| genus | RuminococcaceaeUCG004 | rs12034204 | T | A | 0.126 | 0.026 | 1.26E-06 | 23.884 |
| genus |  | rs1704773 | A | G | -0.052 | 0.011 | 1.03E-06 | 23.884 |
| genus | Haemophilus | rs78909003 | C | T | -0.246 | 0.050 | 1.67E-06 | 23.880 |
| genus | Flavonifractor | rs806808 | C | T | 0.067 | 0.014 | 1.18E-06 | 23.877 |
| genus | Sellimonas | rs10804205 | C | T | 0.123 | 0.025 | 1.20E-06 | 23.876 |
| genus | Coprobacter | rs305411 | G | A | 0.129 | 0.026 | 1.01E-06 | 23.864 |
| genus | Intestinimonas | rs1350893 | G | A | 0.081 | 0.017 | 9.73E-07 | 23.863 |
| genus |  | rs12666346 | T | C | -0.079 | 0.016 | 5.62E-07 | 23.862 |
| genus |  | rs178289 | G | C | 0.057 | 0.012 | 1.00E-06 | 23.858 |
| genus | RuminococcaceaeUCG004 | rs7426795 | C | T | 0.077 | 0.016 | 1.21E-06 | 23.853 |
| genus | Bifidobacterium | rs62168872 | C | T | 0.058 | 0.012 | 7.69E-07 | 23.852 |
| genus | Ruminiclostridium9 | rs73172114 | A | G | -0.064 | 0.013 | 1.21E-06 | 23.848 |
| genus | RuminococcaceaeUCG004 | rs7373223 | T | G | 0.077 | 0.016 | 1.24E-06 | 23.845 |
| genus | Holdemania | rs150096134 | A | T | 0.162 | 0.033 | 2.38E-06 | 23.838 |
| genus | Alloprevotella | rs12675570 | T | C | 0.155 | 0.032 | 3.02E-06 | 23.838 |
| genus | RuminococcaceaeUCG005 | rs34781347 | A | G | 0.189 | 0.039 | 6.05E-07 | 23.835 |
| genus | RuminococcaceaeUCG003 | rs1917270 | C | T | -0.153 | 0.031 | 2.83E-06 | 23.832 |
| genus | LachnospiraceaeUCG001 | rs2050911 | A | G | 0.075 | 0.015 | 1.11E-06 | 23.831 |
| genus | LachnospiraceaeUCG001 | rs6780017 | C | A | 0.091 | 0.019 | 1.58E-06 | 23.828 |
| genus | Bifidobacterium | rs112396243 | C | T | 0.080 | 0.016 | 1.45E-06 | 23.820 |
| genus | LachnospiraceaeUCG001 | rs1851551 | C | T | 0.091 | 0.019 | 1.62E-06 | 23.818 |
| genus | RuminococcaceaeUCG004 | rs1992962 | C | T | 0.077 | 0.016 | 1.24E-06 | 23.810 |
| genus | Methanobrevibacter | rs73457427 | G | A | 0.221 | 0.045 | 1.22E-06 | 23.807 |
| genus |  | rs77910827 | T | C | 0.202 | 0.041 | 9.05E-07 | 23.807 |
| genus |  | rs36058922 | T | A | -0.112 | 0.023 | 1.58E-06 | 23.804 |
| genus | Streptococcus | rs11110084 | G | A | -0.091 | 0.019 | 1.07E-06 | 23.794 |
| genus | Allisonella | rs10521029 | T | G | 0.146 | 0.030 | 1.59E-06 | 23.788 |
| genus | Anaerotruncus | rs1107244 | A | G | 0.086 | 0.018 | 3.66E-07 | 23.786 |
| genus | LachnospiraceaeUCG004 | rs2882478 | A | G | -0.058 | 0.012 | 1.21E-06 | 23.781 |
| genus | Bifidobacterium | rs111682961 | C | T | 0.080 | 0.016 | 1.54E-06 | 23.781 |
| genus | Bifidobacterium | rs16832243 | C | T | 0.080 | 0.016 | 1.40E-06 | 23.770 |
| genus | Intestinimonas | rs67857937 | C | A | 0.081 | 0.017 | 1.09E-06 | 23.767 |
| genus | Intestinimonas | rs11690270 | G | C | 0.081 | 0.017 | 1.02E-06 | 23.766 |
| genus | Dorea | rs78772518 | C | A | 0.138 | 0.028 | 3.38E-06 | 23.766 |
| genus | Blautia | rs67794373 | T | C | 0.060 | 0.012 | 1.00E-06 | 23.760 |
| genus |  | rs4255714 | T | A | -0.164 | 0.034 | 1.39E-06 | 23.750 |
| genus | Romboutsia | rs10279978 | G | A | -0.062 | 0.013 | 1.17E-06 | 23.749 |
| genus | Marvinbryantia | rs41336744 | G | A | -0.076 | 0.016 | 1.32E-06 | 23.749 |
| genus |  | rs112375806 | A | T | 0.143 | 0.029 | 5.82E-06 | 23.744 |
| genus | Intestinimonas | rs1381516 | T | A | 0.081 | 0.017 | 1.03E-06 | 23.742 |
| genus | RuminococcaceaeUCG004 | rs9817603 | T | C | 0.077 | 0.016 | 1.27E-06 | 23.741 |
| genus | Anaerotruncus | rs17055871 | T | A | 0.086 | 0.018 | 3.47E-07 | 23.741 |
| genus | Peptococcus | rs6531351 | A | G | -0.110 | 0.023 | 1.40E-06 | 23.738 |
| genus | RuminococcaceaeUCG002 | rs7155595 | A | C | 0.057 | 0.012 | 1.15E-06 | 23.734 |
| genus | Ruminiclostridium9 | rs10936143 | T | C | -0.064 | 0.013 | 1.34E-06 | 23.733 |
| genus | RuminococcaceaeUCG014 | rs115777838 | C | T | -0.188 | 0.039 | 4.62E-07 | 23.731 |
| genus | Intestinimonas | rs1381515 | C | T | 0.081 | 0.017 | 1.04E-06 | 23.730 |
| genus | Butyricimonas | rs7083431 | C | A | 0.070 | 0.014 | 8.85E-07 | 23.725 |
| genus | RuminococcaceaeUCG003 | rs584768 | G | A | 0.058 | 0.012 | 1.24E-06 | 23.725 |
| genus |  | rs2548459 | T | C | -0.052 | 0.011 | 1.12E-06 | 23.723 |
| genus | Fusicatenibacter | rs4567235 | C | T | -0.061 | 0.013 | 9.91E-07 | 23.719 |
| genus | Ruminiclostridium6 | rs17666271 | G | A | 0.157 | 0.032 | 1.55E-06 | 23.718 |
| genus | Tyzzerella3 | rs17809157 | T | A | -0.164 | 0.034 | 1.54E-06 | 23.717 |
| genus | RuminococcaceaeNK4A214group | rs11704699 | T | G | -0.090 | 0.018 | 8.71E-07 | 23.714 |
| genus | Oxalobacter | rs4287929 | G | A | 0.120 | 0.025 | 1.10E-06 | 23.711 |
| genus |  | rs12434631 | G | A | 0.075 | 0.015 | 2.77E-06 | 23.711 |
| genus | Ruminiclostridium9 | rs12631704 | A | G | -0.063 | 0.013 | 1.29E-06 | 23.709 |
| genus |  | rs56327615 | C | G | -0.079 | 0.016 | 6.62E-07 | 23.705 |
| genus | Adlercreutzia | rs80078995 | T | A | -0.113 | 0.023 | 1.57E-06 | 23.704 |
| genus | Erysipelatoclostridium | rs438876 | G | C | 0.085 | 0.017 | 1.41E-06 | 23.704 |
| genus | Bifidobacterium | rs4954196 | A | G | 0.058 | 0.012 | 8.34E-07 | 23.703 |
| genus | RuminococcaceaeUCG014 | rs12638134 | G | T | 0.058 | 0.012 | 1.21E-06 | 23.702 |
| genus | Intestinimonas | rs62240188 | A | G | 0.130 | 0.027 | 2.20E-06 | 23.701 |
| genus | Paraprevotella | rs9900242 | G | A | -0.085 | 0.018 | 1.14E-06 | 23.699 |
| genus | Faecalibacterium | rs6910935 | G | A | 0.135 | 0.028 | 1.38E-06 | 23.699 |
| genus | Ruminiclostridium9 | rs7617666 | A | C | -0.056 | 0.012 | 1.18E-06 | 23.692 |
| genus | Tyzzerella3 | rs34606911 | T | C | 0.098 | 0.020 | 1.33E-06 | 23.692 |
| genus | Anaerotruncus | rs7984882 | C | T | 0.077 | 0.016 | 5.74E-07 | 23.686 |
| genus | Bifidobacterium | rs309118 | G | C | 0.075 | 0.015 | 1.63E-06 | 23.684 |
| genus | Bifidobacterium | rs1374292 | G | A | 0.058 | 0.012 | 7.72E-07 | 23.683 |
| genus | Lachnoclostridium | rs3827978 | C | T | -0.055 | 0.011 | 1.25E-06 | 23.682 |
| genus | Bilophila | rs7802841 | A | C | 0.067 | 0.014 | 1.77E-06 | 23.681 |
| genus | LachnospiraceaeUCG001 | rs7621964 | T | G | 0.091 | 0.019 | 1.69E-06 | 23.677 |
| genus |  | rs1472609 | C | T | -0.087 | 0.018 | 1.55E-06 | 23.675 |
| genus | Intestinimonas | rs28615759 | C | T | 0.081 | 0.017 | 1.16E-06 | 23.672 |
| genus | LachnospiraceaeUCG001 | rs2639374 | G | A | 0.091 | 0.019 | 1.72E-06 | 23.672 |
| genus |  | rs6890185 | C | T | 0.113 | 0.023 | 1.12E-06 | 23.669 |
| genus | Intestinimonas | rs62109975 | G | C | 0.081 | 0.017 | 1.04E-06 | 23.668 |
| genus |  | rs4001474 | T | G | -0.079 | 0.016 | 6.62E-07 | 23.663 |
| genus | RuminococcaceaeUCG013 | rs11251085 | G | A | -0.055 | 0.011 | 1.17E-06 | 23.662 |
| genus | Tyzzerella3 | rs8102732 | G | C | 0.098 | 0.020 | 1.26E-06 | 23.661 |
| genus |  | rs3771392 | A | G | 0.130 | 0.027 | 9.11E-07 | 23.657 |
| genus | Collinsella | rs9541268 | A | C | 0.096 | 0.020 | 8.79E-07 | 23.657 |
| genus | Hungatella | rs34471047 | G | A | -0.163 | 0.033 | 1.76E-06 | 23.651 |
| genus | Bifidobacterium | rs12469941 | C | T | 0.058 | 0.012 | 7.33E-07 | 23.651 |
| genus | Sellimonas | rs1505357 | C | T | 0.122 | 0.025 | 1.34E-06 | 23.643 |
| genus | RuminococcaceaeUCG004 | rs6804196 | G | A | 0.077 | 0.016 | 1.35E-06 | 23.641 |
| genus | Erysipelatoclostridium | rs393441 | A | T | 0.085 | 0.017 | 1.43E-06 | 23.639 |
| genus | Bifidobacterium | rs7575241 | C | A | 0.072 | 0.015 | 2.25E-06 | 23.635 |
| genus | ErysipelotrichaceaeUCG003 | rs10164067 | G | T | -0.103 | 0.021 | 1.13E-06 | 23.634 |
| genus |  | rs3771394 | C | T | 0.130 | 0.027 | 9.29E-07 | 23.629 |
| genus | Escherichia | rs2267739 | C | G | 0.116 | 0.024 | 1.42E-06 | 23.627 |
| genus | Intestinimonas | rs1037943 | A | G | 0.081 | 0.017 | 1.14E-06 | 23.619 |
| genus | Bifidobacterium | rs6430555 | A | C | 0.058 | 0.012 | 8.75E-07 | 23.618 |
| genus | Haemophilus | rs9328464 | C | T | 0.072 | 0.015 | 1.42E-06 | 23.615 |
| genus | LachnospiraceaeUCG001 | rs16810 | T | C | 0.068 | 0.014 | 1.25E-06 | 23.613 |
| genus | Anaerotruncus | rs3764077 | A | G | 0.086 | 0.018 | 3.62E-07 | 23.611 |
| genus | Peptococcus | rs10824475 | T | G | -0.190 | 0.039 | 3.49E-06 | 23.604 |
| genus | Intestinimonas | rs62109972 | T | C | 0.081 | 0.017 | 1.09E-06 | 23.603 |
| genus | RuminococcaceaeUCG004 | rs6777368 | C | G | 0.077 | 0.016 | 1.38E-06 | 23.601 |
| genus | RuminococcaceaeUCG011 | rs12524558 | A | C | -0.147 | 0.030 | 1.05E-06 | 23.600 |
| genus | Intestinimonas | rs11258177 | T | C | 0.065 | 0.013 | 1.14E-06 | 23.598 |
| genus | Intestinimonas | rs4287737 | G | C | 0.081 | 0.017 | 1.22E-06 | 23.597 |
| genus | Sellimonas | rs2554358 | C | T | -0.125 | 0.026 | 1.40E-06 | 23.596 |
| genus | LachnospiraceaeUCG008 | rs11600957 | T | C | -0.094 | 0.019 | 1.39E-06 | 23.595 |
| genus | Bifidobacterium | rs6711493 | A | G | 0.075 | 0.015 | 1.40E-06 | 23.594 |
| genus | Holdemania | rs150765194 | G | T | 0.161 | 0.033 | 2.76E-06 | 23.589 |
| genus | Streptococcus | rs11110070 | G | A | -0.088 | 0.018 | 1.12E-06 | 23.588 |
| genus | Odoribacter | rs115320778 | G | A | -0.130 | 0.027 | 1.21E-06 | 23.585 |
| genus | Bifidobacterium | rs6430550 | C | T | 0.058 | 0.012 | 7.13E-07 | 23.584 |
| genus | Lachnoclostridium | rs6035303 | G | A | -0.054 | 0.011 | 1.23E-06 | 23.584 |
| genus | Ruminiclostridium9 | rs11707696 | T | G | -0.056 | 0.012 | 1.23E-06 | 23.577 |
| genus | RuminococcaceaeUCG003 | rs4297864 | T | C | 0.096 | 0.020 | 1.98E-06 | 23.571 |
| genus | Intestinimonas | rs62109976 | G | A | 0.081 | 0.017 | 1.09E-06 | 23.567 |
| genus | RuminococcaceaeUCG011 | rs7746459 | T | A | -0.165 | 0.034 | 1.06E-06 | 23.565 |
| genus | Ruminococcus1 | rs7117576 | G | A | 0.083 | 0.017 | 6.48E-07 | 23.561 |
| genus | Tyzzerella3 | rs4807499 | C | T | 0.097 | 0.020 | 1.53E-06 | 23.556 |
| genus | Intestinimonas | rs72774198 | G | A | 0.081 | 0.017 | 1.14E-06 | 23.548 |
| genus | FamilyXIIIUCG001 | rs1426266 | C | T | -0.067 | 0.014 | 1.25E-06 | 23.547 |
| genus | Alloprevotella | rs6472439 | A | C | 0.141 | 0.029 | 2.14E-06 | 23.546 |
| genus | Hungatella | rs34051109 | G | A | -0.163 | 0.033 | 1.89E-06 | 23.546 |
| genus | Streptococcus | rs957755 | G | T | -0.070 | 0.014 | 1.37E-06 | 23.544 |
| genus | RuminococcaceaeUCG004 | rs9884005 | A | G | 0.077 | 0.016 | 1.43E-06 | 23.543 |
| genus | Allisonella | rs4743068 | C | T | 0.130 | 0.027 | 1.53E-06 | 23.542 |
| genus | Parabacteroides | rs4236095 | A | G | 0.076 | 0.016 | 1.93E-06 | 23.542 |
| genus | Intestinimonas | rs28704834 | T | C | 0.081 | 0.017 | 1.24E-06 | 23.537 |
| genus | RuminococcaceaeUCG011 | rs4330884 | A | C | -0.121 | 0.025 | 1.53E-06 | 23.536 |
| genus | Bifidobacterium | rs79826902 | G | A | 0.079 | 0.016 | 1.70E-06 | 23.531 |
| genus | Ruminiclostridium9 | rs6806967 | C | T | -0.063 | 0.013 | 1.47E-06 | 23.531 |
| genus |  | rs13176281 | C | T | -0.112 | 0.023 | 1.16E-06 | 23.527 |
| genus | RuminococcaceaeUCG011 | rs12724320 | T | C | -0.121 | 0.025 | 1.52E-06 | 23.524 |
| genus | Butyrivibrio | rs9689727 | C | T | -0.116 | 0.024 | 1.41E-06 | 23.522 |
| genus | RuminococcaceaeUCG003 | rs4386305 | T | G | 0.096 | 0.020 | 2.17E-06 | 23.520 |
| genus |  | rs838144 | T | C | -0.052 | 0.011 | 1.35E-06 | 23.518 |
| genus | Bifidobacterium | rs2197579 | C | T | 0.058 | 0.012 | 9.35E-07 | 23.501 |
| genus | Butyrivibrio | rs463854 | G | C | -0.116 | 0.024 | 1.42E-06 | 23.501 |
| genus | ChristensenellaceaeR | rs1812307 | G | A | 0.057 | 0.012 | 1.17E-06 | 23.498 |
| genus | Anaerotruncus | rs61946592 | T | C | 0.086 | 0.018 | 4.04E-07 | 23.498 |
| genus | Bifidobacterium | rs16832205 | G | A | 0.079 | 0.016 | 1.68E-06 | 23.497 |
| genus | Lactobacillus | rs921925 | C | A | 0.099 | 0.020 | 9.72E-07 | 23.495 |
| genus | Roseburia | rs9925892 | C | A | 0.060 | 0.012 | 1.74E-06 | 23.494 |
| genus | LachnospiraceaeUCG001 | rs2320002 | T | C | 0.090 | 0.019 | 1.91E-06 | 23.487 |
| genus | Sellimonas | rs2570230 | C | G | -0.124 | 0.026 | 1.46E-06 | 23.487 |
| genus | Bilophila | rs1571225 | T | C | 0.083 | 0.017 | 1.12E-06 | 23.483 |
| genus | Lachnoclostridium | rs6106063 | C | T | -0.053 | 0.011 | 1.26E-06 | 23.479 |
| genus | Bifidobacterium | rs60902646 | T | C | 0.095 | 0.020 | 3.34E-06 | 23.478 |
| genus | Allisonella | rs6478039 | C | T | 0.145 | 0.030 | 1.83E-06 | 23.475 |
| genus | Allisonella | rs6478038 | C | A | 0.145 | 0.030 | 1.83E-06 | 23.475 |
| genus | Hungatella | rs34900903 | C | T | -0.162 | 0.034 | 1.93E-06 | 23.473 |
| genus | LachnospiraceaeFCS020group | rs369444 | G | C | 0.125 | 0.026 | 3.15E-06 | 23.471 |
| genus | LachnospiraceaeND3007group | rs9932954 | G | A | -0.056 | 0.012 | 1.25E-06 | 23.467 |
| genus | Ruminiclostridium9 | rs11719388 | G | T | -0.056 | 0.012 | 1.30E-06 | 23.465 |
| genus | Paraprevotella | rs9602779 | C | A | -0.107 | 0.022 | 6.93E-07 | 23.463 |
| genus | Peptococcus | rs13121056 | C | G | -0.110 | 0.023 | 1.59E-06 | 23.463 |
| genus |  | rs6772337 | G | A | 0.061 | 0.013 | 1.31E-06 | 23.458 |
| genus | Odoribacter | rs17103646 | A | G | -0.130 | 0.027 | 1.29E-06 | 23.456 |
| genus | Anaerofilum | rs4506496 | A | G | 0.103 | 0.021 | 1.49E-06 | 23.455 |
| genus | RuminococcaceaeUCG004 | rs1449332 | T | C | 0.077 | 0.016 | 1.50E-06 | 23.453 |
| genus | LachnospiraceaeUCG001 | rs1608294 | C | T | 0.090 | 0.019 | 1.95E-06 | 23.452 |
| genus | Marvinbryantia | rs1187983 | T | C | -0.094 | 0.019 | 2.02E-06 | 23.450 |
| genus | Bifidobacterium | rs2117511 | T | C | 0.085 | 0.017 | 1.54E-06 | 23.449 |
| genus | Bifidobacterium | rs606101 | G | A | -0.060 | 0.012 | 1.29E-06 | 23.445 |
| genus | Ruminiclostridium5 | rs243585 | G | C | -0.059 | 0.012 | 1.33E-06 | 23.443 |
| genus | Bifidobacterium | rs77985886 | C | T | 0.098 | 0.020 | 2.68E-06 | 23.442 |
| genus | Odoribacter | rs17103637 | C | T | -0.130 | 0.027 | 1.25E-06 | 23.440 |
| genus | Ruminiclostridium9 | rs73172119 | G | A | -0.063 | 0.013 | 1.59E-06 | 23.439 |
| genus | RuminococcaceaeUCG003 | rs11243416 | C | T | -0.093 | 0.019 | 1.67E-06 | 23.437 |
| genus | Prevotella7 | rs55643286 | A | G | 0.154 | 0.032 | 8.96E-07 | 23.432 |
| genus | LachnospiraceaeUCG008 | rs10734276 | T | C | -0.095 | 0.020 | 1.49E-06 | 23.430 |
| genus | Parasutterella | rs10899911 | G | A | -0.072 | 0.015 | 1.15E-06 | 23.429 |
| genus | Streptococcus | rs11769597 | T | C | -0.070 | 0.014 | 1.39E-06 | 23.425 |
| genus |  | rs34860830 | G | A | -0.166 | 0.034 | 1.89E-06 | 23.425 |
| genus | Streptococcus | rs11764382 | G | A | -0.070 | 0.014 | 1.29E-06 | 23.424 |
| genus | Haemophilus | rs76022354 | T | C | 0.245 | 0.051 | 1.83E-06 | 23.421 |
| genus | CandidatusSoleaferrea | rs61825792 | C | T | 0.112 | 0.023 | 1.37E-06 | 23.418 |
| genus | CandidatusSoleaferrea | rs4294381 | C | T | 0.112 | 0.023 | 1.37E-06 | 23.416 |
| genus | RuminococcaceaeUCG003 | rs569970 | T | C | 0.057 | 0.012 | 1.42E-06 | 23.414 |
| genus | Methanobrevibacter | rs11147839 | G | A | 0.219 | 0.045 | 1.62E-06 | 23.414 |
| genus | Intestinimonas | rs60282801 | T | C | 0.080 | 0.017 | 1.30E-06 | 23.413 |
| genus | Bifidobacterium | rs6743537 | G | A | 0.078 | 0.016 | 1.86E-06 | 23.412 |
| genus | Butyrivibrio | rs464401 | A | G | -0.116 | 0.024 | 1.50E-06 | 23.409 |
| genus | Lachnoclostridium | rs6112308 | C | T | -0.054 | 0.011 | 1.56E-06 | 23.407 |
| genus | Dorea | rs4831772 | G | A | -0.094 | 0.019 | 1.64E-06 | 23.404 |
| genus | ChristensenellaceaeR | rs9304850 | C | G | 0.056 | 0.012 | 1.37E-06 | 23.404 |
| genus | Ruminococcus1 | rs7101995 | C | T | 0.054 | 0.011 | 1.54E-06 | 23.403 |
| genus |  | . | T | C | -0.053 | 0.011 | 1.27E-06 | 23.402 |
| genus | Bifidobacterium | rs7592990 | A | G | 0.085 | 0.018 | 1.61E-06 | 23.398 |
| genus | Ruminiclostridium9 | rs115044523 | A | G | -0.098 | 0.020 | 2.37E-06 | 23.395 |
| genus |  | rs73684130 | G | A | -0.080 | 0.016 | 8.61E-07 | 23.389 |
| genus | RuminococcaceaeUCG004 | rs35483270 | C | T | 0.124 | 0.026 | 1.36E-06 | 23.383 |
| genus | Intestinimonas | rs1381514 | C | T | 0.080 | 0.017 | 1.23E-06 | 23.377 |
| genus | LachnospiraceaeUCG001 | rs10493703 | A | T | 0.074 | 0.015 | 1.40E-06 | 23.377 |
| genus | Paraprevotella | rs9531809 | A | C | -0.083 | 0.017 | 1.62E-06 | 23.374 |
| genus | LachnospiraceaeUCG008 | rs7113653 | A | C | -0.094 | 0.019 | 1.41E-06 | 23.372 |
| genus | CandidatusSoleaferrea | rs4653589 | A | G | 0.110 | 0.023 | 1.66E-06 | 23.370 |
| genus | Holdemania | rs3799996 | T | C | 0.087 | 0.018 | 2.21E-06 | 23.369 |
| genus | Alistipes | rs11769002 | A | G | -0.053 | 0.011 | 1.45E-06 | 23.368 |
| genus | Lachnoclostridium | rs3950143 | C | T | -0.052 | 0.011 | 1.42E-06 | 23.368 |
| genus | Lachnoclostridium | rs6081576 | C | T | -0.054 | 0.011 | 1.38E-06 | 23.366 |
| genus | Bifidobacterium | rs6712127 | A | G | 0.071 | 0.015 | 3.11E-06 | 23.358 |
| genus | Intestinimonas | rs60896692 | T | C | 0.080 | 0.017 | 1.26E-06 | 23.355 |
| genus | RuminococcaceaeUCG004 | rs12123966 | A | G | 0.124 | 0.026 | 1.69E-06 | 23.353 |
| genus | Coprococcus1 | rs489284 | T | A | -0.053 | 0.011 | 1.51E-06 | 23.352 |
| genus | Bifidobacterium | rs2053727 | C | T | 0.058 | 0.012 | 9.90E-07 | 23.349 |
| genus | ErysipelotrichaceaeUCG003 | rs73437837 | G | A | -0.085 | 0.018 | 2.39E-06 | 23.347 |
| genus | Hungatella | rs56197253 | G | A | -0.162 | 0.033 | 2.07E-06 | 23.345 |
| genus | Hungatella | rs41530947 | C | T | -0.162 | 0.033 | 2.08E-06 | 23.344 |
| genus | Hungatella | rs34995648 | A | C | -0.162 | 0.034 | 2.17E-06 | 23.343 |
| genus | Sellimonas | rs62016462 | C | T | 0.234 | 0.048 | 1.09E-06 | 23.341 |
| genus | RuminococcaceaeUCG002 | rs10927423 | A | C | -0.071 | 0.015 | 8.50E-07 | 23.340 |
| genus |  | rs8103840 | T | C | -0.052 | 0.011 | 1.28E-06 | 23.340 |
| genus | Intestinimonas | rs62109974 | T | C | 0.080 | 0.017 | 1.23E-06 | 23.339 |
| genus | Lactobacillus | rs11674854 | T | C | -0.085 | 0.018 | 1.59E-06 | 23.338 |
| genus | Marvinbryantia | rs1543595 | T | C | -0.080 | 0.016 | 1.68E-06 | 23.337 |
| genus | Prevotella7 | rs9959718 | A | G | 0.133 | 0.028 | 1.90E-06 | 23.333 |
| genus | Anaerotruncus | rs7995332 | C | A | 0.085 | 0.018 | 4.55E-07 | 23.331 |
| genus |  | rs314726 | C | T | 0.053 | 0.011 | 1.38E-06 | 23.329 |
| genus | Alloprevotella | rs17695059 | G | C | 0.140 | 0.029 | 2.39E-06 | 23.328 |
| genus | Butyricimonas | rs11908936 | A | G | -0.126 | 0.026 | 2.09E-06 | 23.328 |
| genus |  | rs6438086 | G | A | 0.063 | 0.013 | 1.63E-06 | 23.324 |
| genus |  | rs139750 | G | A | -0.083 | 0.017 | 1.36E-06 | 23.323 |
| genus | Flavonifractor | rs4949654 | A | G | -0.066 | 0.014 | 1.52E-06 | 23.320 |
| genus | Tyzzerella3 | rs75091807 | T | G | -0.185 | 0.038 | 1.71E-06 | 23.320 |
| genus | LachnospiraceaeUCG001 | rs1342129 | A | G | 0.074 | 0.015 | 1.46E-06 | 23.315 |
| genus | Lactobacillus | rs7399658 | A | G | -0.107 | 0.022 | 3.12E-06 | 23.313 |
| genus | FamilyXIIIUCG001 | rs12049454 | C | T | -0.065 | 0.013 | 1.17E-06 | 23.312 |
| genus |  | rs12889944 | A | T | -0.165 | 0.034 | 1.77E-06 | 23.311 |
| genus | Dialister | rs7814375 | A | C | 0.064 | 0.013 | 1.13E-06 | 23.309 |
| genus | Butyrivibrio | rs463853 | G | C | -0.116 | 0.024 | 1.56E-06 | 23.308 |
| genus | Intestinibacter | rs6823063 | T | C | 0.069 | 0.014 | 1.60E-06 | 23.301 |
| genus | Allisonella | rs12115447 | A | G | 0.144 | 0.030 | 1.96E-06 | 23.301 |
| genus | Ruminiclostridium9 | rs55875763 | C | G | -0.055 | 0.011 | 1.60E-06 | 23.301 |
| genus | Ruminiclostridium5 | rs2286384 | C | G | -0.052 | 0.011 | 1.44E-06 | 23.300 |
| genus | Hungatella | rs35350084 | T | C | -0.162 | 0.033 | 2.13E-06 | 23.297 |
| genus | Intestinimonas | rs11689582 | A | T | 0.080 | 0.017 | 1.31E-06 | 23.294 |
| genus | LachnospiraceaeND3007group | rs761051 | C | T | -0.056 | 0.012 | 1.38E-06 | 23.293 |
| genus |  | rs35883366 | C | A | -0.165 | 0.034 | 2.12E-06 | 23.286 |
| genus | Gordonibacter | rs34967912 | G | A | -0.226 | 0.047 | 1.24E-06 | 23.285 |
| genus | Ruminococcus1 | rs1348964 | A | G | -0.056 | 0.012 | 1.61E-06 | 23.281 |
| genus | Phascolarctobacterium | rs56069061 | A | G | -0.111 | 0.023 | 1.87E-06 | 23.279 |
| genus | Lactococcus | rs10417872 | G | T | 0.118 | 0.025 | 1.29E-06 | 23.276 |
| genus | Holdemanella | rs73011279 | C | T | -0.096 | 0.020 | 1.36E-06 | 23.274 |
| genus | Bacteroides | rs195028 | A | G | 0.080 | 0.017 | 1.33E-06 | 23.274 |
| genus | RuminococcaceaeUCG004 | rs1337197 | G | A | 0.124 | 0.026 | 1.59E-06 | 23.272 |
| genus | Bifidobacterium | rs4954394 | A | G | 0.066 | 0.014 | 7.84E-07 | 23.272 |
| genus | Barnesiella | rs60316894 | T | C | -0.121 | 0.025 | 1.19E-06 | 23.267 |
| genus | Barnesiella | rs35177866 | G | A | 0.092 | 0.019 | 2.95E-06 | 23.267 |
| genus | Bifidobacterium | rs1960171 | C | T | 0.063 | 0.013 | 1.87E-06 | 23.267 |
| genus | Turicibacter | rs4869133 | A | G | 0.131 | 0.027 | 2.55E-06 | 23.267 |
| genus | Bifidobacterium | rs309161 | G | A | 0.076 | 0.016 | 1.58E-06 | 23.263 |
| genus | Intestinimonas | rs62109973 | T | C | 0.080 | 0.017 | 1.28E-06 | 23.263 |
| genus | Anaerofilum | rs1965460 | C | T | -0.099 | 0.021 | 1.06E-06 | 23.260 |
| genus | Butyrivibrio | rs463242 | A | G | -0.116 | 0.024 | 1.61E-06 | 23.259 |
| genus | Ruminiclostridium9 | rs918449 | G | A | -0.095 | 0.020 | 2.56E-06 | 23.258 |
| genus | Bifidobacterium | rs10841473 | C | G | -0.062 | 0.013 | 1.65E-06 | 23.256 |
| genus | LachnospiraceaeUCG001 | rs7514224 | A | C | 0.078 | 0.016 | 1.93E-06 | 23.254 |
| genus | LachnospiraceaeUCG008 | rs10766568 | C | G | -0.093 | 0.019 | 1.35E-06 | 23.252 |
| genus | ErysipelotrichaceaeUCG003 | rs57486902 | C | T | -0.092 | 0.019 | 3.04E-06 | 23.248 |
| genus | Hungatella | rs34409952 | G | A | -0.161 | 0.034 | 2.19E-06 | 23.236 |
| genus | Odoribacter | rs17103634 | G | T | -0.129 | 0.027 | 1.39E-06 | 23.235 |
| genus | Desulfovibrio | rs16863365 | G | A | 0.109 | 0.023 | 1.79E-06 | 23.235 |
| genus | Roseburia | NA | G | C | -0.232 | 0.048 | 1.48E-07 | 23.235 |
| genus | Hungatella | rs34045034 | A | G | -0.161 | 0.034 | 2.19E-06 | 23.234 |
| genus |  | rs10131724 | C | A | -0.200 | 0.041 | 2.39E-06 | 23.234 |
| genus | Hungatella | rs4691170 | G | A | -0.161 | 0.033 | 2.21E-06 | 23.233 |
| genus | Methanobrevibacter | rs9594530 | T | C | 0.219 | 0.045 | 1.68E-06 | 23.231 |
| genus | Ruminococcus1 | rs10769157 | T | A | -0.056 | 0.012 | 1.66E-06 | 23.228 |
| genus | RuminococcaceaeUCG004 | rs7630070 | G | A | 0.076 | 0.016 | 1.68E-06 | 23.226 |
| genus | Coprococcus2 | rs2426193 | G | C | 0.113 | 0.023 | 2.16E-06 | 23.224 |
| genus | Parasutterella | rs35414597 | A | T | -0.068 | 0.014 | 1.51E-06 | 23.220 |
| genus |  | rs73304492 | C | T | -0.078 | 0.016 | 8.48E-07 | 23.220 |
| genus | Fusicatenibacter | rs41268773 | G | C | -0.060 | 0.012 | 1.50E-06 | 23.218 |
| genus | RuminococcaceaeUCG002 | rs62374283 | C | T | -0.058 | 0.012 | 1.80E-06 | 23.215 |
| genus | Parasutterella | rs7303158 | T | C | 0.065 | 0.013 | 1.33E-06 | 23.214 |
| genus | Blautia | rs55798850 | A | G | 0.060 | 0.012 | 1.47E-06 | 23.214 |
| genus | Bifidobacterium | rs62168891 | C | T | 0.058 | 0.012 | 1.12E-06 | 23.212 |
| genus | LachnospiraceaeUCG008 | rs62277846 | T | C | 0.102 | 0.021 | 1.59E-06 | 23.212 |
| genus | Bifidobacterium | rs72847650 | C | A | -0.060 | 0.012 | 8.44E-07 | 23.212 |
| genus | Fusicatenibacter | rs62353480 | G | A | -0.070 | 0.015 | 1.57E-06 | 23.210 |
| genus | LachnospiraceaeUCG001 | rs4234364 | T | C | 0.088 | 0.018 | 2.00E-06 | 23.208 |
| genus | RuminococcaceaeUCG003 | rs2638282 | G | A | 0.057 | 0.012 | 1.69E-06 | 23.205 |
| genus | Bifidobacterium | rs621341 | C | T | -0.060 | 0.012 | 8.20E-07 | 23.203 |
| genus |  | rs13239072 | A | G | 0.069 | 0.014 | 1.82E-06 | 23.197 |
| genus | Intestinimonas | rs7581071 | G | C | 0.080 | 0.017 | 1.40E-06 | 23.197 |
| genus | LachnospiraceaeUCG001 | rs3920359 | C | A | 0.090 | 0.019 | 2.37E-06 | 23.196 |
| genus | Faecalibacterium | rs1271565 | T | C | -0.058 | 0.012 | 1.30E-06 | 23.195 |
| genus | Anaerotruncus | rs61946576 | T | C | 0.085 | 0.018 | 4.55E-07 | 23.194 |
| genus | RuminococcaceaeUCG011 | rs9377652 | G | T | -0.143 | 0.030 | 1.47E-06 | 23.193 |
| genus | LachnospiraceaeNK4A136group | rs10767692 | G | A | 0.056 | 0.012 | 1.72E-06 | 23.191 |
| genus | Flavonifractor | rs11811696 | C | T | -0.116 | 0.024 | 2.07E-06 | 23.190 |
| genus | Ruminiclostridium9 | rs73592673 | T | A | -0.082 | 0.017 | 2.14E-06 | 23.189 |
| genus |  | rs6036147 | A | G | -0.055 | 0.012 | 1.57E-06 | 23.187 |
| genus | Bifidobacterium | rs4954575 | G | A | 0.062 | 0.013 | 1.14E-06 | 23.183 |
| genus | Hungatella | rs35934050 | C | T | -0.161 | 0.034 | 2.32E-06 | 23.183 |
| genus | RuminococcaceaeUCG005 | rs149070170 | C | T | 0.154 | 0.032 | 1.73E-06 | 23.183 |
| genus | Holdemania | rs77293403 | G | A | 0.165 | 0.034 | 1.77E-06 | 23.182 |
| genus | Butyrivibrio | rs1623806 | C | T | -0.115 | 0.024 | 1.68E-06 | 23.182 |
| genus | Bifidobacterium | rs309165 | T | C | 0.074 | 0.015 | 1.88E-06 | 23.181 |
| genus |  | rs6884406 | G | A | 0.112 | 0.023 | 1.44E-06 | 23.179 |
| genus | Veillonella | rs12679709 | G | C | -0.079 | 0.016 | 1.78E-06 | 23.178 |
| genus | LachnospiraceaeUCG008 | rs10801803 | A | G | -0.117 | 0.024 | 1.40E-06 | 23.178 |
| genus | Fusicatenibacter | rs2132128 | A | G | -0.077 | 0.016 | 1.08E-06 | 23.176 |
| genus | Ruminiclostridium6 | rs12362316 | G | A | 0.074 | 0.015 | 1.52E-06 | 23.174 |
| genus | DefluviitaleaceaeUCG011 | rs55658617 | C | T | 0.174 | 0.036 | 2.15E-06 | 23.173 |
| genus | Butyrivibrio | rs11761679 | C | T | 0.155 | 0.032 | 2.20E-06 | 23.170 |
| genus | Dialister | rs6982344 | A | G | 0.064 | 0.013 | 1.21E-06 | 23.170 |
| genus | DefluviitaleaceaeUCG011 | rs1582238 | C | T | 0.081 | 0.017 | 1.57E-06 | 23.167 |
| genus |  | rs73684129 | G | A | -0.079 | 0.016 | 9.07E-07 | 23.162 |
| genus | Intestinimonas | rs11258179 | G | A | 0.065 | 0.013 | 1.43E-06 | 23.160 |
| genus | Paraprevotella | rs9547271 | T | C | -0.106 | 0.022 | 7.92E-07 | 23.159 |
| genus | ErysipelotrichaceaeUCG003 | rs2052371 | T | C | -0.085 | 0.018 | 2.41E-06 | 23.158 |
| genus | Victivallis | rs7957782 | G | A | 0.119 | 0.025 | 1.51E-06 | 23.157 |
| genus | Butyrivibrio | rs62492398 | G | T | 0.154 | 0.032 | 2.31E-06 | 23.156 |
| genus | Butyrivibrio | rs456569 | G | T | -0.115 | 0.024 | 1.71E-06 | 23.148 |
| genus | Bifidobacterium | rs309119 | T | C | 0.076 | 0.016 | 1.49E-06 | 23.147 |
| genus | Gordonibacter | rs77754772 | C | T | -0.226 | 0.047 | 1.32E-06 | 23.144 |
| genus | Intestinimonas | rs6716504 | T | C | 0.080 | 0.017 | 1.40E-06 | 23.144 |
| genus | Blautia | rs12453000 | T | C | 0.063 | 0.013 | 1.26E-06 | 23.143 |
| genus | Eisenbergiella | rs12278566 | A | T | -0.121 | 0.025 | 1.65E-06 | 23.143 |
| genus | Prevotella7 | rs59826552 | T | G | 0.152 | 0.032 | 9.13E-07 | 23.142 |
| genus | Howardella | rs17167098 | A | G | -0.169 | 0.035 | 1.12E-06 | 23.142 |
| genus | Gordonibacter | rs72714787 | A | C | 0.181 | 0.038 | 1.43E-06 | 23.141 |
| genus | Lactobacillus | rs328312 | A | T | 0.082 | 0.017 | 1.41E-06 | 23.141 |
| genus | Paraprevotella | rs1855665 | C | A | -0.084 | 0.018 | 1.77E-06 | 23.140 |
| genus | Allisonella | rs7872669 | C | G | 0.144 | 0.030 | 2.15E-06 | 23.136 |
| genus | RuminococcaceaeUCG004 | rs6445657 | G | A | 0.076 | 0.016 | 1.77E-06 | 23.136 |
| genus |  | rs692854 | C | A | -0.051 | 0.011 | 1.57E-06 | 23.135 |
| genus | Ruminiclostridium6 | rs77193512 | G | A | 0.074 | 0.015 | 1.30E-06 | 23.125 |
| genus | Sellimonas | rs6754674 | G | T | 0.121 | 0.025 | 1.88E-06 | 23.124 |
| genus | RuminococcaceaeUCG009 | rs4708333 | G | T | -0.084 | 0.017 | 1.56E-06 | 23.122 |
| genus | Bifidobacterium | rs1446523 | G | A | 0.058 | 0.012 | 9.99E-07 | 23.119 |
| genus | RuminococcaceaeUCG002 | rs12431415 | G | A | 0.056 | 0.012 | 1.63E-06 | 23.117 |
| genus |  | rs676388 | T | C | -0.051 | 0.011 | 1.59E-06 | 23.114 |
| genus |  | rs9872758 | C | T | 0.085 | 0.018 | 1.66E-06 | 23.113 |
| genus | LachnospiraceaeUCG008 | rs29362 | A | G | 0.085 | 0.018 | 1.71E-06 | 23.111 |
| genus | Lactococcus | rs4766997 | T | C | 0.115 | 0.024 | 2.06E-06 | 23.109 |
| genus | Alloprevotella | rs7004821 | G | A | 0.139 | 0.029 | 2.67E-06 | 23.108 |
| genus | Bifidobacterium | rs1374291 | T | C | 0.058 | 0.012 | 1.13E-06 | 23.108 |
| genus |  | rs55966090 | C | T | -0.078 | 0.016 | 9.04E-07 | 23.106 |
| genus | Bifidobacterium | rs41269821 | A | G | 0.077 | 0.016 | 1.60E-06 | 23.099 |
| genus | Blautia | rs12670293 | G | A | 0.060 | 0.012 | 1.50E-06 | 23.097 |
| genus | Butyrivibrio | rs455247 | A | G | -0.115 | 0.024 | 1.76E-06 | 23.096 |
| genus | Coprococcus1 | rs56405618 | G | A | -0.090 | 0.019 | 1.57E-06 | 23.095 |
| genus | Gordonibacter | rs112842944 | A | C | 0.181 | 0.038 | 1.48E-06 | 23.095 |
| genus | Lachnoclostridium | rs615997 | C | T | 0.051 | 0.011 | 2.03E-06 | 23.094 |
| genus | Alistipes | rs2871785 | G | T | -0.053 | 0.011 | 1.68E-06 | 23.094 |
| genus | RuminococcaceaeUCG014 | rs9290441 | T | G | -0.057 | 0.012 | 1.61E-06 | 23.089 |
| genus | Bacteroides | rs28757219 | A | T | 0.082 | 0.017 | 1.29E-06 | 23.089 |
| genus | Bifidobacterium | rs512375 | T | G | -0.058 | 0.012 | 1.96E-06 | 23.088 |
| genus | Bifidobacterium | rs177917 | A | G | 0.075 | 0.016 | 1.62E-06 | 23.087 |
| genus |  | rs34085021 | G | A | 0.069 | 0.014 | 2.01E-06 | 23.086 |
| genus | Marvinbryantia | rs61884469 | G | A | 0.119 | 0.025 | 3.00E-06 | 23.085 |
| genus | Adlercreutzia | rs2147798 | G | C | 0.092 | 0.019 | 1.40E-06 | 23.079 |
| genus | RuminococcaceaeUCG011 | rs12724496 | T | C | -0.120 | 0.025 | 1.94E-06 | 23.079 |
| genus | Intestinimonas | rs11679012 | G | A | 0.080 | 0.017 | 1.46E-06 | 23.078 |
| genus | RuminococcaceaeUCG014 | rs72809222 | C | T | 0.067 | 0.014 | 2.41E-06 | 23.078 |
| genus | LachnospiraceaeFCS020group | rs9788306 | T | C | -0.063 | 0.013 | 1.39E-06 | 23.074 |
| genus | RuminococcaceaeNK4A214group | rs73158814 | G | C | -0.109 | 0.023 | 2.20E-06 | 23.073 |
| genus | Bifidobacterium | rs309170 | C | T | 0.076 | 0.016 | 1.70E-06 | 23.070 |
| genus | Allisonella | rs12115448 | A | G | 0.143 | 0.030 | 2.20E-06 | 23.069 |
| genus | Escherichia | rs73208162 | G | A | -0.119 | 0.025 | 2.19E-06 | 23.066 |
| genus | Peptococcus | rs406155 | G | A | -0.109 | 0.023 | 2.37E-06 | 23.066 |
| genus |  | rs2733072 | A | G | 0.110 | 0.023 | 1.49E-06 | 23.064 |
| genus | Oxalobacter | rs62341594 | C | T | 0.203 | 0.042 | 1.71E-06 | 23.064 |
| genus | Alistipes | rs67705352 | G | T | -0.053 | 0.011 | 1.65E-06 | 23.062 |
| genus | Intestinibacter | rs478972 | C | T | -0.143 | 0.030 | 1.82E-06 | 23.061 |
| genus | Intestinibacter | rs1589606 | A | G | -0.143 | 0.030 | 1.82E-06 | 23.061 |
| genus |  | rs56815850 | A | G | -0.079 | 0.016 | 9.91E-07 | 23.061 |
| genus | RuminococcaceaeUCG002 | rs4457071 | T | C | -0.058 | 0.012 | 1.89E-06 | 23.060 |
| genus |  | rs158115 | C | G | 0.092 | 0.019 | 9.74E-06 | 23.057 |
| genus | Intestinimonas | rs57647068 | A | G | 0.080 | 0.017 | 1.47E-06 | 23.055 |
| genus | Lactobacillus | rs7399816 | G | A | -0.107 | 0.022 | 3.67E-06 | 23.055 |
| genus | Coprococcus2 | rs1958519 | A | T | 0.067 | 0.014 | 1.58E-06 | 23.055 |
| genus | Parasutterella | rs6828768 | T | C | 0.064 | 0.013 | 1.78E-06 | 23.051 |
| genus | Lactobacillus | rs16861661 | A | G | -0.183 | 0.038 | 1.28E-06 | 23.049 |
| genus | Paraprevotella | rs4767113 | T | C | 0.088 | 0.018 | 2.14E-06 | 23.047 |
| genus | Streptococcus | rs10028567 | T | C | -0.092 | 0.019 | 7.30E-06 | 23.047 |
| genus | Bifidobacterium | rs4988243 | T | C | 0.114 | 0.024 | 1.37E-06 | 23.043 |
| genus | Sellimonas | rs2448810 | C | T | -0.123 | 0.026 | 1.96E-06 | 23.042 |
| genus | Butyrivibrio | rs7412979 | G | C | 0.187 | 0.039 | 1.71E-06 | 23.041 |
| genus | RuminococcaceaeUCG011 | rs12406974 | A | G | -0.120 | 0.025 | 1.96E-06 | 23.040 |
| genus | Actinomyces | rs17072071 | A | C | -0.120 | 0.025 | 1.33E-06 | 23.039 |
| genus | Sellimonas | rs2606147 | G | A | -0.123 | 0.026 | 1.96E-06 | 23.037 |
| genus | Bifidobacterium | rs3098103 | T | C | 0.078 | 0.016 | 2.28E-06 | 23.036 |
| genus | RuminococcaceaeUCG002 | rs11750293 | T | G | -0.058 | 0.012 | 1.76E-06 | 23.028 |
| genus | Odoribacter | rs552702 | G | T | 0.056 | 0.012 | 1.64E-06 | 23.027 |
| genus | Ruminiclostridium5 | rs4766415 | T | A | -0.052 | 0.011 | 1.82E-06 | 23.026 |
| genus | LachnospiraceaeFCS020group | rs398357 | A | C | 0.125 | 0.026 | 3.28E-06 | 23.022 |
| genus | Parasutterella | rs6851752 | G | A | 0.064 | 0.013 | 1.84E-06 | 23.019 |
| genus | FamilyXIIIAD3011group | rs9813981 | A | G | 0.062 | 0.013 | 1.81E-06 | 23.019 |
| genus | Bifidobacterium | rs1592 | A | C | 0.057 | 0.012 | 1.96E-06 | 23.016 |
| genus | Ruminiclostridium9 | rs73168795 | G | C | -0.056 | 0.012 | 1.64E-06 | 23.015 |
| genus | Peptococcus | rs34282744 | C | G | 0.192 | 0.040 | 1.84E-06 | 23.011 |
| genus |  | rs633372 | G | A | -0.051 | 0.011 | 1.62E-06 | 23.011 |
| genus | Coprococcus2 | rs6012988 | G | C | 0.111 | 0.023 | 1.63E-06 | 23.009 |
| genus | LachnospiraceaeUCG008 | rs7684377 | G | A | -0.081 | 0.017 | 1.78E-06 | 23.008 |
| genus | Oscillibacter | rs36095275 | T | C | -0.075 | 0.016 | 1.40E-06 | 23.005 |
| genus | FamilyXIIIUCG001 | rs17604797 | G | A | -0.066 | 0.014 | 1.65E-06 | 23.004 |
| genus | Peptococcus | rs56981635 | C | T | 0.155 | 0.032 | 1.90E-06 | 23.001 |
| genus | Paraprevotella | rs9547277 | C | T | -0.106 | 0.022 | 7.38E-07 | 22.999 |
| genus |  | rs7154654 | G | T | -0.200 | 0.042 | 2.59E-06 | 22.998 |
| genus | Erysipelatoclostridium | rs2492603 | C | T | 0.084 | 0.017 | 1.97E-06 | 22.992 |
| genus | Parasutterella | rs11731112 | A | T | 0.064 | 0.013 | 1.81E-06 | 22.989 |
| genus | Blautia | rs72607722 | G | A | 0.060 | 0.012 | 1.56E-06 | 22.981 |
| genus | Butyrivibrio | rs455335 | G | A | -0.115 | 0.024 | 1.86E-06 | 22.979 |
| genus | Parasutterella | rs7675533 | C | A | 0.064 | 0.013 | 1.88E-06 | 22.978 |
| genus | CandidatusSoleaferrea | rs1891244 | A | T | 0.111 | 0.023 | 1.72E-06 | 22.977 |
| genus | LachnospiraceaeUCG008 | rs29304 | A | G | -0.081 | 0.017 | 1.79E-06 | 22.975 |
| genus | RuminococcaceaeUCG002 | rs12023531 | T | C | -0.071 | 0.015 | 1.05E-06 | 22.971 |
| genus | Hungatella | rs72699560 | G | A | -0.160 | 0.033 | 2.62E-06 | 22.969 |
| genus | Tyzzerella3 | rs55799124 | G | A | -0.114 | 0.024 | 1.34E-06 | 22.968 |
| genus | Streptococcus | rs11110165 | T | G | -0.083 | 0.017 | 1.62E-06 | 22.967 |
| genus | RuminococcaceaeUCG011 | rs79113084 | T | C | -0.152 | 0.032 | 2.06E-06 | 22.966 |
| genus | Ruminococcus2 | rs7635831 | A | G | 0.062 | 0.013 | 1.98E-06 | 22.965 |
| genus | Dialister | rs4263802 | A | G | 0.063 | 0.013 | 1.33E-06 | 22.964 |
| genus | Alloprevotella | rs909327 | T | C | 0.139 | 0.029 | 3.07E-06 | 22.962 |
| genus | Marvinbryantia | rs78451158 | A | C | -0.074 | 0.016 | 2.01E-06 | 22.961 |
| genus | Oscillibacter | rs9348810 | T | C | -0.072 | 0.015 | 1.97E-06 | 22.961 |
| genus |  | rs139742 | C | T | -0.082 | 0.017 | 1.64E-06 | 22.959 |
| genus | Paraprevotella | rs4756632 | T | G | -0.139 | 0.029 | 3.82E-06 | 22.959 |
| genus | Parabacteroides | rs3860755 | C | G | 0.056 | 0.012 | 1.71E-06 | 22.957 |
| genus | Lachnoclostridium | rs3790181 | T | G | -0.052 | 0.011 | 1.95E-06 | 22.953 |
| genus | Bifidobacterium | rs6430549 | A | G | 0.057 | 0.012 | 1.08E-06 | 22.949 |
| genus | Alistipes | rs7129639 | A | C | -0.052 | 0.011 | 1.78E-06 | 22.948 |
| genus | Ruminiclostridium9 | rs2615052 | G | T | 0.058 | 0.012 | 1.75E-06 | 22.947 |
| genus | Bifidobacterium | rs660002 | C | T | 0.074 | 0.015 | 2.04E-06 | 22.946 |
| genus | Hungatella | rs66832370 | C | G | -0.160 | 0.033 | 2.62E-06 | 22.945 |
| genus | Hungatella | rs35209497 | G | C | -0.160 | 0.033 | 2.59E-06 | 22.941 |
| genus | Eggerthella | rs1860517 | A | G | -0.096 | 0.020 | 1.66E-06 | 22.940 |
| genus | RuminococcaceaeUCG011 | rs56837493 | C | T | -0.120 | 0.025 | 2.11E-06 | 22.928 |
| genus | Butyrivibrio | rs12214380 | C | T | -0.115 | 0.024 | 1.91E-06 | 22.927 |
| genus | RuminococcaceaeUCG005 | rs146613210 | C | T | 0.153 | 0.032 | 2.05E-06 | 22.925 |
| genus | LachnospiraceaeUCG008 | rs4833423 | G | A | -0.081 | 0.017 | 1.85E-06 | 22.922 |
| genus | Butyrivibrio | rs466923 | C | T | -0.115 | 0.024 | 1.93E-06 | 22.919 |
| genus | Bifidobacterium | rs499483 | G | A | 0.078 | 0.016 | 2.33E-06 | 22.918 |
| genus | Hungatella | rs13128780 | C | T | -0.150 | 0.031 | 1.75E-06 | 22.915 |
| genus | Ruminiclostridium9 | rs1845139 | C | T | -0.054 | 0.011 | 1.84E-06 | 22.912 |
| genus | Ruminiclostridium9 | rs1845138 | C | T | -0.054 | 0.011 | 1.84E-06 | 22.912 |
| genus | Parasutterella | rs7673335 | A | G | 0.063 | 0.013 | 1.90E-06 | 22.908 |
| genus |  | rs61969852 | A | T | -0.160 | 0.034 | 2.09E-06 | 22.908 |
| genus | Hungatella | rs13104209 | T | C | -0.150 | 0.031 | 1.77E-06 | 22.907 |
| genus | Bifidobacterium | rs3814358 | A | C | 0.057 | 0.012 | 1.13E-06 | 22.906 |
| genus | Bifidobacterium | rs4954191 | C | G | 0.057 | 0.012 | 1.05E-06 | 22.904 |
| genus | RuminococcaceaeUCG003 | rs692854 | C | A | 0.057 | 0.012 | 1.85E-06 | 22.903 |
| genus | Ruminiclostridium9 | rs62286617 | C | T | -0.054 | 0.011 | 1.86E-06 | 22.898 |
| genus | Streptococcus | rs77628647 | G | A | -0.089 | 0.019 | 1.89E-06 | 22.895 |
| genus | RuminococcaceaeUCG002 | rs116974815 | A | C | -0.190 | 0.040 | 2.03E-06 | 22.890 |
| genus | Ruminiclostridium9 | rs10936144 | C | T | -0.061 | 0.013 | 2.33E-06 | 22.889 |
| genus | Butyrivibrio | rs467042 | T | C | -0.115 | 0.024 | 1.94E-06 | 22.889 |
| genus | Hungatella | rs72699558 | C | T | -0.160 | 0.033 | 2.64E-06 | 22.889 |
| genus | Lachnoclostridium | rs2024617 | C | T | -0.052 | 0.011 | 1.97E-06 | 22.889 |
| genus | Tyzzerella3 | rs74583271 | C | T | -0.132 | 0.028 | 3.45E-06 | 22.883 |
| genus | Anaerostipes | rs2804244 | G | A | -0.053 | 0.011 | 2.04E-06 | 22.882 |
| genus | Odoribacter | rs17781591 | G | A | -0.131 | 0.027 | 1.62E-06 | 22.881 |
| genus |  | rs9627308 | G | C | 0.141 | 0.029 | 7.61E-06 | 22.880 |
| genus | RuminococcaceaeUCG002 | rs58365817 | G | A | -0.058 | 0.012 | 1.90E-06 | 22.880 |
| genus | LachnospiraceaeUCG008 | rs7108105 | G | C | 0.087 | 0.018 | 2.08E-06 | 22.879 |
| genus | Flavonifractor | rs34066017 | G | A | 0.076 | 0.016 | 1.52E-06 | 22.878 |
| genus | Streptococcus | rs114279530 | G | A | -0.087 | 0.018 | 1.39E-06 | 22.878 |
| genus | Lachnoclostridium | rs6035283 | G | T | -0.052 | 0.011 | 2.04E-06 | 22.878 |
| genus | Ruminiclostridium9 | rs7628293 | A | G | -0.054 | 0.011 | 1.91E-06 | 22.877 |
| genus | Hungatella | rs35704070 | C | T | -0.160 | 0.033 | 2.62E-06 | 22.876 |
| genus | Bifidobacterium | rs12469411 | C | T | 0.057 | 0.012 | 1.17E-06 | 22.875 |
| genus | Olsenella | rs72691585 | A | C | -0.249 | 0.052 | 2.95E-06 | 22.872 |
| genus | LachnospiraceaeUCG008 | rs11025180 | C | A | -0.094 | 0.020 | 2.12E-06 | 22.872 |
| genus | Turicibacter | rs55756211 | C | T | -0.115 | 0.024 | 2.81E-06 | 22.871 |
| genus | RuminococcaceaeUCG011 | rs12738929 | C | G | -0.119 | 0.025 | 2.16E-06 | 22.871 |
| genus | Marvinbryantia | rs2815664 | T | C | -0.074 | 0.015 | 2.05E-06 | 22.870 |
| genus | Adlercreutzia | rs34181676 | T | G | -0.135 | 0.028 | 2.34E-06 | 22.867 |
| genus | Dialister | rs11166707 | G | C | 0.063 | 0.013 | 1.43E-06 | 22.865 |
| genus |  | rs4463323 | G | A | -0.077 | 0.016 | 9.72E-07 | 22.863 |
| genus | Blautia | rs66699833 | G | A | 0.059 | 0.012 | 1.73E-06 | 22.861 |
| genus | Romboutsia | rs61841477 | C | A | 0.082 | 0.017 | 1.25E-06 | 22.860 |
| genus | Prevotella9 | rs11685699 | T | C | -0.141 | 0.030 | 2.03E-06 | 22.858 |
| genus | Barnesiella | rs36119320 | G | A | 0.092 | 0.019 | 2.95E-06 | 22.858 |
| genus | RuminococcaceaeUCG003 | rs571689 | C | T | 0.057 | 0.012 | 1.89E-06 | 22.857 |
| genus | Akkermansia | rs9349825 | G | A | -0.070 | 0.015 | 2.60E-06 | 22.856 |
| genus | Bifidobacterium | rs12464050 | G | T | 0.078 | 0.016 | 2.36E-06 | 22.855 |
| genus | Paraprevotella | rs9547270 | C | T | -0.082 | 0.017 | 2.07E-06 | 22.854 |
| genus |  | rs427248 | C | T | -0.052 | 0.011 | 1.52E-06 | 22.851 |
| genus | Parasutterella | rs11131561 | G | A | 0.063 | 0.013 | 2.03E-06 | 22.844 |
| genus | Prevotella9 | rs2683313 | G | A | -0.072 | 0.015 | 1.69E-06 | 22.843 |
| genus | RuminococcaceaeUCG011 | rs1416039 | C | A | -0.142 | 0.030 | 1.74E-06 | 22.843 |
| genus | RuminococcaceaeUCG011 | rs9404561 | C | G | -0.142 | 0.030 | 1.74E-06 | 22.843 |
| genus | Tyzzerella3 | rs7019909 | C | T | 0.144 | 0.030 | 1.76E-06 | 22.841 |
| genus | RuminococcaceaeUCG011 | rs12758559 | A | G | -0.119 | 0.025 | 2.17E-06 | 22.838 |
| genus | Streptococcus | rs111923753 | C | T | -0.088 | 0.018 | 1.80E-06 | 22.836 |
| genus |  | rs12423772 | T | G | 0.141 | 0.030 | 2.63E-06 | 22.834 |
| genus | Parasutterella | rs4860618 | A | G | 0.063 | 0.013 | 2.01E-06 | 22.834 |
| genus | RuminococcaceaeUCG003 | rs492602 | A | G | 0.057 | 0.012 | 2.03E-06 | 22.834 |
| genus | Peptococcus | rs56272717 | C | G | 0.155 | 0.032 | 1.58E-06 | 22.833 |
| genus | RuminococcaceaeUCG011 | rs4652383 | T | A | -0.119 | 0.025 | 2.21E-06 | 22.831 |
| genus | Methanobrevibacter | rs3923545 | G | C | 0.217 | 0.045 | 2.01E-06 | 22.831 |
| genus | Subdoligranulum | . | A | C | -0.053 | 0.011 | 2.08E-06 | 22.828 |
| genus | Bifidobacterium | rs77096272 | C | A | 0.078 | 0.016 | 2.42E-06 | 22.827 |
| genus | Ruminiclostridium6 | rs79968172 | A | G | 0.116 | 0.024 | 1.66E-06 | 22.827 |
| genus | Bifidobacterium | rs6728946 | C | G | 0.076 | 0.016 | 2.13E-06 | 22.826 |
| genus | Parasutterella | rs4304006 | T | C | 0.063 | 0.013 | 2.02E-06 | 22.825 |
| genus | Butyrivibrio | rs465795 | T | C | -0.115 | 0.024 | 2.01E-06 | 22.822 |
| genus | Haemophilus | rs111582866 | A | G | -0.124 | 0.026 | 1.27E-06 | 22.815 |
| genus | Allisonella | rs13286510 | T | A | 0.142 | 0.030 | 2.66E-06 | 22.813 |
| genus | RuminococcaceaeUCG002 | rs117226190 | C | G | -0.134 | 0.028 | 2.54E-06 | 22.812 |
| genus |  | rs440215 | T | C | 0.093 | 0.020 | 1.65E-06 | 22.812 |
| genus | Akkermansia | rs4936098 | G | A | 0.065 | 0.014 | 1.10E-06 | 22.811 |
| genus | LachnospiraceaeUCG001 | rs2148629 | A | G | 0.073 | 0.015 | 1.85E-06 | 22.809 |
| genus |  | rs17169417 | T | C | -0.078 | 0.016 | 1.08E-06 | 22.809 |
| genus | Prevotella7 | rs62115045 | G | A | 0.152 | 0.032 | 1.27E-06 | 22.807 |
| genus | Butyrivibrio | rs456871 | T | C | -0.114 | 0.024 | 2.02E-06 | 22.807 |
| genus | Oscillibacter | rs9380079 | C | T | -0.072 | 0.015 | 2.15E-06 | 22.806 |
| genus | Paraprevotella | rs11653132 | G | T | -0.082 | 0.017 | 2.71E-06 | 22.804 |
| genus | Butyricimonas | rs4746071 | C | T | 0.068 | 0.014 | 1.19E-06 | 22.803 |
| genus | Lactococcus | rs34189344 | G | A | 0.115 | 0.024 | 1.32E-06 | 22.803 |
| genus | Paraprevotella | rs9575904 | A | G | -0.084 | 0.018 | 2.12E-06 | 22.802 |
| genus |  | rs139745 | T | C | -0.082 | 0.017 | 1.78E-06 | 22.802 |
| genus | Ruminiclostridium9 | rs56410505 | G | A | -0.054 | 0.011 | 1.95E-06 | 22.799 |
| genus | RuminococcaceaeUCG011 | rs7521106 | G | A | -0.119 | 0.025 | 2.25E-06 | 22.798 |
| genus | Ruminiclostridium9 | rs6797225 | T | C | -0.054 | 0.011 | 1.95E-06 | 22.798 |
| genus | Tyzzerella3 | rs111752463 | G | A | -0.131 | 0.028 | 3.34E-06 | 22.795 |
| genus | Peptococcus | rs6814734 | C | T | -0.108 | 0.023 | 2.22E-06 | 22.795 |
| genus | Bifidobacterium | rs669759 | T | C | -0.059 | 0.012 | 1.00E-06 | 22.793 |
| genus | Butyrivibrio | rs10456875 | G | A | -0.115 | 0.024 | 2.03E-06 | 22.793 |
| genus | Ruminococcus2 | rs7638158 | T | A | 0.062 | 0.013 | 2.19E-06 | 22.791 |
| genus |  | rs12659207 | C | G | -0.109 | 0.023 | 2.49E-06 | 22.790 |
| genus | Butyrivibrio | rs4537857 | C | T | -0.125 | 0.026 | 1.80E-06 | 22.788 |
| genus | Ruminiclostridium6 | rs77968078 | A | G | 0.119 | 0.025 | 2.63E-06 | 22.788 |
| genus | Anaerostipes | rs62157625 | C | T | 0.089 | 0.019 | 1.45E-06 | 22.787 |
| genus | LachnospiraceaeND3007group | rs11248851 | C | T | -0.055 | 0.012 | 1.80E-06 | 22.786 |
| genus | LachnospiraceaeNK4A136group | rs954878 | G | A | -0.052 | 0.011 | 1.78E-06 | 22.782 |
| genus | Intestinibacter | rs2204169 | C | A | -0.142 | 0.030 | 2.04E-06 | 22.780 |
| genus | Butyrivibrio | rs110732 | T | G | -0.114 | 0.024 | 2.06E-06 | 22.776 |
| genus | Bifidobacterium | rs11688414 | A | G | 0.057 | 0.012 | 1.70E-06 | 22.774 |
| genus | Parasutterella | rs4599442 | T | C | 0.063 | 0.013 | 2.10E-06 | 22.772 |
| genus | RuminococcaceaeUCG002 | rs62374287 | T | C | -0.058 | 0.012 | 2.14E-06 | 22.772 |
| genus | Terrisporobacter | rs11042141 | C | T | 0.208 | 0.044 | 1.72E-06 | 22.770 |
| genus | Akkermansia | rs2602429 | T | C | 0.075 | 0.016 | 2.72E-06 | 22.770 |
| genus | Haemophilus | rs35509 | A | G | 0.128 | 0.027 | 2.01E-06 | 22.768 |
| genus |  | rs646327 | A | G | -0.051 | 0.011 | 1.89E-06 | 22.767 |
| genus | Parasutterella | rs4355422 | C | T | 0.063 | 0.013 | 2.04E-06 | 22.767 |
| genus | Butyrivibrio | rs71640701 | G | A | 0.187 | 0.039 | 2.12E-06 | 22.766 |
| genus | Bilophila | rs10758666 | G | T | 0.082 | 0.017 | 2.22E-06 | 22.766 |
| genus | Bilophila | rs3827020 | T | C | 0.077 | 0.016 | 1.79E-06 | 22.766 |
| genus | Ruminococcus1 | rs1348965 | C | T | -0.055 | 0.012 | 2.11E-06 | 22.764 |
| genus | Ruminiclostridium5 | rs2482038 | A | C | 0.052 | 0.011 | 1.70E-06 | 22.764 |
| genus | Allisonella | rs13300328 | G | C | 0.142 | 0.030 | 2.72E-06 | 22.762 |
| genus | Bifidobacterium | rs7582173 | G | A | 0.074 | 0.015 | 5.46E-06 | 22.761 |
| genus | Paraprevotella | rs1156275 | T | G | -0.084 | 0.018 | 2.25E-06 | 22.761 |
| genus | RuminococcaceaeUCG011 | rs10913766 | A | G | -0.119 | 0.025 | 2.29E-06 | 22.759 |
| genus | Ruminiclostridium5 | rs1044471 | C | T | -0.051 | 0.011 | 2.05E-06 | 22.750 |
| genus | Intestinimonas | rs2930225 | T | G | 0.073 | 0.015 | 1.35E-06 | 22.750 |
| genus | Streptococcus | rs75088533 | C | T | -0.089 | 0.019 | 2.02E-06 | 22.748 |
| genus | Butyrivibrio | rs7992159 | C | T | -0.124 | 0.026 | 1.85E-06 | 22.748 |
| genus | Holdemanella | rs4541991 | C | T | -0.093 | 0.019 | 2.10E-06 | 22.747 |
| genus | Alistipes | rs7783848 | C | G | -0.052 | 0.011 | 2.00E-06 | 22.747 |
| genus | Ruminiclostridium9 | rs55643438 | T | C | -0.054 | 0.011 | 1.99E-06 | 22.746 |
| genus | Bifidobacterium | rs80261410 | T | C | 0.065 | 0.014 | 2.66E-06 | 22.745 |
| genus | Enterorhabdus | rs114731706 | G | T | 0.182 | 0.038 | 2.17E-06 | 22.741 |
| genus | LachnospiraceaeNK4A136group | rs7616165 | T | G | -0.231 | 0.048 | 2.77E-06 | 22.739 |
| genus | Intestinibacter | rs7936728 | G | T | -0.142 | 0.030 | 2.08E-06 | 22.738 |
| genus |  | rs7146815 | G | A | -0.162 | 0.034 | 2.72E-06 | 22.737 |
| genus | RuminococcaceaeUCG011 | rs12758573 | A | C | -0.119 | 0.025 | 2.29E-06 | 22.736 |
| genus | Bifidobacterium | rs6713776 | C | G | 0.073 | 0.015 | 5.58E-06 | 22.735 |
| genus | Bifidobacterium | rs59213715 | A | C | 0.078 | 0.016 | 2.12E-06 | 22.735 |
| genus |  | rs74018587 | T | C | 0.209 | 0.044 | 3.70E-06 | 22.734 |
| genus | Prevotella7 | rs17088300 | T | A | 0.131 | 0.028 | 2.59E-06 | 22.733 |
| genus | DefluviitaleaceaeUCG011 | rs9608282 | G | T | 0.143 | 0.030 | 2.52E-06 | 22.733 |
| genus | LachnospiraceaeUCG001 | rs17429360 | G | C | 0.073 | 0.015 | 1.90E-06 | 22.731 |
| genus | RuminococcaceaeUCG003 | rs57120461 | C | G | 0.095 | 0.020 | 3.85E-06 | 22.728 |
| genus | Paraprevotella | rs1414012 | T | G | -0.084 | 0.018 | 1.93E-06 | 22.728 |
| genus | LachnospiraceaeFCS020group | rs35781607 | T | C | -0.062 | 0.013 | 1.79E-06 | 22.725 |
| genus | Sellimonas | rs2448945 | A | G | -0.123 | 0.026 | 2.41E-06 | 22.724 |
| genus | Parasutterella | rs11942422 | T | A | 0.063 | 0.013 | 2.12E-06 | 22.722 |
| genus | Intestinimonas | rs7568334 | T | A | 0.079 | 0.017 | 1.76E-06 | 22.721 |
| genus | Eisenbergiella | rs2683098 | T | C | 0.107 | 0.023 | 2.24E-06 | 22.720 |
| genus | Bifidobacterium | rs4567981 | A | T | 0.056 | 0.012 | 1.93E-06 | 22.720 |
| genus | RuminococcaceaeUCG003 | rs2251034 | G | A | 0.056 | 0.012 | 2.15E-06 | 22.716 |
| genus | Oscillibacter | rs9393921 | G | T | -0.072 | 0.015 | 2.19E-06 | 22.716 |
| genus | Bifidobacterium | rs2053726 | A | G | 0.057 | 0.012 | 1.21E-06 | 22.716 |
| genus | RuminococcaceaeUCG014 | rs10936708 | C | G | 0.057 | 0.012 | 2.05E-06 | 22.715 |
| genus | Parasutterella | rs4272050 | A | G | 0.063 | 0.013 | 2.13E-06 | 22.712 |
| genus | Butyrivibrio | rs7997900 | T | C | -0.124 | 0.026 | 1.87E-06 | 22.711 |
| genus | Hungatella | rs34258309 | C | A | -0.160 | 0.033 | 2.92E-06 | 22.709 |
| genus | Ruminiclostridium9 | rs15967 | C | A | -0.054 | 0.011 | 2.07E-06 | 22.709 |
| genus | LachnospiraceaeUCG008 | rs13433770 | A | G | -0.105 | 0.022 | 1.84E-06 | 22.709 |
| genus | RuminococcaceaeUCG011 | rs12739116 | C | G | -0.119 | 0.025 | 2.35E-06 | 22.708 |
| genus | RuminococcaceaeUCG011 | rs4233180 | A | G | -0.119 | 0.025 | 2.29E-06 | 22.708 |
| genus | Marvinbryantia | rs75360912 | C | G | -0.074 | 0.016 | 2.25E-06 | 22.707 |
| genus | LachnospiraceaeFCS020group | rs366924 | A | T | 0.123 | 0.026 | 4.13E-06 | 22.707 |
| genus | Intestinibacter | rs16938435 | C | T | -0.112 | 0.024 | 1.80E-06 | 22.706 |
| genus | Subdoligranulum | rs6555306 | C | T | -0.074 | 0.016 | 2.81E-06 | 22.705 |
| genus | LachnospiraceaeUCG008 | rs34527770 | G | A | 0.101 | 0.021 | 2.02E-06 | 22.702 |
| genus | Allisonella | rs1048451 | A | G | 0.142 | 0.030 | 2.93E-06 | 22.697 |
| genus | Lactococcus | rs55910161 | T | C | 0.146 | 0.031 | 2.36E-06 | 22.695 |
| genus | Intestinibacter | rs7949903 | T | C | -0.142 | 0.030 | 2.13E-06 | 22.694 |
| genus | Intestinibacter | rs7950155 | T | C | -0.142 | 0.030 | 2.13E-06 | 22.694 |
| genus |  | rs7720502 | A | G | 0.089 | 0.019 | 1.70E-06 | 22.694 |
| genus | RuminococcaceaeUCG003 | rs16959793 | C | A | -0.063 | 0.013 | 2.22E-06 | 22.692 |
| genus | Ruminiclostridium6 | rs1377110 | G | A | 0.063 | 0.013 | 1.60E-06 | 22.692 |
| genus | Akkermansia | rs74542928 | C | T | 0.113 | 0.024 | 1.48E-06 | 22.690 |
| genus |  | rs1496279 | T | A | -0.088 | 0.018 | 2.31E-06 | 22.688 |
| genus |  | rs139735 | A | C | -0.083 | 0.017 | 1.80E-06 | 22.679 |
| genus | Bifidobacterium | rs7606267 | T | C | 0.076 | 0.016 | 2.19E-06 | 22.678 |
| genus | Butyrivibrio | rs62420437 | C | T | -0.114 | 0.024 | 2.16E-06 | 22.678 |
| genus |  | rs57026722 | A | G | -0.078 | 0.016 | 1.21E-06 | 22.677 |
| genus | Allisonella | rs4743086 | T | G | 0.142 | 0.030 | 2.34E-06 | 22.676 |
| genus | Subdoligranulum | rs9842934 | C | T | 0.052 | 0.011 | 1.79E-06 | 22.674 |
| genus | Sutterella | rs2321387 | A | G | -0.059 | 0.012 | 1.87E-06 | 22.674 |
| genus | Lachnoclostridium | rs10854245 | C | T | -0.052 | 0.011 | 1.88E-06 | 22.673 |
| genus | Bifidobacterium | rs7573555 | T | C | 0.076 | 0.016 | 2.18E-06 | 22.667 |
| genus | Bifidobacterium | rs62181700 | A | G | -0.062 | 0.013 | 2.17E-06 | 22.665 |
| genus | Intestinimonas | rs7568340 | T | G | 0.079 | 0.017 | 1.81E-06 | 22.662 |
| genus | Alistipes | rs34890041 | C | A | -0.052 | 0.011 | 2.13E-06 | 22.662 |
| genus | LachnospiraceaeFCS020group | rs12078956 | G | C | 0.106 | 0.022 | 2.15E-06 | 22.657 |
| genus | Lachnoclostridium | rs62285313 | G | A | 0.086 | 0.018 | 1.58E-06 | 22.655 |
| genus | Bifidobacterium | rs75128007 | A | G | 0.065 | 0.014 | 2.81E-06 | 22.654 |
| genus | ErysipelotrichaceaeUCG003 | rs11994308 | T | C | 0.115 | 0.024 | 1.33E-06 | 22.652 |
| genus | Bifidobacterium | rs586964 | G | A | 0.077 | 0.016 | 2.69E-06 | 22.651 |
| genus | RuminococcaceaeUCG011 | rs12724634 | T | C | -0.119 | 0.025 | 2.42E-06 | 22.651 |
| genus | Parasutterella | rs4860617 | A | G | 0.063 | 0.013 | 2.21E-06 | 22.651 |
| genus | Hungatella | rs34718907 | C | T | -0.159 | 0.033 | 3.01E-06 | 22.651 |
| genus | ChristensenellaceaeR | rs10420576 | T | C | 0.055 | 0.012 | 1.96E-06 | 22.650 |
| genus |  | rs79582700 | G | C | -0.095 | 0.020 | 2.41E-06 | 22.649 |
| genus | Ruminococcus2 | rs10199274 | C | G | 0.066 | 0.014 | 2.45E-06 | 22.649 |
| genus | Erysipelatoclostridium | rs587790 | A | G | 0.083 | 0.017 | 2.34E-06 | 22.648 |
| genus | LachnospiraceaeUCG001 | rs6699460 | G | A | 0.073 | 0.015 | 2.08E-06 | 22.648 |
| genus | Intestinimonas | rs13422601 | A | G | 0.079 | 0.017 | 1.80E-06 | 22.647 |
| genus | LachnospiraceaeNK4A136group | rs68104925 | C | T | -0.055 | 0.012 | 2.37E-06 | 22.647 |
| genus | RuminococcaceaeUCG014 | rs4586787 | C | T | 0.057 | 0.012 | 2.12E-06 | 22.645 |
| genus | Veillonella | rs4831426 | A | G | -0.078 | 0.016 | 2.41E-06 | 22.645 |
| genus | RuminococcaceaeUCG005 | rs6958972 | T | G | 0.054 | 0.011 | 2.21E-06 | 22.644 |
| genus | Ruminiclostridium9 | rs6784019 | G | A | -0.054 | 0.011 | 2.19E-06 | 22.641 |
| genus | Parasutterella | rs1403396 | T | A | -0.076 | 0.016 | 2.76E-06 | 22.641 |
| genus | LachnospiraceaeUCG001 | rs10815577 | G | C | -0.068 | 0.014 | 1.72E-06 | 22.640 |
| genus | Butyrivibrio | rs16941336 | T | C | 0.127 | 0.027 | 1.53E-06 | 22.640 |
| genus | Butyrivibrio | rs9581729 | T | C | -0.124 | 0.026 | 1.95E-06 | 22.639 |
| genus | Ruminococcus1 | rs4148897 | T | C | -0.055 | 0.012 | 2.45E-06 | 22.638 |
| genus | Senegalimassilia | rs57512504 | A | T | 0.082 | 0.017 | 2.03E-06 | 22.637 |
| genus | RuminococcaceaeUCG003 | rs570794 | T | C | 0.056 | 0.012 | 2.12E-06 | 22.636 |
| genus | LachnospiraceaeUCG008 | rs29305 | C | T | -0.081 | 0.017 | 2.13E-06 | 22.632 |
| genus | RuminococcaceaeUCG011 | rs12757935 | A | C | -0.119 | 0.025 | 2.44E-06 | 22.631 |
| genus | FamilyXIIIAD3011group | rs3012191 | T | C | -0.057 | 0.012 | 2.06E-06 | 22.630 |
| genus | Lactobacillus | rs62314653 | A | C | 0.188 | 0.039 | 2.24E-06 | 22.626 |
| genus |  | rs12964517 | A | G | 0.059 | 0.012 | 2.07E-06 | 22.624 |
| genus | LachnospiraceaeNC2004group | rs6116753 | A | G | 0.099 | 0.021 | 2.92E-06 | 22.623 |
| genus | Oxalobacter | rs10868235 | T | C | 0.099 | 0.021 | 2.03E-06 | 22.622 |
| genus | CandidatusSoleaferrea | rs12566578 | T | A | 0.108 | 0.023 | 2.46E-06 | 22.621 |
| genus | Victivallis | rs12512543 | C | A | -0.178 | 0.037 | 2.54E-06 | 22.619 |
| genus | Ruminiclostridium6 | rs17590101 | T | C | 0.153 | 0.032 | 2.73E-06 | 22.619 |
| genus | Parasutterella | rs6837522 | G | A | 0.063 | 0.013 | 2.26E-06 | 22.618 |
| genus | RuminococcaceaeUCG011 | rs11759449 | G | A | -0.141 | 0.030 | 1.90E-06 | 22.618 |
| genus | LachnospiraceaeFCS020group | rs4807301 | C | T | 0.064 | 0.013 | 2.13E-06 | 22.614 |
| genus |  | rs139746 | G | A | 0.077 | 0.016 | 2.27E-06 | 22.613 |
| genus | RuminococcaceaeUCG011 | rs12410988 | C | T | -0.119 | 0.025 | 2.50E-06 | 22.613 |
| genus | Marvinbryantia | rs2768445 | A | G | -0.075 | 0.016 | 2.62E-06 | 22.613 |
| genus | Butyrivibrio | rs9581727 | C | T | -0.124 | 0.026 | 1.99E-06 | 22.613 |
| genus | Romboutsia | rs34683673 | G | C | 0.076 | 0.016 | 2.05E-06 | 22.609 |
| genus | RuminococcaceaeUCG011 | rs10913767 | A | G | -0.119 | 0.025 | 2.47E-06 | 22.608 |
| genus | RuminococcaceaeUCG011 | rs12723027 | G | A | -0.119 | 0.025 | 2.43E-06 | 22.607 |
| genus | Lachnoclostridium | rs721489 | T | C | -0.051 | 0.011 | 2.35E-06 | 22.607 |
| genus | Intestinibacter | rs6590169 | T | C | -0.141 | 0.030 | 2.24E-06 | 22.607 |
| genus | Peptococcus | rs2054133 | A | G | 0.090 | 0.019 | 2.14E-06 | 22.606 |
| genus | Intestinimonas | rs9989754 | G | A | 0.079 | 0.017 | 1.88E-06 | 22.604 |
| genus | Veillonella | rs9792152 | A | G | -0.078 | 0.016 | 2.43E-06 | 22.603 |
| genus | RuminococcaceaeUCG011 | rs12749810 | T | G | -0.119 | 0.025 | 2.46E-06 | 22.603 |
| genus | RuminococcaceaeUCG011 | rs4625244 | A | C | -0.119 | 0.025 | 2.46E-06 | 22.603 |
| genus | RuminococcaceaeUCG011 | rs4562582 | C | A | -0.119 | 0.025 | 2.46E-06 | 22.603 |
| genus | Butyrivibrio | rs1374010 | C | T | -0.124 | 0.026 | 1.97E-06 | 22.603 |
| genus | Butyrivibrio | rs462493 | C | T | -0.114 | 0.024 | 2.25E-06 | 22.601 |
| genus | RuminococcaceaeUCG003 | rs1704773 | A | G | 0.056 | 0.012 | 2.25E-06 | 22.600 |
| genus | LachnospiraceaeNK4A136group | rs160061 | G | A | 0.051 | 0.011 | 2.12E-06 | 22.596 |
| genus | Lachnoclostridium | rs6045976 | G | C | -0.051 | 0.011 | 2.29E-06 | 22.596 |
| genus | RuminococcaceaeUCG011 | rs12746452 | G | A | -0.119 | 0.025 | 2.48E-06 | 22.595 |
| genus | RuminococcaceaeUCG011 | rs4626840 | C | T | -0.119 | 0.025 | 2.48E-06 | 22.595 |
| genus | RuminococcaceaeUCG011 | rs12746612 | C | T | -0.119 | 0.025 | 2.48E-06 | 22.595 |
| genus | RuminococcaceaeUCG011 | rs12741747 | C | G | -0.119 | 0.025 | 2.48E-06 | 22.595 |
| genus | RuminococcaceaeUCG011 | rs12723024 | A | G | -0.119 | 0.025 | 2.48E-06 | 22.595 |
| genus | Ruminiclostridium9 | rs1845140 | G | A | -0.054 | 0.011 | 2.16E-06 | 22.595 |
| genus | Anaerofilum | rs10794359 | C | T | -0.095 | 0.020 | 2.23E-06 | 22.594 |
| genus | RuminococcaceaeUCG011 | rs11802862 | C | A | -0.119 | 0.025 | 2.48E-06 | 22.593 |
| genus | Ruminiclostridium5 | rs10827477 | G | A | -0.055 | 0.012 | 2.19E-06 | 22.592 |
| genus |  | rs12653275 | G | A | -0.109 | 0.023 | 2.96E-06 | 22.592 |
| genus | Collinsella | rs2671662 | G | C | -0.057 | 0.012 | 2.22E-06 | 22.591 |
| genus | Butyrivibrio | rs9581728 | C | T | -0.124 | 0.026 | 2.00E-06 | 22.591 |
| genus | Butyrivibrio | rs9579031 | A | G | -0.124 | 0.026 | 2.00E-06 | 22.591 |
| genus | Parasutterella | rs11945744 | G | A | 0.063 | 0.013 | 2.27E-06 | 22.590 |
| genus | Enterorhabdus | rs73331712 | C | T | 0.262 | 0.055 | 4.85E-06 | 22.589 |
| genus | Dialister | rs4355808 | C | T | 0.063 | 0.013 | 1.65E-06 | 22.589 |
| genus | Butyrivibrio | rs7992015 | C | T | -0.124 | 0.026 | 2.01E-06 | 22.587 |
| genus | Butyrivibrio | rs7990675 | G | T | -0.124 | 0.026 | 2.01E-06 | 22.587 |
| genus | Bifidobacterium | rs766271 | C | T | 0.056 | 0.012 | 2.52E-06 | 22.586 |
| genus | Paraprevotella | rs145020347 | G | A | -0.125 | 0.026 | 4.03E-06 | 22.579 |
| genus | Senegalimassilia | rs11787826 | A | C | 0.081 | 0.017 | 2.63E-06 | 22.578 |
| genus | Parasutterella | rs13113077 | A | T | 0.063 | 0.013 | 2.35E-06 | 22.578 |
| genus | Allisonella | rs60681062 | T | C | 0.142 | 0.030 | 2.89E-06 | 22.573 |
| genus | Sutterella | rs143438747 | C | T | -0.146 | 0.031 | 3.28E-06 | 22.572 |
| genus | Ruminococcus2 | rs12986628 | T | C | 0.067 | 0.014 | 2.14E-06 | 22.572 |
| genus | LachnospiraceaeND3007group | rs761052 | A | G | -0.055 | 0.012 | 2.01E-06 | 22.572 |
| genus | Olsenella | rs17148768 | A | G | 0.140 | 0.030 | 2.20E-06 | 22.570 |
| genus | Sellimonas | rs2371573 | G | A | 0.119 | 0.025 | 2.54E-06 | 22.568 |
| genus | Ruminiclostridium6 | rs1377112 | C | T | 0.063 | 0.013 | 1.69E-06 | 22.568 |
| genus | ChristensenellaceaeR | rs1107331 | A | G | 0.055 | 0.012 | 2.04E-06 | 22.567 |
| genus | Bifidobacterium | rs75753154 | T | C | 0.076 | 0.016 | 2.36E-06 | 22.566 |
| genus | Intestinibacter | rs6823047 | A | C | 0.068 | 0.014 | 2.38E-06 | 22.566 |
| genus | Haemophilus | rs35510 | G | A | 0.128 | 0.027 | 2.20E-06 | 22.565 |
| genus | RuminococcaceaeUCG014 | rs995642 | T | C | 0.060 | 0.013 | 1.90E-06 | 22.562 |
| genus | Peptococcus | rs1530245 | A | G | -0.108 | 0.023 | 2.47E-06 | 22.561 |
| genus | Ruminococcus1 | rs895729 | G | A | -0.053 | 0.011 | 2.59E-06 | 22.561 |
| genus | RuminococcaceaeUCG011 | rs12744535 | A | G | -0.118 | 0.025 | 2.57E-06 | 22.560 |
| genus | RuminococcaceaeUCG003 | rs681343 | C | T | 0.056 | 0.012 | 2.33E-06 | 22.560 |
| genus | Bifidobacterium | rs7588568 | C | A | -0.062 | 0.013 | 2.32E-06 | 22.559 |
| genus | Peptococcus | rs6813111 | G | T | -0.108 | 0.023 | 2.46E-06 | 22.559 |
| genus | Bifidobacterium | rs112758679 | A | C | 0.076 | 0.016 | 2.25E-06 | 22.559 |
| genus | Butyrivibrio | rs9581731 | T | C | -0.124 | 0.026 | 2.03E-06 | 22.558 |
| genus | Bifidobacterium | rs60241067 | A | T | 0.063 | 0.013 | 3.30E-06 | 22.556 |
| genus | Butyrivibrio | rs7992720 | C | G | -0.124 | 0.026 | 2.03E-06 | 22.555 |
| genus | Blautia | rs66932009 | G | A | 0.059 | 0.012 | 2.08E-06 | 22.554 |
| genus | Lachnoclostridium | rs4814843 | T | C | -0.051 | 0.011 | 1.92E-06 | 22.554 |
| genus | RuminococcaceaeUCG002 | rs12033343 | C | G | -0.070 | 0.015 | 1.51E-06 | 22.551 |
| genus | Eisenbergiella | rs3812426 | A | G | 0.106 | 0.022 | 2.72E-06 | 22.551 |
| genus | LachnospiraceaeUCG001 | rs770063 | T | C | 0.089 | 0.019 | 3.22E-06 | 22.550 |
| genus | Desulfovibrio | rs13066142 | A | G | 0.119 | 0.025 | 3.79E-06 | 22.550 |
| genus | Butyrivibrio | rs445349 | G | A | -0.114 | 0.024 | 2.33E-06 | 22.550 |
| genus | Blautia | rs66876068 | A | G | 0.059 | 0.012 | 1.92E-06 | 22.549 |
| genus |  | rs7104872 | A | G | 0.139 | 0.029 | 2.73E-06 | 22.548 |
| genus | Adlercreutzia | rs2717140 | T | C | -0.119 | 0.025 | 2.05E-06 | 22.548 |
| genus |  | rs139756 | A | G | -0.082 | 0.017 | 2.06E-06 | 22.545 |
| genus | ChristensenellaceaeR | rs4805045 | T | C | 0.055 | 0.012 | 1.71E-06 | 22.544 |
| genus | Parasutterella | rs11931587 | T | C | 0.063 | 0.013 | 2.36E-06 | 22.543 |
| genus | Bifidobacterium | rs309167 | C | T | 0.077 | 0.016 | 2.70E-06 | 22.543 |
| genus | Butyrivibrio | rs9901771 | C | T | 0.123 | 0.026 | 1.78E-06 | 22.540 |
| genus | Paraprevotella | rs883630 | G | C | -0.083 | 0.018 | 3.02E-06 | 22.538 |
| genus | Veillonella | rs11203482 | A | G | -0.078 | 0.016 | 2.61E-06 | 22.537 |
| genus | RuminococcaceaeUCG011 | rs12757695 | C | T | -0.118 | 0.025 | 2.55E-06 | 22.537 |
| genus | RuminococcaceaeUCG011 | rs12756900 | G | A | -0.118 | 0.025 | 2.55E-06 | 22.537 |
| genus | RuminococcaceaeUCG011 | rs12738920 | T | G | -0.118 | 0.025 | 2.55E-06 | 22.537 |
| genus | Bifidobacterium | rs7593370 | C | T | 0.057 | 0.012 | 1.38E-06 | 22.536 |
| genus |  | rs4502131 | T | C | -0.162 | 0.034 | 2.72E-06 | 22.536 |
| genus | RuminococcaceaeUCG011 | rs11811198 | T | C | -0.118 | 0.025 | 2.56E-06 | 22.535 |
| genus |  | rs10798999 | T | C | 0.060 | 0.013 | 2.61E-06 | 22.532 |
| genus | Blautia | rs72607721 | C | A | 0.059 | 0.012 | 1.86E-06 | 22.532 |
| genus | Parasutterella | rs7695721 | C | G | 0.063 | 0.013 | 2.30E-06 | 22.529 |
| genus | Bilophila | rs1327489 | T | C | 0.081 | 0.017 | 1.90E-06 | 22.529 |
| genus | Ruminiclostridium9 | rs9852745 | G | A | -0.053 | 0.011 | 2.07E-06 | 22.526 |
| genus | Peptococcus | rs7033353 | G | T | 0.090 | 0.019 | 2.22E-06 | 22.525 |
| genus | Ruminococcus1 | rs11038608 | G | A | -0.055 | 0.012 | 2.74E-06 | 22.525 |
| genus | Butyrivibrio | rs6696235 | C | T | 0.187 | 0.039 | 2.60E-06 | 22.523 |
| genus | Peptococcus | rs12358717 | G | A | -0.226 | 0.048 | 6.53E-06 | 22.521 |
| genus | Ruminiclostridium9 | rs6771341 | A | G | -0.054 | 0.011 | 2.39E-06 | 22.517 |
| genus | Bifidobacterium | rs16833170 | A | G | 0.061 | 0.013 | 1.60E-06 | 22.516 |
| genus | Odoribacter | rs598729 | C | T | 0.055 | 0.012 | 2.16E-06 | 22.516 |
| genus | RuminococcaceaeUCG011 | rs10913770 | C | T | -0.118 | 0.025 | 2.59E-06 | 22.515 |
| genus | Paraprevotella | rs9531805 | T | C | -0.081 | 0.017 | 2.25E-06 | 22.515 |
| genus | Phascolarctobacterium | rs6427992 | C | G | -0.065 | 0.014 | 2.09E-06 | 22.514 |
| genus | Bifidobacterium | rs309142 | T | C | 0.073 | 0.015 | 2.42E-06 | 22.513 |
| genus | LachnospiraceaeUCG008 | rs11236216 | C | T | 0.084 | 0.018 | 2.35E-06 | 22.512 |
| genus | Alistipes | rs11771117 | G | C | -0.052 | 0.011 | 2.29E-06 | 22.511 |
| genus | Lachnoclostridium | rs6045996 | T | C | -0.051 | 0.011 | 2.43E-06 | 22.511 |
| genus | Bifidobacterium | rs7568884 | C | T | 0.061 | 0.013 | 1.62E-06 | 22.511 |
| genus | RuminococcaceaeUCG011 | rs4652379 | T | C | -0.118 | 0.025 | 2.59E-06 | 22.511 |
| genus | RuminococcaceaeUCG011 | rs4651026 | A | G | -0.118 | 0.025 | 2.59E-06 | 22.511 |
| genus | Allisonella | rs12553213 | G | T | 0.141 | 0.030 | 3.04E-06 | 22.508 |
| genus | Oxalobacter | rs36018452 | A | G | 0.201 | 0.042 | 2.78E-06 | 22.508 |
| genus | Peptococcus | rs6814531 | T | A | -0.108 | 0.023 | 2.55E-06 | 22.507 |
| genus | Ruminiclostridium9 | rs62289788 | T | C | -0.054 | 0.011 | 2.34E-06 | 22.507 |
| genus | Haemophilus | rs9574096 | T | A | -0.074 | 0.016 | 2.18E-06 | 22.504 |
| genus | Subdoligranulum | rs10065321 | C | T | -0.051 | 0.011 | 2.10E-06 | 22.503 |
| genus |  | rs7556718 | T | C | 0.126 | 0.027 | 1.71E-06 | 22.501 |
| genus | RuminococcaceaeUCG011 | rs4651027 | A | G | -0.118 | 0.025 | 2.61E-06 | 22.501 |
| genus | Allisonella | rs16919656 | G | T | 0.142 | 0.030 | 2.24E-06 | 22.500 |
| genus | RuminococcaceaeUCG005 | rs112014411 | T | G | 0.152 | 0.032 | 2.56E-06 | 22.497 |
| genus | Hungatella | rs34316562 | C | T | -0.159 | 0.034 | 3.22E-06 | 22.496 |
| genus | Parasutterella | rs2090816 | C | A | 0.084 | 0.018 | 2.90E-06 | 22.494 |
| genus | Ruminococcus2 | rs75140805 | G | T | 0.084 | 0.018 | 3.95E-06 | 22.493 |
| genus | RuminococcaceaeUCG011 | rs12744976 | C | T | -0.118 | 0.025 | 2.66E-06 | 22.486 |
| genus | Paraprevotella | rs9547279 | A | T | -0.105 | 0.022 | 1.04E-06 | 22.484 |
| genus | RuminococcaceaeUCG011 | rs4345774 | G | A | -0.118 | 0.025 | 2.62E-06 | 22.484 |
| genus | Erysipelatoclostridium | rs58236560 | T | G | -0.111 | 0.023 | 2.16E-06 | 22.483 |
| genus | Butyrivibrio | rs9581732 | G | A | -0.124 | 0.026 | 2.10E-06 | 22.483 |
| genus | Bifidobacterium | rs2839740 | G | T | 0.077 | 0.016 | 2.70E-06 | 22.480 |
| genus |  | rs34029790 | C | T | -0.162 | 0.034 | 2.86E-06 | 22.478 |
| genus | Dialister | rs4909362 | C | T | 0.063 | 0.013 | 1.63E-06 | 22.478 |
| genus | Peptococcus | rs13133889 | A | T | -0.108 | 0.023 | 2.59E-06 | 22.478 |
| genus | Ruminiclostridium5 | rs10827478 | G | T | -0.055 | 0.012 | 2.30E-06 | 22.478 |
| genus | Dorea | rs13279148 | A | G | 0.072 | 0.015 | 2.25E-06 | 22.477 |
| genus | RuminococcaceaeUCG011 | rs12729491 | C | A | -0.118 | 0.025 | 2.65E-06 | 22.473 |
| genus | Bifidobacterium | rs309159 | C | T | 0.076 | 0.016 | 2.15E-06 | 22.469 |
| genus |  | rs2229917 | G | A | 0.154 | 0.032 | 2.16E-06 | 22.467 |
| genus | LachnospiraceaeFCS020group | rs72793667 | G | A | -0.117 | 0.025 | 1.63E-06 | 22.467 |
| genus |  | rs329899 | T | C | -0.085 | 0.018 | 2.54E-06 | 22.467 |
| genus | Hungatella | rs13146247 | A | G | -0.159 | 0.033 | 3.40E-06 | 22.466 |
| genus | Ruminiclostridium5 | rs10773991 | C | T | -0.051 | 0.011 | 2.39E-06 | 22.463 |
| genus |  | rs773123 | A | T | 0.082 | 0.017 | 1.59E-06 | 22.462 |
| genus | RuminococcaceaeUCG011 | rs9662026 | G | T | -0.118 | 0.025 | 2.66E-06 | 22.460 |
| genus | RuminococcaceaeUCG011 | rs12409337 | G | A | -0.118 | 0.025 | 2.66E-06 | 22.460 |
| genus | RuminococcaceaeUCG011 | rs12408443 | T | C | -0.118 | 0.025 | 2.66E-06 | 22.460 |
| genus | RuminococcaceaeUCG011 | rs7554103 | A | G | -0.118 | 0.025 | 2.66E-06 | 22.460 |
| genus | RuminococcaceaeUCG011 | rs7513782 | T | C | -0.118 | 0.025 | 2.66E-06 | 22.460 |
| genus | Barnesiella | rs13242616 | C | T | -0.058 | 0.012 | 2.29E-06 | 22.459 |
| genus | RuminococcaceaeUCG011 | rs12755534 | G | T | -0.118 | 0.025 | 2.66E-06 | 22.459 |
| genus | Ruminiclostridium9 | rs7647356 | G | A | -0.054 | 0.011 | 2.38E-06 | 22.458 |
| genus | ChristensenellaceaeR | rs8106037 | C | G | 0.055 | 0.012 | 2.21E-06 | 22.458 |
| genus | Prevotella9 | rs746764 | C | T | -0.092 | 0.019 | 2.04E-06 | 22.457 |
| genus |  | rs492602 | A | G | -0.051 | 0.011 | 2.21E-06 | 22.455 |
| genus | Butyrivibrio | rs9581730 | T | C | -0.124 | 0.026 | 2.12E-06 | 22.454 |
| genus | RuminococcaceaeUCG011 | rs4484557 | C | T | -0.141 | 0.030 | 2.27E-06 | 22.453 |
| genus | RuminococcaceaeUCG002 | rs10927424 | T | G | -0.070 | 0.015 | 1.35E-06 | 22.449 |
| genus | LachnospiraceaeUCG008 | rs10833133 | A | C | -0.091 | 0.019 | 2.21E-06 | 22.447 |
| genus | Intestinimonas | rs9989886 | A | G | 0.079 | 0.017 | 1.98E-06 | 22.445 |
| genus | Streptococcus | rs10025825 | A | G | -0.091 | 0.019 | 9.56E-06 | 22.444 |
| genus | CandidatusSoleaferrea | rs791504 | T | A | 0.108 | 0.023 | 2.64E-06 | 22.440 |
| genus | Gordonibacter | rs322296 | A | G | 0.179 | 0.038 | 4.02E-06 | 22.439 |
| genus | Parasutterella | rs4342214 | G | C | 0.063 | 0.013 | 2.41E-06 | 22.438 |
| genus | RuminococcaceaeUCG011 | rs11577579 | A | C | -0.118 | 0.025 | 2.71E-06 | 22.437 |
| genus | RuminococcaceaeUCG011 | rs12401957 | A | C | -0.118 | 0.025 | 2.71E-06 | 22.437 |
| genus | RuminococcaceaeUCG005 | rs2893871 | A | G | -0.074 | 0.016 | 3.54E-06 | 22.435 |
| genus | Hungatella | rs13122587 | T | C | -0.159 | 0.033 | 3.47E-06 | 22.433 |
| genus | Butyricimonas | rs782080 | A | T | 0.065 | 0.014 | 2.23E-06 | 22.433 |
| genus | Allisonella | rs10981444 | A | C | 0.141 | 0.030 | 3.26E-06 | 22.432 |
| genus | Anaerotruncus | rs9603206 | T | G | 0.078 | 0.017 | 1.17E-06 | 22.431 |
| genus | Sutterella | rs13173038 | G | A | -0.072 | 0.015 | 2.73E-06 | 22.428 |
| genus | Adlercreutzia | rs7656076 | G | C | -0.084 | 0.018 | 2.43E-06 | 22.427 |
| genus | Akkermansia | rs117107102 | G | A | 0.204 | 0.043 | 3.01E-06 | 22.427 |
| genus | ErysipelotrichaceaeUCG003 | rs10502864 | C | A | -0.083 | 0.018 | 3.55E-06 | 22.426 |
| genus | Oscillibacter | rs16866406 | G | A | 0.099 | 0.021 | 3.08E-06 | 22.426 |
| genus | Alistipes | rs11773813 | C | A | -0.052 | 0.011 | 2.42E-06 | 22.424 |
| genus | Ruminiclostridium9 | rs17630086 | C | T | -0.053 | 0.011 | 2.46E-06 | 22.422 |
| genus | Peptococcus | rs7660438 | C | G | -0.107 | 0.023 | 2.72E-06 | 22.421 |
| genus | Ruminiclostridium9 | rs7612520 | T | G | -0.054 | 0.011 | 2.42E-06 | 22.421 |
| genus | RuminococcaceaeUCG011 | rs58836785 | T | C | -0.118 | 0.025 | 2.83E-06 | 22.417 |
| genus | LachnospiraceaeUCG008 | rs12047903 | T | G | -0.115 | 0.024 | 2.11E-06 | 22.417 |
| genus | RuminococcaceaeUCG011 | rs4652370 | G | A | -0.118 | 0.025 | 2.68E-06 | 22.417 |
| genus | Alloprevotella | rs1896135 | A | G | 0.138 | 0.029 | 4.01E-06 | 22.416 |
| genus | LachnospiraceaeUCG008 | rs72958924 | A | G | -0.115 | 0.024 | 1.98E-06 | 22.415 |
| genus | Parasutterella | rs6846697 | A | G | 0.063 | 0.013 | 2.49E-06 | 22.414 |
| genus | RuminococcaceaeUCG011 | rs12525814 | A | G | -0.140 | 0.030 | 2.09E-06 | 22.414 |
| genus | Streptococcus | rs7916711 | G | A | 0.103 | 0.022 | 2.72E-06 | 22.407 |
| genus | Parasutterella | rs12498849 | A | G | 0.063 | 0.013 | 2.54E-06 | 22.404 |
| genus | Bifidobacterium | rs10197646 | G | A | 0.073 | 0.015 | 6.44E-06 | 22.403 |
| genus | FamilyXIIIUCG001 | rs2059580 | C | A | -0.064 | 0.013 | 2.29E-06 | 22.402 |
| genus | Ruminiclostridium9 | rs6808527 | A | G | -0.053 | 0.011 | 2.38E-06 | 22.401 |
| genus | Ruminiclostridium9 | rs62286615 | G | A | -0.053 | 0.011 | 2.39E-06 | 22.401 |
| genus | Bifidobacterium | rs1992764 | C | T | 0.057 | 0.012 | 1.90E-06 | 22.400 |
| genus | Parasutterella | rs35306122 | T | G | 0.063 | 0.013 | 2.50E-06 | 22.398 |
| genus | Subdoligranulum | rs4688291 | C | T | 0.051 | 0.011 | 2.01E-06 | 22.398 |
| genus | Intestinimonas | rs56186705 | T | A | 0.064 | 0.013 | 1.79E-06 | 22.397 |
| genus | Slackia | rs12440440 | G | A | 0.090 | 0.019 | 2.63E-06 | 22.397 |
| genus | Ruminiclostridium6 | rs1377105 | T | C | 0.073 | 0.015 | 2.68E-06 | 22.395 |
| genus | RuminococcaceaeUCG003 | rs11637418 | T | C | -0.062 | 0.013 | 2.46E-06 | 22.394 |
| genus | CandidatusSoleaferrea | rs6665956 | T | A | 0.110 | 0.023 | 2.40E-06 | 22.392 |
| genus | Ruminococcus1 | rs7102941 | T | C | 0.081 | 0.017 | 1.21E-06 | 22.391 |
| genus | RuminococcaceaeUCG011 | rs12744438 | G | A | -0.118 | 0.025 | 2.70E-06 | 22.391 |
| genus | RuminococcaceaeUCG011 | rs4233179 | G | T | -0.118 | 0.025 | 2.70E-06 | 22.391 |
| genus | RuminococcaceaeUCG011 | rs4233178 | T | C | -0.118 | 0.025 | 2.70E-06 | 22.391 |
| genus | RuminococcaceaeUCG011 | rs12726875 | A | G | -0.118 | 0.025 | 2.70E-06 | 22.391 |
| genus | RuminococcaceaeUCG011 | rs11807559 | C | G | -0.118 | 0.025 | 2.70E-06 | 22.391 |
| genus | Parasutterella | rs5005431 | T | C | 0.063 | 0.013 | 2.59E-06 | 22.390 |
| genus | Oscillibacter | rs2288569 | C | T | 0.099 | 0.021 | 3.16E-06 | 22.387 |
| genus | Paraprevotella | rs17785622 | G | A | 0.248 | 0.052 | 1.93E-06 | 22.385 |
| genus | ChristensenellaceaeR | rs4805044 | G | A | 0.055 | 0.012 | 2.30E-06 | 22.385 |
| genus | Ruminiclostridium6 | rs10495465 | A | G | 0.115 | 0.024 | 2.21E-06 | 22.385 |
| genus | RuminococcaceaeUCG011 | rs7513973 | T | A | -0.118 | 0.025 | 2.77E-06 | 22.384 |
| genus |  | rs10241138 | T | C | 0.078 | 0.016 | 1.39E-06 | 22.384 |
| genus | Desulfovibrio | rs6750821 | T | C | 0.106 | 0.022 | 2.18E-06 | 22.383 |
| genus | RuminococcaceaeUCG011 | rs7551894 | C | G | -0.118 | 0.025 | 2.77E-06 | 22.381 |
| genus | LachnospiraceaeUCG008 | rs13024781 | C | T | -0.080 | 0.017 | 2.29E-06 | 22.380 |
| genus | Slackia | rs112764253 | A | T | 0.195 | 0.041 | 3.40E-06 | 22.379 |
| genus | Streptococcus | rs1601915 | C | T | -0.086 | 0.018 | 2.21E-06 | 22.376 |
| genus | Blautia | rs66908300 | C | T | 0.058 | 0.012 | 1.76E-06 | 22.374 |
| genus | Butyrivibrio | rs7763512 | A | G | 0.120 | 0.025 | 3.11E-06 | 22.374 |
| genus | RuminococcaceaeUCG011 | rs11803247 | G | C | -0.118 | 0.025 | 2.75E-06 | 22.374 |
| genus | RuminococcaceaeUCG014 | rs1476123 | C | G | 0.057 | 0.012 | 2.44E-06 | 22.373 |
| genus | RuminococcaceaeUCG009 | rs61779334 | C | G | -0.138 | 0.029 | 1.94E-06 | 22.369 |
| genus | Ruminiclostridium9 | rs6776225 | G | A | -0.053 | 0.011 | 2.50E-06 | 22.367 |
| genus | Ruminiclostridium5 | rs4765844 | A | G | -0.051 | 0.011 | 2.60E-06 | 22.366 |
| genus | Butyrivibrio | rs12745118 | G | A | 0.176 | 0.037 | 3.02E-06 | 22.366 |
| genus |  | rs2039977 | T | C | -0.064 | 0.014 | 1.40E-06 | 22.366 |
| genus | Ruminiclostridium5 | rs7294668 | T | C | -0.051 | 0.011 | 2.34E-06 | 22.364 |
| genus | Alistipes | rs10950069 | C | T | -0.052 | 0.011 | 2.46E-06 | 22.364 |
| genus | RuminococcaceaeUCG011 | rs11803938 | G | A | -0.118 | 0.025 | 2.66E-06 | 22.362 |
| genus | Butyrivibrio | rs11153293 | T | A | -0.113 | 0.024 | 2.55E-06 | 22.360 |
| genus | Butyrivibrio | rs11756643 | T | C | -0.113 | 0.024 | 2.55E-06 | 22.360 |
| genus | RuminococcaceaeUCG002 | rs11749444 | T | C | -0.057 | 0.012 | 2.50E-06 | 22.360 |
| genus | Hungatella | rs67786105 | C | T | -0.158 | 0.033 | 3.57E-06 | 22.359 |
| genus |  | rs55932844 | G | A | -0.171 | 0.036 | 2.39E-06 | 22.357 |
| genus | Butyrivibrio | rs459403 | C | T | -0.113 | 0.024 | 2.57E-06 | 22.357 |
| genus | Hungatella | rs35397148 | A | T | -0.158 | 0.033 | 3.53E-06 | 22.357 |
| genus |  | rs3771391 | C | T | 0.126 | 0.027 | 1.82E-06 | 22.356 |
| genus |  | rs10130434 | G | T | -0.161 | 0.034 | 3.20E-06 | 22.355 |
| genus | Coprococcus1 | rs1010560 | A | C | 0.058 | 0.012 | 1.96E-06 | 22.354 |
| genus | RuminococcaceaeUCG011 | rs11801919 | A | T | -0.118 | 0.025 | 2.66E-06 | 22.353 |
| genus | RuminococcaceaeUCG011 | rs11803484 | G | T | -0.118 | 0.025 | 2.66E-06 | 22.353 |
| genus | RuminococcaceaeUCG011 | rs12757910 | T | A | -0.118 | 0.025 | 2.66E-06 | 22.353 |
| genus | Peptococcus | rs4035124 | T | A | 0.153 | 0.032 | 1.98E-06 | 22.352 |
| genus | Oxalobacter | rs6071442 | C | T | -0.099 | 0.021 | 2.55E-06 | 22.351 |
| genus | Parasutterella | rs55658595 | A | T | 0.063 | 0.013 | 2.59E-06 | 22.351 |
| genus | ChristensenellaceaeR | rs1812308 | T | C | 0.055 | 0.012 | 2.33E-06 | 22.351 |
| genus | RuminococcaceaeUCG011 | rs4651028 | A | G | -0.118 | 0.025 | 2.84E-06 | 22.349 |
| genus | Odoribacter | rs77155862 | G | T | -0.129 | 0.027 | 2.16E-06 | 22.346 |
| genus |  | rs1391597 | T | C | 0.059 | 0.012 | 1.86E-06 | 22.345 |
| genus | RuminococcaceaeUCG011 | rs12750591 | C | G | -0.118 | 0.025 | 2.82E-06 | 22.345 |
| genus | LachnospiraceaeUCG008 | rs10766569 | T | C | -0.090 | 0.019 | 2.14E-06 | 22.345 |
| genus |  | rs10950614 | C | G | -0.077 | 0.016 | 1.26E-06 | 22.344 |
| genus | Bifidobacterium | rs113974791 | T | G | 0.075 | 0.016 | 2.54E-06 | 22.343 |
| genus | Paraprevotella | rs2041113 | C | T | -0.082 | 0.017 | 3.59E-06 | 22.343 |
| genus | Butyricimonas | rs251174 | C | T | 0.069 | 0.015 | 1.75E-06 | 22.343 |
| genus | LachnospiraceaeUCG010 | rs74315802 | T | G | 0.087 | 0.018 | 3.19E-06 | 22.343 |
| genus | Oscillibacter | rs4981911 | A | G | -0.073 | 0.016 | 2.28E-06 | 22.343 |
| genus | Lactobacillus | rs1590596 | G | C | -0.105 | 0.022 | 4.01E-06 | 22.342 |
| genus | Anaerotruncus | rs9594210 | C | T | 0.078 | 0.017 | 1.32E-06 | 22.342 |
| genus | RuminococcaceaeUCG014 | rs13062677 | G | A | 0.057 | 0.012 | 2.46E-06 | 22.341 |
| genus | LachnospiraceaeUCG001 | rs417387 | C | T | 0.067 | 0.014 | 2.28E-06 | 22.341 |
| genus | Victivallis | rs11899949 | A | G | 0.131 | 0.028 | 2.77E-06 | 22.341 |
| genus | Flavonifractor | rs12790684 | G | A | 0.076 | 0.016 | 1.85E-06 | 22.340 |
| genus | RuminococcaceaeUCG011 | rs12751244 | C | T | -0.118 | 0.025 | 2.78E-06 | 22.340 |
| genus | RuminococcaceaeUCG011 | rs12407502 | T | C | -0.118 | 0.025 | 2.87E-06 | 22.339 |
| genus | LachnospiraceaeUCG001 | rs1492259 | A | G | 0.073 | 0.015 | 2.52E-06 | 22.339 |
| genus | Holdemanella | rs75764681 | C | T | -0.283 | 0.060 | 1.94E-06 | 22.338 |
| genus | Parasutterella | rs5005433 | T | C | 0.063 | 0.013 | 2.65E-06 | 22.337 |
| genus | Odoribacter | rs3850601 | A | T | -0.130 | 0.027 | 2.17E-06 | 22.336 |
| genus | Ruminiclostridium9 | rs56089505 | T | A | -0.053 | 0.011 | 2.46E-06 | 22.335 |
| genus | Slackia | rs4492265 | G | A | -0.091 | 0.019 | 2.41E-06 | 22.334 |
| genus | Parasutterella | rs62313332 | C | G | 0.063 | 0.013 | 2.49E-06 | 22.334 |
| genus | Catenibacterium | rs212393 | A | G | -0.135 | 0.029 | 3.62E-06 | 22.332 |
| genus | Bifidobacterium | rs895285 | A | G | 0.077 | 0.016 | 2.65E-06 | 22.332 |
| genus |  | rs2973294 | T | G | 0.092 | 0.020 | 2.39E-06 | 22.331 |
| genus | FamilyXIIIAD3011group | rs2999589 | T | G | -0.057 | 0.012 | 2.40E-06 | 22.328 |
| genus | Holdemania | rs7941067 | T | G | 0.075 | 0.016 | 3.06E-06 | 22.328 |
| genus | RuminococcaceaeUCG003 | rs67401159 | A | G | -0.106 | 0.022 | 3.97E-06 | 22.327 |
| genus | RuminococcaceaeUCG011 | rs12748264 | T | C | -0.118 | 0.025 | 2.82E-06 | 22.327 |
| genus | Prevotella9 | rs117271932 | G | A | 0.208 | 0.044 | 2.82E-06 | 22.326 |
| genus | RuminococcaceaeUCG003 | rs602662 | G | A | 0.056 | 0.012 | 2.62E-06 | 22.326 |
| genus | LachnospiraceaeUCG008 | rs29326 | C | T | 0.080 | 0.017 | 2.63E-06 | 22.326 |
| genus |  | rs10950613 | G | A | -0.077 | 0.016 | 1.28E-06 | 22.325 |
| genus | ChristensenellaceaeR | rs8107807 | T | C | 0.055 | 0.012 | 2.37E-06 | 22.323 |
| genus |  | rs380743 | G | A | -0.051 | 0.011 | 1.99E-06 | 22.323 |
| genus | Ruminiclostridium6 | rs2045348 | T | A | 0.113 | 0.024 | 2.48E-06 | 22.323 |
| genus | RuminococcaceaeUCG014 | . | G | T | 0.126 | 0.027 | 3.02E-06 | 22.321 |
| genus | RuminococcaceaeUCG002 | rs10916131 | T | C | -0.069 | 0.015 | 2.87E-06 | 22.321 |
| genus | Akkermansia | rs112769434 | A | G | 0.112 | 0.024 | 1.96E-06 | 22.320 |
| genus | Ruminiclostridium5 | rs9300298 | A | T | -0.051 | 0.011 | 2.43E-06 | 22.317 |
| genus | Hungatella | rs4691168 | T | C | -0.158 | 0.033 | 3.64E-06 | 22.316 |
| genus | RuminococcaceaeUCG011 | rs12727704 | A | T | -0.118 | 0.025 | 2.87E-06 | 22.313 |
| genus | Intestinibacter | rs4937041 | T | C | -0.140 | 0.030 | 2.49E-06 | 22.313 |
| genus | Victivallis | rs4895919 | C | T | -0.117 | 0.025 | 2.75E-06 | 22.313 |
| genus | RuminococcaceaeUCG011 | rs10913755 | A | C | -0.118 | 0.025 | 2.87E-06 | 22.311 |
| genus |  | rs16884680 | T | G | -0.091 | 0.019 | 1.74E-06 | 22.309 |
| genus | Marvinbryantia | rs117634921 | G | T | 0.117 | 0.025 | 4.32E-06 | 22.306 |
| genus | Bifidobacterium | rs1057031 | G | A | 0.075 | 0.016 | 2.83E-06 | 22.303 |
| genus | Bifidobacterium | rs12472293 | T | C | 0.075 | 0.016 | 2.62E-06 | 22.298 |
| genus | Lachnoclostridium | rs6106066 | G | A | -0.050 | 0.011 | 2.39E-06 | 22.298 |
| genus | Peptococcus | rs317069 | G | A | -0.104 | 0.022 | 3.17E-06 | 22.298 |
| genus | RuminococcaceaeUCG002 | rs11746143 | G | A | -0.058 | 0.012 | 2.57E-06 | 22.298 |
| genus | Alistipes | rs10950070 | G | A | -0.052 | 0.011 | 2.58E-06 | 22.297 |
| genus | Tyzzerella3 | rs11133845 | A | G | -0.103 | 0.022 | 1.76E-06 | 22.297 |
| genus | LachnospiraceaeUCG001 | rs168207 | A | G | 0.067 | 0.014 | 2.77E-06 | 22.296 |
| genus | RikenellaceaeRC9gutgroup | rs2479314 | T | C | -0.180 | 0.038 | 1.32E-06 | 22.293 |
| genus | RuminococcaceaeUCG004 | rs12129927 | G | A | 0.091 | 0.019 | 2.61E-06 | 22.292 |
| genus | LachnospiraceaeUCG001 | rs7618566 | C | T | 0.086 | 0.018 | 3.31E-06 | 22.291 |
| genus | Allisonella | rs7034449 | A | G | 0.141 | 0.030 | 3.35E-06 | 22.289 |
| genus | Paraprevotella | rs883629 | T | C | -0.083 | 0.018 | 3.45E-06 | 22.286 |
| genus |  | rs10950616 | T | C | -0.077 | 0.016 | 1.45E-06 | 22.286 |
| genus | RuminococcaceaeUCG011 | rs36046838 | T | A | -0.118 | 0.025 | 2.90E-06 | 22.285 |
| genus | Bifidobacterium | rs150183636 | C | T | 0.096 | 0.020 | 6.02E-06 | 22.282 |
| genus | Romboutsia | rs61841447 | T | C | 0.075 | 0.016 | 2.61E-06 | 22.278 |
| genus | Ruminococcus1 | rs17781867 | T | C | 0.100 | 0.021 | 1.96E-06 | 22.275 |
| genus | RuminococcaceaeUCG011 | rs10913754 | C | T | -0.118 | 0.025 | 2.95E-06 | 22.273 |
| genus | RuminococcaceaeUCG011 | rs10913752 | G | A | -0.118 | 0.025 | 2.95E-06 | 22.273 |
| genus | RuminococcaceaeUCG009 | rs758191 | G | T | 0.177 | 0.038 | 9.01E-06 | 22.270 |
| genus | Alistipes | rs4068841 | C | G | -0.052 | 0.011 | 2.55E-06 | 22.264 |
| genus | Anaerostipes | rs12259533 | C | G | -0.052 | 0.011 | 2.65E-06 | 22.263 |
| genus | Peptococcus | rs2756907 | C | T | 0.090 | 0.019 | 2.53E-06 | 22.260 |
| genus | Anaerostipes | rs62215703 | A | G | 0.064 | 0.014 | 1.98E-06 | 22.260 |
| genus | Methanobrevibacter | rs10202904 | G | T | -0.113 | 0.024 | 3.09E-06 | 22.260 |
| genus | Marvinbryantia | rs113508927 | C | T | -0.073 | 0.016 | 2.85E-06 | 22.259 |
| genus | RuminococcaceaeUCG011 | rs6666939 | G | T | -0.118 | 0.025 | 2.90E-06 | 22.259 |
| genus | Coprococcus1 | rs73031725 | C | T | 0.168 | 0.036 | 1.98E-06 | 22.259 |
| genus | Streptococcus | rs1863906 | A | C | -0.085 | 0.018 | 3.86E-06 | 22.257 |
| genus | RuminococcaceaeUCG010 | rs682403 | G | A | -0.059 | 0.012 | 2.37E-06 | 22.257 |
| genus |  | rs11508469 | G | A | -0.076 | 0.016 | 1.33E-06 | 22.252 |
| genus | Anaerostipes | rs12259535 | A | G | -0.052 | 0.011 | 2.67E-06 | 22.252 |
| genus | RuminococcaceaeUCG011 | rs12747626 | T | C | -0.118 | 0.025 | 2.91E-06 | 22.251 |
| genus | Bifidobacterium | rs13424016 | G | A | 0.057 | 0.012 | 2.21E-06 | 22.251 |
| genus | Akkermansia | rs12908520 | A | G | 0.062 | 0.013 | 2.26E-06 | 22.251 |
| genus |  | rs6425850 | T | G | 0.063 | 0.013 | 2.71E-06 | 22.250 |
| genus | Butyricimonas | rs867047 | G | T | 0.067 | 0.014 | 1.96E-06 | 22.249 |
| genus | Lachnoclostridium | rs512541 | G | A | 0.050 | 0.011 | 3.21E-06 | 22.247 |
| genus | Ruminiclostridium9 | rs7612250 | G | T | -0.053 | 0.011 | 2.68E-06 | 22.243 |
| genus | Senegalimassilia | rs34653493 | C | T | 0.081 | 0.017 | 3.01E-06 | 22.242 |
| genus | Blautia | rs72973581 | G | A | 0.125 | 0.027 | 1.74E-06 | 22.242 |
| genus | Akkermansia | rs56161035 | C | T | 0.068 | 0.014 | 2.37E-06 | 22.237 |
| genus | Actinomyces | rs34583783 | T | G | 0.127 | 0.027 | 4.49E-06 | 22.237 |
| genus | Bifidobacterium | rs13415439 | C | A | 0.061 | 0.013 | 1.86E-06 | 22.233 |
| genus | Ruminiclostridium5 | rs10430574 | T | C | -0.054 | 0.011 | 2.21E-06 | 22.230 |
| genus | RuminococcaceaeUCG011 | rs10913769 | G | T | -0.117 | 0.025 | 3.03E-06 | 22.229 |
| genus | Adlercreutzia | rs9490822 | T | C | -0.073 | 0.016 | 2.54E-06 | 22.229 |
| genus | LachnospiraceaeUCG008 | rs10741776 | G | A | -0.093 | 0.020 | 2.78E-06 | 22.229 |
| genus | Bifidobacterium | rs16832162 | T | A | 0.075 | 0.016 | 2.79E-06 | 22.228 |
| genus | Oscillospira | rs1954532 | C | T | -0.083 | 0.018 | 2.27E-06 | 22.228 |
| genus | Roseburia | rs2034590 | C | T | 0.058 | 0.012 | 3.51E-06 | 22.225 |
| genus | Bifidobacterium | rs309171 | G | C | 0.075 | 0.016 | 2.76E-06 | 22.219 |
| genus | Sutterella | rs7499539 | G | A | 0.062 | 0.013 | 2.36E-06 | 22.218 |
| genus | RuminococcaceaeUCG011 | rs11804154 | T | G | -0.118 | 0.025 | 2.99E-06 | 22.215 |
| genus | RuminococcaceaeUCG011 | rs11805235 | G | C | -0.118 | 0.025 | 2.99E-06 | 22.215 |
| genus | Flavonifractor | rs12790857 | G | A | 0.076 | 0.016 | 1.99E-06 | 22.212 |
| genus | Prevotella9 | rs113200445 | C | A | -0.091 | 0.019 | 2.16E-06 | 22.211 |
| genus | Allisonella | rs6742198 | A | G | 0.149 | 0.032 | 3.35E-06 | 22.210 |
| genus | Peptococcus | rs2889084 | A | G | -0.107 | 0.023 | 3.11E-06 | 22.210 |
| genus |  | rs7586455 | G | A | 0.126 | 0.027 | 1.94E-06 | 22.209 |
| genus | Butyrivibrio | rs7780509 | G | T | 0.150 | 0.032 | 3.42E-06 | 22.209 |
| genus | Oscillibacter | rs4981912 | C | G | -0.073 | 0.016 | 2.45E-06 | 22.207 |
| genus | LachnospiraceaeUCG010 | rs12346653 | T | C | 0.066 | 0.014 | 2.70E-06 | 22.205 |
| genus | Hungatella | rs67304281 | C | G | -0.158 | 0.033 | 3.83E-06 | 22.205 |
| genus | Odoribacter | rs17103691 | A | G | -0.127 | 0.027 | 2.69E-06 | 22.205 |
| genus | Sellimonas | rs2623258 | A | T | -0.121 | 0.026 | 2.83E-06 | 22.204 |
| genus | Peptococcus | rs12331409 | C | A | -0.107 | 0.023 | 3.13E-06 | 22.203 |
| genus | Butyrivibrio | rs7451051 | A | G | -0.113 | 0.024 | 2.80E-06 | 22.203 |
| genus | Sutterella | rs9527759 | T | C | -0.059 | 0.012 | 2.26E-06 | 22.203 |
| genus | Parasutterella | rs4852276 | T | C | 0.063 | 0.013 | 2.69E-06 | 22.202 |
| genus | Anaerotruncus | rs1272208 | T | G | -0.061 | 0.013 | 4.28E-06 | 22.202 |
| genus | Peptococcus | rs6836511 | A | G | -0.107 | 0.023 | 2.99E-06 | 22.200 |
| genus | Romboutsia | rs41289305 | T | C | 0.075 | 0.016 | 2.77E-06 | 22.200 |
| genus | Ruminiclostridium9 | rs62287861 | G | A | -0.055 | 0.012 | 2.97E-06 | 22.199 |
| genus | LachnospiraceaeNK4A136group | rs7832116 | G | A | -0.071 | 0.015 | 3.57E-06 | 22.199 |
| genus | Bifidobacterium | rs16832624 | T | C | 0.064 | 0.014 | 2.85E-06 | 22.198 |
| genus | LachnospiraceaeFCS020group | rs168737 | A | G | 0.122 | 0.026 | 5.05E-06 | 22.195 |
| genus | Allisonella | rs4743061 | T | C | 0.144 | 0.031 | 2.91E-06 | 22.195 |
| genus | Bifidobacterium | rs6750549 | A | G | 0.076 | 0.016 | 2.94E-06 | 22.194 |
| genus | Bacteroides | rs17619981 | G | T | 0.088 | 0.019 | 2.69E-06 | 22.194 |
| genus | Peptococcus | rs9299351 | C | T | 0.090 | 0.019 | 2.62E-06 | 22.193 |
| genus | RuminococcaceaeUCG011 | rs11802637 | G | A | -0.117 | 0.025 | 3.06E-06 | 22.193 |
| genus | Coprobacter | rs213863 | T | C | -0.089 | 0.019 | 2.35E-06 | 22.192 |
| genus | Alistipes | rs1595991 | T | C | -0.051 | 0.011 | 2.53E-06 | 22.191 |
| genus | Tyzzerella3 | rs77183374 | C | T | -0.130 | 0.027 | 4.45E-06 | 22.190 |
| genus | Hungatella | rs35742826 | C | T | -0.158 | 0.033 | 3.91E-06 | 22.190 |
| genus |  | rs2973293 | C | G | 0.092 | 0.020 | 2.58E-06 | 22.190 |
| genus | RuminococcaceaeUCG011 | rs11803781 | A | G | -0.118 | 0.025 | 3.01E-06 | 22.190 |
| genus | Intestinimonas | rs10262702 | C | T | 0.092 | 0.019 | 2.06E-06 | 22.189 |
| genus | Ruminiclostridium9 | rs62286610 | G | T | -0.053 | 0.011 | 2.66E-06 | 22.188 |
| genus | Lachnoclostridium | rs6045980 | C | T | -0.051 | 0.011 | 2.55E-06 | 22.186 |
| genus |  | rs34157616 | C | T | 0.067 | 0.014 | 2.92E-06 | 22.185 |
| genus | Allisonella | rs7020530 | G | A | 0.141 | 0.030 | 3.51E-06 | 22.184 |
| genus | Lachnoclostridium | rs4813353 | C | T | -0.051 | 0.011 | 2.48E-06 | 22.184 |
| genus | Butyrivibrio | rs4276544 | G | A | -0.113 | 0.024 | 2.79E-06 | 22.183 |
| genus | RuminococcaceaeUCG002 | rs62374285 | G | A | -0.057 | 0.012 | 3.03E-06 | 22.180 |
| genus | LachnospiraceaeUCG008 | rs7928986 | C | G | -0.091 | 0.019 | 2.83E-06 | 22.179 |
| genus |  | rs112617308 | C | T | -0.171 | 0.036 | 2.38E-06 | 22.177 |
| genus |  | rs12155021 | A | T | -0.077 | 0.016 | 1.61E-06 | 22.177 |
| genus | RuminococcaceaeUCG011 | rs12753162 | T | C | -0.118 | 0.025 | 3.02E-06 | 22.177 |
| genus | RuminococcaceaeUCG011 | rs12729623 | C | G | -0.118 | 0.025 | 3.02E-06 | 22.177 |
| genus | Bifidobacterium | rs6746633 | G | A | 0.096 | 0.020 | 6.19E-06 | 22.176 |
| genus | Paraprevotella | rs9547275 | G | A | -0.104 | 0.022 | 1.25E-06 | 22.175 |
| genus | Ruminiclostridium9 | rs62286616 | A | C | -0.053 | 0.011 | 2.79E-06 | 22.173 |
| genus | Oxalobacter | rs10780690 | G | T | 0.100 | 0.021 | 2.56E-06 | 22.173 |
| genus | RuminococcaceaeUCG003 | rs4452755 | C | A | -0.063 | 0.013 | 3.29E-06 | 22.172 |
| genus | Fusicatenibacter | rs2025938 | A | G | -0.097 | 0.021 | 2.99E-06 | 22.172 |
| genus |  | rs13013342 | T | C | 0.126 | 0.027 | 1.97E-06 | 22.169 |
| genus |  | rs289410 | A | G | -0.065 | 0.014 | 2.27E-06 | 22.168 |
| genus | Ruminococcus1 | rs1026078 | T | C | -0.052 | 0.011 | 2.35E-06 | 22.165 |
| genus | Alistipes | rs4717372 | C | G | -0.051 | 0.011 | 2.73E-06 | 22.165 |
| genus | ChristensenellaceaeR | rs8182625 | G | A | 0.054 | 0.012 | 2.54E-06 | 22.165 |
| genus | Bifidobacterium | rs309120 | G | C | 0.074 | 0.016 | 2.38E-06 | 22.164 |
| genus | RuminococcaceaeUCG011 | rs12727075 | A | G | -0.117 | 0.025 | 3.11E-06 | 22.161 |
| genus | Bifidobacterium | rs59869505 | A | C | 0.061 | 0.013 | 1.90E-06 | 22.160 |
| genus | Ruminococcus2 | rs7570274 | C | G | 0.066 | 0.014 | 2.56E-06 | 22.158 |
| genus | Bifidobacterium | rs309166 | C | T | 0.074 | 0.016 | 2.67E-06 | 22.157 |
| genus | Oxalobacter | rs62281780 | T | C | 0.161 | 0.034 | 3.66E-06 | 22.157 |
| genus | Peptococcus | rs6856308 | G | A | -0.107 | 0.023 | 3.04E-06 | 22.154 |
| genus | Intestinimonas | rs12566247 | A | T | 0.064 | 0.014 | 2.19E-06 | 22.153 |
| genus | LachnospiraceaeUCG008 | rs10751237 | C | A | -0.082 | 0.017 | 3.17E-06 | 22.153 |
| genus | LachnospiraceaeFCS020group | rs10418670 | A | G | 0.063 | 0.013 | 2.68E-06 | 22.153 |
| genus | LachnospiraceaeNK4A136group | rs36018216 | G | A | -0.054 | 0.011 | 3.50E-06 | 22.153 |
| genus | RuminococcaceaeUCG011 | rs4652375 | A | G | -0.117 | 0.025 | 3.11E-06 | 22.151 |
| genus | LachnospiraceaeFCS020group | rs2322265 | T | C | -0.067 | 0.014 | 5.21E-06 | 22.149 |
| genus |  | rs35278384 | A | G | 0.067 | 0.014 | 3.03E-06 | 22.148 |
| genus | LachnospiraceaeUCG001 | rs10815579 | C | A | -0.068 | 0.014 | 2.38E-06 | 22.147 |
| genus | ChristensenellaceaeR | rs10426071 | G | A | 0.054 | 0.012 | 2.57E-06 | 22.147 |
| genus | Allisonella | rs4743069 | A | C | 0.141 | 0.030 | 3.45E-06 | 22.146 |
| genus | RuminococcaceaeUCG011 | rs12636310 | A | G | 0.133 | 0.028 | 2.81E-06 | 22.146 |
| genus | Oxalobacter | rs761575 | T | C | -0.100 | 0.021 | 2.72E-06 | 22.144 |
| genus | Romboutsia | rs61841445 | G | C | 0.075 | 0.016 | 2.81E-06 | 22.142 |
| genus | RuminococcaceaeUCG011 | rs12757708 | T | G | -0.118 | 0.025 | 2.97E-06 | 22.139 |
| genus | ChristensenellaceaeR | rs1812306 | C | A | 0.054 | 0.012 | 2.60E-06 | 22.139 |
| genus | RuminococcaceaeUCG011 | rs7550736 | C | T | -0.117 | 0.025 | 3.16E-06 | 22.139 |
| genus | Anaerotruncus | rs9603207 | T | A | 0.078 | 0.017 | 1.45E-06 | 22.139 |
| genus | Dialister | rs10086221 | T | A | 0.062 | 0.013 | 2.00E-06 | 22.138 |
| genus | RuminococcaceaeUCG013 | rs12189346 | A | G | 0.068 | 0.015 | 1.68E-06 | 22.137 |
| genus | Butyrivibrio | rs12943847 | A | C | 0.126 | 0.027 | 2.28E-06 | 22.137 |
| genus | Bifidobacterium | rs78364332 | T | C | 0.075 | 0.016 | 3.06E-06 | 22.135 |
| genus | Parasutterella | rs2797668 | T | C | 0.083 | 0.018 | 2.97E-06 | 22.134 |
| genus | Ruminiclostridium9 | rs62289789 | C | T | -0.053 | 0.011 | 2.84E-06 | 22.133 |
| genus | Anaerotruncus | rs8005030 | T | C | 0.055 | 0.012 | 2.28E-06 | 22.132 |
| genus | Akkermansia | rs1491244 | A | T | 0.111 | 0.024 | 2.25E-06 | 22.132 |
| genus | Paraprevotella | rs1928141 | A | G | -0.080 | 0.017 | 3.00E-06 | 22.131 |
| genus | Prevotella7 | rs9608249 | G | A | -0.158 | 0.034 | 2.07E-06 | 22.130 |
| genus | Oxalobacter | rs73027387 | A | G | 0.152 | 0.032 | 4.07E-06 | 22.130 |
| genus | Ruminiclostridium9 | rs62286618 | G | A | -0.053 | 0.011 | 2.77E-06 | 22.129 |
| genus | Ruminiclostridium5 | rs113753996 | C | T | 0.082 | 0.017 | 3.99E-06 | 22.129 |
| genus |  | rs61227640 | T | C | -0.076 | 0.016 | 1.40E-06 | 22.128 |
| genus | Bifidobacterium | rs3087343 | T | G | 0.075 | 0.016 | 3.00E-06 | 22.128 |
| genus | RuminococcaceaeUCG010 | rs2820282 | C | A | -0.059 | 0.013 | 2.85E-06 | 22.127 |
| genus | RuminococcaceaeUCG002 | rs62374284 | C | T | -0.057 | 0.012 | 3.47E-06 | 22.126 |
| genus | Peptococcus | rs1964138 | G | A | 0.089 | 0.019 | 2.70E-06 | 22.122 |
| genus | Prevotella9 | rs74569930 | C | T | 0.207 | 0.044 | 3.02E-06 | 22.120 |
| genus | Ruminiclostridium9 | rs4318510 | C | T | -0.052 | 0.011 | 2.53E-06 | 22.120 |
| genus | Parasutterella | rs62313293 | G | A | 0.062 | 0.013 | 2.92E-06 | 22.119 |
| genus | Ruminiclostridium9 | rs7613528 | C | T | -0.060 | 0.013 | 3.49E-06 | 22.118 |
| genus | Hungatella | rs12498886 | T | C | -0.157 | 0.033 | 4.04E-06 | 22.118 |
| genus | Hungatella | rs12510196 | G | A | -0.157 | 0.033 | 4.04E-06 | 22.118 |
| genus | FamilyXIIIAD3011group | rs2999588 | A | G | -0.057 | 0.012 | 2.69E-06 | 22.117 |
| genus | RuminococcaceaeUCG011 | rs11586744 | T | C | -0.117 | 0.025 | 3.18E-06 | 22.116 |
| genus | Barnesiella | rs2057922 | C | G | 0.092 | 0.019 | 3.83E-06 | 22.115 |
| genus | Allisonella | rs1057419 | G | A | 0.121 | 0.026 | 3.91E-06 | 22.115 |
| genus | RuminococcaceaeUCG011 | rs12753971 | C | T | -0.118 | 0.025 | 3.14E-06 | 22.111 |
| genus | RuminococcaceaeUCG011 | rs4130266 | T | G | -0.117 | 0.025 | 3.22E-06 | 22.110 |
| genus | Peptococcus | rs6857366 | G | A | -0.107 | 0.023 | 3.11E-06 | 22.109 |
| genus | Ruminiclostridium6 | rs11035753 | C | T | 0.073 | 0.015 | 2.78E-06 | 22.109 |
| genus | Peptococcus | rs6857791 | C | A | -0.107 | 0.023 | 3.13E-06 | 22.108 |
| genus |  | rs61841040 | T | G | 0.161 | 0.034 | 3.56E-06 | 22.108 |
| genus | RuminococcaceaeUCG011 | rs12742172 | G | A | -0.116 | 0.025 | 3.12E-06 | 22.106 |
| genus | Lachnoclostridium | rs6112319 | G | A | -0.053 | 0.011 | 2.57E-06 | 22.105 |
| genus | RuminococcaceaeNK4A214group | rs114244418 | G | C | -0.175 | 0.037 | 3.59E-06 | 22.105 |
| genus | Odoribacter | rs495772 | T | G | 0.056 | 0.012 | 2.68E-06 | 22.103 |
| genus | RuminococcaceaeUCG011 | rs12728680 | C | T | -0.118 | 0.025 | 3.14E-06 | 22.102 |
| genus | Oscillibacter | rs2303539 | G | A | 0.098 | 0.021 | 3.43E-06 | 22.102 |
| genus | ChristensenellaceaeR | rs4413087 | G | A | 0.054 | 0.012 | 2.60E-06 | 22.101 |
| genus | LachnospiraceaeUCG008 | rs29327 | C | T | 0.080 | 0.017 | 2.96E-06 | 22.100 |
| genus | Sellimonas | rs2124587 | T | C | -0.121 | 0.026 | 3.21E-06 | 22.099 |
| genus | ChristensenellaceaeR | rs4419165 | C | T | 0.054 | 0.012 | 2.61E-06 | 22.098 |
| genus | Intestinibacter | rs10811551 | A | T | -0.112 | 0.024 | 4.54E-06 | 22.097 |
| genus | Butyricimonas | rs1466574 | G | T | 0.067 | 0.014 | 2.00E-06 | 22.096 |
| genus | Paraprevotella | rs7981920 | T | C | -0.080 | 0.017 | 2.99E-06 | 22.095 |
| genus |  | rs516316 | G | C | -0.050 | 0.011 | 2.70E-06 | 22.093 |
| genus | Peptococcus | rs9409341 | G | A | 0.089 | 0.019 | 2.74E-06 | 22.090 |
| genus | Coprococcus3 | rs13394391 | T | C | -0.071 | 0.015 | 2.20E-06 | 22.089 |
| genus | RuminococcaceaeUCG003 | rs516246 | C | T | 0.056 | 0.012 | 2.99E-06 | 22.088 |
| genus | Dialister | rs10086225 | G | A | 0.062 | 0.013 | 2.06E-06 | 22.087 |
| genus |  | rs34561138 | A | G | 0.216 | 0.046 | 2.51E-06 | 22.087 |
| genus | Sellimonas | rs691738 | T | C | -0.120 | 0.026 | 2.94E-06 | 22.086 |
| genus | Hungatella | rs67776571 | A | G | -0.157 | 0.033 | 4.12E-06 | 22.085 |
| genus | Clostridiumsensustricto1 | rs2795528 | A | G | -0.184 | 0.039 | 2.72E-06 | 22.085 |
| genus | RuminococcaceaeUCG011 | rs12737345 | G | A | -0.118 | 0.025 | 3.25E-06 | 22.085 |
| genus | Intestinibacter | rs479954 | C | T | -0.140 | 0.030 | 3.02E-06 | 22.082 |
| genus | Prevotella7 | rs55821055 | G | T | 0.156 | 0.033 | 1.49E-06 | 22.080 |
| genus | FamilyXIIIUCG001 | rs60069152 | C | G | -0.074 | 0.016 | 3.26E-06 | 22.075 |
| genus | RuminococcaceaeUCG013 | rs75088940 | C | T | -0.094 | 0.020 | 2.55E-06 | 22.072 |
| genus | RuminococcaceaeUCG005 | rs12604884 | T | G | 0.068 | 0.014 | 2.45E-06 | 22.071 |
| genus | Anaerofilum | rs17096874 | T | C | -0.126 | 0.027 | 2.86E-06 | 22.071 |
| genus | Ruminiclostridium9 | rs9857678 | T | C | -0.053 | 0.011 | 2.81E-06 | 22.070 |
| genus | LachnospiraceaeNK4A136group | rs34832190 | A | G | -0.054 | 0.011 | 4.29E-06 | 22.070 |
| genus |  | rs61416816 | C | T | 0.079 | 0.017 | 1.83E-06 | 22.069 |
| genus | ErysipelotrichaceaeUCG003 | rs59068084 | G | T | 0.056 | 0.012 | 3.12E-06 | 22.068 |
| genus | Ruminococcus1 | rs4755335 | C | A | -0.054 | 0.011 | 2.98E-06 | 22.067 |
| genus |  | rs4542578 | C | G | -0.160 | 0.034 | 3.42E-06 | 22.067 |
| genus | Ruminiclostridium5 | rs4007288 | G | A | -0.054 | 0.011 | 2.67E-06 | 22.065 |
| genus | Methanobrevibacter | rs894996 | A | C | 0.214 | 0.046 | 3.82E-06 | 22.064 |
| genus | Eisenbergiella | rs1580775 | A | G | 0.105 | 0.022 | 2.81E-06 | 22.063 |
| genus | LachnospiraceaeNK4A136group | rs11628089 | C | T | -0.054 | 0.012 | 4.15E-06 | 22.063 |
| genus | Hungatella | rs4691167 | A | G | -0.157 | 0.033 | 4.05E-06 | 22.060 |
| genus | Desulfovibrio | rs11160353 | A | T | -0.069 | 0.015 | 2.70E-06 | 22.059 |
| genus | Bifidobacterium | rs4954555 | T | C | 0.111 | 0.024 | 1.66E-06 | 22.059 |
| genus | Oscillibacter | rs436827 | T | A | -0.071 | 0.015 | 3.00E-06 | 22.057 |
| genus | LachnospiraceaeFCS020group | rs1010831 | A | C | -0.057 | 0.012 | 3.08E-06 | 22.057 |
| genus | Blautia | rs115043014 | A | G | -0.207 | 0.044 | 5.19E-06 | 22.057 |
| genus | Anaerofilum | rs17105491 | C | G | -0.193 | 0.041 | 1.57E-06 | 22.056 |
| genus | Ruminococcus2 | rs4073396 | A | C | 0.065 | 0.014 | 3.27E-06 | 22.051 |
| genus | RuminococcaceaeUCG002 | rs12026045 | G | A | -0.069 | 0.015 | 2.01E-06 | 22.050 |
| genus | Intestinibacter | rs519372 | G | A | -0.140 | 0.030 | 3.08E-06 | 22.049 |
| genus | Parasutterella | rs11131566 | T | A | 0.062 | 0.013 | 3.04E-06 | 22.049 |
| genus | LachnospiraceaeFCS020group | rs10093861 | A | G | -0.057 | 0.012 | 3.06E-06 | 22.048 |
| genus | Parasutterella | rs62313331 | A | G | 0.062 | 0.013 | 2.89E-06 | 22.046 |
| genus | Peptococcus | rs62424012 | A | G | 0.137 | 0.029 | 1.15E-06 | 22.045 |
| genus | RuminococcaceaeUCG011 | rs12722736 | G | T | -0.117 | 0.025 | 3.29E-06 | 22.044 |
| genus | Escherichia | rs1154904 | G | A | -0.061 | 0.013 | 3.04E-06 | 22.043 |
| genus | RuminococcaceaeUCG011 | rs34592589 | T | A | -0.163 | 0.035 | 2.48E-06 | 22.042 |
| genus |  | rs12881844 | C | T | -0.158 | 0.034 | 3.59E-06 | 22.041 |
| genus |  | rs191030074 | G | A | -0.087 | 0.018 | 2.01E-06 | 22.041 |
| genus | Anaerofilum | rs62265650 | G | A | 0.097 | 0.021 | 2.74E-06 | 22.041 |
| genus |  | rs28584818 | G | A | 0.126 | 0.027 | 4.43E-06 | 22.041 |
| genus | LachnospiraceaeUCG008 | rs1992107 | C | T | -0.103 | 0.022 | 2.54E-06 | 22.040 |
| genus | Butyrivibrio | rs74622183 | G | A | -0.201 | 0.043 | 2.46E-06 | 22.040 |
| genus | Lachnoclostridium | rs12481174 | T | C | -0.052 | 0.011 | 2.60E-06 | 22.038 |
| genus | Odoribacter | rs78878135 | T | G | -0.126 | 0.027 | 2.94E-06 | 22.038 |
| genus | Parasutterella | rs7687560 | C | T | 0.062 | 0.013 | 3.10E-06 | 22.038 |
| genus | Prevotella7 | rs385483 | G | A | 0.137 | 0.029 | 2.92E-06 | 22.038 |
| genus | Hungatella | rs7656134 | G | A | -0.157 | 0.033 | 4.18E-06 | 22.037 |
| genus | Sellimonas | rs2470619 | A | G | 0.118 | 0.025 | 2.83E-06 | 22.036 |
| genus | RuminococcaceaeNK4A214group | rs4814689 | T | C | -0.108 | 0.023 | 4.55E-06 | 22.036 |
| genus | Butyrivibrio | rs61269242 | C | T | -0.113 | 0.024 | 3.04E-06 | 22.036 |
| genus | RuminococcaceaeUCG014 | rs10941294 | T | C | -0.122 | 0.026 | 2.40E-06 | 22.036 |
| genus | Marvinbryantia | rs61884470 | A | T | 0.117 | 0.025 | 5.05E-06 | 22.035 |
| genus | Marvinbryantia | rs2768428 | C | T | -0.075 | 0.016 | 3.38E-06 | 22.033 |
| genus | Parasutterella | rs908612 | A | G | 0.063 | 0.013 | 2.81E-06 | 22.031 |
| genus | CandidatusSoleaferrea | rs712066 | C | T | 0.107 | 0.023 | 3.22E-06 | 22.031 |
| genus | Hungatella | rs13110539 | G | A | -0.157 | 0.033 | 4.23E-06 | 22.029 |
| genus | Hungatella | rs13137292 | T | C | -0.157 | 0.033 | 4.23E-06 | 22.029 |
| genus | Hungatella | rs35614407 | C | T | -0.157 | 0.033 | 4.23E-06 | 22.029 |
| genus | Hungatella | rs34311511 | T | G | -0.157 | 0.033 | 4.23E-06 | 22.029 |
| genus | Hungatella | rs68011503 | G | C | -0.157 | 0.033 | 4.23E-06 | 22.029 |
| genus | Hungatella | rs35201973 | T | C | -0.157 | 0.033 | 4.23E-06 | 22.029 |
| genus | Enterorhabdus | rs3017103 | G | A | 0.098 | 0.021 | 2.94E-06 | 22.028 |
| genus | Ruminiclostridium9 | rs60745172 | A | C | -0.055 | 0.012 | 3.29E-06 | 22.028 |
| genus | Streptococcus | rs10826446 | T | C | 0.102 | 0.022 | 3.24E-06 | 22.028 |
| genus |  | rs601338 | G | A | -0.050 | 0.011 | 2.77E-06 | 22.028 |
| genus | Gordonibacter | rs111233479 | A | G | 0.177 | 0.038 | 2.62E-06 | 22.024 |
| genus |  | rs265534 | G | T | -0.056 | 0.012 | 2.27E-06 | 22.024 |
| genus | Anaerotruncus | rs9603208 | T | G | 0.078 | 0.017 | 1.57E-06 | 22.023 |
| genus | RuminococcaceaeUCG011 | rs11802625 | G | A | -0.117 | 0.025 | 3.34E-06 | 22.023 |
| genus | Parasutterella | rs1543164 | G | C | 0.063 | 0.013 | 2.93E-06 | 22.020 |
| genus | Paraprevotella | rs58506674 | T | G | -0.081 | 0.017 | 3.97E-06 | 22.019 |
| genus |  | rs6722245 | G | A | 0.124 | 0.026 | 2.41E-06 | 22.016 |
| genus | RuminococcaceaeUCG011 | rs113805683 | T | C | 0.137 | 0.029 | 2.84E-06 | 22.013 |
| genus | LachnospiraceaeNK4A136group | rs12885960 | A | C | -0.054 | 0.012 | 3.38E-06 | 22.013 |
| genus | RuminococcaceaeUCG003 | rs11831525 | C | A | 0.071 | 0.015 | 2.58E-06 | 22.012 |
| genus | LachnospiraceaeFCS020group | rs11992238 | T | C | -0.057 | 0.012 | 3.12E-06 | 22.012 |
| genus | RuminococcaceaeUCG005 | rs35166120 | G | C | -0.069 | 0.015 | 3.75E-06 | 22.011 |
| genus | Parasutterella | rs1425366 | A | G | 0.062 | 0.013 | 3.23E-06 | 22.011 |
| genus | RuminococcaceaeUCG003 | rs7581860 | G | A | 0.094 | 0.020 | 4.95E-06 | 22.011 |
| genus | Streptococcus | rs11720390 | A | G | 0.107 | 0.023 | 3.59E-06 | 22.010 |
| genus | LachnospiraceaeUCG001 | rs9403580 | T | C | 0.108 | 0.023 | 3.47E-06 | 22.010 |
| genus | Veillonella | rs1882878 | G | A | -0.077 | 0.016 | 2.98E-06 | 22.010 |
| genus | Bifidobacterium | rs614193 | A | T | 0.074 | 0.016 | 3.05E-06 | 22.009 |
| genus | Lachnoclostridium | rs6046013 | A | G | -0.050 | 0.011 | 2.87E-06 | 22.008 |
| genus | Roseburia | rs6930661 | T | C | -0.096 | 0.020 | 2.48E-06 | 22.008 |
| genus | Ruminiclostridium5 | rs7296521 | C | T | -0.050 | 0.011 | 3.15E-06 | 22.001 |
| genus | Bilophila | rs10954636 | C | G | 0.064 | 0.014 | 4.09E-06 | 21.998 |
| genus | Ruminococcus2 | rs4078064 | T | C | 0.065 | 0.014 | 3.48E-06 | 21.998 |
| genus | LachnospiraceaeFCS020group | rs4871049 | G | A | -0.057 | 0.012 | 3.14E-06 | 21.998 |
| genus | Paraprevotella | rs868220 | A | G | -0.082 | 0.018 | 3.83E-06 | 21.998 |
| genus |  | rs12432441 | T | A | 0.072 | 0.015 | 6.17E-06 | 21.996 |
| genus | ChristensenellaceaeR | rs10412550 | C | T | 0.054 | 0.012 | 2.81E-06 | 21.996 |
| genus | Howardella | rs672217 | A | G | 0.164 | 0.035 | 3.52E-06 | 21.996 |
| genus | LachnospiraceaeUCG008 | rs10793104 | A | G | 0.092 | 0.020 | 2.99E-06 | 21.995 |
| genus | RuminococcaceaeUCG010 | rs35506912 | C | G | -0.069 | 0.015 | 3.21E-06 | 21.994 |
| genus | RuminococcaceaeUCG003 | rs10490280 | T | C | -0.067 | 0.014 | 4.16E-06 | 21.994 |
| genus | Odoribacter | rs17103644 | A | G | -0.126 | 0.027 | 2.80E-06 | 21.994 |
| genus |  | rs66522207 | A | C | -0.160 | 0.034 | 4.01E-06 | 21.993 |
| genus | LachnospiraceaeNK4A136group | rs12895121 | C | G | -0.054 | 0.012 | 4.35E-06 | 21.992 |
| genus | LachnospiraceaeNK4A136group | rs34891372 | G | A | -0.053 | 0.011 | 4.66E-06 | 21.991 |
| genus | LachnospiraceaeNC2004group | rs3756315 | G | A | -0.088 | 0.019 | 3.33E-06 | 21.991 |
| genus | LachnospiraceaeUCG001 | rs2201163 | G | A | 0.071 | 0.015 | 3.03E-06 | 21.988 |
| genus | Bifidobacterium | rs3098102 | C | T | 0.074 | 0.016 | 2.95E-06 | 21.986 |
| genus | Coprobacter | rs143662916 | T | C | 0.253 | 0.054 | 3.07E-06 | 21.985 |
| genus | LachnospiraceaeFCS020group | rs811892 | C | G | 0.133 | 0.028 | 5.17E-06 | 21.983 |
| genus | RuminococcaceaeUCG002 | rs2265670 | G | C | -0.051 | 0.011 | 2.99E-06 | 21.983 |
| genus | CandidatusSoleaferrea | rs712065 | T | C | 0.109 | 0.023 | 2.91E-06 | 21.983 |
| genus | Bifidobacterium | rs11895609 | A | T | 0.079 | 0.017 | 1.03E-06 | 21.981 |
| genus | Holdemania | rs116500994 | T | G | -0.138 | 0.029 | 2.34E-06 | 21.981 |
| genus | Dorea | rs4793307 | T | C | 0.057 | 0.012 | 4.01E-06 | 21.979 |
| genus | Desulfovibrio | rs10129801 | C | T | -0.069 | 0.015 | 2.82E-06 | 21.978 |
| genus | Bifidobacterium | rs7574366 | G | A | 0.095 | 0.020 | 6.27E-06 | 21.975 |
| genus | Sutterella | rs1470456 | A | G | -0.059 | 0.012 | 2.57E-06 | 21.974 |
| genus | RuminococcaceaeUCG011 | rs12758415 | T | C | -0.117 | 0.025 | 3.41E-06 | 21.973 |
| genus | Ruminiclostridium5 | rs10735003 | C | T | -0.050 | 0.011 | 3.07E-06 | 21.972 |
| genus | Dialister | rs10106300 | C | A | 0.062 | 0.013 | 2.17E-06 | 21.968 |
| genus | RuminococcaceaeUCG011 | rs10913739 | C | G | -0.117 | 0.025 | 3.38E-06 | 21.968 |
| genus | RuminococcaceaeUCG011 | rs12744251 | G | A | -0.117 | 0.025 | 3.43E-06 | 21.966 |
| genus | Butyricimonas | rs1038524 | G | A | 0.067 | 0.014 | 2.44E-06 | 21.966 |
| genus | Bifidobacterium | rs4954571 | G | A | 0.064 | 0.014 | 1.58E-06 | 21.966 |
| genus | Peptococcus | rs1441210 | T | C | -0.106 | 0.023 | 3.39E-06 | 21.966 |
| genus | Flavonifractor | rs12795061 | C | A | 0.075 | 0.016 | 2.28E-06 | 21.964 |
| genus | RuminococcaceaeUCG002 | rs79016051 | T | C | -0.089 | 0.019 | 2.34E-06 | 21.964 |
| genus | RuminococcaceaeUCG005 | rs12458218 | C | T | 0.068 | 0.014 | 2.41E-06 | 21.964 |
| genus | RuminococcaceaeUCG011 | rs9729514 | G | A | 0.185 | 0.039 | 2.37E-06 | 21.962 |
| genus | Anaerotruncus | rs4669806 | T | G | 0.058 | 0.012 | 2.42E-06 | 21.962 |
| genus | RuminococcaceaeUCG011 | rs7538909 | C | T | -0.117 | 0.025 | 3.41E-06 | 21.960 |
| genus | RuminococcaceaeUCG011 | rs7515710 | G | A | -0.117 | 0.025 | 3.41E-06 | 21.960 |
| genus | RuminococcaceaeUCG011 | rs7539388 | C | T | -0.117 | 0.025 | 3.41E-06 | 21.960 |
| genus | RuminococcaceaeUCG011 | rs7515896 | G | C | -0.117 | 0.025 | 3.41E-06 | 21.960 |
| genus | RuminococcaceaeUCG011 | rs61821011 | A | G | -0.117 | 0.025 | 3.48E-06 | 21.960 |
| genus | Streptococcus | rs7966796 | A | G | -0.084 | 0.018 | 3.99E-06 | 21.959 |
| genus |  | rs142794441 | T | A | -0.171 | 0.037 | 2.59E-06 | 21.959 |
| genus | RuminococcaceaeUCG010 | rs6958419 | T | C | -0.059 | 0.012 | 2.84E-06 | 21.958 |
| genus | RuminococcaceaeUCG011 | rs4454499 | C | G | -0.117 | 0.025 | 3.45E-06 | 21.957 |
| genus | Anaerostipes | rs2769393 | A | G | -0.051 | 0.011 | 3.13E-06 | 21.957 |
| genus | Ruminiclostridium9 | rs62289791 | A | G | -0.053 | 0.011 | 3.13E-06 | 21.956 |
| genus | Victivallis | rs6445926 | C | G | 0.117 | 0.025 | 2.96E-06 | 21.955 |
| genus | Bifidobacterium | rs309162 | T | A | 0.074 | 0.016 | 3.28E-06 | 21.955 |
| genus | Bifidobacterium | rs72976292 | T | A | 0.060 | 0.013 | 2.11E-06 | 21.955 |
| genus | Streptococcus | rs7916740 | C | T | 0.102 | 0.022 | 3.43E-06 | 21.955 |
| genus | Paraprevotella | rs10501247 | C | G | -0.136 | 0.029 | 5.98E-06 | 21.954 |
| genus | Ruminococcus1 | rs7583465 | T | C | 0.053 | 0.011 | 2.56E-06 | 21.952 |
| genus | LachnospiraceaeFCS020group | rs4085481 | T | C | 0.121 | 0.026 | 5.77E-06 | 21.949 |
| genus | Anaerostipes | rs2804213 | T | C | -0.051 | 0.011 | 3.19E-06 | 21.948 |
| genus | Olsenella | rs2759329 | A | G | -0.111 | 0.024 | 3.43E-06 | 21.947 |
| genus | Lachnoclostridium | rs575175 | A | T | -0.050 | 0.011 | 3.75E-06 | 21.945 |
| genus | Anaerostipes | rs10509991 | A | G | -0.052 | 0.011 | 3.46E-06 | 21.945 |
| genus | Prevotella7 | rs430270 | C | A | 0.139 | 0.030 | 2.87E-06 | 21.944 |
| genus | RuminococcaceaeUCG011 | rs11802624 | G | A | -0.117 | 0.025 | 3.47E-06 | 21.944 |
| genus | RuminococcaceaeUCG011 | rs34362270 | T | C | -0.139 | 0.030 | 2.73E-06 | 21.942 |
| genus | Intestinimonas | rs2731794 | T | C | 0.121 | 0.026 | 1.92E-06 | 21.942 |
| genus | RuminococcaceaeUCG011 | rs11582293 | C | G | -0.117 | 0.025 | 3.48E-06 | 21.941 |
| genus | RuminococcaceaeUCG011 | rs11578241 | G | C | -0.117 | 0.025 | 3.48E-06 | 21.941 |
| genus | Marvinbryantia | rs2815602 | C | G | -0.074 | 0.016 | 3.53E-06 | 21.939 |
| genus | Streptococcus | rs7901835 | T | C | 0.102 | 0.022 | 3.43E-06 | 21.939 |
| genus | LachnospiraceaeUCG008 | rs57091572 | G | A | -0.110 | 0.024 | 2.86E-06 | 21.937 |
| genus | Butyrivibrio | rs462432 | A | G | -0.112 | 0.024 | 3.19E-06 | 21.937 |
| genus | RuminococcaceaeUCG011 | rs12729895 | G | A | -0.117 | 0.025 | 3.48E-06 | 21.937 |
| genus | Enterorhabdus | rs73331717 | T | C | 0.258 | 0.055 | 6.39E-06 | 21.936 |
| genus | Oscillibacter | rs12649930 | G | T | 0.122 | 0.026 | 4.09E-06 | 21.935 |
| genus | RuminococcaceaeUCG011 | rs9377649 | G | A | -0.138 | 0.029 | 2.60E-06 | 21.935 |
| genus | LachnospiraceaeUCG010 | rs72761829 | T | A | 0.112 | 0.024 | 2.58E-06 | 21.934 |
| genus | Ruminiclostridium5 | rs6121460 | A | G | 0.093 | 0.020 | 2.64E-06 | 21.934 |
| genus | LachnospiraceaeFCS020group | rs4871050 | C | G | -0.057 | 0.012 | 3.28E-06 | 21.932 |
| genus | RuminococcaceaeUCG005 | rs7555878 | G | A | 0.059 | 0.013 | 2.81E-06 | 21.931 |
| genus | LachnospiraceaeUCG004 | rs12673420 | A | G | 0.055 | 0.012 | 2.98E-06 | 21.930 |
| genus | LachnospiraceaeFCS020group | rs62140927 | T | C | 0.061 | 0.013 | 2.38E-06 | 21.929 |
| genus | Paraprevotella | rs2058085 | A | C | -0.081 | 0.017 | 4.48E-06 | 21.928 |
| genus | RuminococcaceaeUCG011 | rs10913760 | G | A | -0.117 | 0.025 | 3.51E-06 | 21.928 |
| genus | RuminococcaceaeUCG011 | rs10913761 | A | G | -0.117 | 0.025 | 3.51E-06 | 21.928 |
| genus | Lachnoclostridium | rs1033549 | G | T | -0.051 | 0.011 | 3.11E-06 | 21.928 |
| genus | Senegalimassilia | rs11788425 | A | G | 0.080 | 0.017 | 3.75E-06 | 21.927 |
| genus | Bifidobacterium | rs73958638 | A | G | 0.114 | 0.024 | 2.12E-06 | 21.925 |
| genus | Enterorhabdus | rs424715 | C | T | 0.082 | 0.017 | 4.41E-06 | 21.925 |
| genus | LachnospiraceaeFCS020group | rs425629 | A | C | 0.121 | 0.026 | 6.00E-06 | 21.924 |
| genus | RuminococcaceaeUCG011 | rs4233177 | C | T | -0.117 | 0.025 | 3.32E-06 | 21.924 |
| genus | Fusicatenibacter | rs8063430 | C | T | -0.104 | 0.022 | 4.93E-06 | 21.924 |
| genus | ErysipelotrichaceaeUCG003 | rs6875357 | T | C | 0.166 | 0.035 | 6.70E-06 | 21.924 |
| genus | Paraprevotella | rs73011275 | C | T | -0.123 | 0.026 | 4.44E-06 | 21.923 |
| genus | Anaerostipes | rs1581904 | C | A | -0.051 | 0.011 | 3.13E-06 | 21.923 |
| genus | Peptococcus | rs9409014 | C | G | 0.089 | 0.019 | 2.99E-06 | 21.922 |
| genus | LachnospiraceaeNC2004group | rs12127733 | A | G | 0.115 | 0.025 | 3.11E-06 | 21.922 |
| genus | RuminococcaceaeUCG003 | rs2548459 | T | C | 0.055 | 0.012 | 3.18E-06 | 21.921 |
| genus | Oxalobacter | rs7270247 | A | C | -0.108 | 0.023 | 3.37E-06 | 21.920 |
| genus | Anaerostipes | rs2769392 | G | A | -0.051 | 0.011 | 3.19E-06 | 21.918 |
| genus | Intestinibacter | rs530007 | C | G | -0.139 | 0.030 | 3.28E-06 | 21.918 |
| genus | Howardella | rs609430 | G | T | -0.112 | 0.024 | 3.34E-06 | 21.918 |
| genus | Peptococcus | rs6839387 | T | C | -0.106 | 0.023 | 3.42E-06 | 21.918 |
| genus | Howardella | rs525833 | A | G | -0.112 | 0.024 | 3.42E-06 | 21.918 |
| genus | Lachnospira | rs56791201 | C | T | 0.052 | 0.011 | 2.93E-06 | 21.917 |
| genus | RuminococcaceaeUCG011 | rs7532155 | T | C | -0.116 | 0.025 | 3.56E-06 | 21.916 |
| genus | LachnospiraceaeND3007group | rs11861453 | G | A | -0.054 | 0.012 | 2.89E-06 | 21.913 |
| genus | Paraprevotella | rs9547256 | G | T | -0.081 | 0.017 | 3.48E-06 | 21.913 |
| genus | Bacteroides | rs9507307 | T | C | 0.060 | 0.013 | 2.13E-06 | 21.912 |
| genus | Intestinibacter | rs10965008 | A | G | -0.108 | 0.023 | 3.04E-06 | 21.912 |
| genus | DefluviitaleaceaeUCG011 | rs9725395 | G | A | -0.138 | 0.030 | 3.52E-06 | 21.911 |
| genus | Anaerofilum | rs503294 | G | A | -0.096 | 0.021 | 2.35E-06 | 21.908 |
| genus | Senegalimassilia | rs72887800 | A | T | -0.082 | 0.018 | 2.42E-06 | 21.907 |
| genus | Ruminiclostridium6 | rs11554764 | T | C | 0.151 | 0.032 | 3.82E-06 | 21.906 |
| genus | RuminococcaceaeUCG011 | rs12738262 | G | A | -0.117 | 0.025 | 3.19E-06 | 21.906 |
| genus | Tyzzerella3 | rs4904512 | C | T | -0.117 | 0.025 | 3.09E-06 | 21.905 |
| genus | Ruminiclostridium5 | rs9805049 | C | A | 0.050 | 0.011 | 2.93E-06 | 21.903 |
| genus | Erysipelatoclostridium | rs6474512 | C | A | 0.067 | 0.014 | 3.02E-06 | 21.900 |
| genus | LachnospiraceaeNK4A136group | rs73044693 | G | A | -0.108 | 0.023 | 3.57E-06 | 21.900 |
| genus |  | rs10917203 | C | A | 0.061 | 0.013 | 3.15E-06 | 21.898 |
| genus | Haemophilus | rs35508 | A | G | 0.126 | 0.027 | 3.62E-06 | 21.897 |
| genus | Anaerostipes | rs2396460 | C | T | -0.051 | 0.011 | 2.91E-06 | 21.897 |
| genus | Intestinibacter | rs118030283 | A | G | -0.152 | 0.032 | 2.67E-06 | 21.896 |
| genus | Phascolarctobacterium | rs74540770 | A | G | -0.121 | 0.026 | 3.60E-06 | 21.895 |
| genus | Paraprevotella | rs11654896 | A | G | -0.081 | 0.017 | 4.18E-06 | 21.894 |
| genus | Fusicatenibacter | rs704421 | G | C | 0.071 | 0.015 | 3.32E-06 | 21.893 |
| genus | Streptococcus | rs12537178 | G | A | -0.067 | 0.014 | 3.12E-06 | 21.892 |
| genus | Parasutterella | rs11736175 | A | G | 0.062 | 0.013 | 3.28E-06 | 21.892 |
| genus | Streptococcus | rs7316693 | A | G | -0.058 | 0.012 | 2.82E-06 | 21.891 |
| genus |  | rs55716278 | A | G | -0.170 | 0.036 | 2.41E-06 | 21.890 |
| genus | Ruminiclostridium9 | rs12419854 | A | T | -0.073 | 0.016 | 3.18E-06 | 21.890 |
| genus | Anaerotruncus | rs9594211 | C | T | 0.077 | 0.017 | 1.67E-06 | 21.890 |
| genus | Olsenella | rs73440467 | G | A | -0.240 | 0.051 | 3.51E-06 | 21.889 |
| genus | LachnospiraceaeNK4A136group | rs58648175 | A | C | -0.054 | 0.011 | 3.64E-06 | 21.887 |
| genus | RuminococcaceaeUCG011 | rs11808460 | C | T | -0.117 | 0.025 | 3.54E-06 | 21.887 |
| genus | Roseburia | rs16910295 | C | T | -0.098 | 0.021 | 2.91E-06 | 21.887 |
| genus | ErysipelotrichaceaeUCG003 | rs357853 | T | C | -0.099 | 0.021 | 2.98E-06 | 21.884 |
| genus | Parasutterella | rs6826533 | A | G | 0.062 | 0.013 | 3.26E-06 | 21.883 |
| genus | LachnospiraceaeUCG008 | rs9846590 | G | A | -0.115 | 0.025 | 3.11E-06 | 21.882 |
| genus | Lachnoclostridium | rs2024618 | A | G | -0.051 | 0.011 | 3.21E-06 | 21.882 |
| genus | Bacteroides | rs495004 | G | C | -0.061 | 0.013 | 3.42E-06 | 21.881 |
| genus | Victivallis | rs2885255 | C | G | 0.117 | 0.025 | 3.06E-06 | 21.879 |
| genus | Odoribacter | rs3850600 | G | A | -0.128 | 0.027 | 2.76E-06 | 21.879 |
| genus | Bifidobacterium | rs13410301 | T | C | 0.056 | 0.012 | 3.56E-06 | 21.877 |
| genus | Odoribacter | rs11836317 | G | A | -0.126 | 0.027 | 3.05E-06 | 21.877 |
| genus | Odoribacter | rs17184182 | A | G | -0.128 | 0.027 | 2.76E-06 | 21.877 |
| genus | Parasutterella | rs6822428 | C | T | 0.062 | 0.013 | 3.28E-06 | 21.876 |
| genus | Marvinbryantia | rs2492951 | A | G | -0.074 | 0.016 | 3.73E-06 | 21.874 |
| genus | Streptococcus | rs7134384 | T | C | -0.083 | 0.018 | 2.38E-06 | 21.872 |
| genus | Streptococcus | rs7971435 | T | G | -0.086 | 0.018 | 3.22E-06 | 21.872 |
| genus | RuminococcaceaeUCG011 | rs12731823 | A | G | -0.117 | 0.025 | 3.58E-06 | 21.869 |
| genus | RuminococcaceaeUCG014 | . | C | T | 0.127 | 0.027 | 3.85E-06 | 21.867 |
| genus | Prevotella9 | rs2672226 | A | T | -0.070 | 0.015 | 2.43E-06 | 21.867 |
| genus |  | rs13166733 | T | A | -0.108 | 0.023 | 2.74E-06 | 21.867 |
| genus | LachnospiraceaeUCG008 | rs29301 | A | G | 0.080 | 0.017 | 2.99E-06 | 21.865 |
| genus | Olsenella | rs4658950 | A | G | -0.111 | 0.024 | 3.58E-06 | 21.865 |
| genus | Streptococcus | rs10826445 | G | A | 0.102 | 0.022 | 3.46E-06 | 21.864 |
| genus | Alistipes | rs11773263 | C | T | -0.051 | 0.011 | 3.17E-06 | 21.863 |
| genus | Collinsella | rs2103510 | A | G | 0.079 | 0.017 | 2.42E-06 | 21.863 |
| genus | Eisenbergiella | rs1508033 | C | A | 0.092 | 0.020 | 3.23E-06 | 21.863 |
| genus | Butyrivibrio | rs12451977 | C | G | 0.121 | 0.026 | 2.54E-06 | 21.863 |
| genus |  | rs10931481 | A | G | 0.061 | 0.013 | 3.38E-06 | 21.861 |
| genus | Intestinibacter | rs6590163 | A | G | -0.139 | 0.030 | 3.40E-06 | 21.861 |
| genus |  | rs62167033 | C | T | 0.185 | 0.040 | 3.50E-06 | 21.861 |
| genus | Lachnospira | rs4686798 | C | T | 0.053 | 0.011 | 2.74E-06 | 21.860 |
| genus | Hungatella | rs35309983 | A | G | -0.157 | 0.033 | 4.64E-06 | 21.860 |
| genus | Bifidobacterium | rs6430601 | G | T | 0.080 | 0.017 | 1.09E-06 | 21.858 |
| genus | Butyrivibrio | rs8075922 | G | A | 0.120 | 0.026 | 2.42E-06 | 21.858 |
| genus | ErysipelotrichaceaeUCG003 | rs62403464 | C | T | -0.073 | 0.016 | 3.44E-06 | 21.858 |
| genus | Intestinimonas | rs7170984 | C | T | -0.066 | 0.014 | 2.98E-06 | 21.858 |
| genus | LachnospiraceaeUCG008 | rs998759 | C | A | -0.110 | 0.024 | 3.01E-06 | 21.857 |
| genus | Parasutterella | rs10175844 | T | C | 0.062 | 0.013 | 3.02E-06 | 21.856 |
| genus |  | rs3919476 | G | A | -0.077 | 0.016 | 1.92E-06 | 21.856 |
| genus | Peptococcus | rs9995986 | G | A | -0.094 | 0.020 | 3.58E-06 | 21.855 |
| genus | Peptococcus | rs9409013 | G | C | 0.089 | 0.019 | 3.15E-06 | 21.855 |
| genus | Actinomyces | rs7915461 | C | T | 0.188 | 0.040 | 5.92E-06 | 21.854 |
| genus |  | rs681343 | C | T | -0.050 | 0.011 | 3.01E-06 | 21.854 |
| genus | Flavonifractor | rs12790444 | G | A | 0.075 | 0.016 | 2.39E-06 | 21.853 |
| genus | Anaerostipes | rs1336116 | A | C | -0.052 | 0.011 | 3.61E-06 | 21.851 |
| genus | Bifidobacterium | rs7570283 | T | C | 0.091 | 0.020 | 7.21E-06 | 21.851 |
| genus | Lachnoclostridium | rs6046012 | T | C | -0.050 | 0.011 | 3.17E-06 | 21.850 |
| genus | Turicibacter | rs11649454 | C | G | 0.095 | 0.020 | 3.27E-06 | 21.848 |
| genus | RuminococcaceaeUCG002 | rs12031559 | C | T | -0.070 | 0.015 | 1.84E-06 | 21.847 |
| genus |  | rs60254196 | G | A | -0.052 | 0.011 | 2.70E-06 | 21.844 |
| genus |  | rs72506102 | T | G | -0.077 | 0.016 | 1.85E-06 | 21.843 |
| genus | Butyrivibrio | rs11768123 | T | C | 0.162 | 0.035 | 6.38E-06 | 21.842 |
| genus | Barnesiella | rs199035 | A | G | 0.056 | 0.012 | 3.00E-06 | 21.842 |
| genus | Lachnoclostridium | rs2024619 | G | A | -0.050 | 0.011 | 3.10E-06 | 21.841 |
| genus | Blautia | rs67619974 | A | G | 0.058 | 0.012 | 2.67E-06 | 21.839 |
| genus | ErysipelotrichaceaeUCG003 | rs9304371 | T | C | -0.099 | 0.021 | 2.94E-06 | 21.839 |
| genus | RuminococcaceaeUCG011 | rs12730909 | C | T | -0.117 | 0.025 | 3.72E-06 | 21.839 |
| genus | Eggerthella | rs112205261 | C | T | -0.189 | 0.040 | 3.35E-06 | 21.839 |
| genus | Fusicatenibacter | rs3303 | C | T | -0.095 | 0.020 | 3.94E-06 | 21.839 |
| genus | RuminococcaceaeUCG009 | rs2058609 | G | A | 0.082 | 0.017 | 3.12E-06 | 21.837 |
| genus | RuminococcaceaeUCG011 | rs10913753 | A | G | -0.117 | 0.025 | 3.70E-06 | 21.836 |
| genus | Lactobacillus | rs6092149 | T | A | -0.080 | 0.017 | 3.29E-06 | 21.835 |
| genus | Coprococcus3 | rs11681779 | C | T | -0.069 | 0.015 | 4.10E-06 | 21.834 |
| genus | Bifidobacterium | rs79176913 | C | A | 0.074 | 0.016 | 3.62E-06 | 21.834 |
| genus | RuminococcaceaeUCG003 | rs11613919 | T | G | 0.073 | 0.016 | 1.63E-06 | 21.833 |
| genus | Coprobacter | rs55672356 | A | T | -0.193 | 0.041 | 2.74E-06 | 21.832 |
| genus | Peptococcus | rs6837958 | C | G | -0.106 | 0.023 | 3.61E-06 | 21.831 |
| genus | Parasutterella | rs6570145 | C | T | 0.086 | 0.018 | 4.18E-06 | 21.831 |
| genus | Intestinibacter | rs4937042 | T | C | -0.139 | 0.030 | 3.22E-06 | 21.831 |
| genus | Intestinibacter | rs4937044 | C | T | -0.139 | 0.030 | 3.22E-06 | 21.831 |
| genus | Turicibacter | rs75286509 | A | T | 0.101 | 0.022 | 2.83E-06 | 21.830 |
| genus | Parasutterella | rs4860163 | T | C | 0.062 | 0.013 | 3.46E-06 | 21.827 |
| genus | Oscillibacter | rs11156709 | A | G | -0.072 | 0.016 | 2.98E-06 | 21.826 |
| genus | Oscillibacter | rs4506202 | G | A | -0.071 | 0.015 | 3.21E-06 | 21.825 |
| genus | RuminococcaceaeUCG005 | rs62504976 | C | T | -0.068 | 0.015 | 4.14E-06 | 21.825 |
| genus | Bifidobacterium | rs10432371 | G | A | 0.056 | 0.012 | 2.07E-06 | 21.824 |
| genus | Roseburia | rs116270582 | A | T | -0.154 | 0.033 | 1.20E-06 | 21.823 |
| genus | Bifidobacterium | rs3099427 | A | G | 0.073 | 0.016 | 3.32E-06 | 21.823 |
| genus | Ruminiclostridium5 | rs7978783 | A | G | -0.050 | 0.011 | 3.13E-06 | 21.823 |
| genus | LachnospiraceaeUCG008 | rs11098190 | C | T | 0.079 | 0.017 | 3.49E-06 | 21.822 |
| genus | Adlercreutzia | rs1528942 | G | T | -0.083 | 0.018 | 3.25E-06 | 21.821 |
| genus |  | rs17118317 | T | C | 0.076 | 0.016 | 2.23E-06 | 21.818 |
| genus | Intestinibacter | rs2109677 | G | A | 0.069 | 0.015 | 4.15E-06 | 21.818 |
| genus |  | rs4076415 | G | T | 0.052 | 0.011 | 1.99E-06 | 21.815 |
| genus | Oxalobacter | rs10464997 | A | G | 0.138 | 0.029 | 3.30E-06 | 21.815 |
| genus | Paraprevotella | rs34103441 | G | A | -0.104 | 0.022 | 1.48E-06 | 21.814 |
| genus | Anaerofilum | rs62265649 | G | A | 0.097 | 0.021 | 3.05E-06 | 21.814 |
| genus | Anaerostipes | rs10885750 | C | G | -0.051 | 0.011 | 3.37E-06 | 21.812 |
| genus |  | rs12533801 | G | A | -0.076 | 0.016 | 1.89E-06 | 21.812 |
| genus | Intestinibacter | rs4698733 | T | C | 0.065 | 0.014 | 3.18E-06 | 21.811 |
| genus | Parasutterella | rs1991220 | C | T | 0.062 | 0.013 | 3.20E-06 | 21.811 |
| genus | Clostridiumsensustricto1 | rs2817172 | T | C | 0.058 | 0.012 | 2.77E-06 | 21.810 |
| genus | Peptococcus | rs6918730 | A | G | 0.135 | 0.029 | 1.15E-06 | 21.809 |
| genus | RuminococcaceaeUCG011 | rs7553154 | T | C | -0.117 | 0.025 | 3.69E-06 | 21.808 |
| genus | LachnospiraceaeNK4A136group | rs301776 | T | G | 0.050 | 0.011 | 3.21E-06 | 21.808 |
| genus | Eisenbergiella | rs7179235 | T | G | 0.105 | 0.022 | 3.14E-06 | 21.807 |
| genus | Odoribacter | rs76840762 | C | T | -0.126 | 0.027 | 3.11E-06 | 21.806 |
| genus | RuminococcaceaeUCG005 | rs68105234 | G | T | 0.149 | 0.032 | 3.67E-06 | 21.805 |
| genus | Eisenbergiella | rs4462860 | A | G | 0.094 | 0.020 | 4.16E-06 | 21.805 |
| genus | Oscillibacter | rs9380083 | A | G | -0.070 | 0.015 | 3.52E-06 | 21.805 |
| genus | Ruminiclostridium5 | rs2108642 | A | C | -0.050 | 0.011 | 3.16E-06 | 21.804 |
| genus | LachnospiraceaeUCG008 | rs29374 | G | A | 0.082 | 0.018 | 3.41E-06 | 21.804 |
| genus | Paraprevotella | rs12226783 | T | G | -0.135 | 0.029 | 5.79E-06 | 21.803 |
| genus | Fusicatenibacter | rs9727026 | A | G | -0.058 | 0.012 | 2.71E-06 | 21.803 |
| genus | Anaerostipes | rs11197325 | T | C | -0.051 | 0.011 | 3.43E-06 | 21.803 |
| genus | Haemophilus | rs10781340 | A | G | 0.095 | 0.020 | 4.32E-06 | 21.803 |
| genus | Ruminococcus1 | rs11214881 | T | C | 0.080 | 0.017 | 1.65E-06 | 21.803 |
| genus | Anaerostipes | rs2769388 | C | T | -0.051 | 0.011 | 3.40E-06 | 21.803 |
| genus | Sellimonas | rs2570237 | T | C | 0.117 | 0.025 | 3.21E-06 | 21.803 |
| genus | Bifidobacterium | rs7581814 | C | G | 0.074 | 0.016 | 3.59E-06 | 21.801 |
| genus | Escherichia | rs113127095 | G | A | 0.151 | 0.032 | 3.33E-06 | 21.799 |
| genus | Anaerostipes | rs2769391 | G | A | -0.051 | 0.011 | 3.41E-06 | 21.798 |
| genus | Clostridiumsensustricto1 | rs941434 | C | A | -0.183 | 0.039 | 3.22E-06 | 21.798 |
| genus | Allisonella | rs3827485 | G | A | 0.140 | 0.030 | 3.24E-06 | 21.796 |
| genus | LachnospiraceaeND3007group | rs2006002 | G | C | -0.054 | 0.012 | 3.05E-06 | 21.796 |
| genus | RuminococcaceaeUCG004 | rs11164635 | A | T | 0.080 | 0.017 | 2.85E-06 | 21.795 |
| genus | Bifidobacterium | rs309150 | T | A | 0.074 | 0.016 | 3.49E-06 | 21.793 |
| genus | RuminococcaceaeUCG011 | rs2729556 | T | C | -0.109 | 0.023 | 3.19E-06 | 21.793 |
| genus |  | rs876734 | T | C | -0.062 | 0.013 | 2.89E-06 | 21.793 |
| genus | Slackia | rs16894137 | T | C | -0.123 | 0.026 | 2.71E-06 | 21.791 |
| genus | Lachnoclostridium | rs61915992 | T | A | 0.080 | 0.017 | 2.67E-06 | 21.791 |
| genus | Veillonella | rs7845890 | A | T | -0.077 | 0.016 | 4.10E-06 | 21.790 |
| genus | RuminococcaceaeUCG011 | rs10913768 | T | G | -0.116 | 0.025 | 3.79E-06 | 21.789 |
| genus | Prevotella9 | rs11678834 | C | A | -0.138 | 0.030 | 3.49E-06 | 21.788 |
| genus | Ruminiclostridium5 | rs10848571 | G | T | 0.050 | 0.011 | 3.12E-06 | 21.788 |
| genus |  | rs1581240 | A | G | -0.172 | 0.037 | 4.10E-06 | 21.788 |
| genus | Coprococcus2 | rs11159771 | A | C | -0.067 | 0.014 | 3.36E-06 | 21.787 |
| genus | ChristensenellaceaeR | rs7250563 | T | C | 0.054 | 0.012 | 3.36E-06 | 21.786 |
| genus | Gordonibacter | rs111579754 | G | C | 0.176 | 0.038 | 2.97E-06 | 21.785 |
| genus | Butyrivibrio | rs28552674 | C | T | 0.182 | 0.039 | 3.31E-06 | 21.781 |
| genus | Butyrivibrio | rs12758637 | G | A | 0.182 | 0.039 | 3.31E-06 | 21.781 |
| genus | Bacteroides | rs2023437 | C | T | -0.078 | 0.017 | 5.02E-06 | 21.780 |
| genus |  | rs11876297 | C | T | 0.131 | 0.028 | 2.67E-06 | 21.779 |
| genus | Bifidobacterium | rs3099428 | C | G | 0.073 | 0.016 | 3.37E-06 | 21.778 |
| genus | Enterorhabdus | rs3110504 | C | T | 0.097 | 0.021 | 3.37E-06 | 21.778 |
| genus | Anaerotruncus | rs9603209 | T | C | 0.077 | 0.017 | 1.74E-06 | 21.777 |
| genus | Alistipes | rs17675579 | A | G | -0.051 | 0.011 | 3.38E-06 | 21.775 |
| genus | Marvinbryantia | rs2724814 | G | A | -0.077 | 0.016 | 3.90E-06 | 21.772 |
| genus | FamilyXIIIUCG001 | rs745349 | G | C | -0.062 | 0.013 | 2.78E-06 | 21.772 |
| genus | Butyrivibrio | rs8072835 | A | G | 0.120 | 0.026 | 2.54E-06 | 21.770 |
| genus | ChristensenellaceaeR | rs7250370 | C | A | 0.054 | 0.012 | 3.42E-06 | 21.769 |
| genus | Holdemanella | rs1926302 | A | G | -0.108 | 0.023 | 7.50E-06 | 21.768 |
| genus | Coprococcus2 | rs72680320 | C | T | -0.065 | 0.014 | 2.27E-06 | 21.766 |
| genus | Bilophila | rs6793291 | A | C | 0.113 | 0.024 | 3.11E-06 | 21.765 |
| genus | FamilyXIIIUCG001 | rs116979587 | A | T | -0.122 | 0.026 | 3.05E-06 | 21.764 |
| genus | RuminococcaceaeUCG011 | rs10913771 | T | C | -0.116 | 0.025 | 3.85E-06 | 21.763 |
| genus | Lachnoclostridium | rs61915982 | C | T | 0.081 | 0.017 | 3.18E-06 | 21.762 |
| genus | FamilyXIIIAD3011group | rs62029761 | G | A | 0.129 | 0.028 | 3.89E-06 | 21.762 |
| genus | Paraprevotella | rs9547278 | G | A | -0.103 | 0.022 | 1.52E-06 | 21.761 |
| genus | LachnospiraceaeUCG008 | rs7943800 | T | C | -0.090 | 0.019 | 3.45E-06 | 21.761 |
| genus | RuminococcaceaeUCG011 | rs10913744 | C | G | -0.117 | 0.025 | 3.46E-06 | 21.759 |
| genus | RuminococcaceaeUCG004 | rs516741 | G | T | -0.076 | 0.016 | 4.77E-06 | 21.757 |
| genus | Erysipelatoclostridium | rs622418 | G | A | -0.067 | 0.014 | 3.68E-06 | 21.756 |
| genus | RuminococcaceaeUCG003 | rs633372 | G | A | 0.055 | 0.012 | 3.47E-06 | 21.756 |
| genus |  | rs1921839 | T | A | -0.076 | 0.016 | 1.82E-06 | 21.756 |
| genus | RuminococcaceaeUCG005 | rs2893870 | A | G | -0.073 | 0.016 | 5.15E-06 | 21.755 |
| genus | ErysipelotrichaceaeUCG003 | rs7235864 | A | G | -0.100 | 0.021 | 3.06E-06 | 21.753 |
| genus | FamilyXIIIAD3011group | rs12314465 | G | A | -0.092 | 0.020 | 2.86E-06 | 21.752 |
| genus | Streptococcus | rs11925530 | A | C | 0.106 | 0.023 | 4.01E-06 | 21.752 |
| genus | Akkermansia | rs2169719 | A | G | 0.068 | 0.015 | 3.09E-06 | 21.751 |
| genus | Allisonella | rs12555277 | C | G | 0.140 | 0.030 | 3.73E-06 | 21.750 |
| genus |  | rs1999224 | T | G | -0.095 | 0.020 | 3.75E-06 | 21.749 |
| genus | Hungatella | rs35171672 | G | A | -0.156 | 0.033 | 4.90E-06 | 21.748 |
| genus | Desulfovibrio | rs9851810 | A | G | 0.117 | 0.025 | 5.64E-06 | 21.748 |
| genus | Bifidobacterium | rs4245838 | C | T | 0.110 | 0.024 | 1.98E-06 | 21.746 |
| genus | RuminococcaceaeUCG011 | rs4495640 | T | C | -0.117 | 0.025 | 3.77E-06 | 21.745 |
| genus | Ruminiclostridium9 | rs62287884 | G | A | -0.054 | 0.012 | 3.50E-06 | 21.745 |
| genus |  | rs139755 | G | A | -0.080 | 0.017 | 3.11E-06 | 21.744 |
| genus | LachnospiraceaeFCS020group | rs12866856 | T | C | -0.061 | 0.013 | 3.07E-06 | 21.744 |
| genus |  | rs679574 | C | G | -0.050 | 0.011 | 3.26E-06 | 21.743 |
| genus | Paraprevotella | rs10044793 | A | C | -0.099 | 0.021 | 3.95E-06 | 21.742 |
| genus | Allisonella | rs4463540 | G | A | 0.140 | 0.030 | 3.97E-06 | 21.741 |
| genus | Intestinibacter | rs1476854 | A | G | 0.067 | 0.014 | 3.54E-06 | 21.741 |
| genus | Haemophilus | rs4822728 | C | T | 0.071 | 0.015 | 3.48E-06 | 21.740 |
| genus | Veillonella | rs2831941 | A | G | -0.076 | 0.016 | 3.47E-06 | 21.740 |
| genus |  | rs4875590 | T | A | -0.106 | 0.023 | 3.08E-06 | 21.738 |
| genus | Parabacteroides | rs10119172 | G | C | -0.068 | 0.015 | 2.62E-06 | 21.737 |
| genus | RuminococcaceaeUCG013 | rs76973485 | T | G | 0.195 | 0.042 | 3.35E-06 | 21.735 |
| genus | RuminococcaceaeUCG004 | rs511258 | A | G | -0.076 | 0.016 | 4.52E-06 | 21.735 |
| genus | Veillonella | rs62376424 | T | C | -0.076 | 0.016 | 3.65E-06 | 21.733 |
| genus | Ruminiclostridium9 | rs12040548 | T | G | 0.057 | 0.012 | 3.15E-06 | 21.733 |
| genus | Roseburia | rs9300744 | T | C | -0.059 | 0.013 | 4.75E-06 | 21.733 |
| genus | RuminococcaceaeUCG004 | rs11711662 | A | G | 0.072 | 0.016 | 3.70E-06 | 21.733 |
| genus | Parasutterella | rs7967869 | G | A | -0.075 | 0.016 | 3.78E-06 | 21.732 |
| genus | Anaerostipes | rs6854026 | C | T | -0.051 | 0.011 | 3.20E-06 | 21.732 |
| genus | Intestinibacter | rs7952361 | T | C | -0.139 | 0.030 | 3.36E-06 | 21.731 |
| genus |  | rs632111 | A | G | -0.050 | 0.011 | 3.14E-06 | 21.729 |
| genus | Coprobacter | rs3828477 | T | G | -0.091 | 0.020 | 2.89E-06 | 21.728 |
| genus | Parasutterella | rs7669627 | T | A | 0.062 | 0.013 | 3.54E-06 | 21.726 |
| genus | Lactobacillus | rs59849860 | A | G | -0.104 | 0.022 | 4.62E-06 | 21.726 |
| genus | RuminococcaceaeUCG011 | rs12409903 | A | G | -0.116 | 0.025 | 3.95E-06 | 21.725 |
| genus | FamilyXIIIAD3011group | rs13089490 | T | G | 0.057 | 0.012 | 3.35E-06 | 21.724 |
| genus | Sellimonas | rs2622765 | C | T | -0.120 | 0.026 | 4.49E-06 | 21.723 |
| genus | LachnospiraceaeUCG001 | rs7029467 | G | A | -0.067 | 0.014 | 2.74E-06 | 21.722 |
| genus | Anaerostipes | rs2769371 | G | T | -0.051 | 0.011 | 3.52E-06 | 21.720 |
| genus | RuminococcaceaeUCG011 | rs12753310 | G | A | -0.117 | 0.025 | 3.86E-06 | 21.720 |
| genus | LachnospiraceaeND3007group | rs9931821 | T | C | -0.054 | 0.012 | 3.55E-06 | 21.720 |
| genus | Paraprevotella | rs11655964 | T | C | -0.081 | 0.017 | 4.57E-06 | 21.720 |
| genus | Anaerostipes | rs2250761 | C | T | -0.051 | 0.011 | 3.53E-06 | 21.719 |
| genus | Erysipelatoclostridium | rs340991 | G | A | -0.074 | 0.016 | 3.75E-06 | 21.718 |
| genus | Desulfovibrio | rs2853179 | T | C | 0.081 | 0.017 | 2.42E-06 | 21.718 |
| genus |  | rs281395 | C | G | 0.051 | 0.011 | 3.03E-06 | 21.717 |
| genus | LachnospiraceaeUCG001 | rs2639368 | C | T | 0.087 | 0.019 | 4.80E-06 | 21.713 |
| genus | Ruminococcus1 | rs4148893 | C | G | -0.052 | 0.011 | 3.95E-06 | 21.712 |
| genus | RuminococcaceaeUCG011 | rs11582754 | C | G | -0.116 | 0.025 | 3.96E-06 | 21.712 |
| genus | ChristensenellaceaeR | rs10412719 | C | T | 0.054 | 0.012 | 3.20E-06 | 21.711 |
| genus | LachnospiraceaeUCG001 | rs7018735 | T | C | -0.067 | 0.014 | 2.74E-06 | 21.711 |
| genus | RuminococcaceaeUCG005 | rs394449 | T | A | 0.069 | 0.015 | 2.60E-06 | 21.710 |
| genus | Streptococcus | rs7970539 | T | C | -0.086 | 0.018 | 3.28E-06 | 21.709 |
| genus | Anaerostipes | rs2804190 | T | G | -0.051 | 0.011 | 3.53E-06 | 21.709 |
| genus | RuminococcaceaeUCG003 | rs6757232 | A | T | -0.067 | 0.014 | 5.05E-06 | 21.709 |
| genus | Coprococcus1 | rs1576241 | G | A | -0.051 | 0.011 | 3.33E-06 | 21.708 |
| genus | Oxalobacter | rs11108500 | G | A | -0.199 | 0.043 | 3.74E-06 | 21.708 |
| genus | LachnospiraceaeUCG008 | rs29367 | C | T | 0.079 | 0.017 | 3.64E-06 | 21.708 |
| genus | Ruminiclostridium9 | rs11710813 | C | T | -0.059 | 0.013 | 4.60E-06 | 21.707 |
| genus | RuminococcaceaeUCG011 | rs4418554 | T | A | -0.116 | 0.025 | 3.93E-06 | 21.706 |
| genus | Butyrivibrio | rs12744713 | A | G | 0.174 | 0.037 | 3.97E-06 | 21.704 |
| genus | Bifidobacterium | rs4988201 | A | G | 0.075 | 0.016 | 3.63E-06 | 21.703 |
| genus |  | rs2962235 | T | C | -0.083 | 0.018 | 4.57E-06 | 21.703 |
| genus | Lachnoclostridium | rs2024615 | G | A | -0.050 | 0.011 | 3.66E-06 | 21.702 |
| genus | Sutterella | rs4270166 | T | C | 0.061 | 0.013 | 3.09E-06 | 21.700 |
| genus | RuminococcaceaeUCG004 | rs11164636 | C | A | 0.080 | 0.017 | 2.99E-06 | 21.700 |
| genus | Butyricimonas | rs1416263 | G | T | 0.067 | 0.014 | 2.47E-06 | 21.699 |
| genus | RuminococcaceaeUCG004 | rs17127270 | G | A | 0.090 | 0.019 | 3.65E-06 | 21.698 |
| genus | Bilophila | rs4506138 | C | T | 0.064 | 0.014 | 4.54E-06 | 21.697 |
| genus | Alistipes | rs2311504 | G | A | -0.051 | 0.011 | 3.43E-06 | 21.696 |
| genus | RuminococcaceaeUCG002 | rs12463378 | G | A | -0.052 | 0.011 | 2.96E-06 | 21.694 |
| genus | Parasutterella | rs10847032 | A | T | -0.074 | 0.016 | 3.84E-06 | 21.692 |
| genus | Anaerofilum | rs784717 | G | A | 0.096 | 0.021 | 3.23E-06 | 21.691 |
| genus |  | rs16960159 | G | C | -0.157 | 0.034 | 4.10E-06 | 21.691 |
| genus |  | rs12434634 | G | A | 0.071 | 0.015 | 7.73E-06 | 21.691 |
| genus |  | rs78208790 | A | G | -0.076 | 0.016 | 1.66E-06 | 21.690 |
| genus | Intestinibacter | rs10790751 | G | A | -0.138 | 0.030 | 3.43E-06 | 21.689 |
| genus | RuminococcaceaeUCG011 | rs11582995 | A | G | -0.116 | 0.025 | 3.92E-06 | 21.688 |
| genus | Marvinbryantia | rs2863363 | G | A | 0.063 | 0.014 | 3.11E-06 | 21.688 |
| genus | Prevotella9 | rs1193288 | A | G | -0.071 | 0.015 | 2.78E-06 | 21.686 |
| genus | RuminococcaceaeUCG003 | rs11611374 | T | A | 0.073 | 0.016 | 1.76E-06 | 21.685 |
| genus | Ruminococcus2 | rs4073386 | C | A | 0.065 | 0.014 | 4.02E-06 | 21.684 |
| genus | LachnospiraceaeUCG010 | rs12553325 | C | T | 0.087 | 0.019 | 4.21E-06 | 21.684 |
| genus |  | rs3124783 | G | A | -0.116 | 0.025 | 2.67E-06 | 21.682 |
| genus | Allisonella | rs1901739 | G | T | 0.116 | 0.025 | 3.59E-06 | 21.681 |
| genus | RuminococcaceaeUCG014 | . | G | C | 0.133 | 0.029 | 4.05E-06 | 21.681 |
| genus | Anaerostipes | rs9420197 | G | A | -0.051 | 0.011 | 3.61E-06 | 21.681 |
| genus | Bifidobacterium | rs2090660 | C | T | 0.064 | 0.014 | 4.13E-06 | 21.680 |
| genus | Oxalobacter | rs12002250 | C | A | 0.217 | 0.047 | 1.42E-06 | 21.679 |
| genus | Oxalobacter | rs12004269 | T | G | 0.217 | 0.047 | 1.42E-06 | 21.679 |
| genus |  | rs12257053 | T | C | -0.167 | 0.036 | 2.59E-06 | 21.679 |
| genus | RuminococcaceaeUCG014 | rs56105232 | A | G | 0.139 | 0.030 | 2.91E-06 | 21.678 |
| genus | Anaerostipes | rs11197294 | C | T | -0.052 | 0.011 | 4.06E-06 | 21.678 |
| genus | Oxalobacter | rs7614504 | T | G | 0.151 | 0.032 | 5.07E-06 | 21.676 |
| genus |  | rs6861985 | T | C | -0.107 | 0.023 | 3.07E-06 | 21.676 |
| genus | Anaerostipes | rs3900776 | A | G | -0.110 | 0.024 | 2.75E-06 | 21.675 |
| genus | Paraprevotella | rs2159036 | A | C | -0.081 | 0.017 | 5.34E-06 | 21.673 |
| genus | FamilyXIIIAD3011group | rs1997158 | C | A | -0.090 | 0.019 | 3.38E-06 | 21.671 |
| genus | Oscillibacter | rs11624178 | G | A | -0.072 | 0.016 | 3.02E-06 | 21.670 |
| genus |  | rs2939710 | T | C | 0.091 | 0.020 | 3.36E-06 | 21.669 |
| genus | Bifidobacterium | rs3098104 | T | A | 0.075 | 0.016 | 5.25E-06 | 21.667 |
| genus | FamilyXIIIUCG001 | rs34768213 | T | C | -0.063 | 0.013 | 3.29E-06 | 21.667 |
| genus |  | rs62348779 | C | T | -0.201 | 0.043 | 3.78E-06 | 21.666 |
| genus | Bifidobacterium | rs3112496 | C | T | 0.073 | 0.016 | 3.02E-06 | 21.665 |
| genus | Anaerostipes | rs28414370 | G | C | -0.052 | 0.011 | 3.98E-06 | 21.664 |
| genus | Prevotella9 | rs7232121 | C | G | 0.067 | 0.014 | 3.76E-06 | 21.663 |
| genus | Paraprevotella | rs79933388 | A | T | -0.135 | 0.029 | 6.67E-06 | 21.662 |
| genus | Intestinibacter | rs6845847 | C | T | 0.068 | 0.015 | 4.40E-06 | 21.660 |
| genus | LachnospiraceaeUCG008 | rs57846754 | G | A | -0.110 | 0.024 | 3.35E-06 | 21.660 |
| genus | Streptococcus | rs1918540 | A | G | 0.060 | 0.013 | 2.44E-06 | 21.659 |
| genus | Peptococcus | rs1530244 | C | T | -0.106 | 0.023 | 3.94E-06 | 21.657 |
| genus | Parasutterella | rs6551864 | C | G | 0.062 | 0.013 | 3.67E-06 | 21.657 |
| genus | Butyrivibrio | rs8075758 | C | A | 0.121 | 0.026 | 2.83E-06 | 21.655 |
| genus | Intestinibacter | rs552646 | G | A | -0.138 | 0.030 | 3.74E-06 | 21.654 |
| genus | LachnospiraceaeND3007group | rs28491092 | A | G | -0.054 | 0.012 | 3.58E-06 | 21.654 |
| genus | Butyrivibrio | rs12079328 | A | T | 0.174 | 0.037 | 4.37E-06 | 21.653 |
| genus | Parasutterella | rs35055552 | C | T | 0.110 | 0.024 | 3.35E-06 | 21.653 |
| genus | Hungatella | rs67484468 | C | T | -0.156 | 0.033 | 5.00E-06 | 21.653 |
| genus | Streptococcus | rs17708276 | G | A | -0.079 | 0.017 | 3.04E-06 | 21.652 |
| genus | LachnospiraceaeUCG008 | rs36053744 | C | G | 0.099 | 0.021 | 3.40E-06 | 21.652 |
| genus | Odoribacter | rs75953891 | C | T | -0.125 | 0.027 | 3.41E-06 | 21.651 |
| genus | Ruminiclostridium5 | rs8053158 | G | A | -0.074 | 0.016 | 5.90E-06 | 21.651 |
| genus | Blautia | rs28609389 | T | C | 0.056 | 0.012 | 2.65E-06 | 21.649 |
| genus | LachnospiraceaeUCG008 | rs971257 | A | T | -0.079 | 0.017 | 3.25E-06 | 21.649 |
| genus | Streptococcus | rs80003643 | T | C | -0.085 | 0.018 | 3.74E-06 | 21.648 |
| genus | Anaerostipes | rs12765024 | G | A | -0.051 | 0.011 | 3.67E-06 | 21.648 |
| genus |  | rs949971 | G | T | -0.054 | 0.012 | 3.29E-06 | 21.646 |
| genus | Streptococcus | rs10448310 | G | A | -0.052 | 0.011 | 3.31E-06 | 21.646 |
| genus | Bifidobacterium | rs4988163 | G | T | 0.075 | 0.016 | 3.82E-06 | 21.645 |
| genus | Flavonifractor | rs4755897 | C | A | 0.075 | 0.016 | 2.57E-06 | 21.645 |
| genus | Parasutterella | rs10012139 | T | C | 0.062 | 0.013 | 3.80E-06 | 21.645 |
| genus | Lachnospira | rs820968 | T | C | 0.051 | 0.011 | 3.29E-06 | 21.645 |
| genus | RikenellaceaeRC9gutgroup | rs2998141 | C | T | -0.136 | 0.029 | 4.42E-06 | 21.644 |
| genus | Paraprevotella | rs3008582 | C | T | 0.106 | 0.023 | 4.36E-06 | 21.643 |
| genus |  | rs11592691 | A | G | -0.168 | 0.036 | 3.50E-06 | 21.642 |
| genus | Ruminiclostridium9 | rs1105488 | C | T | 0.057 | 0.012 | 3.38E-06 | 21.641 |
| genus |  | rs10248854 | A | C | -0.053 | 0.011 | 4.21E-06 | 21.641 |
| genus | ChristensenellaceaeR | rs8111067 | A | G | 0.054 | 0.012 | 3.32E-06 | 21.639 |
| genus | Hungatella | rs68191502 | C | T | -0.156 | 0.033 | 5.09E-06 | 21.637 |
| genus | Turicibacter | rs59351397 | T | C | -0.113 | 0.024 | 4.58E-06 | 21.637 |
| genus | Anaerostipes | rs1410305 | G | A | -0.051 | 0.011 | 3.70E-06 | 21.635 |
| genus | Prevotella9 | rs2672262 | C | T | -0.070 | 0.015 | 2.79E-06 | 21.635 |
| genus | Peptococcus | rs11030569 | T | A | -0.174 | 0.037 | 3.13E-06 | 21.634 |
| genus | Parasutterella | rs11615654 | G | A | 0.063 | 0.013 | 2.79E-06 | 21.634 |
| genus | Ruminiclostridium9 | rs4805062 | T | A | -0.092 | 0.020 | 4.76E-06 | 21.634 |
| genus | Anaerofilum | rs784716 | G | A | 0.096 | 0.021 | 3.33E-06 | 21.633 |
| genus | LachnospiraceaeUCG008 | rs10833132 | C | G | -0.090 | 0.019 | 3.65E-06 | 21.631 |
| genus | Allisonella | rs3747510 | T | C | 0.139 | 0.030 | 3.92E-06 | 21.631 |
| genus | Oxalobacter | rs7622134 | G | T | 0.151 | 0.032 | 5.20E-06 | 21.628 |
| genus | Hungatella | rs13126727 | A | G | -0.156 | 0.033 | 4.99E-06 | 21.626 |
| genus | Howardella | rs609894 | G | C | -0.111 | 0.024 | 3.90E-06 | 21.625 |
| genus | Butyrivibrio | rs11153292 | A | G | -0.112 | 0.024 | 3.77E-06 | 21.624 |
| genus |  | rs72836424 | T | C | -0.140 | 0.030 | 2.62E-06 | 21.624 |
| genus | Bilophila | rs3818204 | G | A | 0.078 | 0.017 | 3.64E-06 | 21.623 |
| genus | Eggerthella | rs2893553 | G | A | -0.092 | 0.020 | 3.67E-06 | 21.622 |
| genus | Turicibacter | rs11868071 | G | A | 0.105 | 0.023 | 3.20E-06 | 21.622 |
| genus | RuminococcaceaeUCG011 | rs34409173 | A | G | -0.138 | 0.030 | 3.41E-06 | 21.618 |
| genus | Oxalobacter | rs1569853 | C | T | -0.138 | 0.030 | 3.65E-06 | 21.617 |
| genus | LachnospiraceaeFCS020group | rs1010830 | T | G | -0.056 | 0.012 | 3.88E-06 | 21.617 |
| genus | Parasutterella | rs8039785 | G | T | 0.062 | 0.013 | 3.62E-06 | 21.615 |
| genus | Intestinibacter | rs4698730 | A | G | 0.067 | 0.014 | 3.74E-06 | 21.611 |
| genus | Butyrivibrio | rs11657793 | G | T | 0.120 | 0.026 | 2.88E-06 | 21.611 |
| genus | Allisonella | rs6896529 | G | A | 0.116 | 0.025 | 3.73E-06 | 21.610 |
| genus | Butyricimonas | rs1466575 | C | T | 0.066 | 0.014 | 2.31E-06 | 21.610 |
| genus | RuminococcaceaeUCG003 | rs35866622 | C | T | 0.057 | 0.012 | 4.02E-06 | 21.609 |
| genus | Olsenella | rs1555528 | T | A | -0.110 | 0.024 | 4.10E-06 | 21.607 |
| genus | Ruminiclostridium5 | rs11010058 | C | T | -0.053 | 0.011 | 3.08E-06 | 21.607 |
| genus | CandidatusSoleaferrea | rs830149 | G | C | 0.185 | 0.040 | 9.58E-06 | 21.606 |
| genus | Allisonella | rs7859665 | A | G | 0.125 | 0.027 | 4.05E-06 | 21.605 |
| genus | Eisenbergiella | rs12421743 | C | G | -0.117 | 0.025 | 3.73E-06 | 21.603 |
| genus | RuminococcaceaeUCG010 | rs686403 | T | G | -0.058 | 0.012 | 3.27E-06 | 21.603 |
| genus | Lachnoclostridium | rs789029 | T | C | -0.064 | 0.014 | 3.75E-06 | 21.603 |
| genus | Parasutterella | rs883312 | C | T | 0.062 | 0.013 | 3.46E-06 | 21.599 |
| genus | Allisonella | rs4532668 | C | A | 0.119 | 0.026 | 5.01E-06 | 21.598 |
| genus | Adlercreutzia | rs2850883 | A | G | -0.117 | 0.025 | 3.09E-06 | 21.598 |
| genus | ChristensenellaceaeR | rs8103090 | T | G | 0.054 | 0.012 | 3.40E-06 | 21.598 |
| genus | Parasutterella | rs883311 | G | A | 0.062 | 0.013 | 3.50E-06 | 21.596 |
| genus |  | rs1948423 | A | T | -0.109 | 0.023 | 3.49E-06 | 21.596 |
| genus |  | rs28400014 | G | C | -0.049 | 0.011 | 3.21E-06 | 21.595 |
| genus | Ruminococcus2 | rs2997412 | G | A | -0.057 | 0.012 | 4.22E-06 | 21.594 |
| genus | Hungatella | rs66516986 | G | A | -0.156 | 0.033 | 5.04E-06 | 21.594 |
| genus | RuminococcaceaeUCG011 | rs12760048 | C | G | -0.116 | 0.025 | 4.15E-06 | 21.591 |
| genus | Veillonella | rs11993500 | G | A | -0.077 | 0.016 | 4.62E-06 | 21.591 |
| genus | Oscillibacter | rs3731748 | G | A | 0.097 | 0.021 | 4.51E-06 | 21.591 |
| genus | Streptococcus | rs11110079 | C | A | -0.083 | 0.018 | 3.21E-06 | 21.590 |
| genus | Sutterella | rs62501473 | A | G | 0.069 | 0.015 | 5.52E-06 | 21.588 |
| genus | Anaerostipes | rs2804214 | G | C | -0.051 | 0.011 | 3.78E-06 | 21.588 |
| genus | Eggerthella | rs1860519 | G | C | -0.093 | 0.020 | 3.40E-06 | 21.587 |
| genus | CandidatusSoleaferrea | rs10108780 | G | A | -0.093 | 0.020 | 3.64E-06 | 21.587 |
| genus | ErysipelotrichaceaeUCG003 | rs59104037 | G | A | -0.095 | 0.020 | 4.48E-06 | 21.587 |
| genus | ChristensenellaceaeR | rs8110494 | C | A | 0.054 | 0.012 | 3.45E-06 | 21.585 |
| genus | LachnospiraceaeUCG004 | rs10226380 | G | A | 0.055 | 0.012 | 3.57E-06 | 21.585 |
| genus | RuminococcaceaeUCG013 | rs12485353 | A | G | -0.061 | 0.013 | 4.19E-06 | 21.584 |
| genus | FamilyXIIIAD3011group | rs2999591 | G | A | -0.056 | 0.012 | 3.57E-06 | 21.584 |
| genus | Senegalimassilia | rs7225245 | A | G | 0.079 | 0.017 | 4.18E-06 | 21.583 |
| genus | Bifidobacterium | rs6710892 | G | A | 0.084 | 0.018 | 3.21E-06 | 21.582 |
| genus |  | rs117151453 | C | G | -0.113 | 0.024 | 1.84E-06 | 21.582 |
| genus | Streptococcus | rs7316765 | G | A | -0.057 | 0.012 | 5.79E-06 | 21.581 |
| genus | Bifidobacterium | rs7561565 | T | G | 0.074 | 0.016 | 4.09E-06 | 21.580 |
| genus | Subdoligranulum | rs4347804 | G | A | 0.166 | 0.036 | 2.18E-06 | 21.579 |
| genus | RuminococcaceaeUCG010 | rs12597105 | A | G | 0.067 | 0.014 | 4.87E-06 | 21.579 |
| genus | Streptococcus | rs12533001 | G | T | -0.067 | 0.014 | 3.39E-06 | 21.579 |
| genus |  | rs602662 | G | A | -0.049 | 0.011 | 3.47E-06 | 21.578 |
| genus | Subdoligranulum | rs35940633 | A | G | -0.051 | 0.011 | 4.22E-06 | 21.577 |
| genus | Veillonella | rs2013594 | C | T | -0.072 | 0.016 | 3.42E-06 | 21.577 |
| genus | Hungatella | rs35106888 | T | G | -0.156 | 0.033 | 5.19E-06 | 21.576 |
| genus | LachnospiraceaeNK4A136group | rs8011015 | T | G | -0.053 | 0.011 | 4.88E-06 | 21.575 |
| genus | Peptococcus | rs2786718 | A | T | 0.088 | 0.019 | 3.61E-06 | 21.573 |
| genus | Holdemania | rs956437 | G | A | 0.074 | 0.016 | 4.28E-06 | 21.570 |
| genus | RuminococcaceaeUCG011 | rs7524128 | G | C | -0.116 | 0.025 | 4.19E-06 | 21.569 |
| genus | RuminococcaceaeUCG002 | rs10927425 | T | C | -0.069 | 0.015 | 2.20E-06 | 21.568 |
| genus |  | rs2213117 | G | T | 0.088 | 0.019 | 4.21E-06 | 21.568 |
| genus | RuminococcaceaeUCG002 | rs59880871 | G | A | -0.056 | 0.012 | 3.69E-06 | 21.568 |
| genus | LachnospiraceaeUCG001 | rs573933 | C | T | -0.108 | 0.023 | 3.11E-06 | 21.565 |
| genus | Prevotella9 | rs73127833 | G | A | -0.090 | 0.019 | 3.37E-06 | 21.565 |
| genus | Anaerostipes | rs1328008 | A | G | -0.051 | 0.011 | 3.87E-06 | 21.564 |
| genus | Blautia | rs13330279 | A | G | 0.118 | 0.025 | 5.38E-06 | 21.563 |
| genus | ChristensenellaceaeR | rs8100274 | T | C | 0.054 | 0.012 | 3.46E-06 | 21.561 |
| genus | Peptococcus | rs9409346 | A | T | 0.088 | 0.019 | 3.62E-06 | 21.560 |
| genus | Blautia | rs66498553 | A | G | 0.057 | 0.012 | 3.15E-06 | 21.559 |
| genus | Butyricicoccus | rs12585793 | C | T | -0.262 | 0.056 | 5.79E-06 | 21.558 |
| genus | RuminococcaceaeUCG011 | rs11751952 | T | A | -0.138 | 0.030 | 3.53E-06 | 21.557 |
| genus | RuminococcaceaeUCG011 | rs11753518 | G | A | -0.138 | 0.030 | 3.53E-06 | 21.557 |
| genus | Oscillibacter | rs1430431 | C | G | -0.072 | 0.016 | 3.43E-06 | 21.557 |
| genus | Phascolarctobacterium | rs12618201 | G | A | 0.064 | 0.014 | 3.38E-06 | 21.556 |
| genus | Ruminococcus1 | rs12293744 | C | T | 0.079 | 0.017 | 1.79E-06 | 21.556 |
| genus | RuminococcaceaeUCG003 | rs7595179 | T | C | 0.073 | 0.016 | 5.13E-06 | 21.555 |
| genus |  | rs73139629 | C | A | -0.115 | 0.025 | 5.36E-06 | 21.555 |
| genus | Ruminococcus1 | rs10769158 | T | C | -0.051 | 0.011 | 3.43E-06 | 21.554 |
| genus | Ruminiclostridium5 | rs4765843 | C | T | -0.050 | 0.011 | 3.82E-06 | 21.553 |
| genus |  | rs4862235 | A | G | 0.105 | 0.023 | 3.73E-06 | 21.553 |
| genus | Lactococcus | rs66638090 | C | T | 0.112 | 0.024 | 2.55E-06 | 21.553 |
| genus | Victivallis | rs7624585 | T | C | 0.116 | 0.025 | 3.64E-06 | 21.553 |
| genus | Barnesiella | rs12674064 | T | A | -0.059 | 0.013 | 3.56E-06 | 21.553 |
| genus | Anaerofilum | rs4971306 | A | G | 0.099 | 0.021 | 3.99E-06 | 21.553 |
| genus | Oscillibacter | rs2288325 | A | T | 0.097 | 0.021 | 4.58E-06 | 21.553 |
| genus | Oxalobacter | rs10868229 | G | A | 0.098 | 0.021 | 4.17E-06 | 21.552 |
| genus |  | rs3011257 | G | T | -0.116 | 0.025 | 2.87E-06 | 21.552 |
| genus | RuminococcaceaeUCG013 | rs16918863 | C | A | 0.111 | 0.024 | 4.16E-06 | 21.552 |
| genus |  | rs6762473 | A | C | 0.052 | 0.011 | 4.26E-06 | 21.552 |
| genus | Bifidobacterium | rs61253125 | T | C | 0.074 | 0.016 | 3.89E-06 | 21.551 |
| genus | Ruminiclostridium9 | rs62289792 | C | T | -0.052 | 0.011 | 3.89E-06 | 21.551 |
| genus | Adlercreutzia | rs4869118 | T | C | -0.073 | 0.016 | 3.51E-06 | 21.551 |
| genus |  | rs10797540 | G | A | 0.050 | 0.011 | 3.53E-06 | 21.551 |
| genus | LachnospiraceaeUCG004 | rs7531340 | G | T | -0.061 | 0.013 | 4.29E-06 | 21.551 |
| genus | RuminococcaceaeUCG011 | rs12749461 | A | G | -0.116 | 0.025 | 3.86E-06 | 21.548 |
| genus | Sellimonas | rs2606133 | G | A | -0.119 | 0.026 | 4.25E-06 | 21.548 |
| genus |  | rs2973291 | A | G | 0.091 | 0.020 | 3.64E-06 | 21.548 |
| genus | LachnospiraceaeNK4A136group | rs1951576 | A | G | -0.053 | 0.012 | 5.28E-06 | 21.547 |
| genus | RuminococcaceaeUCG011 | rs12746578 | A | G | -0.116 | 0.025 | 4.25E-06 | 21.546 |
| genus | Marvinbryantia | rs72948274 | C | A | -0.126 | 0.027 | 3.26E-06 | 21.546 |
| genus | LachnospiraceaeUCG001 | rs1546749 | A | G | 0.086 | 0.019 | 5.17E-06 | 21.545 |
| genus | Erysipelatoclostridium | rs72671245 | G | A | 0.088 | 0.019 | 3.81E-06 | 21.545 |
| genus | LachnospiraceaeNK4A136group | rs35110752 | C | A | -0.054 | 0.012 | 4.23E-06 | 21.545 |
| genus | Anaerostipes | rs2804187 | A | T | -0.051 | 0.011 | 3.87E-06 | 21.541 |
| genus | Eggerthella | rs6967524 | C | T | -0.092 | 0.020 | 3.89E-06 | 21.540 |
| genus |  | rs2973292 | G | A | 0.091 | 0.020 | 3.60E-06 | 21.540 |
| genus | RuminococcaceaeUCG011 | rs12752452 | A | G | -0.116 | 0.025 | 4.25E-06 | 21.540 |
| genus | RuminococcaceaeUCG011 | rs11583817 | T | A | -0.116 | 0.025 | 4.25E-06 | 21.540 |
| genus | RuminococcaceaeUCG011 | rs12726506 | G | T | -0.116 | 0.025 | 4.25E-06 | 21.540 |
| genus | RuminococcaceaeUCG011 | rs12732167 | C | T | -0.116 | 0.025 | 4.25E-06 | 21.540 |
| genus | RuminococcaceaeUCG011 | rs12760534 | C | T | -0.116 | 0.025 | 4.25E-06 | 21.540 |
| genus | RuminococcaceaeUCG011 | rs12751260 | T | C | -0.116 | 0.025 | 4.25E-06 | 21.540 |
| genus | RuminococcaceaeUCG003 | rs506897 | G | C | 0.055 | 0.012 | 3.77E-06 | 21.538 |
| genus | Akkermansia | rs4242783 | A | G | 0.069 | 0.015 | 3.00E-06 | 21.537 |
| genus | Peptococcus | rs413827 | A | G | 0.110 | 0.024 | 3.30E-06 | 21.537 |
| genus | Holdemania | rs79436239 | A | G | -0.136 | 0.029 | 3.01E-06 | 21.537 |
| genus | RuminococcaceaeUCG011 | rs11582255 | C | T | -0.116 | 0.025 | 4.29E-06 | 21.536 |
| genus | LachnospiraceaeUCG004 | rs2867517 | T | C | 0.055 | 0.012 | 3.65E-06 | 21.536 |
| genus | LachnospiraceaeFCS020group | rs2862811 | C | T | 0.056 | 0.012 | 3.92E-06 | 21.535 |
| genus | Butyrivibrio | rs11153294 | C | T | -0.111 | 0.024 | 3.92E-06 | 21.535 |
| genus | RikenellaceaeRC9gutgroup | rs17582787 | G | A | -0.158 | 0.034 | 3.55E-06 | 21.534 |
| genus | LachnospiraceaeUCG010 | rs12552025 | C | G | 0.087 | 0.019 | 4.18E-06 | 21.534 |
| genus | RuminococcaceaeUCG003 | rs516316 | G | C | 0.055 | 0.012 | 4.00E-06 | 21.532 |
| genus | Oscillibacter | rs3829747 | C | T | 0.097 | 0.021 | 4.70E-06 | 21.531 |
| genus | FamilyXIIIUCG001 | rs3842897 | A | G | -0.113 | 0.024 | 5.20E-06 | 21.531 |
| genus | Anaerostipes | rs4506550 | G | C | -0.051 | 0.011 | 4.08E-06 | 21.530 |
| genus | Hungatella | rs55683737 | T | C | -0.155 | 0.033 | 5.48E-06 | 21.530 |
| genus | Peptococcus | rs1530243 | A | C | -0.105 | 0.023 | 4.21E-06 | 21.528 |
| genus | Barnesiella | rs79795328 | G | A | -0.082 | 0.018 | 4.23E-06 | 21.527 |
| genus | RuminococcaceaeUCG011 | rs2153008 | A | G | 0.183 | 0.039 | 2.96E-06 | 21.527 |
| genus | Howardella | rs113046682 | G | A | -0.159 | 0.034 | 4.49E-06 | 21.526 |
| genus | Lachnoclostridium | rs6112328 | G | A | -0.049 | 0.011 | 3.55E-06 | 21.525 |
| genus |  | rs11598383 | A | C | -0.169 | 0.036 | 3.75E-06 | 21.525 |
| genus | Odoribacter | rs116117929 | T | C | -0.125 | 0.027 | 3.84E-06 | 21.523 |
| genus | Dorea | rs1899291 | T | C | 0.070 | 0.015 | 4.57E-06 | 21.523 |
| genus | LachnospiraceaeFCS020group | rs7017267 | T | C | -0.056 | 0.012 | 4.01E-06 | 21.520 |
| genus | Eggerthella | rs2223081 | A | G | 0.103 | 0.022 | 3.89E-06 | 21.520 |
| genus | Adlercreutzia | rs1528956 | T | C | -0.081 | 0.018 | 4.09E-06 | 21.519 |
| genus | RuminococcaceaeUCG005 | rs114279581 | G | A | -0.147 | 0.032 | 3.22E-06 | 21.519 |
| genus | Streptococcus | rs6806351 | C | T | -0.063 | 0.014 | 4.94E-06 | 21.515 |
| genus | Bifidobacterium | rs309172 | A | G | 0.072 | 0.016 | 3.23E-06 | 21.515 |
| genus | Parasutterella | rs62005974 | C | T | 0.062 | 0.013 | 3.75E-06 | 21.514 |
| genus | LachnospiraceaeNC2004group | rs34514761 | T | C | 0.114 | 0.025 | 3.86E-06 | 21.514 |
| genus | Butyricimonas | rs1982775 | C | T | 0.066 | 0.014 | 2.91E-06 | 21.514 |
| genus | Akkermansia | rs6832119 | A | G | 0.110 | 0.024 | 3.13E-06 | 21.513 |
| genus | Anaerostipes | rs1576149 | G | T | -0.051 | 0.011 | 3.94E-06 | 21.508 |
| genus | Parasutterella | rs61021642 | A | G | -0.065 | 0.014 | 3.46E-06 | 21.505 |
| genus | FamilyXIIIUCG001 | rs12239541 | G | C | -0.062 | 0.013 | 3.18E-06 | 21.505 |
| genus | Oscillibacter | rs3731751 | T | C | 0.097 | 0.021 | 4.76E-06 | 21.504 |
| genus |  | rs11598977 | A | T | -0.171 | 0.037 | 3.68E-06 | 21.504 |
| genus | Alistipes | rs1583863 | G | A | -0.051 | 0.011 | 3.91E-06 | 21.504 |
| genus | LachnospiraceaeNC2004group | rs35598946 | T | G | 0.097 | 0.021 | 4.59E-06 | 21.503 |
| genus | Allisonella | rs2045731 | G | A | 0.139 | 0.030 | 4.26E-06 | 21.503 |
| genus | Allisonella | rs2045730 | A | G | 0.139 | 0.030 | 4.26E-06 | 21.503 |
| genus | Allisonella | rs35066943 | T | C | 0.139 | 0.030 | 4.26E-06 | 21.503 |
| genus | Paraprevotella | rs12226824 | T | A | -0.135 | 0.029 | 7.06E-06 | 21.501 |
| genus | Anaerostipes | rs2769394 | C | A | -0.051 | 0.011 | 3.96E-06 | 21.501 |
| genus | FamilyXIIIAD3011group | rs1997157 | C | T | -0.090 | 0.019 | 3.70E-06 | 21.498 |
| genus | Parasutterella | rs10773221 | A | G | -0.074 | 0.016 | 4.10E-06 | 21.497 |
| genus | Intestinibacter | rs543221 | G | A | -0.135 | 0.029 | 4.32E-06 | 21.496 |
| genus | Anaerostipes | rs10885738 | A | T | -0.051 | 0.011 | 4.01E-06 | 21.495 |
| genus | Anaerotruncus | rs9603205 | C | A | 0.077 | 0.017 | 1.79E-06 | 21.495 |
| genus | Intestinibacter | rs573844 | G | A | -0.135 | 0.029 | 4.32E-06 | 21.495 |
| genus | Intestinibacter | rs519297 | C | G | -0.135 | 0.029 | 4.32E-06 | 21.495 |
| genus | Intestinibacter | rs485463 | T | C | -0.135 | 0.029 | 4.32E-06 | 21.495 |
| genus | Intestinibacter | rs520082 | G | T | -0.135 | 0.029 | 4.32E-06 | 21.495 |
| genus | Veillonella | rs7645873 | T | A | 0.076 | 0.016 | 3.12E-06 | 21.494 |
| genus | Ruminiclostridium9 | rs1017205 | C | T | -0.092 | 0.020 | 4.82E-06 | 21.494 |
| genus | Prevotella9 | rs11686282 | G | A | -0.137 | 0.030 | 4.23E-06 | 21.490 |
| genus | LachnospiraceaeFCS020group | rs2862810 | C | A | 0.056 | 0.012 | 4.02E-06 | 21.488 |
| genus | Oscillospira | rs72866977 | C | A | -0.131 | 0.028 | 5.63E-06 | 21.488 |
| genus | Ruminiclostridium5 | rs10848565 | C | G | -0.050 | 0.011 | 4.18E-06 | 21.487 |
| genus | Blautia | rs4926264 | C | T | 0.083 | 0.018 | 5.10E-06 | 21.487 |
| genus | Olsenella | rs35225860 | G | A | -0.224 | 0.048 | 3.87E-06 | 21.486 |
| genus | Butyricimonas | rs2591756 | A | T | 0.067 | 0.015 | 2.86E-06 | 21.486 |
| genus | Anaerostipes | rs11197320 | C | T | -0.051 | 0.011 | 3.98E-06 | 21.485 |
| genus | RuminococcaceaeUCG002 | rs12434503 | A | G | 0.053 | 0.012 | 4.35E-06 | 21.485 |
| genus | RuminococcaceaeUCG003 | rs601338 | G | A | 0.055 | 0.012 | 4.10E-06 | 21.484 |
| genus | Bifidobacterium | rs111837148 | G | A | 0.074 | 0.016 | 4.32E-06 | 21.483 |
| genus |  | rs34575570 | T | G | 0.082 | 0.018 | 4.58E-06 | 21.480 |
| genus | Parabacteroides | rs6657302 | C | T | -0.105 | 0.023 | 9.76E-06 | 21.479 |
| genus | Anaerostipes | rs2804186 | T | C | -0.051 | 0.011 | 4.00E-06 | 21.478 |
| genus | Paraprevotella | rs11036239 | T | C | -0.136 | 0.029 | 7.12E-06 | 21.478 |
| genus |  | rs503279 | T | C | -0.049 | 0.011 | 3.69E-06 | 21.477 |
| genus | Parasutterella | rs1403394 | T | C | -0.074 | 0.016 | 4.09E-06 | 21.476 |
| genus | Parasutterella | rs57938605 | A | G | 0.062 | 0.013 | 4.03E-06 | 21.475 |
| genus | LachnospiraceaeUCG004 | rs1593706 | C | G | -0.055 | 0.012 | 3.56E-06 | 21.474 |
| genus | Bifidobacterium | rs75524146 | C | T | 0.076 | 0.016 | 4.26E-06 | 21.474 |
| genus | Ruminiclostridium5 | rs2286383 | C | T | 0.050 | 0.011 | 3.69E-06 | 21.474 |
| genus | Hungatella | rs35815424 | A | G | -0.155 | 0.033 | 5.41E-06 | 21.472 |
| genus | RuminococcaceaeUCG011 | rs12744949 | T | G | -0.116 | 0.025 | 4.34E-06 | 21.471 |
| genus | Veillonella | rs2979933 | T | A | 0.076 | 0.016 | 3.17E-06 | 21.470 |
| genus | Anaerostipes | rs1576148 | G | A | -0.051 | 0.011 | 4.03E-06 | 21.467 |
| genus | Ruminiclostridium5 | rs7703519 | C | A | 0.082 | 0.018 | 5.56E-06 | 21.465 |
| genus | Streptococcus | rs6538957 | T | C | -0.084 | 0.018 | 5.92E-06 | 21.465 |
| genus | Peptococcus | rs2013563 | C | T | 0.088 | 0.019 | 3.80E-06 | 21.463 |
| genus | Anaerostipes | rs6726833 | A | C | -0.088 | 0.019 | 3.32E-06 | 21.463 |
| genus | Bifidobacterium | rs10928519 | C | T | 0.056 | 0.012 | 2.42E-06 | 21.463 |
| genus | Victivallis | rs592514 | A | T | -0.181 | 0.039 | 2.60E-06 | 21.461 |
| genus | Fusicatenibacter | rs12742343 | C | T | -0.057 | 0.012 | 3.04E-06 | 21.461 |
| genus | LachnospiraceaeUCG001 | rs3790911 | G | T | 0.070 | 0.015 | 3.64E-06 | 21.460 |
| genus | Allisonella | rs10817491 | G | A | 0.139 | 0.030 | 4.27E-06 | 21.459 |
| genus | Oscillibacter | rs9650608 | A | G | 0.071 | 0.015 | 3.81E-06 | 21.459 |
| genus | Dialister | rs10105942 | G | A | 0.061 | 0.013 | 3.05E-06 | 21.456 |
| genus | Ruminiclostridium5 | rs4007287 | G | A | -0.053 | 0.012 | 3.92E-06 | 21.456 |
| genus | Eggerthella | rs76663501 | T | C | 0.175 | 0.038 | 4.83E-06 | 21.454 |
| genus | LachnospiraceaeNK4A136group | rs1951575 | T | C | -0.053 | 0.011 | 5.44E-06 | 21.452 |
| genus | RuminococcaceaeNK4A214group | rs12642039 | C | T | -0.055 | 0.012 | 3.43E-06 | 21.452 |
| genus | Anaerostipes | rs60983350 | A | G | -0.054 | 0.012 | 4.42E-06 | 21.449 |
| genus | Butyrivibrio | rs12938033 | T | C | 0.119 | 0.026 | 3.02E-06 | 21.449 |
| genus | Akkermansia | rs111862613 | C | T | 0.091 | 0.020 | 3.39E-06 | 21.449 |
| genus | Erysipelatoclostridium | rs67730850 | A | G | 0.086 | 0.019 | 4.14E-06 | 21.447 |
| genus | Ruminococcus1 | rs10742764 | A | T | -0.052 | 0.011 | 4.33E-06 | 21.446 |
| genus | Allisonella | rs3827484 | G | A | 0.138 | 0.030 | 4.18E-06 | 21.443 |
| genus | Allisonella | rs10980890 | T | C | 0.143 | 0.031 | 3.76E-06 | 21.443 |
| genus | Ruminococcus1 | rs11214882 | C | T | 0.079 | 0.017 | 1.94E-06 | 21.443 |
| genus | Butyrivibrio | rs12757197 | G | A | 0.180 | 0.039 | 3.84E-06 | 21.443 |
| genus | Bifidobacterium | rs503227 | T | C | 0.074 | 0.016 | 3.91E-06 | 21.441 |
| genus | Paraprevotella | rs7216323 | C | G | -0.080 | 0.017 | 5.20E-06 | 21.440 |
| genus | RuminococcaceaeUCG011 | rs10913762 | T | C | -0.115 | 0.025 | 4.56E-06 | 21.440 |
| genus | Streptococcus | rs7973815 | A | G | -0.084 | 0.018 | 5.79E-06 | 21.437 |
| genus | Eisenbergiella | rs1603647 | T | C | 0.104 | 0.022 | 3.78E-06 | 21.437 |
| genus | Haemophilus | rs9895850 | C | T | -0.193 | 0.042 | 2.14E-06 | 21.437 |
| genus | Turicibacter | rs2221441 | C | G | 0.071 | 0.015 | 3.46E-06 | 21.436 |
| genus | LachnospiraceaeUCG004 | rs12894272 | G | A | 0.058 | 0.013 | 4.34E-06 | 21.436 |
| genus | Alistipes | rs2119444 | G | A | -0.051 | 0.011 | 3.95E-06 | 21.431 |
| genus | Terrisporobacter | rs2872237 | A | C | -0.081 | 0.018 | 3.97E-06 | 21.431 |
| genus | Paraprevotella | rs9907587 | C | T | -0.080 | 0.017 | 5.20E-06 | 21.431 |
| genus | RuminococcaceaeUCG011 | rs12743585 | T | A | -0.116 | 0.025 | 4.19E-06 | 21.430 |
| genus | Ruminiclostridium5 | rs35980734 | A | G | -0.053 | 0.011 | 3.38E-06 | 21.429 |
| genus | FamilyXIIIUCG001 | rs7119679 | A | G | -0.081 | 0.017 | 3.52E-06 | 21.429 |
| genus | RuminococcaceaeUCG011 | rs12751949 | A | G | -0.116 | 0.025 | 4.50E-06 | 21.428 |
| genus | Anaerostipes | rs7068521 | C | T | -0.051 | 0.011 | 4.30E-06 | 21.427 |
| genus | Paraprevotella | rs58117850 | A | C | -0.150 | 0.032 | 2.34E-06 | 21.427 |
| genus | RuminococcaceaeUCG003 | rs603985 | T | C | 0.055 | 0.012 | 4.11E-06 | 21.427 |
| genus | Butyrivibrio | rs455732 | T | C | -0.111 | 0.024 | 4.17E-06 | 21.427 |
| genus |  | rs7611898 | A | G | 0.059 | 0.013 | 4.52E-06 | 21.426 |
| genus | Holdemanella | rs12513188 | A | G | 0.090 | 0.020 | 4.65E-06 | 21.426 |
| genus | Clostridiumsensustricto1 | rs550843 | C | T | -0.078 | 0.017 | 2.05E-06 | 21.426 |
| genus |  | rs3809430 | C | T | -0.055 | 0.012 | 3.55E-06 | 21.426 |
| genus | Anaerostipes | rs2804212 | G | C | -0.051 | 0.011 | 4.13E-06 | 21.425 |
| genus | Anaerostipes | rs1410306 | G | A | -0.051 | 0.011 | 4.12E-06 | 21.424 |
| genus | Peptococcus | rs2013601 | A | C | 0.088 | 0.019 | 3.87E-06 | 21.423 |
| genus | Akkermansia | rs11252894 | C | A | 0.077 | 0.017 | 3.15E-06 | 21.421 |
| genus | Flavonifractor | rs4755898 | G | A | 0.074 | 0.016 | 2.86E-06 | 21.420 |
| genus | Anaerofilum | rs10802456 | A | G | 0.098 | 0.021 | 4.11E-06 | 21.419 |
| genus | Lachnospira | rs4923324 | A | G | -0.062 | 0.013 | 2.44E-06 | 21.419 |
| genus | Anaerostipes | rs2784811 | C | T | -0.051 | 0.011 | 4.12E-06 | 21.418 |
| genus | LachnospiraceaeUCG001 | rs12079242 | T | G | 0.070 | 0.015 | 4.19E-06 | 21.418 |
| genus | Parabacteroides | rs115602804 | A | G | 0.103 | 0.022 | 1.93E-06 | 21.418 |
| genus | Peptococcus | rs2417299 | C | T | 0.088 | 0.019 | 3.90E-06 | 21.417 |
| genus | Senegalimassilia | rs10036909 | T | C | 0.186 | 0.040 | 8.05E-06 | 21.416 |
| genus | Turicibacter | rs11868511 | G | A | 0.102 | 0.022 | 3.65E-06 | 21.414 |
| genus |  | rs35598171 | T | G | 0.169 | 0.037 | 3.47E-06 | 21.414 |
| genus | Bifidobacterium | rs4954558 | A | G | 0.063 | 0.014 | 4.24E-06 | 21.413 |
| genus |  | rs2380733 | G | T | 0.090 | 0.020 | 3.90E-06 | 21.413 |
| genus | Butyrivibrio | rs458486 | C | T | -0.111 | 0.024 | 4.23E-06 | 21.412 |
| genus | Anaerostipes | rs11197326 | A | G | -0.051 | 0.011 | 4.22E-06 | 21.412 |
| genus | Butyricimonas | rs62130338 | A | G | -0.073 | 0.016 | 3.90E-06 | 21.412 |
| genus | Sutterella | rs154222 | T | C | -0.058 | 0.013 | 4.26E-06 | 21.411 |
| genus | Parasutterella | rs12439987 | G | A | 0.062 | 0.013 | 4.02E-06 | 21.410 |
| genus | LachnospiraceaeUCG008 | rs29329 | G | A | 0.078 | 0.017 | 4.29E-06 | 21.409 |
| genus |  | rs2251034 | G | A | -0.049 | 0.011 | 3.72E-06 | 21.407 |
| genus | Catenibacterium | rs12404911 | T | C | 0.141 | 0.030 | 2.80E-06 | 21.406 |
| genus | Odoribacter | rs75755928 | A | G | -0.124 | 0.027 | 3.83E-06 | 21.406 |
| genus | Bifidobacterium | rs6755391 | C | T | 0.084 | 0.018 | 3.51E-06 | 21.405 |
| genus | Paraprevotella | rs9547280 | G | A | -0.104 | 0.022 | 1.88E-06 | 21.404 |
| genus | LachnospiraceaeUCG004 | rs11128180 | G | A | 0.065 | 0.014 | 4.52E-06 | 21.404 |
| genus | Anaerostipes | rs2769395 | T | A | -0.051 | 0.011 | 4.18E-06 | 21.402 |
| genus | Hungatella | rs71618441 | G | A | -0.155 | 0.033 | 5.76E-06 | 21.402 |
| genus | Bifidobacterium | rs2164918 | T | G | 0.090 | 0.020 | 8.76E-06 | 21.401 |
| genus |  | rs73109678 | A | G | -0.080 | 0.017 | 3.66E-06 | 21.400 |
| genus | Coprococcus2 | rs2391605 | G | A | -0.064 | 0.014 | 2.80E-06 | 21.399 |
| genus | Butyrivibrio | rs10872068 | C | G | -0.111 | 0.024 | 4.14E-06 | 21.399 |
| genus | Prevotella9 | rs2672225 | A | G | -0.070 | 0.015 | 3.06E-06 | 21.397 |
| genus | Bifidobacterium | rs16831899 | G | A | 0.084 | 0.018 | 3.46E-06 | 21.396 |
| genus | Phascolarctobacterium | rs76124218 | G | C | -0.159 | 0.034 | 2.67E-06 | 21.395 |
| genus | Ruminiclostridium9 | rs1589526 | A | C | -0.052 | 0.011 | 3.89E-06 | 21.393 |
| genus |  | rs2939711 | T | A | 0.090 | 0.020 | 3.94E-06 | 21.392 |
| genus | Roseburia | rs2943022 | C | T | 0.049 | 0.011 | 4.11E-06 | 21.391 |
| genus | Butyrivibrio | rs455726 | A | G | -0.111 | 0.024 | 4.26E-06 | 21.390 |
| genus | Parasutterella | rs6844247 | T | C | 0.061 | 0.013 | 4.06E-06 | 21.390 |
| genus | RuminococcaceaeUCG004 | rs12084902 | G | A | 0.090 | 0.019 | 4.25E-06 | 21.388 |
| genus | Ruminococcus1 | rs4148894 | A | G | -0.052 | 0.011 | 4.66E-06 | 21.387 |
| genus | Alistipes | rs4424138 | T | A | -0.050 | 0.011 | 3.87E-06 | 21.385 |
| genus | Anaerostipes | rs2804192 | G | A | -0.051 | 0.011 | 4.21E-06 | 21.385 |
| genus | Barnesiella | rs62251337 | G | A | -0.069 | 0.015 | 4.24E-06 | 21.385 |
| genus | Intestinibacter | rs12508754 | G | C | 0.067 | 0.014 | 4.49E-06 | 21.383 |
| genus | Flavonifractor | rs12789936 | C | T | 0.074 | 0.016 | 3.01E-06 | 21.383 |
| genus | Holdemanella | rs34187114 | A | C | -0.105 | 0.023 | 5.13E-06 | 21.381 |
| genus | Butyrivibrio | rs11153288 | A | T | -0.111 | 0.024 | 4.27E-06 | 21.379 |
| genus | RuminococcaceaeUCG011 | rs12746253 | A | G | -0.116 | 0.025 | 4.59E-06 | 21.379 |
| genus | Sellimonas | rs2570236 | G | A | 0.116 | 0.025 | 4.00E-06 | 21.379 |
| genus | LachnospiraceaeUCG001 | rs7874144 | A | T | -0.066 | 0.014 | 3.32E-06 | 21.379 |
| genus | RuminococcaceaeUCG011 | rs12725414 | G | A | -0.116 | 0.025 | 4.59E-06 | 21.379 |
| genus |  | rs11256279 | T | C | 0.158 | 0.034 | 5.22E-06 | 21.377 |
| genus | Eisenbergiella | rs1486258 | T | C | 0.104 | 0.022 | 4.91E-06 | 21.376 |
| genus | Sellimonas | . | G | A | -0.119 | 0.026 | 5.37E-06 | 21.376 |
| genus | Barnesiella | rs113258194 | G | A | 0.099 | 0.021 | 7.31E-06 | 21.374 |
| genus | RuminococcaceaeUCG004 | rs75388354 | A | C | 0.129 | 0.028 | 4.16E-06 | 21.373 |
| genus | Eisenbergiella | rs1603648 | A | T | 0.104 | 0.022 | 3.89E-06 | 21.373 |
| genus | Lachnoclostridium | rs6046010 | T | G | -0.050 | 0.011 | 3.76E-06 | 21.372 |
| genus | Streptococcus | rs1333637 | C | T | -0.051 | 0.011 | 3.82E-06 | 21.372 |
| genus | Coprococcus2 | rs9323788 | T | C | -0.066 | 0.014 | 4.12E-06 | 21.371 |
| genus | Howardella | rs12452946 | G | A | -0.106 | 0.023 | 3.80E-06 | 21.370 |
| genus | Lactococcus | rs2293361 | T | C | -0.199 | 0.043 | 1.40E-06 | 21.369 |
| genus | Holdemania | rs41438744 | G | C | -0.125 | 0.027 | 2.44E-06 | 21.369 |
| genus | RuminococcaceaeUCG004 | rs10874701 | C | T | 0.116 | 0.025 | 2.52E-06 | 21.368 |
| genus | RuminococcaceaeUCG004 | rs9882285 | C | G | 0.078 | 0.017 | 4.34E-06 | 21.366 |
| genus | Senegalimassilia | rs13293442 | T | G | 0.079 | 0.017 | 4.62E-06 | 21.366 |
| genus | Allisonella | rs3747504 | C | T | 0.139 | 0.030 | 5.82E-06 | 21.366 |
| genus | Bifidobacterium | rs56108664 | C | T | 0.073 | 0.016 | 2.44E-06 | 21.365 |
| genus | Prevotella7 | rs11035469 | G | A | -0.144 | 0.031 | 7.88E-06 | 21.365 |
| genus | Ruminiclostridium5 | rs7974924 | T | A | 0.050 | 0.011 | 3.89E-06 | 21.365 |
| genus | Adlercreutzia | rs34899151 | A | G | -0.130 | 0.028 | 5.32E-06 | 21.364 |
| genus | Hungatella | rs67230009 | T | G | -0.154 | 0.033 | 5.64E-06 | 21.364 |
| genus | Subdoligranulum | rs6555307 | T | A | -0.072 | 0.016 | 5.59E-06 | 21.363 |
| genus | Oxalobacter | rs13264905 | C | T | 0.140 | 0.030 | 3.89E-06 | 21.363 |
| genus | Lactobacillus | rs77478751 | G | A | -0.220 | 0.048 | 7.33E-06 | 21.361 |
| genus | Lachnospira | rs12151435 | G | A | 0.051 | 0.011 | 4.03E-06 | 21.361 |
| genus | Ruminiclostridium9 | rs1105489 | G | A | 0.057 | 0.012 | 3.90E-06 | 21.360 |
| genus |  | rs55654754 | A | G | 0.061 | 0.013 | 4.17E-06 | 21.360 |
| genus | Senegalimassilia | rs12453608 | C | T | -0.081 | 0.018 | 3.57E-06 | 21.359 |
| genus | Turicibacter | rs61265175 | C | G | -0.086 | 0.019 | 4.14E-06 | 21.359 |
| genus | Peptococcus | rs10117790 | A | G | 0.088 | 0.019 | 4.01E-06 | 21.359 |
| genus | Intestinibacter | rs490097 | T | G | -0.135 | 0.029 | 4.71E-06 | 21.358 |
| genus | Intestinibacter | rs540962 | A | G | -0.135 | 0.029 | 4.71E-06 | 21.358 |
| genus |  | rs1908700 | T | C | -0.106 | 0.023 | 5.52E-06 | 21.358 |
| genus | Phascolarctobacterium | rs1264476 | G | T | 0.077 | 0.017 | 4.30E-06 | 21.357 |
| genus | Ruminococcus1 | rs10769164 | T | A | -0.052 | 0.011 | 4.53E-06 | 21.357 |
| genus | RuminococcaceaeUCG003 | rs78720113 | G | A | -0.115 | 0.025 | 7.59E-06 | 21.357 |
| genus | Parasutterella | rs6551841 | T | C | 0.061 | 0.013 | 4.53E-06 | 21.356 |
| genus | Tyzzerella3 | rs4904511 | T | A | -0.116 | 0.025 | 3.82E-06 | 21.353 |
| genus | RuminococcaceaeUCG004 | rs9882299 | C | T | 0.078 | 0.017 | 4.35E-06 | 21.353 |
| genus | Butyrivibrio | rs11153291 | C | T | -0.111 | 0.024 | 4.32E-06 | 21.352 |
| genus | LachnospiraceaeUCG004 | rs2899901 | A | G | 0.056 | 0.012 | 4.38E-06 | 21.352 |
| genus |  | rs62013253 | A | G | -0.055 | 0.012 | 3.67E-06 | 21.351 |
| genus | Butyrivibrio | rs240994 | C | T | -0.112 | 0.024 | 4.29E-06 | 21.350 |
| genus | Allisonella | rs7874652 | A | G | 0.138 | 0.030 | 4.48E-06 | 21.349 |
| genus | Allisonella | rs4743062 | C | A | 0.141 | 0.031 | 4.41E-06 | 21.349 |
| genus | Anaerotruncus | rs9594207 | A | G | 0.076 | 0.017 | 1.81E-06 | 21.348 |
| genus | Romboutsia | rs75987356 | A | G | -0.130 | 0.028 | 6.71E-06 | 21.347 |
| genus | Eggerthella | rs2240832 | T | C | -0.101 | 0.022 | 4.06E-06 | 21.347 |
| genus | LachnospiraceaeUCG001 | rs7858764 | T | C | -0.066 | 0.014 | 3.45E-06 | 21.347 |
| genus | RuminococcaceaeUCG010 | rs10281901 | T | C | -0.058 | 0.012 | 3.89E-06 | 21.347 |
| genus |  | rs1942371 | A | G | -0.158 | 0.034 | 4.06E-06 | 21.343 |
| genus | RuminococcaceaeUCG003 | rs503279 | T | C | 0.055 | 0.012 | 4.16E-06 | 21.343 |
| genus |  | rs516246 | C | T | -0.049 | 0.011 | 3.94E-06 | 21.343 |
| genus | Ruminiclostridium9 | rs56276432 | A | T | -0.054 | 0.012 | 4.36E-06 | 21.342 |
| genus |  | rs76898927 | A | G | 0.123 | 0.027 | 4.79E-06 | 21.341 |
| genus | Bifidobacterium | rs7560412 | C | T | 0.096 | 0.021 | 8.70E-06 | 21.339 |
| genus | Holdemania | rs17073765 | A | G | -0.136 | 0.029 | 4.37E-06 | 21.336 |
| genus |  | rs10910426 | A | G | 0.050 | 0.011 | 3.96E-06 | 21.336 |
| genus | Anaerostipes | rs11197299 | T | G | -0.051 | 0.011 | 4.89E-06 | 21.335 |
| genus | Ruminococcus1 | rs6493760 | T | C | 0.054 | 0.012 | 3.38E-06 | 21.334 |
| genus | Butyrivibrio | rs12190634 | G | A | -0.111 | 0.024 | 4.36E-06 | 21.334 |
| genus | Tyzzerella3 | rs6920448 | T | C | -0.141 | 0.031 | 4.15E-06 | 21.334 |
| genus |  | rs10940770 | A | C | -0.093 | 0.020 | 3.33E-06 | 21.333 |
| genus | Phascolarctobacterium | rs6701725 | G | A | -0.063 | 0.014 | 3.81E-06 | 21.329 |
| genus | RuminococcaceaeUCG003 | rs679574 | C | G | 0.055 | 0.012 | 4.43E-06 | 21.329 |
| genus | Bifidobacterium | rs10171371 | T | C | 0.055 | 0.012 | 4.63E-06 | 21.326 |
| genus | RuminococcaceaeUCG011 | rs2211033 | C | T | -0.137 | 0.030 | 3.87E-06 | 21.326 |
| genus | Howardella | rs58552303 | A | C | -0.112 | 0.024 | 4.79E-06 | 21.324 |
| genus | Parasutterella | rs6845899 | A | G | 0.061 | 0.013 | 4.20E-06 | 21.324 |
| genus | LachnospiraceaeUCG004 | rs7804470 | A | G | 0.055 | 0.012 | 4.25E-06 | 21.323 |
| genus | LachnospiraceaeUCG008 | rs29330 | G | A | -0.079 | 0.017 | 4.40E-06 | 21.322 |
| genus | RikenellaceaeRC9gutgroup | rs7113155 | C | G | 0.114 | 0.025 | 5.26E-06 | 21.322 |
| genus | Butyrivibrio | rs463860 | C | T | -0.111 | 0.024 | 4.35E-06 | 21.321 |
| genus | LachnospiraceaeUCG010 | rs12555625 | T | G | 0.086 | 0.019 | 5.12E-06 | 21.320 |
| genus | Adlercreutzia | rs1406514 | G | A | -0.081 | 0.018 | 4.54E-06 | 21.318 |
| genus | Bifidobacterium | rs1942051 | A | G | 0.055 | 0.012 | 2.59E-06 | 21.318 |
| genus | Dorea | rs56310278 | C | T | 0.057 | 0.012 | 6.00E-06 | 21.317 |
| genus | Peptococcus | rs13139304 | A | T | -0.091 | 0.020 | 4.40E-06 | 21.315 |
| genus | Akkermansia | rs77055143 | C | T | 0.067 | 0.014 | 3.71E-06 | 21.315 |
| genus | Paraprevotella | rs2058084 | C | T | -0.080 | 0.017 | 5.58E-06 | 21.314 |
| genus | Lachnoclostridium | rs6075505 | A | G | -0.049 | 0.011 | 3.91E-06 | 21.314 |
| genus | ChristensenellaceaeR | rs892686 | G | A | 0.051 | 0.011 | 3.97E-06 | 21.313 |
| genus | Prevotella7 | rs2240542 | T | C | 0.121 | 0.026 | 4.84E-06 | 21.312 |
| genus |  | rs112176119 | T | C | -0.113 | 0.025 | 3.33E-06 | 21.312 |
| genus | Ruminiclostridium6 | rs7930449 | C | A | 0.071 | 0.015 | 3.92E-06 | 21.311 |
| genus | Prevotella9 | rs1193309 | G | A | -0.070 | 0.015 | 3.60E-06 | 21.311 |
| genus | LachnospiraceaeFCS020group | rs62140926 | A | T | 0.061 | 0.013 | 3.38E-06 | 21.311 |
| genus |  | rs57340348 | C | T | -0.098 | 0.021 | 4.93E-06 | 21.311 |
| genus | Bifidobacterium | rs309154 | C | T | 0.075 | 0.016 | 4.88E-06 | 21.310 |
| genus | Anaerotruncus | rs17055747 | A | C | 0.076 | 0.017 | 1.81E-06 | 21.309 |
| genus | LachnospiraceaeFCS020group | rs6469903 | T | C | -0.056 | 0.012 | 4.54E-06 | 21.309 |
| genus | Prevotella7 | rs62115054 | C | T | 0.150 | 0.032 | 2.57E-06 | 21.308 |
| genus | Erysipelatoclostridium | rs349424 | T | C | 0.086 | 0.019 | 4.34E-06 | 21.308 |
| genus | RuminococcaceaeUCG005 | rs12288512 | G | A | 0.067 | 0.014 | 3.10E-06 | 21.308 |
| genus | Anaerostipes | rs2769416 | A | G | -0.051 | 0.011 | 4.38E-06 | 21.307 |
| genus | Veillonella | rs4679195 | G | T | 0.076 | 0.016 | 3.42E-06 | 21.306 |
| genus | Faecalibacterium | rs28376661 | G | C | 0.050 | 0.011 | 3.66E-06 | 21.305 |
| genus | Clostridiumsensustricto1 | rs115807074 | G | A | -0.227 | 0.049 | 4.32E-06 | 21.305 |
| genus | Anaerostipes | rs2804204 | G | T | -0.051 | 0.011 | 4.40E-06 | 21.303 |
| genus |  | rs2939709 | A | G | 0.090 | 0.020 | 4.07E-06 | 21.302 |
| genus | Prevotella9 | rs6586820 | G | A | -0.069 | 0.015 | 3.28E-06 | 21.302 |
| genus |  | rs74606150 | G | C | -0.196 | 0.043 | 4.25E-06 | 21.302 |
| genus | ErysipelotrichaceaeUCG003 | rs35470500 | T | C | -0.100 | 0.022 | 3.69E-06 | 21.300 |
| genus | Peptococcus | rs55962838 | G | A | 0.135 | 0.029 | 1.74E-06 | 21.300 |
| genus | Actinomyces | rs4962491 | T | C | 0.169 | 0.037 | 6.70E-06 | 21.299 |
| genus | Streptococcus | rs11006999 | C | T | 0.100 | 0.022 | 4.54E-06 | 21.298 |
| genus | Butyricimonas | rs4747117 | G | T | 0.066 | 0.014 | 2.86E-06 | 21.297 |
| genus | Butyrivibrio | rs10925630 | A | T | 0.172 | 0.037 | 4.94E-06 | 21.296 |
| genus | Butyrivibrio | rs11577669 | T | A | 0.172 | 0.037 | 4.94E-06 | 21.296 |
| genus | Odoribacter | rs116416219 | C | A | -0.124 | 0.027 | 4.16E-06 | 21.296 |
| genus |  | rs606117 | G | A | 0.083 | 0.018 | 4.82E-06 | 21.296 |
| genus |  | rs71564433 | A | T | -0.127 | 0.027 | 7.80E-06 | 21.296 |
| genus | Ruminiclostridium9 | rs12634907 | A | G | -0.054 | 0.012 | 4.56E-06 | 21.295 |
| genus | Turicibacter | rs11054680 | C | T | -0.105 | 0.023 | 2.31E-06 | 21.295 |
| genus | Paraprevotella | rs2367260 | T | C | -0.080 | 0.017 | 5.65E-06 | 21.294 |
| genus | Anaerofilum | rs1710914 | C | T | -0.177 | 0.038 | 3.06E-06 | 21.294 |
| genus | Sutterella | rs9537940 | C | A | -0.058 | 0.012 | 3.60E-06 | 21.294 |
| genus | RuminococcaceaeUCG003 | rs504963 | G | A | 0.055 | 0.012 | 4.28E-06 | 21.294 |
| genus | Butyrivibrio | rs3851225 | G | A | -0.111 | 0.024 | 4.34E-06 | 21.293 |
| genus | ChristensenellaceaeR | rs62132810 | G | A | -0.083 | 0.018 | 5.67E-06 | 21.293 |
| genus |  | rs2939707 | A | C | 0.090 | 0.020 | 4.03E-06 | 21.292 |
| genus | Turicibacter | rs11871873 | G | C | 0.101 | 0.022 | 3.82E-06 | 21.292 |
| genus | LachnospiraceaeUCG004 | rs6665422 | T | A | -0.058 | 0.013 | 4.48E-06 | 21.291 |
| genus | Flavonifractor | rs12795080 | C | G | 0.074 | 0.016 | 3.21E-06 | 21.291 |
| genus | Marvinbryantia | rs17138958 | C | T | -0.126 | 0.027 | 3.59E-06 | 21.290 |
| genus | RuminococcaceaeUCG009 | rs2192926 | G | A | -0.089 | 0.019 | 4.88E-06 | 21.290 |
| genus | LachnospiraceaeUCG004 | rs11157095 | T | G | 0.058 | 0.013 | 4.72E-06 | 21.290 |
| genus | Butyricimonas | rs2591754 | A | T | 0.067 | 0.015 | 3.02E-06 | 21.287 |
| genus | Marvinbryantia | rs11823366 | A | G | 0.106 | 0.023 | 3.55E-06 | 21.286 |
| genus | Sellimonas | rs2187447 | C | A | 0.243 | 0.053 | 3.98E-06 | 21.285 |
| genus | Coprococcus3 | rs7521171 | A | G | -0.060 | 0.013 | 4.32E-06 | 21.285 |
| genus | RuminococcaceaeUCG011 | rs12724525 | C | A | -0.115 | 0.025 | 4.53E-06 | 21.285 |
| genus | Parasutterella | rs4340107 | C | T | -0.074 | 0.016 | 4.57E-06 | 21.285 |
| genus | Ruminiclostridium5 | rs2248896 | T | A | 0.052 | 0.011 | 3.76E-06 | 21.284 |
| genus |  | rs7000472 | G | A | -0.076 | 0.017 | 4.07E-06 | 21.284 |
| genus | Odoribacter | rs76951684 | A | G | -0.124 | 0.027 | 4.35E-06 | 21.284 |
| genus | Ruminiclostridium9 | rs6441175 | G | A | -0.051 | 0.011 | 4.09E-06 | 21.282 |
| genus | Bifidobacterium | rs3769018 | T | C | 0.094 | 0.020 | 8.75E-06 | 21.282 |
| genus | FamilyXIIIAD3011group | rs2999596 | C | A | -0.056 | 0.012 | 4.15E-06 | 21.281 |
| genus | Bilophila | rs1241171 | A | G | -0.069 | 0.015 | 4.24E-06 | 21.281 |
| genus | Holdemania | rs9529719 | C | T | 0.074 | 0.016 | 5.97E-06 | 21.281 |
| genus |  | rs11872789 | C | T | 0.057 | 0.012 | 3.91E-06 | 21.281 |
| genus | Ruminiclostridium5 | rs10773980 | C | T | -0.050 | 0.011 | 4.64E-06 | 21.279 |
| genus | Marvinbryantia | rs2768442 | C | T | -0.072 | 0.016 | 5.09E-06 | 21.276 |
| genus | Oxalobacter | rs9812512 | C | T | 0.156 | 0.034 | 8.03E-06 | 21.275 |
| genus | RuminococcaceaeUCG011 | rs1416002 | A | G | 0.182 | 0.039 | 3.24E-06 | 21.274 |
| genus | FamilyXIIIAD3011group | rs12812672 | C | T | -0.096 | 0.021 | 2.56E-06 | 21.273 |
| genus | Tyzzerella3 | rs76319841 | C | A | -0.127 | 0.028 | 7.02E-06 | 21.273 |
| genus | RikenellaceaeRC9gutgroup | rs9887954 | A | G | -0.115 | 0.025 | 4.81E-06 | 21.272 |
| genus | RuminococcaceaeUCG011 | rs4536002 | G | A | 0.182 | 0.039 | 3.30E-06 | 21.272 |
| genus | Ruminiclostridium9 | rs2117764 | A | G | -0.054 | 0.012 | 4.53E-06 | 21.272 |
| genus | Victivallis | rs173120 | C | T | 0.134 | 0.029 | 7.65E-06 | 21.272 |
| genus | Ruminiclostridium5 | rs1492620 | C | T | -0.083 | 0.018 | 3.53E-06 | 21.271 |
| genus | Subdoligranulum | rs76528319 | T | G | -0.143 | 0.031 | 7.41E-06 | 21.271 |
| genus | Butyricimonas | rs11748099 | C | T | 0.067 | 0.015 | 3.11E-06 | 21.269 |
| genus | RuminococcaceaeUCG010 | rs591790 | C | T | -0.057 | 0.012 | 3.98E-06 | 21.267 |
| genus | Allisonella | rs3747490 | T | G | 0.138 | 0.030 | 4.80E-06 | 21.267 |
| genus | Allisonella | rs3747491 | A | G | 0.138 | 0.030 | 4.80E-06 | 21.267 |
| genus | Allisonella | rs13285567 | A | C | 0.138 | 0.030 | 4.80E-06 | 21.267 |
| genus | RuminococcaceaeUCG011 | rs12729775 | C | T | -0.115 | 0.025 | 4.94E-06 | 21.265 |
| genus | RuminococcaceaeNK4A214group | rs7573569 | C | T | 0.108 | 0.023 | 3.23E-06 | 21.264 |
| genus | Anaerotruncus | rs7147473 | A | G | 0.054 | 0.012 | 3.51E-06 | 21.264 |
| genus | Coprococcus1 | rs10455249 | G | A | -0.051 | 0.011 | 4.20E-06 | 21.263 |
| genus | Butyrivibrio | rs6704046 | G | A | 0.172 | 0.037 | 5.02E-06 | 21.263 |
| genus | Ruminococcus2 | rs7318991 | T | A | -0.056 | 0.012 | 4.86E-06 | 21.262 |
| genus | Akkermansia | rs11184341 | C | G | 0.066 | 0.014 | 4.06E-06 | 21.261 |
| genus | RikenellaceaeRC9gutgroup | rs10980105 | C | T | -0.130 | 0.028 | 4.26E-06 | 21.259 |
| genus | Butyrivibrio | rs72759722 | C | T | 0.172 | 0.037 | 5.03E-06 | 21.259 |
| genus | Anaerostipes | rs2804195 | A | C | -0.051 | 0.011 | 4.51E-06 | 21.258 |
| genus | Anaerostipes | rs2769415 | A | G | -0.051 | 0.011 | 4.51E-06 | 21.257 |
| genus | DefluviitaleaceaeUCG011 | rs73217031 | C | T | 0.166 | 0.036 | 4.91E-06 | 21.257 |
| genus | Ruminococcus1 | rs7115214 | A | G | -0.052 | 0.011 | 4.76E-06 | 21.256 |
| genus | Parasutterella | rs7305839 | C | T | 0.062 | 0.014 | 2.72E-06 | 21.255 |
| genus | CandidatusSoleaferrea | rs10090365 | G | A | -0.083 | 0.018 | 4.17E-06 | 21.255 |
| genus | Allisonella | rs4743085 | T | C | 0.138 | 0.030 | 4.99E-06 | 21.255 |
| genus | RuminococcaceaeUCG002 | rs7120052 | C | A | 0.062 | 0.014 | 1.97E-06 | 21.254 |
| genus |  | rs1158959 | C | T | 0.158 | 0.034 | 5.64E-06 | 21.253 |
| genus |  | rs2357118 | G | T | -0.155 | 0.034 | 5.47E-06 | 21.253 |
| genus | Lactobacillus | rs768253 | G | T | -0.079 | 0.017 | 4.25E-06 | 21.252 |
| genus | Alloprevotella | rs4680035 | G | A | -0.120 | 0.026 | 4.99E-06 | 21.250 |
| genus | Turicibacter | rs2834977 | C | T | -0.096 | 0.021 | 3.96E-06 | 21.248 |
| genus | Adlercreutzia | rs2717136 | T | C | -0.130 | 0.028 | 4.58E-06 | 21.247 |
| genus | Anaerotruncus | rs9594209 | T | C | 0.076 | 0.017 | 1.94E-06 | 21.247 |
| genus | LachnospiraceaeNC2004group | rs12131508 | G | A | 0.113 | 0.025 | 4.25E-06 | 21.247 |
| genus | Anaerostipes | rs1336117 | C | G | -0.050 | 0.011 | 4.49E-06 | 21.247 |
| genus | Sellimonas | rs56203279 | C | T | -0.124 | 0.027 | 3.72E-06 | 21.246 |
| genus | Veillonella | rs2947666 | C | T | 0.077 | 0.017 | 3.67E-06 | 21.246 |
| genus | LachnospiraceaeFCS020group | rs249932 | C | T | 0.120 | 0.026 | 7.97E-06 | 21.245 |
| genus | Butyrivibrio | rs9903225 | A | G | 0.119 | 0.026 | 3.35E-06 | 21.243 |
| genus | Slackia | rs874123 | G | A | -0.122 | 0.026 | 3.93E-06 | 21.242 |
| genus | Escherichia | rs11706043 | A | T | 0.076 | 0.016 | 5.87E-06 | 21.242 |
| genus | Bifidobacterium | rs11896918 | A | G | 0.093 | 0.020 | 8.06E-06 | 21.241 |
| genus | RuminococcaceaeUCG002 | rs113147300 | G | A | -0.076 | 0.016 | 7.69E-06 | 21.240 |
| genus | Oscillibacter | rs16866397 | T | G | 0.096 | 0.021 | 5.39E-06 | 21.238 |
| genus | Methanobrevibacter | rs73475126 | T | G | 0.208 | 0.045 | 4.77E-06 | 21.238 |
| genus | LachnospiraceaeNC2004group | rs12734575 | C | T | 0.113 | 0.025 | 4.58E-06 | 21.237 |
| genus | Howardella | rs61771805 | T | A | -0.137 | 0.030 | 4.03E-06 | 21.236 |
| genus | Fusicatenibacter | rs2039204 | A | T | -0.050 | 0.011 | 3.94E-06 | 21.235 |
| genus | Bifidobacterium | rs6707326 | A | G | 0.055 | 0.012 | 4.56E-06 | 21.235 |
| genus | Anaerostipes | rs2769406 | A | G | -0.050 | 0.011 | 4.56E-06 | 21.234 |
| genus | Odoribacter | rs10093869 | G | A | -0.058 | 0.013 | 3.67E-06 | 21.234 |
| genus | LachnospiraceaeFCS020group | rs6779101 | A | C | 0.059 | 0.013 | 4.15E-06 | 21.233 |
| genus | Marvinbryantia | rs11645029 | C | G | -0.061 | 0.013 | 4.15E-06 | 21.233 |
| genus | Gordonibacter | rs2889871 | C | T | -0.213 | 0.046 | 3.56E-06 | 21.232 |
| genus | Holdemanella | rs35228298 | A | G | 0.093 | 0.020 | 7.30E-06 | 21.231 |
| genus | Victivallis | rs1407532 | G | A | 0.114 | 0.025 | 4.79E-06 | 21.228 |
| genus | Intestinibacter | rs4937048 | G | A | -0.137 | 0.030 | 4.42E-06 | 21.228 |
| genus |  | rs17519472 | T | C | 0.108 | 0.023 | 4.70E-06 | 21.228 |
| genus | Bifidobacterium | rs1435577 | C | G | 0.074 | 0.016 | 4.74E-06 | 21.227 |
| genus | Subdoligranulum | rs12436998 | T | C | -0.051 | 0.011 | 5.01E-06 | 21.226 |
| genus | Bifidobacterium | rs842359 | G | C | 0.055 | 0.012 | 4.48E-06 | 21.226 |
| genus | FamilyXIIIAD3011group | rs11126423 | T | C | 0.090 | 0.020 | 5.91E-06 | 21.226 |
| genus | LachnospiraceaeUCG001 | rs3790905 | A | C | 0.069 | 0.015 | 4.16E-06 | 21.225 |
| genus | Holdemania | rs35979242 | G | T | -0.135 | 0.029 | 4.62E-06 | 21.222 |
| genus | Butyrivibrio | rs12094865 | G | A | 0.171 | 0.037 | 5.12E-06 | 21.222 |
| genus | FamilyXIIIAD3011group | rs2999592 | T | A | -0.056 | 0.012 | 4.25E-06 | 21.222 |
| genus | Anaerostipes | rs2769398 | A | C | -0.050 | 0.011 | 4.60E-06 | 21.222 |
| genus | Anaerostipes | rs2769400 | A | G | -0.050 | 0.011 | 4.60E-06 | 21.222 |
| genus | Anaerostipes | rs2769399 | A | T | -0.050 | 0.011 | 4.60E-06 | 21.222 |
| genus | Peptococcus | rs7856024 | G | A | 0.088 | 0.019 | 4.31E-06 | 21.220 |
| genus | Prevotella9 | rs2683316 | G | A | -0.069 | 0.015 | 3.35E-06 | 21.220 |
| genus | Parasutterella | rs6852253 | T | G | 0.061 | 0.013 | 4.67E-06 | 21.217 |
| genus | LachnospiraceaeUCG008 | rs12058175 | T | C | -0.112 | 0.024 | 3.85E-06 | 21.217 |
| genus |  | rs151257695 | G | A | 0.210 | 0.045 | 3.10E-06 | 21.217 |
| genus | Ruminiclostridium5 | rs7474632 | T | C | -0.053 | 0.011 | 3.77E-06 | 21.216 |
| genus | Eggerthella | rs28609731 | C | G | -0.091 | 0.020 | 4.66E-06 | 21.215 |
| genus | Peptococcus | rs9409342 | C | T | 0.088 | 0.019 | 4.35E-06 | 21.215 |
| genus | Allisonella | rs12551818 | T | C | 0.138 | 0.030 | 4.73E-06 | 21.213 |
| genus | RuminococcaceaeUCG002 | rs72874194 | C | G | -0.077 | 0.017 | 3.47E-06 | 21.212 |
| genus |  | rs12666960 | T | C | -0.079 | 0.017 | 2.69E-06 | 21.212 |
| genus | Anaerostipes | rs1854244 | T | C | -0.050 | 0.011 | 4.58E-06 | 21.211 |
| genus |  | rs2102146 | A | G | -0.082 | 0.018 | 5.04E-06 | 21.211 |
| genus | Coprobacter | rs72821405 | C | T | -0.147 | 0.032 | 4.76E-06 | 21.210 |
| genus | Odoribacter | rs17103688 | A | G | -0.124 | 0.027 | 4.10E-06 | 21.210 |
| genus | Paraprevotella | rs17064682 | C | T | -0.124 | 0.027 | 4.35E-06 | 21.209 |
| genus | Allisonella | rs13289813 | T | C | 0.138 | 0.030 | 4.93E-06 | 21.209 |
| genus | Oscillibacter | rs3731749 | C | T | 0.096 | 0.021 | 5.50E-06 | 21.208 |
| genus | Intestinibacter | rs2702387 | G | A | 0.061 | 0.013 | 4.26E-06 | 21.208 |
| genus | Bifidobacterium | rs857444 | T | C | 0.056 | 0.012 | 3.57E-06 | 21.208 |
| genus | Phascolarctobacterium | rs1264474 | C | T | 0.076 | 0.017 | 4.69E-06 | 21.207 |
| genus | LachnospiraceaeFCS020group | rs9848122 | G | A | 0.056 | 0.012 | 4.62E-06 | 21.207 |
| genus | Faecalibacterium | rs28425620 | C | A | 0.050 | 0.011 | 3.85E-06 | 21.206 |
| genus | Lachnoclostridium | rs62282803 | G | A | 0.083 | 0.018 | 4.93E-06 | 21.206 |
| genus | RuminococcaceaeUCG002 | rs4836088 | G | A | -0.056 | 0.012 | 4.74E-06 | 21.206 |
| genus | RikenellaceaeRC9gutgroup | rs17032291 | C | T | -0.170 | 0.037 | 6.61E-06 | 21.206 |
| genus | LachnospiraceaeUCG008 | rs29328 | C | T | 0.078 | 0.017 | 4.74E-06 | 21.205 |
| genus | Allisonella | rs3747492 | C | T | 0.138 | 0.030 | 4.91E-06 | 21.202 |
| genus | Anaerofilum | rs526159 | G | A | -0.095 | 0.021 | 3.32E-06 | 21.200 |
| genus | Subdoligranulum | rs7737642 | A | C | -0.080 | 0.017 | 5.34E-06 | 21.199 |
| genus | Parabacteroides | rs10811655 | C | G | -0.066 | 0.014 | 3.01E-06 | 21.198 |
| genus | Prevotella9 | rs2943426 | C | G | -0.070 | 0.015 | 3.62E-06 | 21.196 |
| genus | Parasutterella | rs10899913 | A | G | -0.063 | 0.014 | 4.08E-06 | 21.194 |
| genus | Anaerostipes | rs2804208 | T | C | -0.050 | 0.011 | 4.65E-06 | 21.194 |
| genus | Alistipes | rs67281112 | C | G | 0.063 | 0.014 | 3.92E-06 | 21.192 |
| genus | LachnospiraceaeFCS020group | rs9603634 | A | G | -0.060 | 0.013 | 4.23E-06 | 21.192 |
| genus | Butyricimonas | rs270727 | C | G | -0.069 | 0.015 | 5.38E-06 | 21.191 |
| genus | Lachnoclostridium | rs6075502 | T | C | -0.050 | 0.011 | 4.14E-06 | 21.191 |
| genus |  | rs7795339 | C | T | 0.083 | 0.018 | 4.10E-06 | 21.190 |
| genus | FamilyXIIIAD3011group | rs12321489 | T | C | -0.091 | 0.020 | 4.07E-06 | 21.190 |
| genus | CandidatusSoleaferrea | rs256867 | G | C | 0.100 | 0.022 | 5.63E-06 | 21.190 |
| genus | FamilyXIIIAD3011group | rs17469314 | C | T | -0.091 | 0.020 | 3.97E-06 | 21.189 |
| genus | Allisonella | rs2306090 | T | C | 0.138 | 0.030 | 5.00E-06 | 21.188 |
| genus | Allisonella | rs2306092 | A | G | 0.138 | 0.030 | 5.00E-06 | 21.188 |
| genus | Butyrivibrio | rs11579933 | C | A | 0.179 | 0.039 | 4.39E-06 | 21.188 |
| genus | Ruminiclostridium9 | rs2885862 | G | C | -0.052 | 0.011 | 4.54E-06 | 21.188 |
| genus | Slackia | rs87097 | T | C | 0.086 | 0.019 | 4.40E-06 | 21.188 |
| genus | Anaerostipes | rs2769397 | G | T | -0.050 | 0.011 | 4.69E-06 | 21.188 |
| genus | Anaerostipes | rs2769396 | G | A | -0.050 | 0.011 | 4.69E-06 | 21.188 |
| genus |  | rs75091271 | T | C | -0.092 | 0.020 | 5.25E-06 | 21.188 |
| genus | LachnospiraceaeFCS020group | rs10425927 | A | G | 0.063 | 0.014 | 4.47E-06 | 21.187 |
| genus | Anaerostipes | rs7894126 | C | T | -0.050 | 0.011 | 4.68E-06 | 21.186 |
| genus | Victivallis | rs10484765 | A | G | 0.113 | 0.025 | 4.83E-06 | 21.185 |
| genus | RuminococcaceaeUCG011 | rs2434357 | G | A | -0.108 | 0.023 | 4.17E-06 | 21.183 |
| genus | Alistipes | rs12536478 | A | C | -0.050 | 0.011 | 4.58E-06 | 21.183 |
| genus | Allisonella | rs13287095 | T | C | 0.139 | 0.030 | 5.34E-06 | 21.182 |
| genus | Paraprevotella | rs7221230 | T | C | -0.080 | 0.017 | 5.95E-06 | 21.181 |
| genus | FamilyXIIIAD3011group | rs17156849 | A | G | -0.113 | 0.025 | 4.19E-06 | 21.181 |
| genus | Ruminococcus1 | rs7115047 | T | C | -0.051 | 0.011 | 4.95E-06 | 21.181 |
| genus | Anaerostipes | rs2769403 | G | A | -0.050 | 0.011 | 4.70E-06 | 21.181 |
| genus | Parasutterella | rs2881999 | A | G | 0.061 | 0.013 | 4.90E-06 | 21.180 |
| genus | RuminococcaceaeUCG004 | rs11164643 | C | T | 0.089 | 0.019 | 4.77E-06 | 21.180 |
| genus | Parabacteroides | rs114567323 | C | T | 0.186 | 0.041 | 5.65E-06 | 21.180 |
| genus | Butyrivibrio | rs9899961 | C | T | 0.119 | 0.026 | 3.48E-06 | 21.179 |
| genus | Odoribacter | rs113592971 | G | A | -0.124 | 0.027 | 4.40E-06 | 21.179 |
| genus | RuminococcaceaeUCG003 | rs632111 | A | G | 0.055 | 0.012 | 4.74E-06 | 21.178 |
| genus | RuminococcaceaeUCG004 | rs945749 | A | G | 0.117 | 0.025 | 4.73E-06 | 21.178 |
| genus | Anaerotruncus | rs9603202 | A | G | 0.076 | 0.017 | 1.97E-06 | 21.178 |
| genus | Anaerotruncus | rs9603201 | G | C | 0.076 | 0.017 | 1.97E-06 | 21.178 |
| genus | Butyricimonas | rs1338635 | G | A | 0.066 | 0.014 | 3.51E-06 | 21.177 |
| genus | LachnospiraceaeND3007group | rs9921691 | C | T | -0.053 | 0.012 | 4.49E-06 | 21.177 |
| genus | Bifidobacterium | rs4594504 | A | C | 0.074 | 0.016 | 4.91E-06 | 21.175 |
| genus | Oscillibacter | rs16934185 | G | A | -0.130 | 0.028 | 4.38E-06 | 21.175 |
| genus | LachnospiraceaeUCG004 | rs10926163 | C | G | -0.058 | 0.013 | 5.02E-06 | 21.174 |
| genus | Subdoligranulum | rs11167777 | A | C | -0.049 | 0.011 | 4.43E-06 | 21.174 |
| genus | Lachnospira | rs66713776 | G | A | 0.051 | 0.011 | 4.45E-06 | 21.173 |
| genus | Anaerotruncus | rs1923718 | C | T | 0.076 | 0.017 | 2.04E-06 | 21.173 |
| genus | ChristensenellaceaeR | rs10401160 | C | T | 0.054 | 0.012 | 3.44E-06 | 21.172 |
| genus | LachnospiraceaeUCG008 | rs10741779 | A | G | -0.089 | 0.019 | 4.15E-06 | 21.171 |
| genus | Odoribacter | rs77506928 | T | C | -0.124 | 0.027 | 4.38E-06 | 21.171 |
| genus | Coprococcus2 | rs4904315 | A | C | -0.066 | 0.014 | 4.44E-06 | 21.168 |
| genus | Adlercreutzia | rs255375 | T | G | -0.072 | 0.016 | 4.38E-06 | 21.167 |
| genus | Anaerostipes | rs2769375 | T | C | -0.050 | 0.011 | 4.74E-06 | 21.167 |
| genus | Sellimonas | rs2014638 | A | G | 0.116 | 0.025 | 4.47E-06 | 21.166 |
| genus | Anaerostipes | rs1410304 | G | A | -0.050 | 0.011 | 4.67E-06 | 21.166 |
| genus | Anaerotruncus | rs9603203 | G | A | 0.076 | 0.017 | 2.00E-06 | 21.166 |
| genus | RuminococcaceaeUCG004 | rs11164623 | G | T | 0.122 | 0.027 | 3.44E-06 | 21.166 |
| genus | Akkermansia | rs6832927 | C | A | 0.109 | 0.024 | 3.85E-06 | 21.165 |
| genus | Erysipelatoclostridium | rs349421 | G | T | 0.086 | 0.019 | 4.61E-06 | 21.164 |
| genus | Anaerotruncus | rs35308611 | T | C | 0.076 | 0.017 | 1.98E-06 | 21.163 |
| genus | Prevotella9 | rs35790419 | G | A | -0.137 | 0.030 | 5.83E-06 | 21.163 |
| genus | RuminococcaceaeUCG004 | rs73089428 | A | G | 0.093 | 0.020 | 5.01E-06 | 21.162 |
| genus | Parasutterella | rs11058441 | C | T | -0.074 | 0.016 | 6.02E-06 | 21.160 |
| genus | Actinomyces | rs4146653 | A | G | 0.099 | 0.021 | 4.50E-06 | 21.159 |
| genus | Phascolarctobacterium | rs1148491 | A | G | 0.077 | 0.017 | 5.01E-06 | 21.159 |
| genus | Sellimonas | rs10249740 | T | A | -0.124 | 0.027 | 3.88E-06 | 21.159 |
| genus | Tyzzerella3 | rs3752240 | A | G | 0.093 | 0.020 | 4.55E-06 | 21.158 |
| genus | Intestinibacter | rs572034 | A | G | -0.134 | 0.029 | 5.17E-06 | 21.158 |
| genus | FamilyXIIIUCG001 | rs76463770 | G | A | 0.193 | 0.042 | 3.77E-06 | 21.158 |
| genus | Intestinimonas | rs11928843 | A | G | -0.061 | 0.013 | 4.21E-06 | 21.158 |
| genus | LachnospiraceaeFCS020group | rs35035870 | C | T | -0.191 | 0.041 | 2.62E-06 | 21.158 |
| genus | FamilyXIIIUCG001 | rs2276529 | G | C | -0.076 | 0.017 | 5.36E-06 | 21.157 |
| genus |  | rs139757 | C | A | -0.079 | 0.017 | 4.29E-06 | 21.157 |
| genus |  | rs74323458 | C | T | -0.164 | 0.036 | 4.14E-06 | 21.156 |
| genus |  | rs11186828 | G | A | -0.164 | 0.036 | 4.14E-06 | 21.156 |
| genus |  | rs76251755 | C | G | -0.166 | 0.036 | 4.47E-06 | 21.155 |
| genus |  | rs2939708 | T | A | 0.090 | 0.020 | 4.46E-06 | 21.154 |
| genus | Anaerostipes | rs2769405 | G | A | -0.050 | 0.011 | 4.75E-06 | 21.153 |
| genus | Parasutterella | rs10773222 | T | C | -0.073 | 0.016 | 4.95E-06 | 21.153 |
| genus | Peptococcus | rs6816211 | G | A | -0.104 | 0.023 | 5.06E-06 | 21.153 |
| genus | LachnospiraceaeNK4A136group | rs3783321 | T | G | -0.053 | 0.011 | 5.82E-06 | 21.152 |
| genus | Anaerotruncus | rs55963355 | C | T | 0.076 | 0.017 | 2.00E-06 | 21.151 |
| genus | Anaerostipes | rs1854243 | G | C | -0.050 | 0.011 | 4.73E-06 | 21.149 |
| genus |  | rs12906958 | T | C | -0.053 | 0.012 | 4.35E-06 | 21.149 |
| genus |  | rs423478 | C | T | -0.054 | 0.012 | 4.09E-06 | 21.149 |
| genus | Butyricicoccus | rs2017189 | T | G | -0.051 | 0.011 | 3.87E-06 | 21.148 |
| genus | Bilophila | rs542415 | C | T | -0.061 | 0.013 | 4.71E-06 | 21.147 |
| genus | Terrisporobacter | rs7034891 | C | G | -0.080 | 0.017 | 4.54E-06 | 21.147 |
| genus | Anaerotruncus | rs55796046 | G | T | 0.076 | 0.017 | 1.97E-06 | 21.147 |
| genus | Odoribacter | rs74553962 | G | T | 0.121 | 0.026 | 9.49E-06 | 21.146 |
| genus | Eisenbergiella | rs1553971 | G | T | 0.121 | 0.026 | 5.27E-06 | 21.146 |
| genus | LachnospiraceaeNK4A136group | rs61076771 | T | G | -0.053 | 0.011 | 6.32E-06 | 21.145 |
| genus | Roseburia | rs6445851 | A | G | -0.050 | 0.011 | 3.53E-06 | 21.144 |
| genus | Catenibacterium | rs212395 | G | T | -0.133 | 0.029 | 6.69E-06 | 21.143 |
| genus | Anaerotruncus | rs9603195 | G | T | 0.076 | 0.017 | 1.94E-06 | 21.143 |
| genus | Anaerotruncus | rs17055728 | A | G | 0.076 | 0.017 | 2.01E-06 | 21.143 |
| genus | Anaerostipes | rs2804205 | G | C | -0.050 | 0.011 | 4.78E-06 | 21.143 |
| genus | Intestinibacter | rs447950 | G | A | 0.063 | 0.014 | 5.64E-06 | 21.143 |
| genus | Slackia | rs2164672 | T | A | -0.121 | 0.026 | 3.69E-06 | 21.143 |
| genus | Parasutterella | rs1105118 | C | A | 0.061 | 0.013 | 4.53E-06 | 21.142 |
| genus | Bifidobacterium | rs76671854 | G | C | -0.085 | 0.018 | 3.96E-06 | 21.142 |
| genus |  | rs329887 | A | G | -0.082 | 0.018 | 5.20E-06 | 21.142 |
| genus | Lachnoclostridium | rs4814844 | G | T | -0.049 | 0.011 | 4.14E-06 | 21.141 |
| genus | Prevotella9 | rs71439047 | G | C | -0.136 | 0.030 | 5.18E-06 | 21.139 |
| genus | Bilophila | rs45577639 | G | A | 0.069 | 0.015 | 4.25E-06 | 21.138 |
| genus | Alistipes | rs2311507 | C | T | -0.050 | 0.011 | 4.64E-06 | 21.138 |
| genus | Veillonella | rs742016 | G | A | -0.069 | 0.015 | 4.66E-06 | 21.137 |
| genus | Turicibacter | rs76225419 | G | C | 0.102 | 0.022 | 4.45E-06 | 21.137 |
| genus |  | rs7195470 | A | G | 0.058 | 0.013 | 6.22E-06 | 21.137 |
| genus |  | rs12539819 | T | C | 0.111 | 0.024 | 4.49E-06 | 21.137 |
| genus | RuminococcaceaeUCG002 | rs73531566 | G | A | -0.076 | 0.016 | 7.98E-06 | 21.135 |
| genus | Peptococcus | rs17829375 | G | A | 0.134 | 0.029 | 1.80E-06 | 21.135 |
| genus | Erysipelatoclostridium | rs17804233 | C | T | -0.066 | 0.014 | 4.59E-06 | 21.133 |
| genus | RuminococcaceaeUCG003 | rs76377264 | A | G | -0.061 | 0.013 | 4.87E-06 | 21.133 |
| genus | FamilyXIIIAD3011group | rs8179997 | G | A | 0.060 | 0.013 | 4.98E-06 | 21.132 |
| genus | Parasutterella | rs8038877 | G | A | 0.061 | 0.013 | 4.66E-06 | 21.131 |
| genus | Streptococcus | rs9895557 | C | T | -0.051 | 0.011 | 3.92E-06 | 21.130 |
| genus | LachnospiraceaeNK4A136group | rs76193507 | G | A | -0.230 | 0.050 | 2.93E-06 | 21.129 |
| genus | Butyrivibrio | rs6661508 | G | A | 0.171 | 0.037 | 5.66E-06 | 21.129 |
| genus | Akkermansia | rs12498518 | C | T | 0.067 | 0.014 | 3.99E-06 | 21.128 |
| genus | Peptococcus | rs1481445 | C | G | 0.132 | 0.029 | 1.53E-06 | 21.128 |
| genus | Sellimonas | rs2570235 | G | A | 0.115 | 0.025 | 4.55E-06 | 21.128 |
| genus | Ruminiclostridium6 | rs7930568 | C | T | 0.071 | 0.016 | 4.90E-06 | 21.127 |
| genus | Streptococcus | rs4968759 | G | A | -0.052 | 0.011 | 3.78E-06 | 21.127 |
| genus | Anaerostipes | rs2014785 | C | T | 0.052 | 0.011 | 4.68E-06 | 21.125 |
| genus | LachnospiraceaeNK4A136group | rs28540839 | C | A | 0.051 | 0.011 | 9.34E-06 | 21.124 |
| genus | Adlercreutzia | rs13231526 | A | C | 0.143 | 0.031 | 4.81E-06 | 21.123 |
| genus | LachnospiraceaeNK4A136group | rs35965011 | T | C | -0.053 | 0.011 | 5.92E-06 | 21.122 |
| genus | Anaerotruncus | rs1322804 | T | G | 0.076 | 0.017 | 1.98E-06 | 21.121 |
| genus | LachnospiraceaeND3007group | rs9926643 | G | A | -0.053 | 0.012 | 4.69E-06 | 21.121 |
| genus | Bifidobacterium | rs540489 | G | T | -0.064 | 0.014 | 5.19E-06 | 21.121 |
| genus | Blautia | rs682885 | G | A | -0.049 | 0.011 | 4.49E-06 | 21.119 |
| genus | Streptococcus | rs6563952 | C | G | 0.083 | 0.018 | 5.82E-06 | 21.118 |
| genus | Howardella | rs17648393 | T | C | -0.106 | 0.023 | 4.78E-06 | 21.116 |
| genus | Lachnoclostridium | rs6046009 | C | T | -0.049 | 0.011 | 4.67E-06 | 21.115 |
| genus | Bacteroides | rs68118234 | T | C | -0.077 | 0.017 | 5.65E-06 | 21.115 |
| genus | Anaerostipes | rs2804209 | T | C | -0.050 | 0.011 | 4.84E-06 | 21.115 |
| genus | Bifidobacterium | rs3099429 | T | A | 0.073 | 0.016 | 4.73E-06 | 21.114 |
| genus | Butyrivibrio | rs11153290 | C | T | -0.110 | 0.024 | 4.89E-06 | 21.114 |
| genus | Peptococcus | rs10499019 | A | C | 0.134 | 0.029 | 1.88E-06 | 21.114 |
| genus | Butyrivibrio | rs11584386 | A | G | 0.172 | 0.037 | 5.80E-06 | 21.112 |
| genus | Blautia | rs73219072 | T | C | 0.057 | 0.012 | 4.05E-06 | 21.111 |
| genus | RuminococcaceaeUCG002 | rs11241746 | C | G | -0.055 | 0.012 | 4.65E-06 | 21.111 |
| genus | Anaerostipes | rs2769409 | A | G | -0.050 | 0.011 | 4.91E-06 | 21.111 |
| genus | RuminococcaceaeUCG003 | rs507766 | T | C | 0.054 | 0.012 | 4.66E-06 | 21.109 |
| genus | Eggerthella | rs12155081 | C | T | -0.091 | 0.020 | 4.65E-06 | 21.108 |
| genus | Akkermansia | rs12501826 | G | C | 0.067 | 0.014 | 4.11E-06 | 21.107 |
| genus | Butyricimonas | rs71428626 | T | G | -0.133 | 0.029 | 4.80E-06 | 21.107 |
| genus | Parasutterella | rs2863760 | A | G | -0.062 | 0.014 | 4.30E-06 | 21.106 |
| genus | Butyricimonas | rs9657374 | T | C | 0.068 | 0.015 | 4.50E-06 | 21.106 |
| genus | Anaerostipes | rs2769408 | A | G | -0.050 | 0.011 | 4.87E-06 | 21.105 |
| genus | Ruminiclostridium6 | rs72991535 | G | T | 0.136 | 0.030 | 4.95E-06 | 21.105 |
| genus | LachnospiraceaeNK4A136group | rs57090479 | T | C | -0.053 | 0.011 | 7.00E-06 | 21.104 |
| genus | Ruminiclostridium5 | rs928336 | A | G | 0.050 | 0.011 | 4.08E-06 | 21.100 |
| genus |  | rs12889086 | A | G | 0.053 | 0.012 | 4.64E-06 | 21.100 |
| genus | Ruminiclostridium9 | rs10936145 | A | G | -0.059 | 0.013 | 6.34E-06 | 21.099 |
| genus | Parabacteroides | rs114875383 | C | T | 0.102 | 0.022 | 2.22E-06 | 21.098 |
| genus | Lactococcus | rs72799250 | T | C | -0.197 | 0.043 | 1.63E-06 | 21.096 |
| genus | Flavonifractor | rs12794291 | A | G | 0.073 | 0.016 | 3.62E-06 | 21.095 |
| genus | Peptococcus | rs36121075 | G | A | -0.141 | 0.031 | 6.99E-06 | 21.094 |
| genus | RuminococcaceaeUCG009 | rs4079028 | T | C | 0.092 | 0.020 | 3.28E-06 | 21.094 |
| genus | Anaerostipes | rs10885747 | C | T | -0.051 | 0.011 | 5.09E-06 | 21.094 |
| genus | Akkermansia | rs12416161 | C | T | 0.076 | 0.017 | 3.73E-06 | 21.092 |
| genus | LachnospiraceaeUCG004 | rs11849978 | C | A | 0.058 | 0.013 | 5.07E-06 | 21.090 |
| genus | Ruminococcus2 | rs4799823 | T | C | 0.084 | 0.018 | 5.40E-06 | 21.089 |
| genus | Intestinibacter | rs6875660 | T | C | 0.089 | 0.019 | 3.06E-06 | 21.088 |
| genus | Romboutsia | rs11221428 | C | T | -0.073 | 0.016 | 6.49E-06 | 21.088 |
| genus | Anaerostipes | rs3932552 | C | T | -0.050 | 0.011 | 4.93E-06 | 21.088 |
| genus | Bifidobacterium | rs75344046 | T | C | 0.232 | 0.051 | 4.86E-06 | 21.088 |
| genus | Sutterella | rs27641 | A | C | -0.058 | 0.013 | 5.00E-06 | 21.087 |
| genus | LachnospiraceaeUCG008 | rs34065175 | G | A | -0.078 | 0.017 | 4.49E-06 | 21.087 |
| genus | Parasutterella | rs2346210 | A | G | -0.073 | 0.016 | 5.13E-06 | 21.087 |
| genus | Methanobrevibacter | rs7601415 | T | A | -0.110 | 0.024 | 5.73E-06 | 21.086 |
| genus |  | rs10808115 | C | A | -0.050 | 0.011 | 4.42E-06 | 21.085 |
| genus | Sellimonas | rs6433790 | T | C | -0.139 | 0.030 | 3.98E-06 | 21.084 |
| genus | RuminococcaceaeUCG003 | rs507855 | A | G | 0.054 | 0.012 | 4.75E-06 | 21.081 |
| genus | LachnospiraceaeNK4A136group | rs34212776 | A | G | -0.053 | 0.011 | 6.13E-06 | 21.080 |
| genus | Coprococcus2 | rs73314336 | A | G | -0.065 | 0.014 | 4.82E-06 | 21.080 |
| genus | LachnospiraceaeUCG001 | rs75206442 | T | C | 0.106 | 0.023 | 5.64E-06 | 21.080 |
| genus | RikenellaceaeRC9gutgroup | rs10895318 | A | G | 0.113 | 0.025 | 5.63E-06 | 21.080 |
| genus |  | rs34195214 | C | G | 0.167 | 0.036 | 4.17E-06 | 21.080 |
| genus | Prevotella9 | rs1304512 | A | G | 0.076 | 0.017 | 5.29E-06 | 21.078 |
| genus |  | rs2166943 | C | A | 0.057 | 0.012 | 5.28E-06 | 21.077 |
| genus | RuminococcaceaeUCG002 | rs76847269 | G | A | 0.164 | 0.036 | 5.17E-06 | 21.077 |
| genus | Ruminococcus1 | rs4755336 | A | G | -0.051 | 0.011 | 5.21E-06 | 21.077 |
| genus | Parasutterella | rs1403393 | A | G | -0.074 | 0.016 | 4.92E-06 | 21.076 |
| genus |  | rs35106244 | C | T | -0.052 | 0.011 | 4.68E-06 | 21.076 |
| genus | Oxalobacter | rs6128962 | A | G | -0.096 | 0.021 | 4.93E-06 | 21.075 |
| genus | Catenibacterium | rs629326 | T | G | -0.117 | 0.025 | 5.45E-06 | 21.074 |
| genus |  | rs2973295 | G | A | 0.090 | 0.020 | 4.64E-06 | 21.074 |
| genus |  | rs12136548 | T | C | 0.090 | 0.020 | 3.10E-06 | 21.074 |
| genus |  | rs398277 | C | A | -0.054 | 0.012 | 4.35E-06 | 21.072 |
| genus | Sutterella | rs1536384 | T | C | -0.057 | 0.012 | 4.11E-06 | 21.067 |
| genus | Collinsella | rs73052258 | A | G | 0.093 | 0.020 | 1.72E-06 | 21.067 |
| genus | Intestinibacter | rs7034161 | A | T | -0.104 | 0.023 | 4.13E-06 | 21.066 |
| genus | Butyrivibrio | rs406774 | C | T | -0.121 | 0.026 | 4.56E-06 | 21.065 |
| genus | LachnospiraceaeUCG004 | rs2444793 | T | C | -0.054 | 0.012 | 4.77E-06 | 21.064 |
| genus | Anaerostipes | rs2769401 | A | G | -0.050 | 0.011 | 5.00E-06 | 21.064 |
| genus | RuminococcaceaeUCG004 | rs2061707 | A | G | 0.122 | 0.027 | 3.72E-06 | 21.062 |
| genus | Lactococcus | rs12614854 | C | T | -0.197 | 0.043 | 1.42E-06 | 21.062 |
| genus | Roseburia | rs4748237 | C | G | 0.049 | 0.011 | 4.67E-06 | 21.062 |
| genus | Anaerotruncus | rs56406841 | T | A | 0.076 | 0.017 | 2.10E-06 | 21.061 |
| genus | Anaerotruncus | rs1923717 | A | T | 0.076 | 0.017 | 2.12E-06 | 21.060 |
| genus | Bifidobacterium | rs13020688 | A | G | 0.056 | 0.012 | 4.07E-06 | 21.059 |
| genus | RuminococcaceaeUCG014 | rs13382668 | A | G | 0.057 | 0.012 | 4.09E-06 | 21.059 |
| genus | Anaerostipes | rs282969 | T | C | 0.077 | 0.017 | 2.00E-06 | 21.058 |
| genus | Tyzzerella3 | rs7158359 | A | G | -0.114 | 0.025 | 4.40E-06 | 21.058 |
| genus | RuminococcaceaeUCG004 | rs77877730 | T | G | 0.126 | 0.028 | 4.55E-06 | 21.058 |
| genus | Sutterella | rs9537941 | A | C | -0.057 | 0.012 | 4.15E-06 | 21.057 |
| genus | Butyrivibrio | rs12726892 | C | T | 0.179 | 0.039 | 4.77E-06 | 21.055 |
| genus | Anaerotruncus | rs9594208 | T | C | 0.076 | 0.017 | 2.11E-06 | 21.055 |
| genus | Butyricimonas | rs1338636 | C | T | 0.065 | 0.014 | 3.83E-06 | 21.054 |
| genus | Subdoligranulum | rs17122478 | G | C | -0.050 | 0.011 | 4.74E-06 | 21.054 |
| genus | Anaerostipes | rs2769402 | C | T | -0.050 | 0.011 | 5.00E-06 | 21.053 |
| genus | Anaerostipes | rs2804200 | G | C | -0.050 | 0.011 | 5.00E-06 | 21.052 |
| genus | Ruminiclostridium9 | rs13033315 | A | T | 0.051 | 0.011 | 5.68E-06 | 21.051 |
| genus | Coprococcus1 | rs62341981 | A | G | -0.085 | 0.019 | 4.98E-06 | 21.050 |
| genus | Streptococcus | rs10860565 | G | A | -0.078 | 0.017 | 7.74E-06 | 21.050 |
| genus | Lachnospira | rs2326833 | G | C | -0.078 | 0.017 | 4.60E-06 | 21.049 |
| genus | Barnesiella | rs12909713 | T | C | -0.055 | 0.012 | 4.95E-06 | 21.048 |
| genus | Oscillibacter | rs9295772 | A | G | -0.069 | 0.015 | 4.95E-06 | 21.047 |
| genus | Tyzzerella3 | rs7561370 | C | T | 0.131 | 0.029 | 1.52E-06 | 21.047 |
| genus | Eggerthella | rs67968595 | G | A | -0.091 | 0.020 | 5.12E-06 | 21.047 |
| genus | LachnospiraceaeFCS020group | rs9919338 | C | G | -0.055 | 0.012 | 4.91E-06 | 21.044 |
| genus | RuminococcaceaeUCG003 | rs8112983 | T | C | 0.055 | 0.012 | 5.36E-06 | 21.044 |
| genus | Bifidobacterium | rs309157 | G | A | 0.075 | 0.016 | 5.33E-06 | 21.042 |
| genus | Ruminococcus1 | rs4148904 | C | G | -0.053 | 0.012 | 5.12E-06 | 21.041 |
| genus |  | rs40656 | T | C | 0.143 | 0.031 | 8.62E-06 | 21.040 |
| genus | Marvinbryantia | rs3848386 | G | A | -0.061 | 0.013 | 4.43E-06 | 21.039 |
| genus | Victivallis | rs34702749 | A | T | -0.143 | 0.031 | 6.96E-06 | 21.039 |
| genus | Anaerotruncus | rs4436664 | G | C | 0.076 | 0.017 | 2.14E-06 | 21.038 |
| genus |  | rs113893692 | T | C | -0.185 | 0.040 | 5.76E-06 | 21.038 |
| genus | Oxalobacter | rs75151797 | G | C | 0.149 | 0.032 | 6.76E-06 | 21.037 |
| genus | Anaerostipes | rs10165968 | G | A | -0.087 | 0.019 | 4.47E-06 | 21.036 |
| genus | RuminococcaceaeNK4A214group | rs12731 | G | A | -0.053 | 0.012 | 4.87E-06 | 21.035 |
| genus | Ruminiclostridium5 | rs11010060 | A | G | -0.053 | 0.011 | 4.11E-06 | 21.035 |
| genus | Bifidobacterium | rs4988274 | T | A | 0.075 | 0.016 | 5.42E-06 | 21.033 |
| genus | Anaerostipes | rs2804201 | T | A | -0.050 | 0.011 | 5.04E-06 | 21.033 |
| genus | Lachnoclostridium | rs4405817 | A | G | -0.049 | 0.011 | 5.26E-06 | 21.033 |
| genus | FamilyXIIIAD3011group | rs2999594 | A | G | -0.055 | 0.012 | 4.72E-06 | 21.033 |
| genus | LachnospiraceaeUCG008 | rs11025183 | T | C | -0.090 | 0.020 | 5.55E-06 | 21.032 |
| genus | Prevotella9 | rs2672227 | A | G | -0.069 | 0.015 | 3.98E-06 | 21.031 |
| genus | Victivallis | rs12793955 | G | T | -0.145 | 0.032 | 5.81E-06 | 21.031 |
| genus |  | rs34877286 | G | T | -0.053 | 0.012 | 4.35E-06 | 21.030 |
| genus | Alistipes | rs7805099 | G | T | -0.050 | 0.011 | 4.78E-06 | 21.030 |
| genus | Anaerotruncus | rs34027519 | C | T | 0.076 | 0.017 | 2.09E-06 | 21.030 |
| genus | Dialister | rs7829832 | A | C | 0.061 | 0.013 | 3.89E-06 | 21.029 |
| genus | Oscillibacter | rs61883564 | G | A | -0.101 | 0.022 | 3.39E-06 | 21.029 |
| genus | Allisonella | rs1531440 | T | C | 0.138 | 0.030 | 4.99E-06 | 21.029 |
| genus | Veillonella | rs13073802 | C | T | 0.084 | 0.018 | 5.26E-06 | 21.028 |
| genus | Peptococcus | rs1596026 | A | G | -0.104 | 0.023 | 5.58E-06 | 21.028 |
| genus | RuminococcaceaeUCG009 | rs12508214 | T | C | -0.077 | 0.017 | 4.75E-06 | 21.026 |
| genus | Paraprevotella | rs55811511 | A | C | -0.098 | 0.021 | 5.81E-06 | 21.025 |
| genus | LachnospiraceaeNK4A136group | rs7831577 | G | A | -0.070 | 0.015 | 6.41E-06 | 21.025 |
| genus | LachnospiraceaeUCG008 | rs9877951 | C | T | -0.113 | 0.025 | 5.05E-06 | 21.024 |
| genus | Dialister | rs4753063 | A | G | -0.060 | 0.013 | 4.86E-06 | 21.023 |
| genus | Actinomyces | rs114313061 | A | C | 0.082 | 0.018 | 4.19E-06 | 21.022 |
| genus | Ruminiclostridium5 | rs10848563 | T | C | -0.049 | 0.011 | 5.13E-06 | 21.022 |
| genus | Intestinibacter | rs7691215 | G | T | 0.067 | 0.015 | 6.42E-06 | 21.022 |
| genus | Bifidobacterium | rs6430594 | A | G | 0.073 | 0.016 | 5.52E-06 | 21.020 |
| genus |  | rs28650295 | G | C | -0.054 | 0.012 | 4.31E-06 | 21.020 |
| genus | Ruminiclostridium6 | rs11035758 | A | G | 0.071 | 0.015 | 4.94E-06 | 21.020 |
| genus |  | rs8080469 | A | G | 0.049 | 0.011 | 3.50E-06 | 21.019 |
| genus | RuminococcaceaeUCG004 | rs7520435 | G | T | 0.106 | 0.023 | 3.16E-06 | 21.019 |
| genus | LachnospiraceaeFCS020group | rs9831105 | G | T | 0.056 | 0.012 | 5.09E-06 | 21.019 |
| genus | RuminococcaceaeUCG009 | rs7314333 | C | G | 0.080 | 0.017 | 4.90E-06 | 21.019 |
| genus | Butyricimonas | rs11595369 | C | T | 0.066 | 0.014 | 3.48E-06 | 21.019 |
| genus | Anaerostipes | rs1977454 | G | A | -0.050 | 0.011 | 5.07E-06 | 21.019 |
| genus | Paraprevotella | rs140997932 | C | T | -0.162 | 0.035 | 2.11E-06 | 21.018 |
| genus | Parasutterella | rs13103630 | C | G | 0.061 | 0.013 | 4.96E-06 | 21.018 |
| genus | Allisonella | rs898870 | G | A | 0.137 | 0.030 | 5.37E-06 | 21.018 |
| genus | Ruminiclostridium5 | rs11010061 | T | C | -0.053 | 0.011 | 4.15E-06 | 21.017 |
| genus | Bifidobacterium | rs117761464 | G | A | 0.231 | 0.050 | 4.94E-06 | 21.017 |
| genus | RuminococcaceaeUCG002 | rs12513663 | C | T | -0.055 | 0.012 | 5.45E-06 | 21.016 |
| genus | Anaerostipes | rs1927480 | G | A | -0.050 | 0.011 | 5.10E-06 | 21.016 |
| genus | Veillonella | rs11928782 | T | C | 0.075 | 0.016 | 3.98E-06 | 21.015 |
| genus | Sellimonas | rs2570224 | G | A | 0.115 | 0.025 | 4.86E-06 | 21.014 |
| genus |  | rs4073731 | C | T | 0.065 | 0.014 | 4.05E-06 | 21.013 |
| genus | Roseburia | rs28849840 | G | A | 0.052 | 0.011 | 5.33E-06 | 21.013 |
| genus |  | rs72783037 | A | C | 0.066 | 0.014 | 6.55E-06 | 21.012 |
| genus |  | rs34867396 | T | C | -0.105 | 0.023 | 7.06E-06 | 21.011 |
| genus | RuminococcaceaeUCG014 | rs73186226 | A | G | -0.099 | 0.022 | 6.72E-06 | 21.011 |
| genus |  | rs61267978 | C | T | 0.147 | 0.032 | 5.59E-06 | 21.010 |
| genus | Howardella | rs2154047 | A | C | -0.193 | 0.042 | 9.97E-06 | 21.010 |
| genus | Bilophila | rs10954637 | T | G | 0.063 | 0.014 | 6.42E-06 | 21.009 |
| genus | Coprococcus2 | rs35327820 | C | A | -0.064 | 0.014 | 3.39E-06 | 21.009 |
| genus | Odoribacter | rs75779223 | A | G | -0.123 | 0.027 | 4.67E-06 | 21.009 |
| genus | Ruminiclostridium5 | rs3759374 | T | C | 0.049 | 0.011 | 4.99E-06 | 21.008 |
| genus | RuminococcaceaeUCG003 | rs78907377 | G | A | -0.061 | 0.013 | 5.22E-06 | 21.008 |
| genus | Anaerotruncus | rs2224659 | A | G | 0.076 | 0.017 | 2.09E-06 | 21.007 |
| genus | Coprococcus1 | rs7784490 | G | C | -0.052 | 0.011 | 4.55E-06 | 21.007 |
| genus | LachnospiraceaeUCG001 | rs7034160 | A | G | -0.066 | 0.014 | 4.42E-06 | 21.007 |
| genus | Bilophila | rs72676854 | C | T | 0.123 | 0.027 | 5.62E-06 | 21.007 |
| genus | Coprococcus1 | rs62341982 | C | G | -0.085 | 0.019 | 5.03E-06 | 21.006 |
| genus | Oxalobacter | rs73733937 | A | G | -0.136 | 0.030 | 5.08E-06 | 21.006 |
| genus | Lachnoclostridium | rs6075501 | T | C | -0.049 | 0.011 | 5.34E-06 | 21.005 |
| genus | Eggerthella | rs1784405 | G | T | 0.091 | 0.020 | 5.25E-06 | 21.004 |
| genus | Parasutterella | rs12472351 | C | A | 0.061 | 0.013 | 4.83E-06 | 21.004 |
| genus | Oxalobacter | rs6071441 | C | T | -0.106 | 0.023 | 5.23E-06 | 21.004 |
| genus |  | rs2913110 | T | C | 0.105 | 0.023 | 4.56E-06 | 21.004 |
| genus | Akkermansia | rs1605923 | T | G | 0.066 | 0.014 | 4.49E-06 | 21.004 |
| genus | Alistipes | rs1899238 | C | T | -0.050 | 0.011 | 4.96E-06 | 21.003 |
| genus |  | rs61391935 | G | A | -0.074 | 0.016 | 2.70E-06 | 21.003 |
| genus | Intestinibacter | rs4937051 | T | C | -0.136 | 0.030 | 5.03E-06 | 21.003 |
| genus | RuminococcaceaeUCG013 | rs12639557 | A | G | -0.059 | 0.013 | 5.22E-06 | 21.003 |
| genus | Veillonella | rs1454956 | C | T | -0.075 | 0.016 | 5.47E-06 | 21.003 |
| genus | Butyrivibrio | rs75299367 | T | C | -0.203 | 0.044 | 4.51E-06 | 21.002 |
| genus | Coprococcus2 | rs2482516 | T | C | 0.075 | 0.016 | 4.72E-06 | 21.002 |
| genus | Anaerostipes | rs2804206 | T | G | -0.050 | 0.011 | 5.16E-06 | 21.002 |
| genus | Ruminiclostridium9 | rs62287897 | A | G | -0.053 | 0.012 | 5.30E-06 | 21.001 |
| genus | Anaerostipes | rs2804198 | T | C | -0.050 | 0.011 | 5.14E-06 | 20.999 |
| genus | RuminococcaceaeUCG003 | rs3013089 | A | G | -0.055 | 0.012 | 4.38E-06 | 20.998 |
| genus | Holdemania | rs34061796 | G | T | -0.134 | 0.029 | 3.38E-06 | 20.998 |
| genus | LachnospiraceaeFCS020group | rs12330379 | A | G | 0.056 | 0.012 | 5.17E-06 | 20.997 |
| genus | Eggerthella | rs1784446 | A | G | 0.091 | 0.020 | 5.23E-06 | 20.997 |
| genus | RuminococcaceaeUCG011 | rs12746001 | G | A | -0.115 | 0.025 | 5.64E-06 | 20.997 |
| genus | LachnospiraceaeUCG008 | rs72900010 | A | G | -0.108 | 0.024 | 5.37E-06 | 20.996 |
| genus | Coprobacter | rs11532348 | T | C | -0.104 | 0.023 | 5.71E-06 | 20.994 |
| genus | Dorea | rs73729431 | T | C | -0.137 | 0.030 | 3.17E-06 | 20.994 |
| genus | RikenellaceaeRC9gutgroup | rs4717843 | T | G | -0.119 | 0.026 | 4.72E-06 | 20.992 |
| genus | Phascolarctobacterium | rs1264461 | G | T | 0.076 | 0.017 | 5.66E-06 | 20.990 |
| genus | Bifidobacterium | rs139939202 | G | A | 0.075 | 0.016 | 6.82E-06 | 20.990 |
| genus | Hungatella | rs34284687 | G | A | -0.153 | 0.033 | 7.24E-06 | 20.990 |
| genus | FamilyXIIIAD3011group | rs16940167 | T | C | 0.073 | 0.016 | 3.91E-06 | 20.989 |
| genus |  | rs10144730 | C | T | -0.054 | 0.012 | 4.44E-06 | 20.988 |
| genus | Anaerotruncus | rs9347879 | C | T | 0.051 | 0.011 | 4.22E-06 | 20.988 |
| genus | Oxalobacter | rs725475 | T | C | -0.096 | 0.021 | 5.12E-06 | 20.987 |
| genus | RuminococcaceaeUCG011 | rs4946639 | T | C | -0.125 | 0.027 | 4.31E-06 | 20.986 |
| genus | Sellimonas | rs1972689 | G | A | 0.115 | 0.025 | 4.89E-06 | 20.986 |
| genus | Eggerthella | rs1784445 | G | C | 0.091 | 0.020 | 5.27E-06 | 20.986 |
| genus | Barnesiella | rs3816345 | C | T | 0.098 | 0.021 | 9.59E-06 | 20.985 |
| genus | Parasutterella | rs4405788 | C | T | 0.061 | 0.013 | 4.97E-06 | 20.985 |
| genus | RikenellaceaeRC9gutgroup | rs2292730 | G | A | 0.113 | 0.025 | 5.92E-06 | 20.982 |
| genus | Intestinimonas | rs6934519 | T | C | 0.069 | 0.015 | 8.57E-06 | 20.982 |
| genus | Lactococcus | rs6508726 | G | A | 0.113 | 0.025 | 4.47E-06 | 20.982 |
| genus | Intestinimonas | rs1859797 | A | G | 0.060 | 0.013 | 4.12E-06 | 20.981 |
| genus | DefluviitaleaceaeUCG011 | rs4344384 | T | G | 0.072 | 0.016 | 4.83E-06 | 20.981 |
| genus | LachnospiraceaeUCG008 | rs29325 | T | C | 0.078 | 0.017 | 5.28E-06 | 20.980 |
| genus | LachnospiraceaeNK4A136group | rs34315028 | C | A | -0.053 | 0.012 | 7.25E-06 | 20.980 |
| genus | Coprococcus1 | rs12794898 | T | G | 0.090 | 0.020 | 4.92E-06 | 20.980 |
| genus | Ruminiclostridium9 | rs60295840 | T | A | -0.053 | 0.012 | 5.44E-06 | 20.978 |
| genus | Parasutterella | rs764553 | G | A | -0.073 | 0.016 | 5.33E-06 | 20.978 |
| genus | Victivallis | rs9483239 | T | C | 0.113 | 0.025 | 5.48E-06 | 20.978 |
| genus | Roseburia | rs2366612 | C | T | 0.049 | 0.011 | 5.19E-06 | 20.977 |
| genus | Alistipes | rs11763175 | G | A | -0.050 | 0.011 | 4.90E-06 | 20.975 |
| genus | Parasutterella | rs55877868 | C | A | -0.104 | 0.023 | 2.87E-06 | 20.974 |
| genus | RuminococcaceaeUCG005 | rs7449320 | A | C | 0.060 | 0.013 | 4.81E-06 | 20.972 |
| genus | Anaerotruncus | rs1923719 | T | C | 0.076 | 0.017 | 2.25E-06 | 20.971 |
| genus | Anaerotruncus | rs9603204 | C | T | 0.076 | 0.017 | 2.25E-06 | 20.971 |
| genus | Anaerotruncus | rs1590305 | C | T | 0.076 | 0.017 | 2.22E-06 | 20.967 |
| genus | Turicibacter | rs2952020 | A | G | -0.076 | 0.017 | 5.63E-06 | 20.967 |
| genus | Methanobrevibacter | rs1869500 | A | C | -0.110 | 0.024 | 6.05E-06 | 20.965 |
| genus | Anaerotruncus | rs1590303 | C | A | 0.076 | 0.017 | 2.21E-06 | 20.965 |
| genus | Ruminiclostridium9 | rs12634971 | A | T | -0.053 | 0.012 | 5.34E-06 | 20.965 |
| genus | Akkermansia | rs113653192 | C | T | 0.108 | 0.024 | 4.27E-06 | 20.964 |
| genus | Hungatella | rs13112952 | C | T | -0.153 | 0.034 | 7.01E-06 | 20.963 |
| genus | Catenibacterium | rs77285108 | A | G | -0.162 | 0.035 | 3.63E-06 | 20.963 |
| genus | FamilyXIIIAD3011group | rs2999600 | C | G | -0.055 | 0.012 | 4.91E-06 | 20.963 |
| genus | Methanobrevibacter | rs41288287 | C | T | 0.207 | 0.045 | 6.15E-06 | 20.963 |
| genus | LachnospiraceaeUCG004 | rs2444826 | T | C | -0.054 | 0.012 | 5.04E-06 | 20.962 |
| genus | Methanobrevibacter | rs9594529 | A | G | 0.207 | 0.045 | 5.62E-06 | 20.962 |
| genus |  | rs73130967 | T | A | 0.077 | 0.017 | 3.71E-06 | 20.961 |
| genus | Peptococcus | rs10115097 | G | A | 0.087 | 0.019 | 4.92E-06 | 20.960 |
| genus |  | rs10140184 | C | A | 0.058 | 0.013 | 4.96E-06 | 20.959 |
| genus | LachnospiraceaeND3007group | rs9932436 | A | C | -0.053 | 0.012 | 5.13E-06 | 20.959 |
| genus | Prevotella9 | rs2683314 | A | G | -0.069 | 0.015 | 4.05E-06 | 20.958 |
| genus |  | rs10501370 | T | C | -0.116 | 0.025 | 5.42E-06 | 20.958 |
| genus | Subdoligranulum | rs7723123 | C | T | -0.080 | 0.017 | 6.11E-06 | 20.956 |
| genus | RuminococcaceaeUCG004 | rs1597584 | C | A | 0.126 | 0.028 | 4.78E-06 | 20.956 |
| genus | Anaerostipes | rs2250483 | C | A | -0.050 | 0.011 | 5.25E-06 | 20.956 |
| genus | Anaerostipes | rs1576147 | G | A | -0.050 | 0.011 | 5.26E-06 | 20.955 |
| genus | Catenibacterium | rs212388 | T | C | -0.116 | 0.025 | 5.89E-06 | 20.953 |
| genus | Subdoligranulum | rs2171249 | T | C | 0.107 | 0.023 | 4.51E-06 | 20.950 |
| genus | Fusicatenibacter | rs1864685 | C | A | -0.049 | 0.011 | 4.96E-06 | 20.949 |
| genus | Parasutterella | rs7955812 | G | C | -0.073 | 0.016 | 7.04E-06 | 20.949 |
| genus | Coprococcus3 | rs9941669 | C | T | -0.067 | 0.015 | 5.60E-06 | 20.947 |
| genus | LachnospiraceaeFCS020group | rs9816355 | T | C | 0.056 | 0.012 | 5.30E-06 | 20.947 |
| genus | Parasutterella | rs1403397 | T | A | -0.073 | 0.016 | 6.50E-06 | 20.947 |
| genus | RuminococcaceaeUCG010 | rs7935775 | T | A | -0.063 | 0.014 | 4.99E-06 | 20.945 |
| genus | Turicibacter | rs4247078 | G | C | 0.071 | 0.016 | 5.46E-06 | 20.945 |
| genus | Anaerostipes | rs1576144 | C | T | -0.050 | 0.011 | 5.24E-06 | 20.944 |
| genus | CandidatusSoleaferrea | rs10809135 | C | T | 0.083 | 0.018 | 5.47E-06 | 20.944 |
| genus | Alistipes | rs4718473 | C | T | -0.050 | 0.011 | 5.13E-06 | 20.943 |
| genus | LachnospiraceaeUCG001 | rs78848836 | G | A | -0.119 | 0.026 | 3.38E-06 | 20.942 |
| genus | Lachnoclostridium | rs2876527 | T | C | -0.049 | 0.011 | 4.66E-06 | 20.941 |
| genus | Paraprevotella | rs9915190 | C | A | -0.079 | 0.017 | 6.67E-06 | 20.940 |
| genus | Ruminococcus2 | rs55707116 | A | C | 0.087 | 0.019 | 8.01E-06 | 20.940 |
| genus |  | rs390352 | T | G | -0.054 | 0.012 | 4.63E-06 | 20.939 |
| genus | Subdoligranulum | rs12590822 | C | G | -0.050 | 0.011 | 5.43E-06 | 20.939 |
| genus | Victivallis | rs9483237 | G | A | 0.113 | 0.025 | 5.54E-06 | 20.938 |
| genus | Subdoligranulum | rs9813294 | G | A | -0.049 | 0.011 | 5.30E-06 | 20.938 |
| genus | Akkermansia | rs11724791 | G | A | 0.066 | 0.014 | 4.89E-06 | 20.938 |
| genus | Anaerofilum | rs356049 | A | G | 0.133 | 0.029 | 6.56E-06 | 20.936 |
| genus | Oscillibacter | rs2857265 | G | A | 0.095 | 0.021 | 6.23E-06 | 20.936 |
| genus | Phascolarctobacterium | rs12468366 | C | T | 0.063 | 0.014 | 4.48E-06 | 20.936 |
| genus | Coprobacter | rs12684609 | C | T | 0.101 | 0.022 | 6.10E-06 | 20.934 |
| genus |  | rs934940 | C | A | -0.105 | 0.023 | 2.74E-06 | 20.934 |
| genus | Lactococcus | rs1547980 | G | A | -0.196 | 0.043 | 1.54E-06 | 20.934 |
| genus | Escherichia | rs35555519 | G | C | 0.102 | 0.022 | 4.92E-06 | 20.933 |
| genus | Desulfovibrio | rs2853180 | T | A | 0.092 | 0.020 | 2.48E-06 | 20.933 |
| genus | Lachnospira | rs11887924 | G | A | 0.053 | 0.011 | 5.35E-06 | 20.933 |
| genus | LachnospiraceaeFCS020group | rs10936479 | G | T | 0.056 | 0.012 | 5.35E-06 | 20.932 |
| genus | Anaerostipes | rs2784812 | G | A | -0.050 | 0.011 | 5.30E-06 | 20.932 |
| genus | Sellimonas | rs2303443 | G | A | 0.115 | 0.025 | 5.07E-06 | 20.932 |
| genus | Lactococcus | rs6508727 | T | C | 0.113 | 0.025 | 4.41E-06 | 20.931 |
| genus | Allisonella | rs16919675 | T | C | 0.137 | 0.030 | 5.25E-06 | 20.931 |
| genus |  | rs606120 | G | A | 0.083 | 0.018 | 5.83E-06 | 20.931 |
| genus |  | rs11597105 | G | A | 0.115 | 0.025 | 6.95E-06 | 20.930 |
| genus |  | rs36021113 | G | A | -0.054 | 0.012 | 5.19E-06 | 20.930 |
| genus | LachnospiraceaeFCS020group | rs6764789 | G | A | 0.056 | 0.012 | 5.36E-06 | 20.929 |
| genus | Parasutterella | rs7974471 | C | A | 0.062 | 0.013 | 4.22E-06 | 20.929 |
| genus |  | rs57676887 | T | C | 0.147 | 0.032 | 5.84E-06 | 20.928 |
| genus | Phascolarctobacterium | rs1264475 | G | T | 0.076 | 0.017 | 5.32E-06 | 20.928 |
| genus | Barnesiella | rs73216113 | T | A | -0.061 | 0.013 | 4.54E-06 | 20.925 |
| genus | Oxalobacter | rs73733936 | T | C | -0.136 | 0.030 | 5.33E-06 | 20.925 |
| genus | Bifidobacterium | rs72974159 | C | T | 0.064 | 0.014 | 5.50E-06 | 20.925 |
| genus | Oscillibacter | rs6901560 | G | C | 0.086 | 0.019 | 6.21E-06 | 20.922 |
| genus | RuminococcaceaeUCG003 | rs2000751 | A | C | 0.056 | 0.012 | 3.24E-06 | 20.922 |
| genus | Butyrivibrio | rs7211005 | T | C | 0.123 | 0.027 | 4.14E-06 | 20.921 |
| genus | RuminococcaceaeUCG003 | rs485186 | A | G | 0.054 | 0.012 | 5.39E-06 | 20.921 |
| genus | Bifidobacterium | rs4954187 | C | T | 0.055 | 0.012 | 3.37E-06 | 20.921 |
| genus | Sellimonas | rs2570228 | T | C | 0.115 | 0.025 | 5.05E-06 | 20.919 |
| genus | Anaerotruncus | rs7329262 | A | G | 0.076 | 0.017 | 2.33E-06 | 20.919 |
| genus | Allisonella | rs34870571 | G | A | 0.137 | 0.030 | 5.42E-06 | 20.919 |
| genus | Ruminococcus1 | rs7582930 | T | G | 0.052 | 0.011 | 4.33E-06 | 20.917 |
| genus | Fusicatenibacter | rs12727625 | G | A | -0.057 | 0.012 | 4.12E-06 | 20.917 |
| genus | Dorea | rs3005511 | G | A | 0.052 | 0.011 | 5.29E-06 | 20.917 |
| genus | RikenellaceaeRC9gutgroup | rs386756927 | C | T | 0.113 | 0.025 | 6.14E-06 | 20.917 |
| genus | Lachnospira | rs13422545 | T | C | 0.051 | 0.011 | 5.10E-06 | 20.915 |
| genus | Anaerostipes | rs11812371 | C | G | -0.051 | 0.011 | 5.80E-06 | 20.915 |
| genus | Oscillibacter | rs9368568 | T | G | -0.069 | 0.015 | 5.47E-06 | 20.914 |
| genus | Butyrivibrio | rs10457241 | G | A | -0.110 | 0.024 | 5.26E-06 | 20.913 |
| genus | Intestinibacter | rs11722055 | C | G | 0.060 | 0.013 | 5.15E-06 | 20.912 |
| genus | Romboutsia | rs2883972 | A | G | 0.073 | 0.016 | 4.85E-06 | 20.912 |
| genus | CandidatusSoleaferrea | rs4678258 | C | T | 0.099 | 0.022 | 5.53E-06 | 20.912 |
| genus | Turicibacter | rs117846333 | C | A | 0.101 | 0.022 | 4.77E-06 | 20.912 |
| genus | RuminococcaceaeUCG002 | rs11084307 | T | C | -0.053 | 0.012 | 4.29E-06 | 20.911 |
| genus | Intestinimonas | rs10475099 | A | T | 0.117 | 0.026 | 4.06E-06 | 20.911 |
| genus | Parasutterella | rs7133089 | T | C | -0.073 | 0.016 | 5.43E-06 | 20.910 |
| genus | Tyzzerella3 | rs7333521 | C | T | -0.207 | 0.045 | 4.88E-06 | 20.908 |
| genus | Senegalimassilia | rs10972033 | G | T | 0.078 | 0.017 | 5.74E-06 | 20.908 |
| genus | Alistipes | rs8130320 | G | A | -0.049 | 0.011 | 4.84E-06 | 20.906 |
| genus | Coprococcus3 | rs11080344 | T | C | 0.052 | 0.011 | 4.79E-06 | 20.906 |
| genus | Anaerostipes | rs2769424 | C | T | -0.050 | 0.011 | 5.35E-06 | 20.906 |
| genus | Ruminiclostridium9 | rs73172134 | A | G | -0.058 | 0.013 | 7.08E-06 | 20.905 |
| genus | Akkermansia | rs6668273 | G | A | 0.065 | 0.014 | 4.99E-06 | 20.905 |
| genus | Lactobacillus | rs768252 | G | T | -0.079 | 0.017 | 5.09E-06 | 20.905 |
| genus | Akkermansia | rs59317921 | C | T | 0.063 | 0.014 | 4.51E-06 | 20.904 |
| genus | Subdoligranulum | rs3761728 | G | T | -0.054 | 0.012 | 3.87E-06 | 20.903 |
| genus | Paraprevotella | rs17109926 | G | A | -0.099 | 0.022 | 6.75E-06 | 20.903 |
| genus | LachnospiraceaeUCG008 | rs6813622 | G | T | 0.077 | 0.017 | 5.56E-06 | 20.903 |
| genus | Escherichia | rs592299 | C | T | -0.059 | 0.013 | 4.77E-06 | 20.903 |
| genus | Parasutterella | rs7302674 | G | T | -0.073 | 0.016 | 5.58E-06 | 20.902 |
| genus | Anaerostipes | rs3858323 | G | A | -0.051 | 0.011 | 5.83E-06 | 20.901 |
| genus | Tyzzerella3 | rs2326673 | C | A | -0.126 | 0.028 | 6.18E-06 | 20.900 |
| genus | LachnospiraceaeUCG001 | rs11790569 | G | C | -0.066 | 0.014 | 4.39E-06 | 20.900 |
| genus | Parasutterella | rs1403391 | C | T | -0.073 | 0.016 | 5.56E-06 | 20.900 |
| genus | LachnospiraceaeNC2004group | rs12732301 | A | G | 0.112 | 0.025 | 5.84E-06 | 20.900 |
| genus | Allisonella | rs1564048 | C | T | 0.137 | 0.030 | 5.54E-06 | 20.899 |
| genus | Ruminiclostridium6 | rs74930322 | C | T | 0.112 | 0.025 | 8.34E-06 | 20.898 |
| genus | Blautia | rs7860714 | G | A | -0.050 | 0.011 | 4.09E-06 | 20.897 |
| genus | Anaerotruncus | rs4514540 | C | T | 0.076 | 0.017 | 2.28E-06 | 20.897 |
| genus | Anaerotruncus | rs4578521 | C | T | 0.076 | 0.017 | 2.28E-06 | 20.897 |
| genus | Oscillibacter | rs11990279 | C | T | -0.082 | 0.018 | 4.94E-06 | 20.897 |
| genus | Erysipelatoclostridium | rs972352 | A | C | 0.086 | 0.019 | 5.42E-06 | 20.897 |
| genus | Butyrivibrio | rs11653345 | A | G | 0.119 | 0.026 | 4.26E-06 | 20.896 |
| genus | Parasutterella | rs11058437 | A | T | -0.073 | 0.016 | 5.47E-06 | 20.895 |
| genus | RuminococcaceaeUCG003 | rs11780331 | G | A | -0.061 | 0.013 | 5.64E-06 | 20.895 |
| genus | Streptococcus | rs9858426 | A | G | 0.105 | 0.023 | 6.36E-06 | 20.895 |
| genus | Butyrivibrio | rs11204311 | T | C | 0.119 | 0.026 | 4.25E-06 | 20.894 |
| genus |  | rs158487 | G | A | 0.053 | 0.012 | 7.52E-06 | 20.893 |
| genus | Intestinibacter | rs499573 | T | C | -0.133 | 0.029 | 6.01E-06 | 20.893 |
| genus | Intestinibacter | rs472889 | G | A | -0.133 | 0.029 | 6.01E-06 | 20.893 |
| genus | Prevotella9 | rs2943430 | C | T | -0.069 | 0.015 | 4.15E-06 | 20.893 |
| genus | Olsenella | rs7540303 | T | C | 0.108 | 0.024 | 5.32E-06 | 20.892 |
| genus | LachnospiraceaeUCG008 | rs7931514 | C | T | -0.118 | 0.026 | 5.75E-06 | 20.892 |
| genus | RuminococcaceaeUCG004 | rs73000234 | T | C | 0.121 | 0.027 | 4.49E-06 | 20.892 |
| genus | Ruminococcus1 | rs11038597 | C | T | -0.053 | 0.012 | 5.45E-06 | 20.892 |
| genus | Prevotella7 | rs79263163 | C | A | -0.144 | 0.032 | 7.51E-06 | 20.891 |
| genus | Allisonella | rs10817504 | A | G | 0.137 | 0.030 | 6.30E-06 | 20.890 |
| genus | Butyricimonas | rs78453362 | G | A | -0.149 | 0.033 | 4.06E-06 | 20.887 |
| genus | Butyricimonas | rs868015 | G | A | 0.066 | 0.014 | 4.11E-06 | 20.887 |
| genus | Ruminiclostridium6 | rs72640885 | T | A | 0.071 | 0.015 | 4.93E-06 | 20.887 |
| genus | FamilyXIIIAD3011group | rs6037920 | T | C | -0.133 | 0.029 | 5.02E-06 | 20.887 |
| genus | RuminococcaceaeUCG004 | rs11164621 | C | T | 0.121 | 0.027 | 4.62E-06 | 20.887 |
| genus | Oscillospira | rs8076323 | G | A | 0.072 | 0.016 | 5.61E-06 | 20.885 |
| genus | Fusicatenibacter | rs7069626 | C | T | 0.051 | 0.011 | 5.42E-06 | 20.885 |
| genus | Lachnospira | rs13414210 | T | C | 0.051 | 0.011 | 5.18E-06 | 20.885 |
| genus |  | rs10150913 | T | A | -0.054 | 0.012 | 4.68E-06 | 20.884 |
| genus | Anaerofilum | rs1563175 | C | A | 0.092 | 0.020 | 5.54E-06 | 20.884 |
| genus | Butyricicoccus | rs16831418 | G | T | 0.077 | 0.017 | 4.75E-06 | 20.883 |
| genus | Ruminococcus1 | rs1447569 | A | C | -0.053 | 0.012 | 5.58E-06 | 20.882 |
| genus | Parasutterella | rs7601728 | C | G | 0.061 | 0.013 | 5.16E-06 | 20.881 |
| genus | Anaerotruncus | rs7988812 | C | A | 0.076 | 0.017 | 2.32E-06 | 20.880 |
| genus | Odoribacter | rs78179984 | T | C | -0.123 | 0.027 | 5.12E-06 | 20.880 |
| genus | Turicibacter | rs73313230 | G | A | 0.101 | 0.022 | 4.90E-06 | 20.880 |
| genus |  | rs2765994 | T | C | 0.105 | 0.023 | 4.97E-06 | 20.879 |
| genus | LachnospiraceaeUCG010 | rs72894957 | A | G | 0.222 | 0.049 | 5.68E-06 | 20.879 |
| genus | LachnospiraceaeUCG010 | rs72894958 | T | C | 0.222 | 0.049 | 5.68E-06 | 20.879 |
| genus | Veillonella | rs2831938 | A | G | -0.075 | 0.016 | 5.47E-06 | 20.879 |
| genus | Intestinibacter | rs509873 | G | A | -0.133 | 0.029 | 6.03E-06 | 20.878 |
| genus | Allisonella | rs10981800 | C | G | 0.137 | 0.030 | 5.80E-06 | 20.878 |
| genus | Streptococcus | rs9903102 | A | C | -0.071 | 0.016 | 4.18E-06 | 20.878 |
| genus | RuminococcaceaeUCG003 | rs72674077 | G | A | -0.061 | 0.013 | 5.06E-06 | 20.876 |
| genus |  | rs28552670 | G | T | -0.054 | 0.012 | 4.65E-06 | 20.876 |
| genus |  | rs35482477 | C | G | 0.085 | 0.019 | 4.59E-06 | 20.876 |
| genus | ErysipelotrichaceaeUCG003 | rs62403468 | C | G | -0.072 | 0.016 | 4.56E-06 | 20.875 |
| genus |  | rs77700046 | C | T | -0.165 | 0.036 | 5.51E-06 | 20.873 |
| genus | Parasutterella | rs62273907 | G | A | 0.229 | 0.050 | 5.88E-06 | 20.873 |
| genus |  | rs73306417 | T | C | -0.074 | 0.016 | 2.98E-06 | 20.872 |
| genus | RuminococcaceaeUCG013 | rs11581881 | T | C | 0.066 | 0.014 | 4.73E-06 | 20.870 |
| genus | Senegalimassilia | rs11788122 | C | T | 0.078 | 0.017 | 6.19E-06 | 20.868 |
| genus | Intestinibacter | rs940025 | C | T | -0.136 | 0.030 | 5.39E-06 | 20.868 |
| genus | RuminococcaceaeUCG004 | rs6703720 | C | A | 0.126 | 0.028 | 5.04E-06 | 20.868 |
| genus | RuminococcaceaeUCG010 | rs34668774 | A | G | -0.068 | 0.015 | 5.71E-06 | 20.868 |
| genus | Parasutterella | rs7440410 | A | T | -0.061 | 0.013 | 4.80E-06 | 20.868 |
| genus | ErysipelotrichaceaeUCG003 | rs17798136 | A | G | 0.159 | 0.035 | 3.24E-06 | 20.867 |
| genus | Parabacteroides | rs12486680 | C | A | 0.102 | 0.022 | 3.03E-06 | 20.866 |
| genus | Anaerotruncus | rs5000542 | G | A | 0.076 | 0.017 | 2.33E-06 | 20.863 |
| genus | Parabacteroides | rs72893646 | T | A | -0.072 | 0.016 | 8.83E-06 | 20.863 |
| genus | Anaerotruncus | rs35078720 | C | G | 0.076 | 0.017 | 2.33E-06 | 20.862 |
| genus | Lachnospira | rs4923325 | T | C | -0.061 | 0.013 | 3.55E-06 | 20.861 |
| genus | Ruminiclostridium9 | rs12496662 | A | G | -0.052 | 0.011 | 5.76E-06 | 20.861 |
| genus | RuminococcaceaeUCG011 | rs9322807 | A | C | -0.136 | 0.030 | 4.73E-06 | 20.861 |
| genus | Turicibacter | rs113170866 | A | G | 0.101 | 0.022 | 5.04E-06 | 20.861 |
| genus | Phascolarctobacterium | rs1264463 | T | C | 0.076 | 0.017 | 5.56E-06 | 20.860 |
| genus | Eggerthella | rs1784407 | C | T | 0.091 | 0.020 | 5.66E-06 | 20.860 |
| genus | Turicibacter | rs8066252 | G | A | 0.101 | 0.022 | 5.11E-06 | 20.859 |
| genus | Eisenbergiella | rs12421677 | G | C | -0.115 | 0.025 | 5.08E-06 | 20.859 |
| genus |  | rs77352868 | G | A | -0.166 | 0.036 | 5.21E-06 | 20.858 |
| genus | FamilyXIIIAD3011group | rs35280517 | A | G | -0.084 | 0.018 | 3.73E-06 | 20.857 |
| genus | Akkermansia | rs72663787 | G | A | 0.066 | 0.015 | 4.90E-06 | 20.857 |
| genus | Victivallis | rs9492886 | T | C | 0.112 | 0.025 | 5.83E-06 | 20.857 |
| genus | FamilyXIIIAD3011group | rs1848458 | G | A | 0.069 | 0.015 | 7.44E-06 | 20.856 |
| genus | Roseburia | rs10508087 | G | C | -0.058 | 0.013 | 7.77E-06 | 20.856 |
| genus | Anaerostipes | rs3900777 | A | G | -0.108 | 0.024 | 4.19E-06 | 20.856 |
| genus | DefluviitaleaceaeUCG011 | rs12122999 | T | G | -0.083 | 0.018 | 4.83E-06 | 20.855 |
| genus | LachnospiraceaeUCG008 | rs61944774 | G | A | 0.180 | 0.039 | 6.34E-06 | 20.855 |
| genus | Veillonella | rs6656807 | G | A | 0.070 | 0.015 | 5.50E-06 | 20.855 |
| genus | Alistipes | rs11088466 | T | A | -0.049 | 0.011 | 4.99E-06 | 20.854 |
| genus | Anaerostipes | rs2225166 | C | T | -0.050 | 0.011 | 5.56E-06 | 20.854 |
| genus | Intestinibacter | rs500668 | A | G | -0.133 | 0.029 | 6.13E-06 | 20.854 |
| genus | Turicibacter | rs7199484 | A | G | -0.073 | 0.016 | 5.77E-06 | 20.853 |
| genus | Paraprevotella | rs76018378 | G | A | -0.131 | 0.029 | 8.48E-06 | 20.853 |
| genus | Anaerostipes | rs11197319 | T | G | -0.050 | 0.011 | 5.50E-06 | 20.853 |
| genus | Butyricicoccus | rs4380522 | T | C | -0.050 | 0.011 | 4.26E-06 | 20.853 |
| genus | Adlercreutzia | rs255373 | T | A | -0.071 | 0.016 | 5.29E-06 | 20.852 |
| genus | Marvinbryantia | rs2815603 | T | C | -0.073 | 0.016 | 6.03E-06 | 20.852 |
| genus | Ruminiclostridium9 | rs58891024 | A | G | -0.053 | 0.012 | 5.83E-06 | 20.852 |
| genus | Methanobrevibacter | rs9532770 | T | C | 0.128 | 0.028 | 6.50E-06 | 20.851 |
| genus | RuminococcaceaeUCG004 | rs6679803 | G | A | 0.079 | 0.017 | 4.61E-06 | 20.850 |
| genus | FamilyXIIIAD3011group | rs12096170 | G | A | -0.055 | 0.012 | 5.22E-06 | 20.850 |
| genus | Olsenella | rs1035588 | G | A | -0.108 | 0.024 | 4.86E-06 | 20.850 |
| genus | Parasutterella | rs56189010 | A | G | -0.072 | 0.016 | 5.58E-06 | 20.850 |
| genus | Bifidobacterium | rs73958614 | A | C | 0.093 | 0.020 | 9.36E-06 | 20.849 |
| genus | Sutterella | rs9563573 | C | T | -0.057 | 0.012 | 4.52E-06 | 20.849 |
| genus | Coprococcus1 | rs6084702 | A | G | 0.052 | 0.011 | 4.85E-06 | 20.849 |
| genus | LachnospiraceaeFCS020group | rs249938 | C | T | 0.116 | 0.025 | 8.59E-06 | 20.847 |
| genus | Bifidobacterium | rs17789633 | C | A | 0.055 | 0.012 | 5.06E-06 | 20.847 |
| genus | Lachnoclostridium | rs6045989 | G | A | -0.050 | 0.011 | 5.41E-06 | 20.846 |
| genus | RuminococcaceaeUCG003 | rs3013093 | T | C | -0.055 | 0.012 | 4.81E-06 | 20.845 |
| genus | LachnospiraceaeNK4A136group | rs35683282 | A | T | -0.053 | 0.012 | 7.46E-06 | 20.844 |
| genus | FamilyXIIIAD3011group | rs12316111 | G | A | -0.090 | 0.020 | 4.64E-06 | 20.844 |
| genus | Ruminiclostridium9 | rs45471897 | C | G | -0.052 | 0.011 | 5.76E-06 | 20.843 |
| genus | Coprococcus3 | rs10206264 | G | A | -0.070 | 0.015 | 4.97E-06 | 20.843 |
| genus | LachnospiraceaeFCS020group | rs10936480 | G | A | 0.056 | 0.012 | 5.60E-06 | 20.843 |
| genus |  | rs10134680 | T | C | -0.054 | 0.012 | 4.76E-06 | 20.843 |
| genus | CandidatusSoleaferrea | rs9973954 | G | A | 0.089 | 0.020 | 5.95E-06 | 20.842 |
| genus | Allisonella | rs2401384 | A | C | 0.137 | 0.030 | 5.68E-06 | 20.841 |
| genus | Bilophila | rs10230993 | T | C | 0.063 | 0.014 | 6.83E-06 | 20.840 |
| genus | Adlercreutzia | rs9637706 | T | C | -0.082 | 0.018 | 4.74E-06 | 20.840 |
| genus | Prevotella9 | rs1193289 | T | C | -0.069 | 0.015 | 4.45E-06 | 20.839 |
| genus | Allisonella | rs4743083 | A | C | 0.137 | 0.030 | 5.88E-06 | 20.838 |
| genus | Anaerostipes | rs2804202 | T | A | -0.050 | 0.011 | 5.59E-06 | 20.838 |
| genus | LachnospiraceaeFCS020group | rs9881026 | A | G | 0.056 | 0.012 | 5.58E-06 | 20.838 |
| genus | Butyrivibrio | rs12745648 | A | G | 0.170 | 0.037 | 6.48E-06 | 20.838 |
| genus | Anaerostipes | rs1581906 | T | A | -0.050 | 0.011 | 5.58E-06 | 20.836 |
| genus | Intestinibacter | rs506687 | C | T | -0.132 | 0.029 | 6.95E-06 | 20.836 |
| genus | Victivallis | rs3777494 | G | A | 0.112 | 0.025 | 5.78E-06 | 20.835 |
| genus | RikenellaceaeRC9gutgroup | rs6590972 | C | T | 0.113 | 0.025 | 6.42E-06 | 20.835 |
| genus | Escherichia | rs512624 | G | A | -0.059 | 0.013 | 4.83E-06 | 20.834 |
| genus | RuminococcaceaeUCG004 | rs12128743 | C | T | 0.125 | 0.027 | 5.50E-06 | 20.834 |
| genus | Alistipes | rs1689282 | C | A | -0.052 | 0.011 | 5.28E-06 | 20.833 |
| genus | RuminococcaceaeUCG011 | rs12730642 | C | T | -0.114 | 0.025 | 5.74E-06 | 20.832 |
| genus |  | rs12651215 | G | C | 0.103 | 0.023 | 5.37E-06 | 20.832 |
| genus | Victivallis | rs7761587 | A | G | 0.112 | 0.025 | 5.85E-06 | 20.832 |
| genus | Streptococcus | rs395407 | C | G | -0.079 | 0.017 | 4.37E-06 | 20.831 |
| genus | Anaerotruncus | rs7994056 | T | C | 0.075 | 0.017 | 2.39E-06 | 20.830 |
| genus | Anaerotruncus | rs9594205 | G | A | 0.075 | 0.017 | 2.39E-06 | 20.830 |
| genus | Anaerotruncus | rs9805695 | T | C | 0.075 | 0.017 | 2.39E-06 | 20.830 |
| genus | Turicibacter | rs9746981 | A | G | 0.101 | 0.022 | 5.26E-06 | 20.830 |
| genus | Turicibacter | rs116953690 | C | T | 0.101 | 0.022 | 5.20E-06 | 20.830 |
| genus | Anaerotruncus | rs1575381 | A | T | 0.075 | 0.017 | 2.39E-06 | 20.829 |
| genus | Subdoligranulum | rs72793514 | A | G | -0.142 | 0.031 | 9.78E-06 | 20.829 |
| genus |  | rs17169428 | A | T | -0.066 | 0.015 | 4.33E-06 | 20.827 |
| genus | Holdemania | rs113593397 | G | A | -0.129 | 0.028 | 9.36E-06 | 20.827 |
| genus | Coprococcus3 | rs34731438 | G | T | -0.063 | 0.014 | 7.71E-06 | 20.827 |
| genus | Methanobrevibacter | rs9594528 | A | G | 0.207 | 0.045 | 6.09E-06 | 20.826 |
| genus | RuminococcaceaeUCG004 | rs12127710 | A | G | 0.121 | 0.027 | 4.77E-06 | 20.826 |
| genus | Allisonella | rs16919661 | T | C | 0.137 | 0.030 | 5.56E-06 | 20.825 |
| genus |  | rs72836423 | G | T | -0.140 | 0.031 | 3.36E-06 | 20.824 |
| genus | Anaerostipes | rs1581903 | T | C | -0.050 | 0.011 | 5.65E-06 | 20.822 |
| genus | Parasutterella | rs7692363 | T | C | 0.061 | 0.013 | 5.77E-06 | 20.822 |
| genus | LachnospiraceaeUCG004 | rs9638635 | T | C | 0.054 | 0.012 | 4.81E-06 | 20.820 |
| genus | Intestinibacter | rs4698729 | C | T | 0.066 | 0.014 | 5.86E-06 | 20.819 |
| genus | LachnospiraceaeFCS020group | rs1530208 | A | G | 0.056 | 0.012 | 5.67E-06 | 20.819 |
| genus | Butyrivibrio | rs8074459 | A | G | 0.118 | 0.026 | 4.30E-06 | 20.819 |
| genus | Peptococcus | rs9398847 | A | G | 0.133 | 0.029 | 1.83E-06 | 20.819 |
| genus | FamilyXIIIUCG001 | rs13050307 | A | G | -0.075 | 0.017 | 6.21E-06 | 20.818 |
| genus | RuminococcaceaeNK4A214group | rs13087692 | G | T | 0.057 | 0.013 | 8.69E-06 | 20.818 |
| genus |  | rs145236068 | C | T | 0.204 | 0.045 | 4.66E-06 | 20.818 |
| genus | Lachnoclostridium | rs78068103 | G | A | 0.089 | 0.019 | 3.67E-06 | 20.814 |
| genus | Parasutterella | rs737482 | G | A | 0.061 | 0.013 | 4.32E-06 | 20.814 |
| genus | Sellimonas | rs2623259 | C | A | 0.115 | 0.025 | 5.39E-06 | 20.813 |
| genus | Oxalobacter | rs6993398 | A | G | 0.127 | 0.028 | 7.13E-06 | 20.813 |
| genus | Lachnoclostridium | rs4738679 | A | G | -0.052 | 0.011 | 4.42E-06 | 20.813 |
| genus | LachnospiraceaeNC2004group | rs36062763 | C | T | 0.112 | 0.025 | 5.25E-06 | 20.813 |
| genus | Anaerostipes | rs2804197 | T | C | -0.050 | 0.011 | 5.66E-06 | 20.812 |
| genus | RuminococcaceaeUCG011 | rs4945699 | C | T | -0.136 | 0.030 | 5.14E-06 | 20.811 |
| genus | Ruminiclostridium5 | rs2791343 | C | T | 0.052 | 0.011 | 5.54E-06 | 20.810 |
| genus | Phascolarctobacterium | rs130483 | G | A | 0.066 | 0.014 | 6.79E-06 | 20.810 |
| genus | Butyricimonas | rs2591802 | C | A | 0.066 | 0.015 | 4.01E-06 | 20.810 |
| genus | Subdoligranulum | rs17122503 | G | A | -0.050 | 0.011 | 6.01E-06 | 20.808 |
| genus | Peptococcus | rs7766680 | C | G | 0.098 | 0.021 | 3.51E-06 | 20.806 |
| genus | Allisonella | rs34905303 | T | C | 0.137 | 0.030 | 6.01E-06 | 20.805 |
| genus | Allisonella | rs35778718 | A | C | 0.137 | 0.030 | 6.01E-06 | 20.805 |
| genus | Eggerthella | rs1784447 | G | A | 0.090 | 0.020 | 5.79E-06 | 20.804 |
| genus | Turicibacter | rs75622696 | C | G | 0.096 | 0.021 | 4.61E-06 | 20.803 |
| genus | Parasutterella | rs10847029 | G | A | -0.073 | 0.016 | 5.61E-06 | 20.802 |
| genus | Butyrivibrio | rs17163238 | A | G | 0.141 | 0.031 | 5.51E-06 | 20.802 |
| genus | Senegalimassilia | rs62556649 | A | C | 0.078 | 0.017 | 6.22E-06 | 20.801 |
| genus | Ruminococcus2 | rs4386032 | C | T | -0.056 | 0.012 | 6.06E-06 | 20.800 |
| genus | Subdoligranulum | rs72695075 | A | G | -0.051 | 0.011 | 5.81E-06 | 20.799 |
| genus | LachnospiraceaeFCS020group | rs9858926 | T | A | 0.056 | 0.012 | 5.73E-06 | 20.799 |
| genus | RuminococcaceaeUCG004 | rs1992964 | A | G | 0.077 | 0.017 | 5.81E-06 | 20.798 |
| genus |  | rs78250280 | A | G | 0.075 | 0.016 | 3.36E-06 | 20.798 |
| genus | LachnospiraceaeFCS020group | rs1530209 | T | C | 0.056 | 0.012 | 5.73E-06 | 20.798 |
| genus | Actinomyces | rs61937253 | C | T | 0.081 | 0.018 | 4.90E-06 | 20.798 |
| genus | Allisonella | rs35110698 | C | T | -0.146 | 0.032 | 5.72E-06 | 20.797 |
| genus | Ruminiclostridium5 | rs78912587 | A | G | 0.081 | 0.018 | 8.40E-06 | 20.797 |
| genus | Slackia | rs347676 | G | A | 0.085 | 0.019 | 5.32E-06 | 20.797 |
| genus | RuminococcaceaeUCG003 | rs2061406 | G | A | 0.071 | 0.016 | 2.73E-06 | 20.797 |
| genus | RuminococcaceaeUCG003 | rs11666792 | G | A | 0.054 | 0.012 | 6.11E-06 | 20.796 |
| genus | Oscillibacter | rs3829748 | G | A | 0.095 | 0.021 | 6.60E-06 | 20.795 |
| genus | Parasutterella | rs7958917 | T | C | -0.073 | 0.016 | 5.88E-06 | 20.795 |
| genus | Anaerotruncus | rs4435115 | G | A | 0.075 | 0.017 | 2.46E-06 | 20.794 |
| genus | Ruminiclostridium9 | rs73154304 | T | G | -0.058 | 0.013 | 7.18E-06 | 20.794 |
| genus | Faecalibacterium | rs11776390 | C | T | -0.078 | 0.017 | 6.40E-06 | 20.793 |
| genus | Streptococcus | rs55950941 | T | C | -0.061 | 0.013 | 7.56E-06 | 20.793 |
| genus | Ruminiclostridium9 | rs1017203 | T | A | -0.090 | 0.020 | 6.21E-06 | 20.793 |
| genus | Alistipes | rs1960663 | T | C | -0.050 | 0.011 | 5.50E-06 | 20.792 |
| genus |  | rs7867296 | G | T | -0.093 | 0.020 | 6.46E-06 | 20.792 |
| genus | Allisonella | rs16919506 | C | T | 0.136 | 0.030 | 6.07E-06 | 20.791 |
| genus | Ruminococcus1 | rs10929659 | G | A | 0.051 | 0.011 | 4.62E-06 | 20.790 |
| genus | Holdemania | rs9542258 | C | T | 0.075 | 0.016 | 7.92E-06 | 20.787 |
| genus |  | rs7620601 | C | T | 0.057 | 0.012 | 5.81E-06 | 20.785 |
| genus |  | rs12652689 | G | A | -0.105 | 0.023 | 7.78E-06 | 20.785 |
| genus |  | rs12513491 | C | G | -0.092 | 0.020 | 4.55E-06 | 20.783 |
| genus | Catenibacterium | rs73128290 | G | A | 0.130 | 0.028 | 4.29E-06 | 20.783 |
| genus | Sellimonas | rs113379006 | C | T | -0.163 | 0.036 | 7.21E-06 | 20.782 |
| genus |  | rs62449732 | C | T | 0.076 | 0.017 | 4.06E-06 | 20.779 |
| genus |  | rs12251446 | A | G | -0.165 | 0.036 | 5.61E-06 | 20.779 |
| genus | Adlercreutzia | rs1919502 | A | G | -0.082 | 0.018 | 4.73E-06 | 20.778 |
| genus | Intestinibacter | rs472722 | T | C | -0.133 | 0.029 | 6.37E-06 | 20.777 |
| genus | Intestinibacter | rs513164 | A | G | -0.133 | 0.029 | 6.37E-06 | 20.777 |
| genus | Ruminococcus1 | rs7929114 | G | A | -0.050 | 0.011 | 5.50E-06 | 20.777 |
| genus | Butyricimonas | rs1862649 | A | G | 0.113 | 0.025 | 4.76E-06 | 20.777 |
| genus | Collinsella | rs7283749 | C | T | 0.076 | 0.017 | 4.48E-06 | 20.777 |
| genus | Parasutterella | rs7162222 | C | G | 0.061 | 0.013 | 5.59E-06 | 20.775 |
| genus | RuminococcaceaeUCG004 | rs9818949 | T | G | 0.086 | 0.019 | 5.39E-06 | 20.774 |
| genus | RuminococcaceaeUCG011 | rs12724778 | C | G | -0.114 | 0.025 | 5.91E-06 | 20.773 |
| genus | Barnesiella | rs77455852 | G | T | -0.089 | 0.020 | 3.16E-06 | 20.773 |
| genus | LachnospiraceaeUCG008 | rs7932624 | A | G | -0.089 | 0.019 | 5.52E-06 | 20.772 |
| genus | RuminococcaceaeUCG010 | rs686246 | G | A | -0.057 | 0.012 | 5.25E-06 | 20.772 |
| genus | LachnospiraceaeFCS020group | rs1254846 | A | G | 0.106 | 0.023 | 5.60E-06 | 20.771 |
| genus | Allisonella | rs35534888 | C | T | 0.136 | 0.030 | 6.05E-06 | 20.770 |
| genus | Bifidobacterium | rs73797465 | G | T | -0.095 | 0.021 | 4.38E-06 | 20.770 |
| genus | RuminococcaceaeUCG003 | rs485073 | A | G | 0.054 | 0.012 | 5.77E-06 | 20.768 |
| genus | Bifidobacterium | rs60253740 | G | T | 0.073 | 0.016 | 9.02E-06 | 20.768 |
| genus | Butyrivibrio | rs12529158 | G | A | -0.110 | 0.024 | 5.77E-06 | 20.767 |
| genus | Ruminiclostridium9 | rs7624184 | A | G | -0.053 | 0.012 | 6.01E-06 | 20.767 |
| genus | RuminococcaceaeUCG004 | rs114399077 | C | G | 0.127 | 0.028 | 6.00E-06 | 20.767 |
| genus | Holdemanella | rs4802175 | A | G | 0.079 | 0.017 | 4.70E-06 | 20.766 |
| genus |  | rs7880204 | C | T | -0.125 | 0.028 | 6.84E-06 | 20.766 |
| genus | RuminococcaceaeUCG004 | rs151198757 | T | C | 0.127 | 0.028 | 5.87E-06 | 20.766 |
| genus | Anaerotruncus | rs7989845 | A | G | 0.075 | 0.017 | 2.45E-06 | 20.766 |
| genus | Anaerotruncus | rs1575383 | A | G | 0.075 | 0.017 | 2.45E-06 | 20.764 |
| genus | Anaerotruncus | rs4575409 | C | T | 0.075 | 0.017 | 2.45E-06 | 20.764 |
| genus | Anaerotruncus | rs9603200 | G | A | 0.075 | 0.017 | 2.45E-06 | 20.764 |
| genus | Parasutterella | rs62273900 | G | C | 0.228 | 0.050 | 6.08E-06 | 20.762 |
| genus | Eggerthella | rs3851328 | G | T | -0.108 | 0.024 | 4.18E-06 | 20.762 |
| genus | Senegalimassilia | rs59250595 | T | C | 0.079 | 0.017 | 5.23E-06 | 20.761 |
| genus | Lactococcus | rs10425269 | A | G | 0.112 | 0.025 | 4.75E-06 | 20.761 |
| genus |  | rs11878088 | T | C | 0.146 | 0.032 | 6.34E-06 | 20.760 |
| genus | Bacteroides | rs480694 | T | A | -0.059 | 0.013 | 6.16E-06 | 20.760 |
| genus | LachnospiraceaeFCS020group | rs9837621 | A | G | 0.055 | 0.012 | 5.84E-06 | 20.759 |
| genus | Turicibacter | rs111598486 | C | T | 0.099 | 0.022 | 4.86E-06 | 20.759 |
| genus | Lactococcus | rs11672300 | G | C | 0.112 | 0.025 | 4.75E-06 | 20.759 |
| genus | Ruminococcus1 | rs78613526 | A | G | 0.167 | 0.037 | 5.11E-06 | 20.757 |
| genus | RuminococcaceaeUCG014 | rs439810 | C | G | -0.058 | 0.013 | 7.04E-06 | 20.757 |
| genus | Sellimonas | rs2570258 | T | G | 0.114 | 0.025 | 5.52E-06 | 20.756 |
| genus | Ruminococcus2 | rs1819812 | T | G | 0.084 | 0.018 | 5.28E-06 | 20.756 |
| genus | Holdemanella | rs8113760 | A | G | 0.079 | 0.017 | 4.62E-06 | 20.756 |
| genus | Subdoligranulum | rs17122480 | C | T | -0.050 | 0.011 | 6.32E-06 | 20.755 |
| genus |  | rs72864816 | G | T | 0.150 | 0.033 | 5.96E-06 | 20.755 |
| genus | Eggerthella | rs1327257 | A | G | -0.189 | 0.041 | 7.23E-06 | 20.754 |
| genus | Lactococcus | rs9309252 | A | G | -0.141 | 0.031 | 3.59E-06 | 20.754 |
| genus | Eisenbergiella | rs9652495 | A | T | 0.102 | 0.022 | 4.99E-06 | 20.753 |
| genus | LachnospiraceaeFCS020group | rs6775964 | A | G | 0.055 | 0.012 | 5.91E-06 | 20.752 |
| genus | FamilyXIIIAD3011group | rs2999599 | C | T | -0.055 | 0.012 | 5.48E-06 | 20.752 |
| genus |  | rs506897 | G | C | -0.049 | 0.011 | 5.37E-06 | 20.751 |
| genus |  | rs66746423 | T | C | 0.075 | 0.016 | 6.11E-06 | 20.749 |
| genus | Butyrivibrio | rs17539197 | G | C | -0.109 | 0.024 | 5.94E-06 | 20.749 |
| genus | Marvinbryantia | rs1893909 | G | A | -0.124 | 0.027 | 4.82E-06 | 20.748 |
| genus | LachnospiraceaeNK4A136group | rs36112081 | A | G | -0.052 | 0.012 | 7.78E-06 | 20.748 |
| genus | Butyrivibrio | rs240974 | T | C | -0.110 | 0.024 | 5.87E-06 | 20.747 |
| genus | Anaerostipes | rs7095798 | C | T | -0.050 | 0.011 | 6.03E-06 | 20.747 |
| genus | LachnospiraceaeNK4A136group | rs7073658 | G | T | -0.050 | 0.011 | 5.27E-06 | 20.746 |
| genus | RuminococcaceaeUCG010 | rs8056635 | T | C | 0.065 | 0.014 | 7.09E-06 | 20.745 |
| genus | LachnospiraceaeFCS020group | rs12489461 | A | G | 0.055 | 0.012 | 5.90E-06 | 20.744 |
| genus | Anaerotruncus | rs17055723 | C | T | 0.075 | 0.017 | 2.58E-06 | 20.744 |
| genus | Olsenella | rs6046522 | T | C | 0.123 | 0.027 | 4.48E-06 | 20.743 |
| genus | Adlercreutzia | rs10233106 | T | C | 0.140 | 0.031 | 5.51E-06 | 20.743 |
| genus |  | rs55939577 | A | G | -0.054 | 0.012 | 4.98E-06 | 20.743 |
| genus |  | rs11256282 | A | G | 0.155 | 0.034 | 7.70E-06 | 20.742 |
| genus | Anaerostipes | rs2804207 | C | T | -0.050 | 0.011 | 5.95E-06 | 20.742 |
| genus | Bifidobacterium | rs4954189 | G | A | 0.055 | 0.012 | 3.75E-06 | 20.741 |
| genus | RuminococcaceaeUCG014 | rs74060145 | G | C | -0.116 | 0.025 | 8.71E-06 | 20.741 |
| genus | Holdemanella | rs45441892 | G | C | 0.079 | 0.017 | 4.95E-06 | 20.739 |
| genus | Romboutsia | rs34302036 | G | A | 0.055 | 0.012 | 5.88E-06 | 20.737 |
| genus | Alistipes | rs148278255 | A | G | -0.049 | 0.011 | 5.48E-06 | 20.735 |
| genus | Peptococcus | rs74592222 | A | G | 0.138 | 0.030 | 8.55E-06 | 20.735 |
| genus | Tyzzerella3 | rs76361865 | C | T | -0.125 | 0.027 | 9.66E-06 | 20.735 |
| genus | LachnospiraceaeNC2004group | rs1331592 | G | C | 0.095 | 0.021 | 5.34E-06 | 20.734 |
| genus | Ruminococcus2 | rs2368224 | G | T | 0.200 | 0.044 | 3.63E-06 | 20.734 |
| genus |  | rs11637981 | T | G | -0.073 | 0.016 | 5.44E-06 | 20.733 |
| genus | Erysipelatoclostridium | rs72671259 | T | G | 0.086 | 0.019 | 7.98E-06 | 20.733 |
| genus | Prevotella9 | rs2947262 | G | C | -0.069 | 0.015 | 4.89E-06 | 20.733 |
| genus | RuminococcaceaeUCG004 | rs7615599 | C | G | 0.092 | 0.020 | 6.17E-06 | 20.732 |
| genus | LachnospiraceaeUCG008 | rs7677909 | G | A | -0.078 | 0.017 | 6.47E-06 | 20.732 |
| genus |  | rs10141800 | C | T | -0.054 | 0.012 | 5.06E-06 | 20.732 |
| genus | Parasutterella | rs7691912 | T | C | 0.060 | 0.013 | 6.06E-06 | 20.729 |
| genus | Allisonella | rs62557463 | G | C | 0.136 | 0.030 | 5.82E-06 | 20.729 |
| genus | Coprobacter | rs77575394 | C | T | 0.246 | 0.054 | 5.96E-06 | 20.726 |
| genus | Subdoligranulum | rs4347803 | G | A | 0.163 | 0.036 | 3.48E-06 | 20.725 |
| genus | Dorea | rs12216169 | A | T | 0.088 | 0.019 | 5.33E-06 | 20.724 |
| genus | Adlercreutzia | rs306577 | T | C | -0.105 | 0.023 | 7.75E-06 | 20.724 |
| genus |  | rs1959383 | G | C | -0.054 | 0.012 | 5.09E-06 | 20.722 |
| genus | Tyzzerella3 | rs75690843 | T | G | -0.125 | 0.027 | 9.06E-06 | 20.722 |
| genus | Desulfovibrio | rs2590913 | A | G | 0.154 | 0.034 | 6.65E-06 | 20.721 |
| genus | RuminococcaceaeUCG003 | rs507711 | C | T | 0.054 | 0.012 | 5.69E-06 | 20.721 |
| genus | RuminococcaceaeUCG003 | rs3013095 | T | G | -0.055 | 0.012 | 5.14E-06 | 20.721 |
| genus |  | rs11764254 | A | C | 0.076 | 0.017 | 5.55E-06 | 20.719 |
| genus | RuminococcaceaeUCG003 | rs3013105 | T | C | -0.055 | 0.012 | 5.24E-06 | 20.718 |
| genus | Bifidobacterium | rs16832417 | C | T | 0.074 | 0.016 | 7.34E-06 | 20.717 |
| genus | LachnospiraceaeUCG001 | rs4981345 | C | T | -0.068 | 0.015 | 6.09E-06 | 20.717 |
| genus |  | rs8096953 | T | C | 0.146 | 0.032 | 6.48E-06 | 20.717 |
| genus | LachnospiraceaeUCG004 | rs2503728 | C | T | -0.054 | 0.012 | 5.72E-06 | 20.717 |
| genus | LachnospiraceaeUCG004 | rs7629954 | G | A | 0.108 | 0.024 | 5.77E-06 | 20.717 |
| genus | Allisonella | rs898869 | C | T | 0.136 | 0.030 | 6.42E-06 | 20.717 |
| genus | Howardella | rs17529367 | G | A | -0.136 | 0.030 | 5.62E-06 | 20.716 |
| genus | LachnospiraceaeUCG010 | rs77212921 | T | G | 0.082 | 0.018 | 4.02E-06 | 20.716 |
| genus | Bilophila | rs8013541 | T | A | -0.057 | 0.013 | 5.48E-06 | 20.716 |
| genus | LachnospiraceaeUCG004 | rs11621041 | C | A | 0.057 | 0.013 | 6.28E-06 | 20.715 |
| genus | Lactococcus | rs10407387 | A | T | 0.112 | 0.025 | 4.87E-06 | 20.714 |
| genus | Ruminiclostridium6 | rs9555756 | C | A | -0.080 | 0.018 | 7.10E-06 | 20.712 |
| genus | Holdemania | rs12865390 | T | C | -0.134 | 0.029 | 3.94E-06 | 20.712 |
| genus |  | rs485186 | A | G | -0.048 | 0.011 | 5.44E-06 | 20.711 |
| genus | Alistipes | rs12534925 | T | G | -0.049 | 0.011 | 5.52E-06 | 20.710 |
| genus | FamilyXIIIAD3011group | rs35202042 | G | T | -0.090 | 0.020 | 4.72E-06 | 20.710 |
| genus | Lachnoclostridium | rs1529367 | C | A | -0.052 | 0.011 | 5.43E-06 | 20.709 |
| genus | Bifidobacterium | rs7600925 | A | G | 0.093 | 0.020 | 9.63E-06 | 20.708 |
| genus | Parasutterella | rs7959030 | T | C | -0.073 | 0.016 | 6.14E-06 | 20.708 |
| genus | Eggerthella | rs2509016 | A | T | 0.090 | 0.020 | 6.17E-06 | 20.707 |
| genus | Parasutterella | rs6811436 | T | C | 0.060 | 0.013 | 6.07E-06 | 20.707 |
| genus | Oxalobacter | rs73027389 | A | C | 0.147 | 0.032 | 8.71E-06 | 20.706 |
| genus |  | rs504963 | G | A | -0.048 | 0.011 | 5.49E-06 | 20.705 |
| genus | Ruminiclostridium9 | rs11718554 | A | G | -0.053 | 0.012 | 6.23E-06 | 20.705 |
| genus | Eggerthella | rs1784433 | C | T | 0.090 | 0.020 | 6.31E-06 | 20.704 |
| genus | Holdemania | rs34600553 | G | T | -0.134 | 0.029 | 4.86E-06 | 20.704 |
| genus | Erysipelatoclostridium | rs153017 | A | G | -0.072 | 0.016 | 6.18E-06 | 20.704 |
| genus | LachnospiraceaeUCG004 | rs6656451 | T | C | -0.054 | 0.012 | 5.57E-06 | 20.703 |
| genus | Odoribacter | rs145109900 | A | G | -0.122 | 0.027 | 5.66E-06 | 20.703 |
| genus |  | rs35179274 | T | C | -0.063 | 0.014 | 5.76E-06 | 20.703 |
| genus | Butyricimonas | rs12769722 | C | T | 0.065 | 0.014 | 4.03E-06 | 20.703 |
| genus | Holdemania | rs55888180 | G | C | 0.129 | 0.028 | 5.89E-06 | 20.702 |
| genus | Turicibacter | rs12453630 | G | A | 0.098 | 0.022 | 5.05E-06 | 20.700 |
| genus | Odoribacter | rs78962466 | T | C | -0.122 | 0.027 | 5.66E-06 | 20.700 |
| genus | Ruminiclostridium9 | rs1369560 | T | A | -0.053 | 0.012 | 6.20E-06 | 20.699 |
| genus | Coprobacter | rs74919520 | A | G | 0.126 | 0.028 | 5.76E-06 | 20.699 |
| genus | Butyrivibrio | rs28375463 | C | T | 0.122 | 0.027 | 4.78E-06 | 20.698 |
| genus | Anaerostipes | rs11197344 | A | C | -0.050 | 0.011 | 5.92E-06 | 20.698 |
| genus |  | rs56367012 | A | G | 0.146 | 0.032 | 6.50E-06 | 20.697 |
| genus | Butyricimonas | rs9981005 | G | A | -0.117 | 0.026 | 6.90E-06 | 20.697 |
| genus | Bifidobacterium | rs4957061 | C | T | 0.053 | 0.012 | 5.78E-06 | 20.697 |
| genus | Sellimonas | rs2570233 | A | G | 0.114 | 0.025 | 5.69E-06 | 20.697 |
| genus | Romboutsia | rs9567264 | T | C | 0.058 | 0.013 | 5.76E-06 | 20.697 |
| genus | Allisonella | rs12553259 | A | C | 0.136 | 0.030 | 5.86E-06 | 20.696 |
| genus | Sellimonas | rs2593167 | T | G | 0.114 | 0.025 | 5.71E-06 | 20.695 |
| genus | RuminococcaceaeUCG002 | rs7342369 | A | C | -0.053 | 0.012 | 5.66E-06 | 20.694 |
| genus |  | rs11808093 | C | T | 0.079 | 0.017 | 2.97E-06 | 20.694 |
| genus | Erysipelatoclostridium | rs10868677 | C | T | -0.065 | 0.014 | 6.21E-06 | 20.693 |
| genus | LachnospiraceaeUCG001 | rs9386077 | A | G | 0.107 | 0.024 | 6.73E-06 | 20.693 |
| genus | Olsenella | rs12564117 | T | C | 0.108 | 0.024 | 5.90E-06 | 20.693 |
| genus | Eggerthella | rs7791112 | A | G | -0.091 | 0.020 | 6.04E-06 | 20.691 |
| genus | Sellimonas | rs1863429 | C | T | -0.115 | 0.025 | 6.02E-06 | 20.691 |
| genus | Turicibacter | rs112809819 | A | C | 0.098 | 0.022 | 5.07E-06 | 20.689 |
| genus | Sellimonas | rs2652597 | A | G | -0.117 | 0.026 | 6.50E-06 | 20.689 |
| genus | ChristensenellaceaeR | rs78521377 | T | C | 0.125 | 0.027 | 5.61E-06 | 20.689 |
| genus | Ruminococcus1 | rs11783695 | T | G | -0.073 | 0.016 | 4.73E-06 | 20.689 |
| genus | CandidatusSoleaferrea | rs6426133 | G | A | 0.104 | 0.023 | 6.27E-06 | 20.688 |
| genus | Oscillospira | rs2323504 | G | A | 0.071 | 0.016 | 6.26E-06 | 20.688 |
| genus | RuminococcaceaeUCG005 | rs10873449 | C | T | 0.065 | 0.014 | 4.11E-06 | 20.688 |
| genus | Turicibacter | rs4984326 | G | A | 0.071 | 0.016 | 6.26E-06 | 20.687 |
| genus | Parasutterella | rs6825923 | C | G | 0.060 | 0.013 | 5.84E-06 | 20.686 |
| genus | Victivallis | rs9311659 | T | C | 0.113 | 0.025 | 5.69E-06 | 20.686 |
| genus | Anaerotruncus | rs11894866 | A | G | 0.056 | 0.012 | 4.80E-06 | 20.685 |
| genus | Ruminococcus2 | rs4400279 | G | A | 0.055 | 0.012 | 5.80E-06 | 20.684 |
| genus | Intestinimonas | rs72982915 | T | C | 0.183 | 0.040 | 4.91E-06 | 20.682 |
| genus | Marvinbryantia | rs514566 | T | G | 0.062 | 0.014 | 5.28E-06 | 20.682 |
| genus | RuminococcaceaeUCG014 | rs13382580 | T | C | 0.056 | 0.012 | 4.98E-06 | 20.680 |
| genus | Sutterella | rs11591622 | G | T | -0.069 | 0.015 | 6.50E-06 | 20.680 |
| genus | Allisonella | rs13301045 | A | G | 0.136 | 0.030 | 6.27E-06 | 20.680 |
| genus | Roseburia | rs2943023 | C | T | 0.049 | 0.011 | 5.93E-06 | 20.680 |
| genus | Subdoligranulum | rs17122489 | C | G | -0.050 | 0.011 | 6.60E-06 | 20.679 |
| genus | Blautia | rs113271346 | T | C | 0.078 | 0.017 | 6.85E-06 | 20.679 |
| genus | Akkermansia | rs3995795 | T | C | 0.064 | 0.014 | 5.22E-06 | 20.679 |
| genus |  | rs79618182 | G | A | -0.091 | 0.020 | 7.20E-06 | 20.678 |
| genus | LachnospiraceaeFCS020group | . | A | C | 0.059 | 0.013 | 5.58E-06 | 20.678 |
| genus | Lachnospira | rs13430519 | T | C | 0.050 | 0.011 | 5.77E-06 | 20.678 |
| genus | Adlercreutzia | rs11663349 | A | G | -0.131 | 0.029 | 6.27E-06 | 20.677 |
| genus | Parasutterella | rs225465 | G | A | -0.065 | 0.014 | 5.78E-06 | 20.677 |
| genus | FamilyXIIIUCG001 | rs62414802 | T | C | -0.061 | 0.013 | 4.29E-06 | 20.676 |
| genus | Anaerotruncus | rs7140071 | T | C | 0.075 | 0.017 | 2.62E-06 | 20.675 |
| genus | Howardella | rs11871699 | A | T | -0.104 | 0.023 | 5.59E-06 | 20.674 |
| genus | Hungatella | rs66914762 | C | T | -0.152 | 0.033 | 9.17E-06 | 20.674 |
| genus | Lachnospira | rs11029029 | G | C | -0.061 | 0.013 | 3.93E-06 | 20.673 |
| genus |  | rs34008056 | G | C | 0.165 | 0.036 | 5.16E-06 | 20.673 |
| genus | Parasutterella | rs62273906 | A | G | 0.228 | 0.050 | 6.66E-06 | 20.673 |
| genus | Coprococcus2 | rs11734574 | T | C | -0.063 | 0.014 | 4.07E-06 | 20.673 |
| genus | LachnospiraceaeUCG008 | rs10741778 | A | G | -0.088 | 0.019 | 5.85E-06 | 20.673 |
| genus | Prevotella9 | rs10512344 | G | C | 0.247 | 0.054 | 3.19E-06 | 20.671 |
| genus | Lactococcus | rs1943431 | G | A | 0.104 | 0.023 | 5.04E-06 | 20.671 |
| genus | RuminococcaceaeUCG003 | rs34154358 | T | C | -0.061 | 0.013 | 5.69E-06 | 20.671 |
| genus | Butyricicoccus | rs60131947 | C | T | 0.077 | 0.017 | 4.38E-06 | 20.671 |
| genus | Anaerostipes | rs11197301 | A | G | -0.050 | 0.011 | 6.25E-06 | 20.671 |
| genus |  | rs329892 | A | G | -0.081 | 0.018 | 6.50E-06 | 20.671 |
| genus |  | rs7904900 | G | T | -0.168 | 0.037 | 5.87E-06 | 20.670 |
| genus | Victivallis | rs1358962 | C | G | 0.112 | 0.025 | 6.26E-06 | 20.669 |
| genus | Anaerotruncus | rs115414803 | C | A | -0.144 | 0.032 | 6.83E-06 | 20.669 |
| genus | Anaerostipes | rs7823228 | C | G | -0.062 | 0.014 | 5.84E-06 | 20.669 |
| genus | LachnospiraceaeNC2004group | rs12208226 | A | C | -0.155 | 0.034 | 9.75E-06 | 20.668 |
| genus | FamilyXIIIAD3011group | rs12809959 | C | T | -0.090 | 0.020 | 5.04E-06 | 20.668 |
| genus | RuminococcaceaeUCG002 | rs12985584 | C | T | -0.053 | 0.012 | 4.72E-06 | 20.667 |
| genus | Oscillibacter | rs75453768 | T | G | 0.122 | 0.027 | 5.35E-06 | 20.666 |
| genus | Sellimonas | rs4600608 | G | A | -0.137 | 0.030 | 4.95E-06 | 20.666 |
| genus | Butyrivibrio | . | T | A | 0.122 | 0.027 | 4.91E-06 | 20.665 |
| genus | Anaerotruncus | rs4421867 | T | C | 0.075 | 0.017 | 2.59E-06 | 20.665 |
| genus |  | rs1321623 | A | G | 0.060 | 0.013 | 6.13E-06 | 20.664 |
| genus |  | rs35778972 | C | T | 0.165 | 0.036 | 5.18E-06 | 20.664 |
| genus | Anaerotruncus | rs1923716 | C | A | 0.075 | 0.017 | 2.52E-06 | 20.663 |
| genus |  | rs62011174 | A | C | -0.054 | 0.012 | 5.28E-06 | 20.663 |
| genus | Fusicatenibacter | rs2001236 | G | C | -0.063 | 0.014 | 4.71E-06 | 20.663 |
| genus | RuminococcaceaeUCG004 | rs2376261 | A | C | 0.083 | 0.018 | 5.03E-06 | 20.662 |
| genus | Paraprevotella | rs17178062 | A | G | -0.079 | 0.017 | 7.71E-06 | 20.662 |
| genus | Sellimonas | rs2554336 | T | C | 0.114 | 0.025 | 5.77E-06 | 20.662 |
| genus | Prevotella9 | rs1193308 | G | A | -0.069 | 0.015 | 5.27E-06 | 20.662 |
| genus | Bifidobacterium | rs861392 | T | C | 0.055 | 0.012 | 4.87E-06 | 20.661 |
| genus | Parasutterella | rs823424 | A | G | -0.071 | 0.016 | 4.95E-06 | 20.661 |
| genus | Fusicatenibacter | rs11248242 | G | C | 0.051 | 0.011 | 6.25E-06 | 20.661 |
| genus | Veillonella | rs34085484 | T | C | 0.088 | 0.019 | 6.06E-06 | 20.661 |
| genus | Anaerostipes | rs2804224 | G | C | -0.050 | 0.011 | 6.11E-06 | 20.660 |
| genus | LachnospiraceaeFCS020group | rs9821978 | T | C | 0.055 | 0.012 | 6.16E-06 | 20.659 |
| genus | Allisonella | rs35737497 | G | A | 0.136 | 0.030 | 6.08E-06 | 20.659 |
| genus | Holdemania | rs79845664 | G | A | -0.128 | 0.028 | 9.99E-06 | 20.659 |
| genus | Eggerthella | rs2509017 | C | T | 0.090 | 0.020 | 6.32E-06 | 20.658 |
| genus | RuminococcaceaeUCG011 | rs2523007 | C | T | -0.106 | 0.023 | 5.54E-06 | 20.658 |
| genus | Eisenbergiella | rs4523231 | C | T | 0.114 | 0.025 | 7.80E-06 | 20.658 |
| genus | Odoribacter | rs10423795 | T | C | 0.055 | 0.012 | 6.58E-06 | 20.657 |
| genus | Prevotella9 | rs11199734 | T | A | 0.077 | 0.017 | 7.00E-06 | 20.657 |
| genus | Bifidobacterium | rs2278682 | G | C | 0.072 | 0.016 | 5.45E-06 | 20.657 |
| genus | Holdemania | rs76426015 | G | T | -0.128 | 0.028 | 9.99E-06 | 20.656 |
| genus | CandidatusSoleaferrea | rs386526 | G | C | 0.082 | 0.018 | 8.33E-06 | 20.655 |
| genus | Turicibacter | rs10852603 | T | C | -0.073 | 0.016 | 6.37E-06 | 20.655 |
| genus | Allisonella | rs4742688 | G | T | 0.136 | 0.030 | 6.45E-06 | 20.654 |
| genus |  | rs10144298 | T | G | -0.054 | 0.012 | 5.23E-06 | 20.654 |
| genus | Parasutterella | rs898888 | A | G | 0.060 | 0.013 | 5.85E-06 | 20.654 |
| genus | Ruminiclostridium9 | rs7556466 | G | A | 0.056 | 0.012 | 5.64E-06 | 20.653 |
| genus | Holdemanella | rs62113381 | C | T | -0.105 | 0.023 | 5.54E-06 | 20.653 |
| genus | Anaerotruncus | rs1575382 | G | A | 0.075 | 0.017 | 2.64E-06 | 20.653 |
| genus | Victivallis | rs7744734 | A | C | 0.112 | 0.025 | 6.70E-06 | 20.653 |
| genus | Catenibacterium | rs7742829 | T | C | 0.114 | 0.025 | 5.61E-06 | 20.652 |
| genus |  | rs12980122 | G | C | -0.108 | 0.024 | 5.02E-06 | 20.652 |
| genus | Peptococcus | rs113946406 | A | G | 0.109 | 0.024 | 6.20E-06 | 20.651 |
| genus | Faecalibacterium | rs114946999 | T | C | -0.086 | 0.019 | 5.70E-06 | 20.649 |
| genus | Enterorhabdus | rs9470637 | T | A | -0.076 | 0.017 | 5.70E-06 | 20.649 |
| genus | Ruminococcus1 | rs10769163 | A | G | -0.051 | 0.011 | 6.53E-06 | 20.648 |
| genus | Sellimonas | rs2436661 | G | A | 0.114 | 0.025 | 5.84E-06 | 20.647 |
| genus | Streptococcus | rs60486012 | A | G | -0.091 | 0.020 | 6.55E-06 | 20.647 |
| genus | Eggerthella | rs1711432 | C | G | 0.090 | 0.020 | 6.47E-06 | 20.647 |
| genus | Ruminococcus1 | rs7114779 | C | A | -0.051 | 0.011 | 6.53E-06 | 20.646 |
| genus | Prevotella9 | rs1193305 | G | A | -0.069 | 0.015 | 5.14E-06 | 20.645 |
| genus | Coprobacter | rs189356 | A | G | 0.078 | 0.017 | 6.26E-06 | 20.645 |
| genus | Bacteroides | rs66710942 | T | C | 0.049 | 0.011 | 5.86E-06 | 20.644 |
| genus | RuminococcaceaeUCG002 | rs56266601 | C | T | -0.053 | 0.012 | 4.76E-06 | 20.644 |
| genus | RuminococcaceaeUCG004 | rs12126530 | C | T | 0.125 | 0.027 | 6.05E-06 | 20.644 |
| genus | Lachnospira | rs35418758 | C | A | 0.086 | 0.019 | 8.30E-06 | 20.643 |
| genus | Anaerostipes | rs11197324 | G | A | -0.050 | 0.011 | 6.18E-06 | 20.642 |
| genus | Olsenella | rs11736534 | G | A | -0.105 | 0.023 | 5.98E-06 | 20.641 |
| genus | RuminococcaceaeUCG013 | rs7730740 | T | C | 0.066 | 0.015 | 3.35E-06 | 20.641 |
| genus | Actinomyces | rs35011108 | G | A | 0.233 | 0.051 | 6.34E-06 | 20.641 |
| genus |  | rs8022000 | C | T | -0.054 | 0.012 | 5.30E-06 | 20.640 |
| genus |  | rs117175461 | C | T | -0.111 | 0.024 | 3.09E-06 | 20.640 |
| genus | RuminococcaceaeUCG002 | rs56030423 | A | G | -0.098 | 0.022 | 6.30E-06 | 20.640 |
| genus | Streptococcus | rs7957504 | A | G | -0.078 | 0.017 | 9.06E-06 | 20.640 |
| genus | Oscillibacter | rs761240 | G | T | -0.177 | 0.039 | 2.04E-06 | 20.639 |
| genus | Gordonibacter | rs71545975 | G | A | -0.154 | 0.034 | 7.04E-06 | 20.639 |
| genus | LachnospiraceaeUCG010 | rs72894960 | G | A | 0.221 | 0.049 | 6.43E-06 | 20.639 |
| genus |  | rs2973296 | A | G | 0.089 | 0.020 | 5.76E-06 | 20.636 |
| genus | Holdemanella | rs11735091 | C | T | 0.086 | 0.019 | 7.43E-06 | 20.636 |
| genus | RuminococcaceaeUCG004 | rs7653648 | C | G | 0.070 | 0.016 | 6.57E-06 | 20.636 |
| genus | Enterorhabdus | rs11839306 | T | G | 0.086 | 0.019 | 5.26E-06 | 20.635 |
| genus | Odoribacter | rs6856150 | A | G | 0.088 | 0.019 | 6.06E-06 | 20.635 |
| genus | Oscillospira | rs8082421 | G | A | 0.071 | 0.016 | 6.29E-06 | 20.635 |
| genus | Sellimonas | rs1811326 | T | C | 0.114 | 0.025 | 5.85E-06 | 20.635 |
| genus |  | rs61277346 | T | C | 0.146 | 0.032 | 6.73E-06 | 20.634 |
| genus | RikenellaceaeRC9gutgroup | rs7130972 | G | A | 0.112 | 0.025 | 7.05E-06 | 20.634 |
| genus | Oscillospira | rs9712594 | A | C | -0.106 | 0.023 | 6.73E-06 | 20.633 |
| genus | Lactobacillus | rs12542112 | T | C | -0.078 | 0.017 | 5.64E-06 | 20.633 |
| genus | Desulfovibrio | rs6580353 | C | T | 0.077 | 0.017 | 4.94E-06 | 20.632 |
| genus |  | rs28803480 | C | G | -0.054 | 0.012 | 5.32E-06 | 20.632 |
| genus | Intestinimonas | rs4784055 | C | T | -0.175 | 0.039 | 8.72E-07 | 20.631 |
| genus | RuminococcaceaeNK4A214group | rs35559912 | C | T | -0.093 | 0.020 | 4.89E-06 | 20.629 |
| genus | Escherichia | rs2798105 | G | A | -0.101 | 0.022 | 8.25E-06 | 20.629 |
| genus | Anaerostipes | rs10509992 | A | G | -0.050 | 0.011 | 6.22E-06 | 20.629 |
| genus | Ruminiclostridium5 | rs11061971 | A | T | 0.049 | 0.011 | 5.79E-06 | 20.628 |
| genus |  | rs61534193 | G | A | -0.054 | 0.012 | 5.33E-06 | 20.628 |
| genus |  | rs12463876 | T | C | 0.059 | 0.013 | 6.34E-06 | 20.628 |
| genus | Subdoligranulum | rs8015544 | G | A | -0.050 | 0.011 | 6.70E-06 | 20.627 |
| genus | Ruminiclostridium5 | rs1929389 | A | G | 0.049 | 0.011 | 5.16E-06 | 20.627 |
| genus | Allisonella | rs35788892 | A | G | 0.136 | 0.030 | 6.52E-06 | 20.627 |
| genus | RuminococcaceaeUCG004 | rs11801437 | A | G | 0.083 | 0.018 | 5.11E-06 | 20.627 |
| genus | RuminococcaceaeUCG004 | rs10127873 | C | T | 0.083 | 0.018 | 5.11E-06 | 20.627 |
| genus | Paraprevotella | rs3008585 | T | C | 0.102 | 0.022 | 7.77E-06 | 20.627 |
| genus | Sellimonas | rs41816 | G | A | 0.132 | 0.029 | 8.39E-06 | 20.626 |
| genus | Paraprevotella | rs3801748 | A | G | 0.078 | 0.017 | 5.20E-06 | 20.624 |
| genus | Oscillibacter | rs9295776 | C | T | -0.068 | 0.015 | 6.25E-06 | 20.624 |
| genus | Collinsella | rs62102630 | G | A | -0.070 | 0.015 | 8.31E-06 | 20.624 |
| genus | Senegalimassilia | rs61944230 | A | G | 0.079 | 0.017 | 5.61E-06 | 20.624 |
| genus |  | rs79895140 | C | T | -0.064 | 0.014 | 8.62E-06 | 20.623 |
| genus | Bilophila | rs1263061 | G | A | -0.072 | 0.016 | 6.53E-06 | 20.623 |
| genus | Allisonella | rs1123379 | T | C | 0.136 | 0.030 | 6.49E-06 | 20.623 |
| genus | Anaerotruncus | rs10150232 | G | A | 0.057 | 0.012 | 6.68E-06 | 20.622 |
| genus | LachnospiraceaeFCS020group | rs9828890 | T | C | 0.055 | 0.012 | 6.24E-06 | 20.622 |
| genus |  | rs67307009 | A | G | 0.146 | 0.032 | 7.00E-06 | 20.621 |
| genus | Roseburia | rs12740451 | C | T | 0.070 | 0.015 | 7.34E-06 | 20.620 |
| genus | LachnospiraceaeUCG008 | rs73861213 | G | T | -0.112 | 0.025 | 6.02E-06 | 20.620 |
| genus | Lactococcus | rs6674304 | T | C | 0.201 | 0.044 | 6.18E-06 | 20.619 |
| genus | Phascolarctobacterium | rs74847270 | G | A | -0.105 | 0.023 | 5.73E-06 | 20.618 |
| genus | Peptococcus | rs5770862 | C | T | 0.162 | 0.036 | 3.22E-06 | 20.618 |
| genus | Dorea | rs1437568 | G | T | 0.051 | 0.011 | 6.19E-06 | 20.618 |
| genus |  | rs10138634 | A | G | -0.054 | 0.012 | 5.38E-06 | 20.618 |
| genus | Streptococcus | rs10993693 | A | G | -0.051 | 0.011 | 5.67E-06 | 20.617 |
| genus | Eisenbergiella | rs8025430 | C | T | 0.089 | 0.020 | 6.16E-06 | 20.617 |
| genus | Holdemania | rs111745969 | G | A | 0.121 | 0.027 | 3.71E-06 | 20.616 |
| genus | Subdoligranulum | rs75158211 | C | T | -0.072 | 0.016 | 7.52E-06 | 20.616 |
| genus | Paraprevotella | rs2367261 | G | A | -0.079 | 0.017 | 8.76E-06 | 20.615 |
| genus |  | rs12124711 | T | C | 0.089 | 0.020 | 3.59E-06 | 20.615 |
| genus | RuminococcaceaeNK4A214group | rs1374387 | T | G | -0.052 | 0.012 | 6.08E-06 | 20.615 |
| genus | RuminococcaceaeUCG011 | rs6799330 | T | G | 0.132 | 0.029 | 5.79E-06 | 20.615 |
| genus |  | rs1384962 | G | A | 0.121 | 0.027 | 6.99E-06 | 20.613 |
| genus | Akkermansia | rs72663756 | C | T | 0.067 | 0.015 | 4.80E-06 | 20.612 |
| genus | RuminococcaceaeUCG013 | rs4385846 | T | G | 0.060 | 0.013 | 6.46E-06 | 20.612 |
| genus | Turicibacter | rs7197190 | C | A | -0.073 | 0.016 | 6.63E-06 | 20.611 |
| genus | Butyrivibrio | rs9349693 | G | A | 0.118 | 0.026 | 5.55E-06 | 20.610 |
| genus |  | rs2105937 | G | A | 0.058 | 0.013 | 5.10E-06 | 20.610 |
| genus | LachnospiraceaeUCG010 | rs10414815 | C | T | 0.105 | 0.023 | 4.24E-06 | 20.610 |
| genus | Slackia | rs71815 | T | C | 0.084 | 0.019 | 5.93E-06 | 20.609 |
| genus | Ruminococcus1 | rs2012019 | T | C | -0.050 | 0.011 | 5.96E-06 | 20.609 |
| genus |  | rs12261262 | G | C | -0.163 | 0.036 | 3.51E-06 | 20.609 |
| genus | Lactobacillus | rs12693845 | T | C | -0.081 | 0.018 | 8.96E-06 | 20.608 |
| genus | Butyricimonas | rs10823645 | G | T | 0.064 | 0.014 | 5.28E-06 | 20.606 |
| genus |  | rs11750752 | T | C | 0.071 | 0.016 | 7.40E-06 | 20.606 |
| genus | Parasutterella | rs6809952 | A | G | -0.068 | 0.015 | 8.13E-06 | 20.606 |
| genus | FamilyXIIIUCG001 | rs12488120 | T | G | -0.063 | 0.014 | 5.72E-06 | 20.606 |
| genus | RikenellaceaeRC9gutgroup | rs80309088 | A | G | 0.174 | 0.038 | 4.56E-06 | 20.606 |
| genus | Parasutterella | rs740356 | A | G | -0.073 | 0.016 | 6.25E-06 | 20.606 |
| genus | Coprobacter | rs56691857 | A | C | -0.183 | 0.040 | 7.25E-06 | 20.605 |
| genus | Lactococcus | rs4805128 | C | T | 0.112 | 0.025 | 5.40E-06 | 20.605 |
| genus | RuminococcaceaeUCG011 | rs6789741 | G | T | 0.132 | 0.029 | 5.84E-06 | 20.605 |
| genus | LachnospiraceaeNK4A136group | rs307182 | T | A | 0.049 | 0.011 | 5.97E-06 | 20.604 |
| genus | Lactobacillus | rs1434764 | G | T | -0.078 | 0.017 | 5.98E-06 | 20.603 |
| genus | RuminococcaceaeUCG011 | rs9404559 | C | G | -0.134 | 0.030 | 5.52E-06 | 20.603 |
| genus | Alistipes | rs1837664 | A | C | -0.049 | 0.011 | 5.85E-06 | 20.603 |
| genus | Butyrivibrio | rs8070756 | T | C | 0.121 | 0.027 | 4.98E-06 | 20.602 |
| genus |  | rs605239 | G | A | 0.082 | 0.018 | 6.92E-06 | 20.600 |
| genus | Anaerotruncus | rs11841382 | T | G | 0.084 | 0.019 | 2.45E-06 | 20.600 |
| genus | Peptococcus | rs76697713 | A | C | 0.146 | 0.032 | 8.05E-06 | 20.600 |
| genus | Coprococcus3 | rs4368354 | G | A | -0.070 | 0.015 | 7.02E-06 | 20.599 |
| genus | Ruminiclostridium9 | rs55999561 | A | G | -0.052 | 0.011 | 6.61E-06 | 20.599 |
| genus | LachnospiraceaeFCS020group | rs9829411 | T | C | 0.055 | 0.012 | 6.32E-06 | 20.598 |
| genus | Anaerostipes | rs10885752 | A | C | -0.050 | 0.011 | 6.26E-06 | 20.596 |
| genus | RuminococcaceaeUCG014 | rs461493 | C | T | -0.057 | 0.013 | 7.24E-06 | 20.595 |
| genus | Peptococcus | rs77614201 | C | T | -0.176 | 0.039 | 5.68E-06 | 20.595 |
| genus | Oxalobacter | rs62281792 | A | G | 0.147 | 0.032 | 8.41E-06 | 20.594 |
| genus | Holdemania | rs9542254 | T | C | 0.073 | 0.016 | 8.25E-06 | 20.594 |
| genus | LachnospiraceaeUCG004 | rs2726805 | G | A | 0.055 | 0.012 | 6.30E-06 | 20.594 |
| genus | Allisonella | rs10981801 | C | A | 0.136 | 0.030 | 6.72E-06 | 20.591 |
| genus | Bifidobacterium | rs56100046 | T | A | 0.074 | 0.016 | 6.89E-06 | 20.591 |
| genus | Lactococcus | rs12984590 | T | C | 0.112 | 0.025 | 5.21E-06 | 20.591 |
| genus | Butyrivibrio | rs240954 | A | G | -0.110 | 0.024 | 6.35E-06 | 20.591 |
| genus | Holdemania | rs73139538 | A | G | -0.149 | 0.033 | 7.77E-06 | 20.590 |
| genus | Prevotella9 | rs1193287 | G | A | -0.069 | 0.015 | 5.00E-06 | 20.590 |
| genus | Anaerostipes | rs10787582 | C | T | -0.050 | 0.011 | 6.28E-06 | 20.589 |
| genus | Slackia | rs180884 | A | C | 0.084 | 0.019 | 6.04E-06 | 20.588 |
| genus | Akkermansia | rs72663782 | G | C | 0.066 | 0.014 | 5.25E-06 | 20.588 |
| genus | Parasutterella | rs11715853 | A | G | -0.066 | 0.015 | 6.23E-06 | 20.586 |
| genus | Turicibacter | rs113629870 | C | T | 0.089 | 0.020 | 5.23E-06 | 20.585 |
| genus |  | rs10892089 | G | C | -0.064 | 0.014 | 6.22E-06 | 20.585 |
| genus | LachnospiraceaeUCG001 | rs11788322 | A | T | -0.065 | 0.014 | 5.61E-06 | 20.582 |
| genus | Lachnoclostridium | rs1529368 | G | A | -0.052 | 0.011 | 5.83E-06 | 20.581 |
| genus |  | rs17326496 | C | T | 0.071 | 0.016 | 7.34E-06 | 20.581 |
| genus | Subdoligranulum | rs7651544 | C | A | -0.049 | 0.011 | 6.69E-06 | 20.580 |
| genus | Peptococcus | rs61995091 | T | G | 0.108 | 0.024 | 6.26E-06 | 20.579 |
| genus | Peptococcus | rs6573150 | G | A | 0.108 | 0.024 | 5.28E-06 | 20.579 |
| genus | Senegalimassilia | rs10221578 | A | G | 0.078 | 0.017 | 6.10E-06 | 20.578 |
| genus | Eggerthella | rs1784434 | A | G | 0.090 | 0.020 | 6.72E-06 | 20.578 |
| genus | Coprococcus3 | rs78686130 | C | T | -0.056 | 0.012 | 5.84E-06 | 20.577 |
| genus | RuminococcaceaeUCG005 | rs4545506 | C | T | -0.068 | 0.015 | 7.54E-06 | 20.577 |
| genus | RuminococcaceaeUCG005 | rs56163386 | C | T | -0.066 | 0.015 | 7.86E-06 | 20.577 |
| genus | RuminococcaceaeUCG003 | rs78048325 | A | G | -0.061 | 0.013 | 5.89E-06 | 20.576 |
| genus | Barnesiella | rs76181748 | T | C | -0.078 | 0.017 | 6.78E-06 | 20.575 |
| genus | Anaerotruncus | rs7155595 | A | C | 0.054 | 0.012 | 7.55E-06 | 20.575 |
| genus | Coprococcus1 | rs12193787 | T | C | -0.050 | 0.011 | 6.03E-06 | 20.574 |
| genus | LachnospiraceaeUCG010 | rs336138 | T | G | 0.078 | 0.017 | 7.48E-06 | 20.573 |
| genus | Oxalobacter | rs58504958 | A | G | 0.147 | 0.032 | 9.52E-06 | 20.573 |
| genus | Intestinibacter | rs2039380 | C | G | -0.108 | 0.024 | 8.60E-06 | 20.572 |
| genus | LachnospiraceaeNC2004group | rs34421765 | G | A | 0.112 | 0.025 | 7.35E-06 | 20.572 |
| genus | Oscillospira | rs62422654 | T | C | 0.090 | 0.020 | 6.47E-06 | 20.572 |
| genus | RuminococcaceaeUCG011 | rs10274562 | T | C | 0.111 | 0.024 | 6.50E-06 | 20.570 |
| genus | Parasutterella | rs3860462 | C | T | 0.060 | 0.013 | 6.06E-06 | 20.570 |
| genus | FamilyXIIIAD3011group | rs113394511 | A | T | -0.132 | 0.029 | 5.99E-06 | 20.570 |
| genus | RuminococcaceaeUCG010 | rs592858 | C | A | -0.058 | 0.013 | 5.85E-06 | 20.569 |
| genus |  | rs6426744 | A | G | 0.059 | 0.013 | 6.34E-06 | 20.569 |
| genus | Ruminiclostridium6 | rs11992182 | C | A | 0.063 | 0.014 | 4.65E-06 | 20.568 |
| genus | Anaerostipes | rs6823685 | A | C | -0.049 | 0.011 | 5.88E-06 | 20.568 |
| genus | Lactococcus | rs4806175 | A | G | 0.112 | 0.025 | 5.29E-06 | 20.568 |
| genus | Intestinibacter | rs12449107 | C | T | -0.147 | 0.032 | 5.26E-06 | 20.567 |
| genus | Dorea | . | C | T | 0.051 | 0.011 | 6.31E-06 | 20.567 |
| genus | Anaerostipes | rs2769378 | C | T | -0.050 | 0.011 | 6.43E-06 | 20.566 |
| genus | Terrisporobacter | rs11086039 | T | C | -0.080 | 0.018 | 6.19E-06 | 20.565 |
| genus | RuminococcaceaeUCG002 | rs362417 | C | G | -0.055 | 0.012 | 7.80E-06 | 20.564 |
| genus | Escherichia | rs2803250 | G | C | -0.101 | 0.022 | 8.76E-06 | 20.563 |
| genus | Allisonella | rs62557462 | T | C | 0.136 | 0.030 | 6.36E-06 | 20.563 |
| genus | Eisenbergiella | rs13258851 | G | A | 0.137 | 0.030 | 7.75E-06 | 20.563 |
| genus | Sellimonas | rs553697 | C | T | -0.154 | 0.034 | 6.13E-06 | 20.562 |
| genus | Senegalimassilia | rs13383270 | C | G | 0.077 | 0.017 | 6.04E-06 | 20.562 |
| genus | Hungatella | rs66548753 | G | T | -0.151 | 0.033 | 8.45E-06 | 20.562 |
| genus | Coprobacter | rs7679813 | C | A | -0.183 | 0.040 | 7.45E-06 | 20.561 |
| genus | Coprococcus3 | rs13385714 | C | T | -0.069 | 0.015 | 6.13E-06 | 20.561 |
| genus | Subdoligranulum | rs2022769 | T | C | -0.050 | 0.011 | 7.16E-06 | 20.561 |
| genus | Sellimonas | rs2623262 | T | C | 0.114 | 0.025 | 6.13E-06 | 20.561 |
| genus | LachnospiraceaeUCG004 | rs996183 | A | G | 0.057 | 0.013 | 6.65E-06 | 20.560 |
| genus |  | rs11171752 | T | C | 0.076 | 0.017 | 3.41E-06 | 20.558 |
| genus | LachnospiraceaeUCG001 | rs11788340 | A | G | -0.065 | 0.014 | 5.62E-06 | 20.557 |
| genus | Lactococcus | rs10425847 | T | C | 0.112 | 0.025 | 5.44E-06 | 20.556 |
| genus | LachnospiraceaeUCG001 | rs9390193 | A | G | 0.104 | 0.023 | 7.41E-06 | 20.555 |
| genus | Parasutterella | rs11058428 | C | T | -0.073 | 0.016 | 6.38E-06 | 20.555 |
| genus | ErysipelotrichaceaeUCG003 | rs75949021 | C | T | -0.170 | 0.037 | 3.58E-06 | 20.554 |
| genus | LachnospiraceaeNK4A136group | rs943880 | G | C | -0.052 | 0.012 | 9.18E-06 | 20.553 |
| genus | Sellimonas | rs1867984 | T | G | 0.114 | 0.025 | 6.11E-06 | 20.553 |
| genus | Ruminiclostridium5 | rs1929386 | C | G | 0.049 | 0.011 | 5.27E-06 | 20.551 |
| genus |  | rs10137837 | T | C | -0.054 | 0.012 | 5.56E-06 | 20.551 |
| genus | Akkermansia | rs61779207 | A | G | -0.076 | 0.017 | 6.32E-06 | 20.550 |
| genus | Anaerostipes | rs1590736 | A | G | -0.050 | 0.011 | 6.40E-06 | 20.550 |
| genus | Lactococcus | rs10425058 | T | G | 0.112 | 0.025 | 5.35E-06 | 20.550 |
| genus | Anaerostipes | rs1576146 | G | A | -0.050 | 0.011 | 6.46E-06 | 20.550 |
| genus | Senegalimassilia | rs61944227 | T | C | 0.079 | 0.017 | 5.80E-06 | 20.549 |
| genus | Faecalibacterium | rs10927394 | T | G | -0.232 | 0.051 | 7.02E-06 | 20.549 |
| genus | Coprococcus3 | rs13393804 | T | C | -0.069 | 0.015 | 6.26E-06 | 20.549 |
| genus |  | rs17172374 | C | A | 0.076 | 0.017 | 4.52E-06 | 20.549 |
| genus | Senegalimassilia | rs60364969 | C | T | -0.080 | 0.018 | 5.96E-06 | 20.548 |
| genus | Ruminiclostridium9 | rs11571293 | G | T | 0.051 | 0.011 | 7.02E-06 | 20.548 |
| genus |  | rs606082 | G | A | 0.082 | 0.018 | 7.02E-06 | 20.548 |
| genus | Sellimonas | rs114478966 | T | C | -0.162 | 0.036 | 8.02E-06 | 20.548 |
| genus | Flavonifractor | rs4949766 | C | T | -0.069 | 0.015 | 4.83E-06 | 20.547 |
| genus | Subdoligranulum | rs2572344 | A | G | -0.052 | 0.012 | 4.84E-06 | 20.547 |
| genus | Butyricimonas | rs11228830 | G | A | 0.135 | 0.030 | 6.55E-06 | 20.547 |
| genus | Paraprevotella | rs11654749 | G | T | -0.079 | 0.017 | 9.15E-06 | 20.546 |
| genus | RuminococcaceaeUCG003 | rs2359534 | C | T | -0.055 | 0.012 | 5.65E-06 | 20.546 |
| genus | Romboutsia | rs62504452 | G | A | -0.071 | 0.016 | 4.66E-06 | 20.544 |
| genus | Anaerotruncus | rs7334359 | C | T | 0.075 | 0.017 | 2.79E-06 | 20.544 |
| genus | Ruminiclostridium9 | rs79082720 | G | C | 0.093 | 0.020 | 6.47E-06 | 20.543 |
| genus | LachnospiraceaeNK4A136group | rs34513148 | A | G | -0.052 | 0.011 | 8.65E-06 | 20.542 |
| genus | Coprococcus1 | rs7761439 | T | C | -0.050 | 0.011 | 6.11E-06 | 20.542 |
| genus | Butyricimonas | rs326049 | G | C | 0.076 | 0.017 | 8.19E-06 | 20.542 |
| genus | Lachnoclostridium | rs6112331 | T | A | -0.048 | 0.011 | 5.85E-06 | 20.542 |
| genus | Bifidobacterium | rs12465599 | G | A | 0.054 | 0.012 | 6.27E-06 | 20.541 |
| genus | RuminococcaceaeUCG003 | rs11878908 | C | T | 0.054 | 0.012 | 6.98E-06 | 20.541 |
| genus | Bacteroides | rs6015624 | A | G | 0.075 | 0.017 | 5.21E-06 | 20.540 |
| genus |  | rs11740807 | A | T | 0.071 | 0.016 | 7.72E-06 | 20.539 |
| genus | Oxalobacter | rs76405250 | T | C | -0.135 | 0.030 | 6.49E-06 | 20.539 |
| genus | LachnospiraceaeFCS020group | rs1542073 | G | A | 0.055 | 0.012 | 6.56E-06 | 20.537 |
| genus |  | rs11875989 | C | A | 0.145 | 0.032 | 7.21E-06 | 20.536 |
| genus | RuminococcaceaeUCG014 | rs461159 | C | T | -0.057 | 0.013 | 7.64E-06 | 20.535 |
| genus | Paraprevotella | rs2058083 | C | T | -0.078 | 0.017 | 8.38E-06 | 20.535 |
| genus | LachnospiraceaeUCG001 | rs4982387 | T | C | -0.068 | 0.015 | 6.64E-06 | 20.535 |
| genus | RuminococcaceaeNK4A214group | rs147475196 | G | A | -0.134 | 0.030 | 4.72E-06 | 20.535 |
| genus | Bifidobacterium | rs4954556 | G | A | 0.117 | 0.026 | 3.85E-06 | 20.535 |
| genus | Roseburia | rs75326254 | T | C | -0.105 | 0.023 | 7.50E-06 | 20.533 |
| genus | Alistipes | rs62476323 | C | A | -0.049 | 0.011 | 6.29E-06 | 20.533 |
| genus | Blautia | rs2017197 | C | G | 0.115 | 0.025 | 8.26E-06 | 20.531 |
| genus | Holdemania | rs35409370 | T | G | -0.133 | 0.029 | 5.33E-06 | 20.531 |
| genus | Intestinibacter | rs543165 | C | T | -0.132 | 0.029 | 7.21E-06 | 20.530 |
| genus | Butyrivibrio | rs4627235 | G | A | -0.126 | 0.028 | 8.10E-06 | 20.530 |
| genus | Bifidobacterium | rs55809728 | G | A | 0.104 | 0.023 | 3.86E-06 | 20.529 |
| genus | Anaerotruncus | rs9594206 | G | A | 0.075 | 0.017 | 2.90E-06 | 20.528 |
| genus | Anaerostipes | rs10787583 | G | A | -0.050 | 0.011 | 6.48E-06 | 20.528 |
| genus |  | rs329889 | C | A | -0.081 | 0.018 | 7.32E-06 | 20.528 |
| genus | Alloprevotella | rs34619204 | A | G | -0.156 | 0.034 | 8.84E-06 | 20.528 |
| genus | RuminococcaceaeUCG011 | rs4568515 | T | C | 0.111 | 0.025 | 6.55E-06 | 20.527 |
| genus | Coprococcus3 | rs13247359 | A | G | 0.051 | 0.011 | 7.33E-06 | 20.527 |
| genus | Gordonibacter | rs16955299 | A | G | -0.196 | 0.043 | 6.37E-06 | 20.527 |
| genus | Streptococcus | rs57646748 | A | G | -0.091 | 0.020 | 5.48E-06 | 20.527 |
| genus | Coprococcus2 | rs1022078 | T | C | 0.065 | 0.014 | 5.50E-06 | 20.527 |
| genus | Oxalobacter | rs16845257 | C | T | 0.154 | 0.034 | 1.00E-05 | 20.526 |
| genus | Oscillibacter | rs16866373 | A | T | 0.095 | 0.021 | 8.38E-06 | 20.525 |
| genus | Alistipes | rs1822115 | T | G | -0.049 | 0.011 | 6.14E-06 | 20.524 |
| genus | Lachnoclostridium | rs6985620 | C | T | -0.051 | 0.011 | 5.74E-06 | 20.524 |
| genus | Actinomyces | rs2715439 | T | C | 0.075 | 0.016 | 6.27E-06 | 20.523 |
| genus | Hungatella | rs13126978 | A | G | -0.151 | 0.033 | 8.49E-06 | 20.523 |
| genus | Subdoligranulum | rs7674928 | C | G | -0.052 | 0.012 | 4.85E-06 | 20.522 |
| genus | Tyzzerella3 | rs112102233 | G | A | -0.216 | 0.048 | 6.18E-06 | 20.522 |
| genus | Sellimonas | rs2570260 | A | G | 0.114 | 0.025 | 6.24E-06 | 20.522 |
| genus | Anaerostipes | rs4498893 | A | G | -0.050 | 0.011 | 6.53E-06 | 20.522 |
| genus | LachnospiraceaeUCG004 | rs35182105 | G | A | -0.110 | 0.024 | 4.87E-06 | 20.522 |
| genus | Sellimonas | rs7968030 | T | A | -0.127 | 0.028 | 5.56E-06 | 20.518 |
| genus | Bifidobacterium | rs309113 | C | A | 0.071 | 0.016 | 5.85E-06 | 20.518 |
| genus |  | rs10747761 | A | G | 0.074 | 0.016 | 4.63E-06 | 20.517 |
| genus | Butyrivibrio | rs1002475 | C | T | -0.109 | 0.024 | 6.66E-06 | 20.515 |
| genus | Lactobacillus | rs12547259 | A | G | -0.078 | 0.017 | 6.27E-06 | 20.515 |
| genus | Olsenella | rs61090148 | G | A | -0.105 | 0.023 | 6.44E-06 | 20.515 |
| genus | LachnospiraceaeUCG004 | rs4567981 | A | T | -0.055 | 0.012 | 6.55E-06 | 20.513 |
| genus | Howardella | rs73165663 | T | C | -0.170 | 0.037 | 4.40E-06 | 20.512 |
| genus | Eisenbergiella | rs11027642 | T | C | 0.129 | 0.028 | 4.92E-06 | 20.512 |
| genus | Bifidobacterium | rs3769001 | A | G | 0.073 | 0.016 | 6.92E-06 | 20.512 |
| genus | Dialister | rs7816464 | C | T | 0.060 | 0.013 | 5.10E-06 | 20.512 |
| genus | Escherichia | rs113513883 | G | A | 0.172 | 0.038 | 5.28E-06 | 20.511 |
| genus |  | rs28820968 | G | A | -0.054 | 0.012 | 5.68E-06 | 20.510 |
| genus | Ruminiclostridium5 | rs4934686 | C | T | -0.052 | 0.012 | 6.50E-06 | 20.510 |
| genus | Slackia | rs58767323 | C | G | -0.103 | 0.023 | 4.60E-06 | 20.508 |
| genus | Sellimonas | rs2570253 | G | C | 0.114 | 0.025 | 6.31E-06 | 20.507 |
| genus | Sutterella | rs1145877 | G | A | -0.074 | 0.016 | 7.20E-06 | 20.507 |
| genus | ChristensenellaceaeR | rs62467127 | T | C | 0.114 | 0.025 | 3.25E-06 | 20.507 |
| genus | RuminococcaceaeUCG004 | rs1918882 | T | C | 0.114 | 0.025 | 4.06E-06 | 20.504 |
| genus | Ruminiclostridium9 | rs7137760 | T | C | 0.051 | 0.011 | 7.07E-06 | 20.503 |
| genus | RuminococcaceaeUCG003 | rs281379 | G | A | 0.055 | 0.012 | 7.02E-06 | 20.503 |
| genus | Holdemanella | rs777392 | A | T | 0.089 | 0.020 | 7.64E-06 | 20.503 |
| genus | ChristensenellaceaeR | rs1971264 | G | A | 0.124 | 0.027 | 6.25E-06 | 20.503 |
| genus | Collinsella | rs75672793 | G | A | -0.109 | 0.024 | 6.14E-06 | 20.503 |
| genus | Butyricimonas | rs2924726 | G | A | 0.067 | 0.015 | 5.90E-06 | 20.502 |
| genus | Sellimonas | rs56201224 | A | C | -0.162 | 0.036 | 8.00E-06 | 20.501 |
| genus | Ruminiclostridium5 | rs4934685 | G | C | -0.052 | 0.011 | 5.31E-06 | 20.501 |
| genus | Anaerotruncus | rs1575380 | C | T | 0.075 | 0.017 | 2.79E-06 | 20.500 |
| genus | Romboutsia | rs75200530 | G | T | -0.191 | 0.042 | 5.07E-06 | 20.500 |
| genus | Turicibacter | rs77243960 | C | T | 0.095 | 0.021 | 5.28E-06 | 20.499 |
| genus | Eggerthella | rs1784435 | C | T | 0.090 | 0.020 | 7.00E-06 | 20.499 |
| genus | Bifidobacterium | rs6430545 | T | A | 0.054 | 0.012 | 4.24E-06 | 20.499 |
| genus | FamilyXIIIAD3011group | rs3012196 | G | C | -0.055 | 0.012 | 6.25E-06 | 20.498 |
| genus | LachnospiraceaeNC2004group | rs1928659 | C | T | 0.103 | 0.023 | 6.17E-06 | 20.498 |
| genus | LachnospiraceaeNC2004group | rs12863463 | A | G | -0.156 | 0.035 | 6.04E-06 | 20.498 |
| genus | Peptococcus | rs13112950 | C | G | -0.089 | 0.020 | 6.71E-06 | 20.497 |
| genus | Streptococcus | rs77558518 | G | A | -0.104 | 0.023 | 4.71E-06 | 20.497 |
| genus | LachnospiraceaeUCG008 | rs113533446 | T | C | -0.110 | 0.024 | 6.11E-06 | 20.496 |
| genus | Eggerthella | rs12701617 | G | A | -0.090 | 0.020 | 6.75E-06 | 20.496 |
| genus | Dialister | rs11071887 | C | T | 0.066 | 0.015 | 5.91E-06 | 20.496 |
| genus | Romboutsia | rs28603357 | C | T | -0.215 | 0.047 | 8.52E-06 | 20.493 |
| genus | Prevotella9 | rs9613013 | A | G | 0.092 | 0.020 | 6.10E-06 | 20.493 |
| genus | LachnospiraceaeNK4A136group | rs160108 | C | T | 0.049 | 0.011 | 6.30E-06 | 20.493 |
| genus | LachnospiraceaeUCG010 | rs74375131 | T | C | 0.084 | 0.019 | 6.63E-06 | 20.493 |
| genus | Romboutsia | rs77702691 | G | A | -0.094 | 0.021 | 7.37E-06 | 20.493 |
| genus | Tyzzerella3 | rs75684832 | G | A | -0.123 | 0.027 | 9.23E-06 | 20.492 |
| genus | Allisonella | rs35241252 | G | T | 0.139 | 0.031 | 6.91E-06 | 20.492 |
| genus | Butyricimonas | rs12458763 | C | A | 0.122 | 0.027 | 6.37E-06 | 20.492 |
| genus | Anaerotruncus | rs7335244 | A | G | 0.075 | 0.017 | 2.88E-06 | 20.492 |
| genus | Lactococcus | rs4806168 | A | C | 0.112 | 0.025 | 5.72E-06 | 20.491 |
| genus | Dialister | rs4747450 | A | C | 0.067 | 0.015 | 5.84E-06 | 20.490 |
| genus | RuminococcaceaeUCG004 | rs4522018 | G | C | 0.082 | 0.018 | 5.52E-06 | 20.489 |
| genus | Sutterella | rs1581853 | G | A | -0.058 | 0.013 | 5.89E-06 | 20.489 |
| genus | RikenellaceaeRC9gutgroup | rs4754048 | G | T | 0.112 | 0.025 | 7.77E-06 | 20.489 |
| genus | RuminococcaceaeUCG005 | rs17309930 | C | A | 0.065 | 0.014 | 4.71E-06 | 20.489 |
| genus | Butyricicoccus | rs62478070 | G | T | 0.224 | 0.049 | 5.94E-06 | 20.488 |
| genus | LachnospiraceaeFCS020group | rs10936478 | T | A | 0.055 | 0.012 | 6.73E-06 | 20.488 |
| genus | Eggerthella | rs1784437 | C | T | 0.090 | 0.020 | 7.04E-06 | 20.488 |
| genus | Ruminiclostridium6 | rs11584262 | G | A | 0.113 | 0.025 | 8.20E-06 | 20.487 |
| genus | Blautia | rs112309597 | A | T | 0.056 | 0.012 | 6.10E-06 | 20.487 |
| genus |  | rs12402335 | T | A | 0.089 | 0.020 | 3.17E-06 | 20.486 |
| genus | Olsenella | rs4622972 | A | G | -0.105 | 0.023 | 6.51E-06 | 20.485 |
| genus | Parasutterella | rs7311004 | C | T | -0.062 | 0.014 | 5.92E-06 | 20.485 |
| genus |  | rs17132308 | T | C | -0.063 | 0.014 | 6.53E-06 | 20.485 |
| genus | Ruminiclostridium5 | rs12316367 | G | A | -0.049 | 0.011 | 6.87E-06 | 20.484 |
| genus | RikenellaceaeRC9gutgroup | rs7103176 | G | A | 0.112 | 0.025 | 8.13E-06 | 20.483 |
| genus | Anaerostipes | rs2804247 | A | G | -0.050 | 0.011 | 6.72E-06 | 20.483 |
| genus | Ruminiclostridium5 | rs2801960 | G | C | 0.052 | 0.012 | 6.21E-06 | 20.483 |
| genus | LachnospiraceaeUCG008 | rs10899032 | C | T | 0.083 | 0.018 | 6.73E-06 | 20.483 |
| genus | Ruminiclostridium9 | rs113048721 | G | C | 0.060 | 0.013 | 4.10E-06 | 20.482 |
| genus | Dialister | rs10938938 | A | G | -0.077 | 0.017 | 7.37E-06 | 20.482 |
| genus | Anaerostipes | rs7915314 | T | C | -0.050 | 0.011 | 6.65E-06 | 20.482 |
| genus | Prevotella7 | rs6733012 | G | A | 0.146 | 0.032 | 4.93E-06 | 20.482 |
| genus | Subdoligranulum | rs17122514 | G | A | -0.050 | 0.011 | 6.87E-06 | 20.481 |
| genus | Anaerofilum | rs12048664 | C | T | 0.094 | 0.021 | 6.54E-06 | 20.480 |
| genus | Erysipelatoclostridium | rs45480394 | G | T | -0.069 | 0.015 | 7.66E-06 | 20.480 |
| genus | Haemophilus | rs77529231 | T | C | -0.228 | 0.050 | 7.25E-06 | 20.480 |
| genus | Akkermansia | rs7693865 | C | T | 0.107 | 0.024 | 5.54E-06 | 20.480 |
| genus | LachnospiraceaeUCG001 | rs501463 | C | T | -0.106 | 0.023 | 5.52E-06 | 20.480 |
| genus | Roseburia | rs78753150 | C | A | 0.097 | 0.021 | 9.98E-06 | 20.479 |
| genus | Ruminococcus1 | rs11038614 | G | T | -0.051 | 0.011 | 7.06E-06 | 20.479 |
| genus | Lachnoclostridium | rs6035276 | T | C | -0.050 | 0.011 | 6.17E-06 | 20.479 |
| genus | LachnospiraceaeNC2004group | rs12855868 | C | T | -0.156 | 0.035 | 6.56E-06 | 20.478 |
| genus | Marvinbryantia | rs3125832 | C | A | 0.068 | 0.015 | 5.03E-06 | 20.477 |
| genus | Senegalimassilia | rs17350373 | G | A | 0.077 | 0.017 | 7.95E-06 | 20.476 |
| genus | Anaerostipes | rs2769413 | C | A | -0.050 | 0.011 | 6.73E-06 | 20.475 |
| genus | Anaerofilum | rs1552229 | G | A | -0.093 | 0.021 | 4.72E-06 | 20.475 |
| genus | RuminococcaceaeNK4A214group | rs6045163 | C | T | -0.104 | 0.023 | 9.69E-06 | 20.474 |
| genus | CandidatusSoleaferrea | rs6494306 | G | A | -0.097 | 0.021 | 5.80E-06 | 20.474 |
| genus | Anaerostipes | rs2249799 | G | A | -0.050 | 0.011 | 7.03E-06 | 20.472 |
| genus | Streptococcus | rs6949835 | C | T | -0.065 | 0.014 | 5.88E-06 | 20.472 |
| genus | Bifidobacterium | rs56230617 | G | A | 0.071 | 0.016 | 3.68E-06 | 20.471 |
| genus | RuminococcaceaeUCG014 | rs456678 | A | C | -0.057 | 0.013 | 7.90E-06 | 20.471 |
| genus | Oxalobacter | rs13117955 | G | T | 0.193 | 0.043 | 7.06E-06 | 20.471 |
| genus | Peptococcus | rs9299350 | A | G | 0.087 | 0.019 | 7.11E-06 | 20.471 |
| genus | Sellimonas | rs74947509 | T | C | -0.161 | 0.036 | 8.30E-06 | 20.470 |
| genus |  | rs78828165 | C | T | -0.091 | 0.020 | 7.96E-06 | 20.470 |
| genus | Lachnospira | rs12467938 | G | A | 0.050 | 0.011 | 6.43E-06 | 20.469 |
| genus | Anaerotruncus | rs2704155 | A | T | -0.106 | 0.023 | 5.42E-06 | 20.468 |
| genus | Anaerofilum | rs2054677 | C | T | -0.093 | 0.021 | 4.44E-06 | 20.468 |
| genus | RuminococcaceaeUCG003 | rs2523124 | C | T | -0.055 | 0.012 | 5.78E-06 | 20.468 |
| genus | LachnospiraceaeUCG001 | rs897 | C | T | -0.065 | 0.014 | 8.45E-06 | 20.467 |
| genus | Parasutterella | rs77329569 | T | C | 0.226 | 0.050 | 7.23E-06 | 20.467 |
| genus | Subdoligranulum | rs16962433 | T | A | 0.086 | 0.019 | 7.65E-06 | 20.466 |
| genus | Peptococcus | rs12640791 | A | G | -0.089 | 0.020 | 6.81E-06 | 20.466 |
| genus | Oscillospira | rs2211563 | A | C | 0.082 | 0.018 | 9.12E-06 | 20.465 |
| genus | Eisenbergiella | rs969736 | C | A | 0.102 | 0.022 | 6.13E-06 | 20.465 |
| genus | Butyrivibrio | rs12937276 | C | T | 0.117 | 0.026 | 5.31E-06 | 20.464 |
| genus | Blautia | rs3005511 | G | A | 0.050 | 0.011 | 6.19E-06 | 20.464 |
| genus | Turicibacter | rs12602256 | C | T | 0.098 | 0.022 | 5.58E-06 | 20.464 |
| genus | Subdoligranulum | rs17122484 | C | T | -0.050 | 0.011 | 7.37E-06 | 20.463 |
| genus | LachnospiraceaeNK4A136group | rs943881 | T | C | -0.052 | 0.012 | 9.66E-06 | 20.463 |
| genus | Roseburia | rs11169462 | T | C | 0.049 | 0.011 | 7.51E-06 | 20.462 |
| genus | Sellimonas | rs72553859 | C | G | -0.150 | 0.033 | 5.38E-06 | 20.461 |
| genus | Holdemanella | rs1830029 | G | C | -0.095 | 0.021 | 5.35E-06 | 20.460 |
| genus | LachnospiraceaeFCS020group | rs12489401 | T | G | 0.055 | 0.012 | 6.85E-06 | 20.460 |
| genus | RuminococcaceaeUCG011 | rs10513800 | C | A | 0.128 | 0.028 | 6.71E-06 | 20.459 |
| genus | Subdoligranulum | rs9683541 | C | A | -0.052 | 0.012 | 5.06E-06 | 20.459 |
| genus | RuminococcaceaeUCG013 | rs10219135 | C | T | 0.060 | 0.013 | 7.32E-06 | 20.458 |
| genus | Prevotella9 | rs7237249 | T | C | -0.082 | 0.018 | 8.93E-06 | 20.458 |
| genus | Prevotella9 | rs146399833 | A | G | -0.087 | 0.019 | 5.39E-06 | 20.458 |
| genus | Paraprevotella | rs2923900 | C | T | 0.103 | 0.023 | 8.89E-06 | 20.458 |
| genus | Barnesiella | rs28418786 | G | C | -0.079 | 0.017 | 6.48E-06 | 20.457 |
| genus | Oscillibacter | rs73205330 | T | A | -0.082 | 0.018 | 6.29E-06 | 20.456 |
| genus |  | rs13188803 | A | T | 0.071 | 0.016 | 7.28E-06 | 20.455 |
| genus | Hungatella | rs36087611 | G | A | -0.151 | 0.033 | 9.90E-06 | 20.455 |
| genus | Oscillibacter | rs9295777 | T | G | -0.069 | 0.015 | 6.74E-06 | 20.454 |
| genus | Lactococcus | rs7259929 | C | A | 0.112 | 0.025 | 5.74E-06 | 20.454 |
| genus |  | rs2939706 | T | A | 0.088 | 0.020 | 6.30E-06 | 20.454 |
| genus | Anaerostipes | rs35875963 | A | G | -0.050 | 0.011 | 6.73E-06 | 20.454 |
| genus | Prevotella7 | rs2716642 | C | T | 0.146 | 0.032 | 4.63E-06 | 20.454 |
| genus | Lactococcus | rs3972335 | A | G | 0.111 | 0.025 | 5.60E-06 | 20.454 |
| genus | Desulfovibrio | rs4797774 | A | G | 0.213 | 0.047 | 5.64E-06 | 20.454 |
| genus | Ruminiclostridium5 | rs1929387 | A | T | 0.049 | 0.011 | 5.55E-06 | 20.454 |
| genus |  | rs61920452 | G | A | 0.106 | 0.023 | 7.77E-06 | 20.452 |
| genus | Roseburia | rs9554877 | C | T | -0.058 | 0.013 | 7.86E-06 | 20.452 |
| genus | Adlercreutzia | rs6664405 | C | T | -0.095 | 0.021 | 5.23E-06 | 20.451 |
| genus |  | rs7587617 | G | A | 0.129 | 0.029 | 3.91E-06 | 20.450 |
| genus | Eisenbergiella | rs11027643 | A | G | 0.129 | 0.028 | 5.04E-06 | 20.450 |
| genus | Sellimonas | rs55953604 | A | G | -0.161 | 0.036 | 8.27E-06 | 20.449 |
| genus | Oscillibacter | rs80144401 | C | T | -0.127 | 0.028 | 6.83E-06 | 20.449 |
| genus | Intestinibacter | rs488340 | C | T | -0.132 | 0.029 | 7.46E-06 | 20.449 |
| genus | Tyzzerella3 | rs112730207 | A | C | -0.216 | 0.048 | 6.88E-06 | 20.449 |
| genus | RuminococcaceaeUCG013 | rs12336782 | C | T | -0.086 | 0.019 | 8.60E-06 | 20.448 |
| genus | Peptococcus | rs9401968 | C | T | 0.129 | 0.029 | 2.35E-06 | 20.448 |
| genus | Escherichia | rs62043827 | C | T | 0.101 | 0.022 | 7.81E-06 | 20.447 |
| genus | Ruminococcus1 | rs6105066 | C | T | -0.061 | 0.013 | 5.06E-06 | 20.447 |
| genus | ChristensenellaceaeR | rs10461257 | G | A | -0.055 | 0.012 | 6.51E-06 | 20.446 |
| genus | Victivallis | rs1407533 | T | G | 0.111 | 0.025 | 6.87E-06 | 20.446 |
| genus | Collinsella | rs10890671 | C | T | -0.054 | 0.012 | 6.52E-06 | 20.446 |
| genus |  | rs2175593 | G | A | 0.049 | 0.011 | 6.33E-06 | 20.445 |
| genus | LachnospiraceaeUCG008 | rs72900057 | G | T | -0.107 | 0.024 | 6.82E-06 | 20.445 |
| genus | LachnospiraceaeUCG008 | rs7931824 | G | A | -0.116 | 0.026 | 7.11E-06 | 20.443 |
| genus | RuminococcaceaeUCG003 | rs3795750 | G | A | -0.056 | 0.012 | 5.59E-06 | 20.443 |
| genus | RuminococcaceaeUCG004 | rs3800178 | T | C | -0.080 | 0.018 | 7.03E-06 | 20.442 |
| genus | Lactococcus | rs10424088 | T | C | 0.111 | 0.025 | 5.65E-06 | 20.441 |
| genus |  | rs7523423 | C | T | 0.049 | 0.011 | 6.34E-06 | 20.441 |
| genus | Lactococcus | rs10425616 | T | C | 0.112 | 0.025 | 5.82E-06 | 20.440 |
| genus | RuminococcaceaeNK4A214group | rs2121404 | A | G | -0.052 | 0.012 | 6.80E-06 | 20.440 |
| genus |  | rs59596655 | G | T | 0.147 | 0.033 | 7.00E-06 | 20.440 |
| genus | Akkermansia | rs4497215 | C | T | 0.064 | 0.014 | 6.41E-06 | 20.439 |
| genus |  | rs6676699 | T | G | -0.089 | 0.020 | 6.38E-06 | 20.439 |
| genus | ErysipelotrichaceaeUCG003 | rs4758231 | T | G | -0.055 | 0.012 | 6.55E-06 | 20.438 |
| genus |  | rs4717831 | T | A | 0.079 | 0.017 | 9.18E-06 | 20.437 |
| genus | Alistipes | rs10950081 | C | T | -0.049 | 0.011 | 6.43E-06 | 20.437 |
| genus | Roseburia | rs2935503 | T | C | 0.048 | 0.011 | 6.77E-06 | 20.436 |
| genus | Anaerostipes | rs2025912 | G | T | -0.050 | 0.011 | 6.86E-06 | 20.436 |
| genus | LachnospiraceaeNK4A136group | rs12611395 | G | A | -0.090 | 0.020 | 5.83E-06 | 20.435 |
| genus | Oxalobacter | rs2426956 | A | C | -0.097 | 0.022 | 7.07E-06 | 20.435 |
| genus | Peptococcus | rs17795775 | C | T | 0.108 | 0.024 | 6.73E-06 | 20.434 |
| genus |  | rs2973297 | G | A | 0.088 | 0.020 | 6.46E-06 | 20.433 |
| genus | Streptococcus | rs2952251 | G | A | -0.058 | 0.013 | 5.88E-06 | 20.432 |
| genus | Tyzzerella3 | rs1232220 | T | G | -0.144 | 0.032 | 7.91E-06 | 20.432 |
| genus | Romboutsia | rs10261799 | C | A | 0.055 | 0.012 | 6.77E-06 | 20.432 |
| genus | LachnospiraceaeNC2004group | rs6673202 | G | T | 0.111 | 0.025 | 7.40E-06 | 20.432 |
| genus | Peptococcus | rs62424011 | C | T | 0.132 | 0.029 | 2.56E-06 | 20.432 |
| genus | Intestinimonas | rs66542626 | G | A | 0.060 | 0.013 | 5.52E-06 | 20.432 |
| genus | Hungatella | rs35931379 | G | A | -0.153 | 0.034 | 7.40E-06 | 20.432 |
| genus | Ruminiclostridium6 | rs2548459 | T | C | 0.055 | 0.012 | 6.40E-06 | 20.432 |
| genus | Howardella | rs13241441 | G | T | -0.182 | 0.040 | 4.13E-06 | 20.431 |
| genus | Oxalobacter | rs6445050 | A | G | 0.104 | 0.023 | 6.43E-06 | 20.430 |
| genus | Adlercreutzia | rs2717139 | A | G | -0.114 | 0.025 | 5.25E-06 | 20.430 |
| genus | Oscillospira | rs35749994 | C | A | 0.082 | 0.018 | 8.69E-06 | 20.430 |
| genus | Lactococcus | rs10408170 | G | A | 0.111 | 0.025 | 5.65E-06 | 20.429 |
| genus | Olsenella | rs34694585 | T | C | -0.105 | 0.023 | 6.70E-06 | 20.428 |
| genus | Coprococcus2 | rs13110934 | G | A | -0.063 | 0.014 | 5.28E-06 | 20.427 |
| genus | Adlercreutzia | rs36008107 | A | C | -0.128 | 0.028 | 8.11E-06 | 20.427 |
| genus | LachnospiraceaeUCG008 | rs10793102 | G | T | 0.096 | 0.021 | 7.83E-06 | 20.426 |
| genus | FamilyXIIIUCG001 | rs11753849 | T | C | -0.061 | 0.013 | 4.88E-06 | 20.425 |
| genus | Marvinbryantia | rs7202978 | C | T | -0.060 | 0.013 | 6.18E-06 | 20.425 |
| genus | Bifidobacterium | rs12986776 | G | C | 0.055 | 0.012 | 8.19E-06 | 20.424 |
| genus | Ruminiclostridium6 | rs10495466 | G | A | 0.110 | 0.024 | 5.83E-06 | 20.424 |
| genus | LachnospiraceaeUCG001 | rs12131224 | T | C | 0.117 | 0.026 | 7.40E-06 | 20.424 |
| genus |  | rs9831905 | C | T | -0.106 | 0.023 | 6.41E-06 | 20.424 |
| genus | Turicibacter | rs147551457 | A | G | 0.098 | 0.022 | 5.99E-06 | 20.423 |
| genus | Coprococcus1 | rs9342887 | T | G | -0.049 | 0.011 | 6.58E-06 | 20.422 |
| genus | RuminococcaceaeUCG014 | rs425758 | T | C | -0.057 | 0.013 | 8.73E-06 | 20.422 |
| genus | LachnospiraceaeNK4A136group | rs159898 | A | G | 0.049 | 0.011 | 6.45E-06 | 20.421 |
| genus |  | rs7857522 | C | T | 0.081 | 0.018 | 7.76E-06 | 20.421 |
| genus | Anaerostipes | rs10885746 | A | C | -0.050 | 0.011 | 7.28E-06 | 20.421 |
| genus | Anaerostipes | rs10787584 | C | T | -0.050 | 0.011 | 6.85E-06 | 20.421 |
| genus | Butyrivibrio | rs1007475 | T | G | 0.118 | 0.026 | 7.92E-06 | 20.420 |
| genus | RuminococcaceaeUCG003 | rs4642592 | T | G | -0.060 | 0.013 | 7.21E-06 | 20.420 |
| genus | Oxalobacter | rs111966731 | C | T | 0.213 | 0.047 | 7.30E-06 | 20.419 |
| genus | LachnospiraceaeFCS020group | rs3999074 | T | G | -0.055 | 0.012 | 6.55E-06 | 20.418 |
| genus | Senegalimassilia | rs80175780 | C | T | 0.078 | 0.017 | 6.29E-06 | 20.418 |
| genus | Clostridiumsensustricto1 | rs6934446 | G | A | -0.076 | 0.017 | 3.21E-06 | 20.417 |
| genus | Anaerostipes | rs11197336 | T | C | -0.050 | 0.011 | 6.86E-06 | 20.417 |
| genus | Turicibacter | rs12601652 | G | A | 0.098 | 0.022 | 5.74E-06 | 20.417 |
| genus | Ruminococcus1 | rs4756008 | A | C | -0.052 | 0.012 | 7.13E-06 | 20.417 |
| genus | Butyrivibrio | rs10070855 | G | C | 0.140 | 0.031 | 6.74E-06 | 20.416 |
| genus | LachnospiraceaeUCG001 | rs9390194 | G | A | 0.106 | 0.024 | 7.76E-06 | 20.416 |
| genus | Allisonella | rs4014782 | G | T | 0.140 | 0.031 | 6.55E-06 | 20.416 |
| genus | Subdoligranulum | rs17122499 | T | C | -0.050 | 0.011 | 7.69E-06 | 20.415 |
| genus | Sellimonas | rs67895020 | A | G | 0.114 | 0.025 | 6.74E-06 | 20.415 |
| genus | Peptococcus | rs8019363 | T | C | 0.107 | 0.024 | 5.78E-06 | 20.414 |
| genus | Sutterella | rs9292197 | T | C | -0.060 | 0.013 | 5.67E-06 | 20.413 |
| genus | Lactococcus | rs12621813 | A | G | 0.108 | 0.024 | 6.61E-06 | 20.413 |
| genus | Coprococcus3 | rs13390956 | A | G | -0.070 | 0.015 | 7.50E-06 | 20.412 |
| genus | Roseburia | rs12220334 | C | T | 0.048 | 0.011 | 6.53E-06 | 20.411 |
| genus | Peptococcus | rs8019181 | T | C | 0.107 | 0.024 | 5.78E-06 | 20.411 |
| genus | Butyrivibrio | rs7527753 | C | T | 0.169 | 0.037 | 8.19E-06 | 20.411 |
| genus | Ruminiclostridium5 | rs10848560 | C | T | -0.049 | 0.011 | 7.17E-06 | 20.411 |
| genus | LachnospiraceaeUCG001 | rs62496417 | G | T | -0.075 | 0.017 | 5.88E-06 | 20.410 |
| genus | Streptococcus | rs11110077 | C | G | -0.080 | 0.018 | 4.69E-06 | 20.410 |
| genus | Alistipes | rs62476322 | G | A | -0.049 | 0.011 | 6.74E-06 | 20.410 |
| genus | Subdoligranulum | rs8015389 | G | T | -0.050 | 0.011 | 7.49E-06 | 20.410 |
| genus |  | rs4297429 | T | C | -0.063 | 0.014 | 6.77E-06 | 20.409 |
| genus | Alistipes | rs12539972 | G | C | -0.049 | 0.011 | 6.29E-06 | 20.409 |
| genus | Dorea | rs1469588 | C | A | 0.054 | 0.012 | 8.17E-06 | 20.409 |
| genus | Flavonifractor | rs6761463 | C | G | -0.083 | 0.018 | 8.11E-06 | 20.408 |
| genus | Sellimonas | rs17657328 | T | C | 0.115 | 0.025 | 8.25E-06 | 20.408 |
| genus | Akkermansia | rs2199602 | G | T | 0.066 | 0.015 | 7.24E-06 | 20.407 |
| genus | Parasutterella | rs11718634 | C | T | -0.067 | 0.015 | 6.37E-06 | 20.407 |
| genus | LachnospiraceaeUCG010 | rs72894980 | A | C | 0.220 | 0.049 | 7.14E-06 | 20.407 |
| genus | RuminococcaceaeUCG014 | rs375561 | C | A | -0.057 | 0.013 | 8.51E-06 | 20.407 |
| genus | RuminococcaceaeUCG010 | rs73218807 | A | G | -0.166 | 0.037 | 6.43E-06 | 20.406 |
| genus | Marvinbryantia | rs9851939 | C | G | 0.062 | 0.014 | 6.09E-06 | 20.406 |
| genus | Intestinibacter | rs6062862 | G | A | 0.092 | 0.020 | 6.68E-06 | 20.406 |
| genus | Ruminiclostridium6 | rs10829821 | C | T | -0.098 | 0.022 | 3.47E-06 | 20.406 |
| genus | Intestinibacter | rs1608222 | A | G | -0.132 | 0.029 | 7.70E-06 | 20.406 |
| genus | Sellimonas | rs2606130 | C | T | -0.116 | 0.026 | 7.13E-06 | 20.406 |
| genus | Oscillibacter | rs4981910 | T | G | -0.069 | 0.015 | 6.27E-06 | 20.404 |
| genus | Butyricicoccus | rs4962426 | T | G | 0.061 | 0.014 | 7.38E-06 | 20.403 |
| genus | Eggerthella | rs2292733 | T | A | 0.095 | 0.021 | 6.35E-06 | 20.402 |
| genus | Escherichia | rs76027895 | T | C | 0.100 | 0.022 | 6.72E-06 | 20.402 |
| genus | Lactococcus | rs17168302 | A | G | 0.192 | 0.042 | 6.29E-06 | 20.402 |
| genus | Oxalobacter | rs1977135 | A | G | -0.104 | 0.023 | 7.80E-06 | 20.401 |
| genus |  | rs2012708 | G | A | 0.057 | 0.013 | 6.53E-06 | 20.401 |
| genus | Olsenella | rs2208893 | G | T | 0.134 | 0.030 | 6.66E-06 | 20.401 |
| genus | Lachnoclostridium | rs6106056 | T | C | -0.050 | 0.011 | 6.45E-06 | 20.401 |
| genus |  | rs1425962 | C | G | 0.091 | 0.020 | 7.32E-06 | 20.401 |
| genus | Marvinbryantia | rs2815663 | G | C | -0.071 | 0.016 | 7.27E-06 | 20.400 |
| genus | Oscillibacter | rs6988470 | C | T | -0.069 | 0.015 | 6.70E-06 | 20.400 |
| genus | Veillonella | rs55807413 | G | A | 0.107 | 0.024 | 5.51E-06 | 20.400 |
| genus | Oxalobacter | rs58344363 | A | C | 0.146 | 0.032 | 9.43E-06 | 20.399 |
| genus | Bifidobacterium | rs6705916 | T | C | 0.054 | 0.012 | 7.00E-06 | 20.399 |
| genus |  | rs12653463 | A | G | -0.091 | 0.020 | 5.41E-06 | 20.399 |
| genus | Roseburia | NA | G | A | -0.223 | 0.049 | 6.01E-07 | 20.398 |
| genus | LachnospiraceaeFCS020group | rs2902485 | C | T | 0.058 | 0.013 | 6.41E-06 | 20.398 |
| genus | Lactococcus | rs7992246 | C | T | 0.104 | 0.023 | 4.45E-06 | 20.398 |
| genus |  | rs9613196 | A | T | -0.239 | 0.053 | 4.99E-06 | 20.398 |
| genus |  | rs60603763 | G | C | 0.124 | 0.028 | 5.87E-06 | 20.397 |
| genus | LachnospiraceaeUCG008 | rs11025186 | G | A | -0.116 | 0.026 | 8.25E-06 | 20.397 |
| genus | LachnospiraceaeNK4A136group | rs12894973 | C | T | -0.052 | 0.012 | 9.88E-06 | 20.397 |
| genus | Howardella | rs12673422 | T | C | -0.155 | 0.034 | 7.01E-06 | 20.397 |
| genus | Paraprevotella | rs11655006 | A | G | -0.078 | 0.017 | 8.97E-06 | 20.396 |
| genus | Marvinbryantia | rs2863373 | G | T | 0.061 | 0.014 | 6.16E-06 | 20.396 |
| genus | Ruminiclostridium9 | rs9522712 | C | T | 0.070 | 0.015 | 4.66E-06 | 20.396 |
| genus | Marvinbryantia | rs4346511 | T | G | 0.061 | 0.014 | 6.16E-06 | 20.396 |
| genus | Marvinbryantia | rs2863374 | G | A | 0.061 | 0.014 | 6.16E-06 | 20.396 |
| genus | Flavonifractor | rs11642826 | C | G | 0.147 | 0.033 | 6.65E-06 | 20.395 |
| genus | Turicibacter | rs2834975 | A | T | -0.084 | 0.019 | 7.84E-06 | 20.395 |
| genus | Eggerthella | rs1784436 | A | T | 0.090 | 0.020 | 7.39E-06 | 20.395 |
| genus | RuminococcaceaeUCG003 | rs16908686 | C | T | -0.060 | 0.013 | 6.37E-06 | 20.395 |
| genus | RuminococcaceaeUCG003 | . | A | T | 0.117 | 0.026 | 7.21E-06 | 20.394 |
| genus | Oscillospira | rs11541674 | C | A | -0.078 | 0.017 | 8.71E-06 | 20.392 |
| genus | Adlercreutzia | rs6971594 | A | G | 0.139 | 0.031 | 6.65E-06 | 20.391 |
| genus | Oscillibacter | rs1007025 | A | G | 0.094 | 0.021 | 8.48E-06 | 20.390 |
| genus | Dialister | rs2435610 | C | A | 0.065 | 0.014 | 5.93E-06 | 20.389 |
| genus | RuminococcaceaeUCG005 | rs10873448 | C | T | 0.065 | 0.014 | 4.75E-06 | 20.389 |
| genus | Fusicatenibacter | rs8071840 | A | G | -0.049 | 0.011 | 6.92E-06 | 20.389 |
| genus | Roseburia | rs329182 | C | T | 0.069 | 0.015 | 5.90E-06 | 20.389 |
| genus |  | rs2418654 | T | C | -0.075 | 0.017 | 6.17E-06 | 20.388 |
| genus | Turicibacter | rs114145484 | C | G | -0.122 | 0.027 | 5.35E-06 | 20.388 |
| genus | Erysipelatoclostridium | rs72671257 | C | A | 0.085 | 0.019 | 7.57E-06 | 20.387 |
| genus |  | rs12361923 | T | C | -0.063 | 0.014 | 6.82E-06 | 20.386 |
| genus | Fusicatenibacter | rs6515626 | A | G | 0.142 | 0.031 | 7.29E-06 | 20.386 |
| genus | Coprococcus1 | rs4330511 | G | A | -0.049 | 0.011 | 6.71E-06 | 20.386 |
| genus | Eisenbergiella | rs11938607 | C | T | 0.098 | 0.022 | 8.22E-06 | 20.386 |
| genus | Allisonella | rs12554363 | G | T | 0.135 | 0.030 | 6.87E-06 | 20.385 |
| genus | Turicibacter | rs59138429 | T | G | 0.100 | 0.022 | 7.02E-06 | 20.384 |
| genus | Adlercreutzia | rs4692824 | G | A | -0.076 | 0.017 | 7.66E-06 | 20.383 |
| genus | Turicibacter | rs1040364 | C | T | -0.084 | 0.019 | 7.79E-06 | 20.383 |
| genus | Fusicatenibacter | rs16866708 | A | G | -0.070 | 0.016 | 5.23E-06 | 20.382 |
| genus | LachnospiraceaeNC2004group | rs12138162 | G | A | 0.111 | 0.025 | 7.84E-06 | 20.381 |
| genus |  | rs329894 | A | G | -0.081 | 0.018 | 8.35E-06 | 20.381 |
| genus | Butyrivibrio | rs10457240 | A | G | -0.109 | 0.024 | 7.02E-06 | 20.380 |
| genus | RuminococcaceaeUCG003 | rs4629039 | A | T | 0.055 | 0.012 | 6.54E-06 | 20.380 |
| genus | Ruminococcus1 | rs4755332 | C | T | -0.052 | 0.012 | 7.28E-06 | 20.380 |
| genus | Lactococcus | rs3960410 | A | G | 0.111 | 0.025 | 6.06E-06 | 20.380 |
| genus | Senegalimassilia | rs6706152 | G | C | 0.077 | 0.017 | 6.62E-06 | 20.380 |
| genus | Butyrivibrio | rs486484 | G | A | -0.108 | 0.024 | 6.61E-06 | 20.380 |
| genus | Oxalobacter | rs73733942 | C | T | -0.134 | 0.030 | 6.88E-06 | 20.379 |
| genus |  | rs10923018 | A | G | 0.073 | 0.016 | 6.80E-06 | 20.378 |
| genus | Paraprevotella | rs3008584 | T | C | 0.101 | 0.022 | 8.60E-06 | 20.377 |
| genus | Anaerotruncus | rs55903929 | C | G | 0.075 | 0.017 | 2.93E-06 | 20.377 |
| genus |  | rs76214837 | G | T | 0.076 | 0.017 | 4.91E-06 | 20.376 |
| genus | Anaerostipes | rs1927481 | C | T | -0.049 | 0.011 | 7.05E-06 | 20.375 |
| genus | FamilyXIIIAD3011group | rs61914199 | A | T | -0.084 | 0.019 | 5.91E-06 | 20.374 |
| genus | Subdoligranulum | rs1667315 | A | G | 0.049 | 0.011 | 6.72E-06 | 20.374 |
| genus |  | rs1778331 | T | C | 0.104 | 0.023 | 6.41E-06 | 20.374 |
| genus | RuminococcaceaeUCG003 | rs12979891 | C | T | 0.054 | 0.012 | 7.58E-06 | 20.374 |
| genus |  | rs142445524 | G | C | -0.163 | 0.036 | 6.77E-06 | 20.372 |
| genus | FamilyXIIIAD3011group | rs34930668 | G | C | -0.088 | 0.019 | 6.82E-06 | 20.372 |
| genus | LachnospiraceaeUCG004 | rs2706242 | C | G | -0.090 | 0.020 | 9.84E-06 | 20.372 |
| genus | Sellimonas | rs73073226 | T | C | -0.160 | 0.036 | 8.51E-06 | 20.371 |
| genus | Sellimonas | rs1133878 | G | A | -0.116 | 0.026 | 7.26E-06 | 20.370 |
| genus | Coprococcus1 | rs4519976 | C | T | -0.049 | 0.011 | 6.79E-06 | 20.370 |
| genus | Ruminiclostridium5 | rs4934687 | T | A | -0.052 | 0.012 | 6.31E-06 | 20.369 |
| genus | LachnospiraceaeUCG001 | rs8104225 | G | A | 0.089 | 0.020 | 8.04E-06 | 20.369 |
| genus | Subdoligranulum | rs2114677 | T | C | -0.104 | 0.023 | 2.72E-06 | 20.368 |
| genus | Phascolarctobacterium | rs1264471 | G | T | 0.074 | 0.016 | 7.47E-06 | 20.368 |
| genus | Butyricimonas | rs7093802 | G | C | 0.063 | 0.014 | 5.94E-06 | 20.368 |
| genus |  | rs4262735 | G | A | 0.065 | 0.014 | 5.29E-06 | 20.368 |
| genus | RikenellaceaeRC9gutgroup | rs11597218 | C | T | 0.114 | 0.025 | 5.63E-06 | 20.368 |
| genus |  | rs11216331 | A | G | -0.063 | 0.014 | 6.85E-06 | 20.368 |
| genus | RuminococcaceaeUCG014 | rs10512621 | A | G | -0.098 | 0.022 | 9.57E-06 | 20.368 |
| genus | RuminococcaceaeUCG003 | rs4532474 | A | G | 0.077 | 0.017 | 4.82E-06 | 20.367 |
| genus | Coprococcus1 | rs9342888 | A | T | -0.049 | 0.011 | 6.78E-06 | 20.367 |
| genus | Anaerostipes | rs11197330 | T | C | -0.049 | 0.011 | 7.05E-06 | 20.365 |
| genus |  | rs10905594 | T | C | 0.153 | 0.034 | 9.32E-06 | 20.365 |
| genus | Bacteroides | rs13207588 | G | A | -0.059 | 0.013 | 7.49E-06 | 20.365 |
| genus |  | rs6861096 | T | A | -0.091 | 0.020 | 5.69E-06 | 20.364 |
| genus | Collinsella | rs11212094 | C | T | -0.054 | 0.012 | 6.76E-06 | 20.364 |
| genus | Akkermansia | rs12045978 | G | A | 0.064 | 0.014 | 6.73E-06 | 20.363 |
| genus | Akkermansia | rs72663785 | C | G | 0.066 | 0.015 | 7.33E-06 | 20.362 |
| genus | Escherichia | rs4731451 | A | G | -0.061 | 0.014 | 7.47E-06 | 20.362 |
| genus | Oxalobacter | rs7617946 | T | C | 0.104 | 0.023 | 6.56E-06 | 20.362 |
| genus | Barnesiella | rs4387569 | A | T | -0.054 | 0.012 | 6.85E-06 | 20.362 |
| genus | Butyricimonas | rs2114713 | T | G | 0.063 | 0.014 | 6.88E-06 | 20.362 |
| genus | Oxalobacter | rs2144154 | G | A | -0.096 | 0.021 | 6.90E-06 | 20.361 |
| genus |  | rs77034621 | G | T | -0.152 | 0.034 | 6.07E-06 | 20.359 |
| genus | Oscillibacter | rs11990306 | C | G | -0.081 | 0.018 | 6.56E-06 | 20.359 |
| genus | Erysipelatoclostridium | rs66862016 | G | A | -0.106 | 0.024 | 5.89E-06 | 20.359 |
| genus | Oscillibacter | rs12417956 | G | C | 0.079 | 0.017 | 6.03E-06 | 20.358 |
| genus | Desulfovibrio | rs12031543 | C | T | -0.127 | 0.028 | 6.55E-06 | 20.357 |
| genus | Anaerostipes | rs12620003 | C | T | -0.085 | 0.019 | 6.51E-06 | 20.357 |
| genus | Lactococcus | rs12712305 | A | G | 0.108 | 0.024 | 6.97E-06 | 20.357 |
| genus | RuminococcaceaeUCG014 | rs46066 | G | C | -0.057 | 0.013 | 8.20E-06 | 20.357 |
| genus |  | rs10074000 | C | T | -0.103 | 0.023 | 7.00E-06 | 20.357 |
| genus |  | rs11717425 | C | T | -0.052 | 0.012 | 6.98E-06 | 20.356 |
| genus | Ruminiclostridium5 | rs10773989 | T | C | -0.049 | 0.011 | 6.86E-06 | 20.356 |
| genus | Anaerofilum | rs17012738 | G | T | 0.090 | 0.020 | 7.24E-06 | 20.355 |
| genus | RuminococcaceaeUCG010 | rs73218806 | C | T | -0.166 | 0.037 | 6.81E-06 | 20.355 |
| genus | Lactobacillus | rs1530559 | A | G | 0.080 | 0.018 | 4.93E-06 | 20.355 |
| genus | Allisonella | rs3747507 | G | A | 0.137 | 0.030 | 9.95E-06 | 20.355 |
| genus | Dorea | rs34459983 | C | T | -0.065 | 0.014 | 7.24E-06 | 20.354 |
| genus | Fusicatenibacter | rs9905659 | A | G | -0.062 | 0.014 | 7.31E-06 | 20.354 |
| genus | Anaerofilum | rs17507421 | A | G | 0.091 | 0.020 | 7.40E-06 | 20.354 |
| genus | Turicibacter | rs8073198 | G | T | 0.099 | 0.022 | 6.38E-06 | 20.353 |
| genus | Marvinbryantia | rs12963345 | C | G | -0.060 | 0.013 | 6.60E-06 | 20.353 |
| genus | Coprococcus2 | rs9783625 | C | A | -0.065 | 0.014 | 7.37E-06 | 20.353 |
| genus | RuminococcaceaeNK4A214group | rs669271 | A | C | -0.055 | 0.012 | 6.19E-06 | 20.353 |
| genus | Erysipelatoclostridium | rs1346565 | C | G | -0.066 | 0.015 | 6.47E-06 | 20.352 |
| genus |  | rs66960257 | T | C | 0.145 | 0.032 | 7.91E-06 | 20.352 |
| genus | Ruminiclostridium5 | rs9739162 | G | T | -0.048 | 0.011 | 6.76E-06 | 20.350 |
| genus | Phascolarctobacterium | rs1405230 | A | C | 0.064 | 0.014 | 7.04E-06 | 20.350 |
| genus | Anaerofilum | rs9299345 | C | T | -0.136 | 0.030 | 8.04E-06 | 20.349 |
| genus |  | rs139758 | T | C | -0.077 | 0.017 | 6.46E-06 | 20.348 |
| genus | Oscillospira | rs28889936 | C | A | 0.114 | 0.025 | 3.37E-06 | 20.348 |
| genus | Eggerthella | rs2509015 | G | A | 0.089 | 0.020 | 7.39E-06 | 20.348 |
| genus | Oscillibacter | rs2748935 | T | C | 0.068 | 0.015 | 6.29E-06 | 20.347 |
| genus | LachnospiraceaeFCS020group | rs7813903 | A | C | -0.055 | 0.012 | 7.50E-06 | 20.347 |
| genus | RuminococcaceaeUCG011 | rs55701287 | C | T | 0.169 | 0.037 | 7.08E-06 | 20.347 |
| genus | Oscillibacter | rs72681793 | C | G | -0.127 | 0.028 | 6.50E-06 | 20.346 |
| genus | Anaerostipes | rs11492805 | A | G | -0.050 | 0.011 | 7.57E-06 | 20.346 |
| genus | Anaerotruncus | rs8019932 | T | G | 0.053 | 0.012 | 6.01E-06 | 20.345 |
| genus | Alistipes | rs34417064 | G | A | -0.048 | 0.011 | 7.01E-06 | 20.344 |
| genus | Blautia | rs67645219 | T | C | 0.056 | 0.012 | 7.17E-06 | 20.343 |
| genus | Ruminococcus1 | rs7584151 | A | G | 0.051 | 0.011 | 5.90E-06 | 20.343 |
| genus | Ruminiclostridium6 | rs7986731 | G | C | -0.061 | 0.014 | 7.55E-06 | 20.343 |
| genus | Allisonella | rs10981784 | C | T | 0.135 | 0.030 | 7.54E-06 | 20.343 |
| genus | Oscillibacter | rs6926221 | A | G | -0.068 | 0.015 | 7.49E-06 | 20.343 |
| genus | Butyricimonas | rs11738176 | C | A | 0.065 | 0.015 | 5.43E-06 | 20.343 |
| genus | Parasutterella | rs11058430 | A | T | -0.072 | 0.016 | 7.15E-06 | 20.342 |
| genus | Lachnoclostridium | rs6035306 | G | T | -0.049 | 0.011 | 6.90E-06 | 20.341 |
| genus | Ruminiclostridium9 | rs9515627 | G | A | 0.070 | 0.015 | 5.02E-06 | 20.341 |
| genus | Prevotella9 | rs2943422 | G | A | -0.068 | 0.015 | 5.43E-06 | 20.340 |
| genus | Howardella | rs61774465 | A | C | -0.149 | 0.033 | 7.98E-06 | 20.340 |
| genus | Bifidobacterium | rs55888705 | G | A | 0.055 | 0.012 | 6.67E-06 | 20.339 |
| genus | Allisonella | rs35623544 | C | G | 0.135 | 0.030 | 7.65E-06 | 20.339 |
| genus | Odoribacter | rs492325 | T | C | 0.053 | 0.012 | 6.43E-06 | 20.338 |
| genus | RuminococcaceaeUCG014 | rs332731 | A | G | -0.057 | 0.013 | 8.90E-06 | 20.337 |
| genus | Turicibacter | rs41408545 | G | A | 0.100 | 0.022 | 5.95E-06 | 20.337 |
| genus | Bacteroides | rs7322219 | T | C | 0.052 | 0.012 | 5.83E-06 | 20.337 |
| genus | Sellimonas | rs113144620 | A | G | -0.160 | 0.036 | 9.40E-06 | 20.336 |
| genus | Sellimonas | rs919059 | G | A | 0.113 | 0.025 | 6.84E-06 | 20.336 |
| genus | Roseburia | rs11253733 | G | A | 0.048 | 0.011 | 6.84E-06 | 20.336 |
| genus | RuminococcaceaeUCG002 | rs62371738 | T | C | -0.055 | 0.012 | 8.41E-06 | 20.334 |
| genus | Bacteroides | rs2366421 | A | T | -0.053 | 0.012 | 7.65E-06 | 20.334 |
| genus | LachnospiraceaeUCG001 | rs9390195 | G | T | 0.106 | 0.024 | 8.18E-06 | 20.334 |
| genus | Methanobrevibacter | rs1334944 | C | T | 0.115 | 0.026 | 7.61E-06 | 20.329 |
| genus | Adlercreutzia | rs7706425 | G | C | -0.071 | 0.016 | 6.62E-06 | 20.329 |
| genus | LachnospiraceaeUCG001 | rs9376841 | A | G | 0.104 | 0.023 | 8.43E-06 | 20.329 |
| genus | Phascolarctobacterium | rs987268 | G | C | 0.062 | 0.014 | 6.79E-06 | 20.328 |
| genus | RuminococcaceaeUCG014 | rs420342 | T | G | -0.057 | 0.013 | 8.89E-06 | 20.328 |
| genus | Butyrivibrio | rs240991 | G | C | -0.119 | 0.026 | 6.70E-06 | 20.327 |
| genus | LachnospiraceaeUCG008 | rs11025181 | C | T | -0.087 | 0.019 | 7.11E-06 | 20.327 |
| genus | Faecalibacterium | rs79656633 | C | T | 0.146 | 0.032 | 8.14E-06 | 20.326 |
| genus | LachnospiraceaeUCG004 | rs6429189 | T | C | -0.058 | 0.013 | 8.02E-06 | 20.326 |
| genus | Dorea | rs1529992 | T | G | 0.051 | 0.011 | 7.16E-06 | 20.326 |
| genus | RuminococcaceaeUCG003 | rs4593487 | T | C | -0.060 | 0.013 | 7.22E-06 | 20.326 |
| genus |  | rs28420252 | A | G | -0.105 | 0.023 | 7.36E-06 | 20.325 |
| genus | Turicibacter | rs3734633 | A | G | -0.121 | 0.027 | 5.32E-06 | 20.324 |
| genus | Ruminococcus1 | rs7115106 | A | G | -0.050 | 0.011 | 7.74E-06 | 20.324 |
| genus | FamilyXIIIAD3011group | rs34171133 | T | G | -0.091 | 0.020 | 5.70E-06 | 20.324 |
| genus |  | rs11662417 | C | G | 0.145 | 0.032 | 7.98E-06 | 20.323 |
| genus | Lachnospira | rs5030014 | G | C | 0.051 | 0.011 | 5.96E-06 | 20.323 |
| genus | Ruminococcus1 | rs4756009 | G | C | -0.052 | 0.012 | 7.43E-06 | 20.322 |
| genus | Peptococcus | rs12110494 | C | T | 0.130 | 0.029 | 2.64E-06 | 20.322 |
| genus | Sutterella | rs10786607 | A | G | -0.068 | 0.015 | 7.94E-06 | 20.321 |
| genus |  | rs431418 | G | A | -0.095 | 0.021 | 5.54E-06 | 20.321 |
| genus | FamilyXIIIAD3011group | rs149302 | C | T | -0.065 | 0.014 | 7.48E-06 | 20.321 |
| genus | Olsenella | rs6553736 | A | G | -0.104 | 0.023 | 7.11E-06 | 20.321 |
| genus | Victivallis | rs2104209 | G | T | -0.111 | 0.025 | 7.45E-06 | 20.320 |
| genus |  | rs2097532 | T | C | 0.058 | 0.013 | 5.87E-06 | 20.319 |
| genus | RuminococcaceaeUCG003 | rs4452677 | A | G | 0.077 | 0.017 | 4.85E-06 | 20.318 |
| genus | Parasutterella | rs8028823 | A | C | 0.060 | 0.013 | 6.96E-06 | 20.318 |
| genus | RuminococcaceaeUCG004 | rs10127706 | T | C | 0.082 | 0.018 | 5.93E-06 | 20.318 |
| genus | Escherichia | rs7502686 | C | G | -0.136 | 0.030 | 5.90E-06 | 20.317 |
| genus |  | rs7827125 | T | C | 0.122 | 0.027 | 7.17E-06 | 20.317 |
| genus | Paraprevotella | rs7240324 | G | T | -0.102 | 0.023 | 5.96E-06 | 20.315 |
| genus | Peptococcus | rs34419559 | C | T | 0.182 | 0.040 | 7.08E-06 | 20.315 |
| genus |  | rs75448022 | G | A | 0.149 | 0.033 | 7.57E-06 | 20.314 |
| genus |  | rs67260757 | T | C | 0.145 | 0.032 | 7.99E-06 | 20.314 |
| genus | Butyrivibrio | rs7537 | T | A | 0.179 | 0.040 | 8.03E-06 | 20.314 |
| genus | Enterorhabdus | rs7121381 | A | G | 0.095 | 0.021 | 6.99E-06 | 20.312 |
| genus | Coprobacter | rs12908296 | G | A | 0.101 | 0.022 | 9.04E-06 | 20.311 |
| genus | Veillonella | rs11614532 | C | G | 0.074 | 0.017 | 7.13E-06 | 20.309 |
| genus | RuminococcaceaeUCG002 | rs28620610 | A | G | -0.051 | 0.011 | 6.26E-06 | 20.308 |
| genus | FamilyXIIIUCG001 | rs61809296 | G | A | -0.111 | 0.025 | 9.80E-06 | 20.308 |
| genus | Marvinbryantia | rs1320151 | T | C | 0.061 | 0.014 | 6.45E-06 | 20.308 |
| genus | LachnospiraceaeUCG008 | rs10766573 | A | C | -0.109 | 0.024 | 5.84E-06 | 20.307 |
| genus | Lachnoclostridium | rs6035269 | T | C | -0.050 | 0.011 | 7.01E-06 | 20.307 |
| genus |  | rs12989336 | A | G | -0.085 | 0.019 | 7.12E-06 | 20.305 |
| genus | Intestinibacter | rs11109097 | T | C | 0.062 | 0.014 | 5.49E-06 | 20.305 |
| genus | Victivallis | rs911666 | C | T | -0.119 | 0.026 | 7.65E-06 | 20.305 |
| genus | Lactobacillus | rs57326358 | A | G | -0.122 | 0.027 | 8.24E-06 | 20.304 |
| genus | Anaerostipes | rs11197345 | A | C | -0.050 | 0.011 | 7.80E-06 | 20.304 |
| genus | Coprococcus2 | rs35890118 | G | A | -0.067 | 0.015 | 8.26E-06 | 20.304 |
| genus | Butyrivibrio | rs1989574 | C | T | -0.108 | 0.024 | 7.42E-06 | 20.304 |
| genus | Subdoligranulum | rs2572356 | A | G | -0.053 | 0.012 | 6.09E-06 | 20.303 |
| genus | Streptococcus | rs11006989 | A | G | 0.097 | 0.021 | 6.75E-06 | 20.303 |
| genus | Paraprevotella | rs1928132 | G | T | -0.091 | 0.020 | 5.75E-06 | 20.302 |
| genus | Hungatella | rs17092615 | A | G | 0.152 | 0.034 | 7.38E-06 | 20.302 |
| genus | Anaerotruncus | rs12528051 | T | A | 0.050 | 0.011 | 6.10E-06 | 20.302 |
| genus | Oxalobacter | rs2207331 | T | C | -0.096 | 0.021 | 7.12E-06 | 20.301 |
| genus | Tyzzerella3 | rs10414675 | A | G | 0.096 | 0.021 | 8.29E-06 | 20.301 |
| genus |  | rs62051905 | G | A | 0.208 | 0.046 | 6.32E-06 | 20.301 |
| genus | Allisonella | rs16919671 | T | C | 0.135 | 0.030 | 7.36E-06 | 20.301 |
| genus | Enterorhabdus | rs10098492 | C | T | 0.132 | 0.029 | 6.41E-06 | 20.300 |
| genus | Oscillibacter | rs137917150 | A | T | -0.175 | 0.039 | 4.62E-06 | 20.300 |
| genus | Roseburia | rs2943024 | C | G | 0.048 | 0.011 | 7.27E-06 | 20.299 |
| genus | RuminococcaceaeUCG010 | rs886017 | G | A | -0.056 | 0.013 | 6.65E-06 | 20.299 |
| genus | Ruminococcus2 | rs4663243 | G | A | 0.064 | 0.014 | 6.98E-06 | 20.298 |
| genus | Collinsella | rs4753793 | T | A | -0.054 | 0.012 | 7.03E-06 | 20.298 |
| genus |  | rs4792526 | T | C | 0.048 | 0.011 | 6.21E-06 | 20.297 |
| genus | RuminococcaceaeUCG014 | rs62478832 | A | T | -0.058 | 0.013 | 6.04E-06 | 20.296 |
| genus | Barnesiella | rs72684847 | C | T | -0.114 | 0.025 | 6.76E-06 | 20.295 |
| genus | Parasutterella | rs1425386 | C | G | 0.060 | 0.013 | 6.89E-06 | 20.293 |
| genus | Anaerostipes | rs11197343 | G | A | -0.049 | 0.011 | 7.31E-06 | 20.292 |
| genus | Anaerostipes | rs11197342 | C | T | -0.049 | 0.011 | 7.31E-06 | 20.292 |
| genus | RuminococcaceaeUCG003 | rs10504733 | A | G | -0.060 | 0.013 | 6.84E-06 | 20.292 |
| genus | Streptococcus | rs2197262 | T | A | -0.050 | 0.011 | 7.41E-06 | 20.291 |
| genus | Marvinbryantia | rs9854860 | T | G | 0.061 | 0.014 | 6.56E-06 | 20.291 |
| genus | Odoribacter | rs28417404 | G | A | -0.073 | 0.016 | 3.68E-06 | 20.290 |
| genus | CandidatusSoleaferrea | rs6881988 | C | G | -0.082 | 0.018 | 9.23E-06 | 20.290 |
| genus |  | rs6895524 | G | A | -0.082 | 0.018 | 7.76E-06 | 20.290 |
| genus | Marvinbryantia | rs5019680 | G | T | 0.062 | 0.014 | 6.76E-06 | 20.288 |
| genus | Alistipes | rs2836934 | A | C | -0.048 | 0.011 | 6.69E-06 | 20.288 |
| genus | Ruminiclostridium5 | rs2833828 | A | G | 0.049 | 0.011 | 6.82E-06 | 20.288 |
| genus | LachnospiraceaeFCS020group | rs17522496 | C | T | -0.078 | 0.017 | 9.78E-06 | 20.288 |
| genus | Adlercreutzia | rs1881618 | T | C | -0.076 | 0.017 | 7.97E-06 | 20.288 |
| genus | Parasutterella | rs62278065 | G | A | 0.227 | 0.050 | 8.04E-06 | 20.287 |
| genus |  | rs11128172 | A | G | -0.081 | 0.018 | 7.03E-06 | 20.287 |
| genus | Anaerofilum | rs10754535 | G | C | 0.094 | 0.021 | 7.23E-06 | 20.287 |
| genus | Adlercreutzia | rs10279572 | G | C | 0.139 | 0.031 | 6.98E-06 | 20.286 |
| genus | RuminococcaceaeUCG014 | rs332730 | G | A | -0.057 | 0.013 | 9.14E-06 | 20.285 |
| genus |  | rs12129908 | A | C | 0.089 | 0.020 | 5.80E-06 | 20.285 |
| genus | Intestinimonas | rs994794 | C | G | -0.142 | 0.032 | 7.31E-06 | 20.285 |
| genus | Anaerostipes | rs11197357 | C | A | -0.050 | 0.011 | 7.52E-06 | 20.285 |
| genus | Clostridiumsensustricto1 | rs876740 | C | T | 0.055 | 0.012 | 5.83E-06 | 20.285 |
| genus | LachnospiraceaeUCG008 | rs75356640 | A | G | 0.137 | 0.030 | 9.83E-06 | 20.284 |
| genus | Paraprevotella | rs4398141 | G | A | -0.078 | 0.017 | 9.35E-06 | 20.284 |
| genus | LachnospiraceaeUCG004 | rs35816772 | G | C | -0.056 | 0.012 | 9.17E-06 | 20.284 |
| genus | Allisonella | rs7041222 | A | C | 0.135 | 0.030 | 7.90E-06 | 20.283 |
| genus | Oscillibacter | rs7156445 | G | A | -0.069 | 0.015 | 6.72E-06 | 20.283 |
| genus | Anaerofilum | rs12466058 | T | C | 0.091 | 0.020 | 7.60E-06 | 20.282 |
| genus | Parasutterella | rs62273908 | A | G | 0.226 | 0.050 | 8.15E-06 | 20.282 |
| genus | Parasutterella | rs62273909 | T | C | 0.226 | 0.050 | 8.15E-06 | 20.282 |
| genus | Parasutterella | rs79164092 | G | A | 0.226 | 0.050 | 7.98E-06 | 20.281 |
| genus |  | rs6591893 | A | G | 0.108 | 0.024 | 7.34E-06 | 20.281 |
| genus | Parasutterella | rs7670098 | C | T | 0.060 | 0.013 | 7.18E-06 | 20.280 |
| genus | LachnospiraceaeFCS020group | rs12862721 | C | A | -0.059 | 0.013 | 6.97E-06 | 20.279 |
| genus | Eisenbergiella | rs12257723 | C | A | -0.095 | 0.021 | 8.85E-06 | 20.279 |
| genus | Lactobacillus | rs75127669 | A | C | 0.140 | 0.031 | 6.83E-06 | 20.278 |
| genus |  | rs62449702 | G | A | 0.076 | 0.017 | 5.17E-06 | 20.278 |
| genus | Hungatella | rs7851088 | A | C | -0.129 | 0.029 | 4.22E-06 | 20.278 |
| genus | RuminococcaceaeUCG014 | rs457452 | G | A | -0.057 | 0.013 | 9.09E-06 | 20.277 |
| genus | Prevotella9 | rs13385890 | C | G | -0.133 | 0.030 | 8.10E-06 | 20.276 |
| genus | Romboutsia | rs7930091 | A | G | -0.071 | 0.016 | 6.75E-06 | 20.276 |
| genus | LachnospiraceaeNC2004group | rs12137822 | G | T | 0.111 | 0.025 | 8.55E-06 | 20.275 |
| genus |  | rs139759 | G | A | -0.077 | 0.017 | 6.63E-06 | 20.275 |
| genus | RuminococcaceaeUCG003 | rs72674078 | C | T | -0.060 | 0.013 | 6.90E-06 | 20.274 |
| genus | Subdoligranulum | rs936135 | A | G | -0.052 | 0.012 | 5.54E-06 | 20.274 |
| genus | Peptococcus | rs317070 | A | C | -0.099 | 0.022 | 8.57E-06 | 20.274 |
| genus | Anaerostipes | rs10885753 | T | G | -0.049 | 0.011 | 7.40E-06 | 20.274 |
| genus |  | rs7235290 | G | A | 0.147 | 0.033 | 7.92E-06 | 20.273 |
| genus | Fusicatenibacter | rs60254196 | G | A | -0.049 | 0.011 | 5.47E-06 | 20.273 |
| genus | Anaerotruncus | rs12431415 | G | A | 0.054 | 0.012 | 9.02E-06 | 20.273 |
| genus | LachnospiraceaeFCS020group | rs34515898 | G | A | -0.192 | 0.043 | 4.04E-06 | 20.273 |
| genus | Eggerthella | rs2459480 | G | T | 0.089 | 0.020 | 7.66E-06 | 20.272 |
| genus | LachnospiraceaeUCG010 | rs4576377 | C | A | -0.057 | 0.013 | 7.63E-06 | 20.272 |
| genus | Erysipelatoclostridium | rs9590927 | A | G | -0.065 | 0.014 | 6.39E-06 | 20.272 |
| genus |  | rs4791587 | C | T | 0.048 | 0.011 | 5.70E-06 | 20.271 |
| genus | Oscillibacter | rs62206502 | A | C | -0.068 | 0.015 | 6.60E-06 | 20.271 |
| genus | Prevotella9 | rs2672264 | G | A | -0.068 | 0.015 | 5.91E-06 | 20.271 |
| genus | Anaerofilum | rs7555643 | G | A | 0.094 | 0.021 | 7.27E-06 | 20.270 |
| genus | Ruminiclostridium9 | rs7622451 | C | T | -0.051 | 0.011 | 7.91E-06 | 20.269 |
| genus | Anaerotruncus | rs7675045 | T | A | -0.050 | 0.011 | 6.92E-06 | 20.269 |
| genus | LachnospiraceaeNC2004group | rs35914870 | T | C | 0.111 | 0.025 | 9.06E-06 | 20.269 |
| genus | LachnospiraceaeUCG001 | rs77867174 | G | C | 0.106 | 0.024 | 8.43E-06 | 20.269 |
| genus | Ruminococcus2 | rs58681734 | G | A | 0.072 | 0.016 | 4.18E-06 | 20.269 |
| genus | Lactobacillus | rs11619768 | G | A | -0.100 | 0.022 | 9.78E-06 | 20.268 |
| genus | RuminococcaceaeUCG010 | rs7441445 | T | C | -0.057 | 0.013 | 6.80E-06 | 20.267 |
| genus | RuminococcaceaeUCG004 | rs138868222 | T | C | 0.124 | 0.028 | 6.88E-06 | 20.267 |
| genus | Subdoligranulum | rs6555308 | T | C | -0.070 | 0.016 | 9.91E-06 | 20.266 |
| genus | Bilophila | rs1676510 | G | A | -0.071 | 0.016 | 7.86E-06 | 20.265 |
| genus |  | rs36120874 | A | G | 0.071 | 0.016 | 8.42E-06 | 20.263 |
| genus | Anaerostipes | rs78735375 | C | A | -0.137 | 0.031 | 5.33E-06 | 20.262 |
| genus | Butyrivibrio | rs8074123 | G | T | 0.116 | 0.026 | 5.65E-06 | 20.261 |
| genus | Slackia | rs10409783 | G | A | 0.095 | 0.021 | 7.70E-06 | 20.261 |
| genus | LachnospiraceaeNK4A136group | rs59805249 | C | T | 0.094 | 0.021 | 9.45E-06 | 20.260 |
| genus | Allisonella | rs1564050 | A | G | 0.135 | 0.030 | 7.45E-06 | 20.259 |
| genus | RuminococcaceaeUCG014 | rs77627087 | G | C | 0.068 | 0.015 | 7.43E-06 | 20.259 |
| genus | RikenellaceaeRC9gutgroup | rs7945830 | G | A | 0.111 | 0.025 | 8.65E-06 | 20.259 |
| genus | Clostridiumsensustricto1 | rs12341505 | A | G | 0.081 | 0.018 | 4.82E-06 | 20.259 |
| genus | Alistipes | rs2150411 | G | T | -0.048 | 0.011 | 6.84E-06 | 20.258 |
| genus | DefluviitaleaceaeUCG011 | rs2892880 | A | G | 0.082 | 0.018 | 6.83E-06 | 20.258 |
| genus | Sellimonas | rs2570251 | T | C | 0.113 | 0.025 | 7.17E-06 | 20.258 |
| genus | RuminococcaceaeUCG014 | rs332064 | A | G | -0.057 | 0.013 | 8.69E-06 | 20.258 |
| genus | Parasutterella | rs72981403 | C | G | 0.087 | 0.019 | 7.07E-06 | 20.258 |
| genus | Anaerostipes | rs11197322 | C | T | -0.049 | 0.011 | 7.50E-06 | 20.257 |
| genus | Anaerostipes | rs34596478 | A | G | -0.049 | 0.011 | 7.44E-06 | 20.257 |
| genus |  | rs289409 | T | C | -0.063 | 0.014 | 6.58E-06 | 20.256 |
| genus | Eggerthella | rs1784432 | C | G | 0.089 | 0.020 | 7.95E-06 | 20.256 |
| genus | Subdoligranulum | rs2114678 | A | G | -0.104 | 0.023 | 3.03E-06 | 20.255 |
| genus | Tyzzerella3 | rs191093 | A | G | 0.159 | 0.035 | 6.76E-06 | 20.255 |
| genus | Peptococcus | rs401957 | T | G | -0.089 | 0.020 | 7.56E-06 | 20.255 |
| genus |  | rs7858063 | G | C | 0.081 | 0.018 | 8.45E-06 | 20.254 |
| genus | RuminococcaceaeUCG005 | rs1956453 | A | G | 0.065 | 0.014 | 4.76E-06 | 20.254 |
| genus | LachnospiraceaeNK4A136group | rs4955932 | C | T | -0.049 | 0.011 | 7.05E-06 | 20.253 |
| genus |  | rs13025464 | C | T | -0.074 | 0.016 | 6.97E-06 | 20.252 |
| genus | Victivallis | rs7374366 | A | G | -0.182 | 0.041 | 7.23E-06 | 20.251 |
| genus | Sellimonas | rs487637 | A | C | 0.127 | 0.028 | 7.96E-06 | 20.251 |
| genus | Ruminiclostridium9 | rs1609523 | G | A | -0.050 | 0.011 | 7.04E-06 | 20.251 |
| genus | Methanobrevibacter | rs6776814 | C | T | -0.189 | 0.042 | 8.05E-06 | 20.250 |
| genus | Subdoligranulum | rs76664262 | A | T | 0.083 | 0.019 | 4.87E-06 | 20.249 |
| genus | RuminococcaceaeUCG014 | rs455616 | G | C | -0.057 | 0.013 | 8.87E-06 | 20.247 |
| genus | Parasutterella | rs225533 | A | G | -0.064 | 0.014 | 6.74E-06 | 20.246 |
| genus | Lachnoclostridium | rs4814842 | A | T | -0.049 | 0.011 | 6.88E-06 | 20.246 |
| genus | Ruminiclostridium9 | rs6780477 | G | T | -0.051 | 0.011 | 8.35E-06 | 20.246 |
| genus | Parasutterella | rs7956291 | C | T | -0.073 | 0.016 | 7.68E-06 | 20.246 |
| genus | Dorea | rs4831351 | C | T | -0.095 | 0.021 | 7.07E-06 | 20.246 |
| genus | RuminococcaceaeUCG002 | rs4806679 | C | T | -0.053 | 0.012 | 5.59E-06 | 20.244 |
| genus | Prevotella9 | rs1915406 | C | T | -0.069 | 0.015 | 6.03E-06 | 20.244 |
| genus | Eisenbergiella | rs11027644 | C | T | 0.128 | 0.028 | 5.29E-06 | 20.244 |
| genus | Coprococcus2 | rs9426473 | G | A | 0.073 | 0.016 | 6.31E-06 | 20.244 |
| genus | Lachnoclostridium | rs7007181 | C | T | -0.051 | 0.011 | 7.18E-06 | 20.243 |
| genus | LachnospiraceaeFCS020group | rs113859143 | C | G | -0.109 | 0.024 | 2.55E-06 | 20.243 |
| genus | Flavonifractor | rs798674 | C | G | 0.064 | 0.014 | 7.02E-06 | 20.243 |
| genus |  | rs6923695 | G | T | 0.103 | 0.023 | 4.87E-06 | 20.243 |
| genus | Anaerotruncus | rs56098078 | A | C | 0.074 | 0.017 | 3.22E-06 | 20.241 |
| genus | Bilophila | rs2728491 | T | G | -0.063 | 0.014 | 6.33E-06 | 20.241 |
| genus | Sutterella | rs27172 | C | T | -0.057 | 0.013 | 7.80E-06 | 20.239 |
| genus | Parasutterella | rs1357416 | G | A | -0.072 | 0.016 | 7.82E-06 | 20.239 |
| genus | Desulfovibrio | rs1119400 | T | C | -0.066 | 0.015 | 6.91E-06 | 20.239 |
| genus | Anaerostipes | rs1590733 | T | C | -0.049 | 0.011 | 7.56E-06 | 20.237 |
| genus | RuminococcaceaeUCG014 | rs332065 | G | A | -0.057 | 0.013 | 8.56E-06 | 20.237 |
| genus | Anaerostipes | rs2769412 | A | G | -0.049 | 0.011 | 7.61E-06 | 20.236 |
| genus | Gordonibacter | rs117347059 | C | G | -0.128 | 0.029 | 9.17E-06 | 20.236 |
| genus |  | rs61302912 | C | T | -0.052 | 0.012 | 6.47E-06 | 20.236 |
| genus | LachnospiraceaeUCG010 | rs28513422 | G | A | 0.084 | 0.019 | 9.41E-06 | 20.235 |
| genus | Holdemanella | rs62113417 | T | A | -0.104 | 0.023 | 6.84E-06 | 20.235 |
| genus | Eisenbergiella | rs11651545 | C | T | 0.107 | 0.024 | 9.06E-06 | 20.235 |
| genus | Bifidobacterium | rs56302516 | C | T | 0.061 | 0.014 | 6.40E-06 | 20.234 |
| genus | FamilyXIIIAD3011group | rs11043753 | T | G | -0.078 | 0.017 | 7.95E-06 | 20.233 |
| genus | FamilyXIIIUCG001 | rs62414803 | A | G | -0.060 | 0.013 | 7.09E-06 | 20.233 |
| genus | Alistipes | rs4718714 | A | G | -0.049 | 0.011 | 7.35E-06 | 20.232 |
| genus | LachnospiraceaeFCS020group | rs9851162 | A | T | 0.058 | 0.013 | 7.02E-06 | 20.231 |
| genus | LachnospiraceaeUCG004 | rs233486 | G | A | -0.080 | 0.018 | 6.28E-06 | 20.231 |
| genus | Coprobacter | rs5011652 | C | G | 0.090 | 0.020 | 5.51E-06 | 20.230 |
| genus | Gordonibacter | rs13412653 | C | A | 0.108 | 0.024 | 8.61E-06 | 20.229 |
| genus | LachnospiraceaeUCG001 | rs10815582 | T | C | -0.065 | 0.014 | 6.61E-06 | 20.228 |
| genus | Butyricimonas | rs929839 | G | T | 0.112 | 0.025 | 6.16E-06 | 20.228 |
| genus | Bilophila | rs1763359 | T | C | -0.071 | 0.016 | 7.98E-06 | 20.228 |
| genus | Prevotella7 | rs2918132 | T | C | -0.115 | 0.025 | 6.42E-06 | 20.228 |
| genus |  | rs76813986 | T | C | -0.090 | 0.020 | 8.79E-06 | 20.228 |
| genus | Alloprevotella | rs58212166 | G | A | -0.162 | 0.036 | 7.94E-06 | 20.227 |
| genus | Lactococcus | rs10422691 | A | G | 0.111 | 0.025 | 6.48E-06 | 20.227 |
| genus | Streptococcus | rs1518775 | G | T | -0.050 | 0.011 | 6.38E-06 | 20.227 |
| genus | RikenellaceaeRC9gutgroup | rs2408405 | G | A | 0.111 | 0.025 | 8.92E-06 | 20.227 |
| genus | Streptococcus | rs2975635 | C | T | -0.056 | 0.013 | 7.04E-06 | 20.227 |
| genus | LachnospiraceaeUCG001 | rs11790553 | G | T | -0.065 | 0.014 | 5.97E-06 | 20.227 |
| genus | Bifidobacterium | rs4988145 | A | C | 0.072 | 0.016 | 7.85E-06 | 20.227 |
| genus |  | rs188296 | C | T | -0.063 | 0.014 | 6.77E-06 | 20.226 |
| genus | Marvinbryantia | rs9877395 | C | G | 0.061 | 0.014 | 6.78E-06 | 20.226 |
| genus |  | rs7164992 | G | C | -0.074 | 0.017 | 7.15E-06 | 20.226 |
| genus | Bacteroides | rs79471440 | G | T | 0.058 | 0.013 | 5.24E-06 | 20.226 |
| genus | Paraprevotella | rs4471727 | T | A | -0.078 | 0.017 | 9.62E-06 | 20.226 |
| genus |  | rs829969 | G | A | 0.081 | 0.018 | 8.38E-06 | 20.226 |
| genus | Ruminococcus2 | rs3823652 | T | A | 0.054 | 0.012 | 7.45E-06 | 20.226 |
| genus | Prevotella9 | rs16966465 | C | G | 0.074 | 0.017 | 9.33E-06 | 20.225 |
| genus | LachnospiraceaeUCG010 | rs72894970 | G | A | 0.218 | 0.049 | 7.97E-06 | 20.225 |
| genus | Howardella | rs36081916 | C | T | -0.181 | 0.040 | 4.70E-06 | 20.224 |
| genus | LachnospiraceaeUCG001 | rs11790086 | G | A | -0.065 | 0.014 | 6.61E-06 | 20.223 |
| genus | Coprococcus1 | rs7795598 | A | G | -0.054 | 0.012 | 8.53E-06 | 20.223 |
| genus | Eggerthella | rs1784438 | C | T | 0.089 | 0.020 | 8.11E-06 | 20.222 |
| genus | RuminococcaceaeUCG014 | rs162747 | G | A | -0.057 | 0.013 | 9.21E-06 | 20.222 |
| genus | Oxalobacter | rs6071432 | T | C | -0.096 | 0.021 | 7.50E-06 | 20.221 |
| genus | Parasutterella | rs62278066 | G | A | 0.226 | 0.050 | 8.13E-06 | 20.220 |
| genus | Anaerofilum | rs12030877 | T | C | 0.094 | 0.021 | 7.47E-06 | 20.220 |
| genus | Veillonella | rs35949599 | G | A | 0.087 | 0.019 | 7.55E-06 | 20.219 |
| genus | LachnospiraceaeND3007group | rs2861203 | A | G | 0.057 | 0.013 | 7.37E-06 | 20.218 |
| genus | RuminococcaceaeUCG014 | rs458026 | G | A | -0.057 | 0.013 | 9.68E-06 | 20.218 |
| genus | ChristensenellaceaeR | rs60954665 | G | T | 0.050 | 0.011 | 7.13E-06 | 20.218 |
| genus |  | rs57004694 | T | C | 0.144 | 0.032 | 8.26E-06 | 20.218 |
| genus | Parasutterella | rs72979695 | C | T | 0.086 | 0.019 | 7.98E-06 | 20.218 |
| genus | Prevotella9 | rs10204077 | G | A | -0.133 | 0.030 | 8.64E-06 | 20.217 |
| genus | Olsenella | rs11676178 | T | A | -0.107 | 0.024 | 6.51E-06 | 20.216 |
| genus | Oscillospira | rs751183 | C | T | -0.077 | 0.017 | 6.85E-06 | 20.216 |
| genus | Subdoligranulum | rs73289253 | A | C | -0.061 | 0.014 | 5.19E-06 | 20.216 |
| genus | RuminococcaceaeUCG014 | rs466005 | A | T | -0.057 | 0.013 | 8.99E-06 | 20.216 |
| genus | Coprococcus3 | rs4575475 | A | G | 0.062 | 0.014 | 7.04E-06 | 20.215 |
| genus | Romboutsia | rs10893902 | C | T | -0.071 | 0.016 | 9.93E-06 | 20.215 |
| genus | Allisonella | rs7898615 | G | T | 0.168 | 0.037 | 8.87E-06 | 20.214 |
| genus | Lactococcus | rs4806169 | C | T | 0.111 | 0.025 | 6.37E-06 | 20.214 |
| genus | Gordonibacter | rs72714790 | T | C | 0.169 | 0.038 | 7.46E-06 | 20.213 |
| genus | Parasutterella | rs11058438 | G | T | -0.072 | 0.016 | 7.72E-06 | 20.213 |
| genus | Coprococcus2 | rs35916879 | G | C | 0.068 | 0.015 | 5.44E-06 | 20.213 |
| genus |  | rs368871882 | T | C | 0.232 | 0.052 | 8.23E-06 | 20.213 |
| genus | Gordonibacter | rs768830 | A | G | 0.150 | 0.033 | 7.76E-06 | 20.212 |
| genus | Roseburia | rs1897835 | C | T | 0.048 | 0.011 | 7.61E-06 | 20.212 |
| genus |  | rs289414 | C | T | -0.063 | 0.014 | 6.74E-06 | 20.211 |
| genus | Turicibacter | rs11666533 | T | C | -0.112 | 0.025 | 7.37E-06 | 20.211 |
| genus | Ruminiclostridium9 | rs78191726 | C | T | 0.094 | 0.021 | 7.58E-06 | 20.210 |
| genus | Alistipes | rs11958296 | G | A | -0.098 | 0.022 | 9.30E-06 | 20.209 |
| genus | LachnospiraceaeFCS020group | rs1542072 | A | G | 0.055 | 0.012 | 7.80E-06 | 20.209 |
| genus | Parasutterella | rs6551865 | G | A | 0.060 | 0.013 | 7.45E-06 | 20.208 |
| genus | Anaerofilum | rs12030055 | T | C | 0.094 | 0.021 | 7.53E-06 | 20.208 |
| genus | Anaerostipes | rs11197341 | G | A | -0.049 | 0.011 | 7.64E-06 | 20.207 |
| genus | Marvinbryantia | rs9813109 | G | A | 0.061 | 0.014 | 6.76E-06 | 20.206 |
| genus | Ruminiclostridium9 | rs959654 | A | T | -0.051 | 0.011 | 8.17E-06 | 20.206 |
| genus | Butyrivibrio | rs9579032 | A | G | -0.117 | 0.026 | 6.89E-06 | 20.206 |
| genus | Parasutterella | rs62273920 | C | T | 0.226 | 0.050 | 8.39E-06 | 20.205 |
| genus | Eggerthella | rs2459479 | T | C | 0.089 | 0.020 | 7.93E-06 | 20.204 |
| genus | Eggerthella | rs2075012 | A | T | -0.089 | 0.020 | 8.23E-06 | 20.203 |
| genus | Anaerostipes | rs1590734 | G | C | -0.049 | 0.011 | 7.71E-06 | 20.203 |
| genus | Alistipes | rs8132143 | A | T | -0.048 | 0.011 | 7.04E-06 | 20.202 |
| genus | Eisenbergiella | rs12440724 | A | G | 0.101 | 0.022 | 7.34E-06 | 20.200 |
| genus | Ruminiclostridium5 | rs7974422 | G | A | 0.048 | 0.011 | 7.54E-06 | 20.200 |
| genus | Olsenella | rs2424252 | G | A | 0.113 | 0.025 | 6.75E-06 | 20.199 |
| genus | Howardella | rs10515986 | A | G | 0.155 | 0.034 | 9.08E-06 | 20.198 |
| genus | ErysipelotrichaceaeUCG003 | rs8053479 | G | A | -0.084 | 0.019 | 5.83E-06 | 20.198 |
| genus | Lactococcus | rs12619377 | T | G | 0.108 | 0.024 | 7.50E-06 | 20.198 |
| genus | RuminococcaceaeUCG014 | rs457444 | G | A | -0.057 | 0.013 | 8.99E-06 | 20.197 |
| genus | RuminococcaceaeUCG014 | rs16845608 | A | G | -0.093 | 0.021 | 7.82E-06 | 20.197 |
| genus | Anaerofilum | rs4244069 | A | G | -0.147 | 0.033 | 9.81E-06 | 20.197 |
| genus | Sutterella | rs7192289 | A | C | 0.059 | 0.013 | 6.60E-06 | 20.196 |
| genus | LachnospiraceaeUCG004 | rs4134425 | C | T | 0.056 | 0.013 | 8.00E-06 | 20.196 |
| genus |  | rs6991627 | A | C | 0.073 | 0.016 | 7.43E-06 | 20.195 |
| genus | RuminococcaceaeUCG009 | rs7960210 | G | T | 0.078 | 0.017 | 7.68E-06 | 20.195 |
| genus | RuminococcaceaeUCG004 | rs2045819 | C | T | 0.082 | 0.018 | 6.23E-06 | 20.193 |
| genus | Ruminococcus1 | rs10769165 | T | C | -0.050 | 0.011 | 7.59E-06 | 20.193 |
| genus | LachnospiraceaeNC2004group | rs12134680 | A | C | 0.111 | 0.025 | 9.06E-06 | 20.192 |
| genus | CandidatusSoleaferrea | rs6489992 | G | A | -0.084 | 0.019 | 7.89E-06 | 20.192 |
| genus | FamilyXIIIAD3011group | rs71461085 | T | G | -0.087 | 0.019 | 7.67E-06 | 20.192 |
| genus | Roseburia | rs302266 | C | T | -0.078 | 0.017 | 8.13E-06 | 20.192 |
| genus | Collinsella | rs62448869 | A | T | -0.054 | 0.012 | 6.82E-06 | 20.190 |
| genus | Oxalobacter | rs6093023 | C | T | -0.096 | 0.021 | 7.58E-06 | 20.190 |
| genus | Ruminiclostridium9 | rs9809789 | T | C | -0.072 | 0.016 | 8.72E-06 | 20.189 |
| genus | LachnospiraceaeNK4A136group | rs11263806 | G | A | -0.052 | 0.012 | 5.07E-06 | 20.189 |
| genus | Streptococcus | rs11110057 | G | A | -0.084 | 0.019 | 7.44E-06 | 20.189 |
| genus | ErysipelotrichaceaeUCG003 | rs8083334 | T | C | -0.098 | 0.022 | 6.63E-06 | 20.187 |
| genus | RuminococcaceaeUCG005 | rs10937802 | A | G | 0.076 | 0.017 | 8.17E-06 | 20.185 |
| genus |  | rs10506058 | G | A | 0.100 | 0.022 | 8.92E-06 | 20.184 |
| genus | Bifidobacterium | rs79954860 | A | T | 0.110 | 0.025 | 6.52E-06 | 20.184 |
| genus | Roseburia | rs2972974 | C | G | 0.048 | 0.011 | 7.67E-06 | 20.184 |
| genus | Sellimonas | rs2554334 | T | C | 0.113 | 0.025 | 7.40E-06 | 20.184 |
| genus | Turicibacter | rs111439215 | A | C | 0.097 | 0.022 | 6.64E-06 | 20.184 |
| genus | RuminococcaceaeUCG003 | rs16908667 | G | A | -0.060 | 0.013 | 7.27E-06 | 20.184 |
| genus |  | rs1959391 | A | C | -0.053 | 0.012 | 6.64E-06 | 20.184 |
| genus | Coprococcus1 | rs73167075 | C | T | 0.057 | 0.013 | 8.57E-06 | 20.184 |
| genus | Ruminiclostridium6 | rs67479537 | C | T | 0.119 | 0.026 | 9.30E-06 | 20.183 |
| genus | LachnospiraceaeFCS020group | rs9829194 | T | G | 0.058 | 0.013 | 7.54E-06 | 20.182 |
| genus |  | rs2132502 | T | G | -0.082 | 0.018 | 8.11E-06 | 20.182 |
| genus | Bifidobacterium | rs17241561 | G | T | -0.059 | 0.013 | 8.14E-06 | 20.181 |
| genus | Veillonella | rs11141494 | A | G | -0.078 | 0.017 | 9.75E-06 | 20.181 |
| genus | Sellimonas | rs13200972 | G | A | 0.114 | 0.025 | 9.21E-06 | 20.180 |
| genus | LachnospiraceaeUCG008 | rs6808242 | G | T | -0.101 | 0.022 | 5.81E-06 | 20.179 |
| genus | RuminococcaceaeUCG004 | rs3800154 | C | A | -0.080 | 0.018 | 6.12E-06 | 20.178 |
| genus | Intestinimonas | rs2276760 | G | A | -0.069 | 0.015 | 7.84E-06 | 20.178 |
| genus | Ruminococcus2 | rs2997410 | T | C | -0.055 | 0.012 | 8.01E-06 | 20.178 |
| genus | Peptococcus | rs441637 | T | C | -0.098 | 0.022 | 8.90E-06 | 20.176 |
| genus | Erysipelatoclostridium | rs401869 | C | A | 0.076 | 0.017 | 7.82E-06 | 20.175 |
| genus | Coprococcus2 | rs13111161 | G | C | -0.063 | 0.014 | 5.37E-06 | 20.175 |
| genus | Ruminiclostridium9 | rs28431969 | A | C | -0.056 | 0.013 | 6.61E-06 | 20.174 |
| genus | FamilyXIIIUCG001 | rs62414824 | T | C | -0.060 | 0.013 | 8.22E-06 | 20.174 |
| genus | Anaerostipes | rs11197321 | T | C | -0.049 | 0.011 | 7.82E-06 | 20.174 |
| genus | Bifidobacterium | rs1893396 | G | T | 0.054 | 0.012 | 4.95E-06 | 20.173 |
| genus | RuminococcaceaeUCG011 | rs4490371 | C | T | -0.112 | 0.025 | 7.75E-06 | 20.172 |
| genus | Ruminiclostridium9 | rs59979723 | C | A | 0.070 | 0.015 | 5.19E-06 | 20.170 |
| genus | Ruminiclostridium5 | rs2801956 | A | G | 0.052 | 0.012 | 7.15E-06 | 20.169 |
| genus | LachnospiraceaeND3007group | rs11647528 | T | G | -0.052 | 0.012 | 7.27E-06 | 20.169 |
| genus |  | rs289408 | A | G | -0.063 | 0.014 | 6.98E-06 | 20.168 |
| genus | Holdemania | rs4146507 | T | C | 0.079 | 0.018 | 7.23E-06 | 20.167 |
| genus | Akkermansia | rs12026127 | G | A | 0.064 | 0.014 | 6.97E-06 | 20.166 |
| genus | Peptococcus | rs2346470 | G | A | 0.107 | 0.024 | 6.77E-06 | 20.165 |
| genus | Collinsella | rs62448871 | A | C | -0.054 | 0.012 | 6.78E-06 | 20.164 |
| genus | Erysipelatoclostridium | rs1434153 | A | G | -0.068 | 0.015 | 6.85E-06 | 20.163 |
| genus | Howardella | rs6855681 | A | C | -0.107 | 0.024 | 8.29E-06 | 20.163 |
| genus | Eggerthella | rs1784444 | T | C | 0.089 | 0.020 | 8.04E-06 | 20.163 |
| genus | Sellimonas | rs2006772 | A | G | 0.113 | 0.025 | 7.54E-06 | 20.162 |
| genus | Tyzzerella3 | rs60791882 | C | T | -0.214 | 0.048 | 7.97E-06 | 20.162 |
| genus | Coprococcus1 | rs1762123 | T | C | -0.089 | 0.020 | 8.01E-06 | 20.161 |
| genus | Fusicatenibacter | rs7476505 | C | A | -0.088 | 0.020 | 7.60E-06 | 20.161 |
| genus | Peptococcus | rs62003651 | A | G | 0.105 | 0.023 | 8.90E-06 | 20.161 |
| genus | Victivallis | rs2093422 | T | A | -0.118 | 0.026 | 8.31E-06 | 20.160 |
| genus | Lactobacillus | rs1978760 | T | C | -0.077 | 0.017 | 7.53E-06 | 20.159 |
| genus | Butyrivibrio | rs28846706 | G | A | 0.176 | 0.039 | 7.87E-06 | 20.159 |
| genus | Oxalobacter | rs73733941 | C | T | -0.133 | 0.030 | 7.79E-06 | 20.159 |
| genus | Turicibacter | rs113203503 | C | T | 0.097 | 0.022 | 6.89E-06 | 20.158 |
| genus | Coprobacter | rs12231438 | G | T | -0.102 | 0.023 | 8.90E-06 | 20.158 |
| genus | Lachnospira | rs159484 | A | G | 0.079 | 0.018 | 6.68E-06 | 20.157 |
| genus |  | rs833386 | A | G | -0.113 | 0.025 | 7.61E-06 | 20.157 |
| genus | Marvinbryantia | rs4336070 | G | A | 0.061 | 0.014 | 6.97E-06 | 20.157 |
| genus | Alistipes | rs62576416 | C | T | 0.049 | 0.011 | 7.50E-06 | 20.156 |
| genus | Subdoligranulum | rs1954128 | G | A | -0.054 | 0.012 | 8.57E-06 | 20.156 |
| genus | Alistipes | rs12990744 | T | C | -0.078 | 0.017 | 8.21E-06 | 20.156 |
| genus | Marvinbryantia | rs2768426 | T | C | -0.072 | 0.016 | 8.62E-06 | 20.155 |
| genus | Lactococcus | rs4806170 | A | G | 0.111 | 0.025 | 6.56E-06 | 20.154 |
| genus | Coprococcus1 | rs1932315 | A | G | -0.049 | 0.011 | 7.57E-06 | 20.154 |
| genus |  | rs11661723 | A | T | 0.147 | 0.033 | 6.87E-06 | 20.153 |
| genus |  | rs699510 | T | C | 0.065 | 0.015 | 7.42E-06 | 20.153 |
| genus | Barnesiella | rs11155559 | C | T | 0.096 | 0.021 | 8.92E-06 | 20.153 |
| genus | Allisonella | rs12554887 | C | G | 0.142 | 0.032 | 7.78E-06 | 20.153 |
| genus | Veillonella | rs1815011 | G | A | 0.086 | 0.019 | 7.81E-06 | 20.153 |
| genus | RuminococcaceaeUCG005 | rs17541562 | G | C | 0.144 | 0.032 | 8.62E-06 | 20.152 |
| genus | Olsenella | rs12649679 | G | A | -0.104 | 0.023 | 7.75E-06 | 20.152 |
| genus | Ruminiclostridium6 | rs144085351 | C | G | -0.097 | 0.022 | 3.67E-06 | 20.152 |
| genus | RuminococcaceaeUCG004 | rs9839906 | A | C | 0.075 | 0.017 | 8.09E-06 | 20.152 |
| genus | Anaerotruncus | rs9594204 | C | T | 0.074 | 0.017 | 3.35E-06 | 20.151 |
| genus | Adlercreutzia | rs12650418 | C | T | -0.084 | 0.019 | 6.60E-06 | 20.149 |
| genus |  | rs73747127 | A | T | 0.103 | 0.023 | 5.13E-06 | 20.148 |
| genus | Parabacteroides | rs17141986 | C | G | 0.050 | 0.011 | 7.27E-06 | 20.148 |
| genus | Oxalobacter | rs6101123 | T | C | -0.096 | 0.021 | 7.75E-06 | 20.148 |
| genus | Coprobacter | rs6496714 | T | C | 0.099 | 0.022 | 6.93E-06 | 20.147 |
| genus | Peptococcus | rs6573151 | A | C | 0.106 | 0.024 | 6.67E-06 | 20.147 |
| genus | Allisonella | rs34754033 | A | G | 0.136 | 0.030 | 8.54E-06 | 20.146 |
| genus | Lactobacillus | rs78414635 | T | G | -0.172 | 0.038 | 5.85E-06 | 20.145 |
| genus | Lactococcus | rs12620383 | A | T | -0.193 | 0.043 | 2.43E-06 | 20.145 |
| genus | Parasutterella | rs35884570 | T | C | 0.105 | 0.023 | 7.71E-06 | 20.145 |
| genus | Lachnoclostridium | rs6045950 | A | G | -0.049 | 0.011 | 7.74E-06 | 20.144 |
| genus | Lactococcus | . | G | A | 0.111 | 0.025 | 6.58E-06 | 20.144 |
| genus | Lachnoclostridium | rs3821998 | A | C | -0.086 | 0.019 | 6.72E-06 | 20.144 |
| genus |  | rs2348119 | T | C | -0.074 | 0.016 | 7.36E-06 | 20.142 |
| genus | RuminococcaceaeUCG004 | rs114565663 | G | A | 0.131 | 0.029 | 7.27E-06 | 20.142 |
| genus | Methanobrevibacter | rs75597686 | A | G | 0.203 | 0.045 | 9.05E-06 | 20.142 |
| genus | Blautia | rs55821618 | G | A | 0.055 | 0.012 | 5.85E-06 | 20.142 |
| genus | Lachnospira | rs10767477 | A | T | -0.060 | 0.013 | 5.31E-06 | 20.141 |
| genus | Eisenbergiella | rs12710729 | A | C | 0.089 | 0.020 | 9.84E-06 | 20.140 |
| genus | Collinsella | rs7102347 | A | C | -0.053 | 0.012 | 7.61E-06 | 20.140 |
| genus | Butyrivibrio | rs9899333 | T | C | 0.116 | 0.026 | 6.26E-06 | 20.139 |
| genus | RuminococcaceaeUCG003 | rs454715 | G | T | 0.055 | 0.012 | 7.78E-06 | 20.139 |
| genus | Anaerostipes | rs11197323 | C | T | -0.049 | 0.011 | 7.97E-06 | 20.139 |
| genus | Anaerostipes | rs882172 | A | G | 0.050 | 0.011 | 9.89E-06 | 20.139 |
| genus | Anaerofilum | rs56342910 | G | T | 0.112 | 0.025 | 7.56E-06 | 20.138 |
| genus | Holdemania | rs80149660 | T | C | -0.233 | 0.052 | 6.04E-06 | 20.138 |
| genus |  | rs833380 | C | T | -0.113 | 0.025 | 7.70E-06 | 20.137 |
| genus |  | rs17459885 | T | C | 0.105 | 0.023 | 8.62E-06 | 20.137 |
| genus | RikenellaceaeRC9gutgroup | rs2897922 | A | G | 0.111 | 0.025 | 9.18E-06 | 20.137 |
| genus |  | rs482376 | C | T | 0.081 | 0.018 | 8.92E-06 | 20.137 |
| genus | Butyrivibrio | rs4928024 | G | A | -0.175 | 0.039 | 8.19E-06 | 20.137 |
| genus |  | rs4682185 | A | G | -0.052 | 0.012 | 6.95E-06 | 20.137 |
| genus | Butyrivibrio | rs77356209 | C | T | 0.217 | 0.048 | 6.66E-06 | 20.137 |
| genus | LachnospiraceaeUCG008 | . | T | C | 0.077 | 0.017 | 7.33E-06 | 20.136 |
| genus | Ruminiclostridium9 | rs62287934 | C | G | -0.051 | 0.011 | 8.43E-06 | 20.136 |
| genus | RuminococcaceaeUCG004 | rs2248146 | C | T | 0.069 | 0.015 | 8.20E-06 | 20.135 |
| genus |  | rs12515623 | T | G | -0.096 | 0.021 | 6.89E-06 | 20.135 |
| genus | Sellimonas | rs2554345 | G | A | 0.113 | 0.025 | 7.58E-06 | 20.134 |
| genus | Sutterella | rs7638039 | C | T | 0.065 | 0.014 | 8.66E-06 | 20.133 |
| genus | Coprococcus3 | rs58719470 | A | G | -0.063 | 0.014 | 7.92E-06 | 20.133 |
| genus | Collinsella | rs62448870 | A | G | -0.054 | 0.012 | 6.90E-06 | 20.133 |
| genus | RuminococcaceaeUCG014 | rs332654 | A | G | -0.057 | 0.013 | 9.62E-06 | 20.132 |
| genus | Holdemania | rs80134932 | C | G | -0.132 | 0.029 | 6.46E-06 | 20.131 |
| genus | Desulfovibrio | rs746985 | C | T | -0.067 | 0.015 | 7.33E-06 | 20.131 |
| genus | ChristensenellaceaeR | rs78024454 | A | T | 0.112 | 0.025 | 4.39E-06 | 20.129 |
| genus | Bifidobacterium | rs10928507 | G | A | 0.053 | 0.012 | 8.08E-06 | 20.127 |
| genus | RuminococcaceaeUCG002 | rs10927427 | C | A | -0.066 | 0.015 | 4.94E-06 | 20.126 |
| genus |  | rs67084241 | G | A | -0.091 | 0.020 | 6.02E-06 | 20.126 |
| genus | Butyrivibrio | rs240993 | C | T | -0.119 | 0.026 | 7.21E-06 | 20.126 |
| genus | LachnospiraceaeUCG001 | . | A | G | -0.066 | 0.015 | 5.58E-06 | 20.126 |
| genus | Sutterella | rs1859743 | C | T | -0.056 | 0.012 | 6.93E-06 | 20.125 |
| genus | Olsenella | rs13004145 | C | T | -0.106 | 0.024 | 6.86E-06 | 20.125 |
| genus |  | rs16886724 | C | A | -0.091 | 0.020 | 6.25E-06 | 20.125 |
| genus |  | rs67308382 | A | G | -0.091 | 0.020 | 6.25E-06 | 20.125 |
| genus | Ruminococcus2 | rs2846589 | T | G | 0.052 | 0.012 | 7.59E-06 | 20.125 |
| genus |  | rs2817174 | T | C | -0.073 | 0.016 | 7.87E-06 | 20.125 |
| genus | Sutterella | rs2613606 | T | C | -0.056 | 0.012 | 7.20E-06 | 20.124 |
| genus | Eisenbergiella | rs28716358 | C | T | 0.101 | 0.023 | 6.55E-06 | 20.124 |
| genus | CandidatusSoleaferrea | rs11153159 | C | G | -0.128 | 0.029 | 4.42E-06 | 20.124 |
| genus | Coprococcus1 | rs7795435 | C | G | -0.053 | 0.012 | 8.95E-06 | 20.123 |
| genus | Alistipes | rs1822113 | G | T | -0.049 | 0.011 | 7.39E-06 | 20.123 |
| genus | Veillonella | rs35309151 | T | C | 0.086 | 0.019 | 7.90E-06 | 20.123 |
| genus | RikenellaceaeRC9gutgroup | rs7931333 | T | C | 0.111 | 0.025 | 9.27E-06 | 20.122 |
| genus | RuminococcaceaeUCG014 | rs332724 | C | T | -0.057 | 0.013 | 9.81E-06 | 20.121 |
| genus | Paraprevotella | rs72810878 | A | G | -0.121 | 0.027 | 7.55E-06 | 20.121 |
| genus | Senegalimassilia | rs10199944 | C | A | 0.077 | 0.017 | 7.60E-06 | 20.120 |
| genus | FamilyXIIIUCG001 | rs12606 | C | T | -0.060 | 0.013 | 6.99E-06 | 20.120 |
| genus | ChristensenellaceaeR | rs79150079 | A | C | 0.122 | 0.027 | 9.42E-06 | 20.120 |
| genus |  | rs55737168 | A | T | -0.162 | 0.036 | 7.82E-06 | 20.118 |
| genus | Streptococcus | rs991041 | T | A | -0.050 | 0.011 | 7.48E-06 | 20.116 |
| genus | Ruminococcus1 | rs1466068 | C | T | -0.050 | 0.011 | 8.55E-06 | 20.116 |
| genus | CandidatusSoleaferrea | rs13269653 | A | G | -0.090 | 0.020 | 7.67E-06 | 20.115 |
| genus | Butyrivibrio | rs12087848 | C | T | 0.176 | 0.039 | 8.72E-06 | 20.113 |
| genus | Streptococcus | rs57441879 | G | A | 0.096 | 0.021 | 8.25E-06 | 20.113 |
| genus | Butyrivibrio | rs142855850 | G | A | 0.205 | 0.046 | 6.86E-06 | 20.113 |
| genus | Coprococcus3 | rs9655998 | G | T | 0.051 | 0.011 | 9.40E-06 | 20.111 |
| genus | Akkermansia | rs7538356 | A | C | 0.064 | 0.014 | 7.58E-06 | 20.111 |
| genus | Alistipes | rs8132424 | A | T | -0.048 | 0.011 | 7.38E-06 | 20.111 |
| genus | LachnospiraceaeND3007group | rs13110238 | G | C | -0.064 | 0.014 | 4.48E-06 | 20.110 |
| genus | Coprococcus2 | rs6894272 | C | T | -0.113 | 0.025 | 9.53E-06 | 20.110 |
| genus | RuminococcaceaeUCG011 | rs6784050 | C | T | 0.131 | 0.029 | 7.60E-06 | 20.109 |
| genus | Marvinbryantia | rs649360 | T | C | 0.061 | 0.014 | 7.09E-06 | 20.109 |
| genus | Ruminiclostridium6 | rs78755044 | G | A | 0.134 | 0.030 | 9.08E-06 | 20.108 |
| genus | ErysipelotrichaceaeUCG003 | rs62102329 | T | C | -0.098 | 0.022 | 6.94E-06 | 20.107 |
| genus | Butyrivibrio | rs36073314 | A | G | 0.176 | 0.039 | 8.96E-06 | 20.107 |
| genus | Tyzzerella3 | rs73291122 | G | C | -0.214 | 0.048 | 7.69E-06 | 20.107 |
| genus | CandidatusSoleaferrea | rs62203853 | C | A | 0.100 | 0.022 | 8.38E-06 | 20.106 |
| genus |  | rs4656007 | T | C | 0.072 | 0.016 | 7.90E-06 | 20.106 |
| genus |  | rs10858049 | A | G | 0.088 | 0.020 | 5.35E-06 | 20.105 |
| genus | Eisenbergiella | rs12417319 | T | C | -0.113 | 0.025 | 7.93E-06 | 20.104 |
| genus | Clostridiumsensustricto1 | rs12490337 | G | C | -0.062 | 0.014 | 7.49E-06 | 20.104 |
| genus | Victivallis | rs2093420 | C | T | -0.118 | 0.026 | 8.56E-06 | 20.103 |
| genus | Blautia | rs67597570 | T | C | 0.055 | 0.012 | 6.23E-06 | 20.102 |
| genus | Anaerostipes | rs11197337 | C | T | -0.049 | 0.011 | 8.07E-06 | 20.101 |
| genus | Odoribacter | rs2287756 | C | G | 0.054 | 0.012 | 8.73E-06 | 20.101 |
| genus | Intestinibacter | rs7032680 | C | T | -0.104 | 0.023 | 6.54E-06 | 20.101 |
| genus |  | rs115325767 | C | G | 0.078 | 0.017 | 2.62E-06 | 20.100 |
| genus | Haemophilus | rs12876183 | A | T | 0.075 | 0.017 | 9.62E-06 | 20.100 |
| genus | Desulfovibrio | rs1294544 | C | A | 0.069 | 0.015 | 7.78E-06 | 20.099 |
| genus | Streptococcus | rs11006995 | C | T | 0.096 | 0.021 | 7.68E-06 | 20.099 |
| genus | Lachnospira | rs2520509 | G | A | 0.052 | 0.012 | 7.42E-06 | 20.099 |
| genus |  | rs73747128 | T | C | 0.103 | 0.023 | 5.27E-06 | 20.098 |
| genus | Fusicatenibacter | rs4477213 | C | T | -0.052 | 0.012 | 6.89E-06 | 20.096 |
| genus |  | rs2939752 | G | A | 0.088 | 0.020 | 7.69E-06 | 20.096 |
| genus |  | rs10967781 | A | C | 0.051 | 0.011 | 8.37E-06 | 20.095 |
| genus | RuminococcaceaeUCG014 | rs17296933 | G | C | -0.083 | 0.019 | 7.34E-06 | 20.094 |
| genus |  | rs1181573 | A | G | 0.065 | 0.015 | 7.44E-06 | 20.094 |
| genus | LachnospiraceaeFCS020group | rs9818658 | T | G | 0.055 | 0.012 | 8.23E-06 | 20.094 |
| genus | Tyzzerella3 | rs113019116 | A | G | -0.214 | 0.048 | 8.77E-06 | 20.093 |
| genus | Sutterella | rs27727 | T | C | -0.059 | 0.013 | 9.21E-06 | 20.093 |
| genus |  | rs10931918 | G | A | -0.073 | 0.016 | 7.60E-06 | 20.093 |
| genus | Oscillibacter | rs1837782 | C | A | 0.074 | 0.017 | 7.64E-06 | 20.092 |
| genus | Prevotella9 | rs72815774 | C | T | -0.176 | 0.039 | 8.78E-06 | 20.091 |
| genus | LachnospiraceaeFCS020group | rs12221149 | T | G | -0.055 | 0.012 | 7.70E-06 | 20.091 |
| genus | Allisonella | rs3747511 | C | G | 0.134 | 0.030 | 8.20E-06 | 20.091 |
| genus | CandidatusSoleaferrea | rs2193878 | A | T | 0.228 | 0.051 | 9.46E-06 | 20.091 |
| genus | RikenellaceaeRC9gutgroup | rs7941564 | G | A | 0.111 | 0.025 | 9.43E-06 | 20.091 |
| genus | LachnospiraceaeFCS020group | rs9851122 | G | A | 0.055 | 0.012 | 8.23E-06 | 20.090 |
| genus | RuminococcaceaeUCG013 | rs9313055 | C | T | 0.105 | 0.023 | 9.55E-06 | 20.089 |
| genus | RuminococcaceaeUCG011 | rs6966809 | G | A | 0.110 | 0.024 | 8.20E-06 | 20.089 |
| genus |  | rs66830358 | A | T | 0.053 | 0.012 | 6.87E-06 | 20.088 |
| genus |  | rs12117558 | G | A | 0.089 | 0.020 | 6.35E-06 | 20.087 |
| genus | Dorea | rs2917871 | C | G | 0.051 | 0.011 | 8.02E-06 | 20.086 |
| genus | Sutterella | rs607327 | T | C | 0.058 | 0.013 | 6.63E-06 | 20.083 |
| genus | Anaerofilum | rs12057312 | C | T | 0.093 | 0.021 | 8.06E-06 | 20.082 |
| genus | Marvinbryantia | rs9817187 | C | T | 0.061 | 0.014 | 7.35E-06 | 20.082 |
| genus | RuminococcaceaeNK4A214group | rs34576931 | C | G | -0.087 | 0.019 | 4.72E-06 | 20.081 |
| genus | Coprococcus1 | rs1590791 | T | C | -0.049 | 0.011 | 7.87E-06 | 20.081 |
| genus | Sellimonas | rs73077365 | T | C | -0.161 | 0.036 | 8.84E-06 | 20.080 |
| genus | Anaerostipes | rs2149112 | A | G | -0.049 | 0.011 | 8.57E-06 | 20.079 |
| genus | Senegalimassilia | rs7584117 | G | T | 0.077 | 0.017 | 7.79E-06 | 20.079 |
| genus | Oscillibacter | rs744426 | G | A | 0.094 | 0.021 | 9.94E-06 | 20.079 |
| genus | Anaerotruncus | rs5010916 | G | T | 0.074 | 0.017 | 3.56E-06 | 20.078 |
| genus | ErysipelotrichaceaeUCG003 | rs28394575 | T | A | 0.104 | 0.023 | 4.10E-06 | 20.077 |
| genus | Oscillibacter | rs11996437 | C | T | -0.081 | 0.018 | 7.65E-06 | 20.077 |
| genus | Alistipes | rs10950056 | G | C | -0.049 | 0.011 | 7.71E-06 | 20.077 |
| genus |  | rs11256291 | T | C | 0.152 | 0.034 | 9.65E-06 | 20.076 |
| genus | Escherichia | rs112767262 | C | T | 0.073 | 0.016 | 8.21E-06 | 20.075 |
| genus | Anaerotruncus | rs1431492 | T | C | -0.065 | 0.015 | 7.36E-06 | 20.075 |
| genus | LachnospiraceaeUCG004 | rs62256516 | A | G | 0.059 | 0.013 | 9.85E-06 | 20.071 |
| genus | Parasutterella | rs747286 | C | T | 0.060 | 0.013 | 6.48E-06 | 20.070 |
| genus | Intestinibacter | rs2098844 | T | C | -0.058 | 0.013 | 6.79E-06 | 20.070 |
| genus |  | rs2323451 | C | G | 0.048 | 0.011 | 6.07E-06 | 20.069 |
| genus |  | rs6868279 | T | G | -0.081 | 0.018 | 8.58E-06 | 20.069 |
| genus | RuminococcaceaeUCG004 | rs1992963 | C | T | 0.076 | 0.017 | 9.53E-06 | 20.068 |
| genus | Oscillibacter | rs9885928 | G | A | -0.067 | 0.015 | 8.66E-06 | 20.068 |
| genus | Streptococcus | rs2952176 | A | G | -0.056 | 0.013 | 7.85E-06 | 20.067 |
| genus |  | rs11006576 | G | A | -0.110 | 0.025 | 7.99E-06 | 20.067 |
| genus | Ruminiclostridium5 | rs4648667 | A | G | 0.088 | 0.020 | 2.92E-06 | 20.067 |
| genus | Lachnoclostridium | rs1993453 | G | A | -0.051 | 0.011 | 7.62E-06 | 20.067 |
| genus | Alloprevotella | rs4364940 | G | A | 0.126 | 0.028 | 8.58E-06 | 20.067 |
| genus | Ruminiclostridium9 | rs9522719 | G | A | 0.069 | 0.015 | 5.49E-06 | 20.067 |
| genus | Peptococcus | rs437943 | T | C | -0.089 | 0.020 | 8.21E-06 | 20.066 |
| genus | RuminococcaceaeUCG014 | rs162748 | C | T | -0.057 | 0.013 | 1.00E-05 | 20.066 |
| genus | LachnospiraceaeFCS020group | rs10030408 | G | A | 0.055 | 0.012 | 7.60E-06 | 20.066 |
| genus | Coprococcus2 | rs4376165 | T | C | -0.062 | 0.014 | 5.83E-06 | 20.065 |
| genus | Bifidobacterium | rs2686790 | C | T | 0.071 | 0.016 | 7.50E-06 | 20.065 |
| genus | LachnospiraceaeUCG004 | rs9638636 | T | A | 0.053 | 0.012 | 7.12E-06 | 20.064 |
| genus | Actinomyces | rs4073240 | A | G | 0.075 | 0.017 | 7.94E-06 | 20.064 |
| genus | FamilyXIIIAD3011group | rs12311123 | T | C | -0.087 | 0.019 | 7.74E-06 | 20.063 |
| genus | ErysipelotrichaceaeUCG003 | rs2044550 | C | T | -0.061 | 0.014 | 7.25E-06 | 20.062 |
| genus | Olsenella | rs2152319 | A | C | -0.105 | 0.023 | 8.82E-06 | 20.062 |
| genus | Ruminiclostridium9 | rs1435643 | C | T | -0.051 | 0.011 | 8.78E-06 | 20.060 |
| genus | Victivallis | rs7651518 | T | G | 0.112 | 0.025 | 8.21E-06 | 20.059 |
| genus |  | rs4654122 | G | C | 0.055 | 0.012 | 7.20E-06 | 20.058 |
| genus | RuminococcaceaeUCG003 | rs16908643 | G | A | -0.060 | 0.013 | 8.08E-06 | 20.058 |
| genus |  | rs13073982 | T | C | -0.052 | 0.012 | 7.52E-06 | 20.058 |
| genus | RuminococcaceaeUCG002 | rs882348 | G | A | -0.080 | 0.018 | 5.45E-06 | 20.057 |
| genus | LachnospiraceaeFCS020group | rs9308097 | G | A | 0.055 | 0.012 | 7.47E-06 | 20.057 |
| genus | RikenellaceaeRC9gutgroup | rs6912851 | A | G | 0.171 | 0.038 | 7.68E-06 | 20.057 |
| genus | Coprobacter | rs11073942 | C | T | 0.098 | 0.022 | 7.92E-06 | 20.056 |
| genus | LachnospiraceaeUCG001 | rs2371284 | C | T | -0.076 | 0.017 | 7.56E-06 | 20.056 |
| genus |  | rs7212494 | G | C | 0.048 | 0.011 | 5.99E-06 | 20.055 |
| genus | Bifidobacterium | rs78274583 | C | T | 0.073 | 0.016 | 9.03E-06 | 20.055 |
| genus |  | rs2200429 | G | A | -0.089 | 0.020 | 5.30E-06 | 20.054 |
| genus | Parasutterella | rs6551867 | C | A | 0.059 | 0.013 | 8.08E-06 | 20.052 |
| genus | Alloprevotella | rs17380632 | T | A | 0.126 | 0.028 | 9.27E-06 | 20.051 |
| genus | Oscillibacter | rs55633183 | C | T | -0.126 | 0.028 | 7.97E-06 | 20.051 |
| genus |  | rs7844020 | C | G | -0.102 | 0.023 | 7.23E-06 | 20.049 |
| genus | Lactococcus | rs2384050 | A | G | 0.104 | 0.023 | 9.87E-06 | 20.047 |
| genus | Parasutterella | rs10899916 | C | T | -0.061 | 0.014 | 7.52E-06 | 20.047 |
| genus | Streptococcus | rs71481756 | G | T | 0.093 | 0.021 | 6.51E-06 | 20.046 |
| genus | Erysipelatoclostridium | rs34528142 | G | C | -0.088 | 0.020 | 6.13E-06 | 20.046 |
| genus | Bifidobacterium | rs10193171 | G | A | 0.053 | 0.012 | 8.40E-06 | 20.046 |
| genus | Allisonella | rs12550862 | A | C | 0.139 | 0.031 | 8.67E-06 | 20.045 |
| genus | FamilyXIIIUCG001 | rs62414798 | T | G | -0.060 | 0.013 | 6.35E-06 | 20.045 |
| genus | Ruminiclostridium5 | rs2996011 | C | T | 0.052 | 0.012 | 7.68E-06 | 20.045 |
| genus | Lachnoclostridium | rs73232901 | C | A | -0.086 | 0.019 | 7.13E-06 | 20.044 |
| genus | Lactococcus | rs10402279 | G | C | 0.111 | 0.025 | 7.25E-06 | 20.042 |
| genus | Dorea | rs11150408 | G | T | 0.049 | 0.011 | 7.06E-06 | 20.042 |
| genus |  | rs6570197 | C | T | 0.163 | 0.036 | 7.41E-06 | 20.042 |
| genus | Allisonella | rs34954690 | A | G | 0.134 | 0.030 | 7.89E-06 | 20.041 |
| genus | Coprococcus1 | rs12192614 | A | T | -0.049 | 0.011 | 8.02E-06 | 20.041 |
| genus | Anaerofilum | rs59929070 | A | G | 0.111 | 0.025 | 7.97E-06 | 20.040 |
| genus |  | rs4888188 | G | T | -0.053 | 0.012 | 7.74E-06 | 20.040 |
| genus | Bacteroides | rs1340391 | C | T | -0.059 | 0.013 | 6.73E-06 | 20.040 |
| genus | Butyrivibrio | rs6429094 | A | G | 0.171 | 0.038 | 8.79E-06 | 20.039 |
| genus | Lachnoclostridium | rs1031599 | T | G | -0.079 | 0.018 | 6.31E-06 | 20.039 |
| genus | Lactococcus | rs10424823 | G | A | 0.111 | 0.025 | 7.17E-06 | 20.039 |
| genus | Ruminiclostridium9 | rs6082461 | C | A | 0.059 | 0.013 | 4.87E-06 | 20.038 |
| genus | Gordonibacter | rs13033554 | T | C | 0.106 | 0.024 | 9.45E-06 | 20.038 |
| genus | Lactococcus | rs3960411 | T | C | 0.111 | 0.025 | 7.19E-06 | 20.038 |
| genus |  | rs965716 | A | G | 0.107 | 0.024 | 8.08E-06 | 20.038 |
| genus | LachnospiraceaeFCS020group | rs4680693 | A | T | 0.057 | 0.013 | 8.13E-06 | 20.037 |
| genus | Ruminiclostridium5 | rs2482037 | A | G | 0.050 | 0.011 | 7.05E-06 | 20.037 |
| genus | Holdemania | rs10885477 | C | T | -0.135 | 0.030 | 8.60E-06 | 20.037 |
| genus | Marvinbryantia | rs12289963 | G | C | 0.103 | 0.023 | 7.78E-06 | 20.037 |
| genus | Akkermansia | rs79837054 | T | C | 0.106 | 0.024 | 6.96E-06 | 20.037 |
| genus | CandidatusSoleaferrea | rs11071652 | G | C | -0.096 | 0.021 | 7.83E-06 | 20.036 |
| genus |  | rs13179441 | G | A | -0.091 | 0.020 | 6.49E-06 | 20.035 |
| genus |  | rs2884897 | G | A | -0.129 | 0.029 | 6.44E-06 | 20.034 |
| genus |  | rs12079579 | G | A | 0.096 | 0.021 | 5.04E-06 | 20.034 |
| genus | Coprococcus1 | rs946513 | T | C | 0.206 | 0.046 | 8.62E-06 | 20.034 |
| genus | Romboutsia | rs7120903 | T | G | -0.070 | 0.016 | 7.77E-06 | 20.033 |
| genus |  | rs559682 | G | A | 0.081 | 0.018 | 9.35E-06 | 20.032 |
| genus |  | rs2152497 | T | C | -0.053 | 0.012 | 6.86E-06 | 20.032 |
| genus | Dorea | rs3752849 | A | G | 0.164 | 0.037 | 7.68E-06 | 20.032 |
| genus | RuminococcaceaeUCG004 | rs872501 | A | G | 0.116 | 0.026 | 5.81E-06 | 20.031 |
| genus |  | rs6818880 | G | A | -0.110 | 0.025 | 7.83E-06 | 20.030 |
| genus | RikenellaceaeRC9gutgroup | rs7193937 | C | G | 0.124 | 0.028 | 6.19E-06 | 20.030 |
| genus | LachnospiraceaeUCG010 | rs72894975 | C | G | 0.218 | 0.049 | 8.73E-06 | 20.030 |
| genus | FamilyXIIIUCG001 | rs8076666 | G | A | 0.089 | 0.020 | 8.02E-06 | 20.029 |
| genus | Anaerostipes | rs1927482 | T | C | -0.049 | 0.011 | 8.44E-06 | 20.029 |
| genus | Escherichia | rs57024273 | C | T | 0.063 | 0.014 | 9.70E-06 | 20.028 |
| genus |  | rs16891896 | A | G | -0.175 | 0.039 | 2.38E-06 | 20.027 |
| genus | Lactococcus | rs4806166 | T | C | 0.111 | 0.025 | 7.25E-06 | 20.026 |
| genus | Ruminococcus1 | rs78572139 | A | G | 0.125 | 0.028 | 5.23E-06 | 20.026 |
| genus | Faecalibacterium | rs9536330 | C | T | -0.048 | 0.011 | 5.33E-06 | 20.026 |
| genus |  | rs1935864 | G | A | -0.159 | 0.036 | 8.03E-06 | 20.025 |
| genus |  | rs849162 | G | A | 0.065 | 0.015 | 8.00E-06 | 20.025 |
| genus | Lactobacillus | rs1434765 | C | A | -0.077 | 0.017 | 8.10E-06 | 20.024 |
| genus | Streptococcus | rs2440146 | T | C | -0.050 | 0.011 | 8.53E-06 | 20.024 |
| genus | Allisonella | rs12552101 | T | C | 0.136 | 0.030 | 8.10E-06 | 20.024 |
| genus | Ruminiclostridium6 | rs633372 | G | A | 0.055 | 0.012 | 7.94E-06 | 20.024 |
| genus |  | rs8141465 | G | A | 0.048 | 0.011 | 9.65E-06 | 20.023 |
| genus | RuminococcaceaeUCG014 | rs460442 | C | A | -0.057 | 0.013 | 9.82E-06 | 20.023 |
| genus | LachnospiraceaeNK4A136group | rs11030127 | G | A | 0.054 | 0.012 | 7.19E-06 | 20.023 |
| genus | Bifidobacterium | rs112084119 | C | T | 0.071 | 0.016 | 7.57E-06 | 20.023 |
| genus | Lactococcus | rs12712302 | A | G | 0.108 | 0.024 | 8.41E-06 | 20.022 |
| genus | Dialister | rs2257090 | C | T | 0.066 | 0.015 | 9.18E-06 | 20.022 |
| genus | Desulfovibrio | rs6758756 | C | T | 0.101 | 0.023 | 8.94E-06 | 20.021 |
| genus | LachnospiraceaeUCG008 | rs9836360 | G | A | -0.110 | 0.025 | 8.26E-06 | 20.020 |
| genus | RuminococcaceaeUCG004 | rs1003084 | G | T | 0.108 | 0.024 | 7.91E-06 | 20.020 |
| genus | LachnospiraceaeUCG004 | rs1020709 | G | A | -0.056 | 0.013 | 8.59E-06 | 20.020 |
| genus | Intestinibacter | rs10811556 | C | A | -0.104 | 0.023 | 6.70E-06 | 20.019 |
| genus | RuminococcaceaeUCG004 | rs80234555 | C | T | 0.123 | 0.028 | 7.89E-06 | 20.018 |
| genus | FamilyXIIIUCG001 | rs17800625 | C | G | -0.060 | 0.013 | 6.23E-06 | 20.018 |
| genus | Alloprevotella | rs2154444 | G | T | 0.138 | 0.031 | 8.37E-06 | 20.018 |
| genus |  | rs78928087 | C | A | -0.162 | 0.036 | 7.80E-06 | 20.018 |
| genus | Ruminococcus1 | rs7584526 | A | G | 0.050 | 0.011 | 7.12E-06 | 20.018 |
| genus | Subdoligranulum | rs61986650 | G | A | -0.049 | 0.011 | 9.44E-06 | 20.016 |
| genus | Roseburia | rs1416695 | A | G | 0.048 | 0.011 | 7.98E-06 | 20.016 |
| genus | Actinomyces | rs36009731 | G | A | 0.080 | 0.018 | 7.23E-06 | 20.016 |
| genus | RuminococcaceaeUCG010 | rs2773827 | G | A | -0.056 | 0.012 | 7.82E-06 | 20.016 |
| genus | FamilyXIIIAD3011group | rs35087346 | T | A | -0.087 | 0.019 | 8.39E-06 | 20.015 |
| genus | Parasutterella | rs13129739 | C | T | -0.064 | 0.014 | 8.76E-06 | 20.014 |
| genus | Fusicatenibacter | rs56037552 | T | C | -0.054 | 0.012 | 7.78E-06 | 20.014 |
| genus | Lachnoclostridium | rs10957054 | T | C | -0.051 | 0.011 | 7.62E-06 | 20.013 |
| genus |  | rs10458299 | C | T | -0.188 | 0.042 | 8.37E-06 | 20.013 |
| genus | Bifidobacterium | rs2686789 | C | A | 0.071 | 0.016 | 7.65E-06 | 20.013 |
| genus | RuminococcaceaeUCG004 | rs78968459 | G | A | 0.103 | 0.023 | 5.83E-06 | 20.013 |
| genus |  | rs7650657 | A | G | 0.050 | 0.011 | 9.18E-06 | 20.011 |
| genus | Anaerostipes | rs1590735 | G | T | -0.049 | 0.011 | 8.50E-06 | 20.011 |
| genus | Slackia | rs35156985 | C | T | -0.156 | 0.035 | 8.06E-06 | 20.010 |
| genus | RuminococcaceaeUCG004 | rs10976229 | G | T | 0.096 | 0.021 | 7.04E-06 | 20.010 |
| genus | Bifidobacterium | rs6728095 | A | T | 0.053 | 0.012 | 8.62E-06 | 20.010 |
| genus | Intestinibacter | rs10491567 | C | A | -0.104 | 0.023 | 6.85E-06 | 20.010 |
| genus |  | rs17107539 | C | T | -0.162 | 0.036 | 8.55E-06 | 20.010 |
| genus |  | rs10070691 | C | T | -0.091 | 0.020 | 6.43E-06 | 20.009 |
| genus | Eggerthella | rs1559479 | G | A | -0.105 | 0.023 | 8.56E-06 | 20.009 |
| genus | ChristensenellaceaeR | rs62468811 | G | A | 0.130 | 0.029 | 5.47E-06 | 20.009 |
| genus | Anaerostipes | rs11197346 | C | A | -0.050 | 0.011 | 9.18E-06 | 20.009 |
| genus | Dialister | rs517089 | A | T | 0.076 | 0.017 | 5.14E-06 | 20.009 |
| genus | Dorea | rs3005519 | A | G | 0.050 | 0.011 | 8.35E-06 | 20.008 |
| genus | Allisonella | rs7848439 | G | A | 0.138 | 0.031 | 8.68E-06 | 20.008 |
| genus | Bifidobacterium | rs13387501 | C | T | 0.063 | 0.014 | 4.25E-06 | 20.008 |
| genus | RikenellaceaeRC9gutgroup | rs10759383 | C | G | -0.126 | 0.028 | 8.05E-06 | 20.007 |
| genus | Subdoligranulum | rs4695413 | T | C | -0.052 | 0.012 | 6.42E-06 | 20.006 |
| genus | LachnospiraceaeUCG008 | rs9823432 | T | G | -0.110 | 0.025 | 8.30E-06 | 20.005 |
| genus | LachnospiraceaeND3007group | rs28552479 | A | G | -0.052 | 0.012 | 8.39E-06 | 20.005 |
| genus |  | rs57593136 | C | T | 0.079 | 0.018 | 7.79E-06 | 20.005 |
| genus | Prevotella9 | rs13261970 | G | C | -0.068 | 0.015 | 6.88E-06 | 20.005 |
| genus | RuminococcaceaeUCG009 | rs9558661 | C | T | -0.090 | 0.020 | 7.01E-06 | 20.004 |
| genus | Peptococcus | rs12110504 | C | T | 0.129 | 0.029 | 3.53E-06 | 20.004 |
| genus | Intestinimonas | rs6784625 | C | T | -0.059 | 0.013 | 8.94E-06 | 20.004 |
| genus | Ruminiclostridium5 | rs1223978 | C | T | 0.048 | 0.011 | 8.16E-06 | 20.003 |
| genus | Peptococcus | rs6573152 | G | A | 0.106 | 0.024 | 7.17E-06 | 20.003 |
| genus |  | rs11818152 | C | T | -0.113 | 0.025 | 8.12E-06 | 20.003 |
| genus | LachnospiraceaeFCS020group | rs10923439 | A | T | -0.054 | 0.012 | 8.43E-06 | 20.002 |
| genus |  | rs62024432 | T | C | -0.077 | 0.017 | 7.50E-06 | 20.002 |
| genus | RuminococcaceaeUCG005 | rs12273363 | T | C | 0.065 | 0.014 | 6.50E-06 | 20.001 |
| genus | Haemophilus | rs10840326 | G | C | -0.068 | 0.015 | 7.37E-06 | 20.001 |
| genus | FamilyXIIIUCG001 | rs112362903 | G | A | -0.149 | 0.033 | 7.88E-06 | 20.001 |
| genus | Turicibacter | rs10845476 | C | A | -0.095 | 0.021 | 6.47E-06 | 20.001 |
| genus |  | rs570794 | T | C | -0.048 | 0.011 | 8.02E-06 | 20.001 |
| genus | Haemophilus | rs40513 | T | G | 0.118 | 0.026 | 8.28E-06 | 20.000 |
| genus | Lactococcus | rs10402087 | G | C | 0.110 | 0.025 | 7.35E-06 | 20.000 |
| genus | LachnospiraceaeUCG001 | rs7616374 | T | C | 0.065 | 0.014 | 8.12E-06 | 19.999 |
| genus |  | rs1972694 | A | T | -0.061 | 0.014 | 8.93E-06 | 19.999 |
| genus | RuminococcaceaeNK4A214group | rs62027366 | C | T | 0.062 | 0.014 | 6.58E-06 | 19.998 |
| genus | LachnospiraceaeUCG010 | rs17730011 | A | G | -0.070 | 0.016 | 7.85E-06 | 19.998 |
| genus | Clostridiumsensustricto1 | rs116847295 | T | C | 0.110 | 0.025 | 4.58E-06 | 19.997 |
| genus | Allisonella | rs4743082 | T | G | 0.134 | 0.030 | 8.28E-06 | 19.997 |
| genus | Erysipelatoclostridium | rs2114978 | T | C | -0.066 | 0.015 | 8.29E-06 | 19.997 |
| genus |  | rs2973289 | G | A | 0.087 | 0.020 | 8.09E-06 | 19.996 |
| genus | Coprococcus2 | rs7266126 | C | T | 0.107 | 0.024 | 8.89E-06 | 19.995 |
| genus |  | rs1593084 | G | A | -0.141 | 0.031 | 4.74E-06 | 19.994 |
| genus | Streptococcus | rs3842931 | C | T | 0.096 | 0.021 | 7.59E-06 | 19.993 |
| genus | FamilyXIIIAD3011group | rs12820574 | A | G | -0.087 | 0.019 | 8.55E-06 | 19.992 |
| genus |  | rs77845139 | G | A | -0.115 | 0.026 | 8.41E-06 | 19.992 |
| genus | Lactococcus | rs10404086 | A | T | 0.110 | 0.025 | 7.35E-06 | 19.992 |
| genus | Tyzzerella3 | rs73291121 | A | G | -0.214 | 0.048 | 8.42E-06 | 19.991 |
| genus | Tyzzerella3 | rs112715294 | A | C | -0.214 | 0.048 | 8.42E-06 | 19.991 |
| genus | Tyzzerella3 | rs111287803 | T | C | -0.214 | 0.048 | 8.42E-06 | 19.991 |
| genus | Tyzzerella3 | rs113312668 | A | G | -0.214 | 0.048 | 8.42E-06 | 19.991 |
| genus | Coprococcus2 | rs6819699 | C | T | -0.062 | 0.014 | 8.85E-06 | 19.990 |
| genus | FamilyXIIIAD3011group | rs9837139 | G | A | 0.108 | 0.024 | 8.71E-06 | 19.990 |
| genus | LachnospiraceaeFCS020group | rs986977 | T | A | 0.054 | 0.012 | 8.73E-06 | 19.990 |
| genus | Ruminococcus1 | rs6750529 | T | C | 0.057 | 0.013 | 9.90E-06 | 19.989 |
| genus | Veillonella | rs34395919 | G | T | 0.074 | 0.017 | 7.92E-06 | 19.989 |
| genus | Ruminiclostridium5 | rs2482032 | G | A | 0.050 | 0.011 | 7.86E-06 | 19.988 |
| genus | Bifidobacterium | rs10180515 | T | A | 0.063 | 0.014 | 4.53E-06 | 19.987 |
| genus | Intestinibacter | rs9348442 | T | C | 0.099 | 0.022 | 6.26E-06 | 19.987 |
| genus | Veillonella | rs35668111 | C | T | 0.086 | 0.019 | 8.51E-06 | 19.987 |
| genus | LachnospiraceaeFCS020group | rs6771920 | T | A | 0.054 | 0.012 | 8.73E-06 | 19.987 |
| genus | Butyrivibrio | rs8064233 | T | C | 0.115 | 0.026 | 6.43E-06 | 19.986 |
| genus | LachnospiraceaeUCG008 | rs7110926 | A | G | -0.087 | 0.019 | 8.42E-06 | 19.986 |
| genus | Peptococcus | rs72850165 | C | T | -0.134 | 0.030 | 5.74E-06 | 19.985 |
| genus | Gordonibacter | rs35042269 | A | C | -0.180 | 0.040 | 8.11E-06 | 19.985 |
| genus | Marvinbryantia | rs3125831 | T | C | 0.067 | 0.015 | 6.55E-06 | 19.983 |
| genus | ErysipelotrichaceaeUCG003 | rs357893 | C | T | -0.060 | 0.014 | 7.19E-06 | 19.983 |
| genus | Veillonella | rs2831939 | T | C | -0.073 | 0.016 | 8.72E-06 | 19.982 |
| genus | Dialister | rs2314294 | C | T | 0.087 | 0.019 | 8.08E-06 | 19.981 |
| genus | RuminococcaceaeNK4A214group | rs4663856 | C | A | -0.051 | 0.012 | 8.39E-06 | 19.981 |
| genus | Coprobacter | rs1927591 | T | A | 0.078 | 0.017 | 8.11E-06 | 19.980 |
| genus | RuminococcaceaeNK4A214group | rs35829475 | C | T | -0.054 | 0.012 | 8.10E-06 | 19.980 |
| genus | Ruminococcus2 | rs61791565 | C | T | -0.052 | 0.012 | 6.79E-06 | 19.980 |
| genus | Oscillibacter | rs6925714 | C | T | -0.067 | 0.015 | 9.03E-06 | 19.979 |
| genus | Collinsella | rs1496626 | C | T | -0.072 | 0.016 | 6.78E-06 | 19.978 |
| genus | Terrisporobacter | rs58405430 | T | G | 0.135 | 0.030 | 7.94E-06 | 19.978 |
| genus | Turicibacter | rs117207382 | G | A | 0.099 | 0.022 | 7.71E-06 | 19.977 |
| genus | Erysipelatoclostridium | rs2003163 | G | A | -0.066 | 0.015 | 8.31E-06 | 19.977 |
| genus | Ruminiclostridium9 | rs9515633 | T | C | 0.069 | 0.015 | 5.79E-06 | 19.976 |
| genus | CandidatusSoleaferrea | rs7400877 | C | T | -0.095 | 0.021 | 9.29E-06 | 19.976 |
| genus | Anaerotruncus | rs17096167 | A | G | 0.056 | 0.013 | 8.46E-06 | 19.975 |
| genus | Barnesiella | rs6466817 | C | T | -0.054 | 0.012 | 8.33E-06 | 19.975 |
| genus | RuminococcaceaeUCG004 | rs3800155 | A | G | -0.079 | 0.018 | 6.85E-06 | 19.975 |
| genus | Lactococcus | rs6508728 | G | A | 0.110 | 0.025 | 7.45E-06 | 19.973 |
| genus | RuminococcaceaeUCG002 | rs6542556 | G | A | 0.051 | 0.011 | 7.86E-06 | 19.972 |
| genus | Veillonella | rs12815816 | A | T | 0.074 | 0.017 | 7.64E-06 | 19.972 |
| genus | Adlercreutzia | rs12522517 | T | A | -0.105 | 0.023 | 4.41E-06 | 19.972 |
| genus | Eisenbergiella | rs13264385 | C | T | 0.100 | 0.022 | 9.59E-06 | 19.971 |
| genus |  | rs2013977 | T | C | 0.048 | 0.011 | 6.43E-06 | 19.971 |
| genus | Butyricimonas | rs1872571 | C | T | 0.064 | 0.014 | 5.92E-06 | 19.971 |
| genus | Oscillibacter | rs73205327 | C | A | -0.081 | 0.018 | 7.80E-06 | 19.970 |
| genus | Sutterella | rs7988877 | T | A | -0.057 | 0.013 | 7.80E-06 | 19.969 |
| genus | LachnospiraceaeNC2004group | rs147584039 | C | T | -0.154 | 0.035 | 8.16E-06 | 19.969 |
| genus | RuminococcaceaeUCG014 | rs79640386 | A | T | -0.111 | 0.025 | 8.74E-06 | 19.968 |
| genus | Ruminiclostridium6 | rs965686 | T | C | 0.069 | 0.015 | 9.05E-06 | 19.968 |
| genus | Ruminiclostridium6 | rs11035745 | G | T | 0.069 | 0.015 | 7.87E-06 | 19.968 |
| genus |  | rs4682182 | C | T | -0.052 | 0.012 | 8.11E-06 | 19.967 |
| genus | Lactococcus | rs4806172 | T | C | 0.110 | 0.025 | 7.50E-06 | 19.965 |
| genus | Blautia | rs12669850 | C | A | 0.055 | 0.012 | 5.85E-06 | 19.965 |
| genus |  | rs10753292 | A | C | 0.057 | 0.013 | 8.01E-06 | 19.964 |
| genus | RuminococcaceaeUCG004 | rs12055694 | G | A | -0.078 | 0.017 | 7.11E-06 | 19.963 |
| genus | Intestinibacter | rs2614596 | T | C | 0.059 | 0.013 | 8.08E-06 | 19.961 |
| genus | Butyrivibrio | rs16934069 | C | T | -0.134 | 0.030 | 8.86E-06 | 19.961 |
| genus | FamilyXIIIUCG001 | rs17192223 | C | T | -0.060 | 0.013 | 6.71E-06 | 19.960 |
| genus | Coprococcus3 | rs2001666 | G | C | -0.065 | 0.015 | 9.73E-06 | 19.960 |
| genus | Odoribacter | rs16918425 | T | A | 0.100 | 0.022 | 8.85E-06 | 19.959 |
| genus | Slackia | rs77333555 | G | C | -0.117 | 0.026 | 7.13E-06 | 19.958 |
| genus | Coprobacter | rs10852138 | A | C | 0.098 | 0.022 | 8.36E-06 | 19.958 |
| genus | Romboutsia | rs7109293 | G | A | 0.092 | 0.021 | 6.98E-06 | 19.957 |
| genus | Odoribacter | rs80245222 | G | T | -0.120 | 0.027 | 8.44E-06 | 19.957 |
| genus | Lachnoclostridium | rs11996829 | G | T | -0.051 | 0.011 | 7.58E-06 | 19.957 |
| genus | Blautia | rs1529992 | T | G | 0.049 | 0.011 | 8.12E-06 | 19.957 |
| genus | Oxalobacter | rs6065016 | A | G | -0.095 | 0.021 | 8.53E-06 | 19.957 |
| genus | Anaerostipes | rs2420091 | A | G | -0.049 | 0.011 | 9.27E-06 | 19.956 |
| genus | Victivallis | rs1322595 | A | G | -0.110 | 0.025 | 9.33E-06 | 19.956 |
| genus | LachnospiraceaeUCG008 | rs7652397 | A | C | -0.099 | 0.022 | 7.31E-06 | 19.954 |
| genus | Butyrivibrio | rs17163231 | A | G | 0.138 | 0.031 | 8.56E-06 | 19.953 |
| genus | Parasutterella | rs56259060 | C | A | -0.066 | 0.015 | 8.27E-06 | 19.953 |
| genus | RuminococcaceaeUCG004 | rs1963969 | T | A | 0.103 | 0.023 | 5.03E-06 | 19.953 |
| genus | FamilyXIIIUCG001 | rs10498904 | C | A | -0.060 | 0.013 | 6.58E-06 | 19.953 |
| genus | Romboutsia | rs10091895 | C | T | -0.065 | 0.015 | 6.62E-06 | 19.952 |
| genus | Ruminococcus1 | rs10167839 | G | A | 0.052 | 0.012 | 8.09E-06 | 19.952 |
| genus | Parasutterella | rs6778417 | A | C | -0.066 | 0.015 | 8.28E-06 | 19.952 |
| genus | LachnospiraceaeND3007group | rs2549191 | C | G | -0.052 | 0.012 | 8.34E-06 | 19.952 |
| genus | Ruminiclostridium6 | rs1452471 | T | C | 0.061 | 0.014 | 6.15E-06 | 19.951 |
| genus | Oscillibacter | rs35650602 | C | A | -0.068 | 0.015 | 7.92E-06 | 19.951 |
| genus | Ruminiclostridium9 | rs74303178 | C | T | 0.053 | 0.012 | 7.92E-06 | 19.950 |
| genus | RuminococcaceaeUCG002 | rs12971317 | G | A | -0.052 | 0.012 | 6.81E-06 | 19.950 |
| genus | Sutterella | rs2050185 | A | G | 0.058 | 0.013 | 7.97E-06 | 19.950 |
| genus | RuminococcaceaeUCG002 | rs4413527 | C | T | -0.054 | 0.012 | 8.17E-06 | 19.950 |
| genus | Alistipes | rs4818001 | T | C | -0.048 | 0.011 | 8.05E-06 | 19.949 |
| genus | RuminococcaceaeUCG013 | rs2428106 | G | C | -0.049 | 0.011 | 8.38E-06 | 19.948 |
| genus | Adlercreutzia | rs9966313 | C | T | -0.095 | 0.021 | 8.21E-06 | 19.948 |
| genus | Butyrivibrio | rs6702967 | T | C | 0.176 | 0.039 | 9.74E-06 | 19.946 |
| genus | DefluviitaleaceaeUCG011 | rs28696126 | T | A | -0.107 | 0.024 | 6.63E-06 | 19.945 |
| genus |  | rs10826311 | G | A | -0.110 | 0.025 | 8.49E-06 | 19.945 |
| genus | Flavonifractor | rs7538430 | A | G | -0.068 | 0.015 | 6.79E-06 | 19.945 |
| genus |  | rs6550770 | C | T | -0.198 | 0.044 | 4.82E-06 | 19.945 |
| genus | Collinsella | rs59414781 | G | C | 0.067 | 0.015 | 9.15E-06 | 19.944 |
| genus | FamilyXIIIAD3011group | rs9876329 | C | T | 0.059 | 0.013 | 9.83E-06 | 19.943 |
| genus | Anaerofilum | rs10802455 | A | G | 0.093 | 0.021 | 8.66E-06 | 19.943 |
| genus | Turicibacter | rs10845477 | G | A | -0.095 | 0.021 | 6.71E-06 | 19.942 |
| genus | Coprobacter | rs62025078 | T | G | 0.099 | 0.022 | 8.98E-06 | 19.942 |
| genus | Coprococcus2 | rs12634070 | C | T | 0.074 | 0.016 | 9.95E-06 | 19.942 |
| genus | Olsenella | rs9460691 | A | C | 0.120 | 0.027 | 7.28E-06 | 19.941 |
| genus | Terrisporobacter | rs773921 | C | A | -0.083 | 0.019 | 8.91E-06 | 19.941 |
| genus | Peptococcus | rs6573149 | A | G | 0.106 | 0.024 | 7.39E-06 | 19.941 |
| genus | Lachnoclostridium | rs1997204 | C | T | -0.108 | 0.024 | 5.97E-06 | 19.941 |
| genus | Turicibacter | rs12899055 | C | T | 0.069 | 0.016 | 9.12E-06 | 19.941 |
| genus | Parasutterella | rs62273912 | C | T | 0.224 | 0.050 | 9.74E-06 | 19.939 |
| genus | Peptococcus | rs17762161 | G | C | 0.129 | 0.029 | 3.92E-06 | 19.939 |
| genus | Fusicatenibacter | rs792108 | C | T | -0.051 | 0.011 | 8.50E-06 | 19.939 |
| genus | Eggerthella | rs1738091 | A | T | 0.099 | 0.022 | 8.20E-06 | 19.938 |
| genus | Coprococcus1 | rs9360500 | C | A | -0.049 | 0.011 | 8.51E-06 | 19.938 |
| genus | Anaerotruncus | rs6494922 | G | A | 0.090 | 0.020 | 6.62E-06 | 19.938 |
| genus | Hungatella | rs72759041 | T | G | -0.126 | 0.028 | 3.86E-06 | 19.937 |
| genus | Ruminiclostridium6 | rs1433523 | C | A | -0.097 | 0.022 | 4.53E-06 | 19.937 |
| genus | RuminococcaceaeUCG009 | rs113006825 | C | T | -0.093 | 0.021 | 7.98E-06 | 19.937 |
| genus | Eisenbergiella | rs7946438 | C | G | 0.127 | 0.029 | 6.53E-06 | 19.937 |
| genus | Parasutterella | rs62273914 | G | T | 0.224 | 0.050 | 9.74E-06 | 19.936 |
| genus | Parasutterella | rs11720748 | C | A | -0.065 | 0.015 | 8.17E-06 | 19.936 |
| genus |  | rs951265 | G | A | -0.074 | 0.017 | 8.40E-06 | 19.936 |
| genus | FamilyXIIIUCG001 | rs62414800 | C | A | -0.060 | 0.013 | 6.21E-06 | 19.936 |
| genus |  | rs1377730 | C | T | -0.048 | 0.011 | 7.70E-06 | 19.935 |
| genus | Anaerostipes | rs6474958 | G | A | -0.050 | 0.011 | 6.74E-06 | 19.935 |
| genus | RuminococcaceaeUCG004 | rs9378684 | C | T | -0.079 | 0.018 | 7.34E-06 | 19.934 |
| genus | Anaerostipes | rs2532728 | A | C | -0.050 | 0.011 | 9.07E-06 | 19.934 |
| genus | FamilyXIIIAD3011group | rs61914226 | A | G | -0.088 | 0.020 | 6.89E-06 | 19.933 |
| genus | LachnospiraceaeFCS020group | rs1363769 | C | T | -0.201 | 0.045 | 1.58E-06 | 19.933 |
| genus |  | rs11988117 | T | G | 0.072 | 0.016 | 8.48E-06 | 19.933 |
| genus | Parasutterella | rs62273910 | A | G | 0.224 | 0.050 | 9.88E-06 | 19.932 |
| genus | ChristensenellaceaeR | rs4805124 | G | C | 0.049 | 0.011 | 8.27E-06 | 19.931 |
| genus | Hungatella | rs79291467 | T | C | -0.121 | 0.027 | 4.51E-06 | 19.931 |
| genus | Bifidobacterium | rs2176716 | C | T | 0.063 | 0.014 | 4.36E-06 | 19.931 |
| genus | Lachnospira | rs1441493 | C | A | -0.058 | 0.013 | 6.05E-06 | 19.931 |
| genus | Parasutterella | rs12254433 | G | C | -0.061 | 0.014 | 7.99E-06 | 19.931 |
| genus | Oscillibacter | rs4514587 | G | T | -0.068 | 0.015 | 8.05E-06 | 19.930 |
| genus | Anaerofilum | rs34258481 | G | A | 0.089 | 0.020 | 9.47E-06 | 19.930 |
| genus | Victivallis | rs4570771 | A | G | -0.117 | 0.026 | 9.27E-06 | 19.929 |
| genus | Alistipes | rs1115881 | G | A | -0.048 | 0.011 | 8.27E-06 | 19.929 |
| genus | ErysipelotrichaceaeUCG003 | rs8085780 | C | T | -0.094 | 0.021 | 7.31E-06 | 19.928 |
| genus |  | rs11818408 | A | G | 0.106 | 0.024 | 8.20E-06 | 19.928 |
| genus | Enterorhabdus | rs7765821 | G | A | -0.074 | 0.017 | 7.81E-06 | 19.927 |
| genus | Alistipes | rs4810359 | G | A | -0.065 | 0.015 | 7.50E-06 | 19.927 |
| genus | Eggerthella | rs740821 | G | C | -0.089 | 0.020 | 9.12E-06 | 19.925 |
| genus | Allisonella | rs34609282 | C | T | 0.134 | 0.030 | 9.42E-06 | 19.925 |
| genus |  | rs11157826 | C | T | -0.062 | 0.014 | 8.42E-06 | 19.925 |
| genus | Lachnoclostridium | rs13277801 | T | C | -0.051 | 0.011 | 8.06E-06 | 19.924 |
| genus | LachnospiraceaeFCS020group | rs9811284 | C | A | 0.054 | 0.012 | 9.03E-06 | 19.924 |
| genus | Bifidobacterium | rs35596805 | A | G | 0.070 | 0.016 | 8.10E-06 | 19.923 |
| genus | Lachnoclostridium | rs6982486 | A | G | -0.051 | 0.011 | 7.76E-06 | 19.923 |
| genus | CandidatusSoleaferrea | rs12140117 | A | C | 0.102 | 0.023 | 9.41E-06 | 19.922 |
| genus | ChristensenellaceaeR | rs62190261 | C | A | 0.096 | 0.021 | 8.74E-06 | 19.922 |
| genus | Sellimonas | rs12108049 | A | G | -0.160 | 0.036 | 8.95E-06 | 19.922 |
| genus | Erysipelatoclostridium | rs4302942 | A | G | -0.064 | 0.014 | 8.96E-06 | 19.921 |
| genus |  | rs8106205 | C | T | -0.047 | 0.011 | 7.76E-06 | 19.921 |
| genus | Anaerostipes | rs10961808 | C | T | 0.052 | 0.012 | 8.11E-06 | 19.920 |
| genus | Eisenbergiella | rs11079158 | C | T | 0.101 | 0.023 | 7.35E-06 | 19.920 |
| genus | Howardella | rs17529262 | C | G | -0.134 | 0.030 | 9.33E-06 | 19.920 |
| genus | LachnospiraceaeNC2004group | rs1929518 | T | C | 0.093 | 0.021 | 8.06E-06 | 19.919 |
| genus | Odoribacter | rs17781675 | G | A | -0.123 | 0.028 | 8.12E-06 | 19.918 |
| genus | Dorea | rs1370438 | C | A | 0.050 | 0.011 | 8.91E-06 | 19.918 |
| genus | LachnospiraceaeFCS020group | rs6770438 | T | C | 0.057 | 0.013 | 8.63E-06 | 19.917 |
| genus | Parasutterella | rs7170170 | T | C | 0.059 | 0.013 | 8.79E-06 | 19.917 |
| genus | FamilyXIIIUCG001 | rs17192279 | G | A | -0.060 | 0.013 | 7.87E-06 | 19.917 |
| genus | Eggerthella | rs4985746 | A | G | 0.111 | 0.025 | 5.71E-06 | 19.917 |
| genus | Eggerthella | rs6430926 | T | C | 0.088 | 0.020 | 8.37E-06 | 19.916 |
| genus | Butyricimonas | rs605188 | C | T | 0.062 | 0.014 | 8.24E-06 | 19.916 |
| genus | Akkermansia | rs4595371 | A | G | 0.063 | 0.014 | 8.32E-06 | 19.916 |
| genus | FamilyXIIIUCG001 | rs2295936 | C | T | -0.060 | 0.013 | 6.61E-06 | 19.916 |
| genus | Marvinbryantia | rs3991048 | T | G | 0.059 | 0.013 | 7.09E-06 | 19.915 |
| genus | Blautia | rs3005519 | A | G | 0.049 | 0.011 | 8.25E-06 | 19.915 |
| genus | Oxalobacter | rs7616207 | G | A | 0.103 | 0.023 | 8.48E-06 | 19.915 |
| genus | Ruminococcus1 | rs10169634 | C | T | 0.050 | 0.011 | 7.86E-06 | 19.914 |
| genus | Collinsella | rs11597285 | T | G | -0.054 | 0.012 | 9.38E-06 | 19.914 |
| genus | Lactococcus | rs4805130 | T | A | 0.110 | 0.025 | 7.26E-06 | 19.914 |
| genus | Anaerostipes | rs11197369 | A | G | -0.050 | 0.011 | 9.31E-06 | 19.913 |
| genus | FamilyXIIIAD3011group | rs11835648 | T | C | -0.077 | 0.017 | 8.76E-06 | 19.913 |
| genus | Odoribacter | rs4793970 | G | A | -0.058 | 0.013 | 6.03E-06 | 19.912 |
| genus | Butyrivibrio | rs465782 | G | A | -0.118 | 0.026 | 8.35E-06 | 19.912 |
| genus | Anaerostipes | rs11197340 | C | G | -0.049 | 0.011 | 9.34E-06 | 19.912 |
| genus | Fusicatenibacter | rs62187631 | C | T | -0.071 | 0.016 | 4.55E-06 | 19.912 |
| genus | Lactococcus | rs8109234 | T | C | 0.110 | 0.025 | 7.68E-06 | 19.911 |
| genus | Oscillospira | rs75570604 | G | C | 0.137 | 0.031 | 9.66E-06 | 19.911 |
| genus |  | rs9316536 | G | T | -0.082 | 0.018 | 7.84E-06 | 19.911 |
| genus | Lachnoclostridium | rs2016886 | C | T | -0.051 | 0.011 | 7.82E-06 | 19.911 |
| genus |  | rs13139592 | C | T | -0.146 | 0.033 | 7.97E-06 | 19.910 |
| genus |  | rs6437918 | C | T | -0.052 | 0.012 | 8.16E-06 | 19.910 |
| genus | Intestinibacter | rs893394 | A | G | 0.058 | 0.013 | 7.85E-06 | 19.910 |
| genus | Holdemania | rs967319 | C | T | 0.079 | 0.018 | 8.38E-06 | 19.910 |
| genus | Eggerthella | rs1615443 | A | G | 0.088 | 0.020 | 9.60E-06 | 19.909 |
| genus |  | rs62547233 | G | A | 0.054 | 0.012 | 9.90E-06 | 19.908 |
| genus |  | rs289417 | A | G | -0.063 | 0.014 | 7.87E-06 | 19.907 |
| genus | FamilyXIIIUCG001 | rs56006932 | C | G | -0.060 | 0.013 | 6.53E-06 | 19.907 |
| genus | Collinsella | rs2660968 | A | G | 0.067 | 0.015 | 9.68E-06 | 19.907 |
| genus | RuminococcaceaeUCG004 | rs9378319 | T | C | -0.078 | 0.017 | 7.42E-06 | 19.906 |
| genus | Collinsella | rs548708 | C | T | 0.054 | 0.012 | 8.40E-06 | 19.906 |
| genus | LachnospiraceaeUCG008 | rs29321 | C | T | 0.076 | 0.017 | 9.24E-06 | 19.906 |
| genus | Roseburia | rs28040 | C | G | 0.057 | 0.013 | 9.26E-06 | 19.906 |
| genus | LachnospiraceaeUCG004 | rs2444827 | G | A | -0.053 | 0.012 | 8.71E-06 | 19.905 |
| genus | Paraprevotella | rs1928142 | G | A | -0.076 | 0.017 | 9.27E-06 | 19.905 |
| genus |  | rs2323448 | A | T | 0.048 | 0.011 | 7.89E-06 | 19.905 |
| genus | RuminococcaceaeUCG003 | rs11778766 | C | A | -0.060 | 0.013 | 8.23E-06 | 19.905 |
| genus | Lactobacillus | rs1529819 | G | A | -0.077 | 0.017 | 8.62E-06 | 19.905 |
| genus | Eisenbergiella | rs2139516 | C | T | 0.127 | 0.029 | 6.75E-06 | 19.905 |
| genus | CandidatusSoleaferrea | rs12141016 | T | C | 0.102 | 0.023 | 9.13E-06 | 19.904 |
| genus | Alistipes | rs2297256 | G | A | -0.048 | 0.011 | 8.21E-06 | 19.904 |
| genus | Lactococcus | rs12973469 | T | A | 0.111 | 0.025 | 7.99E-06 | 19.903 |
| genus | Lactobacillus | rs7832262 | G | A | -0.077 | 0.017 | 8.58E-06 | 19.903 |
| genus | Akkermansia | rs12024893 | A | G | 0.063 | 0.014 | 8.42E-06 | 19.902 |
| genus | Oscillibacter | rs12879432 | C | T | -0.068 | 0.015 | 8.16E-06 | 19.902 |
| genus | Veillonella | rs7359080 | A | C | -0.135 | 0.030 | 7.40E-06 | 19.898 |
| genus | Butyrivibrio | rs9370258 | G | T | 0.119 | 0.027 | 8.07E-06 | 19.898 |
| genus | LachnospiraceaeFCS020group | rs2024534 | T | C | 0.054 | 0.012 | 9.16E-06 | 19.897 |
| genus | FamilyXIIIUCG001 | rs11755704 | G | A | -0.060 | 0.013 | 6.53E-06 | 19.897 |
| genus | RuminococcaceaeUCG002 | rs6793778 | T | C | -0.056 | 0.013 | 9.81E-06 | 19.896 |
| genus |  | rs78056098 | T | G | -0.051 | 0.011 | 8.29E-06 | 19.895 |
| genus | Faecalibacterium | rs11638158 | C | G | 0.049 | 0.011 | 7.49E-06 | 19.895 |
| genus | Eggerthella | rs13070736 | C | A | -0.121 | 0.027 | 7.62E-06 | 19.894 |
| genus | Veillonella | rs9615062 | T | G | -0.067 | 0.015 | 8.88E-06 | 19.894 |
| genus | Eisenbergiella | rs2133986 | C | T | 0.088 | 0.020 | 9.36E-06 | 19.892 |
| genus | Ruminiclostridium5 | rs12813694 | C | T | 0.051 | 0.012 | 7.58E-06 | 19.892 |
| genus | FamilyXIIIAD3011group | rs12303886 | G | A | -0.087 | 0.019 | 8.44E-06 | 19.892 |
| genus | Parabacteroides | rs2093835 | T | G | -0.064 | 0.014 | 6.09E-06 | 19.891 |
| genus | Romboutsia | rs114398731 | C | G | -0.131 | 0.029 | 7.96E-06 | 19.891 |
| genus |  | rs58433508 | A | G | -0.074 | 0.017 | 8.29E-06 | 19.891 |
| genus |  | rs28374963 | T | C | 0.088 | 0.020 | 5.07E-06 | 19.890 |
| genus | Lactococcus | rs3850347 | C | T | -0.175 | 0.039 | 3.97E-06 | 19.889 |
| genus | Blautia | rs67313756 | G | C | 0.053 | 0.012 | 6.07E-06 | 19.889 |
| genus | RuminococcaceaeUCG003 | rs13262757 | A | T | -0.060 | 0.013 | 8.34E-06 | 19.889 |
| genus | RuminococcaceaeUCG003 | rs7839273 | T | C | -0.059 | 0.013 | 9.92E-06 | 19.889 |
| genus | RuminococcaceaeUCG004 | rs12528102 | T | C | -0.079 | 0.018 | 9.43E-06 | 19.889 |
| genus | Peptococcus | rs991582 | C | T | 0.129 | 0.029 | 3.48E-06 | 19.888 |
| genus | RuminococcaceaeUCG003 | rs11778761 | C | A | -0.060 | 0.013 | 8.37E-06 | 19.888 |
| genus | Phascolarctobacterium | rs10168874 | A | G | 0.062 | 0.014 | 8.39E-06 | 19.887 |
| genus | LachnospiraceaeFCS020group | rs7989718 | G | T | -0.058 | 0.013 | 8.49E-06 | 19.887 |
| genus | LachnospiraceaeNK4A136group | rs301769 | T | C | 0.048 | 0.011 | 8.76E-06 | 19.886 |
| genus | Ruminiclostridium6 | rs16934480 | A | G | 0.069 | 0.015 | 8.12E-06 | 19.886 |
| genus | RikenellaceaeRC9gutgroup | rs7712231 | G | A | 0.156 | 0.035 | 7.97E-06 | 19.886 |
| genus | Ruminiclostridium5 | rs928338 | C | T | 0.050 | 0.011 | 7.56E-06 | 19.886 |
| genus | RuminococcaceaeUCG003 | rs2287921 | T | C | 0.053 | 0.012 | 9.91E-06 | 19.886 |
| genus | Allisonella | rs594561 | T | C | 0.112 | 0.025 | 9.41E-06 | 19.885 |
| genus | Tyzzerella3 | rs73291115 | A | G | -0.213 | 0.048 | 8.91E-06 | 19.884 |
| genus | Ruminiclostridium5 | rs2801958 | C | T | 0.051 | 0.012 | 8.44E-06 | 19.883 |
| genus | Fusicatenibacter | rs9730041 | C | A | -0.055 | 0.012 | 6.72E-06 | 19.883 |
| genus | Ruminococcus2 | rs2997408 | T | C | -0.055 | 0.012 | 9.86E-06 | 19.883 |
| genus | Oscillibacter | rs7101988 | T | A | 0.077 | 0.017 | 7.54E-06 | 19.881 |
| genus | Parasutterella | rs12500587 | T | G | -0.063 | 0.014 | 8.28E-06 | 19.880 |
| genus | Anaerofilum | rs10483366 | C | T | -0.123 | 0.028 | 9.11E-06 | 19.879 |
| genus | Bifidobacterium | rs2491158 | A | G | 0.071 | 0.016 | 8.05E-06 | 19.879 |
| genus | Dorea | rs62583469 | A | T | -0.063 | 0.014 | 5.78E-06 | 19.879 |
| genus | Lactococcus | rs7245626 | G | A | 0.107 | 0.024 | 8.51E-06 | 19.879 |
| genus | ChristensenellaceaeR | rs2188617 | T | C | 0.049 | 0.011 | 8.57E-06 | 19.879 |
| genus | Erysipelatoclostridium | rs113403148 | G | A | -0.105 | 0.024 | 7.98E-06 | 19.878 |
| genus | Coprobacter | rs7163000 | C | T | 0.098 | 0.022 | 8.68E-06 | 19.878 |
| genus | Lactococcus | rs7580916 | C | T | -0.192 | 0.043 | 3.00E-06 | 19.878 |
| genus |  | rs10444197 | G | A | -0.051 | 0.011 | 5.98E-06 | 19.877 |
| genus | Coprobacter | rs6496717 | T | C | 0.089 | 0.020 | 6.75E-06 | 19.876 |
| genus | Bilophila | rs1241179 | G | A | -0.071 | 0.016 | 8.75E-06 | 19.875 |
| genus | Enterorhabdus | rs77655283 | A | G | 0.133 | 0.030 | 5.88E-06 | 19.875 |
| genus | Lactococcus | rs12712303 | C | T | 0.107 | 0.024 | 9.08E-06 | 19.875 |
| genus |  | rs12117793 | T | A | 0.072 | 0.016 | 8.95E-06 | 19.874 |
| genus | FamilyXIIIUCG001 | rs62414804 | C | T | -0.060 | 0.013 | 8.35E-06 | 19.874 |
| genus | RikenellaceaeRC9gutgroup | rs1819327 | A | G | -0.125 | 0.028 | 8.46E-06 | 19.874 |
| genus | Intestinimonas | rs4113676 | C | A | -0.219 | 0.049 | 7.42E-06 | 19.873 |
| genus | Sellimonas | rs2554332 | G | C | 0.112 | 0.025 | 8.70E-06 | 19.873 |
| genus | Methanobrevibacter | rs1889567 | C | T | 0.114 | 0.026 | 9.66E-06 | 19.873 |
| genus | Akkermansia | rs36010840 | C | T | 0.063 | 0.014 | 8.56E-06 | 19.872 |
| genus | Bifidobacterium | rs12022129 | A | G | 0.062 | 0.014 | 8.00E-06 | 19.872 |
| genus | RuminococcaceaeUCG004 | rs73089465 | C | A | 0.090 | 0.020 | 8.99E-06 | 19.872 |
| genus | Erysipelatoclostridium | rs9652192 | A | C | 0.066 | 0.015 | 7.02E-06 | 19.872 |
| genus | RuminococcaceaeUCG009 | rs960892 | A | G | 0.078 | 0.017 | 8.68E-06 | 19.872 |
| genus | Tyzzerella3 | rs2612307 | C | A | 0.127 | 0.029 | 2.85E-06 | 19.872 |
| genus | LachnospiraceaeUCG008 | rs13089098 | G | A | -0.110 | 0.025 | 8.85E-06 | 19.871 |
| genus | Escherichia | rs590488 | C | T | -0.058 | 0.013 | 8.28E-06 | 19.869 |
| genus | RuminococcaceaeUCG004 | rs7569771 | G | A | -0.076 | 0.017 | 8.12E-06 | 19.869 |
| genus |  | rs12151423 | G | A | 0.101 | 0.023 | 9.27E-06 | 19.869 |
| genus | Desulfovibrio | rs7729080 | A | C | -0.070 | 0.016 | 9.96E-06 | 19.867 |
| genus | LachnospiraceaeUCG001 | rs12817350 | A | G | -0.076 | 0.017 | 9.70E-06 | 19.867 |
| genus | Eggerthella | rs2270169 | G | A | -0.089 | 0.020 | 9.14E-06 | 19.866 |
| genus | LachnospiraceaeUCG004 | rs12432325 | A | T | 0.054 | 0.012 | 9.50E-06 | 19.865 |
| genus | Coprococcus3 | rs38665 | T | C | -0.051 | 0.011 | 8.66E-06 | 19.865 |
| genus | Oscillibacter | rs34876895 | G | A | -0.068 | 0.015 | 7.88E-06 | 19.865 |
| genus | RuminococcaceaeUCG013 | rs9565219 | A | T | -0.052 | 0.012 | 8.73E-06 | 19.865 |
| genus | LachnospiraceaeFCS020group | rs9290283 | G | A | 0.054 | 0.012 | 9.31E-06 | 19.864 |
| genus | Blautia | rs2917871 | C | G | 0.049 | 0.011 | 8.47E-06 | 19.863 |
| genus | ErysipelotrichaceaeUCG003 | rs11666127 | G | A | -0.072 | 0.016 | 7.90E-06 | 19.863 |
| genus | Faecalibacterium | rs2175572 | G | A | 0.049 | 0.011 | 7.63E-06 | 19.862 |
| genus | LachnospiraceaeUCG010 | rs75252958 | G | A | 0.080 | 0.018 | 7.04E-06 | 19.862 |
| genus | Turicibacter | rs111885820 | G | A | 0.096 | 0.022 | 7.91E-06 | 19.862 |
| genus | RikenellaceaeRC9gutgroup | rs9299179 | G | C | -0.170 | 0.038 | 4.88E-06 | 19.862 |
| genus | RuminococcaceaeNK4A214group | rs6844250 | T | G | -0.055 | 0.012 | 8.32E-06 | 19.862 |
| genus |  | rs1778353 | C | T | 0.103 | 0.023 | 9.06E-06 | 19.861 |
| genus | LachnospiraceaeFCS020group | rs9848936 | T | G | 0.054 | 0.012 | 9.33E-06 | 19.861 |
| genus | Lachnoclostridium | rs4814838 | A | G | -0.048 | 0.011 | 9.73E-06 | 19.859 |
| genus | LachnospiraceaeFCS020group | rs9809054 | C | G | 0.057 | 0.013 | 8.88E-06 | 19.859 |
| genus | Prevotella9 | rs138897662 | G | A | -0.175 | 0.039 | 9.93E-06 | 19.859 |
| genus | Dorea | rs3005516 | G | A | 0.050 | 0.011 | 9.12E-06 | 19.858 |
| genus | Intestinibacter | rs4935946 | G | A | -0.132 | 0.030 | 9.15E-06 | 19.857 |
| genus | Ruminiclostridium9 | rs73154329 | G | A | -0.056 | 0.013 | 8.78E-06 | 19.856 |
| genus | LachnospiraceaeND3007group | rs8048165 | A | G | -0.052 | 0.012 | 8.47E-06 | 19.856 |
| genus | Ruminococcus1 | rs10734537 | G | C | -0.050 | 0.011 | 9.85E-06 | 19.855 |
| genus | Hungatella | rs35936785 | T | G | -0.151 | 0.034 | 9.23E-06 | 19.852 |
| genus | Alistipes | rs4498432 | G | C | -0.048 | 0.011 | 8.55E-06 | 19.851 |
| genus | RuminococcaceaeUCG005 | rs6674919 | G | A | 0.057 | 0.013 | 8.90E-06 | 19.851 |
| genus | Holdemanella | rs17586763 | C | T | -0.227 | 0.051 | 7.72E-06 | 19.850 |
| genus | Anaerofilum | rs34763136 | G | A | 0.089 | 0.020 | 9.39E-06 | 19.850 |
| genus | Tyzzerella3 | rs10898797 | T | C | 0.122 | 0.027 | 8.85E-06 | 19.850 |
| genus | RuminococcaceaeUCG003 | rs6985148 | A | T | -0.060 | 0.013 | 8.61E-06 | 19.850 |
| genus | Butyricimonas | rs1900181 | T | A | 0.064 | 0.014 | 8.21E-06 | 19.849 |
| genus |  | rs73199919 | C | T | -0.237 | 0.053 | 8.16E-06 | 19.848 |
| genus | Erysipelatoclostridium | rs2114977 | G | C | -0.066 | 0.015 | 8.99E-06 | 19.848 |
| genus | Marvinbryantia | rs6768572 | C | T | 0.061 | 0.014 | 8.26E-06 | 19.848 |
| genus | RuminococcaceaeUCG004 | rs2889331 | G | A | 0.118 | 0.027 | 6.82E-06 | 19.847 |
| genus | Bilophila | rs10974873 | T | C | 0.066 | 0.015 | 9.55E-06 | 19.846 |
| genus | LachnospiraceaeUCG004 | rs59151896 | T | A | 0.054 | 0.012 | 7.85E-06 | 19.846 |
| genus | Intestinibacter | rs9313841 | T | C | 0.087 | 0.020 | 6.29E-06 | 19.846 |
| genus | LachnospiraceaeUCG004 | rs8011680 | C | G | 0.056 | 0.013 | 9.75E-06 | 19.845 |
| genus | Turicibacter | rs41467648 | C | A | 0.096 | 0.022 | 7.86E-06 | 19.845 |
| genus |  | rs9425984 | C | T | -0.130 | 0.029 | 7.21E-06 | 19.845 |
| genus |  | rs720439 | G | A | -0.112 | 0.025 | 7.03E-06 | 19.845 |
| genus | CandidatusSoleaferrea | rs12500231 | T | A | 0.081 | 0.018 | 7.68E-06 | 19.844 |
| genus | Bifidobacterium | rs11684458 | A | G | 0.063 | 0.014 | 4.50E-06 | 19.844 |
| genus | Ruminiclostridium5 | rs2493767 | G | A | 0.050 | 0.011 | 7.78E-06 | 19.843 |
| genus |  | rs11044925 | T | C | -0.097 | 0.022 | 5.28E-06 | 19.842 |
| genus | LachnospiraceaeUCG010 | rs72894963 | G | A | 0.217 | 0.049 | 9.72E-06 | 19.842 |
| genus |  | rs3124075 | C | T | 0.055 | 0.012 | 8.04E-06 | 19.842 |
| genus | LachnospiraceaeNC2004group | rs6478407 | T | C | 0.093 | 0.021 | 8.20E-06 | 19.841 |
| genus | Anaerotruncus | rs9355485 | C | G | 0.049 | 0.011 | 7.65E-06 | 19.841 |
| genus | LachnospiraceaeUCG001 | rs8052586 | C | T | 0.176 | 0.040 | 4.02E-06 | 19.841 |
| genus | Veillonella | rs9759067 | G | A | 0.086 | 0.019 | 9.11E-06 | 19.840 |
| genus |  | rs13180887 | C | T | -0.090 | 0.020 | 7.29E-06 | 19.840 |
| genus | Anaerostipes | rs11197331 | A | G | -0.049 | 0.011 | 9.25E-06 | 19.840 |
| genus | Flavonifractor | rs12038887 | G | C | 0.094 | 0.021 | 9.37E-06 | 19.840 |
| genus | RuminococcaceaeUCG013 | rs9565218 | T | C | -0.053 | 0.012 | 9.63E-06 | 19.840 |
| genus | RuminococcaceaeUCG002 | rs3848208 | A | G | -0.057 | 0.013 | 7.38E-06 | 19.840 |
| genus | Victivallis | rs911673 | T | C | -0.117 | 0.026 | 9.69E-06 | 19.840 |
| genus | Phascolarctobacterium | rs7982713 | A | G | 0.073 | 0.016 | 9.72E-06 | 19.839 |
| genus | Holdemania | rs12855084 | T | A | -0.131 | 0.029 | 5.98E-06 | 19.839 |
| genus | Oscillibacter | rs12432377 | A | G | -0.068 | 0.015 | 8.54E-06 | 19.839 |
| genus |  | rs699512 | A | G | 0.065 | 0.015 | 8.70E-06 | 19.838 |
| genus |  | rs281379 | G | A | -0.050 | 0.011 | 9.33E-06 | 19.838 |
| genus | RuminococcaceaeUCG004 | rs73089420 | A | G | 0.075 | 0.017 | 9.60E-06 | 19.837 |
| genus | LachnospiraceaeUCG008 | rs57254474 | A | G | 0.089 | 0.020 | 6.92E-06 | 19.837 |
| genus | Senegalimassilia | rs4606909 | G | A | 0.076 | 0.017 | 8.67E-06 | 19.836 |
| genus | Desulfovibrio | rs72647089 | G | T | -0.107 | 0.024 | 8.30E-06 | 19.836 |
| genus | Enterorhabdus | rs10113034 | T | G | 0.131 | 0.029 | 8.33E-06 | 19.835 |
| genus | Butyrivibrio | rs6587085 | C | G | 0.115 | 0.026 | 7.26E-06 | 19.834 |
| genus | Butyricicoccus | rs7322368 | C | T | 0.082 | 0.018 | 5.52E-06 | 19.834 |
| genus | Anaerostipes | rs11197339 | G | A | -0.049 | 0.011 | 9.22E-06 | 19.833 |
| genus | RuminococcaceaeUCG004 | rs79746497 | A | G | -0.079 | 0.018 | 9.26E-06 | 19.833 |
| genus | Ruminiclostridium5 | rs4750263 | A | T | 0.049 | 0.011 | 7.79E-06 | 19.832 |
| genus | Butyricimonas | rs113040517 | C | G | -0.070 | 0.016 | 9.68E-06 | 19.832 |
| genus | Ruminiclostridium9 | rs9846988 | A | G | -0.050 | 0.011 | 8.25E-06 | 19.832 |
| genus | Akkermansia | rs72663769 | T | C | 0.066 | 0.015 | 7.03E-06 | 19.831 |
| genus | LachnospiraceaeFCS020group | rs1440673 | G | T | 0.054 | 0.012 | 9.50E-06 | 19.830 |
| genus |  | rs2797645 | A | C | -0.112 | 0.025 | 8.98E-06 | 19.830 |
| genus | Alistipes | rs10434981 | G | A | -0.049 | 0.011 | 9.05E-06 | 19.830 |
| genus | Paraprevotella | rs55906120 | G | A | -0.121 | 0.027 | 9.70E-06 | 19.830 |
| genus | Subdoligranulum | rs875423 | G | A | 0.048 | 0.011 | 8.59E-06 | 19.829 |
| genus | Faecalibacterium | rs61875484 | G | C | 0.082 | 0.018 | 9.18E-06 | 19.828 |
| genus | Roseburia | rs2935500 | A | T | 0.048 | 0.011 | 9.17E-06 | 19.828 |
| genus | Enterorhabdus | rs10097987 | A | G | 0.131 | 0.029 | 7.93E-06 | 19.827 |
| genus |  | rs571689 | C | T | -0.047 | 0.011 | 8.77E-06 | 19.827 |
| genus | Adlercreutzia | rs4557646 | C | T | 0.137 | 0.031 | 8.95E-06 | 19.826 |
| genus | Blautia | rs117001700 | C | T | 0.196 | 0.044 | 8.84E-06 | 19.826 |
| genus | Victivallis | rs911672 | A | G | -0.117 | 0.026 | 9.69E-06 | 19.826 |
| genus | LachnospiraceaeUCG008 | rs7932202 | T | C | -0.086 | 0.019 | 9.55E-06 | 19.824 |
| genus | FamilyXIIIAD3011group | rs34559641 | A | G | -0.087 | 0.020 | 8.70E-06 | 19.823 |
| genus | Butyrivibrio | rs3844555 | C | T | 0.137 | 0.031 | 8.51E-06 | 19.823 |
| genus | Eggerthella | rs1711399 | T | G | 0.088 | 0.020 | 9.66E-06 | 19.822 |
| genus | Ruminococcus1 | rs372099 | G | C | -0.072 | 0.016 | 8.02E-06 | 19.822 |
| genus | Romboutsia | rs9389266 | G | T | 0.072 | 0.016 | 9.38E-06 | 19.821 |
| genus | DefluviitaleaceaeUCG011 | rs6952956 | C | T | -0.108 | 0.024 | 8.66E-06 | 19.821 |
| genus | Intestinimonas | rs80092034 | G | A | -0.140 | 0.032 | 9.07E-06 | 19.821 |
| genus | Alistipes | rs10950094 | G | C | -0.049 | 0.011 | 9.34E-06 | 19.821 |
| genus | Bifidobacterium | rs4954559 | A | G | 0.061 | 0.014 | 9.07E-06 | 19.820 |
| genus | Eggerthella | rs1711434 | T | C | 0.088 | 0.020 | 9.65E-06 | 19.820 |
| genus | Bilophila | rs1261690 | C | T | -0.070 | 0.016 | 9.03E-06 | 19.820 |
| genus | Alistipes | rs10950089 | C | T | -0.049 | 0.011 | 9.09E-06 | 19.819 |
| genus | FamilyXIIIAD3011group | rs61914204 | A | G | -0.086 | 0.019 | 9.16E-06 | 19.819 |
| genus | Victivallis | rs12203075 | A | G | 0.110 | 0.025 | 9.40E-06 | 19.819 |
| genus | Butyrivibrio | rs1970324 | A | G | 0.115 | 0.026 | 7.39E-06 | 19.819 |
| genus | Alistipes | rs8118820 | A | T | -0.063 | 0.014 | 9.69E-06 | 19.819 |
| genus | Butyricimonas | rs2924725 | C | T | 0.068 | 0.015 | 8.42E-06 | 19.818 |
| genus | Lactococcus | rs12712304 | A | C | 0.107 | 0.024 | 9.86E-06 | 19.817 |
| genus | LachnospiraceaeNK4A136group | rs2880566 | C | T | 0.060 | 0.013 | 5.61E-06 | 19.815 |
| genus | Parasutterella | rs7910172 | T | C | -0.064 | 0.014 | 7.53E-06 | 19.815 |
| genus | Marvinbryantia | rs72948275 | T | G | -0.121 | 0.027 | 6.68E-06 | 19.815 |
| genus | Collinsella | rs11600657 | G | T | -0.053 | 0.012 | 8.95E-06 | 19.815 |
| genus | RuminococcaceaeUCG004 | rs7429237 | G | A | 0.075 | 0.017 | 9.55E-06 | 19.813 |
| genus | Lachnoclostridium | rs1006268 | C | T | -0.048 | 0.011 | 9.49E-06 | 19.813 |
| genus |  | rs10130880 | C | G | -0.053 | 0.012 | 8.21E-06 | 19.813 |
| genus | Ruminococcus1 | rs7570714 | C | T | 0.050 | 0.011 | 7.81E-06 | 19.813 |
| genus | Anaerostipes | rs72708371 | G | A | -0.105 | 0.024 | 7.25E-06 | 19.812 |
| genus | Senegalimassilia | rs11869966 | A | G | -0.079 | 0.018 | 8.47E-06 | 19.812 |
| genus | Escherichia | rs9850931 | T | A | -0.070 | 0.016 | 6.62E-06 | 19.811 |
| genus |  | rs58833244 | G | C | 0.067 | 0.015 | 9.42E-06 | 19.811 |
| genus | ChristensenellaceaeR | rs10425605 | T | C | 0.049 | 0.011 | 8.83E-06 | 19.811 |
| genus | Adlercreutzia | rs9915817 | C | T | 0.075 | 0.017 | 8.22E-06 | 19.810 |
| genus | DefluviitaleaceaeUCG011 | rs77041875 | C | T | 0.082 | 0.018 | 9.08E-06 | 19.808 |
| genus | Ruminiclostridium5 | rs10848557 | G | T | -0.048 | 0.011 | 1.00E-05 | 19.808 |
| genus | FamilyXIIIAD3011group | rs12821186 | G | T | -0.086 | 0.019 | 9.14E-06 | 19.808 |
| genus | Lachnoclostridium | rs6035302 | G | C | -0.048 | 0.011 | 9.02E-06 | 19.807 |
| genus |  | rs630939 | T | C | -0.051 | 0.011 | 9.16E-06 | 19.806 |
| genus | Coprobacter | rs6496711 | T | C | 0.098 | 0.022 | 8.44E-06 | 19.805 |
| genus | FamilyXIIIAD3011group | rs12831190 | G | T | -0.092 | 0.021 | 8.70E-06 | 19.805 |
| genus | Prevotella9 | rs2495052 | G | A | 0.084 | 0.019 | 8.97E-06 | 19.805 |
| genus | Alistipes | rs67853823 | C | T | -0.048 | 0.011 | 8.88E-06 | 19.804 |
| genus | Ruminiclostridium9 | rs17628988 | A | G | -0.056 | 0.013 | 9.06E-06 | 19.803 |
| genus | Anaerotruncus | rs9603191 | G | A | 0.074 | 0.017 | 4.05E-06 | 19.803 |
| genus | Bifidobacterium | rs41389446 | G | T | -0.082 | 0.018 | 8.22E-06 | 19.803 |
| genus | Lachnoclostridium | rs7268057 | T | C | -0.048 | 0.011 | 9.37E-06 | 19.802 |
| genus | Prevotella9 | rs2244976 | C | T | -0.067 | 0.015 | 7.25E-06 | 19.802 |
| genus | Dialister | rs12334717 | C | G | 0.059 | 0.013 | 7.08E-06 | 19.800 |
| genus |  | rs289379 | A | G | -0.063 | 0.014 | 7.94E-06 | 19.800 |
| genus | Sellimonas | rs10226023 | G | A | -0.120 | 0.027 | 7.84E-06 | 19.800 |
| genus | LachnospiraceaeUCG010 | rs72894979 | C | G | 0.217 | 0.049 | 9.85E-06 | 19.799 |
| genus | LachnospiraceaeUCG010 | rs72894978 | C | G | 0.217 | 0.049 | 9.85E-06 | 19.799 |
| genus | Oxalobacter | rs17543178 | A | G | -0.132 | 0.030 | 9.37E-06 | 19.798 |
| genus | Adlercreutzia | rs1534655 | T | C | -0.078 | 0.018 | 9.23E-06 | 19.797 |
| genus | LachnospiraceaeNK4A136group | rs10952110 | T | G | 0.049 | 0.011 | 9.08E-06 | 19.796 |
| genus | Subdoligranulum | rs17638405 | C | G | -0.060 | 0.013 | 7.49E-06 | 19.796 |
| genus | Akkermansia | rs4107636 | G | C | 0.063 | 0.014 | 7.47E-06 | 19.795 |
| genus | Parasutterella | rs4948778 | T | C | -0.064 | 0.014 | 7.67E-06 | 19.795 |
| genus | Butyrivibrio | rs8073494 | A | G | 0.115 | 0.026 | 7.12E-06 | 19.794 |
| genus | Victivallis | rs2104151 | C | G | -0.117 | 0.026 | 9.90E-06 | 19.793 |
| genus | Butyricicoccus | rs10084203 | G | A | 0.055 | 0.012 | 8.59E-06 | 19.791 |
| genus | LachnospiraceaeUCG008 | rs11025182 | A | G | -0.086 | 0.019 | 9.26E-06 | 19.791 |
| genus | Lachnoclostridium | rs983812 | T | C | -0.051 | 0.011 | 8.49E-06 | 19.790 |
| genus | Ruminiclostridium6 | rs10105231 | T | C | 0.055 | 0.012 | 9.09E-06 | 19.790 |
| genus | Coprococcus3 | rs6994742 | T | C | 0.055 | 0.012 | 7.84E-06 | 19.790 |
| genus | Sellimonas | rs10229922 | C | G | -0.120 | 0.027 | 8.06E-06 | 19.787 |
| genus | Romboutsia | rs16843578 | T | C | -0.088 | 0.020 | 5.08E-06 | 19.785 |
| genus | Coprococcus1 | rs7774681 | G | T | -0.049 | 0.011 | 9.12E-06 | 19.784 |
| genus | Subdoligranulum | rs11410065 | T | G | -0.052 | 0.012 | 7.21E-06 | 19.784 |
| genus | Enterorhabdus | rs7774871 | G | T | -0.074 | 0.017 | 8.58E-06 | 19.784 |
| genus | Bilophila | rs1969927 | A | G | 0.056 | 0.013 | 9.07E-06 | 19.783 |
| genus | Lachnoclostridium | rs1528479 | A | G | -0.050 | 0.011 | 9.64E-06 | 19.783 |
| genus | Odoribacter | rs150771252 | C | T | -0.120 | 0.027 | 9.40E-06 | 19.783 |
| genus | Ruminiclostridium6 | rs56212330 | G | A | 0.059 | 0.013 | 7.38E-06 | 19.783 |
| genus | Flavonifractor | rs7524151 | G | A | -0.067 | 0.015 | 7.22E-06 | 19.782 |
| genus | Slackia | rs13339230 | G | C | 0.147 | 0.033 | 7.42E-06 | 19.782 |
| genus | Intestinibacter | rs10790752 | C | G | -0.132 | 0.030 | 9.56E-06 | 19.781 |
| genus | Subdoligranulum | rs13124375 | G | T | -0.052 | 0.012 | 7.54E-06 | 19.781 |
| genus | RuminococcaceaeNK4A214group | rs11241747 | T | C | 0.053 | 0.012 | 6.59E-06 | 19.781 |
| genus | FamilyXIIIAD3011group | rs12829962 | C | T | -0.092 | 0.021 | 8.72E-06 | 19.781 |
| genus | Lachnoclostridium | rs7269490 | C | T | -0.052 | 0.012 | 6.65E-06 | 19.781 |
| genus | Coprobacter | rs4932361 | A | G | 0.098 | 0.022 | 9.10E-06 | 19.780 |
| genus | Prevotella7 | rs62115052 | G | T | 0.138 | 0.031 | 5.10E-06 | 19.780 |
| genus | LachnospiraceaeUCG010 | rs1558001 | C | T | -0.056 | 0.012 | 9.25E-06 | 19.779 |
| genus | Eggerthella | rs2877457 | A | G | -0.093 | 0.021 | 9.03E-06 | 19.779 |
| genus | Alistipes | rs4717397 | G | A | -0.049 | 0.011 | 9.29E-06 | 19.779 |
| genus | RuminococcaceaeUCG002 | rs7249614 | G | A | -0.049 | 0.011 | 9.07E-06 | 19.777 |
| genus | FamilyXIIIAD3011group | rs61914210 | A | G | -0.087 | 0.019 | 9.53E-06 | 19.776 |
| genus | Coprococcus3 | rs10810043 | G | A | 0.052 | 0.012 | 9.27E-06 | 19.776 |
| genus |  | rs2175594 | C | T | 0.048 | 0.011 | 8.99E-06 | 19.776 |
| genus | Akkermansia | rs4294419 | G | A | 0.063 | 0.014 | 8.32E-06 | 19.776 |
| genus |  | rs2271192 | T | C | 0.075 | 0.017 | 5.25E-06 | 19.775 |
| genus | RuminococcaceaeUCG004 | rs9405542 | C | T | -0.078 | 0.017 | 8.16E-06 | 19.775 |
| genus |  | rs11587400 | C | T | 0.086 | 0.019 | 6.37E-06 | 19.774 |
| genus | RuminococcaceaeUCG013 | rs7784330 | A | G | -0.050 | 0.011 | 8.16E-06 | 19.774 |
| genus |  | rs2190161 | C | T | -0.101 | 0.023 | 8.72E-06 | 19.773 |
| genus | Intestinibacter | rs7026731 | T | C | -0.102 | 0.023 | 5.80E-06 | 19.773 |
| genus | Holdemanella | rs62113418 | G | A | -0.103 | 0.023 | 8.69E-06 | 19.772 |
| genus | Alistipes | rs13246203 | A | G | -0.049 | 0.011 | 9.16E-06 | 19.771 |
| genus | FamilyXIIIAD3011group | rs6670314 | T | C | -0.055 | 0.012 | 9.14E-06 | 19.771 |
| genus | Anaerofilum | rs7516037 | A | T | 0.093 | 0.021 | 9.44E-06 | 19.771 |
| genus | RuminococcaceaeUCG013 | rs2730183 | A | G | -0.049 | 0.011 | 8.44E-06 | 19.770 |
| genus | Sellimonas | rs2570252 | G | T | 0.112 | 0.025 | 9.27E-06 | 19.769 |
| genus |  | rs4388134 | T | C | -0.090 | 0.020 | 9.12E-06 | 19.769 |
| genus | Adlercreutzia | rs1468569 | G | A | 0.072 | 0.016 | 8.86E-06 | 19.769 |
| genus |  | rs2058238 | T | C | 0.048 | 0.011 | 8.67E-06 | 19.768 |
| genus | Peptococcus | rs991583 | T | C | 0.128 | 0.029 | 3.71E-06 | 19.767 |
| genus | RikenellaceaeRC9gutgroup | rs4978853 | T | G | -0.170 | 0.038 | 5.23E-06 | 19.767 |
| genus | Butyricicoccus | rs141519424 | A | G | 0.075 | 0.017 | 6.67E-06 | 19.766 |
| genus | Streptococcus | rs7916067 | A | G | 0.095 | 0.021 | 9.13E-06 | 19.765 |
| genus | DefluviitaleaceaeUCG011 | rs35251672 | G | T | 0.079 | 0.018 | 9.71E-06 | 19.765 |
| genus | Erysipelatoclostridium | rs11744565 | T | C | -0.064 | 0.014 | 8.88E-06 | 19.765 |
| genus | Butyricimonas | rs1466576 | T | C | 0.063 | 0.014 | 6.74E-06 | 19.764 |
| genus |  | rs6577484 | A | G | 0.160 | 0.036 | 8.41E-06 | 19.764 |
| genus | Butyricimonas | rs4747116 | T | C | 0.064 | 0.014 | 6.73E-06 | 19.761 |
| genus | RuminococcaceaeUCG003 | rs11778780 | G | C | -0.059 | 0.013 | 8.85E-06 | 19.760 |
| genus | Ruminiclostridium9 | rs13086348 | C | A | -0.050 | 0.011 | 8.72E-06 | 19.760 |
| genus | Erysipelatoclostridium | rs61806970 | T | C | 0.143 | 0.032 | 9.09E-06 | 19.759 |
| genus | Allisonella | rs12378350 | T | C | 0.133 | 0.030 | 9.80E-06 | 19.757 |
| genus | Lachnospira | rs213581 | C | T | 0.079 | 0.018 | 8.22E-06 | 19.757 |
| genus | Butyrivibrio | rs56827 | T | C | -0.107 | 0.024 | 9.89E-06 | 19.756 |
| genus | Erysipelatoclostridium | rs2901723 | A | C | 0.064 | 0.014 | 8.79E-06 | 19.755 |
| genus | Eggerthella | rs1711441 | G | T | 0.088 | 0.020 | 9.93E-06 | 19.754 |
| genus | Enterorhabdus | rs6903990 | G | T | -0.074 | 0.017 | 9.12E-06 | 19.753 |
| genus | Ruminiclostridium6 | rs589368 | A | G | -0.147 | 0.033 | 4.59E-06 | 19.753 |
| genus | Lachnoclostridium | rs2179821 | T | G | -0.048 | 0.011 | 9.61E-06 | 19.752 |
| genus |  | rs78401243 | A | G | 0.078 | 0.017 | 3.29E-06 | 19.752 |
| genus | LachnospiraceaeFCS020group | rs7684654 | G | C | 0.054 | 0.012 | 8.89E-06 | 19.752 |
| genus | Streptococcus | rs78621406 | A | G | -0.083 | 0.019 | 8.74E-06 | 19.752 |
| genus |  | rs2644213 | A | G | 0.054 | 0.012 | 9.86E-06 | 19.752 |
| genus | Ruminiclostridium5 | rs1556409 | G | C | 0.050 | 0.011 | 8.82E-06 | 19.751 |
| genus | LachnospiraceaeUCG004 | rs984647 | T | G | 0.054 | 0.012 | 9.98E-06 | 19.751 |
| genus | Actinomyces | rs35110659 | G | T | 0.077 | 0.017 | 9.59E-06 | 19.751 |
| genus | LachnospiraceaeUCG001 | rs6956068 | A | C | -0.074 | 0.017 | 8.41E-06 | 19.750 |
| genus | Subdoligranulum | rs1599742 | G | A | 0.048 | 0.011 | 8.40E-06 | 19.750 |
| genus |  | rs139748 | A | G | -0.089 | 0.020 | 7.70E-06 | 19.750 |
| genus | Ruminococcus1 | rs729646 | C | A | -0.062 | 0.014 | 6.83E-06 | 19.750 |
| genus | Phascolarctobacterium | rs493998 | A | G | 0.061 | 0.014 | 9.18E-06 | 19.749 |
| genus | Gordonibacter | rs4596722 | G | A | 0.103 | 0.023 | 9.06E-06 | 19.748 |
| genus | Lactococcus | rs8105739 | C | G | 0.111 | 0.025 | 8.48E-06 | 19.748 |
| genus | Holdemania | rs4636956 | G | A | -0.067 | 0.015 | 6.71E-06 | 19.748 |
| genus | LachnospiraceaeFCS020group | rs4452603 | G | T | 0.060 | 0.014 | 8.98E-06 | 19.748 |
| genus | Fusicatenibacter | rs206581 | G | A | -0.057 | 0.013 | 8.96E-06 | 19.748 |
| genus |  | rs1020520 | G | T | -0.059 | 0.013 | 8.89E-06 | 19.747 |
| genus | Clostridiumsensustricto1 | rs146962933 | G | A | 0.109 | 0.025 | 5.63E-06 | 19.747 |
| genus | Peptococcus | rs62424017 | T | G | 0.128 | 0.029 | 3.84E-06 | 19.746 |
| genus | LachnospiraceaeFCS020group | rs13108212 | T | C | 0.055 | 0.012 | 8.78E-06 | 19.745 |
| genus | Catenibacterium | rs7795142 | G | T | 0.127 | 0.028 | 8.34E-06 | 19.744 |
| genus | Butyrivibrio | rs34435489 | T | C | -0.107 | 0.024 | 9.96E-06 | 19.744 |
| genus | Methanobrevibacter | rs11018665 | T | A | 0.113 | 0.025 | 7.03E-06 | 19.743 |
| genus | Dorea | rs1595344 | G | A | 0.050 | 0.011 | 9.58E-06 | 19.743 |
| genus | Ruminiclostridium9 | rs9811346 | T | C | -0.050 | 0.011 | 9.21E-06 | 19.743 |
| genus | Sellimonas | rs77979921 | T | A | -0.120 | 0.027 | 8.23E-06 | 19.743 |
| genus | Dialister | rs764177 | A | C | -0.060 | 0.014 | 9.61E-06 | 19.742 |
| genus | Prevotella7 | rs12195431 | C | T | 0.197 | 0.044 | 8.73E-06 | 19.741 |
| genus | Ruminiclostridium6 | rs1871858 | G | C | -0.105 | 0.024 | 9.12E-06 | 19.740 |
| genus | ErysipelotrichaceaeUCG003 | rs12251396 | G | A | -0.071 | 0.016 | 9.52E-06 | 19.740 |
| genus | Coprococcus2 | rs11099043 | T | A | 0.064 | 0.014 | 8.60E-06 | 19.739 |
| genus | Erysipelatoclostridium | rs16936671 | T | C | -0.097 | 0.022 | 6.04E-06 | 19.739 |
| genus | Intestinimonas | rs72895940 | A | G | -0.140 | 0.032 | 9.33E-06 | 19.739 |
| genus | Bifidobacterium | rs4954218 | T | G | -0.061 | 0.014 | 2.72E-06 | 19.738 |
| genus | RuminococcaceaeUCG014 | rs62356877 | T | C | -0.116 | 0.026 | 7.90E-06 | 19.737 |
| genus | Butyrivibrio | rs8068101 | T | C | 0.114 | 0.026 | 7.30E-06 | 19.736 |
| genus | Fusicatenibacter | rs73103914 | G | A | -0.060 | 0.013 | 8.30E-06 | 19.736 |
| genus | Bifidobacterium | rs13399952 | G | A | 0.053 | 0.012 | 9.88E-06 | 19.736 |
| genus | Ruminococcus1 | rs11686947 | G | A | 0.050 | 0.011 | 8.87E-06 | 19.735 |
| genus |  | rs11667321 | G | A | -0.047 | 0.011 | 8.52E-06 | 19.735 |
| genus | LachnospiraceaeUCG008 | rs29302 | C | T | 0.076 | 0.017 | 9.08E-06 | 19.735 |
| genus | Holdemania | rs6762641 | T | C | 0.078 | 0.018 | 9.52E-06 | 19.734 |
| genus | Enterorhabdus | rs6904374 | C | T | -0.074 | 0.017 | 9.19E-06 | 19.733 |
| genus | Ruminiclostridium9 | rs9515631 | T | C | 0.069 | 0.015 | 6.60E-06 | 19.732 |
| genus | Coprococcus2 | rs7685943 | G | A | 0.072 | 0.016 | 6.80E-06 | 19.731 |
| genus | LachnospiraceaeUCG004 | rs868697 | C | A | 0.100 | 0.023 | 8.80E-06 | 19.731 |
| genus |  | rs13234419 | C | T | 0.064 | 0.014 | 9.29E-06 | 19.730 |
| genus | RuminococcaceaeUCG013 | rs10225502 | A | C | -0.050 | 0.011 | 8.27E-06 | 19.730 |
| genus | Dorea | rs3005514 | T | G | 0.050 | 0.011 | 9.75E-06 | 19.729 |
| genus | Ruminiclostridium6 | rs73176030 | C | T | 0.059 | 0.013 | 7.29E-06 | 19.728 |
| genus | Ruminococcus2 | rs12406309 | C | A | -0.063 | 0.014 | 9.79E-06 | 19.728 |
| genus | LachnospiraceaeNK4A136group | rs1564382 | G | A | 0.048 | 0.011 | 9.86E-06 | 19.728 |
| genus | Dorea | rs12451419 | G | A | 0.048 | 0.011 | 9.84E-06 | 19.727 |
| genus | Terrisporobacter | rs12462569 | T | C | -0.078 | 0.018 | 9.45E-06 | 19.727 |
| genus | Enterorhabdus | rs2051957 | T | C | 0.084 | 0.019 | 8.90E-06 | 19.727 |
| genus | Peptococcus | rs17829549 | T | C | 0.128 | 0.029 | 4.24E-06 | 19.727 |
| genus | LachnospiraceaeFCS020group | rs9290282 | G | A | 0.054 | 0.012 | 9.95E-06 | 19.727 |
| genus | RuminococcaceaeUCG014 | rs6430495 | T | A | 0.055 | 0.012 | 7.92E-06 | 19.726 |
| genus | RuminococcaceaeUCG004 | rs72830173 | A | G | -0.078 | 0.018 | 9.79E-06 | 19.726 |
| genus | Dorea | rs1437569 | A | G | 0.050 | 0.011 | 9.80E-06 | 19.725 |
| genus | Lactococcus | rs3972333 | C | T | 0.111 | 0.025 | 8.70E-06 | 19.725 |
| genus | Dialister | rs1601589 | C | T | 0.101 | 0.023 | 7.06E-06 | 19.725 |
| genus | RikenellaceaeRC9gutgroup | rs2095094 | A | G | -0.134 | 0.030 | 9.47E-06 | 19.725 |
| genus | Slackia | rs347677 | C | T | 0.083 | 0.019 | 9.36E-06 | 19.724 |
| genus |  | rs73109677 | G | A | -0.077 | 0.017 | 8.77E-06 | 19.723 |
| genus | LachnospiraceaeUCG001 | rs7213933 | A | T | -0.082 | 0.018 | 9.02E-06 | 19.723 |
| genus | Romboutsia | rs16843588 | T | G | -0.088 | 0.020 | 5.40E-06 | 19.723 |
| genus | Terrisporobacter | rs2569953 | C | A | -0.078 | 0.017 | 8.95E-06 | 19.723 |
| genus | Erysipelatoclostridium | rs9595383 | G | A | 0.066 | 0.015 | 7.35E-06 | 19.722 |
| genus | Blautia | rs12668576 | C | A | 0.055 | 0.012 | 8.46E-06 | 19.722 |
| genus | LachnospiraceaeNK4A136group | rs301772 | C | T | 0.048 | 0.011 | 9.82E-06 | 19.721 |
| genus | Terrisporobacter | rs7184125 | C | T | 0.091 | 0.021 | 8.48E-06 | 19.721 |
| genus | Anaerofilum | rs13417431 | A | G | 0.110 | 0.025 | 8.66E-06 | 19.721 |
| genus |  | rs10987693 | C | T | -0.103 | 0.023 | 8.86E-06 | 19.720 |
| genus | Allisonella | rs538601 | G | A | 0.112 | 0.025 | 9.83E-06 | 19.720 |
| genus | Coprococcus2 | rs34042862 | G | T | -0.065 | 0.015 | 9.34E-06 | 19.718 |
| genus | Ruminiclostridium9 | rs17628958 | G | C | -0.056 | 0.013 | 9.52E-06 | 19.717 |
| genus | Prevotella9 | rs2104588 | C | T | 0.106 | 0.024 | 8.13E-06 | 19.716 |
| genus | Bifidobacterium | rs55912948 | A | G | 0.054 | 0.012 | 9.32E-06 | 19.716 |
| genus | Prevotella9 | rs4968431 | T | G | 0.064 | 0.014 | 8.58E-06 | 19.716 |
| genus |  | rs3862040 | C | T | 0.048 | 0.011 | 9.26E-06 | 19.714 |
| genus | Lachnospira | rs2520511 | T | C | 0.051 | 0.012 | 9.08E-06 | 19.714 |
| genus | RuminococcaceaeUCG004 | rs6545749 | C | G | 0.065 | 0.015 | 9.71E-06 | 19.714 |
| genus |  | . | A | C | 0.088 | 0.020 | 5.94E-06 | 19.714 |
| genus | Erysipelatoclostridium | rs16936674 | G | T | -0.097 | 0.022 | 7.14E-06 | 19.713 |
| genus | Ruminiclostridium6 | rs72848191 | C | T | -0.096 | 0.022 | 4.67E-06 | 19.713 |
| genus | Subdoligranulum | rs2605243 | A | G | -0.051 | 0.012 | 7.55E-06 | 19.713 |
| genus | Prevotella7 | rs9426434 | C | T | -0.124 | 0.028 | 9.72E-06 | 19.713 |
| genus | CandidatusSoleaferrea | rs12681352 | C | T | -0.088 | 0.020 | 9.98E-06 | 19.712 |
| genus | Holdemania | rs11080063 | A | G | -0.067 | 0.015 | 6.67E-06 | 19.711 |
| genus | LachnospiraceaeFCS020group | rs7684645 | G | A | 0.054 | 0.012 | 8.88E-06 | 19.710 |
| genus | Bifidobacterium | rs842356 | A | G | 0.073 | 0.016 | 8.51E-06 | 19.710 |
| genus | Akkermansia | rs72663751 | G | A | 0.065 | 0.015 | 7.55E-06 | 19.710 |
| genus | RuminococcaceaeUCG013 | rs652806 | C | A | -0.049 | 0.011 | 9.68E-06 | 19.707 |
| genus | Gordonibacter | rs61934597 | T | C | -0.172 | 0.039 | 8.37E-06 | 19.707 |
| genus | RuminococcaceaeUCG013 | rs523167 | T | G | -0.049 | 0.011 | 9.67E-06 | 19.706 |
| genus | Subdoligranulum | rs2572343 | T | C | -0.051 | 0.012 | 7.71E-06 | 19.704 |
| genus | Bifidobacterium | rs5746486 | C | T | -0.054 | 0.012 | 9.00E-06 | 19.703 |
| genus | Streptococcus | rs76717940 | A | T | 0.150 | 0.034 | 4.34E-06 | 19.703 |
| genus | LachnospiraceaeNK4A136group | rs169973 | T | C | 0.048 | 0.011 | 9.52E-06 | 19.702 |
| genus | Ruminiclostridium5 | rs914325 | C | T | 0.050 | 0.011 | 9.11E-06 | 19.702 |
| genus | Lachnoclostridium | rs13263105 | G | C | -0.050 | 0.011 | 8.75E-06 | 19.702 |
| genus | Oscillibacter | rs1150668 | T | G | -0.067 | 0.015 | 9.16E-06 | 19.701 |
| genus | Ruminococcus1 | rs7584523 | A | T | 0.050 | 0.011 | 9.03E-06 | 19.700 |
| genus | Collinsella | rs12921100 | T | A | 0.056 | 0.013 | 8.23E-06 | 19.699 |
| genus | RikenellaceaeRC9gutgroup | rs12501673 | G | A | 0.116 | 0.026 | 6.29E-06 | 19.698 |
| genus | Victivallis | rs2546432 | C | T | -0.111 | 0.025 | 9.93E-06 | 19.698 |
| genus | Roseburia | rs12412854 | C | G | 0.047 | 0.011 | 9.50E-06 | 19.697 |
| genus | Coprobacter | rs8041597 | T | G | 0.097 | 0.022 | 9.14E-06 | 19.696 |
| genus | Lachnoclostridium | rs1030431 | G | A | -0.051 | 0.011 | 7.93E-06 | 19.696 |
| genus | Akkermansia | rs736124 | T | A | -0.074 | 0.017 | 8.29E-06 | 19.696 |
| genus | Collinsella | rs2831291 | G | A | 0.074 | 0.017 | 8.59E-06 | 19.695 |
| genus | Olsenella | rs6035503 | A | T | 0.121 | 0.027 | 8.04E-06 | 19.694 |
| genus | Parasutterella | rs4948777 | T | C | -0.064 | 0.014 | 8.06E-06 | 19.694 |
| genus | Peptococcus | rs428521 | A | C | -0.195 | 0.044 | 9.05E-06 | 19.693 |
| genus | Akkermansia | rs72663802 | C | T | 0.065 | 0.015 | 8.50E-06 | 19.692 |
| genus | Enterorhabdus | rs7753616 | G | A | -0.074 | 0.017 | 9.01E-06 | 19.690 |
| genus | RuminococcaceaeUCG002 | rs2073705 | C | T | -0.056 | 0.013 | 6.64E-06 | 19.690 |
| genus |  | rs2229046 | T | C | 0.090 | 0.020 | 7.87E-06 | 19.690 |
| genus |  | rs6861651 | T | C | -0.090 | 0.020 | 8.07E-06 | 19.689 |
| genus | RuminococcaceaeUCG010 | rs3088274 | C | T | 0.061 | 0.014 | 7.82E-06 | 19.689 |
| genus | Peptococcus | rs72850167 | C | T | -0.133 | 0.030 | 7.01E-06 | 19.689 |
| genus | ErysipelotrichaceaeUCG003 | rs79396538 | G | C | 0.085 | 0.019 | 8.63E-06 | 19.688 |
| genus |  | rs8020950 | G | C | -0.053 | 0.012 | 8.80E-06 | 19.688 |
| genus | Slackia | rs79393841 | G | A | -0.117 | 0.026 | 8.37E-06 | 19.687 |
| genus | LachnospiraceaeUCG010 | rs1408275 | T | A | -0.062 | 0.014 | 8.05E-06 | 19.687 |
| genus |  | rs1304116 | T | C | 0.120 | 0.027 | 9.87E-06 | 19.687 |
| genus | Akkermansia | rs4600885 | A | C | 0.063 | 0.014 | 7.76E-06 | 19.686 |
| genus | Sutterella | rs9377065 | G | C | 0.057 | 0.013 | 9.16E-06 | 19.686 |
| genus | Butyricicoccus | rs4129082 | G | C | 0.055 | 0.012 | 9.18E-06 | 19.686 |
| genus | Fusicatenibacter | . | A | T | -0.058 | 0.013 | 5.87E-06 | 19.685 |
| genus | Escherichia | rs117092367 | T | A | 0.117 | 0.026 | 9.65E-06 | 19.683 |
| genus | Peptococcus | rs12351718 | G | A | 0.085 | 0.019 | 9.41E-06 | 19.682 |
| genus | Alistipes | rs2150413 | C | T | -0.048 | 0.011 | 9.23E-06 | 19.681 |
| genus | RuminococcaceaeUCG003 | rs3013098 | A | G | -0.054 | 0.012 | 8.96E-06 | 19.681 |
| genus | Coprobacter | rs5011651 | A | G | 0.089 | 0.020 | 7.43E-06 | 19.681 |
| genus | Clostridiumsensustricto1 | rs11181871 | T | C | 0.109 | 0.025 | 5.36E-06 | 19.679 |
| genus | ChristensenellaceaeR | rs73952017 | T | C | -0.086 | 0.019 | 8.46E-06 | 19.679 |
| genus | Odoribacter | rs491296 | C | A | 0.052 | 0.012 | 9.01E-06 | 19.678 |
| genus | Erysipelatoclostridium | rs2362838 | G | C | -0.066 | 0.015 | 9.82E-06 | 19.677 |
| genus | LachnospiraceaeUCG004 | rs12202257 | A | G | -0.055 | 0.012 | 7.03E-06 | 19.677 |
| genus | Dorea | rs12537781 | C | T | -0.056 | 0.013 | 9.15E-06 | 19.675 |
| genus | Eisenbergiella | rs113812270 | C | T | 0.131 | 0.030 | 6.31E-06 | 19.674 |
| genus | Collinsella | rs2094864 | G | T | 0.074 | 0.017 | 6.61E-06 | 19.673 |
| genus | Odoribacter | rs2899604 | T | A | 0.052 | 0.012 | 9.17E-06 | 19.673 |
| genus | Allisonella | rs76904847 | A | G | 0.149 | 0.033 | 6.09E-06 | 19.673 |
| genus | Lachnoclostridium | rs7005978 | G | A | -0.050 | 0.011 | 8.79E-06 | 19.672 |
| genus |  | rs10054585 | A | C | -0.090 | 0.020 | 7.89E-06 | 19.672 |
| genus |  | rs10054604 | A | C | -0.090 | 0.020 | 7.89E-06 | 19.672 |
| genus | Prevotella7 | rs55795557 | G | A | 0.163 | 0.037 | 7.68E-06 | 19.672 |
| genus | Bifidobacterium | rs6894830 | C | T | -0.094 | 0.021 | 8.00E-06 | 19.671 |
| genus | RikenellaceaeRC9gutgroup | rs61860639 | G | A | 0.112 | 0.025 | 8.41E-06 | 19.670 |
| genus | ErysipelotrichaceaeUCG003 | rs73311093 | C | T | 0.120 | 0.027 | 8.18E-06 | 19.670 |
| genus | Lachnoclostridium | rs62028349 | C | G | 0.047 | 0.011 | 9.17E-06 | 19.670 |
| genus | Ruminiclostridium5 | rs2801961 | A | G | 0.051 | 0.012 | 9.51E-06 | 19.670 |
| genus | Intestinibacter | rs76991165 | G | A | -0.103 | 0.023 | 3.71E-06 | 19.667 |
| genus |  | rs10798998 | C | G | 0.058 | 0.013 | 9.50E-06 | 19.667 |
| genus |  | rs16868806 | C | G | 0.102 | 0.023 | 6.32E-06 | 19.667 |
| genus | Fusicatenibacter | rs2095811 | G | A | -0.051 | 0.012 | 8.42E-06 | 19.666 |
| genus | RikenellaceaeRC9gutgroup | rs11597199 | C | T | 0.112 | 0.025 | 8.24E-06 | 19.666 |
| genus | RuminococcaceaeUCG003 | rs3124989 | T | C | -0.054 | 0.012 | 8.95E-06 | 19.666 |
| genus | Butyricimonas | rs72814525 | G | A | 0.066 | 0.015 | 8.25E-06 | 19.666 |
| genus | Coprococcus1 | rs9351863 | G | C | -0.049 | 0.011 | 9.78E-06 | 19.664 |
| genus | ErysipelotrichaceaeUCG003 | rs60664481 | C | T | 0.120 | 0.027 | 7.82E-06 | 19.662 |
| genus | Ruminiclostridium9 | rs726404 | C | T | 0.058 | 0.013 | 5.92E-06 | 19.662 |
| genus |  | rs6704822 | G | A | 0.074 | 0.017 | 6.62E-06 | 19.661 |
| genus |  | rs13082419 | T | C | -0.072 | 0.016 | 9.56E-06 | 19.661 |
| genus | Dialister | rs4725397 | C | T | 0.065 | 0.015 | 9.71E-06 | 19.660 |
| genus |  | rs73746881 | C | T | 0.110 | 0.025 | 9.42E-06 | 19.660 |
| genus | RuminococcaceaeUCG003 | rs4330642 | T | C | -0.059 | 0.013 | 9.67E-06 | 19.659 |
| genus | Bacteroides | rs17788984 | A | G | 0.075 | 0.017 | 8.47E-06 | 19.658 |
| genus | RuminococcaceaeUCG005 | rs7586445 | A | G | 0.078 | 0.018 | 8.81E-06 | 19.657 |
| genus | Subdoligranulum | rs4695414 | G | A | -0.051 | 0.012 | 7.70E-06 | 19.657 |
| genus | Fusicatenibacter | rs167879 | T | C | -0.066 | 0.015 | 5.87E-06 | 19.656 |
| genus | Parasutterella | rs7133267 | T | C | -0.072 | 0.016 | 9.71E-06 | 19.654 |
| genus |  | rs1409398 | A | T | 0.102 | 0.023 | 9.16E-06 | 19.654 |
| genus | Peptococcus | rs2346471 | A | G | 0.105 | 0.024 | 8.80E-06 | 19.651 |
| genus | RuminococcaceaeUCG003 | rs3013102 | G | A | -0.054 | 0.012 | 9.16E-06 | 19.651 |
| genus | Ruminiclostridium9 | rs6047486 | G | A | 0.058 | 0.013 | 5.92E-06 | 19.651 |
| genus | RuminococcaceaeUCG014 | rs6754184 | A | G | 0.057 | 0.013 | 7.73E-06 | 19.650 |
| genus | Adlercreutzia | rs6811381 | G | T | -0.079 | 0.018 | 9.77E-06 | 19.649 |
| genus | Ruminiclostridium9 | rs147750803 | C | A | -0.049 | 0.011 | 9.83E-06 | 19.648 |
| genus |  | rs11171745 | C | A | 0.073 | 0.016 | 7.23E-06 | 19.646 |
| genus | Parasutterella | rs737483 | A | G | 0.060 | 0.013 | 7.91E-06 | 19.644 |
| genus | Butyricicoccus | rs12034718 | G | A | 0.070 | 0.016 | 9.58E-06 | 19.642 |
| genus | Slackia | rs77641019 | A | T | -0.116 | 0.026 | 8.25E-06 | 19.640 |
| genus | Olsenella | rs8066522 | A | G | -0.107 | 0.024 | 9.70E-06 | 19.640 |
| genus | Bilophila | rs1917709 | T | A | 0.119 | 0.027 | 7.37E-06 | 19.639 |
| genus | Alistipes | rs8188507 | C | T | -0.048 | 0.011 | 9.64E-06 | 19.637 |
| genus | Alistipes | rs1107244 | A | G | 0.076 | 0.017 | 3.59E-06 | 19.636 |
| genus | CandidatusSoleaferrea | rs12679756 | G | A | -0.083 | 0.019 | 9.69E-06 | 19.636 |
| genus |  | rs11052069 | C | T | 0.048 | 0.011 | 9.38E-06 | 19.636 |
| genus | Lactococcus | rs12162342 | G | C | -0.174 | 0.039 | 4.47E-06 | 19.635 |
| genus |  | rs507711 | C | T | -0.047 | 0.011 | 9.63E-06 | 19.634 |
| genus | Turicibacter | rs11868588 | G | A | 0.096 | 0.022 | 9.04E-06 | 19.634 |
| genus | Holdemania | rs75763390 | A | G | -0.130 | 0.029 | 8.30E-06 | 19.634 |
| genus | Slackia | rs7840789 | A | G | -0.116 | 0.026 | 7.99E-06 | 19.634 |
| genus | Odoribacter | rs11041014 | G | A | 0.067 | 0.015 | 9.73E-06 | 19.632 |
| genus | RuminococcaceaeUCG002 | rs57079348 | G | T | -0.077 | 0.017 | 7.22E-06 | 19.632 |
| genus | Tyzzerella3 | rs2311730 | C | A | 0.127 | 0.029 | 5.20E-06 | 19.632 |
| genus | RuminococcaceaeUCG004 | rs12136421 | C | G | 0.120 | 0.027 | 9.55E-06 | 19.630 |
| genus | Sellimonas | rs7182917 | T | C | 0.111 | 0.025 | 9.93E-06 | 19.630 |
| genus | Butyricimonas | rs2591801 | A | G | 0.063 | 0.014 | 8.81E-06 | 19.629 |
| genus | Fusicatenibacter | rs9728894 | C | A | -0.051 | 0.011 | 8.17E-06 | 19.629 |
| genus | Bifidobacterium | rs3739030 | G | A | 0.053 | 0.012 | 6.63E-06 | 19.628 |
| genus |  | rs139737 | C | A | -0.085 | 0.019 | 6.90E-06 | 19.626 |
| genus | Bifidobacterium | rs62287783 | G | A | 0.053 | 0.012 | 9.79E-06 | 19.625 |
| genus | RuminococcaceaeUCG003 | rs2523138 | A | G | -0.054 | 0.012 | 8.96E-06 | 19.624 |
| genus | Clostridiumsensustricto1 | rs11586026 | T | A | 0.111 | 0.025 | 8.85E-06 | 19.624 |
| genus | Roseburia | rs10462485 | T | G | 0.048 | 0.011 | 9.45E-06 | 19.623 |
| genus |  | rs3980709 | T | A | -0.062 | 0.014 | 6.86E-06 | 19.623 |
| genus |  | rs62025228 | G | T | -0.080 | 0.018 | 8.27E-06 | 19.622 |
| genus | Turicibacter | rs111630045 | T | C | 0.096 | 0.022 | 9.13E-06 | 19.622 |
| genus | Butyricimonas | rs1485997 | A | G | 0.062 | 0.014 | 8.85E-06 | 19.622 |
| genus | Coprococcus3 | rs6995587 | T | A | 0.055 | 0.012 | 8.24E-06 | 19.621 |
| genus | Alistipes | rs11766494 | C | T | -0.048 | 0.011 | 9.98E-06 | 19.621 |
| genus | Flavonifractor | rs216583 | G | A | 0.063 | 0.014 | 9.60E-06 | 19.621 |
| genus | ChristensenellaceaeR | rs2188618 | A | T | 0.049 | 0.011 | 9.83E-06 | 19.620 |
| genus | Peptococcus | rs450933 | G | A | 0.105 | 0.024 | 8.71E-06 | 19.620 |
| genus | Peptococcus | rs3759673 | A | T | 0.105 | 0.024 | 8.78E-06 | 19.619 |
| genus | Ruminococcus2 | rs7854299 | A | G | 0.071 | 0.016 | 6.69E-06 | 19.619 |
| genus | Alistipes | rs61946592 | T | C | 0.076 | 0.017 | 3.86E-06 | 19.616 |
| genus | FamilyXIIIAD3011group | rs12911842 | T | A | -0.081 | 0.018 | 6.91E-06 | 19.615 |
| genus |  | rs1475330 | C | T | 0.052 | 0.012 | 8.13E-06 | 19.615 |
| genus | Subdoligranulum | rs1455937 | G | A | -0.051 | 0.012 | 7.97E-06 | 19.614 |
| genus | RuminococcaceaeUCG014 | rs10791168 | G | A | -0.066 | 0.015 | 9.76E-06 | 19.614 |
| genus | Ruminiclostridium6 | rs116969552 | G | A | -0.167 | 0.038 | 9.16E-06 | 19.614 |
| genus | Coprococcus2 | rs61823518 | C | A | -0.096 | 0.022 | 6.68E-06 | 19.613 |
| genus | Parasutterella | rs2190569 | A | G | -0.063 | 0.014 | 9.71E-06 | 19.613 |
| genus | ErysipelotrichaceaeUCG003 | rs214586 | C | T | -0.106 | 0.024 | 8.50E-06 | 19.612 |
| genus | LachnospiraceaeNC2004group | rs117467633 | C | T | -0.170 | 0.038 | 9.13E-06 | 19.612 |
| genus | RikenellaceaeRC9gutgroup | rs2418063 | G | C | -0.134 | 0.030 | 9.50E-06 | 19.611 |
| genus | Anaerofilum | rs2486027 | G | T | -0.182 | 0.041 | 8.29E-06 | 19.609 |
| genus | Slackia | rs72606680 | T | C | -0.116 | 0.026 | 8.26E-06 | 19.609 |
| genus | Sutterella | rs9350083 | G | T | -0.059 | 0.013 | 8.23E-06 | 19.609 |
| genus | Ruminococcus1 | rs4849717 | A | T | 0.133 | 0.030 | 8.83E-06 | 19.609 |
| genus | Alistipes | rs6964425 | T | C | -0.048 | 0.011 | 9.84E-06 | 19.608 |
| genus | Hungatella | rs13249325 | G | T | -0.100 | 0.023 | 9.69E-06 | 19.608 |
| genus | Howardella | rs61771801 | C | G | -0.132 | 0.030 | 9.85E-06 | 19.607 |
| genus | Turicibacter | rs11054677 | T | C | -0.101 | 0.023 | 6.27E-06 | 19.606 |
| genus | Turicibacter | rs2269744 | A | G | 0.096 | 0.022 | 9.11E-06 | 19.605 |
| genus | Terrisporobacter | rs10982246 | G | A | -0.077 | 0.017 | 9.96E-06 | 19.605 |
| genus |  | rs397124 | C | T | -0.056 | 0.013 | 8.45E-06 | 19.604 |
| genus | Lactococcus | rs17045370 | C | T | -0.174 | 0.039 | 4.50E-06 | 19.604 |
| genus | Parasutterella | rs6593368 | G | A | -0.064 | 0.014 | 8.39E-06 | 19.603 |
| genus | Marvinbryantia | rs146541147 | A | G | 0.119 | 0.027 | 6.86E-06 | 19.603 |
| genus | LachnospiraceaeFCS020group | rs9884451 | A | T | 0.055 | 0.012 | 9.45E-06 | 19.603 |
| genus | Anaerotruncus | rs17734739 | C | T | 0.066 | 0.015 | 7.43E-06 | 19.603 |
| genus | ChristensenellaceaeR | rs17081797 | G | A | -0.090 | 0.020 | 3.34E-06 | 19.602 |
| genus |  | rs11684485 | C | T | -0.073 | 0.016 | 9.72E-06 | 19.602 |
| genus | Oscillibacter | rs56246833 | C | T | -0.080 | 0.018 | 9.77E-06 | 19.602 |
| genus | Lactococcus | rs72799257 | T | A | -0.156 | 0.035 | 4.93E-06 | 19.601 |
| genus | Roseburia | rs55858165 | C | A | 0.179 | 0.040 | 9.99E-06 | 19.601 |
| genus |  | rs6502380 | A | G | 0.047 | 0.011 | 8.37E-06 | 19.601 |
| genus | Dialister | rs76680460 | A | G | -0.161 | 0.036 | 8.19E-06 | 19.600 |
| genus | Sutterella | rs1409526 | A | G | -0.055 | 0.012 | 9.06E-06 | 19.600 |
| genus | Coprobacter | rs182453737 | A | T | 0.124 | 0.028 | 7.32E-06 | 19.600 |
| genus | Catenibacterium | rs56327358 | G | A | 0.126 | 0.029 | 9.00E-06 | 19.599 |
| genus | Intestinimonas | rs9823439 | C | T | -0.058 | 0.013 | 9.86E-06 | 19.598 |
| genus | CandidatusSoleaferrea | rs7766248 | C | T | -0.123 | 0.028 | 5.07E-06 | 19.597 |
| genus | Eggerthella | rs4985718 | G | A | 0.114 | 0.026 | 6.14E-06 | 19.596 |
| genus | RuminococcaceaeUCG003 | rs4481546 | T | C | -0.059 | 0.013 | 9.88E-06 | 19.595 |
| genus | Parasutterella | rs7910184 | T | C | -0.064 | 0.014 | 8.46E-06 | 19.594 |
| genus | Fusicatenibacter | rs9727359 | G | T | -0.051 | 0.012 | 8.36E-06 | 19.593 |
| genus | Butyricicoccus | rs75238760 | A | T | 0.062 | 0.014 | 6.80E-06 | 19.592 |
| genus | Ruminiclostridium9 | rs55842512 | A | G | -0.055 | 0.013 | 9.05E-06 | 19.591 |
| genus | Lactobacillus | rs9478791 | T | C | 0.133 | 0.030 | 9.41E-06 | 19.589 |
| genus | Ruminococcus2 | rs58811417 | G | T | 0.071 | 0.016 | 6.42E-06 | 19.589 |
| genus | Parasutterella | rs1425371 | T | C | -0.059 | 0.013 | 9.94E-06 | 19.588 |
| genus | Sellimonas | rs10229799 | C | T | -0.119 | 0.027 | 8.79E-06 | 19.588 |
| genus | Lachnoclostridium | rs6498001 | T | C | 0.047 | 0.011 | 9.57E-06 | 19.587 |
| genus | Intestinimonas | rs1000888 | G | C | -0.059 | 0.013 | 9.71E-06 | 19.587 |
| genus | Senegalimassilia | rs55689333 | C | T | 0.077 | 0.017 | 1.00E-05 | 19.587 |
| genus | CandidatusSoleaferrea | rs7765732 | A | C | -0.123 | 0.028 | 5.10E-06 | 19.587 |
| genus | Olsenella | rs7557702 | A | G | -0.103 | 0.023 | 9.86E-06 | 19.586 |
| genus | RuminococcaceaeUCG014 | rs853612 | G | A | -0.053 | 0.012 | 9.75E-06 | 19.585 |
| genus | Oscillibacter | rs234112 | A | G | 0.067 | 0.015 | 9.35E-06 | 19.584 |
| genus | Subdoligranulum | rs4695415 | G | A | -0.051 | 0.012 | 7.77E-06 | 19.584 |
| genus | Olsenella | rs6035500 | A | G | 0.121 | 0.027 | 8.25E-06 | 19.584 |
| genus |  | rs2559526 | A | G | 0.102 | 0.023 | 9.47E-06 | 19.584 |
| genus | FamilyXIIIUCG001 | rs3798434 | C | T | -0.060 | 0.013 | 7.72E-06 | 19.582 |
| genus | LachnospiraceaeUCG008 | rs34194030 | C | A | 0.088 | 0.020 | 7.89E-06 | 19.582 |
| genus | Prevotella9 | rs1967513 | C | A | -0.065 | 0.015 | 8.46E-06 | 19.581 |
| genus | Lactococcus | rs17045435 | A | G | -0.174 | 0.039 | 4.96E-06 | 19.580 |
| genus | RuminococcaceaeUCG002 | rs11607472 | G | A | -0.078 | 0.018 | 7.19E-06 | 19.580 |
| genus | Sellimonas | rs1476517 | G | A | -0.119 | 0.027 | 9.00E-06 | 19.580 |
| genus | Lachnoclostridium | rs12566975 | C | T | -0.047 | 0.011 | 9.57E-06 | 19.580 |
| genus | Coprococcus1 | rs2907920 | G | A | 0.056 | 0.013 | 7.65E-06 | 19.579 |
| genus | Tyzzerella3 | rs80174235 | G | C | -0.213 | 0.048 | 9.96E-06 | 19.579 |
| genus |  | rs2640569 | C | T | 0.073 | 0.016 | 7.90E-06 | 19.579 |
| genus | Sutterella | rs1033434 | G | T | -0.059 | 0.013 | 8.40E-06 | 19.579 |
| genus | Peptococcus | rs4839724 | T | C | 0.127 | 0.029 | 4.16E-06 | 19.578 |
| genus | Adlercreutzia | rs55719207 | A | G | -0.070 | 0.016 | 9.61E-06 | 19.577 |
| genus | Butyrivibrio | rs2310984 | A | G | 0.114 | 0.026 | 8.19E-06 | 19.576 |
| genus | Eggerthella | rs9898995 | G | A | 0.114 | 0.026 | 5.98E-06 | 19.576 |
| genus |  | rs1999981 | T | G | 0.088 | 0.020 | 8.57E-06 | 19.575 |
| genus | Romboutsia | rs1372227 | G | A | -0.069 | 0.016 | 9.87E-06 | 19.575 |
| genus | Intestinimonas | rs62427239 | A | C | 0.163 | 0.037 | 9.41E-06 | 19.574 |
| genus | Lactococcus | rs10416229 | C | G | 0.109 | 0.025 | 8.58E-06 | 19.574 |
| genus | Eisenbergiella | rs955734 | G | A | 0.100 | 0.023 | 9.07E-06 | 19.574 |
| genus | Parabacteroides | rs7298818 | T | C | 0.089 | 0.020 | 8.54E-06 | 19.574 |
| genus | Peptococcus | rs61995063 | G | A | 0.105 | 0.024 | 9.05E-06 | 19.572 |
| genus | Senegalimassilia | rs1990708 | C | A | -0.110 | 0.025 | 8.91E-06 | 19.572 |
| genus | Peptococcus | rs28384149 | T | C | 0.105 | 0.024 | 9.29E-06 | 19.571 |
| genus | Prevotella9 | rs4821647 | C | G | 0.064 | 0.014 | 9.95E-06 | 19.570 |
| genus | ChristensenellaceaeR | rs7343112 | C | T | 0.049 | 0.011 | 9.99E-06 | 19.570 |
| genus | Oxalobacter | rs13108339 | T | A | 0.199 | 0.045 | 8.70E-06 | 19.570 |
| genus | Subdoligranulum | rs4552435 | T | C | -0.051 | 0.012 | 8.27E-06 | 19.570 |
| genus | Odoribacter | rs76966798 | A | G | -0.119 | 0.027 | 9.99E-06 | 19.569 |
| genus | Fusicatenibacter | rs9727334 | G | A | -0.051 | 0.012 | 8.47E-06 | 19.568 |
| genus | Prevotella9 | rs9428102 | G | A | -0.078 | 0.018 | 4.62E-06 | 19.568 |
| genus | Senegalimassilia | rs2017373 | T | C | 0.078 | 0.018 | 9.50E-06 | 19.567 |
| genus | LachnospiraceaeUCG001 | rs112698185 | G | A | -0.079 | 0.018 | 9.87E-06 | 19.567 |
| genus | Actinomyces | rs71315246 | G | A | -0.097 | 0.022 | 9.83E-06 | 19.566 |
| genus | Prevotella9 | rs12648235 | C | T | 0.079 | 0.018 | 7.39E-06 | 19.565 |
| genus | RuminococcaceaeUCG002 | rs73561250 | C | T | -0.076 | 0.017 | 7.92E-06 | 19.564 |
| genus | ErysipelotrichaceaeUCG003 | rs9963666 | T | G | -0.093 | 0.021 | 8.90E-06 | 19.563 |
| genus | Flavonifractor | rs114873521 | T | C | -0.130 | 0.029 | 7.13E-06 | 19.561 |
| genus |  | rs78399089 | C | T | 0.144 | 0.033 | 6.63E-06 | 19.558 |
| genus | RuminococcaceaeUCG010 | rs7661237 | A | G | -0.055 | 0.012 | 9.79E-06 | 19.557 |
| genus | Ruminococcus1 | rs3819978 | T | C | -0.115 | 0.026 | 8.74E-06 | 19.557 |
| genus | Clostridiumsensustricto1 | rs10804492 | C | T | -0.061 | 0.014 | 9.46E-06 | 19.556 |
| genus | RikenellaceaeRC9gutgroup | rs13223944 | C | T | -0.117 | 0.027 | 8.89E-06 | 19.555 |
| genus | Ruminiclostridium9 | rs4289260 | C | A | 0.058 | 0.013 | 6.11E-06 | 19.554 |
| genus |  | rs59168137 | C | T | 0.077 | 0.017 | 5.35E-06 | 19.554 |
| genus | Bifidobacterium | rs62287778 | T | C | 0.053 | 0.012 | 9.76E-06 | 19.554 |
| genus | Lachnoclostridium | rs7617141 | T | C | -0.078 | 0.018 | 7.37E-06 | 19.554 |
| genus | Olsenella | rs6046512 | T | C | 0.120 | 0.027 | 8.54E-06 | 19.552 |
| genus | Oscillibacter | rs179853 | C | T | 0.067 | 0.015 | 9.53E-06 | 19.552 |
| genus | Butyrivibrio | rs9907578 | A | C | 0.115 | 0.026 | 8.77E-06 | 19.550 |
| genus | RuminococcaceaeUCG003 | rs139730 | C | G | -0.058 | 0.013 | 9.72E-06 | 19.550 |
| genus | Terrisporobacter | rs72944413 | T | C | 0.133 | 0.030 | 9.53E-06 | 19.549 |
| genus |  | rs13161892 | T | C | -0.089 | 0.020 | 8.05E-06 | 19.549 |
| genus | Victivallis | rs1882775 | G | A | -0.138 | 0.031 | 8.73E-06 | 19.549 |
| genus | Methanobrevibacter | rs4779844 | C | G | 0.110 | 0.025 | 9.28E-06 | 19.548 |
| genus | RuminococcaceaeUCG014 | rs11902666 | G | A | 0.055 | 0.012 | 8.74E-06 | 19.547 |
| genus | Turicibacter | rs3734632 | C | T | -0.119 | 0.027 | 8.37E-06 | 19.547 |
| genus | Ruminococcus2 | rs7042369 | T | G | 0.071 | 0.016 | 5.62E-06 | 19.546 |
| genus | Holdemania | rs12701617 | G | A | -0.066 | 0.015 | 9.52E-06 | 19.545 |
| genus | Collinsella | rs149807560 | A | C | -0.104 | 0.024 | 7.10E-06 | 19.545 |
| genus | Ruminiclostridium9 | rs7629021 | T | G | -0.049 | 0.011 | 9.67E-06 | 19.542 |
| genus | Dialister | rs75416973 | G | A | 0.073 | 0.016 | 9.46E-06 | 19.541 |
| genus | LachnospiraceaeUCG001 | rs79476906 | A | T | -0.087 | 0.020 | 8.27E-06 | 19.541 |
| genus | Butyricicoccus | rs7593240 | G | A | 0.055 | 0.012 | 9.84E-06 | 19.540 |
| genus | Senegalimassilia | rs1809460 | C | T | 0.078 | 0.018 | 9.63E-06 | 19.539 |
| genus | ErysipelotrichaceaeUCG003 | rs73311068 | A | G | 0.119 | 0.027 | 8.37E-06 | 19.538 |
| genus | ErysipelotrichaceaeUCG003 | rs73311066 | G | C | 0.119 | 0.027 | 8.37E-06 | 19.538 |
| genus | Eggerthella | rs67490567 | C | T | 0.108 | 0.025 | 8.94E-06 | 19.537 |
| genus | LachnospiraceaeUCG001 | rs10976175 | G | A | -0.064 | 0.014 | 9.65E-06 | 19.537 |
| genus | Alistipes | rs2875322 | C | T | -0.058 | 0.013 | 8.78E-06 | 19.537 |
| genus | Desulfovibrio | rs35710712 | T | C | 0.099 | 0.022 | 8.79E-06 | 19.537 |
| genus | Coprococcus1 | rs12886051 | C | G | -0.052 | 0.012 | 8.01E-06 | 19.536 |
| genus | Desulfovibrio | rs7580530 | T | C | 0.099 | 0.022 | 9.37E-06 | 19.536 |
| genus |  | rs13174809 | G | C | -0.089 | 0.020 | 7.96E-06 | 19.535 |
| genus | Oscillospira | rs12925026 | C | T | 0.136 | 0.031 | 9.31E-06 | 19.535 |
| genus | RuminococcaceaeUCG003 | rs4939837 | G | A | 0.054 | 0.012 | 9.79E-06 | 19.534 |
| genus | Streptococcus | rs8191642 | A | G | -0.060 | 0.014 | 8.09E-06 | 19.532 |
| genus | Coprobacter | rs9972482 | A | T | 0.097 | 0.022 | 9.24E-06 | 19.531 |
| genus | Butyricicoccus | rs10174174 | T | C | 0.055 | 0.012 | 9.91E-06 | 19.531 |
| genus | RuminococcaceaeUCG002 | rs58591149 | T | A | -0.076 | 0.017 | 8.13E-06 | 19.528 |
| genus |  | rs58327496 | T | G | -0.063 | 0.014 | 7.33E-06 | 19.528 |
| genus | Parasutterella | rs10793496 | A | G | -0.063 | 0.014 | 9.27E-06 | 19.528 |
| genus | Akkermansia | rs11184340 | A | G | 0.063 | 0.014 | 9.95E-06 | 19.528 |
| genus | Sutterella | rs4716289 | G | A | -0.059 | 0.013 | 8.67E-06 | 19.527 |
| genus | Ruminiclostridium6 | rs792058 | A | G | 0.055 | 0.013 | 8.58E-06 | 19.527 |
| genus | Coprococcus3 | rs10265310 | T | A | 0.059 | 0.013 | 9.57E-06 | 19.527 |
| genus | Blautia | rs17147246 | C | G | 0.054 | 0.012 | 8.40E-06 | 19.525 |
| genus | Intestinibacter | rs68093214 | T | C | 0.066 | 0.015 | 9.26E-06 | 19.525 |
| genus | Oscillibacter | rs12880119 | T | G | -0.067 | 0.015 | 9.26E-06 | 19.522 |
| genus | Dialister | rs57053627 | A | G | 0.065 | 0.015 | 9.37E-06 | 19.518 |
| genus | Akkermansia | rs12411321 | T | C | 0.073 | 0.017 | 7.80E-06 | 19.518 |
| genus | Escherichia | rs9914886 | T | G | -0.134 | 0.030 | 8.97E-06 | 19.517 |
| genus | Sutterella | rs2142955 | C | T | -0.059 | 0.013 | 8.66E-06 | 19.517 |
| genus | Intestinibacter | rs7041183 | G | A | -0.102 | 0.023 | 6.34E-06 | 19.517 |
| genus | FamilyXIIIUCG001 | rs4907093 | A | G | -0.058 | 0.013 | 8.62E-06 | 19.514 |
| genus | LachnospiraceaeUCG001 | rs7341608 | C | T | -0.078 | 0.018 | 9.48E-06 | 19.514 |
| genus | Oxalobacter | rs62342283 | G | C | 0.199 | 0.045 | 9.04E-06 | 19.514 |
| genus | RuminococcaceaeUCG002 | rs10927426 | G | T | -0.069 | 0.016 | 8.30E-06 | 19.514 |
| genus | Actinomyces | rs10787984 | C | G | 0.094 | 0.021 | 9.62E-06 | 19.513 |
| genus | Holdemanella | rs17527365 | G | A | -0.225 | 0.051 | 9.25E-06 | 19.512 |
| genus | RuminococcaceaeUCG013 | rs1729063 | C | G | -0.053 | 0.012 | 9.64E-06 | 19.512 |
| genus | Peptococcus | rs12069354 | T | C | 0.168 | 0.038 | 9.28E-06 | 19.511 |
| genus | Olsenella | rs2235894 | C | T | 0.120 | 0.027 | 8.20E-06 | 19.507 |
| genus | Olsenella | rs6035499 | A | G | 0.120 | 0.027 | 8.59E-06 | 19.506 |
| genus |  | rs10043038 | G | A | -0.090 | 0.020 | 8.89E-06 | 19.505 |
| genus |  | rs13166543 | T | C | -0.089 | 0.020 | 8.59E-06 | 19.504 |
| genus | Dorea | rs345219 | G | T | -0.050 | 0.011 | 8.80E-06 | 19.504 |
| genus | Tyzzerella3 | rs1957839 | C | T | -0.110 | 0.025 | 9.81E-06 | 19.504 |
| genus | Streptococcus | rs3847387 | G | A | 0.094 | 0.021 | 9.02E-06 | 19.503 |
| genus | Prevotella9 | rs2768367 | C | T | 0.105 | 0.024 | 8.74E-06 | 19.503 |
| genus | Streptococcus | rs4260895 | A | C | -0.056 | 0.013 | 8.87E-06 | 19.503 |
| genus | Bilophila | rs60178956 | A | G | -0.062 | 0.014 | 8.06E-06 | 19.502 |
| genus |  | rs10942121 | G | A | -0.094 | 0.021 | 9.63E-06 | 19.502 |
| genus | Enterorhabdus | rs10101882 | A | G | 0.129 | 0.029 | 7.55E-06 | 19.502 |
| genus |  | rs1465266 | A | G | 0.047 | 0.011 | 9.46E-06 | 19.501 |
| genus | Catenibacterium | rs73117447 | G | T | 0.126 | 0.028 | 8.32E-06 | 19.500 |
| genus |  | rs1409397 | G | C | 0.101 | 0.023 | 9.92E-06 | 19.499 |
| genus | Butyrivibrio | rs34115873 | G | A | 0.114 | 0.026 | 8.31E-06 | 19.499 |
| genus | RuminococcaceaeUCG005 | rs72776570 | A | C | 0.087 | 0.020 | 5.36E-06 | 19.498 |
| genus | ChristensenellaceaeR | rs12983481 | T | C | 0.055 | 0.012 | 9.79E-06 | 19.497 |
| genus | Ruminiclostridium9 | rs4679821 | G | A | -0.049 | 0.011 | 9.93E-06 | 19.495 |
| genus | RuminococcaceaeUCG013 | rs2565137 | T | C | -0.049 | 0.011 | 9.83E-06 | 19.495 |
| genus | Subdoligranulum | rs10497836 | T | C | -0.052 | 0.012 | 8.38E-06 | 19.493 |
| genus | Enterorhabdus | rs7003110 | G | A | 0.131 | 0.030 | 9.86E-06 | 19.493 |
| genus | Enterorhabdus | rs7002267 | C | A | 0.131 | 0.030 | 9.86E-06 | 19.493 |
| genus |  | rs289416 | T | C | -0.062 | 0.014 | 9.74E-06 | 19.493 |
| genus | Akkermansia | rs10005131 | T | G | 0.061 | 0.014 | 9.27E-06 | 19.488 |
| genus | Sellimonas | rs12113766 | C | T | -0.119 | 0.027 | 9.34E-06 | 19.488 |
| genus | Desulfovibrio | rs11902332 | A | G | 0.099 | 0.022 | 9.11E-06 | 19.486 |
| genus | Holdemanella | rs113076513 | C | G | -0.225 | 0.051 | 9.36E-06 | 19.486 |
| genus | Ruminiclostridium9 | rs7629341 | G | C | -0.049 | 0.011 | 9.96E-06 | 19.485 |
| genus | Oscillibacter | rs10834860 | A | G | 0.077 | 0.017 | 9.22E-06 | 19.484 |
| genus | Sutterella | rs9537916 | G | A | -0.056 | 0.013 | 9.96E-06 | 19.484 |
| genus | RuminococcaceaeUCG013 | rs7823627 | G | C | -0.049 | 0.011 | 9.87E-06 | 19.484 |
| genus | Coprobacter | rs11073941 | G | C | 0.097 | 0.022 | 9.44E-06 | 19.481 |
| genus | Lactococcus | rs10423422 | C | T | 0.110 | 0.025 | 9.89E-06 | 19.481 |
| genus | Sutterella | rs10444649 | C | T | -0.055 | 0.012 | 9.70E-06 | 19.480 |
| genus | Phascolarctobacterium | rs11929846 | C | T | -0.070 | 0.016 | 8.88E-06 | 19.479 |
| genus | Turicibacter | rs74516356 | A | T | 0.095 | 0.022 | 9.94E-06 | 19.477 |
| genus | FamilyXIIIAD3011group | rs11736617 | A | G | -0.076 | 0.017 | 9.02E-06 | 19.475 |
| genus | ErysipelotrichaceaeUCG003 | rs11082735 | T | C | -0.094 | 0.021 | 8.82E-06 | 19.475 |
| genus | Turicibacter | rs111931312 | G | A | 0.095 | 0.022 | 9.90E-06 | 19.474 |
| genus | Turicibacter | rs113340666 | G | A | 0.095 | 0.022 | 9.90E-06 | 19.474 |
| genus |  | rs12911214 | T | A | -0.051 | 0.012 | 9.11E-06 | 19.474 |
| genus | ChristensenellaceaeR | rs11881855 | G | A | 0.055 | 0.012 | 9.82E-06 | 19.470 |
| genus | Anaerofilum | rs10744894 | A | G | -0.091 | 0.021 | 9.00E-06 | 19.468 |
| genus |  | rs62025254 | C | A | -0.079 | 0.018 | 9.93E-06 | 19.466 |
| genus | LachnospiraceaeNK4A136group | rs17770545 | C | T | -0.051 | 0.012 | 8.47E-06 | 19.466 |
| genus | Bacteroides | rs1340392 | G | A | -0.058 | 0.013 | 9.60E-06 | 19.463 |
| genus | Olsenella | rs6046515 | T | C | 0.120 | 0.027 | 8.96E-06 | 19.463 |
| genus |  | rs1768152 | C | T | 0.139 | 0.032 | 8.70E-06 | 19.462 |
| genus | Akkermansia | rs28811282 | G | A | 0.063 | 0.014 | 8.75E-06 | 19.460 |
| genus | Holdemania | rs74518299 | T | C | -0.130 | 0.029 | 9.19E-06 | 19.460 |
| genus |  | rs11044927 | G | T | -0.096 | 0.022 | 6.63E-06 | 19.458 |
| genus | Holdemania | rs35245389 | A | T | -0.130 | 0.029 | 9.33E-06 | 19.457 |
| genus | Lachnospira | rs10834854 | G | C | -0.059 | 0.013 | 7.76E-06 | 19.454 |
| genus | Butyricicoccus | rs7320057 | A | G | 0.080 | 0.018 | 7.99E-06 | 19.453 |
| genus | Blautia | rs67402703 | G | A | 0.054 | 0.012 | 8.66E-06 | 19.452 |
| genus | RuminococcaceaeUCG013 | rs17524593 | G | T | 0.068 | 0.015 | 6.30E-06 | 19.452 |
| genus | Prevotella9 | rs10029966 | T | G | 0.078 | 0.018 | 8.74E-06 | 19.452 |
| genus | RuminococcaceaeUCG009 | rs138460696 | G | A | 0.139 | 0.032 | 9.81E-06 | 19.451 |
| genus | Phascolarctobacterium | rs28525131 | A | G | -0.119 | 0.027 | 8.23E-06 | 19.450 |
| genus |  | rs9901465 | C | T | 0.047 | 0.011 | 9.90E-06 | 19.449 |
| genus | Lactococcus | rs34910182 | G | A | 0.103 | 0.023 | 9.58E-06 | 19.449 |
| genus |  | rs1689512 | A | G | 0.072 | 0.016 | 8.22E-06 | 19.448 |
| genus |  | rs4869133 | A | G | -0.181 | 0.041 | 7.24E-06 | 19.448 |
| genus | Prevotella7 | rs12124567 | G | A | -0.121 | 0.028 | 9.49E-06 | 19.448 |
| genus | Olsenella | rs6046513 | T | C | 0.120 | 0.027 | 9.06E-06 | 19.446 |
| genus | Ruminococcus2 | rs61791567 | G | A | -0.052 | 0.012 | 8.78E-06 | 19.446 |
| genus |  | rs887350 | G | A | 0.047 | 0.011 | 8.74E-06 | 19.445 |
| genus |  | rs61838421 | T | C | -0.059 | 0.013 | 6.58E-06 | 19.445 |
| genus | Akkermansia | rs28858161 | T | C | 0.063 | 0.014 | 8.83E-06 | 19.443 |
| genus |  | rs6934739 | G | A | 0.111 | 0.025 | 9.75E-06 | 19.442 |
| genus | Oscillibacter | rs4981915 | C | G | -0.067 | 0.015 | 9.84E-06 | 19.441 |
| genus | Butyrivibrio | rs8079738 | T | C | 0.119 | 0.027 | 9.23E-06 | 19.440 |
| genus | Faecalibacterium | rs2835874 | C | T | -0.087 | 0.020 | 7.54E-06 | 19.440 |
| genus | Sutterella | rs6938698 | G | T | -0.059 | 0.013 | 9.00E-06 | 19.439 |
| genus |  | rs2323450 | G | T | 0.047 | 0.011 | 8.67E-06 | 19.439 |
| genus | Ruminiclostridium6 | rs594106 | C | G | -0.146 | 0.033 | 5.92E-06 | 19.438 |
| genus | Bifidobacterium | rs6711997 | T | C | 0.062 | 0.014 | 5.61E-06 | 19.437 |
| genus |  | rs117748144 | C | T | -0.127 | 0.029 | 7.86E-06 | 19.437 |
| genus | Parasutterella | rs7077599 | T | C | -0.063 | 0.014 | 9.19E-06 | 19.436 |
| genus |  | rs758995 | G | A | 0.076 | 0.017 | 7.91E-06 | 19.434 |
| genus | CandidatusSoleaferrea | rs113393759 | C | T | -0.123 | 0.028 | 5.48E-06 | 19.434 |
| genus | Butyrivibrio | rs68014746 | G | A | 0.114 | 0.026 | 8.63E-06 | 19.433 |
| genus | RuminococcaceaeUCG014 | rs34402072 | T | C | -0.069 | 0.016 | 9.80E-06 | 19.431 |
| genus | Olsenella | rs11673945 | C | T | -0.105 | 0.024 | 9.80E-06 | 19.430 |
| genus | Paraprevotella | rs1487345 | C | A | -0.100 | 0.023 | 9.54E-06 | 19.428 |
| genus | Olsenella | rs11674257 | G | C | -0.105 | 0.024 | 9.81E-06 | 19.428 |
| genus | Fusicatenibacter | rs12066113 | G | A | -0.051 | 0.012 | 9.14E-06 | 19.428 |
| genus | Bifidobacterium | rs79769806 | A | G | -0.081 | 0.018 | 9.91E-06 | 19.427 |
| genus | Eisenbergiella | rs16911971 | G | A | 0.126 | 0.029 | 8.97E-06 | 19.427 |
| genus | Ruminococcus1 | rs11776480 | C | T | -0.073 | 0.016 | 9.36E-06 | 19.427 |
| genus |  | rs10263623 | T | C | 0.193 | 0.044 | 8.91E-06 | 19.425 |
| genus | LachnospiraceaeNK4A136group | rs74167868 | C | T | -0.092 | 0.021 | 9.37E-06 | 19.422 |
| genus | Faecalibacterium | rs10519101 | T | C | 0.048 | 0.011 | 9.82E-06 | 19.422 |
| genus | Coprobacter | rs28402691 | C | T | 0.111 | 0.025 | 9.56E-06 | 19.419 |
| genus | Oscillospira | rs12924124 | C | T | 0.135 | 0.031 | 9.52E-06 | 19.418 |
| genus | RuminococcaceaeNK4A214group | rs10178772 | T | G | 0.071 | 0.016 | 7.72E-06 | 19.417 |
| genus | RuminococcaceaeUCG014 | rs10495392 | T | C | -0.082 | 0.019 | 9.96E-06 | 19.417 |
| genus | RuminococcaceaeNK4A214group | rs113462046 | C | A | 0.061 | 0.014 | 8.33E-06 | 19.416 |
| genus | Akkermansia | rs76564762 | C | T | 0.104 | 0.024 | 9.67E-06 | 19.415 |
| genus | RuminococcaceaeUCG002 | rs73561259 | A | T | -0.076 | 0.017 | 8.34E-06 | 19.414 |
| genus | RuminococcaceaeUCG004 | rs12076769 | T | C | 0.121 | 0.027 | 9.07E-06 | 19.414 |
| genus | ChristensenellaceaeR | rs62467069 | T | C | 0.112 | 0.025 | 6.57E-06 | 19.413 |
| genus | RikenellaceaeRC9gutgroup | rs7796838 | G | A | -0.117 | 0.027 | 9.53E-06 | 19.411 |
| genus | Hungatella | rs10044993 | A | C | 0.140 | 0.032 | 8.07E-06 | 19.409 |
| genus | Turicibacter | rs28784121 | C | T | 0.095 | 0.021 | 9.52E-06 | 19.406 |
| genus | Akkermansia | rs28613890 | C | T | 0.064 | 0.014 | 9.91E-06 | 19.406 |
| genus | Akkermansia | rs1988252 | G | A | 0.063 | 0.014 | 9.27E-06 | 19.406 |
| genus | RuminococcaceaeUCG002 | rs73561254 | C | T | -0.076 | 0.017 | 8.53E-06 | 19.406 |
| genus | Desulfovibrio | rs2032031 | G | A | -0.065 | 0.015 | 9.14E-06 | 19.404 |
| genus | Alistipes | rs6563551 | T | G | 0.075 | 0.017 | 4.10E-06 | 19.402 |
| genus | Akkermansia | rs2865331 | G | A | 0.062 | 0.014 | 9.09E-06 | 19.402 |
| genus | Adlercreutzia | rs1046175 | G | C | 0.113 | 0.026 | 6.36E-06 | 19.401 |
| genus | Holdemanella | rs12415649 | C | G | 0.084 | 0.019 | 7.88E-06 | 19.401 |
| genus | Lactobacillus | rs9986520 | T | C | 0.133 | 0.030 | 7.84E-06 | 19.398 |
| genus | Subdoligranulum | rs1840628 | G | A | -0.051 | 0.012 | 9.07E-06 | 19.395 |
| genus | Holdemania | rs76084714 | A | G | -0.129 | 0.029 | 9.53E-06 | 19.393 |
| genus | Anaerofilum | rs2487275 | G | A | -0.181 | 0.041 | 9.24E-06 | 19.392 |
| genus | Butyricimonas | rs72814526 | A | G | 0.066 | 0.015 | 9.43E-06 | 19.392 |
| genus | Ruminiclostridium9 | rs2377294 | G | T | -0.088 | 0.020 | 8.94E-06 | 19.391 |
| genus | Bilophila | rs2713349 | T | A | 0.062 | 0.014 | 8.63E-06 | 19.389 |
| genus | Fusicatenibacter | rs11172519 | G | T | -0.059 | 0.013 | 9.57E-06 | 19.388 |
| genus | FamilyXIIIAD3011group | rs739451 | T | C | 0.065 | 0.015 | 7.88E-06 | 19.386 |
| genus |  | rs12719051 | G | A | 0.092 | 0.021 | 7.12E-06 | 19.386 |
| genus | LachnospiraceaeNK4A136group | rs301777 | T | G | -0.049 | 0.011 | 9.87E-06 | 19.385 |
| genus | Peptococcus | rs12147893 | A | G | 0.104 | 0.024 | 9.96E-06 | 19.385 |
| genus | Olsenella | rs1983198 | T | A | 0.119 | 0.027 | 8.98E-06 | 19.384 |
| genus | Methanobrevibacter | rs4753222 | G | A | 0.112 | 0.025 | 8.99E-06 | 19.384 |
| genus | Butyricimonas | rs56093015 | T | C | 0.066 | 0.015 | 9.48E-06 | 19.383 |
| genus | Methanobrevibacter | rs3912664 | G | T | 0.112 | 0.025 | 8.68E-06 | 19.381 |
| genus | Akkermansia | rs941682 | A | G | -0.063 | 0.014 | 9.17E-06 | 19.381 |
| genus | RuminococcaceaeUCG009 | rs11577989 | C | T | -0.129 | 0.029 | 9.42E-06 | 19.380 |
| genus | ErysipelotrichaceaeUCG003 | rs6507884 | A | G | -0.094 | 0.021 | 9.99E-06 | 19.379 |
| genus |  | rs59427698 | G | A | -0.058 | 0.013 | 5.37E-06 | 19.379 |
| genus | Coprobacter | rs12996055 | C | A | 0.092 | 0.021 | 8.08E-06 | 19.378 |
| genus | Subdoligranulum | rs28590759 | C | A | -0.051 | 0.012 | 8.85E-06 | 19.376 |
| genus | ErysipelotrichaceaeUCG003 | rs16905331 | T | C | 0.119 | 0.027 | 8.65E-06 | 19.376 |
| genus | RuminococcaceaeUCG014 | rs62478831 | T | C | -0.057 | 0.013 | 9.76E-06 | 19.375 |
| genus | Ruminiclostridium6 | rs848651 | C | T | 0.058 | 0.013 | 8.67E-06 | 19.375 |
| genus | Ruminiclostridium6 | rs10837369 | C | T | 0.060 | 0.014 | 8.37E-06 | 19.375 |
| genus | ErysipelotrichaceaeUCG003 | rs7009613 | G | C | 0.119 | 0.027 | 8.85E-06 | 19.375 |
| genus | Ruminiclostridium6 | rs848653 | C | T | 0.058 | 0.013 | 9.10E-06 | 19.374 |
| genus | Methanobrevibacter | rs4802933 | G | A | -0.136 | 0.031 | 9.74E-06 | 19.374 |
| genus | Holdemanella | rs12414567 | G | A | 0.084 | 0.019 | 8.07E-06 | 19.372 |
| genus |  | rs6807798 | G | T | 0.060 | 0.014 | 8.46E-06 | 19.370 |
| genus | Intestinimonas | rs4722076 | A | G | 0.058 | 0.013 | 9.15E-06 | 19.368 |
| genus | Butyricimonas | rs2591757 | C | G | 0.063 | 0.014 | 9.87E-06 | 19.367 |
| genus | Marvinbryantia | rs72948273 | G | A | -0.120 | 0.027 | 9.09E-06 | 19.367 |
| genus | Fusicatenibacter | rs10439674 | G | A | -0.057 | 0.013 | 7.68E-06 | 19.367 |
| genus | Marvinbryantia | rs3111246 | C | G | 0.066 | 0.015 | 8.98E-06 | 19.366 |
| genus | Holdemania | rs117042274 | A | C | -0.129 | 0.029 | 9.58E-06 | 19.365 |
| genus | Alistipes | rs35909684 | C | A | -0.085 | 0.019 | 7.65E-06 | 19.364 |
| genus | Erysipelatoclostridium | rs62060367 | C | T | -0.080 | 0.018 | 6.70E-06 | 19.362 |
| genus |  | rs12189268 | T | C | -0.087 | 0.020 | 9.83E-06 | 19.361 |
| genus |  | rs329903 | G | A | -0.088 | 0.020 | 9.06E-06 | 19.361 |
| genus | LachnospiraceaeUCG010 | rs2833528 | T | C | -0.056 | 0.013 | 9.92E-06 | 19.359 |
| genus | Lactococcus | rs10190578 | C | G | -0.139 | 0.032 | 7.40E-06 | 19.357 |
| genus | Butyrivibrio | rs7222581 | A | G | 0.118 | 0.027 | 9.77E-06 | 19.356 |
| genus | RuminococcaceaeUCG005 | rs76205969 | C | A | 0.077 | 0.018 | 9.42E-06 | 19.355 |
| genus | RuminococcaceaeUCG014 | rs142598371 | C | T | -0.057 | 0.013 | 9.85E-06 | 19.355 |
| genus | Coprobacter | rs6496712 | T | G | 0.088 | 0.020 | 8.31E-06 | 19.355 |
| genus | RuminococcaceaeUCG002 | rs60613323 | T | C | -0.076 | 0.017 | 9.04E-06 | 19.355 |
| genus | Streptococcus | rs12218294 | A | T | 0.094 | 0.021 | 9.87E-06 | 19.354 |
| genus | CandidatusSoleaferrea | rs6568555 | A | G | -0.123 | 0.028 | 6.16E-06 | 19.353 |
| genus | Dialister | rs7131542 | G | A | 0.101 | 0.023 | 9.48E-06 | 19.353 |
| genus | Prevotella9 | rs7976209 | C | T | -0.087 | 0.020 | 7.28E-06 | 19.351 |
| genus | LachnospiraceaeNC2004group | rs1929743 | C | T | 0.084 | 0.019 | 9.06E-06 | 19.351 |
| genus |  | rs143694765 | C | T | 0.087 | 0.020 | 9.75E-06 | 19.349 |
| genus | RuminococcaceaeUCG002 | rs12405445 | C | T | -0.065 | 0.015 | 7.48E-06 | 19.349 |
| genus | Gordonibacter | rs3765837 | G | T | -0.191 | 0.043 | 7.17E-06 | 19.348 |
| genus | Peptococcus | rs117854205 | C | G | -0.132 | 0.030 | 8.21E-06 | 19.346 |
| genus | LachnospiraceaeNK4A136group | rs77192568 | A | C | 0.059 | 0.013 | 7.12E-06 | 19.345 |
| genus |  | rs61838419 | G | A | -0.059 | 0.013 | 6.97E-06 | 19.344 |
| genus | Coprococcus2 | rs67539765 | G | A | 0.069 | 0.016 | 7.71E-06 | 19.341 |
| genus | Coprococcus3 | rs56112306 | G | A | 0.055 | 0.012 | 9.70E-06 | 19.341 |
| genus | Marvinbryantia | rs11620597 | C | T | 0.119 | 0.027 | 7.80E-06 | 19.340 |
| genus | RikenellaceaeRC9gutgroup | rs13237112 | T | C | -0.117 | 0.027 | 9.97E-06 | 19.337 |
| genus | Turicibacter | rs77854832 | A | G | -0.124 | 0.028 | 9.55E-06 | 19.336 |
| genus | Akkermansia | rs10516447 | A | G | 0.103 | 0.023 | 9.25E-06 | 19.334 |
| genus | Subdoligranulum | rs2605244 | C | T | -0.051 | 0.012 | 9.37E-06 | 19.333 |
| genus | ErysipelotrichaceaeUCG003 | rs73307288 | T | C | 0.118 | 0.027 | 9.01E-06 | 19.332 |
| genus | Flavonifractor | rs7535408 | A | G | -0.067 | 0.015 | 9.39E-06 | 19.332 |
| genus | ChristensenellaceaeR | rs10487399 | T | C | 0.111 | 0.025 | 6.68E-06 | 19.332 |
| genus | Tyzzerella3 | rs2695600 | G | A | 0.125 | 0.029 | 2.92E-06 | 19.331 |
| genus |  | rs11171713 | G | A | 0.087 | 0.020 | 8.30E-06 | 19.330 |
| genus | Alistipes | rs61946576 | T | C | 0.075 | 0.017 | 4.32E-06 | 19.325 |
| genus | Slackia | rs59106370 | C | A | -0.100 | 0.023 | 8.17E-06 | 19.325 |
| genus | Coprococcus1 | rs1519491 | C | T | 0.050 | 0.011 | 8.95E-06 | 19.325 |
| genus | Paraprevotella | rs76403863 | C | T | -0.155 | 0.035 | 5.26E-06 | 19.324 |
| genus |  | rs16886734 | T | C | -0.089 | 0.020 | 9.01E-06 | 19.322 |
| genus | Slackia | rs10853973 | C | T | 0.101 | 0.023 | 8.20E-06 | 19.320 |
| genus | Slackia | rs13335738 | T | C | 0.145 | 0.033 | 9.11E-06 | 19.319 |
| genus | Marvinbryantia | rs8006832 | T | G | -0.095 | 0.022 | 6.58E-06 | 19.317 |
| genus | Streptococcus | rs2370083 | T | G | -0.082 | 0.019 | 9.75E-06 | 19.317 |
| genus | Bacteroides | rs79325005 | A | G | 0.057 | 0.013 | 8.58E-06 | 19.317 |
| genus | Slackia | rs75626975 | C | T | -0.115 | 0.026 | 9.62E-06 | 19.314 |
| genus | Akkermansia | rs12904881 | G | A | 0.058 | 0.013 | 9.78E-06 | 19.313 |
| genus | Alistipes | rs6563550 | C | T | 0.075 | 0.017 | 3.97E-06 | 19.309 |
| genus | CandidatusSoleaferrea | rs12374597 | A | G | -0.122 | 0.028 | 5.87E-06 | 19.309 |
| genus |  | rs1558267 | A | G | 0.047 | 0.011 | 9.45E-06 | 19.307 |
| genus | Paraprevotella | rs10842464 | C | T | -0.076 | 0.017 | 6.60E-06 | 19.305 |
| genus | RuminococcaceaeUCG002 | rs73561239 | C | T | -0.076 | 0.017 | 9.84E-06 | 19.303 |
| genus | Turicibacter | rs10845475 | T | C | -0.093 | 0.021 | 9.32E-06 | 19.303 |
| genus | RuminococcaceaeUCG013 | rs7092659 | T | C | -0.058 | 0.013 | 9.64E-06 | 19.303 |
| genus | Lactococcus | rs17045386 | C | G | -0.173 | 0.039 | 4.75E-06 | 19.299 |
| genus | Intestinibacter | rs12210583 | G | T | 0.155 | 0.035 | 8.20E-06 | 19.298 |
| genus |  | rs17474256 | A | G | 0.081 | 0.018 | 9.45E-06 | 19.297 |
| genus |  | rs11864644 | C | T | -0.140 | 0.032 | 5.01E-06 | 19.297 |
| genus | Butyrivibrio | rs11658561 | T | C | 0.118 | 0.027 | 1.00E-05 | 19.295 |
| genus | Bilophila | rs11069458 | C | T | -0.068 | 0.016 | 7.72E-06 | 19.293 |
| genus | LachnospiraceaeUCG001 | rs74034332 | A | G | 0.168 | 0.038 | 3.33E-06 | 19.288 |
| genus | Anaerostipes | rs176091 | A | T | 0.074 | 0.017 | 5.25E-06 | 19.287 |
| genus | Akkermansia | rs74399905 | T | A | 0.103 | 0.023 | 9.41E-06 | 19.286 |
| genus | Bilophila | rs9899990 | G | A | -0.103 | 0.023 | 9.07E-06 | 19.283 |
| genus | RikenellaceaeRC9gutgroup | rs2074881 | C | T | -0.142 | 0.032 | 9.45E-06 | 19.283 |
| genus | Adlercreutzia | rs6537600 | T | C | 0.112 | 0.026 | 6.71E-06 | 19.282 |
| genus | Blautia | rs16892041 | C | T | -0.062 | 0.014 | 8.82E-06 | 19.277 |
| genus | Ruminiclostridium9 | rs10444612 | C | T | 0.068 | 0.015 | 8.29E-06 | 19.277 |
| genus | Holdemania | rs12877506 | A | G | -0.125 | 0.029 | 8.03E-06 | 19.277 |
| genus | LachnospiraceaeNC2004group | rs17067076 | A | G | -0.155 | 0.035 | 5.61E-06 | 19.277 |
| genus | Flavonifractor | rs4949771 | C | A | -0.067 | 0.015 | 9.54E-06 | 19.275 |
| genus | CandidatusSoleaferrea | rs11752532 | C | G | -0.122 | 0.028 | 6.60E-06 | 19.274 |
| genus | LachnospiraceaeUCG008 | rs35561835 | A | G | 0.087 | 0.020 | 9.46E-06 | 19.273 |
| genus | Coprococcus2 | rs10070053 | G | A | 0.059 | 0.014 | 7.65E-06 | 19.273 |
| genus | Methanobrevibacter | rs10830292 | T | G | 0.112 | 0.025 | 9.44E-06 | 19.272 |
| genus | Lachnospira | rs41526047 | A | C | -0.057 | 0.013 | 8.55E-06 | 19.271 |
| genus | RuminococcaceaeUCG009 | rs6952765 | A | G | 0.073 | 0.017 | 8.13E-06 | 19.269 |
| genus | ErysipelotrichaceaeUCG003 | rs73074432 | T | C | 0.072 | 0.016 | 9.99E-06 | 19.268 |
| genus | Peptococcus | rs56252928 | T | C | 0.126 | 0.029 | 4.68E-06 | 19.268 |
| genus | Subdoligranulum | rs16861606 | T | C | -0.051 | 0.012 | 9.55E-06 | 19.268 |
| genus |  | rs77349995 | C | T | 0.096 | 0.022 | 9.14E-06 | 19.267 |
| genus | Anaerofilum | rs1767111 | C | A | -0.177 | 0.040 | 7.61E-06 | 19.267 |
| genus | Butyrivibrio | rs1970323 | T | C | 0.114 | 0.026 | 9.88E-06 | 19.266 |
| genus | Fusicatenibacter | rs8028026 | G | A | -0.079 | 0.018 | 8.06E-06 | 19.259 |
| genus |  | rs7710079 | T | C | -0.089 | 0.020 | 9.43E-06 | 19.259 |
| genus |  | rs11102814 | C | T | 0.086 | 0.020 | 6.49E-06 | 19.257 |
| genus | RuminococcaceaeUCG014 | rs62354544 | G | T | -0.113 | 0.026 | 9.64E-06 | 19.256 |
| genus | RuminococcaceaeUCG005 | rs7150192 | C | T | 0.063 | 0.014 | 8.63E-06 | 19.252 |
| genus | Haemophilus | rs56310940 | C | G | -0.108 | 0.025 | 7.23E-06 | 19.250 |
| genus | Alistipes | rs7324216 | C | T | 0.075 | 0.017 | 5.00E-06 | 19.249 |
| genus |  | rs2109479 | A | G | 0.056 | 0.013 | 8.37E-06 | 19.248 |
| genus | Butyricimonas | rs77723681 | C | T | -0.143 | 0.033 | 9.60E-06 | 19.245 |
| genus | RuminococcaceaeUCG002 | rs7985747 | G | A | -0.076 | 0.017 | 9.98E-06 | 19.241 |
| genus |  | rs16886738 | G | A | -0.089 | 0.020 | 9.39E-06 | 19.241 |
| genus | Prevotella7 | rs118038478 | G | A | 0.206 | 0.047 | 7.85E-06 | 19.239 |
| genus | RuminococcaceaeUCG004 | rs11961899 | A | G | -0.071 | 0.016 | 9.18E-06 | 19.237 |
| genus |  | rs13409418 | T | G | 0.081 | 0.019 | 9.78E-06 | 19.235 |
| genus |  | rs76496924 | T | A | 0.154 | 0.035 | 6.15E-06 | 19.234 |
| genus | Bacteroides | rs78280928 | G | A | 0.057 | 0.013 | 8.95E-06 | 19.234 |
| genus | Olsenella | rs1980504 | A | C | 0.119 | 0.027 | 9.75E-06 | 19.233 |
| genus | Methanobrevibacter | rs4385899 | C | T | 0.112 | 0.025 | 9.54E-06 | 19.230 |
| genus | Ruminiclostridium9 | rs12856365 | A | T | 0.068 | 0.015 | 8.28E-06 | 19.230 |
| genus | Intestinibacter | rs9644855 | C | G | -0.103 | 0.024 | 8.08E-06 | 19.228 |
| genus | LachnospiraceaeNK4A136group | rs12515958 | C | T | -0.051 | 0.012 | 9.75E-06 | 19.227 |
| genus | LachnospiraceaeUCG004 | rs12072562 | C | T | 0.133 | 0.030 | 7.07E-06 | 19.225 |
| genus | CandidatusSoleaferrea | rs7767882 | C | A | -0.122 | 0.028 | 6.35E-06 | 19.224 |
| genus | Ruminiclostridium6 | rs1452465 | C | T | 0.060 | 0.014 | 9.01E-06 | 19.224 |
| genus | Clostridiumsensustricto1 | rs531166 | C | T | -0.074 | 0.017 | 5.97E-06 | 19.223 |
| genus | ChristensenellaceaeR | rs62467083 | C | T | 0.111 | 0.025 | 7.09E-06 | 19.223 |
| genus | Ruminiclostridium5 | rs73002572 | C | G | 0.182 | 0.041 | 8.82E-06 | 19.223 |
| genus | ErysipelotrichaceaeUCG003 | rs59253454 | A | G | 0.118 | 0.027 | 9.29E-06 | 19.219 |
| genus | CandidatusSoleaferrea | rs36160691 | A | G | 0.104 | 0.024 | 5.73E-06 | 19.218 |
| genus | Marvinbryantia | rs72948271 | A | G | -0.119 | 0.027 | 9.13E-06 | 19.214 |
| genus | Anaerotruncus | rs3915228 | T | C | -0.064 | 0.015 | 9.83E-06 | 19.212 |
| genus | Butyricimonas | rs12304031 | A | G | -0.086 | 0.020 | 6.70E-06 | 19.211 |
| genus | RuminococcaceaeUCG004 | rs58393072 | A | T | -0.071 | 0.016 | 9.47E-06 | 19.208 |
| genus | Anaerotruncus | rs34449434 | C | A | -0.050 | 0.011 | 9.85E-06 | 19.208 |
| genus |  | rs138531890 | G | A | 0.153 | 0.035 | 5.43E-06 | 19.204 |
| genus | Lachnoclostridium | rs72829893 | T | G | 0.117 | 0.027 | 5.58E-06 | 19.199 |
| genus |  | rs77919058 | T | C | 0.076 | 0.017 | 4.32E-06 | 19.198 |
| genus | Bifidobacterium | rs4908116 | C | T | -0.147 | 0.033 | 9.77E-06 | 19.197 |
| genus | ChristensenellaceaeR | rs62467070 | T | C | 0.111 | 0.025 | 7.34E-06 | 19.192 |
| genus | RuminococcaceaeUCG014 | rs61898819 | T | A | 0.061 | 0.014 | 9.92E-06 | 19.190 |
| genus | Bifidobacterium | rs1879087 | T | C | 0.105 | 0.024 | 1.45E-06 | 19.189 |
| genus |  | rs34273234 | C | T | 0.087 | 0.020 | 8.92E-06 | 19.181 |
| genus | Coprobacter | rs76001613 | G | C | 0.216 | 0.049 | 9.13E-06 | 19.179 |
| genus | FamilyXIIIAD3011group | rs28594460 | A | G | 0.070 | 0.016 | 9.76E-06 | 19.177 |
| genus | Howardella | rs10048062 | T | C | -0.147 | 0.034 | 8.59E-06 | 19.172 |
| genus | Bilophila | rs2713348 | G | A | 0.061 | 0.014 | 9.91E-06 | 19.169 |
| genus | CandidatusSoleaferrea | rs117412830 | A | G | -0.122 | 0.028 | 6.40E-06 | 19.168 |
| genus | Eggerthella | rs7222311 | G | A | 0.112 | 0.026 | 7.33E-06 | 19.166 |
| genus |  | rs209813 | A | G | -0.103 | 0.024 | 9.23E-06 | 19.166 |
| genus | Anaerofilum | rs1098567 | G | A | -0.180 | 0.041 | 8.74E-06 | 19.165 |
| genus | Anaerofilum | rs1098566 | G | A | -0.180 | 0.041 | 8.74E-06 | 19.165 |
| genus | Ruminiclostridium6 | rs12360242 | C | T | -0.095 | 0.022 | 6.69E-06 | 19.163 |
| genus | RuminococcaceaeUCG004 | rs7123615 | G | C | -0.079 | 0.018 | 7.09E-06 | 19.162 |
| genus | Tyzzerella3 | rs12713341 | A | G | 0.125 | 0.028 | 4.29E-06 | 19.161 |
| genus | RuminococcaceaeUCG002 | rs59413630 | T | C | -0.076 | 0.017 | 9.29E-06 | 19.159 |
| genus |  | rs58498416 | T | C | -0.052 | 0.012 | 9.68E-06 | 19.156 |
| genus | CandidatusSoleaferrea | rs75942063 | G | A | -0.122 | 0.028 | 7.10E-06 | 19.147 |
| genus | RuminococcaceaeUCG005 | rs4900222 | A | T | 0.063 | 0.014 | 8.90E-06 | 19.147 |
| genus |  | rs35298140 | C | T | 0.075 | 0.017 | 8.35E-06 | 19.143 |
| genus | Intestinibacter | rs10965050 | G | T | -0.103 | 0.023 | 6.02E-06 | 19.137 |
| genus | Collinsella | rs73068166 | T | G | -0.103 | 0.024 | 8.58E-06 | 19.131 |
| genus | Lachnospira | rs7102091 | C | T | -0.057 | 0.013 | 8.46E-06 | 19.130 |
| genus | Ruminococcus2 | rs7693984 | A | G | -0.103 | 0.024 | 9.42E-06 | 19.130 |
| genus | Anaerotruncus | rs7637290 | C | A | -0.066 | 0.015 | 8.71E-06 | 19.129 |
| genus | ChristensenellaceaeR | rs17141346 | C | T | 0.110 | 0.025 | 7.58E-06 | 19.128 |
| genus | Parasutterella | rs472307 | C | T | 0.060 | 0.014 | 6.88E-06 | 19.124 |
| genus | LachnospiraceaeND3007group | rs72776675 | C | T | -0.065 | 0.015 | 8.72E-06 | 19.123 |
| genus | Roseburia | rs147990086 | G | A | -0.058 | 0.013 | 8.93E-06 | 19.120 |
| genus | RuminococcaceaeUCG009 | rs6952633 | A | G | 0.073 | 0.017 | 8.79E-06 | 19.120 |
| genus |  | rs6863988 | T | A | -0.088 | 0.020 | 9.99E-06 | 19.118 |
| genus | Anaerotruncus | rs1920392 | C | T | -0.063 | 0.014 | 8.76E-06 | 19.113 |
| genus | Ruminococcus2 | rs6537941 | C | A | 0.070 | 0.016 | 7.46E-06 | 19.110 |
| genus | Lachnospira | rs111843066 | A | G | 0.086 | 0.020 | 9.47E-06 | 19.109 |
| genus | Escherichia | rs118526 | A | C | -0.059 | 0.014 | 8.00E-06 | 19.108 |
| genus | LachnospiraceaeUCG001 | rs80219526 | G | T | 0.185 | 0.042 | 5.71E-06 | 19.104 |
| genus | RuminococcaceaeUCG013 | rs9324861 | C | T | 0.064 | 0.015 | 8.44E-06 | 19.103 |
| genus | Roseburia | rs113661349 | A | G | -0.130 | 0.030 | 9.11E-06 | 19.099 |
| genus | FamilyXIIIAD3011group | rs9276029 | G | A | -0.081 | 0.019 | 8.93E-06 | 19.097 |
| genus | Anaerotruncus | rs12056802 | G | C | 0.077 | 0.018 | 6.13E-06 | 19.093 |
| genus |  | rs142387626 | C | T | 0.153 | 0.035 | 5.81E-06 | 19.092 |
| genus | Lachnoclostridium | rs825045 | A | T | -0.108 | 0.025 | 9.32E-06 | 19.090 |
| genus | LachnospiraceaeUCG010 | rs2153460 | T | A | -0.068 | 0.016 | 9.17E-06 | 19.089 |
| genus |  | rs6502381 | A | G | 0.047 | 0.011 | 9.63E-06 | 19.089 |
| genus | Oxalobacter | rs3862635 | T | C | -0.172 | 0.039 | 9.19E-06 | 19.086 |
| genus | Ruminococcus2 | rs77214829 | T | C | 0.192 | 0.044 | 8.77E-06 | 19.085 |
| genus | Ruminiclostridium5 | rs17028250 | G | A | 0.181 | 0.041 | 9.40E-06 | 19.084 |
| genus | RikenellaceaeRC9gutgroup | rs1079745 | G | A | 0.167 | 0.038 | 9.90E-06 | 19.074 |
| genus | Faecalibacterium | rs2835873 | G | A | -0.086 | 0.020 | 9.44E-06 | 19.070 |
| genus | Adlercreutzia | rs11604400 | T | C | -0.103 | 0.023 | 9.74E-06 | 19.060 |
| genus | FamilyXIIIAD3011group | rs12185072 | C | T | 0.071 | 0.016 | 9.71E-06 | 19.060 |
| genus |  | rs11720857 | T | C | 0.063 | 0.014 | 9.26E-06 | 19.059 |
| genus | Oscillospira | rs3846286 | A | G | 0.114 | 0.026 | 9.24E-06 | 19.057 |
| genus | RuminococcaceaeUCG004 | rs550351 | C | A | 0.079 | 0.018 | 9.43E-06 | 19.055 |
| genus | Intestinimonas | rs2099538 | A | G | -0.164 | 0.038 | 7.45E-06 | 19.052 |
| genus | LachnospiraceaeNK4A136group | rs74953423 | A | T | 0.059 | 0.013 | 8.70E-06 | 19.049 |
| genus | Peptococcus | rs9458385 | C | A | 0.094 | 0.021 | 8.69E-06 | 19.043 |
| genus | Lactococcus | rs3731972 | T | C | -0.171 | 0.039 | 5.76E-06 | 19.037 |
| genus | RuminococcaceaeUCG009 | rs78410648 | G | A | 0.121 | 0.028 | 9.67E-06 | 19.034 |
| genus | Bilophila | rs4798126 | A | G | 0.073 | 0.017 | 7.15E-06 | 19.033 |
| genus |  | rs2466775 | C | T | 0.061 | 0.014 | 9.42E-06 | 19.029 |
| genus | Ruminococcus1 | rs3000856 | A | T | -0.071 | 0.016 | 9.28E-06 | 19.028 |
| genus | Ruminococcus1 | rs10995816 | G | C | -0.076 | 0.017 | 8.38E-06 | 19.028 |
| genus | Gordonibacter | rs72939513 | G | A | -0.214 | 0.049 | 7.98E-06 | 19.026 |
| genus | Prevotella7 | rs62115051 | G | A | 0.139 | 0.032 | 5.99E-06 | 19.025 |
| genus | Ruminococcus2 | rs6537939 | C | T | 0.070 | 0.016 | 7.57E-06 | 19.024 |
| genus | Butyricicoccus | rs2390280 | G | A | 0.080 | 0.018 | 8.65E-06 | 19.023 |
| genus | Dorea | rs2125444 | T | A | 0.053 | 0.012 | 6.97E-06 | 19.019 |
| genus | Romboutsia | rs12046777 | A | T | -0.086 | 0.020 | 8.32E-06 | 19.013 |
| genus | Bilophila | rs59441360 | A | C | 0.074 | 0.017 | 7.74E-06 | 19.010 |
| genus | RuminococcaceaeUCG002 | rs1044813 | G | C | -0.055 | 0.013 | 9.56E-06 | 19.009 |
| genus | Enterorhabdus | rs6431383 | C | T | 0.128 | 0.029 | 8.09E-06 | 19.009 |
| genus | ChristensenellaceaeR | rs10487398 | T | C | 0.111 | 0.025 | 8.06E-06 | 19.007 |
| genus | Alistipes | rs2450745 | C | A | -0.081 | 0.018 | 7.12E-06 | 19.007 |
| genus | Ruminococcus2 | rs7032486 | C | T | 0.070 | 0.016 | 7.80E-06 | 19.002 |
| genus | Bilophila | rs11081071 | T | C | 0.075 | 0.017 | 7.63E-06 | 19.001 |
| genus | Ruminiclostridium9 | rs9522731 | A | C | 0.068 | 0.016 | 9.38E-06 | 18.995 |
| genus | Butyricimonas | rs1701950 | C | G | -0.105 | 0.024 | 7.68E-06 | 18.995 |
| genus | Barnesiella | rs28479800 | T | A | -0.092 | 0.021 | 7.93E-06 | 18.993 |
| genus | Lactococcus | rs72799248 | T | A | -0.183 | 0.042 | 6.84E-06 | 18.991 |
| genus | Alistipes | rs17055871 | T | A | 0.074 | 0.017 | 5.19E-06 | 18.989 |
| genus | ChristensenellaceaeR | rs62467071 | C | T | 0.111 | 0.025 | 8.33E-06 | 18.987 |
| genus | CandidatusSoleaferrea | rs12374658 | T | C | -0.121 | 0.028 | 7.33E-06 | 18.983 |
| genus | Alistipes | rs2323487 | A | T | 0.074 | 0.017 | 5.52E-06 | 18.982 |
| genus | Turicibacter | rs112044427 | G | C | -0.118 | 0.027 | 9.88E-06 | 18.977 |
| genus | Ruminococcus2 | rs7032489 | C | G | 0.070 | 0.016 | 7.95E-06 | 18.977 |
| genus |  | rs58535382 | C | T | -0.171 | 0.039 | 4.31E-06 | 18.974 |
| genus | Paraprevotella | rs9547347 | A | G | -0.098 | 0.022 | 8.33E-06 | 18.973 |
| genus | CandidatusSoleaferrea | rs36155147 | T | C | 0.105 | 0.024 | 5.41E-06 | 18.971 |
| genus | Coprococcus2 | rs67222397 | G | C | 0.069 | 0.016 | 9.39E-06 | 18.965 |
| genus | RikenellaceaeRC9gutgroup | rs4270579 | A | G | -0.118 | 0.027 | 5.46E-06 | 18.960 |
| genus |  | rs78869071 | T | C | 0.098 | 0.023 | 5.59E-06 | 18.958 |
| genus | CandidatusSoleaferrea | rs12206634 | G | A | -0.122 | 0.028 | 8.25E-06 | 18.957 |
| genus | Ruminiclostridium6 | rs35362464 | A | C | 0.072 | 0.017 | 8.99E-06 | 18.956 |
| genus |  | rs56080211 | T | C | 0.123 | 0.028 | 9.14E-06 | 18.953 |
| genus | Roseburia | rs59070623 | T | C | -0.078 | 0.018 | 9.34E-06 | 18.952 |
| genus | Anaerostipes | rs10502061 | G | A | 0.084 | 0.019 | 7.94E-06 | 18.944 |
| genus | CandidatusSoleaferrea | rs10457187 | G | A | -0.121 | 0.028 | 8.05E-06 | 18.941 |
| genus |  | rs17009692 | A | C | 0.098 | 0.023 | 5.40E-06 | 18.940 |
| genus | Turicibacter | rs2287155 | G | A | -0.099 | 0.023 | 9.30E-06 | 18.935 |
| genus | Lactococcus | rs12623380 | T | C | -0.170 | 0.039 | 6.22E-06 | 18.934 |
| genus | Roseburia | . | A | G | -0.057 | 0.013 | 9.08E-06 | 18.931 |
| genus | Clostridiumsensustricto1 | rs118086821 | C | T | 0.107 | 0.025 | 8.15E-06 | 18.930 |
| genus | ChristensenellaceaeR | rs62467072 | C | T | 0.110 | 0.025 | 8.55E-06 | 18.929 |
| genus | RuminococcaceaeUCG002 | rs15256 | T | C | 0.073 | 0.017 | 9.46E-06 | 18.928 |
| genus |  | rs10147907 | G | T | 0.172 | 0.040 | 8.27E-06 | 18.922 |
| genus | Lactococcus | rs3213775 | A | G | -0.170 | 0.039 | 5.63E-06 | 18.919 |
| genus | Ruminiclostridium9 | rs9522726 | C | A | 0.067 | 0.015 | 9.87E-06 | 18.918 |
| genus |  | rs13154778 | A | T | 0.056 | 0.013 | 7.16E-06 | 18.917 |
| genus | Ruminiclostridium9 | rs10444611 | G | A | 0.067 | 0.015 | 9.96E-06 | 18.912 |
| genus |  | rs73849225 | C | T | 0.098 | 0.022 | 5.21E-06 | 18.908 |
| genus | Sellimonas | rs11686065 | G | C | -0.131 | 0.030 | 9.88E-06 | 18.906 |
| genus | RuminococcaceaeUCG005 | rs55793120 | C | T | 0.122 | 0.028 | 7.37E-06 | 18.905 |
| genus | Roseburia | rs61815620 | A | T | -0.077 | 0.018 | 9.45E-06 | 18.905 |
| genus |  | rs13155135 | A | G | 0.056 | 0.013 | 9.71E-06 | 18.904 |
| genus | ChristensenellaceaeR | rs12374987 | C | T | 0.110 | 0.025 | 8.51E-06 | 18.903 |
| genus |  | rs79970080 | A | G | -0.095 | 0.022 | 9.91E-06 | 18.903 |
| genus | Fusicatenibacter | rs12478647 | T | C | -0.069 | 0.016 | 8.09E-06 | 18.892 |
| genus | Ruminococcus2 | rs12000599 | C | A | 0.070 | 0.016 | 8.32E-06 | 18.890 |
| genus |  | rs75586835 | G | A | -0.114 | 0.026 | 9.39E-06 | 18.888 |
| genus | Lachnoclostridium | rs9901268 | G | A | 0.091 | 0.021 | 8.49E-06 | 18.885 |
| genus | Victivallis | rs342302 | G | A | -0.153 | 0.035 | 8.16E-06 | 18.878 |
| genus | CandidatusSoleaferrea | rs79465564 | C | T | -0.121 | 0.028 | 7.56E-06 | 18.875 |
| genus | Ruminiclostridium6 | rs663262 | C | T | -0.135 | 0.031 | 3.39E-06 | 18.872 |
| genus | Lactococcus | rs3731973 | G | A | -0.134 | 0.031 | 9.12E-06 | 18.860 |
| genus |  | rs73802842 | A | C | 0.074 | 0.017 | 7.48E-06 | 18.859 |
| genus |  | rs763963 | A | G | -0.094 | 0.022 | 8.59E-06 | 18.853 |
| genus | ChristensenellaceaeR | rs62467090 | A | G | 0.109 | 0.025 | 8.74E-06 | 18.846 |
| genus |  | rs62352462 | A | G | -0.164 | 0.038 | 5.07E-06 | 18.837 |
| genus | Peptococcus | rs1383810 | T | C | 0.127 | 0.029 | 6.17E-06 | 18.835 |
| genus | Ruminococcus2 | rs6537942 | A | G | 0.070 | 0.016 | 8.61E-06 | 18.834 |
| genus | CandidatusSoleaferrea | rs12198088 | C | T | -0.121 | 0.028 | 7.75E-06 | 18.831 |
| genus | CandidatusSoleaferrea | rs7759014 | A | G | -0.121 | 0.028 | 8.54E-06 | 18.824 |
| genus | LachnospiraceaeNK4A136group | rs138322411 | G | A | 0.058 | 0.013 | 9.43E-06 | 18.812 |
| genus | Alistipes | rs7337526 | C | T | 0.074 | 0.017 | 5.83E-06 | 18.810 |
| genus |  | rs62451661 | A | C | 0.072 | 0.017 | 9.19E-06 | 18.809 |
| genus | Turicibacter | rs2269746 | C | A | 0.132 | 0.030 | 9.06E-06 | 18.809 |
| genus | Coprococcus3 | rs11077359 | C | T | -0.065 | 0.015 | 9.64E-06 | 18.807 |
| genus | ChristensenellaceaeR | rs16870208 | C | T | 0.110 | 0.025 | 9.14E-06 | 18.803 |
| genus | Fusicatenibacter | rs62187627 | T | G | -0.068 | 0.016 | 9.08E-06 | 18.799 |
| genus | Lactococcus | rs10432674 | A | G | -0.134 | 0.031 | 8.99E-06 | 18.797 |
| genus | Tyzzerella3 | rs1405818 | A | G | 0.129 | 0.030 | 6.10E-06 | 18.796 |
| genus | Anaerotruncus | rs6772640 | G | A | -0.060 | 0.014 | 9.63E-06 | 18.789 |
| genus | Barnesiella | rs28380027 | C | T | -0.081 | 0.019 | 8.66E-06 | 18.788 |
| genus | Ruminiclostridium6 | rs12781529 | C | T | -0.094 | 0.022 | 8.03E-06 | 18.785 |
| genus | Ruminococcus2 | rs78786052 | T | A | 0.070 | 0.016 | 8.29E-06 | 18.781 |
| genus | ChristensenellaceaeR | rs62467082 | C | T | 0.109 | 0.025 | 9.00E-06 | 18.766 |
| genus | ChristensenellaceaeR | rs17141339 | A | G | 0.109 | 0.025 | 9.00E-06 | 18.766 |
| genus | Enterorhabdus | rs6740767 | T | C | 0.127 | 0.029 | 9.56E-06 | 18.765 |
| genus | Allisonella | rs685403 | C | G | -0.175 | 0.040 | 4.88E-06 | 18.763 |
| genus | Lactococcus | rs72799259 | C | T | -0.170 | 0.039 | 6.76E-06 | 18.757 |
| genus | Anaerotruncus | rs9603210 | A | G | 0.083 | 0.019 | 8.63E-06 | 18.751 |
| genus | Butyricimonas | rs67694451 | G | A | -0.086 | 0.020 | 7.70E-06 | 18.750 |
| genus | Barnesiella | rs28483632 | T | C | -0.081 | 0.019 | 8.84E-06 | 18.742 |
| genus | ChristensenellaceaeR | rs62467084 | T | C | 0.109 | 0.025 | 9.10E-06 | 18.737 |
| genus | ChristensenellaceaeR | rs62467085 | C | A | 0.109 | 0.025 | 9.10E-06 | 18.737 |
| genus | Parasutterella | rs575475 | G | A | 0.059 | 0.014 | 8.13E-06 | 18.733 |
| genus | Ruminiclostridium6 | rs77898140 | T | C | 0.106 | 0.024 | 9.17E-06 | 18.733 |
| genus | Streptococcus | rs11006959 | C | G | 0.069 | 0.016 | 7.08E-06 | 18.728 |
| genus | Romboutsia | rs12048003 | C | A | -0.086 | 0.020 | 9.15E-06 | 18.727 |
| genus | CandidatusSoleaferrea | rs11755471 | T | C | -0.121 | 0.028 | 8.76E-06 | 18.724 |
| genus | Alistipes | rs73180681 | C | T | 0.074 | 0.017 | 5.78E-06 | 18.714 |
| genus | Parabacteroides | rs9580476 | T | C | -0.108 | 0.025 | 9.36E-06 | 18.705 |
| genus | Fusicatenibacter | rs6014982 | C | T | -0.064 | 0.015 | 9.94E-06 | 18.696 |
| genus | Prevotella7 | rs60261717 | G | T | 0.143 | 0.033 | 9.85E-06 | 18.693 |
| genus | RuminococcaceaeUCG002 | rs10964441 | A | G | -0.149 | 0.034 | 7.45E-06 | 18.683 |
| genus | Howardella | rs3791893 | G | A | 0.147 | 0.034 | 9.50E-06 | 18.677 |
| genus | Ruminococcus2 | rs6537938 | G | A | 0.069 | 0.016 | 9.05E-06 | 18.670 |
| genus | Roseburia | rs57466170 | T | C | 0.074 | 0.017 | 8.30E-06 | 18.667 |
| genus | CandidatusSoleaferrea | rs12205061 | T | C | -0.120 | 0.028 | 8.81E-06 | 18.665 |
| genus | Parasutterella | rs7962688 | T | A | 0.059 | 0.014 | 8.57E-06 | 18.658 |
| genus | ChristensenellaceaeR | rs62467076 | A | G | 0.109 | 0.025 | 9.47E-06 | 18.658 |
| genus | Barnesiella | rs28575468 | A | T | -0.081 | 0.019 | 9.30E-06 | 18.650 |
| genus | Lactococcus | rs143307941 | C | A | -0.170 | 0.039 | 6.86E-06 | 18.648 |
| genus | Dialister | rs10138457 | C | T | -0.113 | 0.026 | 7.88E-06 | 18.629 |
| genus | Fusicatenibacter | rs61633745 | A | G | -0.068 | 0.016 | 9.45E-06 | 18.622 |
| genus | CandidatusSoleaferrea | rs12205053 | T | C | -0.120 | 0.028 | 9.16E-06 | 18.613 |
| genus | Lactococcus | rs72799268 | T | C | -0.169 | 0.039 | 6.94E-06 | 18.612 |
| genus |  | rs76557701 | G | A | 0.152 | 0.035 | 8.88E-06 | 18.608 |
| genus | Bilophila | rs3862170 | A | G | 0.074 | 0.017 | 8.67E-06 | 18.607 |
| genus | Blautia | rs2788271 | G | T | -0.058 | 0.013 | 7.16E-06 | 18.592 |
| genus | CandidatusSoleaferrea | rs75263286 | G | C | -0.121 | 0.028 | 9.22E-06 | 18.591 |
| genus |  | rs11940874 | G | A | 0.073 | 0.017 | 8.11E-06 | 18.590 |
| genus | Blautia | rs683119 | G | T | -0.058 | 0.013 | 7.46E-06 | 18.581 |
| genus | Tyzzerella3 | rs820796 | T | G | 0.123 | 0.029 | 5.68E-06 | 18.573 |
| genus | Romboutsia | rs79074610 | A | C | -0.086 | 0.020 | 9.38E-06 | 18.562 |
| genus | Alistipes | rs3764077 | A | G | 0.074 | 0.017 | 6.61E-06 | 18.539 |
| genus | Intestinibacter | rs72703447 | A | T | -0.101 | 0.024 | 7.50E-06 | 18.539 |
| genus |  | rs10904297 | G | A | -0.168 | 0.039 | 2.69E-06 | 18.531 |
| genus | Ruminiclostridium5 | rs4955951 | G | A | -0.071 | 0.017 | 9.96E-06 | 18.526 |
| genus |  | rs79860578 | T | C | 0.150 | 0.035 | 7.61E-06 | 18.523 |
| genus | Anaerostipes | rs13376554 | T | A | 0.197 | 0.046 | 7.72E-06 | 18.522 |
| genus | CandidatusSoleaferrea | rs12197912 | C | T | -0.120 | 0.028 | 9.90E-06 | 18.511 |
| genus | Intestinibacter | rs72703450 | C | A | -0.101 | 0.024 | 7.73E-06 | 18.511 |
| genus | Dialister | rs10132469 | G | A | -0.113 | 0.026 | 8.62E-06 | 18.500 |
| genus |  | rs17074066 | C | T | -0.081 | 0.019 | 9.35E-06 | 18.495 |
| genus | Tyzzerella3 | rs1527620 | C | T | 0.123 | 0.029 | 6.21E-06 | 18.482 |
| genus | Intestinibacter | rs62430350 | C | T | 0.151 | 0.035 | 6.84E-06 | 18.480 |
| genus | Intestinibacter | rs10811562 | T | C | -0.101 | 0.023 | 7.84E-06 | 18.453 |
| genus | RuminococcaceaeUCG005 | rs898577 | C | T | -0.123 | 0.029 | 7.46E-06 | 18.413 |
| genus | Intestinimonas | rs17067892 | T | C | 0.107 | 0.025 | 6.38E-06 | 18.383 |
| genus | Ruminiclostridium6 | rs648798 | C | T | -0.140 | 0.033 | 8.33E-06 | 18.379 |
| genus | Clostridiumsensustricto1 | rs484861 | G | A | -0.073 | 0.017 | 9.33E-06 | 18.361 |
| genus | Intestinibacter | rs10811563 | A | T | -0.101 | 0.024 | 8.27E-06 | 18.361 |
| genus | Dialister | rs6575890 | C | T | -0.112 | 0.026 | 9.58E-06 | 18.356 |
| genus | Anaerotruncus | rs875082 | T | A | 0.082 | 0.019 | 7.31E-06 | 18.312 |
| genus | Lactococcus | rs12623273 | G | A | -0.168 | 0.039 | 8.59E-06 | 18.302 |
| genus | Faecalibacterium | rs12753492 | C | A | 0.064 | 0.015 | 8.80E-06 | 18.295 |
| genus | Lactococcus | rs12621783 | C | T | -0.184 | 0.043 | 8.94E-06 | 18.292 |
| genus | Ruminiclostridium6 | rs677700 | G | A | -0.134 | 0.031 | 4.93E-06 | 18.287 |
| genus | Allisonella | rs593213 | A | G | -0.173 | 0.040 | 6.18E-06 | 18.279 |
| genus | Anaerotruncus | rs11018566 | G | A | -0.156 | 0.037 | 6.14E-06 | 18.272 |
| genus | Barnesiella | rs28827481 | C | T | -0.091 | 0.021 | 9.69E-06 | 18.244 |
| genus | Allisonella | rs487355 | G | A | -0.173 | 0.040 | 7.06E-06 | 18.218 |
| genus | LachnospiraceaeFCS020group | rs74515302 | G | A | -0.101 | 0.024 | 9.62E-06 | 18.191 |
| genus | Parabacteroides | rs11965579 | C | G | 0.163 | 0.038 | 8.87E-06 | 18.181 |
| genus | Peptococcus | rs4458693 | G | A | 0.124 | 0.029 | 8.05E-06 | 18.168 |
| genus | Intestinibacter | rs72703449 | C | A | -0.100 | 0.024 | 9.43E-06 | 18.147 |
| genus | Anaerotruncus | rs7338422 | A | G | 0.082 | 0.019 | 7.32E-06 | 18.129 |
| genus | Intestinibacter | rs12379721 | T | C | -0.099 | 0.023 | 7.63E-06 | 18.112 |
| genus | Lactococcus | rs72799262 | G | C | -0.167 | 0.039 | 9.94E-06 | 18.057 |
| genus | LachnospiraceaeFCS020group | rs2061129 | A | G | -0.101 | 0.024 | 8.67E-06 | 18.054 |
| genus | Alistipes | rs2290844 | T | C | 0.081 | 0.019 | 9.10E-06 | 18.042 |
| genus | Streptococcus | rs9919516 | A | G | 0.068 | 0.016 | 9.35E-06 | 18.036 |
| genus | CandidatusSoleaferrea | rs74188131 | T | G | 0.102 | 0.024 | 8.06E-06 | 18.031 |
| genus | Anaerotruncus | rs12584670 | T | C | 0.081 | 0.019 | 8.98E-06 | 17.816 |
| genus | Coprococcus2 | rs10121347 | G | C | 0.093 | 0.022 | 8.31E-06 | 17.796 |
| genus | CandidatusSoleaferrea | rs74214552 | C | G | 0.102 | 0.024 | 9.89E-06 | 17.787 |
| genus |  | rs182318 | A | G | -0.082 | 0.020 | 8.40E-06 | 17.785 |
| genus | Intestinibacter | rs12378286 | G | A | -0.098 | 0.023 | 9.23E-06 | 17.760 |
| genus | Intestinibacter | rs12379709 | T | A | -0.098 | 0.023 | 9.27E-06 | 17.753 |
| genus | Anaerotruncus | rs9594214 | C | T | 0.081 | 0.019 | 9.92E-06 | 17.699 |
| genus | Erysipelatoclostridium | rs3804326 | G | A | 0.141 | 0.034 | 9.85E-06 | 17.691 |
| genus | Faecalibacterium | rs71629856 | G | A | 0.061 | 0.015 | 9.39E-06 | 17.685 |
| genus |  | rs10521010 | T | C | -0.163 | 0.039 | 8.87E-06 | 17.596 |
| genus | RuminococcaceaeNK4A214group | rs6681678 | T | C | -0.100 | 0.024 | 9.05E-06 | 17.422 |
| genus | Clostridiumsensustricto1 | rs11264403 | A | G | -0.139 | 0.033 | 7.76E-06 | 17.288 |
| genus | Prevotella7 | rs16937247 | C | G | 0.146 | 0.035 | 9.64E-06 | 17.273 |
| genus | Alistipes | rs7995332 | C | A | 0.071 | 0.017 | 8.97E-06 | 17.245 |
| genus |  | rs72839198 | G | C | 0.156 | 0.038 | 3.14E-06 | 17.174 |
| genus | Lachnoclostridium | rs2385421 | G | A | 0.075 | 0.018 | 7.14E-06 | 17.046 |
| genus | RuminococcaceaeUCG002 | rs10898505 | T | C | 0.062 | 0.015 | 9.94E-06 | 17.004 |
| genus | ErysipelotrichaceaeUCG003 | rs74988980 | A | G | -0.133 | 0.035 | 8.64E-06 | 14.589 |
| order | Bifidobacteriales | rs182549 | T | C | 0.117 | 0.013 | 5.94E-20 | 85.372 |
| order | Bifidobacteriales | rs4988235 | A | G | 0.116 | 0.013 | 1.30E-19 | 84.054 |
| order | Bifidobacteriales | rs7570971 | C | A | 0.113 | 0.013 | 2.53E-19 | 80.781 |
| order | Bifidobacteriales | rs6754311 | T | C | 0.113 | 0.013 | 8.77E-19 | 79.915 |
| order | Bifidobacteriales | rs1446585 | A | G | 0.112 | 0.013 | 4.82E-18 | 76.954 |
| order | Bifidobacteriales | rs1375131 | T | C | 0.110 | 0.013 | 3.94E-18 | 76.803 |
| order | Bifidobacteriales | rs6730157 | A | G | 0.109 | 0.013 | 4.20E-18 | 76.629 |
| order | Bifidobacteriales | rs12465802 | G | A | 0.110 | 0.013 | 6.15E-18 | 75.683 |
| order | Bifidobacteriales | rs687670 | T | C | 0.111 | 0.013 | 2.49E-17 | 74.007 |
| order | Bifidobacteriales | rs13404551 | T | C | 0.110 | 0.013 | 7.02E-17 | 72.595 |
| order | Bifidobacteriales | rs36084664 | A | G | -0.113 | 0.014 | 1.45E-18 | 70.438 |
| order | Bifidobacteriales | rs309160 | C | T | 0.108 | 0.013 | 3.14E-16 | 69.414 |
| order | Bifidobacteriales | rs309168 | C | T | 0.107 | 0.013 | 5.10E-16 | 68.411 |
| order | Bifidobacteriales | rs218174 | A | G | 0.107 | 0.013 | 7.25E-16 | 68.090 |
| order | Bifidobacteriales | rs309134 | G | A | 0.107 | 0.013 | 1.35E-15 | 66.643 |
| order | Bifidobacteriales | rs309180 | A | G | 0.105 | 0.013 | 1.36E-15 | 66.493 |
| order | Bifidobacteriales | rs632632 | T | C | 0.105 | 0.013 | 2.42E-15 | 65.527 |
| order | Bifidobacteriales | rs12615624 | A | G | 0.105 | 0.013 | 4.78E-15 | 64.191 |
| order | Bifidobacteriales | rs6759321 | G | T | 0.101 | 0.013 | 4.00E-15 | 63.872 |
| order | Bifidobacteriales | rs309125 | C | T | 0.103 | 0.013 | 6.64E-15 | 63.343 |
| order | Bifidobacteriales | rs13023281 | T | C | -0.106 | 0.013 | 1.12E-16 | 62.561 |
| order | Bifidobacteriales | rs1561277 | C | A | 0.100 | 0.013 | 1.28E-14 | 61.678 |
| order | Bifidobacteriales | rs309164 | T | C | 0.103 | 0.013 | 1.74E-14 | 61.436 |
| order | Bifidobacteriales | rs309149 | C | T | 0.101 | 0.013 | 4.39E-14 | 59.450 |
| order | Bifidobacteriales | rs4988183 | T | C | 0.102 | 0.013 | 5.12E-14 | 59.384 |
| order | Bifidobacteriales | rs4954490 | A | G | 0.100 | 0.013 | 5.25E-14 | 59.153 |
| order | Bifidobacteriales | rs6716536 | C | T | 0.098 | 0.013 | 3.56E-14 | 59.134 |
| order | Bifidobacteriales | rs309137 | T | C | 0.099 | 0.013 | 5.22E-14 | 58.752 |
| order | Bifidobacteriales | rs2236783 | A | G | 0.099 | 0.013 | 1.15E-13 | 57.801 |
| order | Bifidobacteriales | rs11681248 | T | C | 0.094 | 0.012 | 9.61E-14 | 57.117 |
| order | Bifidobacteriales | rs12620033 | G | C | 0.098 | 0.013 | 1.68E-13 | 56.975 |
| order | Bifidobacteriales | rs3754686 | C | T | 0.097 | 0.013 | 2.82E-13 | 55.615 |
| order | Bifidobacteriales | rs3769005 | G | C | 0.096 | 0.013 | 5.29E-13 | 54.165 |
| order | Bifidobacteriales | rs745500 | A | G | 0.096 | 0.013 | 6.29E-13 | 54.148 |
| order | Bifidobacteriales | rs6719488 | T | G | 0.096 | 0.013 | 6.53E-13 | 53.989 |
| order | Bifidobacteriales | rs12475139 | T | A | 0.094 | 0.013 | 5.51E-13 | 53.702 |
| order | Bifidobacteriales | rs892715 | C | T | 0.095 | 0.013 | 7.89E-13 | 53.593 |
| order | Bifidobacteriales | rs12988076 | A | C | 0.095 | 0.013 | 8.81E-13 | 53.419 |
| order | Bifidobacteriales | rs2322660 | T | C | 0.095 | 0.013 | 9.97E-13 | 53.059 |
| order | Bifidobacteriales | rs6742283 | G | A | 0.095 | 0.013 | 1.13E-12 | 52.793 |
| order | Bifidobacteriales | rs2164210 | C | T | 0.094 | 0.013 | 1.14E-12 | 52.750 |
| order | Bifidobacteriales | rs749873 | T | C | 0.090 | 0.012 | 8.79E-13 | 52.662 |
| order | Bifidobacteriales | rs748841 | T | C | 0.094 | 0.013 | 1.26E-12 | 52.652 |
| order | Bifidobacteriales | rs17261772 | T | C | 0.095 | 0.013 | 1.55E-12 | 52.416 |
| order | Bifidobacteriales | rs2011946 | A | C | 0.090 | 0.013 | 1.21E-12 | 52.215 |
| order | Bifidobacteriales | rs3739020 | T | G | 0.095 | 0.013 | 3.46E-12 | 50.709 |
| order | Bifidobacteriales | rs2082729 | T | C | 0.093 | 0.013 | 6.59E-12 | 49.356 |
| order | Bifidobacteriales | rs11693502 | T | C | 0.088 | 0.013 | 4.47E-12 | 49.178 |
| order | Bifidobacteriales | rs71348715 | T | G | 0.092 | 0.013 | 8.94E-12 | 48.731 |
| order | Bifidobacteriales | rs1438305 | G | C | 0.092 | 0.013 | 9.19E-12 | 48.673 |
| order | Bifidobacteriales | rs1011361 | A | G | 0.092 | 0.013 | 8.86E-12 | 48.660 |
| order | Bifidobacteriales | rs10202489 | A | G | 0.092 | 0.013 | 9.56E-12 | 48.545 |
| order | Bifidobacteriales | rs313519 | A | C | 0.092 | 0.013 | 8.33E-12 | 48.420 |
| order | Bifidobacteriales | rs11684545 | T | G | 0.092 | 0.013 | 1.02E-11 | 48.386 |
| order | Bifidobacteriales | rs71348714 | A | G | 0.092 | 0.013 | 9.53E-12 | 48.288 |
| order | Bifidobacteriales | rs12998016 | T | C | 0.092 | 0.013 | 1.05E-11 | 48.278 |
| order | Bifidobacteriales | rs6430589 | C | G | 0.092 | 0.013 | 9.08E-12 | 48.250 |
| order | Bifidobacteriales | rs1438304 | G | A | 0.092 | 0.013 | 1.18E-11 | 48.171 |
| order | Bifidobacteriales | rs313518 | C | T | 0.092 | 0.013 | 1.03E-11 | 48.167 |
| order | Bifidobacteriales | rs10928546 | T | C | 0.092 | 0.013 | 1.18E-11 | 48.156 |
| order | Bifidobacteriales | rs1438307 | T | G | 0.092 | 0.013 | 1.16E-11 | 48.137 |
| order | Bifidobacteriales | rs28453840 | A | G | 0.092 | 0.013 | 1.24E-11 | 48.074 |
| order | Bifidobacteriales | rs313528 | G | A | 0.091 | 0.013 | 1.04E-11 | 48.053 |
| order | Bifidobacteriales | rs10195620 | T | A | 0.092 | 0.013 | 1.21E-11 | 48.023 |
| order | Bifidobacteriales | rs313526 | C | T | 0.091 | 0.013 | 1.09E-11 | 47.983 |
| order | Bifidobacteriales | rs11679933 | T | G | 0.087 | 0.013 | 1.00E-11 | 47.873 |
| order | Bifidobacteriales | rs13412397 | A | G | 0.091 | 0.013 | 1.33E-11 | 47.857 |
| order | Bifidobacteriales | rs12998387 | G | A | 0.091 | 0.013 | 1.50E-11 | 47.708 |
| order | Bifidobacteriales | rs313524 | C | T | 0.091 | 0.013 | 1.33E-11 | 47.520 |
| order | Bifidobacteriales | rs3213889 | G | A | 0.091 | 0.013 | 1.68E-11 | 47.511 |
| order | Bifidobacteriales | rs313517 | A | G | 0.091 | 0.013 | 1.38E-11 | 47.493 |
| order | Bifidobacteriales | rs1470457 | A | G | 0.092 | 0.013 | 1.74E-11 | 47.404 |
| order | Bifidobacteriales | rs7355359 | T | C | 0.091 | 0.013 | 1.50E-11 | 47.383 |
| order | Bifidobacteriales | rs12994270 | A | T | 0.091 | 0.013 | 2.13E-11 | 47.110 |
| order | Bifidobacteriales | rs4954280 | A | G | 0.091 | 0.013 | 1.70E-11 | 47.083 |
| order | Bifidobacteriales | rs1446584 | A | G | 0.090 | 0.013 | 1.75E-11 | 47.002 |
| order | Bifidobacteriales | rs10173394 | T | C | 0.091 | 0.013 | 2.21E-11 | 46.996 |
| order | Bifidobacteriales | rs2034276 | A | T | 0.090 | 0.013 | 1.77E-11 | 46.968 |
| order | Bifidobacteriales | rs11679508 | T | A | 0.090 | 0.013 | 2.21E-11 | 46.843 |
| order | Bifidobacteriales | rs6720287 | A | G | 0.090 | 0.013 | 1.88E-11 | 46.839 |
| order | Bifidobacteriales | rs313522 | T | A | 0.090 | 0.013 | 2.09E-11 | 46.597 |
| order | Bifidobacteriales | rs313523 | G | A | 0.090 | 0.013 | 2.08E-11 | 46.591 |
| order | Bifidobacteriales | rs6715856 | T | C | 0.090 | 0.013 | 2.26E-11 | 46.471 |
| order | Bifidobacteriales | rs6755383 | C | T | 0.090 | 0.013 | 2.25E-11 | 46.446 |
| order | Bifidobacteriales | rs12469709 | T | C | 0.090 | 0.013 | 2.72E-11 | 46.390 |
| order | Bifidobacteriales | rs656326 | A | C | 0.090 | 0.013 | 2.34E-11 | 46.376 |
| order | Bifidobacteriales | rs10192827 | A | G | 0.090 | 0.013 | 3.91E-11 | 45.970 |
| order | Bifidobacteriales | rs932206 | T | C | 0.082 | 0.012 | 1.88E-11 | 45.838 |
| order | Bifidobacteriales | rs4954276 | A | G | 0.089 | 0.013 | 3.83E-11 | 45.669 |
| order | Bifidobacteriales | rs10928542 | G | A | 0.089 | 0.013 | 3.35E-11 | 45.654 |
| order | Bifidobacteriales | rs7608045 | T | C | 0.089 | 0.013 | 3.58E-11 | 45.505 |
| order | Bifidobacteriales | rs4954279 | G | A | 0.089 | 0.013 | 4.26E-11 | 45.169 |
| order | Bifidobacteriales | rs2322659 | C | T | 0.089 | 0.013 | 4.94E-11 | 44.875 |
| order | Bifidobacteriales | rs13413639 | C | A | 0.088 | 0.013 | 5.78E-11 | 44.688 |
| order | Bifidobacteriales | rs12619365 | C | T | 0.088 | 0.013 | 5.99E-11 | 44.387 |
| order | Bifidobacteriales | rs2680880 | T | A | 0.080 | 0.012 | 9.86E-11 | 44.287 |
| order | Bifidobacteriales | rs1869829 | A | G | 0.086 | 0.013 | 6.87E-11 | 43.596 |
| order | Bifidobacteriales | rs7587285 | T | C | 0.116 | 0.018 | 3.39E-11 | 43.348 |
| order | Bifidobacteriales | rs2278683 | A | C | 0.115 | 0.018 | 5.39E-11 | 42.739 |
| order | Bifidobacteriales | rs10187402 | C | G | 0.086 | 0.013 | 9.54E-11 | 42.692 |
| order | Bifidobacteriales | rs4954551 | G | A | 0.115 | 0.018 | 5.00E-11 | 42.631 |
| order | Bifidobacteriales | rs11893318 | T | C | 0.115 | 0.018 | 5.47E-11 | 42.486 |
| order | Bifidobacteriales | rs1375132 | A | G | 0.085 | 0.013 | 1.49E-10 | 42.274 |
| order | Bifidobacteriales | rs62160860 | G | C | 0.116 | 0.018 | 7.86E-11 | 42.149 |
| order | Bifidobacteriales | rs935613 | C | T | 0.084 | 0.013 | 2.63E-10 | 40.991 |
| order | Bifidobacteriales | rs2016636 | G | A | 0.084 | 0.013 | 3.23E-10 | 40.741 |
| order | Bifidobacteriales | rs6714750 | A | G | 0.084 | 0.013 | 6.28E-10 | 39.957 |
| order | Bifidobacteriales | rs1374329 | A | G | 0.085 | 0.013 | 5.57E-10 | 39.908 |
| order | Bifidobacteriales | rs10928545 | G | A | 0.086 | 0.014 | 8.21E-10 | 39.645 |
| order | Bifidobacteriales | rs1979033 | G | A | 0.075 | 0.012 | 1.05E-10 | 39.424 |
| order | Bifidobacteriales | rs9287442 | G | A | 0.085 | 0.014 | 1.05E-09 | 39.050 |
| order | Bifidobacteriales | rs12328663 | T | C | 0.103 | 0.017 | 1.19E-10 | 38.929 |
| order | Bifidobacteriales | rs2304599 | G | C | 0.085 | 0.014 | 1.12E-09 | 38.856 |
| order | Bifidobacteriales | rs2874739 | C | T | 0.103 | 0.017 | 1.71E-10 | 38.596 |
| order | Bifidobacteriales | rs12616520 | T | A | 0.084 | 0.014 | 1.34E-09 | 38.570 |
| order | Bifidobacteriales | rs6760329 | G | A | 0.085 | 0.014 | 1.21E-09 | 38.501 |
| order | Bifidobacteriales | rs10193348 | A | G | 0.103 | 0.017 | 1.70E-10 | 38.500 |
| order | Bifidobacteriales | rs4953945 | C | G | 0.103 | 0.017 | 2.92E-10 | 38.497 |
| order | Bifidobacteriales | rs6747073 | T | C | 0.081 | 0.013 | 9.76E-10 | 38.409 |
| order | Bifidobacteriales | rs10188066 | A | G | 0.084 | 0.014 | 1.35E-09 | 38.317 |
| order | Bifidobacteriales | rs1530559 | A | G | 0.074 | 0.012 | 5.23E-10 | 38.029 |
| order | Bifidobacteriales | rs4954513 | G | A | 0.112 | 0.018 | 1.50E-09 | 37.669 |
| order | Bifidobacteriales | rs309126 | T | C | 0.105 | 0.017 | 1.84E-09 | 37.634 |
| order | Bifidobacteriales | rs7568525 | G | A | 0.102 | 0.017 | 2.11E-10 | 37.603 |
| order | Bifidobacteriales | rs13389481 | C | A | 0.104 | 0.017 | 3.31E-10 | 37.518 |
| order | Bifidobacteriales | rs1030764 | T | C | 0.084 | 0.014 | 2.11E-09 | 37.511 |
| order | Bifidobacteriales | rs4346445 | A | C | 0.102 | 0.017 | 2.30E-10 | 37.502 |
| order | Bifidobacteriales | rs10208624 | C | T | 0.101 | 0.017 | 2.26E-10 | 37.480 |
| order | Bifidobacteriales | rs62168942 | T | C | 0.102 | 0.017 | 3.65E-10 | 37.442 |
| order | Bifidobacteriales | rs4954217 | C | T | 0.101 | 0.017 | 3.68E-10 | 37.435 |
| order | Bifidobacteriales | rs58697996 | G | A | 0.101 | 0.017 | 3.09E-10 | 37.287 |
| order | Bifidobacteriales | rs6704561 | C | T | 0.079 | 0.013 | 1.57E-09 | 37.236 |
| order | Bifidobacteriales | rs4954216 | G | A | 0.101 | 0.017 | 3.98E-10 | 37.231 |
| order | Bifidobacteriales | rs10167901 | C | T | 0.101 | 0.017 | 3.96E-10 | 37.230 |
| order | Bifidobacteriales | rs7581382 | T | C | -0.090 | 0.015 | 5.00E-11 | 37.205 |
| order | Bifidobacteriales | rs4953948 | T | G | 0.101 | 0.017 | 3.99E-10 | 37.187 |
| order | Bifidobacteriales | rs1551497 | T | C | 0.101 | 0.017 | 4.34E-10 | 37.157 |
| order | Bifidobacteriales | rs10167809 | C | T | 0.101 | 0.017 | 4.08E-10 | 37.153 |
| order | Bifidobacteriales | rs10167884 | C | T | 0.101 | 0.017 | 4.08E-10 | 37.151 |
| order | Bifidobacteriales | rs7422031 | C | T | 0.101 | 0.017 | 3.80E-10 | 37.135 |
| order | Bifidobacteriales | rs921859 | A | G | 0.101 | 0.017 | 4.38E-10 | 37.106 |
| order | Bifidobacteriales | rs7601696 | C | A | 0.101 | 0.017 | 4.52E-10 | 37.101 |
| order | Bifidobacteriales | rs4954215 | G | A | 0.101 | 0.017 | 4.26E-10 | 37.077 |
| order | Bifidobacteriales | rs7577258 | G | A | 0.101 | 0.017 | 4.61E-10 | 37.065 |
| order | Bifidobacteriales | rs62168941 | C | T | 0.101 | 0.017 | 4.64E-10 | 37.021 |
| order | Bifidobacteriales | rs4953947 | A | C | 0.101 | 0.017 | 4.52E-10 | 36.964 |
| order | Bifidobacteriales | rs2017831 | C | T | 0.101 | 0.017 | 4.80E-10 | 36.958 |
| order | Bifidobacteriales | rs61087958 | C | T | 0.101 | 0.017 | 3.67E-10 | 36.944 |
| order | Bifidobacteriales | rs7573600 | G | C | 0.100 | 0.017 | 3.51E-10 | 36.934 |
| order | Bifidobacteriales | rs3820794 | T | C | 0.083 | 0.014 | 2.82E-09 | 36.933 |
| order | Bifidobacteriales | rs7565268 | T | C | 0.101 | 0.017 | 5.02E-10 | 36.923 |
| order | Bifidobacteriales | rs13409400 | A | C | 0.101 | 0.017 | 4.93E-10 | 36.917 |
| order | Bifidobacteriales | rs56903548 | C | T | 0.100 | 0.017 | 4.29E-10 | 36.876 |
| order | Bifidobacteriales | rs10182750 | T | C | 0.101 | 0.017 | 4.99E-10 | 36.839 |
| order | Bifidobacteriales | rs13402101 | T | A | 0.101 | 0.017 | 5.19E-10 | 36.787 |
| order | Bifidobacteriales | rs13399376 | A | G | 0.101 | 0.017 | 5.19E-10 | 36.787 |
| order | Bifidobacteriales | rs11890844 | A | G | 0.101 | 0.017 | 5.28E-10 | 36.745 |
| order | Bifidobacteriales | rs3213871 | C | T | 0.111 | 0.018 | 1.92E-09 | 36.716 |
| order | Bifidobacteriales | rs7608980 | G | A | 0.110 | 0.018 | 2.78E-09 | 36.715 |
| order | Bifidobacteriales | rs16832138 | G | A | 0.110 | 0.018 | 2.83E-09 | 36.710 |
| order | Bifidobacteriales | rs2278544 | G | A | 0.082 | 0.014 | 3.42E-09 | 36.696 |
| order | Bifidobacteriales | rs3769002 | T | C | 0.110 | 0.018 | 2.22E-09 | 36.695 |
| order | Bifidobacteriales | rs6738563 | G | T | 0.073 | 0.012 | 4.67E-10 | 36.687 |
| order | Bifidobacteriales | rs7603805 | A | G | 0.100 | 0.017 | 4.43E-10 | 36.674 |
| order | Bifidobacteriales | rs4954220 | C | A | 0.100 | 0.017 | 3.75E-10 | 36.622 |
| order | Bifidobacteriales | rs13402112 | A | C | 0.100 | 0.017 | 5.56E-10 | 36.589 |
| order | Bifidobacteriales | rs4954214 | C | T | 0.100 | 0.017 | 5.48E-10 | 36.573 |
| order | Bifidobacteriales | rs6741481 | A | G | 0.100 | 0.017 | 4.46E-10 | 36.504 |
| order | Bifidobacteriales | rs10186594 | A | G | 0.100 | 0.017 | 6.00E-10 | 36.499 |
| order | Bifidobacteriales | rs56263017 | T | C | 0.110 | 0.018 | 2.38E-09 | 36.485 |
| order | Bifidobacteriales | rs4954212 | A | G | 0.100 | 0.017 | 6.59E-10 | 36.479 |
| order | Bifidobacteriales | rs7601683 | C | T | 0.100 | 0.017 | 6.26E-10 | 36.474 |
| order | Bifidobacteriales | rs11898045 | T | C | 0.100 | 0.017 | 4.32E-10 | 36.379 |
| order | Bifidobacteriales | rs6430561 | C | T | 0.100 | 0.017 | 4.49E-10 | 36.360 |
| order | Bifidobacteriales | rs58426504 | T | G | 0.100 | 0.017 | 4.37E-10 | 36.349 |
| order | Bifidobacteriales | rs7565149 | T | A | 0.100 | 0.017 | 6.78E-10 | 36.315 |
| order | Bifidobacteriales | rs6760553 | A | G | 0.100 | 0.017 | 6.22E-10 | 36.287 |
| order | Bifidobacteriales | rs56174523 | A | C | 0.100 | 0.017 | 6.61E-10 | 36.268 |
| order | Bifidobacteriales | rs13413872 | G | T | 0.100 | 0.017 | 6.60E-10 | 36.262 |
| order | Bifidobacteriales | rs11884903 | T | C | 0.101 | 0.017 | 5.72E-10 | 36.251 |
| order | Bifidobacteriales | rs16832275 | C | G | 0.106 | 0.018 | 2.11E-09 | 36.249 |
| order | Bifidobacteriales | rs16832156 | G | A | 0.106 | 0.018 | 2.47E-09 | 36.226 |
| order | Bifidobacteriales | rs4953946 | T | G | 0.100 | 0.017 | 6.49E-10 | 36.166 |
| order | Bifidobacteriales | rs56067372 | C | T | 0.109 | 0.018 | 3.81E-09 | 36.094 |
| order | Bifidobacteriales | rs58327215 | G | C | 0.099 | 0.017 | 4.92E-10 | 36.066 |
| order | Bifidobacteriales | rs57829242 | A | G | 0.101 | 0.017 | 6.48E-10 | 36.060 |
| order | Bifidobacteriales | rs11888951 | T | C | 0.100 | 0.017 | 7.06E-10 | 36.044 |
| order | Bifidobacteriales | rs60350840 | A | G | 0.100 | 0.017 | 8.18E-10 | 35.982 |
| order | Bifidobacteriales | rs6723108 | G | T | 0.073 | 0.012 | 7.70E-10 | 35.971 |
| order | Bifidobacteriales | rs11892750 | G | A | 0.100 | 0.017 | 9.41E-10 | 35.970 |
| order | Bifidobacteriales | rs3768998 | G | T | 0.109 | 0.018 | 1.71E-09 | 35.968 |
| order | Bifidobacteriales | rs11903663 | A | G | 0.099 | 0.017 | 3.66E-10 | 35.926 |
| order | Bifidobacteriales | rs2290518 | G | A | 0.099 | 0.017 | 6.07E-10 | 35.918 |
| order | Bifidobacteriales | rs10174944 | C | T | 0.099 | 0.017 | 8.15E-10 | 35.909 |
| order | Bifidobacteriales | rs10209482 | A | C | 0.100 | 0.017 | 7.31E-10 | 35.786 |
| order | Bifidobacteriales | rs4640444 | T | C | 0.099 | 0.017 | 4.39E-10 | 35.678 |
| order | Bifidobacteriales | rs62168934 | A | G | 0.099 | 0.017 | 9.43E-10 | 35.644 |
| order | Bifidobacteriales | rs16831264 | G | C | 0.099 | 0.017 | 9.62E-10 | 35.529 |
| order | Bifidobacteriales | rs4645053 | T | A | 0.099 | 0.017 | 4.77E-10 | 35.526 |
| order | Bifidobacteriales | rs6721838 | A | G | 0.099 | 0.017 | 6.53E-10 | 35.525 |
| order | Bifidobacteriales | rs2164332 | G | C | 0.104 | 0.018 | 3.03E-09 | 35.435 |
| order | Bifidobacteriales | rs925966 | G | C | 0.076 | 0.013 | 4.52E-09 | 35.335 |
| order | Bifidobacteriales | rs13427494 | G | A | 0.100 | 0.017 | 9.35E-10 | 35.309 |
| order | Bifidobacteriales | rs309132 | C | G | 0.107 | 0.018 | 5.94E-09 | 35.301 |
| order | Bifidobacteriales | rs16831498 | G | C | 0.100 | 0.017 | 9.40E-10 | 35.267 |
| order | Bifidobacteriales | rs13410870 | G | A | 0.098 | 0.017 | 8.43E-10 | 35.171 |
| order | Bifidobacteriales | rs6709763 | C | A | 0.098 | 0.017 | 1.36E-09 | 35.038 |
| order | Bifidobacteriales | rs6752519 | C | T | 0.107 | 0.018 | 4.92E-09 | 34.988 |
| order | Bifidobacteriales | rs6739713 | A | G | 0.079 | 0.013 | 7.39E-09 | 34.953 |
| order | Bifidobacteriales | rs13408096 | G | T | 0.098 | 0.017 | 1.37E-09 | 34.911 |
| order | Bifidobacteriales | rs4494812 | A | G | 0.098 | 0.017 | 7.34E-10 | 34.837 |
| order | Bifidobacteriales | rs4954226 | T | C | 0.099 | 0.017 | 1.17E-09 | 34.697 |
| order | Bifidobacteriales | rs6430593 | G | A | 0.103 | 0.018 | 6.67E-09 | 34.693 |
| order | Bifidobacteriales | rs7371606 | A | G | 0.075 | 0.013 | 6.42E-09 | 34.659 |
| order | Bifidobacteriales | rs6737635 | G | T | 0.098 | 0.017 | 1.65E-09 | 34.645 |
| order | Bifidobacteriales | rs2322725 | T | C | 0.103 | 0.018 | 4.38E-09 | 34.604 |
| order | Bifidobacteriales | rs4954213 | G | T | 0.097 | 0.017 | 1.58E-09 | 34.601 |
| order | Bifidobacteriales | rs6749119 | C | G | 0.097 | 0.017 | 1.10E-09 | 34.550 |
| order | Bifidobacteriales | rs11897204 | T | C | 0.097 | 0.017 | 1.10E-09 | 34.525 |
| order | Bifidobacteriales | rs3754692 | T | C | 0.079 | 0.013 | 9.24E-09 | 34.474 |
| order | Bifidobacteriales | rs7597802 | A | T | 0.097 | 0.017 | 2.04E-09 | 34.314 |
| order | Bifidobacteriales | rs4954221 | G | A | 0.097 | 0.017 | 1.19E-09 | 34.256 |
| order | Bifidobacteriales | rs7563997 | T | A | 0.097 | 0.017 | 2.04E-09 | 34.202 |
| order | Bifidobacteriales | rs11888521 | G | C | 0.097 | 0.017 | 1.02E-09 | 34.158 |
| order | Bifidobacteriales | rs10928529 | G | A | 0.097 | 0.017 | 2.20E-09 | 34.128 |
| order | Bifidobacteriales | rs17293519 | A | G | 0.096 | 0.017 | 1.72E-09 | 34.114 |
| order | Bifidobacteriales | rs11900832 | C | T | 0.097 | 0.017 | 2.27E-09 | 34.111 |
| order | Bifidobacteriales | rs4954242 | A | G | 0.098 | 0.017 | 2.41E-09 | 34.080 |
| order | Bifidobacteriales | rs3754690 | C | T | 0.110 | 0.019 | 8.00E-09 | 33.984 |
| order | Bifidobacteriales | rs2164331 | C | T | 0.106 | 0.018 | 7.53E-09 | 33.959 |
| order | Bifidobacteriales | rs11889843 | G | A | 0.096 | 0.017 | 2.45E-09 | 33.863 |
| order | Bifidobacteriales | rs16832200 | T | C | 0.102 | 0.018 | 6.78E-09 | 33.849 |
| order | Bifidobacteriales | rs6706466 | A | G | 0.096 | 0.017 | 1.54E-09 | 33.819 |
| order | Bifidobacteriales | rs61663334 | A | G | 0.096 | 0.017 | 1.18E-09 | 33.656 |
| order | Bifidobacteriales | rs6742013 | T | C | 0.097 | 0.017 | 2.16E-09 | 33.637 |
| order | Bifidobacteriales | rs1900741 | C | T | 0.083 | 0.014 | 1.91E-08 | 33.624 |
| order | Bifidobacteriales | rs2305248 | A | G | 0.096 | 0.017 | 1.82E-09 | 33.567 |
| order | Bifidobacteriales | rs56369660 | A | G | 0.096 | 0.017 | 2.64E-09 | 33.526 |
| order | Bifidobacteriales | rs12233092 | G | A | 0.102 | 0.018 | 8.26E-09 | 33.473 |
| order | Bifidobacteriales | rs7605229 | C | T | 0.097 | 0.017 | 2.77E-09 | 33.296 |
| order | Bifidobacteriales | rs12478902 | T | C | 0.103 | 0.018 | 9.81E-09 | 33.170 |
| order | Bifidobacteriales | rs2874834 | A | G | 0.077 | 0.013 | 1.94E-08 | 33.095 |
| order | Bifidobacteriales | rs16855656 | A | G | 0.099 | 0.017 | 1.42E-08 | 33.086 |
| order | Bifidobacteriales | rs4954567 | G | T | 0.072 | 0.013 | 9.64E-09 | 33.032 |
| order | Bifidobacteriales | rs3806502 | C | T | 0.096 | 0.017 | 2.90E-09 | 32.981 |
| order | Bifidobacteriales | rs55634455 | T | C | 0.108 | 0.019 | 1.32E-08 | 32.844 |
| order | Bifidobacteriales | rs10179271 | T | C | 0.072 | 0.013 | 1.16E-08 | 32.657 |
| order | Bifidobacteriales | rs6727889 | C | T | 0.096 | 0.017 | 4.85E-09 | 32.629 |
| order | Bifidobacteriales | rs584226 | T | C | 0.101 | 0.018 | 1.91E-08 | 32.529 |
| order | Bifidobacteriales | rs1401687 | G | C | 0.072 | 0.013 | 1.31E-08 | 32.413 |
| order | Bifidobacteriales | rs78141345 | G | A | 0.107 | 0.019 | 1.77E-08 | 32.303 |
| order | Bifidobacteriales | rs4954568 | T | A | 0.071 | 0.013 | 1.51E-08 | 32.136 |
| order | Bifidobacteriales | rs60433564 | A | C | 0.123 | 0.022 | 1.05E-08 | 32.085 |
| order | Bifidobacteriales | rs6745996 | A | G | 0.099 | 0.017 | 8.06E-09 | 32.055 |
| order | Bifidobacteriales | rs7565053 | A | T | 0.128 | 0.023 | 1.04E-08 | 32.053 |
| order | Bifidobacteriales | rs11897217 | C | T | 0.094 | 0.017 | 3.48E-09 | 32.002 |
| order | Bifidobacteriales | rs9941547 | G | A | 0.071 | 0.013 | 1.72E-08 | 31.867 |
| order | Bifidobacteriales | rs10496736 | C | G | 0.122 | 0.022 | 1.09E-08 | 31.818 |
| order | Bifidobacteriales | rs2322445 | T | A | 0.093 | 0.017 | 4.22E-09 | 31.767 |
| order | Bifidobacteriales | rs7594509 | G | T | 0.093 | 0.017 | 4.01E-09 | 31.747 |
| order | Bifidobacteriales | rs6705610 | T | G | 0.093 | 0.017 | 4.25E-09 | 31.718 |
| order | Bifidobacteriales | rs998451 | A | G | 0.068 | 0.012 | 7.44E-09 | 31.361 |
| order | Bifidobacteriales | rs57543738 | T | C | 0.106 | 0.019 | 2.16E-08 | 31.257 |
| order | Bifidobacteriales | rs56211644 | T | C | 0.105 | 0.019 | 3.18E-08 | 30.950 |
| order | Bifidobacteriales | rs4954569 | C | T | 0.070 | 0.013 | 2.86E-08 | 30.854 |
| order | Bifidobacteriales | rs6715785 | G | A | 0.070 | 0.013 | 3.00E-08 | 30.788 |
| order | Bifidobacteriales | rs4954430 | C | T | 0.120 | 0.022 | 1.37E-08 | 30.596 |
| order | Bifidobacteriales | rs950176 | A | G | 0.070 | 0.013 | 3.52E-08 | 30.563 |
| order | Bifidobacteriales | rs4954392 | A | G | 0.070 | 0.013 | 3.21E-08 | 30.559 |
| order | Bifidobacteriales | rs4954570 | G | C | 0.074 | 0.013 | 2.61E-08 | 30.464 |
| order | Bifidobacteriales | rs12616955 | C | T | 0.069 | 0.013 | 3.45E-08 | 30.424 |
| order | Bifidobacteriales | rs60376570 | G | A | 0.104 | 0.019 | 3.46E-08 | 30.350 |
| order | Bifidobacteriales | rs7322849 | C | T | 0.111 | 0.020 | 1.74E-08 | 30.320 |
| order | Bifidobacteriales | rs6430580 | A | G | 0.096 | 0.017 | 1.96E-08 | 30.256 |
| order | Bifidobacteriales | rs4954633 | C | T | 0.118 | 0.022 | 1.67E-08 | 30.235 |
| order | Bifidobacteriales | rs62170081 | A | G | 0.119 | 0.022 | 2.78E-08 | 30.230 |
| order | Bifidobacteriales | rs10928554 | C | T | 0.100 | 0.018 | 1.56E-07 | 30.162 |
| order | Bifidobacteriales | rs4954566 | C | T | 0.069 | 0.013 | 4.15E-08 | 30.081 |
| order | Bifidobacteriales | rs35215526 | G | A | 0.107 | 0.020 | 7.28E-08 | 30.069 |
| order | Gastranaerophilales | rs9864379 | C | T | -0.161 | 0.029 | 4.66E-08 | 30.065 |
| order | Bifidobacteriales | rs4954564 | A | G | 0.069 | 0.013 | 4.58E-08 | 30.042 |
| order | Bifidobacteriales | rs10928513 | C | T | 0.066 | 0.012 | 2.74E-08 | 29.965 |
| order | Gastranaerophilales | rs9830438 | A | C | -0.160 | 0.029 | 5.27E-08 | 29.812 |
| order | Bifidobacteriales | rs6729961 | T | G | 0.118 | 0.022 | 4.11E-08 | 29.806 |
| order | Bifidobacteriales | rs7565871 | G | C | 0.091 | 0.017 | 1.13E-08 | 29.729 |
| order | Bifidobacteriales | rs56982689 | C | T | 0.118 | 0.022 | 1.78E-08 | 29.630 |
| order | Bifidobacteriales | rs2874874 | A | C | 0.103 | 0.019 | 5.28E-08 | 29.597 |
| order | Bifidobacteriales | rs10803550 | C | T | 0.068 | 0.013 | 5.22E-08 | 29.583 |
| order | Bifidobacteriales | rs6720497 | G | A | 0.118 | 0.022 | 4.10E-08 | 29.582 |
| order | Bifidobacteriales | rs6707289 | A | G | 0.118 | 0.022 | 4.10E-08 | 29.581 |
| order | Bifidobacteriales | rs4954396 | A | C | 0.069 | 0.013 | 5.27E-08 | 29.456 |
| order | Bifidobacteriales | rs4954572 | G | T | 0.069 | 0.013 | 5.44E-08 | 29.403 |
| order | Bifidobacteriales | rs953387 | A | C | 0.068 | 0.013 | 5.67E-08 | 29.387 |
| order | Bifidobacteriales | rs58080116 | A | G | 0.102 | 0.019 | 6.55E-08 | 29.305 |
| order | Bifidobacteriales | rs2102896 | T | C | 0.068 | 0.013 | 5.88E-08 | 29.285 |
| order | Bifidobacteriales | rs72972104 | G | A | 0.116 | 0.022 | 2.73E-08 | 29.249 |
| order | Bifidobacteriales | rs74266324 | G | A | 0.082 | 0.015 | 5.45E-08 | 29.201 |
| order | Gastranaerophilales | rs2005426 | C | A | -0.159 | 0.029 | 7.70E-08 | 29.151 |
| order | Bifidobacteriales | rs2322812 | A | G | 0.102 | 0.019 | 7.07E-08 | 29.075 |
| order | Bifidobacteriales | rs72970286 | T | G | 0.115 | 0.021 | 3.92E-08 | 29.057 |
| order | Gastranaerophilales | rs900188 | A | G | -0.158 | 0.029 | 8.47E-08 | 29.038 |
| order | Bifidobacteriales | rs2056297 | G | T | 0.068 | 0.013 | 7.24E-08 | 29.015 |
| order | Bifidobacteriales | rs1807356 | T | C | 0.092 | 0.017 | 4.88E-08 | 28.969 |
| order | Bifidobacteriales | rs62168847 | T | C | 0.105 | 0.019 | 1.08E-07 | 28.935 |
| order | Bifidobacteriales | rs62168846 | G | A | 0.105 | 0.020 | 1.38E-07 | 28.934 |
| order | Bifidobacteriales | rs13010498 | G | C | -0.069 | 0.013 | 6.09E-08 | 28.853 |
| order | Bacillales | rs74887130 | G | C | -0.192 | 0.036 | 1.16E-07 | 28.682 |
| order | Bifidobacteriales | rs74697610 | A | T | 0.112 | 0.021 | 7.76E-08 | 28.663 |
| order | Bifidobacteriales | rs62168843 | T | C | 0.104 | 0.019 | 1.20E-07 | 28.624 |
| order | Bifidobacteriales | rs3769011 | A | T | 0.101 | 0.019 | 9.05E-08 | 28.569 |
| order | Bifidobacteriales | rs4954577 | A | G | 0.068 | 0.013 | 8.89E-08 | 28.491 |
| order | Bifidobacteriales | rs4954445 | T | A | 0.101 | 0.019 | 9.27E-08 | 28.440 |
| order | Bifidobacteriales | rs1123848 | C | T | 0.068 | 0.013 | 8.89E-08 | 28.401 |
| order | Erysipelotrichales | rs62504403 | T | C | 0.068 | 0.013 | 1.12E-07 | 28.371 |
| order | Bifidobacteriales | rs4954397 | A | G | 0.068 | 0.013 | 9.39E-08 | 28.368 |
| order | Bifidobacteriales | rs12473760 | C | A | 0.067 | 0.013 | 9.33E-08 | 28.361 |
| order | Bifidobacteriales | rs16831994 | T | C | 0.114 | 0.022 | 6.36E-08 | 28.314 |
| order | Bifidobacteriales | rs1964776 | C | T | 0.067 | 0.013 | 9.83E-08 | 28.275 |
| order | Bifidobacteriales | rs61470941 | T | C | 0.100 | 0.019 | 8.01E-08 | 28.264 |
| order | Bifidobacteriales | rs16833335 | G | A | 0.067 | 0.013 | 1.13E-07 | 28.086 |
| order | Bifidobacteriales | rs4954388 | T | C | 0.068 | 0.013 | 1.43E-07 | 28.006 |
| order | Gastranaerophilales | rs12493406 | C | T | -0.155 | 0.029 | 2.20E-07 | 27.982 |
| order | Bifidobacteriales | rs7564577 | C | T | 0.089 | 0.017 | 5.26E-08 | 27.907 |
| order | Bifidobacteriales | rs1942055 | G | C | 0.064 | 0.012 | 8.32E-08 | 27.889 |
| order | Bifidobacteriales | rs13008147 | A | G | -0.071 | 0.013 | 2.50E-08 | 27.857 |
| order | Bifidobacteriales | rs4954578 | T | C | 0.067 | 0.013 | 1.37E-07 | 27.842 |
| order | Gastranaerophilales | rs12486770 | T | A | -0.153 | 0.029 | 2.64E-07 | 27.583 |
| order | Bifidobacteriales | rs7609188 | G | C | 0.113 | 0.022 | 8.16E-08 | 27.529 |
| order | Gastranaerophilales | rs62231951 | A | T | -0.153 | 0.029 | 2.82E-07 | 27.452 |
| order | Gastranaerophilales | rs9864035 | G | A | -0.153 | 0.029 | 1.82E-07 | 27.433 |
| order | Bifidobacteriales | rs13007153 | T | C | 0.068 | 0.013 | 2.09E-07 | 27.334 |
| order | Gastranaerophilales | rs6773199 | G | A | -0.150 | 0.029 | 1.37E-07 | 27.333 |
| order | Bifidobacteriales | rs1961273 | T | C | 0.069 | 0.013 | 1.70E-07 | 27.300 |
| order | Bifidobacteriales | rs1519525 | C | T | 0.066 | 0.013 | 1.72E-07 | 27.280 |
| order | Gastranaerophilales | rs9863760 | C | T | -0.148 | 0.028 | 2.28E-07 | 27.196 |
| order | Gastranaerophilales | rs6442436 | C | T | -0.152 | 0.029 | 2.03E-07 | 27.153 |
| order | Bifidobacteriales | rs80274970 | C | T | 0.108 | 0.021 | 2.18E-07 | 27.153 |
| order | Bifidobacteriales | rs62168844 | A | G | 0.101 | 0.019 | 2.74E-07 | 27.040 |
| order | Gastranaerophilales | rs9883109 | T | C | -0.152 | 0.029 | 2.19E-07 | 27.028 |
| order | Gastranaerophilales | rs10510427 | G | A | -0.150 | 0.029 | 3.51E-07 | 27.023 |
| order | Pasteurellales | rs9382510 | T | C | -0.088 | 0.017 | 2.48E-07 | 26.921 |
| order | Bifidobacteriales | rs3739022 | G | A | 0.098 | 0.019 | 1.66E-07 | 26.904 |
| order | Erysipelotrichales | rs11990327 | G | A | 0.067 | 0.013 | 2.45E-07 | 26.880 |
| order | Gastranaerophilales | rs13072729 | T | C | -0.151 | 0.029 | 2.61E-07 | 26.875 |
| order | Pasteurellales | rs12191680 | G | C | 0.102 | 0.020 | 3.05E-07 | 26.869 |
| order | Bifidobacteriales | rs60274701 | G | A | 0.097 | 0.019 | 2.25E-07 | 26.844 |
| order | Gastranaerophilales | rs6780959 | A | G | -0.151 | 0.029 | 2.43E-07 | 26.796 |
| order | Bifidobacteriales | rs6733547 | G | T | 0.087 | 0.017 | 1.45E-07 | 26.789 |
| order | Methanobacteriales | rs10202904 | G | T | -0.122 | 0.024 | 3.01E-07 | 26.761 |
| order | Bifidobacteriales | rs872151 | C | T | 0.096 | 0.019 | 1.81E-07 | 26.746 |
| order | Erysipelotrichales | rs11990200 | G | A | 0.067 | 0.013 | 2.37E-07 | 26.711 |
| order | Bifidobacteriales | rs10928558 | T | C | 0.066 | 0.013 | 2.34E-07 | 26.584 |
| order | Gastranaerophilales | rs13062781 | G | A | -0.146 | 0.028 | 1.44E-07 | 26.545 |
| order | Bifidobacteriales | rs6756200 | T | C | 0.062 | 0.012 | 2.33E-07 | 26.528 |
| order | Pasteurellales | rs7740611 | C | A | -0.088 | 0.017 | 3.08E-07 | 26.505 |
| order | NB1n | rs60583455 | C | T | 0.109 | 0.021 | 2.60E-07 | 26.460 |
| order | Burkholderiales | rs62191117 | G | A | 0.068 | 0.013 | 2.79E-07 | 26.349 |
| order | NB1n | rs2172426 | T | C | -0.102 | 0.020 | 3.17E-07 | 26.348 |
| order | Bacteroidales | rs55993203 | G | A | -0.122 | 0.024 | 3.90E-07 | 26.341 |
| order | Bacteroidales | rs55773148 | A | G | -0.122 | 0.024 | 3.90E-07 | 26.341 |
| order | Bifidobacteriales | rs10171677 | G | A | 0.062 | 0.012 | 1.40E-07 | 26.317 |
| order | Bacteroidales | rs56358950 | T | C | -0.121 | 0.024 | 4.48E-07 | 26.308 |
| order | Bifidobacteriales | rs10193587 | T | C | 0.066 | 0.013 | 2.40E-07 | 26.304 |
| order | Bacteroidales | rs73512608 | A | G | -0.121 | 0.024 | 4.08E-07 | 26.280 |
| order | Bifidobacteriales | rs3820790 | A | T | 0.098 | 0.019 | 3.11E-07 | 26.246 |
| order | Bacteroidales | rs58269684 | C | T | -0.121 | 0.024 | 4.49E-07 | 26.243 |
| order | Bifidobacteriales | rs12992815 | C | G | 0.096 | 0.019 | 2.38E-07 | 26.243 |
| order | Bifidobacteriales | rs2090661 | A | G | 0.066 | 0.013 | 3.34E-07 | 26.189 |
| order | Gastranaerophilales | rs1010755 | C | T | -0.149 | 0.029 | 3.55E-07 | 26.161 |
| order | Bifidobacteriales | rs4954573 | T | G | 0.066 | 0.013 | 2.68E-07 | 26.160 |
| order | Erysipelotrichales | rs62504402 | C | T | 0.066 | 0.013 | 2.88E-07 | 26.152 |
| order | NB1n | rs57230089 | C | T | 0.108 | 0.021 | 3.08E-07 | 26.129 |
| order | Burkholderiales | rs6087811 | G | T | -0.102 | 0.020 | 2.88E-07 | 26.108 |
| order | Bacillales | rs76860190 | A | G | -0.182 | 0.036 | 4.04E-07 | 26.103 |
| order | Rhodospirillales | rs9813022 | G | A | -0.083 | 0.016 | 3.07E-07 | 26.093 |
| order | Bifidobacteriales | rs4954272 | A | G | 0.089 | 0.018 | 3.17E-07 | 26.076 |
| order | Bacteroidales | rs60610351 | T | A | -0.121 | 0.024 | 4.85E-07 | 26.035 |
| order | Gastranaerophilales | rs17039205 | A | G | -0.149 | 0.029 | 3.64E-07 | 25.970 |
| order | Bacteroidales | rs74090639 | G | A | -0.121 | 0.024 | 5.59E-07 | 25.928 |
| order | Bifidobacteriales | rs6716987 | C | A | 0.064 | 0.013 | 3.84E-07 | 25.792 |
| order | Gastranaerophilales | rs17039208 | T | C | -0.148 | 0.029 | 4.15E-07 | 25.725 |
| order | Bacteroidales | rs17085771 | A | T | -0.120 | 0.024 | 6.24E-07 | 25.715 |
| order | Lactobacillales | rs2952251 | G | A | -0.063 | 0.012 | 3.36E-07 | 25.683 |
| order | Bacteroidales | rs58298316 | T | G | -0.120 | 0.024 | 6.30E-07 | 25.596 |
| order | Bacteroidales | rs60614038 | T | A | -0.120 | 0.024 | 6.30E-07 | 25.596 |
| order | Pasteurellales | rs7771624 | C | A | -0.086 | 0.017 | 4.94E-07 | 25.576 |
| order | Bifidobacteriales | rs56036623 | T | C | 0.070 | 0.014 | 3.06E-07 | 25.512 |
| order | Gastranaerophilales | rs13098772 | C | A | -0.147 | 0.029 | 6.08E-07 | 25.497 |
| order | Lactobacillales | rs35344081 | A | G | 0.064 | 0.013 | 4.16E-07 | 25.446 |
| order | Bacteroidales | rs9788409 | T | C | -0.119 | 0.024 | 7.07E-07 | 25.430 |
| order | Methanobacteriales | rs7601415 | T | A | -0.119 | 0.024 | 6.11E-07 | 25.420 |
| order | Bifidobacteriales | rs6708336 | T | A | 0.060 | 0.012 | 3.72E-07 | 25.411 |
| order | NB1n | rs11251024 | A | G | 0.104 | 0.021 | 6.63E-07 | 25.404 |
| order | Desulfovibrionales | rs6058181 | T | C | 0.084 | 0.017 | 2.53E-07 | 25.379 |
| order | NB1n | rs62436708 | C | T | 0.107 | 0.021 | 5.02E-07 | 25.362 |
| order | Bifidobacteriales | rs10496737 | A | G | 0.073 | 0.015 | 1.04E-06 | 25.356 |
| order | Selenomonadales | rs1643968 | C | T | -0.057 | 0.011 | 4.15E-07 | 25.339 |
| order | Bifidobacteriales | rs3814354 | G | A | 0.060 | 0.012 | 3.95E-07 | 25.328 |
| order | NB1n | rs11251023 | T | C | 0.104 | 0.021 | 6.85E-07 | 25.325 |
| order | Gastranaerophilales | rs13088024 | T | A | -0.147 | 0.029 | 5.61E-07 | 25.293 |
| order | NB1n | rs11251025 | G | C | 0.104 | 0.021 | 7.03E-07 | 25.286 |
| order | Bifidobacteriales | rs60963894 | T | G | 0.073 | 0.015 | 1.08E-06 | 25.276 |
| order | Bacteroidales | rs17085818 | A | C | -0.119 | 0.024 | 7.46E-07 | 25.273 |
| order | Bifidobacteriales | rs4988243 | T | C | 0.119 | 0.024 | 3.85E-07 | 25.271 |
| order | NB1n | rs7902814 | G | T | 0.104 | 0.021 | 6.98E-07 | 25.259 |
| order | Methanobacteriales | rs1869500 | A | C | -0.118 | 0.024 | 6.57E-07 | 25.249 |
| order | Lactobacillales | rs17708276 | G | A | -0.084 | 0.017 | 4.38E-07 | 25.207 |
| order | Clostridiales | NA | T | C | 0.255 | 0.051 | 8.95E-07 | 25.206 |
| order | Bifidobacteriales | rs1865452 | C | T | 0.073 | 0.015 | 1.12E-06 | 25.205 |
| order | Bifidobacteriales | rs62168897 | C | T | 0.060 | 0.012 | 4.15E-07 | 25.199 |
| order | Clostridiales | rs112334273 | A | G | 0.064 | 0.013 | 4.07E-07 | 25.186 |
| order | Erysipelotrichales | rs62504370 | C | A | 0.063 | 0.013 | 4.80E-07 | 25.185 |
| order | Bacteroidales | rs2210451 | C | G | -0.119 | 0.024 | 7.02E-07 | 25.164 |
| order | Gastranaerophilales | rs13060131 | C | T | -0.146 | 0.029 | 5.92E-07 | 25.159 |
| order | Bifidobacteriales | rs9677760 | A | T | 0.073 | 0.014 | 1.21E-06 | 25.154 |
| order | Gastranaerophilales | rs13072505 | A | G | -0.146 | 0.029 | 5.82E-07 | 25.147 |
| order | Bifidobacteriales | rs7560535 | G | A | 0.093 | 0.018 | 9.58E-07 | 25.130 |
| order | Bifidobacteriales | rs4954557 | C | T | 0.065 | 0.013 | 5.19E-07 | 25.121 |
| order | NB1n | rs56139471 | T | A | 0.104 | 0.021 | 7.61E-07 | 25.120 |
| order | Bifidobacteriales | rs2034261 | G | T | 0.060 | 0.012 | 4.26E-07 | 25.098 |
| order | Bacteroidales | rs2150960 | G | C | -0.119 | 0.024 | 7.16E-07 | 25.097 |
| order | Burkholderiales | rs78428620 | C | T | -0.102 | 0.020 | 5.28E-07 | 25.088 |
| order | Lactobacillales | rs2009619 | G | A | -0.062 | 0.012 | 4.68E-07 | 25.079 |
| order | Bacteroidales | rs2210454 | C | T | -0.119 | 0.024 | 7.69E-07 | 25.077 |
| order | Gastranaerophilales | rs12714900 | G | T | -0.146 | 0.029 | 6.31E-07 | 24.999 |
| order | Bacteroidales | rs17085798 | C | A | -0.118 | 0.024 | 8.02E-07 | 24.996 |
| order | Bifidobacteriales | rs78793148 | C | A | 0.099 | 0.020 | 1.03E-06 | 24.978 |
| order | Bifidobacteriales | rs625118 | C | A | 0.087 | 0.017 | 6.58E-07 | 24.964 |
| order | Burkholderiales | rs12467854 | C | G | 0.066 | 0.013 | 5.91E-07 | 24.962 |
| order | Gastranaerophilales | rs12491989 | C | G | -0.146 | 0.029 | 6.52E-07 | 24.957 |
| order | Coriobacteriales | rs719099 | G | A | 0.078 | 0.016 | 5.43E-07 | 24.957 |
| order | Bacteroidales | rs74090627 | C | T | -0.118 | 0.024 | 7.95E-07 | 24.946 |
| order | Bifidobacteriales | rs10166142 | A | G | 0.059 | 0.012 | 4.86E-07 | 24.932 |
| order | Verrucomicrobiales | rs11729256 | C | T | 0.075 | 0.015 | 6.73E-07 | 24.928 |
| order | NB1n | rs62436710 | C | T | 0.106 | 0.021 | 5.67E-07 | 24.921 |
| order | Bacteroidales | rs56949730 | A | G | -0.118 | 0.024 | 7.98E-07 | 24.911 |
| order | Gastranaerophilales | rs12714903 | C | T | -0.146 | 0.029 | 6.64E-07 | 24.902 |
| order | Gastranaerophilales | rs13095126 | T | C | -0.145 | 0.029 | 6.82E-07 | 24.884 |
| order | Bifidobacteriales | rs6430601 | G | T | 0.085 | 0.017 | 2.46E-07 | 24.873 |
| order | Bacteroidales | rs112343530 | T | C | -0.118 | 0.024 | 7.32E-07 | 24.870 |
| order | Desulfovibrionales | rs6060239 | T | C | 0.083 | 0.017 | 3.67E-07 | 24.867 |
| order | Bacteroidales | rs80015632 | C | G | -0.118 | 0.024 | 8.23E-07 | 24.865 |
| order | Bifidobacteriales | rs1469996 | A | G | 0.073 | 0.015 | 1.53E-06 | 24.863 |
| order | Gastranaerophilales | rs13072444 | G | A | -0.145 | 0.029 | 6.94E-07 | 24.847 |
| order | Bifidobacteriales | rs35564151 | A | G | 0.060 | 0.012 | 4.51E-07 | 24.846 |
| order | Gastranaerophilales | rs11150282 | C | T | 0.098 | 0.020 | 7.36E-07 | 24.834 |
| order | Enterobacteriales | rs78143293 | G | A | -0.085 | 0.017 | 1.20E-06 | 24.792 |
| order | Gastranaerophilales | rs12714902 | G | A | -0.145 | 0.029 | 7.05E-07 | 24.791 |
| order | Desulfovibrionales | rs6060238 | G | A | 0.083 | 0.017 | 3.70E-07 | 24.786 |
| order | Burkholderiales | rs4033856 | T | C | 0.083 | 0.017 | 5.67E-07 | 24.762 |
| order | Bifidobacteriales | rs3814355 | C | T | 0.059 | 0.012 | 4.83E-07 | 24.742 |
| order | Desulfovibrionales | rs6060237 | A | G | 0.082 | 0.017 | 3.97E-07 | 24.669 |
| order | Bifidobacteriales | rs7595712 | C | T | 0.072 | 0.015 | 1.70E-06 | 24.663 |
| order | Bifidobacteriales | rs7592990 | A | G | 0.087 | 0.017 | 7.55E-07 | 24.662 |
| order | Bifidobacteriales | rs2117511 | T | C | 0.086 | 0.017 | 7.45E-07 | 24.661 |
| order | Bifidobacteriales | rs56271357 | C | T | 0.059 | 0.012 | 5.47E-07 | 24.643 |
| order | Bacteroidales | rs2210452 | G | A | -0.117 | 0.024 | 9.11E-07 | 24.612 |
| order | NB1n | rs17235747 | A | C | 0.103 | 0.021 | 9.92E-07 | 24.609 |
| order | Clostridiales | NA | C | T | 0.251 | 0.051 | 1.28E-06 | 24.575 |
| order | Bifidobacteriales | rs1469995 | G | A | 0.072 | 0.015 | 1.55E-06 | 24.574 |
| order | Bifidobacteriales | rs60453613 | G | C | 0.072 | 0.015 | 1.55E-06 | 24.573 |
| order | Bifidobacteriales | rs309124 | A | G | 0.076 | 0.015 | 9.62E-07 | 24.560 |
| order | Bacteroidales | rs112675712 | T | C | -0.117 | 0.024 | 8.12E-07 | 24.553 |
| order | Bifidobacteriales | rs4954394 | A | G | 0.067 | 0.014 | 4.30E-07 | 24.546 |
| order | Bifidobacteriales | rs12469551 | G | T | 0.072 | 0.014 | 1.80E-06 | 24.520 |
| order | Bifidobacteriales | rs11895609 | A | T | 0.083 | 0.017 | 2.89E-07 | 24.515 |
| order | Coriobacteriales | rs2458655 | C | A | 0.078 | 0.016 | 7.77E-07 | 24.461 |
| order | Bifidobacteriales | rs3769023 | G | A | 0.059 | 0.012 | 6.29E-07 | 24.435 |
| order | Bifidobacteriales | rs1964459 | C | G | 0.080 | 0.016 | 1.17E-06 | 24.419 |
| order | Gastranaerophilales | rs7644367 | G | A | -0.144 | 0.029 | 8.09E-07 | 24.402 |
| order | Bifidobacteriales | rs10187990 | A | G | 0.059 | 0.012 | 5.95E-07 | 24.385 |
| order | Clostridiales | rs6815608 | C | T | 0.104 | 0.021 | 3.72E-07 | 24.363 |
| order | Gastranaerophilales | rs9832125 | C | T | -0.139 | 0.028 | 3.26E-07 | 24.339 |
| order | Methanobacteriales | rs73457410 | G | A | 0.215 | 0.044 | 1.41E-06 | 24.316 |
| order | Gastranaerophilales | rs7206242 | C | T | 0.099 | 0.020 | 1.05E-06 | 24.306 |
| order | Gastranaerophilales | rs13072044 | G | T | -0.148 | 0.030 | 8.25E-07 | 24.300 |
| order | NB1n | rs62436709 | A | G | 0.104 | 0.021 | 7.82E-07 | 24.295 |
| order | Bifidobacteriales | rs6708957 | C | T | 0.072 | 0.015 | 1.76E-06 | 24.294 |
| order | Gastranaerophilales | rs13072359 | A | C | -0.144 | 0.029 | 9.37E-07 | 24.272 |
| order | Bifidobacteriales | rs1947112 | A | G | 0.059 | 0.012 | 6.85E-07 | 24.246 |
| order | Bifidobacteriales | rs6732236 | T | G | 0.072 | 0.015 | 1.82E-06 | 24.238 |
| order | Bifidobacteriales | rs638382 | C | T | -0.060 | 0.012 | 5.56E-07 | 24.230 |
| order | Bifidobacteriales | rs4567981 | A | T | 0.058 | 0.012 | 8.88E-07 | 24.224 |
| order | Lactobacillales | rs76717940 | A | T | 0.161 | 0.033 | 6.55E-07 | 24.224 |
| order | Clostridiales | rs6814436 | C | T | 0.074 | 0.015 | 9.06E-07 | 24.197 |
| order | Bifidobacteriales | rs72847650 | C | A | -0.061 | 0.012 | 5.13E-07 | 24.193 |
| order | Victivallales | rs62570196 | T | C | -0.216 | 0.044 | 1.08E-06 | 24.192 |
| order | Selenomonadales | rs60274479 | C | T | -0.066 | 0.013 | 1.16E-06 | 24.182 |
| order | Gastranaerophilales | rs13084677 | A | T | -0.139 | 0.028 | 3.45E-07 | 24.181 |
| order | Bacteroidales | rs7337863 | C | A | -0.117 | 0.024 | 8.25E-07 | 24.167 |
| order | Lactobacillales | rs2975635 | C | T | -0.060 | 0.012 | 8.37E-07 | 24.155 |
| order | Erysipelotrichales | rs2300774 | A | G | 0.052 | 0.011 | 8.95E-07 | 24.094 |
| order | Verrucomicrobiales | rs113634144 | G | T | 0.074 | 0.015 | 1.12E-06 | 24.092 |
| order | Bifidobacteriales | rs147616090 | C | T | 0.071 | 0.015 | 2.04E-06 | 24.068 |
| order | Bifidobacteriales | rs2289963 | G | A | 0.099 | 0.020 | 1.70E-06 | 24.061 |
| order | Pasteurellales | rs4132989 | T | C | -0.084 | 0.017 | 1.18E-06 | 24.055 |
| order | Bifidobacteriales | rs4953938 | G | A | 0.058 | 0.012 | 6.84E-07 | 24.054 |
| order | Bifidobacteriales | rs309122 | A | G | 0.075 | 0.015 | 1.25E-06 | 24.038 |
| order | Burkholderiales | rs11679161 | C | T | 0.065 | 0.013 | 9.07E-07 | 23.978 |
| order | Burkholderiales | rs4511740 | A | G | 0.063 | 0.013 | 1.01E-06 | 23.976 |
| order | Erysipelotrichales | rs2300775 | C | T | 0.052 | 0.011 | 9.78E-07 | 23.919 |
| order | Desulfovibrionales | rs6060240 | C | A | 0.082 | 0.017 | 7.09E-07 | 23.841 |
| order | Gastranaerophilales | rs4129395 | A | G | 0.090 | 0.019 | 1.22E-06 | 23.826 |
| order | Coriobacteriales | rs7078773 | C | T | 0.076 | 0.016 | 1.12E-06 | 23.780 |
| order | Bifidobacteriales | rs76671854 | G | C | -0.089 | 0.018 | 1.04E-06 | 23.765 |
| order | Bifidobacteriales | rs4957061 | C | T | 0.057 | 0.012 | 1.15E-06 | 23.761 |
| order | Burkholderiales | rs11683769 | A | T | 0.064 | 0.013 | 9.92E-07 | 23.760 |
| order | Lactobacillales | rs2952228 | C | T | -0.060 | 0.012 | 9.17E-07 | 23.760 |
| order | Bifidobacteriales | rs77985886 | C | T | 0.098 | 0.020 | 2.02E-06 | 23.758 |
| order | Gastranaerophilales | rs4129397 | A | G | 0.090 | 0.019 | 1.27E-06 | 23.746 |
| order | Erysipelotrichales | rs7234058 | C | T | -0.095 | 0.019 | 9.12E-07 | 23.744 |
| order | Bifidobacteriales | rs60902646 | T | C | 0.095 | 0.020 | 2.47E-06 | 23.740 |
| order | Erysipelotrichales | rs9858727 | T | A | 0.052 | 0.011 | 1.08E-06 | 23.728 |
| order | Erysipelotrichales | rs2300780 | C | T | 0.052 | 0.011 | 1.10E-06 | 23.722 |
| order | NB1n | rs13219468 | C | G | 0.115 | 0.024 | 1.41E-06 | 23.694 |
| order | Methanobacteriales | rs62241835 | T | G | -0.203 | 0.042 | 1.63E-06 | 23.683 |
| order | Selenomonadales | rs2921533 | A | G | -0.054 | 0.011 | 1.02E-06 | 23.631 |
| order | Bacteroidales | rs73510191 | A | C | -0.115 | 0.024 | 1.65E-06 | 23.628 |
| order | Bacteroidales | rs73846128 | G | A | -0.065 | 0.013 | 8.76E-07 | 23.602 |
| order | NB1n | rs7920017 | A | G | 0.100 | 0.021 | 1.63E-06 | 23.599 |
| order | Erysipelotrichales | rs79996147 | A | T | -0.094 | 0.019 | 9.72E-07 | 23.597 |
| order | Erysipelotrichales | rs2300777 | C | A | 0.052 | 0.011 | 1.18E-06 | 23.592 |
| order | Bacteroidales | rs7631304 | A | G | -0.065 | 0.013 | 8.37E-07 | 23.590 |
| order | Bifidobacteriales | rs10174462 | C | G | 0.071 | 0.015 | 3.08E-06 | 23.565 |
| order | Pasteurellales | rs76022354 | T | C | 0.243 | 0.050 | 1.83E-06 | 23.560 |
| order | Bifidobacteriales | rs578384 | C | T | -0.059 | 0.012 | 7.43E-07 | 23.541 |
| order | Coriobacteriales | rs56374305 | C | G | 0.100 | 0.021 | 1.17E-06 | 23.530 |
| order | Burkholderiales | rs2367850 | C | G | 0.063 | 0.013 | 1.24E-06 | 23.516 |
| order | NB1n | rs7923330 | T | C | 0.100 | 0.021 | 1.75E-06 | 23.490 |
| order | Bifidobacteriales | rs4954575 | G | A | 0.062 | 0.013 | 9.86E-07 | 23.488 |
| order | Bifidobacteriales | rs1374289 | C | T | 0.058 | 0.012 | 9.62E-07 | 23.485 |
| order | Methanobacteriales | rs6776814 | C | T | -0.200 | 0.041 | 1.63E-06 | 23.483 |
| order | Bacteroidales | rs4474577 | G | A | -0.118 | 0.024 | 1.47E-06 | 23.471 |
| order | Bifidobacteriales | rs16832440 | G | A | 0.079 | 0.016 | 1.90E-06 | 23.467 |
| order | Lactobacillales | rs4028634 | C | T | 0.053 | 0.011 | 1.35E-06 | 23.428 |
| order | Pasteurellales | rs78909003 | C | T | -0.241 | 0.050 | 2.05E-06 | 23.415 |
| order | Gastranaerophilales | rs7204337 | G | A | 0.098 | 0.020 | 1.79E-06 | 23.409 |
| order | Bacteroidales | rs56335733 | C | T | -0.115 | 0.024 | 1.66E-06 | 23.389 |
| order | Bacteroidales | rs2225529 | C | T | -0.115 | 0.024 | 1.44E-06 | 23.364 |
| order | Bacteroidales | rs60660711 | G | A | -0.115 | 0.024 | 1.52E-06 | 23.350 |
| order | Gastranaerophilales | rs4129396 | A | G | 0.090 | 0.019 | 1.56E-06 | 23.350 |
| order | Methanobacteriales | rs894996 | A | C | 0.217 | 0.045 | 1.88E-06 | 23.349 |
| order | Bifidobacteriales | rs6430585 | C | A | 0.070 | 0.014 | 3.17E-06 | 23.337 |
| order | Desulfovibrionales | rs16851319 | C | G | -0.073 | 0.015 | 2.70E-06 | 23.331 |
| order | Erysipelotrichales | rs2300778 | G | A | 0.052 | 0.011 | 1.37E-06 | 23.298 |
| order | Bifidobacteriales | rs309123 | C | G | 0.075 | 0.016 | 1.64E-06 | 23.297 |
| order | Bifidobacteriales | rs7604460 | T | C | 0.079 | 0.016 | 2.38E-06 | 23.274 |
| order | Bifidobacteriales | rs6733130 | A | C | 0.058 | 0.012 | 9.71E-07 | 23.265 |
| order | Selenomonadales | rs73232831 | A | G | -0.152 | 0.031 | 1.87E-06 | 23.242 |
| order | Methanobacteriales | rs9942108 | G | A | -0.203 | 0.042 | 1.85E-06 | 23.240 |
| order | Bacteroidales | rs1888900 | T | C | -0.114 | 0.024 | 2.03E-06 | 23.239 |
| order | Bifidobacteriales | rs12471781 | G | A | 0.079 | 0.016 | 2.00E-06 | 23.237 |
| order | Methanobacteriales | rs73031978 | G | C | -0.203 | 0.042 | 1.90E-06 | 23.225 |
| order | Bacteroidales | rs7338117 | A | G | -0.115 | 0.024 | 1.58E-06 | 23.218 |
| order | Bacteroidales | rs17085746 | A | G | -0.115 | 0.024 | 1.63E-06 | 23.214 |
| order | Bacteroidales | rs17085738 | T | G | -0.115 | 0.024 | 1.63E-06 | 23.214 |
| order | Bifidobacteriales | rs309152 | T | C | 0.076 | 0.016 | 1.48E-06 | 23.199 |
| order | Lactobacillales | rs2975647 | G | A | -0.059 | 0.012 | 1.28E-06 | 23.184 |
| order | Lactobacillales | rs2952176 | A | G | -0.059 | 0.012 | 1.44E-06 | 23.178 |
| order | Bifidobacteriales | rs309133 | A | C | 0.075 | 0.016 | 1.65E-06 | 23.174 |
| order | Clostridiales | rs72915163 | C | T | -0.058 | 0.012 | 1.39E-06 | 23.165 |
| order | Lactobacillales | rs9581006 | T | C | 0.226 | 0.047 | 1.77E-06 | 23.163 |
| order | Bacteroidales | rs11929346 | T | C | -0.064 | 0.013 | 1.10E-06 | 23.143 |
| order | Bifidobacteriales | rs4954571 | G | A | 0.065 | 0.014 | 9.01E-07 | 23.140 |
| order | Methanobacteriales | rs76029318 | C | T | 0.215 | 0.045 | 1.58E-06 | 23.140 |
| order | Methanobacteriales | rs62240385 | T | G | -0.201 | 0.042 | 1.77E-06 | 23.135 |
| order | Pasteurellales | rs10840326 | G | C | -0.072 | 0.015 | 1.44E-06 | 23.126 |
| order | Erysipelotrichales | rs35161940 | C | T | -0.081 | 0.017 | 1.85E-06 | 23.118 |
| order | Bifidobacteriales | rs1374287 | C | T | 0.057 | 0.012 | 1.06E-06 | 23.114 |
| order | Bacteroidales | rs73510178 | G | A | -0.115 | 0.024 | 1.67E-06 | 23.113 |
| order | Bacteroidales | rs12938514 | G | A | -0.200 | 0.042 | 1.42E-06 | 23.106 |
| order | Methanobacteriales | rs12711690 | G | A | -0.113 | 0.024 | 1.97E-06 | 23.103 |
| order | Bacteroidales | rs61070599 | C | T | -0.115 | 0.024 | 1.82E-06 | 23.094 |
| order | Lactobacillales | rs11249980 | C | T | -0.059 | 0.012 | 1.45E-06 | 23.085 |
| order | Erysipelotrichales | rs2284890 | C | T | 0.051 | 0.011 | 1.54E-06 | 23.077 |
| order | Bifidobacteriales | rs7575241 | C | A | 0.071 | 0.015 | 3.17E-06 | 23.075 |
| order | Bifidobacteriales | rs112407062 | C | T | 0.078 | 0.016 | 2.02E-06 | 23.056 |
| order | Bifidobacteriales | rs2322251 | C | T | 0.057 | 0.012 | 1.24E-06 | 23.047 |
| order | Bacteroidales | rs111845179 | C | T | 0.103 | 0.021 | 9.24E-07 | 23.041 |
| order | Bacteroidales | rs74937713 | A | T | -0.063 | 0.013 | 1.25E-06 | 23.034 |
| order | Bacteroidales | rs35800684 | G | A | -0.064 | 0.013 | 1.15E-06 | 23.032 |
| order | Gastranaerophilales | rs16851659 | C | G | -0.090 | 0.019 | 1.27E-06 | 23.025 |
| order | Bifidobacteriales | rs75667274 | C | T | 0.078 | 0.016 | 2.13E-06 | 23.019 |
| order | Erysipelotrichales | rs2284889 | A | G | 0.051 | 0.011 | 1.60E-06 | 23.005 |
| order | Victivallales | rs77599476 | G | A | 0.230 | 0.048 | 1.86E-06 | 23.002 |
| order | Bifidobacteriales | rs1374288 | C | T | 0.057 | 0.012 | 1.14E-06 | 22.988 |
| order | Actinomycetales | rs35011108 | G | A | 0.242 | 0.050 | 1.88E-06 | 22.987 |
| order | Bifidobacteriales | rs7568884 | C | T | 0.061 | 0.013 | 1.26E-06 | 22.985 |
| order | Gastranaerophilales | rs7859354 | C | T | 0.089 | 0.019 | 1.89E-06 | 22.984 |
| order | Erysipelotrichales | rs2239641 | C | T | 0.051 | 0.011 | 1.62E-06 | 22.979 |
| order | Methanobacteriales | rs62241834 | G | T | -0.201 | 0.042 | 2.44E-06 | 22.975 |
| order | Erysipelotrichales | rs79329601 | C | T | -0.093 | 0.019 | 1.40E-06 | 22.960 |
| order | Bifidobacteriales | rs115553816 | G | A | 0.078 | 0.016 | 2.18E-06 | 22.959 |
| order | Bifidobacteriales | rs11745923 | T | G | 0.058 | 0.012 | 2.49E-06 | 22.949 |
| order | Bacteroidales | rs7546249 | A | T | -0.057 | 0.012 | 1.55E-06 | 22.948 |
| order | Erysipelotrichales | rs76420385 | G | A | -0.093 | 0.019 | 1.47E-06 | 22.941 |
| order | Gastranaerophilales | rs72765665 | C | T | -0.089 | 0.019 | 1.35E-06 | 22.941 |
| order | Bifidobacteriales | rs10928523 | C | T | 0.057 | 0.012 | 1.16E-06 | 22.940 |
| order | Bacteroidales | rs60869989 | G | A | -0.114 | 0.024 | 1.94E-06 | 22.938 |
| order | Bifidobacteriales | rs12476127 | A | G | 0.076 | 0.016 | 2.51E-06 | 22.921 |
| order | Methanobacteriales | rs6784384 | G | A | -0.198 | 0.041 | 1.90E-06 | 22.909 |
| order | Gastranaerophilales | rs10739388 | T | C | 0.089 | 0.019 | 1.97E-06 | 22.899 |
| order | Verrucomicrobiales | rs9349825 | G | A | -0.070 | 0.015 | 2.54E-06 | 22.898 |
| order | Bacteroidales | rs73512626 | C | A | -0.114 | 0.024 | 2.02E-06 | 22.891 |
| order | Bifidobacteriales | rs7589832 | A | C | 0.070 | 0.015 | 3.88E-06 | 22.890 |
| order | Bifidobacteriales | rs13020688 | A | G | 0.058 | 0.012 | 1.57E-06 | 22.887 |
| order | Rhodospirillales | rs76784716 | G | A | 0.136 | 0.028 | 1.31E-06 | 22.881 |
| order | Verrucomicrobiales | rs2602429 | T | C | 0.075 | 0.016 | 2.58E-06 | 22.863 |
| order | Lactobacillales | rs11248956 | T | C | 0.061 | 0.013 | 1.19E-06 | 22.856 |
| order | Bacteroidales | rs60722059 | A | G | -0.114 | 0.024 | 1.88E-06 | 22.839 |
| order | Methanobacteriales | rs62240384 | C | T | -0.198 | 0.041 | 1.99E-06 | 22.817 |
| order | MollicutesRF9 | rs17235252 | C | T | -0.122 | 0.026 | 2.16E-06 | 22.808 |
| order | Bacteroidales | rs61289215 | C | T | -0.114 | 0.024 | 2.19E-06 | 22.794 |
| order | Methanobacteriales | rs56209241 | G | A | -0.202 | 0.042 | 2.53E-06 | 22.793 |
| order | Methanobacteriales | rs73457427 | G | A | 0.213 | 0.045 | 1.86E-06 | 22.790 |
| order | Bifidobacteriales | rs13415439 | C | A | 0.061 | 0.013 | 1.40E-06 | 22.788 |
| order | Verrucomicrobiales | rs4936098 | G | A | 0.065 | 0.014 | 1.12E-06 | 22.786 |
| order | Erysipelotrichales | rs2239640 | T | G | 0.051 | 0.011 | 1.79E-06 | 22.783 |
| order | Bifidobacteriales | rs72976292 | T | A | 0.061 | 0.013 | 1.41E-06 | 22.783 |
| order | Bifidobacteriales | rs1348790 | C | G | 0.057 | 0.012 | 1.26E-06 | 22.770 |
| order | Lactobacillales | rs2975649 | C | T | -0.059 | 0.012 | 1.61E-06 | 22.761 |
| order | Bifidobacteriales | rs16833170 | A | G | 0.061 | 0.013 | 1.44E-06 | 22.754 |
| order | Erysipelotrichales | rs9852239 | C | T | 0.051 | 0.011 | 1.81E-06 | 22.752 |
| order | Bifidobacteriales | rs150183636 | C | T | 0.096 | 0.020 | 4.08E-06 | 22.750 |
| order | Burkholderiales | rs8119189 | C | A | -0.097 | 0.020 | 1.78E-06 | 22.745 |
| order | Bacteroidales | rs7629132 | C | T | -0.063 | 0.013 | 1.48E-06 | 22.734 |
| order | Erysipelotrichales | rs78782849 | C | T | -0.092 | 0.019 | 1.61E-06 | 22.731 |
| order | Clostridiales | rs4690986 | C | G | 0.072 | 0.015 | 2.01E-06 | 22.711 |
| order | Erysipelotrichales | rs9863872 | G | T | 0.051 | 0.011 | 1.87E-06 | 22.709 |
| order | Erysipelotrichales | rs9846149 | G | C | 0.051 | 0.011 | 1.87E-06 | 22.696 |
| order | Lactobacillales | rs77558518 | G | A | -0.106 | 0.022 | 1.67E-06 | 22.695 |
| order | Bifidobacteriales | rs10496738 | C | T | 0.077 | 0.016 | 2.89E-06 | 22.685 |
| order | Lactobacillales | rs2975655 | G | A | -0.059 | 0.012 | 1.75E-06 | 22.678 |
| order | Bacteroidales | rs74090631 | A | G | -0.114 | 0.024 | 2.10E-06 | 22.673 |
| order | Bacteroidales | rs73510184 | C | T | -0.114 | 0.024 | 2.10E-06 | 22.673 |
| order | Bifidobacteriales | rs59869505 | A | C | 0.061 | 0.013 | 1.46E-06 | 22.671 |
| order | Bacteroidales | rs2032750 | C | T | -0.051 | 0.011 | 1.92E-06 | 22.657 |
| order | Methanobacteriales | rs4848258 | A | T | -0.112 | 0.024 | 2.44E-06 | 22.649 |
| order | Bifidobacteriales | rs10841473 | C | G | -0.061 | 0.013 | 2.28E-06 | 22.638 |
| order | Bifidobacteriales | rs112396243 | C | T | 0.077 | 0.016 | 2.60E-06 | 22.636 |
| order | Bacteroidales | rs60954548 | A | G | -0.113 | 0.024 | 2.38E-06 | 22.634 |
| order | Erysipelotrichales | rs10781552 | T | C | -0.055 | 0.012 | 2.33E-06 | 22.633 |
| order | Coriobacteriales | rs240104 | C | T | -0.060 | 0.013 | 1.52E-06 | 22.630 |
| order | Methanobacteriales | rs4848970 | G | T | 0.116 | 0.024 | 1.89E-06 | 22.619 |
| order | Bifidobacteriales | rs721765 | C | A | 0.077 | 0.016 | 2.94E-06 | 22.598 |
| order | Methanobacteriales | rs62240373 | G | A | -0.199 | 0.042 | 2.75E-06 | 22.598 |
| order | Lactobacillales | rs1962325 | G | C | 0.055 | 0.012 | 1.96E-06 | 22.587 |
| order | Lactobacillales | rs74352383 | A | T | -0.103 | 0.022 | 1.88E-06 | 22.583 |
| order | Rhodospirillales | rs75071199 | C | A | 0.135 | 0.028 | 1.46E-06 | 22.582 |
| order | Methanobacteriales | rs11147839 | G | A | 0.211 | 0.044 | 2.28E-06 | 22.574 |
| order | Methanobacteriales | rs62241836 | C | T | -0.199 | 0.042 | 2.84E-06 | 22.571 |
| order | Victivallales | rs2825714 | G | A | -0.137 | 0.029 | 1.72E-06 | 22.569 |
| order | Gastranaerophilales | rs10817461 | A | T | 0.088 | 0.019 | 2.32E-06 | 22.566 |
| order | Bifidobacteriales | rs73958618 | A | G | 0.069 | 0.015 | 4.27E-06 | 22.566 |
| order | Lactobacillales | rs11967379 | C | T | -0.103 | 0.022 | 2.40E-06 | 22.550 |
| order | Bifidobacteriales | rs12474975 | A | T | 0.077 | 0.016 | 2.48E-06 | 22.549 |
| order | Bifidobacteriales | rs7570283 | T | C | 0.092 | 0.019 | 4.26E-06 | 22.547 |
| order | Selenomonadales | rs71405394 | A | G | -0.114 | 0.024 | 2.17E-06 | 22.539 |
| order | Selenomonadales | rs13086907 | A | G | 0.063 | 0.013 | 1.95E-06 | 22.532 |
| order | Bacteroidales | rs7611599 | C | T | -0.063 | 0.013 | 1.49E-06 | 22.517 |
| order | Rhodospirillales | rs3754624 | T | C | 0.094 | 0.020 | 2.68E-06 | 22.511 |
| order | Bifidobacteriales | rs6746633 | G | A | 0.096 | 0.020 | 4.44E-06 | 22.508 |
| order | Bacteroidales | rs74090626 | A | G | -0.113 | 0.024 | 2.30E-06 | 22.508 |
| order | Verrucomicrobiales | rs74542928 | C | T | 0.112 | 0.024 | 1.63E-06 | 22.508 |
| order | Bacteroidales | rs11920519 | A | G | -0.063 | 0.013 | 1.70E-06 | 22.504 |
| order | NB1n | rs7911787 | T | G | -0.223 | 0.047 | 3.39E-06 | 22.504 |
| order | Bifidobacteriales | rs7574366 | G | A | 0.096 | 0.020 | 4.08E-06 | 22.499 |
| order | Bifidobacteriales | rs56108664 | C | T | 0.075 | 0.016 | 1.37E-06 | 22.497 |
| order | Clostridiales | rs2273429 | G | A | -0.073 | 0.015 | 4.17E-06 | 22.497 |
| order | Verrucomicrobiales | rs117107102 | G | A | 0.205 | 0.043 | 2.92E-06 | 22.493 |
| order | Bacteroidales | rs7337151 | A | C | -0.113 | 0.024 | 2.35E-06 | 22.491 |
| order | Bacteroidales | rs60814075 | C | T | -0.113 | 0.024 | 2.31E-06 | 22.479 |
| order | Bacteroidales | rs12486528 | G | A | -0.064 | 0.013 | 1.55E-06 | 22.469 |
| order | Selenomonadales | rs4722181 | G | T | 0.050 | 0.011 | 2.00E-06 | 22.452 |
| order | Bacteroidales | rs17085776 | A | G | -0.113 | 0.024 | 2.65E-06 | 22.450 |
| order | Rhodospirillales | rs16855101 | T | C | 0.118 | 0.025 | 2.25E-06 | 22.441 |
| order | Bifidobacteriales | rs6710892 | G | A | 0.085 | 0.018 | 1.90E-06 | 22.439 |
| order | Bifidobacteriales | rs10928522 | C | A | 0.057 | 0.012 | 1.43E-06 | 22.435 |
| order | NB1n | rs11606187 | G | A | -0.155 | 0.033 | 3.31E-06 | 22.432 |
| order | Victivallales | rs112022900 | G | A | 0.218 | 0.046 | 2.76E-06 | 22.423 |
| order | Desulfovibrionales | rs4506934 | T | C | -0.095 | 0.020 | 2.43E-06 | 22.416 |
| order | Bifidobacteriales | rs7556781 | A | G | 0.068 | 0.014 | 4.97E-06 | 22.402 |
| order | Bacteroidales | rs17085756 | G | A | -0.113 | 0.024 | 2.66E-06 | 22.399 |
| order | Bacillales | rs4617108 | G | A | 0.249 | 0.053 | 1.98E-06 | 22.390 |
| order | Victivallales | rs45621736 | G | A | 0.218 | 0.046 | 2.75E-06 | 22.387 |
| order | Bifidobacteriales | rs1446524 | A | G | 0.057 | 0.012 | 1.39E-06 | 22.385 |
| order | Bacteroidales | rs7491419 | T | G | -0.113 | 0.024 | 2.53E-06 | 22.385 |
| order | Bifidobacteriales | rs6712127 | A | G | 0.069 | 0.015 | 5.30E-06 | 22.375 |
| order | Bifidobacteriales | rs6755391 | C | T | 0.085 | 0.018 | 2.00E-06 | 22.374 |
| order | MollicutesRF9 | rs7706512 | A | G | 0.066 | 0.014 | 2.27E-06 | 22.371 |
| order | Bacteroidales | rs74090642 | A | G | -0.113 | 0.024 | 2.72E-06 | 22.371 |
| order | Lactobacillales | rs2975653 | G | A | -0.058 | 0.012 | 1.98E-06 | 22.369 |
| order | Bacteroidales | rs11916046 | C | A | -0.063 | 0.013 | 1.79E-06 | 22.359 |
| order | Bacteroidales | rs1888899 | C | T | -0.112 | 0.024 | 3.35E-06 | 22.358 |
| order | Gastranaerophilales | rs68053172 | G | A | -0.088 | 0.019 | 1.84E-06 | 22.355 |
| order | Lactobacillales | rs2952258 | C | T | -0.058 | 0.012 | 2.01E-06 | 22.352 |
| order | Bacteroidales | rs17085809 | A | G | -0.113 | 0.024 | 2.60E-06 | 22.343 |
| order | Verrucomicrobiales | rs12908520 | A | G | 0.062 | 0.013 | 2.17E-06 | 22.340 |
| order | Gastranaerophilales | rs72765667 | A | G | -0.088 | 0.019 | 1.83E-06 | 22.340 |
| order | Bacteroidales | rs17085765 | G | C | -0.113 | 0.024 | 2.81E-06 | 22.332 |
| order | Clostridiales | rs6508055 | G | A | -0.059 | 0.013 | 2.58E-06 | 22.315 |
| order | Bifidobacteriales | rs16831899 | G | A | 0.085 | 0.018 | 2.06E-06 | 22.314 |
| order | Bifidobacteriales | rs177917 | A | G | 0.074 | 0.016 | 2.37E-06 | 22.312 |
| order | Bacteroidales | rs1014718 | G | A | -0.056 | 0.012 | 2.21E-06 | 22.308 |
| order | Bifidobacteriales | rs62168871 | A | G | 0.057 | 0.012 | 1.62E-06 | 22.305 |
| order | Desulfovibrionales | rs11599763 | C | T | -0.055 | 0.012 | 2.61E-06 | 22.305 |
| order | Bacteroidales | rs1022523 | A | G | -0.056 | 0.012 | 2.19E-06 | 22.297 |
| order | Lactobacillales | rs2952262 | T | A | -0.058 | 0.012 | 2.17E-06 | 22.285 |
| order | Methanobacteriales | rs6541968 | T | C | -0.111 | 0.024 | 2.96E-06 | 22.282 |
| order | Verrucomicrobiales | rs56161035 | C | T | 0.068 | 0.014 | 2.32E-06 | 22.280 |
| order | Bifidobacteriales | rs4954193 | A | G | 0.056 | 0.012 | 1.62E-06 | 22.277 |
| order | Bifidobacteriales | rs10176573 | C | A | 0.056 | 0.012 | 1.76E-06 | 22.271 |
| order | Bacteroidales | rs59082732 | T | G | -0.112 | 0.024 | 3.13E-06 | 22.268 |
| order | Bifidobacteriales | rs6743537 | G | A | 0.075 | 0.016 | 3.22E-06 | 22.260 |
| order | Bifidobacteriales | rs60241067 | A | T | 0.062 | 0.013 | 4.15E-06 | 22.241 |
| order | Bifidobacteriales | rs79826902 | G | A | 0.077 | 0.016 | 3.24E-06 | 22.239 |
| order | Lactobacillales | rs2952257 | T | C | -0.058 | 0.012 | 2.10E-06 | 22.233 |
| order | NB1n | rs267959 | G | A | 0.099 | 0.021 | 2.62E-06 | 22.231 |
| order | Bifidobacteriales | rs6430554 | C | T | 0.056 | 0.012 | 1.76E-06 | 22.230 |
| order | Methanobacteriales | rs9594530 | T | C | 0.210 | 0.045 | 2.55E-06 | 22.227 |
| order | Bifidobacteriales | rs111682961 | C | T | 0.077 | 0.016 | 3.32E-06 | 22.225 |
| order | Bifidobacteriales | rs7589297 | C | A | 0.056 | 0.012 | 1.81E-06 | 22.225 |
| order | Burkholderiales | rs6087810 | C | T | -0.096 | 0.020 | 2.42E-06 | 22.215 |
| order | Desulfovibrionales | rs17791387 | G | A | -0.073 | 0.015 | 2.25E-06 | 22.210 |
| order | Bacteroidales | rs7319197 | A | G | -0.112 | 0.024 | 2.48E-06 | 22.207 |
| order | MollicutesRF9 | rs74603314 | C | T | 0.231 | 0.049 | 2.28E-06 | 22.207 |
| order | Bifidobacteriales | rs16832243 | C | T | 0.077 | 0.016 | 3.09E-06 | 22.206 |
| order | Bacteroidales | rs17085785 | C | T | -0.112 | 0.024 | 2.91E-06 | 22.204 |
| order | Bacteroidales | rs7320487 | T | C | -0.112 | 0.024 | 2.48E-06 | 22.204 |
| order | Lactobacillales | rs2271029 | C | A | 0.052 | 0.011 | 2.57E-06 | 22.203 |
| order | Bacteroidales | rs2875575 | G | C | -0.112 | 0.024 | 3.44E-06 | 22.187 |
| order | Bifidobacteriales | rs309161 | G | A | 0.074 | 0.016 | 2.67E-06 | 22.180 |
| order | Bifidobacteriales | rs4334924 | G | A | 0.057 | 0.012 | 4.16E-06 | 22.177 |
| order | Clostridiales | rs11846847 | T | A | -0.072 | 0.015 | 5.16E-06 | 22.167 |
| order | Bifidobacteriales | rs41389446 | G | T | -0.087 | 0.018 | 2.47E-06 | 22.153 |
| order | Bacteroidales | rs35532963 | C | G | -0.062 | 0.013 | 2.01E-06 | 22.152 |
| order | Bifidobacteriales | rs309119 | T | C | 0.074 | 0.016 | 2.33E-06 | 22.149 |
| order | Bacteroidales | rs36076607 | T | C | -0.062 | 0.013 | 1.90E-06 | 22.144 |
| order | Bacteroidales | rs75808613 | G | A | -0.112 | 0.024 | 2.81E-06 | 22.141 |
| order | Bacteroidales | rs78745799 | G | T | -0.112 | 0.024 | 2.81E-06 | 22.141 |
| order | Bacteroidales | rs1996069 | T | C | -0.063 | 0.013 | 1.99E-06 | 22.140 |
| order | Verrucomicrobiales | rs112769434 | A | G | 0.111 | 0.024 | 2.16E-06 | 22.139 |
| order | Bifidobacteriales | rs1446525 | G | A | 0.056 | 0.012 | 1.84E-06 | 22.137 |
| order | Bacteroidales | rs71322866 | A | C | -0.063 | 0.013 | 1.93E-06 | 22.121 |
| order | Lactobacillales | rs2975654 | G | A | -0.058 | 0.012 | 2.43E-06 | 22.116 |
| order | Bifidobacteriales | rs2166480 | G | A | 0.056 | 0.012 | 1.89E-06 | 22.115 |
| order | Bifidobacteriales | rs766270 | G | C | 0.056 | 0.012 | 2.00E-06 | 22.111 |
| order | Lactobacillales | rs12595929 | C | T | 0.060 | 0.013 | 1.89E-06 | 22.110 |
| order | Rhodospirillales | rs77497586 | G | T | 0.134 | 0.028 | 1.82E-06 | 22.096 |
| order | Bifidobacteriales | rs16832205 | G | A | 0.076 | 0.016 | 3.40E-06 | 22.096 |
| order | Lactobacillales | rs11110282 | G | A | -0.102 | 0.022 | 3.96E-06 | 22.090 |
| order | Bifidobacteriales | rs6706537 | T | C | 0.056 | 0.012 | 1.74E-06 | 22.090 |
| order | Lactobacillales | rs4260895 | A | C | -0.058 | 0.012 | 1.75E-06 | 22.083 |
| order | Bacteroidales | rs34519192 | A | G | -0.062 | 0.013 | 2.08E-06 | 22.080 |
| order | Bifidobacteriales | rs1374292 | G | A | 0.056 | 0.012 | 1.80E-06 | 22.079 |
| order | Bifidobacteriales | rs2164918 | T | G | 0.091 | 0.019 | 5.28E-06 | 22.074 |
| order | Victivallales | rs17114848 | A | G | 0.152 | 0.032 | 4.06E-06 | 22.073 |
| order | Rhodospirillales | rs4531902 | T | C | 0.116 | 0.025 | 2.70E-06 | 22.071 |
| order | Bifidobacteriales | rs309170 | C | T | 0.073 | 0.016 | 2.80E-06 | 22.050 |
| order | Methanobacteriales | rs9942109 | G | A | -0.197 | 0.042 | 3.60E-06 | 22.046 |
| order | Methanobacteriales | rs62240388 | A | G | -0.196 | 0.042 | 3.06E-06 | 22.046 |
| order | Bifidobacteriales | rs6430551 | T | C | 0.056 | 0.012 | 1.64E-06 | 22.044 |
| order | Lactobacillales | rs9345899 | G | A | -0.083 | 0.018 | 3.78E-06 | 22.041 |
| order | Bacteroidales | rs1555070 | C | T | -0.056 | 0.012 | 2.53E-06 | 22.036 |
| order | Bacteroidales | rs73846120 | A | G | -0.063 | 0.013 | 2.00E-06 | 22.035 |
| order | Bifidobacteriales | rs55842567 | G | A | 0.057 | 0.012 | 4.73E-06 | 22.032 |
| order | Rhodospirillales | rs55661963 | A | C | 0.133 | 0.028 | 2.24E-06 | 22.026 |
| order | Selenomonadales | rs35797647 | C | G | 0.050 | 0.011 | 2.47E-06 | 22.023 |
| order | Selenomonadales | rs56371515 | G | C | 0.050 | 0.011 | 2.47E-06 | 22.023 |
| order | Burkholderiales | rs36119524 | C | T | 0.063 | 0.013 | 2.57E-06 | 22.020 |
| order | Lactobacillales | rs2952168 | G | C | -0.058 | 0.012 | 2.57E-06 | 22.019 |
| order | Clostridiales | rs11844758 | C | A | -0.072 | 0.015 | 5.09E-06 | 22.018 |
| order | NB1n | rs267958 | G | A | 0.098 | 0.021 | 2.93E-06 | 22.017 |
| order | Bifidobacteriales | rs62168872 | C | T | 0.056 | 0.012 | 2.05E-06 | 22.008 |
| order | Lactobacillales | rs11730038 | A | G | -0.061 | 0.013 | 5.10E-06 | 22.005 |
| order | Bifidobacteriales | rs4954197 | G | A | 0.056 | 0.012 | 2.07E-06 | 21.990 |
| order | Selenomonadales | rs35924322 | A | G | 0.050 | 0.011 | 2.58E-06 | 21.961 |
| order | Gastranaerophilales | rs4846956 | C | T | -0.087 | 0.019 | 2.18E-06 | 21.956 |
| order | Erysipelotrichales | rs9859401 | A | C | 0.050 | 0.011 | 2.70E-06 | 21.954 |
| order | Verrucomicrobiales | rs1491244 | A | T | 0.111 | 0.024 | 2.47E-06 | 21.952 |
| order | Bacteroidales | rs1325597 | G | T | -0.056 | 0.012 | 2.57E-06 | 21.946 |
| order | Selenomonadales | rs9929374 | A | G | -0.050 | 0.011 | 3.11E-06 | 21.940 |
| order | Lactobacillales | rs57872228 | T | C | -0.069 | 0.015 | 2.58E-06 | 21.933 |
| order | NB1n | rs12358808 | T | G | -0.120 | 0.026 | 5.31E-06 | 21.918 |
| order | NB1n | rs267962 | T | C | 0.098 | 0.021 | 3.09E-06 | 21.912 |
| order | Actinomycetales | rs34583783 | T | G | 0.124 | 0.026 | 5.54E-06 | 21.908 |
| order | Bacillales | rs10233278 | C | T | -0.116 | 0.025 | 3.51E-06 | 21.905 |
| order | Bacteroidales | rs34922683 | G | T | -0.062 | 0.013 | 2.32E-06 | 21.904 |
| order | Bifidobacteriales | rs4954196 | A | G | 0.056 | 0.012 | 2.16E-06 | 21.903 |
| order | Coriobacteriales | rs10995776 | C | T | 0.082 | 0.017 | 2.44E-06 | 21.903 |
| order | Methanobacteriales | rs62240380 | C | T | -0.193 | 0.041 | 3.32E-06 | 21.900 |
| order | Erysipelotrichales | rs2300773 | A | G | 0.050 | 0.011 | 2.82E-06 | 21.891 |
| order | Bacteroidales | rs17085791 | G | C | -0.111 | 0.024 | 3.28E-06 | 21.890 |
| order | Lactobacillales | rs4131679 | T | C | -0.060 | 0.013 | 5.46E-06 | 21.888 |
| order | Bacteroidales | rs6794198 | C | T | -0.062 | 0.013 | 2.12E-06 | 21.879 |
| order | Bifidobacteriales | rs11742144 | G | A | 0.057 | 0.012 | 4.22E-06 | 21.878 |
| order | Bacteroidales | rs73975615 | A | G | -0.207 | 0.044 | 1.22E-06 | 21.874 |
| order | Bifidobacteriales | rs56146525 | A | C | 0.057 | 0.012 | 5.54E-06 | 21.868 |
| order | Rhodospirillales | rs11630875 | C | T | 0.095 | 0.020 | 3.70E-06 | 21.865 |
| order | Erysipelotrichales | rs9867473 | C | A | 0.050 | 0.011 | 2.36E-06 | 21.845 |
| order | Bacteroidales | rs2346325 | A | G | -0.062 | 0.013 | 2.40E-06 | 21.834 |
| order | Lactobacillales | rs4293791 | T | C | -0.060 | 0.013 | 5.60E-06 | 21.831 |
| order | Bacteroidales | rs2183908 | T | C | -0.111 | 0.024 | 3.03E-06 | 21.811 |
| order | Bacteroidales | rs1040016 | G | T | -0.062 | 0.013 | 2.43E-06 | 21.810 |
| order | Bifidobacteriales | rs3769018 | T | C | 0.094 | 0.020 | 5.76E-06 | 21.804 |
| order | MollicutesRF9 | rs739151 | G | C | 0.065 | 0.014 | 3.09E-06 | 21.804 |
| order | Bifidobacteriales | rs79769806 | A | G | -0.086 | 0.018 | 2.93E-06 | 21.803 |
| order | Bacteroidales | rs11916939 | G | A | -0.062 | 0.013 | 2.39E-06 | 21.800 |
| order | Burkholderiales | rs76625259 | T | G | -0.094 | 0.020 | 2.91E-06 | 21.788 |
| order | Enterobacteriales | rs6092361 | G | A | 0.060 | 0.013 | 3.27E-06 | 21.786 |
| order | Bacteroidales | rs907713 | A | C | -0.062 | 0.013 | 2.24E-06 | 21.785 |
| order | Verrucomicrobiales | rs4242783 | A | G | 0.069 | 0.015 | 2.64E-06 | 21.781 |
| order | Bifidobacteriales | rs12464050 | G | T | 0.076 | 0.016 | 4.03E-06 | 21.777 |
| order | Bifidobacteriales | rs3098103 | T | C | 0.076 | 0.016 | 4.27E-06 | 21.771 |
| order | Verrucomicrobiales | rs2169719 | A | G | 0.068 | 0.015 | 3.06E-06 | 21.771 |
| order | Pasteurellales | rs6092684 | T | A | 0.068 | 0.015 | 3.44E-06 | 21.769 |
| order | Bifidobacteriales | rs62168891 | C | T | 0.056 | 0.012 | 2.43E-06 | 21.763 |
| order | Selenomonadales | rs12113541 | A | G | 0.049 | 0.011 | 3.28E-06 | 21.763 |
| order | Bifidobacteriales | rs56104584 | T | C | 0.057 | 0.012 | 5.43E-06 | 21.762 |
| order | Methanobacteriales | rs58106718 | A | T | -0.193 | 0.041 | 3.43E-06 | 21.758 |
| order | Rhodospirillales | rs2162883 | T | C | 0.090 | 0.019 | 4.27E-06 | 21.751 |
| order | Clostridiales | rs13179700 | C | T | 0.051 | 0.011 | 3.52E-06 | 21.746 |
| order | Burkholderiales | rs4591362 | G | A | 0.063 | 0.013 | 2.67E-06 | 21.745 |
| order | Enterobacteriales | rs62210023 | G | A | 0.061 | 0.013 | 3.13E-06 | 21.742 |
| order | Erysipelotrichales | rs13072608 | T | C | 0.050 | 0.011 | 3.05E-06 | 21.741 |
| order | Methanobacteriales | rs62241832 | G | A | -0.196 | 0.042 | 4.58E-06 | 21.727 |
| order | Methanobacteriales | rs56001610 | G | A | -0.195 | 0.042 | 4.49E-06 | 21.721 |
| order | Bifidobacteriales | rs11743825 | C | T | 0.056 | 0.012 | 4.71E-06 | 21.720 |
| order | Methanobacteriales | rs75208022 | T | C | -0.227 | 0.049 | 5.92E-06 | 21.717 |
| order | Methanobacteriales | rs62241845 | T | A | -0.195 | 0.042 | 4.55E-06 | 21.716 |
| order | Methanobacteriales | rs62241844 | G | C | -0.195 | 0.042 | 4.55E-06 | 21.716 |
| order | Bifidobacteriales | rs57243841 | A | G | 0.065 | 0.014 | 4.05E-06 | 21.710 |
| order | Victivallales | rs11770843 | T | C | 0.109 | 0.023 | 1.91E-06 | 21.707 |
| order | Bifidobacteriales | rs12469941 | C | T | 0.056 | 0.012 | 2.02E-06 | 21.704 |
| order | Methanobacteriales | rs3923545 | G | C | 0.207 | 0.045 | 3.20E-06 | 21.696 |
| order | Bifidobacteriales | rs12187440 | G | A | 0.056 | 0.012 | 5.25E-06 | 21.691 |
| order | NB1n | rs72671304 | C | T | 0.172 | 0.037 | 3.80E-06 | 21.688 |
| order | Methanobacteriales | rs62241837 | G | A | -0.195 | 0.042 | 4.61E-06 | 21.681 |
| order | Methanobacteriales | rs62241838 | T | C | -0.195 | 0.042 | 4.61E-06 | 21.680 |
| order | Bacteroidales | rs2346324 | A | G | -0.062 | 0.013 | 2.62E-06 | 21.667 |
| order | Bifidobacteriales | rs11896918 | A | G | 0.094 | 0.020 | 5.57E-06 | 21.662 |
| order | Bacteroidales | rs3946805 | A | G | -0.062 | 0.013 | 2.60E-06 | 21.650 |
| order | Methanobacteriales | rs62241839 | A | G | -0.195 | 0.042 | 4.68E-06 | 21.650 |
| order | Bifidobacteriales | rs56042615 | G | A | 0.056 | 0.012 | 5.83E-06 | 21.649 |
| order | NB1n | rs4383094 | C | T | 0.149 | 0.032 | 4.28E-06 | 21.646 |
| order | Bacteroidales | rs71322864 | C | A | -0.062 | 0.013 | 2.42E-06 | 21.637 |
| order | Burkholderiales | rs62395635 | C | T | 0.110 | 0.024 | 2.90E-06 | 21.621 |
| order | Bifidobacteriales | rs7588568 | C | A | -0.061 | 0.013 | 3.73E-06 | 21.612 |
| order | Bacillales | rs74420793 | G | A | -0.164 | 0.035 | 3.07E-06 | 21.600 |
| order | Erysipelotrichales | rs55764236 | T | G | -0.054 | 0.012 | 3.89E-06 | 21.598 |
| order | Coriobacteriales | rs34739816 | T | G | 0.097 | 0.021 | 3.88E-06 | 21.594 |
| order | Bifidobacteriales | rs7560412 | C | T | 0.096 | 0.021 | 6.82E-06 | 21.590 |
| order | Erysipelotrichales | rs3761717 | C | G | 0.050 | 0.011 | 3.30E-06 | 21.588 |
| order | Bifidobacteriales | rs77096272 | C | A | 0.076 | 0.016 | 4.47E-06 | 21.587 |
| order | Bacillales | rs113506874 | A | G | -0.164 | 0.035 | 3.11E-06 | 21.575 |
| order | Desulfovibrionales | rs55640204 | A | G | -0.093 | 0.020 | 3.79E-06 | 21.568 |
| order | Erysipelotrichales | rs7095708 | T | C | -0.053 | 0.011 | 4.01E-06 | 21.568 |
| order | Enterobacteriales | rs73280755 | A | G | 0.060 | 0.013 | 3.43E-06 | 21.562 |
| order | Pasteurellales | rs10965428 | A | C | -0.120 | 0.026 | 4.29E-06 | 21.561 |
| order | Verrucomicrobiales | rs11252894 | C | A | 0.077 | 0.017 | 2.94E-06 | 21.555 |
| order | Selenomonadales | rs34884022 | A | G | 0.049 | 0.011 | 3.60E-06 | 21.553 |
| order | Bifidobacteriales | rs55809728 | G | A | 0.106 | 0.023 | 1.90E-06 | 21.550 |
| order | Bifidobacteriales | rs6430555 | A | C | 0.055 | 0.012 | 2.62E-06 | 21.547 |
| order | Bifidobacteriales | rs6430550 | C | T | 0.055 | 0.012 | 2.05E-06 | 21.544 |
| order | Erysipelotrichales | rs4617508 | T | A | -0.053 | 0.011 | 4.04E-06 | 21.541 |
| order | Bifidobacteriales | rs72717412 | C | T | 0.056 | 0.012 | 5.42E-06 | 21.541 |
| order | Selenomonadales | rs35636125 | A | G | 0.049 | 0.011 | 3.21E-06 | 21.535 |
| order | Bacillales | rs4580666 | C | A | -0.164 | 0.035 | 3.61E-06 | 21.533 |
| order | Burkholderiales | rs2321387 | A | G | -0.051 | 0.011 | 3.26E-06 | 21.532 |
| order | Bifidobacteriales | rs72717410 | A | T | 0.056 | 0.012 | 5.35E-06 | 21.531 |
| order | Coriobacteriales | rs11250875 | C | T | 0.061 | 0.013 | 4.83E-06 | 21.526 |
| order | Erysipelotrichales | rs7911287 | G | T | -0.053 | 0.011 | 4.03E-06 | 21.524 |
| order | Erysipelotrichales | rs11718657 | A | C | 0.050 | 0.011 | 3.40E-06 | 21.513 |
| order | Erysipelotrichales | rs11185506 | C | G | 0.050 | 0.011 | 3.40E-06 | 21.513 |
| order | Bifidobacteriales | rs2053727 | C | T | 0.055 | 0.012 | 2.62E-06 | 21.513 |
| order | Lactobacillales | rs9445783 | A | T | -0.101 | 0.022 | 3.31E-06 | 21.507 |
| order | Bacteroidales | rs13291434 | C | T | 0.069 | 0.015 | 4.28E-06 | 21.507 |
| order | Bifidobacteriales | rs41269821 | A | G | 0.074 | 0.016 | 3.87E-06 | 21.502 |
| order | NB1n | rs7006687 | T | C | -0.092 | 0.020 | 3.99E-06 | 21.495 |
| order | Bacteroidales | rs13291169 | G | C | 0.069 | 0.015 | 3.75E-06 | 21.493 |
| order | Lactobacillales | rs78368499 | C | T | -0.101 | 0.022 | 3.16E-06 | 21.491 |
| order | Coriobacteriales | rs67561917 | G | A | -0.071 | 0.015 | 5.39E-06 | 21.486 |
| order | Bifidobacteriales | rs2197579 | C | T | 0.055 | 0.012 | 2.72E-06 | 21.486 |
| order | Methanobacteriales | rs7635580 | G | A | -0.193 | 0.042 | 4.53E-06 | 21.474 |
| order | Bifidobacteriales | rs6728946 | C | G | 0.073 | 0.016 | 4.39E-06 | 21.472 |
| order | Erysipelotrichales | rs12357537 | T | C | -0.054 | 0.012 | 4.15E-06 | 21.470 |
| order | Gastranaerophilales | rs79790072 | C | T | 0.226 | 0.049 | 3.54E-06 | 21.466 |
| order | Bifidobacteriales | rs2230437 | C | T | 0.055 | 0.012 | 5.16E-06 | 21.462 |
| order | Selenomonadales | rs1447205 | C | G | -0.050 | 0.011 | 2.93E-06 | 21.458 |
| order | Pasteurellales | rs35510 | G | A | 0.123 | 0.026 | 4.02E-06 | 21.456 |
| order | Bacteroidales | rs34046877 | T | C | -0.061 | 0.013 | 2.67E-06 | 21.456 |
| order | Bifidobacteriales | rs1592 | A | C | 0.055 | 0.012 | 4.46E-06 | 21.453 |
| order | Bifidobacteriales | rs309159 | C | T | 0.073 | 0.016 | 3.51E-06 | 21.435 |
| order | Bifidobacteriales | rs1374291 | T | C | 0.055 | 0.012 | 2.73E-06 | 21.433 |
| order | Bacteroidales | rs12496485 | G | A | -0.062 | 0.013 | 2.72E-06 | 21.431 |
| order | Selenomonadales | rs12112489 | C | T | 0.049 | 0.011 | 3.84E-06 | 21.430 |
| order | Bifidobacteriales | rs586964 | G | A | 0.075 | 0.016 | 4.90E-06 | 21.428 |
| order | Lactobacillales | rs12368593 | A | C | -0.102 | 0.022 | 5.44E-06 | 21.406 |
| order | Erysipelotrichales | rs9859260 | T | C | 0.049 | 0.011 | 3.63E-06 | 21.405 |
| order | Bacteroidales | rs13100590 | T | C | -0.061 | 0.013 | 3.00E-06 | 21.404 |
| order | Bifidobacteriales | rs62181700 | A | G | -0.060 | 0.013 | 4.12E-06 | 21.403 |
| order | Bifidobacteriales | rs72711368 | C | T | 0.057 | 0.012 | 4.75E-06 | 21.403 |
| order | Bifidobacteriales | rs56230617 | G | A | 0.073 | 0.016 | 2.30E-06 | 21.402 |
| order | Rhodospirillales | rs1035406 | A | G | -0.115 | 0.025 | 4.07E-06 | 21.401 |
| order | Rhodospirillales | rs7001029 | T | C | 0.121 | 0.026 | 2.83E-06 | 21.397 |
| order | NB1n | rs55921101 | T | A | -0.109 | 0.024 | 4.32E-06 | 21.392 |
| order | Bifidobacteriales | rs73958638 | A | G | 0.112 | 0.024 | 2.36E-06 | 21.387 |
| order | Bifidobacteriales | rs499483 | G | A | 0.075 | 0.016 | 4.99E-06 | 21.383 |
| order | Gastranaerophilales | rs73074665 | T | A | 0.165 | 0.036 | 3.62E-06 | 21.381 |
| order | Desulfovibrionales | rs9928243 | A | C | -0.054 | 0.012 | 3.97E-06 | 21.378 |
| order | Bifidobacteriales | rs4954191 | C | G | 0.055 | 0.012 | 2.34E-06 | 21.372 |
| order | Bifidobacteriales | rs309158 | A | G | 0.071 | 0.015 | 4.45E-06 | 21.372 |
| order | Lactobacillales | rs7959495 | A | C | -0.101 | 0.022 | 6.13E-06 | 21.366 |
| order | Bifidobacteriales | rs520449 | T | C | -0.057 | 0.012 | 1.83E-06 | 21.366 |
| order | Enterobacteriales | rs2374343 | G | C | 0.058 | 0.013 | 4.93E-06 | 21.365 |
| order | Selenomonadales | rs56188349 | C | T | -0.064 | 0.014 | 4.41E-06 | 21.360 |
| order | Bifidobacteriales | rs1056465 | T | A | 0.056 | 0.012 | 7.13E-06 | 21.360 |
| order | Bifidobacteriales | rs59213715 | A | C | 0.075 | 0.016 | 4.30E-06 | 21.355 |
| order | Bacteroidales | rs17026797 | G | T | -0.061 | 0.013 | 3.07E-06 | 21.353 |
| order | Actinomycetales | rs6488662 | T | C | 0.081 | 0.018 | 3.94E-06 | 21.345 |
| order | Bacteroidales | rs17026821 | T | C | -0.061 | 0.013 | 3.09E-06 | 21.345 |
| order | Bifidobacteriales | rs34847072 | G | C | 0.056 | 0.012 | 6.71E-06 | 21.338 |
| order | Enterobacteriales | rs2374342 | A | C | 0.058 | 0.013 | 4.52E-06 | 21.338 |
| order | Bifidobacteriales | rs56302516 | C | T | 0.062 | 0.013 | 3.49E-06 | 21.336 |
| order | Verrucomicrobiales | rs6832119 | A | G | 0.109 | 0.024 | 3.44E-06 | 21.336 |
| order | Victivallales | rs2731834 | G | C | 0.109 | 0.024 | 4.24E-06 | 21.335 |
| order | Rhodospirillales | rs16855116 | C | T | 0.115 | 0.025 | 4.23E-06 | 21.333 |
| order | Erysipelotrichales | rs9877119 | G | A | 0.049 | 0.011 | 3.74E-06 | 21.332 |
| order | Bacteroidales | rs35176045 | C | T | -0.061 | 0.013 | 2.70E-06 | 21.330 |
| order | Bifidobacteriales | rs309120 | G | C | 0.072 | 0.016 | 3.59E-06 | 21.323 |
| order | Bacillales | rs75545728 | C | T | -0.164 | 0.035 | 3.93E-06 | 21.322 |
| order | NB1n | rs13385922 | C | T | 0.093 | 0.020 | 3.97E-06 | 21.311 |
| order | NB1n | rs7088133 | C | T | -0.110 | 0.024 | 4.38E-06 | 21.309 |
| order | Pasteurellales | rs72756943 | A | G | 0.140 | 0.030 | 3.35E-06 | 21.308 |
| order | Gastranaerophilales | rs7549147 | G | A | -0.086 | 0.019 | 3.13E-06 | 21.306 |
| order | Pasteurellales | rs35509 | A | G | 0.122 | 0.027 | 4.39E-06 | 21.298 |
| order | Actinomycetales | rs2110087 | G | A | 0.079 | 0.017 | 4.28E-06 | 21.295 |
| order | Methanobacteriales | rs11123059 | A | G | -0.108 | 0.023 | 4.87E-06 | 21.279 |
| order | Methanobacteriales | rs62241827 | T | G | -0.193 | 0.042 | 5.35E-06 | 21.274 |
| order | Bifidobacteriales | rs73958614 | A | C | 0.093 | 0.020 | 6.78E-06 | 21.270 |
| order | Bifidobacteriales | rs309167 | C | T | 0.075 | 0.016 | 5.12E-06 | 21.266 |
| order | Bifidobacteriales | rs6711493 | A | G | 0.071 | 0.015 | 4.13E-06 | 21.265 |
| order | Bifidobacteriales | rs309166 | C | T | 0.072 | 0.016 | 4.11E-06 | 21.264 |
| order | Methanobacteriales | rs62241829 | C | T | -0.193 | 0.042 | 5.39E-06 | 21.263 |
| order | Bifidobacteriales | rs34453673 | G | C | 0.056 | 0.012 | 6.43E-06 | 21.255 |
| order | Verrucomicrobiales | rs111862613 | C | T | 0.091 | 0.020 | 3.74E-06 | 21.252 |
| order | Erysipelotrichales | rs8003149 | T | C | 0.054 | 0.012 | 4.08E-06 | 21.248 |
| order | Burkholderiales | rs4507125 | A | C | 0.061 | 0.013 | 4.24E-06 | 21.245 |
| order | Methanobacteriales | rs62241828 | A | G | -0.193 | 0.042 | 5.45E-06 | 21.243 |
| order | Selenomonadales | rs72643860 | A | G | -0.050 | 0.011 | 3.35E-06 | 21.239 |
| order | Selenomonadales | rs61249479 | C | A | 0.078 | 0.017 | 2.95E-06 | 21.236 |
| order | Lactobacillales | rs80243853 | A | G | -0.102 | 0.022 | 6.09E-06 | 21.231 |
| order | Verrucomicrobiales | rs11184341 | C | G | 0.066 | 0.014 | 4.13E-06 | 21.230 |
| order | Verrucomicrobiales | rs77055143 | C | T | 0.067 | 0.014 | 3.88E-06 | 21.222 |
| order | Bifidobacteriales | rs12469411 | C | T | 0.055 | 0.012 | 2.81E-06 | 21.219 |
| order | Verrucomicrobiales | rs12416161 | C | T | 0.076 | 0.017 | 3.50E-06 | 21.210 |
| order | Lactobacillales | rs34989881 | G | A | 0.113 | 0.025 | 4.09E-06 | 21.201 |
| order | Lactobacillales | rs2975662 | G | C | -0.056 | 0.012 | 3.91E-06 | 21.201 |
| order | Bifidobacteriales | rs12188164 | C | A | 0.056 | 0.012 | 6.17E-06 | 21.200 |
| order | Bifidobacteriales | rs56346498 | C | T | 0.056 | 0.012 | 6.30E-06 | 21.198 |
| order | Bacillales | rs17265230 | T | C | -0.163 | 0.035 | 4.20E-06 | 21.191 |
| order | Bifidobacteriales | rs2839740 | G | T | 0.075 | 0.016 | 5.22E-06 | 21.182 |
| order | Bacteroidales | rs13060907 | G | A | -0.061 | 0.013 | 3.17E-06 | 21.181 |
| order | Methanobacteriales | rs55721046 | A | G | -0.191 | 0.041 | 6.06E-06 | 21.171 |
| order | Bacillales | rs80042174 | G | A | -0.163 | 0.036 | 3.80E-06 | 21.169 |
| order | Rhodospirillales | rs1549633 | C | A | 0.100 | 0.022 | 3.88E-06 | 21.163 |
| order | Bifidobacteriales | rs12188678 | T | C | 0.056 | 0.012 | 6.26E-06 | 21.161 |
| order | Burkholderiales | rs12477314 | C | T | 0.062 | 0.013 | 3.67E-06 | 21.158 |
| order | NB1n | rs267954 | T | A | 0.096 | 0.021 | 4.65E-06 | 21.157 |
| order | Lactobacillales | rs11110276 | G | T | -0.100 | 0.022 | 6.24E-06 | 21.157 |
| order | Burkholderiales | rs72747231 | G | C | -0.134 | 0.029 | 3.76E-06 | 21.157 |
| order | Pasteurellales | rs4822728 | C | T | 0.069 | 0.015 | 4.72E-06 | 21.156 |
| order | Verrucomicrobiales | rs12498518 | C | T | 0.067 | 0.014 | 3.94E-06 | 21.153 |
| order | Lactobacillales | rs7187700 | G | A | 0.058 | 0.013 | 3.31E-06 | 21.152 |
| order | Bacteroidales | rs6777119 | T | C | -0.061 | 0.013 | 3.35E-06 | 21.148 |
| order | Bacteroidales | rs73510192 | T | C | -0.111 | 0.024 | 4.18E-06 | 21.147 |
| order | Desulfovibrionales | rs112381107 | T | C | 0.210 | 0.046 | 3.22E-06 | 21.136 |
| order | Bifidobacteriales | rs72717432 | G | A | 0.056 | 0.012 | 6.69E-06 | 21.129 |
| order | Pasteurellales | rs55676407 | A | G | 0.134 | 0.029 | 3.41E-06 | 21.127 |
| order | Selenomonadales | rs34660854 | G | A | 0.061 | 0.013 | 4.26E-06 | 21.114 |
| order | Bifidobacteriales | rs309118 | G | C | 0.071 | 0.015 | 5.44E-06 | 21.109 |
| order | NB1n | rs1135727 | A | G | 0.091 | 0.020 | 4.54E-06 | 21.105 |
| order | Clostridiales | rs10774377 | G | A | 0.052 | 0.011 | 3.81E-06 | 21.099 |
| order | MollicutesRF9 | rs62188991 | C | G | -0.111 | 0.024 | 5.27E-06 | 21.099 |
| order | Burkholderiales | rs62191106 | C | T | 0.062 | 0.013 | 4.21E-06 | 21.096 |
| order | Bifidobacteriales | rs309165 | T | C | 0.070 | 0.015 | 5.00E-06 | 21.093 |
| order | Pasteurellales | rs73139353 | C | A | -0.223 | 0.048 | 8.71E-06 | 21.092 |
| order | NB1n | rs6597703 | A | C | -0.109 | 0.024 | 5.14E-06 | 21.087 |
| order | Lactobacillales | rs12642660 | G | A | -0.071 | 0.016 | 6.92E-06 | 21.086 |
| order | Bifidobacteriales | rs7573555 | T | C | 0.073 | 0.016 | 5.12E-06 | 21.086 |
| order | Bifidobacteriales | rs79954860 | A | T | 0.112 | 0.024 | 3.72E-06 | 21.085 |
| order | Burkholderiales | rs1928341 | G | A | 0.051 | 0.011 | 4.52E-06 | 21.084 |
| order | Burkholderiales | rs7988877 | T | A | -0.051 | 0.011 | 4.02E-06 | 21.083 |
| order | Bacillales | rs113037786 | C | T | -0.163 | 0.036 | 4.08E-06 | 21.081 |
| order | Bacteroidales | rs11871360 | A | G | -0.203 | 0.044 | 1.86E-06 | 21.080 |
| order | Lactobacillales | rs8075910 | G | T | 0.051 | 0.011 | 4.86E-06 | 21.079 |
| order | MollicutesRF9 | rs515984 | C | T | -0.088 | 0.019 | 6.61E-06 | 21.078 |
| order | Bifidobacteriales | rs7600925 | A | G | 0.093 | 0.020 | 7.06E-06 | 21.077 |
| order | Bifidobacteriales | rs857444 | T | C | 0.055 | 0.012 | 3.82E-06 | 21.075 |
| order | Bacteroidales | rs17343978 | C | A | -0.055 | 0.012 | 8.36E-06 | 21.067 |
| order | Bifidobacteriales | rs3098102 | C | T | 0.072 | 0.016 | 4.70E-06 | 21.060 |
| order | Bifidobacteriales | rs1446523 | G | A | 0.055 | 0.012 | 2.94E-06 | 21.057 |
| order | Lactobacillales | rs12600469 | T | G | 0.051 | 0.011 | 5.35E-06 | 21.056 |
| order | Bacillales | rs10410917 | C | T | 0.115 | 0.025 | 5.57E-06 | 21.049 |
| order | Enterobacteriales | rs6069871 | G | A | 0.060 | 0.013 | 4.49E-06 | 21.049 |
| order | Enterobacteriales | rs80319214 | G | C | 0.099 | 0.022 | 6.95E-06 | 21.048 |
| order | Bifidobacteriales | rs660002 | C | T | 0.070 | 0.015 | 4.93E-06 | 21.046 |
| order | Methanobacteriales | rs12303159 | C | A | -0.223 | 0.049 | 8.56E-06 | 21.045 |
| order | Erysipelotrichales | rs17530232 | G | A | 0.103 | 0.022 | 2.79E-06 | 21.042 |
| order | Bifidobacteriales | rs72717407 | G | A | 0.056 | 0.012 | 6.68E-06 | 21.042 |
| order | Burkholderiales | rs7593823 | G | A | 0.060 | 0.013 | 4.78E-06 | 21.035 |
| order | Burkholderiales | rs62191107 | T | C | 0.062 | 0.013 | 4.39E-06 | 21.032 |
| order | Bifidobacteriales | rs1057031 | G | A | 0.073 | 0.016 | 5.56E-06 | 21.027 |
| order | Bifidobacteriales | rs1960171 | C | T | 0.060 | 0.013 | 5.82E-06 | 21.026 |
| order | Burkholderiales | rs7638039 | C | T | 0.058 | 0.013 | 4.84E-06 | 21.017 |
| order | Bifidobacteriales | rs79106992 | C | T | -0.084 | 0.018 | 4.83E-06 | 21.017 |
| order | NB1n | rs6051857 | G | A | -0.134 | 0.029 | 7.88E-06 | 21.016 |
| order | Verrucomicrobiales | rs12501826 | G | C | 0.066 | 0.014 | 4.30E-06 | 21.016 |
| order | Bacteroidales | rs6791217 | G | T | -0.061 | 0.013 | 3.44E-06 | 21.010 |
| order | Bifidobacteriales | rs7606267 | T | C | 0.073 | 0.016 | 5.37E-06 | 21.009 |
| order | Bifidobacteriales | rs189135960 | C | T | 0.093 | 0.020 | 8.88E-06 | 21.008 |
| order | Bifidobacteriales | rs12986776 | G | C | 0.056 | 0.012 | 5.91E-06 | 20.999 |
| order | Bifidobacteriales | rs72717436 | C | A | 0.055 | 0.012 | 7.43E-06 | 20.995 |
| order | Verrucomicrobiales | rs6832927 | C | A | 0.108 | 0.024 | 4.23E-06 | 20.989 |
| order | Bacillales | rs6821115 | G | A | -0.162 | 0.035 | 4.90E-06 | 20.985 |
| order | Desulfovibrionales | rs2838334 | A | G | 0.057 | 0.012 | 4.17E-06 | 20.982 |
| order | Bifidobacteriales | rs75753154 | T | C | 0.073 | 0.016 | 5.47E-06 | 20.982 |
| order | Bacillales | rs4429742 | T | A | -0.162 | 0.035 | 4.90E-06 | 20.978 |
| order | Bacillales | rs75921814 | C | A | -0.163 | 0.036 | 4.36E-06 | 20.977 |
| order | Bifidobacteriales | rs3099428 | C | G | 0.072 | 0.016 | 4.96E-06 | 20.972 |
| order | Bifidobacteriales | rs2305165 | A | C | 0.093 | 0.020 | 8.48E-06 | 20.966 |
| order | Lactobacillales | rs11110281 | C | T | -0.102 | 0.022 | 7.03E-06 | 20.961 |
| order | Bifidobacteriales | rs540489 | G | T | -0.063 | 0.014 | 5.37E-06 | 20.957 |
| order | Bifidobacteriales | rs614193 | A | T | 0.072 | 0.016 | 5.14E-06 | 20.947 |
| order | Verrucomicrobiales | rs11724791 | G | A | 0.066 | 0.014 | 4.86E-06 | 20.946 |
| order | Bacillales | rs2391098 | T | C | -0.163 | 0.036 | 4.46E-06 | 20.942 |
| order | Bifidobacteriales | rs72717411 | C | G | 0.055 | 0.012 | 7.15E-06 | 20.939 |
| order | Methanobacteriales | rs62241826 | T | C | -0.190 | 0.041 | 6.95E-06 | 20.937 |
| order | MollicutesRF9 | rs483881 | A | G | -0.087 | 0.019 | 7.18E-06 | 20.935 |
| order | NB1n | rs72834940 | T | G | -0.108 | 0.024 | 5.35E-06 | 20.931 |
| order | Bifidobacteriales | rs112758679 | A | C | 0.073 | 0.016 | 5.42E-06 | 20.930 |
| order | NB1n | rs12358432 | C | T | -0.114 | 0.025 | 9.43E-06 | 20.929 |
| order | Bifidobacteriales | rs13410301 | T | C | 0.054 | 0.012 | 5.79E-06 | 20.925 |
| order | Enterobacteriales | rs111229068 | T | A | 0.111 | 0.024 | 3.65E-06 | 20.921 |
| order | Bifidobacteriales | rs766271 | C | T | 0.054 | 0.012 | 5.98E-06 | 20.918 |
| order | Bacteroidales | rs1996068 | A | G | -0.060 | 0.013 | 3.94E-06 | 20.911 |
| order | Lactobacillales | rs11835118 | C | T | -0.100 | 0.022 | 7.12E-06 | 20.908 |
| order | Erysipelotrichales | rs3804140 | C | T | 0.049 | 0.011 | 4.68E-06 | 20.907 |
| order | Bifidobacteriales | rs67665078 | G | A | 0.053 | 0.012 | 4.93E-06 | 20.906 |
| order | Bifidobacteriales | rs55932239 | G | A | 0.055 | 0.012 | 7.30E-06 | 20.904 |
| order | Burkholderiales | rs2613606 | T | C | -0.050 | 0.011 | 4.13E-06 | 20.904 |
| order | Enterobacteriales | rs7271805 | C | A | 0.059 | 0.013 | 4.77E-06 | 20.898 |
| order | Lactobacillales | rs80205102 | T | C | -0.059 | 0.013 | 9.06E-06 | 20.895 |
| order | Rhodospirillales | rs16855133 | A | G | 0.113 | 0.025 | 4.17E-06 | 20.894 |
| order | Bifidobacteriales | rs75128007 | A | G | 0.062 | 0.014 | 6.87E-06 | 20.892 |
| order | Rhodospirillales | rs61933850 | A | G | 0.165 | 0.036 | 7.00E-06 | 20.888 |
| order | Clostridiales | rs6126494 | T | G | -0.097 | 0.021 | 9.51E-06 | 20.886 |
| order | Burkholderiales | rs1859743 | C | T | -0.050 | 0.011 | 3.94E-06 | 20.884 |
| order | Bifidobacteriales | rs895285 | A | G | 0.074 | 0.016 | 5.52E-06 | 20.882 |
| order | Bacillales | rs74842568 | A | T | -0.162 | 0.036 | 4.58E-06 | 20.876 |
| order | Rhodospirillales | rs11591293 | T | G | 0.072 | 0.016 | 4.69E-06 | 20.876 |
| order | Bacillales | rs12500894 | T | C | -0.162 | 0.036 | 4.62E-06 | 20.871 |
| order | Bifidobacteriales | rs6430549 | A | G | 0.055 | 0.012 | 3.22E-06 | 20.869 |
| order | Rhodospirillales | rs35936958 | G | T | 0.099 | 0.022 | 6.90E-06 | 20.860 |
| order | Verrucomicrobiales | rs59317921 | C | T | 0.063 | 0.014 | 4.62E-06 | 20.858 |
| order | Burkholderiales | rs1511453 | G | A | 0.091 | 0.020 | 8.00E-06 | 20.856 |
| order | Verrucomicrobiales | rs1605923 | T | G | 0.066 | 0.014 | 4.84E-06 | 20.856 |
| order | Rhodospirillales | rs56250014 | T | A | 0.117 | 0.026 | 4.67E-06 | 20.854 |
| order | Bifidobacteriales | rs2053726 | A | G | 0.055 | 0.012 | 3.21E-06 | 20.847 |
| order | Desulfovibrionales | rs72821212 | G | C | -0.092 | 0.020 | 5.32E-06 | 20.846 |
| order | Clostridiales | rs1497088 | G | C | -0.070 | 0.015 | 9.14E-06 | 20.844 |
| order | Burkholderiales | rs9964679 | G | A | 0.053 | 0.012 | 6.06E-06 | 20.834 |
| order | Bifidobacteriales | rs4954275 | C | T | 0.092 | 0.020 | 8.43E-06 | 20.826 |
| order | Bifidobacteriales | rs115554641 | C | G | 0.055 | 0.012 | 8.60E-06 | 20.824 |
| order | NB1n | rs72834933 | A | G | -0.108 | 0.024 | 5.79E-06 | 20.818 |
| order | Bifidobacteriales | rs56182240 | C | T | 0.055 | 0.012 | 7.67E-06 | 20.818 |
| order | Selenomonadales | rs56249650 | C | A | 0.048 | 0.011 | 5.35E-06 | 20.813 |
| order | Rhodospirillales | rs4822789 | C | G | 0.073 | 0.016 | 7.33E-06 | 20.813 |
| order | Bacteroidales | rs73510188 | G | A | -0.110 | 0.024 | 4.75E-06 | 20.810 |
| order | Bacteroidales | rs73510190 | G | C | -0.110 | 0.024 | 4.75E-06 | 20.810 |
| order | Bifidobacteriales | rs309142 | T | C | 0.070 | 0.015 | 5.19E-06 | 20.807 |
| order | Verrucomicrobiales | rs6668273 | G | A | 0.065 | 0.014 | 5.26E-06 | 20.805 |
| order | Bacillales | rs11207728 | A | G | -0.145 | 0.032 | 5.73E-06 | 20.805 |
| order | Pasteurellales | rs731534 | G | C | -0.099 | 0.022 | 9.46E-06 | 20.798 |
| order | Bifidobacteriales | rs3099427 | A | G | 0.071 | 0.016 | 5.48E-06 | 20.797 |
| order | Selenomonadales | rs4236917 | G | C | -0.049 | 0.011 | 4.15E-06 | 20.795 |
| order | Selenomonadales | rs4736971 | C | A | -0.049 | 0.011 | 4.19E-06 | 20.794 |
| order | Verrucomicrobiales | rs113653192 | C | T | 0.108 | 0.024 | 4.68E-06 | 20.789 |
| order | Bifidobacteriales | rs113974791 | T | G | 0.072 | 0.016 | 5.92E-06 | 20.784 |
| order | Verrucomicrobiales | rs72663787 | G | A | 0.066 | 0.015 | 5.08E-06 | 20.783 |
| order | Clostridiales | rs9986136 | G | A | 0.050 | 0.011 | 5.37E-06 | 20.781 |
| order | Actinomycetales | rs114313061 | A | C | 0.080 | 0.018 | 4.73E-06 | 20.765 |
| order | Actinomycetales | rs6488660 | C | T | 0.079 | 0.017 | 5.32E-06 | 20.763 |
| order | Bifidobacteriales | rs6889494 | C | T | 0.055 | 0.012 | 7.91E-06 | 20.759 |
| order | Burkholderiales | rs1581853 | G | A | -0.051 | 0.011 | 4.72E-06 | 20.759 |
| order | Rhodospirillales | rs77468690 | A | T | 0.141 | 0.031 | 6.43E-06 | 20.755 |
| order | Bifidobacteriales | rs479980 | A | C | -0.056 | 0.012 | 4.29E-06 | 20.754 |
| order | Desulfovibrionales | rs7199026 | A | C | -0.054 | 0.012 | 5.54E-06 | 20.749 |
| order | Erysipelotrichales | rs3804139 | C | T | 0.049 | 0.011 | 5.08E-06 | 20.748 |
| order | Bifidobacteriales | rs56784995 | T | C | 0.092 | 0.020 | 8.31E-06 | 20.748 |
| order | Bacillales | rs76274770 | G | C | -0.161 | 0.035 | 5.51E-06 | 20.743 |
| order | Bifidobacteriales | rs3814358 | A | C | 0.054 | 0.012 | 3.52E-06 | 20.741 |
| order | Gastranaerophilales | rs12746891 | A | G | -0.085 | 0.019 | 4.25E-06 | 20.737 |
| order | Bifidobacteriales | rs72717415 | G | A | 0.055 | 0.012 | 7.98E-06 | 20.733 |
| order | Clostridiales | rs7269302 | T | A | -0.097 | 0.021 | 8.84E-06 | 20.733 |
| order | Actinomycetales | rs11055861 | A | G | 0.079 | 0.017 | 5.43E-06 | 20.732 |
| order | Actinomycetales | rs6488659 | T | C | 0.079 | 0.017 | 5.42E-06 | 20.731 |
| order | Pasteurellales | rs62568866 | A | T | -0.118 | 0.026 | 7.23E-06 | 20.730 |
| order | Actinomycetales | rs4073240 | A | G | 0.075 | 0.016 | 5.68E-06 | 20.729 |
| order | Actinomycetales | rs11055860 | A | G | 0.079 | 0.017 | 5.46E-06 | 20.724 |
| order | Coriobacteriales | rs80046645 | G | C | 0.256 | 0.056 | 4.66E-06 | 20.724 |
| order | Erysipelotrichales | rs4078432 | T | C | -0.061 | 0.013 | 4.23E-06 | 20.723 |
| order | Burkholderiales | rs10788864 | A | T | 0.050 | 0.011 | 5.21E-06 | 20.723 |
| order | Methanobacteriales | rs62240390 | C | G | -0.189 | 0.042 | 6.79E-06 | 20.722 |
| order | Methanobacteriales | rs73248946 | A | G | -0.222 | 0.049 | 9.83E-06 | 20.722 |
| order | Bacillales | rs75919743 | G | A | -0.162 | 0.036 | 4.97E-06 | 20.722 |
| order | NB1n | rs72834941 | G | A | -0.108 | 0.024 | 6.10E-06 | 20.708 |
| order | Bifidobacteriales | rs7593370 | C | T | 0.054 | 0.012 | 3.60E-06 | 20.708 |
| order | Selenomonadales | rs2354569 | T | C | -0.049 | 0.011 | 4.39E-06 | 20.707 |
| order | Bifidobacteriales | rs72717440 | A | T | 0.055 | 0.012 | 8.33E-06 | 20.704 |
| order | Lactobacillales | rs113280052 | T | C | -0.102 | 0.022 | 5.78E-06 | 20.701 |
| order | Actinomycetales | rs7488453 | A | G | 0.079 | 0.017 | 5.51E-06 | 20.700 |
| order | Lactobacillales | rs11627423 | A | C | 0.050 | 0.011 | 5.09E-06 | 20.696 |
| order | Rhodospirillales | rs6759037 | G | A | 0.113 | 0.025 | 5.71E-06 | 20.694 |
| order | Bacteroidales | rs60475411 | T | C | -0.060 | 0.013 | 4.32E-06 | 20.694 |
| order | Bacillales | rs112249356 | G | T | -0.161 | 0.035 | 5.19E-06 | 20.693 |
| order | Rhodospirillales | rs1366438 | A | T | 0.088 | 0.019 | 7.56E-06 | 20.688 |
| order | Verrucomicrobiales | rs3995795 | T | C | 0.064 | 0.014 | 5.19E-06 | 20.686 |
| order | Rhodospirillales | rs67973976 | T | C | 0.119 | 0.026 | 3.97E-06 | 20.686 |
| order | Burkholderiales | rs12867878 | G | A | -0.051 | 0.011 | 4.65E-06 | 20.683 |
| order | Burkholderiales | rs9537878 | G | A | -0.050 | 0.011 | 5.09E-06 | 20.680 |
| order | Bifidobacteriales | rs16832162 | T | A | 0.072 | 0.016 | 6.39E-06 | 20.677 |
| order | Actinomycetales | rs17072071 | A | C | -0.112 | 0.025 | 6.45E-06 | 20.676 |
| order | Erysipelotrichales | rs34221479 | C | T | 0.049 | 0.011 | 5.79E-06 | 20.675 |
| order | Bacteroidales | rs35665203 | T | C | -0.061 | 0.013 | 4.04E-06 | 20.672 |
| order | Bifidobacteriales | rs11739543 | G | A | 0.055 | 0.012 | 8.63E-06 | 20.670 |
| order | NB1n | rs3911620 | T | C | -0.108 | 0.024 | 6.02E-06 | 20.669 |
| order | Methanobacteriales | rs56131665 | A | G | 0.179 | 0.039 | 6.18E-06 | 20.669 |
| order | Bifidobacteriales | rs3087343 | T | G | 0.072 | 0.016 | 6.64E-06 | 20.663 |
| order | MollicutesRF9 | rs114184530 | C | T | -0.155 | 0.034 | 7.88E-06 | 20.662 |
| order | Actinomycetales | rs2377095 | C | A | 0.079 | 0.017 | 5.64E-06 | 20.658 |
| order | Bacillales | rs112469872 | G | A | -0.162 | 0.036 | 5.03E-06 | 20.658 |
| order | Clostridiales | rs72738886 | C | T | 0.087 | 0.019 | 8.42E-06 | 20.657 |
| order | Victivallales | rs73113483 | A | T | -0.131 | 0.029 | 8.66E-06 | 20.656 |
| order | Actinomycetales | rs6488665 | T | C | 0.079 | 0.017 | 5.69E-06 | 20.656 |
| order | Coriobacteriales | rs1816223 | G | A | -0.059 | 0.013 | 4.84E-06 | 20.652 |
| order | Erysipelotrichales | rs9990392 | T | C | 0.049 | 0.011 | 4.94E-06 | 20.647 |
| order | Bifidobacteriales | rs3112496 | C | T | 0.071 | 0.016 | 5.05E-06 | 20.643 |
| order | Bifidobacteriales | rs4954555 | T | C | 0.107 | 0.023 | 3.48E-06 | 20.639 |
| order | MollicutesRF9 | rs609343 | A | T | -0.087 | 0.019 | 8.31E-06 | 20.638 |
| order | Bifidobacteriales | rs7569447 | A | C | 0.077 | 0.017 | 2.95E-06 | 20.635 |
| order | Bacillales | rs12509041 | G | A | -0.169 | 0.037 | 5.45E-06 | 20.631 |
| order | Lactobacillales | rs2952183 | C | T | -0.069 | 0.015 | 3.36E-06 | 20.631 |
| order | Selenomonadales | rs9423647 | A | G | 0.048 | 0.011 | 6.06E-06 | 20.628 |
| order | Bifidobacteriales | rs11740891 | A | G | 0.054 | 0.012 | 6.11E-06 | 20.625 |
| order | NB1n | rs9542068 | C | T | 0.099 | 0.022 | 6.52E-06 | 20.625 |
| order | Bacillales | rs62640857 | G | A | 0.148 | 0.033 | 4.49E-06 | 20.619 |
| order | Bifidobacteriales | rs11678502 | T | C | 0.055 | 0.012 | 6.90E-06 | 20.618 |
| order | Bifidobacteriales | rs78364332 | T | C | 0.072 | 0.016 | 6.82E-06 | 20.617 |
| order | Lactobacillales | rs11837200 | T | C | -0.099 | 0.022 | 8.21E-06 | 20.615 |
| order | Bifidobacteriales | rs309171 | G | C | 0.072 | 0.016 | 6.48E-06 | 20.613 |
| order | Erysipelotrichales | rs11146160 | A | G | -0.052 | 0.011 | 6.47E-06 | 20.611 |
| order | Clostridiales | rs11167476 | C | T | 0.050 | 0.011 | 5.96E-06 | 20.608 |
| order | Bacillales | rs11034576 | G | A | 0.206 | 0.045 | 8.86E-06 | 20.604 |
| order | Burkholderiales | rs2094636 | C | G | 0.050 | 0.011 | 5.58E-06 | 20.595 |
| order | Clostridiales | rs7731599 | T | C | 0.050 | 0.011 | 5.93E-06 | 20.592 |
| order | Burkholderiales | rs7324740 | A | G | -0.051 | 0.011 | 5.14E-06 | 20.588 |
| order | Bacillales | rs76491594 | G | A | -0.161 | 0.036 | 5.30E-06 | 20.587 |
| order | Clostridiales | rs10774378 | C | T | 0.052 | 0.011 | 5.04E-06 | 20.583 |
| order | Lactobacillales | rs28491054 | A | G | -0.069 | 0.015 | 3.53E-06 | 20.582 |
| order | Lactobacillales | rs78017832 | G | T | -0.101 | 0.022 | 8.69E-06 | 20.575 |
| order | Burkholderiales | rs9553336 | T | G | 0.093 | 0.020 | 4.23E-06 | 20.574 |
| order | NB1n | rs9317839 | C | T | 0.099 | 0.022 | 6.83E-06 | 20.564 |
| order | Bifidobacteriales | rs12472293 | T | C | 0.072 | 0.016 | 6.61E-06 | 20.561 |
| order | Actinomycetales | rs2889192 | T | G | 0.088 | 0.019 | 3.97E-06 | 20.554 |
| order | Burkholderiales | rs4308141 | C | G | 0.061 | 0.013 | 6.23E-06 | 20.552 |
| order | Lactobacillales | rs67455183 | T | G | -0.071 | 0.016 | 5.51E-06 | 20.549 |
| order | Verrucomicrobiales | rs72663782 | G | C | 0.066 | 0.014 | 5.35E-06 | 20.548 |
| order | Bifidobacteriales | rs72717414 | C | G | 0.055 | 0.012 | 8.75E-06 | 20.544 |
| order | Victivallales | rs11764871 | T | G | 0.106 | 0.023 | 6.39E-06 | 20.535 |
| order | Clostridiales | rs4764517 | T | C | 0.052 | 0.011 | 4.98E-06 | 20.534 |
| order | Pasteurellales | rs7270573 | G | C | -0.068 | 0.015 | 5.58E-06 | 20.534 |
| order | Pasteurellales | rs7115142 | A | G | -0.068 | 0.015 | 5.97E-06 | 20.533 |
| order | Burkholderiales | rs34177861 | G | A | 0.061 | 0.013 | 4.97E-06 | 20.531 |
| order | Desulfovibrionales | rs3935584 | T | C | -0.052 | 0.012 | 7.20E-06 | 20.531 |
| order | Rhodospirillales | rs7098347 | A | G | 0.071 | 0.016 | 5.35E-06 | 20.526 |
| order | Bacillales | rs77552222 | A | G | -0.160 | 0.035 | 5.76E-06 | 20.524 |
| order | Desulfovibrionales | rs35236973 | T | A | -0.053 | 0.012 | 6.98E-06 | 20.522 |
| order | MollicutesRF9 | rs9885169 | A | G | 0.064 | 0.014 | 5.94E-06 | 20.521 |
| order | Bifidobacteriales | rs861392 | T | C | 0.055 | 0.012 | 5.21E-06 | 20.520 |
| order | Bifidobacteriales | rs842356 | A | G | 0.074 | 0.016 | 5.09E-06 | 20.519 |
| order | Erysipelotrichales | rs12258727 | T | C | -0.052 | 0.011 | 6.86E-06 | 20.518 |
| order | Bifidobacteriales | rs1992764 | C | T | 0.054 | 0.012 | 5.09E-06 | 20.515 |
| order | Lactobacillales | rs3817331 | G | A | 0.050 | 0.011 | 6.18E-06 | 20.515 |
| order | Desulfovibrionales | rs55744759 | G | A | -0.077 | 0.017 | 9.02E-06 | 20.514 |
| order | Victivallales | rs72640280 | G | A | 0.220 | 0.049 | 5.18E-06 | 20.513 |
| order | Actinomycetales | rs2110088 | A | T | 0.079 | 0.017 | 6.19E-06 | 20.513 |
| order | Enterobacteriales | rs61973590 | G | C | -0.061 | 0.013 | 8.54E-06 | 20.504 |
| order | Bacillales | rs2174182 | A | C | -0.159 | 0.035 | 6.92E-06 | 20.495 |
| order | Lactobacillales | rs9445784 | C | T | -0.099 | 0.022 | 5.20E-06 | 20.495 |
| order | Bacteroidales | rs13097740 | G | A | -0.060 | 0.013 | 4.05E-06 | 20.493 |
| order | Bifidobacteriales | rs80261410 | T | C | 0.062 | 0.014 | 8.48E-06 | 20.492 |
| order | Bacillales | rs75138708 | A | C | -0.161 | 0.035 | 6.63E-06 | 20.491 |
| order | Victivallales | rs2031282 | G | A | 0.122 | 0.027 | 4.38E-06 | 20.491 |
| order | Gastranaerophilales | rs367480 | A | G | -0.084 | 0.019 | 7.52E-06 | 20.487 |
| order | Rhodospirillales | rs11892008 | C | T | 0.113 | 0.025 | 6.31E-06 | 20.487 |
| order | Bacillales | rs75536913 | T | C | -0.161 | 0.036 | 5.60E-06 | 20.486 |
| order | Erysipelotrichales | rs921306 | C | T | 0.049 | 0.011 | 7.20E-06 | 20.481 |
| order | Actinomycetales | rs61937253 | C | T | 0.079 | 0.018 | 5.52E-06 | 20.480 |
| order | Burkholderiales | rs1410861 | A | G | 0.050 | 0.011 | 6.20E-06 | 20.476 |
| order | Bifidobacteriales | rs2090660 | C | T | 0.062 | 0.014 | 7.78E-06 | 20.475 |
| order | Enterobacteriales | rs62210022 | C | T | 0.070 | 0.016 | 5.30E-06 | 20.472 |
| order | Victivallales | rs2546105 | T | A | 0.106 | 0.023 | 6.53E-06 | 20.472 |
| order | Gastranaerophilales | rs1221147 | A | T | 0.126 | 0.028 | 4.86E-06 | 20.471 |
| order | Erysipelotrichales | rs1074800 | G | A | 0.049 | 0.011 | 6.15E-06 | 20.459 |
| order | Burkholderiales | rs9537886 | C | A | -0.050 | 0.011 | 5.83E-06 | 20.457 |
| order | Bifidobacteriales | rs73797465 | G | T | -0.094 | 0.021 | 4.85E-06 | 20.454 |
| order | Bacillales | rs17205524 | A | C | -0.160 | 0.035 | 6.79E-06 | 20.453 |
| order | Burkholderiales | rs10047202 | C | T | 0.050 | 0.011 | 6.27E-06 | 20.451 |
| order | Bifidobacteriales | rs75524146 | C | T | 0.074 | 0.016 | 7.23E-06 | 20.451 |
| order | NB1n | rs8126061 | C | T | -0.159 | 0.035 | 7.36E-06 | 20.450 |
| order | Gastranaerophilales | rs113884518 | C | T | -0.206 | 0.046 | 7.74E-06 | 20.446 |
| order | Verrucomicrobiales | rs2199602 | G | T | 0.066 | 0.015 | 7.12E-06 | 20.438 |
| order | Burkholderiales | rs7326330 | T | C | -0.050 | 0.011 | 5.76E-06 | 20.437 |
| order | Desulfovibrionales | rs72647048 | C | T | -0.077 | 0.017 | 9.00E-06 | 20.436 |
| order | Gastranaerophilales | rs28678345 | C | T | 0.213 | 0.047 | 8.06E-06 | 20.434 |
| order | Verrucomicrobiales | rs61779207 | A | G | -0.076 | 0.017 | 6.72E-06 | 20.432 |
| order | MollicutesRF9 | rs116086762 | T | C | -0.175 | 0.039 | 6.07E-06 | 20.426 |
| order | Bifidobacteriales | rs11688414 | A | G | 0.054 | 0.012 | 6.08E-06 | 20.423 |
| order | Burkholderiales | rs11128180 | G | A | 0.058 | 0.013 | 5.53E-06 | 20.421 |
| order | Erysipelotrichales | rs9852883 | C | A | 0.048 | 0.011 | 6.33E-06 | 20.416 |
| order | Lactobacillales | rs17151854 | G | T | -0.072 | 0.016 | 3.09E-06 | 20.415 |
| order | Coriobacteriales | rs4880783 | T | G | 0.057 | 0.013 | 5.90E-06 | 20.414 |
| order | Lactobacillales | rs78938557 | C | T | 0.106 | 0.023 | 2.31E-06 | 20.412 |
| order | Bifidobacteriales | rs12188321 | C | G | 0.053 | 0.012 | 6.41E-06 | 20.410 |
| order | Bifidobacteriales | rs16832624 | T | C | 0.061 | 0.014 | 7.07E-06 | 20.409 |
| order | Bacteroidales | rs7857688 | G | A | 0.064 | 0.014 | 5.79E-06 | 20.408 |
| order | Methanobacteriales | rs62240389 | A | G | -0.188 | 0.042 | 7.64E-06 | 20.402 |
| order | Verrucomicrobiales | rs4497215 | C | T | 0.064 | 0.014 | 6.55E-06 | 20.401 |
| order | Bifidobacteriales | rs4072434 | G | C | 0.055 | 0.012 | 8.36E-06 | 20.394 |
| order | Verrucomicrobiales | rs72663756 | C | T | 0.066 | 0.015 | 5.39E-06 | 20.394 |
| order | Selenomonadales | rs34058443 | T | C | 0.060 | 0.013 | 6.37E-06 | 20.381 |
| order | Methanobacteriales | rs74599069 | C | A | 0.221 | 0.049 | 8.22E-06 | 20.378 |
| order | Bacteroidales | rs929878 | T | C | -0.055 | 0.012 | 4.73E-06 | 20.372 |
| order | Lactobacillales | rs2559847 | C | T | -0.099 | 0.022 | 8.75E-06 | 20.372 |
| order | Bifidobacteriales | rs6899771 | G | A | -0.091 | 0.020 | 7.28E-06 | 20.365 |
| order | Bacillales | rs76951419 | A | G | -0.159 | 0.035 | 6.27E-06 | 20.361 |
| order | Bacillales | rs75538955 | C | T | -0.159 | 0.035 | 6.27E-06 | 20.361 |
| order | Bacillales | rs111336356 | G | A | -0.160 | 0.035 | 6.15E-06 | 20.356 |
| order | Bacillales | rs113170277 | G | T | -0.160 | 0.035 | 6.15E-06 | 20.356 |
| order | Bifidobacteriales | rs11655079 | C | T | -0.059 | 0.013 | 5.92E-06 | 20.345 |
| order | Verrucomicrobiales | rs72663785 | C | G | 0.066 | 0.015 | 7.41E-06 | 20.338 |
| order | Bifidobacteriales | rs79176913 | C | A | 0.072 | 0.016 | 8.15E-06 | 20.335 |
| order | Lactobacillales | rs113343986 | G | A | 0.050 | 0.011 | 7.52E-06 | 20.334 |
| order | Bifidobacteriales | rs503227 | T | C | 0.072 | 0.016 | 6.71E-06 | 20.332 |
| order | Bifidobacteriales | rs4988201 | A | G | 0.072 | 0.016 | 7.71E-06 | 20.329 |
| order | Coriobacteriales | rs8010111 | A | G | -0.103 | 0.023 | 6.90E-06 | 20.328 |
| order | Verrucomicrobiales | rs12045978 | G | A | 0.064 | 0.014 | 6.87E-06 | 20.326 |
| order | Coriobacteriales | rs76779974 | G | C | 0.077 | 0.017 | 5.63E-06 | 20.326 |
| order | Bacillales | rs10404781 | G | A | 0.113 | 0.025 | 8.28E-06 | 20.324 |
| order | Burkholderiales | rs9537885 | G | A | -0.049 | 0.011 | 6.24E-06 | 20.319 |
| order | Methanobacteriales | rs4257531 | A | G | 0.164 | 0.036 | 7.44E-06 | 20.316 |
| order | Bacteroidales | rs72706335 | C | T | -0.222 | 0.049 | 7.66E-06 | 20.315 |
| order | Lactobacillales | rs7764486 | A | G | -0.098 | 0.022 | 7.01E-06 | 20.314 |
| order | Bacteroidales | rs7157380 | G | C | 0.096 | 0.021 | 4.63E-06 | 20.312 |
| order | Burkholderiales | rs9537916 | G | A | -0.050 | 0.011 | 6.01E-06 | 20.312 |
| order | Verrucomicrobiales | rs7693865 | C | T | 0.107 | 0.024 | 6.08E-06 | 20.307 |
| order | Bifidobacteriales | rs12465599 | G | A | 0.053 | 0.012 | 7.13E-06 | 20.303 |
| order | Rhodospirillales | rs72714475 | G | C | 0.116 | 0.026 | 6.34E-06 | 20.302 |
| order | Bacillales | rs1910440 | A | G | -0.159 | 0.035 | 7.74E-06 | 20.300 |
| order | Burkholderiales | rs6686895 | A | G | 0.050 | 0.011 | 6.82E-06 | 20.299 |
| order | NB1n | rs166849 | A | G | 0.091 | 0.020 | 7.74E-06 | 20.293 |
| order | Bifidobacteriales | rs7581814 | C | G | 0.071 | 0.016 | 8.01E-06 | 20.291 |
| order | Burkholderiales | rs9537883 | G | T | -0.049 | 0.011 | 6.25E-06 | 20.290 |
| order | Lactobacillales | rs2952172 | T | C | -0.055 | 0.012 | 6.34E-06 | 20.289 |
| order | Pasteurellales | rs40513 | T | G | 0.118 | 0.026 | 7.08E-06 | 20.288 |
| order | Rhodospirillales | rs4918686 | T | G | 0.070 | 0.016 | 7.43E-06 | 20.281 |
| order | Burkholderiales | rs11124193 | A | G | 0.061 | 0.013 | 6.67E-06 | 20.280 |
| order | Bacillales | rs12508795 | C | T | -0.160 | 0.036 | 6.10E-06 | 20.273 |
| order | Coriobacteriales | rs2442778 | A | G | -0.116 | 0.026 | 9.03E-06 | 20.272 |
| order | Burkholderiales | rs2040996 | C | T | -0.049 | 0.011 | 6.21E-06 | 20.265 |
| order | Bacteroidales | rs62575403 | T | C | 0.140 | 0.031 | 7.06E-06 | 20.264 |
| order | Bifidobacteriales | rs621341 | C | T | -0.055 | 0.012 | 3.67E-06 | 20.263 |
| order | NB1n | rs7072640 | G | C | -0.106 | 0.024 | 7.80E-06 | 20.257 |
| order | Rhodospirillales | rs4278423 | C | T | 0.105 | 0.023 | 3.98E-06 | 20.256 |
| order | NB1n | rs60775321 | C | T | -0.096 | 0.021 | 7.10E-06 | 20.256 |
| order | Bifidobacteriales | rs7561565 | T | G | 0.071 | 0.016 | 8.42E-06 | 20.254 |
| order | Bacillales | rs74985627 | G | A | -0.160 | 0.036 | 6.43E-06 | 20.251 |
| order | Selenomonadales | rs1649999 | G | A | 0.075 | 0.017 | 7.58E-06 | 20.246 |
| order | Burkholderiales | rs1926544 | A | C | -0.049 | 0.011 | 6.40E-06 | 20.246 |
| order | Clostridiales | rs6934062 | C | G | 0.052 | 0.012 | 7.09E-06 | 20.240 |
| order | Victivallales | rs1002941 | A | G | 0.105 | 0.023 | 8.15E-06 | 20.233 |
| order | Bacillales | rs74492413 | G | C | -0.160 | 0.036 | 6.44E-06 | 20.228 |
| order | Bifidobacteriales | rs6750549 | A | G | 0.072 | 0.016 | 8.10E-06 | 20.226 |
| order | NB1n | rs8126056 | C | T | -0.158 | 0.035 | 8.32E-06 | 20.226 |
| order | Pasteurellales | rs77529231 | T | C | -0.224 | 0.050 | 8.54E-06 | 20.218 |
| order | Bifidobacteriales | rs4245838 | C | T | 0.105 | 0.023 | 4.34E-06 | 20.214 |
| order | Methanobacteriales | rs10424197 | A | G | -0.111 | 0.025 | 9.28E-06 | 20.211 |
| order | Rhodospirillales | rs72714480 | C | T | 0.115 | 0.026 | 5.52E-06 | 20.210 |
| order | Pasteurellales | rs9938097 | C | T | -0.071 | 0.016 | 8.23E-06 | 20.209 |
| order | Bifidobacteriales | rs10180515 | T | A | 0.063 | 0.014 | 4.10E-06 | 20.209 |
| order | Methanobacteriales | rs73068003 | T | G | -0.158 | 0.035 | 8.45E-06 | 20.206 |
| order | MollicutesRF9 | rs638542 | A | G | -0.071 | 0.016 | 5.17E-06 | 20.204 |
| order | Bacillales | rs75373115 | G | A | -0.159 | 0.035 | 7.12E-06 | 20.199 |
| order | Victivallales | rs6951588 | T | C | 0.104 | 0.023 | 7.63E-06 | 20.199 |
| order | Lactobacillales | rs12797734 | C | T | 0.057 | 0.013 | 7.77E-06 | 20.198 |
| order | Bifidobacteriales | rs309150 | T | A | 0.071 | 0.016 | 8.14E-06 | 20.192 |
| order | Bacillales | rs76884318 | C | G | -0.159 | 0.035 | 6.53E-06 | 20.192 |
| order | Selenomonadales | rs2834062 | G | A | 0.049 | 0.011 | 8.44E-06 | 20.190 |
| order | Bacteroidales | rs11146701 | G | A | 0.047 | 0.011 | 7.08E-06 | 20.186 |
| order | Bifidobacteriales | rs11684458 | A | G | 0.063 | 0.014 | 3.84E-06 | 20.179 |
| order | NB1n | rs59824299 | A | T | -0.107 | 0.024 | 8.10E-06 | 20.178 |
| order | Rhodospirillales | rs72714481 | A | G | 0.115 | 0.026 | 5.65E-06 | 20.172 |
| order | NB1n | rs56328886 | A | T | 0.166 | 0.037 | 7.78E-06 | 20.166 |
| order | Bifidobacteriales | rs9800013 | A | G | 0.065 | 0.015 | 4.84E-06 | 20.164 |
| order | Bifidobacteriales | rs6880820 | T | C | 0.064 | 0.014 | 4.07E-06 | 20.160 |
| order | Rhodospirillales | rs77304857 | A | C | -0.100 | 0.022 | 6.02E-06 | 20.156 |
| order | Burkholderiales | rs4521068 | G | A | 0.060 | 0.013 | 7.03E-06 | 20.156 |
| order | MollicutesRF9 | rs10071529 | C | G | 0.125 | 0.028 | 8.64E-06 | 20.154 |
| order | Methanobacteriales | rs41288287 | C | T | 0.199 | 0.044 | 8.70E-06 | 20.154 |
| order | Actinomycetales | rs34951409 | C | G | 0.078 | 0.017 | 7.37E-06 | 20.149 |
| order | Bacillales | rs7655747 | A | G | -0.159 | 0.035 | 6.68E-06 | 20.149 |
| order | Bifidobacteriales | rs309162 | T | A | 0.071 | 0.016 | 8.46E-06 | 20.146 |
| order | Burkholderiales | rs35945722 | G | A | 0.062 | 0.014 | 5.72E-06 | 20.146 |
| order | MollicutesRF9 | rs11779863 | A | G | -0.077 | 0.017 | 6.69E-06 | 20.146 |
| order | Verrucomicrobiales | rs12026127 | G | A | 0.064 | 0.014 | 7.05E-06 | 20.144 |
| order | Actinomycetales | rs7958229 | C | T | 0.078 | 0.017 | 6.87E-06 | 20.141 |
| order | Lactobacillales | rs11720390 | A | G | 0.100 | 0.022 | 9.73E-06 | 20.137 |
| order | Bifidobacteriales | rs2176716 | C | T | 0.063 | 0.014 | 4.01E-06 | 20.135 |
| order | Actinomycetales | rs58484246 | C | T | 0.076 | 0.017 | 6.67E-06 | 20.133 |
| order | Bacillales | rs75699854 | T | C | -0.158 | 0.035 | 6.88E-06 | 20.132 |
| order | Bifidobacteriales | rs309172 | A | G | 0.070 | 0.016 | 6.64E-06 | 20.129 |
| order | Rhodospirillales | rs6470867 | T | C | 0.117 | 0.026 | 5.88E-06 | 20.126 |
| order | Enterobacteriales | rs4792380 | T | A | 0.116 | 0.026 | 9.49E-06 | 20.125 |
| order | Bacillales | rs75768129 | A | C | -0.158 | 0.035 | 7.27E-06 | 20.123 |
| order | Bacillales | rs78915064 | T | A | -0.158 | 0.035 | 7.27E-06 | 20.123 |
| order | Bifidobacteriales | rs68005421 | G | C | 0.055 | 0.012 | 9.22E-06 | 20.120 |
| order | Erysipelotrichales | rs12938302 | C | T | -0.075 | 0.017 | 9.17E-06 | 20.119 |
| order | NB1n | rs72669400 | T | C | 0.166 | 0.037 | 7.90E-06 | 20.114 |
| order | Erysipelotrichales | rs35587059 | C | T | 0.048 | 0.011 | 7.13E-06 | 20.114 |
| order | Bacteroidales | rs73846124 | G | A | -0.060 | 0.013 | 5.11E-06 | 20.110 |
| order | Bacteroidales | rs71322865 | C | T | -0.059 | 0.013 | 4.90E-06 | 20.108 |
| order | Erysipelotrichales | rs41300431 | G | A | 0.048 | 0.011 | 7.12E-06 | 20.106 |
| order | Lactobacillales | rs111552159 | G | C | 0.129 | 0.029 | 8.31E-06 | 20.102 |
| order | Actinomycetales | rs12230967 | A | G | 0.078 | 0.017 | 7.54E-06 | 20.095 |
| order | Bifidobacteriales | rs3099429 | T | A | 0.071 | 0.016 | 7.65E-06 | 20.086 |
| order | Pasteurellales | rs6100166 | G | C | 0.067 | 0.015 | 8.38E-06 | 20.086 |
| order | Selenomonadales | rs58649672 | C | G | 0.047 | 0.011 | 7.78E-06 | 20.084 |
| order | Rhodospirillales | rs11599418 | T | C | 0.071 | 0.016 | 6.91E-06 | 20.081 |
| order | Burkholderiales | rs1036449 | C | T | -0.049 | 0.011 | 6.86E-06 | 20.077 |
| order | Bacillales | rs79143961 | C | A | -0.159 | 0.035 | 7.71E-06 | 20.075 |
| order | Coriobacteriales | rs13307134 | T | C | 0.057 | 0.013 | 7.80E-06 | 20.072 |
| order | Bacteroidales | rs34986288 | G | A | -0.061 | 0.014 | 4.69E-06 | 20.069 |
| order | Bifidobacteriales | rs61253125 | T | C | 0.071 | 0.016 | 8.75E-06 | 20.069 |
| order | Bifidobacteriales | rs309154 | C | T | 0.072 | 0.016 | 9.29E-06 | 20.066 |
| order | MollicutesRF9 | rs417270 | C | T | -0.070 | 0.016 | 5.81E-06 | 20.062 |
| order | Clostridiales | rs7964593 | T | C | 0.051 | 0.011 | 6.42E-06 | 20.062 |
| order | NB1n | rs80270230 | C | G | 0.176 | 0.039 | 8.41E-06 | 20.060 |
| order | Coriobacteriales | rs7977399 | C | T | -0.058 | 0.013 | 6.07E-06 | 20.058 |
| order | Bacillales | rs1871738 | G | T | -0.166 | 0.037 | 7.58E-06 | 20.056 |
| order | Bacillales | rs28838109 | G | T | -0.166 | 0.037 | 7.20E-06 | 20.056 |
| order | MollicutesRF9 | rs13100746 | T | C | 0.064 | 0.014 | 7.29E-06 | 20.055 |
| order | Bacteroidales | rs2418267 | G | A | 0.061 | 0.014 | 7.16E-06 | 20.054 |
| order | Rhodospirillales | rs3768869 | A | G | 0.089 | 0.020 | 9.12E-06 | 20.052 |
| order | Rhodospirillales | rs3730086 | G | A | 0.080 | 0.018 | 7.98E-06 | 20.051 |
| order | Clostridiales | rs76767978 | A | G | 0.070 | 0.016 | 8.18E-06 | 20.042 |
| order | MollicutesRF9 | rs443345 | G | A | -0.070 | 0.016 | 5.89E-06 | 20.040 |
| order | Burkholderiales | rs9537941 | A | C | -0.049 | 0.011 | 6.81E-06 | 20.035 |
| order | Bacillales | rs10425560 | T | A | 0.112 | 0.025 | 9.60E-06 | 20.029 |
| order | Burkholderiales | rs2528680 | C | T | -0.049 | 0.011 | 6.59E-06 | 20.025 |
| order | Bifidobacteriales | rs10221893 | T | C | 0.054 | 0.012 | 9.68E-06 | 20.020 |
| order | MollicutesRF9 | rs76373661 | A | G | 0.091 | 0.020 | 5.16E-06 | 20.019 |
| order | Rhodospirillales | rs72714479 | G | A | 0.115 | 0.026 | 5.74E-06 | 20.013 |
| order | Rhodospirillales | rs3754623 | G | T | 0.089 | 0.020 | 9.31E-06 | 20.012 |
| order | Bacillales | rs11844714 | G | A | -0.143 | 0.032 | 5.06E-06 | 20.004 |
| order | Rhodospirillales | rs10181850 | A | C | 0.089 | 0.020 | 9.55E-06 | 19.986 |
| order | Methanobacteriales | rs9532770 | T | C | 0.124 | 0.028 | 9.34E-06 | 19.985 |
| order | MollicutesRF9 | rs7801843 | G | A | -0.087 | 0.019 | 9.47E-06 | 19.983 |
| order | Burkholderiales | rs9527747 | C | G | -0.049 | 0.011 | 7.23E-06 | 19.979 |
| order | Verrucomicrobiales | rs7538356 | A | C | 0.064 | 0.014 | 8.12E-06 | 19.978 |
| order | Erysipelotrichales | rs7906464 | T | A | -0.053 | 0.012 | 7.35E-06 | 19.973 |
| order | Actinomycetales | rs59800030 | C | T | 0.076 | 0.017 | 7.31E-06 | 19.970 |
| order | Enterobacteriales | rs6069866 | G | C | 0.058 | 0.013 | 7.83E-06 | 19.968 |
| order | Bifidobacteriales | rs4988163 | G | T | 0.071 | 0.016 | 9.50E-06 | 19.964 |
| order | Bifidobacteriales | rs4954274 | G | A | 0.089 | 0.020 | 9.60E-06 | 19.963 |
| order | Bacillales | rs79653240 | T | A | -0.158 | 0.035 | 7.58E-06 | 19.958 |
| order | NB1n | rs72671309 | T | C | 0.177 | 0.040 | 7.49E-06 | 19.958 |
| order | NB1n | rs267955 | A | G | 0.097 | 0.022 | 9.20E-06 | 19.952 |
| order | Rhodospirillales | rs80346366 | C | T | -0.099 | 0.022 | 6.58E-06 | 19.946 |
| order | MollicutesRF9 | rs367586 | A | C | -0.070 | 0.016 | 6.22E-06 | 19.945 |
| order | Erysipelotrichales | rs290833 | G | T | -0.050 | 0.011 | 8.03E-06 | 19.942 |
| order | Lactobacillales | rs12371930 | G | A | -0.104 | 0.023 | 9.62E-06 | 19.942 |
| order | MollicutesRF9 | rs7853673 | A | G | -0.062 | 0.014 | 6.73E-06 | 19.939 |
| order | Desulfovibrionales | rs186073 | C | T | 0.053 | 0.012 | 8.74E-06 | 19.931 |
| order | Actinomycetales | rs11055873 | A | G | 0.078 | 0.017 | 7.93E-06 | 19.930 |
| order | Erysipelotrichales | rs8096144 | C | G | -0.086 | 0.019 | 9.53E-06 | 19.930 |
| order | Rhodospirillales | rs34142542 | C | T | 0.071 | 0.016 | 7.61E-06 | 19.926 |
| order | Actinomycetales | rs56236273 | T | A | 0.075 | 0.017 | 7.40E-06 | 19.922 |
| order | Rhodospirillales | rs55947871 | G | A | 0.115 | 0.026 | 7.29E-06 | 19.920 |
| order | Clostridiales | rs13105690 | C | T | -0.053 | 0.012 | 9.37E-06 | 19.919 |
| order | Rhodospirillales | rs71498364 | C | T | 0.071 | 0.016 | 7.47E-06 | 19.912 |
| order | Coriobacteriales | rs11073596 | G | T | 0.051 | 0.011 | 8.14E-06 | 19.912 |
| order | Burkholderiales | rs56104821 | C | A | 0.057 | 0.013 | 8.72E-06 | 19.908 |
| order | Verrucomicrobiales | rs4595371 | A | G | 0.063 | 0.014 | 8.42E-06 | 19.894 |
| order | Victivallales | rs1895249 | G | T | 0.105 | 0.023 | 8.27E-06 | 19.893 |
| order | MollicutesRF9 | rs949341 | A | G | 0.066 | 0.015 | 7.73E-06 | 19.893 |
| order | Coriobacteriales | rs62448869 | A | T | -0.049 | 0.011 | 7.87E-06 | 19.891 |
| order | Rhodospirillales | rs57340984 | C | T | 0.111 | 0.025 | 7.87E-06 | 19.887 |
| order | Verrucomicrobiales | rs79837054 | T | C | 0.105 | 0.024 | 7.58E-06 | 19.879 |
| order | Pasteurellales | rs6972479 | G | A | -0.078 | 0.018 | 7.75E-06 | 19.878 |
| order | Burkholderiales | rs6579313 | G | A | -0.090 | 0.020 | 7.99E-06 | 19.877 |
| order | Coriobacteriales | rs12974142 | A | G | 0.079 | 0.018 | 8.51E-06 | 19.865 |
| order | Rhodospirillales | rs17793087 | T | C | 0.070 | 0.016 | 7.77E-06 | 19.857 |
| order | Methanobacteriales | rs6508769 | C | T | 0.154 | 0.034 | 8.23E-06 | 19.856 |
| order | NB1n | rs72671372 | G | A | 0.165 | 0.037 | 9.01E-06 | 19.856 |
| order | Coriobacteriales | rs7898585 | C | T | 0.073 | 0.016 | 5.25E-06 | 19.850 |
| order | Victivallales | rs1363428 | A | G | 0.104 | 0.023 | 9.33E-06 | 19.850 |
| order | Burkholderiales | rs13385989 | A | C | 0.060 | 0.014 | 8.40E-06 | 19.844 |
| order | Bifidobacteriales | rs4954556 | G | A | 0.114 | 0.026 | 4.45E-06 | 19.840 |
| order | Clostridiales | rs11082866 | A | T | -0.051 | 0.012 | 7.89E-06 | 19.840 |
| order | Rhodospirillales | rs12518525 | A | G | -0.110 | 0.025 | 8.09E-06 | 19.831 |
| order | Burkholderiales | rs9537922 | T | C | -0.049 | 0.011 | 7.80E-06 | 19.831 |
| order | Erysipelotrichales | rs28703642 | G | T | 0.053 | 0.012 | 7.64E-06 | 19.830 |
| order | Bifidobacteriales | rs13387501 | C | T | 0.062 | 0.014 | 4.78E-06 | 19.827 |
| order | Burkholderiales | rs1122678 | T | C | -0.049 | 0.011 | 7.41E-06 | 19.819 |
| order | Rhodospirillales | rs80344265 | C | T | -0.099 | 0.022 | 7.03E-06 | 19.818 |
| order | NB1n | rs61833604 | G | A | -0.168 | 0.038 | 8.10E-06 | 19.818 |
| order | NB1n | rs267949 | C | T | 0.096 | 0.022 | 9.55E-06 | 19.817 |
| order | Bifidobacteriales | rs55888705 | G | A | 0.054 | 0.012 | 8.66E-06 | 19.811 |
| order | Verrucomicrobiales | rs4294419 | G | A | 0.063 | 0.014 | 8.19E-06 | 19.808 |
| order | Coriobacteriales | rs45480394 | G | T | -0.050 | 0.011 | 9.71E-06 | 19.806 |
| order | Rhodospirillales | rs11818728 | C | T | 0.070 | 0.016 | 7.84E-06 | 19.803 |
| order | Lactobacillales | rs2370083 | T | G | -0.081 | 0.018 | 8.33E-06 | 19.802 |
| order | Methanobacteriales | rs11018665 | T | A | 0.111 | 0.025 | 6.52E-06 | 19.797 |
| order | Burkholderiales | rs2528681 | G | C | -0.049 | 0.011 | 7.47E-06 | 19.797 |
| order | Bacteroidales | rs1505366 | G | T | -0.107 | 0.024 | 8.89E-06 | 19.796 |
| order | Bifidobacteriales | rs10432371 | G | A | 0.053 | 0.012 | 6.02E-06 | 19.791 |
| order | Verrucomicrobiales | rs12024893 | A | G | 0.063 | 0.014 | 8.96E-06 | 19.784 |
| order | Burkholderiales | rs9537865 | C | T | -0.049 | 0.011 | 8.23E-06 | 19.782 |
| order | Bacillales | rs1871737 | C | A | -0.164 | 0.037 | 8.74E-06 | 19.777 |
| order | Bifidobacteriales | rs10857328 | A | T | 0.056 | 0.013 | 7.24E-06 | 19.770 |
| order | Coriobacteriales | rs7303392 | C | T | -0.057 | 0.013 | 7.32E-06 | 19.770 |
| order | MollicutesRF9 | rs3853498 | C | T | -0.070 | 0.016 | 6.72E-06 | 19.762 |
| order | Selenomonadales | rs1135612 | A | G | 0.053 | 0.012 | 9.26E-06 | 19.761 |
| order | Erysipelotrichales | rs1884466 | T | C | -0.048 | 0.011 | 9.53E-06 | 19.760 |
| order | Selenomonadales | rs12572508 | C | T | 0.047 | 0.011 | 9.58E-06 | 19.757 |
| order | Verrucomicrobiales | rs36010840 | C | T | 0.063 | 0.014 | 9.11E-06 | 19.754 |
| order | Pasteurellales | rs111582866 | A | G | -0.114 | 0.026 | 7.07E-06 | 19.753 |
| order | Bacillales | rs79431317 | T | G | -0.158 | 0.036 | 8.86E-06 | 19.750 |
| order | Burkholderiales | rs9537904 | G | A | -0.049 | 0.011 | 8.17E-06 | 19.740 |
| order | Burkholderiales | rs9537940 | C | A | -0.049 | 0.011 | 7.98E-06 | 19.737 |
| order | Burkholderiales | rs74840119 | G | C | -0.091 | 0.020 | 8.85E-06 | 19.737 |
| order | Rhodospirillales | rs7623329 | C | A | -0.071 | 0.016 | 8.07E-06 | 19.732 |
| order | Rhodospirillales | rs76208018 | A | G | 0.114 | 0.026 | 8.34E-06 | 19.731 |
| order | Burkholderiales | rs9537872 | A | G | -0.049 | 0.011 | 8.24E-06 | 19.730 |
| order | Actinomycetales | rs35110659 | G | T | 0.076 | 0.017 | 9.69E-06 | 19.730 |
| order | Enterobacteriales | rs504442 | G | T | 0.084 | 0.019 | 5.17E-06 | 19.728 |
| order | Verrucomicrobiales | rs72663769 | T | C | 0.065 | 0.015 | 7.43E-06 | 19.727 |
| order | Victivallales | rs62481809 | C | G | 0.103 | 0.023 | 9.20E-06 | 19.726 |
| order | Gastranaerophilales | rs789069 | C | A | -0.104 | 0.023 | 6.50E-06 | 19.725 |
| order | Burkholderiales | rs11124197 | T | C | 0.060 | 0.013 | 9.17E-06 | 19.724 |
| order | Bifidobacteriales | rs606101 | G | A | -0.054 | 0.012 | 7.54E-06 | 19.721 |
| order | Erysipelotrichales | rs7100916 | T | G | -0.053 | 0.012 | 7.62E-06 | 19.721 |
| order | Clostridiales | rs6536526 | T | A | 0.066 | 0.015 | 8.44E-06 | 19.717 |
| order | Rhodospirillales | rs11944431 | G | A | -0.099 | 0.022 | 7.17E-06 | 19.717 |
| order | NB1n | rs72671349 | C | T | 0.165 | 0.037 | 9.92E-06 | 19.714 |
| order | Rhodospirillales | rs55840599 | C | T | 0.114 | 0.026 | 8.20E-06 | 19.714 |
| order | Burkholderiales | rs1036450 | T | A | -0.049 | 0.011 | 8.27E-06 | 19.712 |
| order | Verrucomicrobiales | rs72663802 | C | T | 0.065 | 0.015 | 8.44E-06 | 19.707 |
| order | Burkholderiales | rs9537908 | T | C | -0.049 | 0.011 | 8.34E-06 | 19.703 |
| order | Lactobacillales | rs28450391 | A | G | 0.060 | 0.013 | 9.80E-06 | 19.702 |
| order | Lactobacillales | rs13379080 | T | C | -0.080 | 0.018 | 8.94E-06 | 19.698 |
| order | Bacillales | rs6587921 | C | T | -0.141 | 0.032 | 8.42E-06 | 19.689 |
| order | Burkholderiales | rs9569779 | A | G | -0.049 | 0.011 | 8.63E-06 | 19.686 |
| order | NB1n | rs6741388 | C | T | 0.089 | 0.020 | 9.36E-06 | 19.682 |
| order | Burkholderiales | rs2528678 | C | T | -0.049 | 0.011 | 7.90E-06 | 19.682 |
| order | Coriobacteriales | rs1397793 | A | G | -0.050 | 0.011 | 9.77E-06 | 19.681 |
| order | Burkholderiales | rs9537921 | A | G | -0.049 | 0.011 | 8.41E-06 | 19.676 |
| order | Bacillales | rs6673795 | G | A | -0.140 | 0.032 | 9.03E-06 | 19.675 |
| order | Burkholderiales | rs9527748 | G | A | -0.049 | 0.011 | 8.44E-06 | 19.675 |
| order | Verrucomicrobiales | rs4107636 | G | C | 0.063 | 0.014 | 7.95E-06 | 19.674 |
| order | Actinomycetales | rs12831981 | T | G | 0.077 | 0.017 | 8.93E-06 | 19.672 |
| order | Bifidobacteriales | rs6711997 | T | C | 0.062 | 0.014 | 5.10E-06 | 19.666 |
| order | Verrucomicrobiales | rs4600885 | A | C | 0.063 | 0.014 | 7.86E-06 | 19.661 |
| order | Erysipelotrichales | rs61865709 | G | A | -0.053 | 0.012 | 8.00E-06 | 19.660 |
| order | Verrucomicrobiales | rs12411321 | T | C | 0.074 | 0.017 | 7.24E-06 | 19.656 |
| order | Enterobacteriales | rs35673018 | A | G | 0.090 | 0.020 | 7.63E-06 | 19.653 |
| order | Methanobacteriales | rs4753222 | G | A | 0.111 | 0.025 | 7.45E-06 | 19.651 |
| order | Burkholderiales | rs9569804 | G | A | -0.049 | 0.011 | 8.60E-06 | 19.647 |
| order | Bifidobacteriales | rs10928519 | C | T | 0.053 | 0.012 | 6.26E-06 | 19.645 |
| order | Actinomycetales | rs11055874 | C | T | 0.077 | 0.017 | 9.23E-06 | 19.643 |
| order | Bacteroidales | rs4916508 | A | G | -0.047 | 0.011 | 8.47E-06 | 19.641 |
| order | Rhodospirillales | rs55876211 | T | C | -0.087 | 0.020 | 7.87E-06 | 19.638 |
| order | Clostridiales | rs6536525 | G | C | 0.066 | 0.015 | 8.79E-06 | 19.634 |
| order | Bacillales | rs10025761 | C | T | -0.164 | 0.037 | 9.04E-06 | 19.633 |
| order | Burkholderiales | rs1470456 | A | G | -0.049 | 0.011 | 8.48E-06 | 19.633 |
| order | Desulfovibrionales | rs62020470 | G | A | -0.057 | 0.013 | 7.51E-06 | 19.633 |
| order | Coriobacteriales | rs7540303 | T | C | 0.048 | 0.011 | 9.62E-06 | 19.632 |
| order | Coriobacteriales | rs60426889 | G | T | -0.050 | 0.011 | 8.69E-06 | 19.632 |
| order | Burkholderiales | rs9537900 | A | G | -0.049 | 0.011 | 8.90E-06 | 19.625 |
| order | Selenomonadales | rs2103109 | A | C | 0.047 | 0.011 | 9.81E-06 | 19.622 |
| order | Verrucomicrobiales | rs736124 | T | A | -0.074 | 0.017 | 8.63E-06 | 19.620 |
| order | MollicutesRF9 | rs4970840 | G | A | -0.070 | 0.016 | 7.80E-06 | 19.616 |
| order | Rhodospirillales | rs12522947 | C | G | -0.109 | 0.025 | 9.58E-06 | 19.616 |
| order | Enterobacteriales | rs79757635 | A | C | 0.076 | 0.017 | 9.32E-06 | 19.615 |
| order | MollicutesRF9 | rs12711448 | T | C | -0.069 | 0.016 | 7.43E-06 | 19.612 |
| order | Actinomycetales | rs7133400 | C | G | 0.077 | 0.017 | 9.21E-06 | 19.610 |
| order | Rhodospirillales | rs35982688 | T | C | 0.070 | 0.016 | 8.37E-06 | 19.604 |
| order | Gastranaerophilales | rs8028558 | G | A | 0.083 | 0.019 | 9.78E-06 | 19.603 |
| order | Rhodospirillales | rs13336560 | T | C | -0.070 | 0.016 | 9.75E-06 | 19.598 |
| order | Desulfovibrionales | rs2692012 | G | A | 0.112 | 0.025 | 2.27E-06 | 19.597 |
| order | Burkholderiales | rs1036451 | T | C | -0.049 | 0.011 | 8.80E-06 | 19.597 |
| order | Bifidobacteriales | rs7174549 | T | C | 0.055 | 0.012 | 6.87E-06 | 19.590 |
| order | Desulfovibrionales | rs7164160 | T | A | -0.057 | 0.013 | 7.76E-06 | 19.586 |
| order | Lactobacillales | rs12448994 | C | T | 0.065 | 0.015 | 7.63E-06 | 19.581 |
| order | Rhodospirillales | rs11945729 | G | A | -0.098 | 0.022 | 7.75E-06 | 19.581 |
| order | Rhodospirillales | rs61350907 | T | A | -0.098 | 0.022 | 7.81E-06 | 19.576 |
| order | Burkholderiales | rs7996696 | G | A | -0.049 | 0.011 | 8.95E-06 | 19.574 |
| order | Burkholderiales | rs2217928 | T | C | -0.049 | 0.011 | 9.05E-06 | 19.574 |
| order | Victivallales | rs253710 | C | T | 0.104 | 0.023 | 9.95E-06 | 19.571 |
| order | Burkholderiales | rs9563572 | C | T | -0.049 | 0.011 | 8.96E-06 | 19.571 |
| order | MollicutesRF9 | rs2459830 | C | T | 0.061 | 0.014 | 9.85E-06 | 19.569 |
| order | Coriobacteriales | rs3025411 | G | A | 0.093 | 0.021 | 8.27E-06 | 19.566 |
| order | Erysipelotrichales | rs2638904 | A | G | 0.047 | 0.011 | 9.64E-06 | 19.553 |
| order | MollicutesRF9 | rs4970749 | A | G | -0.069 | 0.016 | 7.46E-06 | 19.553 |
| order | MollicutesRF9 | rs3932485 | T | C | 0.063 | 0.014 | 9.93E-06 | 19.551 |
| order | Burkholderiales | rs1544475 | C | T | -0.048 | 0.011 | 8.54E-06 | 19.550 |
| order | Clostridiales | rs10517722 | G | C | 0.066 | 0.015 | 9.00E-06 | 19.546 |
| order | Bifidobacteriales | rs669759 | T | C | -0.054 | 0.012 | 5.33E-06 | 19.545 |
| order | Bifidobacteriales | rs677208 | C | G | 0.073 | 0.016 | 6.00E-06 | 19.541 |
| order | Rhodospirillales | rs17022723 | T | C | -0.098 | 0.022 | 7.90E-06 | 19.540 |
| order | Bifidobacteriales | rs13424016 | G | A | 0.053 | 0.012 | 9.54E-06 | 19.539 |
| order | Burkholderiales | rs9563571 | T | C | -0.049 | 0.011 | 9.02E-06 | 19.539 |
| order | Burkholderiales | rs1409526 | A | G | -0.049 | 0.011 | 9.10E-06 | 19.536 |
| order | Bacillales | rs1287018 | A | G | 0.141 | 0.032 | 9.87E-06 | 19.534 |
| order | Victivallales | rs35004924 | A | C | 0.104 | 0.024 | 6.42E-06 | 19.533 |
| order | Burkholderiales | rs7322915 | A | T | -0.050 | 0.011 | 8.66E-06 | 19.530 |
| order | Bifidobacteriales | rs11688302 | C | T | 0.057 | 0.013 | 8.32E-06 | 19.515 |
| order | Bacteroidales | rs1365795 | T | C | 0.090 | 0.020 | 8.08E-06 | 19.500 |
| order | Bacteroidales | rs73975618 | A | G | -0.189 | 0.043 | 6.90E-06 | 19.496 |
| order | Coriobacteriales | rs62448870 | A | G | -0.048 | 0.011 | 9.50E-06 | 19.495 |
| order | Bifidobacteriales | rs10496759 | C | G | 0.064 | 0.014 | 8.80E-06 | 19.494 |
| order | Bacillales | rs4834178 | G | A | -0.164 | 0.037 | 9.59E-06 | 19.488 |
| order | Verrucomicrobiales | rs72663751 | G | A | 0.065 | 0.015 | 8.50E-06 | 19.484 |
| order | Burkholderiales | rs976935 | C | A | -0.048 | 0.011 | 8.85E-06 | 19.474 |
| order | Enterobacteriales | rs11026530 | C | T | 0.082 | 0.019 | 9.43E-06 | 19.471 |
| order | Bifidobacteriales | rs1942051 | A | G | 0.053 | 0.012 | 6.80E-06 | 19.468 |
| order | Clostridiales | rs7687278 | T | A | 0.065 | 0.015 | 9.41E-06 | 19.467 |
| order | Burkholderiales | rs986833 | A | G | -0.048 | 0.011 | 9.45E-06 | 19.466 |
| order | Burkholderiales | rs10182740 | G | A | 0.062 | 0.014 | 9.99E-06 | 19.459 |
| order | Clostridiales | rs6442336 | T | C | -0.055 | 0.012 | 9.63E-06 | 19.458 |
| order | Coriobacteriales | rs7912120 | G | A | 0.072 | 0.016 | 6.24E-06 | 19.451 |
| order | Pasteurellales | rs8125536 | C | T | -0.066 | 0.015 | 9.93E-06 | 19.445 |
| order | Lactobacillales | rs1595463 | A | C | 0.048 | 0.011 | 7.44E-06 | 19.443 |
| order | Burkholderiales | rs7988274 | A | C | -0.048 | 0.011 | 9.54E-06 | 19.435 |
| order | Burkholderiales | rs10444649 | C | T | -0.048 | 0.011 | 9.70E-06 | 19.428 |
| order | Burkholderiales | rs9569799 | A | G | -0.048 | 0.011 | 9.57E-06 | 19.426 |
| order | Burkholderiales | rs9527759 | T | C | -0.048 | 0.011 | 9.43E-06 | 19.418 |
| order | Verrucomicrobiales | rs12904881 | G | A | 0.058 | 0.013 | 9.30E-06 | 19.415 |
| order | Verrucomicrobiales | rs28613890 | C | T | 0.063 | 0.014 | 9.91E-06 | 19.405 |
| order | Verrucomicrobiales | rs28858161 | T | C | 0.062 | 0.014 | 9.03E-06 | 19.396 |
| order | Coriobacteriales | rs11656361 | C | A | 0.077 | 0.018 | 8.02E-06 | 19.394 |
| order | Burkholderiales | rs7997285 | C | T | -0.048 | 0.011 | 9.91E-06 | 19.393 |
| order | Verrucomicrobiales | rs28811282 | G | A | 0.062 | 0.014 | 9.06E-06 | 19.391 |
| order | Erysipelotrichales | rs56970041 | G | T | 0.072 | 0.016 | 5.40E-06 | 19.385 |
| order | Pasteurellales | rs12050685 | G | A | -0.067 | 0.015 | 9.19E-06 | 19.385 |
| order | Verrucomicrobiales | rs10005131 | T | G | 0.061 | 0.014 | 9.81E-06 | 19.381 |
| order | MollicutesRF9 | rs652651 | G | A | -0.069 | 0.016 | 7.93E-06 | 19.368 |
| order | Lactobacillales | rs2370086 | T | G | -0.080 | 0.018 | 9.85E-06 | 19.363 |
| order | Burkholderiales | rs2528688 | G | A | -0.048 | 0.011 | 9.55E-06 | 19.363 |
| order | MollicutesRF9 | rs10745353 | G | A | -0.069 | 0.016 | 9.08E-06 | 19.362 |
| order | Burkholderiales | rs7986644 | A | G | -0.048 | 0.011 | 9.91E-06 | 19.358 |
| order | Clostridiales | rs7667168 | T | C | 0.066 | 0.015 | 9.10E-06 | 19.356 |
| order | Bacillales | rs4915738 | G | C | -0.139 | 0.032 | 9.84E-06 | 19.355 |
| order | Methanobacteriales | rs3912664 | G | T | 0.110 | 0.025 | 8.67E-06 | 19.355 |
| order | Verrucomicrobiales | rs1988252 | G | A | 0.062 | 0.014 | 9.56E-06 | 19.347 |
| order | Rhodospirillales | rs67824877 | T | G | 0.070 | 0.016 | 9.63E-06 | 19.334 |
| order | MollicutesRF9 | rs17361688 | G | A | 0.089 | 0.020 | 7.34E-06 | 19.332 |
| order | Bifidobacteriales | rs2349959 | A | T | 0.056 | 0.013 | 9.28E-06 | 19.327 |
| order | Coriobacteriales | rs1834859 | T | G | -0.057 | 0.013 | 7.97E-06 | 19.301 |
| order | MollicutesRF9 | rs11102969 | A | G | -0.069 | 0.016 | 8.61E-06 | 19.300 |
| order | Methanobacteriales | rs12825290 | G | C | -0.217 | 0.049 | 6.08E-06 | 19.296 |
| order | Gastranaerophilales | rs11806158 | C | A | -0.082 | 0.019 | 8.76E-06 | 19.296 |
| order | Verrucomicrobiales | rs941682 | A | G | -0.063 | 0.014 | 9.61E-06 | 19.290 |
| order | MollicutesRF9 | rs12046539 | T | C | -0.069 | 0.016 | 9.52E-06 | 19.282 |
| order | Verrucomicrobiales | rs2865331 | G | A | 0.062 | 0.014 | 9.67E-06 | 19.282 |
| order | MollicutesRF9 | rs6695482 | G | C | -0.069 | 0.016 | 8.75E-06 | 19.271 |
| order | Bifidobacteriales | rs1879087 | T | C | 0.105 | 0.024 | 1.05E-06 | 19.266 |
| order | Bifidobacteriales | rs676239 | A | T | 0.072 | 0.016 | 6.80E-06 | 19.258 |
| order | Rhodospirillales | rs1593368 | T | C | -0.097 | 0.022 | 9.49E-06 | 19.258 |
| order | Methanobacteriales | rs11018664 | G | A | 0.110 | 0.025 | 9.00E-06 | 19.239 |
| order | Rhodospirillales | rs74354280 | T | C | -0.089 | 0.020 | 9.01E-06 | 19.232 |
| order | Methanobacteriales | rs10830292 | T | G | 0.110 | 0.025 | 9.56E-06 | 19.227 |
| order | MollicutesRF9 | rs3967171 | C | T | 0.089 | 0.020 | 7.36E-06 | 19.224 |
| order | Bacteroidales | NA | T | C | -0.223 | 0.051 | 9.93E-06 | 19.221 |
| order | Rhodospirillales | rs79569783 | G | A | -0.098 | 0.022 | 9.37E-06 | 19.214 |
| order | Rhodospirillales | rs2194876 | C | T | -0.097 | 0.022 | 9.79E-06 | 19.210 |
| order | MollicutesRF9 | rs10745352 | C | T | -0.069 | 0.016 | 8.97E-06 | 19.210 |
| order | Rhodospirillales | rs72714477 | A | T | 0.113 | 0.026 | 9.71E-06 | 19.195 |
| order | Bacteroidales | rs12486971 | T | C | -0.060 | 0.014 | 8.30E-06 | 19.190 |
| order | Rhodospirillales | rs11936579 | A | T | -0.097 | 0.022 | 9.70E-06 | 19.177 |
| order | Desulfovibrionales | rs7163129 | T | C | -0.057 | 0.013 | 9.62E-06 | 19.175 |
| order | Methanobacteriales | rs4385899 | C | T | 0.110 | 0.025 | 9.69E-06 | 19.161 |
| order | MollicutesRF9 | rs444387 | G | A | -0.069 | 0.016 | 9.48E-06 | 19.151 |
| order | Bacteroidales | rs62531359 | G | T | 0.066 | 0.015 | 9.09E-06 | 19.138 |
| order | Lactobacillales | rs74663707 | T | C | 0.098 | 0.022 | 8.40E-06 | 19.137 |
| order | Bifidobacteriales | rs4954187 | C | T | 0.052 | 0.012 | 8.71E-06 | 19.115 |
| order | Methanobacteriales | rs10501707 | A | G | 0.110 | 0.025 | 9.72E-06 | 19.112 |
| order | MollicutesRF9 | rs11102971 | T | C | -0.068 | 0.016 | 9.39E-06 | 19.110 |
| order | MollicutesRF9 | rs10510175 | G | A | 0.089 | 0.020 | 8.05E-06 | 19.106 |
| order | Bacteroidales | rs4146051 | G | A | -0.107 | 0.025 | 8.76E-06 | 19.105 |
| order | Lactobacillales | rs57737477 | A | G | -0.053 | 0.012 | 8.14E-06 | 19.062 |
| order | Coriobacteriales | rs7896306 | T | G | 0.072 | 0.016 | 7.82E-06 | 19.059 |
| order | Clostridiales | rs3792064 | A | G | 0.080 | 0.018 | 9.75E-06 | 19.053 |
| order | Pasteurellales | rs16970009 | G | A | 0.187 | 0.043 | 7.32E-06 | 19.028 |
| order | Clostridiales | rs10209007 | C | G | 0.080 | 0.018 | 9.60E-06 | 19.012 |
| order | Bifidobacteriales | rs659440 | T | A | 0.072 | 0.016 | 7.93E-06 | 18.981 |
| order | Bacillales | rs12522021 | A | T | 0.174 | 0.040 | 7.60E-06 | 18.947 |
| order | Burkholderiales | rs74692987 | G | A | -0.054 | 0.013 | 8.84E-06 | 18.927 |
| order | Bifidobacteriales | rs4954189 | G | A | 0.052 | 0.012 | 9.83E-06 | 18.909 |
| order | Bacillales | rs12434451 | T | G | -0.139 | 0.032 | 8.99E-06 | 18.894 |
| order | Bifidobacteriales | rs10831953 | A | G | 0.054 | 0.012 | 9.95E-06 | 18.869 |
| order | NB1n | rs2930903 | G | C | 0.091 | 0.021 | 6.84E-06 | 18.810 |
| order | Bifidobacteriales | rs558520 | T | C | 0.071 | 0.016 | 8.95E-06 | 18.755 |
| order | Rhodospirillales | rs7078649 | C | A | 0.103 | 0.024 | 9.56E-06 | 18.736 |
| order | MollicutesRF9 | rs4524269 | C | T | 0.088 | 0.020 | 9.70E-06 | 18.731 |
| order | Rhodospirillales | rs56021351 | G | C | 0.101 | 0.023 | 8.79E-06 | 18.691 |
| order | Bacteroidales | rs79585701 | C | A | 0.065 | 0.015 | 9.99E-06 | 18.687 |
| order | Erysipelotrichales | rs10134361 | G | A | 0.071 | 0.016 | 8.66E-06 | 18.642 |
| order | Bifidobacteriales | rs6707350 | C | T | 0.081 | 0.019 | 7.70E-06 | 18.554 |
| order | Burkholderiales | rs75242906 | T | C | -0.121 | 0.028 | 9.75E-06 | 18.498 |
| order | Pasteurellales | rs9895850 | C | T | -0.176 | 0.041 | 9.08E-06 | 18.497 |
| order | Bifidobacteriales | rs11674937 | G | C | 0.060 | 0.014 | 9.88E-06 | 18.448 |
| order | Clostridiales | rs290772 | A | G | 0.084 | 0.020 | 1.00E-05 | 18.423 |
| order | Selenomonadales | rs10787507 | C | G | -0.055 | 0.013 | 9.14E-06 | 18.411 |
| order | Erysipelotrichales | rs10144017 | G | A | 0.069 | 0.016 | 8.50E-06 | 18.267 |
| order | Clostridiales | rs1842454 | A | G | -0.054 | 0.013 | 9.92E-06 | 18.228 |
| order | Bifidobacteriales | rs7572152 | C | G | 0.081 | 0.019 | 8.42E-06 | 18.201 |
| order | MollicutesRF9 | rs12566890 | G | T | -0.103 | 0.024 | 8.11E-06 | 18.196 |
| order | Bifidobacteriales | rs4954218 | T | G | -0.059 | 0.014 | 6.71E-06 | 18.113 |
| order | Bifidobacteriales | rs12446429 | C | T | 0.081 | 0.019 | 8.53E-06 | 18.040 |
| order | Erysipelotrichales | rs7826267 | G | T | -0.084 | 0.020 | 9.28E-06 | 17.755 |
| order | Selenomonadales | rs4463806 | C | T | -0.054 | 0.013 | 7.81E-06 | 17.681 |
| order | Clostridiales | rs76860606 | G | C | 0.094 | 0.023 | 8.58E-06 | 17.481 |
| order | Selenomonadales | rs10885514 | A | G | -0.053 | 0.013 | 8.58E-06 | 17.172 |
| phylum | Actinobacteria | rs7570971 | C | A | 0.087 | 0.011 | 1.41E-14 | 58.162 |
| phylum | Actinobacteria | rs6754311 | T | C | 0.085 | 0.011 | 1.24E-13 | 54.299 |
| phylum | Actinobacteria | rs182549 | T | C | 0.085 | 0.011 | 1.13E-13 | 54.294 |
| phylum | Actinobacteria | rs4988235 | A | G | 0.085 | 0.011 | 1.12E-13 | 54.276 |
| phylum | Actinobacteria | rs1375131 | T | C | 0.082 | 0.011 | 4.16E-13 | 51.694 |
| phylum | Actinobacteria | rs6730157 | A | G | 0.081 | 0.011 | 4.54E-13 | 51.332 |
| phylum | Actinobacteria | rs687670 | T | C | 0.083 | 0.012 | 3.02E-12 | 50.137 |
| phylum | Actinobacteria | rs12465802 | G | A | 0.080 | 0.011 | 1.37E-12 | 49.133 |
| phylum | Actinobacteria | rs13404551 | T | C | 0.081 | 0.012 | 1.05E-11 | 48.061 |
| phylum | Actinobacteria | rs309160 | C | T | 0.081 | 0.012 | 1.40E-11 | 47.356 |
| phylum | Actinobacteria | rs1446585 | A | G | 0.080 | 0.012 | 8.22E-12 | 47.283 |
| phylum | Actinobacteria | rs36084664 | A | G | -0.084 | 0.012 | 3.88E-13 | 46.929 |
| phylum | Actinobacteria | rs218174 | A | G | 0.080 | 0.012 | 2.21E-11 | 46.508 |
| phylum | Actinobacteria | rs632632 | T | C | 0.080 | 0.012 | 2.49E-11 | 46.307 |
| phylum | Actinobacteria | rs309168 | C | T | 0.080 | 0.012 | 2.55E-11 | 46.184 |
| phylum | Actinobacteria | rs309180 | A | G | 0.079 | 0.012 | 2.85E-11 | 45.939 |
| phylum | Actinobacteria | rs309125 | C | T | 0.078 | 0.012 | 4.70E-11 | 44.900 |
| phylum | Actinobacteria | rs13023281 | T | C | -0.081 | 0.012 | 2.61E-12 | 43.886 |
| phylum | Actinobacteria | rs4988183 | T | C | 0.079 | 0.012 | 1.10E-10 | 43.826 |
| phylum | Actinobacteria | rs309134 | G | A | 0.078 | 0.012 | 1.74E-10 | 42.892 |
| phylum | Actinobacteria | rs12615624 | A | G | 0.078 | 0.012 | 1.84E-10 | 42.869 |
| phylum | Actinobacteria | rs4954490 | A | G | 0.077 | 0.012 | 1.74E-10 | 42.549 |
| phylum | Actinobacteria | rs2236783 | A | G | 0.076 | 0.012 | 2.68E-10 | 41.969 |
| phylum | Actinobacteria | rs6759321 | G | T | 0.074 | 0.012 | 1.86E-10 | 41.478 |
| phylum | Actinobacteria | rs12620033 | G | C | 0.076 | 0.012 | 3.70E-10 | 41.241 |
| phylum | Actinobacteria | rs309164 | T | C | 0.076 | 0.012 | 5.44E-10 | 40.603 |
| phylum | Actinobacteria | rs1561277 | C | A | 0.073 | 0.012 | 4.41E-10 | 39.784 |
| phylum | Actinobacteria | rs3754686 | C | T | 0.074 | 0.012 | 7.19E-10 | 39.657 |
| phylum | Actinobacteria | rs12988076 | A | C | 0.074 | 0.012 | 7.58E-10 | 39.590 |
| phylum | Actinobacteria | rs309149 | C | T | 0.075 | 0.012 | 8.65E-10 | 39.588 |
| phylum | Actinobacteria | rs6716536 | C | T | 0.073 | 0.012 | 4.47E-10 | 39.481 |
| phylum | Actinobacteria | rs6742283 | G | A | 0.074 | 0.012 | 9.96E-10 | 39.206 |
| phylum | Actinobacteria | rs2322660 | T | C | 0.074 | 0.012 | 1.01E-09 | 38.982 |
| phylum | Actinobacteria | rs6719488 | T | G | 0.074 | 0.012 | 9.99E-10 | 38.977 |
| phylum | Actinobacteria | rs748841 | T | C | 0.073 | 0.012 | 1.16E-09 | 38.736 |
| phylum | Actinobacteria | rs892715 | C | T | 0.073 | 0.012 | 1.14E-09 | 38.704 |
| phylum | Actinobacteria | rs3769005 | G | C | 0.073 | 0.012 | 1.26E-09 | 38.424 |
| phylum | Actinobacteria | rs745500 | A | G | 0.073 | 0.012 | 1.41E-09 | 38.366 |
| phylum | Actinobacteria | rs2164210 | C | T | 0.073 | 0.012 | 1.70E-09 | 37.908 |
| phylum | Actinobacteria | rs3739020 | T | G | 0.074 | 0.012 | 2.26E-09 | 37.847 |
| phylum | Actinobacteria | rs11681248 | T | C | 0.069 | 0.011 | 1.25E-09 | 37.599 |
| phylum | Actinobacteria | rs12475139 | T | A | 0.071 | 0.012 | 1.36E-09 | 37.313 |
| phylum | Actinobacteria | rs2322659 | C | T | 0.073 | 0.012 | 3.28E-09 | 37.119 |
| phylum | Actinobacteria | rs309137 | T | C | 0.070 | 0.012 | 3.76E-09 | 35.973 |
| phylum | Actinobacteria | rs1470457 | A | G | 0.072 | 0.012 | 5.41E-09 | 35.945 |
| phylum | Actinobacteria | rs749873 | T | C | 0.066 | 0.011 | 7.92E-09 | 34.210 |
| phylum | Actinobacteria | rs17261772 | T | C | 0.070 | 0.012 | 8.37E-09 | 34.185 |
| phylum | Actinobacteria | rs11693502 | T | C | 0.066 | 0.011 | 7.15E-09 | 34.170 |
| phylum | Actinobacteria | rs2278683 | A | C | 0.093 | 0.016 | 8.07E-09 | 33.783 |
| phylum | Actinobacteria | rs7587285 | T | C | 0.093 | 0.016 | 7.55E-09 | 33.769 |
| phylum | Actinobacteria | rs71348714 | A | G | 0.069 | 0.012 | 1.40E-08 | 33.422 |
| phylum | Actinobacteria | rs313526 | C | T | 0.069 | 0.012 | 1.54E-08 | 33.225 |
| phylum | Actinobacteria | rs313518 | C | T | 0.069 | 0.012 | 1.56E-08 | 33.197 |
| phylum | Actinobacteria | rs313528 | G | A | 0.069 | 0.012 | 1.54E-08 | 33.197 |
| phylum | Actinobacteria | rs313519 | A | C | 0.069 | 0.012 | 1.46E-08 | 33.163 |
| phylum | Actinobacteria | rs4954551 | G | A | 0.092 | 0.016 | 9.29E-09 | 33.093 |
| phylum | Actinobacteria | rs11893318 | T | C | 0.092 | 0.016 | 1.19E-08 | 32.996 |
| phylum | Actinobacteria | rs1446584 | A | G | 0.068 | 0.012 | 1.83E-08 | 32.781 |
| phylum | Actinobacteria | rs7355359 | T | C | 0.068 | 0.012 | 2.00E-08 | 32.739 |
| phylum | Actinobacteria | rs4954280 | A | G | 0.068 | 0.012 | 2.05E-08 | 32.554 |
| phylum | Actinobacteria | rs11684545 | T | G | 0.068 | 0.012 | 2.18E-08 | 32.550 |
| phylum | Actinobacteria | rs313517 | A | G | 0.068 | 0.012 | 2.14E-08 | 32.530 |
| phylum | Actinobacteria | rs313524 | C | T | 0.068 | 0.012 | 2.16E-08 | 32.507 |
| phylum | Actinobacteria | rs1011361 | A | G | 0.068 | 0.012 | 2.33E-08 | 32.424 |
| phylum | Actinobacteria | rs6720287 | A | G | 0.068 | 0.012 | 2.22E-08 | 32.380 |
| phylum | Actinobacteria | rs2034276 | A | T | 0.068 | 0.012 | 2.25E-08 | 32.368 |
| phylum | Actinobacteria | rs6430589 | C | G | 0.068 | 0.012 | 2.47E-08 | 32.128 |
| phylum | Actinobacteria | rs10928546 | T | C | 0.068 | 0.012 | 2.62E-08 | 32.102 |
| phylum | Actinobacteria | rs313523 | G | A | 0.068 | 0.012 | 2.52E-08 | 32.086 |
| phylum | Actinobacteria | rs6715856 | T | C | 0.068 | 0.012 | 2.55E-08 | 32.068 |
| phylum | Actinobacteria | rs13412397 | A | G | 0.068 | 0.012 | 3.17E-08 | 31.987 |
| phylum | Actinobacteria | rs71348715 | T | G | 0.068 | 0.012 | 3.12E-08 | 31.911 |
| phylum | Actinobacteria | rs656326 | A | C | 0.067 | 0.012 | 2.83E-08 | 31.877 |
| phylum | Actinobacteria | rs313522 | T | A | 0.067 | 0.012 | 2.89E-08 | 31.832 |
| phylum | Actinobacteria | rs6755383 | C | T | 0.067 | 0.012 | 3.11E-08 | 31.684 |
| phylum | Actinobacteria | rs2082729 | T | C | 0.067 | 0.012 | 3.61E-08 | 31.627 |
| phylum | Actinobacteria | rs11679508 | T | A | 0.067 | 0.012 | 3.80E-08 | 31.626 |
| phylum | Actinobacteria | rs10202489 | A | G | 0.067 | 0.012 | 4.16E-08 | 31.418 |
| phylum | Actinobacteria | rs1438305 | G | C | 0.067 | 0.012 | 4.07E-08 | 31.388 |
| phylum | Actinobacteria | rs4954276 | A | G | 0.067 | 0.012 | 4.06E-08 | 31.369 |
| phylum | Actinobacteria | rs7608045 | T | C | 0.067 | 0.012 | 3.70E-08 | 31.355 |
| phylum | Actinobacteria | rs2680880 | T | A | 0.061 | 0.011 | 4.33E-08 | 31.331 |
| phylum | Actinobacteria | rs12998016 | T | C | 0.067 | 0.012 | 4.08E-08 | 31.306 |
| phylum | Actinobacteria | rs1438304 | G | A | 0.067 | 0.012 | 4.33E-08 | 31.276 |
| phylum | Actinobacteria | rs10928542 | G | A | 0.067 | 0.012 | 3.91E-08 | 31.233 |
| phylum | Actinobacteria | rs28453840 | A | G | 0.067 | 0.012 | 4.86E-08 | 30.961 |
| phylum | Actinobacteria | rs12998387 | G | A | 0.067 | 0.012 | 5.20E-08 | 30.955 |
| phylum | Actinobacteria | rs3213889 | G | A | 0.067 | 0.012 | 5.36E-08 | 30.902 |
| phylum | Actinobacteria | rs62160860 | G | C | 0.090 | 0.016 | 3.91E-08 | 30.891 |
| phylum | Actinobacteria | rs4954279 | G | A | 0.066 | 0.012 | 4.64E-08 | 30.878 |
| phylum | Actinobacteria | rs13413639 | C | A | 0.066 | 0.012 | 4.90E-08 | 30.874 |
| phylum | Actinobacteria | rs12619365 | C | T | 0.067 | 0.012 | 4.79E-08 | 30.804 |
| phylum | Actinobacteria | rs10195620 | T | A | 0.066 | 0.012 | 5.63E-08 | 30.677 |
| phylum | Actinobacteria | rs10173394 | T | C | 0.066 | 0.012 | 6.06E-08 | 30.619 |
| phylum | Actinobacteria | rs1438307 | T | G | 0.066 | 0.012 | 6.08E-08 | 30.560 |
| phylum | Actinobacteria | rs10192827 | A | G | 0.066 | 0.012 | 6.52E-08 | 30.457 |
| phylum | Actinobacteria | rs12994270 | A | T | 0.066 | 0.012 | 8.15E-08 | 30.097 |
| phylum | Actinobacteria | rs6723108 | G | T | 0.060 | 0.011 | 1.02E-08 | 30.071 |
| phylum | Actinobacteria | rs1869829 | A | G | 0.065 | 0.012 | 5.72E-08 | 29.930 |
| phylum | Actinobacteria | rs12469709 | T | C | 0.066 | 0.012 | 8.81E-08 | 29.868 |
| phylum | Actinobacteria | rs932206 | T | C | 0.059 | 0.011 | 6.77E-08 | 29.292 |
| phylum | Actinobacteria | rs1374329 | A | G | 0.066 | 0.012 | 1.45E-07 | 29.141 |
| phylum | Actinobacteria | rs10928554 | C | T | 0.089 | 0.017 | 2.51E-07 | 28.954 |
| phylum | Actinobacteria | rs2011946 | A | C | 0.061 | 0.011 | 1.28E-07 | 28.663 |
| phylum | Actinobacteria | rs2304599 | G | C | 0.066 | 0.012 | 1.96E-07 | 28.567 |
| phylum | Actinobacteria | rs9287442 | G | A | 0.065 | 0.012 | 2.66E-07 | 28.270 |
| phylum | Actinobacteria | rs10928545 | G | A | 0.065 | 0.012 | 3.02E-07 | 28.121 |
| phylum | Actinobacteria | rs10188066 | A | G | 0.065 | 0.012 | 2.66E-07 | 27.888 |
| phylum | Firmicutes | rs7557870 | A | T | 0.099 | 0.019 | 3.26E-06 | 27.775 |
| phylum | Actinobacteria | rs2278544 | G | A | 0.065 | 0.012 | 3.36E-07 | 27.762 |
| phylum | Actinobacteria | rs12616520 | T | A | 0.065 | 0.012 | 3.68E-07 | 27.622 |
| phylum | Actinobacteria | rs6760329 | G | A | 0.065 | 0.012 | 3.06E-07 | 27.611 |
| phylum | Firmicutes | rs13421739 | G | C | 0.094 | 0.018 | 1.95E-06 | 27.611 |
| phylum | Firmicutes | rs7600092 | C | T | 0.098 | 0.019 | 4.07E-06 | 27.404 |
| phylum | Cyanobacteria | rs789068 | A | G | -0.111 | 0.021 | 1.57E-07 | 27.366 |
| phylum | Actinobacteria | rs4429415 | T | C | 0.058 | 0.011 | 2.05E-07 | 27.314 |
| phylum | Actinobacteria | rs1030764 | T | C | 0.064 | 0.012 | 4.06E-07 | 27.191 |
| phylum | Cyanobacteria | rs789067 | C | T | -0.111 | 0.021 | 1.78E-07 | 27.140 |
| phylum | Firmicutes | rs13401441 | G | T | 0.097 | 0.019 | 4.38E-06 | 27.095 |
| phylum | Cyanobacteria | rs789069 | C | A | -0.111 | 0.021 | 1.86E-07 | 27.064 |
| phylum | Actinobacteria | rs935613 | C | T | 0.062 | 0.012 | 2.26E-07 | 27.064 |
| phylum | Bacteroidetes | rs73512608 | A | G | -0.123 | 0.024 | 2.54E-07 | 27.045 |
| phylum | Cyanobacteria | rs9864379 | C | T | -0.139 | 0.027 | 2.03E-07 | 26.866 |
| phylum | Actinobacteria | rs3768998 | G | T | 0.085 | 0.016 | 2.66E-07 | 26.853 |
| phylum | Bacteroidetes | rs55773148 | A | G | -0.123 | 0.024 | 2.84E-07 | 26.851 |
| phylum | Cyanobacteria | rs2005426 | C | A | -0.139 | 0.027 | 2.47E-07 | 26.821 |
| phylum | Actinobacteria | rs10187402 | C | G | 0.061 | 0.012 | 2.49E-07 | 26.662 |
| phylum | Cyanobacteria | rs9830438 | A | C | -0.138 | 0.027 | 2.31E-07 | 26.606 |
| phylum | Bacteroidetes | rs58269684 | C | T | -0.122 | 0.024 | 3.66E-07 | 26.546 |
| phylum | Actinobacteria | rs4954513 | G | A | 0.085 | 0.017 | 4.22E-07 | 26.537 |
| phylum | Bacteroidetes | rs55993203 | G | A | -0.122 | 0.024 | 3.40E-07 | 26.529 |
| phylum | Bacteroidetes | rs60610351 | T | A | -0.122 | 0.024 | 3.71E-07 | 26.467 |
| phylum | Firmicutes | rs1374597 | G | T | 0.093 | 0.018 | 3.34E-06 | 26.445 |
| phylum | Cyanobacteria | rs900188 | A | G | -0.138 | 0.027 | 2.88E-07 | 26.435 |
| phylum | Bacteroidetes | rs56358950 | T | C | -0.122 | 0.024 | 4.11E-07 | 26.403 |
| phylum | Firmicutes | rs10169367 | C | T | 0.092 | 0.018 | 2.78E-06 | 26.365 |
| phylum | Bacteroidetes | rs74090639 | G | A | -0.122 | 0.024 | 4.30E-07 | 26.341 |
| phylum | Actinobacteria | rs16832275 | C | G | 0.082 | 0.016 | 5.54E-07 | 26.232 |
| phylum | Actinobacteria | rs6714750 | A | G | 0.061 | 0.012 | 5.82E-07 | 25.955 |
| phylum | Actinobacteria | rs3820794 | T | C | 0.063 | 0.012 | 7.67E-07 | 25.955 |
| phylum | Actinobacteria | rs1375132 | A | G | 0.060 | 0.012 | 4.42E-07 | 25.951 |
| phylum | Proteobacteria | rs922773 | T | C | -0.080 | 0.016 | 3.68E-07 | 25.928 |
| phylum | Cyanobacteria | rs789063 | A | G | -0.106 | 0.021 | 4.78E-07 | 25.909 |
| phylum | Bacteroidetes | rs58298316 | T | G | -0.120 | 0.024 | 5.16E-07 | 25.895 |
| phylum | Actinobacteria | rs309126 | T | C | 0.079 | 0.016 | 6.77E-07 | 25.892 |
| phylum | Actinobacteria | rs4561607 | C | T | 0.057 | 0.011 | 4.31E-07 | 25.885 |
| phylum | Firmicutes | rs4289208 | T | C | 0.091 | 0.018 | 4.99E-06 | 25.818 |
| phylum | Actinobacteria | rs10841473 | C | G | -0.060 | 0.012 | 4.47E-07 | 25.762 |
| phylum | Bacteroidetes | rs17085818 | A | C | -0.120 | 0.024 | 5.49E-07 | 25.751 |
| phylum | Bacteroidetes | rs17085771 | A | T | -0.120 | 0.024 | 5.70E-07 | 25.717 |
| phylum | Bacteroidetes | rs60614038 | T | A | -0.120 | 0.024 | 5.76E-07 | 25.706 |
| phylum | Bacteroidetes | rs2210454 | C | T | -0.120 | 0.024 | 5.38E-07 | 25.658 |
| phylum | Firmicutes | rs13409228 | G | A | 0.095 | 0.019 | 8.45E-06 | 25.647 |
| phylum | Bacteroidetes | rs2210451 | C | G | -0.120 | 0.024 | 5.18E-07 | 25.634 |
| phylum | Bacteroidetes | rs74090627 | C | T | -0.120 | 0.024 | 5.26E-07 | 25.602 |
| phylum | Actinobacteria | rs2164332 | G | C | 0.081 | 0.016 | 7.37E-07 | 25.591 |
| phylum | Bacteroidetes | rs9788409 | T | C | -0.120 | 0.024 | 6.10E-07 | 25.589 |
| phylum | Actinobacteria | rs16832156 | G | A | 0.081 | 0.016 | 9.33E-07 | 25.577 |
| phylum | Bacteroidetes | rs2150960 | G | C | -0.120 | 0.024 | 5.28E-07 | 25.566 |
| phylum | Actinobacteria | rs309132 | C | G | 0.082 | 0.016 | 9.98E-07 | 25.566 |
| phylum | Bacteroidetes | rs112343530 | T | C | -0.120 | 0.024 | 4.87E-07 | 25.508 |
| phylum | Actinobacteria | rs3769002 | T | C | 0.083 | 0.017 | 6.33E-07 | 25.482 |
| phylum | Cyanobacteria | rs67057512 | G | A | -0.108 | 0.021 | 6.67E-07 | 25.480 |
| phylum | Firmicutes | rs2009919 | C | T | 0.054 | 0.011 | 4.95E-07 | 25.471 |
| phylum | Actinobacteria | rs6747073 | T | C | 0.060 | 0.012 | 5.79E-07 | 25.468 |
| phylum | Actinobacteria | rs56263017 | T | C | 0.083 | 0.017 | 6.34E-07 | 25.468 |
| phylum | Actinobacteria | rs7608980 | G | A | 0.083 | 0.017 | 8.75E-07 | 25.386 |
| phylum | Bacteroidetes | rs56949730 | A | G | -0.119 | 0.024 | 5.90E-07 | 25.378 |
| phylum | Euryarchaeota | rs10202904 | G | T | -0.116 | 0.023 | 6.19E-07 | 25.371 |
| phylum | Bacteroidetes | rs80015632 | C | G | -0.119 | 0.024 | 6.09E-07 | 25.332 |
| phylum | Firmicutes | rs10183008 | G | A | 0.091 | 0.018 | 5.60E-06 | 25.305 |
| phylum | Actinobacteria | rs6752519 | C | T | 0.083 | 0.016 | 7.79E-07 | 25.285 |
| phylum | Bacteroidetes | rs17085798 | C | A | -0.119 | 0.024 | 6.59E-07 | 25.284 |
| phylum | Actinobacteria | rs4450544 | C | T | 0.056 | 0.011 | 6.09E-07 | 25.246 |
| phylum | Bacteroidetes | rs2210452 | G | A | -0.119 | 0.024 | 6.13E-07 | 25.240 |
| phylum | Actinobacteria | rs11679933 | T | G | 0.057 | 0.011 | 7.38E-07 | 25.212 |
| phylum | Actinobacteria | rs1530559 | A | G | 0.055 | 0.011 | 3.78E-07 | 25.206 |
| phylum | Bacteroidetes | rs112675712 | T | C | -0.119 | 0.024 | 5.38E-07 | 25.203 |
| phylum | Actinobacteria | rs16832138 | G | A | 0.083 | 0.017 | 9.59E-07 | 25.187 |
| phylum | Firmicutes | rs978023 | T | A | 0.094 | 0.019 | 9.68E-06 | 25.178 |
| phylum | Verrucomicrobia | rs74542928 | C | T | 0.116 | 0.023 | 4.08E-07 | 25.144 |
| phylum | Actinobacteria | rs16832200 | T | C | 0.080 | 0.016 | 9.71E-07 | 25.097 |
| phylum | Actinobacteria | rs10928513 | C | T | 0.055 | 0.011 | 2.61E-07 | 24.968 |
| phylum | Actinobacteria | rs2322725 | T | C | 0.080 | 0.016 | 9.73E-07 | 24.963 |
| phylum | Tenericutes | rs10108398 | A | G | 0.077 | 0.015 | 1.09E-06 | 24.960 |
| phylum | Actinobacteria | rs6430593 | G | A | 0.080 | 0.016 | 1.32E-06 | 24.942 |
| phylum | Actinobacteria | rs4349285 | T | G | 0.056 | 0.011 | 7.41E-07 | 24.931 |
| phylum | Bacteroidetes | rs7337863 | C | A | -0.119 | 0.024 | 5.27E-07 | 24.873 |
| phylum | Actinobacteria | rs12989104 | G | T | 0.056 | 0.011 | 6.93E-07 | 24.854 |
| phylum | Cyanobacteria | rs66464540 | G | A | -0.104 | 0.021 | 9.32E-07 | 24.847 |
| phylum | Verrucomicrobia | rs112769434 | A | G | 0.115 | 0.023 | 5.23E-07 | 24.803 |
| phylum | Verrucomicrobia | rs2602429 | T | C | 0.076 | 0.015 | 8.71E-07 | 24.786 |
| phylum | Bacteroidetes | rs73846128 | G | A | -0.066 | 0.013 | 4.78E-07 | 24.765 |
| phylum | Actinobacteria | rs56067372 | C | T | 0.082 | 0.017 | 1.23E-06 | 24.724 |
| phylum | Euryarchaeota | rs7601415 | T | A | -0.114 | 0.023 | 8.97E-07 | 24.694 |
| phylum | Verrucomicrobia | rs1491244 | A | T | 0.115 | 0.023 | 5.97E-07 | 24.612 |
| phylum | Bacteroidetes | rs7631304 | A | G | -0.066 | 0.013 | 4.90E-07 | 24.611 |
| phylum | Actinobacteria | rs12105842 | T | C | 0.055 | 0.011 | 8.92E-07 | 24.590 |
| phylum | Actinobacteria | rs74037001 | A | G | -0.082 | 0.017 | 6.71E-07 | 24.562 |
| phylum | Firmicutes | rs10189248 | G | A | 0.089 | 0.018 | 5.81E-06 | 24.552 |
| phylum | Euryarchaeota | rs1869500 | A | C | -0.114 | 0.023 | 9.87E-07 | 24.478 |
| phylum | Actinobacteria | rs3754692 | T | C | 0.060 | 0.012 | 1.52E-06 | 24.436 |
| phylum | Actinobacteria | rs3213871 | C | T | 0.082 | 0.017 | 1.05E-06 | 24.431 |
| phylum | Actinobacteria | rs12478902 | T | C | 0.080 | 0.016 | 1.34E-06 | 24.422 |
| phylum | Lentisphaerae | rs62570196 | T | C | -0.217 | 0.044 | 9.64E-07 | 24.410 |
| phylum | Firmicutes | rs112334273 | A | G | 0.063 | 0.013 | 9.26E-07 | 24.251 |
| phylum | Actinobacteria | rs3754690 | C | T | 0.084 | 0.017 | 1.29E-06 | 24.205 |
| phylum | Bacteroidetes | rs11929346 | T | C | -0.065 | 0.013 | 6.30E-07 | 24.204 |
| phylum | Firmicutes | rs3792064 | A | G | 0.090 | 0.018 | 6.75E-07 | 24.168 |
| phylum | Actinobacteria | rs10193348 | A | G | 0.074 | 0.015 | 3.41E-07 | 24.138 |
| phylum | Cyanobacteria | rs789073 | G | A | -0.102 | 0.021 | 1.13E-06 | 24.121 |
| phylum | Cyanobacteria | rs789061 | G | A | -0.102 | 0.021 | 1.36E-06 | 24.107 |
| phylum | Verrucomicrobia | rs6832119 | A | G | 0.114 | 0.023 | 8.09E-07 | 24.098 |
| phylum | Actinobacteria | rs2016636 | G | A | 0.058 | 0.012 | 1.17E-06 | 24.092 |
| phylum | Actinobacteria | rs4530308 | T | C | 0.055 | 0.011 | 1.15E-06 | 24.049 |
| phylum | Actinobacteria | rs12465650 | T | G | 0.055 | 0.011 | 1.12E-06 | 24.020 |
| phylum | Euryarchaeota | rs76029318 | C | T | 0.215 | 0.044 | 1.05E-06 | 24.017 |
| phylum | Bacteroidetes | rs73510191 | A | C | -0.116 | 0.024 | 1.28E-06 | 24.005 |
| phylum | Bacteroidetes | rs35800684 | G | A | -0.065 | 0.013 | 6.99E-07 | 23.987 |
| phylum | Firmicutes | rs10209007 | C | G | 0.089 | 0.018 | 7.40E-07 | 23.959 |
| phylum | Actinobacteria | rs12328663 | T | C | 0.073 | 0.015 | 3.67E-07 | 23.954 |
| phylum | Bacteroidetes | rs17085738 | T | G | -0.117 | 0.024 | 1.04E-06 | 23.921 |
| phylum | Bacteroidetes | rs17085746 | A | G | -0.117 | 0.024 | 1.04E-06 | 23.921 |
| phylum | Bacteroidetes | rs4474577 | G | A | -0.119 | 0.024 | 1.10E-06 | 23.919 |
| phylum | Actinobacteria | rs1842796 | G | A | 0.054 | 0.011 | 1.18E-06 | 23.918 |
| phylum | Firmicutes | rs10201035 | C | T | 0.090 | 0.018 | 4.77E-06 | 23.912 |
| phylum | Actinobacteria | rs2874739 | C | T | 0.073 | 0.015 | 4.09E-07 | 23.910 |
| phylum | Bacteroidetes | rs7338117 | A | G | -0.117 | 0.024 | 1.02E-06 | 23.909 |
| phylum | Bacteroidetes | rs74937713 | A | T | -0.065 | 0.013 | 8.03E-07 | 23.881 |
| phylum | Bacteroidetes | rs2225529 | C | T | -0.117 | 0.024 | 1.05E-06 | 23.831 |
| phylum | Actinobacteria | rs1900741 | C | T | 0.063 | 0.013 | 2.37E-06 | 23.775 |
| phylum | Bacteroidetes | rs111845179 | C | T | 0.104 | 0.021 | 6.47E-07 | 23.734 |
| phylum | Verrucomicrobia | rs113653192 | C | T | 0.113 | 0.023 | 1.01E-06 | 23.713 |
| phylum | Verrucomicrobia | rs2903202 | A | T | 0.099 | 0.020 | 1.59E-06 | 23.699 |
| phylum | Proteobacteria | rs2532663 | A | G | -0.126 | 0.026 | 7.47E-07 | 23.685 |
| phylum | Verrucomicrobia | rs6832927 | C | A | 0.113 | 0.023 | 1.03E-06 | 23.682 |
| phylum | Actinobacteria | rs7422031 | C | T | 0.073 | 0.015 | 4.91E-07 | 23.675 |
| phylum | Actinobacteria | rs10460330 | A | G | 0.054 | 0.011 | 1.52E-06 | 23.654 |
| phylum | Verrucomicrobia | rs7693865 | C | T | 0.112 | 0.023 | 1.07E-06 | 23.653 |
| phylum | Euryarchaeota | rs73457427 | G | A | 0.213 | 0.044 | 1.26E-06 | 23.643 |
| phylum | Tenericutes | rs67374027 | T | A | 0.075 | 0.015 | 9.70E-07 | 23.634 |
| phylum | Bacteroidetes | rs7611599 | C | T | -0.065 | 0.013 | 8.43E-07 | 23.600 |
| phylum | Bacteroidetes | rs7629132 | C | T | -0.064 | 0.013 | 9.39E-07 | 23.599 |
| phylum | Actinobacteria | rs55888705 | G | A | 0.053 | 0.011 | 1.31E-06 | 23.597 |
| phylum | Actinobacteria | rs56903548 | C | T | 0.073 | 0.015 | 5.07E-07 | 23.582 |
| phylum | Verrucomicrobia | rs17533489 | T | C | 0.099 | 0.020 | 1.61E-06 | 23.573 |
| phylum | Euryarchaeota | rs73457410 | G | A | 0.208 | 0.043 | 2.37E-06 | 23.566 |
| phylum | Tenericutes | rs11890098 | G | A | 0.074 | 0.015 | 9.57E-07 | 23.551 |
| phylum | Tenericutes | rs10111506 | A | T | 0.069 | 0.014 | 1.60E-06 | 23.545 |
| phylum | Tenericutes | rs12674568 | A | G | 0.069 | 0.014 | 1.60E-06 | 23.535 |
| phylum | Actinobacteria | rs62448869 | A | T | -0.052 | 0.011 | 1.14E-06 | 23.490 |
| phylum | Bacteroidetes | rs56335733 | C | T | -0.116 | 0.024 | 1.52E-06 | 23.478 |
| phylum | Bacteroidetes | rs60660711 | G | A | -0.115 | 0.024 | 1.41E-06 | 23.427 |
| phylum | Verrucomicrobia | rs111311092 | A | T | 0.112 | 0.023 | 1.15E-06 | 23.426 |
| phylum | Actinobacteria | rs7568525 | G | A | 0.073 | 0.015 | 4.04E-07 | 23.407 |
| phylum | Verrucomicrobia | rs79606973 | T | A | 0.099 | 0.020 | 1.73E-06 | 23.397 |
| phylum | Tenericutes | rs10102709 | C | T | 0.069 | 0.014 | 1.75E-06 | 23.383 |
| phylum | Bacteroidetes | rs61070599 | C | T | -0.115 | 0.024 | 1.50E-06 | 23.380 |
| phylum | Bacteroidetes | rs12938514 | G | A | -0.201 | 0.042 | 1.27E-06 | 23.367 |
| phylum | Actinobacteria | rs4480940 | T | C | 0.054 | 0.011 | 1.62E-06 | 23.352 |
| phylum | Actinobacteria | rs12465088 | G | A | 0.054 | 0.011 | 1.74E-06 | 23.334 |
| phylum | Actinobacteria | rs10208624 | C | T | 0.073 | 0.015 | 4.30E-07 | 23.327 |
| phylum | Actinobacteria | rs6430561 | C | T | 0.073 | 0.015 | 4.90E-07 | 23.327 |
| phylum | Bacteroidetes | rs11920519 | A | G | -0.064 | 0.013 | 1.11E-06 | 23.306 |
| phylum | Actinobacteria | rs1979033 | G | A | 0.053 | 0.011 | 4.76E-07 | 23.305 |
| phylum | Bacteroidetes | rs73510178 | G | A | -0.115 | 0.024 | 1.37E-06 | 23.295 |
| phylum | Cyanobacteria | rs9864035 | G | A | -0.129 | 0.027 | 1.28E-06 | 23.285 |
| phylum | Actinobacteria | rs61087958 | C | T | 0.072 | 0.015 | 5.50E-07 | 23.278 |
| phylum | Euryarchaeota | rs11147839 | G | A | 0.211 | 0.044 | 1.75E-06 | 23.272 |
| phylum | Bacteroidetes | rs60722059 | A | G | -0.115 | 0.024 | 1.43E-06 | 23.251 |
| phylum | Actinobacteria | rs16855656 | A | G | 0.075 | 0.016 | 2.72E-06 | 23.248 |
| phylum | Actinobacteria | rs7419356 | T | A | 0.054 | 0.011 | 1.83E-06 | 23.243 |
| phylum | Bacteroidetes | rs73512626 | C | A | -0.115 | 0.024 | 1.61E-06 | 23.230 |
| phylum | Bacteroidetes | rs1888900 | T | C | -0.114 | 0.024 | 1.95E-06 | 23.206 |
| phylum | Verrucomicrobia | rs11252894 | C | A | 0.078 | 0.016 | 1.11E-06 | 23.203 |
| phylum | Bacteroidetes | rs12486528 | G | A | -0.065 | 0.013 | 1.05E-06 | 23.197 |
| phylum | Euryarchaeota | rs9594530 | T | C | 0.211 | 0.044 | 1.60E-06 | 23.193 |
| phylum | Tenericutes | rs12680996 | T | C | 0.069 | 0.014 | 1.92E-06 | 23.189 |
| phylum | Verrucomicrobia | rs79837054 | T | C | 0.111 | 0.023 | 1.36E-06 | 23.186 |
| phylum | Bacteroidetes | rs71322866 | A | C | -0.064 | 0.013 | 1.11E-06 | 23.182 |
| phylum | Actinobacteria | rs58327215 | G | C | 0.072 | 0.015 | 5.14E-07 | 23.182 |
| phylum | Tenericutes | rs16923562 | A | G | 0.069 | 0.014 | 1.92E-06 | 23.174 |
| phylum | Actinobacteria | rs62448871 | A | C | -0.052 | 0.011 | 1.32E-06 | 23.151 |
| phylum | Bacteroidetes | rs11916046 | C | A | -0.064 | 0.013 | 1.19E-06 | 23.148 |
| phylum | Verrucomicrobia | rs79194015 | A | T | 0.098 | 0.020 | 2.00E-06 | 23.141 |
| phylum | Verrucomicrobia | rs76564762 | C | T | 0.111 | 0.023 | 1.32E-06 | 23.134 |
| phylum | Proteobacteria | rs12150865 | T | C | 0.051 | 0.011 | 1.54E-06 | 23.118 |
| phylum | Bacteroidetes | rs7546249 | A | T | -0.057 | 0.012 | 1.42E-06 | 23.116 |
| phylum | Tenericutes | rs16923564 | T | C | 0.068 | 0.014 | 1.99E-06 | 23.109 |
| phylum | Bacteroidetes | rs60869989 | G | A | -0.115 | 0.024 | 1.66E-06 | 23.101 |
| phylum | Actinobacteria | rs11898045 | T | C | 0.072 | 0.015 | 5.46E-07 | 23.094 |
| phylum | Bacteroidetes | rs74090631 | A | G | -0.115 | 0.024 | 1.60E-06 | 23.084 |
| phylum | Bacteroidetes | rs73510184 | C | T | -0.115 | 0.024 | 1.60E-06 | 23.084 |
| phylum | Euryarchaeota | rs4848970 | G | T | 0.115 | 0.024 | 1.51E-06 | 23.080 |
| phylum | Actinobacteria | rs7573600 | G | C | 0.072 | 0.015 | 5.86E-07 | 23.079 |
| phylum | Bacteroidetes | rs61289215 | C | T | -0.115 | 0.024 | 1.81E-06 | 23.078 |
| phylum | Tenericutes | rs11776003 | G | A | 0.068 | 0.014 | 2.03E-06 | 23.077 |
| phylum | Bacteroidetes | rs36076607 | T | C | -0.063 | 0.013 | 1.17E-06 | 23.059 |
| phylum | Actinobacteria | rs4954556 | G | A | 0.113 | 0.024 | 3.83E-07 | 23.055 |
| phylum | Bacteroidetes | rs1996069 | T | C | -0.064 | 0.013 | 1.25E-06 | 23.040 |
| phylum | Actinobacteria | rs1482581 | T | C | 0.053 | 0.011 | 1.90E-06 | 22.998 |
| phylum | Verrucomicrobia | rs6837699 | G | A | 0.111 | 0.023 | 1.46E-06 | 22.998 |
| phylum | Tenericutes | rs1991359 | A | G | 0.068 | 0.014 | 2.12E-06 | 22.997 |
| phylum | Actinobacteria | rs4954220 | C | A | 0.072 | 0.015 | 5.72E-07 | 22.997 |
| phylum | Tenericutes | rs28650441 | A | C | 0.068 | 0.014 | 2.13E-06 | 22.994 |
| phylum | Bacteroidetes | rs7337151 | A | C | -0.114 | 0.024 | 1.71E-06 | 22.978 |
| phylum | Lentisphaerae | rs77599476 | G | A | 0.230 | 0.048 | 1.90E-06 | 22.964 |
| phylum | Actinobacteria | rs62448887 | A | C | -0.052 | 0.011 | 1.48E-06 | 22.950 |
| phylum | Bacteroidetes | rs74090626 | A | G | -0.114 | 0.024 | 1.73E-06 | 22.938 |
| phylum | Tenericutes | rs7009248 | A | G | 0.068 | 0.014 | 2.24E-06 | 22.924 |
| phylum | Tenericutes | rs74603314 | C | T | 0.222 | 0.046 | 1.56E-06 | 22.924 |
| phylum | Bacteroidetes | rs73846120 | A | G | -0.064 | 0.013 | 1.26E-06 | 22.917 |
| phylum | Actinobacteria | rs13035345 | G | C | 0.053 | 0.011 | 2.12E-06 | 22.911 |
| phylum | Bacteroidetes | rs60814075 | C | T | -0.114 | 0.024 | 1.74E-06 | 22.909 |
| phylum | Actinobacteria | rs2164331 | C | T | 0.079 | 0.017 | 2.82E-06 | 22.900 |
| phylum | Tenericutes | rs6471729 | G | A | 0.068 | 0.014 | 2.22E-06 | 22.899 |
| phylum | Tenericutes | rs7817515 | T | G | 0.068 | 0.014 | 2.23E-06 | 22.891 |
| phylum | Bacteroidetes | rs2032750 | C | T | -0.051 | 0.011 | 1.71E-06 | 22.878 |
| phylum | Verrucomicrobia | rs76819189 | C | T | 0.098 | 0.020 | 2.19E-06 | 22.877 |
| phylum | Bacteroidetes | rs34519192 | A | G | -0.063 | 0.013 | 1.38E-06 | 22.873 |
| phylum | Bacteroidetes | rs60954548 | A | G | -0.114 | 0.024 | 2.01E-06 | 22.872 |
| phylum | Actinobacteria | rs58426504 | T | G | 0.072 | 0.015 | 6.21E-07 | 22.870 |
| phylum | Verrucomicrobia | rs12416161 | C | T | 0.078 | 0.016 | 1.23E-06 | 22.862 |
| phylum | Lentisphaerae | rs2825714 | G | A | -0.138 | 0.029 | 1.50E-06 | 22.861 |
| phylum | Actinobacteria | rs55634455 | T | C | 0.082 | 0.017 | 2.34E-06 | 22.848 |
| phylum | Actinobacteria | rs78141345 | G | A | 0.082 | 0.017 | 2.39E-06 | 22.838 |
| phylum | Verrucomicrobia | rs117107102 | G | A | 0.204 | 0.043 | 2.68E-06 | 22.828 |
| phylum | Actinobacteria | rs2290518 | G | A | 0.072 | 0.015 | 7.22E-07 | 22.803 |
| phylum | Bacteroidetes | rs1040016 | G | T | -0.063 | 0.013 | 1.44E-06 | 22.801 |
| phylum | Bacteroidetes | rs7491419 | T | G | -0.114 | 0.024 | 1.93E-06 | 22.793 |
| phylum | Euryarchaeota | rs3923545 | G | C | 0.209 | 0.044 | 1.88E-06 | 22.789 |
| phylum | Firmicutes | rs3792068 | A | G | 0.087 | 0.018 | 1.36E-06 | 22.779 |
| phylum | Bacteroidetes | rs17085809 | A | G | -0.114 | 0.024 | 1.95E-06 | 22.779 |
| phylum | Tenericutes | rs1583268 | G | A | 0.073 | 0.015 | 1.45E-06 | 22.775 |
| phylum | Actinobacteria | rs2874834 | A | G | 0.058 | 0.012 | 3.79E-06 | 22.764 |
| phylum | Actinobacteria | rs998451 | A | G | 0.053 | 0.011 | 5.35E-07 | 22.755 |
| phylum | Bacteroidetes | rs2346325 | A | G | -0.063 | 0.013 | 1.48E-06 | 22.755 |
| phylum | Bacteroidetes | rs35532963 | C | G | -0.063 | 0.013 | 1.48E-06 | 22.743 |
| phylum | Lentisphaerae | rs60995569 | G | T | -0.161 | 0.034 | 9.19E-06 | 22.743 |
| phylum | Tenericutes | rs28429491 | C | T | 0.068 | 0.014 | 2.39E-06 | 22.741 |
| phylum | Lentisphaerae | rs11770843 | T | C | 0.112 | 0.023 | 1.14E-06 | 22.715 |
| phylum | Euryarchaeota | rs73031978 | G | C | -0.198 | 0.041 | 2.74E-06 | 22.709 |
| phylum | Bacteroidetes | rs17085776 | A | G | -0.114 | 0.024 | 2.24E-06 | 22.688 |
| phylum | Verrucomicrobia | rs111303866 | C | A | 0.110 | 0.023 | 1.65E-06 | 22.684 |
| phylum | Verrucomicrobia | rs112707021 | G | A | 0.110 | 0.023 | 1.65E-06 | 22.684 |
| phylum | Actinobacteria | rs13389481 | C | A | 0.073 | 0.015 | 7.06E-07 | 22.679 |
| phylum | Bacteroidetes | rs2346324 | A | G | -0.063 | 0.013 | 1.54E-06 | 22.678 |
| phylum | Tenericutes | rs7825996 | A | T | 0.068 | 0.014 | 2.60E-06 | 22.677 |
| phylum | Verrucomicrobia | rs112812594 | G | A | 0.109 | 0.023 | 1.97E-06 | 22.677 |
| phylum | Bacteroidetes | rs1888899 | C | T | -0.113 | 0.024 | 2.70E-06 | 22.674 |
| phylum | Bacteroidetes | rs17085756 | G | A | -0.113 | 0.024 | 2.21E-06 | 22.664 |
| phylum | Actinobacteria | rs62448870 | A | G | -0.051 | 0.011 | 1.72E-06 | 22.656 |
| phylum | Proteobacteria | rs12976403 | G | A | 0.051 | 0.011 | 2.04E-06 | 22.651 |
| phylum | Bacteroidetes | rs907713 | A | C | -0.063 | 0.013 | 1.42E-06 | 22.650 |
| phylum | Bacteroidetes | rs7319197 | A | G | -0.114 | 0.024 | 1.86E-06 | 22.650 |
| phylum | Bacteroidetes | rs7320487 | T | C | -0.114 | 0.024 | 1.86E-06 | 22.647 |
| phylum | Tenericutes | rs7014398 | C | T | 0.068 | 0.014 | 2.55E-06 | 22.628 |
| phylum | Bacteroidetes | rs1014718 | G | A | -0.057 | 0.012 | 1.88E-06 | 22.626 |
| phylum | Verrucomicrobia | rs113911713 | T | C | 0.097 | 0.020 | 2.51E-06 | 22.615 |
| phylum | Verrucomicrobia | rs112520120 | T | C | 0.097 | 0.020 | 2.48E-06 | 22.608 |
| phylum | Verrucomicrobia | rs76877520 | G | A | 0.097 | 0.020 | 2.49E-06 | 22.605 |
| phylum | Actinobacteria | rs6741481 | A | G | 0.071 | 0.015 | 8.06E-07 | 22.594 |
| phylum | Bacteroidetes | rs11916939 | G | A | -0.063 | 0.013 | 1.58E-06 | 22.592 |
| phylum | Bacteroidetes | rs74090642 | A | G | -0.113 | 0.024 | 2.35E-06 | 22.592 |
| phylum | Bacteroidetes | rs59082732 | T | G | -0.113 | 0.024 | 2.45E-06 | 22.583 |
| phylum | Verrucomicrobia | rs4242783 | A | G | 0.069 | 0.015 | 1.75E-06 | 22.579 |
| phylum | Verrucomicrobia | rs111441437 | T | A | 0.097 | 0.020 | 2.60E-06 | 22.579 |
| phylum | Bacteroidetes | rs6794198 | C | T | -0.063 | 0.013 | 1.48E-06 | 22.571 |
| phylum | Bacteroidetes | rs17085765 | G | C | -0.113 | 0.024 | 2.38E-06 | 22.569 |
| phylum | Bacteroidetes | rs75808613 | G | A | -0.113 | 0.024 | 2.12E-06 | 22.567 |
| phylum | Bacteroidetes | rs78745799 | G | T | -0.113 | 0.024 | 2.12E-06 | 22.567 |
| phylum | Tenericutes | rs72647435 | G | T | 0.068 | 0.014 | 2.76E-06 | 22.564 |
| phylum | Cyanobacteria | rs2553290 | T | A | 0.096 | 0.020 | 3.61E-06 | 22.549 |
| phylum | Tenericutes | rs55774738 | T | C | 0.067 | 0.014 | 2.61E-06 | 22.544 |
| phylum | Bacteroidetes | rs34922683 | G | T | -0.063 | 0.013 | 1.67E-06 | 22.531 |
| phylum | Bacteroidetes | rs3946805 | A | G | -0.063 | 0.013 | 1.64E-06 | 22.524 |
| phylum | Tenericutes | rs10099153 | C | G | 0.068 | 0.014 | 2.83E-06 | 22.503 |
| phylum | Bacteroidetes | rs12496485 | G | A | -0.063 | 0.013 | 1.56E-06 | 22.497 |
| phylum | Verrucomicrobia | rs11729256 | C | T | 0.070 | 0.015 | 2.23E-06 | 22.495 |
| phylum | Verrucomicrobia | rs7698547 | C | T | 0.097 | 0.020 | 2.66E-06 | 22.489 |
| phylum | Firmicutes | rs28814056 | G | C | 0.086 | 0.018 | 1.67E-06 | 22.482 |
| phylum | Bacteroidetes | rs1022523 | A | G | -0.056 | 0.012 | 2.00E-06 | 22.478 |
| phylum | Cyanobacteria | rs12493406 | C | T | -0.127 | 0.027 | 3.21E-06 | 22.462 |
| phylum | Bacteroidetes | rs17085785 | C | T | -0.113 | 0.024 | 2.43E-06 | 22.460 |
| phylum | Verrucomicrobia | rs2035814 | A | G | 0.097 | 0.020 | 2.73E-06 | 22.448 |
| phylum | Bacteroidetes | rs2875575 | G | C | -0.112 | 0.024 | 2.88E-06 | 22.432 |
| phylum | Tenericutes | rs4325706 | C | T | 0.073 | 0.015 | 1.73E-06 | 22.418 |
| phylum | Bacteroidetes | rs13100590 | T | C | -0.063 | 0.013 | 1.77E-06 | 22.410 |
| phylum | Actinobacteria | rs6739713 | A | G | 0.057 | 0.012 | 3.94E-06 | 22.400 |
| phylum | Bacteroidetes | rs71322864 | C | A | -0.063 | 0.013 | 1.63E-06 | 22.388 |
| phylum | Lentisphaerae | rs112022900 | G | A | 0.218 | 0.046 | 2.82E-06 | 22.388 |
| phylum | Lentisphaerae | rs45621736 | G | A | 0.218 | 0.046 | 2.76E-06 | 22.384 |
| phylum | Verrucomicrobia | rs6532823 | C | A | 0.097 | 0.020 | 2.80E-06 | 22.372 |
| phylum | Verrucomicrobia | rs75487972 | T | C | 0.097 | 0.020 | 2.74E-06 | 22.362 |
| phylum | Tenericutes | rs12335008 | T | A | 0.068 | 0.014 | 3.08E-06 | 22.358 |
| phylum | Bacteroidetes | rs17026821 | T | C | -0.063 | 0.013 | 1.82E-06 | 22.349 |
| phylum | Actinobacteria | rs58697996 | G | A | 0.071 | 0.015 | 8.16E-07 | 22.345 |
| phylum | Tenericutes | rs72901605 | C | T | -0.084 | 0.018 | 3.26E-06 | 22.338 |
| phylum | Proteobacteria | rs10853762 | T | A | 0.050 | 0.011 | 2.36E-06 | 22.327 |
| phylum | Euryarchaeota | rs62241835 | T | G | -0.194 | 0.041 | 3.78E-06 | 22.302 |
| phylum | Tenericutes | rs10099912 | G | T | 0.068 | 0.014 | 3.14E-06 | 22.301 |
| phylum | Proteobacteria | rs7246771 | G | C | 0.050 | 0.011 | 2.37E-06 | 22.301 |
| phylum | Tenericutes | rs9297995 | C | T | 0.067 | 0.014 | 3.00E-06 | 22.283 |
| phylum | Bacteroidetes | rs1555070 | C | T | -0.056 | 0.012 | 2.24E-06 | 22.272 |
| phylum | Tenericutes | rs13025440 | T | G | 0.075 | 0.016 | 2.21E-06 | 22.254 |
| phylum | Proteobacteria | rs9797816 | G | A | 0.050 | 0.011 | 2.45E-06 | 22.242 |
| phylum | Verrucomicrobia | rs41275715 | C | T | 0.096 | 0.020 | 2.92E-06 | 22.225 |
| phylum | Actinobacteria | rs4954214 | C | T | 0.071 | 0.015 | 1.03E-06 | 22.223 |
| phylum | Bacteroidetes | rs2183908 | T | C | -0.112 | 0.024 | 2.30E-06 | 22.219 |
| phylum | Actinobacteria | rs6738563 | G | T | 0.051 | 0.011 | 9.58E-07 | 22.216 |
| phylum | Actinobacteria | rs13410870 | G | A | 0.071 | 0.015 | 9.75E-07 | 22.207 |
| phylum | Bacteroidetes | rs13291434 | C | T | 0.071 | 0.015 | 3.01E-06 | 22.206 |
| phylum | Bacteroidetes | rs13291169 | G | C | 0.070 | 0.015 | 2.61E-06 | 22.202 |
| phylum | Verrucomicrobia | rs45598138 | A | C | -0.144 | 0.031 | 2.19E-06 | 22.202 |
| phylum | Tenericutes | rs10113750 | C | T | 0.067 | 0.014 | 3.15E-06 | 22.199 |
| phylum | Bacteroidetes | rs34046877 | T | C | -0.062 | 0.013 | 1.81E-06 | 22.196 |
| phylum | Actinobacteria | rs1942055 | G | C | 0.052 | 0.011 | 9.78E-07 | 22.192 |
| phylum | Actinobacteria | rs584226 | T | C | 0.076 | 0.016 | 4.02E-06 | 22.192 |
| phylum | Verrucomicrobia | rs745075 | T | C | 0.096 | 0.020 | 2.98E-06 | 22.189 |
| phylum | Cyanobacteria | rs12486770 | T | A | -0.126 | 0.027 | 3.67E-06 | 22.187 |
| phylum | Euryarchaeota | rs62241836 | C | T | -0.194 | 0.041 | 3.77E-06 | 22.187 |
| phylum | Verrucomicrobia | rs2035815 | T | C | 0.096 | 0.020 | 3.39E-06 | 22.178 |
| phylum | Actinobacteria | rs62168941 | C | T | 0.071 | 0.015 | 1.10E-06 | 22.169 |
| phylum | Cyanobacteria | rs6773199 | G | A | -0.124 | 0.026 | 2.75E-06 | 22.155 |
| phylum | Tenericutes | rs7011095 | T | A | 0.068 | 0.014 | 3.41E-06 | 22.154 |
| phylum | Bacteroidetes | rs17026797 | G | T | -0.062 | 0.013 | 2.02E-06 | 22.146 |
| phylum | Bacteroidetes | rs17085791 | G | C | -0.112 | 0.024 | 2.74E-06 | 22.145 |
| phylum | Tenericutes | rs7009374 | C | T | 0.067 | 0.014 | 3.26E-06 | 22.138 |
| phylum | Verrucomicrobia | rs1491237 | A | G | 0.096 | 0.020 | 3.20E-06 | 22.134 |
| phylum | Cyanobacteria | rs62231951 | A | T | -0.126 | 0.027 | 3.77E-06 | 22.128 |
| phylum | Tenericutes | rs59979442 | A | G | 0.067 | 0.014 | 3.43E-06 | 22.119 |
| phylum | Bacteroidetes | rs13060907 | G | A | -0.062 | 0.013 | 1.95E-06 | 22.102 |
| phylum | Tenericutes | rs13024040 | C | T | 0.074 | 0.016 | 2.13E-06 | 22.101 |
| phylum | Verrucomicrobia | rs745074 | T | A | 0.096 | 0.020 | 3.15E-06 | 22.098 |
| phylum | Actinobacteria | rs62168942 | T | C | 0.071 | 0.015 | 1.13E-06 | 22.097 |
| phylum | Tenericutes | rs12676593 | A | G | 0.067 | 0.014 | 3.49E-06 | 22.092 |
| phylum | Cyanobacteria | rs76531781 | C | T | -0.232 | 0.049 | 2.87E-06 | 22.085 |
| phylum | Cyanobacteria | rs9748924 | C | T | -0.100 | 0.021 | 3.77E-06 | 22.079 |
| phylum | Euryarchaeota | rs77658038 | C | A | -0.160 | 0.034 | 4.75E-06 | 22.077 |
| phylum | Cyanobacteria | rs2314810 | G | C | -0.218 | 0.046 | 4.25E-06 | 22.071 |
| phylum | Tenericutes | rs10090964 | A | T | 0.067 | 0.014 | 3.39E-06 | 22.065 |
| phylum | Cyanobacteria | rs9883109 | T | C | -0.126 | 0.027 | 2.38E-06 | 22.059 |
| phylum | Actinobacteria | rs1879087 | T | C | 0.102 | 0.022 | 2.48E-07 | 22.058 |
| phylum | Actinobacteria | rs62287787 | C | T | 0.052 | 0.011 | 2.89E-06 | 22.055 |
| phylum | Verrucomicrobia | rs111664214 | C | T | 0.096 | 0.020 | 3.27E-06 | 22.052 |
| phylum | Euryarchaeota | rs34928225 | C | T | 0.200 | 0.043 | 4.33E-06 | 22.051 |
| phylum | Verrucomicrobia | rs112108820 | C | T | 0.095 | 0.020 | 3.80E-06 | 22.037 |
| phylum | Firmicutes | rs7975768 | G | C | 0.051 | 0.011 | 2.82E-06 | 22.036 |
| phylum | Euryarchaeota | rs12711690 | G | A | -0.108 | 0.023 | 3.44E-06 | 22.033 |
| phylum | Bacteroidetes | rs6777119 | T | C | -0.062 | 0.013 | 2.13E-06 | 22.006 |
| phylum | Actinobacteria | rs10167901 | C | T | 0.071 | 0.015 | 1.14E-06 | 22.004 |
| phylum | Verrucomicrobia | rs1491246 | A | T | 0.096 | 0.020 | 3.31E-06 | 21.996 |
| phylum | Actinobacteria | rs7603805 | A | G | 0.070 | 0.015 | 1.19E-06 | 21.996 |
| phylum | Tenericutes | rs10094910 | T | C | 0.067 | 0.014 | 3.61E-06 | 21.991 |
| phylum | Actinobacteria | rs55912948 | A | G | 0.051 | 0.011 | 3.08E-06 | 21.980 |
| phylum | Euryarchaeota | rs9942108 | G | A | -0.194 | 0.041 | 4.01E-06 | 21.977 |
| phylum | Cyanobacteria | rs145461062 | C | T | -0.100 | 0.021 | 3.98E-06 | 21.975 |
| phylum | Verrucomicrobia | rs10516447 | A | G | 0.108 | 0.023 | 2.38E-06 | 21.966 |
| phylum | Actinobacteria | rs8047955 | G | A | 0.052 | 0.011 | 2.66E-06 | 21.964 |
| phylum | Bacteroidetes | rs35176045 | C | T | -0.062 | 0.013 | 1.96E-06 | 21.943 |
| phylum | Actinobacteria | rs10209482 | A | C | 0.071 | 0.015 | 8.74E-07 | 21.942 |
| phylum | Tenericutes | rs7590919 | G | A | 0.072 | 0.015 | 2.25E-06 | 21.936 |
| phylum | Actinobacteria | rs4953947 | A | C | 0.070 | 0.015 | 1.15E-06 | 21.934 |
| phylum | Actinobacteria | rs6729961 | T | G | 0.092 | 0.020 | 1.93E-06 | 21.920 |
| phylum | Actinobacteria | rs921859 | A | G | 0.070 | 0.015 | 1.23E-06 | 21.912 |
| phylum | Proteobacteria | rs7246428 | G | T | 0.050 | 0.011 | 2.93E-06 | 21.909 |
| phylum | Bacteroidetes | rs73975615 | A | G | -0.207 | 0.044 | 1.20E-06 | 21.905 |
| phylum | Actinobacteria | rs2017831 | C | T | 0.070 | 0.015 | 1.25E-06 | 21.905 |
| phylum | Actinobacteria | rs10167809 | C | T | 0.070 | 0.015 | 1.19E-06 | 21.901 |
| phylum | Actinobacteria | rs12233092 | G | A | 0.075 | 0.016 | 4.66E-06 | 21.899 |
| phylum | Actinobacteria | rs10167884 | C | T | 0.070 | 0.015 | 1.19E-06 | 21.898 |
| phylum | Actinobacteria | rs4953948 | T | G | 0.070 | 0.015 | 1.19E-06 | 21.894 |
| phylum | Verrucomicrobia | rs1491245 | T | G | 0.096 | 0.020 | 3.50E-06 | 21.893 |
| phylum | Bacteroidetes | rs62575403 | T | C | 0.145 | 0.031 | 2.96E-06 | 21.854 |
| phylum | Proteobacteria | rs11083677 | C | T | 0.050 | 0.011 | 3.01E-06 | 21.843 |
| phylum | Actinobacteria | rs7601696 | C | A | 0.070 | 0.015 | 1.34E-06 | 21.833 |
| phylum | Actinobacteria | rs1397793 | A | G | -0.052 | 0.011 | 3.74E-06 | 21.832 |
| phylum | Tenericutes | rs28809811 | G | T | 0.068 | 0.015 | 4.12E-06 | 21.828 |
| phylum | Actinobacteria | rs4954217 | C | T | 0.070 | 0.015 | 1.34E-06 | 21.816 |
| phylum | Firmicutes | rs13386595 | A | G | 0.085 | 0.018 | 2.29E-06 | 21.811 |
| phylum | Actinobacteria | rs4954216 | G | A | 0.070 | 0.015 | 1.25E-06 | 21.810 |
| phylum | Actinobacteria | rs7577258 | G | A | 0.070 | 0.015 | 1.36E-06 | 21.806 |
| phylum | Euryarchaeota | rs62241834 | G | T | -0.192 | 0.041 | 5.07E-06 | 21.793 |
| phylum | Proteobacteria | rs7246855 | T | C | 0.050 | 0.011 | 3.13E-06 | 21.783 |
| phylum | Cyanobacteria | rs2608306 | A | G | 0.095 | 0.020 | 5.37E-06 | 21.767 |
| phylum | Euryarchaeota | rs56209241 | G | A | -0.194 | 0.042 | 4.86E-06 | 21.764 |
| phylum | Proteobacteria | rs11671245 | T | C | 0.050 | 0.011 | 3.12E-06 | 21.761 |
| phylum | Verrucomicrobia | rs6842339 | T | G | 0.107 | 0.023 | 2.82E-06 | 21.751 |
| phylum | Actinobacteria | rs4954221 | G | A | 0.070 | 0.015 | 1.08E-06 | 21.751 |
| phylum | Actinobacteria | rs4953946 | T | G | 0.070 | 0.015 | 1.30E-06 | 21.739 |
| phylum | Actinobacteria | rs13427494 | G | A | 0.071 | 0.015 | 9.66E-07 | 21.739 |
| phylum | Actinobacteria | rs4954215 | G | A | 0.070 | 0.015 | 1.37E-06 | 21.739 |
| phylum | Verrucomicrobia | rs112048442 | G | A | 0.107 | 0.023 | 2.84E-06 | 21.738 |
| phylum | Verrucomicrobia | rs17533517 | C | T | 0.095 | 0.020 | 3.93E-06 | 21.738 |
| phylum | Verrucomicrobia | rs113634144 | G | T | 0.068 | 0.015 | 3.56E-06 | 21.733 |
| phylum | Proteobacteria | rs7249825 | G | T | 0.050 | 0.011 | 3.21E-06 | 21.731 |
| phylum | Bacteroidetes | rs73510192 | T | C | -0.113 | 0.024 | 2.87E-06 | 21.726 |
| phylum | Actinobacteria | rs7565268 | T | C | 0.070 | 0.015 | 1.42E-06 | 21.726 |
| phylum | Proteobacteria | rs4029163 | A | C | 0.050 | 0.011 | 3.20E-06 | 21.716 |
| phylum | Euryarchaeota | rs9532770 | T | C | 0.126 | 0.027 | 3.74E-06 | 21.710 |
| phylum | Actinobacteria | rs4954445 | T | A | 0.080 | 0.017 | 3.22E-06 | 21.700 |
| phylum | Cyanobacteria | rs2553291 | A | G | 0.094 | 0.020 | 5.51E-06 | 21.699 |
| phylum | Verrucomicrobia | rs74399905 | T | A | 0.107 | 0.023 | 2.71E-06 | 21.698 |
| phylum | Actinobacteria | rs10186594 | A | G | 0.070 | 0.015 | 1.41E-06 | 21.697 |
| phylum | Cyanobacteria | rs2608309 | G | A | 0.094 | 0.020 | 5.57E-06 | 21.695 |
| phylum | Actinobacteria | rs13409400 | A | C | 0.070 | 0.015 | 1.41E-06 | 21.693 |
| phylum | Tenericutes | rs11783174 | G | T | 0.067 | 0.014 | 4.33E-06 | 21.688 |
| phylum | Tenericutes | rs6981601 | C | T | 0.066 | 0.014 | 4.05E-06 | 21.679 |
| phylum | Verrucomicrobia | rs114505328 | T | A | 0.095 | 0.020 | 4.48E-06 | 21.679 |
| phylum | Euryarchaeota | rs62240385 | T | G | -0.192 | 0.041 | 4.85E-06 | 21.678 |
| phylum | Actinobacteria | rs10182750 | T | C | 0.070 | 0.015 | 1.38E-06 | 21.675 |
| phylum | Firmicutes | rs1968246 | T | C | 0.050 | 0.011 | 3.71E-06 | 21.663 |
| phylum | Cyanobacteria | rs9863760 | C | T | -0.121 | 0.026 | 3.18E-06 | 21.660 |
| phylum | Proteobacteria | rs7246141 | G | C | 0.049 | 0.011 | 3.35E-06 | 21.652 |
| phylum | Cyanobacteria | rs2714559 | T | C | 0.094 | 0.020 | 5.59E-06 | 21.646 |
| phylum | Actinobacteria | rs3769011 | A | T | 0.080 | 0.017 | 3.37E-06 | 21.645 |
| phylum | Bacteroidetes | rs1996068 | A | G | -0.062 | 0.013 | 2.70E-06 | 21.632 |
| phylum | Bacteroidetes | rs6791217 | G | T | -0.062 | 0.013 | 2.48E-06 | 21.629 |
| phylum | Bacteroidetes | rs1325597 | G | T | -0.055 | 0.012 | 3.05E-06 | 21.622 |
| phylum | Tenericutes | rs6986129 | A | T | 0.066 | 0.014 | 4.19E-06 | 21.619 |
| phylum | Proteobacteria | rs11666257 | A | G | 0.050 | 0.011 | 3.45E-06 | 21.617 |
| phylum | Tenericutes | rs1583269 | C | G | 0.072 | 0.015 | 2.62E-06 | 21.611 |
| phylum | Cyanobacteria | rs2714557 | A | T | 0.094 | 0.020 | 5.84E-06 | 21.603 |
| phylum | Bacteroidetes | rs35665203 | T | C | -0.062 | 0.013 | 2.49E-06 | 21.594 |
| phylum | Lentisphaerae | rs11764871 | T | G | 0.108 | 0.023 | 3.70E-06 | 21.591 |
| phylum | Euryarchaeota | rs9477075 | G | A | -0.155 | 0.033 | 7.59E-06 | 21.590 |
| phylum | Actinobacteria | rs7565149 | T | A | 0.070 | 0.015 | 1.51E-06 | 21.589 |
| phylum | Actinobacteria | rs7601683 | C | T | 0.070 | 0.015 | 1.53E-06 | 21.586 |
| phylum | Tenericutes | rs13027493 | T | A | 0.071 | 0.015 | 2.71E-06 | 21.580 |
| phylum | Proteobacteria | rs55717966 | G | C | 0.049 | 0.011 | 3.46E-06 | 21.579 |
| phylum | Cyanobacteria | rs584122 | T | C | -0.152 | 0.033 | 4.23E-06 | 21.565 |
| phylum | Lentisphaerae | rs2731834 | G | C | 0.110 | 0.024 | 3.80E-06 | 21.544 |
| phylum | Cyanobacteria | rs13062781 | G | A | -0.121 | 0.026 | 2.70E-06 | 21.541 |
| phylum | Verrucomicrobia | rs76826818 | A | G | 0.095 | 0.020 | 4.37E-06 | 21.535 |
| phylum | Verrucomicrobia | rs17533020 | A | G | 0.095 | 0.020 | 4.79E-06 | 21.529 |
| phylum | Verrucomicrobia | rs12908520 | A | G | 0.059 | 0.013 | 3.40E-06 | 21.524 |
| phylum | Actinobacteria | rs56211644 | T | C | 0.079 | 0.017 | 4.40E-06 | 21.514 |
| phylum | Proteobacteria | rs6509051 | A | G | 0.049 | 0.011 | 3.62E-06 | 21.502 |
| phylum | Tenericutes | rs10111052 | T | G | 0.066 | 0.014 | 4.70E-06 | 21.479 |
| phylum | Bacteroidetes | rs60475411 | T | C | -0.061 | 0.013 | 2.86E-06 | 21.472 |
| phylum | Firmicutes | rs11683620 | A | G | 0.084 | 0.018 | 3.31E-06 | 21.460 |
| phylum | Verrucomicrobia | rs7668586 | C | T | 0.095 | 0.020 | 4.59E-06 | 21.451 |
| phylum | Cyanobacteria | rs2608308 | G | A | 0.094 | 0.020 | 6.39E-06 | 21.426 |
| phylum | Actinobacteria | rs56174523 | A | C | 0.070 | 0.015 | 1.60E-06 | 21.418 |
| phylum | Euryarchaeota | rs62240373 | G | A | -0.190 | 0.041 | 5.93E-06 | 21.409 |
| phylum | Euryarchaeota | rs894996 | A | C | 0.204 | 0.044 | 5.12E-06 | 21.404 |
| phylum | Euryarchaeota | rs7635189 | A | G | 0.120 | 0.026 | 4.64E-06 | 21.402 |
| phylum | Tenericutes | rs6043847 | C | T | -0.115 | 0.025 | 4.55E-06 | 21.375 |
| phylum | Bacteroidetes | rs17343978 | C | A | -0.056 | 0.012 | 7.22E-06 | 21.374 |
| phylum | Bacteroidetes | rs13097740 | G | A | -0.061 | 0.013 | 2.54E-06 | 21.372 |
| phylum | Proteobacteria | rs2736151 | C | T | 0.049 | 0.011 | 3.80E-06 | 21.371 |
| phylum | Cyanobacteria | rs10510427 | G | A | -0.122 | 0.026 | 5.69E-06 | 21.369 |
| phylum | Actinobacteria | rs62168934 | A | G | 0.070 | 0.015 | 1.66E-06 | 21.367 |
| phylum | Actinobacteria | rs13402112 | A | C | 0.070 | 0.015 | 1.64E-06 | 21.362 |
| phylum | Proteobacteria | rs11670025 | T | G | 0.049 | 0.011 | 3.83E-06 | 21.360 |
| phylum | Actinobacteria | rs11888951 | T | C | 0.070 | 0.015 | 1.60E-06 | 21.349 |
| phylum | Proteobacteria | rs11881408 | C | T | 0.049 | 0.011 | 3.88E-06 | 21.346 |
| phylum | Actinobacteria | rs10496736 | C | G | 0.090 | 0.020 | 2.24E-06 | 21.341 |
| phylum | Proteobacteria | rs2526711 | A | G | 0.049 | 0.011 | 3.90E-06 | 21.322 |
| phylum | Actinobacteria | rs6760553 | A | G | 0.069 | 0.015 | 1.65E-06 | 21.316 |
| phylum | Actinobacteria | rs857444 | T | C | 0.051 | 0.011 | 3.80E-06 | 21.315 |
| phylum | Verrucomicrobia | rs76430504 | C | T | -0.118 | 0.025 | 3.50E-06 | 21.313 |
| phylum | Actinobacteria | rs57074605 | T | C | 0.051 | 0.011 | 4.32E-06 | 21.307 |
| phylum | Bacteroidetes | rs7857688 | G | A | 0.065 | 0.014 | 3.65E-06 | 21.307 |
| phylum | Proteobacteria | rs12977541 | T | C | 0.049 | 0.011 | 3.99E-06 | 21.301 |
| phylum | Proteobacteria | rs2471952 | C | T | 0.049 | 0.011 | 3.95E-06 | 21.296 |
| phylum | Lentisphaerae | rs1002941 | A | G | 0.108 | 0.023 | 4.31E-06 | 21.280 |
| phylum | Bacteroidetes | rs11871360 | A | G | -0.204 | 0.044 | 1.70E-06 | 21.273 |
| phylum | Lentisphaerae | rs6951588 | T | C | 0.107 | 0.023 | 4.47E-06 | 21.267 |
| phylum | Actinobacteria | rs60475440 | T | C | 0.050 | 0.011 | 4.42E-06 | 21.267 |
| phylum | Actinobacteria | rs13413872 | G | T | 0.069 | 0.015 | 1.72E-06 | 21.263 |
| phylum | Actinobacteria | rs58080116 | A | G | 0.079 | 0.017 | 4.32E-06 | 21.254 |
| phylum | Actinobacteria | rs13008147 | A | G | -0.057 | 0.012 | 1.37E-06 | 21.248 |
| phylum | Proteobacteria | rs2347697 | T | G | 0.050 | 0.011 | 4.27E-06 | 21.247 |
| phylum | Proteobacteria | rs11083672 | G | C | 0.049 | 0.011 | 4.31E-06 | 21.239 |
| phylum | Euryarchaeota | rs6784384 | G | A | -0.187 | 0.041 | 5.47E-06 | 21.238 |
| phylum | Euryarchaeota | rs9942109 | G | A | -0.190 | 0.041 | 6.06E-06 | 21.237 |
| phylum | Proteobacteria | rs7257768 | A | G | 0.049 | 0.011 | 4.11E-06 | 21.234 |
| phylum | Firmicutes | rs11108755 | T | A | 0.050 | 0.011 | 4.31E-06 | 21.229 |
| phylum | Actinobacteria | rs67856226 | A | G | 0.050 | 0.011 | 4.59E-06 | 21.222 |
| phylum | Bacteroidetes | rs73510188 | G | A | -0.111 | 0.024 | 3.63E-06 | 21.209 |
| phylum | Bacteroidetes | rs73510190 | G | C | -0.111 | 0.024 | 3.63E-06 | 21.209 |
| phylum | Proteobacteria | rs2347698 | A | G | 0.050 | 0.011 | 4.37E-06 | 21.206 |
| phylum | Cyanobacteria | rs2261414 | C | T | 0.093 | 0.020 | 7.11E-06 | 21.202 |
| phylum | Firmicutes | rs2332027 | A | G | -0.048 | 0.010 | 4.05E-06 | 21.201 |
| phylum | Actinobacteria | rs74266324 | G | A | 0.063 | 0.014 | 2.70E-06 | 21.198 |
| phylum | Tenericutes | rs12476593 | C | G | 0.073 | 0.016 | 3.84E-06 | 21.194 |
| phylum | Euryarchaeota | rs41288287 | C | T | 0.201 | 0.044 | 5.39E-06 | 21.192 |
| phylum | Actinobacteria | rs60376570 | G | A | 0.079 | 0.017 | 4.31E-06 | 21.189 |
| phylum | Actinobacteria | rs55657163 | G | A | 0.051 | 0.011 | 4.41E-06 | 21.182 |
| phylum | Proteobacteria | rs4803563 | A | G | 0.049 | 0.011 | 4.35E-06 | 21.181 |
| phylum | Cyanobacteria | rs4407129 | C | T | -0.096 | 0.021 | 5.80E-06 | 21.174 |
| phylum | Actinobacteria | rs61470941 | T | C | 0.079 | 0.017 | 3.86E-06 | 21.156 |
| phylum | Actinobacteria | rs9833771 | C | T | 0.049 | 0.011 | 4.07E-06 | 21.150 |
| phylum | Proteobacteria | rs11083679 | G | A | 0.049 | 0.011 | 4.28E-06 | 21.149 |
| phylum | Euryarchaeota | rs62240384 | C | T | -0.187 | 0.041 | 5.72E-06 | 21.144 |
| phylum | Euryarchaeota | rs6541968 | T | C | -0.106 | 0.023 | 5.41E-06 | 21.120 |
| phylum | Lentisphaerae | rs17114848 | A | G | 0.149 | 0.032 | 6.77E-06 | 21.120 |
| phylum | Actinobacteria | rs13006808 | A | C | 0.053 | 0.011 | 3.50E-06 | 21.113 |
| phylum | Verrucomicrobia | rs61779207 | A | G | -0.076 | 0.016 | 5.28E-06 | 21.109 |
| phylum | Actinobacteria | rs58764609 | G | T | 0.050 | 0.011 | 4.83E-06 | 21.099 |
| phylum | Cyanobacteria | rs6442436 | C | T | -0.123 | 0.027 | 3.94E-06 | 21.095 |
| phylum | Tenericutes | rs13015145 | G | A | 0.071 | 0.015 | 3.45E-06 | 21.094 |
| phylum | Actinobacteria | rs6749119 | C | G | 0.069 | 0.015 | 1.69E-06 | 21.081 |
| phylum | Firmicutes | rs3852931 | T | C | -0.048 | 0.010 | 4.53E-06 | 21.080 |
| phylum | Firmicutes | rs6436974 | G | A | 0.083 | 0.018 | 3.57E-06 | 21.059 |
| phylum | Actinobacteria | rs2322812 | A | G | 0.079 | 0.017 | 4.67E-06 | 21.056 |
| phylum | Cyanobacteria | rs4494631 | A | G | -0.095 | 0.021 | 5.98E-06 | 21.056 |
| phylum | Actinobacteria | rs10174944 | C | T | 0.069 | 0.015 | 1.96E-06 | 21.049 |
| phylum | Verrucomicrobia | rs9349825 | G | A | -0.066 | 0.014 | 6.27E-06 | 21.048 |
| phylum | Firmicutes | NA | T | C | 0.233 | 0.051 | 8.52E-06 | 21.042 |
| phylum | Cyanobacteria | rs13098772 | C | A | -0.123 | 0.027 | 5.45E-06 | 21.041 |
| phylum | Firmicutes | rs2273429 | G | A | -0.070 | 0.015 | 9.26E-06 | 21.041 |
| phylum | Actinobacteria | rs12992815 | C | G | 0.078 | 0.017 | 4.64E-06 | 21.036 |
| phylum | Bacteroidetes | rs7157380 | G | C | 0.098 | 0.021 | 3.13E-06 | 21.032 |
| phylum | Actinobacteria | rs60350840 | A | G | 0.069 | 0.015 | 1.99E-06 | 21.028 |
| phylum | Firmicutes | rs72771021 | T | C | -0.141 | 0.031 | 5.12E-06 | 21.022 |
| phylum | Proteobacteria | rs10412444 | C | T | 0.049 | 0.011 | 4.61E-06 | 21.011 |
| phylum | Cyanobacteria | rs1567601 | T | A | -0.099 | 0.022 | 5.66E-06 | 21.009 |
| phylum | Proteobacteria | rs6467551 | G | C | 0.050 | 0.011 | 5.39E-06 | 21.009 |
| phylum | Proteobacteria | rs312757 | G | C | -0.052 | 0.011 | 4.84E-06 | 21.009 |
| phylum | Proteobacteria | rs72771021 | T | C | 0.142 | 0.031 | 7.18E-06 | 21.004 |
| phylum | Cyanobacteria | rs6780959 | A | G | -0.122 | 0.027 | 4.04E-06 | 21.002 |
| phylum | Euryarchaeota | rs56131665 | A | G | 0.177 | 0.039 | 5.30E-06 | 20.993 |
| phylum | Firmicutes | rs13411251 | T | G | 0.083 | 0.018 | 4.07E-06 | 20.992 |
| phylum | Proteobacteria | rs12610565 | G | A | 0.049 | 0.011 | 4.66E-06 | 20.989 |
| phylum | Proteobacteria | rs2347696 | T | C | 0.050 | 0.011 | 4.90E-06 | 20.987 |
| phylum | Actinobacteria | rs60433564 | A | C | 0.090 | 0.020 | 2.80E-06 | 20.978 |
| phylum | Actinobacteria | rs57543738 | T | C | 0.079 | 0.017 | 4.53E-06 | 20.978 |
| phylum | Actinobacteria | rs6430601 | G | T | 0.070 | 0.015 | 2.38E-06 | 20.976 |
| phylum | Cyanobacteria | rs1010755 | C | T | -0.122 | 0.027 | 4.25E-06 | 20.972 |
| phylum | Cyanobacteria | rs13072505 | A | G | -0.122 | 0.027 | 4.12E-06 | 20.972 |
| phylum | Actinobacteria | rs11897204 | T | C | 0.069 | 0.015 | 1.87E-06 | 20.953 |
| phylum | Actinobacteria | rs2874874 | A | C | 0.078 | 0.017 | 4.86E-06 | 20.953 |
| phylum | Actinobacteria | rs16831498 | G | C | 0.070 | 0.015 | 1.55E-06 | 20.949 |
| phylum | Bacteroidetes | rs71322865 | C | T | -0.060 | 0.013 | 3.13E-06 | 20.945 |
| phylum | Euryarchaeota | rs6776814 | C | T | -0.185 | 0.040 | 7.13E-06 | 20.945 |
| phylum | Proteobacteria | rs6509049 | T | G | 0.049 | 0.011 | 4.94E-06 | 20.945 |
| phylum | Cyanobacteria | rs2714558 | G | C | 0.093 | 0.020 | 8.24E-06 | 20.938 |
| phylum | Proteobacteria | rs2526712 | A | G | 0.049 | 0.011 | 4.78E-06 | 20.930 |
| phylum | Actinobacteria | rs3739022 | G | A | 0.078 | 0.017 | 4.33E-06 | 20.928 |
| phylum | Proteobacteria | rs7246662 | G | A | 0.049 | 0.011 | 4.83E-06 | 20.925 |
| phylum | Cyanobacteria | rs3752683 | T | C | 0.093 | 0.020 | 8.17E-06 | 20.922 |
| phylum | Proteobacteria | rs4494654 | C | T | 0.049 | 0.011 | 4.87E-06 | 20.919 |
| phylum | Cyanobacteria | rs615396 | A | G | -0.149 | 0.033 | 5.31E-06 | 20.917 |
| phylum | Proteobacteria | rs79445780 | A | G | -0.116 | 0.025 | 3.58E-06 | 20.913 |
| phylum | Proteobacteria | rs2883539 | G | C | 0.049 | 0.011 | 4.96E-06 | 20.911 |
| phylum | Proteobacteria | rs9789252 | A | C | 0.049 | 0.011 | 4.91E-06 | 20.897 |
| phylum | Bacteroidetes | rs79037 | G | A | -0.055 | 0.012 | 9.13E-06 | 20.892 |
| phylum | Firmicutes | rs28651047 | C | T | 0.083 | 0.018 | 4.34E-06 | 20.880 |
| phylum | Actinobacteria | rs2305248 | A | G | 0.068 | 0.015 | 1.79E-06 | 20.877 |
| phylum | Tenericutes | rs3768491 | G | A | -0.068 | 0.015 | 4.23E-06 | 20.875 |
| phylum | Firmicutes | rs58181266 | G | A | 0.083 | 0.018 | 3.71E-06 | 20.869 |
| phylum | Actinobacteria | rs6742013 | T | C | 0.069 | 0.015 | 1.51E-06 | 20.864 |
| phylum | Proteobacteria | rs7255373 | A | T | 0.049 | 0.011 | 5.25E-06 | 20.862 |
| phylum | Proteobacteria | rs7248256 | T | C | 0.049 | 0.011 | 5.04E-06 | 20.851 |
| phylum | Cyanobacteria | rs2714556 | T | C | 0.093 | 0.020 | 8.38E-06 | 20.846 |
| phylum | Tenericutes | rs3739327 | G | A | 0.066 | 0.014 | 5.75E-06 | 20.843 |
| phylum | Actinobacteria | rs6704561 | C | T | 0.054 | 0.012 | 6.26E-06 | 20.842 |
| phylum | Lentisphaerae | rs73113483 | A | T | -0.132 | 0.029 | 8.79E-06 | 20.833 |
| phylum | Bacteroidetes | rs73846124 | G | A | -0.061 | 0.013 | 3.48E-06 | 20.831 |
| phylum | Tenericutes | rs78169027 | G | A | -0.108 | 0.024 | 5.88E-06 | 20.824 |
| phylum | Proteobacteria | rs11083681 | G | T | 0.049 | 0.011 | 5.08E-06 | 20.813 |
| phylum | Tenericutes | rs10097590 | G | A | 0.067 | 0.015 | 8.52E-06 | 20.812 |
| phylum | Firmicutes | rs6436973 | C | G | 0.083 | 0.018 | 4.28E-06 | 20.807 |
| phylum | Euryarchaeota | rs62241832 | G | A | -0.188 | 0.041 | 8.24E-06 | 20.793 |
| phylum | Verrucomicrobia | rs6844547 | C | T | 0.105 | 0.023 | 4.48E-06 | 20.789 |
| phylum | Firmicutes | rs36111799 | T | C | -0.048 | 0.011 | 5.17E-06 | 20.789 |
| phylum | Tenericutes | rs10085973 | C | G | 0.065 | 0.014 | 6.68E-06 | 20.779 |
| phylum | Proteobacteria | rs11668739 | G | A | 0.049 | 0.011 | 5.37E-06 | 20.775 |
| phylum | Lentisphaerae | rs62481809 | C | G | 0.106 | 0.023 | 5.42E-06 | 20.771 |
| phylum | Proteobacteria | rs11667150 | G | A | 0.048 | 0.011 | 5.24E-06 | 20.770 |
| phylum | Proteobacteria | rs7246092 | T | C | 0.049 | 0.011 | 5.20E-06 | 20.769 |
| phylum | Verrucomicrobia | rs6845301 | G | A | 0.105 | 0.023 | 4.62E-06 | 20.751 |
| phylum | Tenericutes | rs12711448 | T | C | -0.068 | 0.015 | 4.51E-06 | 20.744 |
| phylum | Tenericutes | rs4282557 | C | T | 0.065 | 0.014 | 6.79E-06 | 20.736 |
| phylum | Tenericutes | rs10099240 | G | C | 0.065 | 0.014 | 6.96E-06 | 20.730 |
| phylum | Proteobacteria | rs8106280 | G | A | 0.049 | 0.011 | 5.48E-06 | 20.724 |
| phylum | Euryarchaeota | rs4848258 | A | T | -0.105 | 0.023 | 6.62E-06 | 20.723 |
| phylum | Actinobacteria | rs75211493 | A | G | 0.084 | 0.018 | 9.27E-06 | 20.714 |
| phylum | Cyanobacteria | rs2608307 | C | T | 0.092 | 0.020 | 9.09E-06 | 20.706 |
| phylum | Actinobacteria | rs35215526 | G | A | 0.081 | 0.018 | 7.34E-06 | 20.705 |
| phylum | Cyanobacteria | rs17039205 | A | G | -0.121 | 0.027 | 4.51E-06 | 20.704 |
| phylum | Verrucomicrobia | rs79293902 | C | T | 0.105 | 0.023 | 4.86E-06 | 20.699 |
| phylum | Tenericutes | rs638542 | A | G | -0.068 | 0.015 | 4.49E-06 | 20.696 |
| phylum | Bacteroidetes | rs34986288 | G | A | -0.062 | 0.014 | 3.38E-06 | 20.695 |
| phylum | Cyanobacteria | rs13072729 | T | C | -0.122 | 0.027 | 5.40E-06 | 20.692 |
| phylum | Proteobacteria | rs8102971 | C | T | 0.049 | 0.011 | 5.59E-06 | 20.689 |
| phylum | Euryarchaeota | rs10206171 | C | T | -0.112 | 0.025 | 6.58E-06 | 20.668 |
| phylum | Cyanobacteria | rs13095126 | T | C | -0.121 | 0.027 | 5.00E-06 | 20.658 |
| phylum | Cyanobacteria | rs545090 | T | C | -0.149 | 0.033 | 6.20E-06 | 20.657 |
| phylum | Proteobacteria | rs1058958 | A | G | 0.048 | 0.011 | 5.51E-06 | 20.653 |
| phylum | Actinobacteria | rs10932451 | A | G | 0.050 | 0.011 | 6.29E-06 | 20.653 |
| phylum | Actinobacteria | rs62287783 | G | A | 0.050 | 0.011 | 6.14E-06 | 20.652 |
| phylum | Euryarchaeota | rs62240388 | A | G | -0.187 | 0.041 | 7.62E-06 | 20.651 |
| phylum | Actinobacteria | rs6706466 | A | G | 0.068 | 0.015 | 2.13E-06 | 20.638 |
| phylum | Proteobacteria | rs12610793 | T | G | 0.048 | 0.011 | 5.64E-06 | 20.629 |
| phylum | Cyanobacteria | rs13072444 | G | A | -0.121 | 0.027 | 5.07E-06 | 20.628 |
| phylum | Verrucomicrobia | rs12411321 | T | C | 0.074 | 0.016 | 3.90E-06 | 20.623 |
| phylum | Firmicutes | rs72738886 | C | T | 0.086 | 0.019 | 7.68E-06 | 20.621 |
| phylum | Proteobacteria | rs11670071 | G | A | 0.048 | 0.011 | 5.68E-06 | 20.620 |
| phylum | Proteobacteria | rs1860455 | T | G | 0.048 | 0.011 | 5.76E-06 | 20.610 |
| phylum | Firmicutes | rs113037048 | C | T | 0.082 | 0.018 | 4.83E-06 | 20.606 |
| phylum | Proteobacteria | rs10750258 | C | A | -0.049 | 0.011 | 8.72E-06 | 20.598 |
| phylum | Firmicutes | rs11690370 | C | T | 0.083 | 0.018 | 4.83E-06 | 20.594 |
| phylum | Proteobacteria | rs2526716 | A | G | 0.048 | 0.011 | 5.71E-06 | 20.594 |
| phylum | Proteobacteria | rs7251480 | A | C | 0.048 | 0.011 | 5.77E-06 | 20.589 |
| phylum | Tenericutes | rs6867857 | C | A | 0.122 | 0.027 | 2.42E-06 | 20.589 |
| phylum | Firmicutes | rs7573799 | G | C | -0.070 | 0.015 | 8.05E-06 | 20.587 |
| phylum | Lentisphaerae | rs2546105 | T | A | 0.106 | 0.023 | 7.02E-06 | 20.586 |
| phylum | Cyanobacteria | rs17039208 | T | C | -0.121 | 0.027 | 4.79E-06 | 20.584 |
| phylum | Actinobacteria | rs17293519 | A | G | 0.068 | 0.015 | 2.30E-06 | 20.581 |
| phylum | Firmicutes | rs56199908 | C | T | -0.186 | 0.041 | 8.67E-06 | 20.572 |
| phylum | Euryarchaeota | rs62240380 | C | T | -0.184 | 0.041 | 7.63E-06 | 20.561 |
| phylum | Tenericutes | rs4970749 | A | G | -0.068 | 0.015 | 5.01E-06 | 20.550 |
| phylum | Actinobacteria | rs62160866 | A | G | 0.110 | 0.024 | 1.64E-06 | 20.539 |
| phylum | Proteobacteria | rs6509053 | A | T | 0.048 | 0.011 | 5.92E-06 | 20.539 |
| phylum | Euryarchaeota | rs11022995 | A | G | -0.104 | 0.023 | 7.73E-06 | 20.538 |
| phylum | Cyanobacteria | rs616178 | C | A | -0.148 | 0.033 | 6.44E-06 | 20.538 |
| phylum | Cyanobacteria | rs7148504 | T | G | 0.080 | 0.018 | 6.62E-06 | 20.537 |
| phylum | Bacteroidetes | rs6586324 | C | T | 0.048 | 0.011 | 7.37E-06 | 20.535 |
| phylum | Cyanobacteria | rs2714554 | C | T | 0.092 | 0.020 | 9.99E-06 | 20.534 |
| phylum | Lentisphaerae | rs72640280 | G | A | 0.220 | 0.049 | 5.19E-06 | 20.530 |
| phylum | Actinobacteria | rs11884903 | T | C | 0.069 | 0.015 | 2.00E-06 | 20.520 |
| phylum | Firmicutes | rs7247191 | C | T | -0.071 | 0.016 | 4.73E-06 | 20.515 |
| phylum | Firmicutes | rs7601602 | A | G | 0.082 | 0.018 | 5.56E-06 | 20.509 |
| phylum | Euryarchaeota | rs62241845 | T | A | -0.186 | 0.041 | 9.96E-06 | 20.489 |
| phylum | Euryarchaeota | rs62241844 | G | C | -0.186 | 0.041 | 9.96E-06 | 20.489 |
| phylum | Cyanobacteria | rs789050 | G | A | -0.097 | 0.021 | 8.74E-06 | 20.489 |
| phylum | Firmicutes | rs10164700 | G | A | 0.082 | 0.018 | 4.85E-06 | 20.479 |
| phylum | Proteobacteria | rs6707783 | T | C | 0.085 | 0.019 | 8.09E-06 | 20.470 |
| phylum | Euryarchaeota | rs56001610 | G | A | -0.186 | 0.041 | 9.96E-06 | 20.466 |
| phylum | Proteobacteria | rs11083680 | C | T | 0.048 | 0.011 | 6.16E-06 | 20.465 |
| phylum | Bacteroidetes | rs72706335 | C | T | -0.223 | 0.049 | 7.13E-06 | 20.464 |
| phylum | Tenericutes | rs4970840 | G | A | -0.068 | 0.015 | 5.85E-06 | 20.463 |
| phylum | Actinobacteria | rs62287786 | A | G | 0.050 | 0.011 | 6.73E-06 | 20.459 |
| phylum | Euryarchaeota | rs7635580 | G | A | -0.185 | 0.041 | 9.00E-06 | 20.459 |
| phylum | Actinobacteria | rs7673556 | T | C | 0.050 | 0.011 | 6.78E-06 | 20.457 |
| phylum | Proteobacteria | rs10775540 | A | G | 0.048 | 0.011 | 6.21E-06 | 20.442 |
| phylum | Cyanobacteria | rs13072359 | A | C | -0.121 | 0.027 | 5.69E-06 | 20.428 |
| phylum | Firmicutes | rs6711453 | A | G | 0.082 | 0.018 | 5.55E-06 | 20.426 |
| phylum | Proteobacteria | rs8105730 | T | C | 0.048 | 0.011 | 6.67E-06 | 20.412 |
| phylum | Lentisphaerae | rs35004924 | A | C | 0.106 | 0.024 | 4.10E-06 | 20.403 |
| phylum | Actinobacteria | rs10200912 | G | A | 0.050 | 0.011 | 7.17E-06 | 20.402 |
| phylum | Tenericutes | rs13020096 | T | C | 0.064 | 0.014 | 5.47E-06 | 20.399 |
| phylum | Euryarchaeota | rs73475126 | T | G | 0.199 | 0.044 | 6.89E-06 | 20.389 |
| phylum | Proteobacteria | rs1989369 | C | T | 0.048 | 0.011 | 6.44E-06 | 20.383 |
| phylum | Proteobacteria | rs141155 | A | C | -0.051 | 0.011 | 6.33E-06 | 20.383 |
| phylum | Bacteroidetes | rs2418267 | G | A | 0.061 | 0.014 | 6.05E-06 | 20.379 |
| phylum | Actinobacteria | rs80274970 | C | T | 0.084 | 0.019 | 8.53E-06 | 20.378 |
| phylum | Tenericutes | rs4885016 | C | T | -0.082 | 0.018 | 7.27E-06 | 20.363 |
| phylum | Euryarchaeota | rs45498998 | A | G | -0.132 | 0.029 | 5.32E-06 | 20.359 |
| phylum | Actinobacteria | rs62287778 | T | C | 0.049 | 0.011 | 7.01E-06 | 20.357 |
| phylum | Proteobacteria | rs11083683 | A | T | 0.048 | 0.011 | 6.55E-06 | 20.346 |
| phylum | Tenericutes | rs11678196 | A | G | 0.064 | 0.014 | 5.61E-06 | 20.344 |
| phylum | Firmicutes | rs11689387 | A | G | 0.082 | 0.018 | 5.46E-06 | 20.339 |
| phylum | Firmicutes | rs6814436 | C | T | 0.068 | 0.015 | 6.80E-06 | 20.334 |
| phylum | Actinobacteria | rs638382 | C | T | -0.050 | 0.011 | 5.03E-06 | 20.330 |
| phylum | Proteobacteria | rs8103079 | G | A | 0.048 | 0.011 | 6.80E-06 | 20.320 |
| phylum | Proteobacteria | rs8103457 | T | A | 0.048 | 0.011 | 6.93E-06 | 20.320 |
| phylum | Cyanobacteria | rs13088024 | T | A | -0.120 | 0.027 | 5.89E-06 | 20.306 |
| phylum | Proteobacteria | rs11715072 | A | G | -0.052 | 0.012 | 6.90E-06 | 20.303 |
| phylum | Proteobacteria | rs8102759 | C | G | 0.048 | 0.011 | 6.86E-06 | 20.301 |
| phylum | Tenericutes | rs3931560 | C | A | -0.067 | 0.015 | 5.79E-06 | 20.299 |
| phylum | Proteobacteria | rs10775538 | C | A | 0.048 | 0.011 | 6.66E-06 | 20.294 |
| phylum | Tenericutes | rs652651 | G | A | -0.067 | 0.015 | 5.50E-06 | 20.278 |
| phylum | Actinobacteria | rs6721838 | A | G | 0.068 | 0.015 | 2.28E-06 | 20.271 |
| phylum | Tenericutes | rs11102971 | T | C | -0.067 | 0.015 | 5.84E-06 | 20.254 |
| phylum | Proteobacteria | rs2526706 | G | A | 0.048 | 0.011 | 6.86E-06 | 20.241 |
| phylum | Actinobacteria | rs7594509 | G | T | 0.068 | 0.015 | 2.23E-06 | 20.239 |
| phylum | Cyanobacteria | rs12714902 | G | A | -0.120 | 0.027 | 6.08E-06 | 20.234 |
| phylum | Cyanobacteria | rs13060131 | C | T | -0.120 | 0.027 | 6.08E-06 | 20.231 |
| phylum | Euryarchaeota | rs74599069 | C | A | 0.218 | 0.049 | 8.72E-06 | 20.226 |
| phylum | Cyanobacteria | rs12714903 | C | T | -0.120 | 0.027 | 6.07E-06 | 20.224 |
| phylum | Tenericutes | rs17214486 | A | C | 0.061 | 0.014 | 6.61E-06 | 20.223 |
| phylum | Firmicutes | rs12554342 | C | G | -0.053 | 0.012 | 6.81E-06 | 20.221 |
| phylum | Tenericutes | rs10504256 | G | A | 0.063 | 0.014 | 8.96E-06 | 20.210 |
| phylum | Cyanobacteria | rs2585223 | C | T | 0.111 | 0.025 | 8.86E-06 | 20.206 |
| phylum | Actinobacteria | rs57829242 | A | G | 0.068 | 0.015 | 2.63E-06 | 20.198 |
| phylum | Cyanobacteria | rs12714900 | G | T | -0.120 | 0.027 | 6.17E-06 | 20.197 |
| phylum | Firmicutes | rs74905079 | A | G | 0.081 | 0.018 | 6.46E-06 | 20.194 |
| phylum | Proteobacteria | rs11083676 | G | A | 0.048 | 0.011 | 7.02E-06 | 20.189 |
| phylum | Proteobacteria | rs3890996 | G | T | -0.047 | 0.011 | 6.95E-06 | 20.183 |
| phylum | Proteobacteria | rs2526715 | A | T | 0.048 | 0.011 | 7.10E-06 | 20.183 |
| phylum | Tenericutes | rs10745352 | C | T | -0.067 | 0.015 | 6.13E-06 | 20.181 |
| phylum | Firmicutes | rs6752031 | C | T | 0.082 | 0.018 | 6.16E-06 | 20.170 |
| phylum | Actinobacteria | rs1551497 | T | C | 0.068 | 0.015 | 3.06E-06 | 20.158 |
| phylum | Actinobacteria | rs6496870 | C | T | 0.051 | 0.011 | 4.62E-06 | 20.142 |
| phylum | Actinobacteria | rs62287785 | G | A | 0.049 | 0.011 | 7.92E-06 | 20.141 |
| phylum | Proteobacteria | rs4803564 | G | C | 0.048 | 0.011 | 7.19E-06 | 20.135 |
| phylum | Proteobacteria | rs7246972 | T | C | 0.048 | 0.011 | 7.88E-06 | 20.131 |
| phylum | Cyanobacteria | rs12491989 | C | G | -0.120 | 0.027 | 6.87E-06 | 20.121 |
| phylum | Actinobacteria | rs72970286 | T | G | 0.086 | 0.019 | 6.03E-06 | 20.110 |
| phylum | Actinobacteria | rs4954633 | C | T | 0.087 | 0.019 | 3.46E-06 | 20.102 |
| phylum | Verrucomicrobia | rs61779206 | C | T | -0.073 | 0.016 | 8.82E-06 | 20.101 |
| phylum | Tenericutes | rs4970754 | G | A | -0.067 | 0.015 | 6.38E-06 | 20.100 |
| phylum | Euryarchaeota | rs58106718 | A | T | -0.182 | 0.041 | 9.41E-06 | 20.097 |
| phylum | Euryarchaeota | rs6064552 | C | T | -0.124 | 0.028 | 9.34E-06 | 20.095 |
| phylum | Tenericutes | rs10497163 | G | T | 0.069 | 0.015 | 5.94E-06 | 20.088 |
| phylum | Actinobacteria | rs11897217 | C | T | 0.067 | 0.015 | 2.35E-06 | 20.084 |
| phylum | Tenericutes | rs7843989 | T | G | 0.063 | 0.014 | 9.55E-06 | 20.084 |
| phylum | Bacteroidetes | rs12486971 | T | C | -0.061 | 0.014 | 5.25E-06 | 20.071 |
| phylum | Actinobacteria | rs7605229 | C | T | 0.068 | 0.015 | 2.62E-06 | 20.071 |
| phylum | Firmicutes | rs9973480 | A | C | 0.082 | 0.018 | 6.82E-06 | 20.071 |
| phylum | Actinobacteria | rs7581382 | T | C | -0.061 | 0.014 | 1.06E-06 | 20.060 |
| phylum | Proteobacteria | rs11668675 | A | G | 0.048 | 0.011 | 7.60E-06 | 20.060 |
| phylum | Proteobacteria | rs1962504 | G | C | 0.048 | 0.011 | 7.56E-06 | 20.059 |
| phylum | Tenericutes | rs7827910 | T | A | 0.063 | 0.014 | 9.57E-06 | 20.058 |
| phylum | Actinobacteria | rs6709763 | C | A | 0.067 | 0.015 | 3.33E-06 | 20.050 |
| phylum | Actinobacteria | rs12528285 | T | C | 0.081 | 0.018 | 5.69E-06 | 20.048 |
| phylum | Proteobacteria | rs4029165 | A | G | 0.048 | 0.011 | 7.65E-06 | 20.044 |
| phylum | Actinobacteria | rs11766971 | C | T | 0.048 | 0.011 | 9.40E-06 | 20.023 |
| phylum | Tenericutes | rs6664236 | A | C | -0.067 | 0.015 | 6.74E-06 | 20.014 |
| phylum | Proteobacteria | rs12467198 | T | C | 0.050 | 0.011 | 6.31E-06 | 20.013 |
| phylum | Proteobacteria | rs1962505 | G | T | 0.048 | 0.011 | 7.77E-06 | 20.008 |
| phylum | Proteobacteria | rs6509052 | T | A | 0.048 | 0.011 | 7.85E-06 | 20.004 |
| phylum | Proteobacteria | rs7255021 | G | A | 0.048 | 0.011 | 7.85E-06 | 19.997 |
| phylum | Proteobacteria | rs11673614 | G | T | 0.048 | 0.011 | 7.84E-06 | 19.989 |
| phylum | Euryarchaeota | rs9594528 | A | G | 0.195 | 0.044 | 9.37E-06 | 19.989 |
| phylum | Actinobacteria | rs28385412 | C | T | 0.049 | 0.011 | 8.54E-06 | 19.970 |
| phylum | Verrucomicrobia | rs2169719 | A | G | 0.064 | 0.014 | 7.63E-06 | 19.958 |
| phylum | Euryarchaeota | rs7015093 | A | G | -0.118 | 0.026 | 7.20E-06 | 19.957 |
| phylum | Proteobacteria | rs1036607 | C | T | 0.050 | 0.011 | 6.98E-06 | 19.954 |
| phylum | Firmicutes | rs4750583 | G | A | 0.062 | 0.014 | 5.79E-06 | 19.944 |
| phylum | Euryarchaeota | rs6508769 | C | T | 0.151 | 0.034 | 8.12E-06 | 19.941 |
| phylum | Lentisphaerae | rs1363428 | A | G | 0.104 | 0.023 | 9.95E-06 | 19.939 |
| phylum | Actinobacteria | rs4954570 | G | C | 0.055 | 0.012 | 5.85E-06 | 19.928 |
| phylum | Actinobacteria | rs28539813 | C | T | 0.049 | 0.011 | 8.77E-06 | 19.914 |
| phylum | Actinobacteria | rs6705610 | T | G | 0.067 | 0.015 | 2.68E-06 | 19.909 |
| phylum | Bacteroidetes | rs1808630 | C | T | 0.068 | 0.015 | 9.21E-06 | 19.906 |
| phylum | Cyanobacteria | rs13072044 | G | T | -0.122 | 0.027 | 6.89E-06 | 19.903 |
| phylum | Proteobacteria | rs11668790 | C | G | 0.047 | 0.011 | 8.27E-06 | 19.896 |
| phylum | Euryarchaeota | rs9594529 | A | G | 0.197 | 0.044 | 8.36E-06 | 19.895 |
| phylum | Proteobacteria | rs1989371 | C | A | 0.047 | 0.011 | 8.35E-06 | 19.894 |
| phylum | Actinobacteria | rs80124826 | C | T | -0.124 | 0.028 | 8.75E-06 | 19.876 |
| phylum | Tenericutes | rs2464826 | C | A | 0.094 | 0.021 | 8.40E-06 | 19.874 |
| phylum | Actinobacteria | rs4954242 | A | G | 0.068 | 0.015 | 3.66E-06 | 19.869 |
| phylum | Tenericutes | rs12711447 | T | C | -0.067 | 0.015 | 8.10E-06 | 19.866 |
| phylum | Tenericutes | rs7551176 | T | C | -0.067 | 0.015 | 8.14E-06 | 19.863 |
| phylum | Lentisphaerae | rs2031282 | G | A | 0.120 | 0.027 | 5.86E-06 | 19.850 |
| phylum | Verrucomicrobia | rs736124 | T | A | -0.073 | 0.016 | 8.72E-06 | 19.821 |
| phylum | Actinobacteria | rs4954226 | T | C | 0.068 | 0.015 | 3.22E-06 | 19.818 |
| phylum | Bacteroidetes | rs1365795 | T | C | 0.090 | 0.020 | 6.84E-06 | 19.816 |
| phylum | Tenericutes | rs6695482 | G | C | -0.066 | 0.015 | 7.47E-06 | 19.816 |
| phylum | Tenericutes | rs10858095 | C | G | -0.066 | 0.015 | 7.51E-06 | 19.802 |
| phylum | Actinobacteria | rs6756200 | T | C | 0.049 | 0.011 | 7.54E-06 | 19.800 |
| phylum | Firmicutes | rs7593057 | C | G | 0.081 | 0.018 | 7.38E-06 | 19.798 |
| phylum | Actinobacteria | rs11890844 | A | G | 0.067 | 0.015 | 3.68E-06 | 19.779 |
| phylum | Tenericutes | rs1465607 | C | T | -0.066 | 0.015 | 8.59E-06 | 19.775 |
| phylum | Actinobacteria | rs10871420 | A | G | 0.049 | 0.011 | 8.79E-06 | 19.771 |
| phylum | Actinobacteria | rs11936212 | G | C | 0.049 | 0.011 | 9.52E-06 | 19.767 |
| phylum | Actinobacteria | rs13402101 | T | A | 0.067 | 0.015 | 3.73E-06 | 19.767 |
| phylum | Actinobacteria | rs13399376 | A | G | 0.067 | 0.015 | 3.73E-06 | 19.767 |
| phylum | Tenericutes | rs10745356 | A | C | -0.066 | 0.015 | 8.64E-06 | 19.756 |
| phylum | Bacteroidetes | rs929878 | T | C | -0.054 | 0.012 | 6.51E-06 | 19.745 |
| phylum | Cyanobacteria | rs12555298 | A | G | 0.097 | 0.022 | 8.09E-06 | 19.730 |
| phylum | Verrucomicrobia | rs56161035 | C | T | 0.063 | 0.014 | 8.61E-06 | 19.727 |
| phylum | Actinobacteria | rs13192624 | C | T | -0.052 | 0.012 | 9.33E-06 | 19.720 |
| phylum | Actinobacteria | rs578384 | C | T | -0.049 | 0.011 | 6.68E-06 | 19.698 |
| phylum | Verrucomicrobia | rs77055143 | C | T | 0.063 | 0.014 | 8.39E-06 | 19.696 |
| phylum | Firmicutes | rs6815608 | C | T | 0.094 | 0.021 | 7.24E-06 | 19.693 |
| phylum | Proteobacteria | rs11083675 | C | T | 0.047 | 0.011 | 9.18E-06 | 19.684 |
| phylum | Verrucomicrobia | rs11252899 | G | A | 0.074 | 0.017 | 7.12E-06 | 19.680 |
| phylum | Firmicutes | rs9730854 | A | G | -0.048 | 0.011 | 9.26E-06 | 19.679 |
| phylum | Proteobacteria | rs1439134 | T | C | 0.049 | 0.011 | 7.81E-06 | 19.662 |
| phylum | Firmicutes | rs2547978 | G | A | 0.047 | 0.011 | 8.57E-06 | 19.652 |
| phylum | Actinobacteria | rs2322445 | T | A | 0.067 | 0.015 | 3.21E-06 | 19.651 |
| phylum | Tenericutes | rs12480021 | G | A | -0.111 | 0.025 | 9.55E-06 | 19.640 |
| phylum | Euryarchaeota | rs45567540 | G | C | -0.130 | 0.029 | 7.72E-06 | 19.616 |
| phylum | Firmicutes | rs2220596 | A | G | -0.070 | 0.016 | 6.12E-06 | 19.605 |
| phylum | Tenericutes | rs10858094 | C | T | -0.066 | 0.015 | 8.13E-06 | 19.603 |
| phylum | Cyanobacteria | rs61972390 | C | T | 0.107 | 0.024 | 9.11E-06 | 19.600 |
| phylum | Firmicutes | rs8085381 | A | G | 0.065 | 0.015 | 8.67E-06 | 19.597 |
| phylum | Proteobacteria | rs10853761 | C | T | 0.047 | 0.011 | 9.72E-06 | 19.575 |
| phylum | Tenericutes | rs74338080 | C | G | -0.110 | 0.025 | 9.95E-06 | 19.574 |
| phylum | Tenericutes | rs417270 | C | T | -0.066 | 0.015 | 8.60E-06 | 19.573 |
| phylum | Bacteroidetes | rs73975618 | A | G | -0.189 | 0.043 | 6.77E-06 | 19.552 |
| phylum | Actinobacteria | rs56369660 | A | G | 0.066 | 0.015 | 4.34E-06 | 19.544 |
| phylum | Tenericutes | rs367586 | A | C | -0.066 | 0.015 | 8.77E-06 | 19.543 |
| phylum | Actinobacteria | rs4312299 | G | C | 0.049 | 0.011 | 9.21E-06 | 19.543 |
| phylum | Firmicutes | rs10933355 | G | A | 0.080 | 0.018 | 8.50E-06 | 19.522 |
| phylum | Firmicutes | rs1568247 | G | A | 0.080 | 0.018 | 8.39E-06 | 19.518 |
| phylum | Tenericutes | rs13025367 | C | G | 0.063 | 0.014 | 8.53E-06 | 19.512 |
| phylum | Euryarchaeota | rs12155661 | C | T | -0.117 | 0.026 | 9.16E-06 | 19.502 |
| phylum | Actinobacteria | rs72972104 | G | A | 0.086 | 0.019 | 4.70E-06 | 19.501 |
| phylum | Actinobacteria | rs12474050 | C | T | -0.049 | 0.011 | 9.93E-06 | 19.493 |
| phylum | Actinobacteria | rs16831264 | G | C | 0.066 | 0.015 | 4.58E-06 | 19.487 |
| phylum | Tenericutes | rs443345 | G | A | -0.066 | 0.015 | 9.11E-06 | 19.464 |
| phylum | Tenericutes | rs67450615 | T | G | 0.072 | 0.016 | 9.40E-06 | 19.456 |
| phylum | Verrucomicrobia | rs2895059 | C | T | 0.072 | 0.016 | 7.66E-06 | 19.449 |
| phylum | Tenericutes | rs10776807 | A | G | -0.066 | 0.015 | 9.12E-06 | 19.444 |
| phylum | Tenericutes | rs13023735 | A | G | 0.063 | 0.014 | 8.86E-06 | 19.441 |
| phylum | Actinobacteria | rs62170081 | A | G | 0.086 | 0.020 | 6.51E-06 | 19.437 |
| phylum | Actinobacteria | rs34284163 | T | A | -0.060 | 0.014 | 8.82E-06 | 19.435 |
| phylum | Verrucomicrobia | rs3995795 | T | C | 0.061 | 0.014 | 9.72E-06 | 19.431 |
| phylum | Cyanobacteria | rs7644367 | G | A | -0.118 | 0.027 | 8.98E-06 | 19.424 |
| phylum | Bacteroidetes | rs2138406 | G | T | 0.058 | 0.013 | 8.38E-06 | 19.411 |
| phylum | Tenericutes | rs12136140 | A | G | -0.066 | 0.015 | 9.28E-06 | 19.385 |
| phylum | Actinobacteria | rs6737635 | G | T | 0.066 | 0.015 | 4.90E-06 | 19.373 |
| phylum | Tenericutes | rs11102969 | A | G | -0.066 | 0.015 | 9.44E-06 | 19.357 |
| phylum | Firmicutes | rs12983973 | G | A | -0.069 | 0.016 | 7.95E-06 | 19.347 |
| phylum | Tenericutes | rs10858092 | T | C | -0.065 | 0.015 | 9.40E-06 | 19.298 |
| phylum | Proteobacteria | rs4340523 | T | C | 0.049 | 0.011 | 9.52E-06 | 19.284 |
| phylum | Bacteroidetes | rs62531359 | G | T | 0.066 | 0.015 | 8.42E-06 | 19.260 |
| phylum | Firmicutes | rs3931771 | A | G | -0.069 | 0.016 | 9.51E-06 | 19.248 |
| phylum | Proteobacteria | rs74757828 | T | A | 0.095 | 0.022 | 9.25E-06 | 19.232 |
| phylum | Actinobacteria | rs6745996 | A | G | 0.069 | 0.016 | 4.71E-06 | 19.220 |
| phylum | Bacteroidetes | rs7999780 | A | G | 0.054 | 0.012 | 9.47E-06 | 19.200 |
| phylum | Tenericutes | rs12566890 | G | T | -0.101 | 0.023 | 3.65E-06 | 19.176 |
| phylum | Actinobacteria | rs6743026 | C | T | 0.059 | 0.013 | 9.88E-06 | 19.171 |
| phylum | Firmicutes | rs4933026 | A | T | -0.069 | 0.016 | 8.43E-06 | 19.166 |
| phylum | Firmicutes | rs8112095 | G | A | -0.069 | 0.016 | 9.75E-06 | 19.162 |
| phylum | Actinobacteria | rs4953938 | G | A | 0.047 | 0.011 | 9.30E-06 | 19.141 |
| phylum | Actinobacteria | rs6727889 | C | T | 0.067 | 0.015 | 5.01E-06 | 19.113 |
| phylum | Actinobacteria | rs4954213 | G | T | 0.066 | 0.015 | 5.87E-06 | 19.095 |
| phylum | Actinobacteria | rs13408096 | G | T | 0.066 | 0.015 | 5.69E-06 | 19.094 |
| phylum | Cyanobacteria | rs9832125 | C | T | -0.113 | 0.026 | 8.25E-06 | 19.025 |
| phylum | Tenericutes | rs28537087 | A | G | 0.082 | 0.019 | 8.07E-06 | 19.002 |
| phylum | Bacteroidetes | rs34380401 | T | C | -0.059 | 0.014 | 9.59E-06 | 18.985 |
| phylum | Proteobacteria | rs4340090 | T | C | -0.067 | 0.015 | 9.99E-06 | 18.975 |
| phylum | Firmicutes | rs58880850 | A | C | -0.069 | 0.016 | 8.90E-06 | 18.919 |
| phylum | Actinobacteria | rs4954212 | A | G | 0.065 | 0.015 | 7.41E-06 | 18.818 |
| phylum | Cyanobacteria | rs13084677 | A | T | -0.112 | 0.026 | 9.44E-06 | 18.747 |
| phylum | Actinobacteria | rs9920349 | G | A | 0.049 | 0.011 | 8.57E-06 | 18.743 |
| phylum | Actinobacteria | rs520449 | T | C | -0.048 | 0.011 | 8.74E-06 | 18.716 |
| phylum | Firmicutes | rs35393748 | T | A | -0.068 | 0.016 | 9.97E-06 | 18.699 |
| phylum | Actinobacteria | rs10928529 | G | A | 0.065 | 0.015 | 7.52E-06 | 18.688 |
| phylum | Actinobacteria | rs3806502 | C | T | 0.066 | 0.015 | 5.57E-06 | 18.684 |
| phylum | Firmicutes | rs1963579 | T | A | -0.068 | 0.016 | 9.68E-06 | 18.650 |
| phylum | Firmicutes | rs1984783 | A | G | -0.068 | 0.016 | 9.84E-06 | 18.620 |
| phylum | Actinobacteria | rs11892750 | G | A | 0.065 | 0.015 | 7.84E-06 | 18.589 |
| phylum | Actinobacteria | rs72847650 | C | A | -0.049 | 0.011 | 8.74E-06 | 18.509 |
| phylum | Actinobacteria | rs7597802 | A | T | 0.065 | 0.015 | 8.49E-06 | 18.496 |
| phylum | Actinobacteria | rs11889843 | G | A | 0.065 | 0.015 | 8.37E-06 | 18.477 |
| phylum | Verrucomicrobia | rs12512971 | C | A | 0.171 | 0.040 | 9.81E-06 | 18.467 |
| phylum | Actinobacteria | rs11900832 | C | T | 0.064 | 0.015 | 9.05E-06 | 18.294 |
| phylum | Actinobacteria | rs4954555 | T | C | 0.091 | 0.021 | 9.64E-06 | 18.269 |
| phylum | Proteobacteria | rs11126162 | C | T | -0.077 | 0.019 | 9.26E-06 | 16.971 |

**TableS2. Correlation disease or trait of SNPs in significant IVW results retrievaled by PhenoScanner.**

| **SNP** | **a1** | **a2** | **Trait** |
| --- | --- | --- | --- |
| rs10108398 | A | G | Cholelithiasis |
| rs10108398 | A | G | Impedance of arm left |
| rs10108398 | A | G | Impedance of arm right |
| rs10108398 | A | G | Impedance of whole body |
| rs10108398 | A | G | LDL cholesterol |
| rs10108398 | A | G | Low density lipoprotein |
| rs10108398 | A | G | Low density lipoprotein |
| rs10108398 | A | G | Total cholesterol |
| rs10202904 | G | T | Forced vital capacity |
| rs10217435 | C | T | Impedance of arm right |
| rs10217435 | C | T | Impedance of whole body |
| rs10424197 | A | G | Body mass index |
| rs10490280 | T | C | Impedance of arm left |
| rs10490280 | T | C | Impedance of arm right |
| rs10885477 | C | T | Treatment with cilest tablet |
| rs10950694 | C | T | Height |
| rs10950694 | C | T | Sitting height |
| rs11018566 | A | G | Postpartum haemorrhage |
| rs111810795 | C | T | Mean corpuscular hemoglobin |
| rs11547158 | A | G | Basophil percentage of granulocytes |
| rs11547158 | A | G | Basophil percentage of white cells |
| rs11547158 | A | G | Neutrophil percentage of granulocytes |
| rs115602804 | A | G | Other disorders of eustachian tube |
| rs11613919 | G | T | Neuroticism |
| rs11613919 | G | T | Sensitivity or hurt feelings |
| rs11890098 | A | G | Fluid intelligence score |
| rs11979110 | C | T | Arm fat mass left |
| rs11979110 | C | T | Arm fat mass right |
| rs11979110 | C | T | Arm fat percentage left |
| rs11979110 | C | T | Arm fat percentage right |
| rs11979110 | C | T | Body fat percentage |
| rs11979110 | C | T | Diabetes diagnosed by doctor |
| rs11979110 | C | T | HDL cholesterol |
| rs11979110 | C | T | High density lipoprotein |
| rs11979110 | C | T | Hip circumference |
| rs11979110 | C | T | Hip circumference adjusted for BMI |
| rs11979110 | C | T | Hip circumference in females |
| rs11979110 | C | T | Impedance of arm left |
| rs11979110 | C | T | Impedance of arm right |
| rs11979110 | C | T | Impedance of whole body |
| rs11979110 | C | T | Leg fat mass left |
| rs11979110 | C | T | Leg fat mass right |
| rs11979110 | C | T | Leg fat percentage left |
| rs11979110 | C | T | Leg fat percentage right |
| rs11979110 | C | T | No treatment with medication for cholesterol, blood pressure, diabetes, or take exogenous hormones |
| rs11979110 | C | T | Self-reported hypertension |
| rs11979110 | C | T | Treatment with blood pressure medication |
| rs11979110 | C | T | Treatment with cholesterol lowering medication |
| rs11979110 | C | T | Treatment with simvastatin |
| rs11979110 | C | T | Triglycerides |
| rs11979110 | C | T | Trunk fat mass |
| rs11979110 | C | T | Trunk fat percentage |
| rs11979110 | C | T | Type II diabetes |
| rs11979110 | C | T | Vascular or heart problems diagnosed by doctor: high blood pressure |
| rs11979110 | C | T | Vascular or heart problems diagnosed by doctor: none of the above |
| rs11979110 | C | T | Whole body fat mass |
| rs12050685 | A | G | Body fat percentage |
| rs12050685 | A | G | Heart rate |
| rs12050685 | A | G | Heart rate variability RMSSD |
| rs12050685 | A | G | Heart rate variability SDNN |
| rs12050685 | A | G | Leg fat percentage left |
| rs12050685 | A | G | Leg fat percentage right |
| rs12050685 | A | G | Pulse rate |
| rs1205443 | A | G | Height |
| rs1205443 | A | G | Leg fat-free mass right |
| rs1205443 | A | G | Leg predicted mass left |
| rs1205443 | A | G | Leg predicted mass right |
| rs1205443 | A | G | Pulse rate |
| rs1205443 | A | G | Pulse wave reflection index |
| rs1205443 | A | G | Sitting height |
| rs1205443 | A | G | Treatment with pramipexole |
| rs1205443 | A | G | Trunk fat-free mass |
| rs1205443 | A | G | Trunk predicted mass |
| rs1205443 | A | G | Whole body fat-free mass |
| rs1205443 | A | G | Whole body water mass |
| rs12288512 | A | G | Arm fat mass left |
| rs12288512 | A | G | Arm fat mass right |
| rs12288512 | A | G | Arm fat percentage left |
| rs12288512 | A | G | Arm fat percentage right |
| rs12288512 | A | G | Arm fat-free mass left |
| rs12288512 | A | G | Arm fat-free mass right |
| rs12288512 | A | G | Arm predicted mass left |
| rs12288512 | A | G | Arm predicted mass right |
| rs12288512 | A | G | Basal metabolic rate |
| rs12288512 | A | G | Body fat percentage |
| rs12288512 | A | G | Body mass index |
| rs12288512 | A | G | Body mass index adjusted for physical activity |
| rs12288512 | A | G | Body mass index adjusted for smoking in females |
| rs12288512 | A | G | Body mass index adjusted for smoking in males |
| rs12288512 | A | G | Body mass index ajusted for physical activity in females |
| rs12288512 | A | G | Body mass index in female non-smokers |
| rs12288512 | A | G | Body mass index in females |
| rs12288512 | A | G | Body mass index in females greater than 50 years of age |
| rs12288512 | A | G | Body mass index in males |
| rs12288512 | A | G | Body mass index in males greater than 50 years of age |
| rs12288512 | A | G | Body mass index in physically active indivdiuals |
| rs12288512 | A | G | Body mass index in physically active indivdiuals |
| rs12288512 | A | G | Childhood BMI |
| rs12288512 | A | G | Coffee consumption cups per day |
| rs12288512 | A | G | Comparative body size at age 10 |
| rs12288512 | A | G | Current tobacco smoking |
| rs12288512 | A | G | Diverticular disease of intestine |
| rs12288512 | A | G | Frequency of stair climbing in last 4 weeks |
| rs12288512 | A | G | Hip circumference |
| rs12288512 | A | G | Hip circumference in females |
| rs12288512 | A | G | Impedance of arm left |
| rs12288512 | A | G | Impedance of arm right |
| rs12288512 | A | G | Impedance of leg left |
| rs12288512 | A | G | Impedance of leg right |
| rs12288512 | A | G | Impedance of whole body |
| rs12288512 | A | G | Leg fat mass left |
| rs12288512 | A | G | Leg fat mass right |
| rs12288512 | A | G | Leg fat percentage left |
| rs12288512 | A | G | Leg fat percentage right |
| rs12288512 | A | G | Leg fat-free mass left |
| rs12288512 | A | G | Leg fat-free mass right |
| rs12288512 | A | G | Leg predicted mass left |
| rs12288512 | A | G | Leg predicted mass right |
| rs12288512 | A | G | Nervous feelings |
| rs12288512 | A | G | Obesity class 1 |
| rs12288512 | A | G | Trunk fat mass |
| rs12288512 | A | G | Trunk fat-free mass |
| rs12288512 | A | G | Trunk predicted mass |
| rs12288512 | A | G | Waist circumference |
| rs12288512 | A | G | Waist circumference in females |
| rs12288512 | A | G | Weight |
| rs12288512 | A | G | Whole body fat mass |
| rs12288512 | A | G | Whole body fat-free mass |
| rs12288512 | A | G | Whole body water mass |
| rs12288512 | A | G | Worrier or anxious feelings |
| rs12288512 | A | G | Worry too long after embarrassment |
| rs12341505 | A | G | Treatment with berocca effervescent tablet |
| rs12377846 | A | C | Height |
| rs12566975 | C | T | Hand grip strength left |
| rs12668619 | A | G | Total cholesterol |
| rs12986312 | G | T | High grade serous ovarian cancer |
| rs12986312 | G | T | Serous invasive ovarian cancer |
| rs1340392 | A | G | Mouth or teeth dental problems: dentures |
| rs13409132 | A | G | Nasal polyp |
| rs1397793 | G | A | Birth weight |
| rs1442060 | A | G | Treatment with glyceryl trinitrate |
| rs146541147 | A | G | Hodgkins disease |
| rs1467258 | A | G | Granulocyte count |
| rs1467258 | A | G | Myeloid white cell count |
| rs1467258 | A | G | Neutrophil count |
| rs1467258 | A | G | Sum basophil neutrophil counts |
| rs1467258 | A | G | Sum neutrophil eosinophil counts |
| rs1467258 | A | G | White blood cell count |
| rs147968 | C | T | Basophil percentage of granulocytes |
| rs147968 | C | T | Granulocyte count |
| rs147968 | C | T | Granulocyte percentage of myeloid white cells |
| rs147968 | C | T | Monocyte count |
| rs147968 | C | T | Monocyte percentage of white cells |
| rs147968 | C | T | Neutrophil count |
| rs147968 | C | T | Neutrophil percentage of white cells |
| rs147968 | C | T | Sum basophil neutrophil counts |
| rs147968 | C | T | Sum neutrophil eosinophil counts |
| rs1492620 | C | T | Urticaria |
| rs1511453 | G | A | Fasting blood glucose change in response to hydrochlorothiazide |
| rs1511453 | G | A | Thiazide induced adverse metabolic effects in hypertensive patients |
| rs1520207 | T | C | Hair or balding pattern: pattern 3 |
| rs1520207 | T | C | Hair or balding pattern: pattern 4 |
| rs1530559 | A | G | Arm fat mass left |
| rs1530559 | A | G | Arm fat mass right |
| rs1530559 | A | G | Arm fat percentage left |
| rs1530559 | A | G | Arm fat percentage right |
| rs1530559 | A | G | Body fat percentage |
| rs1530559 | A | G | Body mass index |
| rs1530559 | A | G | Diastolic blood pressure |
| rs1530559 | A | G | Fasting insulin |
| rs1530559 | A | G | Forced expiratory volume in 1-second |
| rs1530559 | A | G | Forced vital capacity |
| rs1530559 | A | G | Hand grip strength left |
| rs1530559 | A | G | Hand grip strength right |
| rs1530559 | A | G | Hip circumference |
| rs1530559 | A | G | Leg fat mass left |
| rs1530559 | A | G | Leg fat mass right |
| rs1530559 | A | G | Leg fat percentage left |
| rs1530559 | A | G | Leg fat percentage right |
| rs1530559 | A | G | log Fasting insulin |
| rs1530559 | A | G | Low density lipoprotein |
| rs1530559 | A | G | Parkinsons disease |
| rs1530559 | A | G | Systolic blood pressure |
| rs1530559 | A | G | Total cholesterol |
| rs1530559 | A | G | Trunk fat mass |
| rs1530559 | A | G | Trunk fat percentage |
| rs1530559 | A | G | Usual walking pace |
| rs1530559 | A | G | Waist circumference |
| rs1530559 | A | G | Weight |
| rs1530559 | A | G | Whole body fat mass |
| rs1582238 | T | C | Hair or balding pattern: pattern 4 |
| rs17379710 | C | T | Self-reported hypothyroidism or myxoedema |
| rs17379710 | C | T | Treatment with levothyroxine sodium |
| rs182549 | C | T | 15 anhydroglucitol levels |
| rs182549 | C | T | Arm fat mass left |
| rs182549 | C | T | Arm fat mass right |
| rs182549 | C | T | Arm fat percentage left |
| rs182549 | C | T | Arm fat percentage right |
| rs182549 | C | T | Body fat percentage |
| rs182549 | C | T | Body mass index |
| rs182549 | C | T | Forced expiratory volume in 1-second |
| rs182549 | C | T | Forced expiratory volume in 1-second, best measure |
| rs182549 | C | T | Forced vital capacity |
| rs182549 | C | T | Forced vital capacity, best measure |
| rs182549 | C | T | Hand grip strength left |
| rs182549 | C | T | Leg fat mass left |
| rs182549 | C | T | Leg fat mass right |
| rs182549 | C | T | Leg fat percentage left |
| rs182549 | C | T | Leg fat percentage right |
| rs182549 | C | T | Parkinsons disease |
| rs182549 | C | T | Potassium in urine |
| rs182549 | C | T | Sleep duration |
| rs182549 | C | T | Time spent watching television |
| rs182549 | C | T | Trunk fat mass |
| rs182549 | C | T | Trunk fat percentage |
| rs182549 | C | T | Weight |
| rs182549 | C | T | Whole body fat mass |
| rs2113833 | T | C | Self-reported thalassaemia |
| rs2155352 | A | G | Height |
| rs2160994 | C | T | Comparative body size at age 10 |
| rs2160994 | C | T | Diastolic blood pressure |
| rs2160994 | C | T | Hair or balding pattern: pattern 4 |
| rs2160994 | C | T | Height |
| rs2160994 | C | T | Impedance of arm left |
| rs2160994 | C | T | Impedance of arm right |
| rs2160994 | C | T | Impedance of leg left |
| rs2160994 | C | T | Impedance of leg right |
| rs2160994 | C | T | Impedance of whole body |
| rs2160994 | C | T | Mean corpuscular volume |
| rs2160994 | C | T | Platelet count |
| rs2160994 | C | T | Plateletcrit |
| rs2160994 | C | T | Sitting height |
| rs2300774 | G | A | High light scatter percentage of red cells |
| rs2300774 | G | A | Mean corpuscular hemoglobin |
| rs2300774 | G | A | Mean corpuscular hemoglobin concentration |
| rs2300774 | G | A | Mean corpuscular hemoglobin concentration |
| rs2300774 | G | A | Mean corpuscular volume |
| rs2300774 | G | A | Platelet count |
| rs2300774 | G | A | Plateletcrit |
| rs2300774 | G | A | Red blood cell count |
| rs2300774 | G | A | Red cell distribution width |
| rs2300774 | G | A | Reticulocyte fraction of red cells |
| rs2321387 | A | G | Mean time to correctly identify matches |
| rs2321387 | A | G | Qualifications: college or university degree |
| rs2321387 | A | G | Years of educational attainment |
| rs2368224 | G | T | Treatment with lisinopril+hydrochlorothiazide 10mg or 12.5mg tablet |
| rs240104 | T | C | Treatment with aprovel 75mg tablet |
| rs2442778 | A | G | Birth weight of first child |
| rs2442778 | A | G | Comparative height size at age 10 |
| rs2442778 | A | G | Height |
| rs2442778 | A | G | Sitting height |
| rs2523124 | T | C | Nervous feelings |
| rs2715439 | T | C | Arm fat mass left |
| rs2715439 | T | C | Arm fat mass right |
| rs2715439 | T | C | Arm fat-free mass left |
| rs2715439 | T | C | Basal metabolic rate |
| rs2715439 | T | C | Comparative height size at age 10 |
| rs2715439 | T | C | Height |
| rs2715439 | T | C | Hip circumference |
| rs2715439 | T | C | Impedance of leg left |
| rs2715439 | T | C | Impedance of leg right |
| rs2715439 | T | C | Leg fat-free mass left |
| rs2715439 | T | C | Leg fat-free mass right |
| rs2715439 | T | C | Leg predicted mass left |
| rs2715439 | T | C | Leg predicted mass right |
| rs2715439 | T | C | Sitting height |
| rs2715439 | T | C | Trunk fat mass |
| rs2715439 | T | C | Trunk fat-free mass |
| rs2715439 | T | C | Trunk predicted mass |
| rs2715439 | T | C | Weight |
| rs2715439 | T | C | Whole body fat mass |
| rs2715439 | T | C | Whole body fat-free mass |
| rs2715439 | T | C | Whole body water mass |
| rs2791343 | C | T | Irritability |
| rs2791343 | C | T | Mean platelet volume |
| rs281379 | A | G | Alcohol intake frequency |
| rs281379 | A | G | Alcohol intake versus 10 years previously |
| rs281379 | A | G | Alkaline phosphatase |
| rs281379 | A | G | Bring up phlegm or sputum/mucus on most days |
| rs281379 | A | G | Cholelithiasis |
| rs281379 | A | G | Comparative body size at age 10 |
| rs281379 | A | G | Comparative height size at age 10 |
| rs281379 | A | G | Crohns disease |
| rs281379 | A | G | Height pubertal |
| rs281379 | A | G | Height SDS for males aged 12 years |
| rs281379 | A | G | LDL cholesterol |
| rs281379 | A | G | Low density lipoprotein |
| rs281379 | A | G | Low density lipoprotein |
| rs281379 | A | G | Mean platelet volume |
| rs281379 | A | G | Medication for cholesterol, blood pressure or diabetes: cholesterol lowering medication |
| rs281379 | A | G | Medication for cholesterol, blood pressure or diabetes: none of the above |
| rs281379 | A | G | Mouth or teeth dental problems: dentures |
| rs281379 | A | G | Mouth or teeth dental problems: none of the above |
| rs281379 | A | G | No treatment with medication for cholesterol, blood pressure, diabetes, or take exogenous hormones |
| rs281379 | A | G | Number of self-reported non-cancer illnesses |
| rs281379 | A | G | Peak expiratory flow |
| rs281379 | A | G | Psoriasis |
| rs281379 | A | G | Psoriasis and Crohns disease combined |
| rs281379 | A | G | Pubertal anthropometrics |
| rs281379 | A | G | Self-reported cholelithiasis or gall stones |
| rs281379 | A | G | Self-reported high cholesterol |
| rs281379 | A | G | Self-reported hypertension |
| rs281379 | A | G | Sitting height |
| rs281379 | A | G | Sodium in urine |
| rs281379 | A | G | Total cholesterol |
| rs281379 | A | G | Treatment with cholesterol lowering medication |
| rs281379 | A | G | Treatment with simvastatin |
| rs281379 | A | G | Vascular or heart problems diagnosed by doctor: high blood pressure |
| rs281379 | A | G | Vascular or heart problems diagnosed by doctor: none of the above |
| rs2833828 | A | G | Impedance of leg left |
| rs2833828 | A | G | Impedance of leg right |
| rs2838334 | A | G | Coronary artery disease |
| rs2838334 | A | G | Mean corpuscular hemoglobin |
| rs2838334 | A | G | Mean corpuscular volume |
| rs2838334 | A | G | Red blood cell count |
| rs2889192 | G | T | mDC:%32+; mDC subset (CD32+) |
| rs2943022 | T | C | Nap during day |
| rs2952251 | G | A | Eosinophil percentage of white cells |
| rs2952251 | G | A | Irritability |
| rs2952251 | G | A | Miserableness |
| rs2952251 | G | A | Mood swings |
| rs2952251 | G | A | Neuroticism |
| rs2952251 | G | A | Neuroticism score |
| rs2952251 | G | A | Past tobacco smoking |
| rs2952251 | G | A | Sensitivity or hurt feelings |
| rs2952251 | G | A | Smoking status: previous |
| rs2952251 | G | A | Worrier or anxious feelings |
| rs3127230 | C | T | Hand grip strength left |
| rs3127230 | C | T | Hand grip strength right |
| rs3127230 | C | T | Height |
| rs3127230 | C | T | Sitting height |
| rs34739816 | G | T | Abnormal involuntary movements |
| rs35233670 | C | T | Hair or balding pattern: pattern 2 |
| rs35233670 | C | T | Hair or balding pattern: pattern 4 |
| rs35344081 | A | G | Arm fat-free mass left |
| rs35344081 | A | G | Arm fat-free mass right |
| rs35344081 | A | G | Arm predicted mass right |
| rs35344081 | A | G | Comparative height size at age 10 |
| rs35344081 | A | G | Height |
| rs35344081 | A | G | Sitting height |
| rs35344081 | A | G | Trunk fat-free mass |
| rs35344081 | A | G | Trunk predicted mass |
| rs35961441 | A | C | Treatment with arcoxia 90mg tablet |
| rs3758087 | T | C | log eGFR creatinine |
| rs3758087 | T | C | log eGFR creatinine in non diabetics |
| rs3758087 | T | C | Serum creatinine |
| rs3768491 | G | A | Arm fat-free mass right |
| rs3768491 | G | A | Arm predicted mass right |
| rs3768491 | G | A | Basal metabolic rate |
| rs3768491 | G | A | Fluid intelligence score |
| rs3768491 | G | A | Impedance of arm left |
| rs3768491 | G | A | Impedance of arm right |
| rs3768491 | G | A | Impedance of leg left |
| rs3768491 | G | A | Impedance of leg right |
| rs3768491 | G | A | Impedance of whole body |
| rs3768491 | G | A | Job involves heavy manual or physical work |
| rs3768491 | G | A | Leg fat-free mass left |
| rs3768491 | G | A | Leg fat-free mass right |
| rs3768491 | G | A | Leg predicted mass left |
| rs3768491 | G | A | Leg predicted mass right |
| rs3768491 | G | A | Qualifications: college or university degree |
| rs3768491 | G | A | Qualifications: none |
| rs3768491 | G | A | Time spent using computer |
| rs3768491 | G | A | Time spent watching television |
| rs3768491 | G | A | Trunk fat-free mass |
| rs3768491 | G | A | Trunk predicted mass |
| rs3768491 | G | A | Whole body fat-free mass |
| rs3768491 | G | A | Whole body water mass |
| rs3821998 | A | C | Eosinophil count |
| rs3821998 | A | C | Eosinophil percentage of granulocytes |
| rs3821998 | A | C | Eosinophil percentage of white cells |
| rs3821998 | A | C | Neutrophil percentage of granulocytes |
| rs3821998 | A | C | Sum eosinophil basophil counts |
| rs4396289 | C | T | Comparative height size at age 10 |
| rs4396289 | C | T | Hip pain for 3+ months |
| rs4396289 | C | T | Trunk fat-free mass |
| rs4396289 | C | T | Trunk predicted mass |
| rs4428215 | A | G | Height |
| rs4461038 | A | G | Hematocrit |
| rs4461038 | A | G | Hemoglobin concentration |
| rs4461038 | A | G | Mean corpuscular hemoglobin concentration |
| rs4461038 | A | G | Platelet count |
| rs4461038 | A | G | Plateletcrit |
| rs4461038 | A | G | Red blood cell count |
| rs4461038 | A | G | Red cell distribution width |
| rs4461038 | A | G | Reticulocyte count |
| rs4461038 | A | G | Reticulocyte fraction of red cells |
| rs4493272 | C | T | Heel bone mineral density |
| rs45497800 | T | C | Current tobacco smoking |
| rs45497800 | T | C | Maternal smoking around birth |
| rs45497800 | T | C | Pack years adult smoking as proportion of life span exposed to smoking |
| rs45497800 | T | C | Pack years of smoking preview only |
| rs45497800 | T | C | Smoking status: current |
| rs4692811 | C | T | Frequency of depressed mood in last 2 weeks |
| rs4738679 | A | G | Cholelithiasis |
| rs4738679 | A | G | Impedance of arm left |
| rs4738679 | A | G | Impedance of arm right |
| rs4738679 | A | G | Impedance of whole body |
| rs4738679 | A | G | LDL cholesterol |
| rs4738679 | A | G | Low density lipoprotein |
| rs4738679 | A | G | Medication for cholesterol, blood pressure or diabetes: cholesterol lowering medication |
| rs4738679 | A | G | Self-reported cholelithiasis or gall stones |
| rs4738679 | A | G | Self-reported high cholesterol |
| rs4738679 | A | G | Total cholesterol |
| rs4738679 | A | G | Treatment with atorvastatin |
| rs4936098 | A | G | Diastolic blood pressure |
| rs4936098 | A | G | Impedance of arm left |
| rs4936098 | A | G | Impedance of arm right |
| rs4936098 | A | G | Impedance of whole body |
| rs4936098 | A | G | Pulse wave reflection index |
| rs4936098 | A | G | Systolic blood pressure |
| rs4936098 | A | G | Trunk fat percentage |
| rs55793120 | C | T | Arm fat-free mass right |
| rs55793120 | C | T | Arm predicted mass right |
| rs55793120 | C | T | Basal metabolic rate |
| rs55793120 | C | T | Height |
| rs55793120 | C | T | Leg fat-free mass left |
| rs55793120 | C | T | Leg predicted mass left |
| rs55793120 | C | T | Leg predicted mass right |
| rs55793120 | C | T | Trunk fat-free mass |
| rs55793120 | C | T | Trunk predicted mass |
| rs55793120 | C | T | Whole body fat-free mass |
| rs55793120 | C | T | Whole body water mass |
| rs56050831 | C | T | Mean corpuscular hemoglobin |
| rs56050831 | C | T | Mean corpuscular volume |
| rs56050831 | C | T | Red blood cell count |
| rs56050831 | C | T | Self-reported asthma |
| rs56050831 | C | T | Sitting height |
| rs56077123 | A | C | High light scatter reticulocyte count |
| rs56077123 | A | C | Reticulocyte count |
| rs56077123 | A | C | Reticulocyte fraction of red cells |
| rs56349194 | A | G | Comparative height size at age 10 |
| rs56349194 | A | G | Hip pain for 3+ months |
| rs56349194 | A | G | Trunk fat-free mass |
| rs56349194 | A | G | Trunk predicted mass |
| rs6000536 | C | T | Hemoglobin concentration |
| rs6000536 | C | T | Mean corpuscular hemoglobin |
| rs6000536 | C | T | Mean corpuscular hemoglobin concentration |
| rs6000536 | C | T | Mean corpuscular volume |
| rs6000536 | C | T | Mineral and other dietary supplements: iron |
| rs6000536 | C | T | Red cell distribution width |
| rs60254196 | A | G | Eosinophil count |
| rs60254196 | A | G | Eosinophil percentage of granulocytes |
| rs60254196 | A | G | Eosinophil percentage of white cells |
| rs60254196 | A | G | Neutrophil percentage of granulocytes |
| rs60254196 | A | G | Sum eosinophil basophil counts |
| rs6058181 | C | T | Arm fat-free mass left |
| rs6058181 | C | T | Arm fat-free mass right |
| rs6058181 | C | T | Arm predicted mass left |
| rs6058181 | C | T | Arm predicted mass right |
| rs6058181 | C | T | Basal metabolic rate |
| rs6058181 | C | T | Cause of death: subarachnoid haemorrhage from intracranial artery, unspecified |
| rs6058181 | C | T | Comparative height size at age 10 |
| rs6058181 | C | T | Creatinine in urine |
| rs6058181 | C | T | Height |
| rs6058181 | C | T | Leg fat-free mass left |
| rs6058181 | C | T | Leg fat-free mass right |
| rs6058181 | C | T | Leg predicted mass left |
| rs6058181 | C | T | Leg predicted mass right |
| rs6058181 | C | T | Plasma protein C levels |
| rs6058181 | C | T | Prothrombin time by INR international normalized ratio |
| rs6058181 | C | T | Self-reported deep venous thrombosis |
| rs6058181 | C | T | Sitting height |
| rs6058181 | C | T | Trunk fat-free mass |
| rs6058181 | C | T | Trunk predicted mass |
| rs6058181 | C | T | Whole body fat-free mass |
| rs6058181 | C | T | Whole body water mass |
| rs6087811 | G | T | Medication for cholesterol, blood pressure or diabetes: blood pressure medication |
| rs6112314 | A | C | Mean platelet volume |
| rs6112314 | A | C | Self-reported female infertility |
| rs61508842 | C | T | Treatment with epilim 100mg crushable tablet |
| rs61702987 | C | T | Treatment with marvelon tablet |
| rs61884471 | A | G | Treatment with adalate 10mg capsule |
| rs62191117 | A | G | Forced expiratory volume in 1-second |
| rs62191117 | A | G | Forced expiratory volume in 1-second, best measure |
| rs62191117 | A | G | Forced expiratory volume in 1-second, predicted percentage |
| rs62532512 | A | C | Body fat percentage |
| rs62532512 | A | C | Leg fat percentage left |
| rs62532512 | A | C | Leg fat percentage right |
| rs62532512 | A | C | Tobacco smoking: smokes on most or all days |
| rs62532512 | A | C | Trunk fat percentage |
| rs6445851 | A | G | Mean platelet volume |
| rs6445851 | A | G | Sitting height |
| rs646327 | A | G | Alcohol intake frequency |
| rs646327 | A | G | Alcohol intake versus 10 years previously |
| rs646327 | A | G | Alkaline phosphatase |
| rs646327 | A | G | Bring up phlegm or sputum/mucus on most days |
| rs646327 | A | G | Cholelithiasis |
| rs646327 | A | G | Comparative body size at age 10 |
| rs646327 | A | G | Crohns disease |
| rs646327 | A | G | Mean platelet volume |
| rs646327 | A | G | Medication for cholesterol, blood pressure or diabetes: cholesterol lowering medication |
| rs646327 | A | G | Medication for cholesterol, blood pressure or diabetes: none of the above |
| rs646327 | A | G | Morning or evening person |
| rs646327 | A | G | Mouth or teeth dental problems: dentures |
| rs646327 | A | G | Mouth or teeth dental problems: none of the above |
| rs646327 | A | G | No treatment with medication for cholesterol, blood pressure, diabetes, or take exogenous hormones |
| rs646327 | A | G | Number of self-reported non-cancer illnesses |
| rs646327 | A | G | Peak expiratory flow |
| rs646327 | A | G | Self-reported cholelithiasis or gall stones |
| rs646327 | A | G | Self-reported high cholesterol |
| rs646327 | A | G | Self-reported hypertension |
| rs646327 | A | G | Sitting height |
| rs646327 | A | G | Sodium in urine |
| rs646327 | A | G | Treatment with cholesterol lowering medication |
| rs646327 | A | G | Treatment with simvastatin |
| rs646327 | A | G | Vascular or heart problems diagnosed by doctor: high blood pressure |
| rs646327 | A | G | Vascular or heart problems diagnosed by doctor: none of the above |
| rs6550770 | C | T | Cause of death: perforation of intestine |
| rs6550770 | C | T | Treatment with salmeterol product |
| rs66710942 | C | T | Morning or evening person |
| rs6776814 | C | T | Treatment with mirtazapine |
| rs6806351 | C | T | Qualifications: college or university degree |
| rs6930661 | C | T | Treatment with clotrimazole |
| rs6953849 | A | G | Hair or balding pattern: pattern 4 |
| rs71315246 | A | G | Mean corpuscular hemoglobin |
| rs71315246 | A | G | Mean corpuscular volume |
| rs7155595 | A | C | Nervous feelings |
| rs7155595 | A | C | Types of transport used, excluding work: walk |
| rs72829893 | G | T | Impedance of leg left |
| rs72829893 | G | T | Impedance of leg right |
| rs72829893 | G | T | Leg fat-free mass left |
| rs72829893 | G | T | Leg fat-free mass right |
| rs72829893 | G | T | Leg predicted mass right |
| rs72829893 | G | T | Trunk fat-free mass |
| rs72829893 | G | T | Trunk predicted mass |
| rs72829893 | G | T | Whole body fat-free mass |
| rs72829893 | G | T | Whole body water mass |
| rs72901605 | C | T | Heel bone mineral density |
| rs72901605 | C | T | Impedance of leg left |
| rs72901605 | C | T | Impedance of leg right |
| rs72901605 | C | T | Impedance of whole body |
| rs72948274 | A | C | Cause of death: acute and subacute infective endocarditis |
| rs7359994 | C | T | Non-accidental death in close genetic family |
| rs74603314 | C | T | Hand grip strength left |
| rs7586445 | A | G | Getting up in morning |
| rs7635831 | A | G | Self-reported venous thromboembolic disease |
| rs76982728 | C | T | Treatment with codipar caplet |
| rs77293403 | A | G | Comparative height size at age 10 |
| rs77293403 | A | G | Forced expiratory volume in 1-second |
| rs77293403 | A | G | Forced expiratory volume in 1-second, best measure |
| rs77293403 | A | G | Forced vital capacity |
| rs77293403 | A | G | Forced vital capacity, best measure |
| rs77293403 | A | G | Height |
| rs77293403 | A | G | Sitting height |
| rs7916711 | A | G | Sitting height |
| rs79535861 | A | C | Self-reported malignant melanoma |
| rs7975087 | A | C | Arm fat-free mass left |
| rs7975087 | A | C | Arm fat-free mass right |
| rs7975087 | A | C | Arm predicted mass left |
| rs7975087 | A | C | Arm predicted mass right |
| rs7975087 | A | C | Basal metabolic rate |
| rs7975087 | A | C | High light scatter percentage of red cells |
| rs7975087 | A | C | High light scatter reticulocyte count |
| rs7975087 | A | C | Leg fat-free mass left |
| rs7975087 | A | C | Leg fat-free mass right |
| rs7975087 | A | C | Leg predicted mass left |
| rs7975087 | A | C | Leg predicted mass right |
| rs7975087 | A | C | Trunk fat-free mass |
| rs7975087 | A | C | Trunk predicted mass |
| rs7975087 | A | C | Waist circumference |
| rs7975087 | A | C | Weight |
| rs7975087 | A | C | Whole body fat-free mass |
| rs7975087 | A | C | Whole body water mass |
| rs8003149 | C | T | Body mass index |
| rs8003149 | C | T | Diastolic blood pressure |
| rs8003149 | C | T | Height |
| rs8003149 | C | T | Impedance of arm left |
| rs8003149 | C | T | Impedance of arm right |
| rs8003149 | C | T | Impedance of whole body |
| rs912860 | G | A | Carcinoma in situ of skin |
| rs9389714 | C | T | Impedance of arm right |
| rs9389714 | C | T | Nap during day |
| rs949971 | T | G | Sensitivity or hurt feelings |
| rs9537886 | A | C | Mean time to correctly identify matches |
| rs9537886 | A | C | Qualifications: college or university degree |
| rs9537886 | A | C | Years of educational attainment |
| rs9725395 | A | G | Senility |
| rs989682 | A | G | Arm fat percentage left |
| rs989682 | A | G | Platelet distribution width |
| rs989682 | A | G | Pulse rate |
| rs9954215 | C | T | Self-reported hypertension |
| rs9954215 | C | T | Vascular or heart problems diagnosed by doctor: high blood pressure |
| rs9954215 | C | T | Vascular or heart problems diagnosed by doctor: none of the above |

**TableS3. Results of analyses of all gut microbiota and he three autoimmune neurologic disorders.**

| **Gut microbiome** | **Methods** | **SNPs** | **beta** | **se** | **P-value** | **OR** | **95%CI** |
| --- | --- | --- | --- | --- | --- | --- | --- |
| ***Multiple sclerosis*** |  |  |  |  |  |  |  |
| class Actinobacteria | IVW | 14 | -0.14 | 0.11 | 0.2 | 0.872 | 0.708-1.075 |
| class Actinobacteria | MR Egger | 14 | 0.259 | 0.37 | 0.5 | 1.296 | 0.624-2.692 |
| class Actinobacteria | Weighted median | 14 | -0.28 | 0.13 | 0.032 | 0.76 | 0.591-0.976 |
| class Actinobacteria | Weighted mode | 14 | -0.33 | 0.22 | 0.15 | 0.718 | 0.47-1.097 |
| class Alphaproteobacteria | IVW | 5 | -0.01 | 0.13 | 0.955 | 0.993 | 0.777-1.268 |
| class Alphaproteobacteria | MR Egger | 5 | -0.53 | 1.24 | 0.697 | 0.589 | 0.052-6.617 |
| class Alphaproteobacteria | Weighted median | 5 | 0.023 | 0.16 | 0.882 | 1.023 | 0.753-1.39 |
| class Alphaproteobacteria | Weighted mode | 5 | 0.074 | 0.22 | 0.75 | 1.077 | 0.702-1.652 |
| class Bacilli | IVW | 12 | -0.05 | 0.13 | 0.69 | 0.948 | 0.73-1.231 |
| class Bacilli | MR Egger | 12 | -0.1 | 0.35 | 0.773 | 0.902 | 0.456-1.784 |
| class Bacilli | Weighted median | 12 | -0.09 | 0.14 | 0.511 | 0.911 | 0.691-1.202 |
| class Bacilli | Weighted mode | 12 | -0.21 | 0.19 | 0.304 | 0.812 | 0.555-1.186 |
| genus Parasutterella | IVW | 11 | -0.05 | 0.1 | 0.624 | 0.953 | 0.786-1.155 |
| genus Parasutterella | MR Egger | 11 | -0.07 | 0.21 | 0.746 | 0.932 | 0.617-1.408 |
| genus Parasutterella | Weighted median | 11 | -0.01 | 0.12 | 0.962 | 0.994 | 0.78-1.266 |
| genus Parasutterella | Weighted mode | 11 | -0.05 | 0.15 | 0.74 | 0.951 | 0.714-1.267 |
| class Betaproteobacteria | IVW | 11 | -0.19 | 0.1 | 0.053 | 0.824 | 0.678-1.003 |
| class Betaproteobacteria | MR Egger | 11 | -0.32 | 0.4 | 0.439 | 0.723 | 0.33-1.584 |
| class Betaproteobacteria | Weighted median | 11 | -0.18 | 0.14 | 0.178 | 0.833 | 0.638-1.087 |
| class Betaproteobacteria | Weighted mode | 11 | -0.2 | 0.22 | 0.372 | 0.818 | 0.537-1.246 |
| class Clostridia | IVW | 11 | 0.015 | 0.1 | 0.871 | 1.016 | 0.843-1.223 |
| class Clostridia | MR Egger | 11 | 0.013 | 0.22 | 0.955 | 1.013 | 0.658-1.558 |
| class Clostridia | Weighted median | 11 | -0.01 | 0.13 | 0.943 | 0.991 | 0.765-1.282 |
| class Clostridia | Weighted mode | 11 | 0.055 | 0.17 | 0.76 | 1.056 | 0.752-1.484 |
| class Coriobacteriia | IVW | 13 | 0.068 | 0.1 | 0.51 | 1.07 | 0.874-1.311 |
| class Coriobacteriia | MR Egger | 13 | 0.009 | 0.39 | 0.981 | 1.009 | 0.47-2.168 |
| class Coriobacteriia | Weighted median | 13 | 0.175 | 0.14 | 0.205 | 1.191 | 0.909-1.561 |
| class Coriobacteriia | Weighted mode | 13 | 0.302 | 0.2 | 0.157 | 1.353 | 0.914-2.004 |
| class Deltaproteobacteria | IVW | 12 | -0.17 | 0.1 | 0.113 | 0.848 | 0.692-1.04 |
| class Deltaproteobacteria | MR Egger | 12 | 0.273 | 0.5 | 0.598 | 1.315 | 0.491-3.518 |
| class Deltaproteobacteria | Weighted median | 12 | -0.09 | 0.12 | 0.482 | 0.917 | 0.721-1.167 |
| class Deltaproteobacteria | Weighted mode | 12 | -0.16 | 0.22 | 0.488 | 0.856 | 0.56-1.308 |
| class Erysipelotrichia | IVW | 13 | 0.127 | 0.1 | 0.199 | 1.135 | 0.936-1.377 |
| class Erysipelotrichia | MR Egger | 13 | 0.542 | 0.47 | 0.271 | 1.719 | 0.688-4.292 |
| class Erysipelotrichia | Weighted median | 13 | 0.149 | 0.14 | 0.269 | 1.16 | 0.891-1.51 |
| class Erysipelotrichia | Weighted mode | 13 | 0.131 | 0.22 | 0.557 | 1.14 | 0.746-1.741 |
| class Gammaproteobacteria | IVW | 4 | 0.096 | 0.25 | 0.701 | 1.101 | 0.674-1.796 |
| class Gammaproteobacteria | MR Egger | 4 | 1.232 | 1.86 | 0.576 | 3.428 | 0.09-131.05 |
| class Gammaproteobacteria | Weighted median | 4 | 0.043 | 0.19 | 0.823 | 1.044 | 0.716-1.521 |
| class Gammaproteobacteria | Weighted mode | 4 | -0.21 | 0.39 | 0.631 | 0.814 | 0.38-1.739 |
| class Lentisphaeria | IVW | 7 | -0.06 | 0.1 | 0.557 | 0.943 | 0.775-1.148 |
| class Lentisphaeria | MR Egger | 7 | 0.286 | 0.4 | 0.509 | 1.331 | 0.605-2.925 |
| class Lentisphaeria | Weighted median | 7 | -0.15 | 0.09 | 0.124 | 0.865 | 0.719-1.041 |
| class Lentisphaeria | Weighted mode | 7 | -0.16 | 0.11 | 0.217 | 0.855 | 0.685-1.068 |
| class Melainabacteria | IVW | 7 | -0.09 | 0.09 | 0.278 | 0.912 | 0.772-1.077 |
| class Melainabacteria | MR Egger | 7 | -0.03 | 0.35 | 0.931 | 0.969 | 0.489-1.919 |
| class Melainabacteria | Weighted median | 7 | -0.11 | 0.1 | 0.262 | 0.894 | 0.734-1.088 |
| class Melainabacteria | Weighted mode | 7 | -0.11 | 0.16 | 0.521 | 0.899 | 0.662-1.221 |
| class Methanobacteria | IVW | 9 | 0.004 | 0.06 | 0.939 | 1.004 | 0.901-1.119 |
| class Methanobacteria | MR Egger | 9 | 0.367 | 0.22 | 0.143 | 1.443 | 0.933-2.231 |
| class Methanobacteria | Weighted median | 9 | 0.061 | 0.08 | 0.411 | 1.063 | 0.919-1.23 |
| class Methanobacteria | Weighted mode | 9 | 0.083 | 0.11 | 0.46 | 1.087 | 0.881-1.341 |
| class Mollicutes | IVW | 10 | -0.12 | 0.09 | 0.204 | 0.89 | 0.744-1.065 |
| class Mollicutes | MR Egger | 10 | 0.117 | 0.53 | 0.83 | 1.125 | 0.398-3.176 |
| class Mollicutes | Weighted median | 10 | -0.01 | 0.13 | 0.932 | 0.989 | 0.772-1.267 |
| class Mollicutes | Weighted mode | 10 | 0.05 | 0.25 | 0.847 | 1.051 | 0.643-1.719 |
| class Negativicutes | IVW | 11 | -0.05 | 0.15 | 0.76 | 0.955 | 0.71-1.284 |
| class Negativicutes | MR Egger | 11 | -0.44 | 0.56 | 0.451 | 0.642 | 0.213-1.933 |
| class Negativicutes | Weighted median | 11 | -0.05 | 0.16 | 0.747 | 0.951 | 0.699-1.294 |
| class Negativicutes | Weighted mode | 11 | 0.123 | 0.25 | 0.633 | 1.131 | 0.693-1.845 |
| class Verrucomicrobiae | IVW | 8 | -0.01 | 0.1 | 0.901 | 0.988 | 0.814-1.198 |
| class Verrucomicrobiae | MR Egger | 8 | 1.427 | 1.31 | 0.317 | 4.167 | 0.32-54.206 |
| class Verrucomicrobiae | Weighted median | 8 | 0.024 | 0.12 | 0.846 | 1.024 | 0.806-1.3 |
| class Verrucomicrobiae | Weighted mode | 8 | 0.041 | 0.19 | 0.832 | 1.042 | 0.722-1.503 |
| family Acidaminococcaceae | IVW | 5 | -0.02 | 0.22 | 0.936 | 0.982 | 0.64-1.508 |
| family Acidaminococcaceae | MR Egger | 5 | -0.65 | 0.64 | 0.385 | 0.521 | 0.148-1.834 |
| family Acidaminococcaceae | Weighted median | 5 | -0.19 | 0.17 | 0.262 | 0.824 | 0.587-1.155 |
| family Acidaminococcaceae | Weighted mode | 5 | -0.27 | 0.2 | 0.234 | 0.761 | 0.519-1.116 |
| family Actinomycetaceae | IVW | 5 | -0.09 | 0.1 | 0.405 | 0.917 | 0.747-1.125 |
| family Actinomycetaceae | MR Egger | 5 | -0.19 | 0.31 | 0.587 | 0.828 | 0.45-1.523 |
| family Actinomycetaceae | Weighted median | 5 | -0.07 | 0.13 | 0.602 | 0.933 | 0.718-1.212 |
| family Actinomycetaceae | Weighted mode | 5 | -0.06 | 0.19 | 0.772 | 0.944 | 0.656-1.359 |
| family Alcaligenaceae | IVW | 8 | 0.041 | 0.14 | 0.77 | 1.042 | 0.789-1.378 |
| family Alcaligenaceae | MR Egger | 8 | -0.82 | 0.65 | 0.252 | 0.439 | 0.123-1.57 |
| family Alcaligenaceae | Weighted median | 8 | -0.07 | 0.17 | 0.684 | 0.934 | 0.672-1.298 |
| family Alcaligenaceae | Weighted mode | 8 | -0.11 | 0.26 | 0.687 | 0.896 | 0.536-1.497 |
| family Bacteroidaceae | IVW | 8 | -0.02 | 0.13 | 0.903 | 0.985 | 0.769-1.261 |
| family Bacteroidaceae | MR Egger | 8 | -0.78 | 0.68 | 0.297 | 0.458 | 0.12-1.751 |
| family Bacteroidaceae | Weighted median | 8 | -0.12 | 0.17 | 0.5 | 0.891 | 0.638-1.245 |
| family Bacteroidaceae | Weighted mode | 8 | -0.13 | 0.22 | 0.581 | 0.881 | 0.574-1.353 |
| family Bacteroidales | IVW | 4 | 0.127 | 0.16 | 0.426 | 1.136 | 0.83-1.554 |
| family Bacteroidales | MR Egger | 4 | -0.51 | 0.57 | 0.463 | 0.601 | 0.199-1.818 |
| family Bacteroidales | Weighted median | 4 | 0.028 | 0.15 | 0.85 | 1.028 | 0.773-1.367 |
| family Bacteroidales | Weighted mode | 4 | -0.09 | 0.2 | 0.686 | 0.916 | 0.624-1.345 |
| family Bifidobacteriaceae | IVW | 12 | -0.07 | 0.11 | 0.55 | 0.935 | 0.751-1.165 |
| family Bifidobacteriaceae | MR Egger | 12 | 0.907 | 0.32 | 0.018 | 2.478 | 1.32-4.65 |
| family Bifidobacteriaceae | Weighted median | 12 | -0.05 | 0.14 | 0.734 | 0.954 | 0.728-1.251 |
| family Bifidobacteriaceae | Weighted mode | 12 | -0.19 | 0.29 | 0.527 | 0.828 | 0.471-1.457 |
| family Clostridiaceae1 | IVW | 10 | 0.08 | 0.12 | 0.502 | 1.083 | 0.858-1.367 |
| family Clostridiaceae1 | MR Egger | 10 | 0.53 | 0.39 | 0.207 | 1.698 | 0.797-3.619 |
| family Clostridiaceae1 | Weighted median | 10 | 0.01 | 0.14 | 0.946 | 1.01 | 0.767-1.329 |
| family Clostridiaceae1 | Weighted mode | 10 | -0.01 | 0.22 | 0.969 | 0.991 | 0.639-1.539 |
| family Clostridiales vadin BB60 group | IVW | 14 | -0.11 | 0.07 | 0.116 | 0.892 | 0.773-1.029 |
| family Clostridiales vadin BB60 group | MR Egger | 14 | -0.09 | 0.26 | 0.75 | 0.918 | 0.55-1.532 |
| family Clostridiales vadin BB60 group | Weighted median | 14 | -0.2 | 0.1 | 0.051 | 0.823 | 0.676-1.001 |
| family Clostridiales vadin BB60 group | Weighted mode | 14 | -0.2 | 0.17 | 0.273 | 0.82 | 0.584-1.152 |
| family Coriobacteriaceae | IVW | 13 | 0.068 | 0.1 | 0.51 | 1.07 | 0.874-1.311 |
| family Coriobacteriaceae | MR Egger | 13 | 0.009 | 0.39 | 0.981 | 1.009 | 0.47-2.168 |
| family Coriobacteriaceae | Weighted median | 13 | 0.175 | 0.14 | 0.194 | 1.191 | 0.915-1.551 |
| family Coriobacteriaceae | Weighted mode | 13 | 0.302 | 0.21 | 0.177 | 1.353 | 0.895-2.045 |
| family Defluviitaleaceae | IVW | 8 | 0.007 | 0.13 | 0.96 | 1.007 | 0.779-1.301 |
| family Defluviitaleaceae | MR Egger | 8 | 0.424 | 0.4 | 0.333 | 1.528 | 0.694-3.365 |
| family Defluviitaleaceae | Weighted median | 8 | -0.13 | 0.14 | 0.352 | 0.88 | 0.672-1.152 |
| family Defluviitaleaceae | Weighted mode | 8 | -0.19 | 0.19 | 0.348 | 0.824 | 0.564-1.202 |
| family Desulfovibrionaceae | IVW | 9 | -0.12 | 0.13 | 0.347 | 0.884 | 0.684-1.143 |
| family Desulfovibrionaceae | MR Egger | 9 | 0.104 | 0.59 | 0.867 | 1.109 | 0.346-3.554 |
| family Desulfovibrionaceae | Weighted median | 9 | -0.03 | 0.15 | 0.87 | 0.975 | 0.722-1.317 |
| family Desulfovibrionaceae | Weighted mode | 9 | 0.042 | 0.24 | 0.865 | 1.043 | 0.651-1.673 |
| family Enterobacteriaceae | IVW | 5 | 0.16 | 0.16 | 0.303 | 1.174 | 0.865-1.592 |
| family Enterobacteriaceae | MR Egger | 5 | 1.066 | 0.91 | 0.325 | 2.903 | 0.489-17.224 |
| family Enterobacteriaceae | Weighted median | 5 | 0.298 | 0.19 | 0.109 | 1.347 | 0.936-1.94 |
| family Enterobacteriaceae | Weighted mode | 5 | 0.356 | 0.27 | 0.251 | 1.428 | 0.849-2.402 |
| family Erysipelotrichaceae | IVW | 13 | 0.127 | 0.1 | 0.199 | 1.135 | 0.936-1.377 |
| family Erysipelotrichaceae | MR Egger | 13 | 0.542 | 0.47 | 0.271 | 1.719 | 0.688-4.292 |
| family Erysipelotrichaceae | Weighted median | 13 | 0.149 | 0.13 | 0.268 | 1.16 | 0.892-1.509 |
| family Erysipelotrichaceae | Weighted mode | 13 | 0.131 | 0.2 | 0.534 | 1.14 | 0.764-1.7 |
| family Family XI | IVW | 7 | -0.03 | 0.09 | 0.779 | 0.976 | 0.821-1.16 |
| family Family XI | MR Egger | 7 | 0.294 | 0.61 | 0.648 | 1.342 | 0.409-4.401 |
| family Family XI | Weighted median | 7 | 0 | 0.08 | 1 | 1 | 0.847-1.18 |
| family Family XI | Weighted mode | 7 | 0.019 | 0.1 | 0.858 | 1.019 | 0.833-1.248 |
| family Family XIII | IVW | 8 | -0.05 | 0.12 | 0.678 | 0.951 | 0.752-1.204 |
| family Family XIII | MR Egger | 8 | -0.84 | 0.76 | 0.311 | 0.431 | 0.097-1.913 |
| family Family XIII | Weighted median | 8 | -0.04 | 0.15 | 0.788 | 0.96 | 0.71-1.296 |
| family Family XIII | Weighted mode | 8 | -0.04 | 0.23 | 0.873 | 0.963 | 0.614-1.508 |
| family Lachnospiraceae | IVW | 13 | -0.19 | 0.12 | 0.091 | 0.824 | 0.658-1.032 |
| family Lachnospiraceae | MR Egger | 13 | -0.51 | 0.37 | 0.197 | 0.601 | 0.291-1.242 |
| family Lachnospiraceae | Weighted median | 13 | -0.28 | 0.14 | 0.041 | 0.755 | 0.576-0.989 |
| family Lachnospiraceae | Weighted mode | 13 | -0.36 | 0.22 | 0.133 | 0.699 | 0.452-1.081 |
| family Lactobacillaceae | IVW | 7 | -0.04 | 0.13 | 0.766 | 0.963 | 0.753-1.232 |
| family Lactobacillaceae | MR Egger | 7 | -0.84 | 0.26 | 0.023 | 0.431 | 0.259-0.716 |
| family Lactobacillaceae | Weighted median | 7 | -0.1 | 0.12 | 0.42 | 0.905 | 0.711-1.153 |
| family Lactobacillaceae | Weighted mode | 7 | -0.13 | 0.16 | 0.446 | 0.876 | 0.638-1.204 |
| family Methanobacteriaceae | IVW | 9 | 0.004 | 0.06 | 0.939 | 1.004 | 0.901-1.119 |
| family Methanobacteriaceae | MR Egger | 9 | 0.367 | 0.22 | 0.143 | 1.443 | 0.933-2.231 |
| family Methanobacteriaceae | Weighted median | 9 | 0.061 | 0.08 | 0.423 | 1.063 | 0.915-1.235 |
| family Methanobacteriaceae | Weighted mode | 9 | 0.083 | 0.11 | 0.466 | 1.087 | 0.878-1.346 |
| family Oxalobacteraceae | IVW | 12 | -0.01 | 0.05 | 0.888 | 0.993 | 0.895-1.101 |
| family Oxalobacteraceae | MR Egger | 12 | -0.1 | 0.24 | 0.69 | 0.907 | 0.571-1.443 |
| family Oxalobacteraceae | Weighted median | 12 | -0.05 | 0.07 | 0.494 | 0.952 | 0.828-1.095 |
| family Oxalobacteraceae | Weighted mode | 12 | -0.09 | 0.13 | 0.472 | 0.91 | 0.71-1.166 |
| family Pasteurellaceae | IVW | 10 | 0.003 | 0.11 | 0.976 | 1.003 | 0.812-1.24 |
| family Pasteurellaceae | MR Egger | 10 | -0.5 | 0.27 | 0.096 | 0.605 | 0.359-1.019 |
| family Pasteurellaceae | Weighted median | 10 | -0.05 | 0.12 | 0.697 | 0.954 | 0.752-1.21 |
| family Pasteurellaceae | Weighted mode | 10 | -0.12 | 0.17 | 0.501 | 0.887 | 0.633-1.241 |
| family Peptococcaceae | IVW | 4 | 0.087 | 0.13 | 0.495 | 1.091 | 0.849-1.403 |
| family Peptococcaceae | MR Egger | 4 | -0.8 | 1.59 | 0.666 | 0.451 | 0.02-10.148 |
| family Peptococcaceae | Weighted median | 4 | 0.08 | 0.16 | 0.611 | 1.083 | 0.797-1.472 |
| family Peptococcaceae | Weighted mode | 4 | 0.07 | 0.23 | 0.784 | 1.072 | 0.68-1.692 |
| family Peptostreptococcaceae | IVW | 10 | -0.11 | 0.15 | 0.455 | 0.895 | 0.669-1.197 |
| family Peptostreptococcaceae | MR Egger | 10 | -0.62 | 0.33 | 0.096 | 0.538 | 0.283-1.024 |
| family Peptostreptococcaceae | Weighted median | 10 | -0.12 | 0.16 | 0.458 | 0.892 | 0.658-1.208 |
| family Peptostreptococcaceae | Weighted mode | 10 | -0.38 | 0.31 | 0.251 | 0.682 | 0.371-1.256 |
| family Porphyromonadaceae | IVW | 8 | -0.1 | 0.12 | 0.414 | 0.905 | 0.712-1.15 |
| family Porphyromonadaceae | MR Egger | 8 | -0.47 | 0.48 | 0.361 | 0.625 | 0.246-1.588 |
| family Porphyromonadaceae | Weighted median | 8 | -0.13 | 0.15 | 0.414 | 0.882 | 0.651-1.193 |
| family Porphyromonadaceae | Weighted mode | 8 | -0.16 | 0.21 | 0.462 | 0.853 | 0.571-1.274 |
| family Prevotellaceae | IVW | 14 | -0.1 | 0.08 | 0.201 | 0.903 | 0.772-1.056 |
| family Prevotellaceae | MR Egger | 14 | -0.04 | 0.28 | 0.901 | 0.964 | 0.552-1.684 |
| family Prevotellaceae | Weighted median | 14 | 0.029 | 0.11 | 0.783 | 1.03 | 0.837-1.267 |
| family Prevotellaceae | Weighted mode | 14 | 0.075 | 0.17 | 0.66 | 1.077 | 0.779-1.49 |
| family Rhodospirillaceae | IVW | 13 | 0.075 | 0.09 | 0.39 | 1.078 | 0.909-1.278 |
| family Rhodospirillaceae | MR Egger | 13 | 0.175 | 0.59 | 0.772 | 1.191 | 0.375-3.78 |
| family Rhodospirillaceae | Weighted median | 13 | 0.133 | 0.1 | 0.177 | 1.142 | 0.942-1.386 |
| family Rhodospirillaceae | Weighted mode | 13 | 0.156 | 0.15 | 0.314 | 1.169 | 0.874-1.564 |
| family Rikenellaceae | IVW | 17 | 0.15 | 0.08 | 0.074 | 1.162 | 0.986-1.369 |
| family Rikenellaceae | MR Egger | 17 | 0.154 | 0.33 | 0.645 | 1.166 | 0.615-2.213 |
| family Rikenellaceae | Weighted median | 17 | 0.099 | 0.11 | 0.369 | 1.104 | 0.89-1.368 |
| family Rikenellaceae | Weighted mode | 17 | 0.075 | 0.18 | 0.683 | 1.078 | 0.758-1.531 |
| family Ruminococcaceae | IVW | 7 | 0.119 | 0.13 | 0.369 | 1.127 | 0.869-1.462 |
| family Ruminococcaceae | MR Egger | 7 | 0.308 | 0.34 | 0.405 | 1.361 | 0.7-2.645 |
| family Ruminococcaceae | Weighted median | 7 | -0.04 | 0.18 | 0.813 | 0.96 | 0.682-1.351 |
| family Ruminococcaceae | Weighted mode | 7 | -0.06 | 0.23 | 0.789 | 0.939 | 0.603-1.462 |
| family Streptococcaceae | IVW | 9 | -0.11 | 0.14 | 0.411 | 0.895 | 0.686-1.167 |
| family Streptococcaceae | MR Egger | 9 | 0.457 | 0.48 | 0.371 | 1.58 | 0.619-4.033 |
| family Streptococcaceae | Weighted median | 9 | 0.056 | 0.15 | 0.714 | 1.058 | 0.784-1.427 |
| family Streptococcaceae | Weighted mode | 9 | 0.146 | 0.24 | 0.559 | 1.157 | 0.723-1.852 |
| family Veillonellaceae | IVW | 15 | 0.107 | 0.08 | 0.157 | 1.113 | 0.959-1.292 |
| family Veillonellaceae | MR Egger | 15 | 0.023 | 0.19 | 0.903 | 1.023 | 0.711-1.473 |
| family Veillonellaceae | Weighted median | 15 | 0.018 | 0.11 | 0.868 | 1.018 | 0.826-1.255 |
| family Veillonellaceae | Weighted mode | 15 | -0.02 | 0.15 | 0.912 | 0.983 | 0.735-1.315 |
| family Verrucomicrobiaceae | IVW | 8 | -0.01 | 0.1 | 0.901 | 0.988 | 0.814-1.198 |
| family Verrucomicrobiaceae | MR Egger | 8 | 1.435 | 1.31 | 0.316 | 4.2 | 0.321-54.896 |
| family Verrucomicrobiaceae | Weighted median | 8 | 0.024 | 0.12 | 0.845 | 1.024 | 0.807-1.3 |
| family Verrucomicrobiaceae | Weighted mode | 8 | 0.041 | 0.18 | 0.829 | 1.042 | 0.727-1.494 |
| family Victivallaceae | IVW | 12 | 0.016 | 0.06 | 0.773 | 1.016 | 0.912-1.131 |
| family Victivallaceae | MR Egger | 12 | 0.555 | 0.22 | 0.032 | 1.742 | 1.126-2.694 |
| family Victivallaceae | Weighted median | 12 | -0.03 | 0.07 | 0.647 | 0.97 | 0.851-1.106 |
| family Victivallaceae | Weighted mode | 12 | -0.05 | 0.1 | 0.632 | 0.954 | 0.792-1.15 |
| genus Actinomyces | IVW | 5 | -0.07 | 0.11 | 0.503 | 0.931 | 0.756-1.147 |
| genus Actinomyces | MR Egger | 5 | -0.27 | 0.3 | 0.443 | 0.766 | 0.424-1.386 |
| genus Actinomyces | Weighted median | 5 | 0.006 | 0.14 | 0.965 | 1.006 | 0.771-1.313 |
| genus Actinomyces | Weighted mode | 5 | 0.022 | 0.2 | 0.915 | 1.023 | 0.696-1.503 |
| genus Adlercreutzia | IVW | 8 | -0.05 | 0.09 | 0.599 | 0.955 | 0.806-1.132 |
| genus Adlercreutzia | MR Egger | 8 | -0.47 | 0.38 | 0.262 | 0.623 | 0.294-1.318 |
| genus Adlercreutzia | Weighted median | 8 | -0.08 | 0.11 | 0.501 | 0.926 | 0.741-1.158 |
| genus Adlercreutzia | Weighted mode | 8 | -0.11 | 0.17 | 0.524 | 0.893 | 0.641-1.244 |
| genus Anaerofilum | IVW | 7 | 0.111 | 0.07 | 0.104 | 1.118 | 0.977-1.278 |
| genus Anaerofilum | MR Egger | 7 | -0.04 | 0.42 | 0.929 | 0.961 | 0.421-2.194 |
| genus Anaerofilum | Weighted median | 7 | 0.023 | 0.09 | 0.81 | 1.023 | 0.851-1.23 |
| genus Anaerofilum | Weighted mode | 7 | -0.01 | 0.13 | 0.924 | 0.987 | 0.759-1.283 |
| genus Anaerostipes | IVW | 10 | 0.102 | 0.1 | 0.324 | 1.107 | 0.905-1.354 |
| genus Anaerostipes | MR Egger | 10 | 0.054 | 0.42 | 0.901 | 1.055 | 0.463-2.405 |
| genus Anaerostipes | Weighted median | 10 | 0.034 | 0.14 | 0.807 | 1.035 | 0.786-1.363 |
| genus Anaerostipes | Weighted mode | 10 | -0.13 | 0.24 | 0.595 | 0.876 | 0.548-1.402 |
| genus Butyrivibrio | IVW | 10 | -0.02 | 0.06 | 0.731 | 0.98 | 0.872-1.1 |
| genus Butyrivibrio | MR Egger | 10 | 0.106 | 0.47 | 0.828 | 1.112 | 0.44-2.807 |
| genus Butyrivibrio | Weighted median | 10 | -0.04 | 0.07 | 0.605 | 0.962 | 0.832-1.113 |
| genus Butyrivibrio | Weighted mode | 10 | 0.009 | 0.11 | 0.93 | 1.01 | 0.822-1.24 |
| genus Catenibacterium | IVW | 3 | -0.03 | 0.08 | 0.758 | 0.975 | 0.827-1.148 |
| genus Catenibacterium | MR Egger | 3 | -0.97 | 0.97 | 0.499 | 0.378 | 0.057-2.528 |
| genus Catenibacterium | Weighted median | 3 | -0.09 | 0.11 | 0.425 | 0.918 | 0.744-1.133 |
| genus Catenibacterium | Weighted mode | 3 | -0.09 | 0.12 | 0.529 | 0.914 | 0.725-1.153 |
| genus Christensenellaceae R 7group | IVW | 6 | -0.3 | 0.18 | 0.105 | 0.743 | 0.518-1.064 |
| genus Christensenellaceae R 7group | MR Egger | 6 | 0.272 | 0.72 | 0.726 | 1.313 | 0.318-5.413 |
| genus Christensenellaceae R 7group | Weighted median | 6 | -0.25 | 0.21 | 0.238 | 0.782 | 0.52-1.176 |
| genus Christensenellaceae R 7group | Weighted mode | 6 | -0.25 | 0.31 | 0.451 | 0.779 | 0.428-1.418 |
| genus Clostridium innocuum group | IVW | 8 | -0.06 | 0.07 | 0.333 | 0.939 | 0.825-1.067 |
| genus Clostridium innocuum group | MR Egger | 8 | 0.304 | 0.33 | 0.391 | 1.356 | 0.711-2.585 |
| genus Clostridium innocuum group | Weighted median | 8 | -0.07 | 0.09 | 0.423 | 0.93 | 0.778-1.111 |
| genus Clostridium innocuum group | Weighted mode | 8 | -0.14 | 0.12 | 0.275 | 0.871 | 0.692-1.095 |
| genus Collinsella | IVW | 7 | 0.015 | 0.14 | 0.916 | 1.015 | 0.769-1.341 |
| genus Collinsella | MR Egger | 7 | 0.656 | 0.58 | 0.312 | 1.927 | 0.614-6.052 |
| genus Collinsella | Weighted median | 7 | -0.02 | 0.17 | 0.908 | 0.98 | 0.701-1.371 |
| genus Collinsella | Weighted mode | 7 | -0.26 | 0.28 | 0.393 | 0.774 | 0.448-1.336 |
| genus Defluviitaleaceae UCG011 | IVW | 7 | 0.125 | 0.14 | 0.355 | 1.134 | 0.869-1.478 |
| genus Defluviitaleaceae UCG011 | MR Egger | 7 | 0.659 | 0.44 | 0.197 | 1.934 | 0.81-4.615 |
| genus Defluviitaleaceae UCG011 | Weighted median | 7 | 0.15 | 0.15 | 0.316 | 1.162 | 0.866-1.559 |
| genus Defluviitaleaceae UCG011 | Weighted mode | 7 | 0.173 | 0.25 | 0.516 | 1.188 | 0.728-1.94 |
| genus Desulfovibrio | IVW | 9 | -0.05 | 0.1 | 0.605 | 0.949 | 0.78-1.156 |
| genus Desulfovibrio | MR Egger | 9 | -0.46 | 0.27 | 0.13 | 0.632 | 0.374-1.068 |
| genus Desulfovibrio | Weighted median | 9 | 0.009 | 0.12 | 0.944 | 1.009 | 0.793-1.283 |
| genus Desulfovibrio | Weighted mode | 9 | 0.001 | 0.19 | 0.996 | 1.001 | 0.692-1.449 |
| genus Dialister | IVW | 9 | 0.003 | 0.1 | 0.975 | 1.003 | 0.832-1.21 |
| genus Dialister | MR Egger | 9 | -0.01 | 0.57 | 0.982 | 0.987 | 0.325-2.996 |
| genus Dialister | Weighted median | 9 | -0.03 | 0.12 | 0.792 | 0.968 | 0.76-1.232 |
| genus Dialister | Weighted mode | 9 | -0.06 | 0.18 | 0.744 | 0.942 | 0.666-1.332 |
| genus Eggerthella | IVW | 7 | -0.13 | 0.1 | 0.158 | 0.875 | 0.726-1.053 |
| genus Eggerthella | MR Egger | 7 | -1.3 | 0.86 | 0.193 | 0.273 | 0.05-1.481 |
| genus Eggerthella | Weighted median | 7 | -0.03 | 0.1 | 0.778 | 0.972 | 0.796-1.186 |
| genus Eggerthella | Weighted mode | 7 | -0.01 | 0.13 | 0.946 | 0.991 | 0.772-1.272 |
| genus Eisenbergiella | IVW | 10 | 0 | 0.06 | 0.999 | 1 | 0.888-1.126 |
| genus Eisenbergiella | MR Egger | 10 | -0.21 | 0.46 | 0.662 | 0.812 | 0.331-1.993 |
| genus Eisenbergiella | Weighted median | 10 | 0.048 | 0.09 | 0.569 | 1.05 | 0.889-1.239 |
| genus Eisenbergiella | Weighted mode | 10 | 0.097 | 0.14 | 0.51 | 1.102 | 0.835-1.453 |
| genus Enterorhabdus | IVW | 4 | 0.037 | 0.12 | 0.755 | 1.037 | 0.824-1.305 |
| genus Enterorhabdus | MR Egger | 4 | -0.04 | 0.35 | 0.918 | 0.96 | 0.483-1.908 |
| genus Enterorhabdus | Weighted median | 4 | 0.035 | 0.13 | 0.783 | 1.036 | 0.805-1.332 |
| genus Enterorhabdus | Weighted mode | 4 | -0.08 | 0.15 | 0.634 | 0.923 | 0.685-1.244 |
| genus Eubacterium fissicatena group | IVW | 9 | -0.02 | 0.07 | 0.767 | 0.98 | 0.858-1.119 |
| genus Eubacterium fissicatena group | MR Egger | 9 | -0.16 | 0.44 | 0.726 | 0.852 | 0.36-2.017 |
| genus Eubacterium fissicatena group | Weighted median | 9 | 0.004 | 0.08 | 0.963 | 1.004 | 0.865-1.164 |
| genus Eubacterium fissicatena group | Weighted mode | 9 | -0.02 | 0.1 | 0.882 | 0.984 | 0.804-1.205 |
| genus Eubacterium rectale group | IVW | 6 | -0.1 | 0.16 | 0.543 | 0.91 | 0.67-1.234 |
| genus Eubacterium rectale group | MR Egger | 6 | -0.63 | 0.51 | 0.281 | 0.531 | 0.196-1.437 |
| genus Eubacterium rectale group | Weighted median | 6 | -0.07 | 0.17 | 0.681 | 0.932 | 0.665-1.306 |
| genus Eubacterium rectale group | Weighted mode | 6 | -0.08 | 0.27 | 0.774 | 0.923 | 0.549-1.551 |
| genus Fusicatenibacter | IVW | 18 | 0.032 | 0.08 | 0.694 | 1.032 | 0.881-1.21 |
| genus Fusicatenibacter | MR Egger | 18 | 0.06 | 0.33 | 0.858 | 1.062 | 0.556-2.027 |
| genus Fusicatenibacter | Weighted median | 18 | 0.085 | 0.1 | 0.417 | 1.088 | 0.887-1.336 |
| genus Fusicatenibacter | Weighted mode | 18 | 0.134 | 0.18 | 0.474 | 1.143 | 0.799-1.636 |
| genus Gordonibacter | IVW | 11 | 0.003 | 0.06 | 0.953 | 1.003 | 0.9-1.118 |
| genus Gordonibacter | MR Egger | 11 | 0.076 | 0.25 | 0.765 | 1.079 | 0.665-1.751 |
| genus Gordonibacter | Weighted median | 11 | -0.02 | 0.07 | 0.823 | 0.985 | 0.861-1.126 |
| genus Gordonibacter | Weighted mode | 11 | -0.07 | 0.13 | 0.586 | 0.929 | 0.721-1.199 |
| genus Intestinimonas | IVW | 15 | 0.023 | 0.08 | 0.764 | 1.023 | 0.883-1.185 |
| genus Intestinimonas | MR Egger | 15 | 0.177 | 0.21 | 0.419 | 1.194 | 0.788-1.81 |
| genus Intestinimonas | Weighted median | 15 | 0.047 | 0.1 | 0.63 | 1.048 | 0.866-1.269 |
| genus Intestinimonas | Weighted mode | 15 | 0.054 | 0.16 | 0.745 | 1.055 | 0.767-1.452 |
| genus Lachnospiraceae FCS020 group | IVW | 12 | 0.005 | 0.08 | 0.955 | 1.005 | 0.854-1.182 |
| genus Lachnospiraceae FCS020 group | MR Egger | 12 | 0.207 | 0.2 | 0.33 | 1.23 | 0.828-1.828 |
| genus Lachnospiraceae FCS020 group | Weighted median | 12 | -0.04 | 0.12 | 0.75 | 0.964 | 0.77-1.208 |
| genus Lachnospiraceae FCS020 group | Weighted mode | 12 | -0.05 | 0.17 | 0.797 | 0.956 | 0.682-1.338 |
| genus Lachnospiraceae UCG001 | IVW | 10 | -0.02 | 0.08 | 0.774 | 0.977 | 0.831-1.148 |
| genus Lachnospiraceae UCG001 | MR Egger | 10 | -0.25 | 0.4 | 0.548 | 0.776 | 0.352-1.711 |
| genus Lachnospiraceae UCG001 | Weighted median | 10 | 0.017 | 0.11 | 0.875 | 1.018 | 0.819-1.264 |
| genus Lachnospiraceae UCG001 | Weighted mode | 10 | 0.035 | 0.13 | 0.796 | 1.036 | 0.799-1.343 |
| genus Lachnospiraceae UCG008 | IVW | 10 | 0.089 | 0.07 | 0.209 | 1.093 | 0.951-1.256 |
| genus Lachnospiraceae UCG008 | MR Egger | 10 | 0.229 | 0.43 | 0.607 | 1.258 | 0.543-2.913 |
| genus Lachnospiraceae UCG008 | Weighted median | 10 | 0.143 | 0.09 | 0.094 | 1.154 | 0.976-1.365 |
| genus Lachnospiraceae UCG008 | Weighted mode | 10 | 0.258 | 0.17 | 0.164 | 1.294 | 0.927-1.807 |
| genus Lactobacillus | IVW | 8 | -0.06 | 0.12 | 0.59 | 0.94 | 0.749-1.178 |
| genus Lactobacillus | MR Egger | 8 | -0.9 | 0.27 | 0.015 | 0.406 | 0.241-0.685 |
| genus Lactobacillus | Weighted median | 8 | -0.14 | 0.12 | 0.239 | 0.869 | 0.688-1.098 |
| genus Lactobacillus | Weighted mode | 8 | -0.2 | 0.16 | 0.261 | 0.818 | 0.593-1.128 |
| genus Marvinbryantia | IVW | 7 | -0.02 | 0.1 | 0.826 | 0.978 | 0.8-1.195 |
| genus Marvinbryantia | MR Egger | 7 | 0.397 | 0.45 | 0.418 | 1.488 | 0.616-3.593 |
| genus Marvinbryantia | Weighted median | 7 | -0.03 | 0.14 | 0.835 | 0.971 | 0.738-1.278 |
| genus Marvinbryantia | Weighted mode | 7 | -0.03 | 0.18 | 0.885 | 0.974 | 0.69-1.375 |
| genus Parabacteroides | IVW | 4 | -0.18 | 0.16 | 0.268 | 0.835 | 0.606-1.149 |
| genus Parabacteroides | MR Egger | 4 | -1.09 | 1.13 | 0.435 | 0.336 | 0.037-3.057 |
| genus Parabacteroides | Weighted median | 4 | -0.22 | 0.2 | 0.261 | 0.8 | 0.542-1.181 |
| genus Parabacteroides | Weighted mode | 4 | -0.33 | 0.26 | 0.303 | 0.723 | 0.432-1.207 |
| genus Peptococcus | IVW | 9 | 0.008 | 0.07 | 0.906 | 1.008 | 0.881-1.154 |
| genus Peptococcus | MR Egger | 9 | 0.168 | 0.3 | 0.594 | 1.183 | 0.656-2.134 |
| genus Peptococcus | Weighted median | 9 | -0.03 | 0.09 | 0.693 | 0.966 | 0.816-1.145 |
| genus Peptococcus | Weighted mode | 9 | -0.05 | 0.14 | 0.712 | 0.949 | 0.726-1.24 |
| genus Romboutsia | IVW | 10 | -0.13 | 0.11 | 0.238 | 0.876 | 0.704-1.091 |
| genus Romboutsia | MR Egger | 10 | -0.04 | 0.42 | 0.926 | 0.96 | 0.419-2.2 |
| genus Romboutsia | Weighted median | 10 | -0.24 | 0.13 | 0.069 | 0.788 | 0.609-1.019 |
| genus Romboutsia | Weighted mode | 10 | -0.29 | 0.21 | 0.196 | 0.749 | 0.499-1.123 |
| genus Ruminiclostridium6 | IVW | 11 | 0.103 | 0.15 | 0.481 | 1.109 | 0.832-1.478 |
| genus Ruminiclostridium6 | MR Egger | 11 | 0.223 | 0.37 | 0.559 | 1.25 | 0.608-2.57 |
| genus Ruminiclostridium6 | Weighted median | 11 | 0.166 | 0.12 | 0.178 | 1.181 | 0.927-1.503 |
| genus Ruminiclostridium6 | Weighted mode | 11 | 0.264 | 0.16 | 0.126 | 1.302 | 0.955-1.775 |
| genus Ruminococcaceae NK4A214 group | IVW | 11 | -0.13 | 0.1 | 0.19 | 0.877 | 0.72-1.067 |
| genus Ruminococcaceae NK4A214 group | MR Egger | 11 | -0.37 | 0.39 | 0.366 | 0.69 | 0.321-1.482 |
| genus Ruminococcaceae NK4A214 group | Weighted median | 11 | -0.1 | 0.13 | 0.432 | 0.903 | 0.699-1.166 |
| genus Ruminococcaceae NK4A214 group | Weighted mode | 11 | -0.07 | 0.19 | 0.712 | 0.932 | 0.647-1.342 |
| genus Ruminococcaceae UCG003 | IVW | 10 | -0.19 | 0.1 | 0.048 | 0.824 | 0.68-0.999 |
| genus Ruminococcaceae UCG003 | MR Egger | 10 | 0.147 | 0.34 | 0.672 | 1.159 | 0.601-2.235 |
| genus Ruminococcaceae UCG003 | Weighted median | 10 | -0.05 | 0.12 | 0.694 | 0.955 | 0.759-1.201 |
| genus Ruminococcaceae UCG003 | Weighted mode | 10 | -0.01 | 0.15 | 0.952 | 0.991 | 0.742-1.324 |
| genus Ruminococcaceae UCG004 | IVW | 8 | 0 | 0.09 | 0.998 | 1 | 0.842-1.188 |
| genus Ruminococcaceae UCG004 | MR Egger | 8 | -0.1 | 0.42 | 0.82 | 0.906 | 0.4-2.05 |
| genus Ruminococcaceae UCG004 | Weighted median | 8 | -0.01 | 0.12 | 0.946 | 0.992 | 0.79-1.246 |
| genus Ruminococcaceae UCG004 | Weighted mode | 8 | 0.005 | 0.16 | 0.976 | 1.005 | 0.741-1.364 |
| genus Ruminococcaceae UCG005 | IVW | 11 | 0.17 | 0.09 | 0.066 | 1.186 | 0.989-1.422 |
| genus Ruminococcaceae UCG005 | MR Egger | 11 | 0.375 | 0.33 | 0.279 | 1.456 | 0.768-2.758 |
| genus Ruminococcaceae UCG005 | Weighted median | 11 | 0.176 | 0.13 | 0.166 | 1.193 | 0.929-1.531 |
| genus Ruminococcaceae UCG005 | Weighted mode | 11 | 0.194 | 0.2 | 0.359 | 1.214 | 0.818-1.801 |
| genus Ruminococcaceae UCG009 | IVW | 10 | -0.01 | 0.07 | 0.907 | 0.991 | 0.858-1.145 |
| genus Ruminococcaceae UCG009 | MR Egger | 10 | -0.12 | 0.45 | 0.8 | 0.889 | 0.369-2.141 |
| genus Ruminococcaceae UCG009 | Weighted median | 10 | 0.015 | 0.1 | 0.875 | 1.015 | 0.838-1.23 |
| genus Ruminococcaceae UCG009 | Weighted mode | 10 | 0.026 | 0.13 | 0.842 | 1.027 | 0.799-1.32 |
| genus Ruminococcaceae UCG010 | IVW | 5 | 0.076 | 0.14 | 0.583 | 1.079 | 0.823-1.414 |
| genus Ruminococcaceae UCG010 | MR Egger | 5 | 0.725 | 0.39 | 0.159 | 2.065 | 0.963-4.431 |
| genus Ruminococcaceae UCG010 | Weighted median | 5 | -0.06 | 0.17 | 0.722 | 0.941 | 0.671-1.319 |
| genus Ruminococcaceae UCG010 | Weighted mode | 5 | -0.16 | 0.27 | 0.586 | 0.851 | 0.499-1.453 |
| genus Ruminococcus1 | IVW | 7 | 0.233 | 0.14 | 0.097 | 1.262 | 0.959-1.661 |
| genus Ruminococcus1 | MR Egger | 7 | -0.65 | 0.52 | 0.265 | 0.523 | 0.19-1.439 |
| genus Ruminococcus1 | Weighted median | 7 | 0.272 | 0.18 | 0.131 | 1.313 | 0.922-1.87 |
| genus Ruminococcus1 | Weighted mode | 7 | 0.508 | 0.33 | 0.175 | 1.662 | 0.87-3.177 |
| genus Ruminococcus2 | IVW | 12 | 0.193 | 0.1 | 0.043 | 1.213 | 1.006-1.462 |
| genus Ruminococcus2 | MR Egger | 12 | 0.236 | 0.3 | 0.446 | 1.266 | 0.707-2.268 |
| genus Ruminococcus2 | Weighted median | 12 | 0.185 | 0.13 | 0.141 | 1.203 | 0.94-1.538 |
| genus Ruminococcus2 | Weighted mode | 12 | 0.18 | 0.18 | 0.326 | 1.198 | 0.849-1.689 |
| genus Ruminococcus gnavus group | IVW | 11 | -0.07 | 0.06 | 0.296 | 0.937 | 0.829-1.059 |
| genus Ruminococcus gnavus group | MR Egger | 11 | 0.157 | 0.31 | 0.623 | 1.17 | 0.639-2.141 |
| genus Ruminococcus gnavus group | Weighted median | 11 | -0.08 | 0.08 | 0.328 | 0.925 | 0.79-1.082 |
| genus Ruminococcus gnavus group | Weighted mode | 11 | -0.17 | 0.14 | 0.258 | 0.841 | 0.634-1.116 |
| genus Senegalimassilia | IVW | 3 | 0.094 | 0.12 | 0.42 | 1.099 | 0.874-1.381 |
| genus Senegalimassilia | MR Egger | 3 | -0.3 | 0.34 | 0.538 | 0.74 | 0.38-1.441 |
| genus Senegalimassilia | Weighted median | 3 | 0.073 | 0.15 | 0.621 | 1.075 | 0.807-1.433 |
| genus Senegalimassilia | Weighted mode | 3 | 0.019 | 0.21 | 0.935 | 1.019 | 0.682-1.524 |
| genus Turicibacter | IVW | 8 | 0.113 | 0.09 | 0.214 | 1.119 | 0.937-1.337 |
| genus Turicibacter | MR Egger | 8 | 0.293 | 0.55 | 0.615 | 1.34 | 0.454-3.95 |
| genus Turicibacter | Weighted median | 8 | 0.134 | 0.11 | 0.239 | 1.144 | 0.915-1.43 |
| genus Turicibacter | Weighted mode | 8 | 0.149 | 0.15 | 0.345 | 1.16 | 0.87-1.547 |
| genus Akkermansia | IVW | 8 | -0.01 | 0.1 | 0.902 | 0.988 | 0.814-1.198 |
| genus Akkermansia | MR Egger | 8 | 1.433 | 1.3 | 0.314 | 4.191 | 0.326-53.902 |
| genus Akkermansia | Weighted median | 8 | 0.024 | 0.12 | 0.843 | 1.024 | 0.809-1.297 |
| genus Akkermansia | Weighted mode | 8 | 0.041 | 0.18 | 0.828 | 1.042 | 0.73-1.488 |
| genus Alloprevotella | IVW | 6 | 0.115 | 0.06 | 0.055 | 1.122 | 0.998-1.262 |
| genus Alloprevotella | MR Egger | 6 | 0.581 | 0.59 | 0.378 | 1.787 | 0.566-5.649 |
| genus Alloprevotella | Weighted median | 6 | 0.11 | 0.08 | 0.141 | 1.117 | 0.964-1.293 |
| genus Alloprevotella | Weighted mode | 6 | 0.116 | 0.1 | 0.288 | 1.123 | 0.928-1.358 |
| genus Bacteroides | IVW | 8 | -0.02 | 0.13 | 0.903 | 0.985 | 0.769-1.261 |
| genus Bacteroides | MR Egger | 8 | -0.78 | 0.68 | 0.297 | 0.458 | 0.12-1.751 |
| genus Bacteroides | Weighted median | 8 | -0.12 | 0.17 | 0.493 | 0.891 | 0.641-1.239 |
| genus Bacteroides | Weighted mode | 8 | -0.13 | 0.22 | 0.59 | 0.881 | 0.568-1.367 |
| genus Barnesiella | IVW | 9 | 0.092 | 0.11 | 0.378 | 1.097 | 0.893-1.346 |
| genus Barnesiella | MR Egger | 9 | 0.205 | 0.3 | 0.521 | 1.227 | 0.678-2.221 |
| genus Barnesiella | Weighted median | 9 | 0 | 0.15 | 0.999 | 1 | 0.751-1.332 |
| genus Barnesiella | Weighted mode | 9 | -0.05 | 0.2 | 0.826 | 0.955 | 0.639-1.425 |
| genus Butyricicoccus | IVW | 7 | -0.17 | 0.17 | 0.33 | 0.846 | 0.603-1.185 |
| genus Butyricicoccus | MR Egger | 7 | -0.05 | 0.36 | 0.894 | 0.952 | 0.475-1.908 |
| genus Butyricicoccus | Weighted median | 7 | -0.05 | 0.14 | 0.717 | 0.95 | 0.722-1.251 |
| genus Butyricicoccus | Weighted mode | 7 | -0.03 | 0.14 | 0.827 | 0.969 | 0.736-1.274 |
| genus Butyricimonas | IVW | 12 | 0.069 | 0.08 | 0.363 | 1.072 | 0.923-1.245 |
| genus Butyricimonas | MR Egger | 12 | 0.121 | 0.26 | 0.658 | 1.128 | 0.673-1.892 |
| genus Butyricimonas | Weighted median | 12 | 0.02 | 0.1 | 0.842 | 1.021 | 0.834-1.249 |
| genus Butyricimonas | Weighted mode | 12 | 0 | 0.17 | 0.995 | 0.999 | 0.718-1.389 |
| genus Clostridium sensustricto1 | IVW | 5 | 0.062 | 0.15 | 0.686 | 1.064 | 0.787-1.44 |
| genus Clostridium sensustricto1 | MR Egger | 5 | -0.01 | 0.43 | 0.982 | 0.99 | 0.429-2.285 |
| genus Clostridium sensustricto1 | Weighted median | 5 | 0.078 | 0.19 | 0.679 | 1.081 | 0.748-1.562 |
| genus Clostridium sensustricto1 | Weighted mode | 5 | 0.12 | 0.23 | 0.636 | 1.127 | 0.712-1.784 |
| genus Coprococcus1 | IVW | 11 | -0.16 | 0.11 | 0.145 | 0.853 | 0.688-1.056 |
| genus Coprococcus1 | MR Egger | 11 | 0.021 | 0.34 | 0.954 | 1.021 | 0.52-2.004 |
| genus Coprococcus1 | Weighted median | 11 | -0.12 | 0.14 | 0.388 | 0.886 | 0.672-1.167 |
| genus Coprococcus1 | Weighted mode | 11 | -0.17 | 0.18 | 0.385 | 0.847 | 0.592-1.212 |
| genus Coprococcus2 | IVW | 8 | -0.13 | 0.14 | 0.348 | 0.877 | 0.668-1.153 |
| genus Coprococcus2 | MR Egger | 8 | -1.15 | 1.28 | 0.404 | 0.317 | 0.026-3.89 |
| genus Coprococcus2 | Weighted median | 8 | 0.045 | 0.16 | 0.774 | 1.046 | 0.772-1.416 |
| genus Coprococcus2 | Weighted mode | 8 | 0.046 | 0.22 | 0.844 | 1.047 | 0.676-1.621 |
| genus Eubacterium hallii group | IVW | 12 | 0.057 | 0.15 | 0.704 | 1.058 | 0.79-1.416 |
| genus Eubacterium hallii group | MR Egger | 12 | -0.05 | 0.37 | 0.898 | 0.953 | 0.467-1.948 |
| genus Eubacterium hallii group | Weighted median | 12 | 0.075 | 0.14 | 0.603 | 1.078 | 0.813-1.429 |
| genus Eubacterium hallii group | Weighted mode | 12 | -0.01 | 0.19 | 0.976 | 0.994 | 0.688-1.438 |
| genus Eubacterium nodatum group | IVW | 8 | -0.05 | 0.06 | 0.382 | 0.952 | 0.851-1.064 |
| genus Eubacterium nodatum group | MR Egger | 8 | -0.22 | 0.29 | 0.467 | 0.8 | 0.455-1.406 |
| genus Eubacterium nodatum group | Weighted median | 8 | -0.11 | 0.07 | 0.144 | 0.9 | 0.78-1.037 |
| genus Eubacterium nodatum group | Weighted mode | 8 | -0.17 | 0.13 | 0.23 | 0.844 | 0.655-1.087 |
| genus Eubacterium oxidoreducens group | IVW | 4 | -0.09 | 0.1 | 0.361 | 0.915 | 0.755-1.108 |
| genus Eubacterium oxidoreducens group | MR Egger | 4 | -0.13 | 0.29 | 0.706 | 0.882 | 0.5-1.555 |
| genus Eubacterium oxidoreducens group | Weighted median | 4 | -0.1 | 0.12 | 0.44 | 0.909 | 0.713-1.159 |
| genus Eubacterium oxidoreducens group | Weighted mode | 4 | -0.1 | 0.14 | 0.517 | 0.906 | 0.696-1.18 |
| genus Eubacterium ventriosum group | IVW | 12 | 0.084 | 0.12 | 0.464 | 1.088 | 0.868-1.364 |
| genus Eubacterium ventriosum group | MR Egger | 12 | -0.14 | 0.51 | 0.786 | 0.867 | 0.319-2.357 |
| genus Eubacterium ventriosum group | Weighted median | 12 | 0 | 0.13 | 0.985 | 0.998 | 0.775-1.283 |
| genus Eubacterium ventriosum group | Weighted mode | 12 | -0.05 | 0.25 | 0.849 | 0.952 | 0.583-1.556 |
| genus Eubacterium xylanophilum group | IVW | 6 | 0.013 | 0.12 | 0.913 | 1.013 | 0.797-1.289 |
| genus Eubacterium xylanophilum group | MR Egger | 6 | 0.672 | 0.41 | 0.178 | 1.959 | 0.873-4.395 |
| genus Eubacterium xylanophilum group | Weighted median | 6 | -0.02 | 0.16 | 0.904 | 0.981 | 0.721-1.335 |
| genus Eubacterium xylanophilum group | Weighted mode | 6 | -0.14 | 0.25 | 0.609 | 0.873 | 0.536-1.423 |
| genus Family XIII AD3011 group | IVW | 12 | 0.058 | 0.13 | 0.646 | 1.059 | 0.829-1.354 |
| genus Family XIII AD3011 group | MR Egger | 12 | -0.43 | 0.55 | 0.45 | 0.65 | 0.222-1.902 |
| genus Family XIII AD3011 group | Weighted median | 12 | -0.11 | 0.13 | 0.423 | 0.898 | 0.69-1.168 |
| genus Family XIII AD3011 group | Weighted mode | 12 | -0.14 | 0.21 | 0.519 | 0.871 | 0.58-1.307 |
| genus Family XIII UCG001 | IVW | 6 | 0.011 | 0.13 | 0.934 | 1.011 | 0.777-1.315 |
| genus Family XIII UCG001 | MR Egger | 6 | 0.104 | 0.78 | 0.9 | 1.11 | 0.242-5.083 |
| genus Family XIII UCG001 | Weighted median | 6 | 0.055 | 0.15 | 0.719 | 1.057 | 0.782-1.429 |
| genus Family XIII UCG001 | Weighted mode | 6 | 0.059 | 0.21 | 0.794 | 1.061 | 0.698-1.613 |
| genus Haemophilus | IVW | 6 | -0.02 | 0.11 | 0.876 | 0.983 | 0.787-1.227 |
| genus Haemophilus | MR Egger | 6 | -0.69 | 0.31 | 0.088 | 0.5 | 0.274-0.915 |
| genus Haemophilus | Weighted median | 6 | -0.09 | 0.12 | 0.482 | 0.917 | 0.719-1.168 |
| genus Haemophilus | Weighted mode | 6 | -0.15 | 0.19 | 0.452 | 0.859 | 0.596-1.238 |
| genus Holdemania | IVW | 10 | 0.027 | 0.08 | 0.742 | 1.028 | 0.873-1.21 |
| genus Holdemania | MR Egger | 10 | 0.443 | 0.4 | 0.303 | 1.557 | 0.708-3.427 |
| genus Holdemania | Weighted median | 10 | 0.107 | 0.11 | 0.324 | 1.113 | 0.9-1.375 |
| genus Holdemania | Weighted mode | 10 | 0.167 | 0.19 | 0.394 | 1.181 | 0.82-1.701 |
| genus Intestinibacter | IVW | 12 | -0.08 | 0.08 | 0.321 | 0.925 | 0.792-1.079 |
| genus Intestinibacter | MR Egger | 12 | -0.34 | 0.29 | 0.264 | 0.713 | 0.407-1.249 |
| genus Intestinibacter | Weighted median | 12 | -0.2 | 0.11 | 0.069 | 0.823 | 0.666-1.015 |
| genus Intestinibacter | Weighted mode | 12 | -0.22 | 0.16 | 0.191 | 0.8 | 0.585-1.095 |
| genus Lachnoclostridium | IVW | 12 | -0.14 | 0.1 | 0.163 | 0.87 | 0.716-1.058 |
| genus Lachnoclostridium | MR Egger | 12 | -0.42 | 0.36 | 0.273 | 0.656 | 0.321-1.338 |
| genus Lachnoclostridium | Weighted median | 12 | -0.09 | 0.13 | 0.502 | 0.916 | 0.708-1.184 |
| genus Lachnoclostridium | Weighted mode | 12 | -0.03 | 0.22 | 0.884 | 0.967 | 0.625-1.497 |
| genus Lachnospiraceae NC2004 group | IVW | 7 | -0.02 | 0.08 | 0.776 | 0.979 | 0.844-1.135 |
| genus Lachnospiraceae NC2004 group | MR Egger | 7 | -0.19 | 0.31 | 0.576 | 0.829 | 0.448-1.533 |
| genus Lachnospiraceae NC2004 group | Weighted median | 7 | 0.009 | 0.1 | 0.933 | 1.009 | 0.825-1.234 |
| genus Lachnospiraceae NC2004 group | Weighted mode | 7 | 0.068 | 0.17 | 0.693 | 1.071 | 0.774-1.481 |
| genus Lachnospiraceae ND3007 group | IVW | 3 | 0 | 0.2 | 0.992 | 0.998 | 0.68-1.464 |
| genus Lachnospiraceae ND3007 group | MR Egger | 3 | 2.003 | 3.59 | 0.676 | 7.412 | 0.006-8496.361 |
| genus Lachnospiraceae ND3007 group | Weighted median | 3 | 0.096 | 0.24 | 0.691 | 1.101 | 0.685-1.769 |
| genus Lachnospiraceae ND3007 group | Weighted mode | 3 | 0.167 | 0.31 | 0.64 | 1.182 | 0.648-2.158 |
| genus Lachnospiraceae NK4A136 group | IVW | 14 | 0.01 | 0.1 | 0.915 | 1.01 | 0.837-1.219 |
| genus Lachnospiraceae NK4A136 group | MR Egger | 14 | -0.13 | 0.24 | 0.614 | 0.883 | 0.55-1.416 |
| genus Lachnospiraceae NK4A136 group | Weighted median | 14 | -0.07 | 0.13 | 0.608 | 0.937 | 0.732-1.2 |
| genus Lachnospiraceae NK4A136 group | Weighted mode | 14 | -0.09 | 0.15 | 0.581 | 0.918 | 0.683-1.235 |
| genus Lactococcus | IVW | 8 | 0.034 | 0.06 | 0.581 | 1.034 | 0.918-1.165 |
| genus Lactococcus | MR Egger | 8 | -0.13 | 0.29 | 0.665 | 0.876 | 0.495-1.55 |
| genus Lactococcus | Weighted median | 8 | 0.022 | 0.08 | 0.783 | 1.022 | 0.876-1.193 |
| genus Lactococcus | Weighted mode | 8 | -0.03 | 0.13 | 0.85 | 0.975 | 0.757-1.256 |
| genus Methanobrevibacter | IVW | 5 | 0.067 | 0.07 | 0.332 | 1.07 | 0.934-1.226 |
| genus Methanobrevibacter | MR Egger | 5 | 0.183 | 0.28 | 0.559 | 1.2 | 0.695-2.072 |
| genus Methanobrevibacter | Weighted median | 5 | 0.061 | 0.08 | 0.467 | 1.062 | 0.903-1.25 |
| genus Methanobrevibacter | Weighted mode | 5 | 0.085 | 0.11 | 0.466 | 1.089 | 0.885-1.339 |
| genus Odoribacter | IVW | 4 | 0.162 | 0.2 | 0.427 | 1.176 | 0.788-1.754 |
| genus Odoribacter | MR Egger | 4 | -1.57 | 0.93 | 0.234 | 0.208 | 0.033-1.294 |
| genus Odoribacter | Weighted median | 4 | 0.142 | 0.22 | 0.516 | 1.152 | 0.751-1.767 |
| genus Odoribacter | Weighted mode | 4 | 0.121 | 0.31 | 0.725 | 1.129 | 0.611-2.085 |
| genus Oscillospira | IVW | 7 | -0.06 | 0.14 | 0.672 | 0.944 | 0.725-1.23 |
| genus Oscillospira | MR Egger | 7 | -0.7 | 0.55 | 0.263 | 0.497 | 0.168-1.471 |
| genus Oscillospira | Weighted median | 7 | 0.043 | 0.15 | 0.782 | 1.043 | 0.773-1.409 |
| genus Oscillospira | Weighted mode | 7 | 0.114 | 0.19 | 0.578 | 1.121 | 0.766-1.64 |
| genus Oxalobacter | IVW | 9 | -0.03 | 0.06 | 0.66 | 0.975 | 0.873-1.09 |
| genus Oxalobacter | MR Egger | 9 | -0.23 | 0.28 | 0.425 | 0.791 | 0.46-1.361 |
| genus Oxalobacter | Weighted median | 9 | -0.04 | 0.08 | 0.563 | 0.957 | 0.823-1.112 |
| genus Oxalobacter | Weighted mode | 9 | 0.009 | 0.13 | 0.946 | 1.009 | 0.787-1.294 |
| genus Prevotella7 | IVW | 10 | -0.1 | 0.05 | 0.059 | 0.906 | 0.818-1.004 |
| genus Prevotella7 | MR Egger | 10 | -0.68 | 0.3 | 0.05 | 0.505 | 0.283-0.903 |
| genus Prevotella7 | Weighted median | 10 | -0.06 | 0.07 | 0.403 | 0.942 | 0.818-1.084 |
| genus Prevotella7 | Weighted mode | 10 | -0.06 | 0.12 | 0.67 | 0.947 | 0.742-1.207 |
| genus Roseburia | IVW | 11 | 0.227 | 0.11 | 0.038 | 1.255 | 1.012-1.556 |
| genus Roseburia | MR Egger | 11 | 0.796 | 0.47 | 0.124 | 2.216 | 0.884-5.555 |
| genus Roseburia | Weighted median | 11 | 0.256 | 0.15 | 0.086 | 1.292 | 0.965-1.73 |
| genus Roseburia | Weighted mode | 11 | 0.295 | 0.23 | 0.22 | 1.343 | 0.863-2.088 |
| genus Ruminiclostridium5 | IVW | 9 | -0.36 | 0.12 | 0.002 | 0.695 | 0.554-0.871 |
| genus Ruminiclostridium5 | MR Egger | 9 | -0.15 | 0.56 | 0.802 | 0.863 | 0.286-2.609 |
| genus Ruminiclostridium5 | Weighted median | 9 | -0.34 | 0.17 | 0.043 | 0.715 | 0.517-0.99 |
| genus Ruminiclostridium5 | Weighted mode | 9 | -0.33 | 0.25 | 0.23 | 0.722 | 0.441-1.181 |
| genus Ruminococcaceae UCG011 | IVW | 7 | -0.03 | 0.07 | 0.64 | 0.966 | 0.836-1.116 |
| genus Ruminococcaceae UCG011 | MR Egger | 7 | 0.154 | 0.39 | 0.71 | 1.167 | 0.542-2.513 |
| genus Ruminococcaceae UCG011 | Weighted median | 7 | -0.07 | 0.08 | 0.339 | 0.931 | 0.804-1.078 |
| genus Ruminococcaceae UCG011 | Weighted mode | 7 | -0.08 | 0.11 | 0.512 | 0.928 | 0.751-1.146 |
| genus Ruminococcaceae UCG013 | IVW | 9 | -0.04 | 0.17 | 0.814 | 0.961 | 0.688-1.341 |
| genus Ruminococcaceae UCG013 | MR Egger | 9 | 0.258 | 0.81 | 0.759 | 1.295 | 0.265-6.333 |
| genus Ruminococcaceae UCG013 | Weighted median | 9 | 0.159 | 0.15 | 0.284 | 1.172 | 0.877-1.568 |
| genus Ruminococcaceae UCG013 | Weighted mode | 9 | 0.164 | 0.2 | 0.445 | 1.178 | 0.79-1.755 |
| genus Ruminococcaceae UCG014 | IVW | 10 | -0.07 | 0.11 | 0.526 | 0.934 | 0.756-1.153 |
| genus Ruminococcaceae UCG014 | MR Egger | 10 | -0.02 | 0.41 | 0.971 | 0.985 | 0.443-2.191 |
| genus Ruminococcaceae UCG014 | Weighted median | 10 | -0.07 | 0.13 | 0.614 | 0.935 | 0.72-1.214 |
| genus Ruminococcaceae UCG014 | Weighted mode | 10 | -0.21 | 0.2 | 0.327 | 0.809 | 0.542-1.208 |
| genus Ruminococcus gauvreauii group | IVW | 11 | -0.08 | 0.12 | 0.524 | 0.925 | 0.727-1.177 |
| genus Ruminococcus gauvreauii group | MR Egger | 11 | -0.2 | 0.6 | 0.745 | 0.819 | 0.255-2.63 |
| genus Ruminococcus gauvreauii group | Weighted median | 11 | 0.031 | 0.13 | 0.813 | 1.032 | 0.796-1.337 |
| genus Ruminococcus gauvreauii group | Weighted mode | 11 | 0.165 | 0.22 | 0.475 | 1.179 | 0.764-1.82 |
| genus Streptococcus | IVW | 14 | -0.11 | 0.12 | 0.347 | 0.896 | 0.713-1.127 |
| genus Streptococcus | MR Egger | 14 | 0.36 | 0.41 | 0.401 | 1.433 | 0.638-3.216 |
| genus Streptococcus | Weighted median | 14 | 0.093 | 0.13 | 0.482 | 1.098 | 0.846-1.424 |
| genus Streptococcus | Weighted mode | 14 | 0.132 | 0.18 | 0.477 | 1.141 | 0.802-1.623 |
| genus Tyzzerella3 | IVW | 10 | 0.035 | 0.06 | 0.578 | 1.036 | 0.915-1.173 |
| genus Tyzzerella3 | MR Egger | 10 | 0.075 | 0.4 | 0.856 | 1.078 | 0.491-2.367 |
| genus Tyzzerella3 | Weighted median | 10 | 0.082 | 0.08 | 0.316 | 1.085 | 0.925-1.273 |
| genus Tyzzerella3 | Weighted mode | 10 | 0.081 | 0.11 | 0.482 | 1.084 | 0.873-1.347 |
| genus Veillonella | IVW | 7 | 0.2 | 0.14 | 0.158 | 1.222 | 0.925-1.614 |
| genus Veillonella | MR Egger | 7 | -0.25 | 1.22 | 0.845 | 0.777 | 0.071-8.511 |
| genus Veillonella | Weighted median | 7 | 0.265 | 0.15 | 0.076 | 1.303 | 0.972-1.747 |
| genus Veillonella | Weighted mode | 7 | 0.294 | 0.2 | 0.182 | 1.341 | 0.916-1.965 |
| genus Victivallis | IVW | 8 | 0.024 | 0.06 | 0.674 | 1.024 | 0.916-1.145 |
| genus Victivallis | MR Egger | 8 | -0.55 | 0.47 | 0.283 | 0.576 | 0.23-1.441 |
| genus Victivallis | Weighted median | 8 | 0.042 | 0.07 | 0.562 | 1.043 | 0.904-1.203 |
| genus Victivallis | Weighted mode | 8 | 0.046 | 0.09 | 0.625 | 1.047 | 0.878-1.249 |
| genus Alistipes | IVW | 11 | 0.047 | 0.11 | 0.659 | 1.048 | 0.851-1.29 |
| genus Alistipes | MR Egger | 11 | 0.382 | 0.49 | 0.453 | 1.465 | 0.564-3.804 |
| genus Alistipes | Weighted median | 11 | 0.184 | 0.14 | 0.193 | 1.202 | 0.911-1.584 |
| genus Alistipes | Weighted mode | 11 | 0.261 | 0.22 | 0.252 | 1.299 | 0.852-1.98 |
| genus Allisonella | IVW | 6 | 0.03 | 0.06 | 0.612 | 1.03 | 0.919-1.155 |
| genus Allisonella | MR Egger | 6 | 0.169 | 0.36 | 0.662 | 1.185 | 0.586-2.396 |
| genus Allisonella | Weighted median | 6 | 0.013 | 0.07 | 0.859 | 1.013 | 0.881-1.164 |
| genus Allisonella | Weighted mode | 6 | 0.013 | 0.1 | 0.904 | 1.013 | 0.834-1.23 |
| genus Anaerotruncus | IVW | 11 | -0.32 | 0.1 | 0.002 | 0.726 | 0.592-0.89 |
| genus Anaerotruncus | MR Egger | 11 | -0.1 | 0.39 | 0.813 | 0.909 | 0.421-1.963 |
| genus Anaerotruncus | Weighted median | 11 | -0.4 | 0.14 | 0.006 | 0.673 | 0.508-0.891 |
| genus Anaerotruncus | Weighted mode | 11 | -0.41 | 0.21 | 0.082 | 0.663 | 0.437-1.005 |
| genus Bifidobacterium | IVW | 10 | -0.07 | 0.12 | 0.534 | 0.931 | 0.742-1.167 |
| genus Bifidobacterium | MR Egger | 10 | 0.745 | 0.33 | 0.055 | 2.106 | 1.098-4.04 |
| genus Bifidobacterium | Weighted median | 10 | -0.18 | 0.14 | 0.197 | 0.832 | 0.629-1.1 |
| genus Bifidobacterium | Weighted mode | 10 | -0.29 | 0.28 | 0.333 | 0.75 | 0.433-1.301 |
| genus Bilophila | IVW | 13 | 0.085 | 0.08 | 0.31 | 1.088 | 0.924-1.281 |
| genus Bilophila | MR Egger | 13 | 0.229 | 0.4 | 0.575 | 1.257 | 0.578-2.734 |
| genus Bilophila | Weighted median | 13 | 0.081 | 0.12 | 0.488 | 1.085 | 0.862-1.364 |
| genus Bilophila | Weighted mode | 13 | -0.12 | 0.24 | 0.623 | 0.884 | 0.548-1.426 |
| genus Candidatus Soleaferrea | IVW | 9 | -0.06 | 0.07 | 0.377 | 0.942 | 0.825-1.076 |
| genus Candidatus Soleaferrea | MR Egger | 9 | 0.426 | 0.74 | 0.584 | 1.53 | 0.358-6.54 |
| genus Candidatus Soleaferrea | Weighted median | 9 | 0.033 | 0.1 | 0.733 | 1.033 | 0.856-1.247 |
| genus Candidatus Soleaferrea | Weighted mode | 9 | 0.043 | 0.14 | 0.763 | 1.044 | 0.797-1.366 |
| genus Coprobacter | IVW | 8 | -0.04 | 0.08 | 0.643 | 0.964 | 0.824-1.127 |
| genus Coprobacter | MR Egger | 8 | 0.034 | 0.43 | 0.939 | 1.035 | 0.446-2.402 |
| genus Coprobacter | Weighted median | 8 | -0.04 | 0.1 | 0.672 | 0.958 | 0.785-1.169 |
| genus Coprobacter | Weighted mode | 8 | -0.07 | 0.13 | 0.615 | 0.933 | 0.722-1.206 |
| genus Coprococcus3 | IVW | 7 | -0.08 | 0.15 | 0.601 | 0.926 | 0.692-1.237 |
| genus Coprococcus3 | MR Egger | 7 | 0.638 | 0.43 | 0.195 | 1.892 | 0.821-4.361 |
| genus Coprococcus3 | Weighted median | 7 | 0.043 | 0.18 | 0.809 | 1.044 | 0.736-1.481 |
| genus Coprococcus3 | Weighted mode | 7 | 0.197 | 0.26 | 0.479 | 1.217 | 0.73-2.029 |
| genus Dorea | IVW | 9 | 0.1 | 0.11 | 0.353 | 1.105 | 0.895-1.364 |
| genus Dorea | MR Egger | 9 | 0.432 | 0.29 | 0.185 | 1.541 | 0.865-2.744 |
| genus Dorea | Weighted median | 9 | 0.099 | 0.15 | 0.518 | 1.104 | 0.818-1.49 |
| genus Dorea | Weighted mode | 9 | 0.1 | 0.23 | 0.68 | 1.106 | 0.698-1.75 |
| genus Erysipelatoclostridium | IVW | 15 | 0.006 | 0.07 | 0.927 | 1.006 | 0.876-1.156 |
| genus Erysipelatoclostridium | MR Egger | 15 | -0.24 | 0.32 | 0.466 | 0.787 | 0.421-1.47 |
| genus Erysipelatoclostridium | Weighted median | 15 | 0.014 | 0.1 | 0.888 | 1.014 | 0.838-1.227 |
| genus Erysipelatoclostridium | Weighted mode | 15 | -0.03 | 0.16 | 0.851 | 0.97 | 0.711-1.323 |
| genus Escherichia Shigella | IVW | 7 | -0.02 | 0.12 | 0.893 | 0.984 | 0.781-1.241 |
| genus Escherichia Shigella | MR Egger | 7 | -0.37 | 0.59 | 0.554 | 0.689 | 0.218-2.176 |
| genus Escherichia Shigella | Weighted median | 7 | 0.057 | 0.15 | 0.71 | 1.058 | 0.785-1.427 |
| genus Escherichia Shigella | Weighted mode | 7 | 0.142 | 0.23 | 0.566 | 1.152 | 0.729-1.82 |
| genus Eubacterium brachy group | IVW | 8 | 0.047 | 0.07 | 0.486 | 1.048 | 0.919-1.195 |
| genus Eubacterium brachy group | MR Egger | 8 | -0.29 | 0.32 | 0.406 | 0.752 | 0.402-1.406 |
| genus Eubacterium brachy group | Weighted median | 8 | 0.001 | 0.08 | 0.988 | 1.001 | 0.854-1.173 |
| genus Eubacterium brachy group | Weighted mode | 8 | 0.017 | 0.11 | 0.879 | 1.017 | 0.828-1.249 |
| genus Eubacterium coprostanoligenes group | IVW | 11 | 0 | 0.15 | 0.989 | 0.998 | 0.744-1.338 |
| genus Eubacterium coprostanoligenes group | MR Egger | 11 | -0.65 | 0.72 | 0.391 | 0.523 | 0.128-2.143 |
| genus Eubacterium coprostanoligenes group | Weighted median | 11 | -0.05 | 0.15 | 0.768 | 0.956 | 0.709-1.29 |
| genus Eubacterium coprostanoligenes group | Weighted mode | 11 | -0.25 | 0.26 | 0.351 | 0.775 | 0.466-1.29 |
| genus Eubacterium eligens group | IVW | 7 | 0.164 | 0.17 | 0.336 | 1.179 | 0.843-1.647 |
| genus Eubacterium eligens group | MR Egger | 7 | 0.47 | 0.74 | 0.553 | 1.6 | 0.376-6.81 |
| genus Eubacterium eligens group | Weighted median | 7 | 0.036 | 0.2 | 0.859 | 1.037 | 0.695-1.548 |
| genus Eubacterium eligens group | Weighted mode | 7 | -0.09 | 0.27 | 0.76 | 0.918 | 0.542-1.554 |
| genus Eubacterium ruminantium group | IVW | 15 | -0.07 | 0.07 | 0.308 | 0.935 | 0.823-1.064 |
| genus Eubacterium ruminantium group | MR Egger | 15 | 0.402 | 0.23 | 0.103 | 1.495 | 0.954-2.342 |
| genus Eubacterium ruminantium group | Weighted median | 15 | -0.03 | 0.08 | 0.758 | 0.976 | 0.834-1.142 |
| genus Eubacterium ruminantium group | Weighted mode | 15 | -0.05 | 0.13 | 0.715 | 0.952 | 0.737-1.231 |
| genus Faecalibacterium | IVW | 7 | -0.14 | 0.13 | 0.279 | 0.873 | 0.684-1.116 |
| genus Faecalibacterium | MR Egger | 7 | -0.02 | 0.28 | 0.957 | 0.984 | 0.569-1.703 |
| genus Faecalibacterium | Weighted median | 7 | -0.13 | 0.16 | 0.385 | 0.874 | 0.646-1.184 |
| genus Faecalibacterium | Weighted mode | 7 | -0.14 | 0.22 | 0.551 | 0.868 | 0.56-1.346 |
| genus Flavonifractor | IVW | 4 | -0.21 | 0.12 | 0.083 | 0.81 | 0.638-1.028 |
| genus Flavonifractor | MR Egger | 4 | 0.67 | 0.65 | 0.409 | 1.955 | 0.55-6.95 |
| genus Flavonifractor | Weighted median | 4 | -0.27 | 0.15 | 0.063 | 0.761 | 0.571-1.015 |
| genus Flavonifractor | Weighted mode | 4 | -0.29 | 0.21 | 0.261 | 0.746 | 0.492-1.13 |
| genus Holdemanella | IVW | 9 | 0.116 | 0.1 | 0.254 | 1.123 | 0.92-1.372 |
| genus Holdemanella | MR Egger | 9 | 1.01 | 0.3 | 0.012 | 2.747 | 1.526-4.943 |
| genus Holdemanella | Weighted median | 9 | -0.01 | 0.11 | 0.96 | 0.995 | 0.807-1.226 |
| genus Holdemanella | Weighted mode | 9 | -0.05 | 0.14 | 0.733 | 0.95 | 0.716-1.261 |
| genus Howardella | IVW | 9 | -0.02 | 0.05 | 0.769 | 0.985 | 0.888-1.092 |
| genus Howardella | MR Egger | 9 | 0.032 | 0.22 | 0.892 | 1.032 | 0.666-1.6 |
| genus Howardella | Weighted median | 9 | -0.05 | 0.07 | 0.443 | 0.947 | 0.824-1.088 |
| genus Howardella | Weighted mode | 9 | -0.07 | 0.11 | 0.518 | 0.929 | 0.749-1.151 |
| genus Hungatella | IVW | 4 | -0.04 | 0.1 | 0.682 | 0.959 | 0.786-1.17 |
| genus Hungatella | MR Egger | 4 | 0.189 | 1.87 | 0.929 | 1.208 | 0.031-47.246 |
| genus Hungatella | Weighted median | 4 | -0.04 | 0.11 | 0.734 | 0.962 | 0.769-1.203 |
| genus Hungatella | Weighted mode | 4 | 0.076 | 0.18 | 0.702 | 1.079 | 0.757-1.538 |
| genus Lachnospiraceae UCG004 | IVW | 12 | 0.043 | 0.09 | 0.625 | 1.044 | 0.878-1.241 |
| genus Lachnospiraceae UCG004 | MR Egger | 12 | 0 | 0.34 | 0.999 | 0.999 | 0.511-1.954 |
| genus Lachnospiraceae UCG004 | Weighted median | 12 | 0.016 | 0.12 | 0.895 | 1.016 | 0.804-1.284 |
| genus Lachnospiraceae UCG004 | Weighted mode | 12 | -0.01 | 0.19 | 0.977 | 0.994 | 0.686-1.442 |
| genus Lachnospiraceae UCG010 | IVW | 7 | -0.04 | 0.1 | 0.674 | 0.96 | 0.792-1.163 |
| genus Lachnospiraceae UCG010 | MR Egger | 7 | -0.3 | 0.23 | 0.262 | 0.744 | 0.47-1.177 |
| genus Lachnospiraceae UCG010 | Weighted median | 7 | -0.12 | 0.13 | 0.354 | 0.886 | 0.686-1.144 |
| genus Lachnospiraceae UCG010 | Weighted mode | 7 | -0.13 | 0.15 | 0.422 | 0.876 | 0.649-1.183 |
| genus Olsenella | IVW | 10 | 0.029 | 0.08 | 0.717 | 1.029 | 0.881-1.203 |
| genus Olsenella | MR Egger | 10 | 0.231 | 0.32 | 0.495 | 1.26 | 0.668-2.377 |
| genus Olsenella | Weighted median | 10 | 0.039 | 0.09 | 0.652 | 1.039 | 0.879-1.23 |
| genus Olsenella | Weighted mode | 10 | 0.114 | 0.13 | 0.397 | 1.121 | 0.872-1.442 |
| genus Oscillibacter | IVW | 11 | -0.04 | 0.08 | 0.628 | 0.96 | 0.816-1.131 |
| genus Oscillibacter | MR Egger | 11 | 0.226 | 0.34 | 0.518 | 1.254 | 0.648-2.425 |
| genus Oscillibacter | Weighted median | 11 | 0 | 0.1 | 0.987 | 0.998 | 0.827-1.206 |
| genus Oscillibacter | Weighted mode | 11 | 0.046 | 0.16 | 0.778 | 1.047 | 0.769-1.425 |
| genus Paraprevotella | IVW | 10 | 0.006 | 0.07 | 0.933 | 1.006 | 0.884-1.144 |
| genus Paraprevotella | MR Egger | 10 | 0.325 | 0.24 | 0.217 | 1.384 | 0.86-2.228 |
| genus Paraprevotella | Weighted median | 10 | -0.05 | 0.09 | 0.535 | 0.947 | 0.797-1.125 |
| genus Paraprevotella | Weighted mode | 10 | -0.13 | 0.15 | 0.41 | 0.877 | 0.65-1.182 |
| genus Parasutterella | IVW | 12 | -0.11 | 0.09 | 0.193 | 0.896 | 0.758-1.057 |
| genus Parasutterella | MR Egger | 12 | -0.47 | 0.24 | 0.08 | 0.627 | 0.392-1.002 |
| genus Parasutterella | Weighted median | 12 | -0.05 | 0.11 | 0.648 | 0.949 | 0.759-1.187 |
| genus Parasutterella | Weighted mode | 12 | -0.36 | 0.21 | 0.115 | 0.699 | 0.464-1.054 |
| ebi-a-GCST90017043 | IVW | 6 | -0.1 | 0.15 | 0.483 | 0.901 | 0.673-1.206 |
| ebi-a-GCST90017043 | MR Egger | 6 | 0 | 0.91 | 0.999 | 0.998 | 0.167-5.979 |
| ebi-a-GCST90017043 | Weighted median | 6 | -0.19 | 0.14 | 0.184 | 0.829 | 0.628-1.094 |
| ebi-a-GCST90017043 | Weighted mode | 6 | -0.2 | 0.21 | 0.372 | 0.817 | 0.545-1.224 |
| genus Prevotella9 | IVW | 11 | -0.13 | 0.09 | 0.148 | 0.882 | 0.743-1.046 |
| genus Prevotella9 | MR Egger | 11 | 0.18 | 0.52 | 0.735 | 1.197 | 0.436-3.284 |
| genus Prevotella9 | Weighted median | 11 | -0.09 | 0.12 | 0.432 | 0.912 | 0.725-1.148 |
| genus Prevotella9 | Weighted mode | 11 | -0.02 | 0.2 | 0.925 | 0.981 | 0.668-1.441 |
| genus Rikenellaceae RC9 gut group | IVW | 9 | -0.01 | 0.05 | 0.803 | 0.987 | 0.89-1.094 |
| genus Rikenellaceae RC9 gut group | MR Egger | 9 | 0.029 | 0.32 | 0.93 | 1.03 | 0.547-1.938 |
| genus Rikenellaceae RC9 gut group | Weighted median | 9 | -0.04 | 0.07 | 0.556 | 0.961 | 0.841-1.097 |
| genus Rikenellaceae RC9 gut group | Weighted mode | 9 | -0.07 | 0.1 | 0.506 | 0.933 | 0.766-1.135 |
| genus Ruminiclostridium9 | IVW | 5 | -0.1 | 0.14 | 0.473 | 0.903 | 0.682-1.194 |
| genus Ruminiclostridium9 | MR Egger | 5 | 0.555 | 1.1 | 0.648 | 1.741 | 0.203-14.969 |
| genus Ruminiclostridium9 | Weighted median | 5 | -0.12 | 0.18 | 0.483 | 0.883 | 0.624-1.25 |
| genus Ruminiclostridium9 | Weighted mode | 5 | -0.19 | 0.22 | 0.442 | 0.828 | 0.536-1.278 |
| genus Ruminococcaceae UCG002 | IVW | 16 | -0.04 | 0.08 | 0.675 | 0.966 | 0.82-1.137 |
| genus Ruminococcaceae UCG002 | MR Egger | 16 | -0.4 | 0.32 | 0.229 | 0.67 | 0.359-1.25 |
| genus Ruminococcaceae UCG002 | Weighted median | 16 | -0.05 | 0.12 | 0.678 | 0.953 | 0.757-1.198 |
| genus Ruminococcaceae UCG002 | Weighted mode | 16 | 0.191 | 0.21 | 0.377 | 1.211 | 0.802-1.827 |
| genus Ruminococcus torques group | IVW | 7 | 0.149 | 0.23 | 0.521 | 1.161 | 0.736-1.829 |
| genus Ruminococcus torques group | MR Egger | 7 | 0.298 | 0.82 | 0.732 | 1.347 | 0.27-6.732 |
| genus Ruminococcus torques group | Weighted median | 7 | 0.046 | 0.19 | 0.806 | 1.047 | 0.725-1.513 |
| genus Ruminococcus torques group | Weighted mode | 7 | 0.026 | 0.27 | 0.928 | 1.026 | 0.603-1.744 |
| genus Sellimonas | IVW | 8 | -0.02 | 0.06 | 0.797 | 0.986 | 0.882-1.101 |
| genus Sellimonas | MR Egger | 8 | 0.505 | 0.33 | 0.181 | 1.657 | 0.861-3.189 |
| genus Sellimonas | Weighted median | 8 | -0.06 | 0.07 | 0.41 | 0.941 | 0.814-1.088 |
| genus Sellimonas | Weighted mode | 8 | -0.09 | 0.1 | 0.387 | 0.912 | 0.749-1.109 |
| genus Slackia | IVW | 6 | 0.013 | 0.08 | 0.875 | 1.013 | 0.859-1.195 |
| genus Slackia | MR Egger | 6 | 0.005 | 0.55 | 0.994 | 1.005 | 0.341-2.963 |
| genus Slackia | Weighted median | 6 | -0.04 | 0.1 | 0.722 | 0.964 | 0.786-1.181 |
| genus Slackia | Weighted mode | 6 | -0.05 | 0.18 | 0.787 | 0.951 | 0.671-1.347 |
| genus Subdoligranulum | IVW | 8 | -0.03 | 0.13 | 0.798 | 0.967 | 0.75-1.248 |
| genus Subdoligranulum | MR Egger | 8 | -0.07 | 0.38 | 0.86 | 0.932 | 0.44-1.973 |
| genus Subdoligranulum | Weighted median | 8 | -0.08 | 0.16 | 0.619 | 0.923 | 0.674-1.264 |
| genus Subdoligranulum | Weighted mode | 8 | -0.12 | 0.2 | 0.578 | 0.889 | 0.598-1.321 |
| genus Sutterella | IVW | 11 | -0.02 | 0.11 | 0.87 | 0.983 | 0.796-1.213 |
| genus Sutterella | MR Egger | 11 | 0.226 | 1.23 | 0.858 | 1.254 | 0.113-13.94 |
| genus Sutterella | Weighted median | 11 | 0.038 | 0.13 | 0.765 | 1.039 | 0.811-1.33 |
| genus Sutterella | Weighted mode | 11 | 0.27 | 0.26 | 0.331 | 1.31 | 0.78-2.198 |
| genus Terrisporobacter | IVW | 5 | -0.09 | 0.1 | 0.354 | 0.913 | 0.753-1.107 |
| genus Terrisporobacter | MR Egger | 5 | -0.26 | 0.34 | 0.496 | 0.77 | 0.397-1.494 |
| genus Terrisporobacter | Weighted median | 5 | -0.07 | 0.12 | 0.561 | 0.933 | 0.737-1.18 |
| genus Terrisporobacter | Weighted mode | 5 | -0.05 | 0.15 | 0.748 | 0.949 | 0.706-1.276 |
| order Actinomycetales | IVW | 5 | -0.09 | 0.11 | 0.406 | 0.917 | 0.747-1.125 |
| order Actinomycetales | MR Egger | 5 | -0.19 | 0.31 | 0.59 | 0.829 | 0.45-1.526 |
| order Actinomycetales | Weighted median | 5 | -0.07 | 0.13 | 0.598 | 0.933 | 0.72-1.208 |
| order Actinomycetales | Weighted mode | 5 | -0.06 | 0.18 | 0.764 | 0.944 | 0.664-1.342 |
| order Bacillales | IVW | 8 | 0.073 | 0.05 | 0.172 | 1.076 | 0.968-1.196 |
| order Bacillales | MR Egger | 8 | 0.308 | 0.28 | 0.319 | 1.36 | 0.78-2.371 |
| order Bacillales | Weighted median | 8 | 0.038 | 0.07 | 0.593 | 1.039 | 0.904-1.193 |
| order Bacillales | Weighted mode | 8 | 0.013 | 0.11 | 0.909 | 1.013 | 0.819-1.253 |
| order Bacteroidales | IVW | 11 | -0.05 | 0.1 | 0.624 | 0.953 | 0.786-1.155 |
| order Bacteroidales | MR Egger | 11 | -0.07 | 0.21 | 0.746 | 0.932 | 0.617-1.408 |
| order Bacteroidales | Weighted median | 11 | -0.01 | 0.13 | 0.965 | 0.994 | 0.767-1.289 |
| order Bacteroidales | Weighted mode | 11 | -0.05 | 0.16 | 0.766 | 0.951 | 0.69-1.311 |
| order Bifidobacteriales | IVW | 12 | -0.07 | 0.11 | 0.55 | 0.935 | 0.751-1.165 |
| order Bifidobacteriales | MR Egger | 12 | 0.907 | 0.32 | 0.018 | 2.478 | 1.32-4.65 |
| order Bifidobacteriales | Weighted median | 12 | -0.05 | 0.14 | 0.739 | 0.954 | 0.724-1.257 |
| order Bifidobacteriales | Weighted mode | 12 | -0.19 | 0.27 | 0.504 | 0.828 | 0.486-1.413 |
| order Burkholderiales | IVW | 10 | -0.22 | 0.1 | 0.032 | 0.8 | 0.653-0.981 |
| order Burkholderiales | MR Egger | 10 | -0.36 | 0.39 | 0.385 | 0.701 | 0.329-1.495 |
| order Burkholderiales | Weighted median | 10 | -0.24 | 0.14 | 0.078 | 0.787 | 0.604-1.027 |
| order Burkholderiales | Weighted mode | 10 | -0.29 | 0.21 | 0.201 | 0.752 | 0.502-1.127 |
| order Clostridiales | IVW | 12 | 0.028 | 0.09 | 0.769 | 1.028 | 0.854-1.237 |
| order Clostridiales | MR Egger | 12 | 0.065 | 0.23 | 0.784 | 1.067 | 0.68-1.674 |
| order Clostridiales | Weighted median | 12 | 0.03 | 0.13 | 0.815 | 1.031 | 0.799-1.33 |
| order Clostridiales | Weighted mode | 12 | 0.108 | 0.16 | 0.523 | 1.115 | 0.808-1.538 |
| order Coriobacteriales | IVW | 13 | 0.068 | 0.1 | 0.51 | 1.07 | 0.874-1.311 |
| order Coriobacteriales | MR Egger | 13 | 0.009 | 0.39 | 0.981 | 1.009 | 0.47-2.168 |
| order Coriobacteriales | Weighted median | 13 | 0.175 | 0.14 | 0.209 | 1.191 | 0.907-1.565 |
| order Coriobacteriales | Weighted mode | 13 | 0.302 | 0.22 | 0.202 | 1.353 | 0.872-2.1 |
| order Desulfovibrionales | IVW | 11 | -0.12 | 0.12 | 0.313 | 0.89 | 0.711-1.116 |
| order Desulfovibrionales | MR Egger | 11 | 0.039 | 0.55 | 0.945 | 1.04 | 0.356-3.04 |
| order Desulfovibrionales | Weighted median | 11 | -0.02 | 0.14 | 0.858 | 0.976 | 0.749-1.273 |
| order Desulfovibrionales | Weighted mode | 11 | 0.106 | 0.23 | 0.658 | 1.112 | 0.705-1.755 |
| order Enterobacteriales | IVW | 5 | 0.16 | 0.16 | 0.303 | 1.174 | 0.865-1.592 |
| order Enterobacteriales | MR Egger | 5 | 1.066 | 0.91 | 0.325 | 2.903 | 0.489-17.224 |
| order Enterobacteriales | Weighted median | 5 | 0.298 | 0.19 | 0.122 | 1.347 | 0.924-1.965 |
| order Enterobacteriales | Weighted mode | 5 | 0.356 | 0.27 | 0.261 | 1.428 | 0.838-2.434 |
| order Erysipelotrichales | IVW | 13 | 0.127 | 0.1 | 0.199 | 1.135 | 0.936-1.377 |
| order Erysipelotrichales | MR Egger | 13 | 0.542 | 0.47 | 0.271 | 1.719 | 0.688-4.292 |
| order Erysipelotrichales | Weighted median | 13 | 0.149 | 0.14 | 0.276 | 1.16 | 0.888-1.516 |
| order Erysipelotrichales | Weighted mode | 13 | 0.131 | 0.2 | 0.524 | 1.14 | 0.772-1.683 |
| order Gastranaerophilales | IVW | 7 | -0.1 | 0.08 | 0.259 | 0.91 | 0.772-1.072 |
| order Gastranaerophilales | MR Egger | 7 | -0.03 | 0.35 | 0.926 | 0.966 | 0.486-1.922 |
| order Gastranaerophilales | Weighted median | 7 | -0.11 | 0.1 | 0.257 | 0.895 | 0.738-1.084 |
| order Gastranaerophilales | Weighted mode | 7 | -0.11 | 0.15 | 0.504 | 0.899 | 0.669-1.207 |
| order Lactobacillales | IVW | 9 | -0.16 | 0.16 | 0.325 | 0.851 | 0.617-1.174 |
| order Lactobacillales | MR Egger | 9 | -0.34 | 0.39 | 0.412 | 0.711 | 0.33-1.53 |
| order Lactobacillales | Weighted median | 9 | -0.15 | 0.16 | 0.339 | 0.858 | 0.627-1.174 |
| order Lactobacillales | Weighted mode | 9 | -0.17 | 0.19 | 0.406 | 0.847 | 0.584-1.228 |
| order Methanobacteriales | IVW | 9 | 0.004 | 0.06 | 0.939 | 1.004 | 0.901-1.119 |
| order Methanobacteriales | MR Egger | 9 | 0.367 | 0.22 | 0.143 | 1.443 | 0.933-2.231 |
| order Methanobacteriales | Weighted median | 9 | 0.061 | 0.07 | 0.396 | 1.063 | 0.923-1.225 |
| order Methanobacteriales | Weighted mode | 9 | 0.083 | 0.11 | 0.47 | 1.087 | 0.876-1.348 |
| order Mollicutes RF9 | IVW | 9 | 0.034 | 0.1 | 0.739 | 1.035 | 0.847-1.264 |
| order Mollicutes RF9 | MR Egger | 9 | 0.443 | 0.76 | 0.578 | 1.557 | 0.352-6.886 |
| order Mollicutes RF9 | Weighted median | 9 | 0.09 | 0.13 | 0.482 | 1.094 | 0.851-1.407 |
| order Mollicutes RF9 | Weighted mode | 9 | 0.093 | 0.18 | 0.629 | 1.097 | 0.764-1.574 |
| order NB1n | IVW | 13 | 0.053 | 0.07 | 0.477 | 1.054 | 0.912-1.219 |
| order NB1n | MR Egger | 13 | 0.381 | 0.29 | 0.222 | 1.463 | 0.822-2.604 |
| order NB1n | Weighted median | 13 | 0.075 | 0.08 | 0.316 | 1.078 | 0.931-1.248 |
| order NB1n | Weighted mode | 13 | 0.079 | 0.12 | 0.534 | 1.082 | 0.85-1.377 |
| order Pasteurellales | IVW | 10 | 0.003 | 0.11 | 0.976 | 1.003 | 0.812-1.24 |
| order Pasteurellales | MR Egger | 10 | -0.5 | 0.27 | 0.096 | 0.605 | 0.359-1.019 |
| order Pasteurellales | Weighted median | 10 | -0.05 | 0.12 | 0.687 | 0.954 | 0.758-1.2 |
| order Pasteurellales | Weighted mode | 10 | -0.12 | 0.16 | 0.468 | 0.887 | 0.649-1.211 |
| order Rhodospirillales | IVW | 12 | 0.087 | 0.09 | 0.329 | 1.091 | 0.916-1.3 |
| order Rhodospirillales | MR Egger | 12 | 0.332 | 0.58 | 0.577 | 1.394 | 0.451-4.314 |
| order Rhodospirillales | Weighted median | 12 | 0.116 | 0.1 | 0.26 | 1.124 | 0.917-1.376 |
| order Rhodospirillales | Weighted mode | 12 | 0.136 | 0.13 | 0.313 | 1.146 | 0.89-1.476 |
| order Selenomonadales | IVW | 11 | -0.05 | 0.15 | 0.76 | 0.955 | 0.71-1.284 |
| order Selenomonadales | MR Egger | 11 | -0.44 | 0.56 | 0.451 | 0.642 | 0.213-1.933 |
| order Selenomonadales | Weighted median | 11 | -0.05 | 0.16 | 0.751 | 0.951 | 0.695-1.3 |
| order Selenomonadales | Weighted mode | 11 | 0.123 | 0.25 | 0.638 | 1.131 | 0.688-1.86 |
| order Verrucomicrobiales | IVW | 8 | -0.01 | 0.1 | 0.901 | 0.988 | 0.814-1.198 |
| order Verrucomicrobiales | MR Egger | 8 | 1.427 | 1.31 | 0.317 | 4.167 | 0.32-54.206 |
| order Verrucomicrobiales | Weighted median | 8 | 0.024 | 0.12 | 0.842 | 1.024 | 0.811-1.293 |
| order Verrucomicrobiales | Weighted mode | 8 | 0.041 | 0.19 | 0.832 | 1.042 | 0.723-1.502 |
| order Victivallales | IVW | 7 | -0.06 | 0.1 | 0.557 | 0.943 | 0.775-1.148 |
| order Victivallales | MR Egger | 7 | 0.286 | 0.4 | 0.509 | 1.331 | 0.605-2.925 |
| order Victivallales | Weighted median | 7 | -0.15 | 0.09 | 0.094 | 0.865 | 0.73-1.025 |
| order Victivallales | Weighted mode | 7 | -0.16 | 0.11 | 0.221 | 0.855 | 0.683-1.07 |
| phylum Actinobacteria | IVW | 12 | 0.044 | 0.14 | 0.745 | 1.045 | 0.801-1.364 |
| phylum Actinobacteria | MR Egger | 12 | 1.232 | 0.65 | 0.089 | 3.43 | 0.953-12.34 |
| phylum Actinobacteria | Weighted median | 12 | -0.03 | 0.14 | 0.84 | 0.972 | 0.735-1.285 |
| phylum Actinobacteria | Weighted mode | 12 | -0.17 | 0.27 | 0.538 | 0.843 | 0.497-1.429 |
| phylum Bacteroidetes | IVW | 11 | -0.04 | 0.1 | 0.702 | 0.964 | 0.801-1.161 |
| phylum Bacteroidetes | MR Egger | 11 | -0.04 | 0.21 | 0.851 | 0.961 | 0.644-1.436 |
| phylum Bacteroidetes | Weighted median | 11 | 0 | 0.13 | 0.974 | 0.996 | 0.777-1.276 |
| phylum Bacteroidetes | Weighted mode | 11 | -0.02 | 0.16 | 0.92 | 0.984 | 0.722-1.341 |
| phylum Cyanobacteria | IVW | 6 | -0.05 | 0.11 | 0.653 | 0.952 | 0.769-1.179 |
| phylum Cyanobacteria | MR Egger | 6 | -0.1 | 0.54 | 0.86 | 0.903 | 0.314-2.602 |
| phylum Cyanobacteria | Weighted median | 6 | -0.15 | 0.13 | 0.273 | 0.865 | 0.666-1.122 |
| phylum Cyanobacteria | Weighted mode | 6 | -0.21 | 0.18 | 0.305 | 0.811 | 0.567-1.162 |
| phylum Euryarchaeota | IVW | 10 | -0.1 | 0.07 | 0.162 | 0.907 | 0.791-1.04 |
| phylum Euryarchaeota | MR Egger | 10 | -0.12 | 0.36 | 0.753 | 0.889 | 0.437-1.806 |
| phylum Euryarchaeota | Weighted median | 10 | -0.05 | 0.08 | 0.486 | 0.948 | 0.816-1.102 |
| phylum Euryarchaeota | Weighted mode | 10 | -0.04 | 0.12 | 0.749 | 0.962 | 0.765-1.21 |
| phylum Firmicutes | IVW | 14 | 0.01 | 0.11 | 0.921 | 1.011 | 0.821-1.244 |
| phylum Firmicutes | MR Egger | 14 | 0.306 | 0.24 | 0.223 | 1.358 | 0.852-2.165 |
| phylum Firmicutes | Weighted median | 14 | -0.02 | 0.14 | 0.899 | 0.983 | 0.752-1.284 |
| phylum Firmicutes | Weighted mode | 14 | 0.038 | 0.17 | 0.827 | 1.039 | 0.744-1.451 |
| phylum Lentisphaerae | IVW | 8 | -0.04 | 0.09 | 0.622 | 0.957 | 0.805-1.139 |
| phylum Lentisphaerae | MR Egger | 8 | 0.338 | 0.37 | 0.39 | 1.402 | 0.685-2.869 |
| phylum Lentisphaerae | Weighted median | 8 | -0.14 | 0.09 | 0.111 | 0.874 | 0.74-1.031 |
| phylum Lentisphaerae | Weighted mode | 8 | -0.17 | 0.12 | 0.208 | 0.846 | 0.667-1.071 |
| phylum Proteobacteria | IVW | 11 | 0.093 | 0.15 | 0.547 | 1.097 | 0.811-1.486 |
| phylum Proteobacteria | MR Egger | 11 | -0.56 | 0.5 | 0.289 | 0.571 | 0.215-1.514 |
| phylum Proteobacteria | Weighted median | 11 | 0.13 | 0.16 | 0.409 | 1.139 | 0.837-1.55 |
| phylum Proteobacteria | Weighted mode | 11 | 0.113 | 0.25 | 0.659 | 1.119 | 0.689-1.818 |
| phylum Tenericutes | IVW | 10 | -0.12 | 0.09 | 0.204 | 0.89 | 0.744-1.065 |
| phylum Tenericutes | MR Egger | 10 | 0.117 | 0.53 | 0.83 | 1.125 | 0.398-3.176 |
| phylum Tenericutes | Weighted median | 10 | -0.01 | 0.13 | 0.932 | 0.989 | 0.773-1.266 |
| phylum Tenericutes | Weighted mode | 10 | 0.05 | 0.22 | 0.825 | 1.051 | 0.684-1.614 |
| phylum Verrucomicrobia | IVW | 9 | -0.09 | 0.09 | 0.346 | 0.916 | 0.763-1.099 |
| phylum Verrucomicrobia | MR Egger | 9 | -0.56 | 0.31 | 0.115 | 0.57 | 0.309-1.052 |
| phylum Verrucomicrobia | Weighted median | 9 | -0.01 | 0.13 | 0.961 | 0.994 | 0.778-1.27 |
| phylum Verrucomicrobia | Weighted mode | 9 | 0.049 | 0.2 | 0.815 | 1.05 | 0.708-1.558 |
| ***Guillain-barre syndrome*** | |  |  |  |  |  |  |
| class Actinobacteria | IVW | 14 | 0.051 | 0.47 | 0.913 | 1.053 | 0.419-2.646 |
| class Actinobacteria | MR Egger | 14 | -2.23 | 1.25 | 0.1 | 0.108 | 0.009-1.247 |
| class Actinobacteria | Weighted median | 14 | 0.338 | 0.64 | 0.598 | 1.402 | 0.399-4.926 |
| class Actinobacteria | Weighted mode | 14 | 0.325 | 0.84 | 0.705 | 1.384 | 0.267-7.174 |
| class Alphaproteobacteria | IVW | 7 | -0.13 | 0.7 | 0.854 | 0.88 | 0.224-3.458 |
| class Alphaproteobacteria | MR Egger | 7 | 1.267 | 2.79 | 0.669 | 3.55 | 0.015-840.522 |
| class Alphaproteobacteria | Weighted median | 7 | -0.9 | 0.73 | 0.217 | 0.405 | 0.097-1.698 |
| class Alphaproteobacteria | Weighted mode | 7 | -1.36 | 1.06 | 0.244 | 0.256 | 0.032-2.027 |
| class Bacilli | IVW | 18 | -0.59 | 0.43 | 0.174 | 0.554 | 0.236-1.298 |
| class Bacilli | MR Egger | 18 | -2.67 | 1.19 | 0.039 | 0.069 | 0.007-0.715 |
| class Bacilli | Weighted median | 18 | -0.87 | 0.61 | 0.158 | 0.421 | 0.127-1.398 |
| class Bacilli | Weighted mode | 18 | -1.44 | 1.02 | 0.173 | 0.236 | 0.032-1.73 |
| genus Parasutterella | IVW | 13 | -0.24 | 0.72 | 0.739 | 0.787 | 0.192-3.23 |
| genus Parasutterella | MR Egger | 13 | -2.75 | 1.55 | 0.103 | 0.064 | 0.003-1.32 |
| genus Parasutterella | Weighted median | 13 | -0.56 | 0.8 | 0.483 | 0.572 | 0.12-2.719 |
| genus Parasutterella | Weighted mode | 13 | -1.09 | 1.1 | 0.339 | 0.336 | 0.039-2.882 |
| class Betaproteobacteria | IVW | 10 | -0.65 | 0.6 | 0.281 | 0.525 | 0.162-1.695 |
| class Betaproteobacteria | MR Egger | 10 | 2.066 | 1.87 | 0.302 | 7.897 | 0.201-310.078 |
| class Betaproteobacteria | Weighted median | 10 | -0.56 | 0.79 | 0.476 | 0.57 | 0.121-2.675 |
| class Betaproteobacteria | Weighted mode | 10 | -0.41 | 1.11 | 0.722 | 0.664 | 0.075-5.886 |
| class Clostridia | IVW | 12 | 0.254 | 0.52 | 0.625 | 1.289 | 0.465-3.574 |
| class Clostridia | MR Egger | 12 | -0.93 | 1.41 | 0.523 | 0.393 | 0.025-6.257 |
| class Clostridia | Weighted median | 12 | 0.467 | 0.71 | 0.512 | 1.595 | 0.395-6.434 |
| class Clostridia | Weighted mode | 12 | 0.543 | 1.06 | 0.619 | 1.721 | 0.215-13.77 |
| class Coriobacteriia | IVW | 13 | -0.42 | 0.65 | 0.515 | 0.655 | 0.184-2.34 |
| class Coriobacteriia | MR Egger | 13 | 2.357 | 2.75 | 0.409 | 10.56 | 0.049-2295.843 |
| class Coriobacteriia | Weighted median | 13 | -0.29 | 0.77 | 0.71 | 0.751 | 0.166-3.395 |
| class Coriobacteriia | Weighted mode | 13 | 1.092 | 1.5 | 0.482 | 2.981 | 0.156-56.795 |
| class Deltaproteobacteria | IVW | 11 | -0.21 | 0.74 | 0.777 | 0.812 | 0.192-3.431 |
| class Deltaproteobacteria | MR Egger | 11 | -3.29 | 3.89 | 0.42 | 0.037 | 0-76.011 |
| class Deltaproteobacteria | Weighted median | 11 | -0.99 | 0.76 | 0.192 | 0.371 | 0.083-1.648 |
| class Deltaproteobacteria | Weighted mode | 11 | -1.2 | 1.01 | 0.261 | 0.3 | 0.042-2.169 |
| class Erysipelotrichia | IVW | 13 | -0.2 | 0.59 | 0.739 | 0.821 | 0.257-2.621 |
| class Erysipelotrichia | MR Egger | 13 | -6.11 | 2.43 | 0.028 | 0.002 | 0-0.256 |
| class Erysipelotrichia | Weighted median | 13 | -0.26 | 0.79 | 0.736 | 0.768 | 0.165-3.575 |
| class Erysipelotrichia | Weighted mode | 13 | -1.12 | 1.47 | 0.463 | 0.328 | 0.018-5.862 |
| class Gammaproteobacteria | IVW | 6 | 0.033 | 0.77 | 0.966 | 1.034 | 0.23-4.637 |
| class Gammaproteobacteria | MR Egger | 6 | 0.88 | 2.39 | 0.731 | 2.412 | 0.022-259.73 |
| class Gammaproteobacteria | Weighted median | 6 | -0.25 | 1.03 | 0.811 | 0.783 | 0.105-5.841 |
| class Gammaproteobacteria | Weighted mode | 6 | -0.5 | 1.39 | 0.735 | 0.608 | 0.04-9.256 |
| class Lentisphaeria | IVW | 8 | 0.28 | 0.36 | 0.431 | 1.323 | 0.659-2.657 |
| class Lentisphaeria | MR Egger | 8 | 0.077 | 1.26 | 0.953 | 1.08 | 0.091-12.814 |
| class Lentisphaeria | Weighted median | 8 | 0.475 | 0.46 | 0.298 | 1.608 | 0.657-3.936 |
| class Lentisphaeria | Weighted mode | 8 | 0.537 | 0.64 | 0.426 | 1.711 | 0.492-5.959 |
| class Melainabacteria | IVW | 10 | -0.21 | 0.51 | 0.682 | 0.812 | 0.301-2.193 |
| class Melainabacteria | MR Egger | 10 | 1.823 | 1.44 | 0.24 | 6.193 | 0.37-103.713 |
| class Melainabacteria | Weighted median | 10 | -0.24 | 0.57 | 0.668 | 0.784 | 0.257-2.393 |
| class Melainabacteria | Weighted mode | 10 | 1.051 | 1.15 | 0.386 | 2.859 | 0.299-27.362 |
| class Methanobacteria | IVW | 9 | -0.15 | 0.37 | 0.697 | 0.865 | 0.417-1.794 |
| class Methanobacteria | MR Egger | 9 | -0.93 | 1.54 | 0.563 | 0.394 | 0.019-7.981 |
| class Methanobacteria | Weighted median | 9 | -0.25 | 0.46 | 0.584 | 0.778 | 0.317-1.911 |
| class Methanobacteria | Weighted mode | 9 | -0.93 | 0.74 | 0.243 | 0.394 | 0.093-1.675 |
| class Mollicutes | IVW | 12 | 1.104 | 0.46 | 0.016 | 3.016 | 1.228-7.411 |
| class Mollicutes | MR Egger | 12 | 1.941 | 1.62 | 0.258 | 6.963 | 0.292-166.094 |
| class Mollicutes | Weighted median | 12 | 1.204 | 0.61 | 0.048 | 3.332 | 1.008-11.017 |
| class Mollicutes | Weighted mode | 12 | 1.054 | 0.79 | 0.21 | 2.869 | 0.609-13.526 |
| class Negativicutes | IVW | 12 | -0.91 | 0.63 | 0.153 | 0.404 | 0.117-1.4 |
| class Negativicutes | MR Egger | 12 | -3.52 | 2.13 | 0.129 | 0.029 | 0-1.907 |
| class Negativicutes | Weighted median | 12 | -1.01 | 0.82 | 0.217 | 0.363 | 0.073-1.812 |
| class Negativicutes | Weighted mode | 12 | -0.46 | 1.22 | 0.712 | 0.63 | 0.058-6.88 |
| class Verrucomicrobiae | IVW | 11 | -0.25 | 0.47 | 0.593 | 0.779 | 0.312-1.945 |
| class Verrucomicrobiae | MR Egger | 11 | -1.64 | 1.57 | 0.324 | 0.195 | 0.009-4.213 |
| class Verrucomicrobiae | Weighted median | 11 | -0.92 | 0.61 | 0.133 | 0.4 | 0.121-1.321 |
| class Verrucomicrobiae | Weighted mode | 11 | -0.88 | 0.91 | 0.358 | 0.415 | 0.069-2.488 |
| family Acidaminococcaceae | IVW | 7 | 0.375 | 0.61 | 0.539 | 1.455 | 0.441-4.803 |
| family Acidaminococcaceae | MR Egger | 7 | 1.592 | 1.94 | 0.448 | 4.913 | 0.111-218.179 |
| family Acidaminococcaceae | Weighted median | 7 | 0.531 | 0.73 | 0.469 | 1.701 | 0.403-7.174 |
| family Acidaminococcaceae | Weighted mode | 7 | 0.653 | 1.18 | 0.599 | 1.921 | 0.192-19.228 |
| family Actinomycetaceae | IVW | 4 | -0.13 | 0.59 | 0.822 | 0.876 | 0.276-2.776 |
| family Actinomycetaceae | MR Egger | 4 | 0.472 | 1.39 | 0.767 | 1.603 | 0.105-24.592 |
| family Actinomycetaceae | Weighted median | 4 | 0.271 | 0.71 | 0.703 | 1.312 | 0.326-5.284 |
| family Actinomycetaceae | Weighted mode | 4 | 0.331 | 0.81 | 0.709 | 1.393 | 0.287-6.762 |
| family Alcaligenaceae | IVW | 11 | 0.275 | 0.59 | 0.638 | 1.317 | 0.419-4.142 |
| family Alcaligenaceae | MR Egger | 11 | 1.559 | 2.68 | 0.576 | 4.753 | 0.025-914.244 |
| family Alcaligenaceae | Weighted median | 11 | 0.313 | 0.79 | 0.692 | 1.367 | 0.291-6.413 |
| family Alcaligenaceae | Weighted mode | 11 | 0.709 | 1.27 | 0.59 | 2.032 | 0.167-24.683 |
| family Bacteroidaceae | IVW | 7 | -0.36 | 0.74 | 0.628 | 0.698 | 0.163-2.983 |
| family Bacteroidaceae | MR Egger | 7 | -1.37 | 4 | 0.745 | 0.253 | 0-645.73 |
| family Bacteroidaceae | Weighted median | 7 | -0.29 | 0.97 | 0.766 | 0.749 | 0.112-4.998 |
| family Bacteroidaceae | Weighted mode | 7 | -1.72 | 1.45 | 0.278 | 0.179 | 0.01-3.037 |
| family Bacteroidales | IVW | 8 | 0.344 | 0.44 | 0.43 | 1.41 | 0.6-3.312 |
| family Bacteroidales | MR Egger | 8 | 0.997 | 1.75 | 0.589 | 2.71 | 0.088-83.299 |
| family Bacteroidales | Weighted median | 8 | 0.618 | 0.58 | 0.286 | 1.855 | 0.596-5.774 |
| family Bacteroidales | Weighted mode | 8 | 0.909 | 0.87 | 0.333 | 2.481 | 0.448-13.743 |
| family Bifidobacteriaceae | IVW | 11 | 0.375 | 0.47 | 0.426 | 1.454 | 0.578-3.657 |
| family Bifidobacteriaceae | MR Egger | 11 | 0.701 | 1.53 | 0.658 | 2.015 | 0.1-40.535 |
| family Bifidobacteriaceae | Weighted median | 11 | 0.625 | 0.65 | 0.333 | 1.868 | 0.527-6.614 |
| family Bifidobacteriaceae | Weighted mode | 11 | 0.686 | 0.75 | 0.381 | 1.986 | 0.457-8.627 |
| family Clostridiaceae1 | IVW | 10 | -0.53 | 0.69 | 0.443 | 0.591 | 0.154-2.267 |
| family Clostridiaceae1 | MR Egger | 10 | 1.375 | 2 | 0.512 | 3.955 | 0.078-200.248 |
| family Clostridiaceae1 | Weighted median | 10 | 0.228 | 0.78 | 0.77 | 1.257 | 0.272-5.814 |
| family Clostridiaceae1 | Weighted mode | 10 | 0.488 | 0.97 | 0.629 | 1.629 | 0.241-10.991 |
| family Clostridiales vadin BB60 group | IVW | 15 | -0.14 | 0.37 | 0.707 | 0.869 | 0.418-1.808 |
| family Clostridiales vadin BB60 group | MR Egger | 15 | -0.9 | 1.02 | 0.394 | 0.406 | 0.055-3.014 |
| family Clostridiales vadin BB60 group | Weighted median | 15 | -0.23 | 0.51 | 0.654 | 0.795 | 0.292-2.164 |
| family Clostridiales vadin BB60 group | Weighted mode | 15 | -0.43 | 0.7 | 0.55 | 0.652 | 0.166-2.559 |
| family Coriobacteriaceae | IVW | 13 | -0.42 | 0.65 | 0.515 | 0.655 | 0.184-2.34 |
| family Coriobacteriaceae | MR Egger | 13 | 2.357 | 2.75 | 0.409 | 10.56 | 0.049-2295.843 |
| family Coriobacteriaceae | Weighted median | 13 | -0.29 | 0.76 | 0.704 | 0.751 | 0.171-3.3 |
| family Coriobacteriaceae | Weighted mode | 13 | 1.092 | 1.39 | 0.448 | 2.981 | 0.194-45.765 |
| family Defluviitaleaceae | IVW | 11 | 0.025 | 0.41 | 0.953 | 1.025 | 0.456-2.304 |
| family Defluviitaleaceae | MR Egger | 11 | 1.18 | 1.41 | 0.425 | 3.253 | 0.205-51.725 |
| family Defluviitaleaceae | Weighted median | 11 | 0.194 | 0.52 | 0.711 | 1.214 | 0.435-3.389 |
| family Defluviitaleaceae | Weighted mode | 11 | 0.131 | 0.88 | 0.885 | 1.14 | 0.202-6.429 |
| family Desulfovibrionaceae | IVW | 8 | -0.14 | 0.96 | 0.884 | 0.869 | 0.131-5.738 |
| family Desulfovibrionaceae | MR Egger | 8 | -4.13 | 4.7 | 0.413 | 0.016 | 0-159.984 |
| family Desulfovibrionaceae | Weighted median | 8 | -1.08 | 0.82 | 0.188 | 0.341 | 0.069-1.694 |
| family Desulfovibrionaceae | Weighted mode | 8 | -1.23 | 1 | 0.259 | 0.292 | 0.041-2.078 |
| family Enterobacteriaceae | IVW | 7 | 0.653 | 0.67 | 0.331 | 1.921 | 0.515-7.164 |
| family Enterobacteriaceae | MR Egger | 7 | 2.22 | 4.01 | 0.604 | 9.208 | 0.004-23861.452 |
| family Enterobacteriaceae | Weighted median | 7 | 1.03 | 0.88 | 0.24 | 2.8 | 0.503-15.577 |
| family Enterobacteriaceae | Weighted mode | 7 | 1.119 | 1.28 | 0.415 | 3.061 | 0.25-37.424 |
| family Erysipelotrichaceae | IVW | 13 | -0.2 | 0.59 | 0.739 | 0.821 | 0.257-2.621 |
| family Erysipelotrichaceae | MR Egger | 13 | -6.11 | 2.43 | 0.028 | 0.002 | 0-0.256 |
| family Erysipelotrichaceae | Weighted median | 13 | -0.26 | 0.76 | 0.73 | 0.768 | 0.172-3.436 |
| family Erysipelotrichaceae | Weighted mode | 13 | -1.12 | 1.46 | 0.458 | 0.328 | 0.019-5.678 |
| family Family XI | IVW | 8 | -0.01 | 0.36 | 0.971 | 0.987 | 0.485-2.01 |
| family Family XI | MR Egger | 8 | 2.127 | 2.33 | 0.397 | 8.388 | 0.087-811.844 |
| family Family XI | Weighted median | 8 | -0.35 | 0.41 | 0.391 | 0.702 | 0.313-1.575 |
| family Family XI | Weighted mode | 8 | -0.77 | 0.72 | 0.316 | 0.461 | 0.113-1.881 |
| family Family XIII | IVW | 7 | 0.604 | 0.73 | 0.409 | 1.829 | 0.436-7.678 |
| family Family XIII | MR Egger | 7 | -0.19 | 2.74 | 0.947 | 0.825 | 0.004-176.589 |
| family Family XIII | Weighted median | 7 | 0.105 | 0.96 | 0.913 | 1.11 | 0.17-7.261 |
| family Family XIII | Weighted mode | 7 | 0.01 | 1.3 | 0.994 | 1.01 | 0.079-12.982 |
| family Lachnospiraceae | IVW | 16 | -0.77 | 0.51 | 0.132 | 0.464 | 0.171-1.26 |
| family Lachnospiraceae | MR Egger | 16 | -0.7 | 1.81 | 0.706 | 0.498 | 0.014-17.382 |
| family Lachnospiraceae | Weighted median | 16 | -0.98 | 0.7 | 0.163 | 0.377 | 0.096-1.484 |
| family Lachnospiraceae | Weighted mode | 16 | -1.22 | 1.31 | 0.369 | 0.297 | 0.023-3.891 |
| family Lactobacillaceae | IVW | 8 | -0.16 | 0.45 | 0.724 | 0.853 | 0.353-2.061 |
| family Lactobacillaceae | MR Egger | 8 | -1.74 | 0.99 | 0.131 | 0.176 | 0.025-1.236 |
| family Lactobacillaceae | Weighted median | 8 | -0.4 | 0.54 | 0.464 | 0.673 | 0.233-1.942 |
| family Lactobacillaceae | Weighted mode | 8 | -0.53 | 0.62 | 0.421 | 0.587 | 0.173-1.992 |
| family Methanobacteriaceae | IVW | 9 | -0.15 | 0.37 | 0.697 | 0.865 | 0.417-1.794 |
| family Methanobacteriaceae | MR Egger | 9 | -0.93 | 1.54 | 0.563 | 0.394 | 0.019-7.981 |
| family Methanobacteriaceae | Weighted median | 9 | -0.25 | 0.46 | 0.587 | 0.778 | 0.314-1.927 |
| family Methanobacteriaceae | Weighted mode | 9 | -0.93 | 0.78 | 0.268 | 0.394 | 0.085-1.827 |
| family Oxalobacteraceae | IVW | 14 | 0.285 | 0.27 | 0.292 | 1.33 | 0.783-2.261 |
| family Oxalobacteraceae | MR Egger | 14 | 0.229 | 1.05 | 0.831 | 1.257 | 0.162-9.756 |
| family Oxalobacteraceae | Weighted median | 14 | 0.232 | 0.36 | 0.523 | 1.261 | 0.619-2.57 |
| family Oxalobacteraceae | Weighted mode | 14 | 0.146 | 0.53 | 0.785 | 1.158 | 0.413-3.247 |
| family Pasteurellaceae | IVW | 13 | -0.14 | 0.34 | 0.687 | 0.872 | 0.447-1.699 |
| family Pasteurellaceae | MR Egger | 13 | 0.203 | 0.73 | 0.787 | 1.226 | 0.291-5.169 |
| family Pasteurellaceae | Weighted median | 13 | 0.096 | 0.5 | 0.847 | 1.1 | 0.417-2.906 |
| family Pasteurellaceae | Weighted mode | 13 | 0.272 | 1 | 0.789 | 1.313 | 0.186-9.252 |
| family Peptococcaceae | IVW | 9 | -0.02 | 0.49 | 0.973 | 0.983 | 0.375-2.577 |
| family Peptococcaceae | MR Egger | 9 | -0.26 | 1.46 | 0.862 | 0.769 | 0.044-13.364 |
| family Peptococcaceae | Weighted median | 9 | -0.57 | 0.61 | 0.345 | 0.564 | 0.172-1.852 |
| family Peptococcaceae | Weighted mode | 9 | -0.97 | 1 | 0.36 | 0.379 | 0.054-2.688 |
| family Peptostreptococcaceae | IVW | 13 | 0.845 | 0.54 | 0.12 | 2.327 | 0.802-6.755 |
| family Peptostreptococcaceae | MR Egger | 13 | 0.988 | 1.29 | 0.46 | 2.685 | 0.214-33.653 |
| family Peptostreptococcaceae | Weighted median | 13 | 0.782 | 0.67 | 0.245 | 2.186 | 0.584-8.18 |
| family Peptostreptococcaceae | Weighted mode | 13 | 0.578 | 0.79 | 0.476 | 1.782 | 0.382-8.31 |
| family Porphyromonadaceae | IVW | 9 | 0.187 | 0.83 | 0.822 | 1.205 | 0.237-6.131 |
| family Porphyromonadaceae | MR Egger | 9 | -1.34 | 3.88 | 0.741 | 0.263 | 0-530.967 |
| family Porphyromonadaceae | Weighted median | 9 | -1.05 | 0.95 | 0.27 | 0.351 | 0.055-2.252 |
| family Porphyromonadaceae | Weighted mode | 9 | -1.08 | 1.35 | 0.449 | 0.34 | 0.024-4.828 |
| family Prevotellaceae | IVW | 15 | -0.49 | 0.45 | 0.279 | 0.616 | 0.256-1.483 |
| family Prevotellaceae | MR Egger | 15 | -1.84 | 1.59 | 0.27 | 0.159 | 0.007-3.624 |
| family Prevotellaceae | Weighted median | 15 | -0.11 | 0.61 | 0.862 | 0.899 | 0.271-2.986 |
| family Prevotellaceae | Weighted mode | 15 | -0.01 | 1.03 | 0.994 | 0.992 | 0.131-7.53 |
| family Rhodospirillaceae | IVW | 15 | -0.35 | 0.34 | 0.302 | 0.706 | 0.364-1.368 |
| family Rhodospirillaceae | MR Egger | 15 | -0.04 | 1.47 | 0.978 | 0.959 | 0.053-17.194 |
| family Rhodospirillaceae | Weighted median | 15 | -0.43 | 0.46 | 0.351 | 0.652 | 0.266-1.599 |
| family Rhodospirillaceae | Weighted mode | 15 | -0.3 | 0.72 | 0.689 | 0.744 | 0.181-3.068 |
| family Rikenellaceae | IVW | 16 | 0.089 | 0.47 | 0.848 | 1.093 | 0.439-2.724 |
| family Rikenellaceae | MR Egger | 16 | 1.888 | 1.43 | 0.208 | 6.608 | 0.401-108.767 |
| family Rikenellaceae | Weighted median | 16 | 0.244 | 0.63 | 0.7 | 1.277 | 0.369-4.415 |
| family Rikenellaceae | Weighted mode | 16 | 0.521 | 1.08 | 0.635 | 1.684 | 0.204-13.904 |
| family Ruminococcaceae | IVW | 9 | 0.222 | 0.56 | 0.693 | 1.249 | 0.415-3.757 |
| family Ruminococcaceae | MR Egger | 9 | -1.18 | 1.27 | 0.382 | 0.306 | 0.026-3.672 |
| family Ruminococcaceae | Weighted median | 9 | 0.524 | 0.72 | 0.469 | 1.688 | 0.409-6.967 |
| family Ruminococcaceae | Weighted mode | 9 | 1.331 | 1.14 | 0.278 | 3.784 | 0.402-35.577 |
| family Streptococcaceae | IVW | 11 | -0.3 | 0.57 | 0.605 | 0.745 | 0.244-2.274 |
| family Streptococcaceae | MR Egger | 11 | -1.86 | 2.35 | 0.448 | 0.156 | 0.002-15.432 |
| family Streptococcaceae | Weighted median | 11 | -0.5 | 0.74 | 0.499 | 0.607 | 0.143-2.582 |
| family Streptococcaceae | Weighted mode | 11 | -0.88 | 1.08 | 0.438 | 0.417 | 0.05-3.478 |
| family Veillonellaceae | IVW | 19 | -0.46 | 0.37 | 0.221 | 0.632 | 0.304-1.317 |
| family Veillonellaceae | MR Egger | 19 | -1.39 | 0.76 | 0.084 | 0.249 | 0.056-1.101 |
| family Veillonellaceae | Weighted median | 19 | -0.45 | 0.56 | 0.416 | 0.635 | 0.213-1.897 |
| family Veillonellaceae | Weighted mode | 19 | -0.73 | 0.69 | 0.303 | 0.48 | 0.124-1.864 |
| family Verrucomicrobiaceae | IVW | 11 | -0.25 | 0.47 | 0.594 | 0.78 | 0.312-1.946 |
| family Verrucomicrobiaceae | MR Egger | 11 | -1.63 | 1.57 | 0.325 | 0.195 | 0.009-4.228 |
| family Verrucomicrobiaceae | Weighted median | 11 | -0.91 | 0.62 | 0.14 | 0.402 | 0.12-1.348 |
| family Verrucomicrobiaceae | Weighted mode | 11 | -0.88 | 0.89 | 0.344 | 0.414 | 0.073-2.355 |
| family Victivallaceae | IVW | 12 | 0.007 | 0.25 | 0.977 | 1.007 | 0.612-1.658 |
| family Victivallaceae | MR Egger | 12 | 1.826 | 1.21 | 0.162 | 6.208 | 0.58-66.445 |
| family Victivallaceae | Weighted median | 12 | 0.014 | 0.34 | 0.967 | 1.014 | 0.518-1.987 |
| family Victivallaceae | Weighted mode | 12 | 0.038 | 0.51 | 0.942 | 1.039 | 0.384-2.806 |
| genus Actinomyces | IVW | 7 | -0.01 | 0.45 | 0.988 | 0.993 | 0.409-2.415 |
| genus Actinomyces | MR Egger | 7 | -0.17 | 1.11 | 0.886 | 0.846 | 0.097-7.415 |
| genus Actinomyces | Weighted median | 7 | 0.085 | 0.58 | 0.883 | 1.089 | 0.352-3.371 |
| genus Actinomyces | Weighted mode | 7 | 0.182 | 0.74 | 0.813 | 1.2 | 0.282-5.114 |
| genus Adlercreutzia | IVW | 8 | -0.19 | 0.49 | 0.689 | 0.824 | 0.318-2.132 |
| genus Adlercreutzia | MR Egger | 8 | -0.55 | 2.18 | 0.808 | 0.576 | 0.008-40.857 |
| genus Adlercreutzia | Weighted median | 8 | -0.26 | 0.58 | 0.653 | 0.771 | 0.248-2.394 |
| genus Adlercreutzia | Weighted mode | 8 | -0.26 | 0.85 | 0.771 | 0.774 | 0.146-4.091 |
| genus Anaerofilum | IVW | 11 | -0.26 | 0.32 | 0.422 | 0.772 | 0.411-1.451 |
| genus Anaerofilum | MR Egger | 11 | -0.2 | 1.75 | 0.913 | 0.821 | 0.027-25.213 |
| genus Anaerofilum | Weighted median | 11 | -0.34 | 0.44 | 0.436 | 0.711 | 0.302-1.676 |
| genus Anaerofilum | Weighted mode | 11 | -0.54 | 0.73 | 0.475 | 0.583 | 0.14-2.427 |
| genus Anaerostipes | IVW | 13 | 0.06 | 0.54 | 0.912 | 1.062 | 0.367-3.075 |
| genus Anaerostipes | MR Egger | 13 | 1.495 | 1.97 | 0.463 | 4.458 | 0.095-210.303 |
| genus Anaerostipes | Weighted median | 13 | -0.31 | 0.76 | 0.689 | 0.737 | 0.165-3.281 |
| genus Anaerostipes | Weighted mode | 13 | -0.38 | 1.25 | 0.766 | 0.685 | 0.059-7.881 |
| genus Butyrivibrio | IVW | 15 | -0.2 | 0.22 | 0.367 | 0.822 | 0.536-1.26 |
| genus Butyrivibrio | MR Egger | 15 | -1.57 | 0.94 | 0.118 | 0.207 | 0.033-1.309 |
| genus Butyrivibrio | Weighted median | 15 | 0.052 | 0.29 | 0.856 | 1.054 | 0.598-1.856 |
| genus Butyrivibrio | Weighted mode | 15 | 0.224 | 0.57 | 0.702 | 1.251 | 0.407-3.841 |
| genus Catenibacterium | IVW | 4 | -0.51 | 0.43 | 0.233 | 0.598 | 0.257-1.392 |
| genus Catenibacterium | MR Egger | 4 | 1.544 | 5.4 | 0.802 | 4.681 | 0-182994.107 |
| genus Catenibacterium | Weighted median | 4 | -0.51 | 0.49 | 0.299 | 0.6 | 0.229-1.572 |
| genus Catenibacterium | Weighted mode | 4 | -0.76 | 0.64 | 0.321 | 0.47 | 0.135-1.635 |
| genus Christensenellaceae R 7group | IVW | 8 | -0.07 | 0.7 | 0.92 | 0.932 | 0.237-3.665 |
| genus Christensenellaceae R 7group | MR Egger | 8 | 1.155 | 2.45 | 0.654 | 3.175 | 0.026-386.919 |
| genus Christensenellaceae R 7group | Weighted median | 8 | -0.27 | 0.91 | 0.767 | 0.765 | 0.13-4.511 |
| genus Christensenellaceae R 7group | Weighted mode | 8 | -0.42 | 1.36 | 0.766 | 0.657 | 0.046-9.433 |
| genus Clostridium innocuum group | IVW | 7 | 0.135 | 0.34 | 0.691 | 1.145 | 0.588-2.231 |
| genus Clostridium innocuum group | MR Egger | 7 | 1.123 | 1.67 | 0.532 | 3.075 | 0.116-81.836 |
| genus Clostridium innocuum group | Weighted median | 7 | 0.046 | 0.46 | 0.92 | 1.047 | 0.422-2.601 |
| genus Clostridium innocuum group | Weighted mode | 7 | -0.07 | 0.68 | 0.92 | 0.931 | 0.244-3.551 |
| genus Collinsella | IVW | 9 | -0.82 | 0.63 | 0.191 | 0.439 | 0.128-1.509 |
| genus Collinsella | MR Egger | 9 | -4.76 | 2.35 | 0.083 | 0.009 | 0-0.862 |
| genus Collinsella | Weighted median | 9 | -0.53 | 0.83 | 0.523 | 0.589 | 0.116-2.987 |
| genus Collinsella | Weighted mode | 9 | -0.52 | 1.24 | 0.689 | 0.597 | 0.052-6.828 |
| genus Defluviitaleaceae UCG011 | IVW | 9 | -0.01 | 0.46 | 0.977 | 0.987 | 0.399-2.441 |
| genus Defluviitaleaceae UCG011 | MR Egger | 9 | 1.274 | 1.67 | 0.47 | 3.577 | 0.136-93.919 |
| genus Defluviitaleaceae UCG011 | Weighted median | 9 | -0.17 | 0.61 | 0.777 | 0.842 | 0.257-2.763 |
| genus Defluviitaleaceae UCG011 | Weighted mode | 9 | -0.8 | 0.94 | 0.419 | 0.448 | 0.071-2.84 |
| genus Desulfovibrio | IVW | 9 | 0.325 | 0.48 | 0.494 | 1.384 | 0.546-3.507 |
| genus Desulfovibrio | MR Egger | 9 | -1.19 | 1.41 | 0.424 | 0.303 | 0.019-4.777 |
| genus Desulfovibrio | Weighted median | 9 | 0.317 | 0.63 | 0.614 | 1.373 | 0.4-4.717 |
| genus Desulfovibrio | Weighted mode | 9 | 0.419 | 0.95 | 0.671 | 1.52 | 0.237-9.769 |
| genus Dialister | IVW | 11 | 0.307 | 0.49 | 0.528 | 1.36 | 0.524-3.529 |
| genus Dialister | MR Egger | 11 | -0.15 | 2 | 0.944 | 0.865 | 0.017-43.347 |
| genus Dialister | Weighted median | 11 | 0.429 | 0.67 | 0.524 | 1.535 | 0.41-5.747 |
| genus Dialister | Weighted mode | 11 | 0.975 | 1.13 | 0.408 | 2.65 | 0.291-24.153 |
| genus Eggerthella | IVW | 10 | 0.036 | 0.35 | 0.918 | 1.037 | 0.519-2.071 |
| genus Eggerthella | MR Egger | 10 | -0.22 | 1.73 | 0.903 | 0.804 | 0.027-23.81 |
| genus Eggerthella | Weighted median | 10 | 0.402 | 0.48 | 0.404 | 1.494 | 0.582-3.837 |
| genus Eggerthella | Weighted mode | 10 | 0.737 | 0.86 | 0.412 | 2.09 | 0.39-11.201 |
| genus Eisenbergiella | IVW | 11 | -0.42 | 0.33 | 0.209 | 0.658 | 0.343-1.263 |
| genus Eisenbergiella | MR Egger | 11 | 0.896 | 2.46 | 0.724 | 2.45 | 0.02-304.042 |
| genus Eisenbergiella | Weighted median | 11 | -0.26 | 0.43 | 0.546 | 0.773 | 0.335-1.785 |
| genus Eisenbergiella | Weighted mode | 11 | -0.23 | 0.63 | 0.718 | 0.792 | 0.232-2.705 |
| genus Enterorhabdus | IVW | 6 | -0.35 | 0.72 | 0.629 | 0.706 | 0.172-2.895 |
| genus Enterorhabdus | MR Egger | 6 | -2.62 | 1.73 | 0.205 | 0.073 | 0.002-2.164 |
| genus Enterorhabdus | Weighted median | 6 | -0.29 | 0.76 | 0.708 | 0.752 | 0.169-3.345 |
| genus Enterorhabdus | Weighted mode | 6 | -0.16 | 0.98 | 0.88 | 0.855 | 0.125-5.872 |
| genus Eubacterium fissicatena group | IVW | 9 | 0.321 | 0.42 | 0.447 | 1.379 | 0.603-3.153 |
| genus Eubacterium fissicatena group | MR Egger | 9 | -2.64 | 2.04 | 0.237 | 0.071 | 0.001-3.91 |
| genus Eubacterium fissicatena group | Weighted median | 9 | 0.442 | 0.46 | 0.333 | 1.556 | 0.636-3.806 |
| genus Eubacterium fissicatena group | Weighted mode | 9 | 0.752 | 0.8 | 0.372 | 2.121 | 0.446-10.095 |
| genus Eubacterium rectale group | IVW | 8 | 0.166 | 0.66 | 0.801 | 1.181 | 0.323-4.315 |
| genus Eubacterium rectale group | MR Egger | 8 | -0.01 | 2.42 | 0.996 | 0.988 | 0.009-112.234 |
| genus Eubacterium rectale group | Weighted median | 8 | 0.47 | 0.86 | 0.583 | 1.6 | 0.299-8.564 |
| genus Eubacterium rectale group | Weighted mode | 8 | 0.728 | 1.25 | 0.579 | 2.071 | 0.179-23.977 |
| genus Fusicatenibacter | IVW | 18 | 0.312 | 0.46 | 0.498 | 1.366 | 0.554-3.366 |
| genus Fusicatenibacter | MR Egger | 18 | -0.2 | 1.73 | 0.91 | 0.821 | 0.028-24.222 |
| genus Fusicatenibacter | Weighted median | 18 | -0.01 | 0.65 | 0.994 | 0.995 | 0.279-3.549 |
| genus Fusicatenibacter | Weighted mode | 18 | -0.45 | 1.27 | 0.727 | 0.636 | 0.052-7.717 |
| genus Gordonibacter | IVW | 10 | -0.15 | 0.29 | 0.593 | 0.858 | 0.489-1.505 |
| genus Gordonibacter | MR Egger | 10 | -0.7 | 1.15 | 0.563 | 0.498 | 0.052-4.776 |
| genus Gordonibacter | Weighted median | 10 | 0.094 | 0.36 | 0.795 | 1.099 | 0.54-2.238 |
| genus Gordonibacter | Weighted mode | 10 | 0.168 | 0.54 | 0.764 | 1.183 | 0.408-3.431 |
| genus Intestinimonas | IVW | 16 | -0.15 | 0.39 | 0.696 | 0.859 | 0.4-1.845 |
| genus Intestinimonas | MR Egger | 16 | 0.119 | 1.08 | 0.914 | 1.126 | 0.136-9.322 |
| genus Intestinimonas | Weighted median | 16 | 0.229 | 0.53 | 0.664 | 1.258 | 0.446-3.547 |
| genus Intestinimonas | Weighted mode | 16 | 0.48 | 0.88 | 0.593 | 1.616 | 0.288-9.066 |
| genus Lachnospiraceae FCS020 group | IVW | 12 | -0.16 | 0.5 | 0.745 | 0.851 | 0.321-2.252 |
| genus Lachnospiraceae FCS020 group | MR Egger | 12 | -1.86 | 1.25 | 0.167 | 0.155 | 0.013-1.802 |
| genus Lachnospiraceae FCS020 group | Weighted median | 12 | 0.118 | 0.65 | 0.856 | 1.125 | 0.315-4.017 |
| genus Lachnospiraceae FCS020 group | Weighted mode | 12 | 0.525 | 0.98 | 0.604 | 1.69 | 0.247-11.587 |
| genus Lachnospiraceae UCG001 | IVW | 12 | -0.38 | 0.41 | 0.343 | 0.681 | 0.308-1.506 |
| genus Lachnospiraceae UCG001 | MR Egger | 12 | -0.41 | 1.68 | 0.814 | 0.667 | 0.025-17.828 |
| genus Lachnospiraceae UCG001 | Weighted median | 12 | -0.06 | 0.57 | 0.914 | 0.941 | 0.31-2.853 |
| genus Lachnospiraceae UCG001 | Weighted mode | 12 | 0.043 | 0.91 | 0.963 | 1.044 | 0.176-6.2 |
| genus Lachnospiraceae UCG008 | IVW | 10 | 0.22 | 0.4 | 0.577 | 1.246 | 0.575-2.7 |
| genus Lachnospiraceae UCG008 | MR Egger | 10 | -1.88 | 1.99 | 0.374 | 0.153 | 0.003-7.599 |
| genus Lachnospiraceae UCG008 | Weighted median | 10 | 0.316 | 0.49 | 0.521 | 1.372 | 0.522-3.607 |
| genus Lachnospiraceae UCG008 | Weighted mode | 10 | 0.334 | 0.75 | 0.666 | 1.396 | 0.323-6.043 |
| genus Lactobacillus | IVW | 8 | 0.08 | 0.45 | 0.858 | 1.083 | 0.453-2.592 |
| genus Lactobacillus | MR Egger | 8 | -1.75 | 0.96 | 0.12 | 0.175 | 0.026-1.153 |
| genus Lactobacillus | Weighted median | 8 | -0.25 | 0.5 | 0.623 | 0.781 | 0.291-2.095 |
| genus Lactobacillus | Weighted mode | 8 | -0.34 | 0.63 | 0.608 | 0.713 | 0.206-2.459 |
| genus Marvinbryantia | IVW | 10 | -1.4 | 0.57 | 0.014 | 0.247 | 0.081-0.755 |
| genus Marvinbryantia | MR Egger | 10 | 0.323 | 2.28 | 0.891 | 1.382 | 0.016-121.625 |
| genus Marvinbryantia | Weighted median | 10 | -0.99 | 0.7 | 0.16 | 0.372 | 0.094-1.476 |
| genus Marvinbryantia | Weighted mode | 10 | -1.05 | 0.96 | 0.302 | 0.35 | 0.054-2.291 |
| genus Parabacteroides | IVW | 5 | 0.548 | 0.81 | 0.497 | 1.729 | 0.355-8.412 |
| genus Parabacteroides | MR Egger | 5 | 5.458 | 5.02 | 0.357 | 234.5 | 0.012-4425569.245 |
| genus Parabacteroides | Weighted median | 5 | 1.02 | 1 | 0.306 | 2.772 | 0.393-19.569 |
| genus Parabacteroides | Weighted mode | 5 | 1.233 | 1.45 | 0.444 | 3.43 | 0.199-59.006 |
| genus Peptococcus | IVW | 12 | -0.25 | 0.37 | 0.492 | 0.776 | 0.377-1.599 |
| genus Peptococcus | MR Egger | 12 | -1.23 | 1.45 | 0.418 | 0.294 | 0.017-5.052 |
| genus Peptococcus | Weighted median | 12 | 0.032 | 0.45 | 0.944 | 1.032 | 0.427-2.493 |
| genus Peptococcus | Weighted mode | 12 | 0.143 | 0.7 | 0.842 | 1.154 | 0.292-4.562 |
| genus Romboutsia | IVW | 13 | -0.37 | 0.47 | 0.437 | 0.693 | 0.275-1.745 |
| genus Romboutsia | MR Egger | 13 | 0.513 | 1.37 | 0.715 | 1.671 | 0.114-24.428 |
| genus Romboutsia | Weighted median | 13 | 0.013 | 0.63 | 0.984 | 1.013 | 0.293-3.502 |
| genus Romboutsia | Weighted mode | 13 | 0.645 | 1.15 | 0.585 | 1.906 | 0.2-18.142 |
| genus Ruminiclostridium6 | IVW | 14 | 0.207 | 0.46 | 0.654 | 1.23 | 0.498-3.039 |
| genus Ruminiclostridium6 | MR Egger | 14 | 0.828 | 1.12 | 0.473 | 2.289 | 0.256-20.455 |
| genus Ruminiclostridium6 | Weighted median | 14 | 0.145 | 0.61 | 0.812 | 1.156 | 0.349-3.83 |
| genus Ruminiclostridium6 | Weighted mode | 14 | 0.275 | 0.93 | 0.772 | 1.316 | 0.213-8.117 |
| genus Ruminococcaceae NK4A214 group | IVW | 13 | 0.408 | 0.49 | 0.405 | 1.504 | 0.576-3.924 |
| genus Ruminococcaceae NK4A214 group | MR Egger | 13 | 1.848 | 1.62 | 0.277 | 6.345 | 0.267-150.555 |
| genus Ruminococcaceae NK4A214 group | Weighted median | 13 | 0.25 | 0.68 | 0.711 | 1.284 | 0.342-4.828 |
| genus Ruminococcaceae NK4A214 group | Weighted mode | 13 | 0.059 | 1.13 | 0.96 | 1.06 | 0.115-9.778 |
| genus Ruminococcaceae UCG003 | IVW | 12 | -0.84 | 0.47 | 0.078 | 0.434 | 0.171-1.097 |
| genus Ruminococcaceae UCG003 | MR Egger | 12 | -0.15 | 1.54 | 0.925 | 0.862 | 0.042-17.788 |
| genus Ruminococcaceae UCG003 | Weighted median | 12 | -0.79 | 0.63 | 0.21 | 0.456 | 0.134-1.555 |
| genus Ruminococcaceae UCG003 | Weighted mode | 12 | -0.57 | 0.88 | 0.531 | 0.568 | 0.102-3.154 |
| genus Ruminococcaceae UCG004 | IVW | 11 | -0.17 | 0.59 | 0.777 | 0.846 | 0.266-2.69 |
| genus Ruminococcaceae UCG004 | MR Egger | 11 | -1.86 | 3.41 | 0.599 | 0.156 | 0-124.776 |
| genus Ruminococcaceae UCG004 | Weighted median | 11 | 0.363 | 0.64 | 0.572 | 1.438 | 0.408-5.074 |
| genus Ruminococcaceae UCG004 | Weighted mode | 11 | 0.463 | 1.07 | 0.674 | 1.589 | 0.195-12.935 |
| genus Ruminococcaceae UCG005 | IVW | 14 | -0.61 | 0.5 | 0.221 | 0.543 | 0.204-1.443 |
| genus Ruminococcaceae UCG005 | MR Egger | 14 | 0.574 | 1.36 | 0.681 | 1.775 | 0.123-25.655 |
| genus Ruminococcaceae UCG005 | Weighted median | 14 | -0.07 | 0.64 | 0.914 | 0.933 | 0.267-3.266 |
| genus Ruminococcaceae UCG005 | Weighted mode | 14 | -0.02 | 0.83 | 0.982 | 0.981 | 0.193-4.998 |
| genus Ruminococcaceae UCG009 | IVW | 12 | 0.152 | 0.36 | 0.672 | 1.164 | 0.577-2.348 |
| genus Ruminococcaceae UCG009 | MR Egger | 12 | 0.183 | 1.43 | 0.901 | 1.201 | 0.072-19.926 |
| genus Ruminococcaceae UCG009 | Weighted median | 12 | 0.052 | 0.49 | 0.915 | 1.053 | 0.404-2.746 |
| genus Ruminococcaceae UCG009 | Weighted mode | 12 | 0.076 | 0.76 | 0.922 | 1.079 | 0.243-4.786 |
| genus Ruminococcaceae UCG010 | IVW | 6 | 0.71 | 0.69 | 0.304 | 2.034 | 0.525-7.872 |
| genus Ruminococcaceae UCG010 | MR Egger | 6 | 0.919 | 2.13 | 0.688 | 2.507 | 0.039-161.667 |
| genus Ruminococcaceae UCG010 | Weighted median | 6 | 0.424 | 0.83 | 0.61 | 1.527 | 0.3-7.774 |
| genus Ruminococcaceae UCG010 | Weighted mode | 6 | 0.48 | 1.09 | 0.68 | 1.615 | 0.189-13.802 |
| genus Ruminococcus1 | IVW | 10 | 0.343 | 0.64 | 0.594 | 1.409 | 0.4-4.966 |
| genus Ruminococcus1 | MR Egger | 10 | 1.452 | 1.77 | 0.436 | 4.271 | 0.133-137.604 |
| genus Ruminococcus1 | Weighted median | 10 | 0.048 | 0.76 | 0.95 | 1.049 | 0.235-4.689 |
| genus Ruminococcus1 | Weighted mode | 10 | 0.049 | 1.2 | 0.968 | 1.051 | 0.1-11.085 |
| genus Ruminococcus2 | IVW | 15 | 0.61 | 0.41 | 0.137 | 1.841 | 0.823-4.115 |
| genus Ruminococcus2 | MR Egger | 15 | 1.311 | 0.99 | 0.208 | 3.709 | 0.533-25.796 |
| genus Ruminococcus2 | Weighted median | 15 | 0.965 | 0.58 | 0.098 | 2.625 | 0.837-8.231 |
| genus Ruminococcus2 | Weighted mode | 15 | 0.932 | 0.76 | 0.24 | 2.539 | 0.573-11.26 |
| genus Ruminococcus gnavus group | IVW | 11 | 0.767 | 0.41 | 0.06 | 2.152 | 0.968-4.785 |
| genus Ruminococcus gnavus group | MR Egger | 11 | 1.83 | 2.03 | 0.39 | 6.234 | 0.118-330.237 |
| genus Ruminococcus gnavus group | Weighted median | 11 | 0.816 | 0.5 | 0.104 | 2.262 | 0.844-6.061 |
| genus Ruminococcus gnavus group | Weighted mode | 11 | 0.641 | 0.8 | 0.441 | 1.898 | 0.396-9.098 |
| genus Senegalimassilia | IVW | 5 | 0.47 | 0.68 | 0.49 | 1.6 | 0.421-6.085 |
| genus Senegalimassilia | MR Egger | 5 | -2.39 | 2.51 | 0.411 | 0.092 | 0.001-12.505 |
| genus Senegalimassilia | Weighted median | 5 | 0.727 | 0.79 | 0.36 | 2.068 | 0.436-9.809 |
| genus Senegalimassilia | Weighted mode | 5 | 0.706 | 0.97 | 0.507 | 2.026 | 0.303-13.557 |
| genus Turicibacter | IVW | 9 | -0.69 | 0.48 | 0.152 | 0.501 | 0.194-1.29 |
| genus Turicibacter | MR Egger | 9 | 1.4 | 1.93 | 0.492 | 4.057 | 0.092-178.724 |
| genus Turicibacter | Weighted median | 9 | -0.96 | 0.6 | 0.111 | 0.385 | 0.119-1.246 |
| genus Turicibacter | Weighted mode | 9 | -1.21 | 0.93 | 0.23 | 0.298 | 0.048-1.853 |
| genus Akkermansia | IVW | 11 | -0.25 | 0.47 | 0.594 | 0.78 | 0.312-1.946 |
| genus Akkermansia | MR Egger | 11 | -1.63 | 1.57 | 0.326 | 0.196 | 0.009-4.231 |
| genus Akkermansia | Weighted median | 11 | -0.91 | 0.58 | 0.12 | 0.404 | 0.129-1.268 |
| genus Akkermansia | Weighted mode | 11 | -0.89 | 0.93 | 0.358 | 0.41 | 0.067-2.513 |
| genus Alloprevotella | IVW | 5 | -0.23 | 0.36 | 0.533 | 0.797 | 0.391-1.625 |
| genus Alloprevotella | MR Egger | 5 | 1.175 | 3.15 | 0.734 | 3.239 | 0.007-1563.453 |
| genus Alloprevotella | Weighted median | 5 | -0.35 | 0.49 | 0.471 | 0.705 | 0.273-1.824 |
| genus Alloprevotella | Weighted mode | 5 | -0.36 | 0.68 | 0.621 | 0.696 | 0.184-2.624 |
| genus Bacteroides | IVW | 7 | -0.36 | 0.74 | 0.628 | 0.698 | 0.163-2.983 |
| genus Bacteroides | MR Egger | 7 | -1.37 | 4 | 0.745 | 0.253 | 0-645.73 |
| genus Bacteroides | Weighted median | 7 | -0.29 | 0.97 | 0.765 | 0.749 | 0.113-4.962 |
| genus Bacteroides | Weighted mode | 7 | -1.72 | 1.53 | 0.304 | 0.179 | 0.009-3.614 |
| genus Barnesiella | IVW | 12 | -0.65 | 0.51 | 0.202 | 0.524 | 0.194-1.415 |
| genus Barnesiella | MR Egger | 12 | -2.45 | 2.07 | 0.266 | 0.087 | 0.001-5.056 |
| genus Barnesiella | Weighted median | 12 | -0.67 | 0.69 | 0.335 | 0.512 | 0.131-1.997 |
| genus Barnesiella | Weighted mode | 12 | -0.71 | 1.04 | 0.506 | 0.49 | 0.064-3.751 |
| genus Butyricicoccus | IVW | 8 | -0.14 | 0.59 | 0.809 | 0.866 | 0.271-2.773 |
| genus Butyricicoccus | MR Egger | 8 | 0.762 | 1.17 | 0.538 | 2.142 | 0.218-21.054 |
| genus Butyricicoccus | Weighted median | 8 | 0.058 | 0.75 | 0.939 | 1.059 | 0.244-4.608 |
| genus Butyricicoccus | Weighted mode | 8 | 0.149 | 0.9 | 0.873 | 1.161 | 0.2-6.751 |
| genus Butyricimonas | IVW | 13 | 0.184 | 0.65 | 0.777 | 1.202 | 0.336-4.303 |
| genus Butyricimonas | MR Egger | 13 | -1.61 | 2.35 | 0.508 | 0.2 | 0.002-19.973 |
| genus Butyricimonas | Weighted median | 13 | -0.03 | 0.68 | 0.963 | 0.969 | 0.258-3.638 |
| genus Butyricimonas | Weighted mode | 13 | -0.3 | 1.02 | 0.773 | 0.74 | 0.1-5.463 |
| genus Clostridium sensustricto1 | IVW | 7 | -0.04 | 0.56 | 0.948 | 0.964 | 0.32-2.904 |
| genus Clostridium sensustricto1 | MR Egger | 7 | 1.705 | 1.44 | 0.288 | 5.5 | 0.33-91.687 |
| genus Clostridium sensustricto1 | Weighted median | 7 | 0.412 | 0.73 | 0.574 | 1.51 | 0.359-6.345 |
| genus Clostridium sensustricto1 | Weighted mode | 7 | 0.477 | 0.86 | 0.599 | 1.611 | 0.299-8.686 |
| genus Coprococcus1 | IVW | 11 | -0.3 | 0.56 | 0.596 | 0.741 | 0.245-2.24 |
| genus Coprococcus1 | MR Egger | 11 | -0.16 | 1.47 | 0.916 | 0.852 | 0.048-15.094 |
| genus Coprococcus1 | Weighted median | 11 | 0.145 | 0.72 | 0.84 | 1.156 | 0.282-4.732 |
| genus Coprococcus1 | Weighted mode | 11 | 0.273 | 0.93 | 0.775 | 1.314 | 0.211-8.171 |
| genus Coprococcus2 | IVW | 8 | 0.426 | 0.57 | 0.453 | 1.532 | 0.503-4.662 |
| genus Coprococcus2 | MR Egger | 8 | 1.835 | 4.56 | 0.701 | 6.262 | 0.001-47605.222 |
| genus Coprococcus2 | Weighted median | 8 | 0.552 | 0.77 | 0.476 | 1.737 | 0.381-7.911 |
| genus Coprococcus2 | Weighted mode | 8 | 0.579 | 1.13 | 0.624 | 1.784 | 0.195-16.312 |
| genus Eubacterium hallii group | IVW | 15 | 1.025 | 0.46 | 0.025 | 2.787 | 1.14-6.816 |
| genus Eubacterium hallii group | MR Egger | 15 | 1.308 | 0.99 | 0.209 | 3.7 | 0.533-25.685 |
| genus Eubacterium hallii group | Weighted median | 15 | 1.132 | 0.61 | 0.063 | 3.103 | 0.94-10.245 |
| genus Eubacterium hallii group | Weighted mode | 15 | 0.977 | 0.92 | 0.304 | 2.656 | 0.442-15.969 |
| genus Eubacterium nodatum group | IVW | 11 | -0.28 | 0.26 | 0.278 | 0.753 | 0.452-1.257 |
| genus Eubacterium nodatum group | MR Egger | 11 | 0.271 | 1.16 | 0.821 | 1.311 | 0.134-12.825 |
| genus Eubacterium nodatum group | Weighted median | 11 | -0.04 | 0.35 | 0.919 | 0.965 | 0.489-1.906 |
| genus Eubacterium nodatum group | Weighted mode | 11 | -0.07 | 0.53 | 0.904 | 0.936 | 0.329-2.661 |
| genus Eubacterium oxidoreducens group | IVW | 5 | 0.339 | 0.48 | 0.48 | 1.403 | 0.548-3.594 |
| genus Eubacterium oxidoreducens group | MR Egger | 5 | -0.51 | 1.79 | 0.793 | 0.598 | 0.018-19.864 |
| genus Eubacterium oxidoreducens group | Weighted median | 5 | 0.156 | 0.6 | 0.795 | 1.169 | 0.36-3.795 |
| genus Eubacterium oxidoreducens group | Weighted mode | 5 | 0.05 | 0.8 | 0.954 | 1.051 | 0.218-5.074 |
| genus Eubacterium ventriosum group | IVW | 15 | 0.913 | 0.62 | 0.142 | 2.493 | 0.737-8.433 |
| genus Eubacterium ventriosum group | MR Egger | 15 | 1.882 | 2.87 | 0.523 | 6.565 | 0.024-1818.328 |
| genus Eubacterium ventriosum group | Weighted median | 15 | 1.753 | 0.69 | 0.011 | 5.769 | 1.491-22.317 |
| genus Eubacterium ventriosum group | Weighted mode | 15 | 2.708 | 1.23 | 0.045 | 14.99 | 1.341-167.635 |
| genus Eubacterium xylanophilum group | IVW | 9 | -0.08 | 0.51 | 0.877 | 0.924 | 0.338-2.527 |
| genus Eubacterium xylanophilum group | MR Egger | 9 | 1.24 | 1.54 | 0.446 | 3.457 | 0.17-70.274 |
| genus Eubacterium xylanophilum group | Weighted median | 9 | 0.208 | 0.65 | 0.749 | 1.231 | 0.343-4.414 |
| genus Eubacterium xylanophilum group | Weighted mode | 9 | 0.208 | 0.99 | 0.838 | 1.231 | 0.179-8.486 |
| genus Family XIII AD3011 group | IVW | 13 | -0.41 | 0.49 | 0.403 | 0.665 | 0.256-1.73 |
| genus Family XIII AD3011 group | MR Egger | 13 | 0.026 | 2.31 | 0.991 | 1.026 | 0.011-94.52 |
| genus Family XIII AD3011 group | Weighted median | 13 | -0.58 | 0.64 | 0.372 | 0.563 | 0.159-1.988 |
| genus Family XIII AD3011 group | Weighted mode | 13 | -0.55 | 1.07 | 0.616 | 0.578 | 0.072-4.669 |
| genus Family XIII UCG001 | IVW | 8 | 0.136 | 0.57 | 0.81 | 1.145 | 0.378-3.467 |
| genus Family XIII UCG001 | MR Egger | 8 | -1.45 | 1.74 | 0.438 | 0.235 | 0.008-7.145 |
| genus Family XIII UCG001 | Weighted median | 8 | 0.213 | 0.73 | 0.771 | 1.237 | 0.295-5.192 |
| genus Family XIII UCG001 | Weighted mode | 8 | 1.018 | 1.16 | 0.41 | 2.767 | 0.284-26.973 |
| genus Haemophilus | IVW | 9 | 0.075 | 0.46 | 0.87 | 1.078 | 0.438-2.654 |
| genus Haemophilus | MR Egger | 9 | 1.352 | 0.96 | 0.2 | 3.867 | 0.593-25.234 |
| genus Haemophilus | Weighted median | 9 | 0.243 | 0.56 | 0.666 | 1.275 | 0.424-3.831 |
| genus Haemophilus | Weighted mode | 9 | 0.226 | 0.73 | 0.764 | 1.253 | 0.301-5.216 |
| genus Holdemania | IVW | 14 | -0.65 | 0.37 | 0.082 | 0.523 | 0.252-1.085 |
| genus Holdemania | MR Egger | 14 | 0.199 | 1.1 | 0.859 | 1.22 | 0.141-10.519 |
| genus Holdemania | Weighted median | 14 | -0.22 | 0.52 | 0.674 | 0.805 | 0.293-2.212 |
| genus Holdemania | Weighted mode | 14 | -0.06 | 0.77 | 0.938 | 0.941 | 0.21-4.225 |
| genus Intestinibacter | IVW | 15 | 0.003 | 0.41 | 0.995 | 1.003 | 0.45-2.232 |
| genus Intestinibacter | MR Egger | 15 | -1.43 | 1.33 | 0.304 | 0.24 | 0.018-3.264 |
| genus Intestinibacter | Weighted median | 15 | 0.061 | 0.54 | 0.909 | 1.063 | 0.372-3.037 |
| genus Intestinibacter | Weighted mode | 15 | -0.43 | 0.92 | 0.652 | 0.654 | 0.107-3.985 |
| genus Lachnoclostridium | IVW | 13 | -0.18 | 0.6 | 0.76 | 0.833 | 0.257-2.696 |
| genus Lachnoclostridium | MR Egger | 13 | 0.532 | 2.12 | 0.806 | 1.702 | 0.027-108.223 |
| genus Lachnoclostridium | Weighted median | 13 | 0.442 | 0.76 | 0.56 | 1.555 | 0.352-6.867 |
| genus Lachnoclostridium | Weighted mode | 13 | 0.521 | 1.16 | 0.661 | 1.685 | 0.173-16.365 |
| genus Lachnospiraceae NC2004 group | IVW | 9 | 0.06 | 0.43 | 0.889 | 1.062 | 0.46-2.449 |
| genus Lachnospiraceae NC2004 group | MR Egger | 9 | 1.126 | 1.81 | 0.553 | 3.084 | 0.09-106.194 |
| genus Lachnospiraceae NC2004 group | Weighted median | 9 | -0.39 | 0.55 | 0.482 | 0.68 | 0.233-1.99 |
| genus Lachnospiraceae NC2004 group | Weighted mode | 9 | -0.38 | 0.92 | 0.688 | 0.682 | 0.113-4.12 |
| genus Lachnospiraceae ND3007 group | IVW | 3 | -1.33 | 1.1 | 0.223 | 0.263 | 0.031-2.252 |
| genus Lachnospiraceae ND3007 group | MR Egger | 3 | 1.825 | 18.4 | 0.937 | 6.202 | 0-30112716984393300 |
| genus Lachnospiraceae ND3007 group | Weighted median | 3 | -1.42 | 1.31 | 0.277 | 0.241 | 0.019-3.134 |
| genus Lachnospiraceae ND3007 group | Weighted mode | 3 | -1.46 | 1.44 | 0.418 | 0.232 | 0.014-3.933 |
| genus Lachnospiraceae NK4A136 group | IVW | 15 | -0.64 | 0.51 | 0.208 | 0.526 | 0.194-1.43 |
| genus Lachnospiraceae NK4A136 group | MR Egger | 15 | -0.52 | 1.06 | 0.633 | 0.597 | 0.075-4.731 |
| genus Lachnospiraceae NK4A136 group | Weighted median | 15 | -0.77 | 0.65 | 0.237 | 0.461 | 0.128-1.663 |
| genus Lachnospiraceae NK4A136 group | Weighted mode | 15 | -0.85 | 0.72 | 0.26 | 0.428 | 0.104-1.764 |
| genus Lactococcus | IVW | 9 | 0.329 | 0.32 | 0.303 | 1.39 | 0.743-2.6 |
| genus Lactococcus | MR Egger | 9 | 0.192 | 1.44 | 0.897 | 1.212 | 0.073-20.185 |
| genus Lactococcus | Weighted median | 9 | 0.01 | 0.44 | 0.981 | 1.01 | 0.43-2.374 |
| genus Lactococcus | Weighted mode | 9 | -0.21 | 0.62 | 0.746 | 0.811 | 0.239-2.748 |
| genus Methanobrevibacter | IVW | 6 | -0.01 | 0.4 | 0.978 | 0.989 | 0.452-2.163 |
| genus Methanobrevibacter | MR Egger | 6 | 0.637 | 1.64 | 0.717 | 1.892 | 0.076-46.793 |
| genus Methanobrevibacter | Weighted median | 6 | -0.07 | 0.52 | 0.889 | 0.93 | 0.335-2.583 |
| genus Methanobrevibacter | Weighted mode | 6 | -0.74 | 0.87 | 0.433 | 0.475 | 0.086-2.628 |
| genus Odoribacter | IVW | 7 | -0.36 | 0.82 | 0.657 | 0.695 | 0.14-3.454 |
| genus Odoribacter | MR Egger | 7 | 4.041 | 2.04 | 0.104 | 56.91 | 1.053-3075.526 |
| genus Odoribacter | Weighted median | 7 | -0.23 | 0.9 | 0.8 | 0.795 | 0.136-4.662 |
| genus Odoribacter | Weighted mode | 7 | -0.31 | 1.31 | 0.821 | 0.733 | 0.056-9.635 |
| genus Oscillospira | IVW | 8 | 0.482 | 0.51 | 0.346 | 1.62 | 0.594-4.415 |
| genus Oscillospira | MR Egger | 8 | 0.02 | 2.2 | 0.993 | 1.021 | 0.014-75.96 |
| genus Oscillospira | Weighted median | 8 | 0.948 | 0.69 | 0.168 | 2.581 | 0.67-9.946 |
| genus Oscillospira | Weighted mode | 8 | 1.016 | 0.94 | 0.314 | 2.763 | 0.441-17.32 |
| genus Oxalobacter | IVW | 11 | 0.359 | 0.29 | 0.21 | 1.433 | 0.817-2.512 |
| genus Oxalobacter | MR Egger | 11 | -0.25 | 1.35 | 0.859 | 0.781 | 0.056-10.917 |
| genus Oxalobacter | Weighted median | 11 | 0.17 | 0.36 | 0.64 | 1.185 | 0.582-2.415 |
| genus Oxalobacter | Weighted mode | 11 | 0.208 | 0.52 | 0.697 | 1.231 | 0.446-3.401 |
| genus Prevotella7 | IVW | 11 | -0.13 | 0.27 | 0.643 | 0.882 | 0.518-1.501 |
| genus Prevotella7 | MR Egger | 11 | 2.144 | 1.58 | 0.207 | 8.534 | 0.388-187.724 |
| genus Prevotella7 | Weighted median | 11 | -0.05 | 0.36 | 0.899 | 0.955 | 0.469-1.942 |
| genus Prevotella7 | Weighted mode | 11 | -0.14 | 0.54 | 0.797 | 0.866 | 0.299-2.512 |
| genus Roseburia | IVW | 13 | 0.614 | 0.54 | 0.252 | 1.847 | 0.646-5.283 |
| genus Roseburia | MR Egger | 13 | 1.3 | 1.61 | 0.438 | 3.671 | 0.155-86.861 |
| genus Roseburia | Weighted median | 13 | 0.954 | 0.73 | 0.188 | 2.597 | 0.627-10.764 |
| genus Roseburia | Weighted mode | 13 | 1.254 | 1.17 | 0.303 | 3.503 | 0.356-34.436 |
| genus Ruminiclostridium5 | IVW | 11 | -0.34 | 0.62 | 0.576 | 0.709 | 0.212-2.368 |
| genus Ruminiclostridium5 | MR Egger | 11 | -0.34 | 2.71 | 0.902 | 0.709 | 0.003-144.071 |
| genus Ruminiclostridium5 | Weighted median | 11 | 0.269 | 0.81 | 0.738 | 1.309 | 0.27-6.342 |
| genus Ruminiclostridium5 | Weighted mode | 11 | -0.24 | 1.21 | 0.85 | 0.79 | 0.074-8.474 |
| genus Ruminococcaceae UCG011 | IVW | 8 | 0.332 | 0.32 | 0.304 | 1.394 | 0.74-2.623 |
| genus Ruminococcaceae UCG011 | MR Egger | 8 | 1.369 | 1.69 | 0.448 | 3.93 | 0.144-107.461 |
| genus Ruminococcaceae UCG011 | Weighted median | 8 | 0.327 | 0.41 | 0.426 | 1.387 | 0.62-3.101 |
| genus Ruminococcaceae UCG011 | Weighted mode | 8 | 0.31 | 0.59 | 0.614 | 1.363 | 0.432-4.303 |
| genus Ruminococcaceae UCG013 | IVW | 11 | 0.15 | 0.65 | 0.817 | 1.162 | 0.326-4.138 |
| genus Ruminococcaceae UCG013 | MR Egger | 11 | 2.264 | 1.82 | 0.244 | 9.62 | 0.274-338.04 |
| genus Ruminococcaceae UCG013 | Weighted median | 11 | 0.406 | 0.76 | 0.593 | 1.5 | 0.339-6.637 |
| genus Ruminococcaceae UCG013 | Weighted mode | 11 | 0.733 | 1.11 | 0.525 | 2.082 | 0.235-18.457 |
| genus Ruminococcaceae UCG014 | IVW | 11 | 0.557 | 0.53 | 0.293 | 1.745 | 0.618-4.927 |
| genus Ruminococcaceae UCG014 | MR Egger | 11 | -0.53 | 1.26 | 0.685 | 0.59 | 0.05-6.941 |
| genus Ruminococcaceae UCG014 | Weighted median | 11 | 0.253 | 0.71 | 0.721 | 1.287 | 0.322-5.144 |
| genus Ruminococcaceae UCG014 | Weighted mode | 11 | -0.05 | 0.79 | 0.95 | 0.95 | 0.202-4.474 |
| genus Ruminococcus gauvreauii group | IVW | 11 | 0.293 | 0.5 | 0.555 | 1.34 | 0.507-3.543 |
| genus Ruminococcus gauvreauii group | MR Egger | 11 | 1.532 | 2.04 | 0.472 | 4.629 | 0.085-252.752 |
| genus Ruminococcus gauvreauii group | Weighted median | 11 | 0.016 | 0.66 | 0.98 | 1.017 | 0.282-3.67 |
| genus Ruminococcus gauvreauii group | Weighted mode | 11 | -0.21 | 1.01 | 0.84 | 0.811 | 0.111-5.903 |
| genus Streptococcus | IVW | 12 | 0 | 0.55 | 0.998 | 0.999 | 0.342-2.919 |
| genus Streptococcus | MR Egger | 12 | -2.58 | 2.02 | 0.229 | 0.076 | 0.001-3.943 |
| genus Streptococcus | Weighted median | 12 | -0.4 | 0.7 | 0.571 | 0.673 | 0.171-2.649 |
| genus Streptococcus | Weighted mode | 12 | -0.52 | 1.09 | 0.647 | 0.598 | 0.07-5.085 |
| genus Tyzzerella3 | IVW | 13 | -0.25 | 0.3 | 0.399 | 0.776 | 0.43-1.4 |
| genus Tyzzerella3 | MR Egger | 13 | 1.622 | 1.73 | 0.369 | 5.061 | 0.171-150.077 |
| genus Tyzzerella3 | Weighted median | 13 | -0.09 | 0.41 | 0.82 | 0.911 | 0.407-2.037 |
| genus Tyzzerella3 | Weighted mode | 13 | 0.012 | 0.67 | 0.986 | 1.012 | 0.271-3.779 |
| genus Veillonella | IVW | 6 | 0.987 | 0.62 | 0.113 | 2.682 | 0.791-9.095 |
| genus Veillonella | MR Egger | 6 | 1.662 | 5.51 | 0.778 | 5.272 | 0-260100.321 |
| genus Veillonella | Weighted median | 6 | 1.214 | 0.78 | 0.119 | 3.366 | 0.731-15.511 |
| genus Veillonella | Weighted mode | 6 | 1.586 | 1.21 | 0.245 | 4.884 | 0.461-51.782 |
| genus Victivallis | IVW | 10 | 0.304 | 0.35 | 0.389 | 1.355 | 0.678-2.709 |
| genus Victivallis | MR Egger | 10 | 2.351 | 2.76 | 0.42 | 10.49 | 0.047-2358.17 |
| genus Victivallis | Weighted median | 10 | 0.399 | 0.42 | 0.345 | 1.491 | 0.651-3.411 |
| genus Victivallis | Weighted mode | 10 | 0.851 | 0.65 | 0.22 | 2.341 | 0.662-8.283 |
| genus Alistipes | IVW | 12 | -0.09 | 0.61 | 0.888 | 0.918 | 0.28-3.013 |
| genus Alistipes | MR Egger | 12 | -3.95 | 2.89 | 0.201 | 0.019 | 0-5.485 |
| genus Alistipes | Weighted median | 12 | -0.39 | 0.82 | 0.629 | 0.674 | 0.137-3.329 |
| genus Alistipes | Weighted mode | 12 | -0.94 | 1.4 | 0.517 | 0.391 | 0.025-6.106 |
| genus Allisonella | IVW | 8 | -0.14 | 0.3 | 0.653 | 0.874 | 0.486-1.571 |
| genus Allisonella | MR Egger | 8 | 2.309 | 1.94 | 0.279 | 10.07 | 0.225-451.247 |
| genus Allisonella | Weighted median | 8 | -0.29 | 0.37 | 0.438 | 0.75 | 0.363-1.552 |
| genus Allisonella | Weighted mode | 8 | -0.22 | 0.55 | 0.697 | 0.8 | 0.272-2.351 |
| genus Anaerotruncus | IVW | 13 | -0.29 | 0.52 | 0.582 | 0.752 | 0.272-2.079 |
| genus Anaerotruncus | MR Egger | 13 | -1.84 | 1.51 | 0.25 | 0.159 | 0.008-3.09 |
| genus Anaerotruncus | Weighted median | 13 | -0.64 | 0.66 | 0.329 | 0.526 | 0.145-1.912 |
| genus Anaerotruncus | Weighted mode | 13 | -1.08 | 1.07 | 0.336 | 0.341 | 0.042-2.798 |
| genus Bifidobacterium | IVW | 12 | 0.446 | 0.43 | 0.298 | 1.563 | 0.674-3.621 |
| genus Bifidobacterium | MR Egger | 12 | 0.558 | 1.05 | 0.605 | 1.747 | 0.225-13.561 |
| genus Bifidobacterium | Weighted median | 12 | 0.324 | 0.56 | 0.565 | 1.382 | 0.459-4.164 |
| genus Bifidobacterium | Weighted mode | 12 | 0.36 | 0.67 | 0.599 | 1.433 | 0.39-5.271 |
| genus Bilophila | IVW | 13 | 0.303 | 0.48 | 0.532 | 1.354 | 0.524-3.497 |
| genus Bilophila | MR Egger | 13 | -2.59 | 2.38 | 0.3 | 0.075 | 0.001-7.973 |
| genus Bilophila | Weighted median | 13 | 0.301 | 0.66 | 0.648 | 1.351 | 0.371-4.921 |
| genus Bilophila | Weighted mode | 13 | 0.338 | 1.13 | 0.769 | 1.403 | 0.154-12.771 |
| genus Blautia | IVW | 2 | -0.19 | 1.41 | 0.892 | 0.826 | 0.053-12.968 |
| genus Candidatus Soleaferrea | IVW | 9 | -0.22 | 0.38 | 0.558 | 0.8 | 0.378-1.691 |
| genus Candidatus Soleaferrea | MR Egger | 9 | 4.769 | 3.9 | 0.261 | 117.8 | 0.056-246831.933 |
| genus Candidatus Soleaferrea | Weighted median | 9 | -0.47 | 0.54 | 0.382 | 0.626 | 0.219-1.792 |
| genus Candidatus Soleaferrea | Weighted mode | 9 | -0.38 | 0.81 | 0.655 | 0.687 | 0.141-3.353 |
| genus Coprobacter | IVW | 10 | -0.46 | 0.38 | 0.224 | 0.63 | 0.299-1.326 |
| genus Coprobacter | MR Egger | 10 | 0.293 | 1.42 | 0.842 | 1.34 | 0.083-21.685 |
| genus Coprobacter | Weighted median | 10 | -0.13 | 0.49 | 0.783 | 0.874 | 0.336-2.276 |
| genus Coprobacter | Weighted mode | 10 | 0.082 | 0.76 | 0.916 | 1.086 | 0.245-4.808 |
| genus Coprococcus3 | IVW | 9 | -0.67 | 0.64 | 0.301 | 0.514 | 0.146-1.813 |
| genus Coprococcus3 | MR Egger | 9 | -1.4 | 3.55 | 0.706 | 0.247 | 0-259.585 |
| genus Coprococcus3 | Weighted median | 9 | -0.89 | 0.86 | 0.301 | 0.41 | 0.075-2.223 |
| genus Coprococcus3 | Weighted mode | 9 | -1.1 | 1.33 | 0.435 | 0.334 | 0.025-4.552 |
| genus Dorea | IVW | 10 | 0.408 | 0.66 | 0.537 | 1.504 | 0.412-5.494 |
| genus Dorea | MR Egger | 10 | 3.376 | 1.73 | 0.087 | 29.25 | 0.981-872.081 |
| genus Dorea | Weighted median | 10 | -0.12 | 0.9 | 0.896 | 0.89 | 0.154-5.139 |
| genus Dorea | Weighted mode | 10 | -0.49 | 1.68 | 0.776 | 0.612 | 0.023-16.385 |
| genus Erysipelatoclostridium | IVW | 15 | 0.491 | 0.39 | 0.203 | 1.634 | 0.767-3.478 |
| genus Erysipelatoclostridium | MR Egger | 15 | 1.368 | 1.55 | 0.394 | 3.926 | 0.188-82.08 |
| genus Erysipelatoclostridium | Weighted median | 15 | 0.64 | 0.52 | 0.216 | 1.896 | 0.688-5.224 |
| genus Erysipelatoclostridium | Weighted mode | 15 | 0.916 | 0.9 | 0.328 | 2.499 | 0.425-14.679 |
| genus Escherichia Shigella | IVW | 10 | -0.66 | 0.84 | 0.431 | 0.515 | 0.099-2.678 |
| genus Escherichia Shigella | MR Egger | 10 | 0.679 | 2.73 | 0.809 | 1.973 | 0.009-411.403 |
| genus Escherichia Shigella | Weighted median | 10 | -0.82 | 0.81 | 0.314 | 0.442 | 0.09-2.163 |
| genus Escherichia Shigella | Weighted mode | 10 | -1.05 | 1.18 | 0.397 | 0.349 | 0.034-3.542 |
| genus Eubacterium brachy group | IVW | 10 | 0.459 | 0.3 | 0.126 | 1.583 | 0.878-2.854 |
| genus Eubacterium brachy group | MR Egger | 10 | -0.01 | 1.21 | 0.992 | 0.987 | 0.092-10.542 |
| genus Eubacterium brachy group | Weighted median | 10 | 0.479 | 0.38 | 0.213 | 1.614 | 0.76-3.426 |
| genus Eubacterium brachy group | Weighted mode | 10 | 0.671 | 0.61 | 0.298 | 1.956 | 0.595-6.428 |
| genus Eubacterium coprostanoligenes group | IVW | 13 | 0.1 | 0.55 | 0.857 | 1.105 | 0.373-3.274 |
| genus Eubacterium coprostanoligenes group | MR Egger | 13 | 0.717 | 2.18 | 0.748 | 2.048 | 0.029-145.549 |
| genus Eubacterium coprostanoligenes group | Weighted median | 13 | -0.07 | 0.72 | 0.923 | 0.933 | 0.227-3.831 |
| genus Eubacterium coprostanoligenes group | Weighted mode | 13 | -0.19 | 1.22 | 0.882 | 0.83 | 0.076-9.114 |
| genus Eubacterium eligens group | IVW | 6 | 0.151 | 0.72 | 0.834 | 1.163 | 0.282-4.802 |
| genus Eubacterium eligens group | MR Egger | 6 | -2.75 | 2.67 | 0.361 | 0.064 | 0-11.897 |
| genus Eubacterium eligens group | Weighted median | 6 | -0.21 | 0.95 | 0.826 | 0.811 | 0.126-5.228 |
| genus Eubacterium eligens group | Weighted mode | 6 | -1.02 | 1.45 | 0.512 | 0.359 | 0.021-6.167 |
| genus Eubacterium ruminantium group | IVW | 18 | 0.018 | 0.3 | 0.953 | 1.018 | 0.569-1.819 |
| genus Eubacterium ruminantium group | MR Egger | 18 | -1.17 | 0.97 | 0.246 | 0.31 | 0.046-2.085 |
| genus Eubacterium ruminantium group | Weighted median | 18 | 0.091 | 0.43 | 0.831 | 1.095 | 0.474-2.529 |
| genus Eubacterium ruminantium group | Weighted mode | 18 | 0.643 | 0.86 | 0.463 | 1.902 | 0.355-10.201 |
| genus Faecalibacterium | IVW | 10 | -0.82 | 0.6 | 0.176 | 0.442 | 0.136-1.441 |
| genus Faecalibacterium | MR Egger | 10 | -0.38 | 1.23 | 0.769 | 0.687 | 0.061-7.716 |
| genus Faecalibacterium | Weighted median | 10 | -0.57 | 0.64 | 0.373 | 0.566 | 0.162-1.98 |
| genus Faecalibacterium | Weighted mode | 10 | -0.42 | 0.7 | 0.559 | 0.656 | 0.168-2.561 |
| genus Flavonifractor | IVW | 5 | -0.2 | 0.66 | 0.767 | 0.822 | 0.224-3.016 |
| genus Flavonifractor | MR Egger | 5 | -0.57 | 2.65 | 0.844 | 0.567 | 0.003-101.699 |
| genus Flavonifractor | Weighted median | 5 | 0.224 | 0.83 | 0.787 | 1.252 | 0.245-6.391 |
| genus Flavonifractor | Weighted mode | 5 | 0.365 | 1.12 | 0.761 | 1.44 | 0.16-12.986 |
| genus Holdemanella | IVW | 11 | 0.201 | 0.42 | 0.63 | 1.222 | 0.541-2.763 |
| genus Holdemanella | MR Egger | 11 | -0.68 | 1.22 | 0.591 | 0.508 | 0.047-5.516 |
| genus Holdemanella | Weighted median | 11 | 0.753 | 0.51 | 0.136 | 2.123 | 0.789-5.712 |
| genus Holdemanella | Weighted mode | 11 | 1.04 | 0.88 | 0.266 | 2.829 | 0.501-15.98 |
| genus Howardella | IVW | 9 | 0.197 | 0.28 | 0.485 | 1.218 | 0.701-2.116 |
| genus Howardella | MR Egger | 9 | 0.513 | 1.12 | 0.662 | 1.67 | 0.185-15.091 |
| genus Howardella | Weighted median | 9 | 0.309 | 0.38 | 0.418 | 1.361 | 0.645-2.874 |
| genus Howardella | Weighted mode | 9 | 0.411 | 0.54 | 0.471 | 1.508 | 0.521-4.366 |
| genus Hungatella | IVW | 5 | 0.295 | 0.43 | 0.494 | 1.342 | 0.578-3.121 |
| genus Hungatella | MR Egger | 5 | 0.758 | 2.61 | 0.79 | 2.134 | 0.013-354.522 |
| genus Hungatella | Weighted median | 5 | 0.468 | 0.52 | 0.372 | 1.597 | 0.572-4.462 |
| genus Hungatella | Weighted mode | 5 | 0.533 | 0.61 | 0.433 | 1.705 | 0.514-5.655 |
| genus Lachnospiraceae UCG004 | IVW | 12 | -0.06 | 0.6 | 0.928 | 0.947 | 0.29-3.087 |
| genus Lachnospiraceae UCG004 | MR Egger | 12 | 2.162 | 2.53 | 0.413 | 8.689 | 0.061-1234.161 |
| genus Lachnospiraceae UCG004 | Weighted median | 12 | 0.4 | 0.74 | 0.588 | 1.491 | 0.351-6.339 |
| genus Lachnospiraceae UCG004 | Weighted mode | 12 | 1.059 | 1.22 | 0.403 | 2.884 | 0.265-31.409 |
| genus Lachnospiraceae UCG010 | IVW | 10 | -0.09 | 0.55 | 0.866 | 0.912 | 0.312-2.667 |
| genus Lachnospiraceae UCG010 | MR Egger | 10 | 0.984 | 1.67 | 0.571 | 2.676 | 0.102-70.42 |
| genus Lachnospiraceae UCG010 | Weighted median | 10 | 0.161 | 0.68 | 0.813 | 1.175 | 0.31-4.444 |
| genus Lachnospiraceae UCG010 | Weighted mode | 10 | 0.309 | 0.92 | 0.744 | 1.362 | 0.226-8.219 |
| genus Olsenella | IVW | 10 | 0.33 | 0.38 | 0.388 | 1.391 | 0.657-2.944 |
| genus Olsenella | MR Egger | 10 | 1.194 | 1.26 | 0.371 | 3.3 | 0.279-39.013 |
| genus Olsenella | Weighted median | 10 | 0.734 | 0.38 | 0.055 | 2.083 | 0.983-4.414 |
| genus Olsenella | Weighted mode | 10 | 0.818 | 0.49 | 0.13 | 2.267 | 0.866-5.932 |
| genus Oscillibacter | IVW | 13 | -0.38 | 0.37 | 0.31 | 0.684 | 0.329-1.424 |
| genus Oscillibacter | MR Egger | 13 | 0.406 | 1.42 | 0.78 | 1.501 | 0.093-24.134 |
| genus Oscillibacter | Weighted median | 13 | -0.34 | 0.48 | 0.482 | 0.715 | 0.28-1.824 |
| genus Oscillibacter | Weighted mode | 13 | -0.28 | 0.76 | 0.72 | 0.758 | 0.172-3.345 |
| genus Paraprevotella | IVW | 13 | 0.128 | 0.32 | 0.689 | 1.137 | 0.608-2.126 |
| genus Paraprevotella | MR Egger | 13 | 1.873 | 1.21 | 0.15 | 6.506 | 0.606-69.841 |
| genus Paraprevotella | Weighted median | 13 | 0.479 | 0.43 | 0.266 | 1.614 | 0.694-3.752 |
| genus Paraprevotella | Weighted mode | 13 | 0.709 | 0.63 | 0.279 | 2.032 | 0.597-6.917 |
| genus Parasutterella | IVW | 14 | 0.169 | 0.54 | 0.754 | 1.184 | 0.413-3.396 |
| genus Parasutterella | MR Egger | 14 | -0.05 | 1.55 | 0.974 | 0.951 | 0.046-19.823 |
| genus Parasutterella | Weighted median | 14 | 0.538 | 0.57 | 0.349 | 1.712 | 0.556-5.271 |
| genus Parasutterella | Weighted mode | 14 | 0.645 | 0.72 | 0.385 | 1.906 | 0.467-7.774 |
| ebi-a-GCST90017043 | IVW | 7 | -0.08 | 0.59 | 0.893 | 0.924 | 0.288-2.958 |
| ebi-a-GCST90017043 | MR Egger | 7 | -3.08 | 2.82 | 0.325 | 0.046 | 0-11.629 |
| ebi-a-GCST90017043 | Weighted median | 7 | -0.07 | 0.76 | 0.929 | 0.935 | 0.211-4.131 |
| ebi-a-GCST90017043 | Weighted mode | 7 | -1.3 | 1.29 | 0.355 | 0.274 | 0.022-3.446 |
| genus Prevotella9 | IVW | 15 | -0.33 | 0.36 | 0.351 | 0.717 | 0.357-1.442 |
| genus Prevotella9 | MR Egger | 15 | -0.73 | 1.07 | 0.508 | 0.482 | 0.059-3.943 |
| genus Prevotella9 | Weighted median | 15 | -0.75 | 0.49 | 0.125 | 0.473 | 0.182-1.23 |
| genus Prevotella9 | Weighted mode | 15 | -1.13 | 0.79 | 0.174 | 0.325 | 0.07-1.514 |
| genus Rikenellaceae RC9 gut group | IVW | 11 | -0.02 | 0.35 | 0.946 | 0.977 | 0.493-1.935 |
| genus Rikenellaceae RC9 gut group | MR Egger | 11 | 0.475 | 2.29 | 0.84 | 1.607 | 0.018-141.875 |
| genus Rikenellaceae RC9 gut group | Weighted median | 11 | -0.21 | 0.39 | 0.585 | 0.808 | 0.377-1.735 |
| genus Rikenellaceae RC9 gut group | Weighted mode | 11 | -0.67 | 0.68 | 0.343 | 0.51 | 0.136-1.921 |
| genus Ruminiclostridium9 | IVW | 7 | 0.194 | 0.74 | 0.792 | 1.214 | 0.288-5.124 |
| genus Ruminiclostridium9 | MR Egger | 7 | 1.879 | 3.34 | 0.598 | 6.544 | 0.009-4580.608 |
| genus Ruminiclostridium9 | Weighted median | 7 | -0.01 | 0.94 | 0.994 | 0.993 | 0.157-6.285 |
| genus Ruminiclostridium9 | Weighted mode | 7 | -0.07 | 1.33 | 0.962 | 0.936 | 0.069-12.694 |
| genus Ruminococcaceae UCG002 | IVW | 19 | 0.092 | 0.39 | 0.815 | 1.096 | 0.507-2.372 |
| genus Ruminococcaceae UCG002 | MR Egger | 19 | -0.06 | 1.01 | 0.957 | 0.946 | 0.131-6.827 |
| genus Ruminococcaceae UCG002 | Weighted median | 19 | 0.325 | 0.57 | 0.565 | 1.385 | 0.457-4.193 |
| genus Ruminococcaceae UCG002 | Weighted mode | 19 | 0.519 | 0.8 | 0.525 | 1.68 | 0.35-8.062 |
| genus Ruminococcus torques group | IVW | 7 | -0.44 | 1.02 | 0.666 | 0.645 | 0.088-4.744 |
| genus Ruminococcus torques group | MR Egger | 7 | -0.91 | 3.39 | 0.799 | 0.401 | 0.001-310.469 |
| genus Ruminococcus torques group | Weighted median | 7 | 0.889 | 1.1 | 0.417 | 2.433 | 0.285-20.795 |
| genus Ruminococcus torques group | Weighted mode | 7 | 1.215 | 1.36 | 0.405 | 3.369 | 0.237-47.985 |
| genus Sellimonas | IVW | 9 | 0.005 | 0.27 | 0.984 | 1.005 | 0.597-1.692 |
| genus Sellimonas | MR Egger | 9 | -0.46 | 1.64 | 0.786 | 0.631 | 0.026-15.543 |
| genus Sellimonas | Weighted median | 9 | -0.06 | 0.35 | 0.869 | 0.944 | 0.475-1.875 |
| genus Sellimonas | Weighted mode | 9 | 0.062 | 0.51 | 0.905 | 1.064 | 0.394-2.878 |
| genus Slackia | IVW | 6 | 0.408 | 0.55 | 0.457 | 1.504 | 0.513-4.411 |
| genus Slackia | MR Egger | 6 | -4.89 | 3.18 | 0.199 | 0.008 | 0-3.806 |
| genus Slackia | Weighted median | 6 | 0.289 | 0.63 | 0.648 | 1.335 | 0.386-4.615 |
| genus Slackia | Weighted mode | 6 | -0.42 | 1.07 | 0.715 | 0.661 | 0.081-5.408 |
| genus Subdoligranulum | IVW | 10 | 0.272 | 0.55 | 0.62 | 1.313 | 0.447-3.857 |
| genus Subdoligranulum | MR Egger | 10 | 0.089 | 1.41 | 0.951 | 1.093 | 0.07-17.173 |
| genus Subdoligranulum | Weighted median | 10 | -0.04 | 0.75 | 0.953 | 0.957 | 0.222-4.127 |
| genus Subdoligranulum | Weighted mode | 10 | -0.02 | 1.01 | 0.984 | 0.979 | 0.135-7.077 |
| genus Sutterella | IVW | 12 | 0.182 | 0.6 | 0.76 | 1.2 | 0.373-3.859 |
| genus Sutterella | MR Egger | 12 | 0.728 | 2.71 | 0.794 | 2.07 | 0.01-416.871 |
| genus Sutterella | Weighted median | 12 | 0.503 | 0.73 | 0.489 | 1.653 | 0.398-6.87 |
| genus Sutterella | Weighted mode | 12 | 0.573 | 1.11 | 0.615 | 1.774 | 0.202-15.557 |
| genus Terrisporobacter | IVW | 5 | -0.2 | 0.64 | 0.756 | 0.821 | 0.236-2.855 |
| genus Terrisporobacter | MR Egger | 5 | -2.74 | 1.68 | 0.201 | 0.064 | 0.002-1.729 |
| genus Terrisporobacter | Weighted median | 5 | -1.11 | 0.72 | 0.125 | 0.331 | 0.08-1.362 |
| genus Terrisporobacter | Weighted mode | 5 | -1.22 | 1.14 | 0.347 | 0.297 | 0.032-2.777 |
| order Actinomycetales | IVW | 4 | -0.13 | 0.59 | 0.821 | 0.876 | 0.276-2.777 |
| order Actinomycetales | MR Egger | 4 | 0.472 | 1.4 | 0.767 | 1.603 | 0.104-24.756 |
| order Actinomycetales | Weighted median | 4 | 0.272 | 0.69 | 0.696 | 1.312 | 0.336-5.116 |
| order Actinomycetales | Weighted mode | 4 | 0.331 | 0.75 | 0.69 | 1.392 | 0.318-6.093 |
| order Bacillales | IVW | 8 | 0.329 | 0.29 | 0.26 | 1.39 | 0.784-2.465 |
| order Bacillales | MR Egger | 8 | 0.719 | 1.18 | 0.564 | 2.053 | 0.204-20.655 |
| order Bacillales | Weighted median | 8 | 0.539 | 0.38 | 0.156 | 1.715 | 0.814-3.611 |
| order Bacillales | Weighted mode | 8 | 0.644 | 0.55 | 0.279 | 1.904 | 0.649-5.591 |
| order Bacteroidales | IVW | 13 | -0.24 | 0.72 | 0.739 | 0.787 | 0.192-3.23 |
| order Bacteroidales | MR Egger | 13 | -2.75 | 1.55 | 0.103 | 0.064 | 0.003-1.32 |
| order Bacteroidales | Weighted median | 13 | -0.56 | 0.79 | 0.479 | 0.572 | 0.122-2.686 |
| order Bacteroidales | Weighted mode | 13 | -1.09 | 1.13 | 0.352 | 0.336 | 0.037-3.061 |
| order Bifidobacteriales | IVW | 11 | 0.375 | 0.47 | 0.426 | 1.454 | 0.578-3.657 |
| order Bifidobacteriales | MR Egger | 11 | 0.701 | 1.53 | 0.658 | 2.015 | 0.1-40.535 |
| order Bifidobacteriales | Weighted median | 11 | 0.625 | 0.67 | 0.351 | 1.868 | 0.503-6.934 |
| order Bifidobacteriales | Weighted mode | 11 | 0.686 | 0.76 | 0.39 | 1.986 | 0.444-8.886 |
| order Burkholderiales | IVW | 10 | -0.68 | 0.58 | 0.241 | 0.505 | 0.161-1.581 |
| order Burkholderiales | MR Egger | 10 | 2.229 | 1.84 | 0.261 | 9.29 | 0.252-343.02 |
| order Burkholderiales | Weighted median | 10 | -0.56 | 0.75 | 0.458 | 0.572 | 0.131-2.506 |
| order Burkholderiales | Weighted mode | 10 | -0.59 | 1.04 | 0.586 | 0.556 | 0.072-4.273 |
| order Clostridiales | IVW | 13 | -0.21 | 0.57 | 0.713 | 0.811 | 0.267-2.469 |
| order Clostridiales | MR Egger | 13 | -0.47 | 1.61 | 0.778 | 0.627 | 0.027-14.839 |
| order Clostridiales | Weighted median | 13 | 0.307 | 0.72 | 0.667 | 1.359 | 0.335-5.516 |
| order Clostridiales | Weighted mode | 13 | 0.251 | 0.99 | 0.804 | 1.286 | 0.185-8.943 |
| order Coriobacteriales | IVW | 13 | -0.42 | 0.65 | 0.515 | 0.655 | 0.184-2.34 |
| order Coriobacteriales | MR Egger | 13 | 2.357 | 2.75 | 0.409 | 10.56 | 0.049-2295.843 |
| order Coriobacteriales | Weighted median | 13 | -0.29 | 0.72 | 0.691 | 0.751 | 0.183-3.078 |
| order Coriobacteriales | Weighted mode | 13 | 1.092 | 1.41 | 0.454 | 2.981 | 0.188-47.278 |
| order Desulfovibrionales | IVW | 10 | -0.24 | 0.8 | 0.763 | 0.787 | 0.165-3.745 |
| order Desulfovibrionales | MR Egger | 10 | -3.13 | 4.04 | 0.461 | 0.044 | 0-119.797 |
| order Desulfovibrionales | Weighted median | 10 | -1.11 | 0.81 | 0.174 | 0.331 | 0.067-1.629 |
| order Desulfovibrionales | Weighted mode | 10 | -1.33 | 0.97 | 0.203 | 0.265 | 0.04-1.766 |
| order Enterobacteriales | IVW | 7 | 0.653 | 0.67 | 0.331 | 1.921 | 0.515-7.164 |
| order Enterobacteriales | MR Egger | 7 | 2.22 | 4.01 | 0.604 | 9.208 | 0.004-23861.452 |
| order Enterobacteriales | Weighted median | 7 | 1.03 | 0.87 | 0.237 | 2.8 | 0.509-15.412 |
| order Enterobacteriales | Weighted mode | 7 | 1.119 | 1.28 | 0.417 | 3.061 | 0.247-37.943 |
| order Erysipelotrichales | IVW | 13 | -0.2 | 0.59 | 0.739 | 0.821 | 0.257-2.621 |
| order Erysipelotrichales | MR Egger | 13 | -6.11 | 2.43 | 0.028 | 0.002 | 0-0.256 |
| order Erysipelotrichales | Weighted median | 13 | -0.26 | 0.82 | 0.746 | 0.768 | 0.155-3.807 |
| order Erysipelotrichales | Weighted mode | 13 | -1.12 | 1.37 | 0.432 | 0.328 | 0.022-4.829 |
| order Gastranaerophilales | IVW | 9 | -0.13 | 0.52 | 0.808 | 0.881 | 0.318-2.446 |
| order Gastranaerophilales | MR Egger | 9 | 1.739 | 1.49 | 0.28 | 5.69 | 0.31-104.478 |
| order Gastranaerophilales | Weighted median | 9 | -0.39 | 0.58 | 0.506 | 0.681 | 0.219-2.117 |
| order Gastranaerophilales | Weighted mode | 9 | -0.68 | 1.11 | 0.558 | 0.509 | 0.058-4.433 |
| order Lactobacillales | IVW | 15 | -0.7 | 0.48 | 0.147 | 0.497 | 0.194-1.278 |
| order Lactobacillales | MR Egger | 15 | -2.01 | 1.24 | 0.129 | 0.134 | 0.012-1.527 |
| order Lactobacillales | Weighted median | 15 | -0.83 | 0.65 | 0.199 | 0.434 | 0.122-1.55 |
| order Lactobacillales | Weighted mode | 15 | -0.55 | 1.06 | 0.61 | 0.575 | 0.072-4.596 |
| order Methanobacteriales | IVW | 9 | -0.15 | 0.37 | 0.697 | 0.865 | 0.417-1.794 |
| order Methanobacteriales | MR Egger | 9 | -0.93 | 1.54 | 0.563 | 0.394 | 0.019-7.981 |
| order Methanobacteriales | Weighted median | 9 | -0.25 | 0.45 | 0.577 | 0.778 | 0.321-1.883 |
| order Methanobacteriales | Weighted mode | 9 | -0.93 | 0.79 | 0.27 | 0.394 | 0.084-1.841 |
| order Mollicutes RF9 | IVW | 12 | -0.09 | 0.42 | 0.838 | 0.917 | 0.4-2.105 |
| order Mollicutes RF9 | MR Egger | 12 | 1.376 | 1.3 | 0.313 | 3.96 | 0.312-50.194 |
| order Mollicutes RF9 | Weighted median | 12 | 0.516 | 0.61 | 0.397 | 1.676 | 0.507-5.534 |
| order Mollicutes RF9 | Weighted mode | 12 | 0.657 | 0.84 | 0.45 | 1.928 | 0.373-9.962 |
| order NB1n | IVW | 12 | 0.249 | 0.3 | 0.403 | 1.283 | 0.716-2.302 |
| order NB1n | MR Egger | 12 | -0.3 | 1.21 | 0.81 | 0.742 | 0.069-7.942 |
| order NB1n | Weighted median | 12 | 0.216 | 0.41 | 0.597 | 1.241 | 0.558-2.758 |
| order NB1n | Weighted mode | 12 | 0.833 | 0.73 | 0.277 | 2.301 | 0.552-9.599 |
| order Pasteurellales | IVW | 13 | -0.14 | 0.34 | 0.687 | 0.872 | 0.447-1.699 |
| order Pasteurellales | MR Egger | 13 | 0.203 | 0.73 | 0.787 | 1.226 | 0.291-5.169 |
| order Pasteurellales | Weighted median | 13 | 0.096 | 0.51 | 0.852 | 1.1 | 0.403-3.003 |
| order Pasteurellales | Weighted mode | 13 | 0.272 | 0.98 | 0.787 | 1.313 | 0.191-9.036 |
| order Rhodospirillales | IVW | 14 | -0.21 | 0.39 | 0.596 | 0.811 | 0.375-1.758 |
| order Rhodospirillales | MR Egger | 14 | 0.147 | 1.66 | 0.931 | 1.158 | 0.045-30.01 |
| order Rhodospirillales | Weighted median | 14 | -0.45 | 0.49 | 0.353 | 0.635 | 0.243-1.657 |
| order Rhodospirillales | Weighted mode | 14 | -0.37 | 0.71 | 0.611 | 0.693 | 0.174-2.762 |
| order Selenomonadales | IVW | 12 | -0.91 | 0.63 | 0.153 | 0.404 | 0.117-1.4 |
| order Selenomonadales | MR Egger | 12 | -3.52 | 2.13 | 0.129 | 0.029 | 0-1.907 |
| order Selenomonadales | Weighted median | 12 | -1.01 | 0.82 | 0.215 | 0.363 | 0.073-1.801 |
| order Selenomonadales | Weighted mode | 12 | -0.46 | 1.26 | 0.721 | 0.63 | 0.054-7.414 |
| order Verrucomicrobiales | IVW | 11 | -0.25 | 0.47 | 0.593 | 0.779 | 0.312-1.945 |
| order Verrucomicrobiales | MR Egger | 11 | -1.64 | 1.57 | 0.324 | 0.195 | 0.009-4.213 |
| order Verrucomicrobiales | Weighted median | 11 | -0.92 | 0.61 | 0.134 | 0.4 | 0.12-1.328 |
| order Verrucomicrobiales | Weighted mode | 11 | -0.88 | 0.88 | 0.34 | 0.415 | 0.074-2.322 |
| order Victivallales | IVW | 8 | 0.28 | 0.36 | 0.431 | 1.323 | 0.659-2.657 |
| order Victivallales | MR Egger | 8 | 0.077 | 1.26 | 0.953 | 1.08 | 0.091-12.814 |
| order Victivallales | Weighted median | 8 | 0.475 | 0.44 | 0.282 | 1.608 | 0.677-3.822 |
| order Victivallales | Weighted mode | 8 | 0.537 | 0.66 | 0.443 | 1.711 | 0.469-6.244 |
| phylum Actinobacteria | IVW | 14 | 0.243 | 0.51 | 0.63 | 1.275 | 0.473-3.436 |
| phylum Actinobacteria | MR Egger | 14 | -0.25 | 2.1 | 0.907 | 0.779 | 0.013-47.44 |
| phylum Actinobacteria | Weighted median | 14 | 0.523 | 0.67 | 0.438 | 1.687 | 0.45-6.315 |
| phylum Actinobacteria | Weighted mode | 14 | 0.525 | 0.97 | 0.599 | 1.69 | 0.25-11.411 |
| phylum Bacteroidetes | IVW | 10 | 0.057 | 0.63 | 0.928 | 1.059 | 0.308-3.641 |
| phylum Bacteroidetes | MR Egger | 10 | -2.77 | 1.33 | 0.07 | 0.062 | 0.005-0.845 |
| phylum Bacteroidetes | Weighted median | 10 | -0.04 | 0.85 | 0.965 | 0.964 | 0.184-5.059 |
| phylum Bacteroidetes | Weighted mode | 10 | -0.71 | 1.37 | 0.619 | 0.493 | 0.033-7.278 |
| phylum Cyanobacteria | IVW | 8 | 0.309 | 0.62 | 0.618 | 1.362 | 0.404-4.588 |
| phylum Cyanobacteria | MR Egger | 8 | 1.892 | 2.29 | 0.44 | 6.633 | 0.075-588.182 |
| phylum Cyanobacteria | Weighted median | 8 | 0.52 | 0.62 | 0.399 | 1.682 | 0.503-5.627 |
| phylum Cyanobacteria | Weighted mode | 8 | 0.787 | 0.95 | 0.435 | 2.197 | 0.341-14.154 |
| phylum Euryarchaeota | IVW | 11 | -0.16 | 0.27 | 0.57 | 0.856 | 0.502-1.461 |
| phylum Euryarchaeota | MR Egger | 11 | 1.099 | 1.21 | 0.387 | 3.002 | 0.281-32.049 |
| phylum Euryarchaeota | Weighted median | 11 | 0.018 | 0.37 | 0.961 | 1.018 | 0.497-2.084 |
| phylum Euryarchaeota | Weighted mode | 11 | 0.271 | 0.7 | 0.706 | 1.312 | 0.333-5.16 |
| phylum Firmicutes | IVW | 15 | 0.011 | 0.46 | 0.98 | 1.011 | 0.413-2.479 |
| phylum Firmicutes | MR Egger | 15 | -0.9 | 1.07 | 0.414 | 0.406 | 0.05-3.3 |
| phylum Firmicutes | Weighted median | 15 | 0.165 | 0.65 | 0.798 | 1.18 | 0.331-4.2 |
| phylum Firmicutes | Weighted mode | 15 | 0.575 | 0.9 | 0.533 | 1.778 | 0.305-10.35 |
| phylum Lentisphaerae | IVW | 9 | 0.143 | 0.33 | 0.667 | 1.154 | 0.601-2.217 |
| phylum Lentisphaerae | MR Egger | 9 | -0.16 | 1.27 | 0.905 | 0.855 | 0.072-10.216 |
| phylum Lentisphaerae | Weighted median | 9 | 0.468 | 0.43 | 0.282 | 1.596 | 0.681-3.74 |
| phylum Lentisphaerae | Weighted mode | 9 | 0.568 | 0.72 | 0.451 | 1.765 | 0.433-7.191 |
| phylum Proteobacteria | IVW | 12 | 0.55 | 0.53 | 0.303 | 1.734 | 0.609-4.935 |
| phylum Proteobacteria | MR Egger | 12 | -0.43 | 1.49 | 0.778 | 0.65 | 0.035-11.991 |
| phylum Proteobacteria | Weighted median | 12 | 0.687 | 0.7 | 0.325 | 1.988 | 0.506-7.807 |
| phylum Proteobacteria | Weighted mode | 12 | 1.082 | 1.05 | 0.326 | 2.95 | 0.376-23.16 |
| phylum Tenericutes | IVW | 12 | 1.104 | 0.46 | 0.016 | 3.016 | 1.228-7.411 |
| phylum Tenericutes | MR Egger | 12 | 1.941 | 1.62 | 0.258 | 6.963 | 0.292-166.094 |
| phylum Tenericutes | Weighted median | 12 | 1.204 | 0.63 | 0.056 | 3.332 | 0.972-11.426 |
| phylum Tenericutes | Weighted mode | 12 | 1.054 | 0.9 | 0.266 | 2.869 | 0.492-16.73 |
| phylum Verrucomicrobia | IVW | 12 | -0.31 | 0.44 | 0.491 | 0.737 | 0.31-1.754 |
| phylum Verrucomicrobia | MR Egger | 12 | -1.2 | 1.16 | 0.327 | 0.302 | 0.031-2.937 |
| phylum Verrucomicrobia | Weighted median | 12 | -0.15 | 0.59 | 0.796 | 0.859 | 0.271-2.725 |
| phylum Verrucomicrobia | Weighted mode | 12 | -0.51 | 0.78 | 0.524 | 0.6 | 0.131-2.748 |
| ***Myasthenia gravis*** |  |  |  |  |  |  |  |
| class Actinobacteria | IVW | 14 | -0.43 | 0.58 | 0.463 | 0.653 | 0.209-2.037 |
| class Actinobacteria | MR Egger | 14 | 2.544 | 1.44 | 0.102 | 12.73 | 0.759-213.449 |
| class Actinobacteria | Weighted median | 14 | -0.02 | 0.67 | 0.979 | 0.983 | 0.264-3.664 |
| class Actinobacteria | Weighted mode | 14 | 0.869 | 0.92 | 0.362 | 2.384 | 0.393-14.475 |
| class Alphaproteobacteria | IVW | 7 | 0.493 | 0.55 | 0.366 | 1.637 | 0.562-4.77 |
| class Alphaproteobacteria | MR Egger | 7 | 3.782 | 1.85 | 0.096 | 43.92 | 1.171-1647.625 |
| class Alphaproteobacteria | Weighted median | 7 | 1.156 | 0.72 | 0.108 | 3.176 | 0.776-13 |
| class Alphaproteobacteria | Weighted mode | 7 | 1.378 | 1.17 | 0.284 | 3.967 | 0.4-39.381 |
| class Bacilli | IVW | 17 | -1.03 | 0.43 | 0.018 | 0.358 | 0.153-0.838 |
| class Bacilli | MR Egger | 17 | -0.56 | 1.22 | 0.654 | 0.573 | 0.053-6.23 |
| class Bacilli | Weighted median | 17 | -0.38 | 0.62 | 0.533 | 0.681 | 0.203-2.28 |
| class Bacilli | Weighted mode | 17 | -0.28 | 0.88 | 0.756 | 0.757 | 0.134-4.261 |
| genus Parasutterella | IVW | 12 | -0.12 | 0.55 | 0.827 | 0.887 | 0.304-2.593 |
| genus Parasutterella | MR Egger | 12 | -1.88 | 1.27 | 0.171 | 0.153 | 0.013-1.848 |
| genus Parasutterella | Weighted median | 12 | -0.82 | 0.75 | 0.275 | 0.442 | 0.102-1.912 |
| genus Parasutterella | Weighted mode | 12 | -1.03 | 1.09 | 0.363 | 0.357 | 0.043-2.998 |
| class Betaproteobacteria | IVW | 10 | 0.324 | 0.78 | 0.676 | 1.383 | 0.302-6.339 |
| class Betaproteobacteria | MR Egger | 10 | 2.979 | 2.42 | 0.254 | 19.66 | 0.17-2277.03 |
| class Betaproteobacteria | Weighted median | 10 | 0.201 | 0.8 | 0.802 | 1.223 | 0.254-5.896 |
| class Betaproteobacteria | Weighted mode | 10 | 0.204 | 1.25 | 0.874 | 1.226 | 0.105-14.332 |
| class Clostridia | IVW | 13 | 0.286 | 0.49 | 0.556 | 1.331 | 0.514-3.446 |
| class Clostridia | MR Egger | 13 | -1.39 | 1.32 | 0.314 | 0.248 | 0.019-3.295 |
| class Clostridia | Weighted median | 13 | -0.02 | 0.68 | 0.98 | 0.983 | 0.262-3.692 |
| class Clostridia | Weighted mode | 13 | 0.882 | 1.14 | 0.456 | 2.416 | 0.256-22.754 |
| class Coriobacteriia | IVW | 13 | -0.15 | 0.51 | 0.772 | 0.862 | 0.316-2.354 |
| class Coriobacteriia | MR Egger | 13 | -1.51 | 2.17 | 0.502 | 0.221 | 0.003-15.683 |
| class Coriobacteriia | Weighted median | 13 | -0.83 | 0.67 | 0.216 | 0.436 | 0.117-1.622 |
| class Coriobacteriia | Weighted mode | 13 | -0.98 | 1.03 | 0.356 | 0.374 | 0.05-2.786 |
| class Deltaproteobacteria | IVW | 11 | 0.522 | 0.53 | 0.328 | 1.685 | 0.592-4.795 |
| class Deltaproteobacteria | MR Egger | 11 | 2.826 | 2.77 | 0.334 | 16.88 | 0.074-3848.502 |
| class Deltaproteobacteria | Weighted median | 11 | 0.422 | 0.71 | 0.551 | 1.526 | 0.38-6.12 |
| class Deltaproteobacteria | Weighted mode | 11 | 0.796 | 1.18 | 0.516 | 2.216 | 0.219-22.442 |
| class Erysipelotrichia | IVW | 13 | -0.23 | 0.54 | 0.667 | 0.794 | 0.278-2.268 |
| class Erysipelotrichia | MR Egger | 13 | 1.474 | 2.32 | 0.539 | 4.366 | 0.046-413.985 |
| class Erysipelotrichia | Weighted median | 13 | -0.26 | 0.73 | 0.723 | 0.772 | 0.184-3.235 |
| class Erysipelotrichia | Weighted mode | 13 | -0.14 | 1.13 | 0.906 | 0.872 | 0.096-7.965 |
| class Gammaproteobacteria | IVW | 6 | -0.03 | 0.73 | 0.965 | 0.969 | 0.232-4.052 |
| class Gammaproteobacteria | MR Egger | 6 | 0.066 | 2.21 | 0.978 | 1.068 | 0.014-80.577 |
| class Gammaproteobacteria | Weighted median | 6 | -0.38 | 0.92 | 0.677 | 0.682 | 0.113-4.131 |
| class Gammaproteobacteria | Weighted mode | 6 | -0.5 | 1.16 | 0.685 | 0.609 | 0.063-5.855 |
| class Lentisphaeria | IVW | 7 | 0.123 | 0.43 | 0.774 | 1.131 | 0.488-2.623 |
| class Lentisphaeria | MR Egger | 7 | 1.058 | 1.53 | 0.519 | 2.88 | 0.144-57.397 |
| class Lentisphaeria | Weighted median | 7 | 0.253 | 0.53 | 0.63 | 1.288 | 0.46-3.605 |
| class Lentisphaeria | Weighted mode | 7 | 0.148 | 0.62 | 0.817 | 1.16 | 0.347-3.874 |
| class Melainabacteria | IVW | 9 | 0.478 | 0.41 | 0.244 | 1.613 | 0.722-3.602 |
| class Melainabacteria | MR Egger | 9 | 0.088 | 1.28 | 0.947 | 1.092 | 0.09-13.33 |
| class Melainabacteria | Weighted median | 9 | 0.689 | 0.5 | 0.172 | 1.991 | 0.742-5.345 |
| class Melainabacteria | Weighted mode | 9 | 1.027 | 0.87 | 0.271 | 2.793 | 0.509-15.343 |
| class Methanobacteria | IVW | 9 | -0.04 | 0.3 | 0.896 | 0.962 | 0.539-1.717 |
| class Methanobacteria | MR Egger | 9 | 0.601 | 1.16 | 0.622 | 1.823 | 0.186-17.854 |
| class Methanobacteria | Weighted median | 9 | -0.07 | 0.39 | 0.864 | 0.936 | 0.439-1.996 |
| class Methanobacteria | Weighted mode | 9 | -0.28 | 0.54 | 0.617 | 0.755 | 0.262-2.174 |
| class Mollicutes | IVW | 12 | -0.31 | 0.42 | 0.465 | 0.735 | 0.321-1.68 |
| class Mollicutes | MR Egger | 12 | -0.63 | 1.44 | 0.672 | 0.534 | 0.032-8.965 |
| class Mollicutes | Weighted median | 12 | -0.15 | 0.55 | 0.784 | 0.859 | 0.291-2.538 |
| class Mollicutes | Weighted mode | 12 | 0.268 | 0.85 | 0.757 | 1.308 | 0.249-6.86 |
| class Negativicutes | IVW | 12 | 0.502 | 0.53 | 0.347 | 1.652 | 0.58-4.702 |
| class Negativicutes | MR Egger | 12 | -1.31 | 1.83 | 0.492 | 0.271 | 0.007-9.856 |
| class Negativicutes | Weighted median | 12 | 0.255 | 0.71 | 0.72 | 1.291 | 0.32-5.214 |
| class Negativicutes | Weighted mode | 12 | -0.43 | 1.13 | 0.712 | 0.651 | 0.071-5.993 |
| class Verrucomicrobiae | IVW | 11 | -0.41 | 0.45 | 0.358 | 0.664 | 0.278-1.59 |
| class Verrucomicrobiae | MR Egger | 11 | -1.62 | 1.49 | 0.305 | 0.197 | 0.011-3.67 |
| class Verrucomicrobiae | Weighted median | 11 | -0.36 | 0.6 | 0.552 | 0.7 | 0.216-2.268 |
| class Verrucomicrobiae | Weighted mode | 11 | -1.32 | 1.06 | 0.24 | 0.267 | 0.034-2.119 |
| family Acidaminococcaceae | IVW | 7 | -0.18 | 0.52 | 0.727 | 0.835 | 0.304-2.296 |
| family Acidaminococcaceae | MR Egger | 7 | -0.79 | 1.56 | 0.634 | 0.453 | 0.021-9.697 |
| family Acidaminococcaceae | Weighted median | 7 | -0.33 | 0.64 | 0.608 | 0.719 | 0.204-2.533 |
| family Acidaminococcaceae | Weighted mode | 7 | -0.41 | 0.82 | 0.635 | 0.665 | 0.135-3.291 |
| family Actinomycetaceae | IVW | 4 | -0.43 | 0.72 | 0.548 | 0.649 | 0.158-2.658 |
| family Actinomycetaceae | MR Egger | 4 | -1.56 | 1.7 | 0.457 | 0.211 | 0.007-5.936 |
| family Actinomycetaceae | Weighted median | 4 | -0.68 | 0.74 | 0.356 | 0.505 | 0.118-2.158 |
| family Actinomycetaceae | Weighted mode | 4 | -0.99 | 0.69 | 0.248 | 0.371 | 0.095-1.443 |
| family Alcaligenaceae | IVW | 9 | 0.32 | 0.76 | 0.672 | 1.377 | 0.312-6.07 |
| family Alcaligenaceae | MR Egger | 9 | -0.17 | 5.72 | 0.978 | 0.848 | 0-62908.858 |
| family Alcaligenaceae | Weighted median | 9 | 0.494 | 0.87 | 0.572 | 1.638 | 0.296-9.065 |
| family Alcaligenaceae | Weighted mode | 9 | 0.948 | 1.39 | 0.515 | 2.58 | 0.169-39.326 |
| family Bacteroidaceae | IVW | 5 | -0.15 | 0.81 | 0.853 | 0.86 | 0.176-4.21 |
| family Bacteroidaceae | MR Egger | 5 | -2.89 | 3.58 | 0.479 | 0.056 | 0-62.593 |
| family Bacteroidaceae | Weighted median | 5 | -0.38 | 1.01 | 0.705 | 0.683 | 0.095-4.925 |
| family Bacteroidaceae | Weighted mode | 5 | -0.31 | 1.31 | 0.824 | 0.733 | 0.057-9.485 |
| family Bacteroidales | IVW | 7 | 0.384 | 0.45 | 0.388 | 1.468 | 0.614-3.512 |
| family Bacteroidales | MR Egger | 7 | 1.411 | 1.69 | 0.441 | 4.1 | 0.15-112.005 |
| family Bacteroidales | Weighted median | 7 | 0.323 | 0.55 | 0.555 | 1.382 | 0.472-4.045 |
| family Bacteroidales | Weighted mode | 7 | 0.381 | 0.75 | 0.631 | 1.463 | 0.335-6.387 |
| family Bifidobacteriaceae | IVW | 9 | -0.37 | 0.51 | 0.471 | 0.691 | 0.253-1.888 |
| family Bifidobacteriaceae | MR Egger | 9 | 2.526 | 1.61 | 0.162 | 12.5 | 0.528-295.84 |
| family Bifidobacteriaceae | Weighted median | 9 | -0.78 | 0.72 | 0.277 | 0.458 | 0.112-1.873 |
| family Bifidobacteriaceae | Weighted mode | 9 | 1.157 | 0.75 | 0.16 | 3.181 | 0.736-13.759 |
| family Clostridiaceae1 | IVW | 10 | -0.75 | 0.67 | 0.268 | 0.474 | 0.127-1.777 |
| family Clostridiaceae1 | MR Egger | 10 | -0.87 | 2.09 | 0.689 | 0.42 | 0.007-25.178 |
| family Clostridiaceae1 | Weighted median | 10 | -1.2 | 0.74 | 0.108 | 0.303 | 0.07-1.301 |
| family Clostridiaceae1 | Weighted mode | 10 | -1.4 | 1.11 | 0.241 | 0.247 | 0.028-2.188 |
| family Clostridiales vadin BB60 group | IVW | 14 | -0.07 | 0.48 | 0.88 | 0.93 | 0.366-2.366 |
| family Clostridiales vadin BB60 group | MR Egger | 14 | 0.121 | 1.32 | 0.929 | 1.128 | 0.085-14.892 |
| family Clostridiales vadin BB60 group | Weighted median | 14 | 0.364 | 0.54 | 0.499 | 1.44 | 0.501-4.137 |
| family Clostridiales vadin BB60 group | Weighted mode | 14 | 0.666 | 0.71 | 0.363 | 1.947 | 0.487-7.779 |
| family Coriobacteriaceae | IVW | 13 | -0.15 | 0.51 | 0.772 | 0.862 | 0.316-2.354 |
| family Coriobacteriaceae | MR Egger | 13 | -1.51 | 2.17 | 0.502 | 0.221 | 0.003-15.683 |
| family Coriobacteriaceae | Weighted median | 13 | -0.83 | 0.63 | 0.189 | 0.436 | 0.126-1.507 |
| family Coriobacteriaceae | Weighted mode | 13 | -0.98 | 0.99 | 0.34 | 0.374 | 0.054-2.6 |
| family Defluviitaleaceae | IVW | 11 | -0.43 | 0.39 | 0.274 | 0.65 | 0.3-1.407 |
| family Defluviitaleaceae | MR Egger | 11 | 0.273 | 1.35 | 0.844 | 1.314 | 0.094-18.369 |
| family Defluviitaleaceae | Weighted median | 11 | -0.63 | 0.51 | 0.215 | 0.533 | 0.197-1.442 |
| family Defluviitaleaceae | Weighted mode | 11 | -0.71 | 0.86 | 0.425 | 0.49 | 0.091-2.637 |
| family Desulfovibrionaceae | IVW | 9 | 0.287 | 0.59 | 0.625 | 1.332 | 0.422-4.204 |
| family Desulfovibrionaceae | MR Egger | 9 | 3.241 | 2.89 | 0.299 | 25.56 | 0.089-7358.155 |
| family Desulfovibrionaceae | Weighted median | 9 | 0.037 | 0.74 | 0.96 | 1.038 | 0.241-4.462 |
| family Desulfovibrionaceae | Weighted mode | 9 | 0.136 | 1.36 | 0.923 | 1.146 | 0.08-16.443 |
| family Enterobacteriaceae | IVW | 6 | -2.2 | 0.74 | 0.003 | 0.111 | 0.026-0.477 |
| family Enterobacteriaceae | MR Egger | 6 | -8.81 | 4.01 | 0.093 | 0 | 0-0.387 |
| family Enterobacteriaceae | Weighted median | 6 | -1.9 | 0.99 | 0.055 | 0.149 | 0.021-1.045 |
| family Enterobacteriaceae | Weighted mode | 6 | -3.39 | 1.47 | 0.07 | 0.034 | 0.002-0.605 |
| family Erysipelotrichaceae | IVW | 13 | -0.24 | 0.53 | 0.657 | 0.789 | 0.277-2.244 |
| family Erysipelotrichaceae | MR Egger | 13 | 1.487 | 2.31 | 0.533 | 4.425 | 0.048-409.303 |
| family Erysipelotrichaceae | Weighted median | 13 | -0.24 | 0.72 | 0.737 | 0.785 | 0.192-3.215 |
| family Erysipelotrichaceae | Weighted mode | 13 | -0.09 | 1.18 | 0.939 | 0.911 | 0.091-9.177 |
| family Family XI | IVW | 8 | 0.18 | 0.33 | 0.59 | 1.197 | 0.622-2.3 |
| family Family XI | MR Egger | 8 | -2.33 | 2.05 | 0.299 | 0.098 | 0.002-5.384 |
| family Family XI | Weighted median | 8 | 0.165 | 0.39 | 0.669 | 1.18 | 0.554-2.514 |
| family Family XI | Weighted mode | 8 | 0.412 | 0.67 | 0.557 | 1.509 | 0.408-5.588 |
| family Family XIII | IVW | 7 | -0.63 | 0.91 | 0.493 | 0.534 | 0.089-3.207 |
| family Family XIII | MR Egger | 7 | 0.999 | 3.66 | 0.796 | 2.716 | 0.002-3554.757 |
| family Family XIII | Weighted median | 7 | -0.58 | 1.02 | 0.571 | 0.559 | 0.075-4.166 |
| family Family XIII | Weighted mode | 7 | -1.04 | 1.51 | 0.516 | 0.352 | 0.018-6.8 |
| family Lachnospiraceae | IVW | 14 | -0.67 | 0.55 | 0.225 | 0.511 | 0.173-1.512 |
| family Lachnospiraceae | MR Egger | 14 | -4.61 | 2.19 | 0.057 | 0.01 | 0-0.721 |
| family Lachnospiraceae | Weighted median | 14 | -0.16 | 0.74 | 0.829 | 0.852 | 0.198-3.655 |
| family Lachnospiraceae | Weighted mode | 14 | 0.099 | 1.22 | 0.936 | 1.105 | 0.102-12.014 |
| family Lactobacillaceae | IVW | 6 | 0.197 | 0.45 | 0.658 | 1.218 | 0.509-2.914 |
| family Lactobacillaceae | MR Egger | 6 | 0.032 | 1.12 | 0.979 | 1.032 | 0.114-9.319 |
| family Lactobacillaceae | Weighted median | 6 | 0.272 | 0.56 | 0.63 | 1.312 | 0.434-3.966 |
| family Lactobacillaceae | Weighted mode | 6 | 0.303 | 0.66 | 0.664 | 1.354 | 0.374-4.906 |
| family Methanobacteriaceae | IVW | 9 | -0.04 | 0.3 | 0.896 | 0.962 | 0.539-1.717 |
| family Methanobacteriaceae | MR Egger | 9 | 0.601 | 1.16 | 0.622 | 1.823 | 0.186-17.854 |
| family Methanobacteriaceae | Weighted median | 9 | -0.07 | 0.36 | 0.854 | 0.936 | 0.464-1.889 |
| family Methanobacteriaceae | Weighted mode | 9 | -0.28 | 0.59 | 0.648 | 0.755 | 0.237-2.409 |
| family Oxalobacteraceae | IVW | 13 | 0.212 | 0.27 | 0.426 | 1.236 | 0.733-2.083 |
| family Oxalobacteraceae | MR Egger | 13 | 0.298 | 1.02 | 0.776 | 1.347 | 0.182-9.963 |
| family Oxalobacteraceae | Weighted median | 13 | -0.07 | 0.35 | 0.85 | 0.936 | 0.472-1.857 |
| family Oxalobacteraceae | Weighted mode | 13 | -0.05 | 0.59 | 0.94 | 0.956 | 0.302-3.029 |
| family Pasteurellaceae | IVW | 13 | -0.04 | 0.45 | 0.935 | 0.964 | 0.402-2.314 |
| family Pasteurellaceae | MR Egger | 13 | 1.546 | 0.84 | 0.094 | 4.691 | 0.898-24.498 |
| family Pasteurellaceae | Weighted median | 13 | 0.728 | 0.46 | 0.109 | 2.071 | 0.849-5.053 |
| family Pasteurellaceae | Weighted mode | 13 | 0.774 | 0.56 | 0.189 | 2.169 | 0.73-6.442 |
| family Peptococcaceae | IVW | 8 | 0.446 | 0.55 | 0.421 | 1.562 | 0.527-4.63 |
| family Peptococcaceae | MR Egger | 8 | -1.14 | 1.42 | 0.452 | 0.32 | 0.02-5.154 |
| family Peptococcaceae | Weighted median | 8 | 1.149 | 0.72 | 0.109 | 3.154 | 0.773-12.861 |
| family Peptococcaceae | Weighted mode | 8 | 1.577 | 1.38 | 0.29 | 4.839 | 0.325-71.985 |
| family Peptostreptococcaceae | IVW | 13 | 0.164 | 0.42 | 0.694 | 1.179 | 0.519-2.675 |
| family Peptostreptococcaceae | MR Egger | 13 | -0.4 | 0.95 | 0.679 | 0.668 | 0.104-4.29 |
| family Peptostreptococcaceae | Weighted median | 13 | 0.043 | 0.58 | 0.94 | 1.044 | 0.337-3.235 |
| family Peptostreptococcaceae | Weighted mode | 13 | -0.26 | 0.76 | 0.736 | 0.77 | 0.174-3.408 |
| family Porphyromonadaceae | IVW | 7 | -1.08 | 0.76 | 0.155 | 0.341 | 0.077-1.502 |
| family Porphyromonadaceae | MR Egger | 7 | 0.637 | 3.08 | 0.844 | 1.891 | 0.005-791.645 |
| family Porphyromonadaceae | Weighted median | 7 | -0.79 | 0.98 | 0.421 | 0.456 | 0.067-3.083 |
| family Porphyromonadaceae | Weighted mode | 7 | -0.51 | 1.41 | 0.728 | 0.598 | 0.038-9.491 |
| family Prevotellaceae | IVW | 15 | 0.55 | 0.43 | 0.198 | 1.734 | 0.751-4.006 |
| family Prevotellaceae | MR Egger | 15 | -1.45 | 1.51 | 0.353 | 0.234 | 0.012-4.496 |
| family Prevotellaceae | Weighted median | 15 | 0.263 | 0.58 | 0.651 | 1.3 | 0.417-4.051 |
| family Prevotellaceae | Weighted mode | 15 | 0.356 | 1.02 | 0.733 | 1.427 | 0.192-10.616 |
| family Rhodospirillaceae | IVW | 12 | -0.04 | 0.35 | 0.92 | 0.966 | 0.486-1.917 |
| family Rhodospirillaceae | MR Egger | 12 | 1.188 | 1.44 | 0.427 | 3.28 | 0.197-54.587 |
| family Rhodospirillaceae | Weighted median | 12 | -0.19 | 0.48 | 0.688 | 0.825 | 0.323-2.109 |
| family Rhodospirillaceae | Weighted mode | 12 | -0.32 | 0.71 | 0.66 | 0.727 | 0.182-2.898 |
| family Rikenellaceae | IVW | 16 | 0.688 | 0.49 | 0.161 | 1.99 | 0.76-5.207 |
| family Rikenellaceae | MR Egger | 16 | 0.216 | 1.55 | 0.891 | 1.242 | 0.06-25.891 |
| family Rikenellaceae | Weighted median | 16 | 0.437 | 0.64 | 0.492 | 1.548 | 0.446-5.376 |
| family Rikenellaceae | Weighted mode | 16 | 0.027 | 0.96 | 0.978 | 1.027 | 0.155-6.794 |
| family Ruminococcaceae | IVW | 9 | -0.49 | 0.62 | 0.431 | 0.614 | 0.182-2.071 |
| family Ruminococcaceae | MR Egger | 9 | 0.807 | 1.39 | 0.58 | 2.242 | 0.146-34.388 |
| family Ruminococcaceae | Weighted median | 9 | 0.267 | 0.74 | 0.718 | 1.307 | 0.306-5.586 |
| family Ruminococcaceae | Weighted mode | 9 | 0.405 | 0.84 | 0.643 | 1.499 | 0.288-7.795 |
| family Streptococcaceae | IVW | 11 | -0.66 | 0.55 | 0.229 | 0.519 | 0.178-1.511 |
| family Streptococcaceae | MR Egger | 11 | 2.236 | 2.24 | 0.344 | 9.354 | 0.116-755.638 |
| family Streptococcaceae | Weighted median | 11 | -0.74 | 0.72 | 0.307 | 0.479 | 0.117-1.967 |
| family Streptococcaceae | Weighted mode | 11 | -0.79 | 1.09 | 0.485 | 0.454 | 0.054-3.833 |
| family Veillonellaceae | IVW | 19 | 0.079 | 0.39 | 0.84 | 1.082 | 0.502-2.335 |
| family Veillonellaceae | MR Egger | 19 | 0.522 | 0.81 | 0.529 | 1.686 | 0.344-8.268 |
| family Veillonellaceae | Weighted median | 19 | 0.558 | 0.54 | 0.301 | 1.747 | 0.607-5.03 |
| family Veillonellaceae | Weighted mode | 19 | 0.646 | 0.77 | 0.411 | 1.908 | 0.424-8.579 |
| family Verrucomicrobiaceae | IVW | 11 | -0.41 | 0.45 | 0.358 | 0.664 | 0.278-1.59 |
| family Verrucomicrobiaceae | MR Egger | 11 | -1.62 | 1.49 | 0.305 | 0.197 | 0.011-3.675 |
| family Verrucomicrobiaceae | Weighted median | 11 | -0.36 | 0.56 | 0.525 | 0.701 | 0.234-2.098 |
| family Verrucomicrobiaceae | Weighted mode | 11 | -1.32 | 0.97 | 0.204 | 0.267 | 0.04-1.789 |
| family Victivallaceae | IVW | 11 | 0.184 | 0.27 | 0.492 | 1.202 | 0.71-2.035 |
| family Victivallaceae | MR Egger | 11 | 3.304 | 1.25 | 0.027 | 27.22 | 2.356-314.488 |
| family Victivallaceae | Weighted median | 11 | 0.208 | 0.34 | 0.536 | 1.231 | 0.637-2.379 |
| family Victivallaceae | Weighted mode | 11 | 0.269 | 0.53 | 0.62 | 1.308 | 0.467-3.663 |
| genus Actinomyces | IVW | 7 | -0.98 | 0.46 | 0.036 | 0.377 | 0.152-0.937 |
| genus Actinomyces | MR Egger | 7 | -1.94 | 1.15 | 0.152 | 0.144 | 0.015-1.369 |
| genus Actinomyces | Weighted median | 7 | -1.29 | 0.56 | 0.022 | 0.276 | 0.092-0.827 |
| genus Actinomyces | Weighted mode | 7 | -1.28 | 0.72 | 0.127 | 0.279 | 0.068-1.144 |
| genus Adlercreutzia | IVW | 7 | -0.38 | 0.49 | 0.443 | 0.685 | 0.26-1.802 |
| genus Adlercreutzia | MR Egger | 7 | 2.839 | 2.1 | 0.234 | 17.1 | 0.279-1046.772 |
| genus Adlercreutzia | Weighted median | 7 | -0.16 | 0.63 | 0.798 | 0.851 | 0.248-2.92 |
| genus Adlercreutzia | Weighted mode | 7 | -0.14 | 0.92 | 0.887 | 0.873 | 0.143-5.317 |
| genus Anaerofilum | IVW | 11 | -0.48 | 0.33 | 0.148 | 0.619 | 0.323-1.185 |
| genus Anaerofilum | MR Egger | 11 | -1.44 | 1.87 | 0.462 | 0.238 | 0.006-9.325 |
| genus Anaerofilum | Weighted median | 11 | -0.45 | 0.45 | 0.317 | 0.635 | 0.261-1.544 |
| genus Anaerofilum | Weighted mode | 11 | -1.11 | 0.8 | 0.196 | 0.331 | 0.069-1.58 |
| genus Anaerostipes | IVW | 10 | 0.019 | 0.6 | 0.975 | 1.019 | 0.316-3.288 |
| genus Anaerostipes | MR Egger | 10 | -0.75 | 1.99 | 0.718 | 0.474 | 0.01-23.59 |
| genus Anaerostipes | Weighted median | 10 | -0.24 | 0.77 | 0.751 | 0.784 | 0.174-3.532 |
| genus Anaerostipes | Weighted mode | 10 | -0.87 | 1.22 | 0.497 | 0.421 | 0.038-4.618 |
| genus Butyrivibrio | IVW | 15 | -0.15 | 0.22 | 0.492 | 0.86 | 0.559-1.323 |
| genus Butyrivibrio | MR Egger | 15 | 0.485 | 0.97 | 0.624 | 1.624 | 0.244-10.805 |
| genus Butyrivibrio | Weighted median | 15 | -0.4 | 0.29 | 0.168 | 0.673 | 0.383-1.183 |
| genus Butyrivibrio | Weighted mode | 15 | -0.56 | 0.47 | 0.254 | 0.57 | 0.226-1.439 |
| genus Catenibacterium | IVW | 4 | -0.48 | 0.41 | 0.248 | 0.622 | 0.278-1.391 |
| genus Catenibacterium | MR Egger | 4 | 0.462 | 5.02 | 0.935 | 1.587 | 0-29773.608 |
| genus Catenibacterium | Weighted median | 4 | -0.49 | 0.48 | 0.315 | 0.615 | 0.238-1.588 |
| genus Catenibacterium | Weighted mode | 4 | -0.72 | 0.64 | 0.342 | 0.489 | 0.141-1.7 |
| genus Christensenellaceae R 7group | IVW | 8 | 0.586 | 0.75 | 0.437 | 1.797 | 0.41-7.873 |
| genus Christensenellaceae R 7group | MR Egger | 8 | 2.336 | 2.76 | 0.429 | 10.34 | 0.047-2295.676 |
| genus Christensenellaceae R 7group | Weighted median | 8 | -0.17 | 0.93 | 0.853 | 0.843 | 0.137-5.178 |
| genus Christensenellaceae R 7group | Weighted mode | 8 | -0.43 | 1.6 | 0.798 | 0.654 | 0.029-14.904 |
| genus Clostridium innocuum group | IVW | 7 | -0.15 | 0.33 | 0.654 | 0.864 | 0.457-1.635 |
| genus Clostridium innocuum group | MR Egger | 7 | -0.75 | 1.59 | 0.656 | 0.472 | 0.021-10.592 |
| genus Clostridium innocuum group | Weighted median | 7 | -0.24 | 0.41 | 0.566 | 0.789 | 0.352-1.771 |
| genus Clostridium innocuum group | Weighted mode | 7 | -0.31 | 0.54 | 0.593 | 0.736 | 0.254-2.134 |
| genus Collinsella | IVW | 9 | 0.653 | 0.69 | 0.344 | 1.921 | 0.496-7.435 |
| genus Collinsella | MR Egger | 9 | 1.026 | 2.76 | 0.722 | 2.789 | 0.012-628.388 |
| genus Collinsella | Weighted median | 9 | 1.154 | 0.87 | 0.182 | 3.17 | 0.582-17.259 |
| genus Collinsella | Weighted mode | 9 | 1.37 | 1.3 | 0.323 | 3.934 | 0.307-50.445 |
| genus Defluviitaleaceae UCG011 | IVW | 9 | -0.59 | 0.44 | 0.179 | 0.554 | 0.234-1.312 |
| genus Defluviitaleaceae UCG011 | MR Egger | 9 | 0.033 | 1.59 | 0.984 | 1.034 | 0.046-23.266 |
| genus Defluviitaleaceae UCG011 | Weighted median | 9 | -0.64 | 0.57 | 0.262 | 0.528 | 0.173-1.611 |
| genus Defluviitaleaceae UCG011 | Weighted mode | 9 | -0.64 | 0.87 | 0.484 | 0.529 | 0.096-2.904 |
| genus Desulfovibrio | IVW | 8 | -0.44 | 0.47 | 0.357 | 0.646 | 0.255-1.636 |
| genus Desulfovibrio | MR Egger | 8 | -0.49 | 1.43 | 0.745 | 0.615 | 0.038-10.094 |
| genus Desulfovibrio | Weighted median | 8 | -0.55 | 0.61 | 0.371 | 0.58 | 0.176-1.915 |
| genus Desulfovibrio | Weighted mode | 8 | -0.62 | 0.78 | 0.449 | 0.536 | 0.117-2.458 |
| genus Dialister | IVW | 11 | 0.438 | 0.46 | 0.345 | 1.55 | 0.624-3.85 |
| genus Dialister | MR Egger | 11 | -0.69 | 1.91 | 0.728 | 0.504 | 0.012-21.428 |
| genus Dialister | Weighted median | 11 | -0.24 | 0.65 | 0.714 | 0.788 | 0.221-2.811 |
| genus Dialister | Weighted mode | 11 | -0.75 | 1.13 | 0.524 | 0.474 | 0.052-4.336 |
| genus Eggerthella | IVW | 10 | 0.27 | 0.33 | 0.412 | 1.309 | 0.688-2.494 |
| genus Eggerthella | MR Egger | 10 | 0.184 | 1.52 | 0.907 | 1.202 | 0.061-23.619 |
| genus Eggerthella | Weighted median | 10 | 0.105 | 0.45 | 0.817 | 1.11 | 0.457-2.696 |
| genus Eggerthella | Weighted mode | 10 | -0.53 | 0.71 | 0.475 | 0.589 | 0.146-2.374 |
| genus Eisenbergiella | IVW | 11 | -0.09 | 0.4 | 0.829 | 0.917 | 0.418-2.011 |
| genus Eisenbergiella | MR Egger | 11 | -1.99 | 3.06 | 0.532 | 0.137 | 0-54.948 |
| genus Eisenbergiella | Weighted median | 11 | -0.13 | 0.45 | 0.768 | 0.877 | 0.365-2.105 |
| genus Eisenbergiella | Weighted mode | 11 | -0.42 | 0.61 | 0.509 | 0.658 | 0.199-2.178 |
| genus Enterorhabdus | IVW | 6 | 0.206 | 0.67 | 0.757 | 1.229 | 0.333-4.542 |
| genus Enterorhabdus | MR Egger | 6 | 0.041 | 1.96 | 0.984 | 1.042 | 0.022-48.611 |
| genus Enterorhabdus | Weighted median | 6 | -0.31 | 0.68 | 0.646 | 0.732 | 0.194-2.767 |
| genus Enterorhabdus | Weighted mode | 6 | -0.57 | 0.75 | 0.483 | 0.568 | 0.131-2.46 |
| genus Eubacterium fissicatena group | IVW | 8 | 0.631 | 0.33 | 0.052 | 1.88 | 0.995-3.554 |
| genus Eubacterium fissicatena group | MR Egger | 8 | -0.46 | 1.64 | 0.789 | 0.632 | 0.025-15.695 |
| genus Eubacterium fissicatena group | Weighted median | 8 | 0.795 | 0.42 | 0.06 | 2.214 | 0.966-5.076 |
| genus Eubacterium fissicatena group | Weighted mode | 8 | 0.929 | 0.65 | 0.195 | 2.533 | 0.71-9.036 |
| genus Eubacterium rectale group | IVW | 5 | 0.73 | 1 | 0.465 | 2.075 | 0.293-14.717 |
| genus Eubacterium rectale group | MR Egger | 5 | -7.72 | 3.61 | 0.122 | 0 | 0-0.521 |
| genus Eubacterium rectale group | Weighted median | 5 | 1.206 | 1.07 | 0.259 | 3.34 | 0.412-27.105 |
| genus Eubacterium rectale group | Weighted mode | 5 | 1.292 | 1.86 | 0.525 | 3.639 | 0.095-138.702 |
| genus Fusicatenibacter | IVW | 18 | 0.008 | 0.44 | 0.986 | 1.008 | 0.426-2.384 |
| genus Fusicatenibacter | MR Egger | 18 | 0.37 | 1.65 | 0.825 | 1.448 | 0.057-36.636 |
| genus Fusicatenibacter | Weighted median | 18 | 0.104 | 0.6 | 0.863 | 1.109 | 0.342-3.599 |
| genus Fusicatenibacter | Weighted mode | 18 | 0.392 | 0.94 | 0.681 | 1.48 | 0.236-9.281 |
| genus Gordonibacter | IVW | 7 | 0.262 | 0.37 | 0.475 | 1.3 | 0.633-2.667 |
| genus Gordonibacter | MR Egger | 7 | 0.434 | 1.86 | 0.825 | 1.543 | 0.04-59.104 |
| genus Gordonibacter | Weighted median | 7 | 0.463 | 0.44 | 0.296 | 1.589 | 0.666-3.787 |
| genus Gordonibacter | Weighted mode | 7 | 0.576 | 0.65 | 0.408 | 1.778 | 0.501-6.313 |
| genus Intestinimonas | IVW | 16 | -0.2 | 0.42 | 0.643 | 0.822 | 0.358-1.883 |
| genus Intestinimonas | MR Egger | 16 | 1.16 | 1.15 | 0.329 | 3.19 | 0.337-30.175 |
| genus Intestinimonas | Weighted median | 16 | -0.63 | 0.52 | 0.228 | 0.532 | 0.191-1.484 |
| genus Intestinimonas | Weighted mode | 16 | -0.65 | 0.82 | 0.445 | 0.524 | 0.104-2.632 |
| genus Lachnospiraceae FCS020 group | IVW | 12 | 0.338 | 0.45 | 0.449 | 1.403 | 0.584-3.372 |
| genus Lachnospiraceae FCS020 group | MR Egger | 12 | 0.342 | 1.18 | 0.778 | 1.408 | 0.138-14.321 |
| genus Lachnospiraceae FCS020 group | Weighted median | 12 | 0.466 | 0.58 | 0.418 | 1.594 | 0.516-4.927 |
| genus Lachnospiraceae FCS020 group | Weighted mode | 12 | 0.853 | 0.79 | 0.303 | 2.347 | 0.499-11.038 |
| genus Lachnospiraceae UCG001 | IVW | 12 | 0.466 | 0.39 | 0.235 | 1.594 | 0.739-3.438 |
| genus Lachnospiraceae UCG001 | MR Egger | 12 | -2.71 | 1.54 | 0.109 | 0.066 | 0.003-1.369 |
| genus Lachnospiraceae UCG001 | Weighted median | 12 | 0.62 | 0.53 | 0.239 | 1.859 | 0.662-5.219 |
| genus Lachnospiraceae UCG001 | Weighted mode | 12 | 0.709 | 0.89 | 0.443 | 2.033 | 0.355-11.644 |
| genus Lachnospiraceae UCG008 | IVW | 9 | 0.486 | 0.38 | 0.196 | 1.625 | 0.778-3.396 |
| genus Lachnospiraceae UCG008 | MR Egger | 9 | 3.058 | 1.99 | 0.168 | 21.28 | 0.432-1048.068 |
| genus Lachnospiraceae UCG008 | Weighted median | 9 | 0.363 | 0.52 | 0.481 | 1.438 | 0.524-3.943 |
| genus Lachnospiraceae UCG008 | Weighted mode | 9 | 0.44 | 0.81 | 0.6 | 1.553 | 0.319-7.549 |
| genus Lactobacillus | IVW | 8 | 0.114 | 0.36 | 0.75 | 1.121 | 0.556-2.258 |
| genus Lactobacillus | MR Egger | 8 | -0.14 | 0.92 | 0.883 | 0.868 | 0.143-5.265 |
| genus Lactobacillus | Weighted median | 8 | 0.099 | 0.51 | 0.846 | 1.104 | 0.405-3.009 |
| genus Lactobacillus | Weighted mode | 8 | 0.101 | 0.63 | 0.878 | 1.106 | 0.322-3.801 |
| genus Marvinbryantia | IVW | 10 | -0.57 | 0.66 | 0.392 | 0.568 | 0.156-2.071 |
| genus Marvinbryantia | MR Egger | 10 | 0.176 | 2.73 | 0.95 | 1.193 | 0.006-251.195 |
| genus Marvinbryantia | Weighted median | 10 | -0.21 | 0.74 | 0.772 | 0.808 | 0.191-3.414 |
| genus Marvinbryantia | Weighted mode | 10 | 0.52 | 1.21 | 0.678 | 1.682 | 0.156-18.089 |
| genus Parabacteroides | IVW | 5 | -1.8 | 0.77 | 0.02 | 0.166 | 0.037-0.752 |
| genus Parabacteroides | MR Egger | 5 | -3.7 | 4.8 | 0.497 | 0.025 | 0-302.019 |
| genus Parabacteroides | Weighted median | 5 | -1.71 | 0.97 | 0.078 | 0.181 | 0.027-1.208 |
| genus Parabacteroides | Weighted mode | 5 | -1.91 | 1.26 | 0.204 | 0.149 | 0.013-1.747 |
| genus Peptococcus | IVW | 9 | -0.58 | 0.35 | 0.1 | 0.559 | 0.28-1.117 |
| genus Peptococcus | MR Egger | 9 | -0.28 | 1.52 | 0.86 | 0.758 | 0.039-14.809 |
| genus Peptococcus | Weighted median | 9 | -0.9 | 0.48 | 0.059 | 0.405 | 0.159-1.036 |
| genus Peptococcus | Weighted mode | 9 | -1.15 | 0.8 | 0.188 | 0.317 | 0.066-1.516 |
| genus Romboutsia | IVW | 13 | -0.25 | 0.55 | 0.645 | 0.777 | 0.266-2.273 |
| genus Romboutsia | MR Egger | 13 | 1.874 | 1.51 | 0.242 | 6.511 | 0.335-126.536 |
| genus Romboutsia | Weighted median | 13 | 0.188 | 0.62 | 0.761 | 1.206 | 0.36-4.047 |
| genus Romboutsia | Weighted mode | 13 | -0.11 | 0.97 | 0.914 | 0.899 | 0.133-6.064 |
| genus Ruminiclostridium6 | IVW | 14 | -0.97 | 0.55 | 0.081 | 0.381 | 0.129-1.128 |
| genus Ruminiclostridium6 | MR Egger | 14 | -3 | 1.24 | 0.032 | 0.05 | 0.004-0.565 |
| genus Ruminiclostridium6 | Weighted median | 14 | -0.67 | 0.66 | 0.31 | 0.513 | 0.141-1.86 |
| genus Ruminiclostridium6 | Weighted mode | 14 | 0.32 | 1.28 | 0.806 | 1.377 | 0.112-16.884 |
| genus Ruminococcaceae NK4A214 group | IVW | 13 | 0.092 | 0.47 | 0.844 | 1.096 | 0.439-2.741 |
| genus Ruminococcaceae NK4A214 group | MR Egger | 13 | 3.027 | 1.54 | 0.076 | 20.64 | 1.003-424.766 |
| genus Ruminococcaceae NK4A214 group | Weighted median | 13 | 0.352 | 0.64 | 0.584 | 1.421 | 0.404-4.994 |
| genus Ruminococcaceae NK4A214 group | Weighted mode | 13 | 0.86 | 1.03 | 0.422 | 2.362 | 0.312-17.911 |
| genus Ruminococcaceae UCG003 | IVW | 12 | -0.04 | 0.45 | 0.926 | 0.959 | 0.395-2.324 |
| genus Ruminococcaceae UCG003 | MR Egger | 12 | -2.67 | 1.47 | 0.1 | 0.07 | 0.004-1.238 |
| genus Ruminococcaceae UCG003 | Weighted median | 12 | -0.18 | 0.62 | 0.772 | 0.836 | 0.248-2.82 |
| genus Ruminococcaceae UCG003 | Weighted mode | 12 | -0.36 | 0.89 | 0.695 | 0.699 | 0.122-3.999 |
| genus Ruminococcaceae UCG004 | IVW | 8 | 0.461 | 0.54 | 0.397 | 1.586 | 0.546-4.607 |
| genus Ruminococcaceae UCG004 | MR Egger | 8 | 2.302 | 2.65 | 0.418 | 9.999 | 0.056-1789.881 |
| genus Ruminococcaceae UCG004 | Weighted median | 8 | 0.1 | 0.67 | 0.881 | 1.105 | 0.299-4.08 |
| genus Ruminococcaceae UCG004 | Weighted mode | 8 | -0.72 | 1.25 | 0.583 | 0.487 | 0.042-5.65 |
| genus Ruminococcaceae UCG005 | IVW | 14 | 0.896 | 0.42 | 0.034 | 2.45 | 1.072-5.598 |
| genus Ruminococcaceae UCG005 | MR Egger | 14 | 0.323 | 1.18 | 0.788 | 1.381 | 0.138-13.855 |
| genus Ruminococcaceae UCG005 | Weighted median | 14 | 0.672 | 0.57 | 0.239 | 1.958 | 0.64-5.985 |
| genus Ruminococcaceae UCG005 | Weighted mode | 14 | 0.669 | 0.8 | 0.415 | 1.953 | 0.411-9.268 |
| genus Ruminococcaceae UCG009 | IVW | 12 | -0.08 | 0.35 | 0.821 | 0.924 | 0.467-1.828 |
| genus Ruminococcaceae UCG009 | MR Egger | 12 | 1.476 | 1.37 | 0.307 | 4.377 | 0.298-64.225 |
| genus Ruminococcaceae UCG009 | Weighted median | 12 | 0.074 | 0.46 | 0.871 | 1.077 | 0.442-2.625 |
| genus Ruminococcaceae UCG009 | Weighted mode | 12 | 0.081 | 0.66 | 0.905 | 1.084 | 0.298-3.938 |
| genus Ruminococcaceae UCG010 | IVW | 6 | 0.37 | 0.82 | 0.651 | 1.448 | 0.291-7.193 |
| genus Ruminococcaceae UCG010 | MR Egger | 6 | -2.99 | 1.76 | 0.164 | 0.05 | 0.002-1.577 |
| genus Ruminococcaceae UCG010 | Weighted median | 6 | 0.726 | 0.83 | 0.38 | 2.067 | 0.408-10.465 |
| genus Ruminococcaceae UCG010 | Weighted mode | 6 | -0.75 | 1.21 | 0.562 | 0.472 | 0.044-5.038 |
| genus Ruminococcus1 | IVW | 10 | 0.496 | 0.51 | 0.333 | 1.641 | 0.602-4.473 |
| genus Ruminococcus1 | MR Egger | 10 | 0.382 | 1.37 | 0.787 | 1.465 | 0.101-21.359 |
| genus Ruminococcus1 | Weighted median | 10 | 0.773 | 0.7 | 0.269 | 2.165 | 0.551-8.514 |
| genus Ruminococcus1 | Weighted mode | 10 | 0.69 | 1.21 | 0.582 | 1.994 | 0.186-21.379 |
| genus Ruminococcus2 | IVW | 12 | -0.41 | 0.5 | 0.405 | 0.662 | 0.25-1.75 |
| genus Ruminococcus2 | MR Egger | 12 | -2.56 | 1.49 | 0.115 | 0.077 | 0.004-1.416 |
| genus Ruminococcus2 | Weighted median | 12 | -0.38 | 0.67 | 0.567 | 0.682 | 0.184-2.524 |
| genus Ruminococcus2 | Weighted mode | 12 | 0.078 | 1.22 | 0.95 | 1.081 | 0.099-11.873 |
| genus Ruminococcus gnavus group | IVW | 11 | -0.08 | 0.33 | 0.823 | 0.928 | 0.482-1.786 |
| genus Ruminococcus gnavus group | MR Egger | 11 | -2.13 | 1.6 | 0.215 | 0.119 | 0.005-2.717 |
| genus Ruminococcus gnavus group | Weighted median | 11 | 0.087 | 0.43 | 0.838 | 1.091 | 0.474-2.509 |
| genus Ruminococcus gnavus group | Weighted mode | 11 | 0.443 | 0.73 | 0.556 | 1.557 | 0.375-6.469 |
| genus Senegalimassilia | IVW | 5 | -0.3 | 0.68 | 0.657 | 0.739 | 0.194-2.806 |
| genus Senegalimassilia | MR Egger | 5 | -2.91 | 2.61 | 0.345 | 0.054 | 0-8.991 |
| genus Senegalimassilia | Weighted median | 5 | -0.47 | 0.75 | 0.528 | 0.623 | 0.143-2.709 |
| genus Senegalimassilia | Weighted mode | 5 | -0.62 | 1.02 | 0.576 | 0.538 | 0.073-3.971 |
| genus Turicibacter | IVW | 9 | -0.38 | 0.44 | 0.385 | 0.685 | 0.292-1.609 |
| genus Turicibacter | MR Egger | 9 | 4.007 | 1.76 | 0.057 | 54.99 | 1.732-1745.831 |
| genus Turicibacter | Weighted median | 9 | -0.24 | 0.6 | 0.688 | 0.786 | 0.242-2.549 |
| genus Turicibacter | Weighted mode | 9 | -0.02 | 0.9 | 0.981 | 0.978 | 0.169-5.678 |
| genus Akkermansia | IVW | 11 | -0.41 | 0.45 | 0.36 | 0.665 | 0.278-1.592 |
| genus Akkermansia | MR Egger | 11 | -1.6 | 1.49 | 0.31 | 0.201 | 0.011-3.739 |
| genus Akkermansia | Weighted median | 11 | -0.35 | 0.61 | 0.571 | 0.708 | 0.215-2.335 |
| genus Akkermansia | Weighted mode | 11 | -1.32 | 0.99 | 0.211 | 0.267 | 0.038-1.855 |
| genus Alloprevotella | IVW | 5 | 0.032 | 0.35 | 0.925 | 1.033 | 0.524-2.037 |
| genus Alloprevotella | MR Egger | 5 | 2.929 | 3.01 | 0.402 | 18.71 | 0.051-6843.368 |
| genus Alloprevotella | Weighted median | 5 | 0 | 0.48 | 0.999 | 1 | 0.39-2.563 |
| genus Alloprevotella | Weighted mode | 5 | 0.155 | 0.59 | 0.805 | 1.168 | 0.369-3.696 |
| genus Bacteroides | IVW | 7 | -0.74 | 0.67 | 0.268 | 0.476 | 0.128-1.768 |
| genus Bacteroides | MR Egger | 7 | -3.57 | 3.32 | 0.332 | 0.028 | 0-19.035 |
| genus Bacteroides | Weighted median | 7 | -0.6 | 0.86 | 0.487 | 0.55 | 0.102-2.971 |
| genus Bacteroides | Weighted mode | 7 | -0.57 | 1.27 | 0.67 | 0.567 | 0.047-6.775 |
| genus Barnesiella | IVW | 12 | -0.29 | 0.48 | 0.547 | 0.747 | 0.29-1.927 |
| genus Barnesiella | MR Egger | 12 | -0.61 | 1.97 | 0.762 | 0.541 | 0.011-25.742 |
| genus Barnesiella | Weighted median | 12 | -0.59 | 0.67 | 0.38 | 0.556 | 0.15-2.062 |
| genus Barnesiella | Weighted mode | 12 | -0.69 | 0.96 | 0.489 | 0.502 | 0.076-3.319 |
| genus Butyricicoccus | IVW | 8 | 0.122 | 0.77 | 0.874 | 1.13 | 0.251-5.091 |
| genus Butyricicoccus | MR Egger | 8 | -0.02 | 1.63 | 0.989 | 0.977 | 0.04-23.674 |
| genus Butyricicoccus | Weighted median | 8 | -0.46 | 0.8 | 0.568 | 0.632 | 0.131-3.055 |
| genus Butyricicoccus | Weighted mode | 8 | -0.41 | 1.06 | 0.713 | 0.666 | 0.084-5.293 |
| genus Butyricimonas | IVW | 13 | -0.15 | 0.43 | 0.726 | 0.861 | 0.372-1.992 |
| genus Butyricimonas | MR Egger | 13 | 0.26 | 1.52 | 0.868 | 1.296 | 0.065-25.657 |
| genus Butyricimonas | Weighted median | 13 | -0.26 | 0.58 | 0.649 | 0.768 | 0.246-2.395 |
| genus Butyricimonas | Weighted mode | 13 | -0.27 | 0.95 | 0.781 | 0.763 | 0.118-4.931 |
| genus Clostridium sensustricto1 | IVW | 7 | -0.83 | 0.78 | 0.285 | 0.436 | 0.095-1.997 |
| genus Clostridium sensustricto1 | MR Egger | 7 | -0.58 | 2.18 | 0.801 | 0.56 | 0.008-40.177 |
| genus Clostridium sensustricto1 | Weighted median | 7 | -0.81 | 0.78 | 0.298 | 0.443 | 0.096-2.049 |
| genus Clostridium sensustricto1 | Weighted mode | 7 | -1.37 | 1.28 | 0.323 | 0.254 | 0.021-3.089 |
| genus Coprococcus1 | IVW | 9 | -0.55 | 0.78 | 0.483 | 0.579 | 0.126-2.667 |
| genus Coprococcus1 | MR Egger | 9 | 2.167 | 2.6 | 0.432 | 8.732 | 0.053-1426.697 |
| genus Coprococcus1 | Weighted median | 9 | -1.02 | 0.87 | 0.24 | 0.361 | 0.066-1.975 |
| genus Coprococcus1 | Weighted mode | 9 | -1.73 | 1.49 | 0.28 | 0.178 | 0.01-3.296 |
| genus Coprococcus2 | IVW | 8 | 0.752 | 0.54 | 0.165 | 2.122 | 0.733-6.141 |
| genus Coprococcus2 | MR Egger | 8 | -5.63 | 4.29 | 0.237 | 0.004 | 0-15.928 |
| genus Coprococcus2 | Weighted median | 8 | 1.113 | 0.71 | 0.116 | 3.044 | 0.76-12.201 |
| genus Coprococcus2 | Weighted mode | 8 | 1.425 | 1.07 | 0.224 | 4.156 | 0.511-33.791 |
| genus Eubacterium hallii group | IVW | 15 | -0.85 | 0.45 | 0.057 | 0.426 | 0.177-1.025 |
| genus Eubacterium hallii group | MR Egger | 15 | 0.295 | 0.9 | 0.749 | 1.343 | 0.228-7.905 |
| genus Eubacterium hallii group | Weighted median | 15 | -0.81 | 0.59 | 0.175 | 0.447 | 0.14-1.431 |
| genus Eubacterium hallii group | Weighted mode | 15 | -0.66 | 0.8 | 0.424 | 0.517 | 0.108-2.485 |
| genus Eubacterium nodatum group | IVW | 11 | -0.04 | 0.25 | 0.881 | 0.963 | 0.591-1.57 |
| genus Eubacterium nodatum group | MR Egger | 11 | -0.01 | 1.11 | 0.993 | 0.99 | 0.112-8.711 |
| genus Eubacterium nodatum group | Weighted median | 11 | -0.08 | 0.31 | 0.801 | 0.924 | 0.499-1.711 |
| genus Eubacterium nodatum group | Weighted mode | 11 | -0.11 | 0.46 | 0.81 | 0.893 | 0.364-2.191 |
| genus Eubacterium oxidoreducens group | IVW | 5 | -0.21 | 0.78 | 0.792 | 0.815 | 0.177-3.744 |
| genus Eubacterium oxidoreducens group | MR Egger | 5 | 2.631 | 2.88 | 0.428 | 13.89 | 0.049-3921.697 |
| genus Eubacterium oxidoreducens group | Weighted median | 5 | 0.752 | 0.69 | 0.278 | 2.122 | 0.545-8.266 |
| genus Eubacterium oxidoreducens group | Weighted mode | 5 | 1.002 | 0.85 | 0.302 | 2.724 | 0.519-14.312 |
| genus Eubacterium ventriosum group | IVW | 15 | -0.02 | 0.48 | 0.963 | 0.978 | 0.385-2.482 |
| genus Eubacterium ventriosum group | MR Egger | 15 | 2.88 | 2.04 | 0.181 | 17.81 | 0.329-965.683 |
| genus Eubacterium ventriosum group | Weighted median | 15 | 0.162 | 0.64 | 0.798 | 1.176 | 0.338-4.09 |
| genus Eubacterium ventriosum group | Weighted mode | 15 | -0.03 | 1.12 | 0.98 | 0.972 | 0.108-8.748 |
| genus Eubacterium xylanophilum group | IVW | 7 | 0.539 | 0.56 | 0.337 | 1.715 | 0.57-5.159 |
| genus Eubacterium xylanophilum group | MR Egger | 7 | 1.964 | 1.65 | 0.286 | 7.126 | 0.284-179.093 |
| genus Eubacterium xylanophilum group | Weighted median | 7 | 1.001 | 0.71 | 0.156 | 2.722 | 0.682-10.863 |
| genus Eubacterium xylanophilum group | Weighted mode | 7 | 1.05 | 0.98 | 0.324 | 2.858 | 0.421-19.379 |
| genus Family XIII AD3011 group | IVW | 13 | 0.039 | 0.46 | 0.933 | 1.04 | 0.418-2.584 |
| genus Family XIII AD3011 group | MR Egger | 13 | -1.19 | 2.2 | 0.598 | 0.303 | 0.004-22.654 |
| genus Family XIII AD3011 group | Weighted median | 13 | 0.099 | 0.63 | 0.876 | 1.104 | 0.32-3.801 |
| genus Family XIII AD3011 group | Weighted mode | 13 | 0.247 | 0.98 | 0.806 | 1.28 | 0.188-8.73 |
| genus Family XIII UCG001 | IVW | 7 | -0.51 | 0.62 | 0.412 | 0.602 | 0.179-2.023 |
| genus Family XIII UCG001 | MR Egger | 7 | -1.37 | 1.94 | 0.512 | 0.254 | 0.006-11.419 |
| genus Family XIII UCG001 | Weighted median | 7 | -0.36 | 0.83 | 0.666 | 0.699 | 0.137-3.568 |
| genus Family XIII UCG001 | Weighted mode | 7 | 0.358 | 1.29 | 0.791 | 1.43 | 0.114-17.99 |
| genus Haemophilus | IVW | 6 | 0.046 | 0.74 | 0.95 | 1.047 | 0.247-4.441 |
| genus Haemophilus | MR Egger | 6 | 2.319 | 1.41 | 0.176 | 10.17 | 0.638-162.116 |
| genus Haemophilus | Weighted median | 6 | 0.933 | 0.63 | 0.141 | 2.543 | 0.734-8.812 |
| genus Haemophilus | Weighted mode | 6 | 1.05 | 0.74 | 0.216 | 2.856 | 0.667-12.224 |
| genus Holdemania | IVW | 14 | 0.891 | 0.36 | 0.012 | 2.437 | 1.215-4.888 |
| genus Holdemania | MR Egger | 14 | 1.934 | 1.05 | 0.089 | 6.917 | 0.892-53.642 |
| genus Holdemania | Weighted median | 14 | 0.949 | 0.47 | 0.045 | 2.582 | 1.021-6.529 |
| genus Holdemania | Weighted mode | 14 | 1.526 | 0.85 | 0.094 | 4.602 | 0.876-24.161 |
| genus Intestinibacter | IVW | 11 | 0.816 | 0.46 | 0.074 | 2.26 | 0.923-5.533 |
| genus Intestinibacter | MR Egger | 11 | 3.163 | 1.5 | 0.064 | 23.64 | 1.253-446.035 |
| genus Intestinibacter | Weighted median | 11 | 0.858 | 0.62 | 0.165 | 2.359 | 0.702-7.923 |
| genus Intestinibacter | Weighted mode | 11 | 0.784 | 0.9 | 0.403 | 2.19 | 0.378-12.702 |
| genus Lachnoclostridium | IVW | 13 | 1.303 | 0.54 | 0.015 | 3.681 | 1.288-10.521 |
| genus Lachnoclostridium | MR Egger | 13 | 2.701 | 1.86 | 0.174 | 14.89 | 0.391-567.03 |
| genus Lachnoclostridium | Weighted median | 13 | 1.375 | 0.72 | 0.055 | 3.955 | 0.971-16.103 |
| genus Lachnoclostridium | Weighted mode | 13 | 1.425 | 1.12 | 0.226 | 4.159 | 0.465-37.159 |
| genus Lachnospiraceae NC2004 group | IVW | 8 | 0.435 | 0.39 | 0.263 | 1.546 | 0.721-3.315 |
| genus Lachnospiraceae NC2004 group | MR Egger | 8 | 0.45 | 1.69 | 0.799 | 1.568 | 0.057-43.257 |
| genus Lachnospiraceae NC2004 group | Weighted median | 8 | 0.204 | 0.53 | 0.702 | 1.227 | 0.431-3.489 |
| genus Lachnospiraceae NC2004 group | Weighted mode | 8 | 0.247 | 0.79 | 0.762 | 1.28 | 0.275-5.972 |
| genus Lachnospiraceae ND3007 group | IVW | 2 | 0.602 | 1.29 | 0.64 | 1.826 | 0.147-22.723 |
| genus Lachnospiraceae NK4A136 group | IVW | 15 | -0.65 | 0.4 | 0.103 | 0.523 | 0.24-1.141 |
| genus Lachnospiraceae NK4A136 group | MR Egger | 15 | -1.3 | 0.79 | 0.126 | 0.273 | 0.058-1.294 |
| genus Lachnospiraceae NK4A136 group | Weighted median | 15 | -0.3 | 0.61 | 0.618 | 0.738 | 0.224-2.432 |
| genus Lachnospiraceae NK4A136 group | Weighted mode | 15 | -0.36 | 0.65 | 0.591 | 0.701 | 0.198-2.484 |
| genus Lactococcus | IVW | 8 | 0.038 | 0.33 | 0.909 | 1.039 | 0.541-1.996 |
| genus Lactococcus | MR Egger | 8 | -2.11 | 1.7 | 0.262 | 0.121 | 0.004-3.417 |
| genus Lactococcus | Weighted median | 8 | 0.309 | 0.44 | 0.485 | 1.362 | 0.572-3.24 |
| genus Lactococcus | Weighted mode | 8 | 0.451 | 0.58 | 0.465 | 1.569 | 0.5-4.923 |
| genus Methanobrevibacter | IVW | 5 | 0.06 | 0.42 | 0.886 | 1.062 | 0.47-2.4 |
| genus Methanobrevibacter | MR Egger | 5 | 1.509 | 1.39 | 0.356 | 4.521 | 0.298-68.521 |
| genus Methanobrevibacter | Weighted median | 5 | -0.16 | 0.5 | 0.759 | 0.857 | 0.319-2.3 |
| genus Methanobrevibacter | Weighted mode | 5 | -0.27 | 0.64 | 0.695 | 0.764 | 0.219-2.662 |
| genus Odoribacter | IVW | 7 | -0.19 | 0.63 | 0.767 | 0.83 | 0.242-2.849 |
| genus Odoribacter | MR Egger | 7 | 2.781 | 1.95 | 0.212 | 16.14 | 0.357-729.988 |
| genus Odoribacter | Weighted median | 7 | 0.707 | 0.85 | 0.404 | 2.028 | 0.385-10.673 |
| genus Odoribacter | Weighted mode | 7 | 0.899 | 1.16 | 0.469 | 2.457 | 0.251-24.066 |
| genus Oscillospira | IVW | 8 | 0.45 | 0.49 | 0.357 | 1.569 | 0.602-4.086 |
| genus Oscillospira | MR Egger | 8 | 0.617 | 2.06 | 0.774 | 1.854 | 0.033-104.453 |
| genus Oscillospira | Weighted median | 8 | 0.874 | 0.61 | 0.154 | 2.395 | 0.721-7.959 |
| genus Oscillospira | Weighted mode | 8 | 0.941 | 0.96 | 0.357 | 2.563 | 0.394-16.657 |
| genus Oxalobacter | IVW | 11 | 0.215 | 0.3 | 0.474 | 1.24 | 0.688-2.235 |
| genus Oxalobacter | MR Egger | 11 | -1.13 | 1.42 | 0.446 | 0.324 | 0.02-5.18 |
| genus Oxalobacter | Weighted median | 11 | 0.172 | 0.38 | 0.649 | 1.188 | 0.566-2.494 |
| genus Oxalobacter | Weighted mode | 11 | 0.195 | 0.6 | 0.75 | 1.215 | 0.379-3.901 |
| genus Prevotella7 | IVW | 11 | -0.07 | 0.26 | 0.799 | 0.936 | 0.563-1.556 |
| genus Prevotella7 | MR Egger | 11 | -0.88 | 1.53 | 0.579 | 0.414 | 0.021-8.327 |
| genus Prevotella7 | Weighted median | 11 | -0.1 | 0.36 | 0.782 | 0.905 | 0.445-1.838 |
| genus Prevotella7 | Weighted mode | 11 | -0.64 | 0.61 | 0.316 | 0.528 | 0.161-1.729 |
| genus Roseburia | IVW | 13 | 0.464 | 0.51 | 0.364 | 1.591 | 0.584-4.333 |
| genus Roseburia | MR Egger | 13 | 0.55 | 1.53 | 0.727 | 1.734 | 0.086-35.092 |
| genus Roseburia | Weighted median | 13 | 1.018 | 0.7 | 0.145 | 2.768 | 0.705-10.864 |
| genus Roseburia | Weighted mode | 13 | 1.651 | 1.35 | 0.244 | 5.215 | 0.371-73.386 |
| genus Ruminiclostridium5 | IVW | 12 | 0.137 | 0.46 | 0.764 | 1.147 | 0.468-2.814 |
| genus Ruminiclostridium5 | MR Egger | 12 | 0.982 | 1.11 | 0.396 | 2.671 | 0.305-23.42 |
| genus Ruminiclostridium5 | Weighted median | 12 | 0.381 | 0.62 | 0.54 | 1.463 | 0.433-4.947 |
| genus Ruminiclostridium5 | Weighted mode | 12 | 0.487 | 0.75 | 0.53 | 1.627 | 0.373-7.09 |
| genus Ruminococcaceae UCG011 | IVW | 8 | -0.06 | 0.33 | 0.867 | 0.946 | 0.492-1.816 |
| genus Ruminococcaceae UCG011 | MR Egger | 8 | 2.014 | 1.58 | 0.249 | 7.491 | 0.339-165.366 |
| genus Ruminococcaceae UCG011 | Weighted median | 8 | 0.235 | 0.4 | 0.56 | 1.265 | 0.574-2.79 |
| genus Ruminococcaceae UCG011 | Weighted mode | 8 | 0.315 | 0.57 | 0.597 | 1.37 | 0.449-4.181 |
| genus Ruminococcaceae UCG013 | IVW | 10 | 0.326 | 0.63 | 0.606 | 1.385 | 0.401-4.779 |
| genus Ruminococcaceae UCG013 | MR Egger | 10 | -0.24 | 1.88 | 0.9 | 0.783 | 0.02-31.156 |
| genus Ruminococcaceae UCG013 | Weighted median | 10 | -0.58 | 0.76 | 0.448 | 0.563 | 0.127-2.486 |
| genus Ruminococcaceae UCG013 | Weighted mode | 10 | -1.19 | 1.11 | 0.312 | 0.304 | 0.034-2.695 |
| genus Ruminococcaceae UCG014 | IVW | 11 | 0.16 | 0.45 | 0.722 | 1.174 | 0.485-2.839 |
| genus Ruminococcaceae UCG014 | MR Egger | 11 | 1.539 | 1.07 | 0.183 | 4.658 | 0.575-37.722 |
| genus Ruminococcaceae UCG014 | Weighted median | 11 | 0.048 | 0.63 | 0.94 | 1.049 | 0.305-3.608 |
| genus Ruminococcaceae UCG014 | Weighted mode | 11 | 1.297 | 0.81 | 0.141 | 3.657 | 0.745-17.96 |
| genus Ruminococcus gauvreauii group | IVW | 10 | -0.04 | 0.49 | 0.938 | 0.963 | 0.366-2.533 |
| genus Ruminococcus gauvreauii group | MR Egger | 10 | -0.85 | 1.99 | 0.679 | 0.427 | 0.009-20.955 |
| genus Ruminococcus gauvreauii group | Weighted median | 10 | -0.18 | 0.64 | 0.777 | 0.835 | 0.24-2.905 |
| genus Ruminococcus gauvreauii group | Weighted mode | 10 | -0.12 | 1.03 | 0.908 | 0.885 | 0.119-6.602 |
| genus Streptococcus | IVW | 12 | 0.095 | 0.52 | 0.857 | 1.099 | 0.394-3.07 |
| genus Streptococcus | MR Egger | 12 | 0.018 | 1.92 | 0.993 | 1.018 | 0.023-44.243 |
| genus Streptococcus | Weighted median | 12 | -0.05 | 0.69 | 0.942 | 0.951 | 0.247-3.659 |
| genus Streptococcus | Weighted mode | 12 | -0.29 | 1.08 | 0.793 | 0.748 | 0.09-6.216 |
| genus Tyzzerella3 | IVW | 13 | -0.09 | 0.35 | 0.796 | 0.912 | 0.456-1.826 |
| genus Tyzzerella3 | MR Egger | 13 | 2.791 | 1.93 | 0.176 | 16.3 | 0.372-713.152 |
| genus Tyzzerella3 | Weighted median | 13 | -0.21 | 0.41 | 0.612 | 0.812 | 0.362-1.817 |
| genus Tyzzerella3 | Weighted mode | 13 | -0.19 | 0.59 | 0.749 | 0.823 | 0.257-2.635 |
| genus Veillonella | IVW | 6 | -0.33 | 0.61 | 0.593 | 0.721 | 0.217-2.395 |
| genus Veillonella | MR Egger | 6 | -1.66 | 5.38 | 0.773 | 0.19 | 0-7174.059 |
| genus Veillonella | Weighted median | 6 | 0.082 | 0.7 | 0.906 | 1.086 | 0.278-4.238 |
| genus Veillonella | Weighted mode | 6 | 0.122 | 1.11 | 0.916 | 1.13 | 0.129-9.911 |
| genus Victivallis | IVW | 10 | -0.68 | 0.27 | 0.011 | 0.509 | 0.302-0.857 |
| genus Victivallis | MR Egger | 10 | 0.449 | 2.03 | 0.831 | 1.567 | 0.029-84.087 |
| genus Victivallis | Weighted median | 10 | -0.93 | 0.36 | 0.011 | 0.397 | 0.195-0.808 |
| genus Victivallis | Weighted mode | 10 | -1.16 | 0.64 | 0.102 | 0.315 | 0.091-1.093 |
| genus Alistipes | IVW | 12 | -0.57 | 0.58 | 0.328 | 0.568 | 0.183-1.766 |
| genus Alistipes | MR Egger | 12 | 1.488 | 2.75 | 0.601 | 4.427 | 0.02-978.046 |
| genus Alistipes | Weighted median | 12 | -0.96 | 0.78 | 0.219 | 0.382 | 0.082-1.774 |
| genus Alistipes | Weighted mode | 12 | -1.14 | 1.24 | 0.377 | 0.32 | 0.028-3.619 |
| genus Allisonella | IVW | 7 | -0.06 | 0.29 | 0.847 | 0.946 | 0.54-1.658 |
| genus Allisonella | MR Egger | 7 | -2.53 | 1.85 | 0.231 | 0.08 | 0.002-3.013 |
| genus Allisonella | Weighted median | 7 | 0.126 | 0.39 | 0.747 | 1.135 | 0.526-2.447 |
| genus Allisonella | Weighted mode | 7 | 0.448 | 0.63 | 0.5 | 1.565 | 0.46-5.33 |
| genus Anaerotruncus | IVW | 13 | -0.08 | 0.59 | 0.893 | 0.923 | 0.288-2.957 |
| genus Anaerotruncus | MR Egger | 13 | 0.984 | 1.78 | 0.591 | 2.675 | 0.082-87.341 |
| genus Anaerotruncus | Weighted median | 13 | 0.285 | 0.69 | 0.68 | 1.329 | 0.343-5.147 |
| genus Anaerotruncus | Weighted mode | 13 | 1.101 | 1.3 | 0.415 | 3.006 | 0.234-38.653 |
| genus Bifidobacterium | IVW | 10 | -0.33 | 0.51 | 0.52 | 0.72 | 0.264-1.96 |
| genus Bifidobacterium | MR Egger | 10 | 1.669 | 1.03 | 0.142 | 5.308 | 0.711-39.612 |
| genus Bifidobacterium | Weighted median | 10 | -0.42 | 0.65 | 0.517 | 0.655 | 0.183-2.35 |
| genus Bifidobacterium | Weighted mode | 10 | -0.81 | 0.87 | 0.374 | 0.445 | 0.082-2.428 |
| genus Bilophila | IVW | 13 | -0.13 | 0.49 | 0.792 | 0.879 | 0.337-2.291 |
| genus Bilophila | MR Egger | 13 | 2.959 | 2.32 | 0.229 | 19.28 | 0.203-1828.055 |
| genus Bilophila | Weighted median | 13 | 0.553 | 0.67 | 0.407 | 1.739 | 0.47-6.428 |
| genus Bilophila | Weighted mode | 13 | 0.936 | 1.26 | 0.473 | 2.551 | 0.215-30.329 |
| genus Blautia | IVW | 2 | 1.731 | 1.33 | 0.193 | 5.647 | 0.416-76.582 |
| genus Candidatus Soleaferrea | IVW | 9 | -0.43 | 0.37 | 0.238 | 0.65 | 0.318-1.329 |
| genus Candidatus Soleaferrea | MR Egger | 9 | 0.09 | 3.72 | 0.981 | 1.094 | 0.001-1606.966 |
| genus Candidatus Soleaferrea | Weighted median | 9 | -0.59 | 0.48 | 0.213 | 0.552 | 0.216-1.408 |
| genus Candidatus Soleaferrea | Weighted mode | 9 | -0.91 | 0.79 | 0.285 | 0.404 | 0.086-1.904 |
| genus Coprobacter | IVW | 10 | 0.036 | 0.42 | 0.931 | 1.037 | 0.458-2.345 |
| genus Coprobacter | MR Egger | 10 | -0.04 | 1.65 | 0.983 | 0.965 | 0.038-24.548 |
| genus Coprobacter | Weighted median | 10 | 0.452 | 0.49 | 0.361 | 1.571 | 0.596-4.14 |
| genus Coprobacter | Weighted mode | 10 | 0.561 | 0.74 | 0.47 | 1.752 | 0.408-7.514 |
| genus Coprococcus3 | IVW | 9 | 0.231 | 0.61 | 0.706 | 1.26 | 0.379-4.188 |
| genus Coprococcus3 | MR Egger | 9 | -0.25 | 3.35 | 0.943 | 0.781 | 0.001-555.971 |
| genus Coprococcus3 | Weighted median | 9 | 0.322 | 0.79 | 0.682 | 1.38 | 0.296-6.442 |
| genus Coprococcus3 | Weighted mode | 9 | 0.88 | 1.13 | 0.458 | 2.412 | 0.264-22.074 |
| genus Dorea | IVW | 10 | -0.36 | 0.59 | 0.541 | 0.696 | 0.218-2.225 |
| genus Dorea | MR Egger | 10 | 0.107 | 1.64 | 0.95 | 1.113 | 0.044-27.873 |
| genus Dorea | Weighted median | 10 | -0.34 | 0.73 | 0.644 | 0.714 | 0.17-2.989 |
| genus Dorea | Weighted mode | 10 | -0.32 | 1.04 | 0.763 | 0.724 | 0.095-5.539 |
| genus Erysipelatoclostridium | IVW | 14 | -0.13 | 0.37 | 0.719 | 0.877 | 0.429-1.793 |
| genus Erysipelatoclostridium | MR Egger | 14 | 0.671 | 1.4 | 0.64 | 1.957 | 0.126-30.307 |
| genus Erysipelatoclostridium | Weighted median | 14 | -0.17 | 0.45 | 0.715 | 0.847 | 0.348-2.063 |
| genus Erysipelatoclostridium | Weighted mode | 14 | -0.56 | 0.81 | 0.502 | 0.574 | 0.118-2.78 |
| genus Escherichia Shigella | IVW | 10 | -0.12 | 0.68 | 0.863 | 0.889 | 0.234-3.376 |
| genus Escherichia Shigella | MR Egger | 10 | 1.541 | 2.15 | 0.494 | 4.671 | 0.069-315.834 |
| genus Escherichia Shigella | Weighted median | 10 | 0.137 | 0.72 | 0.85 | 1.147 | 0.278-4.733 |
| genus Escherichia Shigella | Weighted mode | 10 | 0.205 | 1.2 | 0.868 | 1.228 | 0.118-12.799 |
| genus Eubacterium brachy group | IVW | 10 | 0.342 | 0.29 | 0.233 | 1.408 | 0.803-2.471 |
| genus Eubacterium brachy group | MR Egger | 10 | 0.264 | 1.15 | 0.825 | 1.302 | 0.136-12.495 |
| genus Eubacterium brachy group | Weighted median | 10 | 0.166 | 0.38 | 0.659 | 1.18 | 0.565-2.468 |
| genus Eubacterium brachy group | Weighted mode | 10 | -0.01 | 0.63 | 0.993 | 0.994 | 0.291-3.4 |
| genus Eubacterium coprostanoligenes group | IVW | 13 | 0.569 | 0.63 | 0.365 | 1.767 | 0.515-6.06 |
| genus Eubacterium coprostanoligenes group | MR Egger | 13 | 3.459 | 2.42 | 0.181 | 31.77 | 0.277-3640.186 |
| genus Eubacterium coprostanoligenes group | Weighted median | 13 | 0.406 | 0.78 | 0.604 | 1.5 | 0.324-6.937 |
| genus Eubacterium coprostanoligenes group | Weighted mode | 13 | 1.815 | 1.4 | 0.22 | 6.142 | 0.394-95.726 |
| genus Eubacterium eligens group | IVW | 6 | -2.04 | 0.68 | 0.003 | 0.13 | 0.034-0.495 |
| genus Eubacterium eligens group | MR Egger | 6 | -2.12 | 2.54 | 0.451 | 0.12 | 0.001-17.489 |
| genus Eubacterium eligens group | Weighted median | 6 | -2.2 | 0.88 | 0.012 | 0.111 | 0.02-0.616 |
| genus Eubacterium eligens group | Weighted mode | 6 | -2.94 | 1.24 | 0.064 | 0.053 | 0.005-0.604 |
| genus Eubacterium ruminantium group | IVW | 18 | 0.769 | 0.3 | 0.009 | 2.157 | 1.211-3.843 |
| genus Eubacterium ruminantium group | MR Egger | 18 | 0.467 | 1.01 | 0.651 | 1.595 | 0.219-11.61 |
| genus Eubacterium ruminantium group | Weighted median | 18 | 0.636 | 0.39 | 0.105 | 1.889 | 0.875-4.076 |
| genus Eubacterium ruminantium group | Weighted mode | 18 | 0.607 | 0.63 | 0.349 | 1.836 | 0.533-6.326 |
| genus Faecalibacterium | IVW | 10 | 0.131 | 0.45 | 0.77 | 1.14 | 0.473-2.752 |
| genus Faecalibacterium | MR Egger | 10 | -0.19 | 0.88 | 0.834 | 0.828 | 0.149-4.606 |
| genus Faecalibacterium | Weighted median | 10 | 0 | 0.58 | 0.999 | 0.999 | 0.318-3.136 |
| genus Faecalibacterium | Weighted mode | 10 | 0.026 | 0.75 | 0.973 | 1.026 | 0.234-4.493 |
| genus Flavonifractor | IVW | 5 | 0.182 | 0.63 | 0.774 | 1.2 | 0.347-4.152 |
| genus Flavonifractor | MR Egger | 5 | 2.304 | 2.53 | 0.43 | 10.01 | 0.07-1427.599 |
| genus Flavonifractor | Weighted median | 5 | 0.225 | 0.83 | 0.785 | 1.252 | 0.248-6.33 |
| genus Flavonifractor | Weighted mode | 5 | 0.213 | 1.04 | 0.847 | 1.238 | 0.162-9.459 |
| genus Holdemanella | IVW | 11 | -0.06 | 0.35 | 0.867 | 0.943 | 0.476-1.869 |
| genus Holdemanella | MR Egger | 11 | 0.367 | 1.04 | 0.733 | 1.443 | 0.188-11.079 |
| genus Holdemanella | Weighted median | 11 | 0.022 | 0.46 | 0.961 | 1.023 | 0.416-2.512 |
| genus Holdemanella | Weighted mode | 11 | 0.079 | 0.76 | 0.919 | 1.082 | 0.245-4.784 |
| genus Howardella | IVW | 9 | 0.014 | 0.31 | 0.964 | 1.014 | 0.553-1.859 |
| genus Howardella | MR Egger | 9 | -0.79 | 1.28 | 0.556 | 0.453 | 0.037-5.557 |
| genus Howardella | Weighted median | 9 | -0.1 | 0.39 | 0.803 | 0.909 | 0.427-1.931 |
| genus Howardella | Weighted mode | 9 | -0.27 | 0.51 | 0.605 | 0.762 | 0.282-2.053 |
| genus Hungatella | IVW | 3 | 0.589 | 0.53 | 0.27 | 1.802 | 0.632-5.136 |
| genus Hungatella | MR Egger | 3 | -3.53 | 6.95 | 0.701 | 0.029 | 0-24390.833 |
| genus Hungatella | Weighted median | 3 | 0.632 | 0.63 | 0.319 | 1.881 | 0.542-6.521 |
| genus Hungatella | Weighted mode | 3 | 0.314 | 0.74 | 0.713 | 1.369 | 0.32-5.859 |
| genus Lachnospiraceae UCG004 | IVW | 12 | -0.19 | 0.56 | 0.738 | 0.828 | 0.274-2.502 |
| genus Lachnospiraceae UCG004 | MR Egger | 12 | -0.62 | 2.46 | 0.806 | 0.538 | 0.004-66.327 |
| genus Lachnospiraceae UCG004 | Weighted median | 12 | 0.064 | 0.69 | 0.926 | 1.066 | 0.278-4.082 |
| genus Lachnospiraceae UCG004 | Weighted mode | 12 | 1.128 | 1.13 | 0.34 | 3.088 | 0.337-28.285 |
| genus Lachnospiraceae UCG010 | IVW | 9 | 0.379 | 0.86 | 0.659 | 1.461 | 0.271-7.889 |
| genus Lachnospiraceae UCG010 | MR Egger | 9 | -1.57 | 2.5 | 0.551 | 0.209 | 0.002-28.198 |
| genus Lachnospiraceae UCG010 | Weighted median | 9 | -0.44 | 0.8 | 0.584 | 0.647 | 0.136-3.082 |
| genus Lachnospiraceae UCG010 | Weighted mode | 9 | -1.06 | 1.19 | 0.398 | 0.346 | 0.034-3.558 |
| genus Olsenella | IVW | 10 | -0.09 | 0.34 | 0.781 | 0.91 | 0.467-1.771 |
| genus Olsenella | MR Egger | 10 | -1.51 | 1.03 | 0.182 | 0.222 | 0.029-1.669 |
| genus Olsenella | Weighted median | 10 | -0.39 | 0.37 | 0.292 | 0.676 | 0.326-1.401 |
| genus Olsenella | Weighted mode | 10 | -0.62 | 0.52 | 0.257 | 0.536 | 0.195-1.474 |
| genus Oscillibacter | IVW | 13 | -0.18 | 0.36 | 0.622 | 0.838 | 0.416-1.69 |
| genus Oscillibacter | MR Egger | 13 | 0.313 | 1.35 | 0.821 | 1.367 | 0.096-19.402 |
| genus Oscillibacter | Weighted median | 13 | -0.51 | 0.49 | 0.301 | 0.6 | 0.228-1.579 |
| genus Oscillibacter | Weighted mode | 13 | -0.7 | 0.83 | 0.413 | 0.497 | 0.099-2.504 |
| genus Paraprevotella | IVW | 13 | -0.39 | 0.31 | 0.199 | 0.676 | 0.372-1.229 |
| genus Paraprevotella | MR Egger | 13 | -1.33 | 1.16 | 0.275 | 0.265 | 0.027-2.557 |
| genus Paraprevotella | Weighted median | 13 | -0.3 | 0.42 | 0.478 | 0.742 | 0.325-1.693 |
| genus Paraprevotella | Weighted mode | 13 | -0.41 | 0.65 | 0.541 | 0.663 | 0.185-2.384 |
| genus Parasutterella | IVW | 14 | 0.149 | 0.36 | 0.682 | 1.161 | 0.569-2.366 |
| genus Parasutterella | MR Egger | 14 | 1.912 | 1.01 | 0.082 | 6.765 | 0.936-48.885 |
| genus Parasutterella | Weighted median | 14 | 0.346 | 0.51 | 0.495 | 1.413 | 0.523-3.813 |
| genus Parasutterella | Weighted mode | 14 | 0.438 | 0.83 | 0.608 | 1.549 | 0.303-7.915 |
| ebi-a-GCST90017043 | IVW | 6 | -0.01 | 0.58 | 0.993 | 0.995 | 0.319-3.101 |
| ebi-a-GCST90017043 | MR Egger | 6 | 0.852 | 3.31 | 0.81 | 2.345 | 0.004-1545.738 |
| ebi-a-GCST90017043 | Weighted median | 6 | 0.109 | 0.7 | 0.877 | 1.115 | 0.281-4.431 |
| ebi-a-GCST90017043 | Weighted mode | 6 | 0.007 | 0.92 | 0.994 | 1.007 | 0.166-6.112 |
| genus Prevotella9 | IVW | 15 | 0.077 | 0.38 | 0.841 | 1.08 | 0.509-2.292 |
| genus Prevotella9 | MR Egger | 15 | 0.12 | 1.16 | 0.919 | 1.127 | 0.116-11.004 |
| genus Prevotella9 | Weighted median | 15 | 0.101 | 0.47 | 0.83 | 1.107 | 0.44-2.783 |
| genus Prevotella9 | Weighted mode | 15 | 0.224 | 0.65 | 0.737 | 1.251 | 0.347-4.51 |
| genus Rikenellaceae RC9 gut group | IVW | 9 | -0.26 | 0.27 | 0.336 | 0.768 | 0.449-1.315 |
| genus Rikenellaceae RC9 gut group | MR Egger | 9 | 0.461 | 1.86 | 0.811 | 1.585 | 0.042-60.507 |
| genus Rikenellaceae RC9 gut group | Weighted median | 9 | -0.19 | 0.33 | 0.563 | 0.824 | 0.428-1.586 |
| genus Rikenellaceae RC9 gut group | Weighted mode | 9 | 0.01 | 0.51 | 0.985 | 1.01 | 0.372-2.74 |
| genus Ruminiclostridium9 | IVW | 6 | 0.146 | 0.76 | 0.847 | 1.157 | 0.262-5.121 |
| genus Ruminiclostridium9 | MR Egger | 6 | 1.088 | 3.68 | 0.782 | 2.969 | 0.002-4029.497 |
| genus Ruminiclostridium9 | Weighted median | 6 | 0.431 | 0.95 | 0.65 | 1.539 | 0.239-9.925 |
| genus Ruminiclostridium9 | Weighted mode | 6 | 0.906 | 1.3 | 0.516 | 2.474 | 0.194-31.485 |
| genus Ruminococcaceae UCG002 | IVW | 17 | -0.57 | 0.45 | 0.203 | 0.565 | 0.234-1.362 |
| genus Ruminococcaceae UCG002 | MR Egger | 17 | -1.03 | 1.24 | 0.418 | 0.355 | 0.031-4.06 |
| genus Ruminococcaceae UCG002 | Weighted median | 17 | -0.07 | 0.61 | 0.904 | 0.929 | 0.284-3.044 |
| genus Ruminococcaceae UCG002 | Weighted mode | 17 | -0.02 | 0.77 | 0.985 | 0.985 | 0.219-4.433 |
| genus Ruminococcus torques group | IVW | 7 | 0.022 | 0.74 | 0.976 | 1.022 | 0.24-4.345 |
| genus Ruminococcus torques group | MR Egger | 7 | 2.144 | 3.29 | 0.544 | 8.534 | 0.013-5436.318 |
| genus Ruminococcus torques group | Weighted median | 7 | 0.007 | 0.93 | 0.994 | 1.007 | 0.163-6.211 |
| genus Ruminococcus torques group | Weighted mode | 7 | -1.34 | 1.38 | 0.37 | 0.263 | 0.018-3.919 |
| genus Sellimonas | IVW | 9 | -0.07 | 0.25 | 0.791 | 0.935 | 0.569-1.537 |
| genus Sellimonas | MR Egger | 9 | -0.59 | 1.48 | 0.704 | 0.556 | 0.031-10.137 |
| genus Sellimonas | Weighted median | 9 | 0.04 | 0.31 | 0.899 | 1.041 | 0.563-1.924 |
| genus Sellimonas | Weighted mode | 9 | 0.045 | 0.5 | 0.93 | 1.046 | 0.395-2.773 |
| genus Slackia | IVW | 7 | 0.594 | 0.46 | 0.196 | 1.811 | 0.736-4.453 |
| genus Slackia | MR Egger | 7 | -2.26 | 2.37 | 0.385 | 0.104 | 0.001-10.944 |
| genus Slackia | Weighted median | 7 | 0.693 | 0.59 | 0.239 | 2 | 0.631-6.339 |
| genus Slackia | Weighted mode | 7 | 0.845 | 0.88 | 0.374 | 2.329 | 0.414-13.093 |
| genus Subdoligranulum | IVW | 8 | 0.618 | 0.68 | 0.361 | 1.855 | 0.493-6.979 |
| genus Subdoligranulum | MR Egger | 8 | -0.19 | 1.95 | 0.926 | 0.829 | 0.018-37.589 |
| genus Subdoligranulum | Weighted median | 8 | 0.287 | 0.78 | 0.712 | 1.333 | 0.289-6.135 |
| genus Subdoligranulum | Weighted mode | 8 | 0.321 | 0.99 | 0.756 | 1.378 | 0.197-9.643 |
| genus Sutterella | IVW | 12 | 0.264 | 0.62 | 0.669 | 1.302 | 0.388-4.362 |
| genus Sutterella | MR Egger | 12 | 0.404 | 2.79 | 0.888 | 1.497 | 0.006-357.467 |
| genus Sutterella | Weighted median | 12 | 1.197 | 0.71 | 0.091 | 3.311 | 0.824-13.297 |
| genus Sutterella | Weighted mode | 12 | 1.939 | 1.19 | 0.132 | 6.949 | 0.674-71.625 |
| genus Terrisporobacter | IVW | 4 | -0.69 | 0.59 | 0.243 | 0.501 | 0.157-1.598 |
| genus Terrisporobacter | MR Egger | 4 | -0.87 | 1.69 | 0.658 | 0.419 | 0.015-11.563 |
| genus Terrisporobacter | Weighted median | 4 | -0.84 | 0.68 | 0.22 | 0.433 | 0.113-1.651 |
| genus Terrisporobacter | Weighted mode | 4 | -1.12 | 0.97 | 0.331 | 0.326 | 0.049-2.171 |
| order Actinomycetales | IVW | 4 | -0.43 | 0.72 | 0.548 | 0.649 | 0.158-2.66 |
| order Actinomycetales | MR Egger | 4 | -1.56 | 1.71 | 0.456 | 0.21 | 0.007-5.934 |
| order Actinomycetales | Weighted median | 4 | -0.68 | 0.72 | 0.345 | 0.505 | 0.122-2.085 |
| order Actinomycetales | Weighted mode | 4 | -0.99 | 0.73 | 0.266 | 0.37 | 0.089-1.543 |
| order Bacillales | IVW | 8 | -0.27 | 0.3 | 0.365 | 0.761 | 0.421-1.375 |
| order Bacillales | MR Egger | 8 | -0.41 | 1.31 | 0.764 | 0.661 | 0.05-8.695 |
| order Bacillales | Weighted median | 8 | -0.31 | 0.38 | 0.417 | 0.734 | 0.347-1.55 |
| order Bacillales | Weighted mode | 8 | -0.47 | 0.54 | 0.417 | 0.626 | 0.216-1.815 |
| order Bacteroidales | IVW | 9 | 0.196 | 0.67 | 0.77 | 1.217 | 0.327-4.534 |
| order Bacteroidales | MR Egger | 9 | -1.32 | 1.45 | 0.392 | 0.266 | 0.015-4.572 |
| order Bacteroidales | Weighted median | 9 | 0.066 | 0.86 | 0.939 | 1.069 | 0.197-5.801 |
| order Bacteroidales | Weighted mode | 9 | -0.33 | 1.19 | 0.789 | 0.72 | 0.07-7.43 |
| order Bifidobacteriales | IVW | 9 | -0.68 | 0.62 | 0.271 | 0.506 | 0.151-1.699 |
| order Bifidobacteriales | MR Egger | 9 | 3.049 | 1.61 | 0.1 | 21.09 | 0.903-492.777 |
| order Bifidobacteriales | Weighted median | 9 | -0.98 | 0.8 | 0.219 | 0.377 | 0.079-1.788 |
| order Bifidobacteriales | Weighted mode | 9 | 1.125 | 0.81 | 0.202 | 3.08 | 0.631-15.029 |
| order Burkholderiales | IVW | 8 | 0.569 | 0.93 | 0.542 | 1.767 | 0.283-11.022 |
| order Burkholderiales | MR Egger | 8 | 3.089 | 2.91 | 0.329 | 21.96 | 0.073-6565.585 |
| order Burkholderiales | Weighted median | 8 | 0.742 | 0.9 | 0.409 | 2.1 | 0.36-12.239 |
| order Burkholderiales | Weighted mode | 8 | 1.076 | 1.24 | 0.415 | 2.933 | 0.257-33.456 |
| order Clostridiales | IVW | 13 | 0.111 | 0.49 | 0.819 | 1.118 | 0.431-2.898 |
| order Clostridiales | MR Egger | 13 | -1.59 | 1.32 | 0.255 | 0.204 | 0.015-2.732 |
| order Clostridiales | Weighted median | 13 | -0.07 | 0.69 | 0.918 | 0.932 | 0.241-3.597 |
| order Clostridiales | Weighted mode | 13 | 0.166 | 1.09 | 0.881 | 1.181 | 0.139-10.011 |
| order Coriobacteriales | IVW | 12 | -0.49 | 0.52 | 0.351 | 0.616 | 0.222-1.705 |
| order Coriobacteriales | MR Egger | 12 | -1.22 | 2.13 | 0.578 | 0.295 | 0.005-19.052 |
| order Coriobacteriales | Weighted median | 12 | -0.87 | 0.69 | 0.208 | 0.418 | 0.108-1.626 |
| order Coriobacteriales | Weighted mode | 12 | -1.1 | 1.11 | 0.346 | 0.334 | 0.038-2.968 |
| order Desulfovibrionales | IVW | 10 | 0.153 | 0.56 | 0.783 | 1.165 | 0.392-3.464 |
| order Desulfovibrionales | MR Egger | 10 | 3.44 | 2.75 | 0.246 | 31.18 | 0.143-6798.511 |
| order Desulfovibrionales | Weighted median | 10 | 0.027 | 0.77 | 0.972 | 1.027 | 0.227-4.647 |
| order Desulfovibrionales | Weighted mode | 10 | -0.29 | 1.21 | 0.816 | 0.747 | 0.069-8.076 |
| order Enterobacteriales | IVW | 3 | -1.98 | 1.48 | 0.181 | 0.138 | 0.008-2.515 |
| order Enterobacteriales | MR Egger | 3 | -13.4 | 5.42 | 0.245 | 0 | 0-0.063 |
| order Enterobacteriales | Weighted median | 3 | -2.76 | 1.26 | 0.028 | 0.064 | 0.005-0.748 |
| order Enterobacteriales | Weighted mode | 3 | -3.57 | 2.21 | 0.247 | 0.028 | 0-2.136 |
| order Erysipelotrichales | IVW | 13 | -0.24 | 0.53 | 0.657 | 0.789 | 0.277-2.244 |
| order Erysipelotrichales | MR Egger | 13 | 1.487 | 2.31 | 0.533 | 4.425 | 0.048-409.303 |
| order Erysipelotrichales | Weighted median | 13 | -0.24 | 0.73 | 0.739 | 0.785 | 0.19-3.25 |
| order Erysipelotrichales | Weighted mode | 13 | -0.09 | 1.09 | 0.934 | 0.911 | 0.107-7.786 |
| order Gastranaerophilales | IVW | 9 | 0.415 | 0.4 | 0.299 | 1.514 | 0.693-3.309 |
| order Gastranaerophilales | MR Egger | 9 | 0.152 | 1.27 | 0.908 | 1.165 | 0.097-13.967 |
| order Gastranaerophilales | Weighted median | 9 | 0.437 | 0.5 | 0.385 | 1.548 | 0.578-4.148 |
| order Gastranaerophilales | Weighted mode | 9 | 0.641 | 0.78 | 0.437 | 1.899 | 0.409-8.808 |
| order Lactobacillales | IVW | 15 | -0.55 | 0.46 | 0.234 | 0.578 | 0.235-1.426 |
| order Lactobacillales | MR Egger | 15 | -0.42 | 1.18 | 0.727 | 0.656 | 0.065-6.662 |
| order Lactobacillales | Weighted median | 15 | -0.36 | 0.64 | 0.567 | 0.695 | 0.2-2.412 |
| order Lactobacillales | Weighted mode | 15 | -0.3 | 0.91 | 0.746 | 0.74 | 0.123-4.435 |
| order Methanobacteriales | IVW | 9 | 0 | 0.3 | 0.988 | 0.996 | 0.558-1.776 |
| order Methanobacteriales | MR Egger | 9 | 0.566 | 1.16 | 0.64 | 1.761 | 0.182-17.012 |
| order Methanobacteriales | Weighted median | 9 | 0.013 | 0.39 | 0.974 | 1.013 | 0.471-2.18 |
| order Methanobacteriales | Weighted mode | 9 | -0.17 | 0.54 | 0.762 | 0.844 | 0.293-2.435 |
| order Mollicutes RF9 | IVW | 12 | -0.34 | 0.41 | 0.397 | 0.71 | 0.321-1.571 |
| order Mollicutes RF9 | MR Egger | 12 | -0.25 | 1.23 | 0.846 | 0.782 | 0.07-8.744 |
| order Mollicutes RF9 | Weighted median | 12 | -0.18 | 0.57 | 0.755 | 0.837 | 0.274-2.56 |
| order Mollicutes RF9 | Weighted mode | 12 | 0.154 | 0.85 | 0.86 | 1.166 | 0.221-6.156 |
| order NB1n | IVW | 12 | -0.03 | 0.29 | 0.922 | 0.972 | 0.557-1.699 |
| order NB1n | MR Egger | 12 | -0.5 | 1.2 | 0.687 | 0.608 | 0.058-6.367 |
| order NB1n | Weighted median | 12 | -0.12 | 0.38 | 0.759 | 0.891 | 0.425-1.866 |
| order NB1n | Weighted mode | 12 | -0.31 | 0.62 | 0.626 | 0.732 | 0.216-2.48 |
| order Pasteurellales | IVW | 13 | -0.04 | 0.45 | 0.935 | 0.964 | 0.402-2.314 |
| order Pasteurellales | MR Egger | 13 | 1.546 | 0.84 | 0.094 | 4.691 | 0.898-24.498 |
| order Pasteurellales | Weighted median | 13 | 0.728 | 0.48 | 0.131 | 2.071 | 0.804-5.334 |
| order Pasteurellales | Weighted mode | 13 | 0.774 | 0.59 | 0.212 | 2.169 | 0.686-6.853 |
| order Rhodospirillales | IVW | 12 | 0.213 | 0.37 | 0.57 | 1.237 | 0.595-2.573 |
| order Rhodospirillales | MR Egger | 12 | 3.646 | 2.08 | 0.111 | 38.31 | 0.646-2272.726 |
| order Rhodospirillales | Weighted median | 12 | 0.422 | 0.55 | 0.446 | 1.525 | 0.514-4.522 |
| order Rhodospirillales | Weighted mode | 12 | 0.842 | 1.01 | 0.422 | 2.321 | 0.321-16.773 |
| order Selenomonadales | IVW | 10 | 0.476 | 0.58 | 0.41 | 1.609 | 0.519-4.988 |
| order Selenomonadales | MR Egger | 10 | -1.42 | 1.9 | 0.477 | 0.242 | 0.006-10.089 |
| order Selenomonadales | Weighted median | 10 | 0.197 | 0.75 | 0.793 | 1.218 | 0.278-5.341 |
| order Selenomonadales | Weighted mode | 10 | -0.55 | 1.25 | 0.671 | 0.578 | 0.05-6.683 |
| order Verrucomicrobiales | IVW | 11 | -0.41 | 0.45 | 0.358 | 0.664 | 0.278-1.59 |
| order Verrucomicrobiales | MR Egger | 11 | -1.62 | 1.49 | 0.305 | 0.197 | 0.011-3.67 |
| order Verrucomicrobiales | Weighted median | 11 | -0.36 | 0.58 | 0.539 | 0.7 | 0.224-2.185 |
| order Verrucomicrobiales | Weighted mode | 11 | -1.32 | 1 | 0.219 | 0.267 | 0.037-1.915 |
| order Victivallales | IVW | 7 | -0.28 | 0.36 | 0.428 | 0.753 | 0.373-1.519 |
| order Victivallales | MR Egger | 7 | -0.03 | 1.33 | 0.981 | 0.968 | 0.072-13.062 |
| order Victivallales | Weighted median | 7 | -0.09 | 0.45 | 0.842 | 0.915 | 0.38-2.201 |
| order Victivallales | Weighted mode | 7 | 0.166 | 0.67 | 0.813 | 1.18 | 0.318-4.385 |
| phylum Actinobacteria | IVW | 14 | -0.1 | 0.53 | 0.85 | 0.905 | 0.321-2.554 |
| phylum Actinobacteria | MR Egger | 14 | 0.307 | 2.28 | 0.895 | 1.359 | 0.016-118.473 |
| phylum Actinobacteria | Weighted median | 14 | 0.442 | 0.68 | 0.518 | 1.555 | 0.408-5.927 |
| phylum Actinobacteria | Weighted mode | 14 | 0.963 | 1 | 0.351 | 2.62 | 0.372-18.437 |
| phylum Bacteroidetes | IVW | 9 | -0.52 | 0.62 | 0.4 | 0.595 | 0.177-1.995 |
| phylum Bacteroidetes | MR Egger | 9 | -0.58 | 1.44 | 0.699 | 0.56 | 0.033-9.385 |
| phylum Bacteroidetes | Weighted median | 9 | -0.81 | 0.8 | 0.308 | 0.445 | 0.094-2.114 |
| phylum Bacteroidetes | Weighted mode | 9 | -0.99 | 1.2 | 0.435 | 0.372 | 0.035-3.924 |
| phylum Cyanobacteria | IVW | 6 | 0.487 | 0.51 | 0.34 | 1.627 | 0.598-4.425 |
| phylum Cyanobacteria | MR Egger | 6 | 1.489 | 1.77 | 0.447 | 4.431 | 0.139-141.036 |
| phylum Cyanobacteria | Weighted median | 6 | 0.395 | 0.63 | 0.531 | 1.484 | 0.431-5.111 |
| phylum Cyanobacteria | Weighted mode | 6 | 0.28 | 0.95 | 0.78 | 1.323 | 0.206-8.517 |
| phylum Euryarchaeota | IVW | 11 | -0.16 | 0.26 | 0.537 | 0.851 | 0.511-1.418 |
| phylum Euryarchaeota | MR Egger | 11 | 0.381 | 1.16 | 0.75 | 1.464 | 0.151-14.18 |
| phylum Euryarchaeota | Weighted median | 11 | -0.08 | 0.34 | 0.816 | 0.925 | 0.48-1.784 |
| phylum Euryarchaeota | Weighted mode | 11 | -0.42 | 0.52 | 0.435 | 0.657 | 0.239-1.808 |
| phylum Firmicutes | IVW | 11 | -0.33 | 0.64 | 0.61 | 0.722 | 0.206-2.526 |
| phylum Firmicutes | MR Egger | 11 | 0.963 | 1.4 | 0.51 | 2.618 | 0.167-40.981 |
| phylum Firmicutes | Weighted median | 11 | 0.527 | 0.7 | 0.448 | 1.694 | 0.434-6.614 |
| phylum Firmicutes | Weighted mode | 11 | 0.561 | 0.8 | 0.498 | 1.753 | 0.367-8.377 |
| phylum Lentisphaerae | IVW | 8 | -0.04 | 0.4 | 0.921 | 0.961 | 0.435-2.12 |
| phylum Lentisphaerae | MR Egger | 8 | 1.757 | 1.62 | 0.32 | 5.794 | 0.243-138.429 |
| phylum Lentisphaerae | Weighted median | 8 | 0.021 | 0.46 | 0.964 | 1.021 | 0.418-2.494 |
| phylum Lentisphaerae | Weighted mode | 8 | 0.201 | 0.61 | 0.753 | 1.222 | 0.368-4.063 |
| phylum Proteobacteria | IVW | 11 | 0.082 | 0.54 | 0.879 | 1.085 | 0.378-3.113 |
| phylum Proteobacteria | MR Egger | 11 | -0.16 | 1.52 | 0.92 | 0.855 | 0.043-16.898 |
| phylum Proteobacteria | Weighted median | 11 | -0.58 | 0.74 | 0.433 | 0.56 | 0.131-2.391 |
| phylum Proteobacteria | Weighted mode | 11 | -0.63 | 0.95 | 0.521 | 0.533 | 0.084-3.4 |
| phylum Tenericutes | IVW | 12 | -0.31 | 0.42 | 0.465 | 0.734 | 0.321-1.68 |
| phylum Tenericutes | MR Egger | 12 | -0.63 | 1.44 | 0.673 | 0.535 | 0.032-8.976 |
| phylum Tenericutes | Weighted median | 12 | -0.15 | 0.54 | 0.774 | 0.858 | 0.301-2.446 |
| phylum Tenericutes | Weighted mode | 12 | 0.268 | 0.87 | 0.765 | 1.308 | 0.236-7.244 |
| phylum Verrucomicrobia | IVW | 12 | -0.61 | 0.42 | 0.151 | 0.546 | 0.239-1.247 |
| phylum Verrucomicrobia | MR Egger | 12 | -1.33 | 1.11 | 0.256 | 0.264 | 0.03-2.301 |
| phylum Verrucomicrobia | Weighted median | 12 | -0.53 | 0.56 | 0.347 | 0.589 | 0.196-1.772 |
| phylum Verrucomicrobia | Weighted mode | 12 | -0.2 | 0.84 | 0.815 | 0.817 | 0.157-4.26 |

**Figure S1 Leave-one-out analysis of the causal effects of the significant microbial taxa on the three autoimmune neurologic disorders.** (A) Causal effect of gut microbiota on MS. (B) Causal effect of gut microbiota on GBS. (C) Causal effect of gut microbiota on MG.

**Table S4. Reverse MR results of causal links between the three autoimmune neurologic disorders and gut microbiome.**

| **Gut microbiome** | **Methods** | **SNPs** | **beta** | **P-value** | **OR** | **95%CI** |
| --- | --- | --- | --- | --- | --- | --- |
| ***Multiple sclerosis*** |  |  |  |  |  |  |
| genus Anaerotruncus | IVW | 118 | -0.007 | 0.443 | 0.993 | 0.976-1.011 |
|  | MR Egger | 118 | 0.002 | 0.932 | 1.002 | 0.965-1.040 |
|  | Weighted median | 118 | 0.011 | 0.454 | 1.011 | 0.982-1.041 |
|  | Weighted mode | 118 | 0.021 | 0.230 | 1.021 | 0.987-1.056 |
| genus Roseburia | IVW | 116 | -0.014 | 0.099 | 0.986 | 0.969-1.003 |
|  | MR Egger | 116 | -0.005 | 0.792 | 0.995 | 0.958-1.033 |
|  | Weighted median | 116 | -0.005 | 0.777 | 0.995 | 0.964-1.028 |
|  | Weighted mode | 116 | -0.005 | 0.798 | 0.995 | 0.961-1.031 |
| genus Ruminiclostridium 5 | IVW | 109 | 0.003 | 0.764 | 1.003 | 0.985-1.021 |
|  | MR Egger | 109 | 0.027 | 0.171 | 1.027 | 0.989-1.066 |
|  | Weighted median | 109 | 0.011 | 0.482 | 1.011 | 0.980-1.043 |
|  | Weighted mode | 109 | 0.013 | 0.488 | 1.013 | 0.977-1.05 |
| genus Ruminococcaceae | IVW | 115 | 0.005 | 0.631 | 1.005 | 0.986-1.024 |
|  | MR Egger | 115 | 0.019 | 0.361 | 1.019 | 0.979-1.061 |
|  | Weighted median | 115 | 0.010 | 0.506 | 1.010 | 0.980-1.041 |
|  | Weighted mode | 115 | 0.018 | 0.381 | 1.018 | 0.978-1.060 |
| genus Ruminococcus2 | IVW | 117 | 0.000 | 0.959 | 1.000 | 0.981-1.018 |
|  | MR Egger | 117 | -0.001 | 0.972 | 0.999 | 0.961-1.039 |
|  | Weighted median | 117 | -0.005 | 0.709 | 0.995 | 0.968-1.023 |
|  | Weighted mode | 117 | -0.010 | 0.609 | 0.990 | 0.953-1.029 |
| order Burkholderiales | IVW | 115 | -0.002 | 0.810 | 0.998 | 0.998-1.015 |
|  | MR Egger | 115 | -0.022 | 0.239 | 0.978 | 0.942-1.015 |
|  | Weighted median | 115 | -0.015 | 0.330 | 0.985 | 0.955-1.016 |
|  | Weighted mode | 115 | -0.012 | 0.520 | 0.988 | 0.951-1.025 |
| ***Guillain-barre syndrome*** |  |  |  |  |  |  |
| class Mollicutes | IVW | 4 | -0.002 | 0.902 | 0.998 | 0.970-1.027 |
|  | MR Egger | 4 | -0.117 | 0.253 | 0.889 | 0.770-1.028 |
|  | Weighted median | 4 | -0.004 | 0.797 | 0.996 | 0.962-1.030 |
|  | Weighted mode | 4 | -0.022 | 0.458 | 0.978 | 0.930-1.029 |
| genus Eubacterium （hallii group） | IVW | 6 | -0.008 | 0.381 | 0.992 | 0.974-1.010 |
|  | MR Egger | 6 | -0.005 | 0.913 | 0.995 | 0.914-1.083 |
|  | Weighted median | 6 | -0.008 | 0.455 | 0.992 | 0.971-1.013 |
|  | Weighted mode | 6 | -0.009 | 0.578 | 0.991 | 0.962-1.021 |
| genus Marvinbryantia | IVW | 4 | -0.015 | 0.256 | 0.985 | 0.959-1.011 |
|  | MR Egger | 4 | 0.005 | 0.937 | 1.005 | 0.894-1.131 |
|  | Weighted median | 4 | -0.018 | 0.214 | 0.982 | 0.954-1.011 |
|  | Weighted mode | 4 | -0.020 | 0.394 | 0.980 | 0.942-1.020 |
| phylum Tenericutes | IVW | 4 | -0.002 | 0.902 | 0.998 | 0.970-1.027 |
|  | MR Egger | 4 | -0.117 | 0.253 | 0.889 | 0.770-1.028 |
|  | Weighted median | 4 | -0.004 | 0.799 | 0.996 | 0.962-1.030 |
|  | Weighted mode | 4 | -0.022 | 0.501 | 0.978 | 0.924-1.035 |
| ***Myasthenia gravis*** |  |  |  |  |  |  |
| class Bacilli | IVW | 4 | -0.003 | 0.783 | 0.997 | 0.974-1.020 |
|  | MR Egger | 4 | -0.008 | 0.826 | 0.992 | 0.934-1.054 |
|  | Weighted median | 4 | -0.008 | 0.573 | 0.992 | 0.93-1.021 |
|  | Weighted mode | 4 | -0.012 | 0.533 | 0.988 | 0.954-1.023 |
| family Enterobacteriaceae | IVW | 4 | -0.005 | 0.718 | 0.995 | 0.968-1.023 |
|  | MR Egger | 4 | 0.007 | 0.928 | 1.007 | 0.884-1.147 |
|  | Weighted median | 4 | -0.007 | 0.690 | 0.993 | 0.962-1.026 |
|  | Weighted mode | 4 | -0.009 | 0.702 | 0.991 | 0.951-1.033 |
| genus Actinomyces | IVW | 4 | 0.007 | 0.722 | 1.007 | 0.969-1.046 |
|  | MR Egger | 4 | -0.047 | 0.648 | 0.9547 | 0.804-1.133 |
|  | Weighted median | 4 | 0.006 | 0.792 | 1.0067 | 0.962-1.053 |
|  | Weighted mode | 4 | 0.007 | 0.844 | 1.007 | 0.948-1.069 |
| genus Eubacterium （eligens group） | IVW | 3 | -0.012 | 0.407 | 0.988 | 0.961-1.016 |
|  | MR Egger | 3 | -0.013 | 0.760 | 0.987 | 0.928-1.051 |
|  | Weighted median | 3 | -0.013 | 0.442 | 0.987 | 0.955-1.020 |
|  | Weighted mode | 3 | -0.013 | 0.572 | 0.987 | 0.949-1.026 |
| genus Eubacterium （ruminantium group） | IVW | 5 | 0.001 | 0.936 | 1.001 | 0.970-1.033 |
|  | MR Egger | 5 | -0.034 | 0.472 | 0.966 | 0.891-1.049 |
|  | Weighted median | 5 | 0.004 | 0.841 | 1.004 | 0.967-1.042 |
|  | Weighted mode | 5 | 0.015 | 0.588 | 1.015 | 0.966-1.066 |
| genus Holdemania | IVW | 4 | 0.019 | 0.214 | 1.020 | 0.989-1.051 |
|  | MR Egger | 4 | 0.016 | 0.734 | 1.016 | 0.938-1.100 |
|  | Weighted median | 4 | 0.018 | 0.327 | 1.018 | 0.982-1.055 |
|  | Weighted mode | 4 | 0.016 | 0.521 | 1.016 | 0.973-1.061 |
| genus Lachnoclostridium | IVW | 5 | 0.004 | 0.736 | 1.004 | 0.983-1.025 |
|  | MR Egger | 5 | 0.006 | 0.842 | 1.006 | 0.953-1.062 |
|  | Weighted median | 5 | 0.002 | 0.881 | 1.002 | 0.977-1.028 |
|  | Weighted mode | 5 | -0.001 | 0.950 | 0.999 | 0.967-1.032 |
| genus Parabacteroides | IVW | 5 | 0.007 | 0.533 | 1.007 | 0.985-1.030 |
|  | MR Egger | 5 | 0.037 | 0.550 | 1.038 | 0.931-1.158 |
|  | Weighted median | 5 | 0.017 | 0.229 | 1.017 | 0.989-1.046 |
|  | Weighted mode | 5 | 0.021 | 0.338 | 1.022 | 0.983-1.062 |
| genus Ruminococcaceae UCG005 | IVW | 5 | -0.010 | 0.371 | 0.990 | 0.969-1.012 |
|  | MR Egger | 5 | -0.033 | 0.390 | 0.967 | 0.906-1.032 |
|  | Weighted median | 5 | -0.008 | 0.545 | 0.992 | 0.965-1.019 |
|  | Weighted mode | 5 | -0.009 | 0.646 | 0.991 | 0955-1.028 |
